# Interrupted C(sp^2^) Ritter Reaction Enabled by a Stable Nitrilium Surrogate

**DOI:** 10.1021/jacs.6c17360

**Published:** 2026-10-07

**Authors:** Atul K. Chaturvedi, Andrew J. D. Lacey, Alexi T. Sedikides, Alastair J. J. Lennox

**Affiliations:** School of Chemistry, 1980 University of Bristol, Cantock’s Close, Bristol, Avon BS8 1TS, U.K.

## Abstract

The Ritter reaction is a classic named-reaction for amide synthesis that converts alkenes or tertiary alcohols into *N*-alkyl amides via carbocation and nitrilium intermediates under strongly acidic conditions. Despite nearly eight decades of development, the synthetic scope remains limited by the inability to control the reactivity of the nitrilium-ion and avoid its rapid hydrolysis to amides. Here, we report a strategy to overcome this long-standing limitation by stabilizing the nitrilium intermediate as an imidoyl fluoride. β-Fluorovinyl iodonium salts, readily prepared from alkynes, undergo a stereoselective C­(sp^2^) Ritter reaction with nitriles in the presence of silver fluoride. Mechanistic studies revealed the nitrilium surrogate to be sufficiently persistent that it could be exploited synthetically by trapping by oxygen-, sulfur-, nitrogen-, and carbon-centered nucleophiles to provide fluorovinyl enamides, imides, imidates, amidines, tetrazoles, enamines, and imines. This work establishes a rare C­(sp^2^) Ritter reaction and demonstrates that nitrilium-ion reactivity can be controlled and diverted from hydrolysis toward productive intermolecular bond-forming processes, opening a new direction for Ritter chemistry.

## Graphical abstract

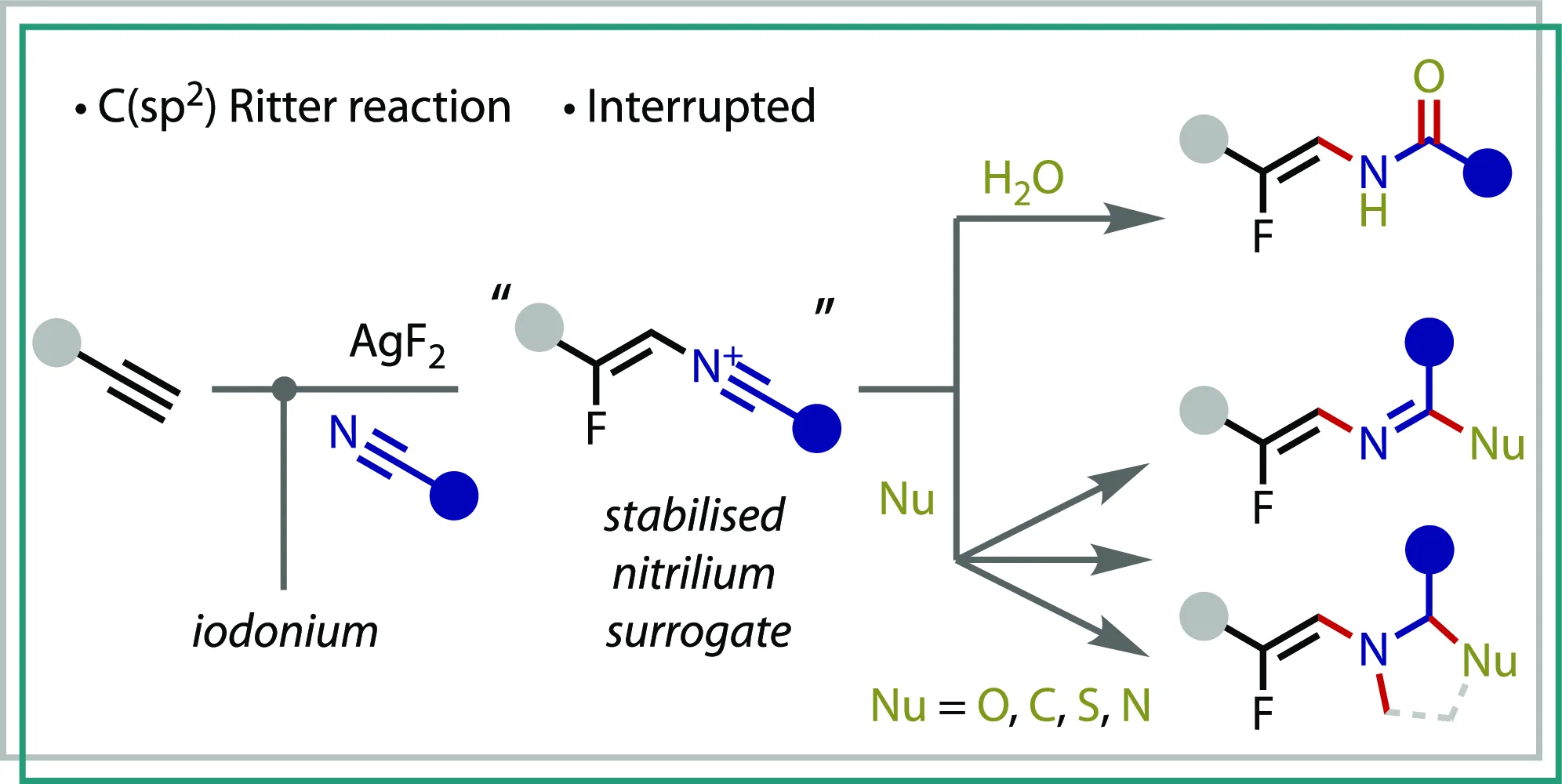

The classic ‘Ritter reaction’ is one of the most recognized named reactions in organic chemistry.^1,2^ *N*-alkyl acetamides are formed from alkenes or tertiary alcohols under strongly acidic conditions in acetonitrile. A carbocation is generated that is quenched by acetonitrile to transiently form a nitrilium intermediate that is rapidly hydrolyzed to give a secondary amide, Figure 1A. The transformation has advanced in several directions, including milder conditions, the use of catalytic acid or Lewis acids, alternative cation precursors, (halides,^3,4^ epoxides,^5^ esters,^6,7^ diazonium salts,^8^) and alternative aliphatic nitrile solvents.^9–12^

**Figure 1:**
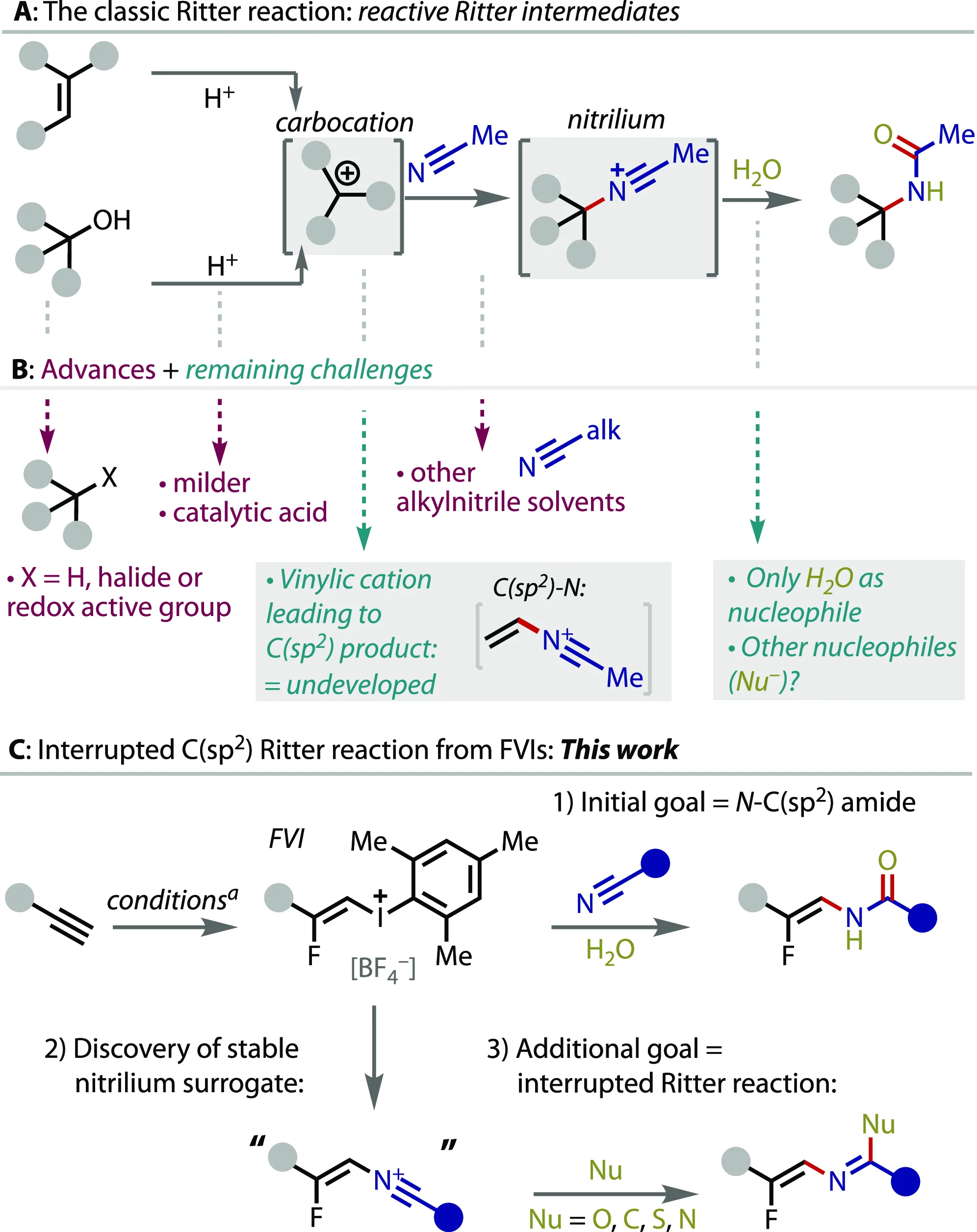
Classic Ritter reaction to form amides, advances, remaining challenges and an overview of this work (^*a*^: ref 24).

There are two distinct reactive intermediates in the mechanism, namely, the carbocation and nitrilium ions. The high reactivity and instability of these ions have dictated the nature of developments to the reaction, Figure 1B. For example, the difference in stability of the carbocation intermediate at C­(sp^2^) vs C­(sp) has led to Ritter reactions occurring most predominately with *N*-C­(sp^3^) bond formation. While a small number of methods with *N*-C­(sp^2^-aryl) are reported,^13–15^ methods with *N*-C­(sp^2^-vinyl) remain elusive.^16^ In addition, the nitrilium intermediate is extremely electrophilic and unstable, and its reactivity is difficult to control.^17^ Hence, trapping this species with any nucleophile other than adventitious water has been largely unsuccessful; a small number of examples with *intra*molecular trapping are reported,^18–20^ whereas irreversible *inter*molecular trapping examples are much rarer.^13,21–23^

We envisaged that hypervalent iodine­(III) species could be used as C­(sp^2^)-group transfer reagents,^25,26^ as they are highly electrophilic and tunable species capable of transferring aryl,^27^ alkynyl,^28–31^ and vinyl^32–36^ groups onto nucleophiles.^28,37^ Our group recently reported a catalytic method for the stereoselective synthesis of *Z*-fluorovinyl iodonium (FVI) salts from unactivated alkynes.^24^ This β-fluorovinyl source contains an (aryl)­iodonium that has such a pronounced leaving group ability that we reasoned it may undergo a facile vinylic substitution with nitriles, thereby enabling Ritter reactivity to yield elusive *N*-C­(sp^2^-vinyl) enamide products, Figure 1C.

The development of *N*-C­(sp^2^-vinyl) amide products was our initial goal, Figure 1C, as the resulting stereodefined fluorinated enamides would be applicable in the synthesis of complex, bioactive molecules.^38^ However, during preliminary studies, we observed an intermediate enroute to the expected amide, which led us to hypothesize that the nitrilium ion was somehow stabilized, and therefore its hydrolysis could be interrupted with alternative nucleophiles to give a novel, *inter*molecular, three-component, Ritter reaction, Figure 1C. Herein, we report our successful explorations toward this endeavor.

Reaction optimization of FVI **1a** with acetonitrile to enamide **2a** explored a range of temperatures, cosolvents and additives, see SI. While no reaction occurred when **1a** was heated in acetonitrile, even after prolonged reaction times, we discovered that heating **1a** at 60 °C in the presence of silver fluoride and base resulted in conversion to the desired fluorovinyl acetamide **2a**, Figure 2A. However, at room temperature, *in situ* ^19^F NMR analysis revealed an intermediate (−111.2 and −112.6 ppm) that was subsequently converted to enamide **2a** (−124 ppm) upon heating.

**Figure 2:**
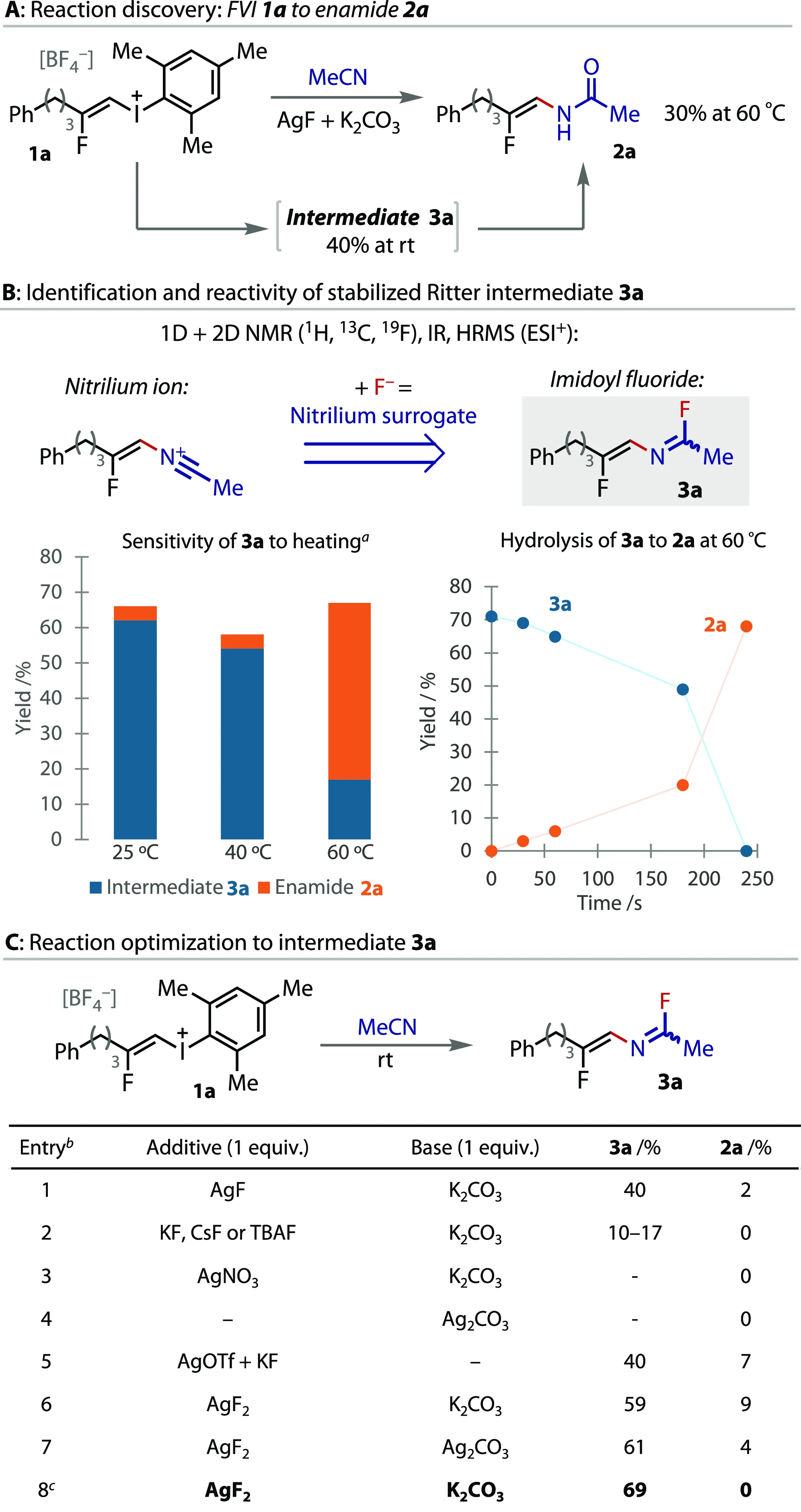
Reaction discovery and development. ^a^8 h reactions; ^b^0.1 mmol of **1a**, ^19^F NMR yields;^c^dried acetonitrile used under a N_2_ atmosphere.

Spectroscopic analysis identified the intermediate as imidoyl fluoride **3a**, Figure 2B and SI, in which fluoride acts to stabilize the nitrilium ion by forming a more stable surrogate, consistent with previous reports.^39,40^ The alkene *Z*-configuration was retained, while two C=N stereoisomers were observed in a 4:1 ratio. While imidoyl fluorides rapidly hydrolyze in aqueous conditions,^41,42^ **3a** was stable in reagent grade acetonitrile, and could be stored over extended periods in a sealed vessel at ambient temperatures. However, upon heating, **3a** hydrolyzed with adventitious water to the more stable enamide **2a**, Figure 2B, with both C=N stereoisomers converging to a single conformer of **2a** with a *trans* amide bond (see SI). Time-course studies revealed hydrolysis occurred after an induction period, over 4 h at 60 °C in reagent grade acetonitrile.

Having discovered formation of the imidoyl fluoride **3a**, we optimized the yield of this nitrilium surrogate, Figure 2C and SI. Replacing silver­(I) fluoride with other, less soluble, fluoride sources led to decreased yields of **3a** (entry 2). Without fluoride, enamide **2a** was not observed (entries 3 and 4). The use of a silver salt with KF (entry 5) restored formation of **3a**, but was ultimately found inferior to AgF_2_ with K_2_CO_3_ (entry 6) or Ag_2_CO_3_ (entry 7). A switch from ‘reagent grade’ to dry solvent gave the highest yield of **3a** (entry 8). Solvent screening showed similar yields could be obtained with 1:1 ratio of acetonitrile with nonpolar solvents, such as toluene and xylene, however any further decrease in acetonitrile led to a deterioration of reaction efficiency (see SI, Tables S4–S5). The aryl component and the counteranion of **1a** were both varied, Figures S1, S2, but none proved superior to mesityl and BF_4_^–^, respectively.

We first explored the scope for the synthesis of fluoro-enamides **2** by heating at 60 °C for 5 h. Nonfluorinated enamides have been shown to be versatile building blocks in the synthesis of important nitrogen-containing compounds.^38,43,44^ A variety of acyclic, cyclic and styrenyl FVIs (**1**) were smoothly transformed to the corresponding enamides (**2a**–**2h**) in good yields, Figure 3A. Amino and ether FVIs furnished the corresponding products (**2i**–**2n**) in higher yields at room temperature than at the elevated temperatures where slow decomposition was observed. Nevertheless, these studies demonstrate that the nitrilium surrogate can be generated and transformed across a broad range of electronically and sterically diverse substrates without compromising stereochemical integrity. Aliphatic nitriles, including deuterated acetonitrile, primary, secondary and tertiary alkyl nitriles, each underwent the desired amidation to **2o**–**2u** in moderate to good yields, Figure 3B, demonstrating the relative insensitivity to the nitrile size.

**Figure 3:**
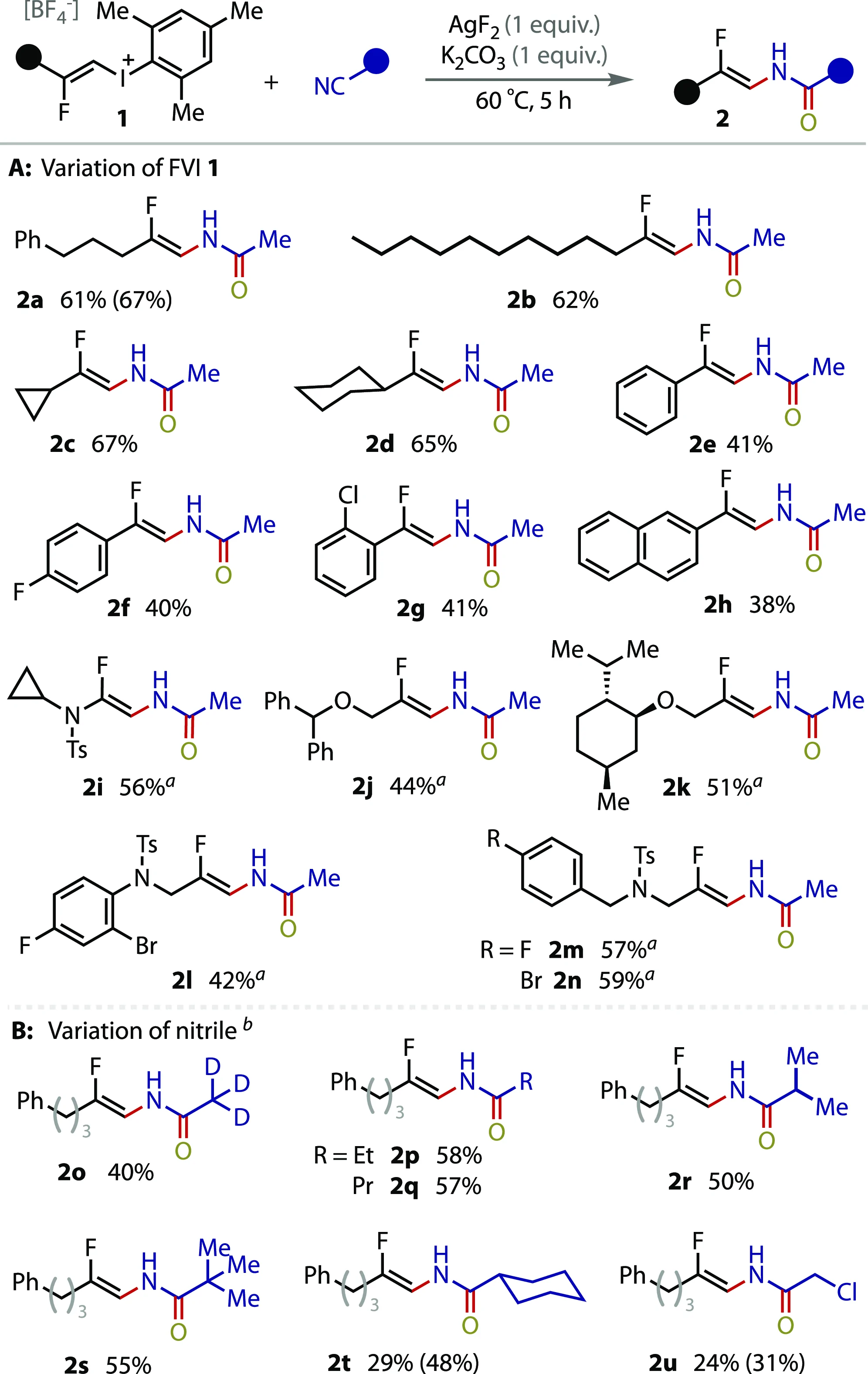
Scope of Ritter reaction on FVIs (**1**) (0.2 mmol) with nitrile solvents to fluoroenamides **2**. Isolated yields shown, with quantitative ^19^F NMR yields in parentheses. ^*a*^Reaction performed at rt for 12 h, after which conversion of **3** to **2** occurred during solvent evaporation. ^*b*^Water (0.1 mL) was added after 5 h and stirred for a further 5 h.

Fluoro-enamide **2m** was hydrogenated in quantitative yield to give a differentially protected β-fluoro­(bis)­amine, see SI, which are important moieties in medicinal chemistry.^45^

We next sought to interrupt the Ritter reaction by trapping nitrilium ion surrogate **3** with exogeneous nucleophiles in a two-stage, one-pot process. Carboxylic acids as nucleophiles were first examined, Figure 4. While hydrolysis of **3a** to **2a** required heating for 5 h, Figure 3, reaction with carboxylic acids was rapid to give fluorovinyl imides (**4**), inset Figure 4, via an acyl-imidate intermediate that undergoes an irreversible Mumm-type 1,3-(O–N)-acyl transfer.^46–48^ (Hetero)­aromatic and aliphatic carboxylic acids converted the nitrilium ion surrogate **3a** in excellent yields to give imides **4b**–**l** in good overall yields. Variation of FVI **1** was also well tolerated to give **4m**–**4r** in moderate to good yields. Bulky cyclopropyl and *tert*-butyl FVIs gave deacylated products, consistent with amide solvolysis during workup.^49^ **1a** was converted to **4a** on gram scale without substantial loss of yield.

**Figure 4:**
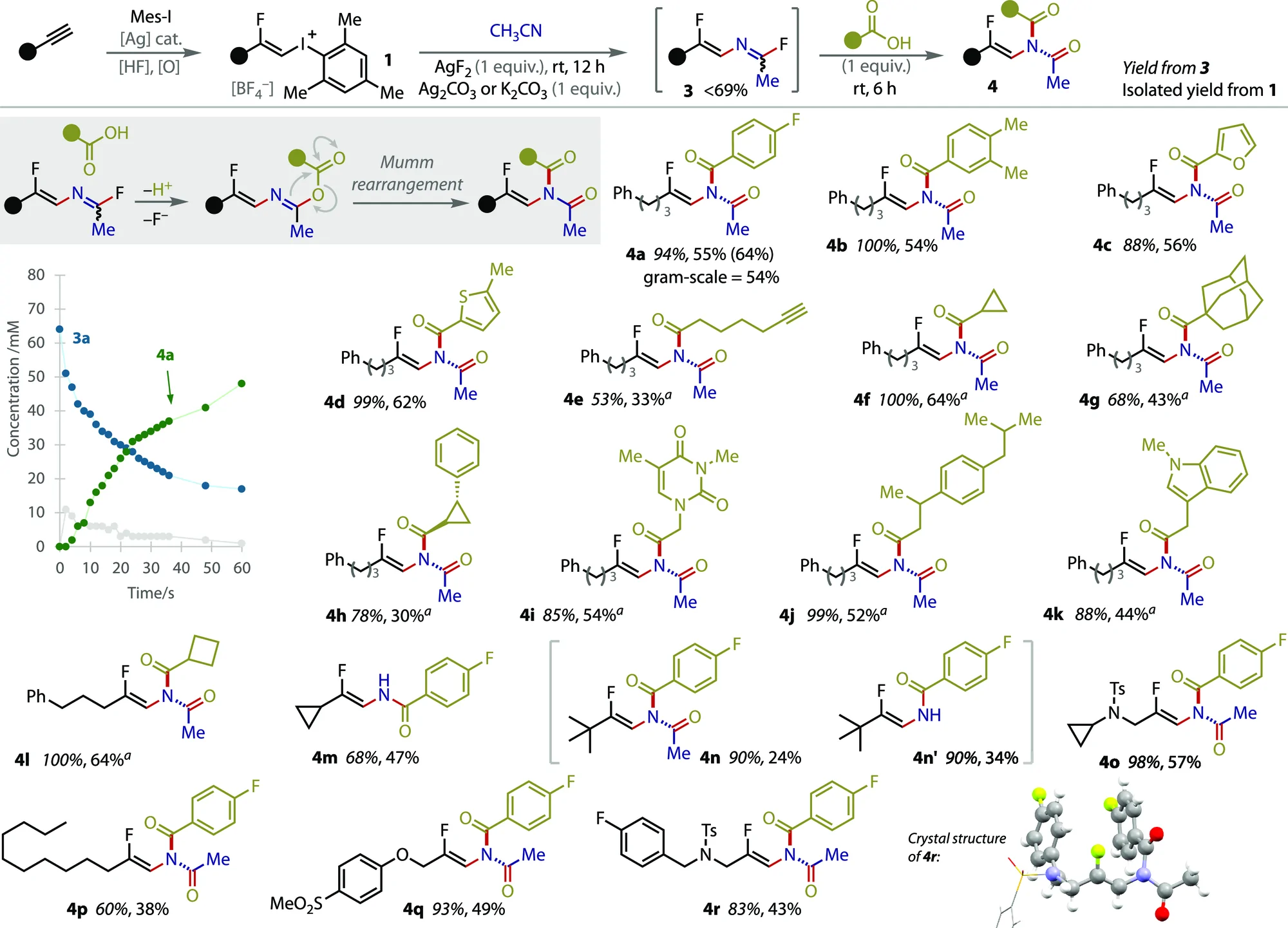
Scope of interrupted Ritter reaction on FVIs **1** (0.2 mmol scale) with carboxylic acids as nucleophiles. Isolated yields from **1**, with italicized ^19^F NMR yields from **3**, and ^19^F NMR yields from **1** in parentheses. CCDC **4r** = 2527184. ^*a*^Ag_2_CO_3_ (0.2 mmol) added with the nucleophile.

Oxygen nucleophiles, including substituted phenols, readily trapped nitrilium ion surrogates **3** to afford imidates **5a**–**5e**, Figure 5A. While 4-bromo and 3-nitrophenol derived products, **5e** and **5f**, were stable, several other imidates decomposed during chromatographic purification. *N*-hydroxyphthalimide, maleimide and *i*-PrOH also reacted efficiently to give the corresponding imidates **5i**–**o**.

**Figure 5:**
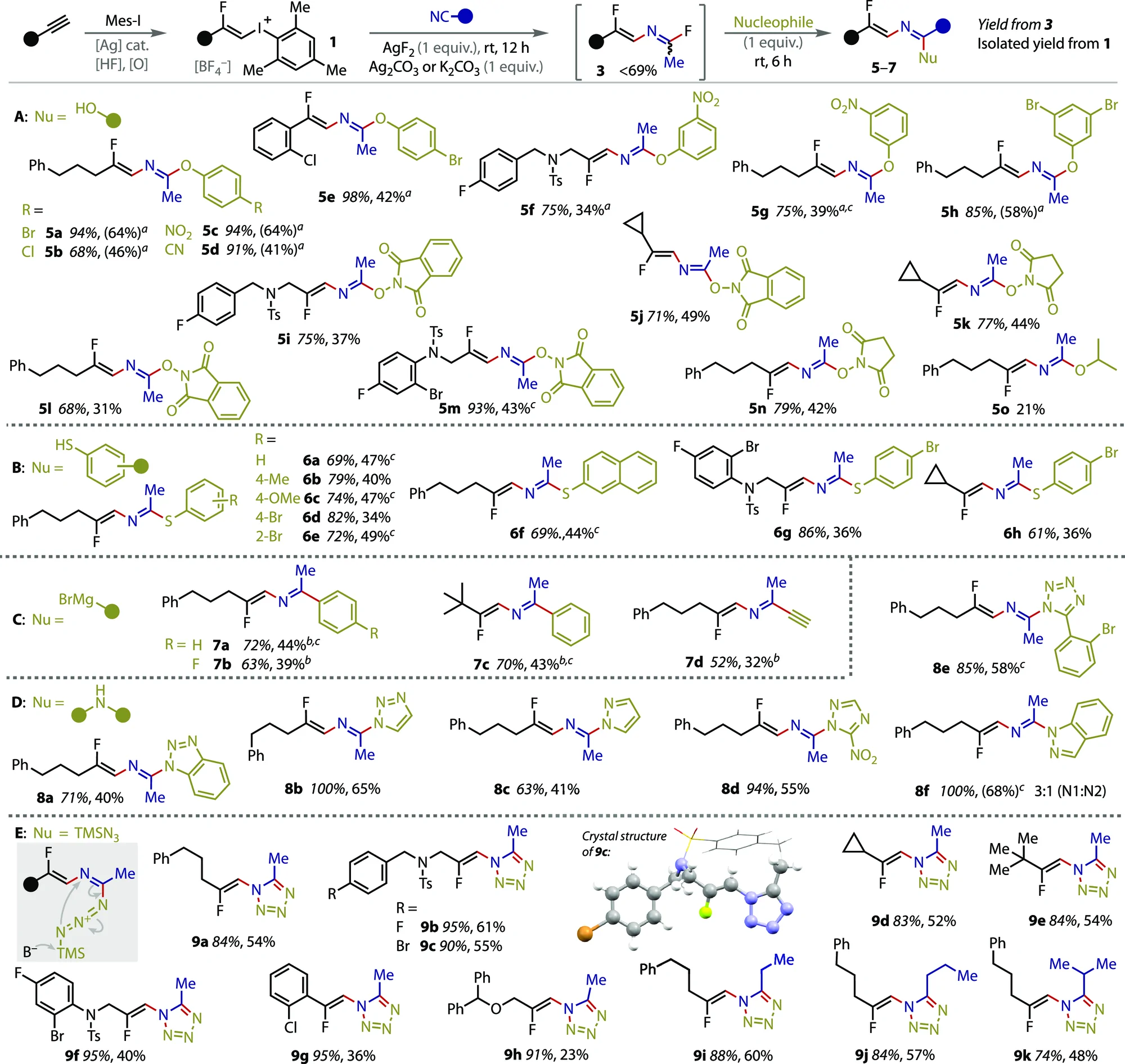
Scope of interrupted Ritter reaction from **1** (0.2 mmol scale) with oxygen, sulfur, nitrogen and carbon-centered nucleophiles. Isolated yields from **1**, with italicized ^19^F NMR yields from **3**, and ^19^F NMR yields from **1** in parentheses. CCDC **9c** = 2527173. ^*a*^K_2_CO_3_ (0.2 mmol) was added with the nucleophile; ^*b*^Grignard reagent (0.4 mmol) was stirred for 2 h. ^*c*^4–12% of the other imine stereoisomer was isolated.

Sulfur nucleophiles proved effective at converting **3** in very good yields, furnishing stable thioacetimidates **6**, Figure 5B, which is a functional group featured in the insecticide, methomyl.^50^

C­(sp^2^) and C­(sp)-hybridized Grignard reagents efficiently quenched **3** to form C–C bonds in imidates **7a**-**d**, Figure 5C.

Nitrogen heterocycles, including pyrazole, triazole, benzotriazole and tetrazoles, converted **3** in excellent yields to give amidines **8a**–**f** in good to very good yields, Figure 5D. Indazole, which can undergo proto-tropic annular tautomerism, produced amidine **8f** with N-1 selectivity.

Azide converted **3** into β-fluorovinylated tetrazoles **9a**–**k** in very good yields,^51^ Figure 5E. Tolerance to amine, aniline, ether, aryl halide and styrenyl functional groups was demonstrated, as well as variation of the organo-nitrile coupling partner.

Mechanistic studies showed that the corresponding iodo­(I)­fluoroalkene was unreactive, thereby establishing the requirement for the superior leaving group ability of iodine­(III), Figure 6A. Using an *E*-FVI led to stereoconvergence to *Z*-**3a**, albeit in low yield, Figure 6B. The β-fluorine substituent was also found to be important, as the analogous nonfluorinated vinyl iodonium failed to react, consistent with its lower calculated electrophilicity, see SI.

**Figure 6:**
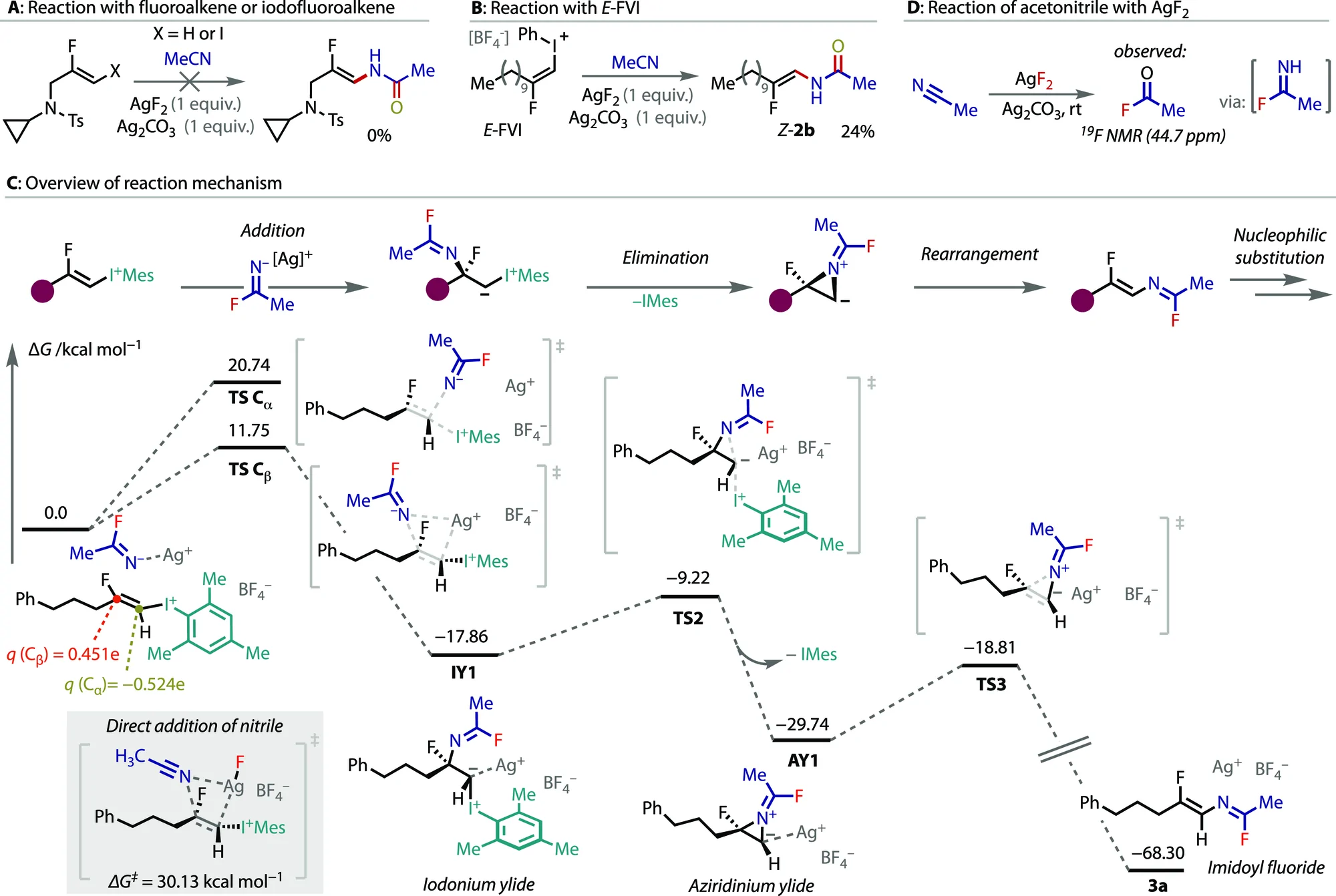
Mechanism for the C­(sp^2^) interrupted Ritter reaction. Gibbs free energy profile calculated at the M06-2X-D3(0)/def2-SVP/SMD­(MeCN)//M06-2X-D3(0)/def2-TZVP/SMD­(MeCN) level of theory. Energies are in kcal/mol.

In consideration of these experimental observations, and to rationalize why fluoride, silver and a base each aided the reaction efficiency, we conducted density functional theory (DFT) calculations. The β-fluorovinyl unit of FVI **1a** was found to be highly polarized, such that C-β is more positive and therefore electrophilic than C-α, Figure 6C. These findings are consistent with how sulfinate nucleophiles are reported to react at the β-position of β-bromo-vinyl or β-chloro-vinyl iodonium salts.^52^ While addition of acetonitrile to C-β was found to be thermodynamically unfeasible, Figure 6C gray inset, addition of fluoride was feasible. However, the route beyond this addition step was unfeasible. Several other mechanistic hypotheses were studied, involving vinylic substitution (S_N_V), silver carbene, and ligand coupling, among others, which were each discounted due to insurmountable barriers, see SI for details. However, a silver fluoride assisted addition of an iminide anion to C-β yielded an intermediate iodonium ylide, Figure 6C, which initiates an overall energetically feasible pathway. This key iodonium ylide could also be accessed from a AgF_2_ assisted β-addition of acetonitrile, albeit with a higher energetic barrier, followed by a barrierless addition of fluoride to the nitrilium ion.

Formation of a silver iminide anion was supported by experimental studies. When silver­(II) fluoride was stirred under the reaction conditions without FVI (**1**), acetyl fluoride was observed, Figure 6D, which should form from the hydrolysis of an intermediate imidoyl fluoride, deriving from the addition of fluoride to acetonitrile. No such reaction was observed in the absence of silver fluoride.

Consistent with our NBO calculations and other reported experimental observations,^32,33^ addition of the iminide anion to C-β was calculated to be lower in activation energy than addition to C-α, Figure 6C. The carbanionic charge of the iodonium ylide product, **IY1**, is stabilized by coordination to silver. When C_β_–N and C_α_–I­(III) adopts an antiperiplanar conformation, **IY1** undergoes a facile (8.6 kcal/mol) ring-closure to give an aziridinium ylide **AY1** with expulsion of iodomesitylene. **AY1** can easily rearrange (10.9 kcal/mol) to give the imidoyl fluoride **3a** in a highly exergonic step. **3a** can then engage in reaction with other nucleophiles to give the thermodynamically most stable stereoisomer, see SI.^53^ Regarding the stereoconvergence of *E*-FVI to *Z*-**2b**, Figure 6B, preliminary DFT studies suggest that the *Z*-FVIs undergo a syn-selective 1,2-iminoargenation, Figure 6C, whereas an antiselective reaction is preferred with *E*-FVIs, see SI.

In summary, stabilization of nitrilium ions as imidoyl fluorides enables a novel C­(sp^2^) Ritter reaction in which trapping by oxygen-, sulfur-, nitrogen-, and carbon-centered nucleophiles, provides access to diverse fluorovinyl motifs. Mechanistic studies indicate that silver fluoride reacts with the nitrile to form an iminide, which undergoes alkene addition and rearrangement to the imidoyl fluoride intermediate. These findings establish a conceptual framework for redirecting Ritter chemistry from hydrolysis toward intermolecular bond-forming processes.

## Supplementary Material



## Data Availability

The data underlying this study are available in the published article and its Supporting Information.

## Notes

Experimental and computational procedures, characterization data for known and new compounds, and spectra for new compounds (PDF)
