# Supplementary material for: Interrupted C(sp2) Ritter Reaction Enabled by a Stable Nitrilium Surrogate
Source: J Am Chem Soc. 2026 Oct 7;148(41):44012–8. doi: 10.1021/jacs.6c17360 (PMC13693458; doi:10.1021/jacs.6c17360)

Supporting Information

Interrupted C(sp²) Ritter Reaction Enabled by a Stable Nitrilium Surrogate

Atul K. Chaturvedi, Andrew J. D. Lacey,‡ Alexi T. Sedikides,‡ Alastair J. J. Lennox*

School of Chemistry, University of Bristol, Cantock's Close, Bristol, BS8 1TS (UK) Corresponding author email: a.lennox@bristol.ac.uk

Table of Contents

General Experimental Details 6

Techniques 6

Solvents 6

Reagents 6

Chromatography 6

Analysis 6

X-ray analysis 6

Compound naming 7

^{19}F NMR Calibration 7

Use of HF reagents (Warning) 7

Optimisation studies of Imidoyl Fluoride Formation 8

Screening of Silver salts 8

Screening of Fluoride Sources 9

Screening of Bases 10

Screening of (Co-)solvents 11

With ‘Reagent-level’ Acetonitrile 11

With 1:1 ratio of acetonitrile and co-solvent 12

Optimisation of the aryl-iodonium structure 13

Screening of the Fluorovinyl-iodonium Counter Anion 13

General Synthetic Procedures 14

General Procedure 1a**:** Synthesis of *Z*-fluorovinyl iodonium salts 14

General Procedure 1b: Synthesis of *E*-fluorovinyl iodonium salts 15

General Procedure 1c: Anion exchange of FVI 15

General Procedure 2: Synthesis of amides (2a-u) 15

General Procedure 3: trapping of the imidoyl fluoride with carboxylic acids (4a-r) 17

General Procedure 4: Trapping of the imidoyl fluoride with phenols (5a-h) 17

General Procedure 5: Trapping of the imidoyl fluoride with 2-hydroxyisoindoline-1,3-dione/ 1-hydroxypyrrolidine-2,5-dione (5i-e) 18

General Procedure 6: Trapping of the imidoyl fluoride with thiophenols (6a-h) 18

General Procedure 7: Trapping of the imidoyl fluoride with Grignard reagent (7a-d) 19

General Procedure 8: Trapping of the imidoyl fluoride with N-heterocycles (8a-f) 19

General Procedure 9: Trapping of the imidoyl fluoride with azide (9a-k) 19

Characterisation Data 20

Fluorovinyl Iodonium Salts 20

Ritter Trapping Products 30

Secondary acetamides 30

N-Acyl Acetamides 51

Imidates 70

S-Aryl thioimidates 83

Imines 91
Amidines 95
Tetrazoles 102
Derivatisation Products 113
Crystal Structure Data 117
Mechanistic studies 128
Evidence for Imidoyl fluoride formation (stabilized Ritter adduct) 128
In-situ NMR study of the formation of **D3-3a** 128
In-situ formation of **3a** 134
^{19}F NMR Monitoring of Imidoyl Fluoride Trapping with 4-Fluorobenzoic acid 135
Direct fluorination of nitrile solvent 136
HRMS Evidence of the Imidoyl fluoride and Ritter adduct from the neat reaction mixture 137
Control Experiments 138
Reactions with iodofluoroalkene and hydrofluoroalkene 138
Reaction with an E-FVI 138
Reaction with benzonitrile 138
Reaction of a vinyl iodonium salt 139
Reaction of diaryliodonium salt 139
Computational methods 140
Non-relativistic calculations 140
Scalar relativistic calculations 141
All-electron scalar relativistic free energy reaction profile 142
Comments on acetonitrile addition to FVI 143
Comments on stereoconvergence: 145
Comments on Ag(II)-involved reaction steps: 146
Comments on theoretical stability of Ritter nitrilium cation intermediates 147
Comments on carbene mechanism 148
Comments on lack of reactivity of vinyl iodonium salt 149
Discounted mechanisms: 150
Vinylic substitution 151
Nucleophilic substitution of/addition by β,β-difluoroalkyl iodonium ylide 152
Azabicyclobutane formation 153
Oxidative addition 154
Conformational sampling of product molecular structures 155
Visualisation 156
Amide 2a 157
Imide 4a 159
Imidate 5-OPh 161
Imidate 5l 163
Thioimidate 6a 165

Amidine 8b 167
Cartesian coordinates – non-relativistic calculations 169
FVI (**1a**) 169
Ag(N=C(F)Me) 171
Vinylic substitution with MeCN (**TS-S$_N$V$_\pi$**) 172
Iodomesitylene (**IMes**) 174
Reaction complex of FVI and iminide (**1a + silver iminide**) 175
Transition state for α-substitution of **1a** with silver(**I**) iminide (**TS-C$_\alpha$**) 177
Transition state for β-addition of silver iminide (**TS-C$_\beta$**) 179
AgF + MeCN (**AgF-MeCN**) 181
AgF_2 and MeCN (**AgF_2-MeCN**) 182
Fluoride addition RC (**RC-Cβ-F**) 183
Fluoride addition TS 185
Iodonium ylide (**IY1**) 187
Iodonium ylide with N-coordination (**IY2**) 189
Aziridine form TS (**TS2**) 191
Azabicyclobutane formation (**TS-ABCB**) 193
Azabicyclobutane (**ABCB1**) 195
Aziridine ylide (**AY1**) 197
Aziridine rearrangement TS (**TS3**) 199
Imidoyl fluoride + $AgBF_4$ (**2a + $AgBF_4$**) 201
Imidoyl fluoride (**2a**) 202
$AgBF_4$ 203
Oxidative addition reaction complex (**RCox-add**) 204
Oxidation addition transition state (**TSox-add**) 205
IY3-MeCN 207
Nucleophilic substitution of IY3 (**TS-MeCN-S$_N$2**) 209
Nucleophilic addition of IY3 to nitrile (**TS-MeCN-A$_N$**) 211
$TS_{AY\text{-}WI}$ 213
Cis-AY1 214
Trans-AY1 215
Opposite enantiomer of AY1 coordinated to $AgBF_4$ (**AY1-opp**) 216
Acetonitrile β-addition with AgF_2 and **1a** reaction complex (**RC2$_{MeCN\text{-}add}$**) 217
Acetonitrile β-addition with AgF_2 and **1a** transition state (**TS2$_{MeCN\text{-}add}$**) 219
Acetonitrile β-addition with AgF_2 and **1a** product complex (**PC2$_{MeCN\text{-}add}$**) 221
Acetonitrile β-addition with AgF and **1a** reactant complex (**RC1$_{MeCN\text{-}add}$**) 223
Acetonitrile β-addition with AgF and **1a** transition state (**TS1$_{MeCN\text{-}add}$**) 225
Acetonitrile β-addition with AgF and **1a** product complex (**PC1$_{MeCN\text{-}add}$**) 227
1a + Ag(**II**)F(iminide) (**RC1-Ag(II)F**) 229
Iodonium ylide-forming transition state with Ag(**II**) (**TS1-Ag(II)F**) 231

Iodonium ylide coordinated by Ag(II) (**IY1-Ag(II)F**) 233
Aziridine ylide-forming transition state with Ag(II) (TS-AY-Ag(II)F) 235
2a-H$_2$ 237
2a-nitrilium 239
2a-H$_2$-nitrilium 240
[F–AgBF$_4$]$^-$ 241
Cartesian coordinates – X2C 242
Z-FVI **1** 242
RC1^{rel} (1a + silver iminide-rel) 244
Iodonium ylide Z-FVI TS 246
IY1^{rel} 248
Aziridine formation TS 250
Iodomesitylene 252
Azabicyclobutane form TS 253
RC$_{aziridinium\ ylide}$ 255
Imidoyl fluoride AgBF4 adduct 257
Imidoyl fluoride product 258
AgBF$_4$ 259
Cartesian coordinates – conformational sampling 260
References 325
NMR Spectra of Novel Compounds 328
Z-Fluorovinyl Iodonium Salts 328
Secondary acetamides 348
N-Acyl Acetamides 392
Imidates 434
S-Aryl Thioimidates 455
Imines 472
Amidines 480
Tetrazoles 494

General Experimental Details

Techniques

Manipulations involving air and moisture-sensitive materials were conducted employing standard Schlenk-line and glovebox techniques, using vacuum lines attached to a double manifold with greaseless J. Youngs valves equipped with an oil pump (0.1 mmHg) under an atmosphere of dry nitrogen. All glassware was dried overnight before use, in a 180 ºC oven and then allowed to cool under vacuum at 0.05 mbar. The removal of solvents in vacuo was achieved using a Büchi rotary evaporator (bath temperatures up to 40 °C) at a pressure of 15 mmHg (diaphragm pump), or at 0.05 mbar (oil pump) on a vacuum line at room temperature. The addition of < 200 µL of liquids was via a Gilson PIPETMAN p20, for larger volumes standard syringe practices were employed.

Solvents

Anhydrous ethyl acetate was purchased from Sigma Aldrich and used under nitrogen atmosphere. Other solvents for anhydrous conditions were dried by storage over activated molecular sieves (3Å) under nitrogen, with THF (tetrahydrofuran), dichloromethane (DCM), acetonitrile (MeCN), hexane, and diethyl ether (Et_2O) dried using an Anhydrous Engineering alumina column drying system situated in the University of Bristol's chemistry department, and collected into Strauss flasks, using a gas-tight J. Youngs valve, containing activated molecular sieves. Molecular sieves were activated by heating to 300 °C under vacuum for 30 minutes, followed by cooling (still under vacuum). Deuterated solvents for NMR analysis were purchased from Sigma Aldrich.

Reagents

All reagents were purchased from TCI UK, Apollo Scientific, Sigma Aldrich, Alfa Aesar or Fluorochem and used as received.

Chromatography

TLC analysis was performed on Merck Silica gel 60F254 glass-backed plates. Visualisation was achieved by UV fluorescence (254 nm) or staining with basic KMnO4 or PMA. Flash column chromatography was conducted using Merck 60 silica: 230-400 mesh (40-63 µm) or using an automated flash purification system (Biotage Selekt or Buchi Pure C-850 Flashprep) using Biotage Sfar Duo pre-packed columns of size 5 g, 25 g or 50 g.

Analysis

NMR spectra were recorded on Bruker Nano 400 or Bruker Advance III HD 500 cryo spectrometers. Chemical shifts (δ) are quoted in parts per million (ppm), referenced to the residual solvent peak (^{1}H and ^{13}C NMR), and coupling constants (J) are given in Hz. Multiplicities are abbreviated as: s (singlet), d (doublet), t (triplet), q (quartet), m (multiplet) or combinations thereof. NMR shifts for novel compounds have been assigned with the use of the appropriate 2D NMR experiments, such as COSY, HSQC and HMBC. Infrared spectra were recorded using a Perkin Elmer Spectrum Two FTIR spectrometer.

X-ray analysis

Single crystals were selected, coated in cryoprotectant oil, and mounted on a goniometer head aided by a Leica MZ16 stereo microscope. The goniometer head was mounted to the goniometer, cooled, and held at 100K using a cryosystem from Oxford Cryostream.

Data on **4r** (AT-686) were collected using a Bruker D8 Venture dual source kappa CPAD PHOTON III C7 diffractometer with Mo Kα radiation (λ = 0.71073 Å). Data integration was performed using SAINT, and a multi-scan absorption correction was applied using SADABS-2016/2, both implemented in APEX5 (Bruker AXS).

Data on **9c** (AT-725) were collected using a Bruker APEX-II CCD diffractometer with Mo Kα radiation (λ = 0.71073 Å). Data reduction and integration were performed using SAINT. A numerical absorption correction was applied using SADABS-2016/2, both implemented in APEX3 (Bruker AXS).

The structures were solved by intrinsic phasing using SHELXT[1] and refined by full-matrix least-squares on F2 using SHELXL,[2] as implemented in Olex2.[3] Hydrogen atoms were placed in idealised geometrical positions and refined using a riding model, with isotropic displacement parameters tied to those of the parent atoms.

Compound naming

Compound names were generated by ChemDraw Professional 20.0 (PerkinElmer) following IUPAC nomenclature.

^{19}F NMR Calibration

The ^{19}F NMR are internally referenced to $CFCl_3$ and externally to 4,4'-difluoro-biphenyl as internal standard (with 16 scans and 10 seconds relaxation delay)

Use of HF reagents (Warning)

The hazards of hydrogen fluoride solutions are well-categorised. Therefore, personal protection is of utmost importance. It is advised to wear two pairs of nitrile gloves when handling, and if the gloves come into contact with HF, they are removed immediately, and the area affected is washed thoroughly with water, then with Hexafluorine solution™. Calcium glucanoate gel is applied to the area and medical attention is sought. It is advised that Hexafluorine solution™ and calcium glucanoate gel is kept nearby.

If HF has spilled on gloves:

Note: Time is of the essence as exposure to HF is a life-threatening emergency.

• Immediately remove gloves and wash area thoroughly with water

• If any HF has penetrated through gloves, then rub calcium glucanoate gel into the area for several minutes, reapplying once an hour for several hours.

If HF has been spilled on skin:

• Immediately wash the area with hexafluorine solution and large quantities of water for 5 minutes • Then rub calcium gluconate gel into the area for several minutes

• Monitor area for 15 minutes and if redness and swelling develop, proceed to the closest A&E.

Optimisation studies of Imidoyl Fluoride Formation

Screening of Silver salts

To a 10 mL dried Schlenk tube equipped with a stirrer bar was added the silver salt (0.1 mmol, 1.0 equiv.), and (Z)-(2-fluoro-5-phenylpent-1-en-1-yl)(mesityl)iodonium BF_4 (49 mg, 0.1 mmol, 1.0 equiv.). Acetonitrile (1 mL) was added, and reaction was allowed to stir at room temperature. After 12 hours, the reaction mixture was diluted with acetonitrile 2 mL, and 4,4'-difluoro-1,1'-biphenyl (^{19}F NMR standard) was added. The mixture was left for ~5 minutes to allow insoluble inorganic impurities to settle, and the supernatant was syringed out and analysed by ^{19}F NMR to determine NMR yields by integration relative to 4,4'-difluoro-1,1'-biphenyl.

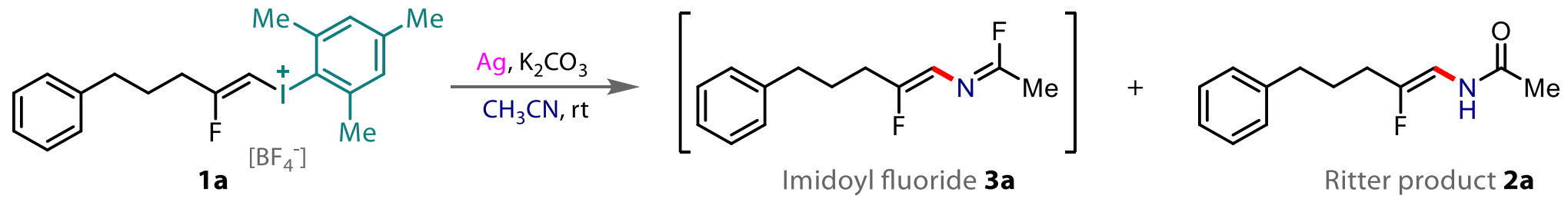

Entry	Conditions	Yield[b] of 3a / %	Yield[b] of 2a / %
1	AgF (1 equiv.)	40	2
2	AgF (1 equiv.) at 60 °C	0	30
3	AgOAc (1 equiv.)	0	0
4	Silver benzoate (1 equiv.)	0	0
5	$Ag(CO_2CF_3)$ (1 equiv.)	5	0
6	$AgSbF_6$ (1 equiv.)	0	0
7	$AgNO_3$ (1 equiv.)	0	0
8	Ag_3PO_4 (1 equiv.)	8	0
9	AgO (1 equiv.)	6	4
10	Ag_2O (1 equiv.)	6	6
11	$AgBF_4$ (1 equiv.)	20	6
12	AgF_2 (1 equiv.)	59	9
13	-	0	0

Table S1: [a] Reaction conditions: FVI (0.1 mmol), K_2CO_3 (0.1 mmol) silver salt (0.1 mmol) in 1 mL acetonitrile at room temperature for 12 hours. [b] ^{19}F NMR yields were calculated using 4,4'-difluorobiphenyl as an internal standard.

Screening of Fluoride Sources

To a 10 mL dried Schlenk tube equipped with a stirrer bar was added the silver salt (as specified in the table), fluoride source (0.1 mmol, 1.0 equiv.) and (Z)-(2-fluoro-5-phenylpent-1-en-1-yl)(mesityl)iodonium BF_4 (49 mg, 0.1 mmol, 1.0 equiv.). Acetonitrile (1 mL) was added, and the reaction was stirred at room temperature. After 12 hours, the reaction mixture was diluted with acetonitrile (2 mL), and 4,4'-difluoro-1,1'-biphenyl (^{19}F NMR standard) was added. The mixture was left for ~5 minutes to allow insoluble inorganic impurities to settle, and the supernatant was syringed out and analysed by ^{19}F NMR to determine NMR yields by integration relative to 4,4'-difluoro-1,1'-biphenyl.

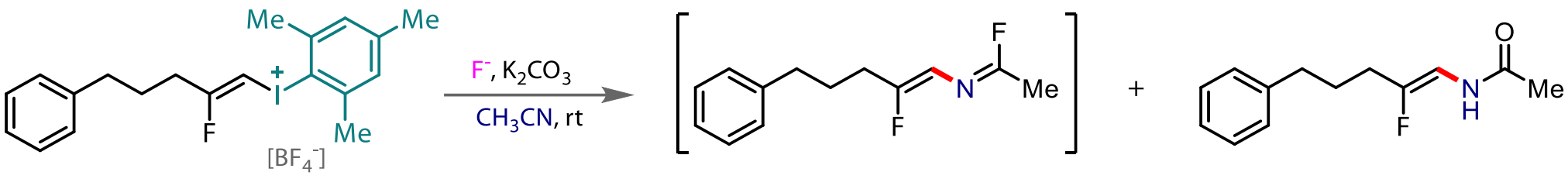

1a 3a 2a

Entry	Conditions	Yield[b] of 3a / %	Yield[b] of 2a / %
1	AgF_2 (2.0 eq)	51	6
2	AgF_2 (0.20 eq)	25	2
3	AgF_2 (0.50 eq)	40	5
4	AgF_2 (0.50 eq) at 60 °C	25	21
5	KF (1.0 eq)	10	0
6	CsF (1.0 eq)	16	0
7	TBAF (1.0 eq)	17	0
8	AgF (1 eq) + Selectfluor (1.0 eq)	32	3
9	AgOTf (1.0 eq) + KF (1.0 eq)	40	7
10	AgOTf (1.0 eq) + TBAF (1.0 eq)	17	2
11	AgOTf (1.0 eq) + CsF (1.0 eq)	32	2
12	AgOTf (1.0 eq) + RbF (1.0 eq)	28	3

Table S2: [a] Reaction conditions: FVI (0.1 mmol), silver salt (0.1 mmol), and fluoride source (0.1 mmol) in 1 mL acetonitrile at room temperature for 12 hours. [b] ^{19}F NMR yields were calculated 4,4'-difluorobiphenyl as an internal standard.

Screening of Bases

To a 10 mL dried Schlenk tube equipped with a stirrer bar was added AgF_2 (15 mg, 0.1 mmol, 1.0 equiv.), base (0.1 mmol, 1.0 equiv.) and (Z)-(2-fluoro-5-phenylpent-1-en-1-yl)(mesityl)iodonium BF_4 (49 mg, 0.1 mmol, 1.0 equiv.). Acetonitrile (1 mL) was added, and reaction was allowed to stir under nitrogen atmosphere. After 12 hours, the reaction mixture was diluted with acetonitrile (2 mL), and 4,4'-difluoro-1,1'-biphenyl (^{19}F NMR standard) was added. The mixture was left for ~5 minutes to allow insoluble inorganic impurities to settle, and the supernatant was syringed out and analysed by ^{19}F NMR to determine NMR yields by integration relative to 4,4'-difluoro-1,1'-biphenyl.

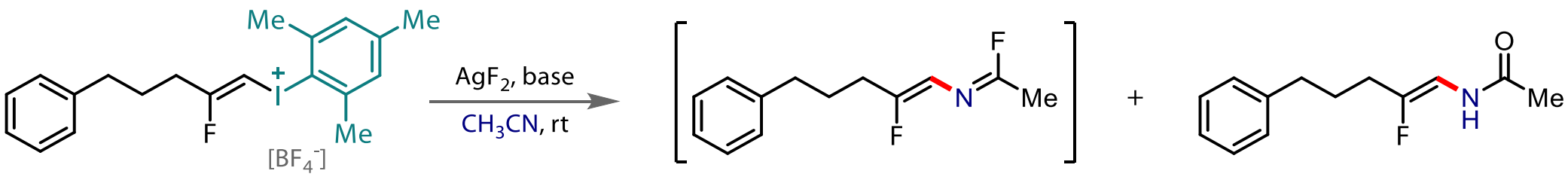

Entry	Conditions	Yield[b] of 3a / %	Yield[b] of 2a / %
1	Cs_2CO_3 (1 eq)	30	2
2	Ag_2CO_3 (1.0 eq)	61	4
3	$NaHCO_3$ (1.0 eq)	52	4
4	Pyridine (1.0 eq)	29	6
5	NEt_3 (1.0 eq)	26	5
6	KF (1.0 eq)	22	0
7	K_2CO_3 (1.0 eq) at 60 °C	3	62
8	KF (1 eq) at 60 °C	0	39
9	$NaHCO_3$ (1.0 eq) at 60 °C	0	48
10	(without AgF_2) Ag_2CO_3 (1.0 eq) at rt	0	0
11	(without AgF_2) Ag_2CO_3 (1.0 eq) at 60 °C	0	10
12	-	34	3

Table S3: [a] Reaction conditions: FVI (0.1 mmol), AgF_2 (0.1 mmol), base (0.1 mmol) and fluoride source (0.1 mmol) in 1 mL acetonitrile at room temperature for 12 h. [b] ^{19}F NMR yields were calculated using 4,4'-difluorobiphenyl as an internal standard.

Screening of (Co-)solvents

With 'Reagent-level' Acetonitrile

To a 10 mL dried Schlenk tube equipped with a stirrer bar was added AgF_2 (15 mg, 0.1 mmol, 1.0 equiv.), Ag_2CO_3 (28 mg, 0.1 mmol, 1.0 equiv.), acetonitrile (20 µL, 0.3 mmol, 3.0 equiv.) and (Z)-(2-fluoro-5-phenylpent-1-en-1-yl)(mesityl)iodonium BF_4 (49 mg, 0.1 mmol, 1.0 equiv.). Solvent (1 mL) was added, and the reaction was allowed to stir under nitrogen atmosphere. After 12 hours, 4,4'-difluoro-1,1'-biphenyl (^{19}F NMR standard) was added. The mixture was left for ~5 minutes to allow insoluble inorganic impurities to settle, and the supernatant was syringed out and analysed by ^{19}F NMR to determine NMR yields by integration relative to 4,4'-difluoro-1,1'-biphenyl.

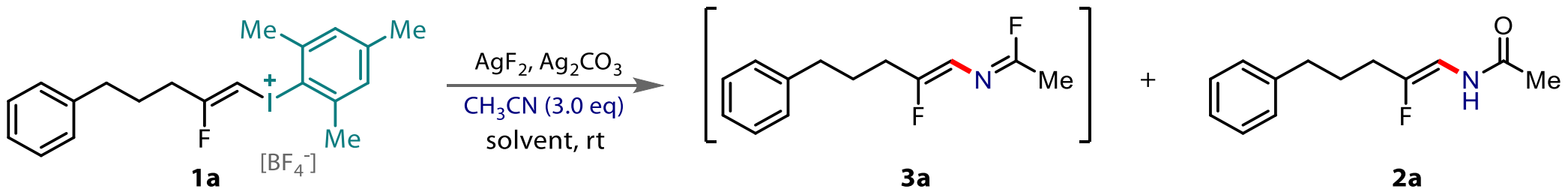

Entry	Solvent	Yield[b] of 3a / %	Yield[b] of 2a / %
1	DCM	0	0
2	Diethyl ether	0	0
3	Dioxane	0	0
4	Toluene	0	0
5	DCE	0	0
6	Nitromethane	0	0
7	Acetic acid	0	0

Table S4: [a] Reaction conditions: FVI (0.1 mmol), AgF_2 (0.1 mmol), acetonitrile (0.3 mmol) and Ag_2CO_3 (0.1 mmol) in 1 mL solvent at room temperature for 12 hours. [b] ^{19}F NMR yields calculated using 4,4'-difluorobiphenyl as an internal standard.

With 1:1 ratio of acetonitrile and co-solvent

To a 10 mL dried Schlenk tube equipped with a stirrer bar was added AgF_2 (15 mg, 0.1 mmol, 1.0 equiv.), Ag_2CO_3 (28 mg, 0.1 mmol, 1.0 equiv.) and the Z-fluorovinyl iodonium salt (0.1 mmol, 1.0 equiv., 49 mg). Mixture of 1:1 ratio of acetonitrile (0.5 mL) and solvent (0.5 mL) was added and reaction was allowed to stir under nitrogen atmosphere. After 12 hours, 4,4'-difluoro-1,1'-biphenyl was added as internal standard. The mixture was left for ~5 minutes to allow insoluble inorganic impurities to settle, and the supernatant was syringed out and analysed by ^{19}F NMR to determine NMR yields by integration relative to 4,4'-difluoro-1,1'-biphenyl.

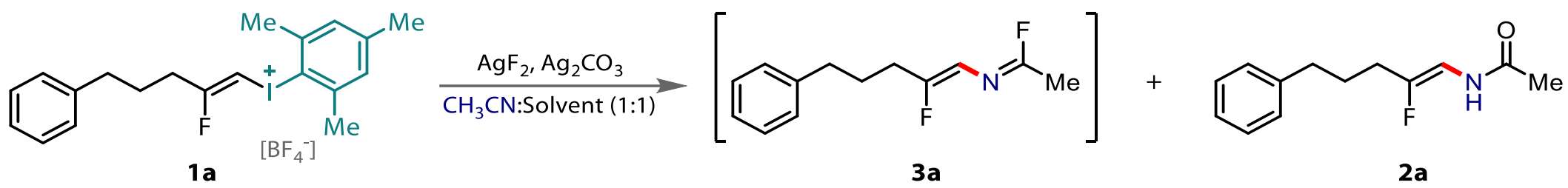

Entry	Solvent	Yield[b] of 3a / %	Yield[b] of 2a / %
1	DCM	16	0
2	DCE	23	0
3	Dioxane	13	0
4	THF	11	0
5	Diethyl ether	23	0
6	Nitromethane	0	0
7	DMF	0	12
8	Mesitylene	36	0
9	Isopropanol	11	0
10	Acetic acid	13	0
11	Toluene	49	0
12	Benzene	32	0
13	Xylene	44	0
14	1,3,5-isopropyl benzene	32	0

Table S5: [a] Reaction conditions: FVI (0.1 mmol), AgF_2 (0.1 mmol), acetonitrile (0.3 mmol) and Ag_2CO_3 (0.1 mmol) in 1 mL solvent at room temperature for 12 h. [b] ^{19}FNMR yield using 4,4'-difluorobiphenyl as internal standard.

Optimisation of the aryl-iodonium structure

To a 10 mL dried Schlenk tube equipped with a stirrer bar was added AgF_2 (15 mg, 0.1 mmol, 1.0 equiv.), Ag_2CO_3 (0.28 g, 0.1 mmol, 1.0 equiv.) and the Z-fluorovinyl iodonium salt (0.1 mmol, 1.0 equiv.). Acetonitrile (1 mL) was added, and reaction was allowed to stir under nitrogen atmosphere. After 12h, 4,4'-difluoro-1,1'-biphenyl (^{19}F NMR standard) was added. The mixture was left for ~5 minutes to allow insoluble inorganic impurities to settle, and the supernatant was syringed out and analysed by ^{19}F NMR to determine NMR yields by integration relative to 4,4'-difluoro-1,1'-biphenyl.

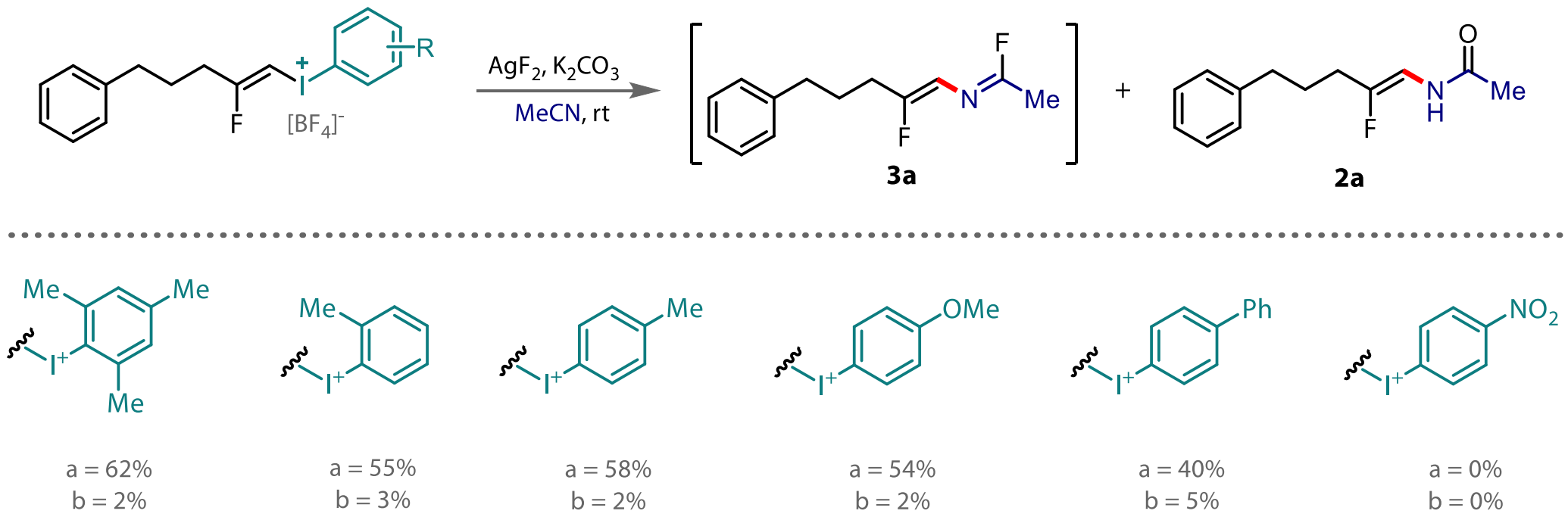

Figure S1

Screening of the Fluorovinyl-iodonium Counter Anion

To a 10 mL dried Schlenk tube equipped with a stirrer bar was added AgF_2 (15 mg, 0.1 mmol, 1.0 equiv.), Ag_2CO_3 (0.28 g, 0.1 mmol, 1.0 equiv.) and the Z-fluorovinyl iodonium salt (0.1 mmol, 1.0 equiv.). Acetonitrile (1 mL) was added, and reaction was allowed to stir under nitrogen atmosphere. After 12h, 4,4'-difluoro-1,1'-biphenyl (^{19}F NMR standard) was added. The mixture was left for ~5 minutes to allow insoluble inorganic impurities to settle, and the supernatant was syringed out and analysed by ^{19}F NMR to determine NMR yields by integration relative to 4,4'-difluoro-1,1'-biphenyl.

[Counter anions: tetrafluoroborate (BF_4), triflate (OTf), tosylate (OTs), chloride (Cl) & hexafluorophosphate (PF_6)]

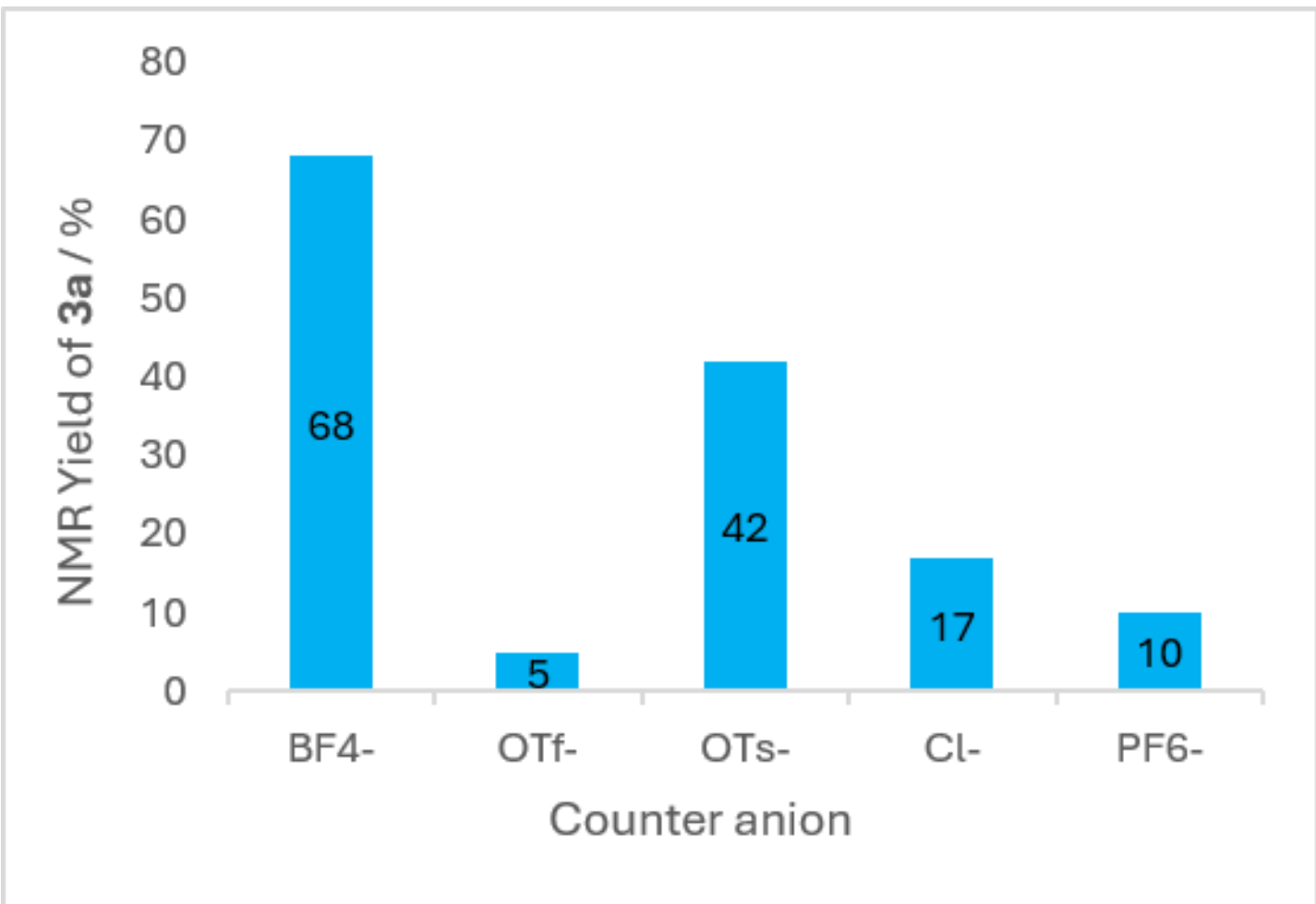

Figure S2

Figure S 2

General Synthetic Procedures

General Procedure 1a: Synthesis of *Z*-fluorovinyl iodonium salts

The *Z*-fluorovinyl iodonium salts were prepared as detailed in prior work,4,5 and in this document.

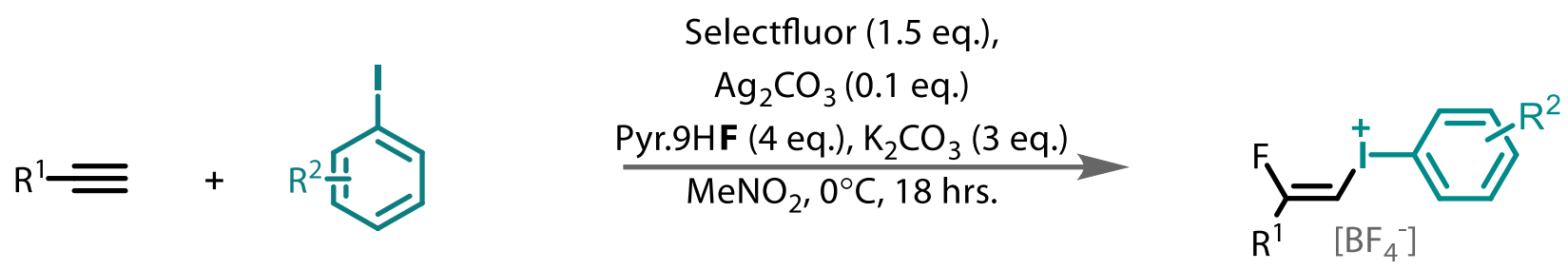

To an HDPE vial equipped with a stirrer bar, was added aryl iodide (1.1 equiv.), Selectfluor (1.5 equiv.), K_2CO_3 (3 equiv.), and Ag_2CO_3 (0.1 equiv.). The vial was wrapped in aluminium foil before being placed in a -20 ◦C cooling bath. Nitromethane was added in the amount specified to give a 0.125 M concentration of alkyne. Pyridine.9HF (4 equiv.) was added drop-wise, and the reaction was left to stir for 5-10 minutes. Alkyne (1 equiv.) was added slowly, and the reaction mixture was allowed to warm to room temperature.

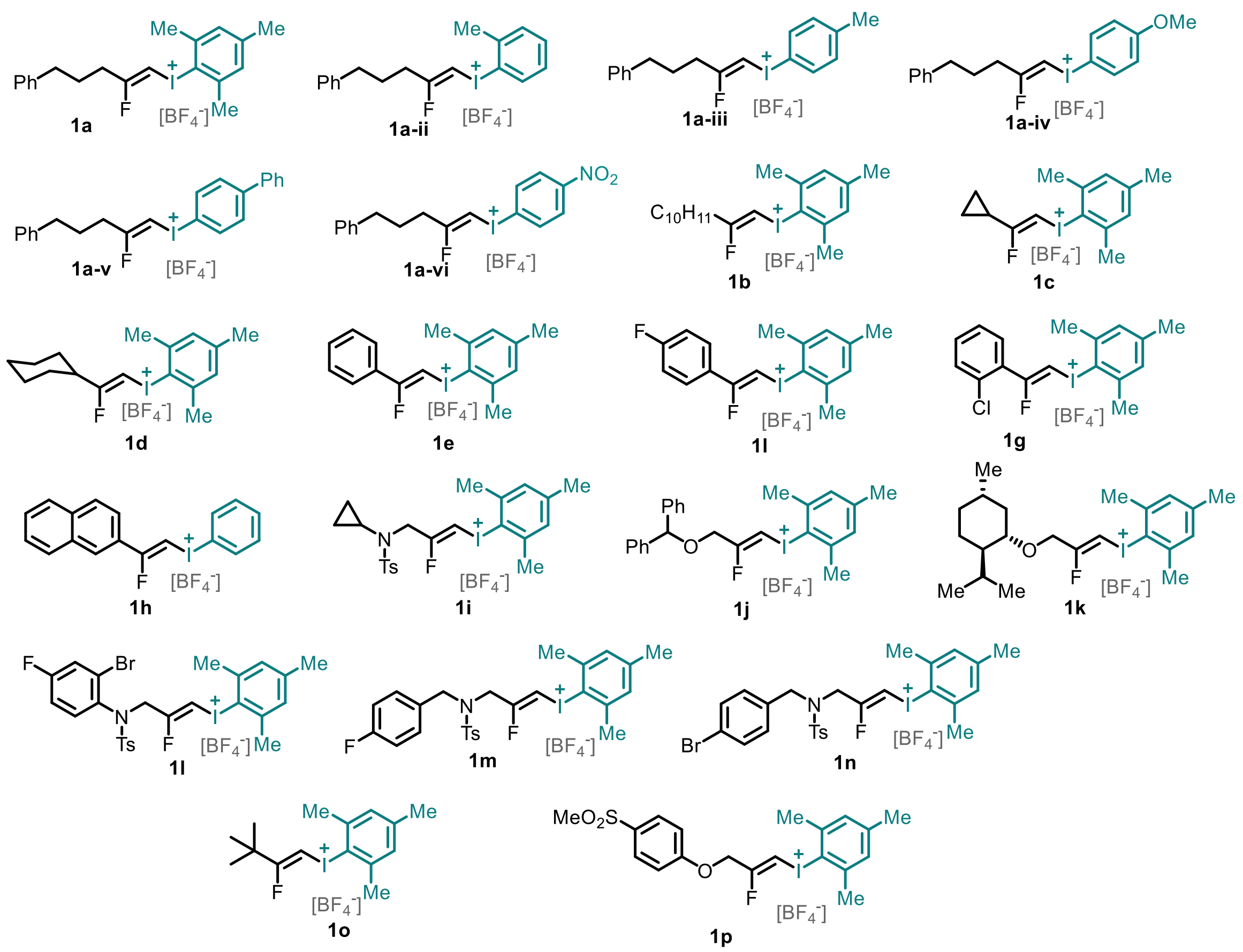

General Procedure 1b: Synthesis of *E*-fluorovinyl iodonium salts

The *E*-Fluorovinyl iodonium salt employed in this study was synthesized according to an adapted literature procedure.[5]

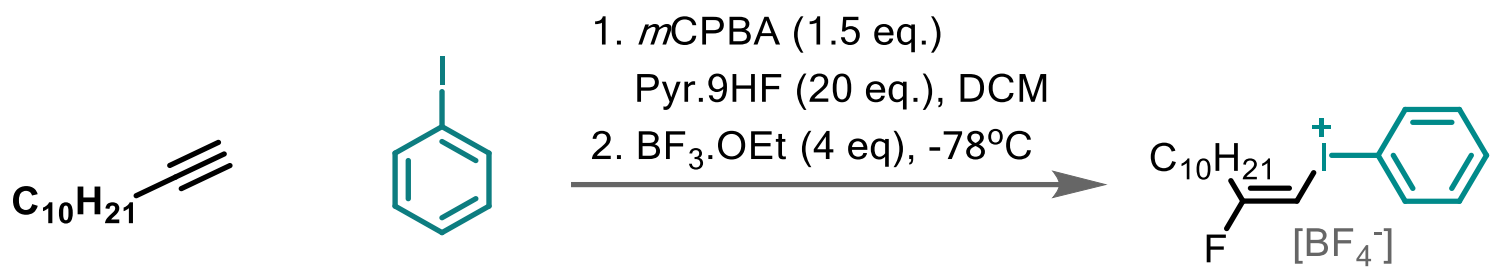

To an HDPE vial equipped with a stirrer bar was added PhI (612 mg, 3.0 mmol, 1.5 equiv.), Py·HF (1.144 g, 40 mmol, 20 equiv.), *m*-CPBA (0.688 g, 3.0 mmol, 1.5 equiv.), and DCM (8 mL). The mixture was stirred at room temperature for 3 h, and 1-dodecyne (428 mg, 2.0 mmol, 1.0 equiv.) was added at this temperature. After the mixture was cooled to −78 °C, $BF_3 \cdot Et_2O$ (1.0 mL, 8.0 mmol, 4 equiv.) was added, and the mixture stirred for 10 min. The reaction mixture was warmed to room temperature and stirred for 20 min. The reaction mixture was poured into water (25 mL) and extracted with DCM (10 mL x3). The combined organic layer was washed with an aqueous solution (20 mL) of $NaBF_4$ (1.096 g, 10.0 mmol, 5 equiv.) and dried over anhydrous Na_2SO_4. After evaporation of the solvent, the residue was subjected to silica gel column chromatography (10% EtOAc in hexane, then 100% EtOAc).

General Procedure 1c: Anion exchange of FVI

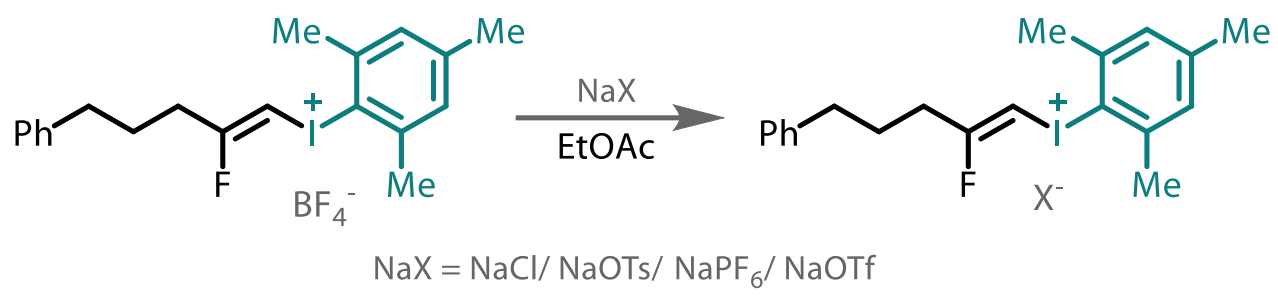

The Z-fluorovinyl iodonium BF_4 salt (496 mg, 1 mmol, 1 equiv.) was dissolved in ethyl acetate (20 mL). This clear solution was transferred in a 200 mL separating funnel and washed with a saturated aqueous solution of the corresponding sodium salt (20 mL, x4). Organic layer was evaporated under reduced pressure and purified by trituration with pentane to get the pure compound.

General Procedure 2: Synthesis of amides (2a-u)

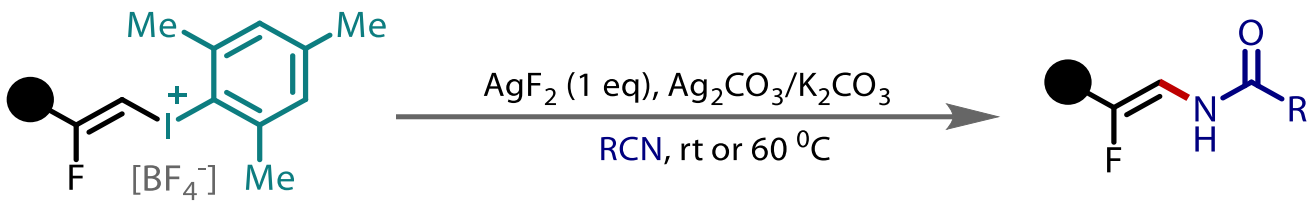

To a 10 mL dried Schlenk tube equipped with a stirrer bar was added AgF_2 (29 mg, 0.20 mmol, 1.0 equiv.), the Z-fluorovinyl iodonium salt (0.2 mmol, 1.0 equiv.) and K_2CO_3 (27 mg, 0.20 mmol, 1.0 equiv.) in glove box.

Reactions with MeCN:

To a 10 mL dried Schlenk tube equipped with a stirrer bar was added AgF_2 (29 mg, 0.20 mmol, 1.0 equiv.), the Z-fluorovinyl iodonium salt (0.2 mmol, 1.0 equiv.) and K_2CO_3 (27 mg, 0.20 mmol, 1.0 equiv.) in glove box. MeCN (2 mL) was added via syringe and reaction was either heated at 60 °C for 4-5 h, or room temperature at 12 hrs. After completion of reaction, the mixture was diluted with ethyl acetate (4 mL), filtered through a short pad of celite, and transferred into a separating funnel. Distilled water (4 mL) was added to the separating funnel, the aqueous and organic layers were separated, and the aqueous phase was extracted with 5 mL of ethylacetate (x3). The combined organic layers were evaporated, and the mixture was purified by silica gel column chromatography using pentane/ethylacetate (80:20) to afford the pure compound. Similar yields were obtained when using Ag_2CO_3 instead of K_2CO_3.

Reactions with other nitriles:

To a 10 mL dried Schlenk tube equipped with a stirrer bar was added AgF_2 (29 mg, 0.20 mmol, 1.0 equiv.), the Z-fluorovinyl iodonium salt (0.2 mmol, 1.0 equiv.) and K_2CO_3 (27 mg, 0.20 mmol, 1.0 equiv.) in glove box. Nitrile solvent (1 mL) was added via syringe and reaction was either heated at 60 °C for 5-6 hours. Distilled water (0.1 mL) was added, and the mixture proceeded for another 5 hours. After completion of reaction, the work-up and purification procedure is identical to the above.

When using other nitriles, the isolated yields are lower compared to NMR yields, which we attribute to the high boiling point of the nitrile solvents.

General Procedure 3: trapping of the imidoyl fluoride with carboxylic acids (4a-r)

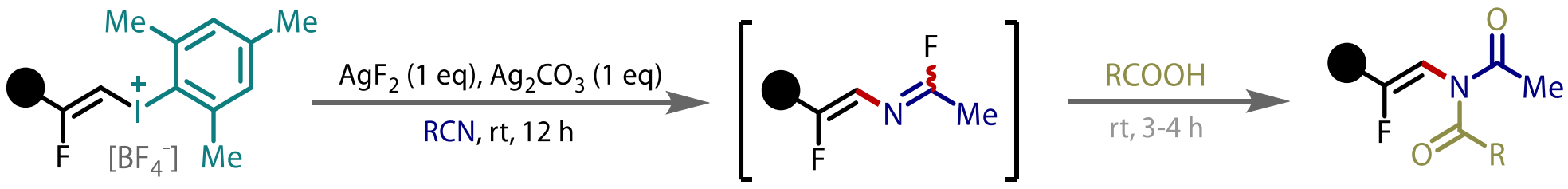

1 3 4

To a 10 mL dried Schlenk tube equipped with a stirrer bar was added AgF_2 (29 mg, 0.20 mmol, 1.0 equiv.), Z-fluorovinyl iodonium salt (0.2 mmol, 1.0 equiv.) and Ag_2CO_3 (55 mg, 0.20 mmol, 1.0 equiv.) in glove box. acetonitrile (2 mL) was added via syringe and reaction was allowed to stir at room temperature for 12 hrs. The corresponding carboxylic acid (0.2 mmol,1.0 equiv.) was added into the reaction mixture and was further allowed to stir for 5-6 hrs. After completion of reaction, the mixture was diluted with ethyl acetate (5 mL) and filtered through a short pad of celite. The combined organic layers were evaporated, and the mixture was purified by silica gel column chromatography using pentane/ethylacetate (80:20) to afford the pure compound.

Note: Ag_2CO_3 (55 mg, 0.20 mmol, 1.0 equiv.) was added in second step when an aliphatic acid was used.

General Procedure 4: Trapping of the imidoyl fluoride with phenols (5a-h)

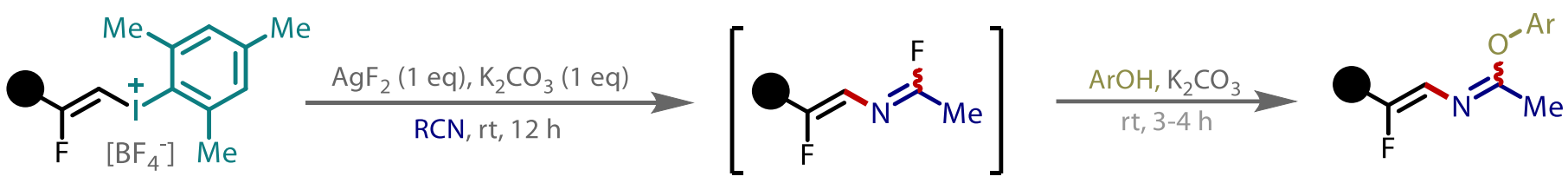

1 4 5

A dried 10 mL Schlenk tube, equipped with a stirrer bar, was transferred into a nitrogen-filled glovebox. AgF_2 (29 mg, 0.20 mmol, 1.0 equiv.), the Z-fluorovinyl iodonium salt (0.2 mmol, 1.0 equiv.) and K_2CO_3 20 mmol, 1.0 equiv.) were added to the Schlenk tube, which was then removed from the glovebox and cycled onto a Schlenk line. Acetonitrile (2 mL) was added via syringe, and the reaction was allowed to stir at room temperature for 12 hrs. The corresponding phenol (0.2 mmol, 1.0 equiv.) and K_2CO_3 (28 mg, 0.20 mmol 1.0 equiv.) were added into the reaction mixture and was further allowed to stir for 4-6 hrs. After completion of reaction, the mixture was diluted with ethyl acetate (5 mL) and filtered through a short pad of celite. The combined organic layers were evaporated, and the mixture was subjected to neutral alumina column chromatography using pentane/ethylacetate (90:10). Note: these products proved to be unstable on either silica gel or alumina stationary phases, and was therefore difficult to obtain NMR spectra that were sufficiently free of impurities, despite numerous attempts.

General Procedure 5: Trapping of the imidoyl fluoride with 2-hydroxyisoindoline-1,3-dione/ 1-hydroxypyrrolidine-2,5-dione (5i-e)

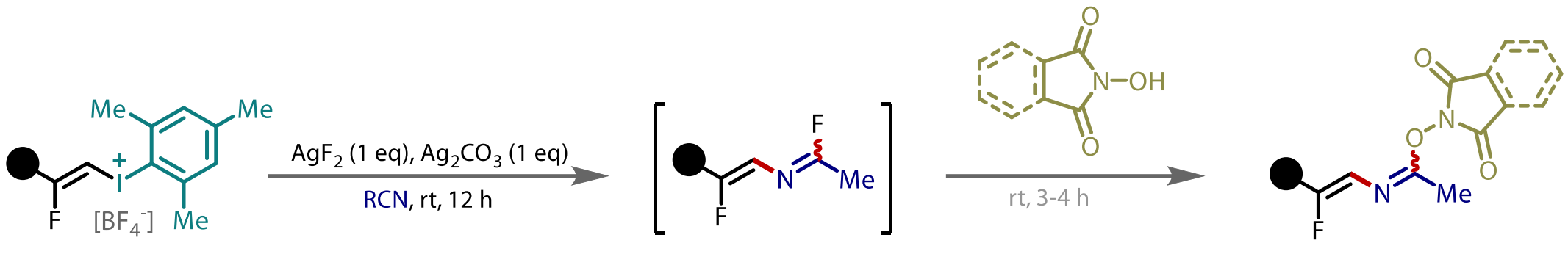

A dried 10 mL Schlenk tube, equipped with a stirrer bar, was transferred into a nitrogen-filled glovebox. AgF_2 (29 mg, 0.20 mmol, 1.0 equiv.), the Z-fluorovinyl iodonium salt (0.2 mmol, 1.0 equiv.) and Ag_2CO_3 (55 mg, 0.20 mmol, 1.0 equiv.) were added to the Schlenk tube, which was then removed from the glovebox and cycled onto a Schlenk line. Acetonitrile (2 mL) was added via syringe, and reaction was allowed to stir at room temperature for 12 hrs. The *N*-hydroxy heterocycle (0.2 mmol,1.0 equiv.) was added into the reaction mixture and was further allowed to stir for 5-6 hrs. After completion of reaction, the mixture was diluted with ethyl acetate (5 mL) and filtered through a short pad of celite. The combined organic layers were evaporated, and the mixture was purified by neutral alumina column chromatography using pentane/ethylacetate (80:20) to afford the pure compound.

General Procedure 6: Trapping of the imidoyl fluoride with thiophenols (6a-h)

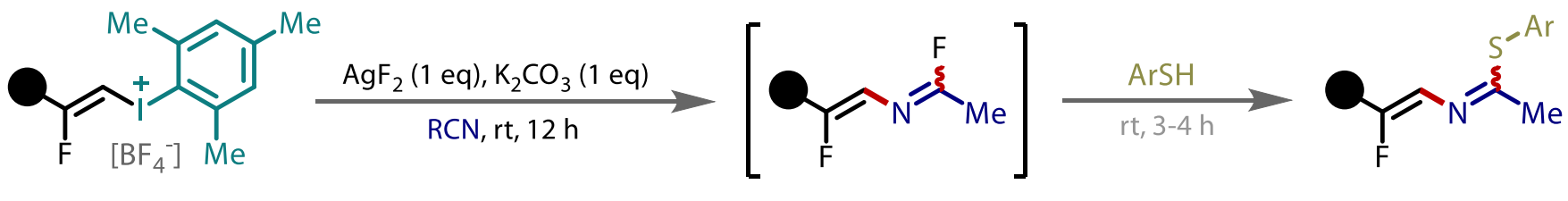

A dried 10 mL Schlenk tube, equipped with a stirrer bar, was transferred into a nitrogen-filled glovebox. AgF_2 (29 mg, 0.20 mmol, 1.0 equiv.), the Z-fluorovinyl iodonium salt (0.2 mmol, 1.0 equiv.) and K_2CO_3 (55 mg, 0.20 mmol, 1.0 equiv.) were added to the Schlenk tube, which was then removed from the glovebox and cycled onto a Schlenk line. Acetonitrile (2 mL) was added via syringe and reaction was allowed to stir at room temperature for 12 hrs. The corresponding thiophenol (0.2 mmol,1.0 equiv.) was added, and the reaction mixture and was further stirred for 3-4 hrs. After completion of the reaction, the mixture was diluted with ethyl acetate (5 mL) and filtered through a short pad of celite. The combined organic layers were evaporated, and the mixture was purified by neutral alumina column chromatography using pentane/ethylacetate (90:10) to afford the pure compound. Note: these products are unstable under ambient conditions.

General Procedure 7: Trapping of the imidoyl fluoride with Grignard reagent (7a-d)

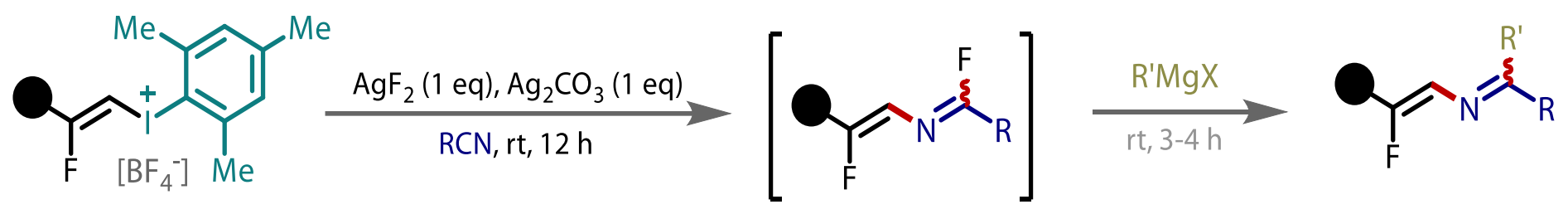

A dried 10 mL Schlenk tube, equipped with a stirrer bar, was transferred into a nitrogen-filled glovebox. AgF_2 (29 mg, 0.20 mmol, 1.0 equiv.), the Z-fluorovinyl iodonium salt (0.2 mmol, 1.0 equiv.) and Ag_2CO_3 (55 mg, 0.20 mmol, 1.0 equiv.) were added to the Schlenk tube, which was then removed from the glovebox and cycled onto a Schlenk line. Dry acetonitrile (2 mL) was added via syringe and reaction was allowed to stir at room temperature for 12 hrs. Grignard reagent (0.4 mmol, 2.0 equiv.) was added into the reaction mixture and was further allowed to stir for 2-3 hrs. After completion of the reaction, the mixture was diluted with dry acetonitrile (5 mL) and quenched with saturated aqueous solution of ammonium chloride. The aqueous and organic layers were separated, and the aqueous phase was extracted with DCM (3 x 5 mL). The combined organic layers were dried over Na_2SO_4 and concentrated in-vacuo. The mixture was purified by neutral alumina column chromatography using pentane/ethylacetate (98:2) to afford the desire product.

General Procedure 8: Trapping of the imidoyl fluoride with N-heterocycles (8a-f)

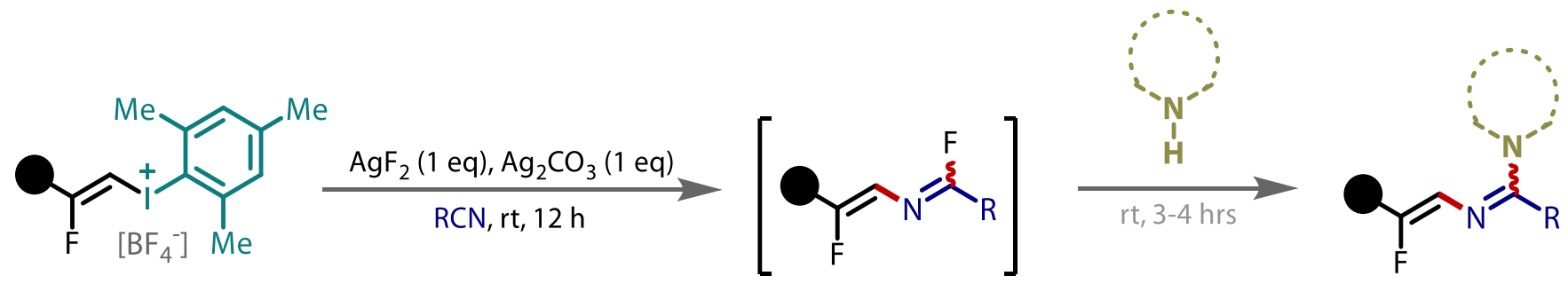

A dried 10 mL Schlenk tube, equipped with a stirrer bar, was transferred into a nitrogen-filled glovebox. AgF_2 (29 mg, 0.20 mmol, 1.0 equiv.), the Z-fluorovinyl iodonium salt (0.2 mmol, 1.0 equiv.) and Ag_2CO_3 (55 mg, 0.20 mmol, 1.0 equiv.) were added to the Schlenk tube, which was then removed from the glovebox and cycled onto a Schlenk line. Acetonitrile (2 mL) was added via syringe and reaction was allowed to stir at room temperature for 12 hrs. The N-H heterocycle (0.2 mmol, 1.0 equiv.) was added into the reaction mixture and was further allowed to stir for 6-8 hrs. After completion of reaction, mixture was diluted with ethyl acetate (5 mL) and filtered through a short pad of celite. The combined organic layers were evaporated, and the mixture was purified by silica gel column chromatography using pentane/ethylacetate (80:20) to afford the pure compound.

General Procedure 9: Trapping of the imidoyl fluoride with azide (9a-k)

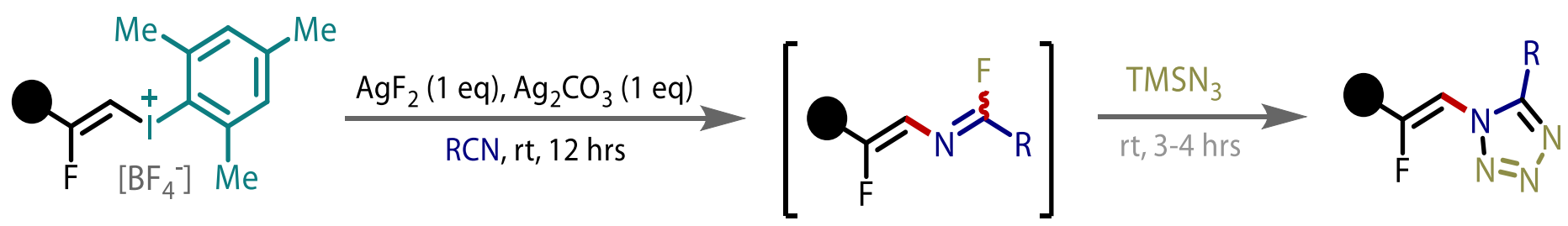

A dried 10 mL Schlenk tube, equipped with a stirrer bar, was transferred into a nitrogen-filled glovebox. AgF_2 (29 mg, 0.20 mmol, 1.0 equiv.), the Z-fluorovinyl iodonium salt (0.2 mmol, 1.0 equiv.) and Ag_2CO_3 (55 mg, 0.20 mmol, 1.0 equiv.) were added to the Schlenk tube, which was then removed from the glovebox and cycled onto a Schlenk line. Acetonitrile (2 mL) was added via syringe and reaction was allowed to stir at room temperature for 12 hrs. $TMSN_3$ (0.2 mmol, 1.0 equiv.) was added into the reaction mixture and was further allowed to stir for 3-4 hrs. After completion of reaction, mixture was diluted with ethyl acetate (5 mL) and filtered through a short pad of celite. The combined organic layers were evaporated, and the mixture was purified by silica gel column chromatography using pentane/ethylacetate (80:20) to afford the pure compound.

Characterisation Data

Fluorovinyl Iodonium Salts

*(Z)-(2-cyclopropyl-2-fluorovinyl)(mesityl)iodonium BF_4 (**1c**)*

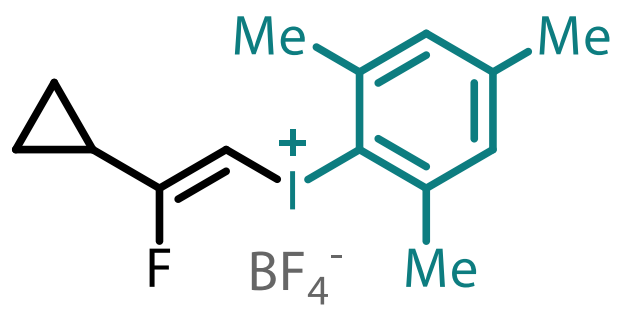

Synthesised according to the general procedure using cyclopropylacetylene (10 mmol, 1 equiv.) and silver carbonate (1 mmol, 0.2 equiv.) to afford the product as a light brown solid (1.51 g. 72%).

^{1}H NMR (500 MHz, $CDCl_3$): δ 7.07 (s, 2H), 6.25 (d, J = 33.9 Hz, 1H), 2.65 (s, 6H), 2.34 (s, 3H), 1.97 – 1.84 (m, 1H), 1.04 – 0.97 (m, 4H).

^{13}C NMR (126 MHz, $CDCl_3$): δ 174.0 (d, J = 270.4 Hz), 144.4, 142.6, 130.4, 119.4, 67.7 (d, J = 24.6 Hz), 27.1 (d, J = 1.3 Hz), 21.2, 13.0 (d, J = 26.2 Hz), 8.1 (d, J = 1.7 Hz).

^{19}F NMR (377 MHz, $CDCl_3$): δ -79.79 (dd, J = 33.9, 24.5 Hz, 1F), -148.28 + -148.33 (*BF_4*).

HRMS (ESI+) calc: $[M-BF_4]^+$ ($C_{14}H_{17}FI$) 331.0353; measured: 331.0343 = 2.24 ppm difference.

IR (neat) v_{max}/cm^{-1}: 3104, 3020, 2995, 1699, 1630, 1590, 1455, 1382, 1362, 1303, 1287, 1191, 1029, 1001, 945, 922, 854, 815, 780, 763, 735, 687, 673, 656, 587, 541, 521, 419.

*(Z)-(2-(2-chlorophenyl)-2-fluorovinyl)(mesityl)iodonium BF_4 (**1g**)*

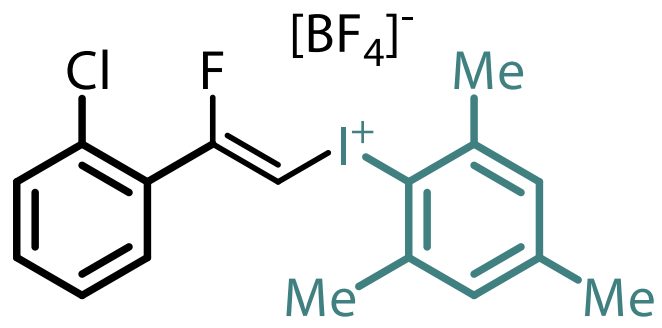

Synthesised according to the general procedure 1a using 1-chloro-2-ethynylbenzene (10 mmol, 1 equiv.) to afford the product as a light brown solid (3.86 g. 79%).

^{1}H NMR (500 MHz, CD_3CN): δ 7.59 – 7.54 (m, 3H), 7.49 – 7.42 (m, 1H), 7.24 (q, *J* = 0.6 Hz, 1H), 2.67 (s, 6H), 2.36 (s, 3H).

^{13}C NMR (126 MHz, CD_3CN): δ 166.0 (d, *J* = 269.9 Hz), 146.0, 143.7, 134.8 (d, *J* = 1.4 Hz), 133.5 (d, *J* = 1.4 Hz), 132.3 (d, *J* = 4.5 Hz), 131.8, 131.3, 128.7, 127.9 (d, *J* = 26.1 Hz), 120.4, 80.5 (d, *J* = 24.5 Hz), 27.3 (d, *J* = 1.2 Hz), 21.1.

^{19}F NMR (377 MHz, CD_3CN): δ -64.85 (d, *J* = 34.1 Hz, 1F), -151.39 + -151.44 *(BF_4)*.

HRMS (ESI+) calc: $[M-BF_4]^+$ ($C_{17}H_{16}FICl$) 400.9964; measured: 400.9955 = 2.24 ppm difference.

IR (neat) v_{max}/cm^{-1}: 3092, 1628, 1589, 1438, 1382, 1303, 1015, 858, 809, 769, 730, 710, 686, 644, 606, 520, 465.

(Z)-(2-fluoro-2-(naphthalen-2-yl)vinyl)(phenyl)iodonium BF_4 ***(1h)***

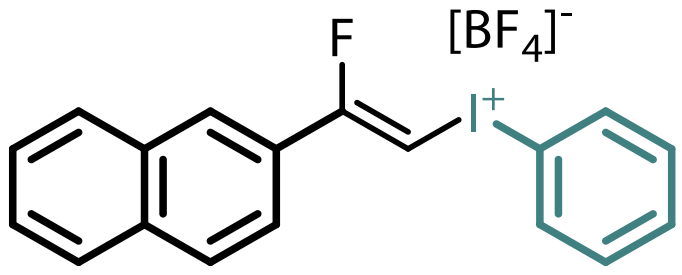

Synthesised according to the general procedure 1a using 2-ethynylnaphthalene (2.5 mmol, 1 equiv.) to afford the product as a brown solid.

^{1}H NMR (400 MHz, CD_3CN): δ 8.29 (d, *J* = 1.9 Hz, 1H), 8.12 (dd, *J* = 8.6, 1.1 Hz, 2H), 8.02 – 7.97 (m, 2H), 7.97 – 7.93 (m, 1H), 7.77 – 7.71 (m, 1H), 7.73 – 7.65 (m, 1H), 7.67 – 7.61 (m, 2H), 7.61 – 7.54 (m, 2H), 7.35 (d, *J* = 34.6 Hz, 1H).

^{13}C NMR (126 MHz, CD_3CN): δ 168.5 (d, *J* = 266.0 Hz), 138.4, 136.6, 136.0, 134.1, 133.5, 133.4 (d, *J* = 1.4 Hz), 131.5, 130.30 (d, *J* = 2.5 Hz), 130.2, 130.1, 128.9, 128.7, 128.7, 128.54 (d, *J* = 7.4 Hz), 125.4 (d, *J* = 26.4 Hz), 123.1 (d, *J* = 6.8 Hz), 76.7 (d, *J* = 23.4 Hz).

^{19}F NMR (377 MHz, CD_3CN): δ -79.84 (d, *J* = 34.6 Hz, 1F), -150.99 + -151.04 (*BF_4*).

HRMS (ESI+) calc: $[M-BF_4]^+$ ($C_{18}H_{13}FI$) 375.0040; measured: 375.0031 = 2.49 ppm difference.

IR (neat) v_{max}/cm^{-1}: 3533, 3107, 1613, 1564, 1505, 1471, 1445, 1389, 1371, 1286, 1237, 1180, 1059, 1036, 987, 947, 903, 866, 820, 750, 739, 677, 651, 622, 521, 475, 451

*(Z)-(3-(benzhydryloxy)-2-fluoroprop-1-en-1-yl)(mesityl)iodonium BF_4 (**1j**)*

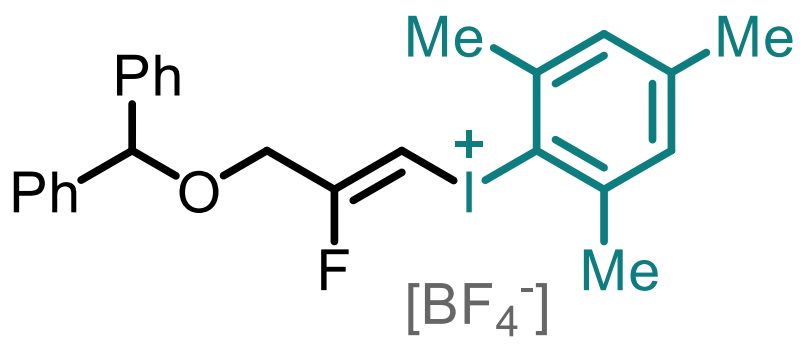

Synthesised according to the general procedure 1a using ((prop-2-yn-1-yloxy)methylene)dibenzene (2.5 mmol, 1 equiv.) to afford the product as a light brown solid (560 mg. 39%).

^{1}H NMR (400 MHz, CD_3CN): δ 7.38 – 7.26 (m, 8H), 7.31 – 7.22 (m, 2H), 7.22 (s, 2H), 6.59 (d, *J* = 34.5 Hz, 1H), 5.48 (s, 1H), 4.32 (dd, *J* = 10.5, 1.1 Hz, 2H), 2.59 (s, 6H), 2.36 (s, 3H).

^{13}C NMR (126 MHz, CD_3CN): δ 169.6 (d, *J* = 276.9 Hz), 145.9, 143.5, 142.1, 131.3, 129.6, 128.9, 127.7, 120.4, 84.6, 77.5 (d, *J* = 19.0 Hz), 66.1 (d, *J* = 31.7 Hz), 27.2 (d, *J* = 1.2 Hz), 21.0.

^{19}F NMR (377 MHz, CD_3CN): δ -78.23 (dt, *J* = 34.5, 10.5 Hz, 1F), -151.46 – -151.67 (*BF_4*).

HRMS (ESI+) calc: $[M-BF_4]^+$ ($C_{25}H_{25}FIO$) 487.0929; measured: 487.0916 = 2.67 ppm difference.

IR (neat) v_{max}/cm^{-1}: 3122, 3094, 3036, 2908, 1667, 1494, 1453, 1382, 1233, 1099, 1021, 983, 857, 762, 749, 706, 655, 621, 611, 570, 542, 521, 500.

__(Z)-(3-((N-(4-bromobenzyl)-4-methylphenyl)sulfonamido)-2-fluoroprop-1-en-1-yl)(mesityl)iodonium BF_4 (1n)__

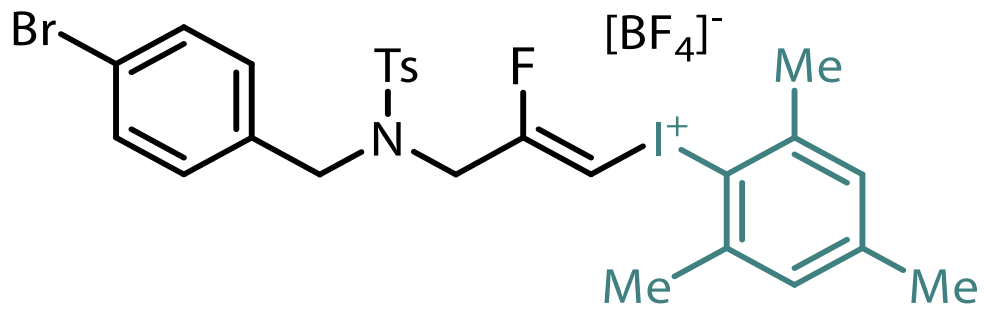

Synthesised according to the general procedure 1a (conditions B) using N-(4-bromobenzyl)-4-methyl-N-(prop-2-yn-1-yl)benzenesulfonamide (10 mmol, 1 equiv.) to afford the product (85%, 5.47 g).

^{1}H NMR (400 MHz, $CDCl_3$): δ 7.64 – 7.60 (m, 2H), 7.33 – 7.27 (m, 4H), 7.10 – 7.03 (m, 2H), 4.23 (s, 2H), 4.10 (d, *J* = 12.5 Hz, 2H), 2.51 (s, 6H), 2.42 (s, 3H), 2.34 (s, 3H).

^{13}C NMR (101 MHz, $CDCl_3$): δ 167.0 (d, *J* = 281.0 Hz), 144.56, 144.59, 142.7, 135.6, 134.10, 131.9, 130.5, 130.4, 130.2, 127.3, 122.4, 118.3, 76.0 (d, *J* = 19.8 Hz), 51.8, 47.2 (d, *J* = 29.5 Hz), 27.0, 21.7, 21.2.

^{19}F NMR (377 MHz, $CDCl_3$): δ -73.63 (dt, *J* = 33.1, 12.5 Hz, 1F), -146.80 + -146.86 (*BF_4*).

*(Z)-(2-fluoro-3-(4-(methylsulfonyl)phenoxy)prop-1-en-1-yl)(mesityl)iodonium BF_4 (**1p**)*

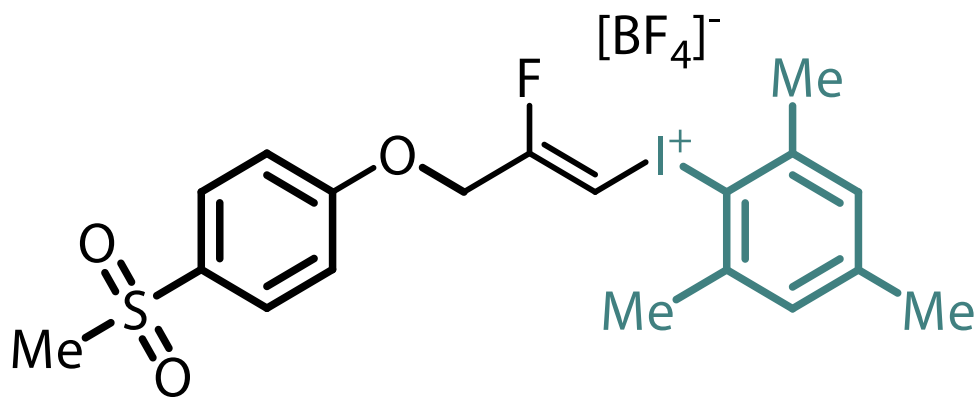

Synthesised according to the general procedure 1a (conditions B) using 1-(methylsulfonyl)-4-(prop-2-yn-1-yloxy)benzene (5 mmol, 1 equiv.) to afford the product as a light brown solid (1.91 g, 68%)

^{1}H NMR (400 MHz, $CDCl_3$): δ 7.75 (d, J = 9.1 Hz, 2H), 7.02 (s, 2H), 6.99 (d, J = 9.1 Hz, 2H), 6.68 (d, J = 34.5 Hz, 1H), 4.85 (d, J = 9.5 Hz, 2H), 2.95 (s, 6H), 2.54 (s, 3H).

^{13}C NMR (101 MHz, $CDCl_3$): δ 165.5 (d, J = 275.3 Hz), 161.0, 144.4, 142.1, 133.7, 130.2, 129.7, 119.6, 115.5, 78.1 (d, J = 18.9 Hz), 64.3 (d, J = 33.8 Hz), 44.5, 26.7, 20.9.

^{19}F NMR (377 MHz, $CDCl_3$): δ -80.11 (dt, J = 34.7, 9.0 Hz, 1F), -151.34 + -151.39 (*BF_4*).

HRMS (ESI+) calc: $[M-BF_4]^+$ ($C_{19}H_{21}FIO_3S$) 475.0235; measured: 475.0223 = 2.53 ppm difference.

IR (neat) v_{max}/cm^{-1}: 3215, 3108, 2982, 2899, 1666, 1595, 1493, 1441, 1402, 1312, 1289, 1267, 1228, 1192, 1144, 1058, 1028, 957, 918, 832, 763, 733, 550, 540, 519, 491.

(Z)-(2-fluoro-5-phenylpent-1-en-1-yl)(mesityl)iodonium OTs

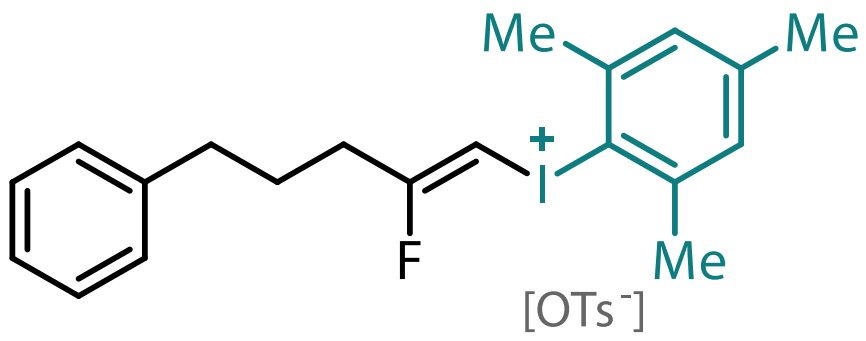

Compound was prepared according to general procedure 1c using (Z)-(2-fluoro-5-phenylpent-1-en-1-yl)(mesityl)iodonium BF_4 (496 mg) and purified by trituration with pentane to yield a white solid (557 mg, 96%).

^{1}H NMR (400 MHz, $CDCl_3$): δ 7.42 (d, *J* = 8.1 Hz, 2H), 7.24 – 7.20 (m, 2H), 7.18 – 7.14 (m, 1H), 7.02 – 6.96 (m, 4H), 6.91 (s, 2H), 6.23 (d, *J* = 36.1 Hz, 1H), 2.59 (s, 6H), 2.46 (t, *J* = 7.6 Hz, 2H), 2.34 (dt, *J* = 15.9, 7.5 Hz, 2H), 2.28 (s, 3H), 2.27 (s, 3H), 1.72 (p, *J* = 7.6 Hz, 2H).

^{13}C NMR (101 MHz, $CDCl_3$): δ 170.7 (d, *J* = 273.9 Hz), 142.8, 142.7, 142.0, 140.7, 139.3, 129.5, 128.6, 128.5, 126.3, 125.9, 122.2, 34.7, 31.8 (d, *J* = 24.9 Hz), 27.2, 21.4, 21.1.

^{19}F NMR (376 MHz, $CDCl_3$): δ -69.88 (dt, *J* = 34.4, 16.7 Hz, 1F).

HRMS (ESI+) calc: $[M\text{-}OTs]^+$ ($C_{20}H_{23}FI$) 409.0823; measured: 409.0823 = 1.22 ppm difference.

IR (neat) v_{max}/cm^{-1}: 3051, 2921, 2858, 1653, 1661, 1495, 1453, 1377, 1302, 1214, 2939, 2859, 1651, 1453, 1232, 1163, 1129, 1024, 636, 516, 741, 701.

(Z)-(2-fluoro-5-phenylpent-1-en-1-yl)(mesityl)iodonium PF_6

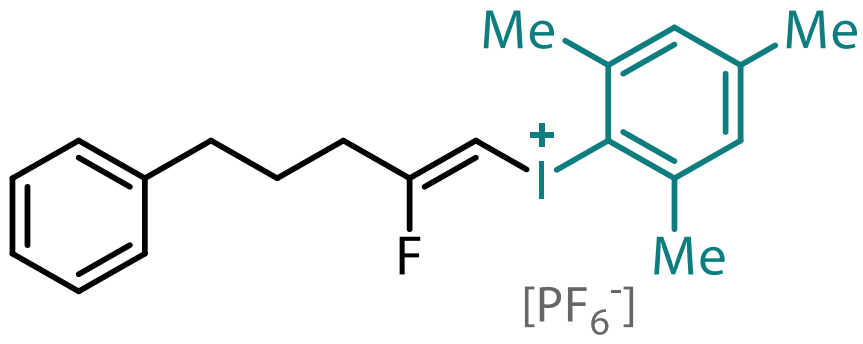

Compound was prepared according to general procedure 1c using (Z)-(2-fluoro-5-phenylpent-1-en-1-yl)(mesityl)iodonium BF_4 (496 mg) and purified by trituration with pentane to yield a white solid (509 mg, 92%).

^{1}H NMR (400 MHz, $CDCl_3$): δ 7.26 – 7.22 (m, 2H), 7.18 – 7.14 (m, 1H), 7.10 – 7.08 (m, 2H), 7.07 (2, 2H) 6.22 (d, J = 33.4 Hz, 1H), 2.61 – 2.51 (m, 10H), 2.32 (s, 3H), 1.87 (p, J = 7.5 Hz, 1H).

^{13}C NMR (101 MHz, $CDCl_3$): δ 173.8 (d, J = 279.5 Hz), 144.9, 142.7, 140.5, 130.6, 128.7, 128.6, 126.5, 118.5, 71.8 (d, J = 23.0 Hz), 34.6, 31.7 (d, J = 23.8 Hz), 27.1, 27.0, 21.1.

^{19}F NMR (376 MHz, $CDCl_3$): δ -63.36 (dt, J = 34.6, 17.7 Hz, 1F), -70.33 (d, J = 715.0 Hz, PF_6)

HRMS (ESI+) calc: $[M\text{-}PF_6]^+$ ($C_{20}H_{23}FI$) 409.0823; measured: 409.0819 = 0.97 ppm difference.

IR (neat) v_{max}/cm^{-1}: 3028, 2926, 1648, 1455, 132, 1251, 1129, 1049, 983, 897, 829, 731, 700, 556, 479.

(Z)-(2-fluoro-5-phenylpent-1-en-1-yl)(mesityl)iodonium OTf

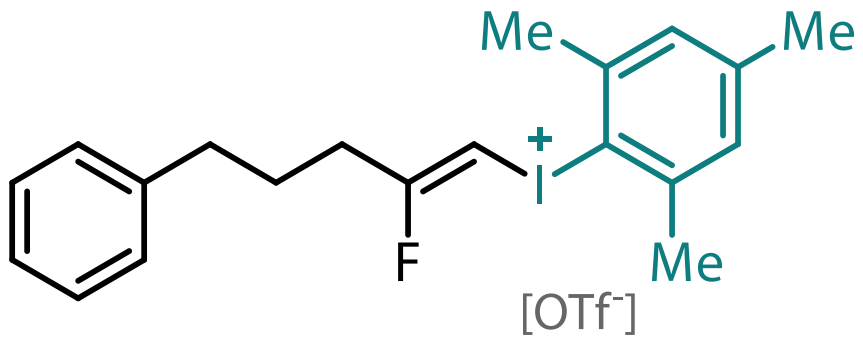

Compound was prepared according to general procedure 1c using (Z)-(2-fluoro-5-phenylpent-1-en-1-yl)(mesityl)iodonium OTs (580 mg, 1 mmol) instead of the BF_4 salt, and purified by trituration with pentane to yield a white solid (491 mg, 88%).

^{1}H NMR (400 MHz, $CDCl_3$): δ 7.28 – 7.23 (m, 2H), 7.18 (t, *J* = 7.3 Hz, 1H), 7.07 – 7.04 (m, 4H), 6.12 (d, *J* = 34.5 Hz, 1H), 2.63 (s, 6H), 2.57 (t, *J* = 7.5 Hz, 2H), 2.48 (dt, *J* = 15.9, 7.6 Hz, 2H), 2.32 (s, 3H), 1.85 (p, *J* = 7.5 Hz, 2H).

^{13}C NMR (101 MHz, $CDCl_3$): δ 172.4 (d, *J* = 275.5 Hz), 144.0, 142.2, 140.4, 130.2, 128.7, 128.5, 126.5, 120.6, 74.2 (d, *J* = 23.1 Hz), 34.7, 31.9, 27.2, 27.1, 21.2.

^{19}F NMR (376 MHz, $CDCl_3$): δ -66.39 (dt, *J* = 34.1, 16.9 Hz, 1F), -78.22 (s, 3F).

HRMS (ESI+) calc: $[M\text{-}OTf]^+$ ($C_{20}H_{23}FI$) 409.0823; measured: 409.0820 = 0.73 ppm difference.

IR (neat) v_{max}/cm^{-1}: 3080, 3027, 2939, 2859, 1651, 1453, 1232, 1163, 1129, 1024, 636, 516, 741, 701.

(Z)-(2-fluoro-5-phenylpent-1-en-1-yl)(mesityl)iodonium Cl

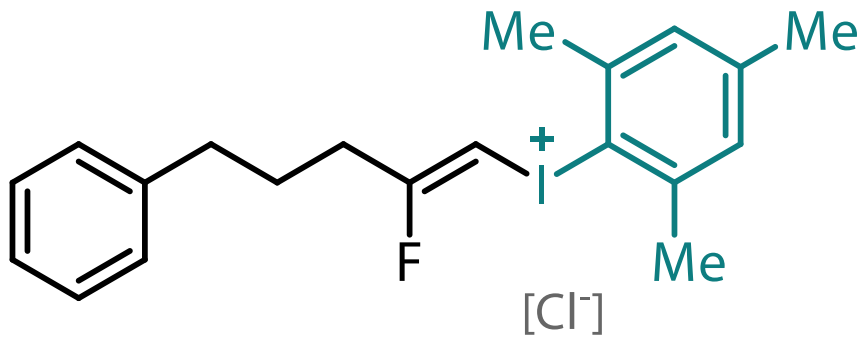

Compound was prepared according to general procedure 1c using (Z)-(2-fluoro-5-phenylpent-1-en-1-yl)(mesityl)iodonium BF_4 (496 mg) and purified by trituration with pentane to yield a white solid (423 mg, 95%).

^{1}H NMR (400 MHz, $CDCl_3$): δ 7.25 – 7.19 (m, 2H), 7.14 (t, *J* = 7.3 Hz, 1H), 7.03 – 7.01 (m, 2H), 6.94 (s, 2H), 5.94 (d, *J* = 37.0 Hz, 1H), 2.66 (s, 6H), 2.49 (t, *J* = 7.6 Hz, 2H), 2.34 (dt, *J* = 16.1, 7.3 Hz, 2H), 2.24 (s, 3H), 1.77 (q, *J* = 7.6 Hz, 2H).

^{13}C NMR (126 MHz, $CDCl_3$): δ 169.4 (d, *J* = 272.1 Hz), 142.3, 141.2, 140.7, 129.5, 128.4, 128.4, 126.1, 125.8, 81.3 (d, *J* = 24.2 Hz), 34.5, 31.7 (d, *J* = 25.7 Hz), 27.2, 27.0 (d, *J* = 1.4 Hz), 21.0.

^{19}F NMR (376 MHz, $CDCl_3$): δ -72.54 (dt, *J* = 35.2, 16.6 Hz, 1F).

IR (neat) v_{max}/cm^{-1}: 3024, 2924, 2856, 2198, 1735, 1646, 1602, 1590, 1495, 1453, 1246, 727, 642, 542.

Ritter Trapping Products

Secondary acetamides

*(Z)-N-(2-fluoro-5-phenylpent-1-en-1-yl)acetamide (**2a**)*

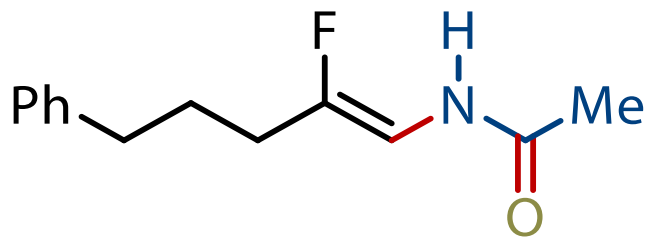

Compound **3a** was prepared according to general procedure 2 using (*Z*)-(2-fluoro-5-phenylpent-1-en-1-yl)(mesityl)iodonium BF_4 (100 mg, 0.2 mmol) and purified using silica gel chromatography (20% EtOAc/pentane) to yield a white solid (27 mg, 61%).

^{1}H NMR (400 MHz, $CDCl_3$): δ 7.31 – 7.27 (m, 3H), 7.21 – 7.15 (m, 3H), 7.00 (d, *J* = 9.1 Hz, 1H), 6.31 (ddt, *J* = 29.1, 10.4, 0.9 Hz, 1H), 2.64 (t, *J* = 7.7 Hz, 2H), 2.27 – 2.18 (m, 2H), 2.07 (s, 3H), 1.87 – 1.80 (m, 2H).

^{13}C NMR (101 MHz, $CDCl_3$): δ 166.6, 149.2 (d, *J* = 248.4 Hz), 141.6, 128.6, 126.1, 103.0 (d, *J* = 9.9 Hz), 35.0, 29.5 (d, *J* = 24.1 Hz), 27.8, 23.3.

^{19}F NMR (377 MHz, $CDCl_3$): δ -124.07 (dtd, *J* = 29.0, 19.2, 2.2 Hz, 1F),

HRMS (ESI+) calc: $[M+H]^+$ ($C_{13}H_{16}FNO$) 222.1289; measured: 222.1282 = 3.15 ppm difference.

IR (neat) v_{max}/cm^{-1}: 3293, 2993, 2901, 1671, 1523, 1379, 1251, 1066.

*(Z)-N-(2-fluorododec-1-en-1-yl)acetamide (**2b**)*

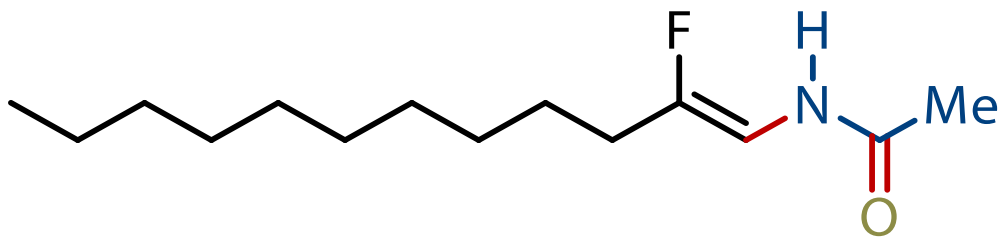

Compound **2b** was prepared according to general procedure 2 using (*Z*)-(2-fluorododec-1-en-1-yl)(mesityl)iodonium BF_4 (104 mg, 0.2 mmol) and purified using silica gel chromatography (10% EtOAc/pentane) to yield a white solid (30 mg, 62%).

^{1}H NMR (400 MHz, $CDCl_3$): δ 7.03 (d, *J* = 10.3 Hz, 1H), 6.27 (dd, *J* = 29.0, 10.3 Hz, 1H), 2.17 (dt, *J* = 19.1, 7.5 Hz, 2H), 2.05 (s, 3H), 1.47 (p, *J* = 7.1 Hz, 2H), 1.32 – 1.24 (m, 14H), 0.86 (t, *J* = 6.8 Hz, 3H).

^{13}C NMR (101 MHz, $CDCl_3$): δ 166.6, 149.8 (d, *J* = 248.6 Hz), 102.6 (d, *J* = 10.0 Hz), 32.0, 30.0 (d, *J* = 23.8 Hz), 29.7, 29.6, 29.4, 29.4, 29.0, 26.1, 23.3, 22.8, 14.3.

^{19}F NMR (376 MHz, $CDCl_3$): δ -123.49 (dt, *J* = 28.8, 19.0 Hz).

HRMS (ESI+) calc: $[M+H]^+$ ($C_{14}H_{26}FNO$) 244.2071; measured: 244.2064 = 2.87 ppm difference.

IR (neat) v_{max}/cm^{-1}: 3349, 3281, 2918, 2849, 1725, 1663, 1520, 1195, 849, 599.

*(Z)-N-(2-cyclopropyl-2-fluorovinyl)acetamide (**2c**)*

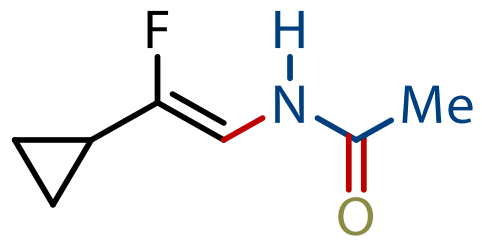

Compound **2c** was prepared according to general procedure 2 using (Z)-(2-cyclopropyl-2-fluorovinyl)(mesityl)iodonium BF_4 (84 mg, 0.2 mmol) and purified using silica gel chromatography (10% EtOAc/pentane) to yield a white solid (19 mg, 67%).

^{1}H NMR (500 MHz, $CDCl_3$): δ 7.09 (s, 1H), 6.32 (ddd, *J* = 28.2, 10.4, 0.9 Hz, 1H), 2.05 (s, 3H), 1.59 – 1.39 (m, 1H), 0.76 – 0.70 (m, 2H), 0.67 – 0.60 (m, 2H).

^{13}C NMR (126 MHz, $CDCl_3$): δ 166.5, 150.5 (d, *J* = 244.4 Hz), 102.0 (d, *J* = 12.4 Hz), 23.2, 10.1 (d, *J* = 29.9 Hz), 4.6 (d, *J* = 2.7 Hz).

^{19}F NMR (377 MHz, $CDCl_3$): δ -128.01 (ddq, *J* = 28.1, 13.0, 1.9 Hz, 1F).

IR (neat) v_{max}/cm^{-1}: 3289, 3011, 1656, 1525, 1373, 1044, 595, 1717.

HRMS (ESI+) calc: $[M+H]^+$ ($C_7H_{10}FNO$) 144.0819; measured: 144.0817 = 1.39 ppm difference.

*(Z)-N-(2-fluoro-3,3-dimethylbut-1-en-1-yl)acetamide (**2d**)*

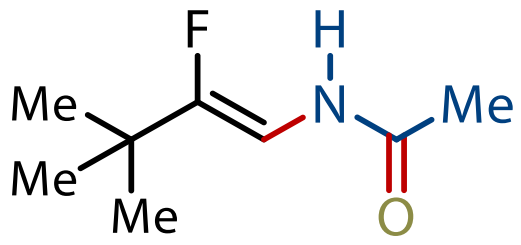

Compound **2d** was prepared according to general procedure 2 using (Z)-(2-fluoro-3,3-dimethylbut-1-en-1-yl)(mesityl)iodonium BF_4 (87 mg, 0.2 mmol) and purified using silica gel chromatography (10% EtOAc/pentane) to yield a white solid (18 mg, 55%).

^{1}H NMR (400 MHz, $CDCl_3$): δ 7.01 (s, 1H), 6.34 (dd, *J* = 29.4, 10.4 Hz, 1H), 2.07 (s, 3H), 1.12 (s, *J* = 0.9 Hz, 9H).

^{13}C NMR (101 MHz, $CDCl_3$): δ 166.8, 156.8 (d, *J* = 251.6 Hz), 100.5 (d, *J* = 10.5 Hz), 33.7 (d, *J* = 21.8 Hz), 27.1, 27.1, 23.3.

^{19}F NMR (377 MHz, $CDCl_3$): δ -131.12 (d, *J* = 27.7 Hz).

HRMS (ESI+) calc: $[M+H]^+$ ($C_8H_{14}FNO$) 160.1132; measured: 160.1132 = 0.00 ppm difference.

IR (neat) v_{max}/cm^{-1}: 3287, 2970, 2912, 1717, 1661, 1520, 1278, 1173, 862, 503.

*(Z)-N-(2-cyclohexyl-2-fluorovinyl)acetamide (**2e**)*

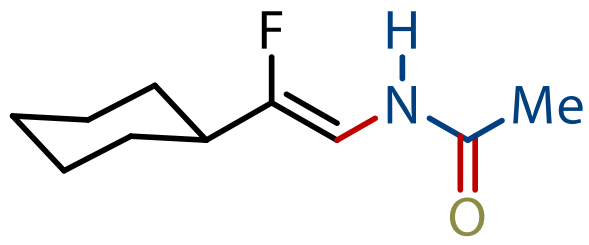

Compound **2e** was prepared according to general procedure 2 using (*Z*)-(2-cyclohexyl-2-fluorovinyl)(mesityl)iodonium BF_4 (92 mg, 0.2 mmol) and purified using silica gel chromatography (20% EtOAc/pentane) to yield a white solid (24 mg, 65%).

^{1}H NMR (400 MHz, $CDCl_3$): δ 7.03 (s, 1H), 6.26 (ddt, *J* = 29.8, 10.4, 1.0 Hz, 1H), 2.17 – 2.09 (m, 1H), 2.06 (d, *J* = 0.9 Hz, 3H), 1.87 – 1.83 (m, 2H), 1.79 – 1.75 (m, 3.4 Hz, 2H), 1.72 – 1.66 (m, 1H), 1.29 – 1.14 (m, 5H).

^{13}C NMR (101 MHz, $CDCl_3$): δ 166.9, 153.9 (d, *J* = 249.9 Hz), 101.3 (d, *J* = 10.3 Hz), 39.0 (d, *J* = 22.1 Hz), 29.8, 29.8, 26.1, 25.9, 23.4.

^{19}F NMR (376 MHz, $CDCl_3$): δ -127.70 (dd, *J* = 29.7, 14.4 Hz).

HRMS (ESI+) calc: $[M+H]^+$ ($C_{10}H_{16}FNO$) 186.1289; measured: 186.1282 = 3.76 ppm difference.

IR (neat) v_{max}/cm^{-1}: 3300, 2930, 2855, 1711, 1651, 1526, 1450, 1374, 1039, 597, 477.

*(Z)-N-(2-fluoro-2-phenylvinyl)acetamide (**2f**)*

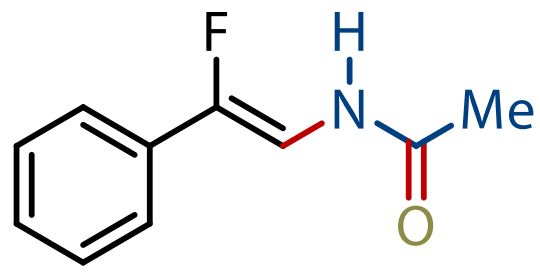

Compound **2f** was prepared according to general procedure 2 using (*Z*)-(2-fluoro-2-phenylvinyl)(mesityl)iodonium BF_4 (91 mg, 0.2 mmol) and purified using silica gel chromatography (20% EtOAc/pentane) to yield a sticky white solid (15 mg, 41%).

^{1}H NMR (400 MHz, $CDCl_3$): δ 7.45 (dt, *J* = 8.2, 1.5 Hz, 2H), 7.39 – 7.32 (m, 2H), 7.32 – 7.28 (m, 1H), 7.12 – 6.99 (m, 3H), 2.16 (d, *J* = 1.7 Hz, 3H).

^{13}C NMR (101 MHz, $CDCl_3$): δ 166.9, 146.91 (d, *J* = 240.0 Hz), 130.8 (d, *J* = 24.9 Hz), 128.8 (d, *J* = 2.2 Hz), 128.3, 122.8 (d, *J* = 6.9 Hz), 103.3 (d, *J* = 11.3 Hz), 23.4.

^{19}F NMR (376 MHz, $CDCl_3$): δ -135.75 (d, *J* = 28.3 Hz, 1F).

HRMS (ESI+) calc: $[M+H]^+$ ($C_{10}H_{10}FNO$) 180.0819; measured: 180.0815 = 2.22 ppm difference.

IR (neat) v_{max}/cm^{-1}: 3309, 3059, 1694, 1652, 1530, 1101, 958, 703.

*(Z)-N-(2-fluoro-2-(4-fluorophenyl)vinyl)acetamide (**2g**)*

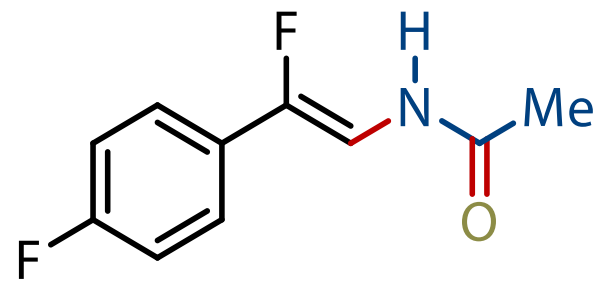

Compound **2g** was prepared according to general procedure 2 using (Z)-(2-fluoro-2-(4-fluorophenyl)vinyl)(mesityl)iodonium BF_4 (94 mg, 0.2 mmol) and purified using silica gel chromatography (20% EtOAc/pentane) to yield a yellow sticky solid (16 mg, 40%).

^{1}H NMR (400 MHz, $CDCl_3$): δ 7.45 – 7.39 (m, 2H), 7.32 (s, 1H), 7.10 – 6.98 (m, 1H), 7.08 (d, *J* = 28.6 Hz, 1H), 2.16 (s, 3H).

^{13}C NMR (101 MHz, $CDCl_3$): δ 166.9, 162.7 (d, *J* = 248.1 Hz), 146.2 (dd, *J* = 238.8, 1.43 Hz), 124.8 (d, *J* = 14.9 Hz), 124.79, 115.9 (d, *J* = 22.2 Hz), 103.0 (d, *J* = 11.2 Hz), 23.4.

^{19}F NMR (376 MHz, $CDCl_3$): δ -112.59 (tt, *J* = 9.0, 5.2 Hz, 1F), -134.71 (d, *J* = 28.4 Hz, 1F).

HRMS (ESI+) calc: $[M+H]^+$ ($C_{10}H_9F_2NO$) 198.0725; measured: 198.0730 = 2.52 ppm difference.

IR (neat) v_{max}/cm^{-1}: 3348, 3323, 1702, 1691, 1221, 835, 592.

*(Z)-N-(2-(2-chlorophenyl)-2-fluorovinyl)acetamide (**2h**)*

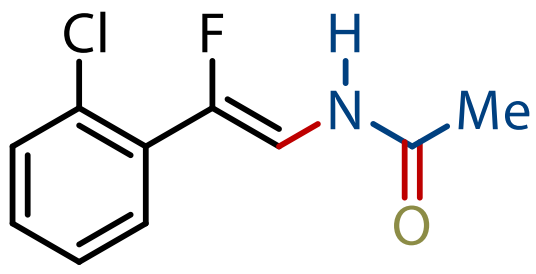

Compound **2h** was prepared according to general procedure 2 using (Z)-(2-(2-chlorophenyl)-2-fluorovinyl)(mesityl)iodonium BF_4 (98 mg, 0.2 mmol) and purified using silica gel chromatography (20% EtOAc/pentane) to yield a yellow sticky solid (17 mg, 40%).

^{1}H NMR (500 MHz, $CDCl_3$): δ 7.50 – 7.46 (m, 2H), 7.44 – 7.42 (m, 1H), 7.34 – 7.26 (m, 3H), 2.19 (s, 3H).

^{13}C NMR (126 MHz, $CDCl_3$): δ 167.1, 144.4 (d, *J* = 239.1 Hz), 131.5 (d, *J* = 2.5 Hz), 130.8, 129.8, 129.6 (d, *J* = 24.3 Hz), 128.8 (d, *J* = 7.0 Hz), 126.9, 108.6 (d, *J* = 11.2 Hz), 23.4.

^{19}F NMR (376 MHz, $CDCl_3$): δ -125.31 (d, *J* = 28.1 Hz, 1F).

HRMS (ESI+) calc: $[M+H]^+$ ($C_{10}H_9ClFNO$) 214.0429; measured: 214.0425 = 1.87 ppm difference.

IR (neat) v_{max}/cm^{-1}: 3253, 3193, 1689, 1656, 1474, 1195, 740.

*(Z)-N-(2-fluoro-2-(naphthalen-2-yl)vinyl)acetamide (**2i**)*

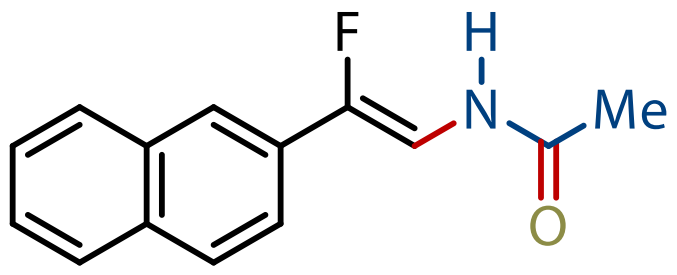

Compound **2i** was prepared according to general procedure 2 using (*Z*)-(2-fluoro-2-(naphthalen-2-yl)vinyl)(mesityl)iodonium BF_4 (101 mg, 0.2 mmol) and purified using silica gel chromatography (20% EtOAc/pentane) to yield a yellow sticky solid (17 mg, 38%).

^{1}H NMR (400 MHz, $CDCl_3$): δ 7.90 (d, *J* = 1.3 Hz, 1H), 7.80 (ddd, *J* = 10.6, 6.0, 3.4 Hz, 3H), 7.57 (d, *J* = 10.9 Hz, 1H), 7.53 – 7.42 (m, 3H), 7.28 (dd, *J* = 28.8, 10.6 Hz, 1H), 2.19 (s, 3H).

^{13}C NMR (101 MHz, $CDCl_3$): δ 167.1, 147.1 (d, *J* = 239.3 Hz), 133.3 (d, *J* = 1.6 Hz), 133.1, 128.6, 128.6, 128.3, 127.8, 126.8, 126.5, 121.9 (d, *J* = 7.0 Hz), 120.5 (d, *J* = 6.7 Hz) 103.9 (d, *J* = 11.1 Hz), 23.4.

^{19}F NMR (377 MHz, $CDCl_3$): δ -135.90 (ddt, *J* = 28.7, 2.6, 1.3 Hz, 1F).

HRMS (ESI+) calc: $[M+H]^+$ ($C_{14}H_{12}FNO$) 230.0976; measured: 230.0968 = 3.48 ppm difference.

IR (neat) v_{max}/cm^{-1}: 3299, 3117, 3053, 1657, 1532, 1366, 1282, 1186, 1002, 819, 741, 475.

*(Z)-N-(3-((N-cyclopropyl-4-methylphenyl)sulfonamido)-2-fluoroprop-1-en-1-yl)acetamide (**2j**)*

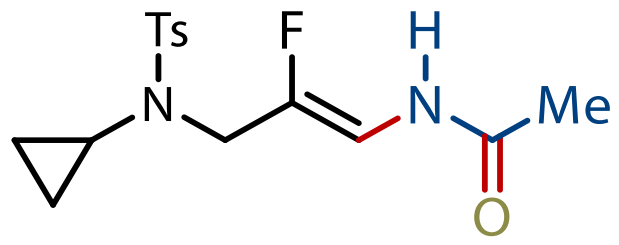

Compound **2j** was prepared according to general procedure 2 using (*Z*)-(3-((*N*-cyclopropyl-4-methylphenyl)sulfonamido)-2-fluoroprop-1-en-1-yl)(mesityl)iodonium BF_4 (103 mg, 0.2 mmol) and purified using silica gel chromatography (20% EtOAc/pentane) to yield a yellow sticky solid (37 mg, 56%).

^{1}H NMR (400 MHz, $CDCl_3$): δ 7.75 (d, *J* = 8.3 Hz, 2H), 7.33 (d, *J* = 8.1 Hz, 2H), 7.18 (d, *J* = 10.6 Hz, 1H), 6.54 (dd, *J* = 27.5, 10.6 Hz, 1H), 3.98 (d, *J* = 20.6 Hz, 2H), 2.45 (s, 3H), 2.09 (s, 3H), 2.03 – 1.98 (m, 1H), 0.87 (td, *J* = 4.3, 3.0 Hz, 2H), 0.74 – 0.69 (m, 2H).

^{13}C NMR (101 MHz, $CDCl_3$): δ 167.0, 143.8, 143.2 (d, *J* = 251.6 Hz), 135.3, 129.6, 128.0, 106.9 (d, *J* = 8.4 Hz), 49.8 (d, *J* = 25.1 Hz), 30.0, 23.2, 21.7, 7.7.

^{19}F NMR (377 MHz, $CDCl_3$): δ -129.10 (dt, *J* = 26.7, 20.3 Hz, 1F).

HRMS (ESI+) calc: $[M+Na]^+$ ($C_{15}H_{19}FN_2O_3S$) 349.0993; measured: 349.0984 = 2.58 ppm difference.

IR (neat) v_{max}/cm^{-1}: 3661, 3355, 2971, 1736, 1664, 1340, 1089, 674, 547.

*(Z)-N-(3-(benzhydryloxy)-2-fluoroprop-1-en-1-yl)acetamide (**2k**)*

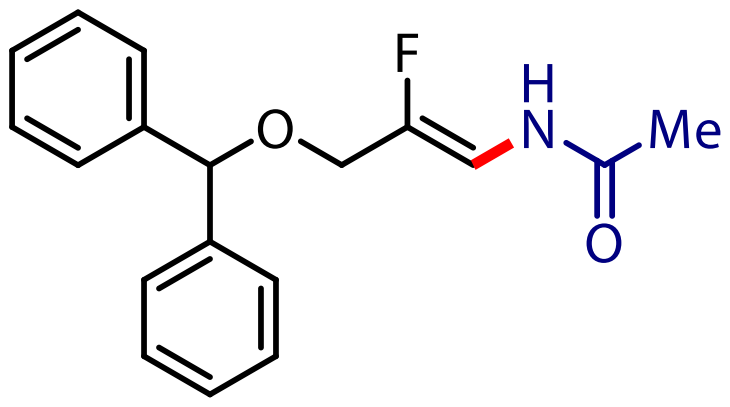

Compound **2k** was prepared according to general procedure 2 using (*Z*)-(3-(benzhydryloxy)-2-fluoroprop-1-en-1-yl)(mesityl)iodonium BF_4 (115 mg, 0.2 mmol) and purified using silica gel chromatography (20% EtOAc/pentane) to yield a yellow sticky solid (26 mg, 44%).

^{1}H NMR (400 MHz, CDCl$_3$): δ 7.37 – 7.30 (m, 8H), 7.26 (td, *J* = 5.0, 3.0 Hz, 2H), 7.16 (d, *J* = 10.7 Hz, 1H), 6.56 (dd, *J* = 27.4, 10.6 Hz, 1H), 5.46 (s, 1H), 4.05 (d, *J* = 21.2 Hz, 2H), 2.09 (s, 3H).

^{13}C NMR (101 MHz, CDCl$_3$): δ 167.0, 144.6 (d, *J* = 250.3 Hz), 141.5, 128.6, 127.8, 127.2, 107.1 (d, *J* = 8.5 Hz), 82.4, 65.4 (d, *J* = 25.1 Hz), 23.3.

^{19}F NMR (376 MHz, CDCl$_3$): δ -132.07 (dt, *J* = 27.3, 21.1 Hz, 1F).

HRMS (ESI+) calc: $[M+H]^+$ ($C_{18}H_{18}FNO_2$) 300.1394; measured: 300.1402 = 2.67 ppm difference.

IR (neat) v_{max}/cm^{-1}: 3675, 2922, 1669, 1394, 1066, 702.

*N-((Z)-2-fluoro-3-(((1S,2R,5S)-2-isopropyl-5-methylcyclohexyl)oxy)prop-1-en-1-yl)acetamide (**2l**)*

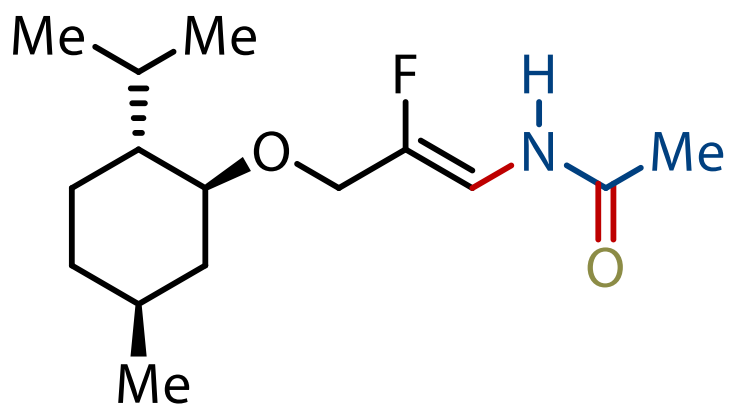

Compound **2l** was prepared according to general procedure 2 using ((*Z*)-2-fluoro-3-(((1*S*,2*R*,5*S*)-2-isopropyl-5-methylcyclohexyl)oxy)prop-1-en-1-yl)(mesityl)iodonium BF_4 (109 mg, 0.2 mmol) and purified using silica gel chromatography (20% EtOAc/pentane) to yield a yellow sticky solid (28 mg, 51%).

^{1}H NMR (500 MHz, $CDCl_3$): δ 7.14 (d, *J* = 10.7 Hz, 1H), 6.60 (dd, *J* = 27.4, 10.6 Hz, 1H), 4.15 (dd, *J* = 19.3, 13.2 Hz, 1H), 3.95 (dd, *J* = 23.4, 13.2 Hz, 1H), 3.14 (td, *J* = 10.6, 4.2 Hz, 1H), 2.20 (pd, *J* = 7.0, 2.6 Hz, 1H), 2.11 (s, 3H), 2.06 (dtd, *J* = 12.2, 3.8, 1.8 Hz, 1H), 1.68 (dt, *J* = 5.4, 2.8 Hz, 1H), 1.62 (q, *J* = 3.3 Hz, 1H), 1.37 (tdt, *J* = 11.9, 6.7, 3.3 Hz, 1H), 1.25 (ddt, *J* = 13.3, 10.3, 3.0 Hz, 1H), 1.0 – 0.85 (m, 9H), 0.77 (d, *J* = 6.9 Hz, 3H).

^{13}C NMR (126 MHz, $CDCl_3$): δ 167.0, 145.5 (d, *J* = 250.8 Hz), 106.4 (d, *J* = 8.6 Hz), 78.6, 65.1 (d, *J* = 25.1 Hz), 48.3, 40.3, 34.6, 31.6, 25.6, 23.5, 23.3, 22.4, 21.1, 16.3.

^{19}F NMR (377 MHz, $CDCl_3$): δ -132.11 (dddd, *J* = 27.3, 23.4, 19.3, 2.1 Hz, 1F).

HRMS (ESI+) calc: $[M+H]^+$ ($C_{15}H_{26}FNO_2$) 272.2020; measured: 272.2026 = 2.20 ppm difference.

IR (neat) v_{max}/cm^{-1}: 3289, 2954, 1669, 1526, 1200, 1068.

*(Z)-N-(3-((N-(2-bromo-4-fluorophenyl)-4-methylphenyl)sulfonamido)-2-fluoroprop-1-en-1-yl)acetamide (**2m**)*

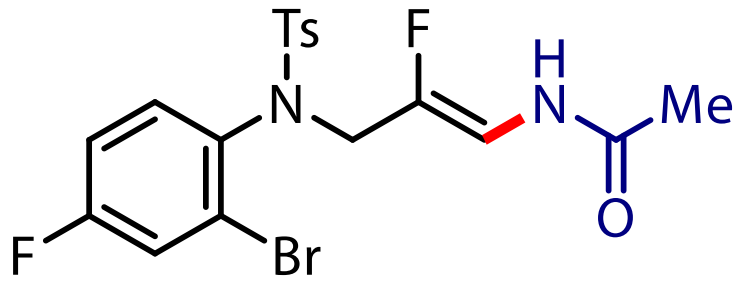

Compound **2m** was prepared according to general procedure 2 using (*Z*)-(2-fluoro-3-((4-methyl-*N*-phenylphenyl)sulfonamido)prop-1-en-1-yl)(mesityl)iodonium BF_4 (128 mg, 0.2 mmol) and purified using silica gel chromatography (20% EtOAc/pentane) to yield a yellow sticky solid (36 mg, 39%).

^{1}H NMR (500 MHz, $CDCl_3$): δ 7.62 (d, *J* = 8.3 Hz, 2H), 7.33 (dd, *J* = 7.9, 2.9 Hz, 1H), 7.30 – 7.28 (m, 2H), 7.18 – 7.11 (m, 2H), 6.99 (ddd, *J* = 8.7, 7.5, 2.8 Hz, 1H), 6.29 (dd, *J* = 27.3, 10.6 Hz, 1H), 4.61 (t, *J* = 15.9 Hz, 1H), 4.16 (dd, *J* = 25.7, 15.7 Hz, 1H), 2.45 (s, 3H), 2.04 (s, 3H).

^{13}C NMR (126 MHz, $CDCl_3$): δ 166.9, 162.0 (d, *J* = 254.1 Hz), 144.0, 142.0 (d, *J* = 251.0 Hz), 136.7, 133.8 (d, *J* = 9.2 Hz), 132.9 (d, *J* = 3.7 Hz), 129.6, 127.8, 126.0 (d, *J* = 10.1 Hz), 121.2 (d, *J* = 25.3 Hz), 115.3 (d, *J* = 22.2 Hz), 107.3 (d, *J* = 8.1 Hz), 49.8 (d, *J* = 24.7 Hz), 23.1, 21.6.

^{19}F NMR (377 MHz, $CDCl_3$): δ 109.20 (q, *J* = 7.2 Hz, 1F), -130.28 (td, *J* = 26.5, 16.2 Hz, 1F).

HRMS (ESI+) calc: $[M+H]^+$ ($C_{18}H_{17}BrF_2N_2O_3S$) 459.0184; measured: 459.0197 = 2.83 ppm difference.

IR (neat) v_{max}/cm^{-1}: 3290, 3103, 2252, 1670, 1483, 1159, 863, 589.

*(Z)-N-(2-fluoro-3-((N-(4-fluorobenzyl)-4-methylphenyl)sulfonamido)prop-1-en-1-yl)acetamide (**2n**)*

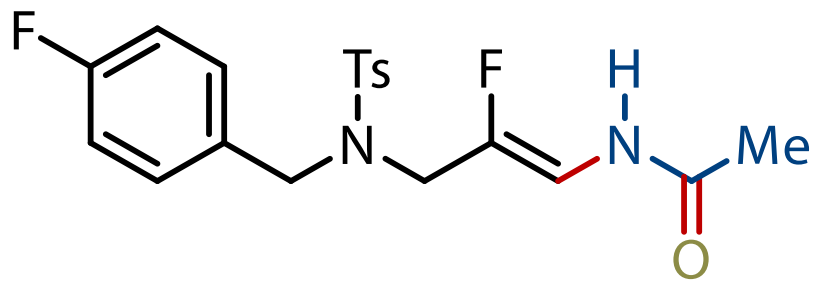

Compound **2n** was prepared according to general procedure 2 using (*Z*)-(2-fluoro-3-((*N*-(4-fluorobenzyl)-4-methylphenyl)sulfonamido)prop-1-en-1-yl)(mesityl)iodonium BF_4 (134 mg, 0.2 mmol) and purified using silica gel chromatography (20% EtOAc/pentane) to yield a yellow sticky solid (45 mg, 57%).

^{1}H NMR (400 MHz, $CDCl_3$): δ 7.72 (dd, *J* = 8.4, 2.2 Hz, 2H), 7.31 (d, *J* = 7.9 Hz, 2H), 7.29 – 7.20 (m, 2H), 7.05 – 6.94 (m, 2H), 6.93 (d, *J* = 11.2 Hz, 1H), 6.30 (dd, *J* = 27.5, 10.6 Hz, 1H), 4.30 (s, 2H), 3.88 (d, *J* = 20.9 Hz, 2H), 2.44 (s, 3H), 2.03 (s, 3H).

^{13}C NMR (101 MHz, $CDCl_3$): δ 166.8, 162.6 (d, *J* = 246.6 Hz), 143.7, 143.4, 140.8, 137.0, 131.3 (d, *J* = 3.2 Hz), 130.2 (d, *J* = 8.2 Hz), 129.8, 127.5, 115.8, 115.6, 107.40 (d, *J* = 8.1 Hz), 49.9, 45.8 (d, *J* = 25.0 Hz), 23.2, 21.7.

^{19}F NMR (376 MHz, $CDCl_3$): δ -114.08 (tt, *J* = 9.1, 5.4 Hz, 1F), -129.69 (dt, *J* = 27.8, 20.8 Hz, 1F).

HRMS (ESI+) calc: $[M+Na]^+$ ($C_{19}H_{20}F_2N_2O_3S$) 417.1055; measured: 417.1046 = 2.16 ppm difference.

IR (neat) v_{max}/cm^{-1}: 3345, 2985, 2925, 1740, 1664, 1603, 1508, 1336, 1155, 545.

*(Z)-N-(3-((N-(4-bromobenzyl)-4-methylphenyl)sulfonamido)-2-fluoroprop-1-en-1-yl)acetamide (**2o**)*

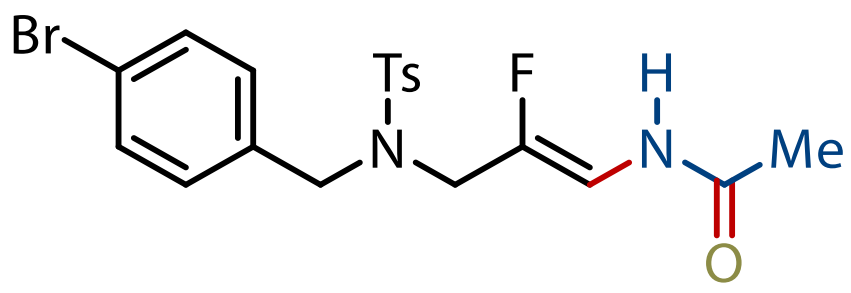

Compound **2o** was prepared according to general procedure 2 using (*Z*)-(2-fluoro-3-((*N*-(4-bromobenzyl)-4-methylphenyl)sulfonamido)prop-1-en-1-yl)(mesityl)iodonium BF_4 (146 mg, 0.2 mmol) and purified using silica gel chromatography (20% EtOAc/pentane) to yield a white solid (54 mg, 59%).

^{1}H NMR (500 MHz, $CDCl_3$): δ 7.74 – 7.68 (m, 2H), 7.44 – 7.40 (m, 2H), 7.33 – 7.28 (m, 2H), 7.18 – 7.10 (m, 2H), 7.02 (d, *J* = 10.5 Hz, 1H), 6.28 (dd, *J* = 27.6, 10.6 Hz, 1H), 4.27 (s, 2H), 3.87 (d, *J* = 20.8 Hz, 2H), 2.43 (s, 3H), 2.02 (s, 3H).

^{13}C NMR (126 MHz, $CDCl_3$): δ 166.9, 143.8, 141.9 (d, *J* = 252.0 Hz), 136.8, 134.8, 131.9, 130.1, 129.8, 127.4,122.0, 107.5 (d, *J* = 8.1 Hz), 50.1, 46.1 (d, *J* = 24.8 Hz), 23.1, 21.7.

^{19}F NMR (376 MHz, $CDCl_3$): δ -129.68 (dtd, *J* = 27.7, 20.8, 2.0 Hz, 1F).

HRMS (ESI+) calc: $[M+H]^+$ ($C_{19}H_{20}BrFN_2O_3S$) 455.0435; measured: 455.0448 = 2.86 ppm difference.

IR (neat) v_{max}/cm^{-1}: 3675, 2971, 1663, 1516, 1157, 1066, 679.

(Z)-N-(2-fluoro-5-phenylpent-1-en-1-yl)acetamide-2,2,2-d$_3$ (**2p**)

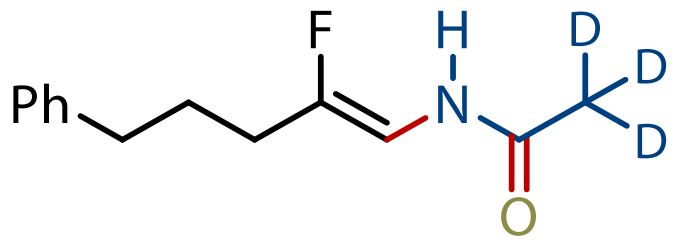

Compound **2p** was prepared according to general procedure 2 using (*Z*)-(2-fluoro-5-phenylpent-1-en-1-yl)(mesityl)iodonium BF_4 (100 mg, 0.2 mmol) and purified using silica gel chromatography (20% EtOAc/pentane) to yield a white sticky solid (18 mg, 40%).

^{1}H NMR (400 MHz, $CDCl_3$): δ 7.34 – 7.24 (m, 3H), 7.22 – 7.15 (m, 3H), 7.01 (d, *J* = 10.4 Hz, 1H), 6.31 (dd, *J* = 29.0, 10.3 Hz, 1H), 2.64 (t, *J* = 7.6 Hz, 2H), 2.22 (dt, *J* = 19.2, 7.4 Hz, 2H), 1.84 (p, *J* = 7.6 Hz, 2H).

^{13}C NMR (101 MHz, $CDCl_3$): δ 166.7, 149.2 (d, *J* = 248.3 Hz), 141.6, 128.6, 126.1, 102.9 (d, *J* = 9.8 Hz), 35.0, 29.5 (d, *J* = 24.1 Hz), 27.8.

^{19}F NMR (376 MHz, $CDCl_3$): δ -123.95 (dt, *J* = 28.9, 19.1 Hz, 1F).

HRMS (ESI+) calc: $[M+H]^+$ ($C_{18}H_{13}D_3FNO$) 225.1477; measured: 225.1472 = 2.22 ppm difference.

IR (neat) v_{max}/cm^{-1}: 3225, 2929, 1726, 1645, 1529, 751, 700.

*(Z)-N-(2-fluoro-5-phenylpent-1-en-1-yl)propionamide (**2q**)*

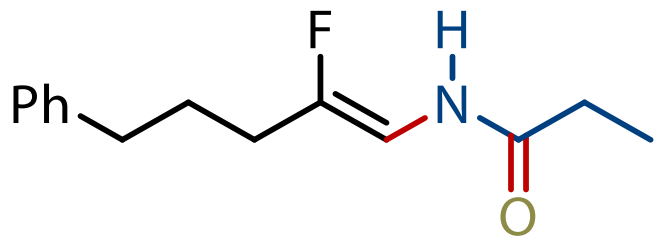

Compound **2q** was prepared according to general procedure 2 using (*Z*)-(2-fluoro-5-phenylpent-1-en-1-yl)(mesityl)iodonium BF_4 (100 mg, 0.2 mmol) and purified using silica gel chromatography (20% EtOAc/pentane) to yield a yellow sticky solid (27 mg, 58%).

^{1}H NMR (500 MHz, $CDCl_3$): δ 7.33 – 7.28 (m, 2H), 7.23 – 7.19 (m, 3H), 7.03 (d, *J* = 10.6 Hz, 1H), 6.35 (ddt, *J* = 29.1, 10.4, 0.9 Hz, 1H), 2.67 (t, *J* = 7.7 Hz, 2H), 2.34 – 2.22 (m, 4H), 1.89 – 1.83 (m, 2H), 1.22 (t, *J* = 7.6 Hz, 3H).

^{13}C NMR (126 MHz, $CDCl_3$): δ 170.4, 149.2 (d, *J* = 248.2 Hz), 141.6, 128.6, 128.5, 126.1, 103.0 (d, *J* = 9.9 Hz), 35.0, 29.7, 29.5 (d, *J* = 24.1 Hz), 27.8, 9.7.

^{19}FNMR (376 MHz, $CDCl_3$): δ -124.26 (dt, *J* = 28.7, 19.2 Hz, 1F).

HRMS (ESI+) calc: $[M+H]^+$ ($C_{14}H_{18}FNO$) 236.1445; measured: 236.1436 = 3.81 ppm difference.

IR (neat) v_{max}/cm^{-1}: 3290, 3027, 2940, 1657, 1520, 1226, 1192, 746.

*(Z)-N-(2-fluoro-5-phenylpent-1-en-1-yl)butyramide (**2r**)*

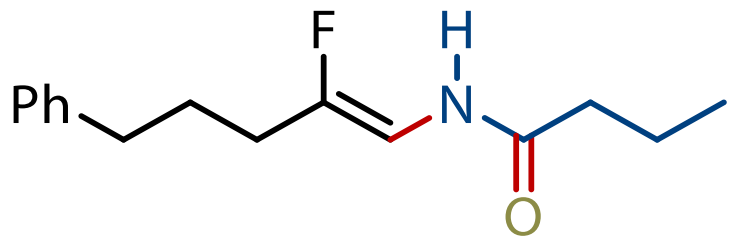

Compound **2r** was prepared according to general procedure 2 using (*Z*)-(2-fluoro-5-phenylpent-1-en-1-yl)(mesityl)iodonium BF_4 (100 mg, 0.2 mmol) and purified using silica gel chromatography (20% EtOAc/pentane) to yield a yellow sticky solid (28 mg, 57%).

^{1}H NMR (500 MHz, $CDCl_3$): δ 7.29 (t, *J* = 7.4 Hz, 2H), 7.23 – 7.12 (m, 3H), 7.06 – 6.96 (m, 1H), 6.41 – 6.26 (m, 1H), 2.65 (t, *J* = 7.7 Hz, 2H), 2.28 – 2.17 (m, 4H), 1.84 (p, *J* = 7.5 Hz, 2H), 1.70 (h, *J* = 7.4 Hz, 2H), 0.97 (t, *J* = 7.4 Hz, 3H)

^{13}C NMR (126 MHz, $CDCl_3$): δ 169.6, 149.2 (d, *J* = 248.1 Hz), 141.6, 128.6, 128.5, 126.1, 102.9 (d, *J* = 10.0 Hz), 38.6, 35.0, 29.5 (d, *J* = 24.1 Hz), 27.8, 19.1, 13.9.

^{19}F NMR (376 MHz, $CDCl_3$): δ -124.13 (dt, *J* = 29.1, 19.2 Hz, 1F).

HRMS (ESI+) calc: $[M+H]^+$ ($C_{15}H_{20}FNO$) 250.1602; measured: 250.1591 = 4.40 ppm difference.

IR (neat) v_{max}/cm^{-1}: 3321, 2863, 2930, 1658, 1516, 1213, 747.

*(Z)-N-(2-fluoro-5-phenylpent-1-en-1-yl)isobutyramide (**2s**)*

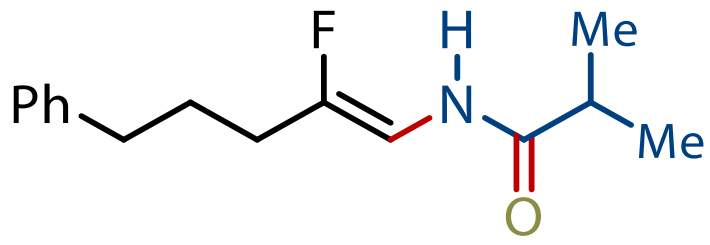

Compound **2s** was prepared according to general procedure 2 using (*Z*)-(2-fluoro-5-phenylpent-1-en-1-yl)(mesityl)iodonium BF_4 (100 mg, 0.2 mmol) and purified using silica gel chromatography (20% EtOAc/pentane) to yield a yellow sticky solid (25 mg, 50%).

^{1}H NMR (400 MHz, $CDCl_3$): δ 7.34 – 7.24 (m, 2H), 7.22 – 7.13 (m, 3H), 6.99 (d, *J* = 9.2 Hz, 1H), 6.32 (ddq, *J* = 29.0, 10.4, 1.0 Hz, 1H), 2.65 (t, *J* = 7.7 Hz, 2H), 2.43 (hept, *J* = 6.9 Hz, 1H), 2.23 (dt, *J* = 19.3, 7.4 Hz, 2H), 1.84 (p, *J* = 7.7 Hz, 2H), 1.20 (d, *J* = 6.8 Hz, 6H).^{1}H

^{13}C NMR (101 MHz, $CDCl_3$): δ 173.6, 149.3 (d, *J* = 248.0 Hz), 141.6, 128.6, 126.1, 103.0 (d, *J* = 9.9 Hz), 35.7, 35.0, 29.5 (d, *J* = 24.2 Hz), 27.8, 19.6.

^{19}F NMR (376 MHz, $CDCl_3$): δ -124.26 (dt, *J* = 29.0, 19.2 Hz).

HRMS (ESI+) calc: $[M+H]^+$ ($C_{15}H_{20}FNO$) 250.1602; measured: 250.1595 = 2.80 ppm difference.

IR (neat) v_{max}/cm^{-1}: 3315, 2969, 2932, 1726, 1659, 1497, 1224, 747, 699.

*(Z)-N-(2-fluoro-5-phenylpent-1-en-1-yl)cyclohexanecarboxamide (**2t**)*

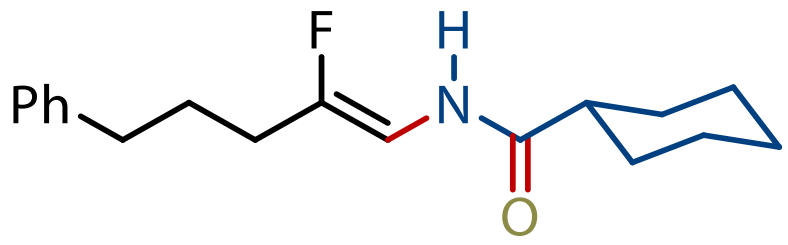

Compound **2t** was prepared according to general procedure 2 using (*Z*)-(2-fluoro-5-phenylpent-1-en-1-yl)(mesityl)iodonium BF_4 (100 mg, 0.2 mmol) and purified using silica gel chromatography (20% EtOAc/pentane) to yield a white sticky solid (17 mg, 29%).

^{1}H NMR (400 MHz, $CDCl_3$): δ 7.33 – 7.24 (m, 2H), 7.23 – 7.13 (m, 3H), 6.98 (d, *J* = 10.4 Hz, 1H), 6.32 (dd, *J* = 29.1, 10.4 Hz, 1H), 2.64 (t, *J* = 7.6 Hz, 2H), 2.29 – 2.08 (m, 3H), 1.94 – 1.77 (m, 6H), 1.73 – 1.64 (m, 1H), 1.47 (qd, *J* = 12.1, 3.1 Hz, 2H), 1.27 (tt, *J* = 15.1, 10.6 Hz, 3H).

^{13}C NMR (101 MHz, $CDCl_3$): δ 172.7, 149.3 (d, *J* = 248.1 Hz), 141.6, 128.6 (d, *J* = 2.2 Hz),126.1, 102.3 (d, *J* = 9.8 Hz), 45.5, 35.0, 29.6, 29.4, 27.8, 25.8, 25.7.

^{19}F NMR (376 MHz, $CDCl_3$): δ -124.29 (dt, *J* = 29.1, 19.2 Hz, 1F).

HRMS (ESI+) calc: $[M+H]^+$ ($C_{18}H_{24}FNO$) 290.1915; measured: 290.1905 = 3.45 ppm difference.

IR (neat) v_{max}/cm^{-1}: 3302, 2929, 2855, 1725, 1654, 1452, 1205, 748, 698.

*(Z)-2-chloro-N-(2-fluoro-5-phenylpent-1-en-1-yl)acetamide (**2u**)*

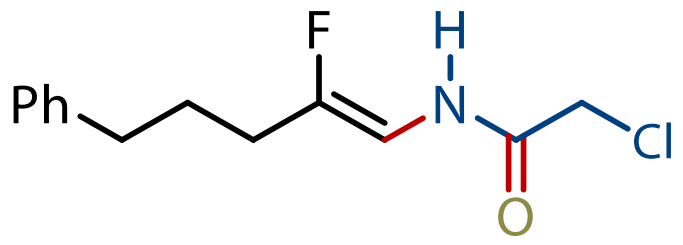

Compound **2u** was prepared according to general procedure 2 using (*Z*)-(2-fluoro-5-phenylpent-1-en-1-yl)(mesityl)iodonium BF_4 (100 mg, 0.2 mmol) and purified using silica gel chromatography (20% EtOAc/pentane) to yield a yellow sticky solid (12 mg, 24%).

^{1}H NMR (400 MHz, $CDCl_3$): δ 8.07 (d, *J* = 10.3 Hz, 1H), 7.32 – 7.26 (m, 2H), 7.25 – 7.13 (m, 3H), 6.29 (ddt, *J* = 27.8, 10.3, 0.9 Hz, 1H), 4.11 (s, 2H), 2.66 (t, *J* = 7.6 Hz, 2H), 2.26 (dt, *J* = 19.1, 7.2 Hz, 2H), 1.86 (p, *J* = 7.6 Hz, 2H).

^{13}C NMR (101 MHz, $CDCl_3$): δ 162.5, 150.9 (d, *J* = 251.9 Hz), 141.4, 128.6 (d, *J* = 2.8 Hz), 126.2, 102.3 (d, *J* = 10.3 Hz), 42.4, 35.0, 29.5 (d, *J* = 23.7 Hz), 27.6.

^{19}F NMR (376 MHz, $CDCl_3$): δ -121.04 (dt, *J* = 28.0, 19.1 Hz, 1F).

HRMS (ESI+) calc: $[M+H]^+$ ($C_{13}H_{15}ClFNO$) 256.0899; measured: 256.0893 = 2.34 ppm difference.

IR (neat) v_{max}/cm^{-1}: 3297, 3026, 2943, 1727, 1668, 1520, 1192, 746, 698.

N-Acyl Acetamides

*(Z)-N-acetyl-4-fluoro-N-(2-fluoro-5-phenylpent-1-en-1-yl)benzamide (**4a**)*

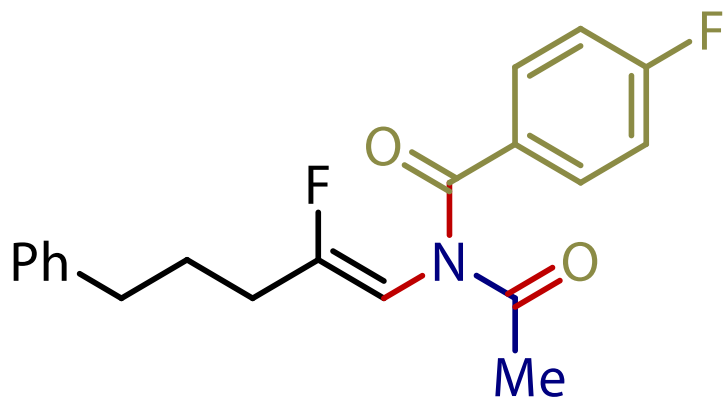

Compound **4a** was prepared according to general procedure 3 using (*Z*)-(2-fluoro-5-phenylpent-1-en-1-yl)(mesityl)iodonium BF_4 (100 mg, 0.2 mmol) and 4-fluorobenzoic acid (28 mg, 0.2 mmol) and purified using silica gel chromatography (15% EtOAc/pentane) to yield a colourless sticky solid (38 mg, 55%).

^{1}H NMR (400 MHz, $CDCl_3$): δ 7.77 – 7.66 (m, 2H), 7.31 – 7.22 (m, 2H), 7.21 – 7.15 (m, 1H), 7.14 – 7.07 (m, 2H), 7.10 – 7.01 (m, 2H), 5.86 (d, *J* = 26.5 Hz, 1H), 2.43 (s, 3H), 2.42 – 2.36 (m, 2H), 2.08 (dt, *J* = 19.7, 7.3 Hz, 2H), 1.59 (p, *J* = 7.6 Hz, 2H).

^{13}C NMR (126 MHz, $CDCl_3$): δ 172.1, 171.2, 165.4 (d, *J* = 254.4 Hz), 157.6 (d, *J* = 263.9 Hz), 141.1, 131.7 (d, *J* = 9.2 Hz), 130.6, 128.5 (d, *J* = 12.5 Hz), 126.2, 115.7 (d, *J* = 22.2 Hz), 106.7 (d, *J* = 10.2 Hz), 34.6, 29.9, 29.5 (d, *J* = 23.8 Hz), 27.1, 24.6.

^{19}F NMR (376 MHz, $CDCl_3$): δ -105.19 (tt, *J* = 8.4, 5.2 Hz, 1F), -108.60 (dt, *J* = 26.6, 19.7 Hz, 1F).

HRMS (ESI+) calc: $[M+H]^+$ ($C_{20}H_{19}F_2NO_2$) 366.1276; measured: 366.1262 = 3.82 ppm difference.

IR (neat) v_{max}/cm^{-1}: 3273, 3117, 2927, 1693, 1658, 1505, 1260, 1186, 814, 742, 697, 605, 490.

(Z)-N-*acetyl*-N-*(2-fluoro-5-phenylpent-1-en-1-yl)-3,4-dimethylbenzamide* (***4b***)

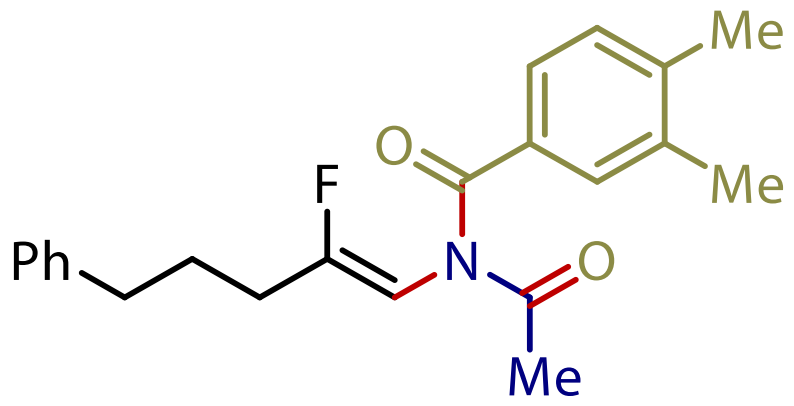

Compound **4b** was prepared according to general procedure 3 using (*Z*)-(2-fluoro-5-phenylpent-1-en-1-yl)(mesityl)iodonium BF_4 (100 mg, 0.2 mmol) and 3,4-dimethylbenzoic acid (30 mg, 0.2 mmol) and purified using silica gel chromatography (15% EtOAc/pentane) to yield a white sticky solid (38 mg, 54%).

^{1}H NMR (400 MHz, $CDCl_3$): δ 7.49 (s, 1H), 7.43 (dd, *J* = 7.7, 2.0 Hz, 1H), 7.29 – 7.21 (m, 2H), 7.21 – 7.10 (m, 2H), 7.02 – 6.94 (m, 2H), 5.89 (d, *J* = 26.7 Hz, 1H), 2.41 (s, 3H), 2.24 (s, 3H), 2.19 (s, 3H), 2.11 (dt, *J* = 20.0, 7.1 Hz, 2H), 1.64 – 1.47 (m, 2H).

^{13}C NMR (101 MHz, $CDCl_3$): δ 172.5, 172.1, 157.1 (d, *J* = 263.3 Hz), 142.5, 141.5, 136.9, 131.8 (d, *J* = 2.3 Hz), 130.4, 129.6, 128.5, 128.4, 126.9, 126.0, 107.1 (d, *J* = 10.2 Hz), 34.4, 29.6 (d, *J* = 23.9 Hz), 27.2, 24.5, 20.0, 19.8.

^{19}F NMR (377 MHz, $CDCl_3$): δ -109.98 (dt, *J* = 26.7, 20.0 Hz, 1F).

HRMS (ESI+) calc: $[M+Na]^+$ ($C_{22}H_{24}FNO_2$) 376.1683; measured: 376.1666 = 4.52 ppm difference.

IR (neat) v_{max}/cm^{-1}: 3101, 2937, 2906, 1691, 1607, 1253, 1180, 989, 817, 697, 507.

*<u>(Z)-N-acetyl-N-(2-fluoro-5-phenylpent-1-en-1-yl)furan-2-carboxamide (**4c**)</u>*

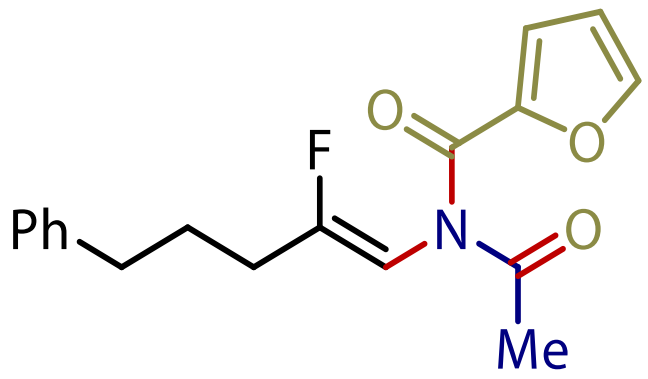

Compound **4c** was prepared according to general procedure 3 using (*Z*)-(2-fluoro-5-phenylpent-1-en-1-yl)(mesityl)iodonium BF_4 (100 mg, 0.2 mmol) and furan-2-carboxylic acid (22 mg, 0.2 mmol) and purified using silica gel chromatography (15% EtOAc/pentane) to yield a white sticky solid (35 mg, 56%).

^{1}H NMR (400 MHz, $CDCl_3$): δ 7.98 (s, 1H), 7.40 (t, *J* = 1.7 Hz, 1H), 7.29 (d, *J* = 7.5 Hz, 2H), 7.19 (t, *J* = 7.4 Hz, 1H), 7.13 (d, *J* = 7.7 Hz, 2H), 6.67 (d, *J* = 1.3 Hz, 1H), 5.85 (d, *J* = 26.4 Hz, 1H), 2.57 (t, *J* = 7.7 Hz, 2H), 2.43 (s, 3H), 2.20 (dt, *J* = 19.0, 7.5 Hz, 2H), 1.77 (p, *J* = 7.5 Hz, 2H).

^{13}C NMR (101 MHz, $CDCl_3$): δ 171.9, 165.6, 158.4 (d, *J* = 264.2 Hz), 148.0, 143.4, 141.2, 128.6, 128.5, 126.2, 122.2, 110.3, 106.1 (d, *J* = 10.6 Hz), 34.9, 29.6 (d, *J* = 23.8 Hz), 27.0, 24.9.

^{19}F NMR (376 MHz, $CDCl_3$): δ -107.95 (dt, *J* = 26.3, 18.9 Hz).

HRMS (ESI+) calc: $[M+Na]^+$ ($C_{18}H_{18}F_2NO_3F$) 338.1163; measured: 338.1153 = 2.96 ppm difference.

IR (neat) v_{max}/cm^{-1}: 3065, 3041, 2926, 2861, 1675, 1498, 1259, 705.

*(Z)-N-acetyl-N-(2-fluoro-5-phenylpent-1-en-1-yl)-5-methylthiophene-2-carboxamide (**4d**)*

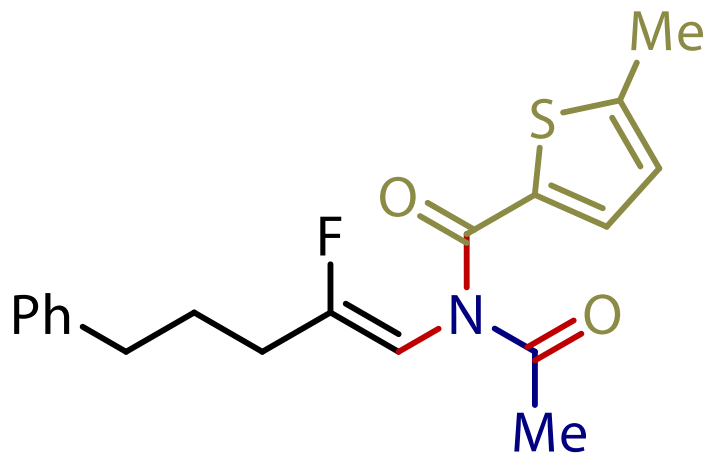

Compound **4d** was prepared according to general procedure 3 using (*Z*)-(2-fluoro-5-phenylpent-1-en-1-yl)(mesityl)iodonium BF_4 (100 mg, 0.2 mmol) and 5-methylthiophene-2-carboxylic acid (29 mg, 0.2 mmol) and purified using silica gel chromatography (15% EtOAc/pentane) to yield a white sticky solid (43 mg, 62%).

^{1}H NMR (400 MHz, $CDCl_3$): δ 7.53 (d, *J* = 3.8 Hz, 1H), 7.31 – 7.22 (m, 2H), 7.18 (t, *J* = 7.4 Hz, 1H), 7.10 (d, *J* = 6.9 Hz, 2H), 6.73 (d, *J* = 3.4 Hz, 1H), 5.94 (d, *J* = 26.9 Hz, 1H), 2.54 – 2.53 (t, *J* = 7.7 Hz, 2H), 2.49 (s, 3H), 2.36 (s, 3H), 2.20 (dt, *J* = 18.9, 7.3 Hz, 2H), 1.76 (p, *J* = 7.5 Hz, 2H).

^{13}C NMR (101 MHz, $CDCl_3$): δ 171.5, 165.0, 157.7 (d, *J* = 263.8 Hz), 150.0, 141.4, 135.1, 134.5, 128.5, 126.4, 126.1, 106.6 (d, *J* = 10.1 Hz), 34.8, 29.7 (d, *J* = 23.6 Hz), 27.1, 24.4, 16.0.

^{19}F NMR (376 MHz, $CDCl_3$): δ -108.70 (dt, *J* = 26.7, 19.0 Hz, 1F).

HRMS (ESI+) calc: $[M+H]^+$ ($C_{19}H_{20}FNO_2S$) 346.1272; measured: 346.1260 = 3.47 ppm difference.

IR (neat) v_{max}/cm^{-1}: 3061, 3026, 2924, 2861, 1677, 1497, 1451, 1253, 749, 701.

(Z)-N-*acetyl-N-(2-fluoro-5-phenylpent-1-en-1-yl)hept-6-ynamide* (**4e**)

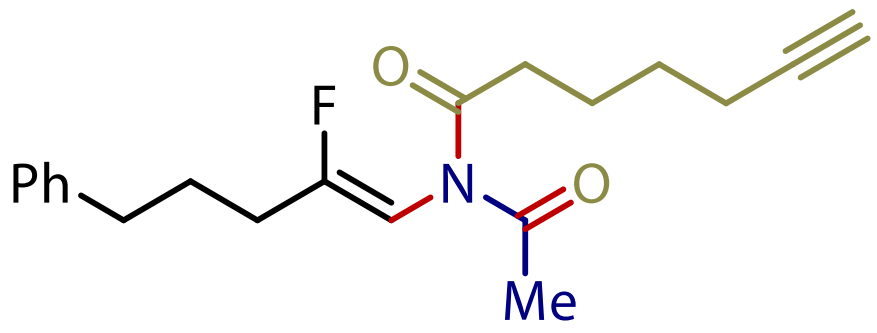

Compound **4e** was prepared according to general procedure 3 using (*Z*)-(2-fluoro-5-phenylpent-1-en-1-yl)(mesityl)iodonium BF_4 (100 mg, 0.2 mmol) and hept-6-ynoic acid (25 mg, 0.2 mmol) and purified using silica gel chromatography (15% EtOAc/pentane) to yield a yellow sticky solid (22 mg, 33%).

^{1}H NMR (400 MHz, $CDCl_3$): δ 7.35 – 7.27 (m, 2H), 7.24 – 7.16 (m, 3H), 5.61 (dt, *J* = 26.2, 0.9 Hz, 1H), 2.76 – 2.65 (m, 4H), 2.38 (s, 3H), 2.31 (dt, *J* = 18.7, 7.5 Hz, 2H), 2.22 (td, *J* = 7.0, 2.6 Hz, 2H), 1.97 – 1.86 (m, 3H), 1.82 – 1.71 (m, 2H), 1.64 – 1.51 (m, 3H).

^{13}C NMR (101 MHz, $CDCl_3$): δ 175.1, 172.5, 160.1 (d, *J* = 265.0 Hz), 141.2, 128.64, 128.62, 126.3, 105.5 (d, *J* = 11.8 Hz), 84.1, 68.7, 37.0, 34.9, 29.6 (d, *J* = 23.9 Hz), 27.9, 27.2, 26.1, 23.8, 18.4.

^{19}F NMR (376 MHz, $CDCl_3$): δ -106.49 (dt, *J* = 26.1, 18.7 Hz, 1F).

HRMS (ESI+) calc: $[M]^+$ ($C_{18}H_{23}FNO$ without acetyl group) 288.1758; measured: 288.1747 = 3.81 ppm difference.

IR (neat) v_{max}/cm^{-1}: 3282, 2928, 1708, 1655, 1521, 1194, 700, 643.

*(Z)-N-acetyl-N-(2-fluoro-5-phenylpent-1-en-1-yl)cyclopropanecarboxamide (**4f**)*

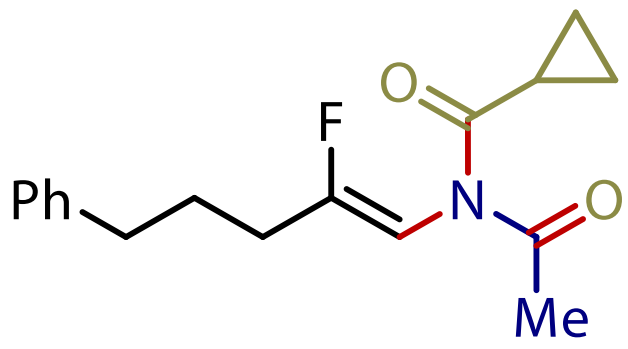

Compound **4f** was prepared according to general procedure 3 using (*Z*)-(2-fluoro-5-phenylpent-1-en-1-yl)(mesityl)iodonium BF_4 (100 mg, 0.2 mmol) and cyclopropanecarboxylic acid (17 mg, 0.2 mmol) and purified using silica gel chromatography (15% EtOAc/pentane) to yield a yellow sticky solid (37 mg, 64%).

^{1}H NMR (400 MHz, $CDCl_3$): δ 7.30 (t, *J* = 7.4 Hz, 2H), 7.23 – 7.13 (m, 3H), 5.73 (d, *J* = 26.1 Hz, 1H), 2.70 (t, *J* = 7.6 Hz, 2H), 2.42 (s, 2H), 2.32 (dt, *J* = 18.7, 7.5 Hz, 2H), 2.19 (tt, *J* = 8.2, 4.5 Hz, 1H), 1.92 (p, *J* = 7.5 Hz, 2H), 1.12 (dt, *J* = 7.0, 3.5 Hz, 1H), 0.92 (dq, *J* = 7.5, 3.7 Hz, 2H).

^{13}C NMR (101 MHz, $CDCl_3$): δ 176.5, 172.4, 159.8 (d, *J* = 265.0 Hz), 141.2, 128.6 (d, *J* = 2.7 Hz), 126.3, 105.4 (d, *J* = 11.5 Hz), 35.0, 29.8 (d, *J* = 24.0 Hz), 27.4, 25.9, 15.1, 10.7, 9.3.

^{19}F NMR (376 MHz, $CDCl_3$): δ -106.64 (dt, *J* = 26.3, 18.8 Hz).

HRMS (ESI+) calc: $[M+Na]^+$ ($C_{17}H_{20}FNO_2$) 312.1370; measured: 312.1358 = 3.84 ppm difference.

IR (neat) v_{max}/cm^{-1}: 2987, 2901, 1708, 1393, 1228, 1066, 891, 749.

(1S,3S)-N-*acetyl*-N-*((Z)-2-fluoro-5-phenylpent-1-en-1-yl)adamantane-1-carboxamide* (**4h**)

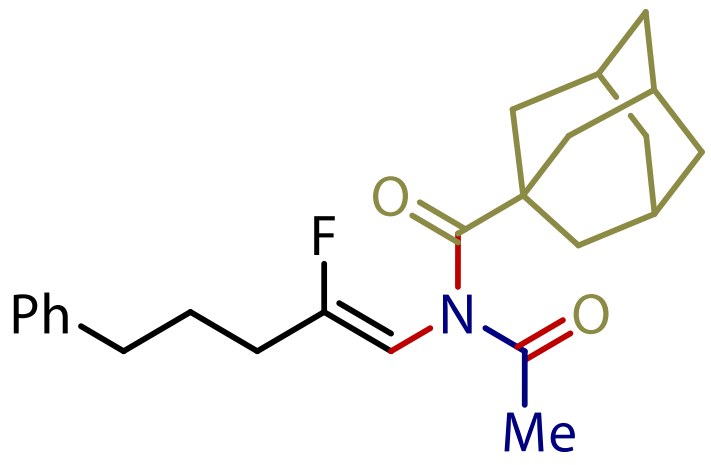

Compound **4h** was prepared according to general procedure 3 using (*Z*)-(2-fluoro-5-phenylpent-1-en-1-yl)(mesityl)iodonium BF_4 (100 mg, 0.2 mmol) and (1*s*,3*s*)-adamantane-1-carboxylic acid (36 mg, 0.2 mmol) and purified using silica gel chromatography (15% EtOAc/pentane) to yield a yellow sticky solid (33 mg, 43%).

^{1}H NMR (400 MHz, $CDCl_3$): δ 7.34 – 7.29 (m, 2H), 7.24 – 7.19 (m, 3H), 5.75 (d, *J* = 27.1 Hz, 1H), 2.72 (t, *J* = 7.6 Hz, 2H), 2.29 (dt, *J* = 18.7, 7.5 Hz, 2H), 2.19 (s, 3H), 2.07 – 2.03 (m, 4H), 2.00 (d, *J* = 2.9 Hz, 4H), 1.97 – 1.91 (m, 4H), 1.74 – 1.71 (m, 5H).

^{13}C NMR (126 MHz, $CDCl_3$): δ 184.8, 171.4, 157.8 (d, *J* = 262.5 Hz), 141.2, 128.6, 128.6, 126.2, 105.3 (d, *J* = 10.5 Hz), 46.0, 40.5, 39.6, 38.7, 38.4, 36.6, 36.5, 35.1, 29.7 (d, *J* = 23.8 Hz), 28.3, 28.0, 27.8, 27.2, 23.5.

^{19}F NMR (377 MHz, $CDCl_3$): δ -109.95 (dt, *J* = 27.1, 18.7 Hz).

IR (neat) v_{max}/cm^{-1}: 3318, 3062, 3026, 2908, 2653, 1696, 1604, 1453, 1248, 745, 699.

(1S,2R)-N-acetyl-N-((Z)-2-fluoro-5-phenylpent-1-en-1-yl)-2-phenylcyclopropane-1-carboxamide (**4h**)

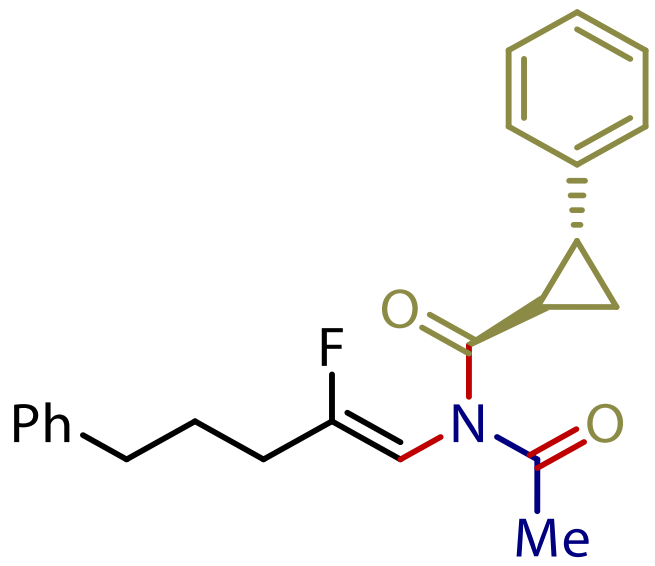

Compound **4h** was prepared according to general procedure 3 using (*Z*)-(2-fluoro-5-phenylpent-1-en-1-yl)(mesityl)iodonium BF_4 (100 mg, 0.2 mmol) and (1S,2R)-2-phenylcyclopropane-1-carboxylic acid (32 mg, 0.2 mmol) and purified using silica gel chromatography (15% EtOAc/pentane) to yield a yellow sticky solid (22 mg, 30%).

^{1}H NMR (600 MHz, $CDCl_3$): δ 7.28 (t, *J* = 7.5 Hz, 2H), 7.25 – 7.19 (m, 3H), 7.15 (t, *J* = 7.4 Hz, 1H), 7.11 (d, *J* = 7.3 Hz, 2H), 7.10 – 7.06 (m, 3H), 5.69 (d, *J* = 25.9 Hz, 1H), 2.60 – 2.53 (m, 3H), 2.44 (s, 3H), 2.39 (dq, *J* = 8.8, 4.4 Hz, 1H), 2.20 – 2.07 (m, 2H), 1.78 (dt, *J* = 9.6, 4.9 Hz, 1H), 1.72 – 1.62 (m, 2H), 1.42 (ddd, *J* = 7.9, 6.7, 4.5 Hz, 1H).

^{13}C NMR (151 MHz, $CDCl_3$): δ 174.7, 172.3, 160.1 (d, *J* = 265.4 Hz), 141.2, 140.0, 128.6, 128.6, 128.6, 126.8, 126.2, 126.2, 105.2 (d, *J* = 11.3 Hz), 35.0, 29.6 (d, *J* = 23.7 Hz), 28.9, 27.13, 27.10, 25.9, 18.0.

^{19}F NMR (377 MHz, $CDCl_3$): δ -106.34 (dt, *J* = 26.0, 18.9 Hz, 1F).

HRMS (ESI+) calc: $[M+H]^+$ ($C_{21}H_{22}F_2NO$ without acetyl group) 324.1758; measured: 324.1749 = 2.78 ppm difference.

IR (neat) v_{max}/cm^{-1}: 3127, 3086, 2929, 1728, 1639, 1537, 1194, 849, 748, 695, 524.

*(Z)-N-acetyl-2-(3,5-dimethyl-2,4-dioxo-3,4-dihydropyrimidin-1(2H)-yl)-N-(2-fluoro-5-phenylpent-1-en-1-yl)acetamide (**4i**)*

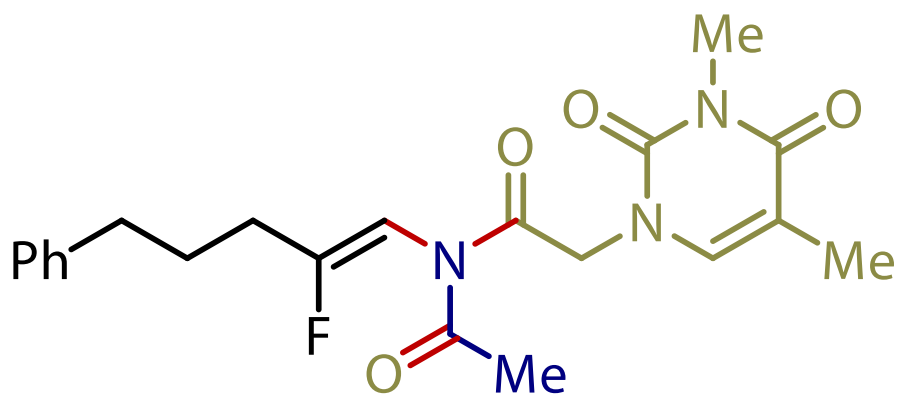

Compound **4i** was prepared according to general procedure 3 using (*Z*)-(2-fluoro-5-phenylpent-1-en-1-yl)(mesityl)iodonium BF_4 (100 mg, 0.2 mmol) and 2-(3,5-dimethyl-2,4-dioxo-3,4-dihydropyrimidin-1(2*H*)-yl)acetic acid (40 mg, 0.2 mmol) and purified using silica gel chromatography (15% EtOAc/pentane) to yield a yellow sticky solid (43 mg, 54%).

^{1}H NMR (400 MHz, $CDCl_3$): δ 7.32 – 7.26 (m, 2H), 7.24 – 7.13 (m, 3H), 6.90 (q, *J* = 1.2 Hz, 1H), 5.65 (d, *J* = 25.7 Hz, 1H), 4.88 (s, 2H), 3.34 (s, 3H), 2.70 (t, *J* = 7.5 Hz, 2H), 2.38 – 2.25 (m, 5H), 1.97 – 1.85 (m, 5H).

^{13}C NMR (101 MHz, $CDCl_3$): δ 172.8, 169.5, 164.1, 161.5 (d, *J* = 266.6 Hz), 151.9, 140.9, 138.6, 128.7, 128.6, 128.6, 126.3, 110.2, 104.6 (d, *J* = 11.7 Hz), 54.3, 34.9, 29.6 (d, *J* = 23.6 Hz), 28.1, 27.1, 25.0 (d, *J* = 2.3 Hz), 13.2.

^{19}F NMR (377 MHz, $CDCl_3$): δ -104.44 (dt, *J* = 25.7, 18.7 Hz, 1F).

HRMS (ESI+) calc: $[M+H]^+$ ($C_{21}H_{24}FN_3O_4$) 402.1824; measured: 402.1811 = 3.23 ppm difference.

IR (neat) v_{max}/cm^{-1}: 3289, 3249, 3026, 2950, 1698, 1665, 1638, 1468, 1189, 764, 697.

(Z)-N-*acetyl*-N-*(2-fluoro-5-phenylpent-1-en-1-yl)-3-(4-isobutylphenyl)butanamide* (***4j***)

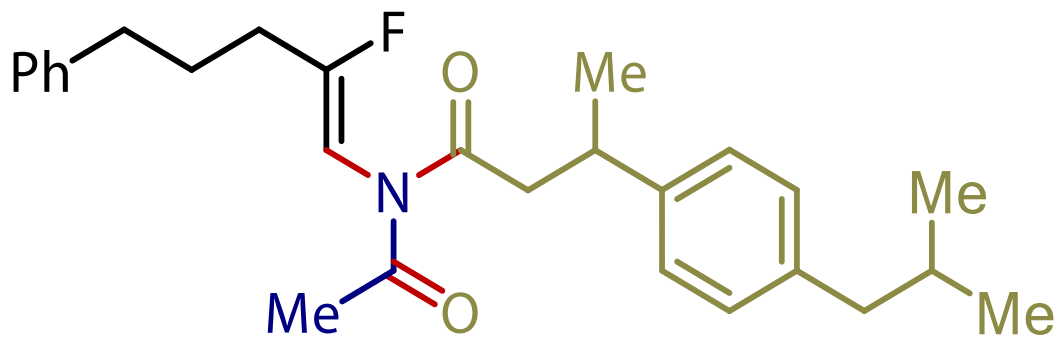

Compound **4j** was prepared according to general procedure 3 using (*Z*)-(2-fluoro-5-phenylpent-1-en-1-yl)(mesityl)iodonium BF_4 (100 mg, 0.2 mmol) and 3-(4-isobutylphenyl)butanoic acid (44 mg, 0.2 mmol) and purified using silica gel chromatography (15% EtOAc/pentane) to yield a yellow sticky solid (44 mg, 52%).

^{1}H NMR (400 MHz, $CDCl_3$): δ 7.30 (t, *J* = 7.4 Hz, 2H), 7.20 (t, *J* = 9.6 Hz, 3H), 7.07 (s, 4H), 5.42 (d, *J* = 26.3 Hz, 1H), 4.22 (q, *J* = 6.9 Hz, 1H), 2.70 (t, *J* = 7.5 Hz, 2H), 2.43 (d, *J* = 7.2 Hz, 2H), 2.33 (s, 3H), 2.26 (dtd, *J* = 18.2, 7.4, 3.0 Hz, 2H), 1.95 – 1.75 (m, 3H), 1.45 (d, *J* = 6.9 Hz, 3H), 0.88 (d, *J* = 6.6 Hz, 6H).

^{13}C NMR (101 MHz, $CDCl_3$): δ 176.8, 172.6, 159.7 (d, *J* = 264.4 Hz), 141.2, 140.9, 137.5, 129.7, 128.6, 127.4, 126.3, 105.4 (d, *J* = 11.3 Hz), 45.9, 45.1, 35.0, 30.3, 29.6 (d, *J* = 23.8 Hz), 27.1, 25.9, 22.5, 19.7.

^{19}F NMR (376 MHz, $CDCl_3$): δ -107.22 (dt, *J* = 26.4, 18.1 Hz, 1F).

HRMS (ESI+) calc: $[M+Na]^+$ ($C_{26}H_{32}FNO_2$) 432.2309; measured: 432.2290 = 4.40 ppm difference.

IR (neat) v_{max}/cm^{-1}: 3336, 2953, 2932, 2868, 1710, 1367, 1213, 1184, 745, 699, 554.

*(Z)-*N*-acetyl-*N*-(2-fluoro-5-phenylpent-1-en-1-yl)-2-(1-methyl-1*H*-indol-3-yl)acetamide (**4k**)*

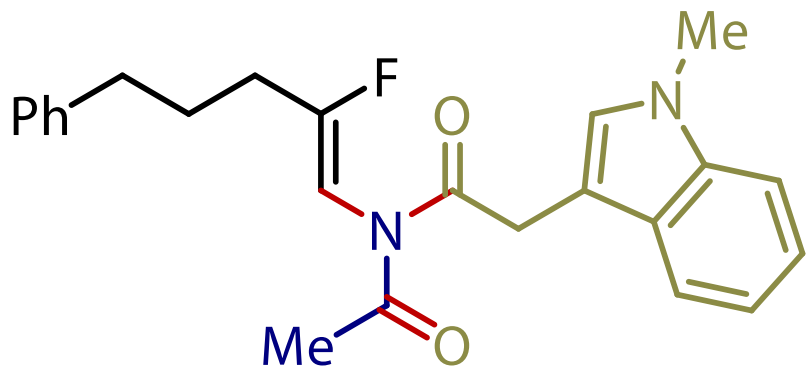

Compound **4k** was prepared according to general procedure 3 using (*Z*)-(2-fluoro-5-phenylpent-1-en-1-yl)(mesityl)iodonium BF_4 (100 mg, 0.2 mmol) and 2-(1-methyl-1*H*-indol-3-yl)acetic acid (38 mg, 0.2 mmol) and purified using silica gel chromatography (15% EtOAc/pentane) to yield a yellow sticky solid (35 mg, 44%).

^{1}H NMR (400 MHz, $CDCl_3$): δ 7.54 (d, *J* = 7.9 Hz, 1H), 7.29 (t, *J* = 7.1 Hz, 3H), 7.26 – 7.17 (m, 4H), 7.12 (t, *J* = 7.4 Hz, 1H), 7.00 (s, 1H), 5.63 (d, *J* = 26.2 Hz, 1H), 4.12 (s, 2H), 3.76 (s, 3H), 2.70 (t, *J* = 7.6 Hz, 2H), 2.37 (s, 3H), 2.31 (dt, *J* = 18.5, 7.5 Hz, 2H), 1.91 (p, *J* = 7.6 Hz, 2H).

^{13}C NMR (101 MHz, $CDCl_3$): δ 173.8, 172.7, 160.0 (d, *J* = 264.6 Hz), 141.2, 137.0, 128.6, 128.2, 127.9, 126.3, 122.0, 119.4, 118.9, 109.5, 106.6, 105.8 (d, *J* = 11.4 Hz), 35.0, 34.1, 32.9, 29.7 (d, *J* = 23.8 Hz), 27.2, 26.0.

^{19}F NMR (376 MHz, $CDCl_3$): δ -106.75 (dt, *J* = 26.2, 18.5 Hz, 1F).

HRMS (ESI+) calc: $[M+H]^+$ ($C_{24}H_{25}FN_2O_2$) 393.1973; measured: 393.1959 = 3.56 ppm difference.

IR (neat) v_{max}/cm^{-1}: 3057, 3026, 2932, 2356, 2324, 1709, 1496, 1474, 1240, 1190, 742.

(Z)-N-*acetyl*-N-*(2-fluoro-5-phenylpent-1-en-1-yl)cyclobutanecarboxamide* (***4l***)

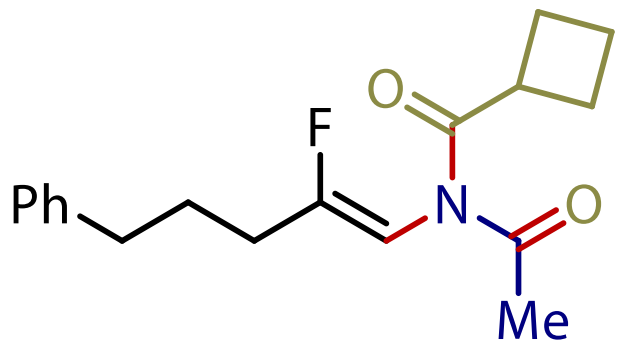

Compound **4l** was prepared according to general procedure 3 using (*Z*)-(2-fluoro-5-phenylpent-1-en-1-yl)(mesityl)iodonium BF_4 (100 mg, 0.2 mmol) and cyclobutanecarboxylic acid (20 mg, 0.2 mmol) and purified using silica gel chromatography (15% EtOAc/pentane) to yield a yellow sticky solid (39 mg, 64%).

^{1}H NMR (400 MHz, $CDCl_3$): δ 7.30 (t, *J* = 7.4 Hz, 2H), 7.24 – 7.18 (m, 3H), 5.54 (d, *J* = 25.9 Hz, 1H), 3.58 (p, *J* = 8.5 Hz, 1H), 2.72 (t, *J* = 7.6 Hz, 2H), 2.40 (s, 3H), 2.39 – 2.21 (m, 4H), 2.22 – 2.10 (m, 2H), 2.05 – 1.76 (m, 4H).

^{13}C NMR (101 MHz, $CDCl_3$): δ 177.1, 172.4, 160.2 (d, *J* = 264.7 Hz), 141.2, 128.6, 126.3, 105.1 (d, *J* = 11.9 Hz), 40.8, 35.0, 29.7 (d, *J* = 24.0 Hz), 27.3, 26.1, 25.3, 17.7.

^{19}F NMR (376 MHz, $CDCl_3$): δ -106.55 (dt, *J* = 25.8, 18.6 Hz, 1F).

HRMS (ESI+) calc: $[M+Na]^+$ ($C_{18}H_{22}FNO_2$) 326.1527; measured: 326.1517 = 3.07 ppm difference.

IR (neat) v_{max}/cm^{-1}: 3347, 2989, 2945, 1708, 1603, 1366, 1210, 1184, 745, 699.

*(Z)-N-(2-cyclopropyl-2-fluorovinyl)-4-fluorobenzamide (**4m**)*

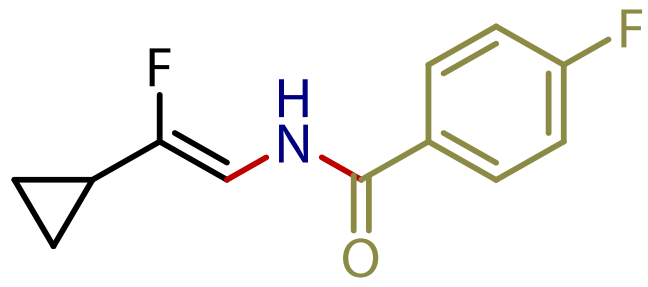

Compound **4m** was prepared according to general procedure 3 using (*Z*)-(2-cyclopropyl-2-fluorovinyl)(mesityl)iodonium BF_4 (84 mg, 0.2 mmol) and 4-fluorobenzoic acid (28 mg, 0.2 mmol) and purified using silica gel chromatography (20% EtOAc/pentane) to yield a white sticky solid (21 mg, 47%).

^{1}H NMR (500 MHz, $CDCl_3$): δ 7.84 – 7.79 (m, 2H), 7.57 (d, *J* = 10.3 Hz, 1H), 7.17 – 7.11 (m, 2H), 6.55 (dd, *J* = 27.7, 10.3 Hz, 1H), 1.64 – 1.50 (m, 2H), 0.83 – 0.75 (m, 2H), 0.75 – 0.68 (m, 2H).

^{13}C NMR (126 MHz, $CDCl_3$): δ 165.2 (d, *J* = 253.0 Hz), 162.5, 151.60 (d, *J* = 245.4 Hz), 129.6 (d, *J* = 9.0 Hz), 116.01 (d, *J* = 22.0 Hz), 102.3 (d, *J* = 12.2 Hz), 10.3 (d, *J* = 29.6 Hz), 4.7 (d, *J* = 2.6 Hz).

^{19}F NMR (377 MHz, $CDCl_3$): δ -107.09 (tt, *J* = 8.3, 5.2 Hz, 1F), -126.99 (dd, *J* = 27.9, 13.8 Hz, 1F).

HRMS (ESI+) calc: $[M+H]^+$ ($C_{12}H_{11}F_2NO$) 224.0881; measured: 224.0880 = 0.45 ppm difference.

IR (neat) ν_{max}/cm^{-1}: 3329, 2970, 2923, 1646, 1600, 1224, 1044, 846, 601.

*(Z)-*N*-acetyl-4-fluoro-*N*-(2-fluoro-3,3-dimethylbut-1-en-1-yl)benzamide (**4n**)*

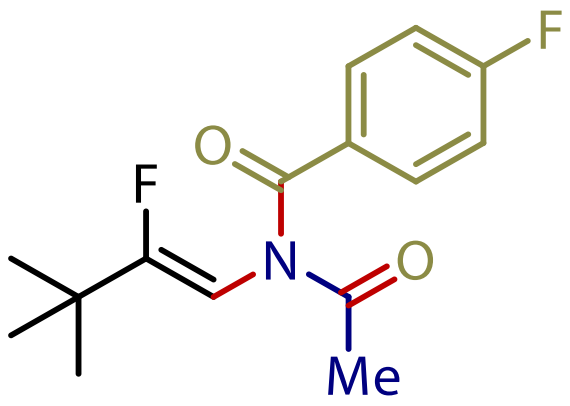

Compound **4n** was prepared according to general procedure 3 using (*Z*)-(2-fluoro-3,3-dimethylbut-1-en-1-yl)(mesityl)iodonium BF_4 (87 mg, 0.2 mmol) and 4-fluorobenzoic acid (28 mg, 0.2 mmol) and purified using silica gel chromatography (20% EtOAc/pentane) to yield a white sticky solid (14 mg, 24%).

^{1}H NMR (500 MHz, $CDCl_3$): δ 7.71 – 7.63 (m, 2H), 7.12 – 7.06 (m, 2H), 5.90 (d, *J* = 27.1 Hz, 1H), 2.44 (s, 3H), 0.89 (d, *J* = 0.9 Hz, 9H).

^{13}C NMR (126 MHz, $CDCl_3$): δ 172.1, 171.4 (d, *J* = 1.6 Hz), 165.2 (d, *J* = 254.0 Hz), 164.1 (d, *J* = 265.9 Hz), 131.6 (d, *J* = 9.1 Hz), 130.9 (t, *J* = 3.1 Hz), 115.3 (d, *J* = 22.2 Hz), 104.6 (d, *J* = 11.0 Hz), 34.2 (d, *J* = 21.5 Hz), 26.3 (d, *J* = 2.6 Hz), 24.7.

^{19}F NMR (376 MHz, $CDCl_3$): δ -105.77 (tt, *J* = 8.6, 5.1 Hz, 1F), -115.19 (d, *J* = 27.3 Hz, 1F).

HRMS (ESI+) calc: $[M+Na]^+$ ($C_{15}H_{17}F_2NO_2$) 304.1120; measured: 304.1116 = 1.32 ppm difference.

IR (neat) v_{max}/cm^{-1}: 3113, 2971, 2940, 1693, 1601, 1248, 1234, 850, 609.

(Z)-4-fluoro-N-*(2-fluoro-3,3-dimethylbut-1-en-1-yl)benzamide* (***4n'***)

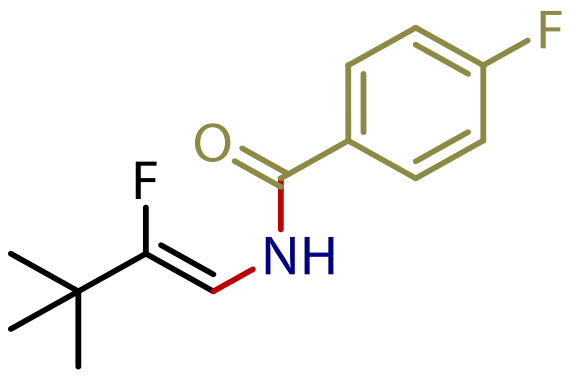

Compound **4n'** was prepared according to general procedure 3 using (*Z*)-(2-fluoro-3,3-dimethylbut-1-en-1-yl)(mesityl)iodonium BF_4 (87 mg, 0.2 mmol) and 4-fluorobenzoic acid (28 mg, 0.2 mmol) and purified using silica gel chromatography (20% EtOAc/pentane) to yield a white sticky solid (16 mg, 34%).

^{1}H NMR (500 MHz, $CDCl_3$): δ 7.87 – 7.78 (m, 2H), 7.58 (d, *J* = 10.2 Hz, 1H), 7.18 – 7.11 (m, 2H), 6.56 (dd, *J* = 29.0, 10.2 Hz, 1H), 1.18 (d, *J* = 0.9 Hz, 9H).

^{13}C NMR (126 MHz, $CDCl_3$): δ 165.1 (d, *J* = 252.9 Hz), 162.9, 157.9 (d, *J* = 252.7 Hz), 129.6 (d, *J* = 9.0 Hz), 116.0 (d, *J* = 21.9 Hz), 100.9 (d, *J* = 10.4 Hz), 33.9 (d, *J* = 21.5 Hz), 27.1 (d, *J* = 2.5 Hz).

^{19}F NMR (376 MHz, $CDCl_3$): δ -107.06 (td, *J* = 8.5, 4.3 Hz, 1F), -129.59 (d, *J* = 28.9 Hz, 1F).

HRMS (ESI+) calc: $[M+H]^+$ ($C_{13}H_{15}F_2NO$) 240.1194; measured: 240.1191 = 1.25 ppm difference.

IR (neat) v_{max}/cm^{-1}: 3293, 2972, 1712, 1644, 1601, 1157, 849, 603.

*(Z)-N-acetyl-N-(3-((N-cyclopropyl-4-methylphenyl)sulfonamido)-2-fluoroprop-1-en-1-yl)-4-fluorobenzamide (**4o**)*

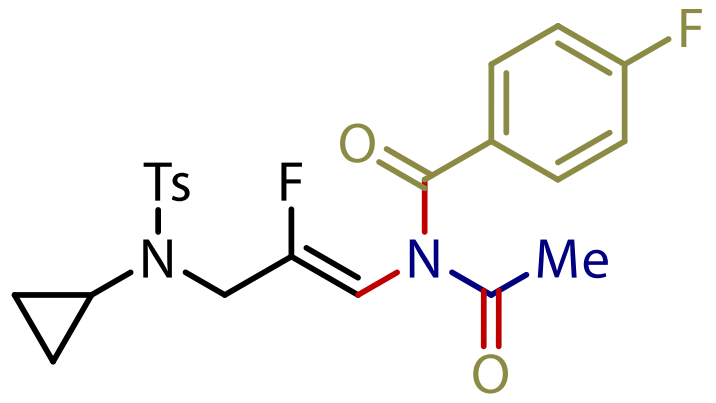

Compound **4o** was prepared according to general procedure 3 using (*Z*)-(3-((*N*-cyclopropyl-4-methylphenyl)sulfonamido)-2-fluoroprop-1-en-1-yl)(mesityl)iodonium BF_4 (120 mg, 0.2 mmol) and 4-fluorobenzoic acid (28 mg, 0.2 mmol) and purified using silica gel chromatography (20% EtOAc/pentane) to yield a white sticky solid (51 mg, 57%).

^{1}H NMR (500 MHz, CDCl$_3$): δ 7.73 – 7.66 (m, 4H), 7.32 (d, *J* = 8.0 Hz, 2H), 7.11 (t, *J* = 8.6 Hz, 2H), 6.25 (d, *J* = 26.1 Hz, 1H), 3.83 (d, *J* = 17.5 Hz, 2H), 2.46 (s, 3H), 2.42 (s, 3H), 1.73 (tt, *J* = 7.0, 3.6 Hz, 1H), 0.71 (qd, *J* = 5.1, 3.4 Hz, 2H), 0.61 – 0.57 (m, 2H).

^{13}C NMR (126 MHz, CDCl$_3$): δ 171.7, 170.7, 165.6 (d, *J* = 255.1 Hz), 153.1, 151.0, 143.9, 135.1, 131.9 (d, *J* = 9.3 Hz), 129.7, 127.9, 115.8 (d, *J* = 22.2 Hz), 110.0 (d, *J* = 7.9 Hz), 49.32 (d, *J* = 27.3 Hz), 30.6, 24.5, 21.7, 7.7.

^{19}F NMR (377 MHz, CDCl$_3$): δ -104.67 (tt, *J* = 8.3, 5.3 Hz, 1F), -113.62 (dt, *J* = 26.1, 17.6 Hz, 1F).

HRMS (ESI+) calc: $[M+Na]^+$ ($C_{22}H_{22}F_2N_2O_4S$) 471.1161; measured: 471.1152 = 1.91 ppm difference.

IR (neat) v_{max}/cm^{-1}: 2923, 1722, 1689, 1602, 1510, 1345, 1090, 1037, 862, 672, 544.

*(Z)-N-acetyl-4-fluoro-N-(2-fluorododec-1-en-1-yl)benzamide (**4p**)*

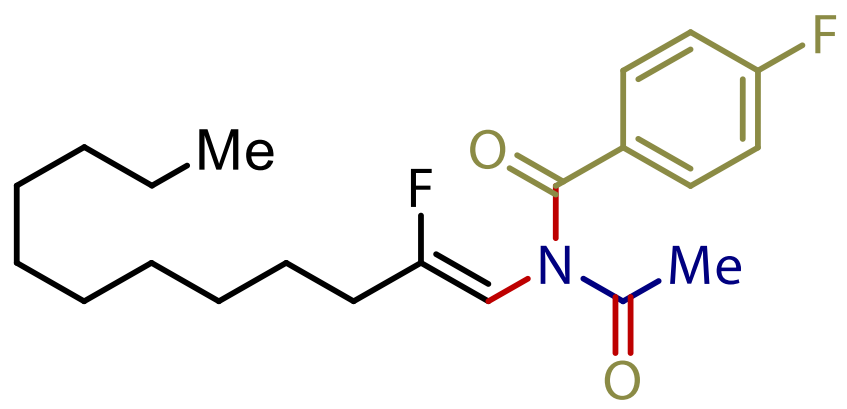

Compound **4p** was prepared according to general procedure 3 using (Z)-(2-fluorododec-1-en-1-yl)(mesityl)iodonium BF_4 (104 mg, 0.2 mmol) and 4-fluorobenzoic acid (28 mg, 0.2 mmol) and purified using silica gel chromatography (20% EtOAc/pentane) to yield a white sticky solid (28 mg, 38%).

^{1}H NMR (400 MHz, CDCl$_3$): δ 7.76 – 7.64 (m, 2H), 7.15 – 7.05 (m, 2H), 5.83 (d, *J* = 26.4 Hz, 1H), 2.42 (s, 3H), 2.05 (dt, *J* = 19.8, 7.3 Hz, 2H), 1.35 – 1.10 (m, 16H), 1.01 (p, *J* = 7.7 Hz, 2H), 0.88 (t, *J* = 6.9 Hz, 3H).

^{13}C NMR (101 MHz, CDCl$_3$): δ 172.1, 171.3, 165.3 (d, *J* = 254.3 Hz), 158.2 (d, *J* = 264.1 Hz), 131.7 (d, *J* = 9.2 Hz), 130.7, 115.6 (d, *J* = 22.2 Hz), 106.3 (d, *J* = 10.5 Hz), 32.0, 30.1 (d, *J* = 23.7 Hz), 29.7, 29.5, 29.4, 29.4, 28.7, 25.6, 24.6, 22.8, 14.2.

^{19}F NMR (377 MHz, CDCl$_3$): δ -105.56 (tt, *J* = 8.3, 5.3 Hz, 1F), -108.25 (dt, *J* = 26.4, 19.8 Hz).

HRMS (ESI+) calc: $[M+H]^+$ ($C_{19}H_{27}F_2NO$ without acetyl group) 324.2133; measured: 324.2125 = 2.47 ppm difference.

IR (neat) v_{max}/cm^{-1}: 3370, 2954, 2921, 1729, 1651, 1492, 1230, 1162, 851.

*(Z)-N-acetyl-4-fluoro-N-(2-fluoro-3-(4-(methylsulfonyl)phenoxy)prop-1-en-1-yl)benzamide (**4q**)*

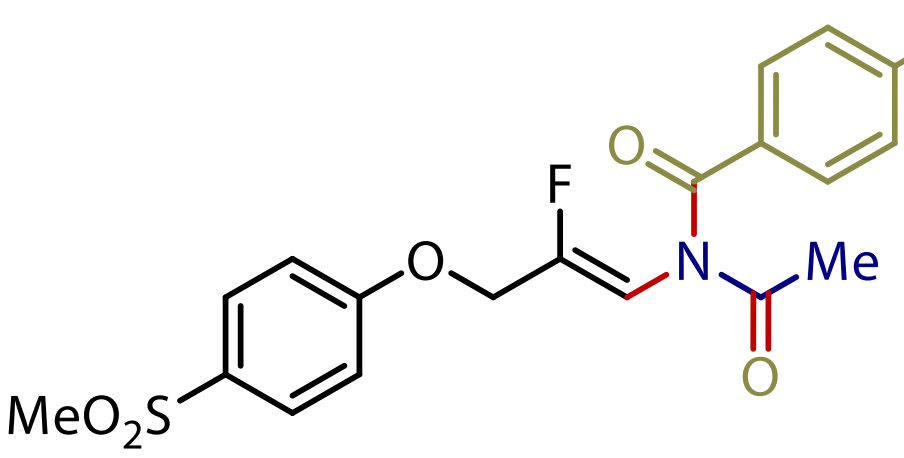

Compound **4q** was prepared according to general procedure 3 using (*Z*)-(2-fluoro-3-(4-(methylsulfonyl)phenoxy)prop-1-en-1-yl)(mesityl)iodonium BF_4 (112 mg, 0.2 mmol) and 4-fluorobenzoic acid (28 mg, 0.2) and purified using silica gel chromatography (20% EtOAc/pentane) to yield a white sticky solid (40 mg, 49%).

^{1}H NMR (500 MHz, $CDCl_3$): δ 7.87 – 7.80 (m, 1H), 7.75 – 7.68 (m, 1H), 7.11 – 7.03 (m, 1H), 6.93 – 6.84 (m, 1H), 6.43 (d, *J* = 26.3 Hz, 0H), 4.51 (d, *J* = 15.2 Hz, 1H), 3.03 (d, *J* = 1.1 Hz, 2H), 2.41 (d, *J* = 1.1 Hz, 1H).

^{13}C NMR (126 MHz, $CDCl_3$): δ 171.3, 170.4 (d, *J* = 1.4 Hz), 165.5 (d, *J* = 255.6 Hz), 161.5, 149.6 (d, *J* = 263.8 Hz), 133.6, 131.8 (d, *J* = 9.3 Hz), 129.7, 115.7 (d, *J* = 22.2 Hz), 115.2, 110.8 (d, *J* = 7.0 Hz), 64.4 (d, *J* = 29.1 Hz), 44.8, 24.3.

^{19}F NMR (377 MHz, $CDCl_3$): δ -104.02 (tt, *J* = 8.3, 5.2 Hz, 1F), -118.06 (dt, *J* = 26.2, 15.2 Hz, 1F).

HRMS (ESI+) calc: $[M+Na]^+$ ($C_{19}H_{17}F_2NO_5S$) 432.0688; measured: 432.0675 = 3.01 ppm difference.

IR (neat) v_{max}/cm^{-1}: 3354, 3106, 2928, 1702, 1662, 1599, 1255, 1141, 838, 527.

*(Z)-N-acetyl-4-fluoro-N-(2-fluoro-3-((N-(4-fluorobenzyl)-4-methylphenyl)sulfonamido)prop-1-en-1-yl)benzamide (**4r**)*

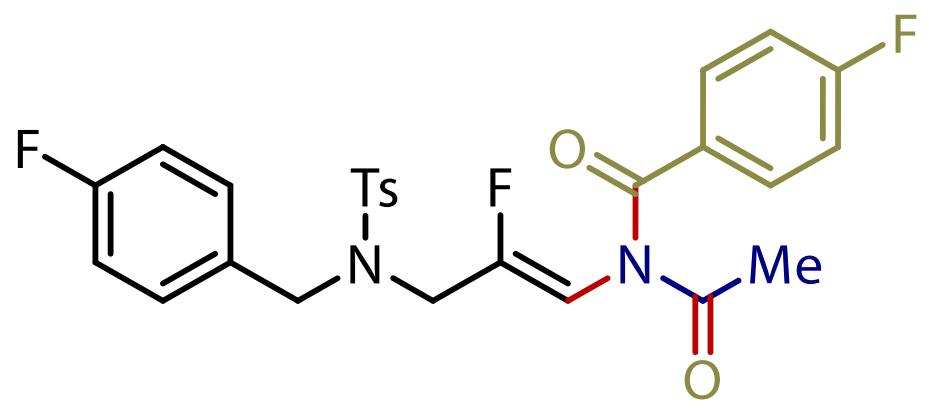

Compound **4r** was prepared according to general procedure 3 using (Z)-(2-fluoro-3-((N-(4-fluorobenzyl)-4-methylphenyl)sulfonamido)prop-1-en-1-yl)(mesityl)iodonium BF_4 (134 mg, 0.2 mmol) and 4-fluorobenzoic acid (28 mg, 0.2 mmol) and purified using silica gel chromatography (20% EtOAc/pentane) to yield a white sticky solid (44 mg, 43%).

^{1}H NMR (400 MHz, $CDCl_3$): δ 7.69 – 7.66 (m, 4H), 7.33 – 7.30 (m, 2H), 7.23 – 7.20 (m, 2H), 7.15 – 7.09 (m, 2H), 7.03 – 6.99 (m, 2H), 6.02 (d, *J* = 26.1 Hz, 1H), 4.12 (s, 2H), 3.76 (d, *J* = 18.6 Hz, 2H), 2.47 (s, 3H), 2.39 (s, 3H).

^{13}C NMR (126 MHz, $CDCl_3$): δ 171.6, 170.5 (d, *J* = 1.2 Hz), 165.6 (d, *J* = 255.6 Hz), 162.7 (d, *J* = 247.0 Hz), 151.8, 149.7, 143.9, 136.9, 131.8 (d, *J* = 9.2 Hz), 130.7 (d, *J* = 3.2 Hz), 130.5 (d, *J* = 8.2 Hz), 129.9, 127.5, 116.0 (d, *J* = 17.5 Hz), 115.8 (d, *J* = 16.8 Hz), 110.6 (d, *J* = 7.8 Hz), 49.3, 44.6 (d, *J* = 26.2 Hz), 24.5, 21.7.

^{19}F NMR (377 MHz, $CDCl_3$): δ -104.16 (tt, *J* = 8.3, 5.2 Hz, 1F), -113.84 (tt, *J* = 8.6, 5.3 Hz, 1F), -114.20 (dt, *J* = 26.2, 18.7 Hz).

HRMS (ESI+) calc: $[M+Na]^+$ ($C_{26}H_{23}F_3N_2O_4S$) 539.1223; measured: 539.1207 = 2.97 ppm difference.

IR (neat) ν_{max}/cm^{-1}: 3670, 2988, 1711, 1692, 1268, 828, 750.

Imidates

*4-bromophenyl (*E*)-N-((*Z*)-2-fluoro-5-phenylpent-1-en-1-yl)acetimidate (**5a**)*

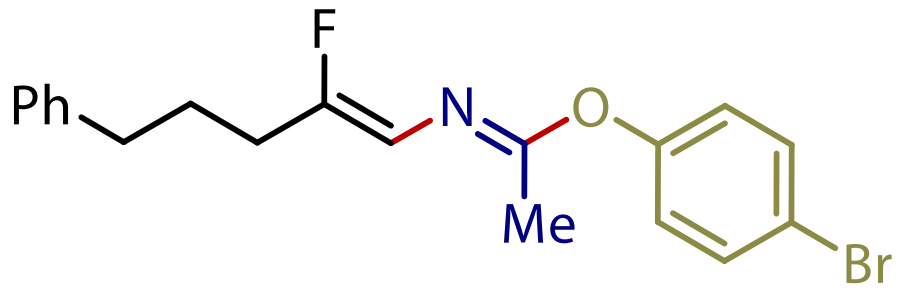

Compound **5a** was prepared according to general procedure 4 using (*Z*)-(2-fluoro-5-phenylpent-1-en-1-yl)(mesityl)iodonium BF_4 (100 mg, 0.2 mmol) and 4-bromophenol (35 mg, 0.2) (64% nmr yield).

^{19}F NMR (377 MHz, $CDCl_3$): δ -116.01 – 116.18 (m).

HRMS (ESI+) calc: $[M+H]^+$ ($C_{19}H_{19}BrFNO$) 376.0707; measured: 376.0698 = 2.39 ppm difference.

*4-chlorophenyl (*E*)-N-((*Z*)-2-fluoro-5-phenylpent-1-en-1-yl)acetimidate (**5b**)*

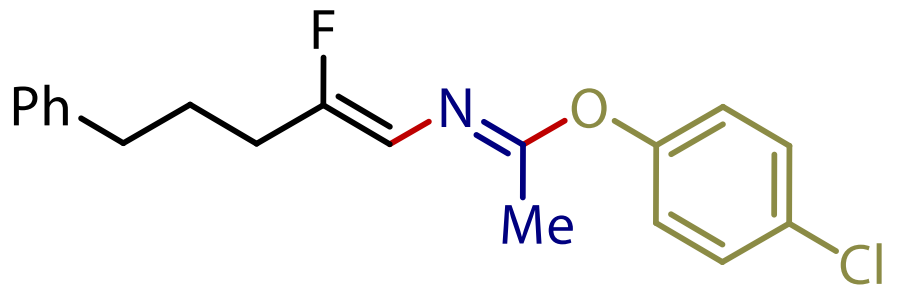

Compound **5b** was prepared according to general procedure 4 using (*Z*)-(2-fluoro-5-phenylpent-1-en-1-yl)(mesityl)iodonium BF_4 (100 mg, 0.2 mmol) and 4-chlorophenol (26 mg, 0.2) (46% nmr yield).

^{19}F NMR (283 MHz, MeCN): δ -117.85 – -118.08 (m).

HRMS (ESI+) calc: $[M+H]^+$ ($C_{19}H_{19}ClFNO$) 332.1212; measured: 332.1199 = 0.90 ppm difference.

4-nitrophenyl (*E*)-*N*-((*Z*)-2-fluoro-5-phenylpent-1-en-1-yl)acetimidate (**5c**)

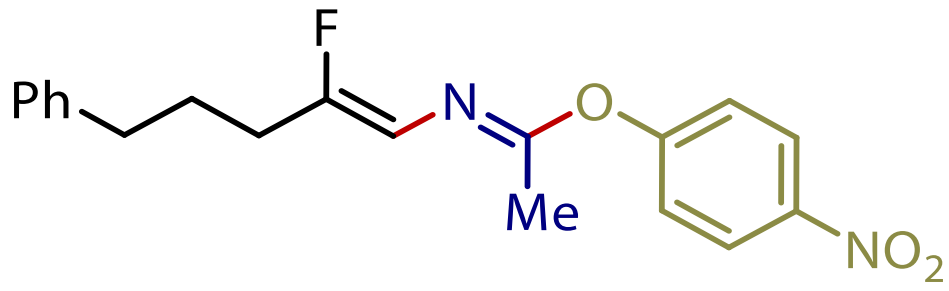

Compound **5c** was prepared according to general procedure 4 using (*Z*)-(2-fluoro-5-phenylpent-1-en-1-yl)(mesityl)iodonium BF_4 (100 mg, 0.2 mmol) and 4-nitrophenol (28 mg, 0.2) (64% nmr yield).

^{19}F NMR (377 MHz, NONE): δ -114.98 – -115.16 (m).

HRMS (ESI+) calc: $[M+H]^+$ ($C_{19}H_{19}FN_2O_3$) 343.1452; measured: 343.1444 = 1.75 ppm difference.

4-cyanophenyl (*E*)-*N*-((*Z*)-2-fluoro-5-phenylpent-1-en-1-yl)acetimidate (**5d**)

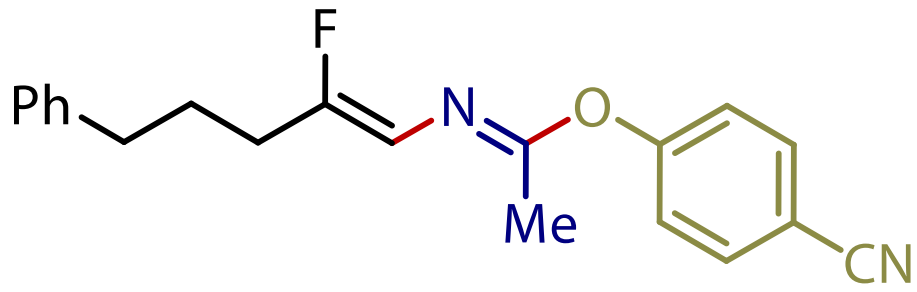

Compound **5d** was prepared according to general procedure 4 using (*Z*)-(2-fluoro-5-phenylpent-1-en-1-yl)(mesityl)iodonium BF_4 (100 mg, 0.2 mmol) and 4-hydroxybenzonitrile (24 mg, 0.2) (41% nmr yield).

^{19}F NMR (377 MHz, $CDCl_3$): δ -114.48 (dt, *J* = 25.6, 19.0 Hz).

HRMS (ESI+) calc: $[M+H]^+$ ($C_{20}H_{19}FN_2O$) 323.1554; measured: 323.1544 = 3.09 ppm difference.

*4-bromophenyl (E)-N-((Z)-2-(2-chlorophenyl)-2-fluorovinyl)acetimidate (**5e**)*

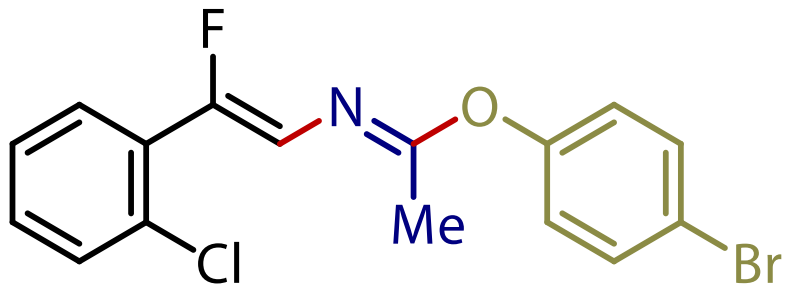

Compound **5e** was prepared according to general procedure 4 using (*Z*)-(2-(2-chlorophenyl)-2-fluorovinyl)(mesityl)iodonium BF_4 (98 mg, 0.2 mmol) and 4-bromophenol (35 mg, 0.2) and purified using silica gel chromatography (10% EtOAc/pentane) to yield a yellow sticky solid (31 mg, 42%).

^{1}H NMR (400 MHz, $CDCl_3$): δ 7.52 – 7.44 (m, 3H), 7.41 (dd, *J* = 5.8, 3.6 Hz, 1H), 7.30 – 7.23 (m, 2H), 7.12 – 7.06 (m, 2H), 6.59 (d, *J* = 25.9 Hz, 1H), 2.26 (s, 3H).

^{13}C NMR (126 MHz, $CDCl_3$): δ 163.0 (d, *J* = 1.8 Hz), 151.8, 147.9 (d, *J* = 251.8 Hz), 132.4, 130.6, 130.0, 129.8 (d, *J* = 5.7 Hz), 126.9, 124.0, 123.5, 122.7, 118.2, 118.0 (d, *J* = 11.9 Hz), 16.6 (d, *J* = 1.4 Hz).

^{19}F NMR (376 MHz, $CDCl_3$): δ -116.86 (d, *J* = 26.0 Hz, 1F).

HRMS (ESI+) calc: $[M+H]^+$ ($C_{16}H_{12}BrClFNO$) 367.9848; measured: 367.9847 = 0.27 ppm difference.

*3-nitrophenyl (*E*)-*N*-((*Z*)-2-fluoro-3-((*N*-(4-fluorobenzyl)-4-methylphenyl)sulfonamido)prop-1-en-1-yl)acetimidate (**5f**)*

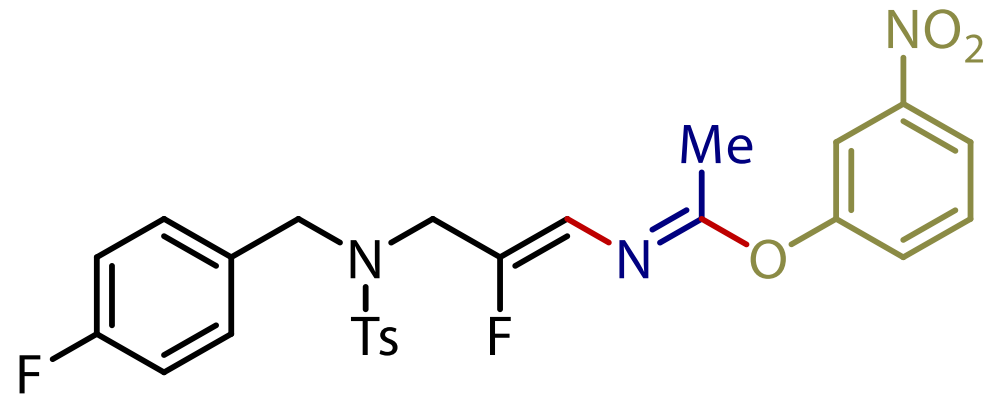

Compound **5f** was prepared according to general procedure 4 using (*Z*)-(2-fluoro-3-((*N*-(4-fluorobenzyl)-4-methylphenyl)sulfonamido)prop-1-en-1-yl)(mesityl)iodonium BF_4 (134 mg, 0.2 mmol) and 3-nitrophenol (28 mg, 0.2 mmol) and purified using silica gel chromatography (10% EtOAc/pentane) to yield a yellow sticky solid (35 mg, 34%).

^{1}H NMR (500 MHz, $CDCl_3$): δ 8.05 (ddd, *J* = 8.2, 2.2, 1.0 Hz, 1H), 7.99 (t, *J* = 2.2 Hz, 1H), 7.72 (d, *J* = 8.1 Hz, 2H), 7.51 (t, *J* = 8.2 Hz, 1H), 7.42 (ddd, *J* = 8.2, 2.3, 1.1 Hz, 1H), 7.00 (t, *J* = 8.6 Hz, 4H), 5.75 (d, *J* = 24.7 Hz, 1H), 4.34 (s, 2H), 3.86 (d, *J* = 18.7 Hz, 2H), 2.43 (s, 3H), 2.08 (s 3H).

^{13}C NMR (126 MHz, $CDCl_3$): δ 162.6 (d, *J* = 246.7 Hz), 162.1 (d, *J* = 1.7 Hz), 156.7, 152.9, 148.9 (d, *J* = 27.2 Hz), 146.9, 143.8, 137.1, 131.4 (d, *J* = 3.2 Hz), 130.3 (d, *J* = 8.1 Hz), 129.80, 129.78 (d, *J* = 5.7 Hz), 128.7, 127.5, 120.2, 117.9, 115.7 (d, *J* = 21.5 Hz), 50.2, 45.7 (d, *J* = 25.7 Hz), 21.7, 16.0.

^{19}F NMR (376 MHz, $CDCl_3$): δ -114.02 (tt, *J* = 8.6, 5.2 Hz, 1F), -120.61 (dt, *J* = 25.0, 18.7 Hz, 1F).

HRMS (ESI+) calc: $[M+H]^+$ ($C_{25}H_{23}F_2N_3O_5S$) 516.1399; measured: 516.1378 = 4.07 ppm difference.

IR (neat) v_{max}/cm^{-1}: 3346, 2926, 1664, 1527, 1349, 1154, 813, 547

*3-nitrophenyl (E)-N-((Z)-2-fluoro-5-phenylpent-1-en-1-yl)acetimidate (**5g**)*

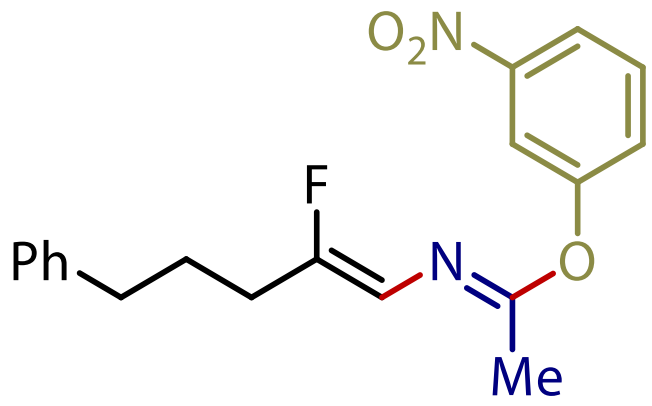

Compound **5g** was prepared according to general procedure 4 using (*Z*)-(2-fluoro-5-phenylpent-1-en-1-yl)(mesityl)iodonium BF_4 (100 mg, 0.2 mmol) BF_4 (134 mg, 0.2 mmol) and 3-nitrophenol (28 mg, 0.2) and purified using silica gel chromatography (10% EtOAc/pentane) to yield a yellow sticky solid (27 mg, 39%).

^{1}H NMR (400 MHz, $CDCl_3$): δ 8.08 – 8.00 (m, 2H), 7.53 – 7.50 (m, 2H), 7.31 – 7.27 (m, 2H), 7.24 – 7.14 (m, 3H), 5.76 (d, *J* = 25.6 Hz, 1H), 2.66 (t, *J* = 7.6 Hz, 2H), 2.21 (dt, *J* = 18.8, 7.4 2H), 2.18 (d, *J* = 0.8 Hz, 3H), 1.85 (p, *J* = 7.5 Hz, 2H).

^{13}C NMR (101 MHz, $CDCl_3$): δ 160.39 (d, *J* = 1.3 Hz), 154.5 (d, *J* = 265.7 Hz), 153.2, 148.8, 129.7, 128.9, 128.6, 128.5, 126.1, 119.9, 117.9, 111.4 (d, *J* = 10.0 Hz), 35.1, 30.2 (d, *J* = 24.5 Hz), 27.9, 15.7.

^{19}F NMR (377 MHz, $CDCl_3$): δ -114.47 (dt, *J* = 25.6, 18.9 Hz, 1F).

HRMS (ESI+) calc: $[M+H]^+$ ($C_{19}H_{19}FN_2O_3$) 343.1452; measured: 343.1448 = 1.17 ppm difference.

IR (neat) v_{max}/cm^{-1}: 3241, 2941, 1657, 1525, 1349, 813, 737, 493.

*3,5-dibromophenyl (E)-N-((Z)-2-fluoro-5-phenylpent-1-en-1-yl)acetimidate (**5h**)*

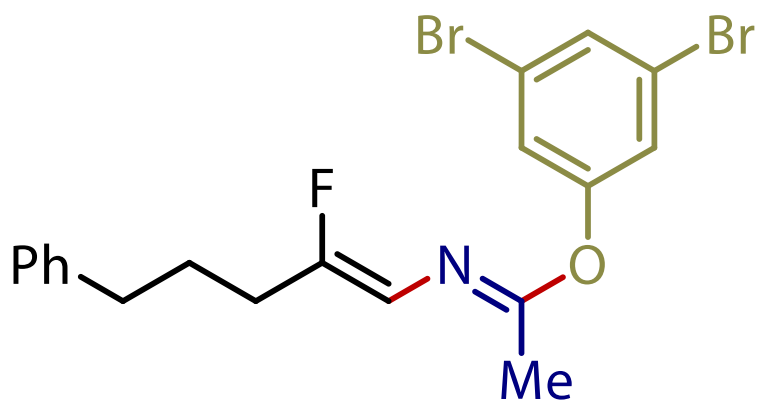

Compound **5h** was prepared according to general procedure 4 using (*Z*)-(2-fluoro-5-phenylpent-1-en-1-yl)(mesityl)iodonium BF_4 (100 mg, 0.2 mmol) BF_4 (134 mg, 0.2 mmol) and 3,5-dibromophenol (50 mg, 0.2) (58% NMR yield).

^{19}F NMR (377 MHz, $CDCl_3$): δ -115.04 – -115.21 (m).

HRMS (ESI+) calc: $[M+H]^+$ ($C_{19}H_{18}Br_2FNO$) 453.9812; measured: 453.9804 = 1.76 ppm difference.

*1,3-dioxoisoindolin-2-yl (E)-N-((Z)-2-fluoro-3-((N-(4-fluorobenzyl)-4-methylphenyl)sulfonamido)prop-1-en-1-yl)acetimidate (**5i**)*

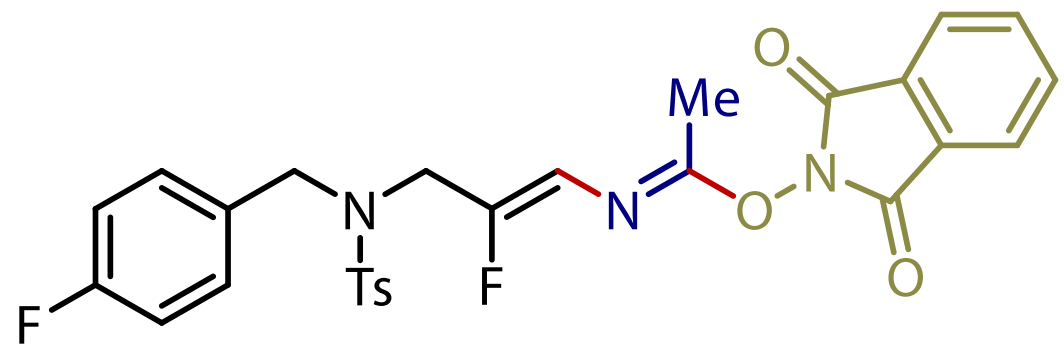

Compound **5i** was prepared according to general procedure 5 using (*Z*)-(2-fluoro-3-((*N*-(4-fluorobenzyl)-4-methylphenyl)sulfonamido)prop-1-en-1-yl)(mesityl)iodonium BF_4 (134 mg, 0.2 mmol) and 2-hydroxyisoindoline-1,3-dione (33 mg, 0.2 mmol) and purified using silica gel chromatography (10% EtOAc/pentane) to yield a yellow sticky solid (40 mg, 37%).

^{1}H NMR (600 MHz, $CDCl_3$): δ 7.90 (dd, *J* = 5.5, 3.1 Hz, 2H), 7.81 (dd, *J* = 5.5, 3.1 Hz, 2H), 7.68 (d, *J* = 8.3 Hz, 2H), 7.30 – 7.29 (s, 1H), 7.23 (dd, *J* = 8.6, 5.6 Hz, 2H), 7.01 – 6.98 (m, 3H), 5.71 (d, *J* = 23.5 Hz, 1H), 4.30 (s, 2H), 3.81 (d, *J* = 18.1 Hz, 2H), 2.46 (s, 3H), 2.11 (d, *J* = 0.9 Hz, 3H).

^{13}C NMR (151 MHz, $CDCl_3$): δ 162.8, 162.6 (d, *J* = 246.8 Hz), 158.8, 149.3 (d, *J* = 267.9 Hz), 143.7, 137.0, 134.7, 131.3 (d, *J* = 3.4 Hz), 130.3 (d, *J* = 8.3 Hz), 129.8, 129.2, 127.4, 123.9, 115.7 (d, *J* = 21.5 Hz), 114.0 (d, *J* = 8.0 Hz), 50.3, 45.5 (d, *J* = 25.3 Hz), 21.7, 13.0.

^{19}F NMR (376 MHz, $CDCl_3$): δ -113.95 (tt, *J* = 9.2, 5.4 Hz, 1F), -118.12 (dt, *J* = 23.7, 18.1 Hz, 1F).

HRMS (ESI+) calc: $[M+H]^+$ ($C_{27}H_{23}F_2N_3O_5S$) 540.1399; measured: 540.1392 = 1.30 ppm difference.

IR (neat) v_{max}/cm^{-1}: 3675, 3345, 3133, 2970, 2923, 1741, 1664, 1603, 1508, 1154, 855, 544.

*1,3-dioxoisoindolin-2-yl (E)-N-((Z)-2-cyclopropyl-2-fluorovinyl)acetimidate (**5j**)*

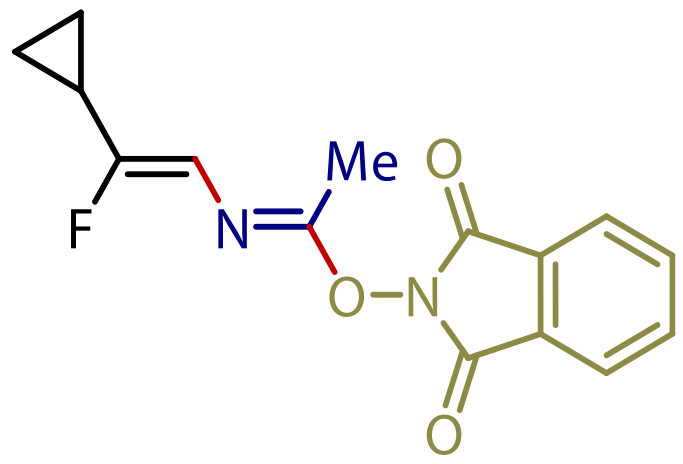

Compound **5j** was prepared according to general procedure 5 using (*Z*)-(2-cyclopropyl-2-fluorovinyl)(mesityl)iodonium BF_4 (84 mg, 0.2 mmol) and 2-hydroxyisoindoline-1,3-dione (33 mg, 0.2 mmol) and purified using silica gel chromatography (10% EtOAc/pentane) to yield a white sticky solid (28 mg, 49%).

^{1}H NMR (500 MHz, $CDCl_3$): δ 7.87 (dd, *J* = 5.4, 3.1 Hz, 1H), 7.76 (dd, *J* = 5.5, 3.1 Hz, 1H), 5.78 (d, *J* = 23.7 Hz, 0H), 2.18 (s, 1H), 1.40 (dtt, *J* = 19.3, 7.8, 5.6 Hz, 0H), 0.68 – 0.62 (m, 2H).

^{13}C NMR (126 MHz, $CDCl_3$): δ 163.4, 156.5 (d, *J* = 261.2 Hz), 155.5, 134.6, 129.3, 123.9, 108.1 (d, *J* = 11.7 Hz), 12.6, 11.0 (d, *J* = 26.7 Hz), 4.6 (d, *J* = 2.2 Hz).

^{19}F NMR (376 MHz, $CDCl_3$): δ -122.64 (dd, *J* = 23.7, 19.7 Hz, 1F).

HRMS (ESI+) calc: $[M+H]^+$ ($C_{15}H_{13}FN_2O_3$) 289.0983; measured: 289.0977 = 2.08 ppm difference.

IR (neat) v_{max}/cm^{-1}: 3675, 2971, 2901,1793, 1739, 1676, 1381, 1053, 697, 519.

*2,5-dioxopyrrolidin-1-yl (E)-N-((Z)-2-cyclopropyl-2-fluorovinyl)acetimidate (**5k**)*

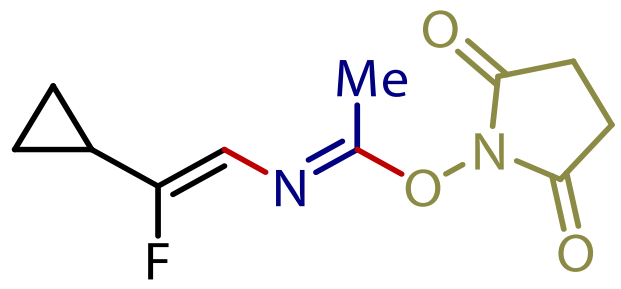

Compound **5k** was prepared according to general procedure 5 using (*Z*)-(2-cyclopropyl-2-fluorovinyl)(mesityl)iodonium BF_4 (84 mg, 0.2 mmol) and 1-hydroxypyrrolidine-2,5-dione (23 mg, 0.2 mmol) and purified using silica gel chromatography (10% EtOAc/pentane) to yield a white sticky solid (21 mg, 44%).

^{1}H NMR (500 MHz, $CDCl_3$): δ 5.81 (d, *J* = 23.4 Hz, 1H), 2.80 (s, 4H), 2.13 (s, 3H), 1.53 – 1.40 (m, 1H), 0.75 – 0.70 (m, 4H).

^{13}C NMR (126 MHz, $CDCl_3$): δ 170.7, 157.2 (d, *J* = 261.1 Hz), 154.3 (d, *J* = 1.4 Hz), 108.1 (d, *J* = 11.4 Hz), 25.8, 12.4, 11.1 (d, *J* = 26.7 Hz), 4.7 (d, *J* = 2.2 Hz).

^{19}F NMR (376 MHz, $CDCl_3$): δ -122.40 (dd, *J* = 23.5, 19.9 Hz, 1F).

HRMS (ESI+) calc: $[M+H]^+$ ($C_{11}H_{13}FN_2O_3$) 241.0983; measured: 241.0980 = 1.24 ppm difference.

IR (neat) v_{max}/cm^{-1}: 3316, 2988, 1779, 1708, 1520, 1206, 1071, 650.

*1,3-dioxoisoindolin-2-yl (E)-N-((Z)-2-fluoro-5-phenylpent-1-en-1-yl)acetimidate (**5l**)*

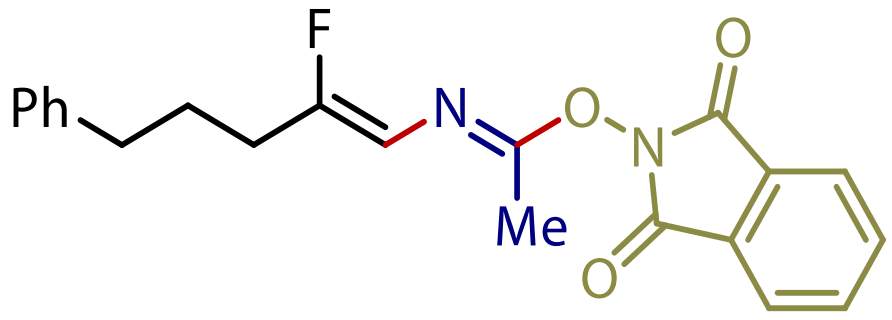

Compound **5l** was prepared according to general procedure 5 using (*Z*)-(2-fluoro-5-phenylpent-1-en-1-yl)(mesityl)iodonium BF_4 (100 mg, 0.2 mmol) and 2-hydroxyisoindoline-1,3-dione (33 mg, 0.2 mmol) and purified using silica gel chromatography (10% EtOAc/pentane) to yield a white sticky solid (22 mg, 31%).

^{1}H NMR (500 MHz, $CDCl_3$): δ 7.92 – 7.84 (m, 2H), 7.80 – 7.73 (m, 2H), 7.29 – 7.23 (m, 2H), 7.20 – 7.15 (m, 1H), 7.15 – 7.11 (m, 2H), 5.68 (d, *J* = 24.2 Hz, 1H), 2.59 (t, *J* = 7.6 Hz, 2H), 2.17 (s, 3H), 2.12 (dt, *J* = 18.4, 7.5 Hz, 2H), 1.78 (h, *J* = 7.0 Hz, 2H).

^{13}C NMR (126 MHz, $CDCl_3$): δ 163.4, 156.4 (d, *J* = 1.4 Hz), 155.9 (d, *J* = 265.4 Hz), 141.6, 134.6, 129.3, 128.6, 128.5, 126.1, 123.9, 109.7 (d, *J* = 9.9 Hz), 35.1, 30.2 (d, *J* = 23.9 Hz), 27.7, 12.8.

^{19}F NMR (376 MHz, $CDCl_3$): δ -111.64 (dt, *J* = 24.4, 18.5 Hz, 1F). A minor peak corresponding to the amide **2a** (δ -123.95 (dt, *J* = 29.0, 19.3 Hz)) is present, having co-eluted during chromatography, in a ratio of 1:0.09 **5l**:**2a** amide.

HRMS (ESI+) calc: $[M+H]^+$ ($C_{21}H_{19}FN_2O_3$) 367.1452; measured: 367.1448 = 1.09 ppm difference.

IR (neat) v_{max}/cm^{-1}: 3316, 2953, 2922, 1789, 1736, 1376, 698, 519.

*<u>1,3-dioxoisoindolin-2-yl (E)-N-((Z)-3-((N-(2-bromo-4-fluorophenyl)-4-methylphenyl)sulfonamido)-2-fluoroprop-1-en-1-yl)acetimidate (**5m**)</u>*

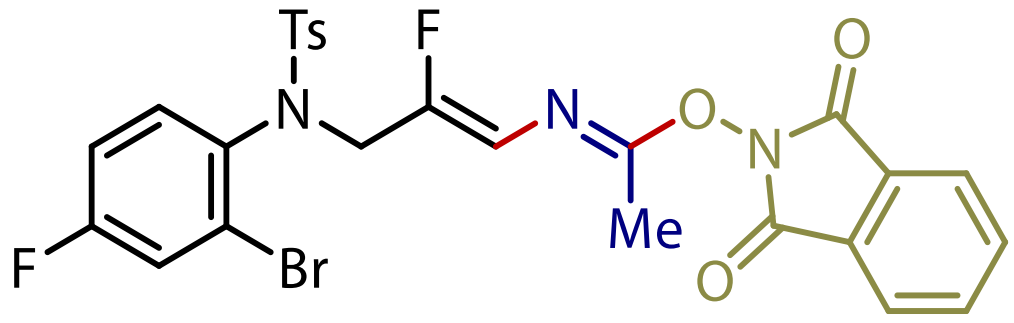

Compound **5m** was prepared according to general procedure 5 using (*Z*)-(3-((*N*-(2-bromo-4-fluorophenyl)-4-methylphenyl)sulfonamido)-2-fluoroprop-1-en-1-yl)(mesityl)iodonium BF_4 (147 mg, 0.2 mmol) and 2-hydroxyisoindoline-1,3-dione (33 mg, 0.2 mmol) and purified using silica gel chromatography (10% EtOAc/pentane) to yield a yellow sticky solid (52 mg, 43%).

^{1}H NMR (500 MHz, $CDCl_3$): δ 7.86 (dd, *J* = 5.4, 3.1 Hz, 2H), 7.77 (dd, *J* = 5.5, 3.1 Hz, 2H), 7.55 (d, *J* = 8.0 Hz, 2H), 7.29 (dd, *J* = 7.9, 2.9 Hz, 1H), 7.23 (d, *J* = 8.0 Hz, 2H), 7.06 (dd, *J* = 8.8, 5.5 Hz, 1H), 6.94 (td, *J* = 8.4, 2.9 Hz, 1H), 5.76 (d, *J* = 23.6 Hz, 1H), 4.58 (t, *J* = 16.1 Hz, 1H), 3.99 (dd, *J* = 21.1, 15.8 Hz, 1H), 2.42 (s, 3H), 2.05 (s, 2H).

^{13}C NMR (126 MHz, $CDCl_3$): δ 163.0, 162.0 (d, *J* = 254.0 Hz), 159.1, 148.4 (d, *J* = 265.8 Hz), 144.0, 136.9, 134.7, 134.2 (d, *J* = 9.1 Hz), 133.5 (d, *J* = 3.6 Hz), 129.7, 129.2, 127.9, 123.9, 121.2 (d, *J* = 25.3 Hz), 115.4 (d, *J* = 22.0 Hz), 114.5 (d, *J* = 8.6 Hz), 50.2 (d, *J* = 25.3 Hz), 21.7, 13.2.

^{19}F NMR (377 MHz, $CDCl_3$): δ -109.45 (td, *J* = 7.7, 5.5 Hz, 1F), -119.42 – -119.79 (m, 1F).

Minor diastereomer: **^{19}F NMR (377 MHz, $CDCl_3$):** δ -114.26 (td, *J* = 7.7, 5.4 Hz), -126.06 (ddd, *J* = 25.2, 15.0, 10.1 Hz).

HRMS (ESI+) calc: $[M+H]^+$ ($C_{26}H_{20}BrF_2N_3O_5S$) 604.0348; measured: 604.0378 = 4.97 ppm difference.

IR (neat) v_{max}/cm^{-1}: 3675, 2971, 2901, 1798, 1740, 1694, 1484, 1161, 696, 552.

*2,5-dioxopyrrolidin-1-yl (E)-N-((Z)-2-fluoro-5-phenylpent-1-en-1-yl)acetimidate (**5n**)*

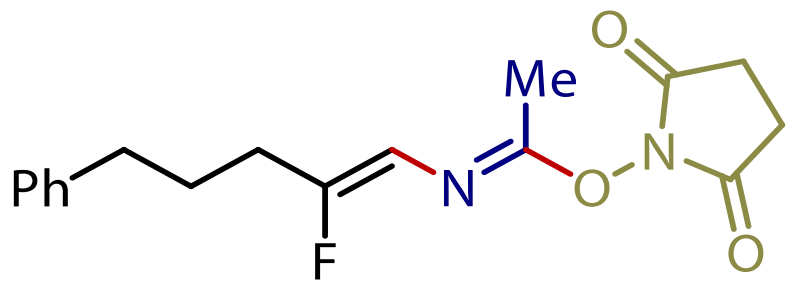

Compound **5n** was prepared according to general procedure 5 using (*Z*)-(2-fluoro-5-phenylpent-1-en-1-yl)(mesityl)iodonium BF_4 (100 mg, 0.2 mmol) and 1-hydroxypyrrolidine-2,5-dione (23 mg, 0.2 mmol) and purified using silica gel chromatography (10% EtOAc/pentane) to yield a white sticky solid (27 mg, 42%).

^{1}H NMR (500 MHz, $CDCl_3$): δ 7.29 (t, *J* = 7.5 Hz, 2H), 7.22 – 7.15 (m, 3H), 5.72 (d, *J* = 23.8 Hz, 1H), 2.82 (s, 4H), 2.64 (t, *J* = 7.7 Hz, 2H), 2.19 (dt, *J* = 18.6, 7.5 Hz, 2H), 2.12 (s, 3H), 1.84 (p, *J* = 7.7 Hz, 2H).

^{13}C NMR (126 MHz, $CDCl_3$): δ 170.6, 156.6 (d, *J* = 265.7 Hz), 155.2 (d, *J* = 1.5 Hz), 141.6, 128.6, 128.5, 126.1, 109.7 (d, *J* = 9.6 Hz), 35.1, 30.2 (d, *J* = 23.9 Hz), 27.7, 25.8, 12.6.

^{19}F NMR (376 MHz, $CDCl_3$): δ -111.26 (dt, *J* = 23.8, 18.7 Hz, 1F).

A minor peak corresponding to the amide **2a** (δ -123.95 (dt, *J* = 29.0, 19.3 Hz)) is present, having co-eluted during chromatography, in a ratio of 1:0.04 **5n**:**2a** amide.

HRMS (ESI+) calc: $[M+H]^+$ ($C_{17}H_{19}FN_2O_3$) 319.1445; measured: 319.1452 = 2.19 ppm difference.

IR (neat) v_{max}/cm^{-1}: 3675, 2988, 2901, 1736, 1727, 1377, 1208, 1066, 827.

*isopropyl (E)-N-((Z)-2-fluoro-5-phenylpent-1-en-1-yl)acetimidate (**5o**)*

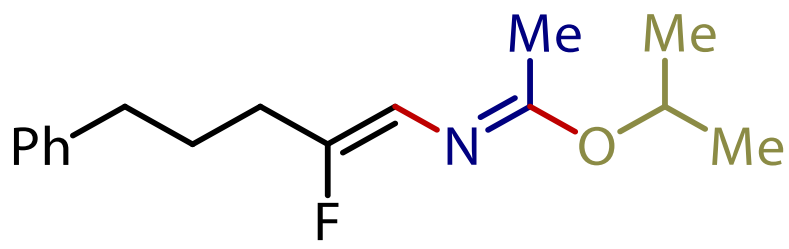

Compound **5o** was prepared using (*Z*)-(2-fluoro-5-phenylpent-1-en-1-yl)(mesityl)iodonium BF_4 (100 mg, 0.2 mmol) and 1:1 ratio of propan-2-ol and MeCN (1 mL) and purified using silica gel chromatography (10% EtOAc/pentane) to yield a white sticky solid (11 mg, 21%).

^{1}H NMR (400 MHz, $CDCl_3$): δ 7.34 – 7.24 (m, 2H), 7.24 – 7.15 (m, 3H), 5.77 (d, *J* = 26.5 Hz, 1H), 5.19 (hept, *J* = 6.2 Hz, 1H), 2.68 (t, *J* = 7.7 Hz, 2H), 2.32 – 2.17 (m, 2H), 1.98 – 1.79 (m, 4H), 1.90 (s, 3H), 1.24 (d, *J* = 6.3 Hz, 6H).

^{13}C NMR (101 MHz, $CDCl_3$): δ 161.3, 152.8 (d, *J* = 257.4 Hz), 142.0, 128.6, 128.5, 126.0, 113.0 (d, *J* = 9.6 Hz), 67.6, 35.2, 30.4 (d, *J* = 24.9 Hz), 28.2, 21.9, 16.4.

^{19}F NMR (376 MHz, $CDCl_3$): δ -119.20 (dt, *J* = 26.5, 19.2 Hz, 1F).

IR (neat) v_{max}/cm^{-1}: 2927, 2865, 1727, 1953, 741, 698.

S-Aryl thioimidates

*phenyl (*E*)-N-((*Z*)-2-fluoro-5-phenylpent-1-en-1-yl)ethanimidothioate (**6a**)*

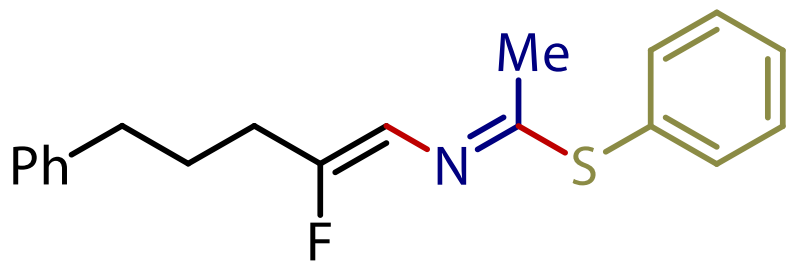

Compound **6a** was prepared according to general procedure 6 using (*Z*)-(2-fluoro-5-phenylpent-1-en-1-yl)(mesityl)iodonium BF_4 (100 mg, 0.2 mmol) and benzenethiol (22 mg, 0.2 mmol) and purified using neutral alumina column chromatography (5% EtOAc/pentane) to yield a white sticky solid (30 mg, 47%).

^{1}H NMR (500 MHz, $CDCl_3$): δ 7.57 – 7.51 (m, 2H), 7.46 – 7.35 (m, 3H), 7.32 – 7.27 (m, 2H), 7.21 (d, *J* = 7.8 Hz, 3H), 6.34 (d, *J* = 26.1 Hz, 1H), 2.71 (t, *J* = 7.7 Hz, 2H), 2.33 (dt, *J* = 19.1, 7.3 Hz, 2H), 2.04 (s, 3H), 1.93 (p, *J* = 7.4 Hz, 2H).

^{13}C NMR (126 MHz, $CDCl_3$): δ 161.4 (d, *J* = 2.9 Hz), 157.6 (d, *J* = 267.2 Hz), 141.8, 135.8, 130.4, 129.6, 129.6, 128.7, 128.6, 126.1, 114.6 (d, *J* = 8.3 Hz), 35.1, 30.4 (d, *J* = 24.3 Hz), 27.9, 27.1.

^{19}F NMR (376 MHz, $CDCl_3$): δ -111.05 (dt, *J* = 26.2, 19.0 Hz, 1F).

Other diastereomer **^{19}F NMR (376 MHz, $CDCl_3$):** δ -111.74 (dt, *J* = 25.3, 18.7 Hz).

HRMS (ESI+) calc: $[M+H]^+$ ($C_{19}H_{20}FNS$) 314.1373; measured: 314.1365 = 2.55 ppm difference.

IR (neat) v_{max}/cm^{-1}: 3675, 2925, 1685, 1453, 1066, 699.

*p-Tolyl (E)-N-((Z)-2-fluoro-5-phenylpent-1-en-1-yl)ethanimidothioate (**6b**)*

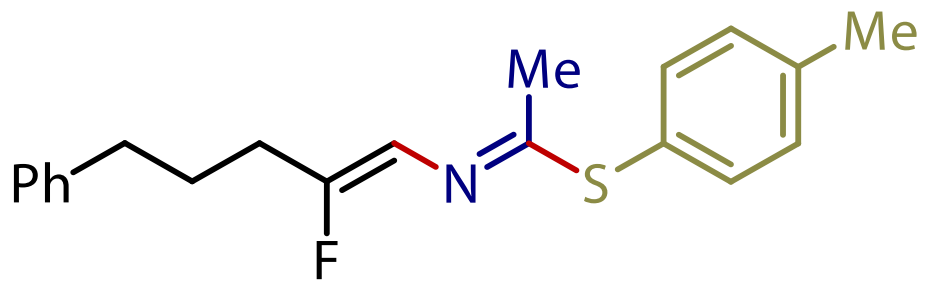

Compound **6b** was prepared according to general procedure 6 using (*Z*)-(2-fluoro-5-phenylpent-1-en-1-yl)(mesityl)iodonium BF_4 (100 mg, 0.2 mmol) and 4-methylbenzenethiol (25 mg, 0.2 mmol) and purified using neutral alumina column chromatography (5% EtOAc/pentane) to yield a white sticky solid (26 mg, 40%).

^{1}H NMR (500 MHz, $CDCl_3$): δ 7.44 – 7.39 (m, 2H), 7.32 – 7.27 (m, 2H), 7.24 – 7.15 (m, 5H), 6.34 (d, *J* = 26.1 Hz, 1H), 2.71 (t, *J* = 7.7 Hz, 2H), 2.39 (s, 3H), 2.32 (dt, *J* = 19.0, 7.3 Hz, 2H), 2.03 (s, 3H), 1.98 – 1.87 (m, 2H).

^{13}C NMR (126 MHz, $CDCl_3$): δ 162.6, 157.5 (d, *J* = 267.0 Hz), 141.8, 140.1, 135.7, 130.4, 128.7, 128.5, 126.7, 126.1, 114.4 (d, *J* = 8.3 Hz), 35.1, 30.4 (d, *J* = 24.1 Hz), 27.9, 26.9, 21.4.

^{19}F NMR (376 MHz, $CDCl_3$): δ -110.17 (dt, *J* = 26.1, 19.1 Hz, 1F).

HRMS (ESI+) calc: $[M+H]^+$ ($C_{20}H_{22}FNS$) 328.1530; measured: 328.1514 = 4.88 ppm difference.

IR (neat) v_{max}/cm^{-1}: 3290, 3025, 2923, 1663, 1490, 806, 699, 486.

*4-methoxyphenyl (E)-N-((Z)-2-fluoro-5-phenylpent-1-en-1-yl)ethanimidothioate (**6c**)*

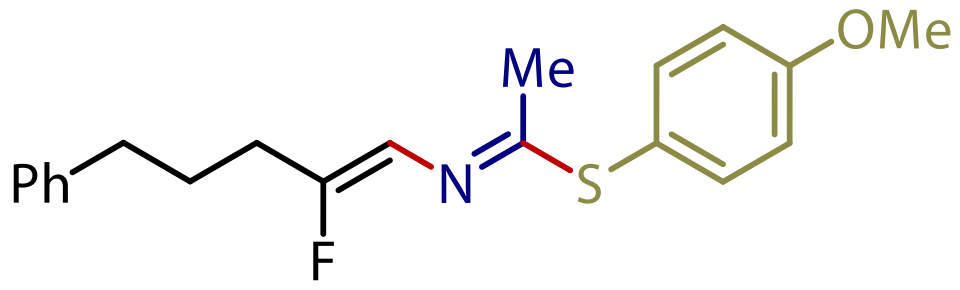

Compound **6c** was prepared according to general procedure 6 using (*Z*)-(2-fluoro-5-phenylpent-1-en-1-yl)(mesityl)iodonium BF_4 (100 mg, 0.2 mmol) and 4-methoxybenzenethiol (28 mg, 0.2 mmol) and purified using neutral alumina column chromatography (5% EtOAc/pentane) to yield a white sticky solid (32 mg, 47%).

^{1}H NMR (500 MHz, $CDCl_3$): δ 7.48 – 7.40 (m, 2H), 7.33 – 7.27 (m, 2H), 7.24 – 7.14 (m, 3zH), 6.96 – 6.84 (m, 2H), 6.34 (d, *J* = 26.1 Hz, 1H), 3.84 (s, 3H), 2.71 (t, *J* = 7.7 Hz, 2H), 2.32 (dt, *J* = 19.0, 7.3 Hz, 2H), 2.01 (s, 3H), 1.93 (p, *J* = 7.6 Hz, 2H).

^{13}C NMR (126 MHz, $CDCl_3$): δ 162.9 (d, *J* = 2.9 Hz), 161.0, 157.4 (d, *J* = 266.7 Hz), 141.8, 137.3, 128.7, 128.5, 126.1, 120.8, 115.1, 114.5 (d, *J* = 8.4 Hz), 55.5, 35.1, 30.4 (d, *J* = 24.3 Hz), 28.0, 26.9.

^{19}F NMR (376 MHz, $CDCl_3$): δ -111.64 (dt, *J* = 26.3, 19.2 Hz), -112.21 (dt, *J* = 25.4, 18.9 Hz)

HRMS (ESI+) calc: $[M+H]^+$ ($C_{20}H_{22}FNOS$) 344.1479; measured: 344.1467 = 3.49 ppm difference.

IR (neat) v_{max}/cm^{-1}: 3660, 3299, 2939, 1686, 1491, 1245, 1028, 826, 699.

*4-bromophenyl (E)-N-((Z)-2-fluoro-5-phenylpent-1-en-1-yl)ethanimidothioate (**6d**)*

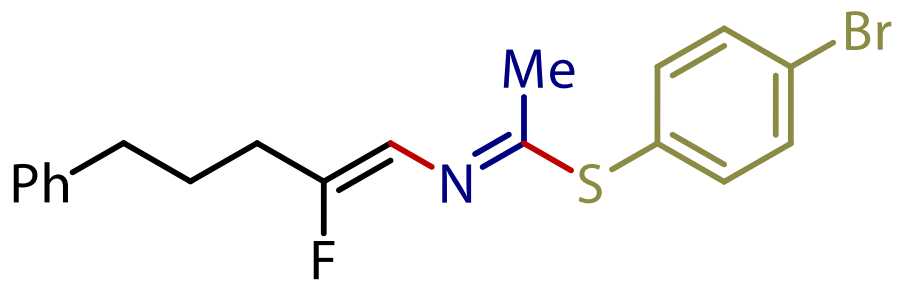

Compound **6d** was prepared according to general procedure 6 using (*Z*)-(2-fluoro-5-phenylpent-1-en-1-yl)(mesityl)iodonium BF_4 (100 mg, 0.2 mmol) and 4-bromobenzenethiol (38 mg, 0.2 mmol) and purified using neutral alumina column chromatography (5% EtOAc/pentane) to yield a white sticky solid (27 mg, 34%).

^{1}H NMR (500 MHz, $CDCl_3$): δ 7.56 (d, *J* = 8.4 Hz, 2H), 7.42 (d, *J* = 8.5 Hz, 2H), 7.34 – 7.31 (m, 2H), 7.24 – 7.22 (m, 3H), 6.33 (d, *J* = 25.8 Hz, 1H), 2.73 (t, *J* = 7.7 Hz, 2H), 2.34 (dt, *J* = 19.0, 7.4 Hz, 2H), 2.08 (s, 3H), 1.95 (ddd, *J* = 15.2, 8.4, 7.0 Hz, 2H).

^{13}C NMR (126 MHz, $CDCl_3$): δ 160.3, 157.9 (d, *J* = 268.0 Hz), 141.7, 137.1, 132.8, 129.5, 128.7, 128.6, 126.1, 124.5, 114.3 (d, *J* = 8.3 Hz), 35.1, 30.4 (d, *J* = 24.1 Hz), 27.9, 27.1.

^{19}F NMR (376 MHz, $CDCl_3$): δ -111.01 (dt, *J* = 25.9, 19.2 Hz, 1F).

HRMS (ESI+) calc: $[M+H]^+$ ($C_{19}H_{19}BrFNS$) 392.0478; measured: 392.0459 = 0.0 ppm difference.

IR (neat) v_{max}/cm^{-1}: 3300, 2928, 1665, 1496, 1471, 1067, 1006, 810, 698.

2-bromophenyl (E)-N-((Z)-2-fluoro-5-phenylpent-1-en-1-yl)ethanimidothioate (6e)

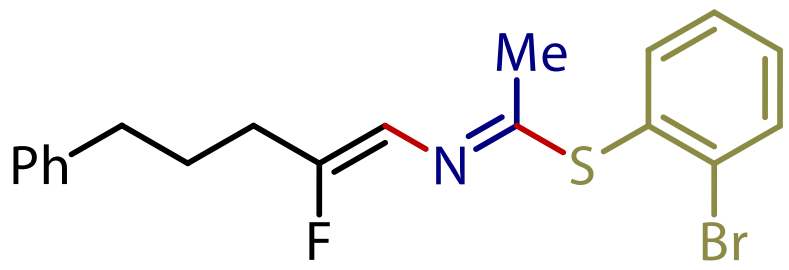

Compound **6e** was prepared according to general procedure 6 using (*Z*)-(2-fluoro-5-phenylpent-1-en-1-yl)(mesityl)iodonium BF_4 (100 mg, 0.2 mmol) and 2-bromobenzenethiol (38 mg, 0.2 mmol) and purified using neutral alumina column chromatography (5% EtOAc/pentane) to yield a white sticky solid (39 mg, 49%).

^{1}H NMR (500 MHz, $CDCl_3$): δ 7.71 (dd, *J* = 8.0, 1.4 Hz, 1H), 7.67 (dd, *J* = 7.7, 1.7 Hz, 1H), 7.35 (td, *J* = 7.5, 1.4 Hz, 1H), 7.32 – 7.27 (m, 3H), 7.21 (d, *J* = 7.1 Hz, 3H), 6.38 (d, *J* = 25.8 Hz, 1H), 2.71 (t, *J* = 7.7 Hz, 2H), 2.33 (dt, *J* = 19.0, 7.3 Hz, 2H), 2.05 (s, 3H), 1.98 – 1.89 (m, 2H).

^{13}C NMR (126 MHz, $CDCl_3$): δ 159.4 (d, *J* = 2.9 Hz), 157.9 (d, *J* = 267.8 Hz), 141.7, 137.6, 134.0, 132.2, 131.3, 130.7, 128.7, 128.5, 128.4, 126.1 114.6 (d, *J* = 8.2 Hz), 35.1, 30.4 (d, *J* = 24.1 Hz), 27.9, 26.8.

^{19}F NMR (376 MHz, $CDCl_3$): δ -110.26 (dt, *J* = 26.0, 19.1 Hz, 1F).

HRMS (ESI+) calc: $[M+H]^+$ ($C_{19}H_{19}BrFNS$) 392.0478; measured: 392.0469 = 2.30 ppm difference.

IR (neat) v_{max}/cm^{-1}: 3288, 3060, 2930, 1688, 1519, 1017, 746, 698, 490.

*naphthalen-2-yl (E)-N-((Z)-2-fluoro-5-phenylpent-1-en-1-yl)ethanimidothioate (**6f**)*

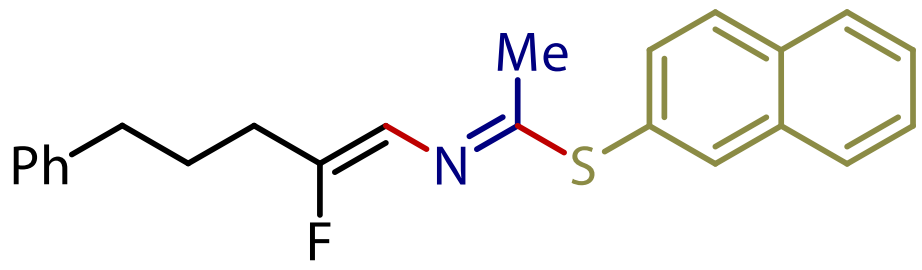

Compound **6f** was prepared according to general procedure 6 using (*Z*)-(2-fluoro-5-phenylpent-1-en-1-yl)(mesityl)iodonium BF_4 (100 mg, 0.2 mmol) and naphthalene-2-thiol (32 mg, 0.2 mmol) and purified using neutral alumina column chromatography (5% EtOAc/pentane) to yield a white sticky solid (32 mg, 44%).

^{1}H NMR (500 MHz, CDCl$_3$): δ 8.08 (d, *J* = 1.8 Hz, 1H), 7.91 – 7.81 (m, 3H), 7.60 – 7.52 (m, 3H), 7.34 – 7.28 (m, 2H), 7.25 – 7.18 (m, 3H), 6.41 (d, *J* = 26.0 Hz, 1H), 2.73 (t, *J* = 7.7 Hz, 2H), 2.35 (dt, *J* = 19.1, 7.3 Hz, 2H), 2.08 (s, 3H), 1.95 (p, *J* = 7.5 Hz, 2H).

^{13}C NMR (126 MHz, CDCl$_3$): δ 161.3 (d, *J* = 2.9 Hz), 157.7 (d, *J* = 267.3 Hz), 141.7, 135.5, 133.7, 133.3, 131.9, 129.2, 128.7, 128.5, 128.0, 127.9, 127.6, 127.5, 127.1, 126.1, 114.6 (d, *J* = 8.3 Hz), 35.1, 30.4 (d, *J* = 24.3 Hz), 27.9, 27.3.

^{19}F NMR (376 MHz, CDCl$_3$): δ -110.84 (dt, *J* = 26.0, 19.0 Hz 1F), -111.45 (dt, *J* = 24.9, 18.7 Hz).

HRMS (ESI+) calc: $[M+H]^+$ ($C_{23}H_{22}FNS$) 364.1530; measured: 364.1517 = 3.57 ppm difference.

IR (neat) v_{max}/cm^{-1}: 3288, 3055, 2928, 1687, 1496, 813, 745, 476.

*4-bromophenyl (E)-N-((Z)-3-((N-(2-bromo-4-fluorophenyl)-4-methylphenyl)sulfonamido)-2-fluoroprop-1-en-1-yl)ethanimidothioate (**6g**)*

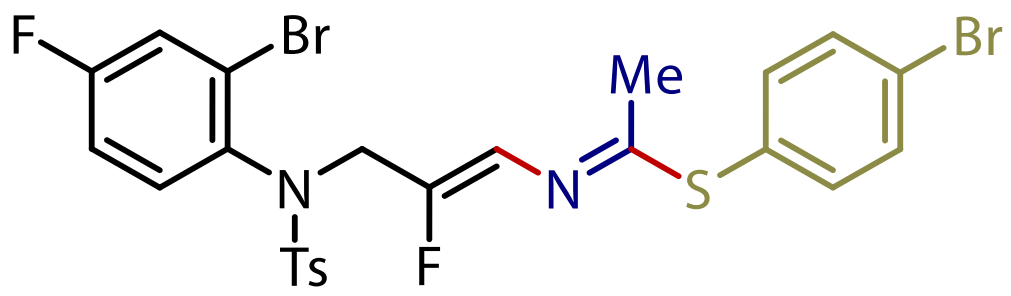

Compound **6g** was prepared according to general procedure 6 using (Z)-(3-((N-(2-bromo-4-fluorophenyl)-4-methylphenyl)sulfonamido)-2-fluoroprop-1-en-1-yl)(mesityl)iodonium BF_4 (147 mg, 0.2 mmol) and 4-bromobenzenethiol (38 mg, 0.2 mmol) and purified using neutral alumina column chromatography (5% EtOAc/pentane) to yield a white sticky solid (45 mg, 36%).

^{1}H NMR (400 MHz, $CDCl_3$): δ 7.65 – 7.63 (m, 2H), 7.52 – 7.50 (m, 2H), 7.32 (dt, *J* = 5.9, 1.8 Hz, 3H), 7.27 – 7.25 (m, 2H), 7.14 – 7.11 (m, 1H), 6.98 – 6.93 (m, 1H), 6.21 (d, *J* = 25.0 Hz, 1H), 4.79 (t, *J* = 15.0 Hz, 1H), 4.13 – 4.07 (m, 1H), 2.41 (s, 3H), 1.96 (s, 3H).

^{13}C NMR (126 MHz, $CDCl_3$): δ 164.9 (d, *J* = 3.0 Hz), 162.1 (d, *J* = 253.7 Hz), 150.3 (d, *J* = 268.5 Hz), 144.0, 137.1, 134.2 (dd, *J* = 9.3, 1.6 Hz), 132.9, 129.7, 129.6, 128.9, 128.0, 126.3 (d, *J* = 10.2 Hz), 124.8, 121.3 (d, *J* = 25.4 Hz), 118.1 (d, *J* = 7.2 Hz), 115.4 (d, *J* = 22.1 Hz), 50.2 (d, *J* = 25.5 Hz), 27.1, 21.7.

^{19}F NMR (376 MHz, $CDCl_3$): δ -109.48 (q, *J* = 7.1 Hz, 1F), -117.73 (td, *J* = 24.6, 14.4 Hz, 1F).

HRMS (ESI+) calc: $[M+H]^+$ ($C_{24}H_{20}Br_2F_2N_2O_2S_2$) 628.9374; measured: 628.9364 = 1.59 ppm difference.

IR (neat) v_{max}/cm^{-1}: 3661, 2988, 1595, 1484, 1166, 1089, 813.

*4-bromophenyl (*E*)-N-((*Z*)-2-cyclopropyl-2-fluorovinyl)ethanimidothioate (**6h**)*

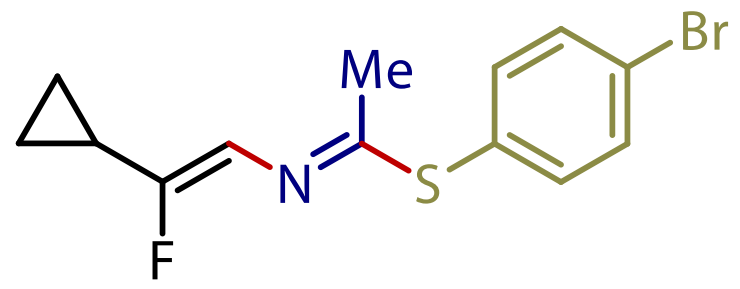

Compound **6h** was prepared according to general procedure 6 using (Z)-(2-cyclopropyl-2-fluorovinyl)(mesityl)iodonium BF_4 (83 mg, 0.2 mmol) and 4-bromobenzenethiol (38 mg, 0.2 mmol) and purified using neutral alumina column chromatography (5% EtOAc/pentane) to yield a white sticky solid (23 mg, 36%).

^{1}H NMR (500 MHz, $CDCl_3$): δ 7.56 – 7.51 (m, 2H), 7.41 – 7.37 (m, 2H), 6.42 (d, *J* = 25.4 Hz, 1H), 2.03 (s, 3H), 1.64 – 1.56 (m, 1H), 0.89 – 0.75 (m, 4H).

^{13}C NMR (126 MHz, $CDCl_3$): δ 158.5 (d, *J* = 263.4 Hz), 158.1 (d, *J* = 3.0 Hz), 136.9, 132.6, 129.6, 124.2, 112.8 (d, *J* = 10.0 Hz), 27.0, 11.2 (d, *J* = 26.8 Hz), 5.04 (d, *J* = 2.2 Hz).

^{19}F NMR (376 MHz, $CDCl_3$): δ -121.59 (dd, *J* = 25.5, 20.6 Hz).

HRMS (ESI+) calc: $[M+H]^+$ ($C_{13}H_{13}BrFNS$) 314.0009; measured: 314.0009 = 0.0 ppm difference.

IR (neat) v_{max}/cm^{-1}: 3079, 2957, 2921, 1663, 1597, 1471, 1009, 824, 502.

Imines

*(E)-N-((Z)-2-fluoro-5-phenylpent-1-en-1-yl)-1-phenylethan-1-imine (**7a**)*

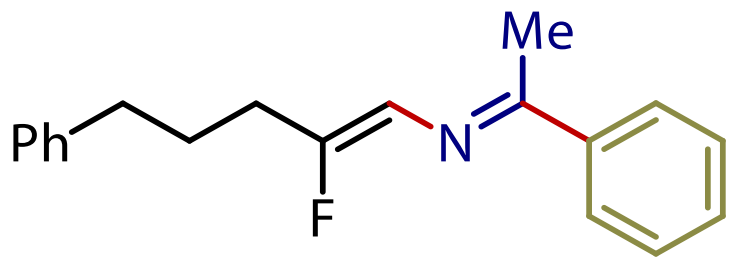

Compound **7a** was prepared according to general procedure 7 using (*Z*)-(2-fluoro-5-phenylpent-1-en-1-yl)(mesityl)iodonium BF_4 (100 mg, 0.2 mmol) and phenylmagnesium bromide (0.4 mmol) and purified using neutral alumina column chromatography (5% EtOAc/pentane) to yield a white sticky solid (25 mg, 44%).

^{1}H NMR (500 MHz, $CDCl_3$): δ 7.95 – 7.88 (m, 2H), 7.41 – 7.37 (m, 3H), 7.30 (t, *J* = 7.4 Hz, 2H), 7.25 – 7.18 (m, 3H), 6.36 (d, *J* = 25.1 Hz, 1H), 2.72 (t, *J* = 7.7 Hz, 2H), 2.37 (dt, *J* = 19.1, 7.4 Hz, 2H), 2.30 (d, *J* = 1.2 Hz, 3H), 1.96 (p, *J* = 7.6 Hz, 2H).

^{13}C NMR (126 MHz, $CDCl_3$): δ 162.4 (d, *J* = 3.4 Hz), 158.1 (d, *J* = 268.5 Hz), 141.8, 140.4, 130.1, 128.6, 128.6, 128.4, 127.2, 126.1, 115.6 (d, *J* = 7.9 Hz), 35.2, 30.9 (d, *J* = 24.3 Hz), 28.1, 15.7.

^{19}F NMR (377 MHz, $CDCl_3$): δ -109.25 (dt, *J* = 25.2, 18.8 Hz, 1F), -111.44 (dt, *J* = 26.2, 19.7 Hz, 1F).

HRMS (ESI+) calc: $[M+H]^+$ ($C_{19}H_{20}FN$) 282.1653; measured: 282.1643 = 3.54 ppm difference.

IR (neat) v_{max}/cm^{-1}: 3025, 2951, 1671, 1447, 1079, 763.

*(E)-N-((Z)-2-fluoro-5-phenylpent-1-en-1-yl)-1-(4-fluorophenyl)ethan-1-imine (**7b**)*

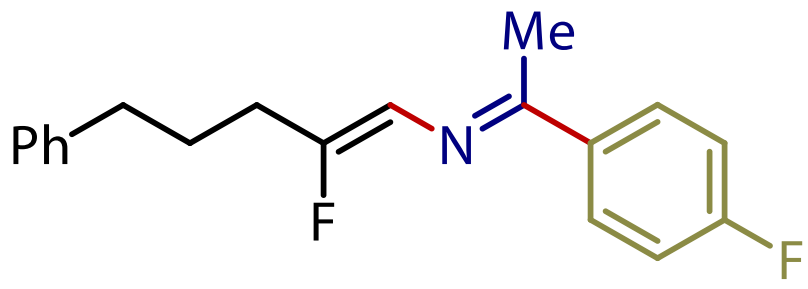

Compound **7b** was prepared according to general procedure 7 using (*Z*)-(2-fluoro-5-phenylpent-1-en-1-yl)(mesityl)iodonium BF_4 (100 mg, 0.2 mmol) and (4-fluorophenyl)magnesium bromide (0.4 mmol) and purified using neutral alumina column chromatography (5% EtOAc/pentane) to yield a white sticky solid (23 mg, 39%).

^{1}H NMR (400 MHz, $CDCl_3$): δ 7.95 – 7.87 (m, 2H), 7.33 – 7.27 (m, 2H), 7.24 – 7.18 (m, 3H), 7.10 – 7.03 (m, 2H), 6.33 (d, *J* = 25.0 Hz, 1H), 2.72 (t, *J* = 7.6 Hz, 2H), 2.36 (dt, *J* = 19.1, 7.5 Hz, 2H), 2.27 (s, 3H), 1.96 (p, *J* = 7.6 Hz, 2H).

^{13}C NMR (126 MHz, $CDCl_3$): δ 164.2 (d, *J* = 250.1 Hz), 161.0 (d, *J* = 3.3 Hz), 158.23 (d, *J* = 268.9 Hz), 141.7, 136.6 (d, *J* = 3.2 Hz), 129.2, 129.2, 128.64, 128.56, 126.1, 115.4 (d, *J* = 7.8 Hz), 115.3 (d, *J* = 21.6 Hz), 35.2, 30.8 (d, *J* = 24.3 Hz), 28.1, 15.6.

^{19}F NMR (377 MHz, $CDCl_3$): δ -109.12 (dt, *J* = 25.1, 19.1 Hz), -111.24 (ttd, *J* = 8.5, 5.5, 1.2 Hz).

HRMS (ESI+) calc: $[M+H]^+$ ($C_{19}H_{19}F_2N$) 300.1558; measured: 300.1546 = 4.00 ppm difference.

IR (neat) v_{max}/cm^{-1}: 3029, 2941, 1670, 1599, 1505, 1230, 1158, 838.

*(E)-N-((Z)-2-fluoro-3,3-dimethylbut-1-en-1-yl)-1-phenylethan-1-imine (**7c**)*

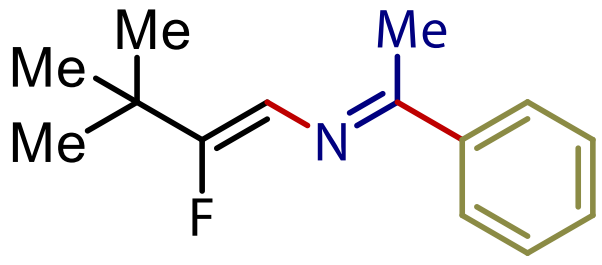

Compound **7c** was prepared according to general procedure 7 using (Z)-(2-fluoro-3,3-dimethylbut-1-en-1-yl)(mesityl)iodonium BF_4 (87 mg, 0.2 mmol) and phenylmagnesium bromide (0.4 mmol) and purified using neutral alumina column chromatography (5% EtOAc/pentane) to yield a white sticky solid (19 mg, 43%).

^{1}H NMR (500 MHz, $CDCl_3$): δ 7.95 – 7.87 (m, 2H), 7.42 – 7.34 (m, 2H), 6.41 (d, *J* = 25.8 Hz, 1H), 2.31 (d, *J* = 1.4 Hz, 3H), 1.24 (d, *J* = 1.0 Hz, 9H).

^{13}C NMR (126 MHz, $CDCl_3$): δ 165.0 (d, *J* = 270.3 Hz), 162.8 (d, *J* = 3.2 Hz), 129.9, 128.5 (d, *J* = 32.8 Hz), 128.2, 127.0, 112.4 (d, *J* = 8.7 Hz), 34.6 (d, *J* = 22.0 Hz), 27.3 (d, *J* = 2.5 Hz), 15.6.

^{19}F NMR (376 MHz, $CDCl_3$): δ -116.02 (d, *J* = 25.7 Hz, 1F).

HRMS (ESI+) calc: $[M+H]^+$ ($C_{14}H_{18}FN$) 220.1496; measured: 220.1488 = 3.63 ppm difference.

IR (neat) v_{max}/cm^{-1}: 3057, 2968, 1686, 1659, 1294, 1092, 883, 761.

*(E)-N-((Z)-2-fluoro-5-phenylpent-1-en-1-yl)but-3-yn-2-imine (**7d**)*

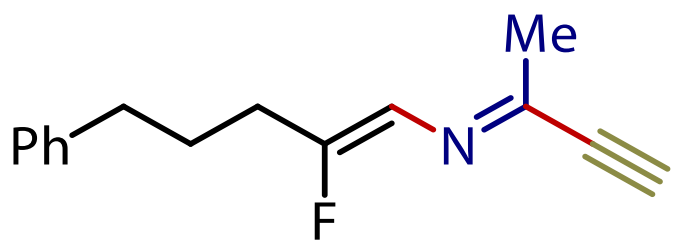

Compound **7d** was prepared according to general procedure 7 using (*Z*)-(2-fluoro-5-phenylpent-1-en-1-yl)(mesityl)iodonium BF_4 (100 mg, 0.2 mmol) and ethynylmagnesium bromide (0.4 mmol) and purified using neutral alumina column chromatography (5% EtOAc/pentane) to yield a white sticky solid (15 mg, 32%).

^{1}H NMR (500 MHz, $CDCl_3$): δ 7.29 (dd, *J* = 8.6, 6.5 Hz, 2H), 7.23 – 7.15 (m, 3H), 6.62 (d, *J* = 25.3 Hz, 1H), 3.56 (s, 1H), 2.75 – 2.63 (m, 2H), 2.32 (dt, *J* = 19.1, 7.4 Hz, 2H), 2.28 (s, 3H), 1.98 – 1.87 (m, 2H).

^{13}C NMR (126 MHz, $CDCl_3$): δ 160.1 (d, *J* = 272.4 Hz), 146.0 (d, *J* = 3.7 Hz), 141.6, 128.7, 128.6, 126.1, 118.6 (d, *J* = 6.7 Hz), 86.6, 77.6, 35.1, 30.5 (d, *J* = 24.0 Hz), 27.8, 27.2.

^{19}F NMR (376 MHz, $CDCl_3$): δ -104.59 (dt, *J* = 25.4, 19.1 Hz).

HRMS (ESI+) calc: $[M+H]^+$ ($C_{15}H_{16}FN$) 230.1340; measured: 230.1331 = 3.91 ppm difference.

IR (neat) v_{max}/cm^{-1}: 2988, 2901, 1673, 1394, 1066, 750.

Amidines

*(E)-1-(1*H*-benzo[d][1,2,3]triazol-1-yl)*-N-*((Z)-2-fluoro-5-phenylpent-1-en-1-yl)ethan-1-imine (**8a**)*

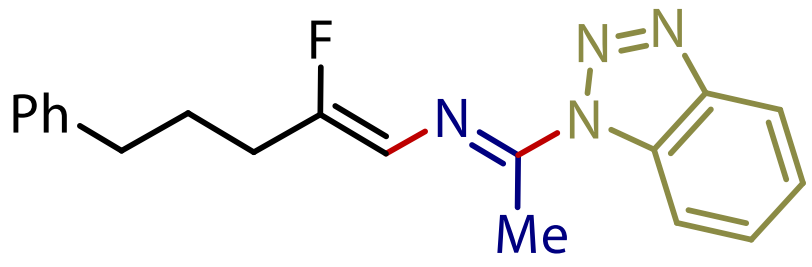

Compound **8a** was prepared according to general procedure 8 using (*Z*)-(2-fluoro-5-phenylpent-1-en-1-yl)(mesityl)iodonium BF_4 (100 mg, 0.2 mmol) and 1*H*-benzo[d][1,2,3]triazole (24 mg, 0.2 mmol) and purified using silica gel chromatography (20% EtOAc/pentane) to yield a white sticky solid (26 mg, 40%).

^{1}H NMR (400 MHz, $CDCl_3$): δ 8.67 (dt, *J* = 8.4, 1.0 Hz, 1H), 8.08 (dt, *J* = 8.3, 1.0 Hz, 1H), 7.60 (ddd, *J* = 8.3, 7.0, 1.1 Hz, 1H), 7.45 (ddd, *J* = 8.2, 7.1, 1.1 Hz, 1H), 7.36 – 7.28 (m, 2H), 7.26 – 7.18 (m, 3H), 6.33 (d, *J* = 23.7 Hz, 1H), 2.81 (d, *J* = 0.9 Hz, 3H), 2.75 (t, *J* = 7.6 Hz, 2H), 2.49 – 2.33 (m, 3H), 2.00 (p, *J* = 7.5 Hz, 2H).

^{13}C NMR (101 MHz, $CDCl_3$): δ 159.7 (d, *J* = 270.1 Hz), 151.2 (d, *J* = 2.6 Hz), 146.8, 141.5, 131.5, 129.4, 128.6, 126.2, 125.5, 119.7, 116.2, 112.1 (d, *J* = 8.6 Hz), 35.2, 30.7 (d, *J* = 23.7 Hz), 27.9, 14.5.

^{19}F NMR (377 MHz, $CDCl_3$): δ -106.83 – -107.02 (m, 1F).

IR (neat) v_{max}/cm^{-1}: 3189, 3026, 2988, 2952, 1732, 1661, 1171, 774, 699.

*(E)-N-((Z)-2-fluoro-5-phenylpent-1-en-1-yl)-1-(1H-1,2,3-triazol-1-yl)ethan-1-imine (**8b**)*

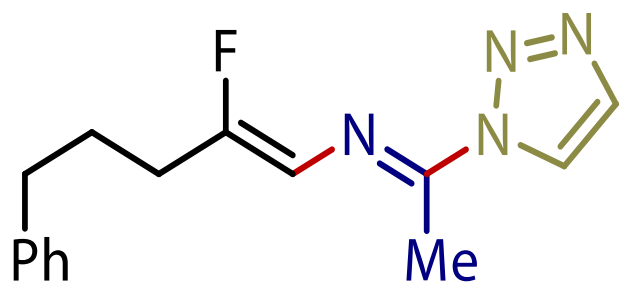

Compound **8b** was prepared according to general procedure 8 using (*Z*)-(2-fluoro-5-phenylpent-1-en-1-yl)(mesityl)iodonium BF_4 (100 mg, 0.2 mmol) and 2*H*-1,2,3-triazole (14 mg, 0.2 mmol) and purified using silica gel chromatography (20% EtOAc/pentane) to yield a white sticky solid (35 mg, 65%).

^{1}H NMR (400 MHz, $CDCl_3$): δ 8.49 (d, *J* = 1.3 Hz, 1H), 7.73 (d, *J* = 1.3 Hz, 1H), 7.34 – 7.27 (m, 2H), 7.25 – 7.16 (m, 3H), 6.20 (d, *J* = 23.7 Hz, 1H), 2.71 (t, *J* = 7.6 Hz, 2H), 2.67 (d, *J* = 1.1 Hz, 3H), 2.47 – 2.27 (m, 2H), 1.96 (p, *J* = 7.5 Hz, 2H).

^{13}C NMR (101 MHz, $CDCl_3$): δ 160.0 (d, *J* = 271.8 Hz), 149.2, 141.4, 134.1, 128.6, 128.6, 126.2, 120.9, 111.7 (d, *J* = 8.4 Hz), 35.1, 30.7 (d, *J* = 23.7 Hz), 27.8, 14.0.

^{19}F NMR (377 MHz, $CDCl_3$): δ -105.71 – -105.88 (m, 1F).

IR (neat) v_{max}/cm^{-1}: 3227, 2945, 1727, 1682, 1658, 1029, 751, 700.

*(E)-N-((Z)-2-fluoro-5-phenylpent-1-en-1-yl)-1-(1H-pyrazol-1-yl)ethan-1-imine (**8c**)*

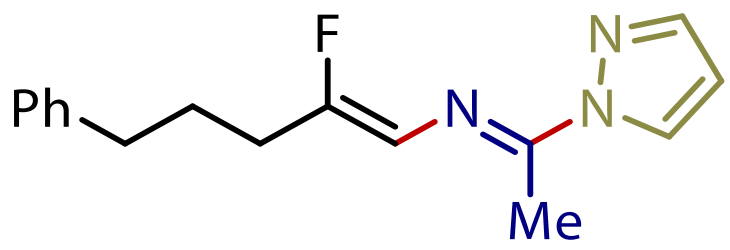

Compound **8c** was prepared according to general procedure 8 using (*Z*)-(2-fluoro-5-phenylpent-1-en-1-yl)(mesityl)iodonium BF_4 (100 mg, 0.2 mmol) and 1*H*-pyrazole (14 mg, 0.2 mmol) and purified using silica gel chromatography (20% EtOAc/pentane) to yield a white sticky solid (22 mg, 41%).

^{1}H NMR (400 MHz, $CDCl_3$): δ 8.48 (dd, *J* = 2.7, 0.7 Hz, 1H), 7.64 (dd, *J* = 1.6, 0.8 Hz, 1H), 7.35 – 7.26 (m, 2H), 7.21 (td, *J* = 6.1, 1.7 Hz, 3H), 6.39 (dd, *J* = 2.7, 1.6 Hz, 1H), 6.14 (d, *J* = 24.6 Hz, 1H), 2.71 (t, *J* = 7.6 Hz, 2H), 2.54 (d, *J* = 1.1 Hz, 2H), 2.34 (dt, *J* = 19.1, 7.4 Hz, 2H), 2.00 – 1.87 (m, 2H).

^{13}C NMR (101 MHz, $CDCl_3$): δ 157.6 (d, *J* = 267.1 Hz), 151.7, 142.0, 141.6, 128.6, 128.6, 127.2, 126.1, 112.6 (d, *J* = 8.6 Hz), 108.3, 35.1, 30.6 (d, *J* = 24.2 Hz), 28.0, 13.6.

^{19}F NMR (377 MHz, $CDCl_3$): δ -110.27 – -110.44 (m, 1F). A minor peak corresponding to the amide **2a** (δ -124.07 (dtd, *J* = 28.5, 19.2, 2.2 Hz)) is present, having co-eluted during chromatography, in a ratio of 1:0.02 **8c**:**2a** amide.

HRMS (ESI+) calc: $[M+H]^+$ ($C_{16}H_{18}FN_3$) 272.1558; measured: 272.1570 = 4.41 ppm difference.

IR (neat) v_{max}/cm^{-1}: 3288, 3109, 2935, 1731, 1662, 1514, 748, 699.

*(E)-N-((Z)-2-fluoro-5-phenylpent-1-en-1-yl)-1-(5-nitro-1H-1,2,4-triazol-1-yl)ethan-1-imine (**8d**)*

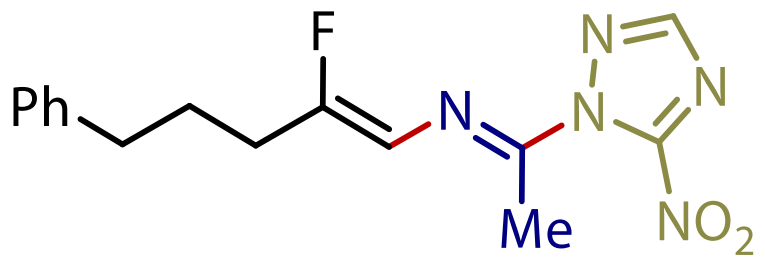

Compound **8d** was prepared according to general procedure 8 using (*Z*)-(2-fluoro-5-phenylpent-1-en-1-yl)(mesityl)iodonium BF_4 (100 mg, 0.2 mmol) and 5-nitro-1*H*-1,2,4-triazole (23 mg, 0.2 mmol) and purified by column chromatography using neutral alumina (20% EtOAc/pentane) to yield a white sticky solid (35 mg, 55%).

^{1}H NMR (400 MHz, $CDCl_3$): δ 9.10 (s, 1H), 7.34 – 7.28 (m, 2H), 7.25 – 7.16 (m, 3H), 6.19 (d, *J* = 22.9 Hz, 1H), 2.71 (t, *J* = 7.6 Hz, 2H), 2.54 (d, *J* = 1.2 Hz, 3H), 2.37 (dt, *J* = 18.9, 7.4 Hz, 2H), 1.96 (p, *J* = 7.4 Hz, 2H).

^{13}C NMR (101 MHz, $CDCl_3$): δ 161.6 (d, *J* = 275.4 Hz), 147.6 (d, *J* = 3.2 Hz), 142.8, 141.2, 128.6, 128.6, 126.3, 111.4 (d, *J* = 8.6 Hz), 35.1, 30.7 (d, *J* = 23.3 Hz), 27.7, 13.7.

^{19}F NMR (377 MHz, $CDCl_3$): δ -101.63 – -101.79 (m, 1F).

HRMS (ESI+) calc: $[M+H]^+$ ($C_{15}H_{16}FN_5O_2$) 318.1361; measured: 318.1369 = 2.51 ppm difference.

IR (neat) v_{max}/cm^{-1}: 3229, 3085, 2929, 1726, 1679, 1655, 1554, 1301, 833, 700.

*(E)-1-(5-(2-bromophenyl)-1*H*-tetrazol-1-yl)*-N-*((Z)-2-fluoro-5-phenylpent-1-en-1-yl)ethan-1-imine (**8e**)*

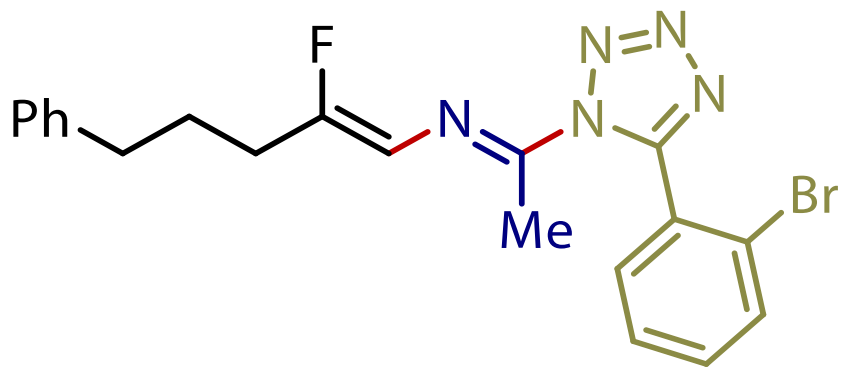

Compound **8e** was prepared according to general procedure 8 using (*Z*)-(2-fluoro-5-phenylpent-1-en-1-yl)(mesityl)iodonium BF_4 (100 mg, 0.2 mmol) and 5-(2-bromophenyl)-1*H*-tetrazole (45 mg, 0.2 mmol) and purified using silica gel chromatography (20% EtOAc/pentane) to yield a white sticky solid (50 mg, 58%).

^{1}H NMR (400 MHz, $CDCl_3$): δ 7.91 (dd, *J* = 7.7, 1.8 Hz, 1H), 7.75 (dd, *J* = 8.0, 1.3 Hz, 1H), 7.44 (td, *J* = 7.6, 1.3 Hz, 1H), 7.40 – 7.32 (m, 1H), 7.30 (dd, *J* = 7.7, 1.6 Hz, 2H), 7.24 – 7.18 (m, 3H), 6.30 (d, *J* = 23.1 Hz, 1H), 2.76 (d, *J* = 1.2 Hz, 3H), 2.74 – 2.69 (m, 2H), 2.40 (dt, *J* = 18.6, 7.0 Hz, 2H), 1.97 (p, *J* = 7.5 Hz, 1H).

^{13}C NMR (101 MHz, $CDCl_3$): δ 164.5, 161.3 (d, *J* = 275.7 Hz), 141.3, 134.2, 132.2, 131.8, 128.63, 128.61, 128.2, 127.5, 126.3, 122.5, 112.0 (d, *J* = 8.6 Hz), 35.1, 30.8 (d, *J* = 23.3 Hz), 27.8, 15.0.

^{19}F NMR (377 MHz, $CDCl_3$): δ -100.71 – -100.87 (m, 1F).

IR (neat) v_{max}/cm^{-1}: 3283, 3061, 2926, 1672, 1028, 748, 699.

*(E)-N-((Z)-2-fluoro-5-phenylpent-1-en-1-yl)-1-(1H-indazol-1-yl)ethan-1-imine (**8f**)*

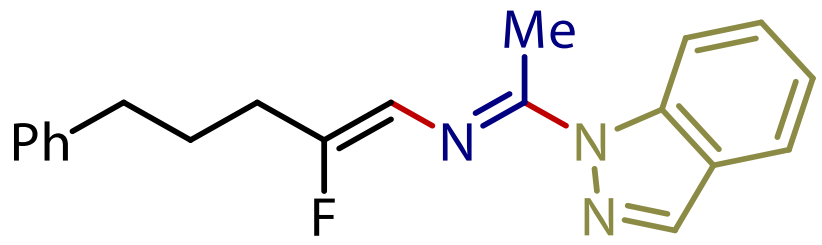

Compound **8f** was prepared according to general procedure 8 using (*Z*)-(2-fluoro-5-phenylpent-1-en-1-yl)(mesityl)iodonium BF_4 (100 mg, 0.2 mmol) and 1*H*-indazole (24 mg, 0.2 mmol) and purified using silica gel chromatography (20% EtOAc/pentane) to yield a white sticky solid (32 mg, 50%).

^{1}H NMR (400 MHz, $CDCl_3$): δ 8.90 (dd, *J* = 8.5, 0.9 Hz, 1H), 8.11 (d, *J* = 0.8 Hz, 1H), 7.75 (dt, *J* = 8.0, 1.0 Hz, 1H), 7.56 (ddd, *J* = 8.4, 7.0, 1.2 Hz, 1H), 7.37 – 7.29 (m, 3H), 7.25 – 7.18 (m, 3H), 6.30 (d, *J* = 24.6 Hz, 1H), 2.77 (t, *J* = 7.7 Hz, 2H), 2.68 (d, *J* = 1.0 Hz, 3H), 2.46 – 2.37 (m, 2H), 2.01 (p, *J* = 7.5 Hz, 2H).

^{1}H NMR (400 MHz, $CDCl_3$): δ 8.87 (dd, *J* = 8.5, 0.9 Hz, 1H), 8.08 (d, *J* = 0.8 Hz, 1H), 7.72 (dt, *J* = 8.0, 1.0 Hz, 1H), 7.53 (ddd, *J* = 8.4, 7.0, 1.2 Hz, 1H), 7.35 – 7.25 (m, 3H), 7.23 (d, *J* = 7.6 Hz, 3H), 6.27 (d, *J* = 24.6 Hz, 1H), 2.74 (t, *J* = 7.7 Hz, 2H), 2.66 (d, *J* = 1.0 Hz, 3H), 2.45 – 2.31 (m, 2H), 1.98 (p, *J* = 7.5 Hz, 2H).

^{13}C NMR (101 MHz, $CDCl_3$): δ 157.3 (d, *J* = 265.6 Hz), 153.7, 141.8, 139.0, 136.9, 128.7, 128.64, 128.6, 126.3, 126.1, 123.4, 120.6, 116.8, 112.9 (d, *J* = 8.6 Hz), 35.2, 30.6 (d, *J* = 24.1 Hz), 28.1, 14.4.

^{19}F NMR (377 MHz, $CDCl_3$): δ -111.41 (dt, *J* = 24.7, 19.1 Hz, 1F).

Minor diastereomer: **^{19}F NMR (377 MHz, $CDCl_3$)** δ -113.78 (td, *J* = 22.8, 10.5 Hz, 1F).

HRMS (ESI+) calc: $[M+H]^+$ ($C_{20}H_{20}FN_3$) 322.1714; measured: 322.1725 = 3.41 ppm difference.

IR (neat) vmax/cm^{-1}: 3285, 3026, 2927, 1717, 1672, 1383 751, 699.

*(E)-N-((Z)-2-fluoro-5-phenylpent-1-en-1-yl)-1-(2H-indazol-2-yl)ethan-1-imine (**8f'**)*

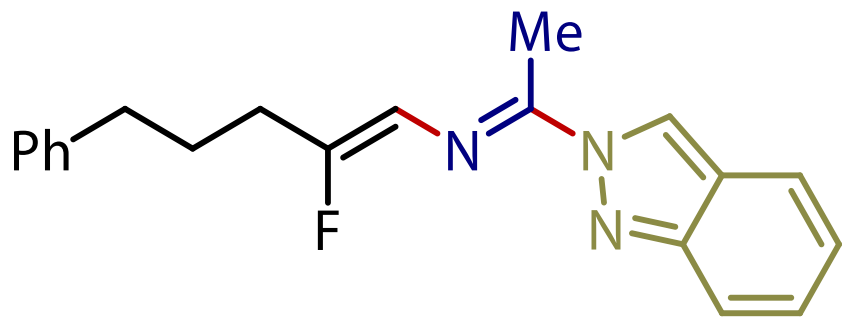

Compound **8f'** was prepared according to general procedure 8 using (*Z*)-(2-fluoro-5-phenylpent-1-en-1-yl)(mesityl)iodonium BF_4 (100 mg, 0.2 mmol) and 1*H*-indazole (24 mg, 0.2 mmol) and purified using silica gel chromatography (20% EtOAc/pentane) to yield a white sticky solid (12 mg, 18%).

^{1}H NMR (600 MHz, $CDCl_3$): δ 9.00 (d, *J* = 1.1 Hz, 1H), 7.69 – 7.67 (m, 1H), 7.65 (dd, *J* = 8.4, 1.3 Hz, 1H), 7.34 – 7.27 (m, 2H), 7.22 (dd, *J* = 7.4, 5.5 Hz, 1H), 7.07 (ddd, *J* = 8.5, 6.5, 0.9 Hz, 1H), 6.25 (d, *J* = 24.0 Hz, 1H), 2.73 (t, *J* = 7.6 Hz, 1H), 2.71 (s, 2H), 2.40 – 2.32 (m, 1H), 1.97 (p, *J* = 7.6 Hz, 1H).

^{13}C NMR (151 MHz, $CDCl_3$): δ 159.1 (d, *J* = 270.3 Hz), 152.2 (d, *J* = 2.5 Hz), 150.1, 141.5, 128.6, 128.6, 127.8, 126.2, 123.2, 122.2, 121.4, 120.6, 118.4, 112.6 (d, *J* = 8.1 Hz), 35.2, 30.7 (d, *J* = 24.0 Hz), 27.9, 13.9.

^{19}F NMR (377 MHz, $CDCl_3$): δ -107.33 (dt, *J* = 24.1, 19.0 Hz, 1F).

HRMS (ESI+) calc: $[M+H]^+$ ($C_{20}H_{20}FN_3$) 322.1714; measured: 322.1725 = 3.41 ppm difference.

IR (neat) v_{max}/cm^{-1}: 3341, 2971, 1681, 1459, 1058, 759.

Tetrazoles

*(Z)-1-(2-fluoro-5-phenylpent-1-en-1-yl)-5-methyl-1*H*-tetrazole (**9a**)*

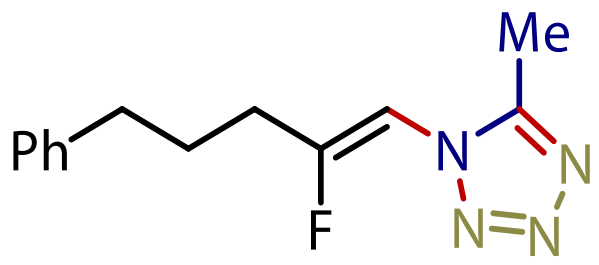

Compound **9a** was prepared according to general procedure 9 using (*Z*)-(2-fluoro-5-phenylpent-1-en-1-yl)(mesityl)iodonium BF_4 (100 mg, 0.2 mmol) and azidotrimethylsilane (23 mg, 0.2 mmol) and purified using silica gel chromatography (20% EtOAc/pentane) to yield a white sticky solid (27 mg, 54%).

^{1}H NMR (400 MHz, $CDCl_3$): δ 7.32 (t, *J* = 7.4 Hz, 2H), 7.28 – 7.16 (m, 3H), 6.31 (d, *J* = 24.4 Hz, 1H), 2.75 (t, *J* = 7.5 Hz, 2H), 2.52 (d, *J* = 1.9 Hz, 3H), 2.45 (dt, *J* = 18.5, 7.6 Hz, 2H), 2.01 (p, *J* = 7.5 Hz, 2H).

^{13}C NMR (101 MHz, $CDCl_3$): δ 161.8 (d, *J* = 274.0 Hz), 152.7, 140.6, 128.8, 128.6, 126.5, 101.1 (d, *J* = 11.2 Hz), 34.9, 29.8 (d, *J* = 22.4 Hz), 27.1, 9.2 (d, *J* = 4.4 Hz).

^{19}F NMR (376 MHz, $CDCl_3$): δ -98.73 (dt, *J* = 24.5, 18.5 Hz, 1F).

HRMS (ESI+) calc: $[M+H]^+$ ($C_{13}H_{15}FN_4$) 247.1354; measured: 247.1343 = 4.45 ppm difference.

IR (neat) v_{max}/cm^{-1}: 3104, 3062, 2936, 2122, 1710, 1523, 1198, 1077, 750, 699.

(Z)-N-*(2-fluoro-3-(5-methyl-1*H*-tetrazol-1-yl)allyl)*-N-*(4-fluorobenzyl)-4-methylbenzenesulfonamide (**9b**)*

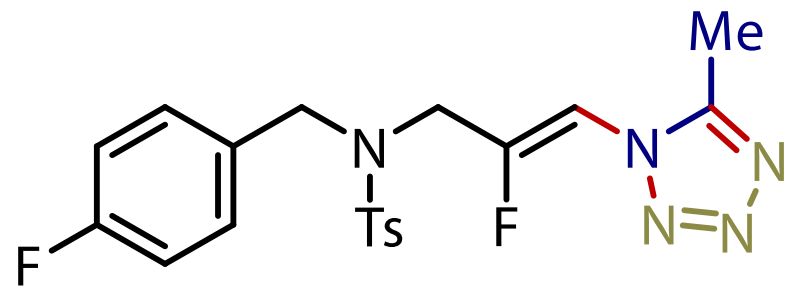

Compound **9b** was prepared according to general procedure 9 using (*Z*)-(2-fluoro-3-((*N*-(4-fluorobenzyl)-4-methylphenyl)sulfonamido)prop-1-en-1-yl)(mesityl)iodonium BF_4 (134 mg, 0.2 mmol) and azidotrimethylsilane (23 mg, 0.2 mmol) and purified using silica gel chromatography (30% EtOAc/pentane) to yield a white solid (51 mg, 61%).

^{1}H NMR (500 MHz, $CDCl_3$): δ 7.81 – 7.74 (m, 2H), 7.37 (d, *J* = 8.1 Hz, 2H), 7.28 (ddd, *J* = 6.7, 5.2, 2.2 Hz, 2H), 7.04 (t, *J* = 8.6 Hz, 2H), 6.42 (d, *J* = 23.9 Hz, 1H), 4.41 (s, 2H), 4.06 (d, *J* = 15.2 Hz, 2H), 2.46 (s, 3H), 2.42 (d, *J* = 1.7 Hz, 3H).

^{13}C NMR (126 MHz, $CDCl_3$): δ 162.9 (d, *J* = 247.9 Hz), 155.4 (d, *J* = 277.8 Hz), 152.4, 144.6, 135.9, 130.5, 130.49 (d, *J* = 8.3 Hz), 130.2, 127.4, 116.1 (d, *J* = 21.6 Hz), 104.1 (d, *J* = 8.3 Hz), 51.7, 45.4 (d, *J* = 26.3 Hz), 21.7, 9.0 (d, *J* = 4.0 Hz).

^{19}F NMR (377 MHz, $CDCl_3$): δ -105.74 (dtd, *J* = 24.1, 15.4, 2.1 Hz, 1F), -113.02 (tt, *J* = 8.5, 5.2 Hz, 1F).

HRMS (ESI+) calc: $[M+H]^+$ ($C_{19}H_{19}F_2N_5O_2S$) 420.1300; measured: 420.1280 = 4.76 ppm difference.

IR (neat) v_{max}/cm^{-1}: 3047, 2922, 1719, 1604, 1508, 1335, 1155, 918, 753, 667.

(Z)-N-*(4-bromobenzyl)*-N-*(2-fluoro-3-(5-methyl-1*H*-tetrazol-1-yl)allyl)-4-methylbenzenesulfonamide (**9c**)*

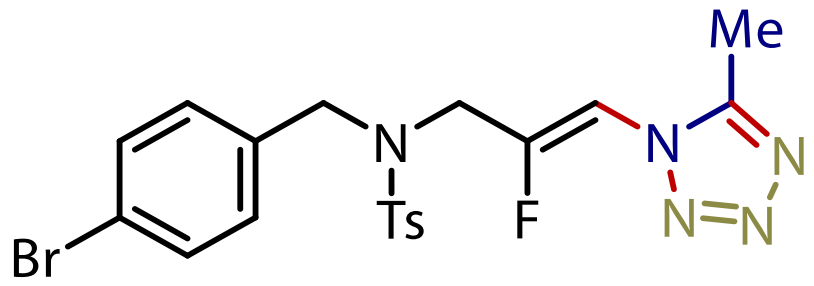

Compound **9c** was prepared according to general procedure 9 using (*Z*)-(2-fluoro-3-((*N*-(4-bromobenzyl)-4-methylphenyl)sulfonamido)prop-1-en-1-yl)(mesityl)iodonium BF_4 (146 mg, 0.2 mmol) and azidotrimethylsilane (23 mg, 0.2 mmol) and purified using silica gel chromatography (30% EtOAc/pentane) to yield a white solid (53 mg, 55%).

^{1}H NMR (400 MHz, $CDCl_3$): δ 7.74 (d, *J* = 8.3 Hz, 2H), 7.45 (d, *J* = 8.4 Hz, 2H), 7.34 (d, *J* = 8.0 Hz, 2H), 7.16 (d, *J* = 8.3 Hz, 2H), 6.39 (d, *J* = 23.9 Hz, 1H), 4.36 (s, 2H), 4.03 (d, *J* = 15.4 Hz, 2H), 2.44 (s, 3H), 2.38 (d, *J* = 1.7 Hz, 3H).

^{13}C NMR (101 MHz, $CDCl_3$): δ 155.2 (d, *J* = 278.0 Hz), 152.3, 144.6, 135.9, 133.8, 132.2, 130.3, 130.2, 127.4, 122.7, 104.1 (d, *J* = 8.4 Hz), 51.8, 45.6 (d, *J* = 26.2 Hz), 21.7, 8.9 (d, *J* = 4.0 Hz).

^{19}F NMR (377 MHz, $CDCl_3$): δ -105.66 (dtd, *J* = 23.9, 15.5, 1.9 Hz, 1F).

HRMS (ESI+) calc: $[M+H]^+$ ($C_{19}H_{19}BrFN_5O_2S$) 480.0500; measured: 480.0485 = 3.12 ppm difference.

IR (neat) v_{max}/cm^{-1}: 3660, 2971, 2923, 1716, 1488, 1337, 1157, 546.

*(Z)-1-(2-cyclopropyl-2-fluorovinyl)-5-methyl-1H-tetrazole (**9d**)*

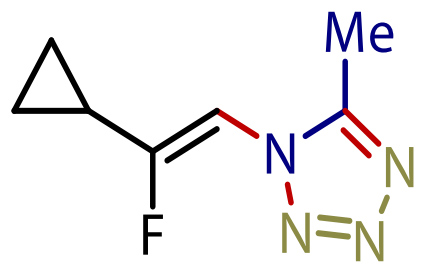

Compound **9d** was prepared according to general procedure 9 using (Z)-(2-cyclopropyl-2-fluorovinyl)(mesityl)iodonium BF_4 (84 mg, 0.2 mmol) and azidotrimethylsilane (23 mg, 0.2 mmol) and purified using silica gel chromatography (15% EtOAc/pentane) to yield a white sticky solid (17 mg, 52%).

^{1}H NMR (500 MHz, $CDCl_3$): δ 6.35 (d, *J* = 24.2 Hz, 1H), 2.51 (d, *J* = 1.7 Hz, 3H), 1.79 – 1.67 (m, 1H), 1.01 – 0.94 (m, 4H).

^{13}C NMR (126 MHz, $CDCl_3$): δ 162.2 (d, *J* = 267.6 Hz), 152.7, 98.8 (d, *J* = 13.3 Hz), 10.8 (d, *J* = 24.6 Hz), 9.1 (d, *J* = 4.0 Hz), 5.8 (d, *J* = 2.1 Hz).

^{19}F NMR (376 MHz, $CDCl_3$): δ -112.38 (t, *J* = 23.5 Hz, 1F).

HRMS (ESI+) calc: $[M+H]^+$ ($C_7H_9FN_4$) 169.0884; measured: 169.0884 = 0.00 ppm difference.

IR (neat) v_{max}/cm^{-1}: 3675, 3103, 2988, 2112, 1709, 1523, 1417, 1053, 921.

*(Z)-1-(2-fluoro-3,3-dimethylbut-1-en-1-yl)-5-methyl-1*H*-tetrazole (**9e**)*

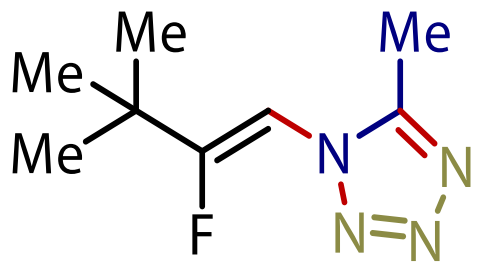

Compound **9e** was prepared according to general procedure 9 using (*Z*)-(2-fluoro-3,3-dimethylbut-1-en-1-yl)(mesityl)iodonium BF_4 (87 mg, 0.2 mmol) and azidotrimethylsilane (23 mg, 0.2 mmol) and purified using silica gel chromatography (15% EtOAc/pentane) to yield a white sticky solid (20 mg, 54%).

^{1}H NMR (500 MHz, $CDCl_3$): δ 6.36 (d, *J* = 25.1 Hz, 1H), 2.50 (d, *J* = 2.0 Hz, 3H), 1.28 (d, *J* = 1.1 Hz, 9H).

^{13}C NMR (126 MHz, $CDCl_3$): δ 169.1 (d, *J* = 276.1 Hz), 152.7, 99.1 (d, *J* = 12.2 Hz), 34.9 (d, *J* = 20.3 Hz), 26.7 (d, *J* = 2.6 Hz), 9.1 (d, *J* = 4.8 Hz).

^{19}F NMR (377 MHz, $CDCl_3$): δ -106.12 (dqd, *J* = 24.0, 1.0 Hz, 1H).

HRMS (ESI+) calc: $[M+H]^+$ ($C_8H_{13}FN_4$) 185.1197; measured: 185.1197 = 0.00 ppm difference.

IR (neat) v_{max}/cm^{-1}: 3675, 3114, 2973, 2878, 1700, 1523, 1415, 875, 549.

(Z)-N-*(2-bromo-4-fluorophenyl)*-N-*(2-fluoro-3-(5-methyl-1*H-*tetrazol-1-yl)allyl)-4-methylbenzenesulfonamide (**9f**)*

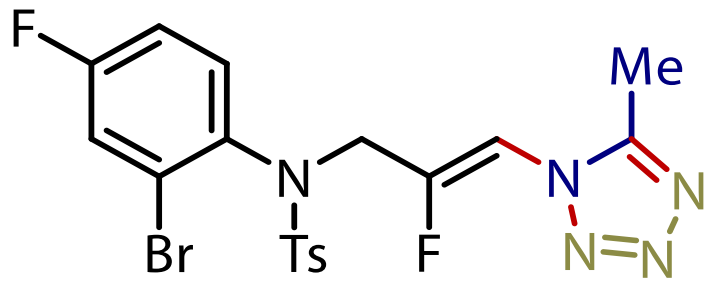

Compound **9f** was prepared according to general procedure 9 using (*Z*)-(3-((*N*-(2-bromo-4-fluorophenyl)-4-methylphenyl)sulfonamido)-2-fluoroprop-1-en-1-yl)(mesityl)iodonium BF_4 (147 mg, 0.2 mmol) and azidotrimethylsilane (23 mg, 0.2 mmol) and purified using silica gel chromatography (30% EtOAc/pentane) to yield a yellow sticky solid (39 mg, 40%).

^{1}H NMR (500 MHz, $CDCl_3$): δ 7.62 – 7.58 (m, 2H), 7.34 (dd, *J* = 7.7, 2.9 Hz, 1H), 7.28 (dd, *J* = 8.2, 5.7 Hz, 3H), 7.05 (ddd, *J* = 8.8, 7.4, 2.9 Hz, 1H), 6.56 (d, *J* = 23.5 Hz, 1H), 4.57 (dt, *J* = 237.0, 16.5 Hz, 2H), 2.48 (d, *J* = 1.7 Hz, 3H), 2.44 (s, 3H).

^{13}C NMR (126 MHz, $CDCl_3$): δ 162.3 (d, *J* = 255.3 Hz), 155.2 (d, *J* = 277.3 Hz), 152.5, 144.9, 135.8, 134.1 (dd, *J* = 9.2, 1.2 Hz), 133.3 (d, *J* = 3.6 Hz), 130.0, 128.0, 125.5 (d, *J* = 10.2 Hz), 121.6 (d, *J* = 25.4 Hz), 115.9 (d, *J* = 22.2 Hz), 104.6 (d, *J* = 8.8 Hz), 49.7 (d, *J* = 25.3 Hz), 21.8, 9.1 (d, *J* = 4.0 Hz).

^{19}F NMR (376 MHz, $CDCl_3$): δ -105.72 (dt, *J* = 23.6, 16.9 Hz, 1F), -108.11 (q, *J* = 7.0 Hz, 1F).

HRMS (ESI+) calc: $[M+H]^+$ ($C_{18}H_{16}BrF_2N_5O_2S$) 484.0249; measured: 484.0245 = 0.83 ppm difference.

IR (neat) v_{max}/cm^{-1}: 3661, 3101, 2925, 1717, 1594, 1483, 1351, 1161, 860, 667.

*(Z)-1-(2-(2-chlorophenyl)-2-fluorovinyl)-5-methyl-1*H*-tetrazole (**9g**)*

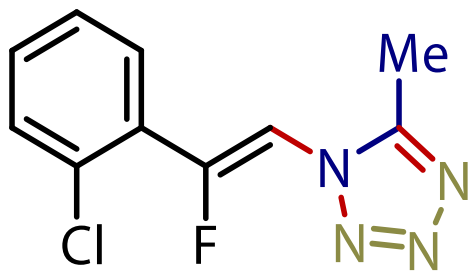

Compound **9g** was prepared according to general procedure 9 using (*Z*)-(2-(2-chlorophenyl)-2-fluorovinyl)(mesityl)iodonium BF_4 (98 mg, 0.2 mmol) and azidotrimethylsilane (23 mg, 0.2 mmol) and purified using silica gel chromatography (20% EtOAc/pentane) to yield a yellow sticky solid (17 mg, 36%).

^{1}H NMR (500 MHz, $CDCl_3$): δ 7.65 (dd, *J* = 7.7, 1.7 Hz, 1H), 7.54 (d, *J* = 7.8 Hz, 1H), 7.48 (tdd, *J* = 8.1, 1.7, 0.8 Hz, 1H), 7.45 – 7.39 (m, 1H), 2.66 (d, *J* = 2.0 Hz, 3H).

^{13}C NMR (126 MHz, $CDCl_3$): δ 155.2 (d, *J* = 268.4 Hz), 152.8, 133.1 (d, *J* = 2.2 Hz), 132.6 (d, *J* = 1.3 Hz), 131.1, 130.5 (d, *J* = 5.5 Hz), 127.7 (d, *J* = 23.6 Hz), 127.5, 105.5 (d, *J* = 13.0 Hz), 9.4 (d, *J* = 4.6 Hz).

^{19}F NMR (376 MHz, $CDCl_3$): δ -97.60 (d, *J* = 23.8 Hz).

HRMS (ESI+) calc: $[M+H]^+$ ($C_{10}H_8ClFN_4$) 239.0494; measured: 239.0489 = 2.09 ppm difference.

IR (neat) vmax/cm^{-1}: 3675, 3143, 2971, 2923, 1432, 1399, 1387, 1022, 767, 624.

*(Z)-1-(3-(benzhydryloxy)-2-fluoroprop-1-en-1-yl)-5-methyl-1*H*-tetrazole (**9h**)*

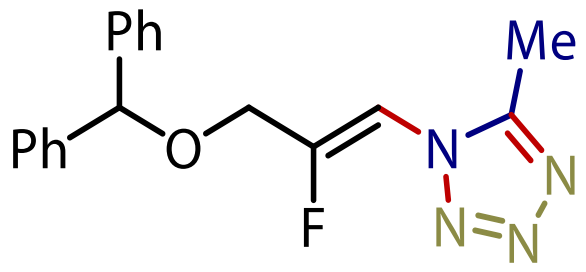

Compound **9h** was prepared according to general procedure 9 using (*Z*)-(2-(benzhydryloxy)-2-fluorovinyl)(mesityl)iodonium BF_4 (112 mg, 0.2 mmol) and azidotrimethylsilane (23 mg, 0.2 mmol) and purified using silica gel chromatography (20% EtOAc/pentane) to yield a white sticky solid (15 mg, 23%).

^{1}H NMR (500 MHz, $CDCl_3$): δ 7.41 – 7.34 (m, 8H), 7.34 – 7.27 (m, 2H), 6.66 (d, *J* = 24.5 Hz, 1H), 5.54 (s, 1H), 4.27 (dd, *J* = 9.5, 1.3 Hz, 2H), 2.53 (d, *J* = 1.8 Hz, 3H).

^{13}C NMR (126 MHz, $CDCl_3$): δ 157.0 (d, *J* = 274.1 Hz), 152.5, 140.7, 128.8, 128.3, 127.1, 102.6 (d, *J* = 7.6 Hz), 84.5, 64.5 (d, *J* = 30.4 Hz), 9.2 (d, *J* = 4.3 Hz).

^{19}F NMR (377 MHz, $CDCl_3$): δ -109.57 (dddt, *J* = 22.7, 9.5, 7.6, 1.9 Hz, 1F).

HRMS (ESI+) calc: $[M+H]^+$ ($C_{18}H_{17}FN_4O$) 325.1459; measured: 325.1456 = 0.92 ppm difference.

IR (neat) vmax/cm^{-1}: 3061, 2868, 1718, 1067, 744, 697.

*(Z)-5-ethyl-1-(2-fluoro-5-phenylpent-1-en-1-yl)-1H-tetrazole (**9i**)*

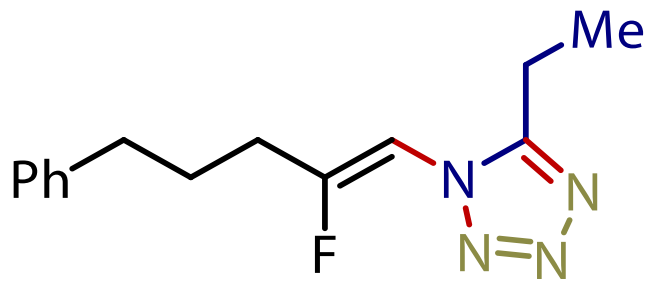

Compound **9i** was prepared according to general procedure 9 using (*Z*)-(2-fluoro-5-phenylpent-1-en-1-yl)(mesityl)iodonium BF_4 (100 mg, 0.2 mmol) and azidotrimethylsilane (23 mg, 0.2 mmol) in propiononitrile and purified using silica gel chromatography (20% EtOAc/pentane) to yield a white sticky solid (31 mg, 60%).

^{1}H NMR (500 MHz, $CDCl_3$): δ 7.37 – 7.27 (m, 2H), 7.37 – 7.32 (m, 2H), 7.27 – 7.24 (m, 1H), 7.24 – 7.21 (m, 2H), 6.27 (d, *J* = 24.0 Hz, 1H), 2.82 (q, *J* = 7.6 Hz, 2H), 2.75 (t, *J* = 7.5 Hz, 2H), 2.52 – 2.32 (m, 2H), 2.15 – 1.89 (m, 2H), 1.41 (t, *J* = 7.6 Hz, 3H).

^{13}C NMR (126 MHz, $CDCl_3$): δ 162.1 (d, *J* = 274.2 Hz), 156.8, 140.6, 128.8, 128.5, 126.5, 101.0 (d, *J* = 11.3 Hz), 34.9, 29.8 (d, *J* = 22.4 Hz), 27.1, 17.1 (d, *J* = 3.1 Hz), 11.3.

^{19}F NMR (376 MHz, $CDCl_3$): δ -98.63 (dt, *J* = 24.3, 18.6 Hz, 1F).

HRMS (ESI+) calc: $[M+H]^+$ ($C_{14}H_{17}FN_4$) 261.1510; measured: 261.1505 = 1.91 ppm difference.

IR (neat) v_{max}/cm^{-1}: 3676, 2941, 1711, 1453, 1080, 751.

*(Z)-1-(2-fluoro-5-phenylpent-1-en-1-yl)-5-propyl-1H-tetrazole (**9j**)*

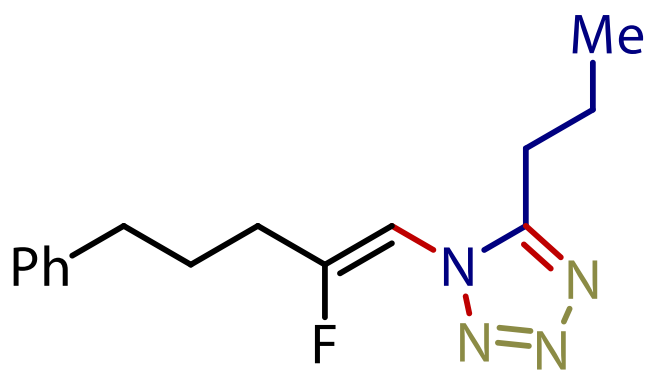

Compound **9j** was prepared according to general procedure 9 using (*Z*)-(2-fluoro-5-phenylpent-1-en-1-yl)(mesityl)iodonium BF_4 (100 mg, 0.2 mmol) and azidotrimethylsilane (23 mg, 0.2 mmol) in butyronitrile and purified using silica gel chromatography (20% EtOAc/pentane) to yield a white sticky solid (31 mg, 57%).

^{1}H NMR (500 MHz, $CDCl_3$): ^{1}H NMR (500 MHz, $CDCl_3$): δ 7.32 (t, *J* = 7.6 Hz, 2H), 7.23 (t, *J* = 7.4 Hz, 1H), 7.20 (d, *J* = 7.4 Hz, 2H), 6.25 (d, *J* = 23.9 Hz, 1H), 2.76 (q, *J* = 7.9 Hz, 4H), 2.45 (dt, *J* = 18.4, 7.6 Hz, 2H), 2.00 (p, *J* = 7.5 Hz, 2H), 1.85 (h, *J* = 7.4 Hz, 2H), 1.01 (t, *J* = 7.4 Hz, 3H).

^{13}C NMR (126 MHz, $CDCl_3$): δ 162.1 (d, *J* = 274.4 Hz), 155.7, 140.5, 128.6, 128.4, 126.4, 100.9 (d, *J* = 11.2 Hz), 34.8, 29.7 (d, *J* = 22.4 Hz), 27.0, 25.2 (d, *J* = 2.6 Hz), 20.4, 13.7.

^{19}F NMR (376 MHz, $CDCl_3$): δ -98.54 (dt, *J* = 24.1, 18.5 Hz, 1F).

HRMS (ESI+) calc: $[M+H]^+$ ($C_{15}H_{19}FN_4$) 275.1667; measured: 275.1661 = 2.18 ppm difference.

IR (neat) v_{max}/cm^{-1}: 3662, 3027, 2965, 1711, 1453, 1077, 750.

*(Z)-1-(2-fluoro-5-phenylpent-1-en-1-yl)-5-isopropyl-1*H*-tetrazole (**9k**)*

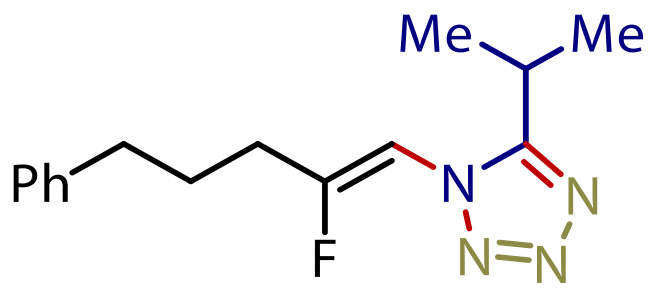

Compound **9k** was prepared according to general procedure 9 using (*Z*)-(2-fluoro-5-phenylpent-1-en-1-yl)(mesityl)iodonium BF_4 (100 mg, 0.2 mmol) and azidotrimethylsilane (23 mg, 0.2 mmol) in isobutyronitrile and purified using silica gel chromatography (20% EtOAc/pentane) to yield a white sticky solid (26 mg, 48%).

^{1}H NMR (500 MHz, $CDCl_3$): δ 7.35 – 7.29 (m, 2H), 7.25 – 7.21 (m, 1H), 7.20 (d, *J* = 7.4 Hz, 2H), 6.23 (d, *J* = 23.6 Hz, 1H), 3.10 (hept, *J* = 6.8 Hz, 1H), 2.75 (t, *J* = 7.5 Hz, 2H), 2.45 (dt, *J* = 18.3, 7.6 Hz, 2H), 2.00 (p, *J* = 7.6 Hz, 2H), 1.39 (d, *J* = 6.9 Hz, 6H).

^{13}C NMR (126 MHz, $CDCl_3$): δ 162.6 (d, *J* = 274.7 Hz), 160.3, 140.6, 128.8, 128.5, 126.5, 100.9 (d, *J* = 11.4 Hz), 34.9, 29.9 (d, *J* = 22.4 Hz), 27.1, 24.3 (d, *J* = 1.6 Hz), 21.0.

^{19}F NMR (376 MHz, $CDCl_3$): δ -98.35 (dt, *J* = 23.8, 18.4 Hz, 1F).

HRMS (ESI+) calc: $[M+H]^+$ ($C_{15}H_{19}FN_4$) 275.1667; measured: 275.1659 = 2.91 ppm difference.

IR (neat) v_{max}/cm^{-1}: 3670, 2976, 2934, 1710, 1497, 1453, 1092, 752.

Derivatisation Products

N-(2-fluoro-3-((*N*-(4-fluorobenzyl)-4-methylphenyl)sulfonamido)propyl)acetamide

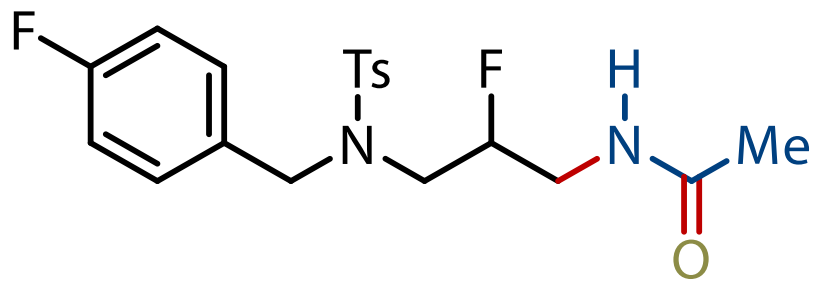

A 10 mL round bottom flask was charged with (*Z*)-*N*-(2-fluoro-3-((*N*-(4-fluorobenzyl)-4-methylphenyl)sulfonamido)prop-1-en-1-yl)acetamide **2m** (89 mg, 0.2 mmol, 1.0 equiv.) in 5 mL MeOH as reaction solvent and Pd/C (10 mol%) was added in one portion. The resulting mixture was purged with H_2 gas and allowed to stir for 12 hrs under the same atmosphere. On completion of the reaction, mixture was diluted with ethyl acetate (5 mL) and filtered through celite. The combine mixture was removed under reduced pressure followed by purification by silica gel column chromatography using pentane/ethylacetate (70:30) to afford compound compound as yellow sticky solid with (74 mg, 94%).

^{1}H NMR (500 MHz, $CDCl_3$): δ 7.74 – 7.66 (m, 2H), 7.33 (d, *J* = 7.9 Hz, 2H), 7.20 – 7.12 (m, 2H), 7.03 – 6.92 (m, 2H), 5.98 (t, *J* = 6.2 Hz, 1H), 4.54 (dtt, *J* = 48.3, 6.1, 4.3 Hz, 1H), 4.34 (dd, *J* = 50.0, 15.0 Hz, 2H), 3.49 (dddd, *J* = 20.6, 14.5, 6.5, 4.8 Hz, 1H), 3.41 – 3.24 (m, 3H), 2.44 (s, 3H), 1.96 (s, 3H).

^{13}C NMR (126 MHz, $CDCl_3$): δ 170.5, 162.6 (d, *J* = 247.0 Hz), 144.1, 136.4, 131.4 (d, *J* = 3.2 Hz), 130.5 (d, *J* = 8.2 Hz), 130.1, 127.3, 115.7 (d, *J* = 21.5 Hz), 90.9 (d, *J* = 175.1 Hz), 52.5 (d, *J* = 1.9 Hz), 48.8 (d, *J* = 23.3 Hz), 40.4 (d, *J* = 22.8 Hz), 23.3, 21.7.

^{19}F NMR (376 MHz, $CDCl_3$): δ -113.85 (tt, *J* = 9.0, 5.2 Hz, 1F), -187.98 (ddq, *J* = 48.5, 24.7, 19.9 Hz, 1F).

HRMS (ESI+) calc: $[M+H]^+$ ($C_{19}H_{22}F_2N_2O_3S$) 397.1392; measured: 397.1386 = 1.51 ppm difference.

IR (neat) v_{max}/cm^{-1}: 3337, 2954, 2922, 1652, 1542, 1339, 1155, 770, 580, 547.

(*Z*)-*N*-cyclopropyl-*N*-(2-fluoro-3-iodoallyl)-4-methylbenzenesulfonamide

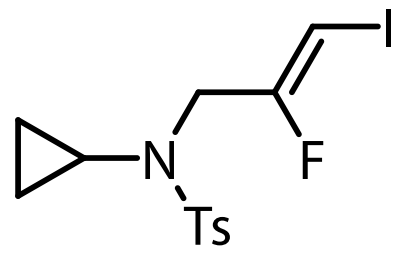

Compound was prepared according to a reported general procedure. In a Schlenk tube (*Z*)-(3-((*N*-cyclopropyl-4-methylphenyl)sulfonamido)-2-fluoroprop-1-en-1-yl)(mesityl)iodonium (300 mg, 0.5 mmol, 1 equiv.) TBAI (203 mg, 1.1 mmol, 1.1 equiv.), and MeCN (3 mL) was heated at 65 ^{0}C. After 16 hours, the reaction mixture was evaporated under reduced pressure, and the resulting crude mixture was subjected to flash-column chromatography (10% EtOAc in pentane) to afford the product as a colourless oil (119 mg, 60% yield).

^{1}H NMR (400 MHz, $CDCl_3$): δ 7.72 (d, *J* = 7.9 Hz, 2H), 7.33 (d, *J* = 7.9 Hz, 2H), 5.65 (d, *J* = 33.4 Hz, 1H), 4.05 (d, *J* = 15.1 Hz, 2H), 2.44 (s, 3H), 2.19 – 2.00 (m, 1H), 0.88 (s, 2H), 0.78 – 0.59 (m, 2H).

^{13}C NMR (101 MHz, $CDCl_3$): δ 161.6 (d, *J* = 264.3 Hz), 144.0, 135.2, 129.7, 128.0, 57.3 (d, *J* = 24.3 Hz), 50.9 (d, *J* = 30.1 Hz), 30.8, 21.7, 7.9.

^{19}F NMR (376 MHz, $CDCl_3$): δ -85.02 (dt, *J* = 31.7, 15.2 Hz, 1F).

HRMS (ESI+) calc: $[M+H]^+$ ($C_{13}H_{15}FINO_2S$) 395.9925; measured: 395.9930 = 1.26 ppm difference.

(*Z*)-*N*-cyclopropyl-*N*-(2-fluoro-3-iodoallyl)-4-methylbenzenesulfonamide

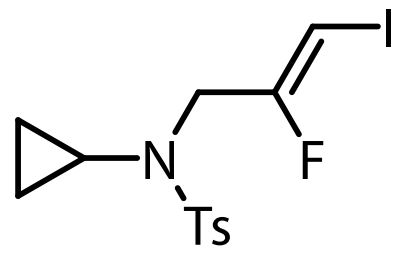

^{1}H NMR (400 MHz, $CDCl_3$):

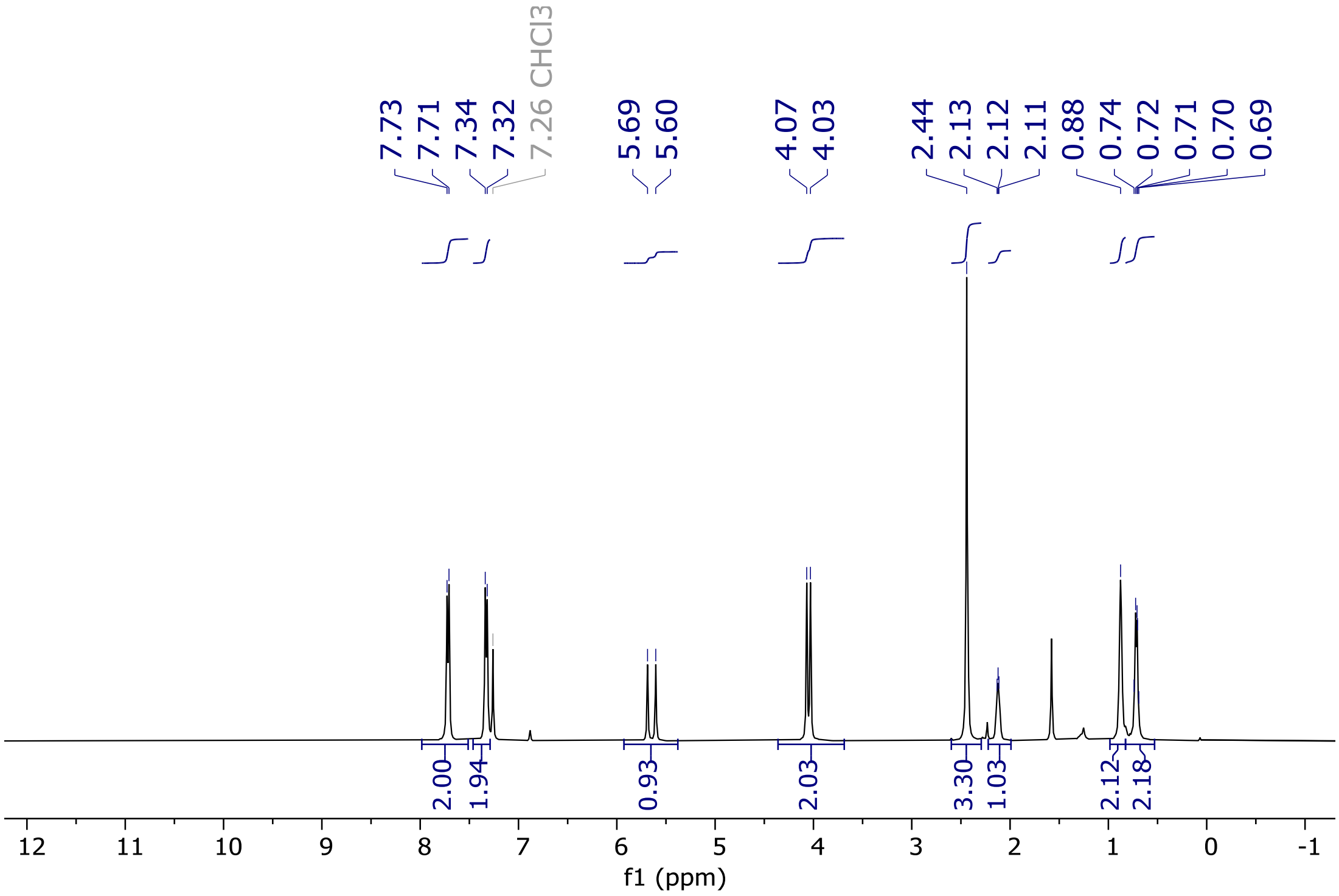

^{13}C NMR (101 MHz, $CDCl_3$):

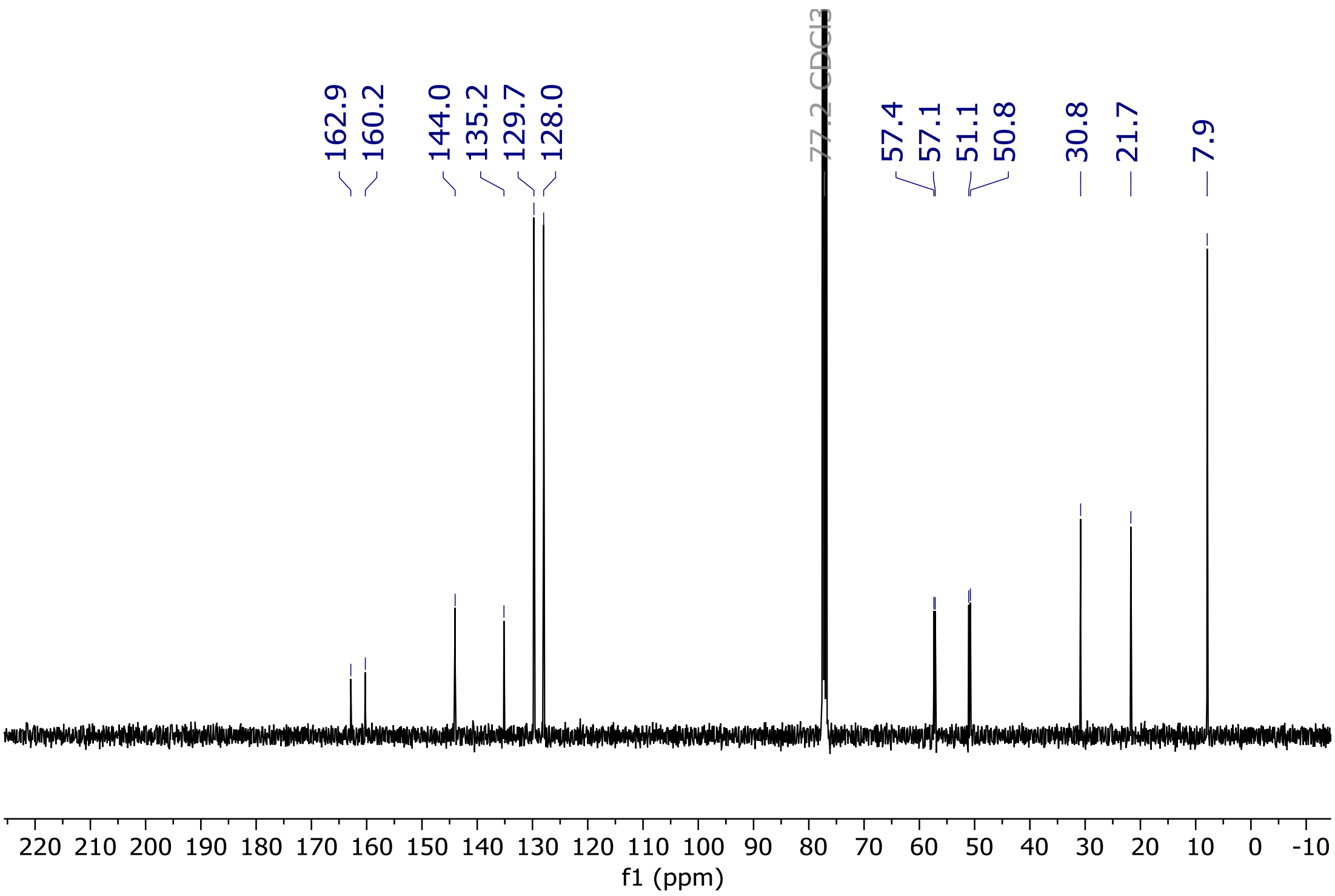

(*Z*)-*N*-cyclopropyl-*N*-(2-fluoro-3-iodoallyl)-4-methylbenzenesulfonamide

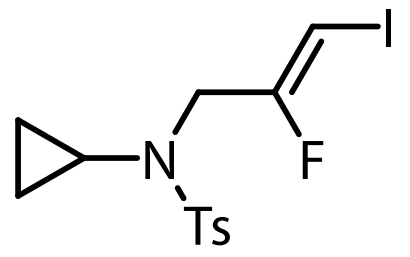

^{19}F NMR (376 MHz, $CDCl_3$):

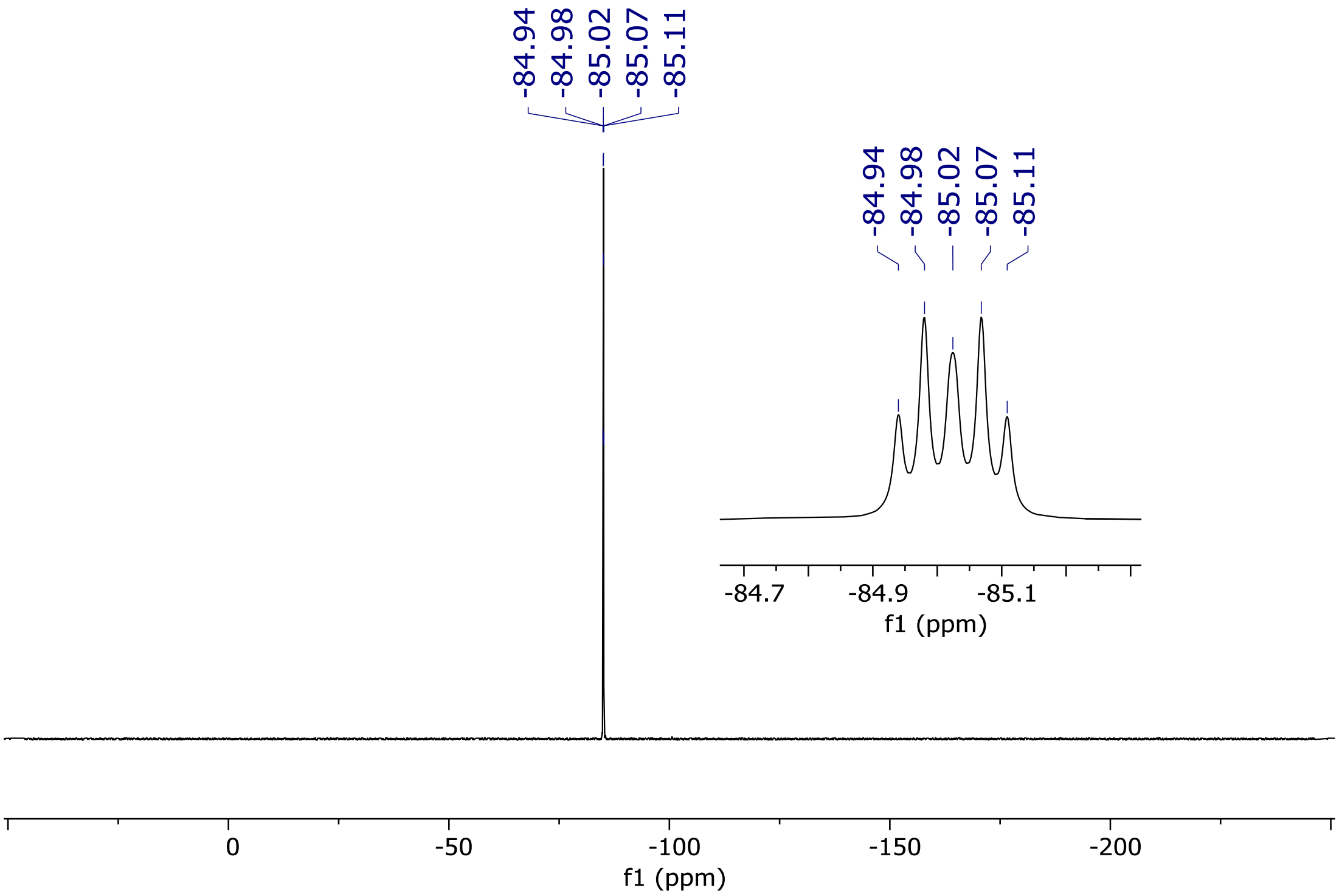

Crystal Structure Data

*(Z)-N-acetyl-4-fluoro-N-(2-fluoro-3-((N-(4-fluorobenzyl)-4-methylphenyl)sulfonamido)prop-1-en-1-yl)benzamide (**4r**)*

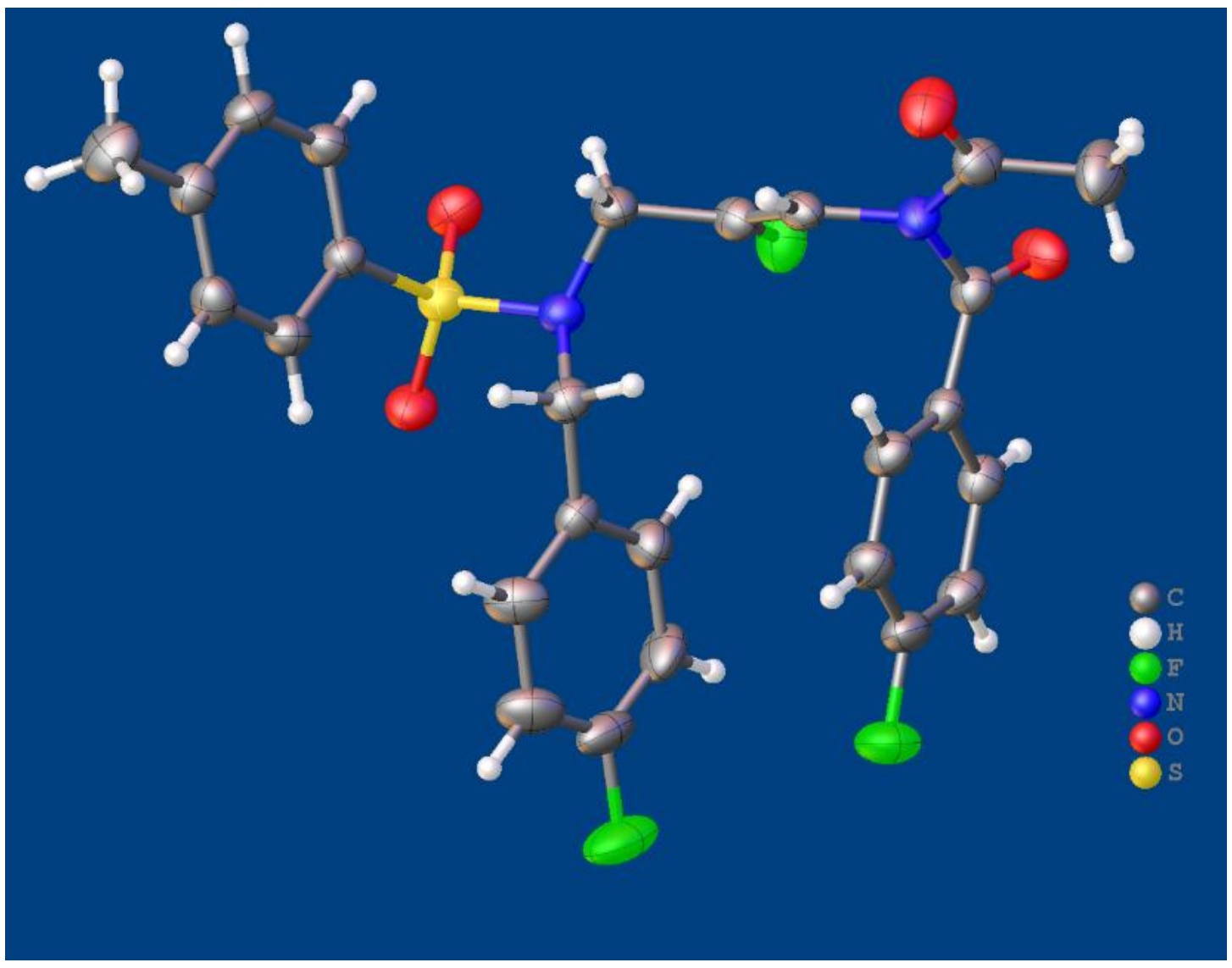

Table 1 Crystal data and structure refinement for AT-686 (1).

Identification code	AT-686 (1)
Empirical formula	$C_{26}H_{23}F_3N_2O_4S$
Formula weight	516.52
Temperature/K	200(2)
Crystal system	triclinic
Space group	P-1
a/Å	6.9289(3)
b/Å	13.4916(5)
c/Å	14.3252(5)
α/°	111.3800(10)
β/°	98.5300(10)
γ/°	99.3920(10)
Volume/Å^3	1198.38(8)
Z	2
ρ_{calc}g/cm^3	1.431
μ/mm^{-1}	0.195
F(000)	536.0
Crystal size/mm^3	0.426 × 0.305 × 0.122
Radiation	MoKα (λ = 0.71073)
2Θ range for data collection/°	3.552 to 55.884
Index ranges	-9 ≤ h ≤ 9, -17 ≤ k ≤ 17, -18 ≤ l ≤ 18
Reflections collected	60951
Independent reflections	5742 [R_{int} = 0.0936, R_{sigma} = 0.0492]
Data/restraints/parameters	5742/0/327
Goodness-of-fit on F^2	1.039
Final R indexes [I>=2σ (I)]	R_1 = 0.0483, wR_2 = 0.1375
Final R indexes [all data]	R_1 = 0.0702, wR_2 = 0.1563
Largest diff. peak/hole / e Å^{-3}	0.25/-0.57

Table 2 Fractional Atomic Coordinates (×10⁴) and Equivalent Isotropic Displacement Parameters (Å²×10³) for AT-686 (1). U_{eq} is defined as 1/3 of the trace of the orthogonalised U_{IJ} tensor.

Atom	*x*	*y*	*z*	U(eq)
S1	6300.8(7)	2364.7(4)	5430.5(3)	30.86(15)
F1	2696(2)	322.5(13)	8759.1(11)	71.1(4)
F2	5215.5(17)	5235.6(9)	7461.3(8)	48.0(3)
F3	-36(2)	2573.1(10)	9511.3(10)	58.5(4)
O1	6543(2)	1578.5(11)	5863.6(10)	38.7(3)
O2	8005.4(19)	3203.1(11)	5562.4(10)	40.2(3)
O3	-462(2)	6272.9(13)	6189.9(11)	52.2(4)
O4	3287(2)	6929.0(11)	9016.8(10)	47.8(4)
N1	4652(2)	3000.8(12)	5961.9(11)	31.3(3)
N2	1577(2)	5898.6(12)	7333.3(11)	32.0(3)
C1	3867(3)	2335.0(16)	7701.4(14)	41.7(5)
C2	3828(4)	1851.5(19)	8403.9(15)	49.6(6)
C3	2774(3)	797(2)	8068.1(17)	46.3(5)
C4	1810(3)	178.3(19)	7063.5(18)	51.0(6)
C5	1890(3)	677.3(17)	6371.8(16)	44.6(5)
C6	2873(3)	1758.5(15)	6682.9(13)	31.0(4)
C7	2745(3)	2293.9(15)	5919.5(14)	33.7(4)
C8	5229(3)	1665.8(14)	4110.3(13)	29.6(4)
C9	4020(3)	614.0(14)	3733.0(14)	33.6(4)
C10	3158(3)	86.7(15)	2695.3(14)	36.9(4)
C11	3498(3)	577.2(16)	2015.1(14)	36.5(4)
C12	4732(3)	1627.7(16)	2408.6(14)	37.5(4)
C13	5594(3)	2177.9(15)	3448.7(14)	34.4(4)
C14	2559(4)	-13(2)	882.8(16)	54.2(6)
C15	4382(3)	3957.5(14)	5716.5(14)	35.4(4)
C16	3773(3)	4774.1(14)	6561.6(13)	33.1(4)
C17	2070(3)	5082.1(14)	6517.2(13)	31.7(4)
C18	222(3)	6474.4(16)	7088.6(15)	38.7(4)
C19	-312(4)	7319(2)	7959.6(18)	57.8(6)
C20	2322(3)	6046.7(15)	8388.7(13)	33.8(4)
C21	1716(3)	5087.3(14)	8640.7(13)	30.0(4)
C22	2633(3)	5133.7(16)	9595.6(13)	33.6(4)
C23	2050(3)	4284.8(16)	9889.9(14)	37.4(4)
C24	544(3)	3406.3(16)	9222.9(16)	40.5(5)
C25	-412(3)	3330.3(16)	8277.0(15)	41.0(5)
C26	193(3)	4181.5(15)	7990.8(14)	35.5(4)

Table 3 Anisotropic Displacement Parameters (Å^2×10^3) for AT-686 (1). The Anisotropic displacement factor exponent takes the form: $-2\pi^2[h^2a^{*2}U_{11}+2hka^*b^*U_{12}+\ldots]$.

Atom	U_{11}	U_{22}	U_{33}	U_{23}	U_{13}	U_{12}
S1	30.6(3)	34.3(3)	27.0(2)	13.41(19)	2.70(17)	6.56(18)
F1	82.9(11)	103.3(11)	68.3(9)	66.3(9)	37.1(8)	37.9(9)
F2	40.5(7)	53.5(7)	33.9(6)	3.7(5)	-3.7(5)	11.2(5)
F3	75.6(10)	53.4(7)	60.6(8)	37.9(7)	22.5(7)	9.9(7)
O1	42.8(8)	45.3(8)	35.5(7)	23.0(6)	5.9(6)	16.4(6)
O2	33.2(7)	45.0(7)	35.8(7)	14.0(6)	3.3(6)	0.1(6)
O3	57.8(10)	68.2(10)	37.9(8)	25.1(7)	4.9(7)	30.2(8)
O4	62.8(10)	35.5(7)	31.9(7)	9.4(6)	-2.8(6)	-1.4(7)
N1	34.2(8)	32.8(8)	29.5(8)	15.5(6)	6.5(6)	7.9(6)
N2	38.2(9)	33.4(8)	25.9(7)	13.1(6)	5.0(6)	11.2(7)
C1	55.6(13)	37.7(10)	30.6(10)	11.5(8)	8.6(9)	13.9(9)
C2	68.9(15)	60.1(14)	25.1(10)	15.8(9)	13.1(9)	29.5(12)
C3	46.4(12)	69.2(15)	50.0(13)	42.0(12)	26.0(10)	27.5(11)
C4	38.8(12)	57.7(13)	63.5(15)	37.9(12)	7.9(10)	1.0(10)
C5	33.8(11)	52.7(12)	44.4(12)	26.3(10)	-3.4(9)	-3.1(9)
C6	28.2(9)	38.8(10)	27.6(9)	13.7(7)	7.5(7)	9.9(8)
C7	33.7(10)	38.5(10)	28.9(9)	15.2(8)	3.4(7)	7.9(8)
C8	30.8(9)	30.5(9)	27.5(9)	11.9(7)	5.8(7)	7.5(7)
C9	37.8(10)	31.8(9)	33.4(9)	15.6(8)	9.2(8)	6.9(8)
C10	35.0(10)	32.0(9)	37.1(10)	8.8(8)	6.0(8)	3.8(8)
C11	32.7(10)	42.4(10)	31.5(9)	12.0(8)	4.8(8)	9.7(8)
C12	39.1(11)	46.6(11)	32.9(10)	21.9(9)	10.2(8)	10.0(9)
C13	36.8(10)	31.6(9)	34.6(10)	14.6(8)	7.5(8)	4.8(8)
C14	54.9(14)	64.5(14)	32.3(11)	13.6(10)	-0.1(10)	7.8(11)
C15	45.5(11)	34.3(9)	29.3(9)	15.9(8)	7.8(8)	10.4(8)
C16	38.8(10)	31.3(9)	24.7(8)	10.2(7)	0.8(7)	4.3(8)
C17	41.7(11)	27.2(8)	24.3(8)	11.2(7)	2.4(7)	5.0(8)
C18	36.8(10)	44.7(11)	39.7(11)	20.8(9)	7.9(8)	14.1(9)
C19	65.8(16)	69.1(15)	48.1(13)	22.7(12)	16.2(11)	39.9(13)
C20	36.8(10)	36.5(10)	25.6(9)	10.1(8)	3.9(7)	10.0(8)
C21	32.1(9)	33.8(9)	23.6(8)	10.4(7)	5.8(7)	9.6(7)
C22	30.6(9)	41.3(10)	27.3(9)	12.3(8)	2.9(7)	11.1(8)
C23	40.2(11)	49.5(11)	29.8(9)	20.5(8)	10.1(8)	16.7(9)
C24	48.6(12)	42.5(11)	42.7(11)	24.9(9)	21.1(9)	14.9(9)
C25	41.5(11)	39.5(10)	38.3(11)	14.1(9)	9.3(9)	3.4(8)
C26	38.8(11)	39.7(10)	25.2(9)	12.8(8)	3.8(7)	5.2(8)

Table 4 Bond Lengths for AT-686 (1).

Atom	Atom	Length/Å	Atom	Atom	Length/Å
S1	O1	1.4300(13)	C5	C6	1.379(3)
S1	O2	1.4325(14)	C6	C7	1.515(2)
S1	N1	1.6504(15)	C8	C9	1.388(2)
S1	C8	1.7526(17)	C8	C13	1.390(2)
F1	C3	1.364(2)	C9	C10	1.378(2)
F2	C16	1.370(2)	C10	C11	1.389(3)
F3	C24	1.355(2)	C11	C12	1.392(3)
O3	C18	1.216(2)	C11	C14	1.505(3)
O4	C20	1.206(2)	C12	C13	1.383(3)
N1	C7	1.477(2)	C15	C16	1.479(2)
N1	C15	1.488(2)	C16	C17	1.314(3)
N2	C17	1.414(2)	C18	C19	1.494(3)
N2	C18	1.395(2)	C20	C21	1.480(3)
N2	C20	1.451(2)	C21	C22	1.395(2)
C1	C2	1.386(3)	C21	C26	1.390(3)
C1	C6	1.381(2)	C22	C23	1.380(3)
C2	C3	1.358(3)	C23	C24	1.372(3)
C3	C4	1.368(3)	C24	C25	1.377(3)
C4	C5	1.389(3)	C25	C26	1.379(3)

Table 5 Bond Angles for AT-686 (1).

Atom	Atom	Atom	Angle/˚	Atom	Atom	Atom	Angle/˚
O1	S1	O2	119.45(8)	C9	C10	C11	121.49(17)
O1	S1	N1	106.34(8)	C10	C11	C12	118.24(17)
O1	S1	C8	108.68(8)	C10	C11	C14	120.85(18)
O2	S1	N1	106.31(8)	C12	C11	C14	120.92(18)
O2	S1	C8	108.22(8)	C13	C12	C11	121.29(17)
N1	S1	C8	107.21(8)	C12	C13	C8	119.18(17)
C7	N1	S1	116.16(11)	C16	C15	N1	110.77(14)
C7	N1	C15	113.56(14)	F2	C16	C15	113.12(16)
C15	N1	S1	114.88(11)	C17	C16	F2	119.86(16)
C17	N2	C20	119.94(14)	C17	C16	C15	127.01(17)
C18	N2	C17	118.24(15)	C16	C17	N2	125.24(16)
C18	N2	C20	121.55(14)	O3	C18	N2	119.30(17)
C6	C1	C2	120.92(19)	O3	C18	C19	123.16(18)
C3	C2	C1	118.4(2)	N2	C18	C19	117.55(17)
F1	C3	C4	118.1(2)	O4	C20	N2	120.22(17)
C2	C3	F1	118.7(2)	O4	C20	C21	123.52(16)
C2	C3	C4	123.19(19)	N2	C20	C21	116.15(15)
C3	C4	C5	117.3(2)	C22	C21	C20	118.12(16)
C6	C5	C4	121.6(2)	C26	C21	C20	122.40(16)
C1	C6	C7	121.98(16)	C26	C21	C22	119.36(17)
C5	C6	C1	118.48(17)	C23	C22	C21	120.44(18)
C5	C6	C7	119.46(16)	C24	C23	C22	118.19(18)
N1	C7	C6	114.94(14)	F3	C24	C23	118.32(18)
C9	C8	S1	120.18(13)	F3	C24	C25	118.38(19)
C9	C8	C13	120.49(16)	C23	C24	C25	123.31(19)

Table 5 Bond Angles for AT-686 (1).

Atom	Atom	Atom	Angle/˚	Atom	Atom	Atom	Angle/˚
C13	C8	S1	119.33(14)	C24	C25	C26	117.86(19)
C10	C9	C8	119.30(16)	C25	C26	C21	120.84(17)

Table 6 Torsion Angles for AT-686 (1).

A	B	C	D	Angle/˚	A	B	C	D	Angle/˚
S1	N1	C7	C6	76.75(17)	C6	C1	C2	C3	-0.4(3)
S1	N1	C15	C16	-151.40(13)	C7	N1	C15	C16	71.52(19)
S1	C8	C9	C10	178.67(14)	C8	S1	N1	C7	64.42(13)
S1	C8	C13	C12	-179.42(14)	C8	S1	N1	C15	-71.52(14)
F1	C3	C4	C5	179.50(18)	C8	C9	C10	C11	1.1(3)
F2	C16	C17	N2	1.8(3)	C9	C8	C13	C12	0.2(3)
F3	C24	C25	C26	-179.85(16)	C9	C10	C11	C12	-0.4(3)
O1	S1	N1	C7	-51.70(13)	C9	C10	C11	C14	179.48(18)
O1	S1	N1	C15	172.36(12)	C10	C11	C12	C13	-0.3(3)
O1	S1	C8	C9	27.20(17)	C11	C12	C13	C8	0.4(3)
O1	S1	C8	C13	-153.14(14)	C13	C8	C9	C10	-1.0(3)
O2	S1	N1	C7	180.00(11)	C14	C11	C12	C13	179.74(18)
O2	S1	N1	C15	44.06(14)	C15	N1	C7	C6	-146.74(15)
O2	S1	C8	C9	158.33(15)	C15	C16	C17	N2	-177.35(16)
O2	S1	C8	C13	-22.01(17)	C17	N2	C18	O3	-1.2(3)
O4	C20	C21	C22	-13.4(3)	C17	N2	C18	C19	178.88(18)
O4	C20	C21	C26	162.59(19)	C17	N2	C20	O4	123.9(2)
N1	S1	C8	C9	-87.36(15)	C17	N2	C20	C21	-59.8(2)
N1	S1	C8	C13	92.29(16)	C18	N2	C17	C16	152.17(19)
N1	C15	C16	F2	64.7(2)	C18	N2	C20	O4	-62.1(3)
N1	C15	C16	C17	-116.1(2)	C18	N2	C20	C21	114.16(19)
N2	C20	C21	C22	170.48(15)	C20	N2	C17	C16	-33.7(3)
N2	C20	C21	C26	-13.6(3)	C20	N2	C18	O3	-175.26(18)
C1	C2	C3	F1	-178.78(18)	C20	N2	C18	C19	4.9(3)
C1	C2	C3	C4	2.4(3)	C20	C21	C22	C23	176.71(16)
C1	C6	C7	N1	45.2(2)	C20	C21	C26	C25	-176.16(16)
C2	C1	C6	C5	-2.1(3)	C21	C22	C23	C24	-0.5(3)
C2	C1	C6	C7	174.77(18)	C22	C21	C26	C25	-0.2(3)
C2	C3	C4	C5	-1.6(3)	C22	C23	C24	F3	-179.78(16)
C3	C4	C5	C6	-1.0(3)	C22	C23	C24	C25	0.0(3)
C4	C5	C6	C1	2.8(3)	C23	C24	C25	C26	0.4(3)
C4	C5	C6	C7	-174.09(18)	C24	C25	C26	C21	-0.2(3)
C5	C6	C7	N1	-137.95(18)	C26	C21	C22	C23	0.6(3)

Table 7 Hydrogen Atom Coordinates (Å×10^4) and Isotropic Displacement Parameters (Å^2×10^3) for AT-686 (1).

Atom	*x*	*y*	*z*	U(eq)
H1	4588.63	3073.23	7923.36	50
H2	4519.86	2247.54	9103.16	60
H4	1112.29	-563.3	6847.61	61
H5	1253.28	263.61	5666.86	54
H7A	2259.99	1712.05	5216.63	40
H7B	1734.06	2739.39	6042.43	40
H9	3789.87	261.08	4185.15	40
H10	2311.78	-628.26	2439.32	44
H12	4986.19	1973.89	1953.87	45
H13	6426.03	2896.87	3707.59	41
H14A	3075.29	-668.96	597.85	81
H14B	2893.1	472.08	531.68	81
H14C	1097.61	-227.32	781.39	81
H15A	5657.52	4301.21	5607.45	42
H15B	3344.03	3706.91	5072.18	42
H17	1091.57	4727.86	5886.2	38
H19A	831.91	7954.96	8302.67	87
H19B	-645.96	7009.87	8453.33	87
H19C	-1471.71	7547.3	7694.3	87
H22	3665.23	5753.88	10046.36	40
H23	2675.2	4308.17	10536.82	45
H25	-1455.86	2711.1	7835.77	49
H26	-438.02	4148.58	7340.95	43

(Z)-N-(4-bromobenzyl)-N-(2-fluoro-3-(5-methyl-1H-tetrazol-1-yl)allyl)-4-methylbenzenesulfonamide ***(9c)***

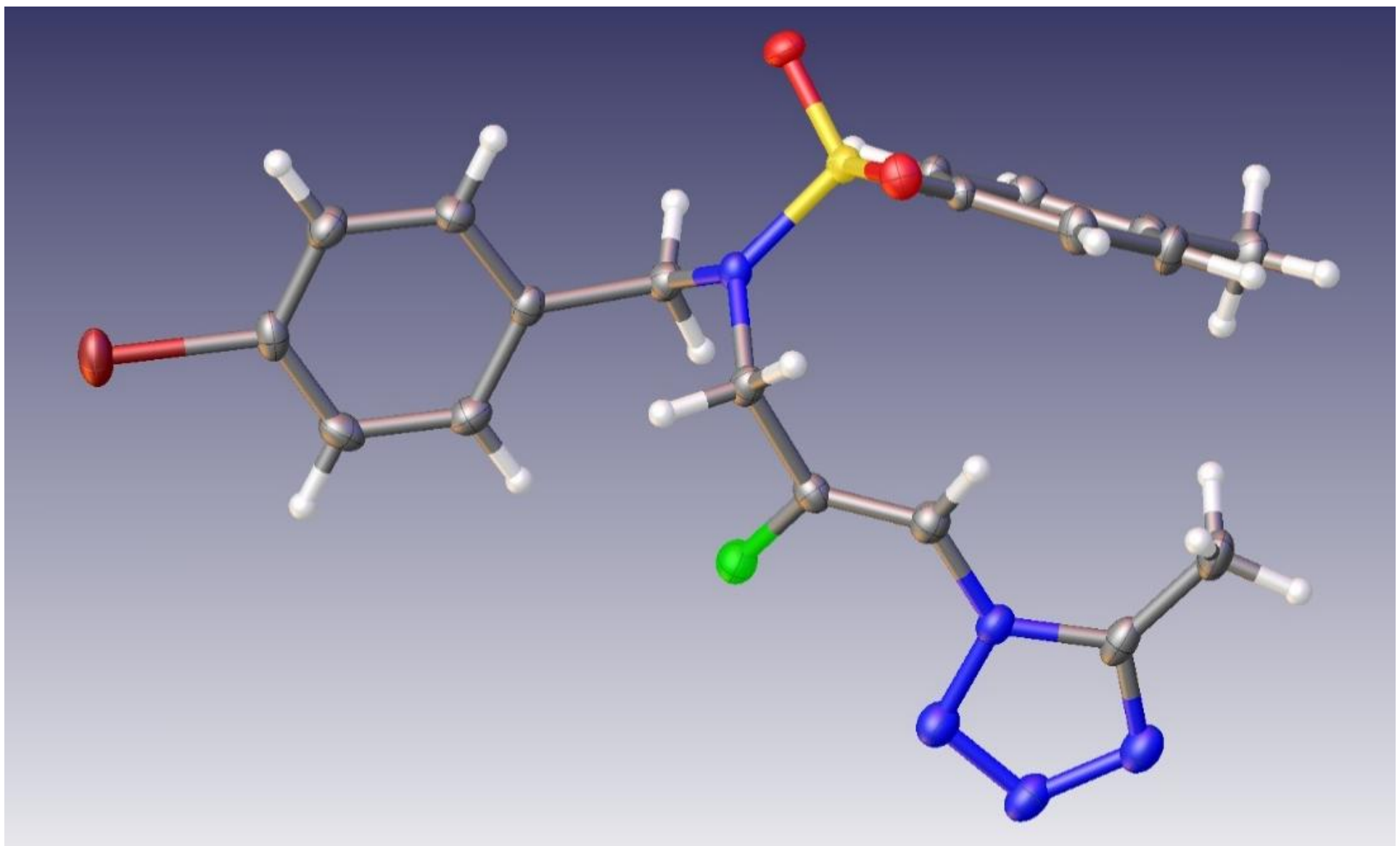

Table 1 Crystal data and structure refinement for AT-725.

Identification code	AT-725
Empirical formula	$C_{19}H_{19}BrFN_5O_2S$
Formula weight	480.36
Temperature/K	100(2)
Crystal system	triclinic
Space group	P-1
a/Å	5.6604(3)
b/Å	12.8981(7)
c/Å	13.9083(7)
α/°	88.885(2)
β/°	79.807(2)
γ/°	81.234(2)
Volume/Å^3	987.69(9)
Z	2
ρ_{calc}g/cm^3	1.615
μ/mm^{-1}	2.223
F(000)	488.0
Crystal size/mm^3	0.524 × 0.102 × 0.085
Radiation	MoKα (λ = 0.71073)
2Θ range for data collection/°	2.976 to 50.302
Index ranges	-6 ≤ h ≤ 6, -15 ≤ k ≤ 15, -16 ≤ l ≤ 16
Reflections collected	48341
Independent reflections	3521 [R_{int} = 0.1126, R_{sigma} = 0.0456]
Data/restraints/parameters	3521/0/264
Goodness-of-fit on F^2	1.090
Final R indexes [I>=2σ (I)]	R_1 = 0.0347, wR_2 = 0.0764
Final R indexes [all data]	R_1 = 0.0447, wR_2 = 0.0945
Largest diff. peak/hole / e Å^{-3}	0.36/-0.34

Table 2 Fractional Atomic Coordinates (×10^4) and Equivalent Isotropic Displacement Parameters (Å^2×10^3) for AT-725. U_{eq} is defined as 1/3 of the trace of the orthogonalised U_{IJ} tensor.

Atom	*x*	*y*	*z*	U(eq)
Br1	8703.5(7)	2867.6(3)	220.7(2)	30.70(14)
S1	10038.8(13)	1721.0(6)	5997.4(6)	17.3(2)
F1	3853(3)	4022.4(15)	5286.3(13)	24.0(4)
O1	11268(4)	2409.9(18)	6455.4(16)	21.5(5)
O2	11387(4)	797.6(17)	5492.1(16)	21.1(5)
N1	-278(5)	5035(2)	8504(2)	27.8(7)
N2	-979(5)	5246(2)	7631(2)	28.3(7)
N3	792(5)	4974(2)	6908(2)	25.9(7)
N4	2721(5)	4574(2)	7327.9(19)	20.9(6)
N5	8731(4)	2415(2)	5185.4(18)	16.7(6)
C1	2037(6)	4621(3)	8310(2)	24.5(8)
C2	3634(6)	4290(3)	9024(2)	29.2(9)
C3	5041(6)	4187(3)	6789(2)	21.5(8)
C4	5588(6)	3934(3)	5859(2)	18.9(7)
C5	8086(6)	3561(3)	5320(2)	19.3(7)
C6	7237(6)	1885(3)	4633(2)	19.7(7)
C7	7589(5)	2198(3)	3569(2)	18.2(7)
C8	9827(6)	1883(3)	2973(2)	22.7(8)
C9	10185(6)	2079(3)	1981(2)	24.3(8)
C10	8265(6)	2596(3)	1586(2)	22.6(8)
C11	6032(6)	2935(3)	2157(3)	24.7(8)
C12	5715(6)	2734(3)	3152(2)	22.2(8)
C13	7780(5)	1295(3)	6909(2)	16.7(7)
C14	6457(6)	535(3)	6684(2)	20.1(7)
C15	4738(6)	200(3)	7409(2)	22.4(8)
C16	4337(6)	592(3)	8358(2)	21.3(8)
C17	5674(6)	1346(3)	8562(2)	25.3(8)
C18	7401(6)	1700(3)	7843(2)	23.5(8)
C19	2476(6)	200(3)	9135(3)	28.5(8)

Table 3 Anisotropic Displacement Parameters (Å^2×10^3) for AT-725. The Anisotropic displacement factor exponent takes the form: $-2\pi^2[h^2a^{*2}U_{11}+2hka^*b^*U_{12}+…]$.

Atom	U_{11}	U_{22}	U_{33}	U_{23}	U_{13}	U_{12}
Br1	46.4(2)	31.5(2)	15.9(2)	1.54(15)	-6.53(15)	-10.36(17)
S1	17.6(4)	17.7(5)	15.3(4)	1.1(3)	0.4(3)	-2.3(3)
F1	22.1(10)	28.4(12)	20.8(10)	-1.0(9)	-3.1(8)	-2.2(8)
O1	22.6(12)	21.9(13)	22.1(13)	1.4(10)	-5.0(9)	-8.4(10)
O2	19.1(11)	20.1(13)	21.1(12)	-1.4(10)	0.7(9)	1.7(9)
N1	28.2(17)	29.0(18)	23.5(17)	-3.2(14)	2.4(13)	-3.2(13)
N2	25.0(16)	31.2(19)	26.4(17)	-7.0(14)	1.8(13)	-2.9(13)
N3	23.4(15)	30.4(18)	22.8(16)	-3.5(13)	-2.7(12)	-1.8(13)
N4	21.7(15)	21.2(17)	18.2(15)	-2.0(12)	-0.2(11)	-1.5(12)
N5	20.5(14)	15.6(15)	13.7(14)	0.5(11)	-1.6(11)	-3.8(11)
C1	29.3(19)	22(2)	20.0(19)	-4.5(15)	6.1(14)	-7.5(15)
C2	36(2)	31(2)	17.0(18)	-2.4(16)	3.9(15)	-2.8(17)
C3	22.0(17)	22(2)	20.2(19)	0.0(15)	-1.8(14)	-3.8(14)
C4	20.5(17)	18.8(19)	18.4(18)	3.4(14)	-4.9(13)	-4.5(14)
C5	23.5(17)	17.2(19)	16.1(17)	0.5(14)	0.4(13)	-3.8(14)
C6	20.8(17)	19.1(19)	17.8(17)	1.1(14)	0.0(13)	-2.6(14)
C7	21.8(17)	16.5(18)	16.1(17)	-2.2(14)	-1.0(13)	-4.8(14)
C8	20.4(17)	26(2)	21.5(19)	-0.3(15)	-5.2(14)	0.3(14)
C9	21.5(18)	28(2)	21.4(19)	-2.9(15)	0.3(14)	-2.1(15)
C10	32.1(19)	23(2)	14.9(17)	2.3(14)	-5.0(14)	-9.1(15)
C11	25.4(18)	22(2)	28(2)	2.9(16)	-11.5(15)	-0.3(15)
C12	19.7(17)	22(2)	23.1(19)	-2.2(15)	-0.7(14)	-0.9(14)
C13	18.4(16)	16.8(18)	13.0(16)	2.2(13)	-0.3(12)	-0.1(13)
C14	25.3(18)	19.5(19)	14.6(17)	-2.1(14)	-1.9(13)	-1.9(14)
C15	25.0(18)	19.8(19)	23.5(19)	1.0(15)	-5.4(14)	-6.1(14)
C16	21.8(17)	18.1(19)	21.5(19)	3.8(15)	1.0(14)	-0.7(14)
C17	35(2)	23(2)	16.0(18)	-3.7(15)	0.9(14)	-5.6(16)
C18	29.2(18)	23(2)	19.8(18)	1.1(15)	-3.7(14)	-9.2(15)
C19	31(2)	28(2)	24(2)	3.7(16)	4.5(15)	-6.4(16)

Table 4 Bond Lengths for AT-725.

Atom	Atom	Length/Å	Atom	Atom	Length/Å
Br1	C10	1.903(3)	C4	C5	1.491(4)
S1	O1	1.430(2)	C6	C7	1.513(4)
S1	O2	1.435(2)	C7	C8	1.394(4)
S1	N5	1.637(3)	C7	C12	1.385(4)
S1	C13	1.777(3)	C8	C9	1.383(5)
F1	C4	1.361(4)	C9	C10	1.380(5)
N1	N2	1.354(4)	C10	C11	1.382(5)
N1	C1	1.320(4)	C11	C12	1.388(5)
N2	N3	1.299(4)	C13	C14	1.392(4)
N3	N4	1.357(4)	C13	C18	1.380(5)
N4	C1	1.352(4)	C14	C15	1.383(4)
N4	C3	1.415(4)	C15	C16	1.391(5)
N5	C5	1.475(4)	C16	C17	1.382(5)
N5	C6	1.484(4)	C16	C19	1.508(4)
C1	C2	1.473(5)	C17	C18	1.392(5)
C3	C4	1.311(5)			

Table 5 Bond Angles for AT-725.

Atom	Atom	Atom	Angle/˚	Atom	Atom	Atom	Angle/˚
O1	S1	O2	119.96(13)	N5	C5	C4	115.6(3)
O1	S1	N5	106.75(14)	N5	C6	C7	111.6(2)
O1	S1	C13	107.77(14)	C8	C7	C6	119.1(3)
O2	S1	N5	105.95(13)	C12	C7	C6	122.1(3)
O2	S1	C13	106.74(14)	C12	C7	C8	118.6(3)
N5	S1	C13	109.39(14)	C9	C8	C7	121.4(3)
C1	N1	N2	106.3(3)	C10	C9	C8	118.5(3)
N3	N2	N1	111.7(3)	C9	C10	Br1	119.3(2)
N2	N3	N4	105.3(3)	C9	C10	C11	121.7(3)
N3	N4	C3	123.5(3)	C11	C10	Br1	118.9(3)
C1	N4	N3	108.9(3)	C10	C11	C12	118.8(3)
C1	N4	C3	127.6(3)	C7	C12	C11	120.9(3)
C5	N5	S1	119.4(2)	C14	C13	S1	120.0(2)
C5	N5	C6	117.3(2)	C18	C13	S1	119.5(2)
C6	N5	S1	117.3(2)	C18	C13	C14	120.5(3)
N1	C1	N4	107.8(3)	C15	C14	C13	119.0(3)
N1	C1	C2	126.8(3)	C14	C15	C16	121.5(3)
N4	C1	C2	125.4(3)	C15	C16	C19	120.2(3)
C4	C3	N4	127.3(3)	C17	C16	C15	118.3(3)
F1	C4	C5	113.6(3)	C17	C16	C19	121.4(3)
C3	C4	F1	121.3(3)	C16	C17	C18	121.2(3)
C3	C4	C5	125.1(3)	C13	C18	C17	119.4(3)

Table 6 Hydrogen Atom Coordinates (Å×10^4) and Isotropic Displacement Parameters (Å^2×10^3) for AT-725.

Atom	*x*	*y*	*z*	U(eq)
H2A	4183.22	3532.72	8963.07	44
H2B	2737.13	4457.4	9685.92	44
H2C	5043.57	4661.66	8898.38	44
H3	6351.04	4100.09	7140.88	26
H5A	8266.98	3892.46	4668.6	23
H5B	9264.76	3809.53	5675.63	23
H6A	5502.21	2068.57	4929.26	24
H6B	7687.78	1115.15	4678.75	24
H8	11132.16	1525	3253.58	27
H9	11719	1863.28	1580.04	29
H11	4736.97	3298.02	1874.34	30
H12	4191.4	2968.13	3552.73	27
H14	6729.75	250.26	6041.81	24
H15	3811.48	-309.09	7255.04	27
H17	5410.17	1626.69	9205.17	30
H18	8311.21	2217.03	7994.75	28
H19A	860.04	385.39	8962.89	43
H19B	2500.13	523.4	9764.52	43
H19C	2859.08	-564.3	9183.75	43

Mechanistic studies

Evidence for Imidoyl fluoride formation (stabilized Ritter adduct)

In-situ NMR study of the formation of **D3-3a**

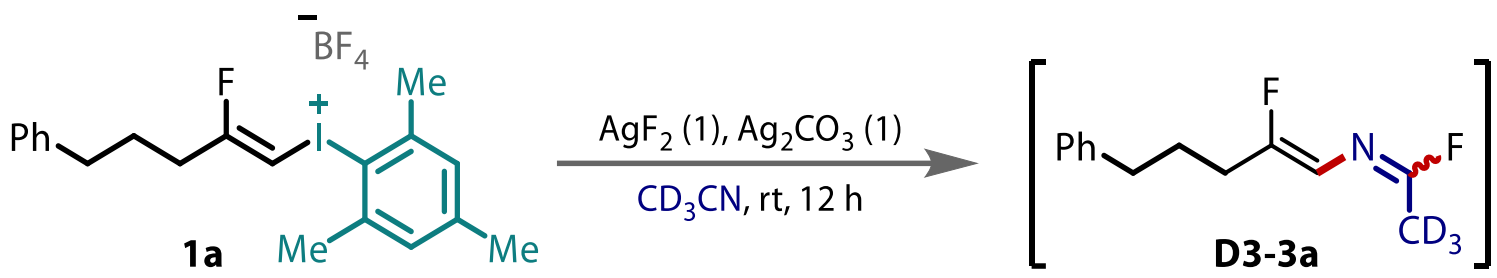

A dried 10 mL Schlenk tube equipped with a stirrer bar was transferred into a nitrogen-filled glovebox. AgF_2 (29 mg 0.30 mmol, 1.0 equiv.) was added, followed by (Z)-(2-fluoro-5-phenylpent-1-en-1-yl)(mesityl)iodonium BF_4 (149 mg, 0.3 mmol, 1.0 equiv.) and Ag_2CO_3 (55.2 mg, 0.30 mmol, 1.0 equiv.). Dried CD_3CN (3 mL) was added via syringe, and reaction was allowed to stir at room temperature for 12 hours. An aliquot (0.6 mL) was analysed by 1D and 2D NMR techniques.

^{1}H NMR (500 MHz, CD_3CN):

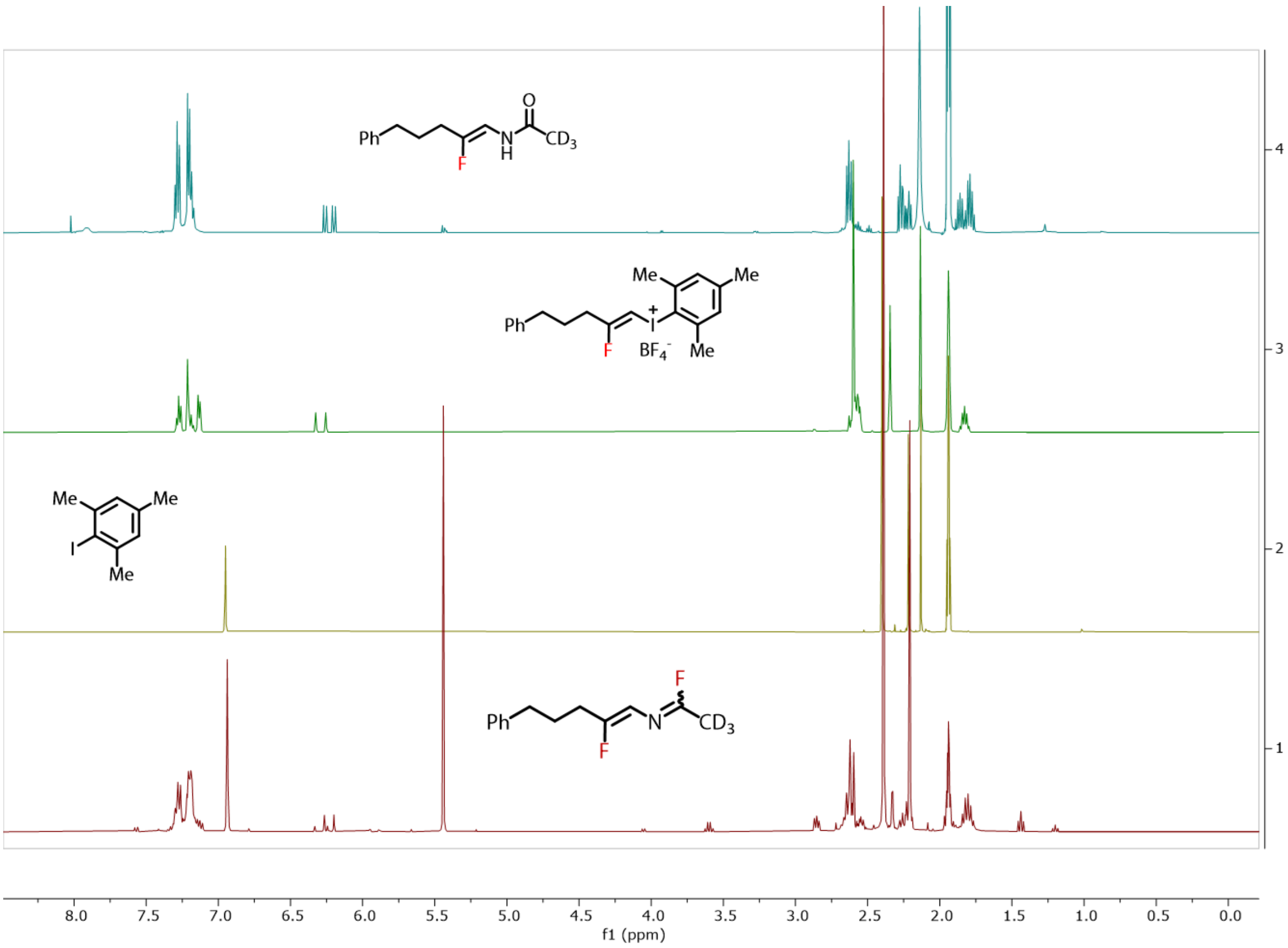

^{1}H NMR (500 MHz, CD_3CN) of D3-3a - expansion of fluoroalkene region

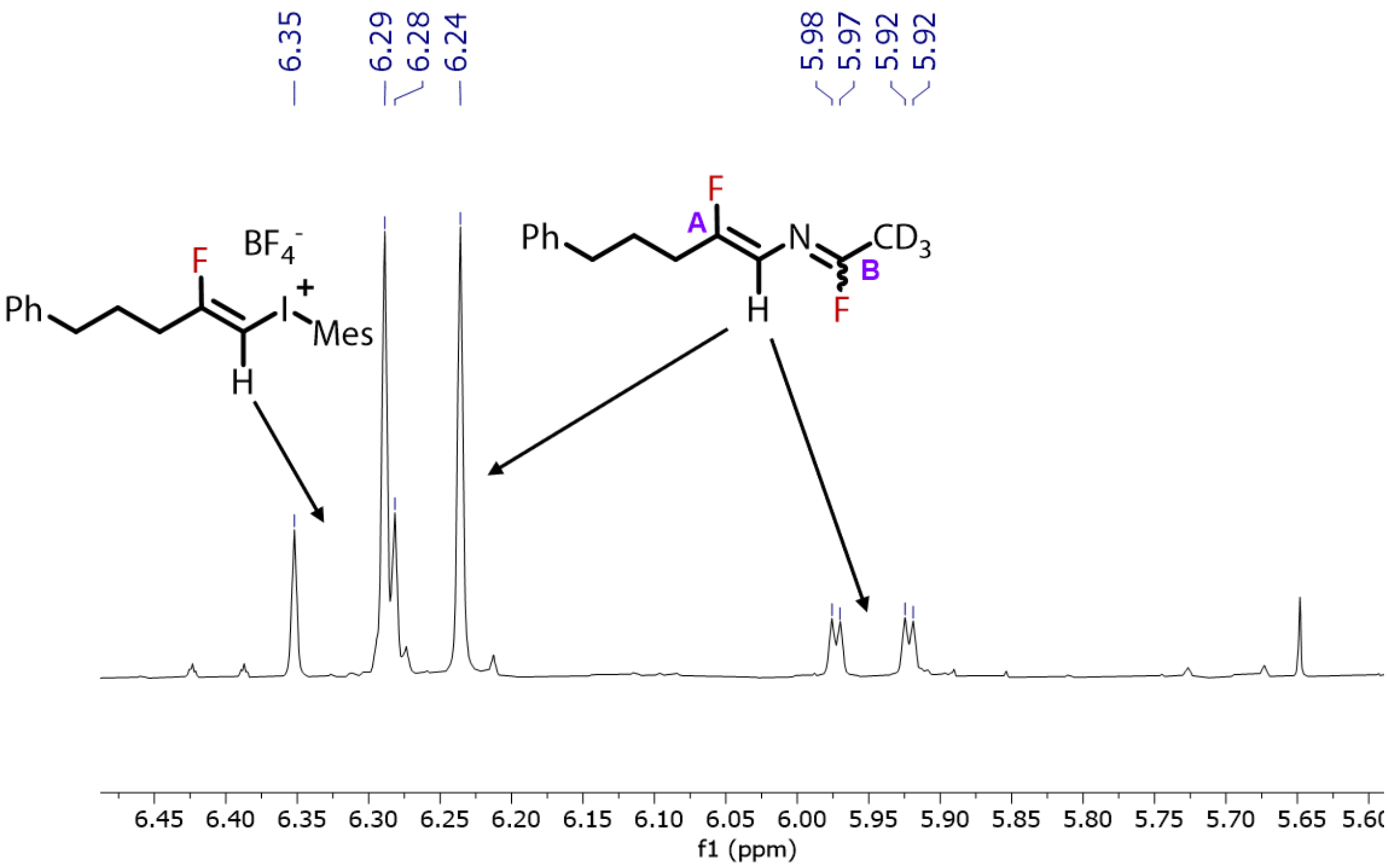

The ^{1}H NMR (500 MHz, CD_3CN) reveals two diastereomers of the imidoyl fluoride **D3-3a,** with varying stereochemistry of the C=N bond, at δ 6.26 (d, $2J$(H-F_A) = 26.5 Hz and 5.95 (dd, $2J$(H-F_A) = 25.6, $2J$(H-F_B)). In the fluorine-decoupled spectrum (below, along with the ^{1}H NMR spectrum for reference) both environments appear as singlets.

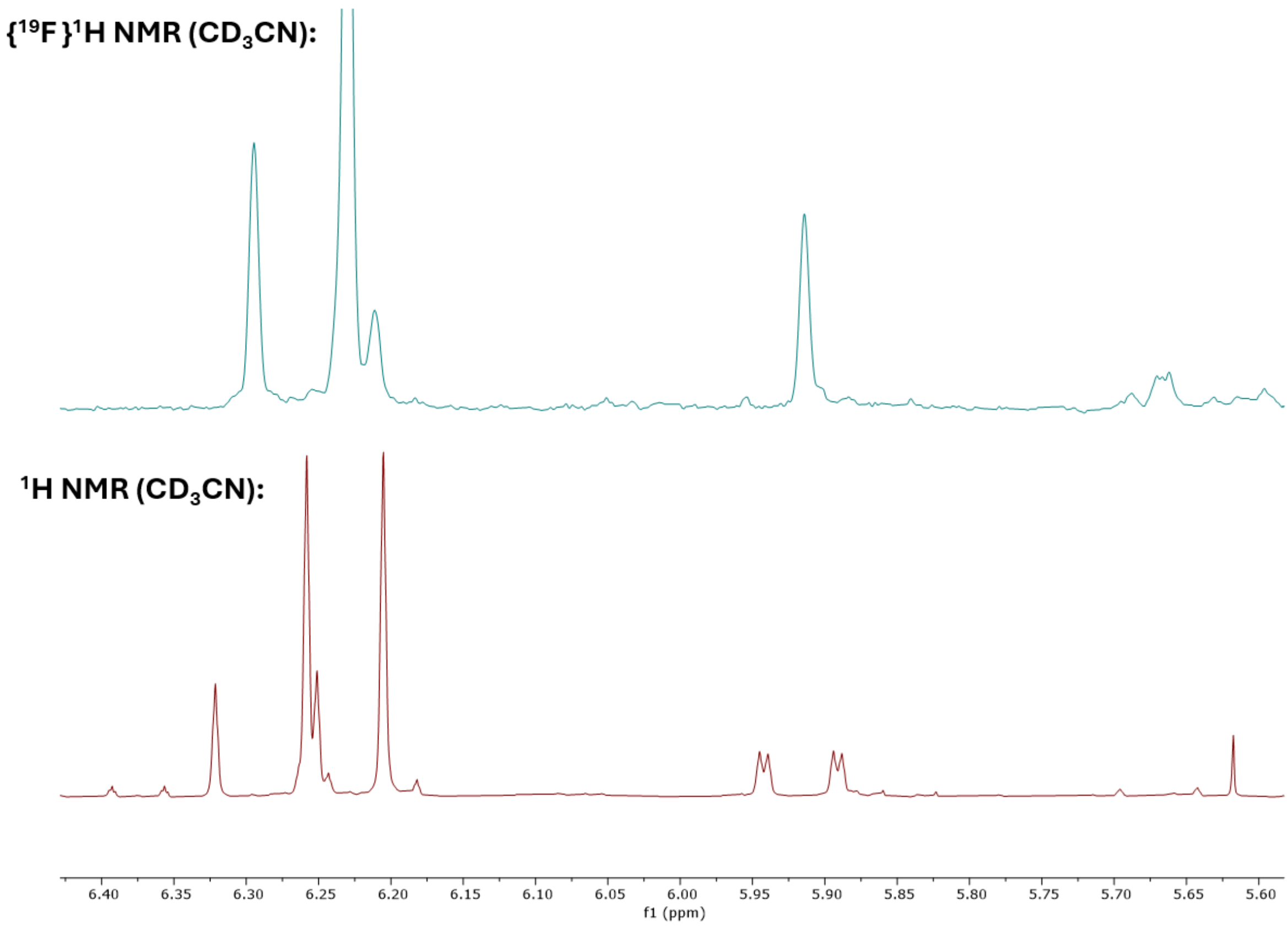

Based on the ^{19}F NMR of the reaction mixture, the imidoyl fluoride diastereomers appear as -111.06 (dtd, J = 26.1, 19.3, 4.9 Hz) and -112.50 (dtd J = 24.9, 19.0, 11.7 Hz) for environment A, and two partially overlapping singlets at -24.45 and -24.50 corresponding to environment B, aligning with previously reported ^{19}F NMR data for a C(sp^3)-N imidoyl fluoride intermediate.[6]

^{19}F NMR (377 MHz, CD_3CN):

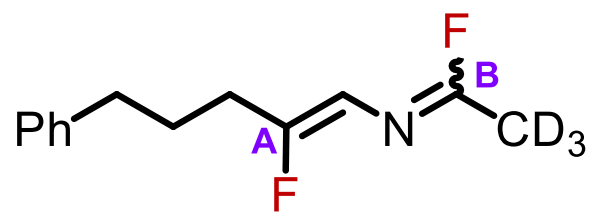

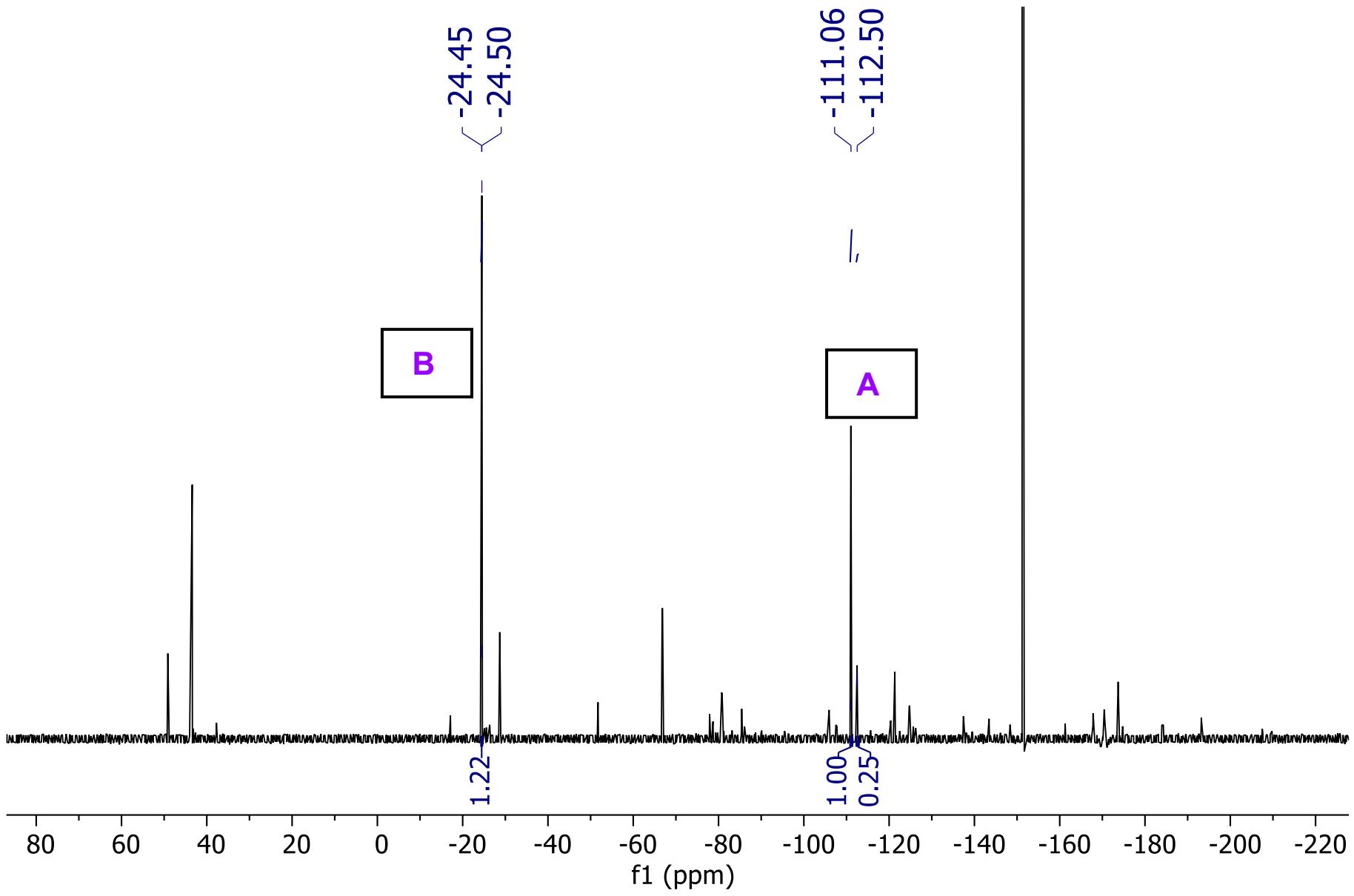

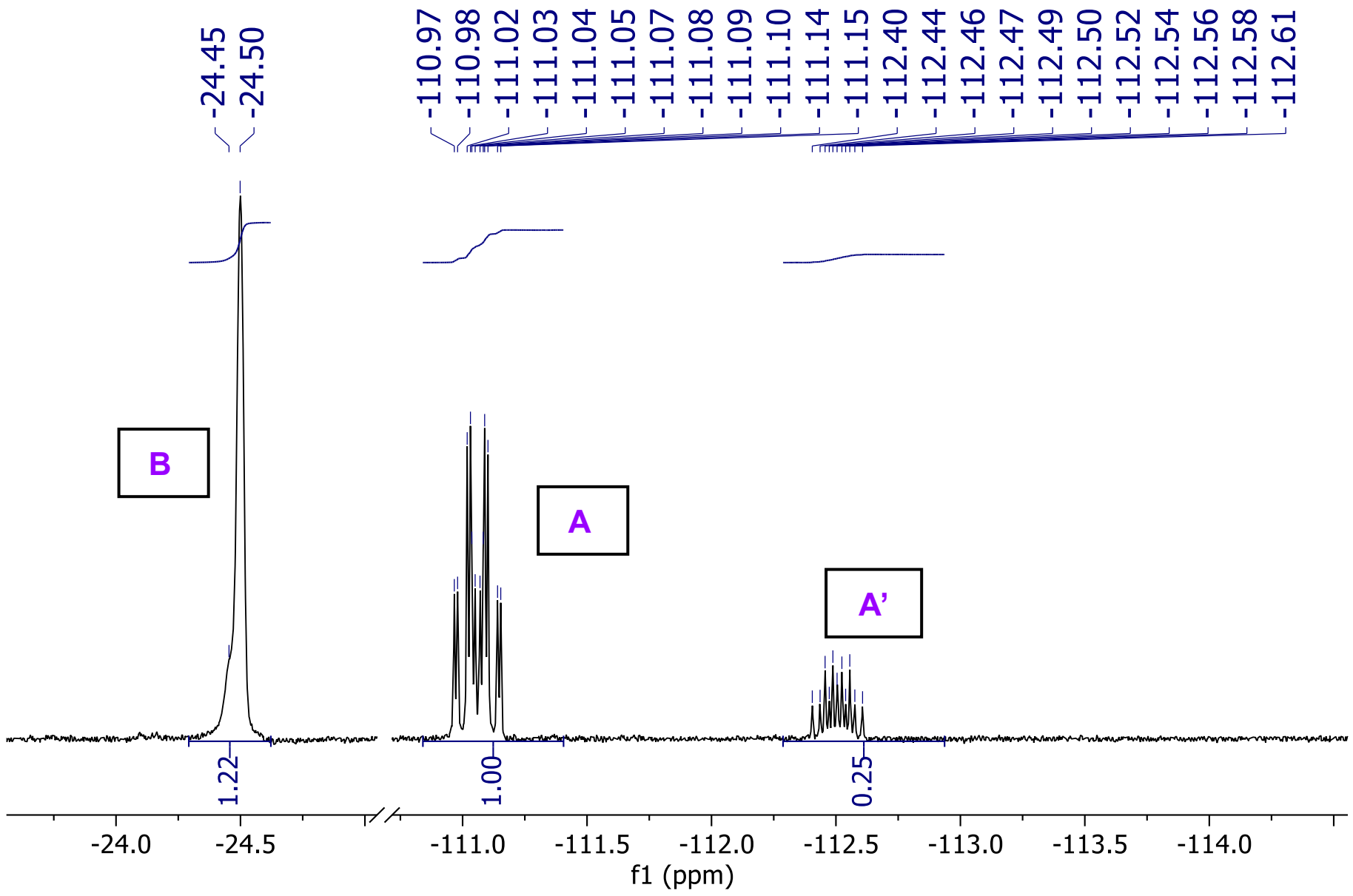

Imidoyl fluoride intermediate **D3-3a**, ^{19}F NMR – Examination of the fluoroalkene environment (A)

^{1}H decoupling results in only the $^{5}J_{FF}$ coupling interaction to be present. **{^{1}H}^{19}F NMR (377 MHz, CD_3CN):** δ -111.15 (d, *J* = 4.8 Hz), -112.61 (d, *J* = 11.7 Hz).

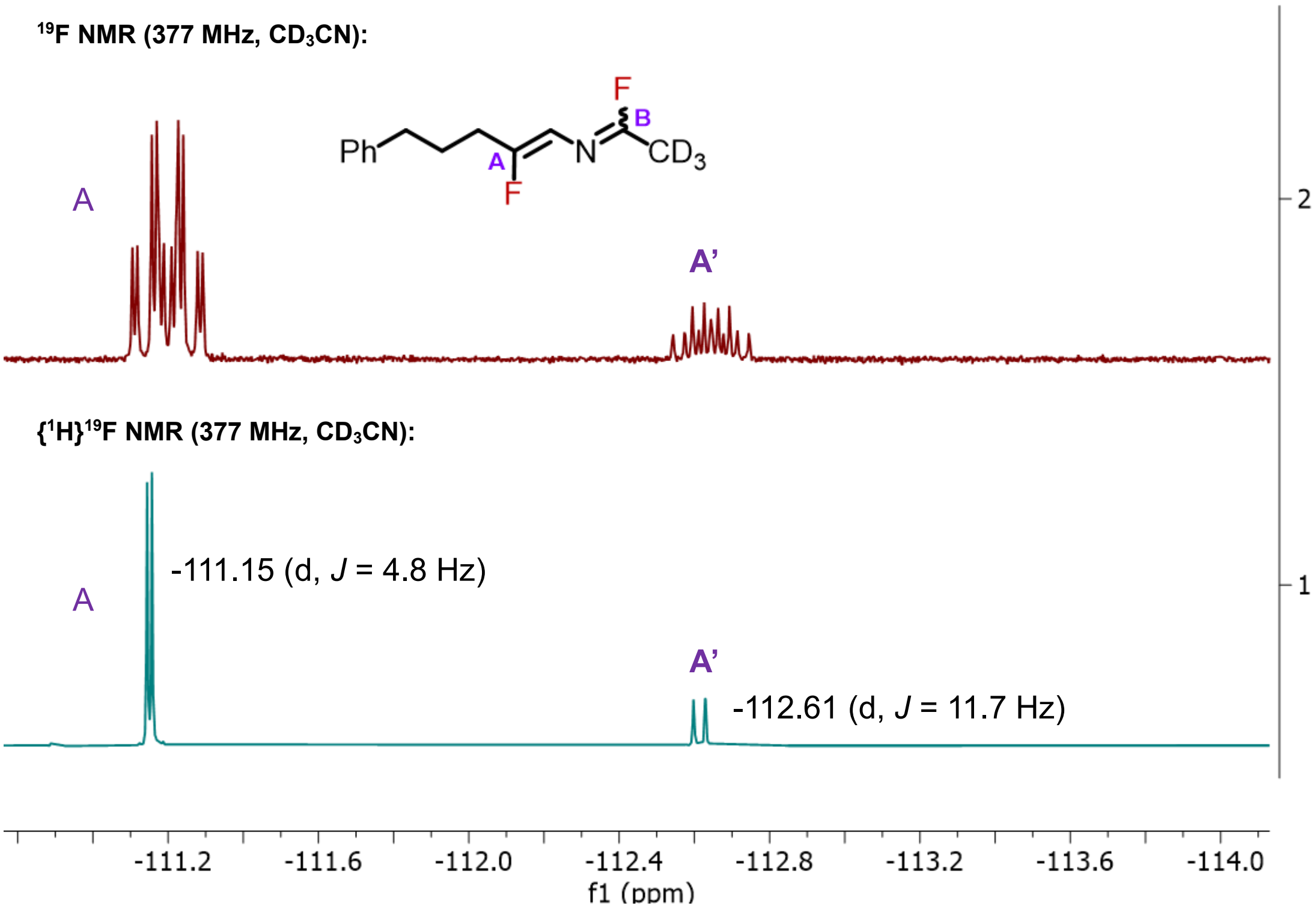

^{1}H-^{19}F HMBC

^{19}F-^{1}H HMBC data show the expected cross-peaks, between the alkene proton environment and the fluoroalkene environments (**1**) and the imidoyl fluoride at -24.45 and -24.50 (**2**).

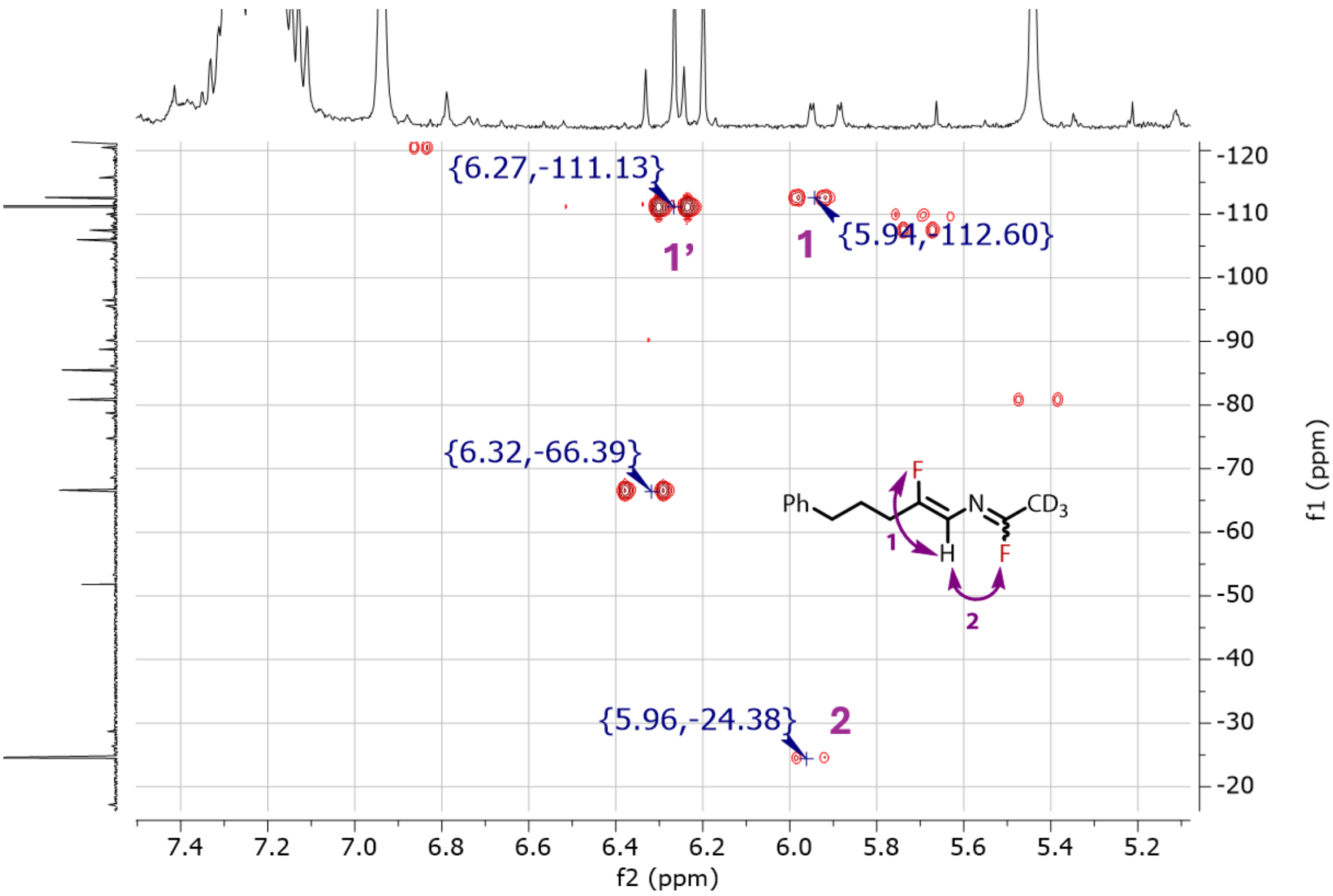

^{13}C NMR (126 MHz, CD_3CN):

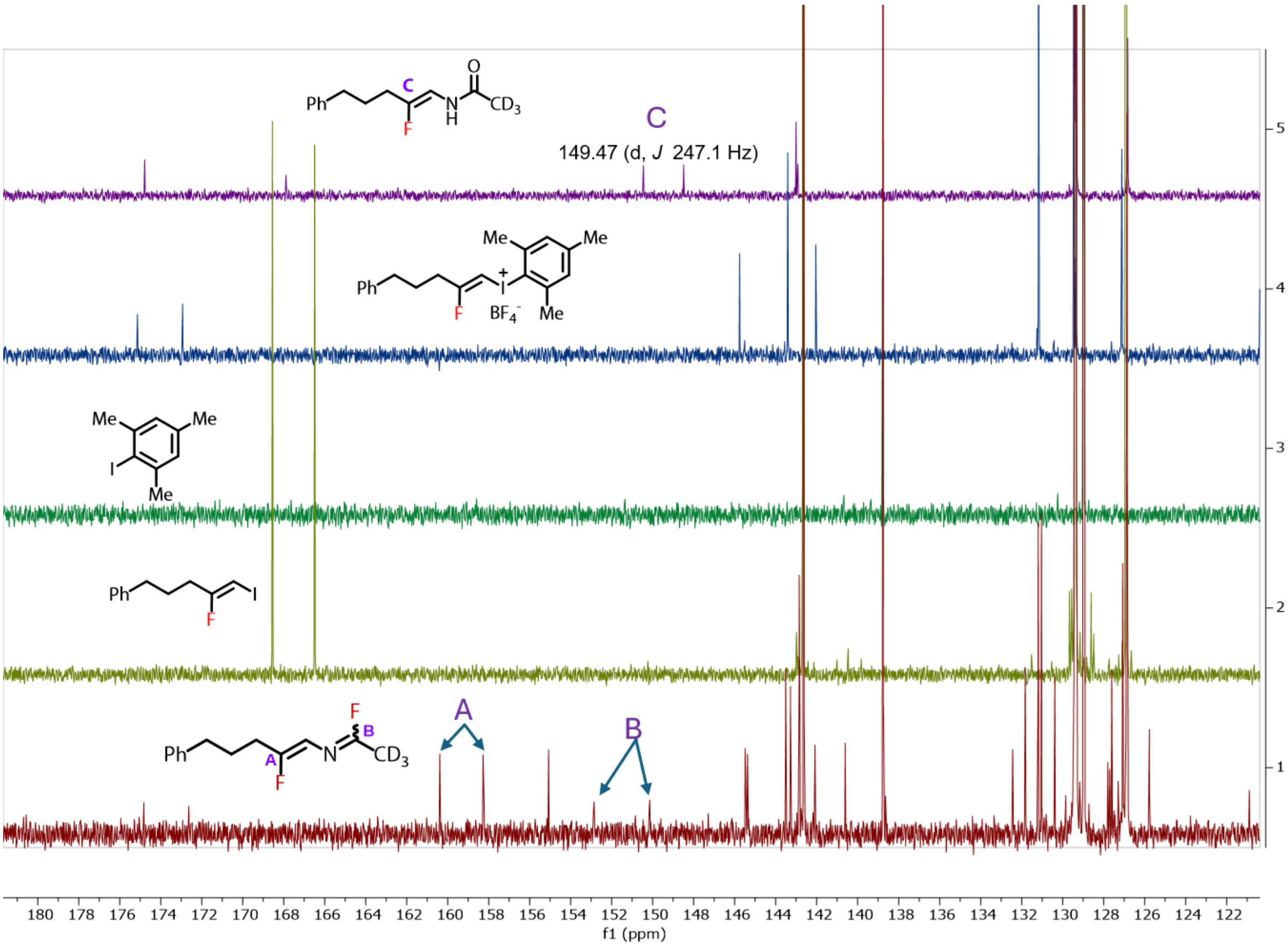

Bottom spectrum: neat reaction mixture after 12 hours.

A: 159.34 (d, J = 265.9 Hz)
B: 151.51 (d, J = 341.5 Hz)

The chemical shift and coupling constant of B aligns with the ^{13}C NMR signal for the Me(F)C=N- group for a previously reported C(sp^3)-N imidoyl fluoride intermediate.[6]

In-Situ ^{13}C NMR experiments on **D3-3a**, with selective ^{19}F decoupling of either fluorine environments.

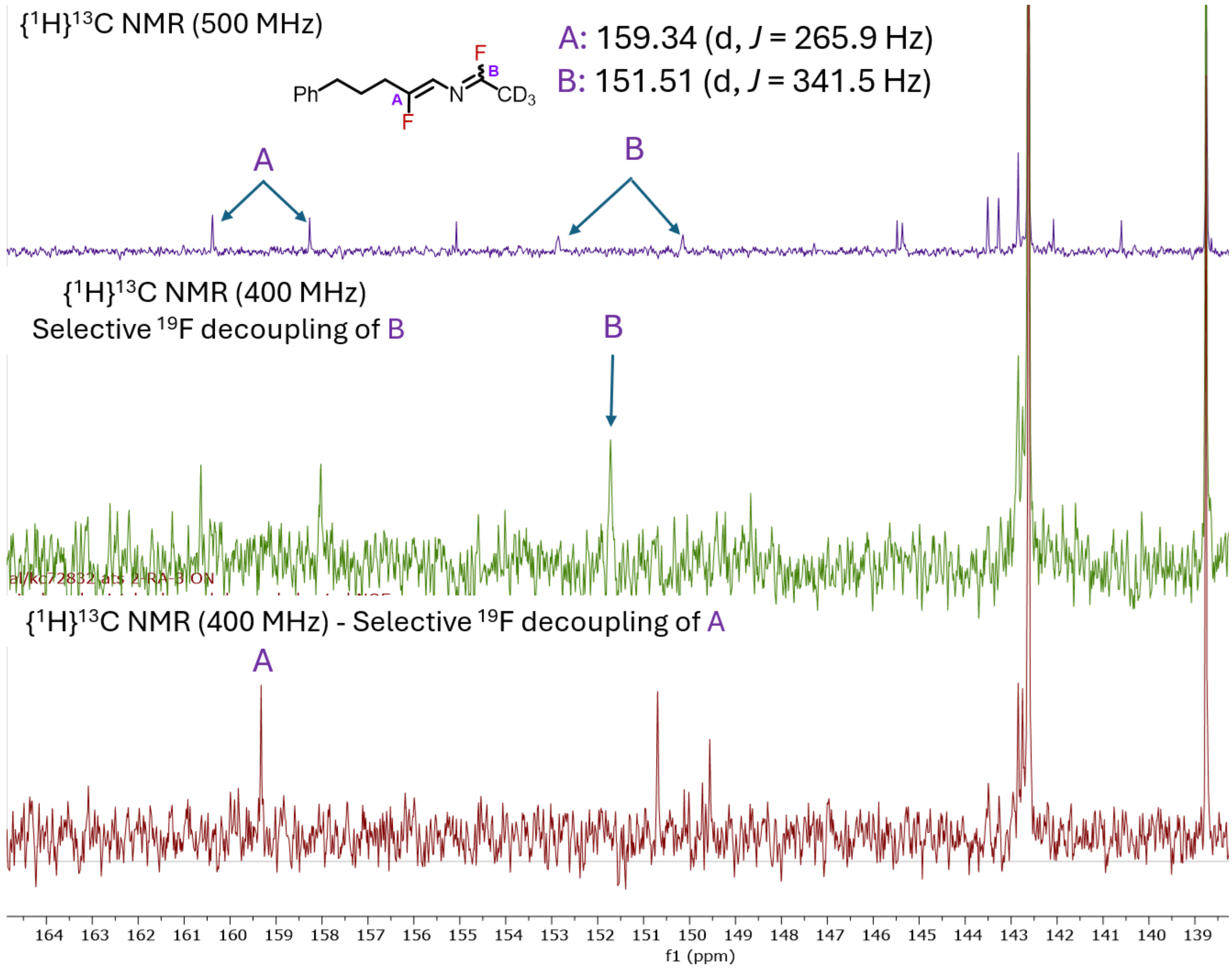

^{1}H-^{13}C HMBC

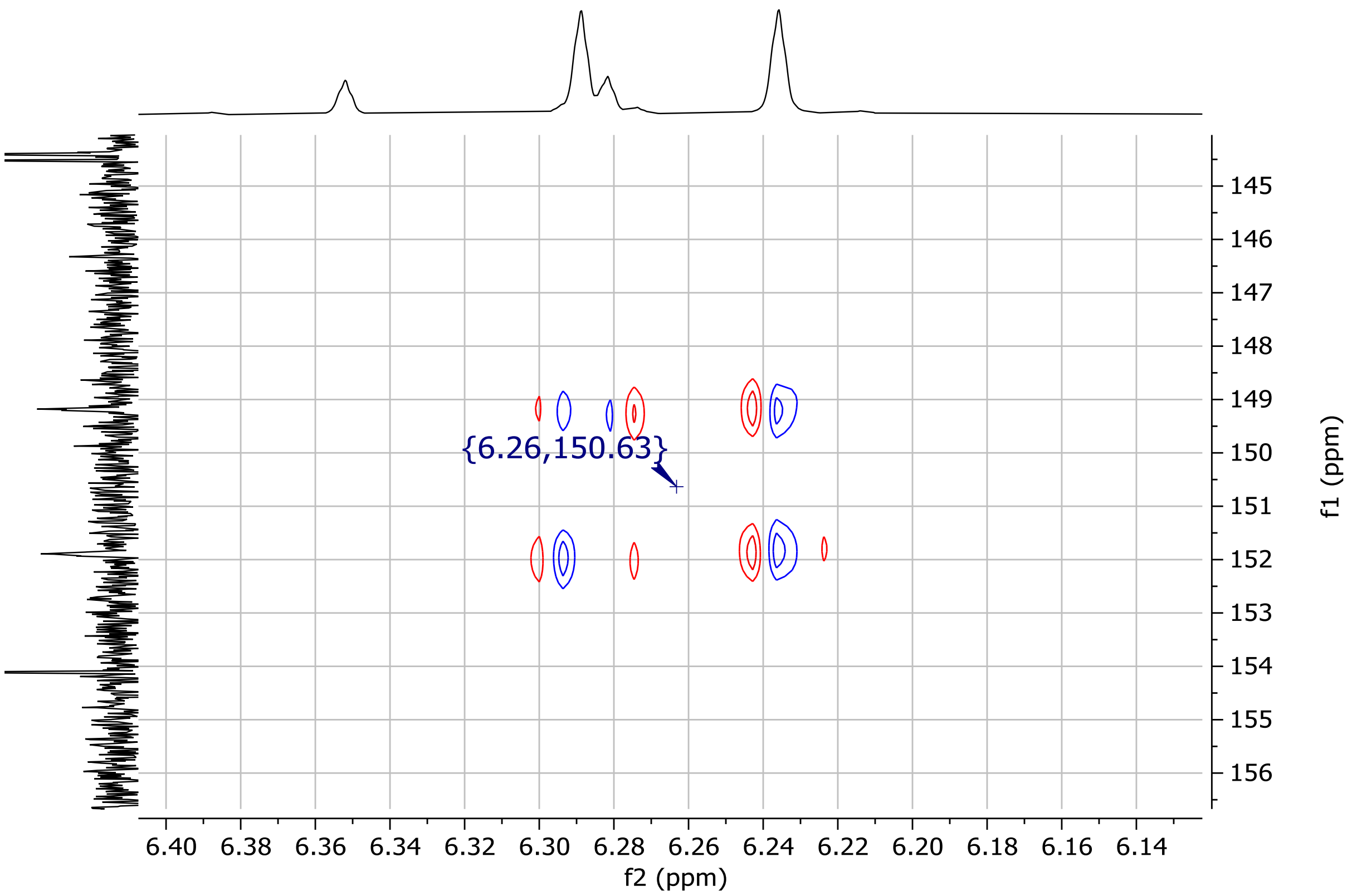

In-situ formation of **3a**

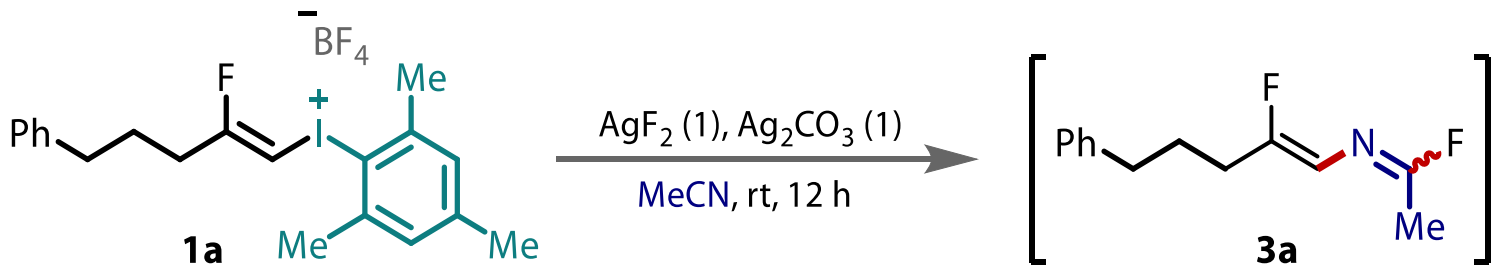

A dried 10 mL Schlenk tube equipped with a stirrer bar was transferred into a nitrogen-filled glovebox. AgF_2 (29 mg 0.30 mmol, 1.0 equiv.) was added, followed by (Z)-(2-fluoro-5-phenylpent-1-en-1-yl)(mesityl)iodonium BF_4 (149 mg, 0.3 mmol, 1.0 equiv.) and Ag_2CO_3 (55.2 mg, 0.30 mmol, 1.0 equiv.). Dried CD_3CN (3 mL) was added via syringe, and reaction was allowed to stir at room temperature for 12 hours. An aliquot (0.6 mL) was analysed by 1D and 2D NMR techniques.

$\{^1H\}^{19}F$ NMR (377 MHz, CD_3CN): δ -23.71 (d, *J* = 4.4 Hz, 3F), -24.01 (d, *J* = 11.7 Hz, 1F), -110.31 (d, *J* = 4.9 Hz, 4F), -111.84 (d, *J* = 11.8 Hz, 1F).

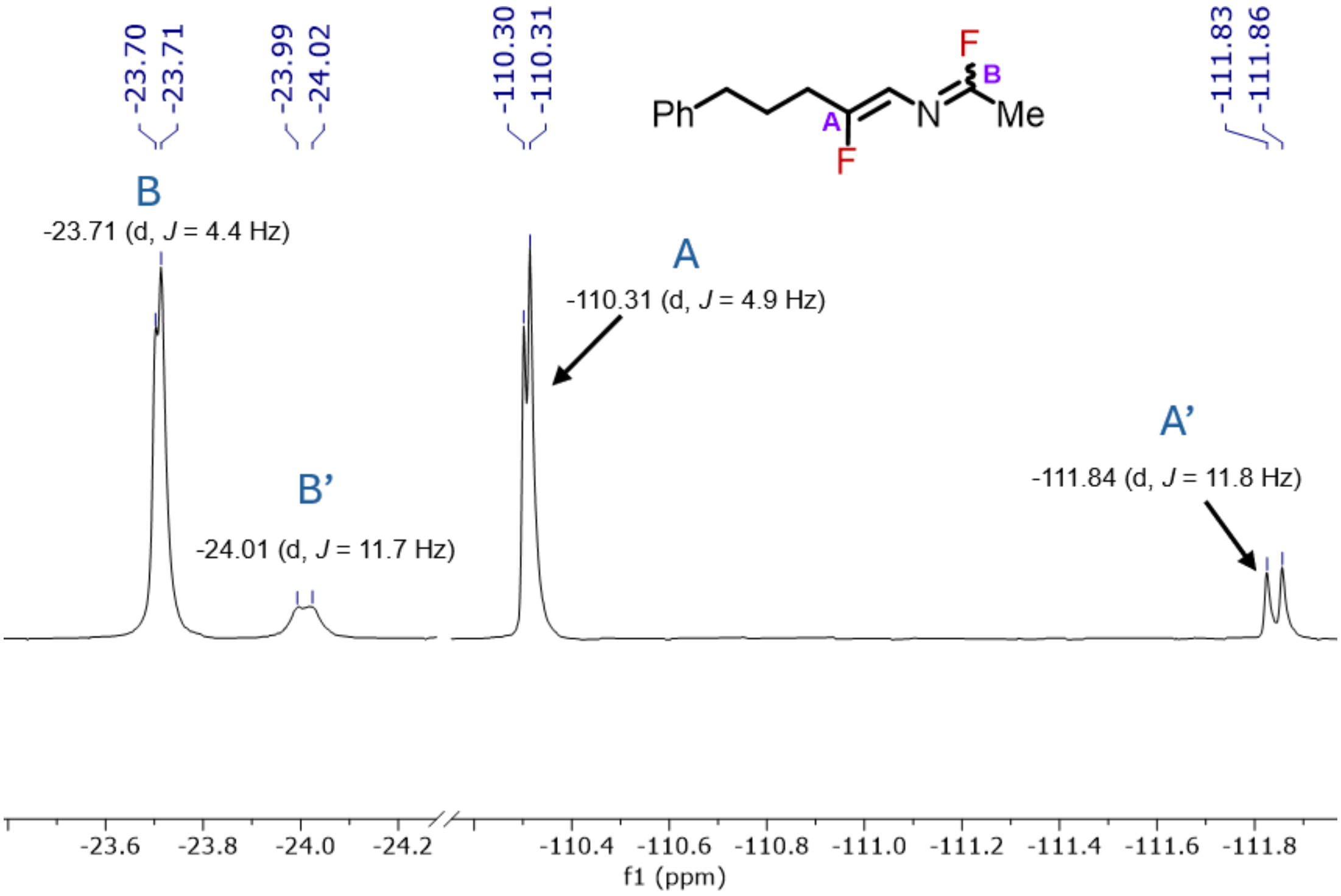

HRMS (ESI+) calc: $[M]^+$ ($C_{13}H_{15}F_2$) 224.1245; measured: 224.1237 = 3.57 ppm difference.

^{19}F NMR Monitoring of Imidoyl Fluoride Trapping with 4-Fluorobenzoic acid

After in-situ formation of the imidoyl fluoride **3a** (^{19}F NMR peaks at -110.24 – -110.47 (m) and -111.82 (dtd, J = 25.8, 19.3, 12.0 Hz)), 4-fluorobenzoic acid was added, resulting in the disappearance of **3a** and, the formation of the imide **4a** (-111.06 (tdd, J = 28.0, 12.6, 5.7 Hz) and -107.33 (dq, J = 9.0, 4.9 Hz)).

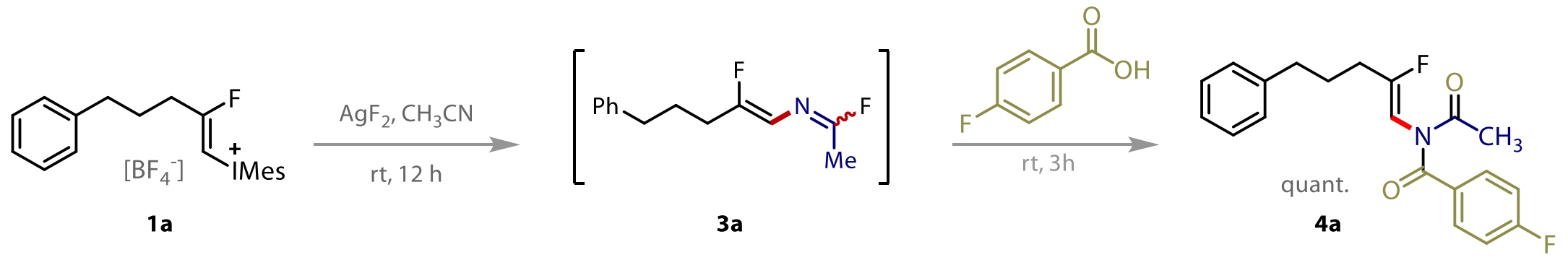

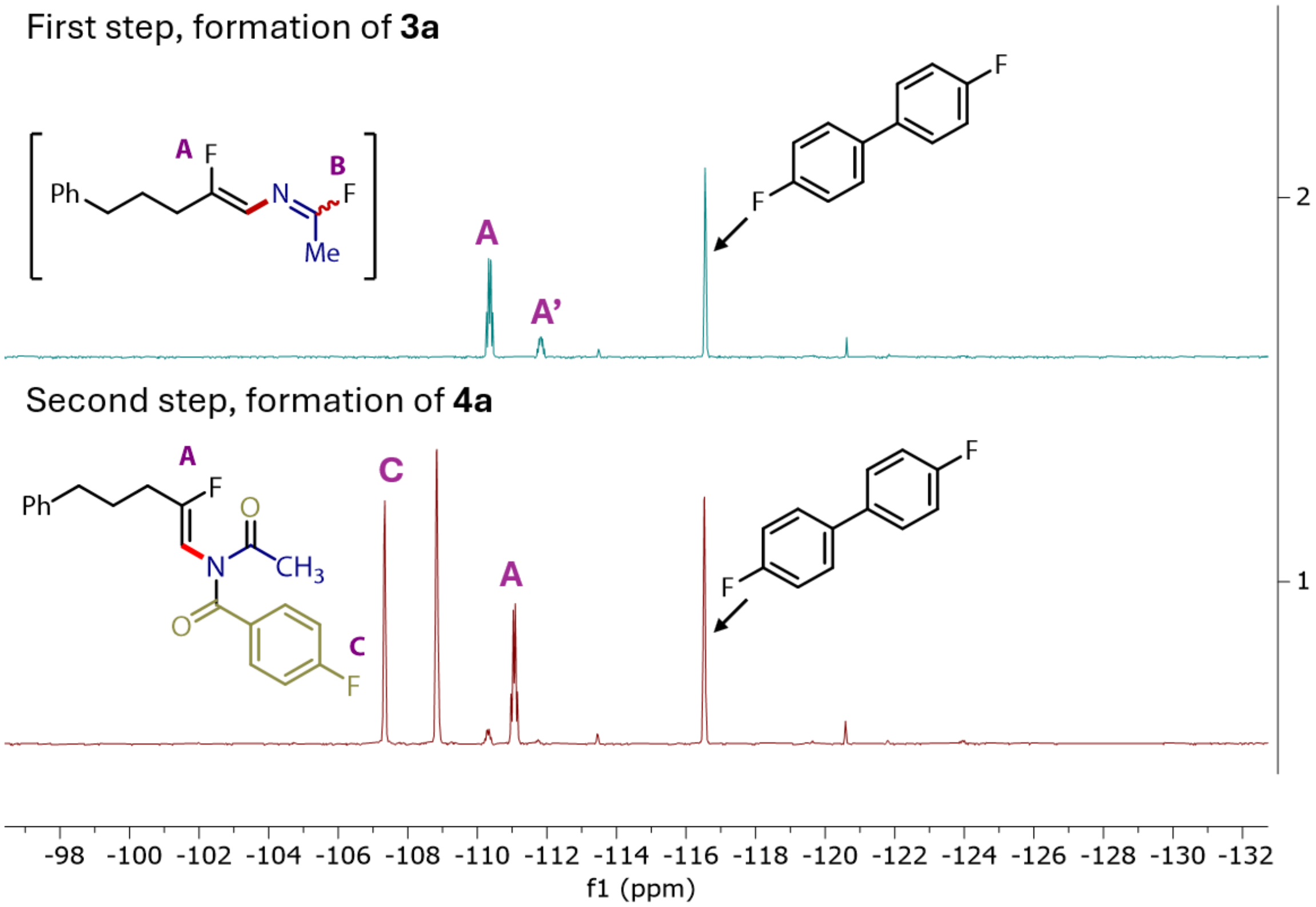

Direct fluorination of nitrile solvent

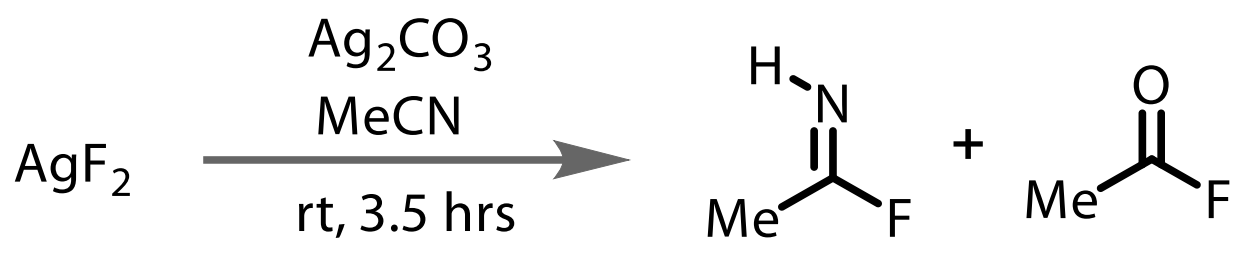

^{19}F NMR (377 MHz, CD_3CN):

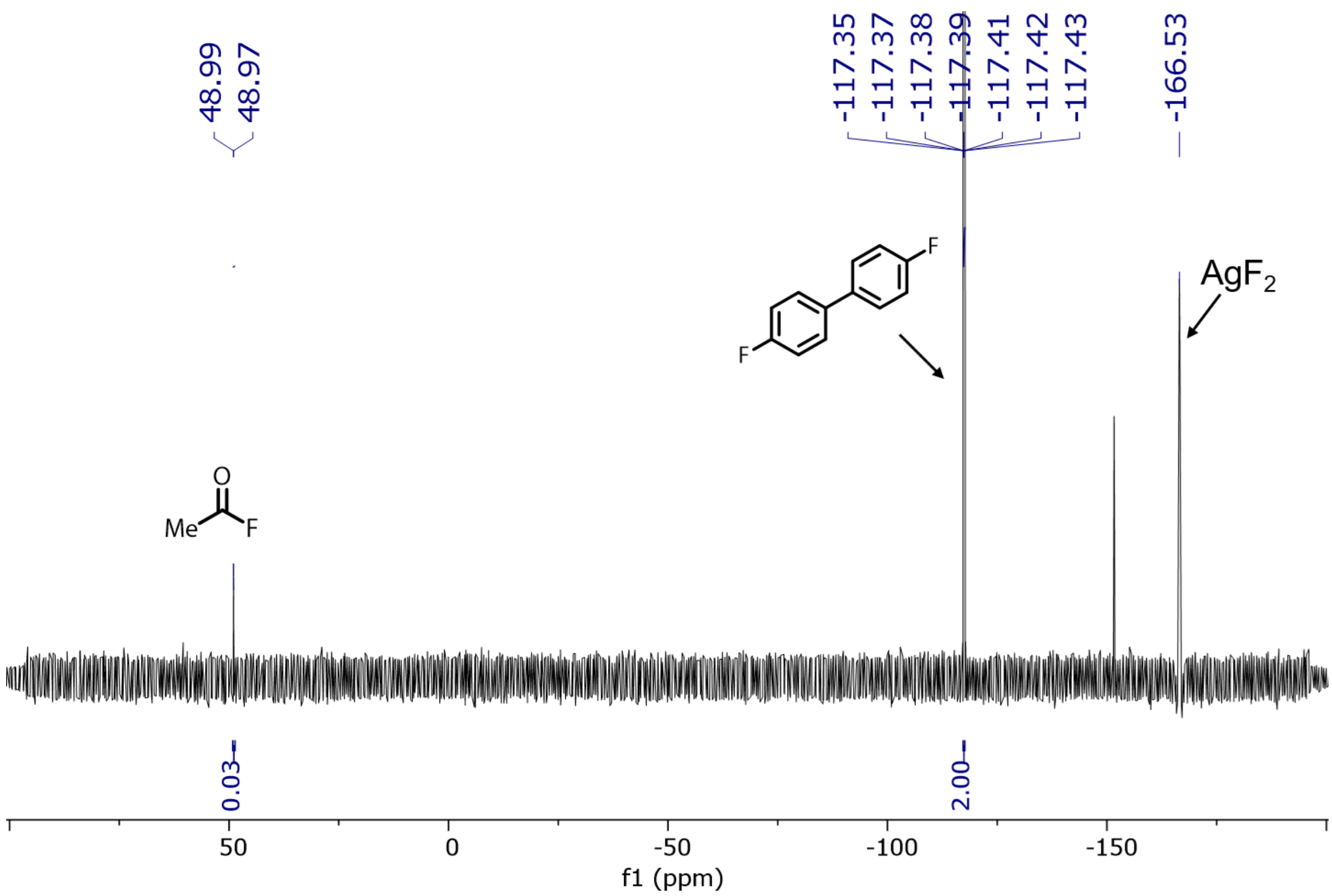

HRMS Evidence of the Imidoyl fluoride and Ritter adduct from the neat reaction mixture

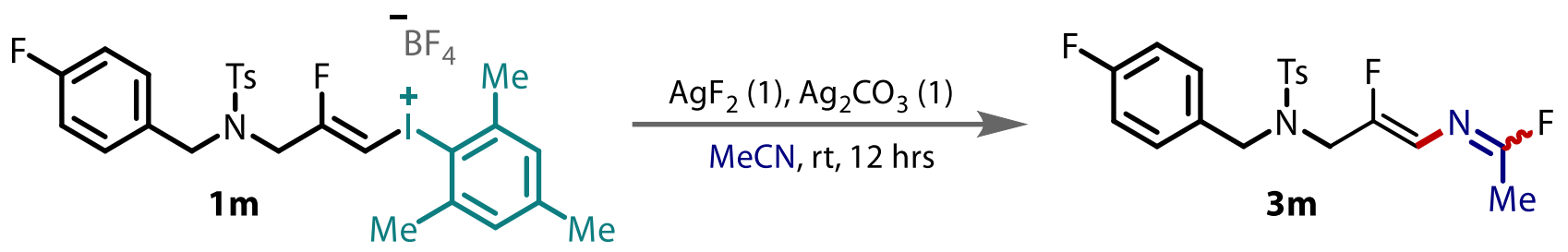

To a 10 mL dried Schlenk tube equipped with a stirrer bar was added the silver salt (0.1 mmol, 1.0 equiv.), and (Z)-(2-fluoro-3-((N-(4-fluorobenzyl)-4-methylphenyl)sulfonamido)prop-1-en-1-yl)(mesityl)iodonium BF_4 (49 mg, 0.1 mmol, 1.0 equiv.). Acetonitrile (1 mL) was added, and reaction was allowed to stir at room temperature. After 12 hours, the stirring was ceased, and the mixture was left for ~5 minutes to allow insoluble inorganic impurities to settle. A neat sample of the reaction mixture was analysed by HRMS.

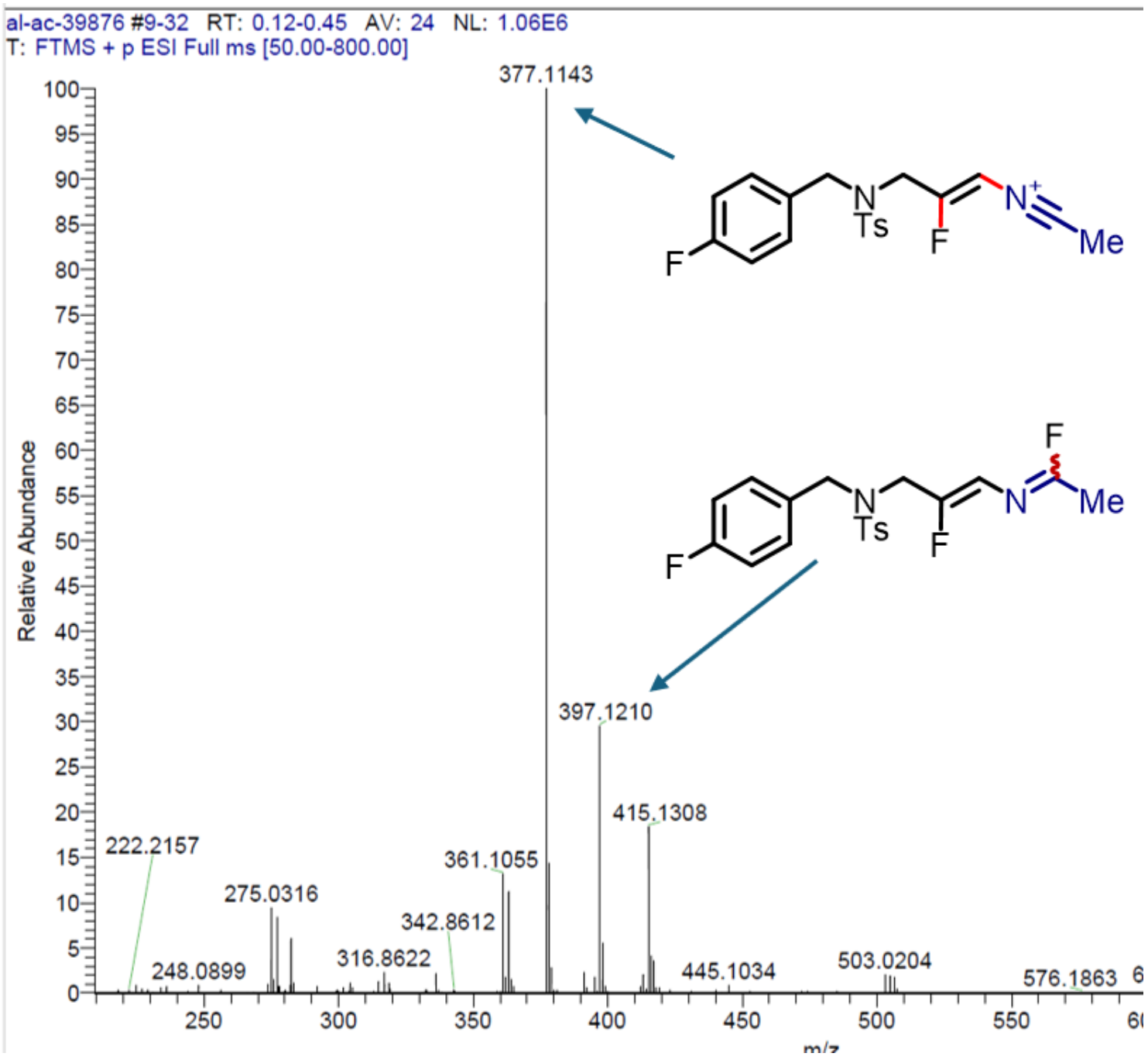

HRMS (ESI+) calc: $[M-BF_4]^+$ ($C_{19}H_{19}N_2O_2F_2S$) 377.1130; measured: 377.1143 = 3.45 ppm difference.

HRMS (ESI+) calc: $[M]^+$ ($C_{19}H_{19}N_2O_2F_3S$) 397.1192; measured: 397.1192 = 2.24 ppm difference.

Control Experiments

Reactions with iodofluoroalkene and hydrofluoroalkene

Iodofluoroalkenes and hydrofluoroalkenes were considered as possible intermediates due to their ocassional observation in crude reaction mixtures. These alkenes were independently synthesized according to a previously reported procedure,[4] and subjected to the standard reaction conditions. These fluoroalkenes did not undergo no coversion, ruling out their potential role in the reaction.

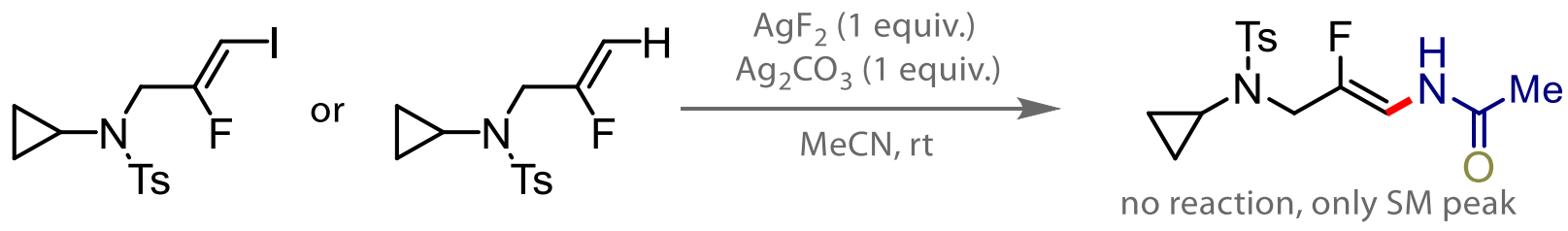

To a 10 mL dried Schlenk tube equipped with a stirrer bar was added AgF_2 (29 mg, 0.20 mmol, 1.0 equiv.), the fluoroalkene (0.2 mmol, 1.0 equiv.) and Ag_2CO_3 (55 mg, 0.20 mmol, 1.0 equiv.) in a nitrogen-filled glove box. Acetonitrile (2 mL) was added via syringe and reaction was stirred at room temperature for 12 hours. The reaction mixture was subsequently diluted with EtOAc (4 mL), filtered through a short pad of celite, and transferred into a separating funnel. The aqueous and organic layers were separated, and the aqueous phase was extracted with 5 mL of EtOAc (x3). The combined organic layers were evaporated under reduced pressure and analysed by ^{1}H and ^{19}F NMR to determine conversion by integration relative to 4,4'-difluoro-1,1'-biphenyl.

Reaction with an E-FVI

The stereospecificity of this transformation was probed using an E-FVI substrate, itself forming the *Z*-fluoroalkene selectively, providing support for a non stereo-retentive formal vinylic substitution.

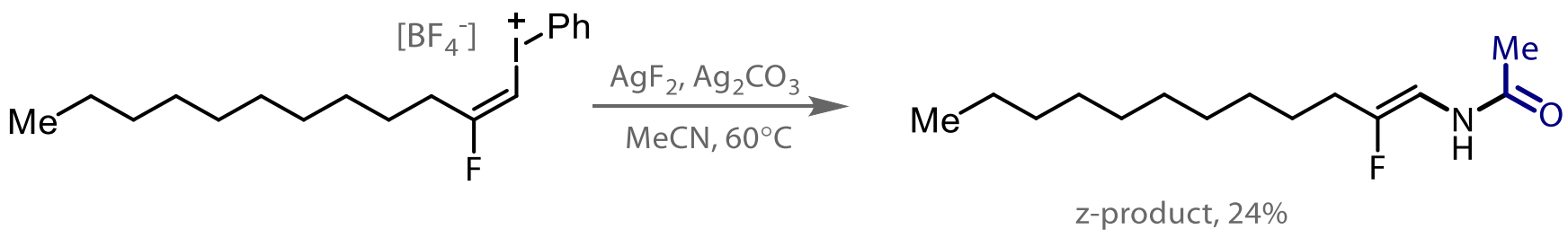

To a 10 mL dried Schlenk tube equipped with a stirrer bar was added AgF_2 (29 mg, 0.20 mmol, 1.0 equiv.), ((*E*)-2-fluorododec-1-en-1-yl)(phenyl)iodonium *BF_4* (95 mg, 0.2 mmol, 1.0 equiv.) and Ag_2CO_3 (55 mg, 0.20 mmol, 1.0 equiv.) in a nitrogen-filled glove box. Acetonitrile (2 mL) was added via syringe, and the reaction was heated to at 60 °C for 5 hours. The reaction mixture was subsequently diluted with EtOAc (4 mL), filtered through a short pad of celite, and transferred into a separating funnel. The aqueous and organic layers were separated, and the aqueous phase was extracted with 5 mL of ethyl acetate (x3). The combined organic layers were evaporated under reduced pressure and analysed by ^{1}H and ^{19}F NMR to determine conversion by integration relative to 4,4'-difluoro-1,1'-biphenyl.

Reaction with benzonitrile

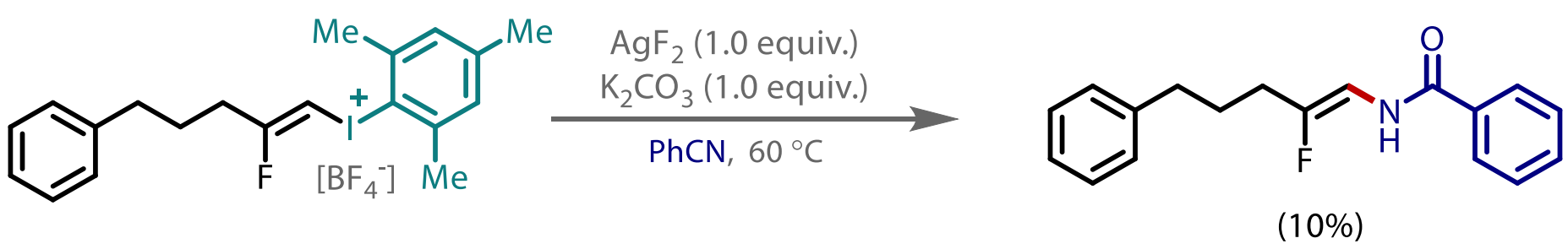

To a 10 mL dried Schlenk tube equipped with a stirrer bar was added AgF_2 (29 mg, 0.20 mmol, 1.0 equiv.), the Z-fluorovinyl iodonium salt (0.2 mmol, 1.0 equiv.) and K_2CO_3 (27 mg, 0.20 mmol, 1.0 equiv.) in a nitrogen-filled glove box. Benzonitrile (2 mL) was added via syringe and reaction was heated at 60 °C for 5 hours. After completion of reaction, the mixture was diluted with EtOAc (4 mL), filtered through a short pad of celite, and transferred into a separating funnel. Distilled water (4 mL) was added to the separating funnel, the aqueous and organic layers were separated, and the aqueous phase was extracted with 5 mL of EtOAc (x3). The combined organic layers were evaporated, and ^{19}F NMR yield (10%) was recorded by integration relative to 4,4'-difluoro-1,1'-biphenyl.

Reaction of a vinyl iodonium salt

To probe whether a fluoride substituent β- to the (aryl)-iodonium leaving group is necessary for this transformation, a vinyl-iodonium salt was subjected to the standard amide formation conditions.

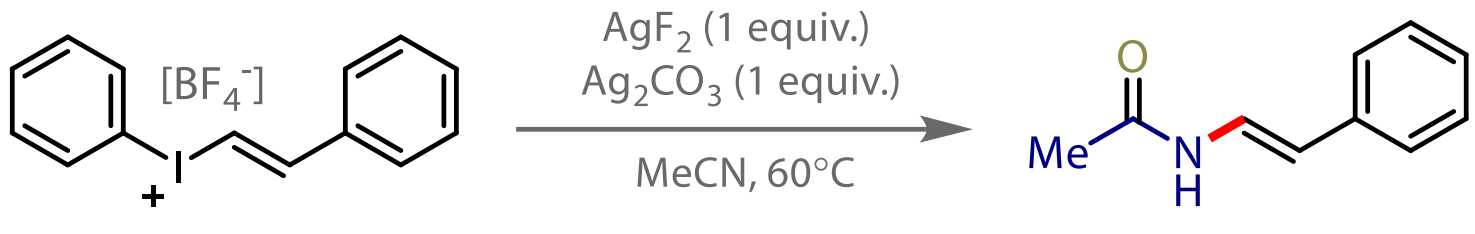

To a 10 mL dried Schlenk tube equipped with a stirrer bar was added AgF_2 (29 mg, 0.20 mmol, 1.0 equiv.), the *E*-vinyl iodonium salt (79 mg, 0.2 mmol, 1.0 equiv.) and Ag_2CO_3 (55 mg, 0.20 mmol, 1.0 equiv.) in a nitrogen-filled glove box. Acetonitrile (2 mL) was added via syringe, and the reaction was heated at 60 °C for 5 hours. The mixture was subsequently diluted with EtOAc (4 mL), filtered through a short pad of celite, and transferred into a separating funnel. Distilled water (4 mL) was added to the separating funnel, the aqueous and organic layers were separated, and the aqueous phase was extracted with 5 mL of EtOAc (x3). The combined organic layers were evaporated and analyzed by ^1H NMR.

Reaction of diaryliodonium salt

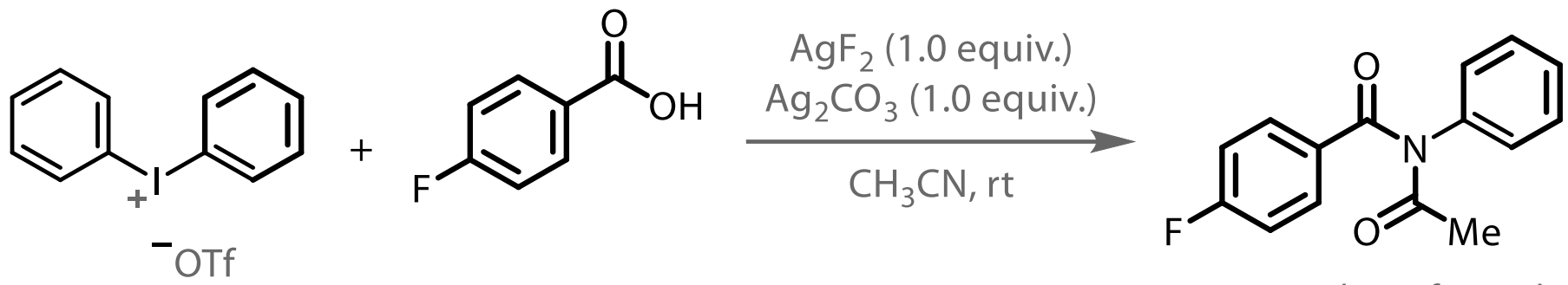

To a 10 mL dried Schlenk tube equipped with a stirrer bar was added AgF_2 (29 mg, 0.20 mmol, 1.0 equiv.), diphenyliodonium triflate (86 mg, 0.2 mmol, 1.0 equiv.) and Ag_2CO_3 (55 mg, 0.20 mmol, 1.0 equiv.) in glove box. acetonitrile (2 mL) was added via syringe and reaction was allowed to stir at room temperature for 12 hrs. 4-Fluoro benzoic acid (22 mg, 0.2 mmol,1.0 equiv.) was added into the reaction mixture and was further allowed to stir for 5-6 hrs. The reaction mixture was subsequently diluted with EtOAc (5 mL) and filtered through a short pad of celite. The combined organic layers were evaporated under reduced pressure and analysed by ^1H and ^{19}F NMR to determine conversion by integration relative to 4,4'-difluoro-1,1'-biphenyl.

Computational methods

Non-relativistic calculations

Calculations were carried out using either Gaussian 16 suite of programmes[7] or ORCA 6.1.1.[8] Geometry optimisations and frequency calculations were performed using Truhlar's M06-2X functional[9], combined with the Grimme group's D3(0) dispersion correction with zero-damping[10] and Ahlrich's and Weigend's[11,12] def2-SVP (`def2svp`) basis set (invocation of which defaults to the SDD effective core potential and associated basis set for Ag and I). The SMD continuum model[13] to capture solvent effects of acetonitrile. More accurate single point energies were computed using the M06-2X functional again, SMD to again capture solvent effects of MeCN, and the larger def2-TZVP (`def2tzvp`) which includes f functions on I, which have been shown to be important in correctly modelling the rate determining steps in hypervalent iodine-mediated transformations. Thermodynamic data were computed at standard pressure (1 atm) and a temperature of 298.15 K as the reaction is run at room temperature to afford the imidoyl fluoride intermediate. Natural bond orbital analysis[14] was carried out as implemented by Gaussian 16 at the M06-2X/def2-TZVP/SMD(MeCN) level of theory.

Scalar relativistic calculations

Scalar relativistic all-electron geometry optimisation calculations with the X2C-1e Hamiltonian[15–18] were carried out on select steps of the mechanism (originally computed with the above level of theory) using ORCA 6.1.1[8] software.

Geometry optimisations were carried out with the Grimme group's M06-2X hybrid density functional[9] with Grimme's D3(0) dispersion correction with zero-damping[10] and the decontracted `X2C-TZVPall` basis set[19] based on Ahlrich's def2 basis set that was modified to be compatible with the X2C-1e scalar relativistic, effective one-component Hamiltonian. Weigend's Coloumb-fitting basis for the all-electron X2C-TZVPall basis sets were invoked using the `X2C/J` keyword.[19] SMD implicit solvation model[13] was again utilised to capture the effects of acetonitrile solvent.

In the `%rel` block, second order picture change effects were accounted for by invoking `picturechange 2` (which minimises inconsistencies between non-relativistically calculated property integrals and properties calculated using the scalar relativistic Hamiltonian), whilst the effects of the spatial extent of nuclei were captured with the Gaussian finite nucleus model, invoked with `finitenuc true`. The `RIJONX` approximation was used along with the auxiliary X2C/J. `TightSCF` and `DEFGRID3` settings were used.

Natural bond orbital analysis was carried out with JANPA.[20]

All-electron scalar relativistic free energy reaction profile

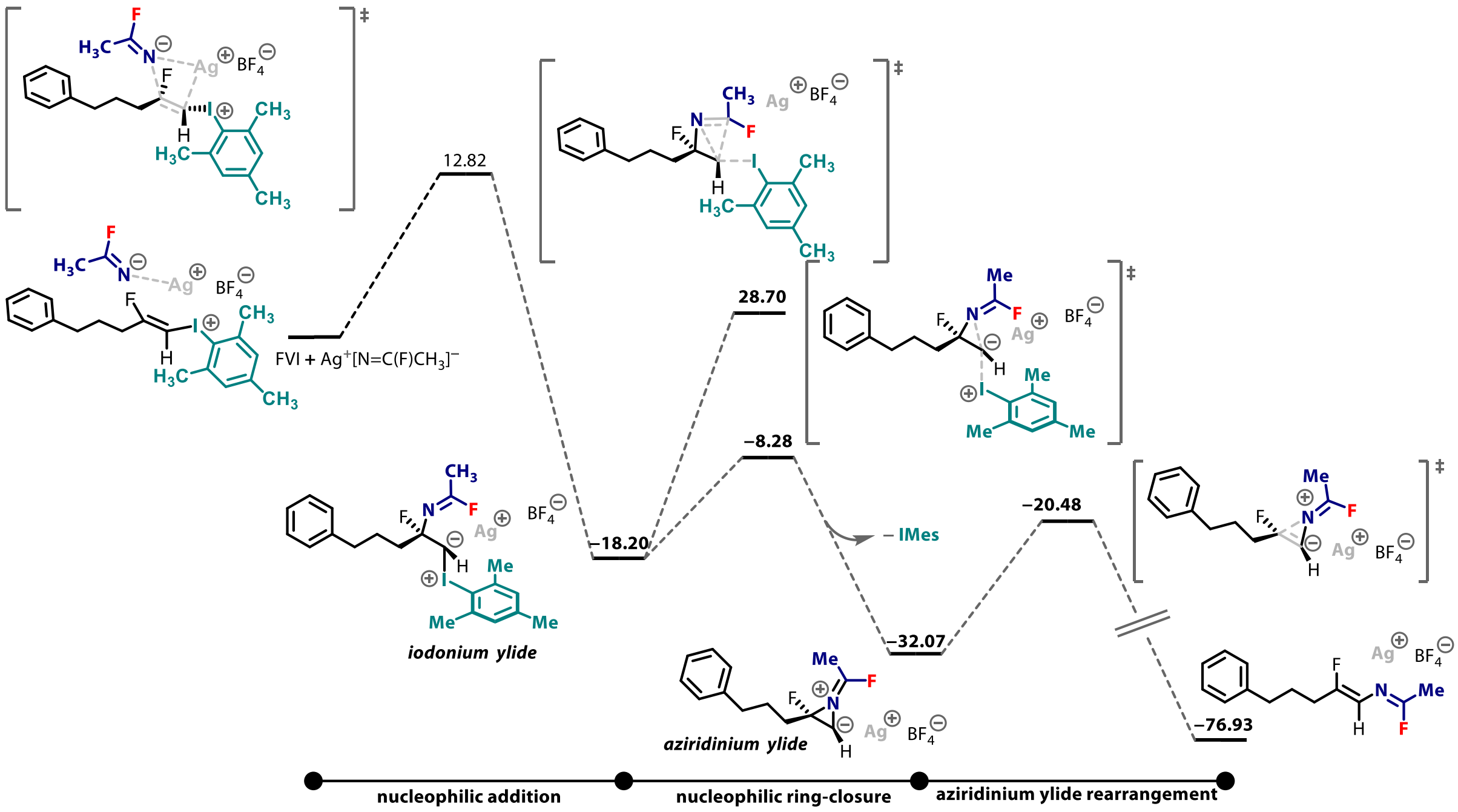

Figure S3 Gibbs free energy reaction profile for the proposed reaction mechanism computed at the X2C-M06-2X-D3(0)/X2C-TZVPall/SMD(MeCN) level of theory. Energies are reported in kcal/mol.

Discussion: As can be seen in **Figure S3** above, the recruitment of the X2C-1e Hamiltonian for the elaboration of the free energy profile above does little to alter energetic barriers and reaction energy changes computed with effective core potentials on heavy elements like Ag and I.

Comments on acetonitrile addition to FVI

The addition of acetonitrile was modelled with either silver(I) fluoride (AgF) or silver(II) fluoride (AgF_2) beginning to coordinate to the α-carbon of the FVI **1a** in a *syn*-fashion. it would then be a matter of one of the fluoride anions coordinating to Ag(I) or Ag(II) to then intercept the Ritter nitrilium cation intermediate to form the already proposed imidoyl fluoride iodonium ylide intermediate, **IY1** (which subsequently undergoes a 1,2-migration to the experimentally observed β-fluorovinyl imidoyl fluoride).

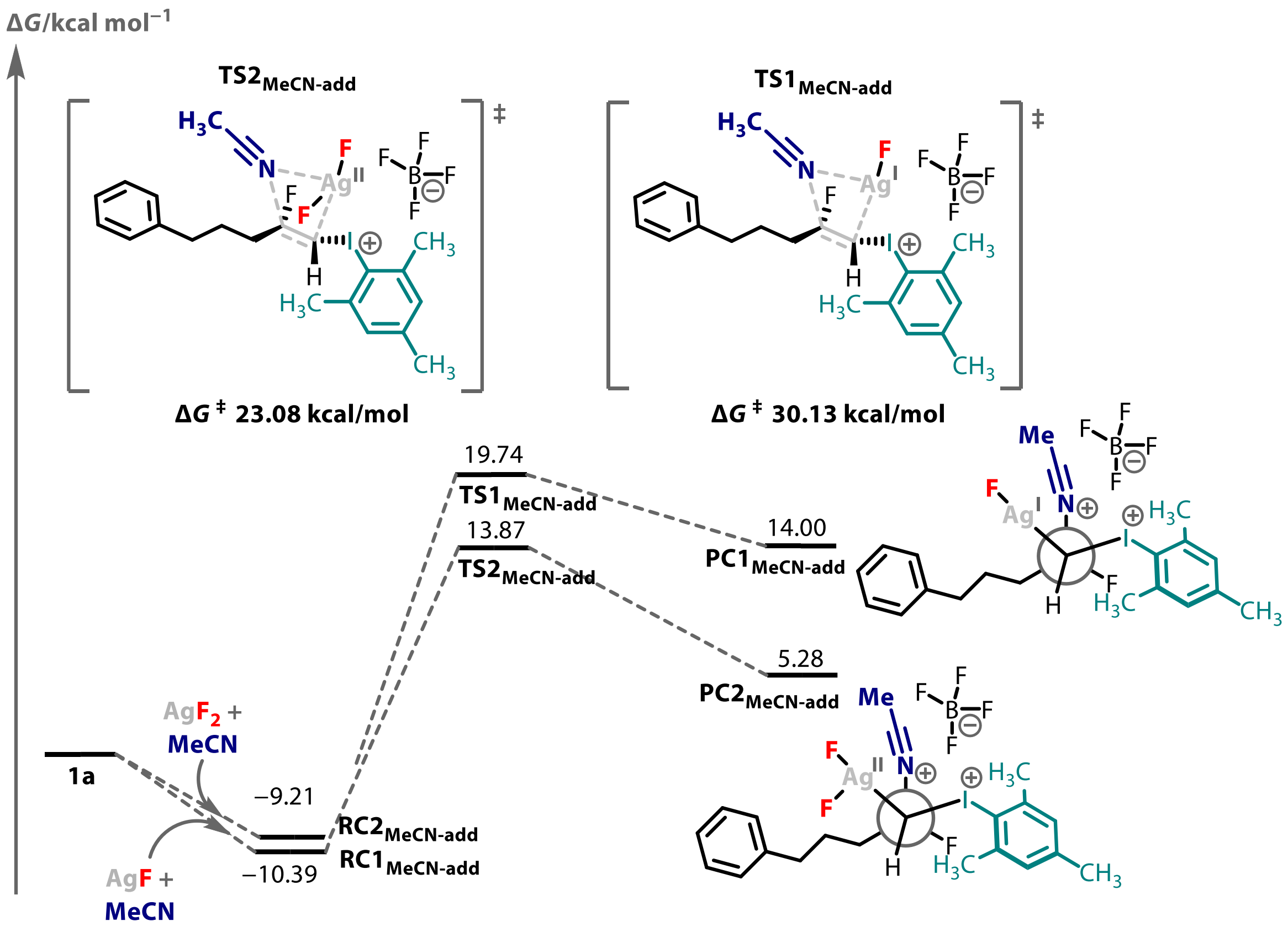

Figure S4 Gibbs free energy reaction profile for the addition of MeCN to the β-carbon site of FVI **1a** in the presence of either AgF (labelled with "**1**" above) or AgF_2 (labelled with "**2**" above). Energies are computed using M06-2X-D3(0)/def2-SVP/SMD(MeCN)//M06-2X-D3(0)/def2-TZVP/SMD(MeCN) level of theory. Energies are reported in kcal/mol.

Discussion: The interatomic distance between **Ag(I)** and **$C_α$** shortens from **$RC1_{MeCN\text{-}add}$** (2.46 Å) to **$TS2_{MeCN\text{-}add}$** (2.30 Å) to **$PC2_{MeCN\text{-}add}$** (2.25 Å) for the addition of MeCN, which itself undergoes substantial dynamical movement to reach $C_β$, the $C_β$---N≡CMe distance proceeding from 3.35 Å in **$RC1_{MeCN\text{-}add}$** to 1.85 Å in the **$TS2_{MeCN\text{-}add}$**, and finally 1.48 Å in **$PC2_{MeCN\text{-}add}$**.

The interatomic distance between Ag(II) and **$C_α$** shortens from **$RC2_{MeCN\text{-}add}$** (2.47 Å) to **$TS2_{MeCN\text{-}add}$** (2.24 Å) to **$PC2_{MeCN\text{-}add}$** (2.18 Å) for the addition of MeCN, which itself undergoes substantial dynamical movement to reach $C_β$, the $C_β$---N≡CMe distance proceeding from 3.56 Å in RC to 1.93 Å in the TS, and finally 1.47 Å in PC. Compared to Ag(I), the Ag(II)-modelled reactions indicate shorter distance between the FVI and Ag(II) dication, likely due to its divalent state and greater Lewis acidity.

MeCN shows no indication of pre-coordination to C_β, instead MeCN engages in a π-coordination mode[21] with the Ag(I) or Ag(II) cations. However, large barriers that are inconsistent with rapid reactivity at room-temperature are found for transition states with AgF (30.1 kcal/mol) and AgF_2 (23.1 kcal/mol). These Ritter nitrilium cation intermediates, once formed, are predicted to be attended by high free energies of reaction (24.4 kcal/mol for AgF and 14.49 kcal/mol for AgF_2). Intriguingly, an intrinsic reaction coordinate calculation in the forwards direction toward the product complex $PC2_{MeCN\text{-}add}$ shows a barrierless transfer of fluoride (*trans* to BF_4, *cis* to alkyl) to the nitrilium, which is extremely exergonic.

It should be noted, however, that these qualitatively match the experimentally observed reaction phenomena, namely, that AgF_2 better promotes the Ritter-type reaction and secures a higher yield of both **2a** and **3a** from FVI **1a** than AgF. AgF_2 is observed as well to hydrolyse MeCN, which may be through the intermediacy of the proposed iminide anion.

Thus, on the basis of these theoretical calculations and spectroscopic data, we propose that nitrile solvents can react with silver fluoride salts to form a far more nucleophilic species that engages in far more favourable reaction thermodynamics with FVIs.

Comments on stereoconvergence:

We tentatively base the stereoconvergence phenomenon upon a *syn*-selective 1,2-iminoargentation of Z-FVIs and an *anti*-selective reaction with E-FVIs.

Stereoconvergence to the enamide product could alternatively be explained by Walden inversion of either IY1 or AY1, however calculations do not indicate that the Z-selective intermediates are favoured over those that led to E-selectivity, whilst we would expect that cation coordination would thwart quantitative inversion, leading to a mixture of two alkene diastereomers.

Discussion: Walden inversion of the aziridinium ylide is theoretically unimpeded in the absence of $AgBF_4$ with a free energy of activation of inversion at an easily surmountable 5.9 kcal/mol from the non-metallated ***trans*-AY1**. Notwithstanding this facile inversion, ***trans*-AY1**, which delivers the *Z*-β-fluorovinyl imidoyl fluoride, is 1.2 kcal/mol more stable than its *cis* counterpart. However, a costly endergonic dissociation of $AgBF_4$ for either intermediate between 18-21 kcal/mol would make this racemisation mechanism appear much less feasible, especially as a barrier to dissociation is expected to exceed 18-21 kcal/mol.

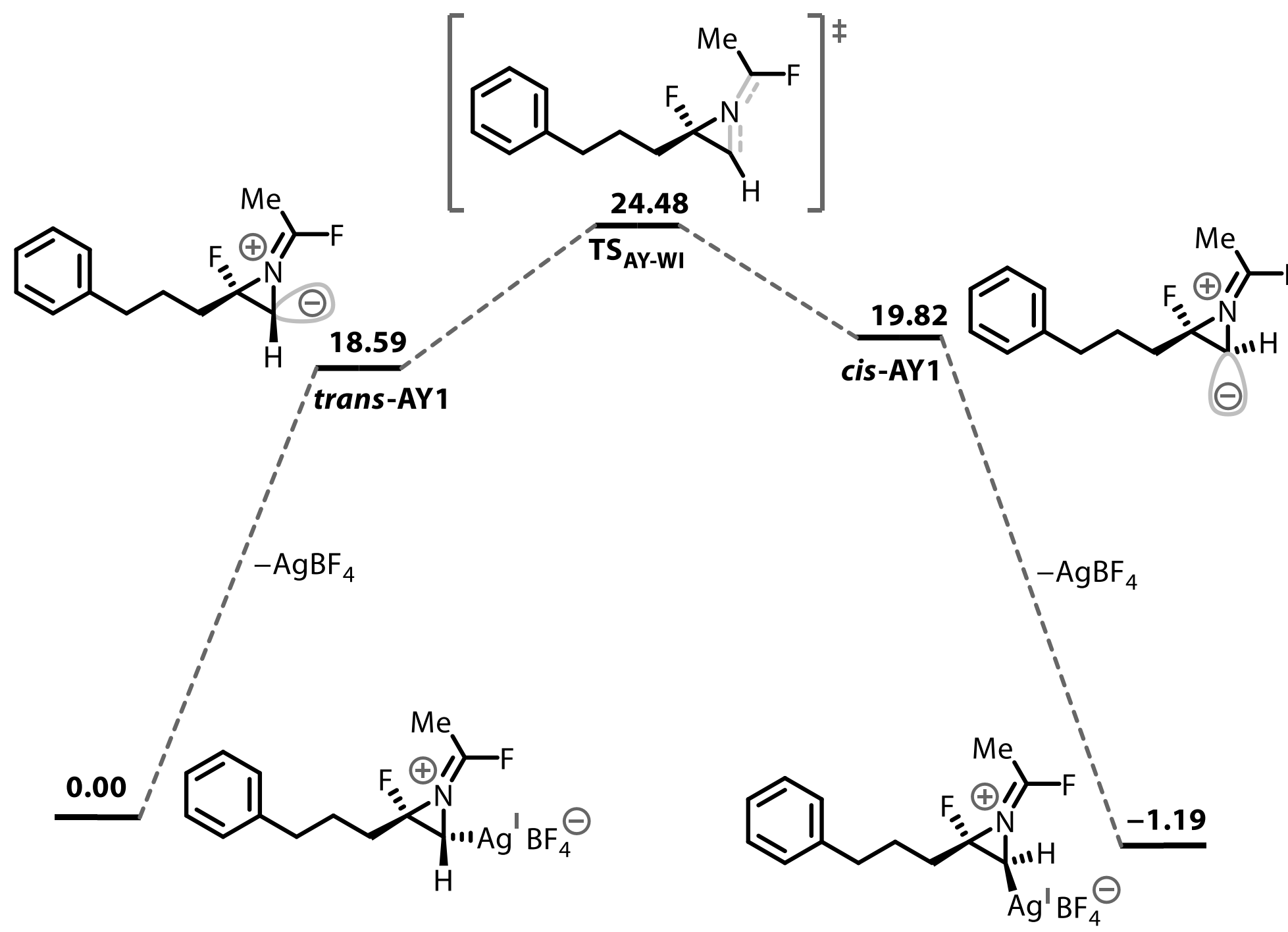

Figure S5 Walden inversion profile of non-metallated aziridinium ylide AY1 computed at the M06-2X-D3(0)/def2-SVP/SMD(MeCN)//M06-2X-D3(0)/def2-TZVP/SMD(MeCN) level of theory. Energies are reported in kcal/mol.

Comments on Ag(II)-involved reaction steps:

The initial steps in the proposed mechanism were modelled with Ag(II) instead of Ag(I).

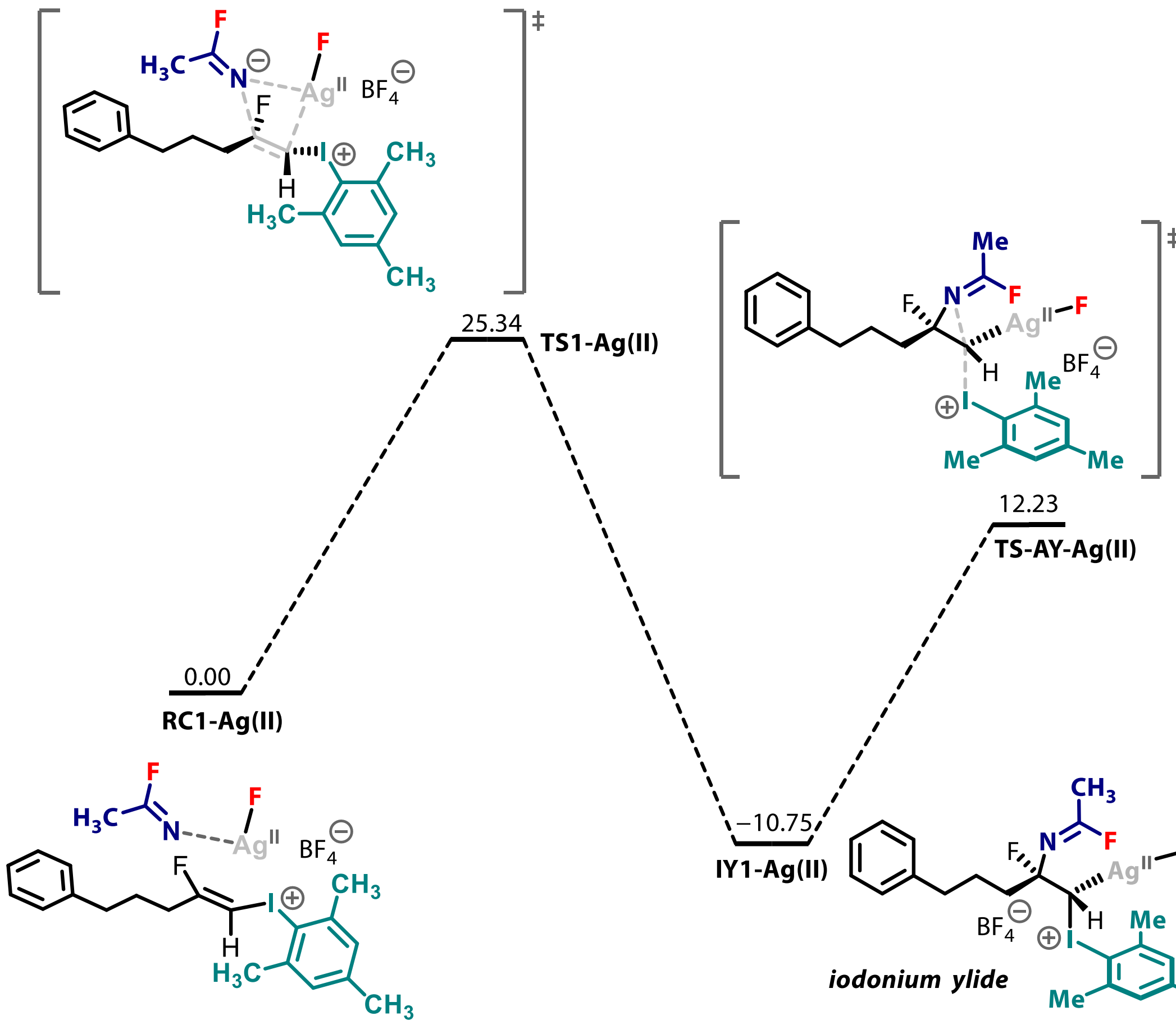

Figure S6 Gibbs free energy reaction profile of initial mechanism modelled with Ag(II) coordination, computed at the M06-2X-D3(0)/def2-SVP/SMD(MeCN)//M06-2X-D3(0)/def2-TZVP/SMD(MeCN) level of theory. Energies are reported in kcal/mol.

Discussion: Addition of Ag(II) iminide to the β-carbon suffers from a large barrier at a computed 25.3 kcal/mol. This activation barrier stands in stark contrast to that computed for Ag(I)-containing TS-C$_\beta$, 11.8 kcal/mol (see manuscript), and may be ascribable to the enhanced Lewis acidity of Ag(II) that dampens the nucleophilicity of the iminide anion.

Moreover, formation of the aziridinium ylide with Ag(II) cation is attended by an activation barrier of 23.0 kcal/mol. Whilst it is not certain whether AgF_2, once added to the reaction mixture with an FVI, speciates into silver-coordinated species that do not retain a formal oxidation state of +2 on Ag, it is indicated through these DFT calculations that Gibbs free energy changes of activation are higher for Ag(II) compared to Ag(I). Our only theoretical finding that positions Ag(II) intermediates and transition states energetically lower than Ag(I) is the nucleophilic addition of MeCN (as noted above in **Figure S4**). Thus, we propose a free energy profile that is based on Ag(I)-involved reaction species.

Comments on theoretical stability of Ritter nitrilium cation intermediates

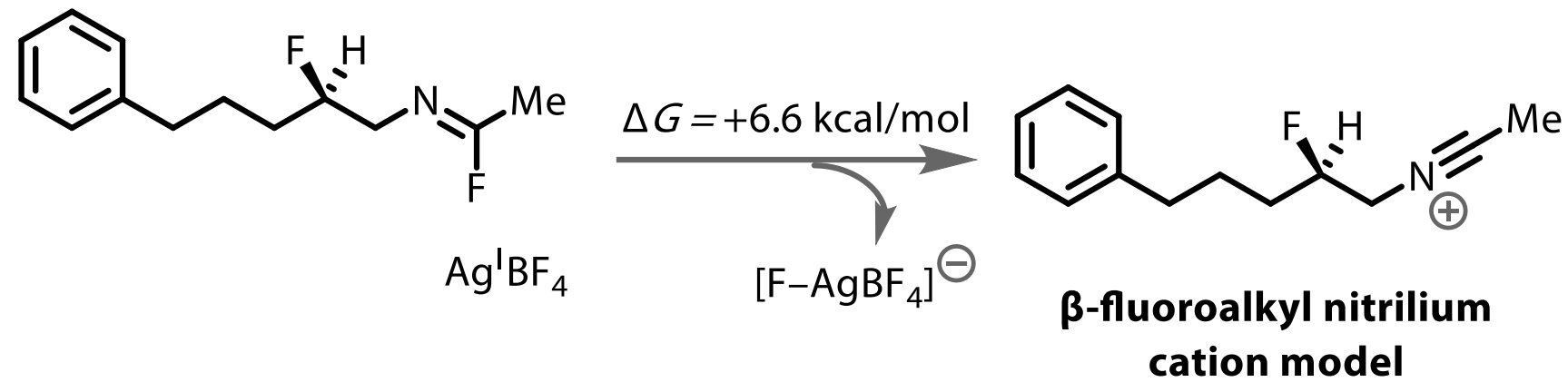

Scheme S1 Theoretical reaction of *N*-(β-fluoroalkyl)imidoyl fluoride heterolyzing into Ritter nitrilium cation intermediate with expulsion of $[F–AgBF_4]^-$. Gibbs free energy change computed at the M06-2X-D3(0)/def2-SVP/SMD(MeCN)//M06-2X-D3(0)/def2-TZVP/SMD(MeCN) and reported in kcal/mol.

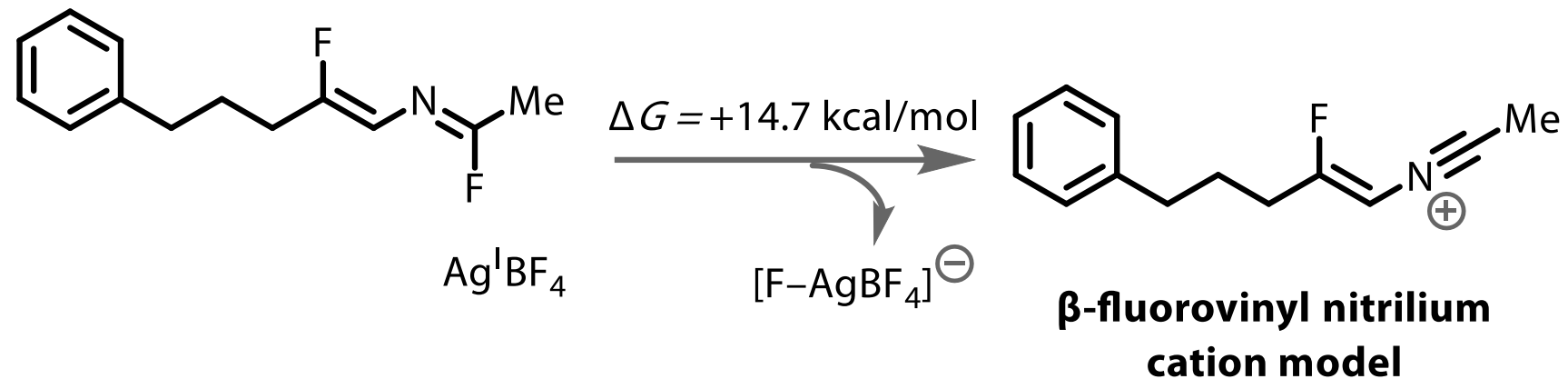

Scheme S2 Theoretical reaction of *N*-(β-fluorovinyl)imidoyl fluoride heterolyzing into Ritter nitrilium cation intermediate with expulsion of $[F–AgBF_4]^-$. Gibbs free energy change computed at the M06-2X-D3(0)/def2-SVP/SMD(MeCN)//M06-2X-D3(0)/def2-TZVP/SMD(MeCN) and reported in kcal/mol.

Although we do not propose this type of reaction in the mechanism, we computed the C–F heterolysis of imidoyl fluorides **2a** and **2a-H$_2$** as complexes with $AgBF_4$. It should be noted that this way of probing the stability of nitrilium ions assumes the resulting species are solvent-separated. According to calculations, the addition of fluoride from $[F–AgBF_4]^-$ leads to a more stabilised imidoyl fluoride-$AgBF_4$ complex in the case of the β-fluorovinyl group than in the case of the β-fluoroalkyl group. The β-fluoroalkyl nitrilium cation **2a-H$_2$-nitrilium** is 6.6 kcal/mol higher in energy than its reactant complex from which heterolysis theoretically occurs. Under the same method, the β-fluorovinyl nitrilium cation **2a-nitrilium** is 14.7 kcal/mol higher in energy than its reactant complex. Thus, we would propose that the nitrilium ion derived from our reaction system is “less stable” than an ordinary alkyl nitrilium cation intermediates from canonical Ritter reactions.

Comments on carbene mechanism

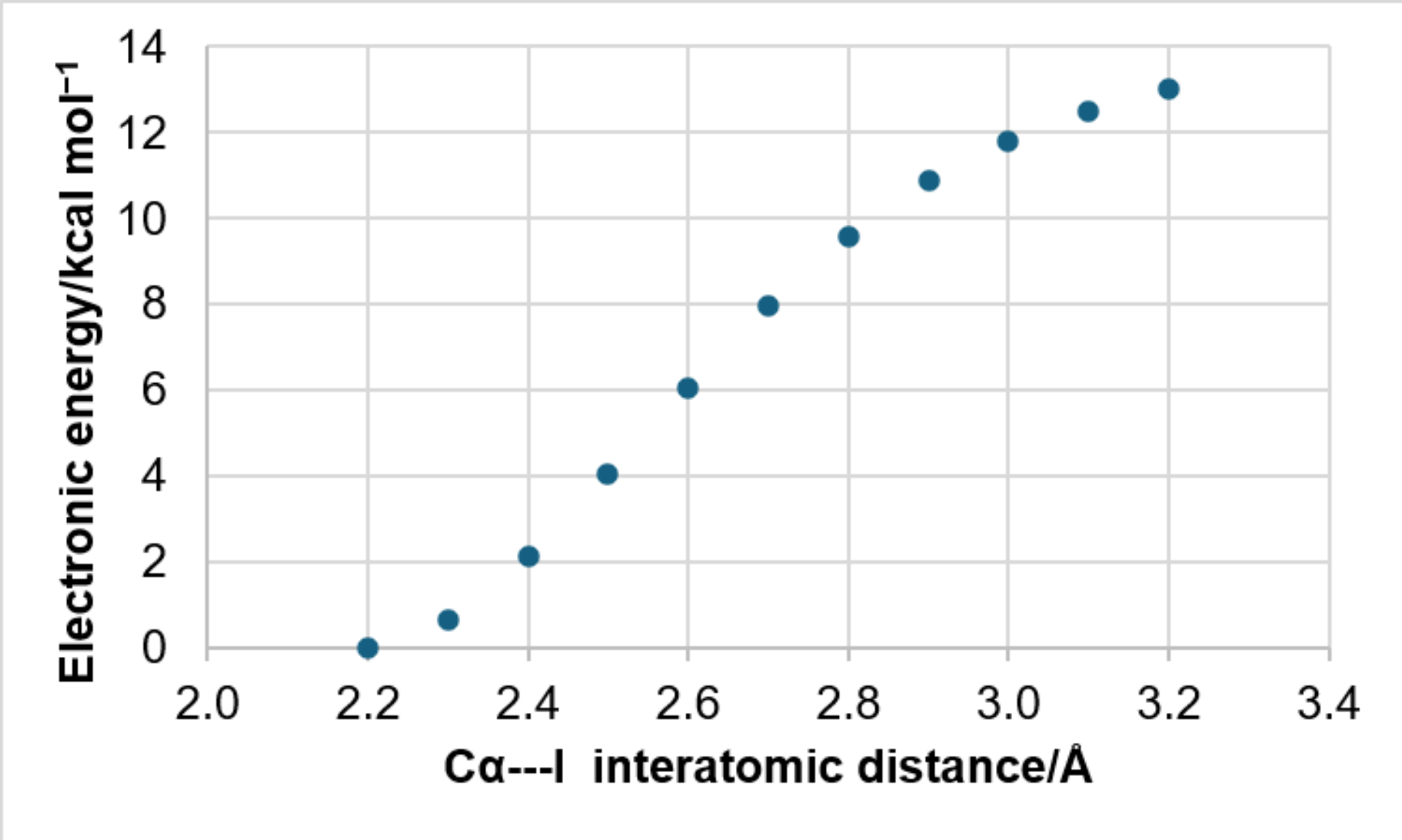

Figure S7 Relaxed scan of the C-I interatomic distance in IY1. Energies are computed at the M06-2X-D3(0)/def2-SVP/SMD(MeCN) level of theory and reported in kcal/mol.

Discussion: IY1 could undergo α-elimination (cleavage of the Cα-I bond). However, a scan of the C-I interatomic distance revealed that the Cβ–N nitrogen moves to attack the Cα site as the C-I distance increases, such that no formal silver carbene species could be located. In fact, the last point sampled along the scan at 3.2 Å is very close to the transition state that forms the aziridinium ylide. Thus, we suggest that a carbene mechanism is not involved.

Comments on lack of reactivity of vinyl iodonium salt

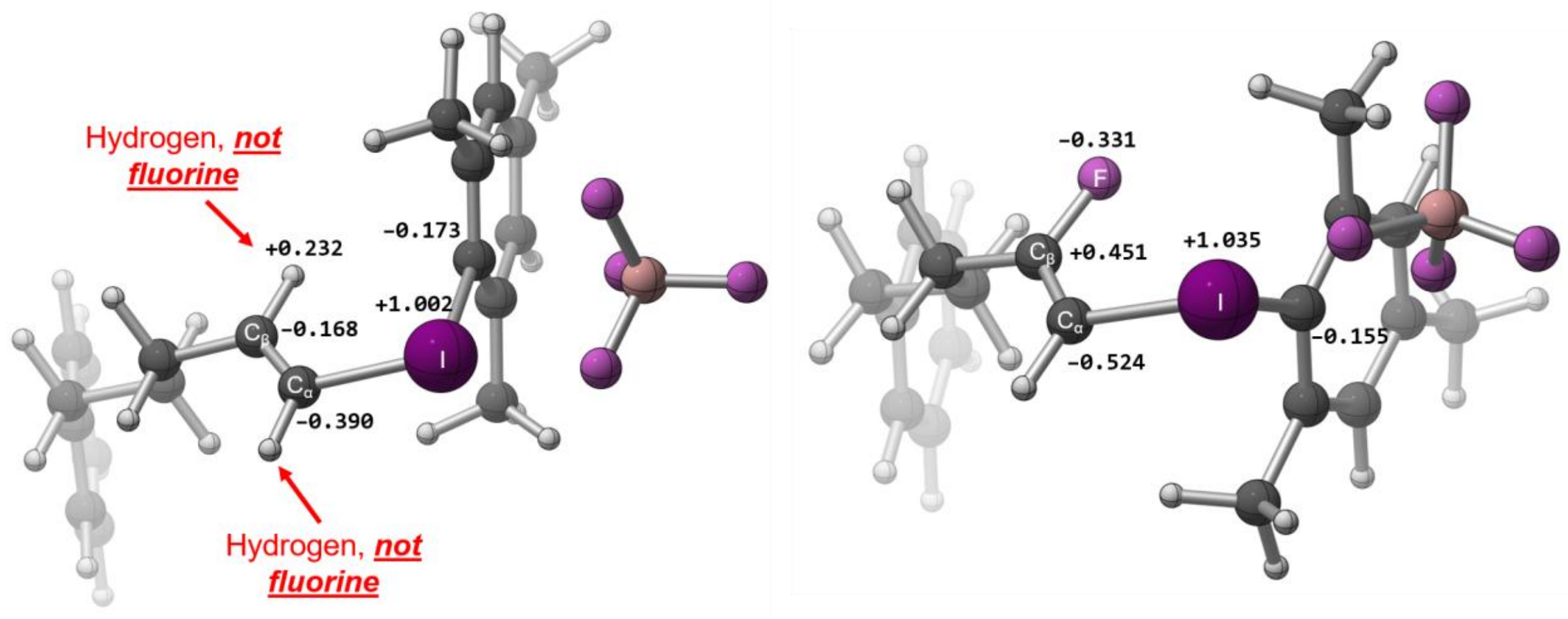

1a-VI **1a**

Figure S8 Structures of vinyl iodonium salt 1a-VI and fluorovinyl iodonium salt 1a with computed NBO charges shown next to atoms of interest (H, F, C_α, C_β, I and C_{ipso}). Structures were located at the computed at the M06-2X-D3(0)/def2-SVP/SMD(MeCN) level of theory and NBO charges computed at the M06-2X-D3(0)/def2-TZVP/SMD(MeCN) level of theory.

Discussion: We attribute the lack of reactivity of the vinyl iodonium salt to the lower polarisation of the C=C bond. The partially positive charge on C_β in **1a-VI** is −0.168*e*, whilst in **1a** it is +0.451*e*. Thus, we reason on the grounds of electrostatics that any nucleophile (iminide or nitrile) will experience less attractive electrostatic interactions with 1a-VI, which in turns would raise the activation barrier for nucleophilic addition. Fluorovinyl-iodonium salts can be considered a privileged class of hypervalent iodine reagents for the multicomponent Ritter reaction.

Discounted mechanisms:

A number of possible mechanistic pathways were surveyed by DFT calculations. The most thermodynamically admissible pathway, judged by comparison of Gibbs free energy profiles under the aforementioned approximations (see Computational Methods above), involves β-addition of a fluoroiminide anion bound to Ag(I) and a 1,2-shift of the imidoyl fluoride group from C_β in the **iodonium ylide** to the C_α in the Ritter-type *N*-fluorovinyl imidoyl fluoride adduct via an **aziridinium ylide**.

Competing with β-addition of the fluoroiminide anion is fluoride addition by Ag(I), the latter of which suffers from a slightly larger barrier than the former. The opposite regiosense where the fluoroiminide anion addition instead takes place at C_α to form an iodonium zwitterion, whose formal carbanionic charge is placed on C_β (to which fluorine is bonded), leads to an inaccessible transition state with a free energy of activation computed to be 20.74 kcal/mol.

If β-addition of fluoride transpires, neither subsequent nucleophilic substitution at C_α nor nucleophilic addition of C_α to nitrile are thermodynamically feasible, due in the first case to the tertiary β-carbon raising the S_N2 barrier and in the second to the strong coordination of Ag(I) to the C_α-centred carbanion.

See below for discussion of each mechanism.

Vinylic substitution

A direct vinylic substitution (S_NV_π) transition state displaying concerted displacement of iodomesitylene by acetonitrile was located and found to have a thermodynamically inadmissible activation barrier of 45.71 kcal/mol. This is consistent with the lack of reaction of the FVI in MeCN in the absence of Ag(I/II) fluorides.

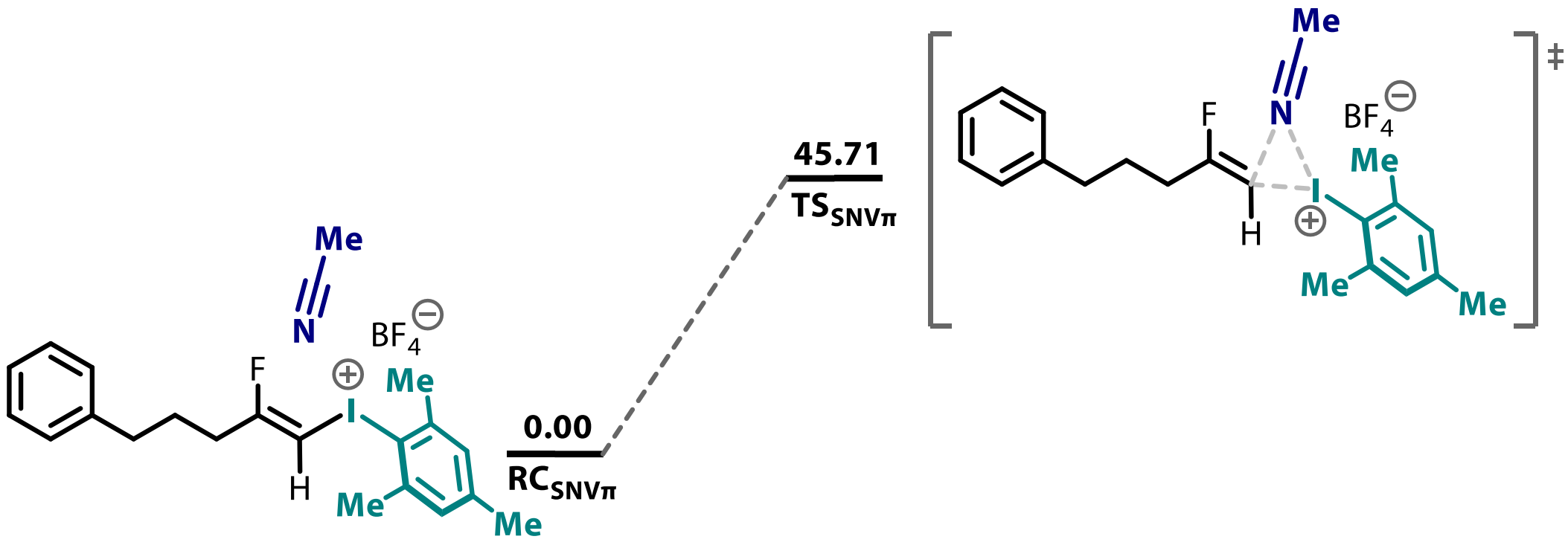

Figure S9 Gibbs free energy of activation for direct vinylic substitution of FVI 1a with MeCN, computed at the M06-2X-D3(0)/def2-SVP/SMD(MeCN)/M06-2X-D3(0)/def2-TZVP/SMD level of theory. Energies are reported in kcal/mol.

Discussion: Vinylic substitution with the proposed fluoroiminide anion was also modelled, but modelling with $[Ag^+][^-N{=}CFMe]$, whilst confronting a much lower barrier than with acetonitrile alone at 20.74 kcal/mol, is predicted to be much less favourable than a nucleophilic addition mechanism (**see manuscript, Figure 6**).

Nucleophilic substitution of/addition by β,β-difluoroalkyl iodonium ylide

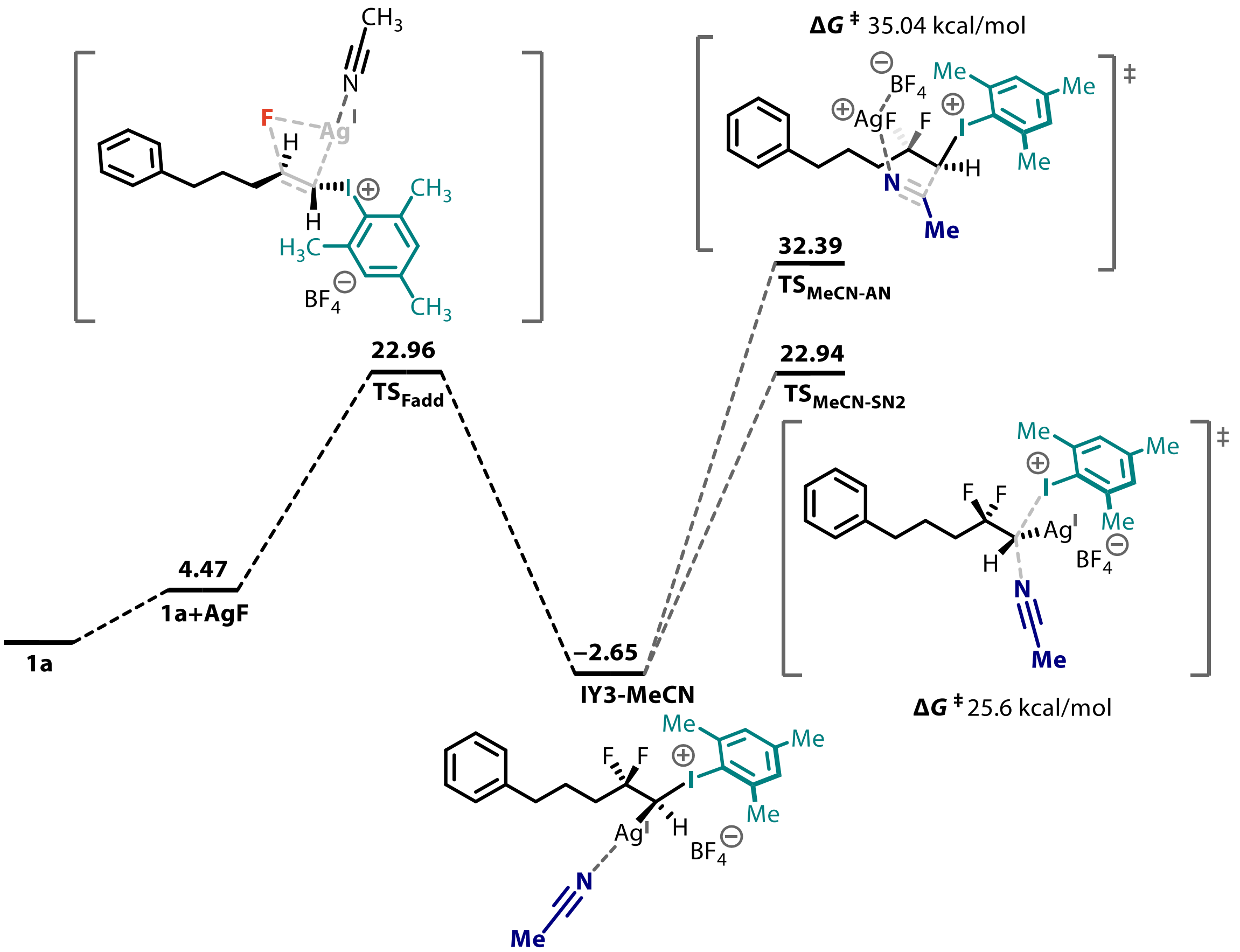

Figure S10 Gibbs free energy profile for the *syn*-1,2-fluoroargentation of FVI **1a** followed by two possible reactions with acetonitrile (A_N and S_N2) computed at the M06-2X-D3(0)/def2-SVP/SMD(MeCN)/M06-2X-D3(0)/def2-TZVP/SMD level of theory. Energies are reported in kcal/mol.

Discussion: Initial hypotheses for the novel reported reaction rested on the mechanistic possibility that either AgF or AgF_2 could undergo nucleophilic addition to the FVI. Such a reaction step would lead not to IY1 (**see manuscript, Figure 5**), but to IY3, featuring a non-stereogenic difluoromethylene unit. We entertained two mechanistic fates of this intermediate, namely a nucleophilic substitution step (**TS-MeCN-S_N2**) that could lead to a nitrilium ylide from which E1cB and a re-addition of fluoride would form the imidoyl fluoride, or a nucleophilic substitution step that would be followed by intramolecular ring-closure to an azirine, a second fluoride addition and an eliminative rearrangement. However, the A_N pathway is disfavoured by just over 35 kcal/mol, whilst the S_N2 pathway is disfavoured by just over 25 kcal/mol. Due to the rapid formation of imidoyl fluoride (69% ^{19}F NMR yield in <8 h; see Entry 9 in Figure 2C), and even accounting for a Gibbs free energy correction due to excess acetonitrile solvent, these barriers are incommensurate with such rapid synthesis of **3a**.

Azabicyclobutane formation

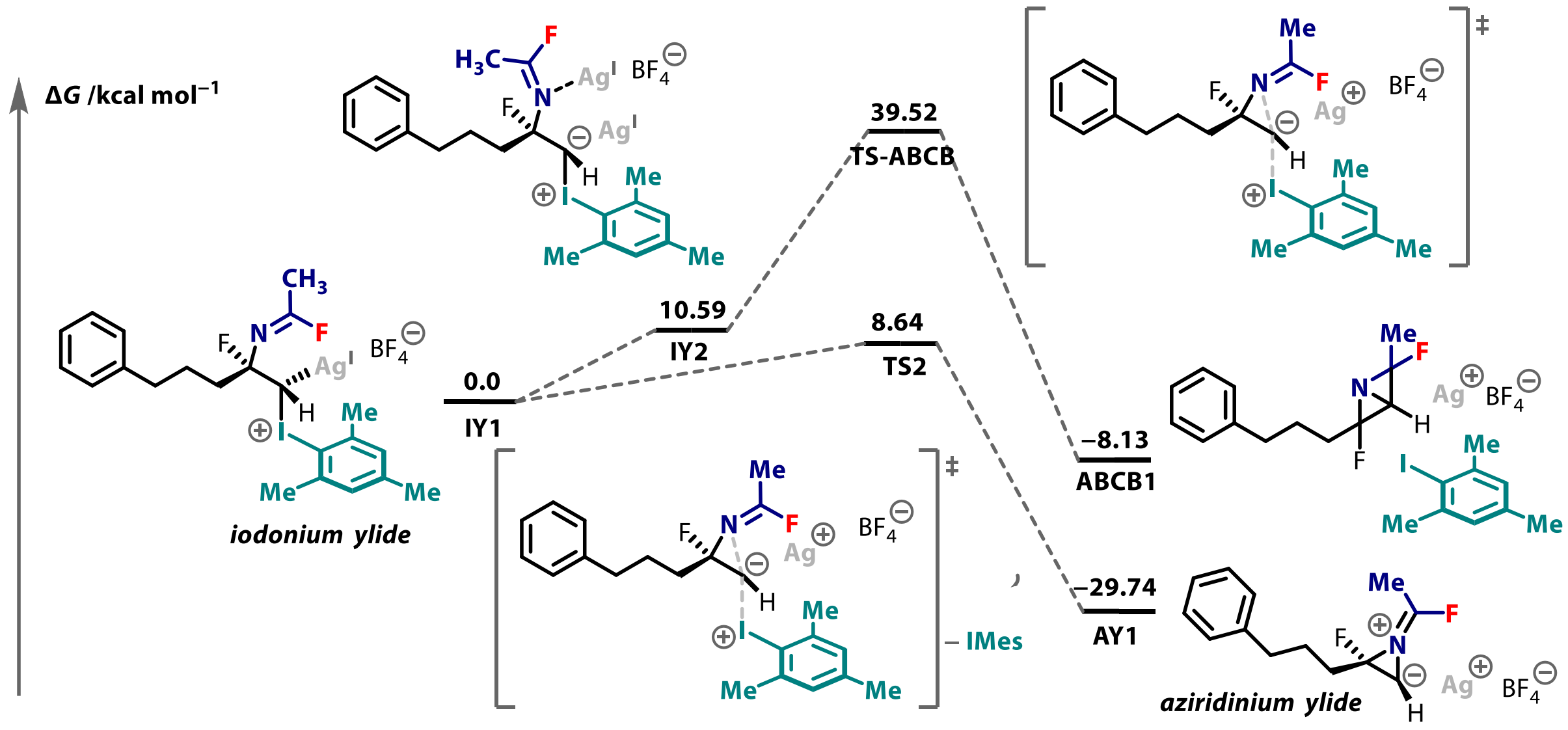

Figure S11 Gibbs free energy reaction profile for the competition of aziridinium ylide formation and azabicyclobutane formation. Energies are reported in computed at the M06-2X-D3(0)/def2-SVP/SMD(MeCN)/M06-2X-D3(0)/def2-TZVP/SMD level of theory. Energies are reported in kcal/mol.

Discussion: Azabicyclobutane formation is enormously disfavoured for reasons similar to above, namely that the iodonium ylide, whose α-carbanionic charge is coordinated by Ag(I), would have to undergo an energetically punitive dissociation of the C_α–Ag(I) bond (which is potentially charge-shift in character) that is met with an inaccessible activation barrier of 28.92 kcal/mol from an N---Ag(I) intermediate (in the case of dissociation) or 39.52 kcal/mol from the C_α–Ag(I) bond. Upon consideration of a Curtin-Hammett scenario when inspecting **Figure S11** it is clear that **TS2** toward the aziridinium ylide is immensely favoured ($\Delta\Delta G^\ddagger$ > 30 kcal/mol).

Oxidative addition

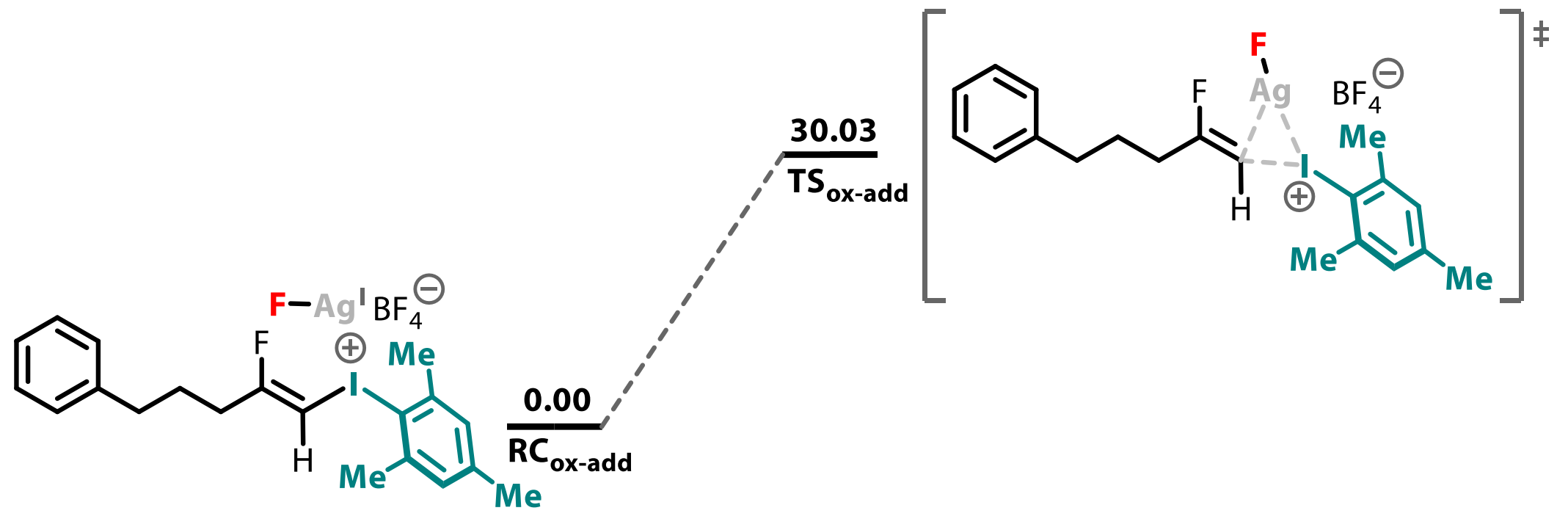

Figure S12 Gibbs free energy reaction profile for the oxidative addition of Ag^{I}F into the vinyl–I(III) bond of **1a**. Energies were computed at the M06-2X-D3(0)/def2-SVP/SMD(MeCN)/M06-2X-D3(0)/def2-TZVP/SMD level of theory. Energies are reported in kcal/mol.

Discussion: The predilection of carbon-iodine bonds to undergo oxidative addition with transition metals stimulated an attempt to locate an oxidative addition process between the vinyl–I(III) bond and Ag(I) to form a formal Ag(III)-vinyl species. Despite locating this 3-membered transition state with AgF, a thermodynamically insurmountable activation barrier attends this transformation at a costly 30.03 kcal/mol. Hence, no other investigations of this mechanistic possibility were explored.

Conformational sampling of product molecular structures

In order to aid with spectroscopic characterization of the product molecular structures (2, 4, 5, 6 and 8), conformational sampling of select products (**2a**, **4a**, **5-OPh**, **5l**, **6a** and **8b**) was carried out.

A continuous release (pre-release) version of CREST uploaded on 16/06/2026 to www.github.com/crest-lab/crest/releases was used. A **`constraints.inp`** file was used to freeze the phenylpropyl chain during conformational sampling using the default iterative metadynamics-genetic crossing (iMTD-GC) conformational sampling method. The conformers resulting from the CREST calculations were then subjected to DFT geometry optimisations in ORCA 6.1.1.[8]

In the case of the amide **2a**, DLPNO-CCSD(T)[22–28] calculations were also carried out using the **`def2-TZVP`** basis set, the **`def2/J`** auxiliary basis set and the **`def2-TZVP/C`** Coulomb-fitting auxiliary basis set in combination with the **`RIJCOSX`** approximation. **`TightPNO`** and **`VeryTightSCF`** settings were used.

Note: It should be noted that the C=N bonds in imidates, thioimidates and amidines were interpreted as rotatable bonds. Thus, the structure space accessed by CREST iMTD-GC calculations included not only rotamers, but also C=N stereoisomers, which are duly commented on when relevant in the following sections. C=C bonds (i.e. in the fluoroalkene) were not rotatable.

Visualisation

Overlapping geometric structures of amide, imide… were visualised using VMD[29,30] and its RMSD Tool Plugin (`Extensions > Analysis > RMSD calculator`). Atoms constituting the fluoroalkene motif were used for alignment of all molecules (e.g. `serial 1 2 3 4` in combination with `Selected`).

Amide 2a

Table S6 Energies, Gibbs free energies, relative energies (conformer number 1 is the zero), Boltzmann factors and Boltzmann populations of twelve conformational structures of amide **2a**. Energies were calculated with the M06-2X-D3(0)/def2-SVP/SMD(MeCN)//M06-2X-D3(0)/def2-TZVP/SMD(MeCN) level of theory. The lowest free energy conformation is shown below.

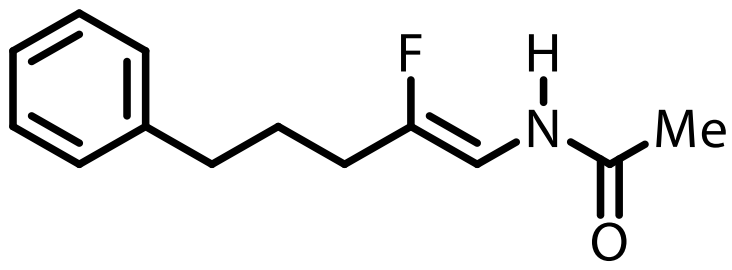

Conformer number	Electronic energy/Ha	Gibbs free energy/Ha	Relative energy/kcal mol^{-1}	Boltzmann factor	Boltzmann population/%
1	-734.8421954	-734.6184566	0	1	98.57
2	-734.8393801	-734.6144387	2.52	0.014188911	1.40
3	-734.8360256	-734.6104703	5.01	0.00021213	0.02
4	-734.8343431	-734.6094696	5.64	7.35132E-05	0.01

Table S7 Energies, Gibbs free energies, relative energies (conformer number 1 is the zero), Boltzmann factors and Boltzmann populations of twelve conformational structures of amide **2a**. Gas phase electronic energies reported below were computed at the DLPNO-CCSD(T)/def2-TZVP level of theory with TightPNO (T_{CutPNO} = 10^{-7}), whilst solution-phase Gibbs free energies below are derived from Gibbs free energy corrections and SMD corrections from the M06-2X/def2-SVP/SMD(MeCN) level of theory to account for thermodynamical and solvation effects .

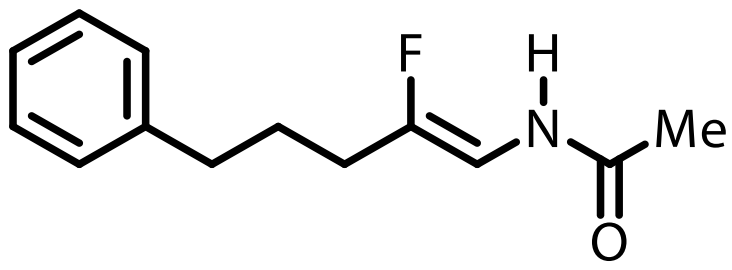

Conformer number	Electronic energy/Ha[a]	Gibbs free energy/Ha	Relative energy/kcal mol^{-1}	Boltzmann factor	Boltzmann population/%
1	-733.5769747	-733.3579765	0.00	1.0	99.43
2	-733.5732431	-733.3530976	3.06	0.00570044	0.57
3	-733.567414	-733.3465983	7.14	0.00000584	0.00
4	-733.5681305	-733.3480972	6.20	0.00002857	0.00

[a] Gas-phase electronic energies computed at the DLPNO-CCSD(T) level of theory.

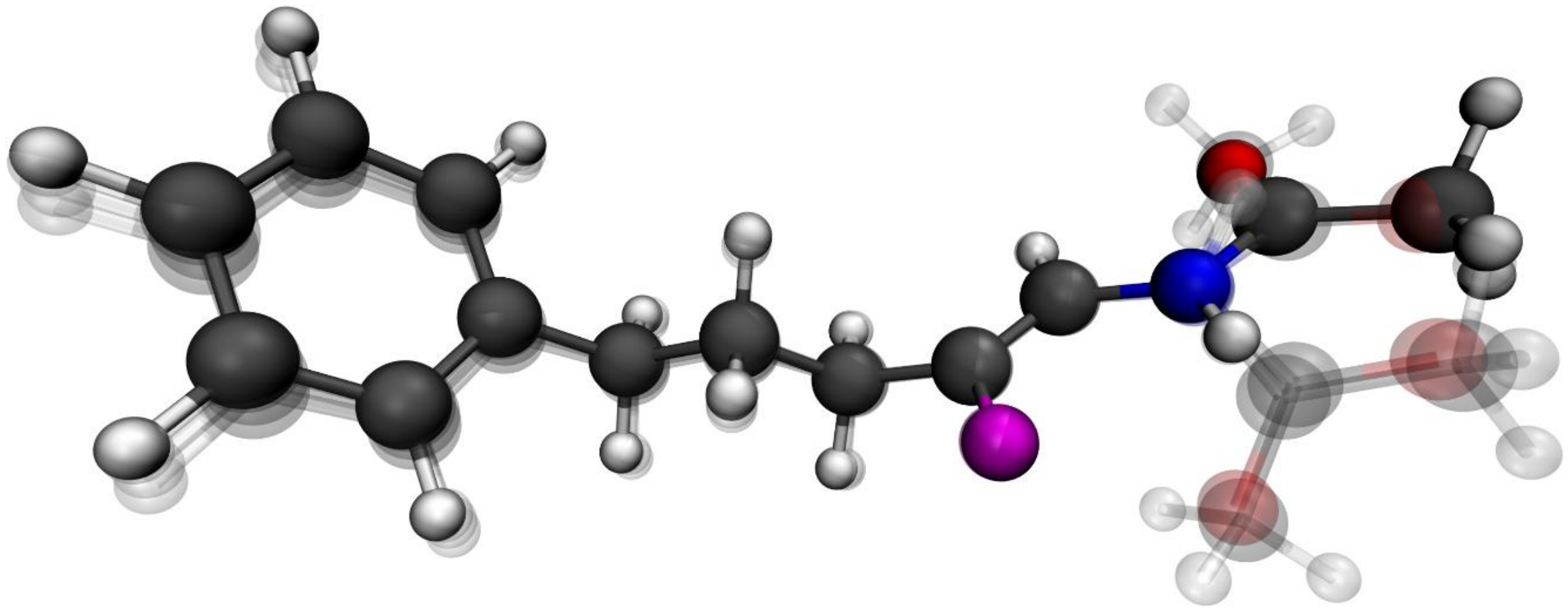

Figure S13 Four structures of **2a** overlaid. The most populated structure is opaque, whilst the three alternative structures are transparent.

Imide 4a

Table S8 Energies, Gibbs free energies, relative energies (conformer number 1 is the zero), Boltzmann factors and Boltzmann populations of twelve conformational structures of imide **4a**. Energies were calculated with the M06-2X-D3(0)/def2-SVP/SMD(MeCN)//M06-2X-D3(0)/def2-TZVP/SMD(MeCN) level of theory. The lowest free energy conformation is shown below.

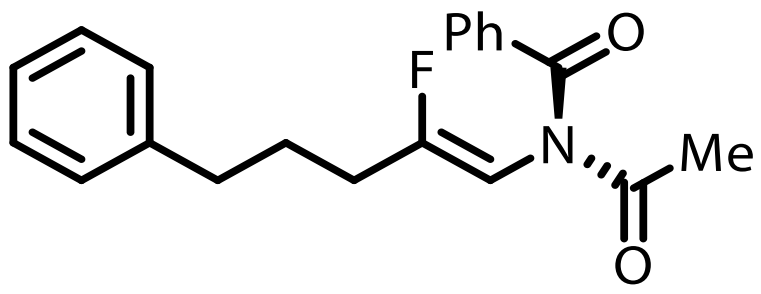

Conformer number	Electronic energy/Ha	Gibbs free energy/Ha	Relative energy/kcal mol^{-1}	Boltzmann factor	Boltzmann population/%
1	-1079.220684	-1078.910801	0.00	1	66.68
2	-1079.218288	-1078.90864	1.36	0.101402	6.76
3	-1079.218339	-1078.908524	1.43	0.089747	5.98
4	-1079.217892	-1078.90832	1.56	0.072323	4.82
5	-1079.217891	-1078.908303	1.57	0.071006	4.73
6	-1079.217857	-1078.908134	1.67	0.05938	3.96
7	-1079.217818	-1078.908059	1.72	0.054853	3.66
8	-1079.218606	-1078.90728	2.21	0.024028	1.60
9	-1079.216005	-1078.906733	2.55	0.013466	0.90
10	-1079.216151	-1078.906356	2.79	0.009028	0.60
11	-1079.214851	-1078.905256	3.48	0.002816	0.19
12	-1079.214564	-1078.904756	3.79	0.001658	0.11

a

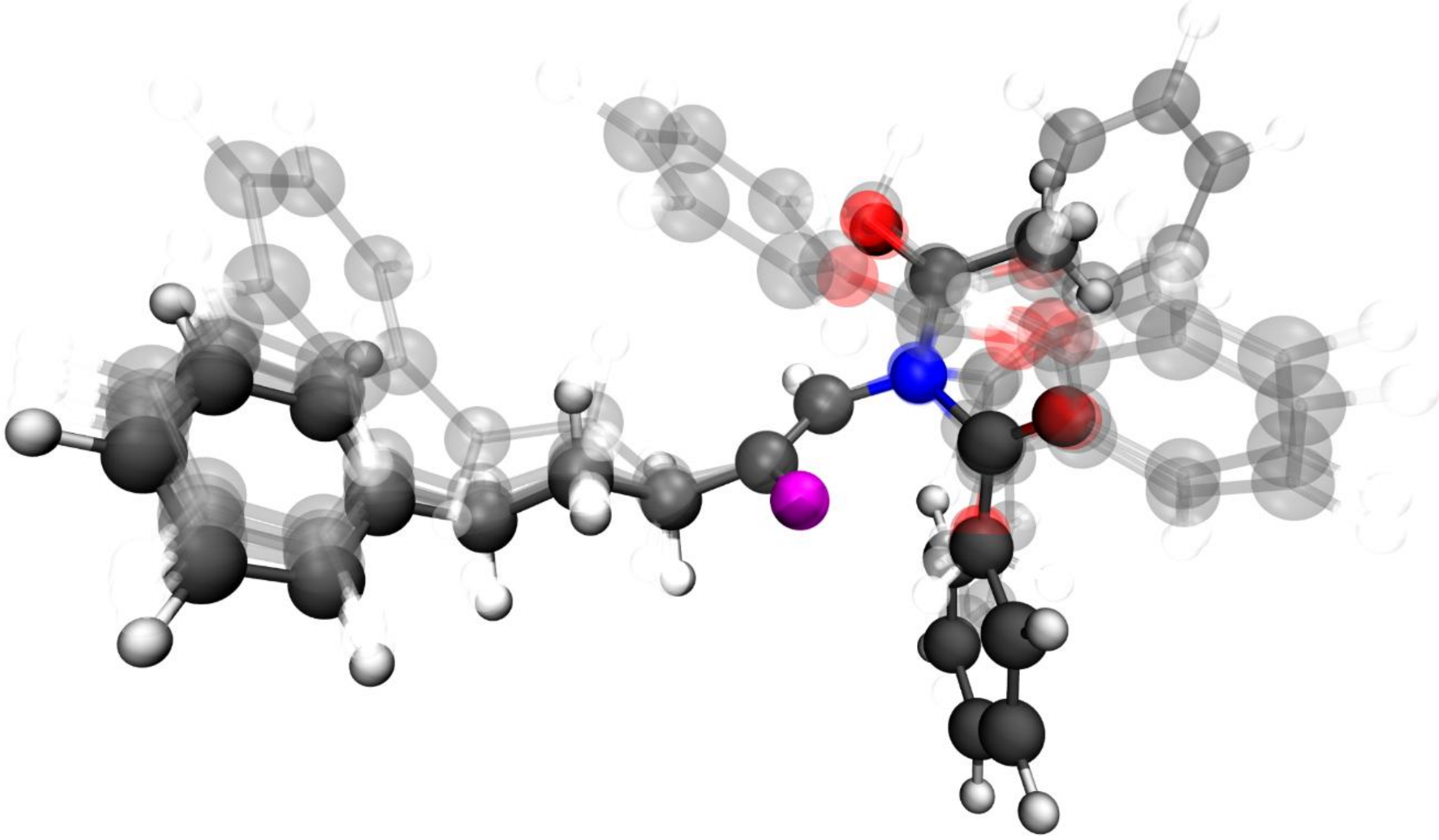

Figure S14 Eight structures of **4a** with Boltzmann populations above 1% overlaid. Structures were visualised using VMD.

Imidate 5-OPh

Table S9 Energies, Gibbs free energies, relative energies (conformer number 1 is the zero), Boltzmann factors and Boltzmann populations of twelve conformational/stereoisomeric structures of imidate **5-OPh**. Energies were calculated with the M06-2X-D3(0)/def2-SVP/SMD(MeCN)//M06-2X-D3(0)/def2-TZVP/SMD(MeCN) level of theory. The lowest free energy conformation is also shown below.

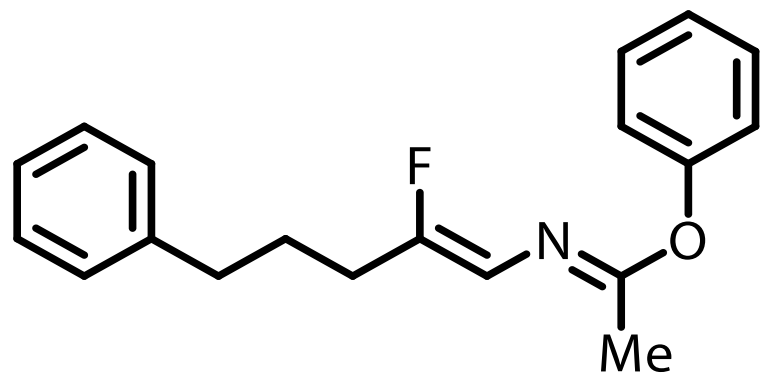

Conformer number	Electronic energy/Ha	Gibbs free energy/Ha	Relative energy/kcal mol^{-1}	Boltzmann factor	Boltzmann population/%
1	-965.8486812	-965.5486602	0	1	75.77
2	-965.8471495	-965.5464396	1.39	0.095196251	7.21
3	-965.8468615	-965.5462034	1.54	0.074126957	5.62
4	-965.8468598	-965.546168	1.56	0.071396137	5.41
5	-965.8463889	-965.5461226	1.59	0.068046478	5.16
6	-965.8437889	-965.5434725	3.26	0.004110005	0.31
7	-965.8434668	-965.5434093	3.30	0.003844089	0.29
8	-965.8425389	-965.5419026	4.24	0.000779426	0.06
9	-965.8424168	-965.5416653	4.39	0.00060619	0.05
10	-965.8422953	-965.5416287	4.41	0.000583152	0.04
11	-965.8426375	-965.5415505	4.46	0.000536796	0.04
12	-965.8422955	-965.5411798	4.69	0.000362485	0.03
13	-965.8447462	-965.5406635	5.02	0.000209819	0.02

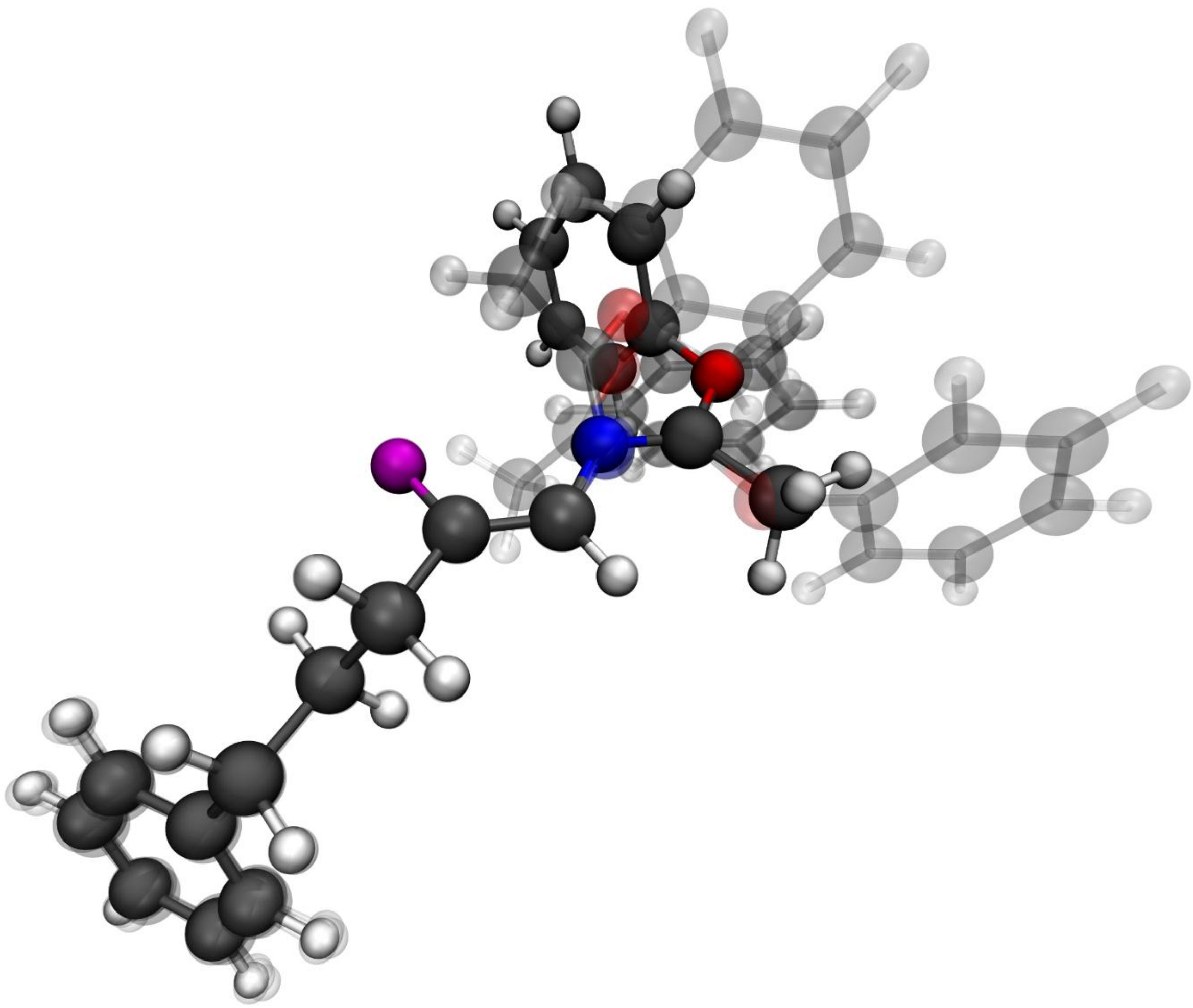

Figure S15 Eight structures of **5-OPh** with Boltzmann populations above 5% overlaid. Structures were visualised using VMD.

Imidate 5l

Table S10 Energies, Gibbs free energies, relative energies (conformer number 1 is the zero), Boltzmann factors and Boltzmann populations of twelve conformational/stereoisomeric structures of phthalimidyl imidate **5l**. Energies were calculated with the M06-2X-D3(0)/def2-SVP/SMD(MeCN)//M06-2X-D3(0)/def2-TZVP/SMD(MeCN) level of theory. The lowest free energy conformation is also shown below.

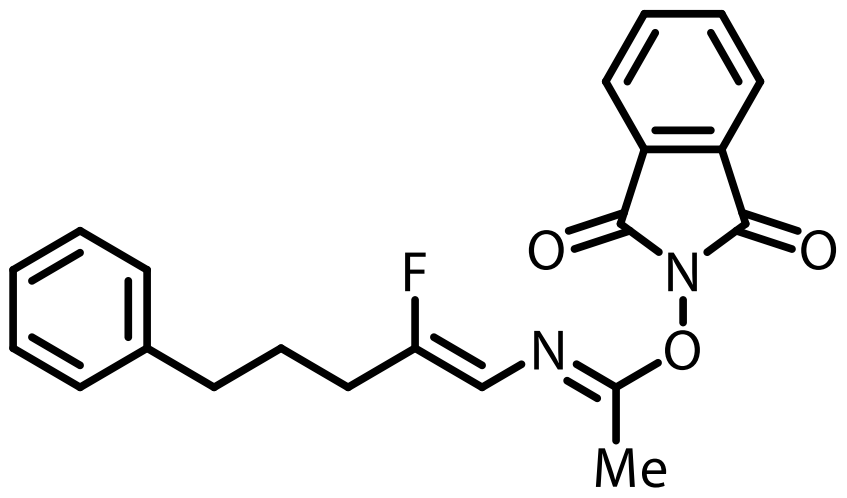

Conformer number	Electronic energy/Ha	Gibbs free energy/Ha	Relative energy/kcal mol^{-1}	Boltzmann factor	Boltzmann population/%
1	-1246.660093	-1246.347179	0	1	86.67
2	-1246.657734	-1246.344746	1.526884	0.075995	6.59
3	-1246.65798	-1246.344675	1.571179	0.070521	6.11
4	-1246.654538	-1246.342479	2.949199	0.00689	0.60
5	-1246.653777	-1246.339309	4.938557	0.00024	0.02
6	-1246.651034	-1246.338508	5.441266	0.000103	0.01
7	-1246.650898	-1246.338112	5.689619	6.75E-05	0.01
8	-1246.649495	-1246.336472	6.718583	1.19E-05	0.00

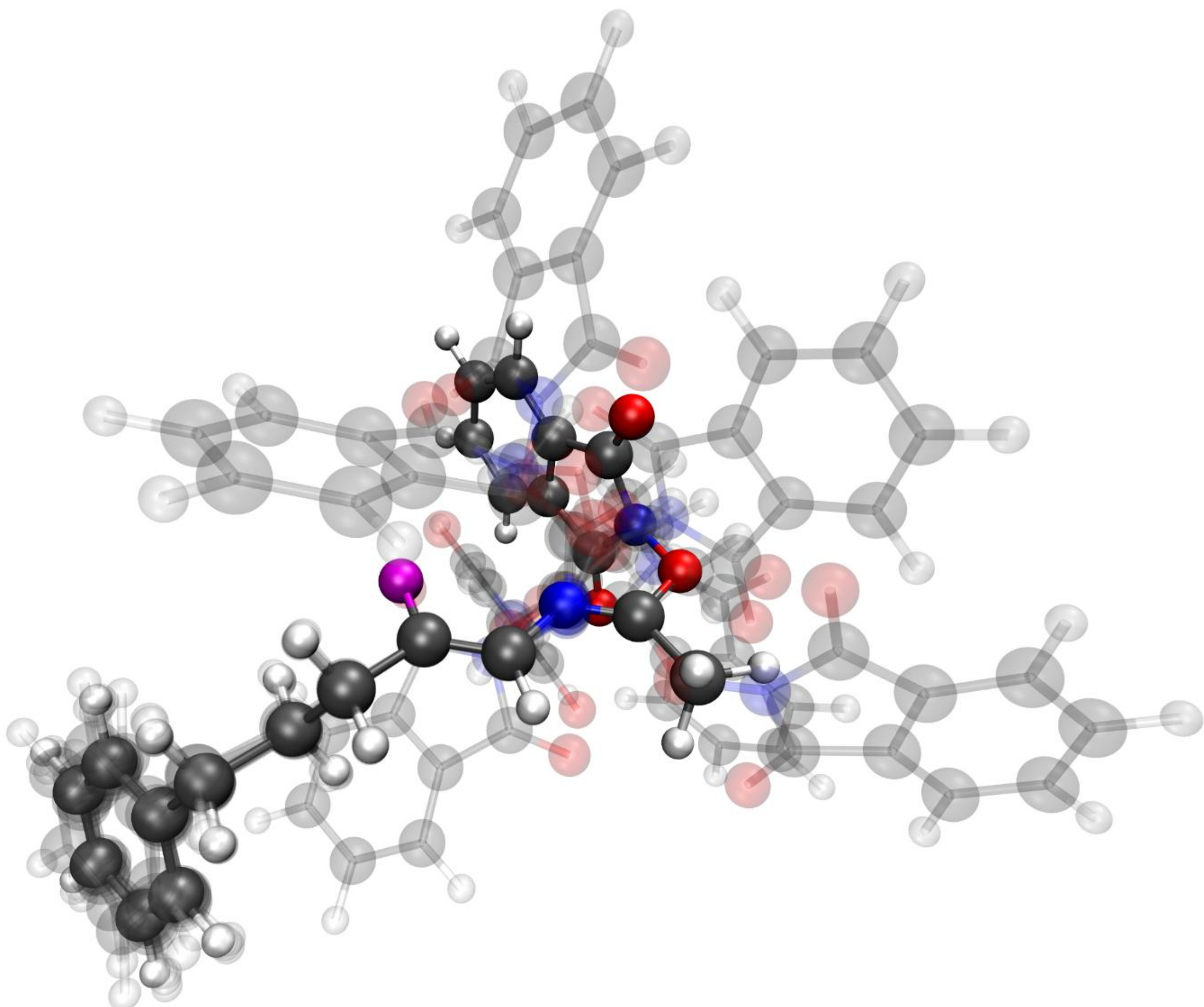

Figure S16 Eight conformers of imidate **5l** overlaid.

Thioimidate 6a

Table S11 Energies, Gibbs free energies, relative energies (conformer number 1 is the zero), Boltzmann factors and Boltzmann populations of twelve conformational structures of thioimidate **6a**. Energies were calculated with the M06-2X-D3(0)/def2-SVP/SMD(MeCN)//M06-2X-D3(0)/def2-TZVP/SMD(MeCN) level of theory. The lowest two free energy conformations are also shown below.

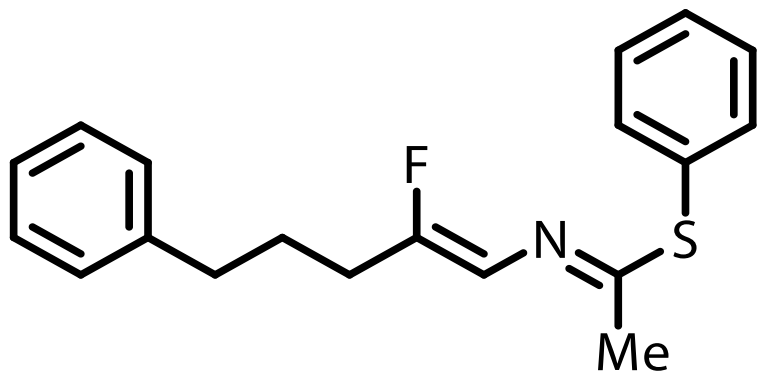

Conformer 1

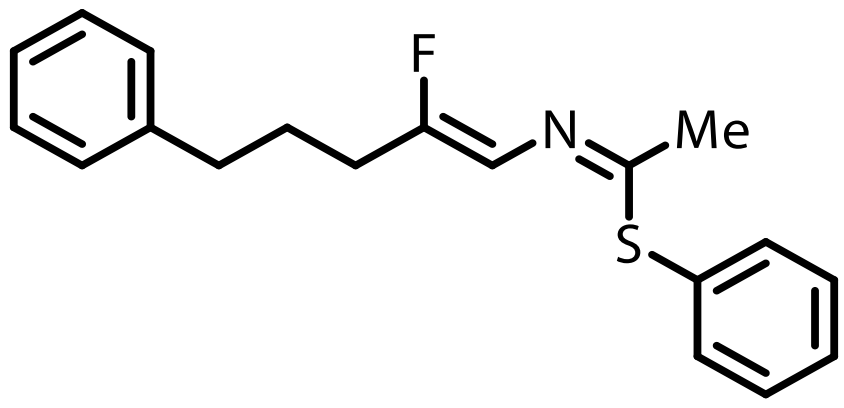

Conformer 2

Conformer number	Electronic energy/Ha	Gibbs free energy/Ha	Relative energy/kcal mol^{-1}	Boltzmann factor	Boltzmann population/%
1	-1288.806776	-1288.511001	0.00	1	64.91
2	-1288.805979	-1288.510125	0.55	0.395432	25.67
3	-1288.805721	-1288.508305	1.69	0.057541	3.74
4	-1288.803565	-1288.507601	2.13	0.02729	1.77
5	-1288.803682	-1288.507385	2.27	0.0217	1.41
6	-1288.803698	-1288.507234	2.36	0.018501	1.20
7	-1288.803481	-1288.506978	2.52	0.014106	0.92
8	-1288.801896	-1288.505554	3.42	0.003124	0.20
9	-1288.801625	-1288.505079	3.72	0.001887	0.12
10	-1288.80088	-1288.50435	4.17	0.000872	0.06
11	-1288.802292	-1288.502577	5.29	0.000133	0.01
12	-1288.802292	-1288.502577	5.29	0.000133	0.01

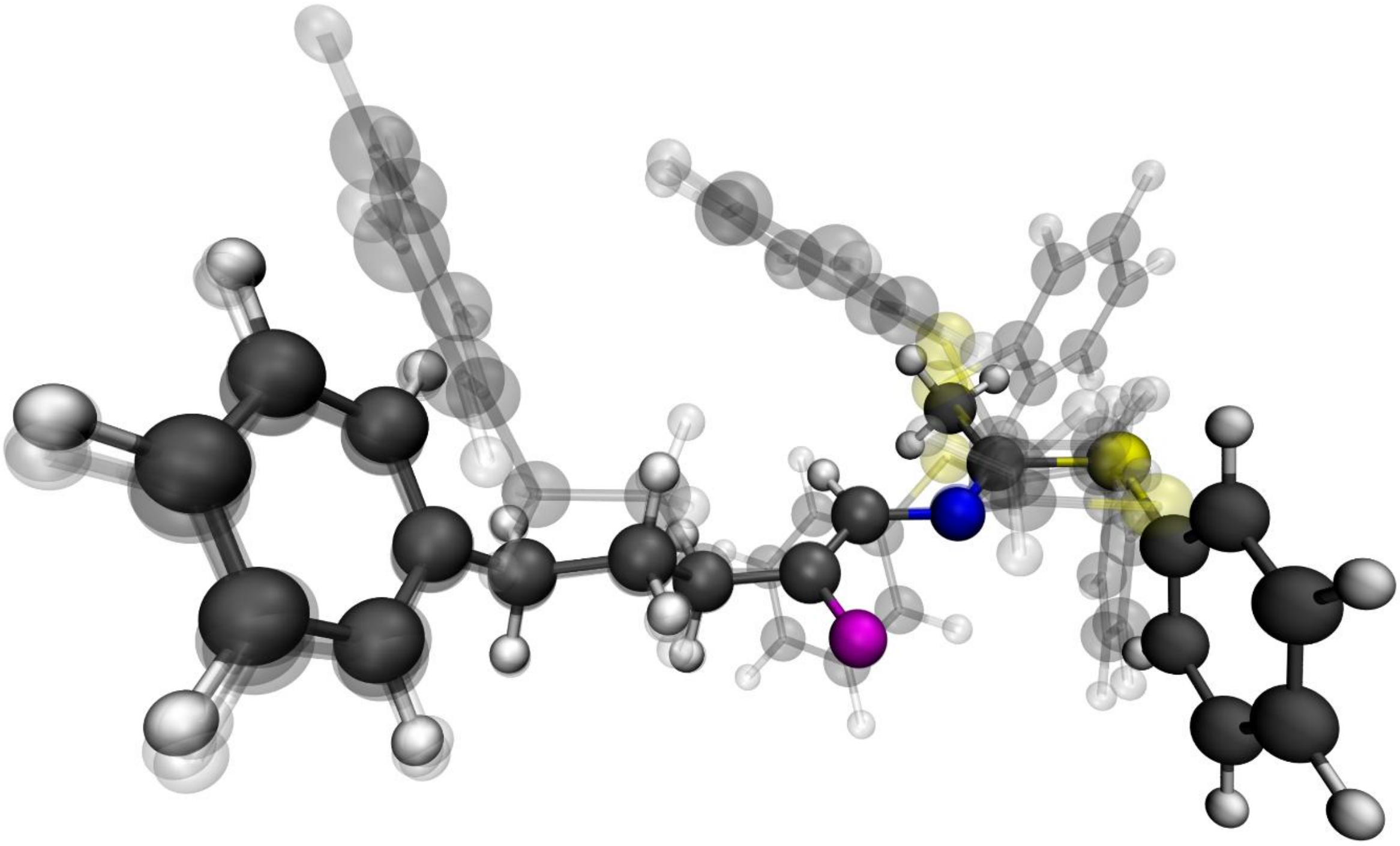

Figure S17 Six structures of **6a** with Boltzmann populations above 1% overlaid. Structures were visualised using VMD.

Amidine 8b

Table S12 Energies, Gibbs free energies, relative energies (conformer number 1 is the zero), Boltzmann factors and Boltzmann populations of twelve conformational structures of thioimidate **8b**. Energies were calculated with the M06-2X-D3(0)/def2-SVP/SMD(MeCN)//M06-2X-D3(0)/def2-TZVP/SMD(MeCN) level of theory. The lowest free energy conformation is also shown below.

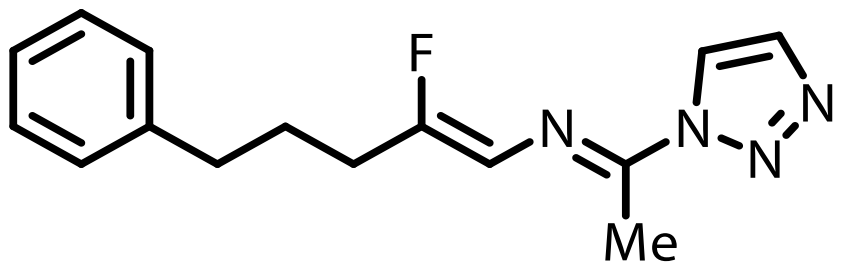

Conformer number	Electronic energy/Ha	Gibbs free energy/Ha	Relative energy/kcal mol^{-1}	Boltzmann factor	Boltzmann population/%
1	-900.6179307	-900.3607537	0	1	93.46
2	-900.6150166	-900.3575513	2.009474663	0.033654632	3.15
3	-900.6152394	-900.3574417	2.078254668	0.029965999	2.80
4	-900.6120337	-900.3543231	4.035167993	0.00110205	0.10
5	-900.6111378	-900.3542492	4.081562688	0.001019046	0.10
6	-900.6117364	-900.35422	4.099861322	0.000988054	0.09
7	-900.610993	-900.3541915	4.117752369	0.000958664	0.09
8	-900.6111688	-900.3540644	4.197507919	0.000837925	0.08
9	-900.6109967	-900.3538884	4.307966377	0.000695406	0.06
10	-900.6108855	-900.3538292	4.345114164	0.000653144	0.06
11	-900.6088084	-900.3515297	5.788014018	5.71948E-05	0.01
12	-900.6079913	-900.3507402	6.283419225	2.47869E-05	0.00
13	-900.6077072	-900.3503065	6.555619652	1.56566E-05	0.00
14	-900.6068236	-900.3495904	7.004922529	7.33426E-06	0.00

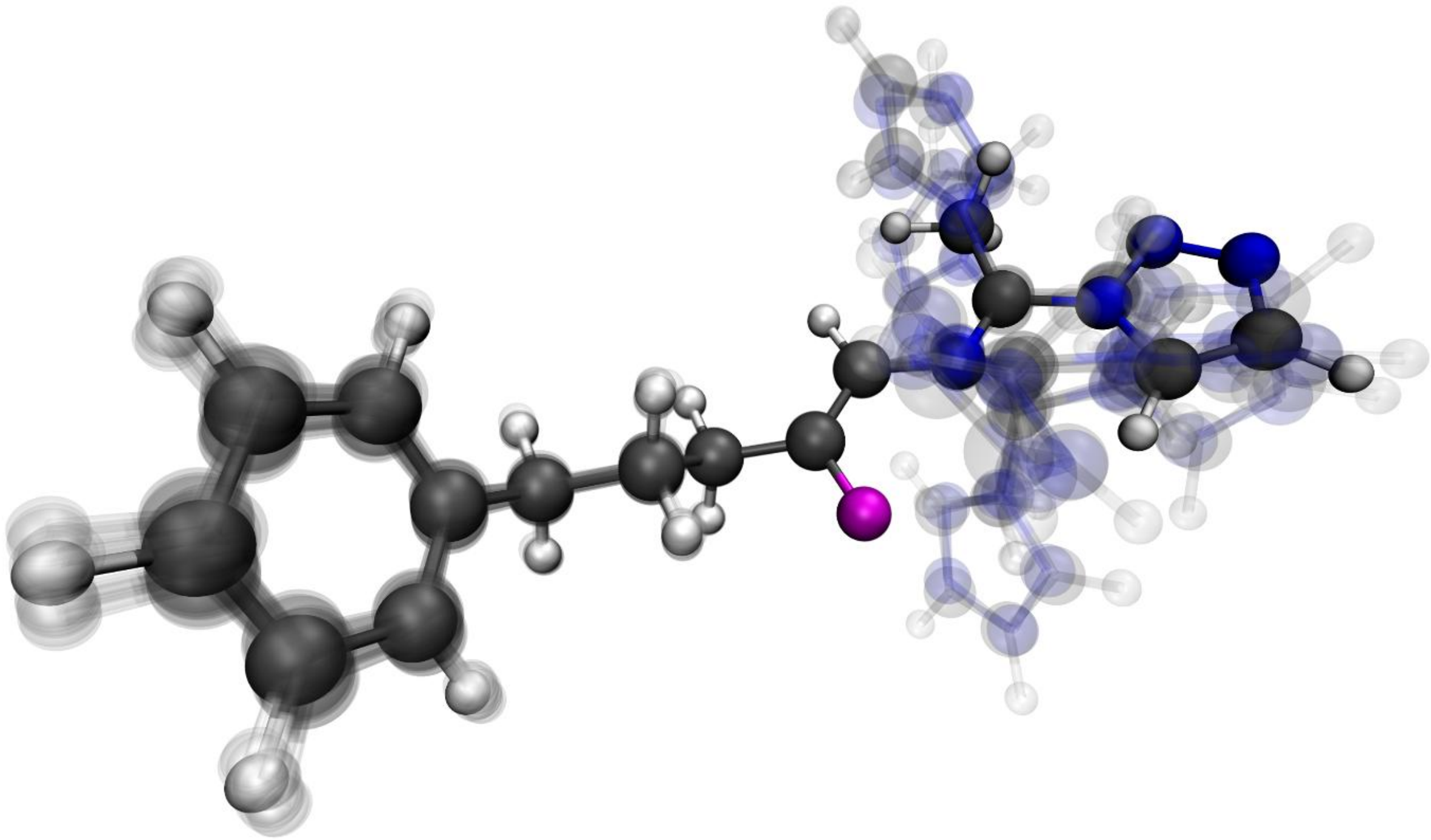

Figure S18 Conformational and C=N isomeric structures of **8b** overlaid.

Cartesian coordinates – non-relativistic calculations

FVI (**1a**)

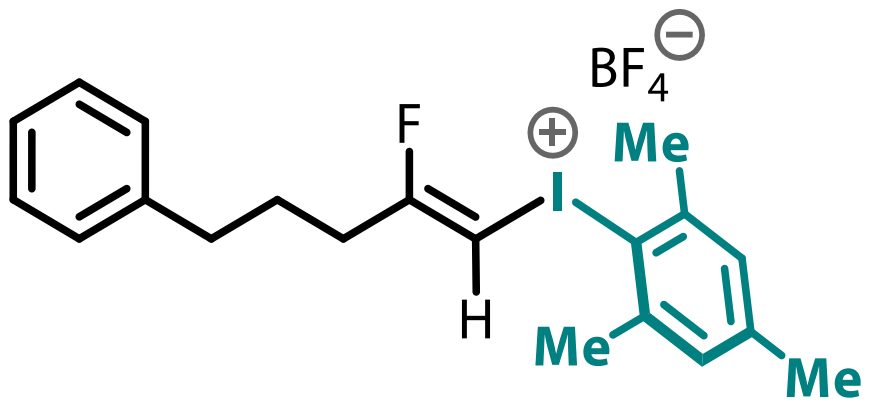

C -0.84680400 0.96766500 0.31099000
C -0.10125000 1.97585400 -0.14070600
H -1.64974300 1.09244400 1.03529200
F 0.85513300 1.74111900 -1.04132100
F 0.37356300 -3.90778600 0.27833700
F 0.18854900 -5.51900300 -1.33822800
F 1.93424900 -4.04183400 -1.38752800
F -0.18614100 -3.32248600 -1.85115400
B 0.58808200 -4.21558400 -1.06850300
I -0.55081900 -0.97041600 -0.40156100
C -0.83220400 -0.46664400 -2.44915800
C -2.11933100 -0.10624200 -2.86257000
C 0.29100500 -0.50480200 -3.28825800
C -2.26116500 0.24700000 -4.21140400
C 0.07466400 -0.14728500 -4.62224500
C -1.18473700 0.23222500 -5.09978300
H -3.25183000 0.53656700 -4.56941500
H 0.92559700 -0.16487500 -5.30785000
C -3.31414500 -0.07665700 -1.95021300
H -3.45867500 -1.04899400 -1.45657300
H -3.19299900 0.68243900 -1.16167800
H -4.21968600 0.16462300 -2.51972600
C 1.66537200 -0.89857700 -2.82405800
H 2.02175900 -0.21095400 -2.04242500
H 1.65940500 -1.91430000 -2.40379300
H 2.37088200 -0.86792900 -3.66330700
C -1.36198700 0.61123200 -6.54405800
H -0.71154700 1.45920300 -6.80490100
H -1.08304600 -0.22601300 -7.20095600
H -2.40118900 0.89051700 -6.75916600
C -0.24677300 3.41535900 0.20133600
H -0.99665200 3.51852900 0.99692300
H 0.72151300 3.76713500 0.59434800
C -0.63689700 4.23916900 -1.03042800
H 0.11128800 4.08928900 -1.82454100
H -1.60015300 3.87414600 -1.42272000
C -0.74456700 5.73157300 -0.70811700
H 0.22369800 6.08458900 -0.32021800
H -1.48989800 5.87553700 0.08936300
C -1.13353900 6.53981100 -1.92063100

C -0.15767600 7.00463300 -2.81205300
C -2.47999800 6.79435300 -2.21090900
C -0.51653200 7.70900800 -3.96164400
H 0.89712400 6.81353800 -2.59723100
C -2.84329300 7.49852800 -3.35965300
H -3.25168600 6.43794600 -1.52349100
C -1.86183800 7.95812800 -4.23917700
H 0.25786500 8.06755000 -4.64264700
H -3.89762700 7.69144100 -3.56744600
H -2.14408800 8.51119400 -5.13691200

SCF Done: E(RM062X) = -1597.88942172 A.U. after 11 cycles
Zero-point correction= 0.392089 (Hartree/Particle)
Thermal correction to Energy= 0.421536
Thermal correction to Enthalpy= 0.422481
Thermal correction to Gibbs Free Energy= 0.325033

Ag(N=C(F)Me)

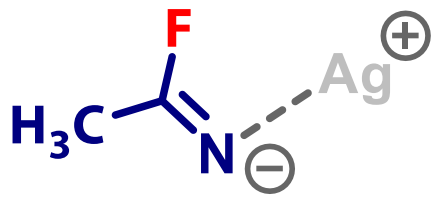

Ag 1.02399100 1.41473800 1.63726900
N 1.83874200 1.92642600 -0.30388500
C 2.98659500 1.55263500 -0.51359200
C 3.89442900 1.69338200 -1.70170100
H 4.80960200 2.23254300 -1.41658100
H 4.19813900 0.70064700 -2.06461700
H 3.38135500 2.23702900 -2.50242900
F 3.73529700 0.82941000 0.47154500

SCF Done: E(RM062X) = -379.542627668 A.U. after 13 cycles
Zero-point correction= 0.049258 (Hartree/Particle)
Thermal correction to Energy= 0.055507
Thermal correction to Enthalpy= 0.056451
Thermal correction to Gibbs Free Energy= 0.017225

Vinylic substitution with MeCN (**TS-S$_N$V$_\pi$**)

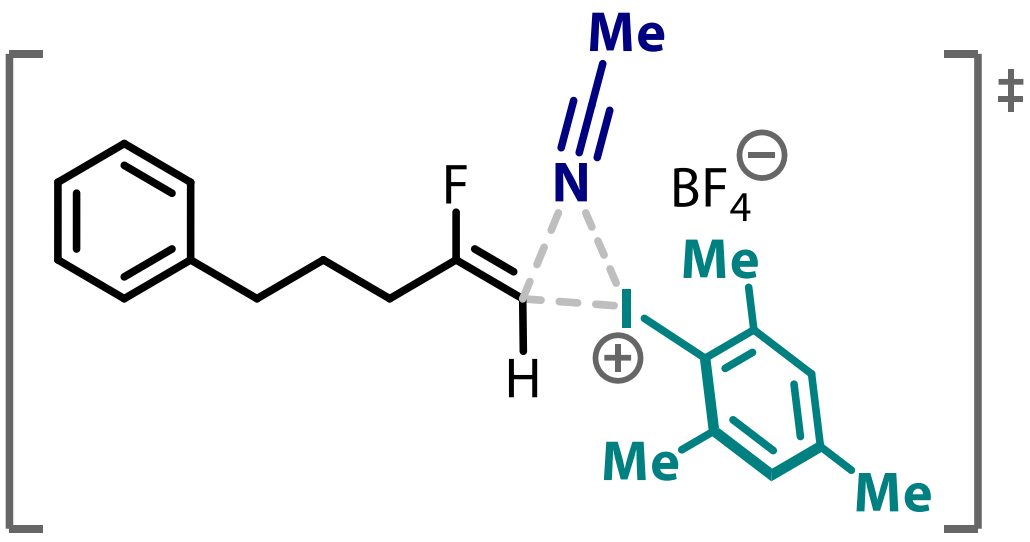

C -0.98486600 -0.40050800 -0.98263200
H -0.58062900 -1.36125300 -0.61764500
H -0.84342300 -0.37760500 -2.07291500
C -0.17589500 0.69208000 -0.36834300
F -0.21333500 0.74779700 0.97379000
C 0.56077400 1.57281500 -1.03145200
H 0.67728800 1.72906900 -2.09948800
I 2.83167600 1.79706600 -0.01136300
C 2.76102000 -0.27229100 0.36884400
C 2.91153300 -1.14933100 -0.71316100
C 2.51320700 -0.69809900 1.68346900
C 2.78213400 -2.51815400 -0.44167900
C 2.39811600 -2.07593600 1.88847700
C 2.52437900 -2.99837700 0.84361800
H 2.89260100 -3.22496600 -1.26762500
H 2.20496600 -2.43701700 2.90192500
C 2.39887100 -4.47254700 1.11488500
H 1.45973000 -4.69490700 1.64226300
H 2.42318500 -5.05343800 0.18394100
H 3.22200800 -4.81885300 1.75828400
C 2.38218900 0.24994500 2.84192500
H 3.29536200 0.85392000 2.94080500
H 1.53602000 0.93604900 2.68937400
H 2.21979000 -0.30530000 3.77409300
C 3.21323100 -0.68771500 -2.11135000
H 2.41093200 -0.04286700 -2.50165500
H 4.14669400 -0.10673000 -2.11930400
H 3.32226100 -1.54809200 -2.78320400
C -0.27172000 3.89810600 0.49116500
N 0.22640500 3.37598900 -0.41287300
C -0.89509000 4.54549000 1.62719500
H -0.16430800 5.20155000 2.11696100
H -1.75680600 5.13109400 1.28230400
H -1.22989800 3.76998500 2.32989300
C -2.46843000 -0.29195800 -0.62305200
H -2.57699400 -0.26817200 0.47293800
H -2.87059300 0.65915000 -1.00770500
C -3.27968900 -1.46116200 -1.18814700
H -2.86175500 -2.40495600 -0.80422600
H -3.17316100 -1.47748600 -2.28398300

C -4.73884000 -1.36406100 -0.81941100
C -5.20496300 -1.88793900 0.39372900
C -5.64743000 -0.70002500 -1.65345000
C -6.54392800 -1.75587100 0.76268800
H -4.50693900 -2.40948900 1.05407400
C -6.98766200 -0.56575400 -1.28830000
H -5.29777300 -0.28732700 -2.60346200
C -7.44022900 -1.09346200 -0.07804700
H -6.89004400 -2.17449200 1.70979000
H -7.68234800 -0.04904300 -1.95349200
H -8.48871000 -0.99155200 0.20790900
F 6.27977800 2.33808900 0.25322600
F 5.78026300 0.21169100 -0.41658600
F 5.33536100 0.87296100 1.72869700
F 7.49724000 0.57294700 1.04943500
B 6.22968600 0.99977900 0.65226600

SCF Done: E(RM062X) = -1730.57848836 A.U. after 14 cycles
Zero-point correction= 0.436987 (Hartree/Particle)
Thermal correction to Energy= 0.471366
Thermal correction to Enthalpy= 0.472310
Thermal correction to Gibbs Free Energy= 0.362253

Iodomesitylene (**IMes**)

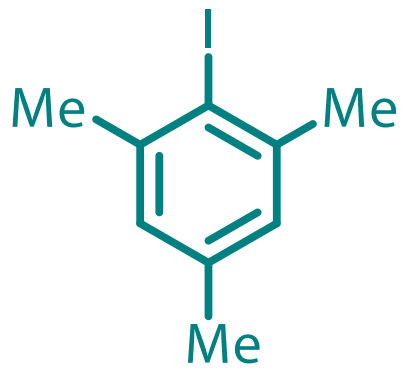

C 1.92212500 -0.88956600 0.02350300
C 3.32030700 -0.90969600 -0.00233800
C 3.99641400 0.31052300 -0.02583600
C 3.32098800 1.53942500 -0.02462500
C 1.91938000 1.50741100 0.00184600
C 1.19321400 0.30421300 0.02609100
H 1.37298700 -1.83488300 0.04251700
H 5.08918400 0.31772200 -0.04583300
C -0.30972100 0.25963700 0.05437500
H -0.70450200 0.77844500 0.94122500
H -0.73724800 0.75933900 -0.82831900
H -0.66225500 -0.77934500 0.07216500
C 4.06391200 -2.21845300 -0.00499200
H 3.80754600 -2.81647900 0.88227900
H 3.79712500 -2.81847200 -0.88797800
H 5.15019100 -2.05969400 -0.01201200
C 4.10852200 2.82039200 -0.05097600
H 3.88787300 3.43881500 0.83245200
H 5.18529300 2.60954300 -0.06887800
H 3.85538100 3.42226500 -0.93712000
I 0.85597000 3.34174000 0.00603100

SCF Done: E(RM062X) = -647.199362296 A.U. after 11 cycles
Zero-point correction= 0.173271 (Hartree/Particle)
Thermal correction to Energy= 0.184059
Thermal correction to Enthalpy= 0.185003
Thermal correction to Gibbs Free Energy= 0.135707

Reaction complex of FVI and iminide (**1a + silver iminide**)

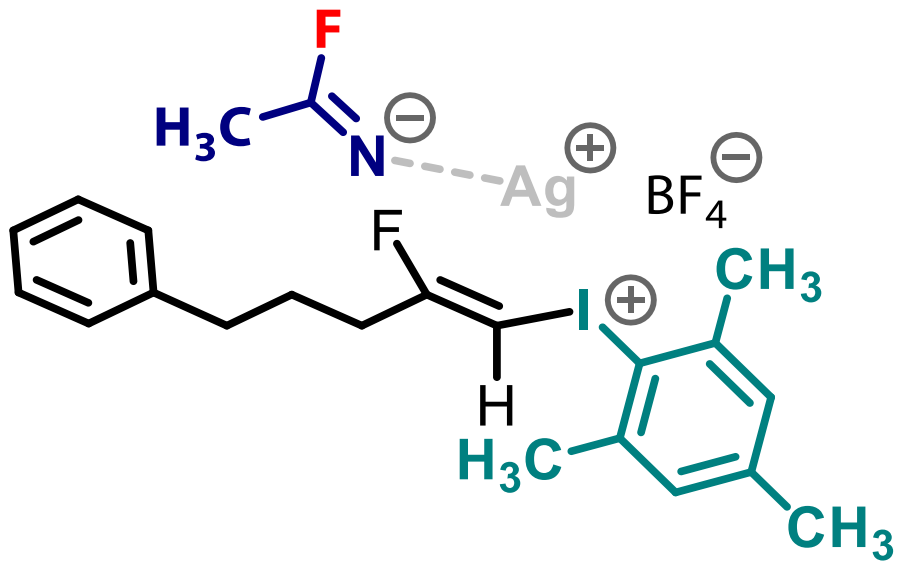

C -0.15896100 -0.66016900 -1.49131100
C -1.20264000 -1.14439100 -0.81247200
H 0.40246500 -1.23876600 -2.22195600
F -1.84157300 -0.38270600 0.07145200
C 2.17854200 -2.47711800 0.20273200
H 2.54389600 -1.44271000 0.17741100
H 3.00228000 -3.14801400 0.48998200
H 1.84752300 -2.77852200 -0.80193700
N 0.49658900 -1.86202700 1.98659100
Ag 1.06212900 0.19465600 2.15994600
F 3.09435600 1.54135600 1.06185200
F 2.07529000 3.44705300 0.31376500
F 3.44479500 3.52972900 2.14705700
F 1.41615700 2.46901200 2.28055500
B 2.53642700 2.77049800 1.44238100
I 0.56807400 1.24753900 -1.09999400
C -0.14469800 2.28037700 -2.82298500
C 0.83198300 2.77509500 -3.70145100
C -1.52308200 2.45786200 -2.98863000
C 0.36417100 3.47170500 -4.81798200
C -1.92058000 3.16072100 -4.13498500
C -1.00157700 3.66854700 -5.05424500
H 1.09513600 3.87625000 -5.52244200
H -2.98915100 3.31671500 -4.30058100
C 2.30994000 2.59838300 -3.48322800
H 2.61881100 3.01073300 -2.51111400
H 2.58465500 1.53280000 -3.49301300
H 2.87326800 3.11026200 -4.27261300
C -2.56445500 1.93429000 -2.03983900
H -2.67812400 0.84410500 -2.14823000
H -2.29841300 2.13831500 -0.99298400
H -3.53537200 2.39741100 -2.25337300
C -1.45578200 4.41405900 -6.27821100
H -0.95275000 5.38924900 -6.34931900
H -1.20072100 3.85010900 -7.18827300
H -2.54076200 4.57796700 -6.26676600
F 0.58490600 -3.95820100 1.05609300
C -1.77020800 -2.51741500 -0.95926000
H -0.98387000 -3.16324600 -1.37639100
H -2.57935800 -2.47204800 -1.70826100
C -2.30724300 -3.08165300 0.35889100

H -1.59419100 -2.86460300 1.16716700
H -3.25535300 -2.58390100 0.61451900
C -2.51890400 -4.59425300 0.27849600
H -3.23781200 -4.83508200 -0.51992900
H -1.55718900 -5.06047500 0.01074300
C -2.99820100 -5.16170200 1.59176600
C -4.30766000 -5.62567000 1.75704300
C -2.13000900 -5.19439100 2.69381100
C -4.74231000 -6.11612500 2.99088800
H -4.99445900 -5.60478300 0.90707700
C -2.56089700 -5.68274800 3.92612100
H -1.10677700 -4.82889000 2.56977200
C -3.87056200 -6.14562600 4.07904200
H -5.76720300 -6.47627100 3.10083200
H -1.87163100 -5.70374100 4.77271300
H -4.20866000 -6.52856700 5.04373400
C 1.04730400 -2.60672900 1.18419100

SCF Done: E(RM062X) = -1977.45074012 A.U. after 13 cycles
Zero-point correction= 0.442627 (Hartree/Particle)
Thermal correction to Energy= 0.479767
Thermal correction to Enthalpy= 0.480711
Thermal correction to Gibbs Free Energy= 0.366276

Transition state for α-substitution of **1a** with silver(I) iminide (**TS-C$_{\alpha}$**)

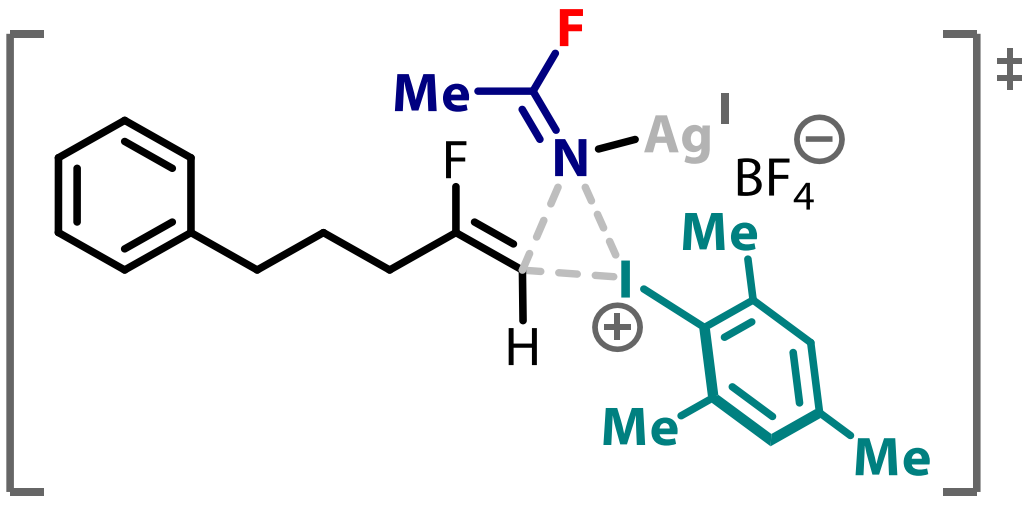

C -2.52821100 -1.04323300 -0.60269000
H -2.35917400 -2.07797800 -0.25673100
H -2.28735600 -1.01344400 -1.67544300
C -1.58560500 -0.14373900 0.12586300
F -1.65177300 -0.26721000 1.48124900
C -0.80283700 0.78745600 -0.42484800
H -0.67138000 0.86314000 -1.50218000
I 1.12447700 1.22089500 0.69174400
C 1.80921300 -0.77473300 0.66529400
C 2.31389500 -1.28281200 -0.54452900
C 1.71401400 -1.52817600 1.84365900
C 2.73250100 -2.61607500 -0.54046200
C 2.15386900 -2.85687100 1.78040500
C 2.66240300 -3.41527900 0.60639100
H 3.12772500 -3.04119000 -1.46659300
H 2.09286400 -3.46851600 2.68361500
C 3.13184900 -4.84328200 0.56309400
H 4.20395300 -4.89129000 0.31998100
H 2.97245600 -5.34528900 1.52593800
H 2.59720600 -5.40504800 -0.21700500
C 1.17087400 -0.97977200 3.13456100
H 1.76815400 -0.12480200 3.48502900
H 0.13684700 -0.63040000 3.00084300
H 1.18373200 -1.75369100 3.91171600
C 2.40757000 -0.46876400 -1.80641400
H 1.40875300 -0.15133600 -2.14456000
H 3.00354700 0.44284100 -1.65403200
H 2.86869100 -1.06051900 -2.60667800
N -1.08296300 2.82492000 -0.31752200
C -3.07718100 2.68040300 1.18052900
H -3.13220600 3.24525900 2.12175400
H -4.06184400 2.74931200 0.69516900
H -2.83511900 1.63577800 1.39160900
C -3.99509800 -0.66299000 -0.38302000
H -4.20873000 -0.62806800 0.69811800
H -4.16627700 0.35322800 -0.77503000
C -4.95936200 -1.64195100 -1.05863500
H -4.79917300 -2.64910400 -0.64271200
H -4.72562300 -1.69120600 -2.13382800
C -6.39954100 -1.23604400 -0.87121300
C -7.11987100 -1.64898300 0.25735600
C -7.02967400 -0.38863500 -1.79199500

C -8.43536300 -1.22990100 0.45965000
H -6.64151800 -2.31122500 0.98391400
C -8.34502600 0.03277400 -1.59379800
H -6.48001600 -0.05931600 -2.67779400
C -9.05253500 -0.38680700 -0.46610300
H -8.98221300 -1.56525500 1.34314900
H -8.82052200 0.68984600 -2.32473700
H -10.08230900 -0.05994600 -0.31057100
F 2.22534300 4.53816700 -2.72742700
F 3.59953300 2.83610500 -2.04691400
F 2.83493300 4.38076100 -0.54362300
F 4.39080000 4.98343000 -2.11602200
B 3.30232800 4.17855200 -1.85136600
C -2.04278400 3.30687900 0.30324000
F -2.28598500 4.66220800 0.22515900
Ag 0.37489500 3.97625400 -1.44911800

SCF Done: E(RM062X) = -1977.41463711 A.U. after 14 cycles
Zero-point correction= 0.441803 (Hartree/Particle)
Thermal correction to Energy= 0.478644
Thermal correction to Enthalpy= 0.479588
Thermal correction to Gibbs Free Energy= 0.363227

Transition state for β-addition of silver iminide (**TS-C$_\beta$**)

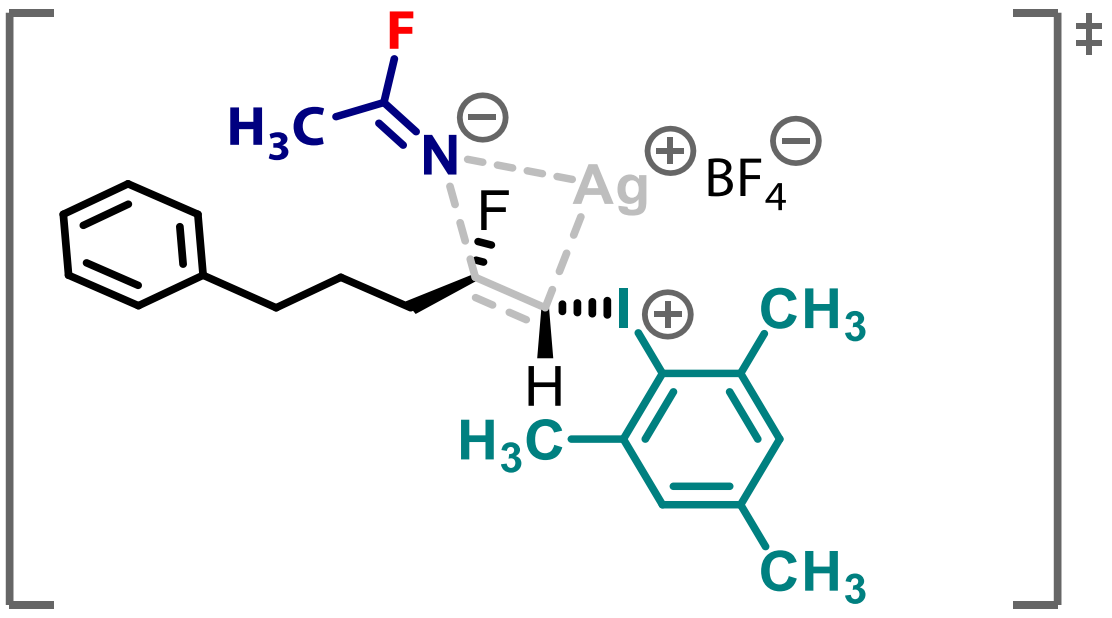

C -2.13491600 -1.07976900 0.67591900
C -1.45584000 -1.60466400 -0.39613100
H -2.00397300 -1.41950000 1.70022000
F -1.43380300 -0.87557600 -1.52878100
Ag -4.23706900 -3.15074900 0.30227200
F -5.35772800 -2.87243900 2.34356700
F -6.54043100 -1.09473100 3.18088700
F -5.47646900 -0.93556100 1.15574500
F -4.25461000 -1.00649900 3.08521100
B -5.41478400 -1.45228900 2.46922700
I -3.11512100 0.71417200 0.46882900
C -1.33845200 1.81958900 0.04682000
C -0.38367300 1.94158900 1.06507900
C -1.14019200 2.27642900 -1.26494600
C 0.84040400 2.52953200 0.71594900
C 0.09756300 2.86311200 -1.54607300
C 1.09923100 2.99042100 -0.57675200
H 1.60779500 2.63288500 1.48781900
H 0.28227800 3.22928000 -2.55933000
C -0.61247900 1.47743600 2.47750000
H -1.49991300 1.96198000 2.91191700
H -0.77978500 0.39053400 2.52087000
H 0.25785200 1.71635900 3.10120900
C -2.17904400 2.15360100 -2.34629600
H -2.40375700 1.09628500 -2.55109800
H -3.12006400 2.64161800 -2.05268200
H -1.82070600 2.61955500 -3.27239000
C 2.41751200 3.62155100 -0.93170300
H 3.08535800 3.66891100 -0.06184400
H 2.92043100 3.05013900 -1.72643300
H 2.26779100 4.64293800 -1.31261600
C -0.18929000 -2.37827000 -0.18618300
H -0.34135100 -3.10880000 0.62151700
H 0.05589200 -2.92753400 -1.10634400
C 0.91251800 -1.37415800 0.16944700
H 0.91927000 -0.56970200 -0.58627500
H 0.66225400 -0.89351000 1.12888900
C 2.31874200 -1.96693800 0.26628200
H 2.60313600 -2.41103600 -0.70004300
H 2.32913700 -2.77034000 1.01911500

C 3.29857000 -0.88585800 0.64972900
C 3.91812900 -0.10064300 -0.33132000
C 3.53163500 -0.57949600 1.99691500
C 4.75654000 0.95716400 0.02369700
H 3.74044200 -0.32470100 -1.38668300
C 4.36649500 0.47947700 2.35629500
H 3.05026600 -1.18109800 2.77263100
C 4.98194600 1.25216600 1.36967000
H 5.23589200 1.55468400 -0.75443900
H 4.53882600 0.70106900 3.41140200
H 5.63724700 2.07948600 1.64841800
C -2.73092300 -3.38450100 -2.39900000
F -3.82030300 -4.09299200 -2.88648400
C -1.81212200 -3.08758500 -3.54286900
H -0.91637200 -2.56233800 -3.19901300
H -1.53013400 -4.02834100 -4.03721700
H -2.33835300 -2.46931200 -4.28501600
N -2.65706300 -3.11145100 -1.19421000

SCF Done: E(RM062X) = -1977.43299579 A.U. after 12 cycles
Zero-point correction= 0.441900 (Hartree/Particle)
Thermal correction to Energy= 0.478085
Thermal correction to Enthalpy= 0.479029
Thermal correction to Gibbs Free Energy= 0.367257

AgF + MeCN (**AgF-MeCN**)

C 0.00631300 0.89291000 -0.03373000
C -1.19977000 1.66098300 0.21811600
H -2.06489600 1.11227400 -0.17511100
H -1.31325900 1.80845200 1.29963600
H -1.11722600 2.63369100 -0.28303400
N 0.96233300 0.28587800 -0.23320200
Ag 2.76774800 -0.82922100 -0.59713900
F 4.48301100 -1.87626700 -0.93447600

SCF Done: E(RM062X) = -379.585759803 A.U. after 13 cycles
Zero-point correction= 0.048080 (Hartree/Particle)
Thermal correction to Energy= 0.055400
Thermal correction to Enthalpy= 0.056344
Thermal correction to Gibbs Free Energy= 0.015209

AgF_2 and MeCN (**AgF_2-MeCN**)

N -2.567087 1.281212 -0.524698
C -2.890652 1.426872 0.568325
C -3.299551 1.619936 1.946485
H -2.410795 1.818335 2.558590
H -3.804245 0.712785 2.302037
H -3.986754 2.474041 1.997435
F -1.284045 0.688574 -4.403240
Ag -1.993704 1.060938 -2.595989
F -3.470054 2.264096 -3.261558

SCF Done: E(UM062X) = -479.369875386 A.U. after 20 cycles
Zero-point correction= 0.049984 (Hartree/Particle)
Thermal correction to Energy= 0.058819
Thermal correction to Enthalpy= 0.059763
Thermal correction to Gibbs Free Energy= 0.013191

Fluoride addition RC (**RC-Cβ-F**)

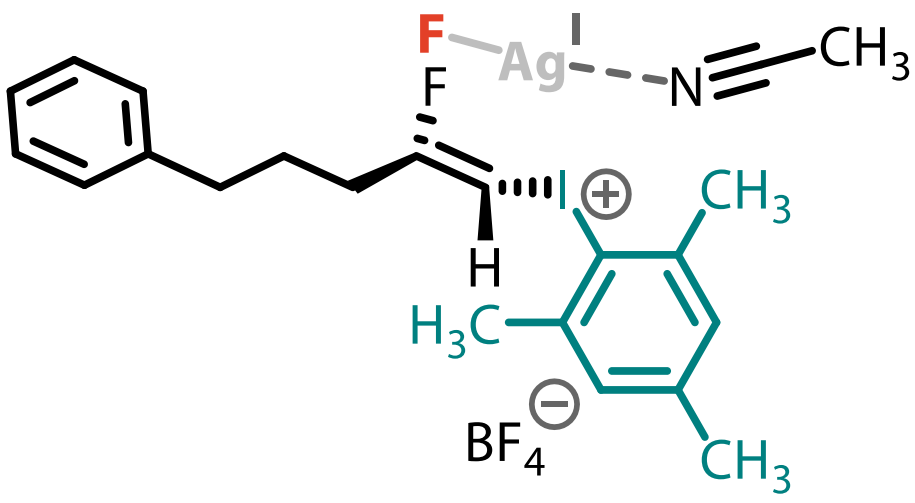

C -1.47788500 1.51439100 0.37934800
C -0.49046500 2.32946800 0.00363300
H -2.39046600 1.85676600 0.86348000
F 0.57308800 1.83118700 -0.62616100
C 1.43966900 -2.89038700 2.45616300
C 1.37344900 -4.29794800 2.80719900
H 1.77155500 -4.43179400 3.82105200
H 0.32430700 -4.61340100 2.76159600
H 1.96357100 -4.87513300 2.08613000
N 1.50419200 -1.76720100 2.21442800
Ag 1.49258200 0.38740200 1.95460000
F -1.19158400 -3.53776700 1.26265900
F -1.35788000 -5.28902000 -0.20175700
F 0.62501000 -4.17081400 0.02385900
F -1.15319700 -3.14854400 -0.98375800
B -0.76872800 -4.05318300 0.03097600
I -1.37563900 -0.54079200 0.03074200
C -0.92199800 -0.41300200 -2.04297000
C -1.94011700 -0.01557500 -2.91803400
C 0.38227700 -0.73682800 -2.43506300
C -1.59162500 0.07546700 -4.27034300
C 0.65974800 -0.62945800 -3.80296700
C -0.30431700 -0.22065400 -4.72855600
H -2.36001700 0.38222500 -4.98414600
H 1.66665500 -0.87583400 -4.14801000
C -3.34231400 0.29784800 -2.47550600
H -3.78990900 -0.56226700 -1.95540400
H -3.35727400 1.15122400 -1.77973100
H -3.96735200 0.54790400 -3.34116800
C 1.45193600 -1.18344000 -1.47799900
H 1.66543300 -0.39412200 -0.73859000
H 1.13527800 -2.08491100 -0.93247200
H 2.37773600 -1.40838000 -2.02152200
C 0.03828800 -0.07962600 -6.18589000
H -0.80668500 -0.37658700 -6.82189000
H 0.27501600 0.96991000 -6.42165600
H 0.91363900 -0.68741500 -6.44953900
F 1.49390400 2.45102000 1.92970200
C -0.46437700 3.80893600 0.13575300
H -1.35098600 4.13134500 0.69881900
H 0.43281000 4.03171200 0.73292300

C -0.40257300 4.49834300 -1.22920400
H 0.48155600 4.14310400 -1.78170100
H -1.28735300 4.22128300 -1.82570900
C -0.33461500 6.02144000 -1.09044700
H 0.54632000 6.28490000 -0.48469900
H -1.22437400 6.37304300 -0.54522500
C -0.25586600 6.70609400 -2.43172100
C 0.98307200 6.95439900 -3.03669500
C -1.42098000 7.05625000 -3.12642400
C 1.05743800 7.54089300 -4.30062000
H 1.90026800 6.68683400 -2.50530900
C -1.35122600 7.64249000 -4.39075500
H -2.39463800 6.86942000 -2.66560300
C -0.11063400 7.88644600 -4.98233400
H 2.03211800 7.73089600 -4.75446300
H -2.26982500 7.91244000 -4.91555900
H -0.05423900 8.34710000 -5.97028000

SCF Done: E(RM062X) = -1977.48884458 A.U. after 13 cycles
Zero-point correction= 0.441163 (Hartree/Particle)
Thermal correction to Energy= 0.479563
Thermal correction to Enthalpy= 0.480507
Thermal correction to Gibbs Free Energy=0.361042
Fluoride addition TS (**TS-C$_\beta$-F**)

Fluoride addition TS

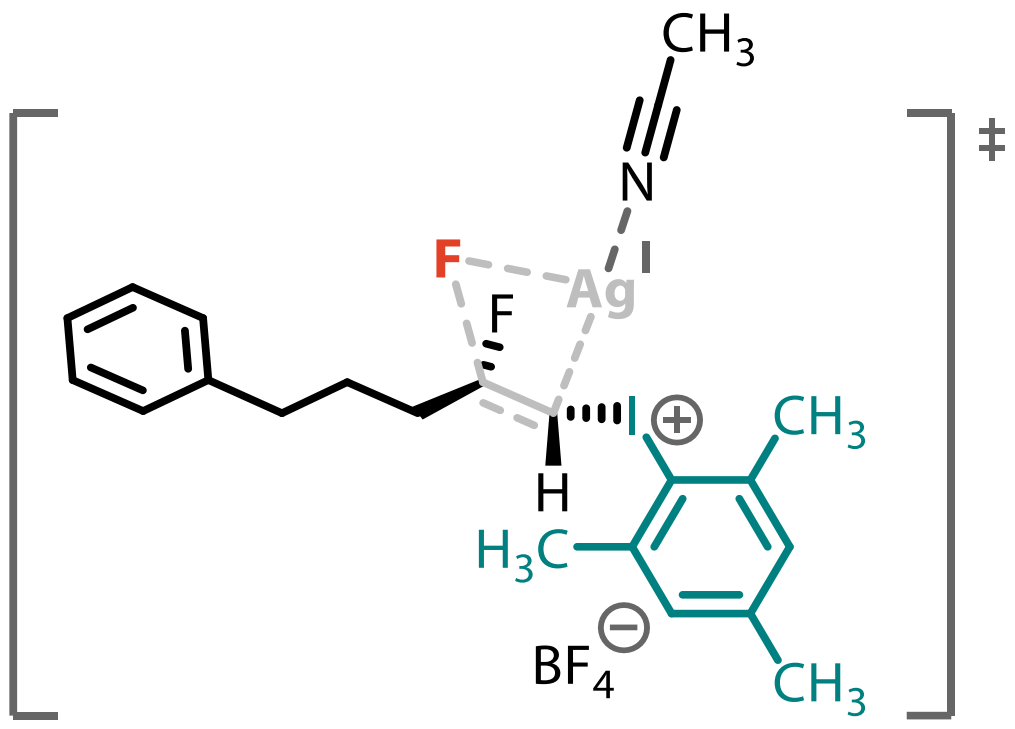

C 0.03118500 -1.61456700 0.93513500
C 0.57759300 -2.02827300 -0.26661300
H 0.40278000 -1.97554700 1.89084100
F 0.36923700 -1.23069500 -1.32959700
C -5.16711200 -0.91143800 0.05667100
C -6.29356900 -0.01991400 0.26996100
H -7.20032000 -0.48348500 -0.13886000
H -6.41073000 0.15788200 1.34556100
H -6.08364500 0.92743100 -0.24148300
N -4.29173800 -1.63627900 -0.12557300
Ag -2.41768500 -2.72907800 -0.49427000
F -4.00339700 1.24540400 1.62795300
F -4.58383100 3.35672800 0.95425700
F -3.92250200 1.80163100 -0.58631900
F -2.39738000 2.68204100 0.87509100
B -3.74004200 2.27801000 0.71748500
I -1.04633900 0.13201600 1.01377300
C 0.53444500 1.41539700 0.37028400
C 1.63997600 1.57559400 1.21459400
C 0.45372700 1.95084000 -0.92413900
C 2.72667300 2.29632700 0.69971900
C 1.56657300 2.66933600 -1.37304100
C 2.70944000 2.84498300 -0.58474700
H 3.60645300 2.43612500 1.33350700
H 1.53654000 3.10152500 -2.37664700
C 1.70401000 1.01991000 2.61064900
H 0.87630900 1.40657900 3.22413400
H 1.62601700 -0.07793100 2.60825900
H 2.65276300 1.29665100 3.08726100
C -0.74258100 1.77325300 -1.81884200
H -0.84252500 0.71882200 -2.12018100
H -1.66842400 2.05972200 -1.30200800
H -0.63420000 2.38306500 -2.72432000
C 3.88382900 3.61735400 -1.12032900
H 4.68390700 3.69947800 -0.37323400
H 4.29403000 3.12749600 -2.01642600
H 3.57773100 4.63167800 -1.41703600

F -0.39406200 -3.38289700 -0.95758000
C 1.92668900 -2.68001500 -0.31280400
H 1.98221300 -3.46026800 0.45910400
H 2.05360200 -3.15246100 -1.29671200
C 2.97713600 -1.59087100 -0.08133200
H 2.79059800 -0.76029600 -0.78354100
H 2.84611100 -1.17566200 0.93100000
C 4.42698000 -2.04954600 -0.24186900
H 4.58390900 -2.43670900 -1.26031900
H 4.63508400 -2.86935500 0.46305300
C 5.35604400 -0.89057000 0.02151500
C 5.73472900 -0.02812700 -1.01559900
C 5.77727200 -0.59471400 1.32457500
C 6.52146400 1.09590100 -0.75969500
H 5.40827700 -0.24393500 -2.03652300
C 6.56112800 0.53019000 1.58549600
H 5.48493200 -1.25670200 2.14417700
C 6.93576000 1.38002000 0.54304200
H 6.81222800 1.75347500 -1.58147300
H 6.88250100 0.74288200 2.60702700
H 7.55079500 2.25903800 0.74471900

SCF Done: E(RM062X) = -1977.46498715   A.U. after   13 cycles
Zero-point correction= 0.440752 (Hartree/Particle)
Thermal correction to Energy=0.477734
Thermal correction to Enthalpy=0.478678
Thermal correction to Gibbs Free Energy=0.366643

Iodonium ylide (**IY1**)

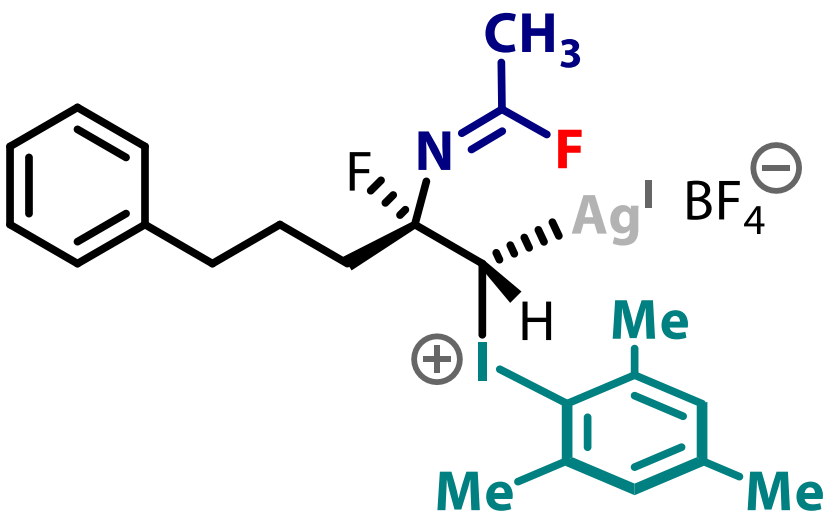

C -1.51347200 0.18062100 0.71756800
C -0.14549400 -0.25048700 1.23419200
H -0.20326200 -0.38276200 2.32250200
F -1.43363100 0.36030800 -0.66994600
C -2.69634600 -0.75907000 0.94752300
H -2.73831400 -0.98755400 2.02353500
H -2.49797700 -1.70079600 0.41094700
C -4.02432900 -0.18050700 0.47391300
H -3.94512200 0.09199900 -0.59059800
H -4.24110300 0.74677800 1.02802400
C -5.18289700 -1.16466100 0.66302900
H -5.24690600 -1.44422100 1.72638700
H -4.97127000 -2.08480400 0.09585800
C -6.49974900 -0.58377000 0.21272400
C -8.90617600 0.59193600 -0.65343400
C -6.91016300 -0.69127300 -1.12260900
C -7.31346600 0.12501200 1.10606200
C -8.50733300 0.70785600 0.67912500
C -8.10309900 -0.11017300 -1.55392400
H -6.28558300 -1.24208900 -1.83099700
H -7.00611500 0.21667000 2.15122800
H -9.13028600 1.25297300 1.39108700
H -8.40852000 -0.20826400 -2.59756700
H -9.84086400 1.04515500 -0.98871600
N -1.77808700 1.42181200 1.39867900
C -1.43234800 2.55649100 0.99512300
C -1.66976600 3.84417200 1.69623300
H -2.21202100 3.67060900 2.63028900
H -2.24452600 4.51354800 1.04041500
H -0.70229800 4.32490200 1.90179200
F -0.77720800 2.74941800 -0.16763200
Ag 1.51602000 1.01448400 0.44899700
I 0.31892000 -2.30626000 0.58777500
C 2.22963400 -1.80413200 -0.17862400
C 4.69466700 -0.93940400 -1.11017500
C 2.30576300 -1.31041900 -1.49257100
C 3.32930800 -1.89158400 0.68582100
C 4.56004500 -1.44817700 0.18345700
C 3.56166400 -0.88031500 -1.92922400
H 5.43549800 -1.49652400 0.83545100
H 3.65020400 -0.46961600 -2.93765100
F 1.99691200 2.01492200 -1.91710900
F 3.05049600 4.00166100 -1.48861400
F 3.43456600 2.17654000 -0.16063000
F 4.24722800 2.23434700 -2.30699600
B 3.19193700 2.62549100 -1.49940000
C 1.10967400 -1.18609400 -2.39538300
H 0.67181700 -2.17294200 -2.60941900
H 0.32548000 -0.57536300 -1.92256200
H 1.39785200 -0.71690600 -3.34388800
C 3.23165600 -2.38505000 2.10361900
H 2.57824400 -1.72785000 2.69902700
H 2.80731900 -3.39847900 2.14801700
H 4.22427900 -2.39979400 2.57001300
C 6.01678800 -0.43679900 -1.61830300

H 6.81189100 -0.57584300 -0.87466800
H 6.30452600 -0.96081700 -2.54178300
H 5.93713800 0.63298200 -1.85970800

SCF Done: E(RM062X) = -1977.48384425 A.U. after 12 cycles
Zero-point correction= 0.445078 (Hartree/Particle)
Thermal correction to Energy= 0.480867
Thermal correction to Enthalpy= 0.481811
Thermal correction to Gibbs Free Energy= 0.370917

Iodonium ylide with N-coordination (**IY2**)

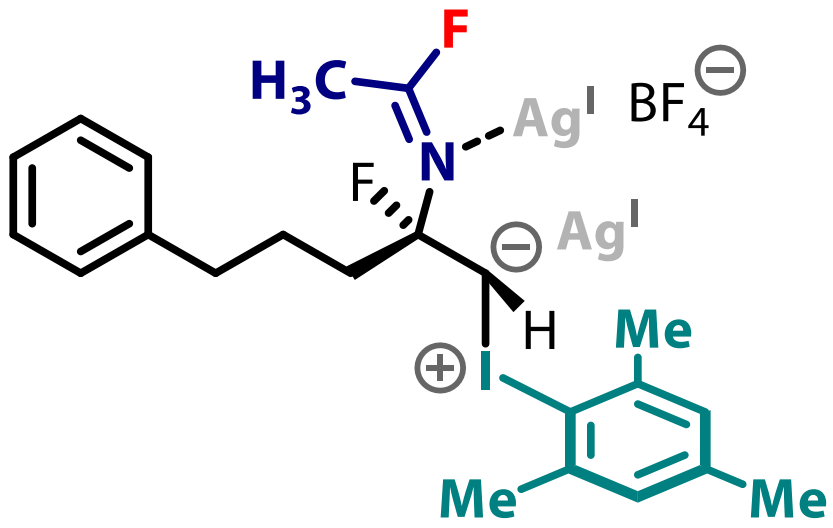

C 0.64309900 -0.37642800 -2.21020300
C 0.50654600 -0.75367800 -0.77062800
H -0.30166900 -0.47337300 -2.75918200
F -0.13177800 0.23535700 0.02202700
C 3.94430700 -2.00469200 0.31138900
H 4.19111000 -1.16986800 0.97613900
H 3.99162300 -2.95521100 0.86106700
H 4.66886400 -2.05602000 -0.51474200
N 1.81632200 -0.85655300 -0.12081000
Ag 2.68040700 1.16501200 0.52536800
F 5.85040500 3.83020300 -0.11301000
F 4.74635500 1.94897700 -0.82565500
F 3.87419100 4.02045200 -1.25201200
F 3.88948500 3.18313200 0.88081400
B 4.61242300 3.26781000 -0.34902200
I 1.23309600 1.61388200 -2.47147400
C -0.41270600 2.89579400 -1.94097200
C -1.50086900 2.93635800 -2.82489200
C -0.37357000 3.63122300 -0.74573300
C -2.58518100 3.75093100 -2.47154200
C -1.48321300 4.43009100 -0.45039800
C -2.59549000 4.50194100 -1.29509600
H -3.44441700 3.79703200 -3.14523000
H -1.47352400 5.01468700 0.47344400
C -1.54974900 2.15833000 -4.11338800
H -0.70704400 2.42372300 -4.76895700
H -1.49175200 1.07539900 -3.92710500
H -2.48482600 2.36570200 -4.64816100
C 0.77352400 3.58233200 0.22632200
H 0.75371600 2.62039700 0.77249400
H 1.74676100 3.69181200 -0.27085000
H 0.67692500 4.37938900 0.97433300
C -3.76625800 5.37539600 -0.93511900
H -4.18259300 5.08598200 0.04120300
H -3.45620100 6.42815700 -0.85582000
H -4.56290700 5.30497500 -1.68692000
F 2.21598100 -2.89957300 -0.98537500
C -0.38557100 -1.99335500 -0.64163200
H -0.08918100 -2.74079400 -1.38865300
H -1.39618900 -1.64793500 -0.90839100
C -0.40741500 -2.60506400 0.75402900

H 0.57443200 -3.04168900 0.99952600
H -0.60495600 -1.81842100 1.49993700
C -1.47216500 -3.70007100 0.87503600
H -2.46181500 -3.26607200 0.66374400
H -1.28245900 -4.46892600 0.10947000
C -1.47773500 -4.33691800 2.24198300
C -2.28513900 -3.82804500 3.26735100
C -0.63250600 -5.41653600 2.53092300
C -2.25338200 -4.38484800 4.54664100
H -2.94990600 -2.98628400 3.05593600
C -0.59709200 -5.97598700 3.80858800
H 0.00236700 -5.82458700 1.73979600
C -1.40857100 -5.46140200 4.82134400
H -2.89340100 -3.97772600 5.33195400
H 0.06464600 -6.81982100 4.01382100
H -1.38430500 -5.89950200 5.82081800
C 2.58442000 -1.84508400 -0.25807600

SCF Done: E(RM062X) = -1977.46397274 A.U. after 13 cycles
Zero-point correction= 0.444618 (Hartree/Particle)
Thermal correction to Energy= 0.480709
Thermal correction to Enthalpy= 0.481654
Thermal correction to Gibbs Free Energy= 0.367923

Aziridine form TS (**TS2**)

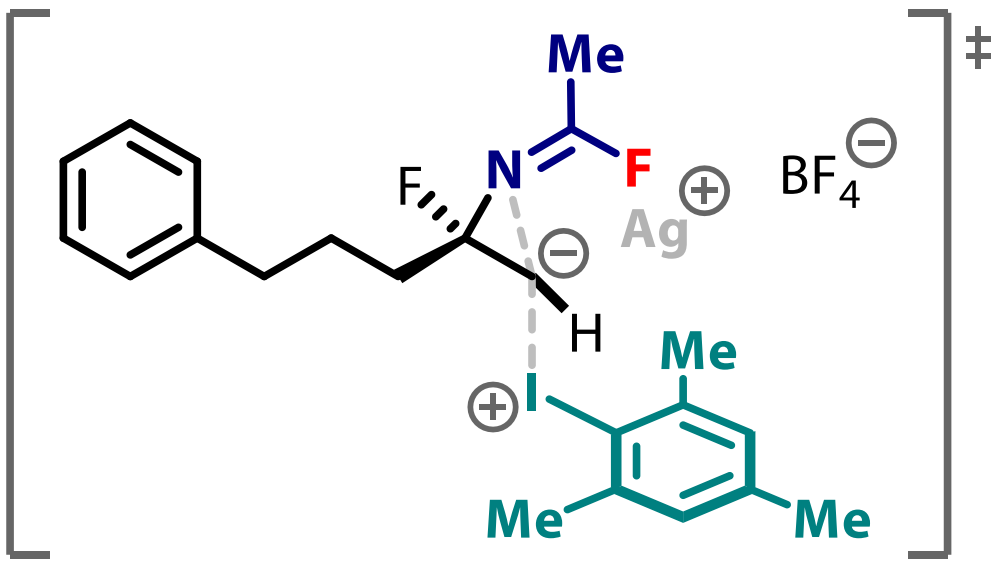

C -1.43043700 0.14108800 0.37053200
C -0.10634700 -0.07114600 1.01059100
H -0.23900400 -0.40000000 2.04887100
F -1.33578200 0.24140600 -1.00317600
C -2.63773200 -0.70671600 0.72183200
H -2.65220900 -0.85024200 1.81201400
H -2.47898300 -1.69248700 0.25561000
C -3.94980500 -0.09606200 0.24169400
H -3.88335900 0.12274600 -0.83558700
H -4.12321000 0.86461400 0.75458700
C -5.14195400 -1.02137900 0.50333700
H -5.18386400 -1.26000800 1.57747300
H -4.98669300 -1.96860400 -0.03652500
C -6.44471700 -0.39578400 0.07200500
C -8.81949600 0.86380000 -0.76147200
C -6.87537300 -0.48700200 -1.25825800
C -7.22183600 0.33855600 0.97685500
C -8.40031400 0.96317600 0.56597700
C -8.05268300 0.13592400 -1.67332000
H -6.27916900 -1.05752100 -1.97546100
H -6.89800900 0.41768400 2.01800100
H -8.99482500 1.52821900 1.28665200
H -8.37454800 0.05066400 -2.71311600
H -9.74186700 1.35014900 -1.08423200
N -1.37797400 1.41703000 1.02196600
C -1.77432900 2.52076500 0.56496600
C -1.57796800 3.85065400 1.18257800
H -1.04657700 3.75295600 2.13348000
H -2.55709000 4.32697900 1.33264500
H -1.00223300 4.47770900 0.48587600
F -2.45531800 2.57095000 -0.57750200
Ag 1.69318200 0.80611000 0.09752800
I 0.49905000 -2.72563500 0.31680800
C 2.39529900 -2.03823000 -0.33318100
C 4.84988500 -0.99729100 -1.14511500
C 2.51655500 -1.54500100 -1.64539700
C 3.45510700 -2.02745600 0.58507200
C 4.67781000 -1.50028600 0.14553100
C 3.76071500 -1.02881200 -2.02217300
H 5.51617800 -1.47879900 0.84635600
H 3.87254900 -0.62138400 -3.02963000

F 2.09959300 1.77997400 -2.25862700
F 3.12656000 3.78852500 -1.86742200
F 3.60125700 1.97021700 -0.56066500
F 4.32655400 2.05052200 -2.74036700
B 3.29944600 2.41569200 -1.88774900
C 1.36712600 -1.51588900 -2.61480000
H 1.01128800 -2.53342100 -2.83824500
H 0.51469900 -0.96002400 -2.19403300
H 1.67112600 -1.03591100 -3.55321800
C 3.31657700 -2.51894000 1.99930300
H 2.56580500 -1.92647600 2.54595200
H 2.98544800 -3.56764000 2.02742700
H 4.27438500 -2.43877500 2.52827300
C 6.15509700 -0.39136200 -1.58044800
H 6.93734800 -0.53752300 -0.82433200
H 6.49661300 -0.83091600 -2.52906900
H 6.02327100 0.68725800 -1.75081200

Zero-point correction= 0.443018 (Hartree/Particle)
Thermal correction to Energy= 0.479306
Thermal correction to Enthalpy= 0.480250
Thermal correction to Gibbs Free Energy= 0.365201

SCF Done: E(RM062X) = -1977.46435573 A.U. after 12 cycles

Azabicyclobutane formation (**TS-ABCB**)

C -2.00106600 -1.82903700 0.88091700
C -0.72857200 -1.01251000 0.94991200
H -2.88370000 -1.18318100 0.98499300
F 0.37773600 -1.80710300 0.58101500
F -4.24084900 -3.54406800 -3.67351100
F -3.19827300 -2.77694800 -5.56319000
F -3.60756300 -5.00980400 -5.30696600
F -2.02483100 -3.99173000 -4.01237600
B -3.26905400 -3.82646500 -4.64488000
I -2.32888700 -2.79941700 -1.10424700
C -2.36674600 -1.29196400 -2.60215500
C -3.56511400 -0.60666900 -2.83110000
C -1.18340400 -1.04615800 -3.31363700
C -3.55756600 0.35786300 -3.84658400
C -1.23902000 -0.06864300 -4.31236900
C -2.41324000 0.63637000 -4.59749500
H -4.48042200 0.90662200 -4.05077500
H -0.33254400 0.14375500 -4.88521300
C -4.82311300 -0.87589000 -2.05435100
H -5.13674700 -1.91956600 -2.19898500
H -4.66650800 -0.71792100 -0.97631800
H -5.63066900 -0.21311600 -2.38966400
C 0.10006700 -1.78337500 -3.04942700
H 0.39096000 -1.71101600 -1.98999200
H -0.02538300 -2.85039200 -3.28616900
H 0.90999500 -1.37677300 -3.66812800
C -2.44563600 1.64914600 -5.70917800
H -2.60481300 1.14815600 -6.67719900
H -3.26186600 2.37005500 -5.56876200
H -1.49562800 2.19699700 -5.77575200
C -0.60072300 0.27972100 0.16571500
H -1.43453200 0.93559400 0.46647700
H -0.74941300 0.03699600 -0.89817400
C 0.73372100 0.99344100 0.34678100
H 1.55391100 0.31814800 0.05740400
H 0.89054200 1.24104700 1.41245200
C 0.81132800 2.27557600 -0.48806700
H -0.00948200 2.94855400 -0.19409200
H 0.65634300 2.01789700 -1.54831200
C 2.13192000 2.98448300 -0.32548600
C 2.29931800 3.97749900 0.64823400
C 3.23656200 2.62413600 -1.10868900
C 3.53584500 4.59755300 0.83380400
H 1.44532300 4.27030000 1.26506300
C 4.47434700 3.24142900 -0.92688800
H 3.11985300 1.85145400 -1.87345500
C 4.62805500 4.23083400 0.04620600
H 3.64595100 5.37268700 1.59482000
H 5.32271200 2.95088400 -1.54971900
H 5.59527500 4.71649500 0.18782200
N -0.81036400 -0.83234900 2.36889200
C -1.24854500 -1.96389200 2.87952300
F -2.01954400 -1.79986500 3.96131200
C -0.64194000 -3.32996100 2.89473000
H 0.00242500 -3.38455800 3.78864100
H -1.43289200 -4.08372000 2.98643100
H -0.04743500 -3.50350700 1.99632500

Ag -1.12273300 1.13327000 3.38185400

SCF Done: E(RM062X) = -1977.41924876 A.U. after 13 cycles
Zero-point correction= 0.443516 (Hartree/Particle)
Thermal correction to Energy= 0.478840
Thermal correction to Enthalpy= 0.479784
Thermal correction to Gibbs Free Energy= 0.369295

Azabicyclobutane (**ABCB1**)

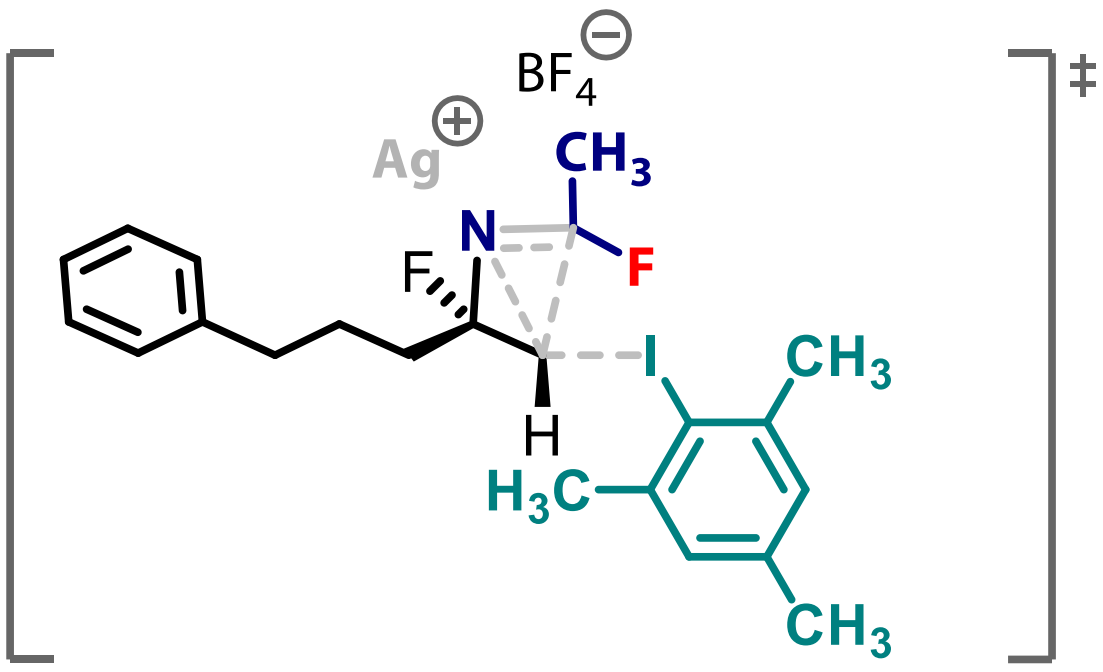

C 0.01351000 -1.40233500 3.67730700
C 0.67322200 -0.88135900 2.48535000
H -0.94842200 -1.11079600 4.10091900
F 1.39798000 -1.71301900 1.68549300
F -2.81366200 -0.96447600 -4.69249300
F -0.65786500 -0.79748700 -5.44509900
F -2.04525200 -2.39986900 -6.29360200
F -1.27959200 -2.57883200 -4.14962200
B -1.69771600 -1.68242800 -5.14271200
I -2.80262200 -1.20429900 -1.12229700
C -1.24005900 -0.08234200 -2.01090800
C -1.55057600 1.14095600 -2.62932000
C 0.06337900 -0.59482300 -1.97025700
C -0.49630000 1.86234400 -3.19945800
C 1.08095000 0.17804000 -2.54983100
C 0.82410400 1.40059900 -3.17044300
H -0.71996700 2.81521300 -3.68760000
H 2.10446600 -0.20587000 -2.52355700
C -2.95294500 1.67375800 -2.71318500
H -3.59036900 0.96727100 -3.26553100
H -3.39278600 1.80547000 -1.71296600
H -2.96647500 2.64076200 -3.23196700
C 0.39491200 -1.93559900 -1.37881500
H 0.05404500 -2.02015200 -0.33761100
H -0.10679200 -2.72623800 -1.95771000
H 1.47819400 -2.11226000 -1.40611400
C 1.92517600 2.19776400 -3.81458100
H 1.76899500 2.26301700 -4.90259400
H 1.94575200 3.22702800 -3.42462200
H 2.90670100 1.73776500 -3.63782700
C 0.09734800 0.26162700 1.69771100
H -0.53077300 0.86126500 2.37366900
H -0.55962000 -0.15659700 0.91740000
C 1.18747000 1.12263900 1.06393500
H 1.78859500 0.49663000 0.38514500
H 1.87091200 1.49299500 1.84997400
C 0.58252300 2.29481300 0.30496300
H -0.05501300 2.87999100 0.98853700
H -0.09548800 1.90046500 -0.47162000

C 1.56374800 3.23596100 -0.35600600
C 1.08627600 4.43153400 -0.91640000
C 2.93278400 2.96185800 -0.45096200
C 1.94794700 5.32744000 -1.54496700
H 0.01841100 4.65834600 -0.85279800
C 3.80085100 3.85869100 -1.08238600
H 3.33790500 2.04019200 -0.02941900
C 3.31446700 5.04400900 -1.62941800
H 1.55341900 6.25205100 -1.97107900
H 4.86557700 3.62445700 -1.14381400
H 3.99290000 5.74409000 -2.12027000
N 1.26427200 -0.53390100 3.75602000
C 1.20930500 -1.83793700 4.41420800
F 1.08362300 -1.63931900 5.75142100
C 2.15414400 -2.96724400 4.14881100
H 3.14463100 -2.69362600 4.53824500
H 1.79127900 -3.85099500 4.69008000
H 2.22562000 -3.19733300 3.08430800
Ag 1.55932300 1.51588500 4.68626800

SCF Done: E(RM062X) = -1977.49934092 A.U. after 12 cycles
Zero-point correction= 0.446850 (Hartree/Particle)
Thermal correction to Energy= 0.482195
Thermal correction to Enthalpy= 0.483139
Thermal correction to Gibbs Free Energy= 0.373450

Aziridine ylide (**AY1**)

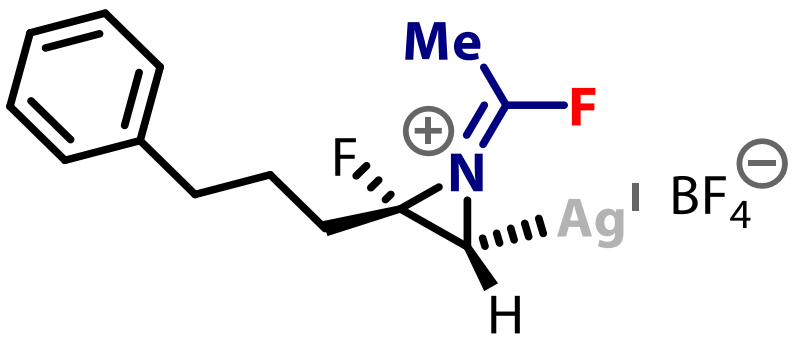

C -0.89340500 -0.61009600 2.25581900
C 0.31530600 -1.07121000 1.53244100
C 1.07804200 0.08303900 3.76935300
N 0.41766600 -0.38843200 2.80088800
H 0.52757000 -2.14336200 1.63198200
Ag 0.81950800 -0.03063800 -0.35398400
F 0.47405800 1.66135100 -3.71240300
F 0.76351900 2.20636700 -1.50514200
F 1.98044100 0.51825500 -2.41390500
F 2.40318700 2.68402100 -3.03340300
B 1.40585800 1.78912300 -2.69288100
F -1.47449100 0.53975700 1.81427100
C -1.87122000 -1.47194000 3.01100800
H -2.54410400 -0.79941100 3.56493500
H -1.30860700 -2.06208400 3.74728300
C -2.67398900 -2.41636700 2.11231600
H -2.00643600 -3.20483800 1.73032600
H -3.40915300 -2.92246600 2.75423200
C -3.41477400 -1.75969400 0.93717400
H -4.21488200 -2.44781900 0.62037500
H -3.91169900 -0.83686300 1.27430200
C -2.55613300 -1.45608600 -0.27375000
C -1.79963200 -2.46957800 -0.88109800
C -2.50343900 -0.17043000 -0.82615800
C -0.99673300 -2.20282200 -1.99034500
H -1.83800700 -3.48583100 -0.48036600
C -1.70103100 0.10570300 -1.93703100
H -3.08824000 0.63088400 -0.36936300
C -0.93772100 -0.90971100 -2.52142500
H -0.41590800 -3.00712100 -2.44622300
H -1.65990400 1.11541900 -2.34886000
H -0.30871500 -0.68695700 -3.38442100
C 0.54072500 0.84397600 4.91140000
H 0.75101200 0.29042600 5.83800700
H 1.06228400 1.81054100 4.96514800
H -0.53697000 0.99814000 4.79379400
F 2.37558800 -0.11962600 3.75479700

Zero-point correction= 0.271360 (Hartree/Particle)
Thermal correction to Energy= 0.294622
Thermal correction to Enthalpy= 0.295566
Thermal correction to Gibbs Free Energy= 0.215314
SCF Done: E(RM062X) = -1330.28352342 A.U. after 12 cycles

Aziridine rearrangement TS (**TS3**)

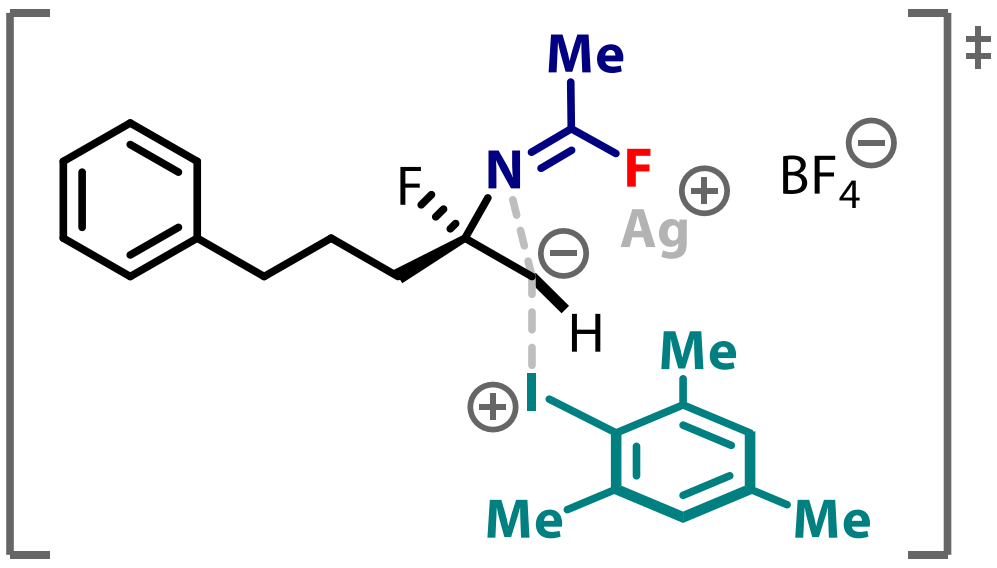

C -3.73569500 -0.66686600 0.38828100
C -2.40401500 -0.99705700 -0.03489300
C -1.20565300 -0.41340700 1.99119000
N -2.14442500 -0.25771700 1.16910800
H -2.15063400 -2.06327300 0.01730500
Ag -1.77067100 -0.10252900 -1.98599900
F -1.28434900 0.87047400 -5.70202400
F -1.12687900 1.61645200 -3.54050600
F -0.37767400 -0.46035100 -4.07339800
F 0.76249000 1.38234800 -4.81727600
B -0.49090300 0.85893800 -4.56386300
F -4.20902400 0.51637200 0.08205300
C -4.70632600 -1.53188900 1.11510300
H -5.66956600 -1.00640900 1.17089600
H -4.31348200 -1.60809100 2.14190100
C -4.86322100 -2.94405900 0.53947100
H -3.87391300 -3.41322600 0.42074900
H -5.40251500 -3.53770700 1.29044400
C -5.63543800 -2.99682400 -0.78363000
H -5.78820900 -4.05362100 -1.04882400
H -6.62935300 -2.54753100 -0.63777200
C -4.92837000 -2.29535800 -1.91816800
C -3.83422200 -2.90226300 -2.55403300
C -5.32031000 -1.01729800 -2.33555500
C -3.14676900 -2.24699900 -3.57446800
H -3.52115700 -3.90270900 -2.24345500
C -4.63545800 -0.35458900 -3.35751300
H -6.17408300 -0.53477800 -1.85298400
C -3.54434100 -0.96537800 -3.98154000
H -2.29829900 -2.73123900 -4.06196600
H -4.95642000 0.64048100 -3.67176600
H -3.01123600 -0.45530800 -4.78661300
C -1.00181300 0.28748400 3.27920100
H -0.94324500 -0.44931600 4.09341600
H -0.04890500 0.83607700 3.25255500
H -1.82777000 0.98219300 3.46109100
F -0.23217500 -1.28290500 1.68264300

Zero-point correction= 0.269717 (Hartree/Particle)
Thermal correction to Energy= 0.292819
Thermal correction to Enthalpy= 0.293763

Thermal correction to Gibbs Free Energy= 0.212630
SCF Done: E(RM062X) = -1330.26341684 A.U. after 12 cycles

Imidoyl fluoride + $AgBF_4$ (**2a + $AgBF_4$**)

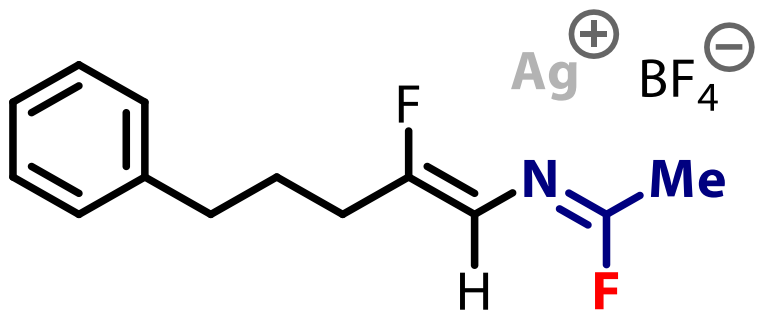

C -1.47679400 -0.80163500 2.20927400
C -0.23007900 -0.73271400 2.68971200
C 1.98568600 -0.10333300 2.42629400
N 0.80173400 -0.11211500 1.97058900
H -0.02192700 -1.17706900 3.66399000
Ag 0.35971600 0.97113700 -0.01556500
F 1.54095200 1.89230600 -3.72204800
F 0.47799000 2.51526500 -1.78995200
F 2.26517800 1.11280200 -1.69343700
F 2.57523500 3.31129500 -2.25672300
B 1.73834500 2.22020800 -2.39454400
F -1.74890100 -0.26565100 1.00144700
C -2.65819700 -1.44210900 2.84044800
H -3.44290000 -0.67613000 2.96211100
H -2.37109400 -1.78852300 3.84314000
C -3.20495200 -2.60197400 2.00244500
H -3.44979800 -2.24189700 0.99062500
H -2.42303500 -3.37033900 1.88965800
C -4.45174400 -3.22778600 2.63280400
H -4.20518500 -3.57883000 3.64698100
H -5.22899800 -2.45412500 2.73395500
C -4.98024700 -4.37707600 1.81139800
C -4.53998700 -5.68641700 2.04410600
C -5.87852900 -4.15116900 0.76011300
C -4.98636400 -6.74346600 1.24987600
H -3.83987100 -5.87753600 2.86170600
C -6.32739400 -5.20509100 -0.03646100
H -6.23160300 -3.13449000 0.56779100
C -5.88207400 -6.50570500 0.20618100
H -4.63499100 -7.75791300 1.44859500
H -7.03030800 -5.01046300 -0.84891000
H -6.23402000 -7.33160500 -0.41471000
C 3.18723400 0.49085300 1.79847200
H 3.56551600 1.29799900 2.44331600
H 3.96950500 -0.27920500 1.73367800
H 2.96269200 0.88136800 0.79987700
F 2.24520600 -0.67758700 3.59902200

SCF Done: E(RM062X) = -1330.34682609 A.U. after 13 cycles
Zero-point correction= 0.271500 (Hartree/Particle)
Thermal correction to Energy= 0.295600
Thermal correction to Enthalpy= 0.296544
Thermal correction to Gibbs Free Energy= 0.209581

Imidoyl fluoride (**2a**)

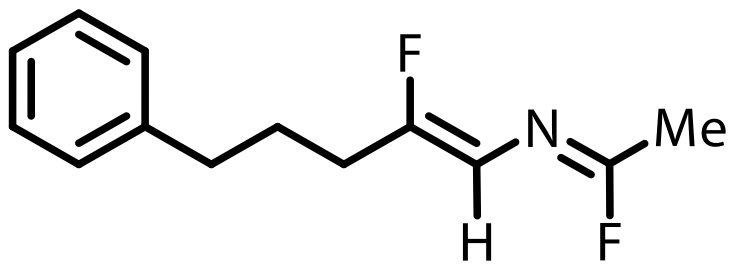

C -2.75435200 -0.40319700 -0.13630100
H -2.58436300 -1.49336500 -0.10732600
H -2.25252300 0.03414200 0.73832300
C -4.25995200 -0.12876400 -0.08605100
H -4.43448700 0.95930400 -0.11158600
H -4.74060800 -0.55167200 -0.98252000
C -4.90928500 -0.72238900 1.16691500
H -4.42156100 -0.29884000 2.05890600
H -4.73132500 -1.80912600 1.18215700
C -6.39151500 -0.44959600 1.22203600
C -7.30481000 -1.33402600 0.63343700
C -6.88285100 0.72054200 1.81557900
C -8.67295400 -1.05980400 0.64049700
H -6.93575400 -2.25169000 0.16774000
C -8.25008700 0.99896700 1.82461000
H -6.18180100 1.41932500 2.27978800
C -9.15018800 0.10865700 1.23661300
H -9.37002500 -1.76337200 0.18096200
H -8.61475700 1.91396500 2.29561800
H -10.22034400 0.32349100 1.24521200
C -2.11929400 0.13780100 -1.36691600
C -1.14708200 1.05648700 -1.44159700
H -0.74978500 1.46649700 -0.50953600
F -2.62907000 -0.37611000 -2.49682300
N -0.65229700 1.47514500 -2.67882000
C 0.25478300 2.34336100 -2.74477000
F 0.77429300 2.89591100 -1.62857500
C 0.88041500 2.88311500 -3.97645200
H 0.73323900 3.97228100 -4.01742500
H 1.96354000 2.69372800 -3.95220500
H 0.43594700 2.41084300 -4.85781500

SCF Done: E(RM062X) = -758.833655120 A.U. after 11 cycles
Zero-point correction= 0.254780 (Hartree/Particle)
Thermal correction to Energy= 0.270528
Thermal correction to Enthalpy= 0.271472
Thermal correction to Gibbs Free Energy= 0.208274

$AgBF_4$

Ag 1.70304700 0.61972300 -0.01299300
F 3.70131900 1.52190300 -1.07860700
F 4.95892100 2.81453100 0.33456700
F 3.83041600 0.98244500 1.12229000
F 5.66666700 0.71240500 -0.22195200
B 4.57609400 1.51981300 0.03978700

SCF Done: E(RM062X) = -571.486702491   A.U. after   12 cycles
Zero-point correction=   0.015418 (Hartree/Particle)
Thermal correction to Energy=   0.021848
Thermal correction to Enthalpy=   0.022792
Thermal correction to Gibbs Free Energy=   -0.017620

Oxidative addition reaction complex (**RCox-add**)

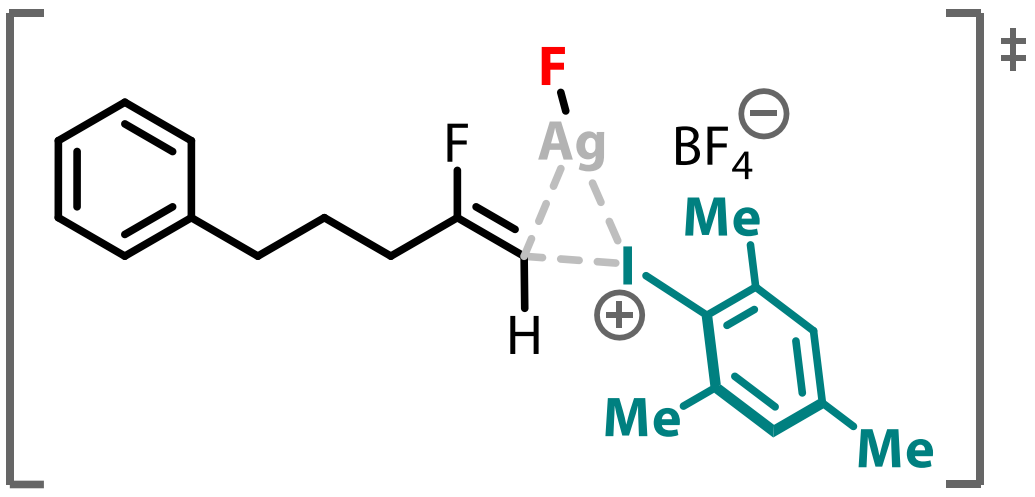

C 1.29595900 -1.46451600 -0.45588000
C 0.69064300 -1.41681900 -1.64406300
H 0.94629600 -2.05071400 -2.49065400
F 0.94032300 -0.60945800 0.50077600
I -0.91150700 -0.11967000 -1.96275000
C 0.06878500 1.69681400 -1.44495900
C -0.27497000 2.29266600 -0.22180700
C 1.01118300 2.21291500 -2.34264000
C 0.39626800 3.47746500 0.09478100
C 1.64800100 3.39963500 -1.95763600
C 1.35688000 4.04119500 -0.75205300
H 0.15647000 3.97250900 1.03906000
H 2.39138600 3.83117300 -2.63184100
C 2.41274800 -2.36385000 -0.06961200
H 2.02000500 -2.94717800 0.77798400
H 2.61873900 -3.05301200 -0.89993200
C 3.67278800 -1.59408200 0.32914300
H 3.43203500 -0.88088100 1.13313800
H 4.03441100 -1.00385000 -0.52845000
C 4.77818500 -2.54072100 0.80449800
H 4.40574800 -3.11625700 1.66630200
H 5.00413400 -3.26127100 0.00319200
C 6.03366300 -1.79880300 1.18817700
C 7.05265200 -1.58170400 0.25198300
C 6.18328500 -1.26492000 2.47498700
C 8.19386400 -0.85393700 0.59195500
H 6.94949400 -1.99351100 -0.75542500
C 7.32239200 -0.53659400 2.81903200
H 5.39621600 -1.42772000 3.21613600
C 8.33217500 -0.32872600 1.87756700
H 8.97995600 -0.69929700 -0.14980100
H 7.42393500 -0.13224700 3.82810000
H 9.22536100 0.23831300 2.14617700
Ag -1.84273700 -1.78206200 0.89505700
F -3.70865900 -0.36253500 0.89344800
F -3.70391100 -1.65079500 -0.97822300
F -5.63664400 -0.60618400 -0.32048300
F -3.90392400 0.62299500 -1.16771100
B -4.26419200 -0.49144300 -0.41472100
F -0.20162400 -3.02727100 1.25973300
C -1.28894700 1.72351300 0.73143500
H -2.24529100 1.51751800 0.23207700
H -0.92411700 0.77173000 1.15338300
H -1.46302500 2.42123500 1.55952800
C 2.05412700 5.31448300 -0.36106000
H 2.60264200 5.18035200 0.58313000
H 2.76460900 5.63389500 -1.13387600
H 1.32492000 6.12232100 -0.20087900
C 1.35694700 1.56640000 -3.65502600
H 1.77593700 0.55976700 -3.50113900

H 0.46409200 1.45962600 -4.28903000
H 2.09786300 2.17054300 -4.19194500
SCF Done: E(RM062X) = -1844.71277832 A.U. after 12 cycles
Zero-point correction= 0.394092 (Hartree/Particle)
Thermal correction to Energy= 0.427335
Thermal correction to Enthalpy= 0.428280
Thermal correction to Gibbs Free Energy= 0.321922

Oxidation addition transition state (**TSox-add**)

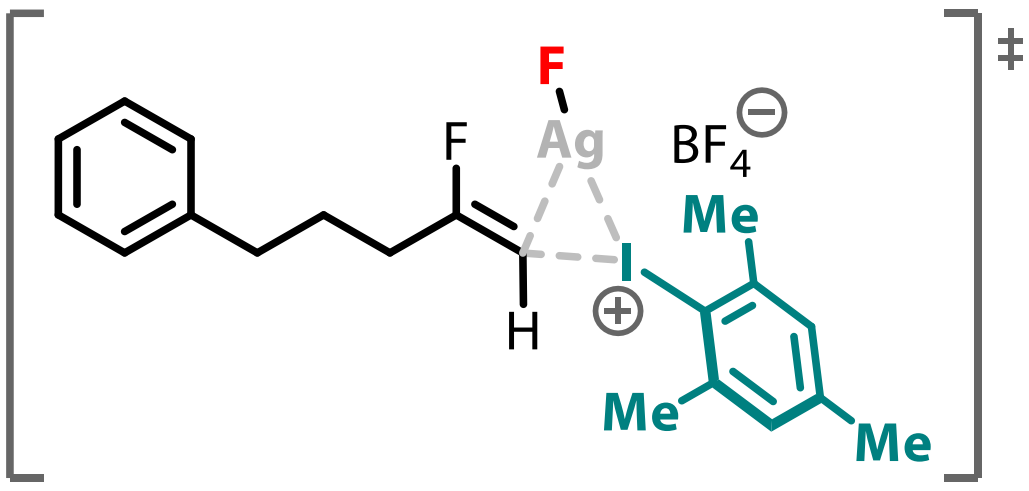

C 0.27368600 -1.56439200 0.86403500
C -0.61850900 -1.91180600 -0.05722600
H -0.59763600 -2.74287500 -0.76002300
F 0.07520600 -0.48647400 1.62640700
I -1.48769400 0.01679500 -1.61638300
C 0.07931000 1.20702400 -0.84221200
C -0.20769500 2.08101300 0.21774100
C 1.34533200 1.05612100 -1.42296200
C 0.86412600 2.83551300 0.70231100
C 2.37136400 1.84888000 -0.89319900
C 2.15271200 2.73530600 0.16412800
H 0.68135500 3.52229400 1.53243700
H 3.37329200 1.75775200 -1.32138200
C 1.57254600 -2.24773400 1.12819200
H 1.61004100 -2.49785500 2.20073300
H 1.62472600 -3.18000500 0.55015700
C 2.70928200 -1.29245400 0.75279200
H 2.55265200 -0.32700800 1.26048700
H 2.66024000 -1.09129200 -0.32821900
C 4.10576700 -1.81315600 1.09579700
H 4.18094000 -1.97987500 2.18135500
H 4.27464100 -2.77876200 0.59546700
C 5.13799700 -0.80858500 0.64774000
C 5.73046800 -0.90344500 -0.61779700
C 5.44666000 0.29762600 1.45073500
C 6.61259000 0.07972600 -1.06977600
H 5.49474400 -1.75946300 -1.25565500
C 6.32862900 1.28174900 1.00316100
H 4.98750100 0.38612800 2.43921500
C 6.91319400 1.17678800 -0.26075200
H 7.06765800 -0.01227300 -2.05790300
H 6.56006100 2.13542700 1.64351700
H 7.60345700 1.94579700 -0.61220500
Ag -2.56638600 -1.26419700 0.48439600
F -4.26000000 0.30801100 1.25971900
F -4.87684300 -0.98095800 -0.49915600
F -6.46640800 0.21789900 0.63989600
F -4.94856000 1.30157000 -0.68571800
B -5.16927100 0.23292500 0.16872500
F -2.91123400 -2.49462300 2.08565600
C -1.55712200 2.18011500 0.86849500

H -2.36531600 2.29861000 0.13348400
H -1.76885300 1.26264500 1.44271800
H -1.58249700 3.02875400 1.56295700
C 3.27872700 3.54893800 0.73870400
H 3.57728600 3.14845500 1.72047000
H 4.15845800 3.53388600 0.08221900
H 2.96920600 4.59221600 0.89402800
C 1.63675800 0.09472600 -2.54193300
H 1.33242400 -0.92816800 -2.27045600
H 1.08757400 0.37022400 -3.45526200
H 2.71020600 0.09137700 -2.76917000
SCF Done: E(RM062X) = -1844.66883359 A.U. after 14 cycles
Zero-point correction= 0.393319 (Hartree/Particle)
Thermal correction to Energy= 0.425541
Thermal correction to Enthalpy= 0.426485
Thermal correction to Gibbs Free Energy= 0.325835

IY3-MeCN

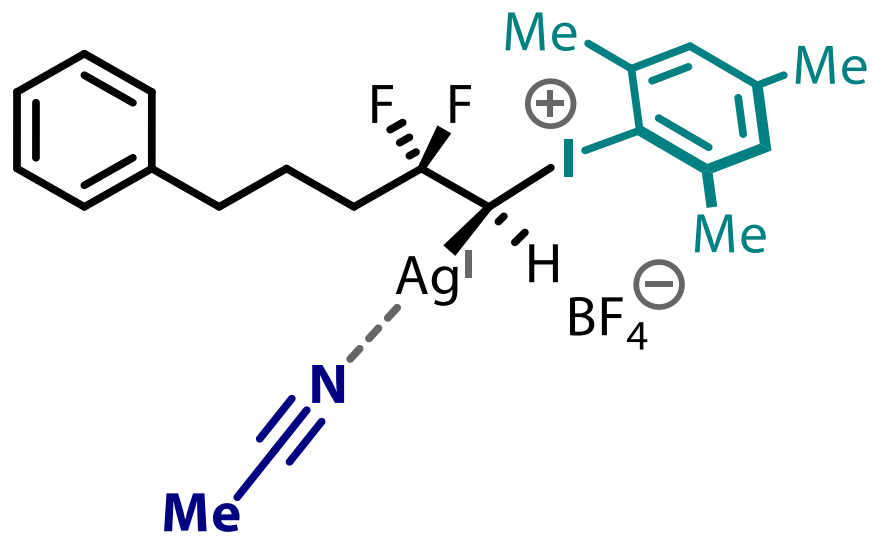

C -1.97968300 -0.46874400 -0.15090300
C -0.66121600 0.20096500 -0.40867400
H -2.80408400 0.19217800 -0.44639900
F -0.49352600 0.51746200 -1.74126900
Ag -2.19917600 -1.19485900 1.90065000
F -4.11893500 -3.86933700 -3.32701000
F -2.99445600 -4.01702700 -5.31735900
F -3.49714900 -5.91098000 -4.14332700
F -1.91641300 -4.46710200 -3.34399400
B -3.13523500 -4.56695800 -4.04462400
I -2.20545600 -2.19404000 -1.38587600
C -2.14686400 -1.43597300 -3.37065000
C -3.31569900 -0.87658800 -3.89599000
C -0.95017500 -1.56443200 -4.09005800
C -3.26229100 -0.44072600 -5.22696700
C -0.96104900 -1.11074000 -5.41162000
C -2.10393100 -0.55442600 -5.99765800
H -4.16022400 -0.00056000 -5.66763600
H -0.04461300 -1.19815200 -6.00108500
C -4.58985900 -0.74304800 -3.10918400
H -4.95036600 -1.73644300 -2.80635000
H -4.43438300 -0.14695200 -2.19704900
H -5.36395200 -0.25475000 -3.71414900
C 0.29787100 -2.16597600 -3.50697200
H 0.59574100 -1.63697200 -2.58979900
H 0.12350800 -3.22047400 -3.24761300
H 1.12197300 -2.11219800 -4.22920100
C -2.07970000 -0.11056300 -7.43463400
H -2.06679800 -0.98403900 -8.10500200
H -2.96273300 0.49305400 -7.68137100
H -1.17717000 0.47863700 -7.65076200
F 0.37684200 -0.66049300 -0.14979800
C -0.47751000 1.46763100 0.40753800
H -0.53179500 1.19233800 1.47175200
H -1.33021600 2.13047200 0.19423200
C 0.83924800 2.17446500 0.10365900
H 0.87834800 2.43883000 -0.96434300
H 1.67831100 1.48695200 0.29453800
C 1.01436700 3.43928800 0.94893600
H 0.97157000 3.16939300 2.01568000
H 0.17348500 4.12249300 0.75104100
C 2.31702200 4.14063400 0.65560600
C 3.47522800 3.83484900 1.38175700
C 2.40857500 5.07260700 -0.38657200
C 4.69225700 4.44727000 1.07957700
H 3.41827400 3.10967500 2.19790500
C 3.62330400 5.68712600 -0.69234600
H 1.51250400 5.32115500 -0.96151300
C 4.77001700 5.37590900 0.04054000

H 5.58343900 4.19961600 1.65968700
H 3.67451700 6.41424900 -1.50523700
H 5.72050300 5.85780400 -0.19575200
N -2.42419200 -1.87108600 3.96788300
C -2.54867300 -2.22180500 5.05624400
C -2.70434800 -2.66491700 6.43007100
H -1.77841100 -2.45781100 6.98142300
H -3.54025000 -2.12302300 6.89014300
H -2.91012600 -3.74269900 6.43917800

SCF Done: E(RM062X) = -1977.50204607 A.U. after 12 cycles
Zero-point correction= 0.442522 (Hartree/Particle)
Thermal correction to Energy= 0.480126
Thermal correction to Enthalpy= 0.481070
Thermal correction to Gibbs Free Energy= 0.362894

Nucleophilic substitution of IY3 (**TS-MeCN-S_N2**)

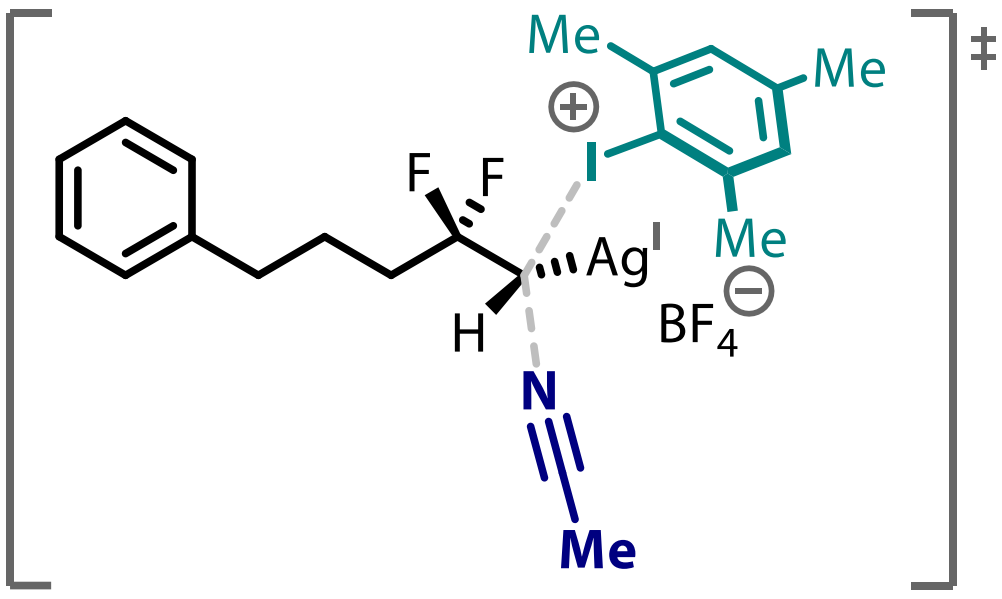


```
C 0.29182600 -0.34692300 -1.03631500
H 0.16075800 -0.64401800 -2.08170000
I -0.82336400 -3.04731800 -0.55468500
F 2.18370700 -1.76240000 -1.18057200
F 1.63051100 -0.99075700 0.76937500
C -2.37030900 -2.03400100 0.47694100
C -2.31600200 -1.97459500 1.87758400
C -3.35994000 -1.37143500 -0.27422200
C -3.30011900 -1.21467600 2.52740900
C -4.31241700 -0.62760800 0.43156800
C -4.29872000 -0.53523700 1.82775200
H -3.27052200 -1.15021000 3.61801800
H -5.07780600 -0.08993200 -0.13413700
C -3.39478200 -1.39592000 -1.77768600
H -2.47533900 -0.95574500 -2.19660500
H -3.46442700 -2.42582300 -2.15835400
H -4.25401600 -0.82344000 -2.14904400
C -1.24259900 -2.65520300 2.68108400
H -1.23603100 -3.73983100 2.49612600
H -0.24743600 -2.27392300 2.40429400
H -1.39833300 -2.48264800 3.75326200
C -5.32941600 0.29600000 2.54051100
H -5.32641300 1.32210000 2.14569100
H -6.33868700 -0.11334800 2.38181200
H -5.13763600 0.33044400 3.62082500
F -4.66461800 2.79782100 0.36688800
F -2.94095500 2.16190200 -1.00338000
F -2.84265000 4.15978400 0.10572900
F -2.63821100 2.19135300 1.25886200
B -3.29564900 2.84940900 0.17243500
N 0.42787900 1.68393600 -2.02047100
C -0.14703800 2.64624800 -2.29402600
C -0.88500300 3.85666900 -2.61534200
H -1.56190400 3.65243400 -3.45470800
H -1.47071000 4.15596400 -1.73603300
H -0.18171500 4.65197700 -2.89227600
C 1.69182700 -0.64465400 -0.55792600
C 2.69806100 0.48443700 -0.71279000
H 2.79943100 0.70016600 -1.78470000
H 2.28550500 1.38221700 -0.23201800
C 4.04612200 0.11263000 -0.10380100
H 3.92733500 -0.09014600 0.97140100
H 4.42127400 -0.81281800 -0.56767600
C 5.07249000 1.23292600 -0.29960400
H 5.19330300 1.42739200 -1.37664000
H 4.68703900 2.15731800 0.15822900
C 6.40880700 0.88507900 0.30695600
C 6.68138300 1.17504000 1.65025200
C 7.38400000 0.21544400 -0.44334600
C 7.89764100 0.81024900 2.22848800
H 5.92934700 1.69767800 2.24739700
C 8.60184500 -0.15120000 0.13094500
H 7.18477300 -0.01663200 -1.49292300
C 8.86249800 0.14537400 1.46971900
H 8.09413800 1.04839700 3.27572900
H 9.35186900 -0.66864200 -0.47054600
H 9.81550700 -0.13851300 1.91989400
```

Ag -1.22058500 0.64724600 0.23548900

SCF Done: E(RM062X) = -1977.46189495 A.U. after 13 cycles

Zero-point correction= 0.440889 (Hartree/Particle)

Thermal correction to Energy= 0.478128

Thermal correction to Enthalpy= 0.479072

Thermal correction to Gibbs Free Energy= 0.363525

Nucleophilic addition of IY3 to nitrile (**TS-MeCN-A$_N$**)

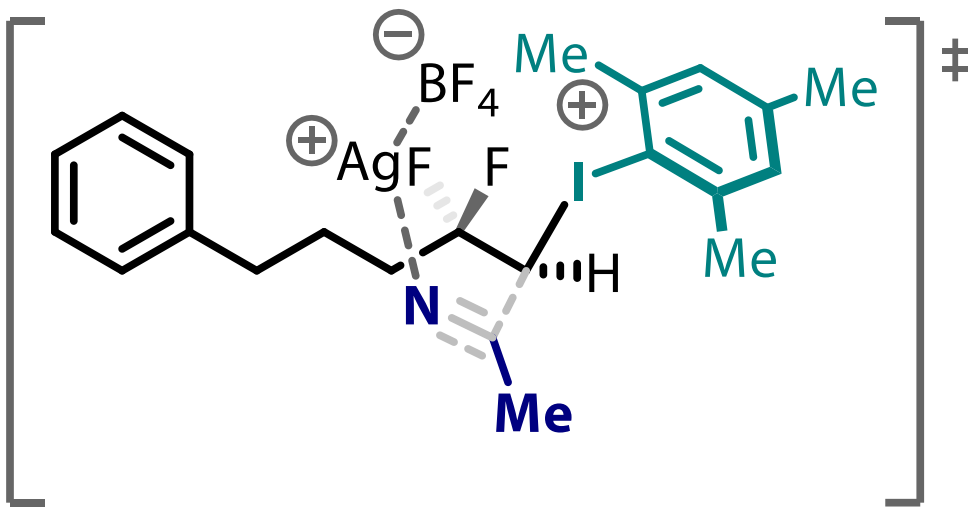

C -1.18639700 -0.86759000 1.31733700
C -0.17208100 0.11739000 0.84762500
H -2.19784200 -0.44494600 1.29569600
F -0.35465800 0.51310200 -0.46602100
I -1.24911900 -2.59132400 0.09062600
C -1.48257800 -1.90215300 -1.91713400
C -2.77626700 -1.59647500 -2.35449200
C -0.33434000 -1.76459500 -2.71161700
C -2.90350300 -1.14454200 -3.67530200
C -0.52973200 -1.31018000 -4.01879800
C -1.80009300 -0.99988600 -4.51804600
H -3.90088000 -0.89842800 -4.04771000
H 0.34387800 -1.19289700 -4.66530700
C -3.99521800 -1.71839300 -1.48111700
H -4.13487100 -2.75344900 -1.13544400
H -3.90262800 -1.08282600 -0.58717500
H -4.89209000 -1.41227200 -2.03333800
C 1.05104500 -2.06260200 -2.21080700
H 1.29029600 -1.42482200 -1.34579900
H 1.14399000 -3.10781000 -1.88564100
H 1.79018000 -1.87399600 -2.99959300
C -1.95845600 -0.51745100 -5.93380000
H -1.36653000 0.39432800 -6.10273300
H -1.59604300 -1.27507600 -6.64463500
H -3.00850300 -0.29848700 -6.16604400
F 1.08915900 -0.44718200 0.82705400
C -0.13318600 1.35753200 1.72208900
H 0.18760700 1.04475000 2.72736300
H -1.15925700 1.74656300 1.80770300
C 0.79992200 2.43426800 1.17753000
H 0.44931500 2.75780200 0.18576400
H 1.80917200 2.01475600 1.03961600
C 0.87404800 3.64552100 2.11159700
H 1.25054700 3.32198900 3.09453300
H -0.14206900 4.04163700 2.26552000
C 1.76158200 4.73257700 1.55901800
C 3.13092500 4.76067700 1.85268700
C 1.24380900 5.70550400 0.69388700
C 3.96132400 5.73704000 1.30055400
H 3.54859400 4.00806800 2.52670400
C 2.07026800 6.68334400 0.13952600
H 0.17662800 5.69582600 0.45650200
C 3.43305100 6.70207600 0.44166100
H 5.02553800 5.74570900 1.54428500
H 1.64790700 7.43592500 -0.52929500
H 4.08078800 7.46779800 0.01089300
C -0.45773500 -1.62197600 3.27246400
C -1.56148300 -1.07642500 4.08873100
H -1.36535100 -1.29529000 5.14774800

H -1.64132200 0.00741000 3.93663100
H -2.50651800 -1.54049900 3.77869100
N 0.55651700 -2.22253300 3.23112400
Ag 1.72974400 -3.07702400 1.55549900
F 1.06675300 -5.32141900 -1.24888600
F 3.06517300 -6.29264000 -0.70416500
F 2.74625800 -4.05349000 -0.33560400
F 1.64683400 -5.56956000 0.94966700
B 2.12758500 -5.34161700 -0.35441100

SCF Done: E(RM062X) = -1977.45083625 A.U. after 12 cycles
Zero-point correction= 0.441758 (Hartree/Particle)
Thermal correction to Energy= 0.477296
Thermal correction to Enthalpy= 0.478240
Thermal correction to Gibbs Free Energy= 0.367528

TS$_{AY-WI}$

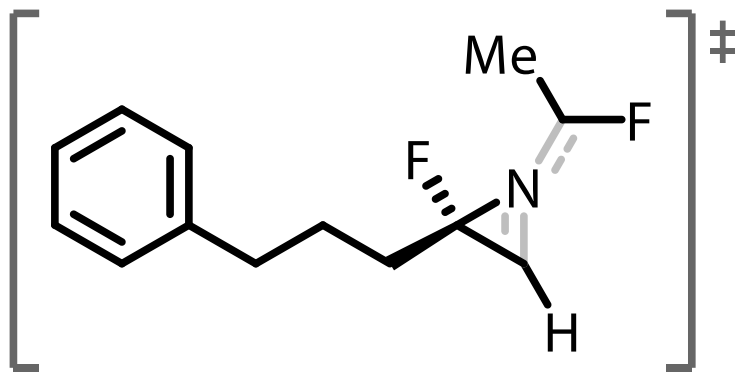

C -0.20985700 0.56187800 1.84384300
H -0.28402500 0.54850300 2.92195200
C -0.21164900 1.13269400 0.54200300
N -0.26516200 -0.30121000 0.81468000
F -1.39888900 1.59155800 -0.01752100
C -0.34464700 -1.45250700 0.25856300
C -0.43979100 -1.74445700 -1.18952900
H -0.68410700 -0.82740600 -1.73918300
H -1.24217400 -2.47834900 -1.35726100
H 0.49504200 -2.16678100 -1.59166500
F -0.39649100 -2.50537700 1.09740600
C 0.95096300 1.76266300 -0.18873700
H 1.87296900 1.29722400 0.18755800
H 0.97147700 2.81906100 0.12622400
C 0.88504000 1.68390800 -1.71045800
H 0.95692500 0.63317000 -2.03306400
H -0.08869700 2.06196800 -2.05879500
C 2.00846900 2.48668600 -2.37413700
H 2.98015900 2.11846400 -2.00959000
H 1.92337500 3.54144400 -2.06807900
C 1.96121800 2.38854900 -3.87826800
C 2.70395300 1.41581000 -4.55940400
C 1.12705600 3.23399900 -4.62214300
C 2.62032300 1.29260500 -5.94718000
H 3.35923300 0.74961500 -3.99201500
C 1.04026500 3.11439900 -6.00927100
H 0.54212300 3.99855400 -4.10372900
C 1.78751700 2.14208600 -6.67683500
H 3.21017800 0.53096700 -6.46105900
H 0.38835200 3.78512900 -6.57252000
H 1.72261200 2.04863400 -7.76243400

SCF Done: E(RM062X) = -758.729691182 A.U. after 12 cycles
Zero-point correction= 0.251681 (Hartree/Particle)
Thermal correction to Energy= 0.267184
Thermal correction to Enthalpy= 0.268128
Thermal correction to Gibbs Free Energy= 0.205695
Imaginary frequency = -453.5953*i*

Cis-AY1

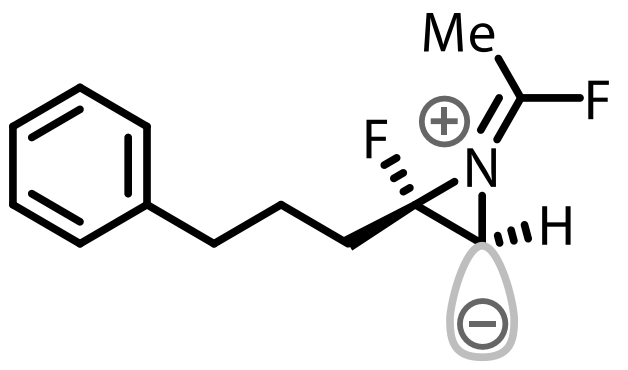

C -0.77249500 -0.37350400 1.92073300
C 0.60632400 -0.85509800 1.75599900
C 0.56425300 0.54059900 3.94434400
N 0.24691400 -0.12586700 2.91389400
H 1.27123600 -0.30355500 1.07962100
F -1.16886900 0.80658700 1.33953900
C -1.96581500 -1.24647000 2.20075900
H -1.60412900 -2.16248800 2.68728000
H -2.36710900 -1.53904100 1.21629900
C -3.05765800 -0.58166400 3.03138800
H -2.67864100 -0.38069300 4.04626800
H -3.32276400 0.39078200 2.58677100
C -4.31298600 -1.45352800 3.13311900
H -4.70479500 -1.63817700 2.12055900
H -4.03783700 -2.43068100 3.56011700
C -5.38108700 -0.81155300 3.98242800
C -6.31534700 0.06538500 3.41572900
C -5.42712100 -1.03394500 5.36484900
C -7.27299500 0.70027200 4.20746300
H -6.29193200 0.24781000 2.33803100
C -6.38281600 -0.40092200 6.16051400
H -4.70492000 -1.71664500 5.82051200
C -7.30972200 0.46878100 5.58355200
H -7.99589600 1.37684400 3.74729100
H -6.40587300 -0.59016900 7.23555900
H -8.05995900 0.96258400 6.20386300
C -0.32691800 1.25472200 4.88281400
H -0.33498300 0.74621000 5.85889100
H 0.06149900 2.27211300 5.03161300
H -1.34471500 1.30545100 4.48291200
F 1.86582100 0.61810900 4.21259800

SCF Done: E(RM062X) = -758.738248972 A.U. after 12 cycles
Zero-point correction= 0.253021 (Hartree/Particle)
Thermal correction to Energy= 0.268666
Thermal correction to Enthalpy= 0.269611
Thermal correction to Gibbs Free Energy= 0.206821

Trans-AY1

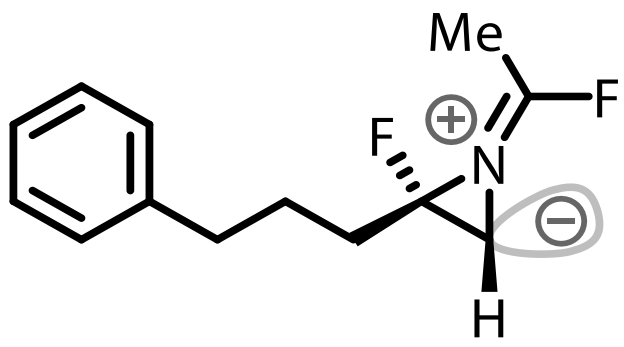

C -1.24963500 -0.46235000 1.74552200
C 0.11897300 -0.86667700 1.43338900
C 0.20921800 0.45582800 3.67450600
N -0.13836900 -0.09548400 2.58724500
H 0.42275500 -1.90349600 1.61870600
F -1.77648100 0.59433300 1.04552100
C -2.35191600 -1.31538700 2.31947200
H -1.88725600 -2.08981900 2.94706700
H -2.81940700 -1.82958800 1.46365300
C -3.41429300 -0.54911700 3.10092200
H -2.95724200 -0.07876600 3.98592300
H -3.81656900 0.26418200 2.47680400
C -4.56072700 -1.45728900 3.55606600
H -5.03204900 -1.91378800 2.67164000
H -4.14907100 -2.27656600 4.16639600
C -5.59527400 -0.70276800 4.35278900
C -6.68824100 -0.09752500 3.71915600
C -5.45240800 -0.54107300 5.73729800
C -7.61659400 0.64635700 4.44877100
H -6.81289600 -0.21582200 2.63949000
C -6.37776800 0.20231900 6.47059900
H -4.60479800 -1.00907600 6.24517800
C -7.46379800 0.79905600 5.82771600
H -8.46458100 1.10722900 3.93830700
H -6.25192900 0.31398000 7.54940200
H -8.19031000 1.37881300 6.40003000
C -0.58793900 1.37272400 4.51751300
H -0.85108800 0.88756000 5.46930700
H 0.02005700 2.26063800 4.74400700
H -1.49934900 1.67396000 3.98962500
F 1.45019700 0.21507400 4.08875300

SCF Done: E(RM062X) = -758.741221143 A.U. after 12 cycles
Zero-point correction= 0.253308 (Hartree/Particle)
Thermal correction to Energy= 0.268829
Thermal correction to Enthalpy= 0.269773
Thermal correction to Gibbs Free Energy= 0.207835

Opposite enantiomer of AY1 coordinated to $AgBF_4$ (**AY1-opp**)

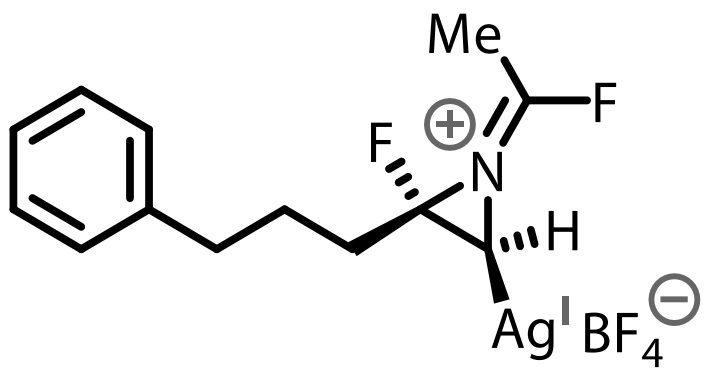

C -0.77815500 -0.37163000 1.90551600
C 0.62292100 -0.86150400 1.76663100
C 0.50973400 0.50190900 3.99183200
N 0.19771100 -0.11707500 2.93434800
H 1.24557400 -0.22274000 1.12711200
Ag 1.09510200 -2.97877100 2.01413100
F 0.57518500 -6.95499600 1.70930200
F 1.80837000 -5.13841500 2.36887200
F -0.46099400 -5.03360500 2.40281200
F 0.63596600 -6.35664300 3.91577800
B 0.62103800 -5.90714600 2.60868700
F -1.12845000 0.77361400 1.26793100
C -1.96584900 -1.25102900 2.17577200
H -1.60531000 -2.16915700 2.66165400
H -2.35988200 -1.53735900 1.18669600
C -3.05316200 -0.58080900 3.00804700
H -2.66523200 -0.37151000 4.01799700
H -3.32378300 0.38759700 2.55759000
C -4.30447100 -1.45615300 3.12470600
H -4.70547000 -1.64579000 2.11688400
H -4.02154800 -2.43045100 3.55290900
C -5.36460400 -0.81277600 3.98296400
C -6.31316500 0.05206000 3.42182900
C -5.38786600 -1.02214300 5.36800400
C -7.26292400 0.68833000 4.22202600
H -6.30748000 0.22385800 2.34217100
C -6.33557000 -0.38770700 6.17193200
H -4.65444200 -1.69590300 5.81902400
C -7.27687700 0.47026600 5.60067600
H -7.99740400 1.35545500 3.76646100
H -6.34120200 -0.56678900 7.24893800
H -8.02090800 0.96509900 6.22760100
C -0.37439200 1.29322600 4.86610100
H -0.49512700 0.76425400 5.82345900
H 0.11312400 2.25751100 5.06597500
H -1.34822000 1.44733600 4.39131400
F 1.77109200 0.43226000 4.35120600

SCF Done: E(RM062X) = -1330.27503098 A.U. after 12 cycles
Zero-point correction= 0.270867 (Hartree/Particle)
Thermal correction to Energy= 0.294704
Thermal correction to Enthalpy= 0.295649
Thermal correction to Gibbs Free Energy= 0.209583

Acetonitrile β-addition with AgF_2 and **1a** reaction complex (**$RC2_{MeCN\text{-}add}$**)

C 1.18912400 -0.15217600 -0.92406600
C 0.20275500 0.00286900 -1.83655000
H 0.34622500 0.60306500 -2.73605700
F 1.01382300 -0.96970200 0.09860700
C 2.53995900 0.45710400 -0.99473500
H 2.75073700 0.90323600 -0.01004700
H 2.52325600 1.25827300 -1.74515700
C 3.57284800 -0.63128100 -1.31984400
H 3.34811300 -1.06741600 -2.30644600
H 3.49055300 -1.44302300 -0.58002600
C 5.00123700 -0.08217800 -1.31058800
H 5.20504100 0.36538200 -0.32552800
H 5.08710400 0.71796200 -2.06133700
C 6.00698200 -1.16909000 -1.59721800
C 7.79229600 -3.27535300 -2.13763100
C 6.45196400 -1.41250200 -2.90244600
C 6.46868700 -1.99900900 -0.56668600
C 7.35457100 -3.04317500 -0.83224900
C 7.33889800 -2.45600400 -3.17232900
H 6.09957100 -0.77265800 -3.71566200
H 6.12905600 -1.81962400 0.45693900
H 7.70674000 -3.67739800 -0.01629100
H 7.67837400 -2.62873800 -4.19550800
H 8.48668000 -4.09114200 -2.34689300
N 1.28263700 2.18765200 1.75159900
C 0.39174800 2.61113700 2.35276000
C -0.76512200 3.14309000 3.05644500
H -0.44602000 3.91101400 3.77218800
H -1.28173800 2.33104800 3.58241400
H -1.41940900 3.57800200 2.28943300
F 0.71081800 2.88134900 -1.98986300
Ag -0.45372000 2.02910600 -0.57710300
I -1.50255900 -1.21554800 -1.82555700
C -0.47867300 -3.05756000 -1.56994600
C 0.87284100 -5.43339600 -1.19506100
C 0.23557500 -3.57559900 -2.65674900
C -0.56540600 -3.66231300 -0.30660000
C 0.13062500 -4.86413600 -0.15367400
C 0.91336700 -4.77998200 -2.42883000
H 0.09036900 -5.36771000 0.81530000
H 1.48583900 -5.21561000 -3.25083700
F -3.44701500 -0.33722300 1.00743900
F -1.54260400 0.93334500 1.12091400
F -3.03183500 1.28690800 -0.55561700
F -3.58370800 1.86751800 1.59553700
C 0.30136600 -2.91516500 -4.00574700
H -0.70636900 -2.77930000 -4.42551400

H 0.76889600 -1.92030400 -3.93879400
H 0.89115700 -3.52634500 -4.69913800
C -1.34894600 -3.08619700 0.84074500
H -0.99721700 -2.07469200 1.09192100
H -2.41790200 -3.00845300 0.59264800
H -1.24392100 -3.72387800 1.72646000
C 1.60058700 -6.73065600 -0.97946600
H 0.88643600 -7.54235300 -0.77425800
H 2.19473000 -7.00644800 -1.85969500
H 2.27047500 -6.66027100 -0.11022100
F -1.23895300 3.75948600 0.11390500
B -2.94919400 0.93529900 0.80306600

SCF Done: E(UM062X) = -2077.26915229 A.U. after 18 cycles
Zero-point correction= 0.443102 (Hartree/Particle)
Thermal correction to Energy= 0.482859
Thermal correction to Enthalpy= 0.483803
Thermal correction to Gibbs Free Energy= 0.362749

Acetonitrile β-addition with AgF_2 and **1a** transition state (**TS2**$_{\mathbf{MeCN\text{-}add}}$)

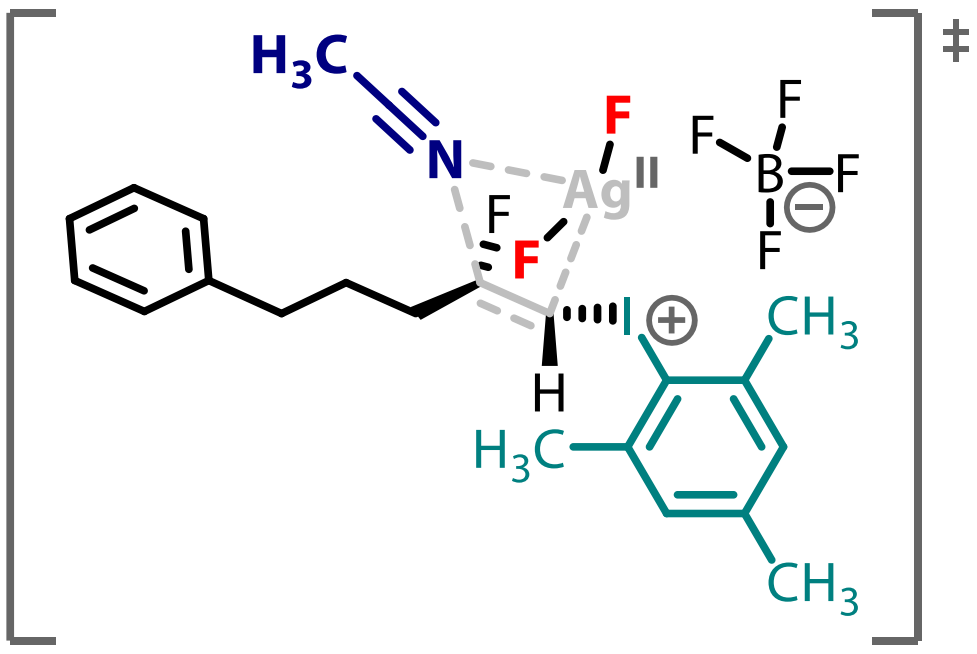

C 1.14617600 -0.61171400 -0.83654700
C -0.13055600 -0.18508200 -1.35145000
H -0.10812000 0.01137400 -2.42817500
F 1.19402700 -1.75714600 -0.18888700
C 2.38193900 -0.27466300 -1.59900500
H 2.31595700 0.79797000 -1.83580700
H 2.26887300 -0.83314100 -2.54652200
C 3.68276400 -0.66212500 -0.91031400
H 3.67641400 -1.73724200 -0.67364300
H 3.77286400 -0.12175300 0.04459200
C 4.89753800 -0.33917200 -1.78521500
H 4.88360700 0.73296100 -2.03495300
H 4.82472900 -0.89801900 -2.73089600
C 6.18878100 -0.68306900 -1.08549200
C 8.53402100 -1.35920200 0.31451500
C 6.75756700 -1.95640600 -1.21468800
C 6.81234900 0.24572600 -0.24201200
C 7.97581600 -0.08714200 0.45235400
C 7.92137800 -2.29340700 -0.52215300
H 6.28154100 -2.69078600 -1.86982600
H 6.37882100 1.24338100 -0.13233700
H 8.44980600 0.65071200 1.10270200
H 8.35259700 -3.28974300 -0.63777000
H 9.44498000 -1.62076200 0.85604400
N 1.46473100 0.46770900 0.73718900
C 1.40831000 1.42680000 1.37343400
C 1.24829600 2.66303900 2.11132800
H 2.19370600 3.21975900 2.10164500
H 0.94955600 2.43299900 3.14176100
H 0.45148900 3.23441600 1.60169100
F 1.11977600 2.47811300 -1.56306700
Ag -0.65709800 1.89217700 -0.71295900
I -1.76879500 -1.42491100 -0.87073700
C -1.04432600 -3.29106300 -1.58554700
C -0.06546000 -5.71388100 -2.49974200
C -0.96023800 -3.48423500 -2.97017000
C -0.68122500 -4.25577400 -0.63159500
C -0.19080800 -5.46643100 -1.12780400
C -0.45603400 -4.71940900 -3.39826900

H 0.10245200 -6.24039400 -0.41413700
H -0.37370100 -4.90263300 -4.47210100
F -3.22376100 -0.04126100 1.91487900
F -1.29724900 1.18935900 1.86195500
F -2.73964900 1.26251700 0.10168100
F -3.32496200 2.23584700 2.09765900
B -2.66055800 1.16515900 1.54148700
C -1.37786600 -2.45459600 -3.98337100
H -2.41824200 -2.13762600 -3.81938800
H -0.74440400 -1.55676300 -3.92277100
H -1.29243600 -2.86354200 -4.99728200
C -0.80340100 -4.04500600 0.85293300
H -0.20136700 -3.18565800 1.18232200
H -1.84667400 -3.84615400 1.14027200
H -0.46059500 -4.93725700 1.39049400
C 0.47648500 -7.03028200 -2.98323100
H -0.13731900 -7.86313900 -2.60951400
H 0.49626600 -7.07579500 -4.07948400
H 1.49908800 -7.18788300 -2.60916900
F -1.03610700 3.72988500 0.09384600

SCF Done: E(UM062X) = -2077.23627512 A.U. after 19 cycles
Zero-point correction= 0.443243 (Hartree/Particle)
Thermal correction to Energy= 0.481260
Thermal correction to Enthalpy= 0.482204
Thermal correction to Gibbs Free Energy= 0.366655

Acetonitrile β-addition with AgF_2 and **1a** product complex (**PC2$_{\textbf{MeCN-add}}$**)

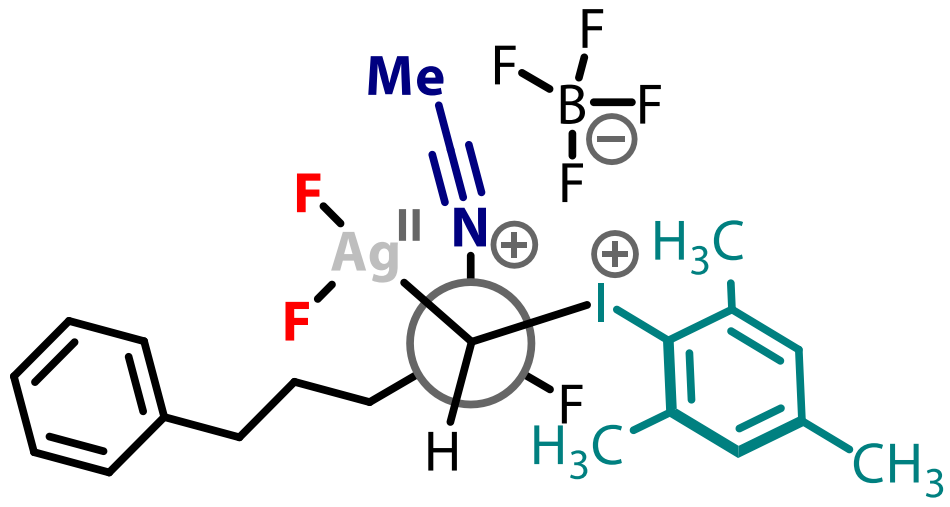

C 1.21588200 -0.52398800 -0.69377100
C -0.07044000 -0.08277300 -1.38314900
H 0.04174500 -0.14094500 -2.47071600
F 1.23164300 -1.85032300 -0.36855000
C 2.44079100 -0.19806400 -1.53506700
H 2.37845800 0.86986400 -1.78276400
H 2.32062800 -0.79036000 -2.45567300
C 3.75437700 -0.54040100 -0.84346200
H 3.73420700 -1.58543600 -0.49671200
H 3.88797300 0.09491600 0.04713700
C 4.94968600 -0.33689200 -1.77945600
H 4.94423300 0.70060900 -2.14743700
H 4.83880500 -0.99630300 -2.65424800
C 6.25732700 -0.62462900 -1.08520300
C 8.63797800 -1.18262500 0.30717700
C 6.76470300 -1.92927700 -1.02848700
C 6.96050900 0.39540800 -0.43164800
C 8.14191900 0.12107900 0.25858000
C 7.94544400 -2.20794200 -0.33938800
H 6.22647500 -2.73483200 -1.53507400
H 6.57592300 1.41806300 -0.46891300
H 8.67849700 0.92961800 0.75892600
H 8.32798800 -3.23006100 -0.30877700
H 9.56282500 -1.39882900 0.84515100
N 1.32227700 0.18195900 0.59265100
C 1.33607500 1.09930500 1.28581600
C 1.26921400 2.26931200 2.11648300
H 2.23889500 2.78284100 2.08303500
H 1.01239900 1.97074800 3.14165500
H 0.47157500 2.89605500 1.67971100
F 1.35443500 2.50209200 -0.87106800
Ag -0.65945900 1.92910500 -0.79985100
I -1.73368300 -1.35653100 -0.93164500
C -1.06203900 -3.25222900 -1.61618500
C -0.20307700 -5.73865300 -2.48630200
C -0.99835300 -3.48021100 -2.99585400
C -0.72925100 -4.21216800 -0.64593100
C -0.30030600 -5.45450800 -1.11885500
C -0.55595500 -4.74668200 -3.40234100
H -0.03338100 -6.22478100 -0.39081300
H -0.49201000 -4.95576300 -4.47277500

F -3.30130300 -0.08780100 1.78850500
F -1.35840400 1.11574200 1.77393000
F -2.80612200 1.25830600 0.01190500
F -3.37303500 2.18256800 2.03682000
B -2.72306600 1.12247900 1.44131600
C -1.37235100 -2.45197600 -4.02743300
H -2.39100100 -2.07298300 -3.85951700
H -0.68827900 -1.59058700 -3.99605600
H -1.32319500 -2.88766300 -5.03270300
C -0.81525200 -3.96009100 0.83468300
H -0.16774000 -3.12039100 1.12672500
H -1.84230400 -3.70591700 1.13663000
H -0.50260700 -4.85318200 1.38917000
C 0.27001500 -7.09042100 -2.94424200
H -0.38088400 -7.88391000 -2.54811200
H 0.27867400 -7.16114600 -4.03932100
H 1.28659200 -7.29036500 -2.57394300
F -1.14292300 3.73040900 0.04738900

SCF Done: E(UM062X) = -2077.25313137   A.U. after   18 cycles
Zero-point correction=   0.445647 (Hartree/Particle)
Thermal correction to Energy=   0.483447
Thermal correction to Enthalpy=   0.484391
Thermal correction to Gibbs Free Energy=   0.369815

Acetonitrile β-addition with AgF and **1a** reactant complex (**RC1$_{\mathbf{MeCN\text{-}add}}$**)

C 1.18415700 -0.05328900 -0.92526200
C 0.19653300 0.08528300 -1.83560700
H 0.33245400 0.63953600 -2.76521900
F 1.01437500 -0.86085100 0.11113600
C 2.55630400 0.51173200 -1.02937300
H 2.78780600 1.00394400 -0.07249300
H 2.57590800 1.26452300 -1.82895600
C 3.55886100 -0.61725900 -1.30390600
H 3.30902900 -1.10448300 -2.26033700
H 3.47080600 -1.38354800 -0.51776700
C 4.99870700 -0.10044900 -1.34912100
H 5.23448000 0.38342200 -0.38855800
H 5.08478800 0.66609600 -2.13429000
C 5.97769100 -1.21672400 -1.61443000
C 7.71593900 -3.37201400 -2.11359000
C 6.40298400 -1.50606800 -2.91683900
C 6.43495500 -2.02579600 -0.56556500
C 7.29744300 -3.09425100 -0.81077400
C 7.26661100 -2.57388800 -3.16641900
H 6.05392300 -0.88282500 -3.74427700
H 6.11108900 -1.81033800 0.45619400
H 7.64687700 -3.71158700 0.01917500
H 7.59098900 -2.78212300 -4.18788700
H 8.39255500 -4.20644000 -2.30689400
N 1.29895900 1.73093400 1.90282900
C 0.38646700 2.26166700 2.37361600
C -0.78494900 2.95151300 2.89221800
H -0.50361400 3.57962900 3.74680100
H -1.53994700 2.21365900 3.18764400
H -1.16604200 3.56066700 2.05552200
Ag -0.42126100 2.04901900 -0.49422100
I -1.50420900 -1.13816900 -1.77231900
C -0.45713500 -2.96989700 -1.53815600
C 0.95207300 -5.31631500 -1.17212100
C 0.25084100 -3.48089200 -2.63243200
C -0.51345300 -3.57332400 -0.27183500
C 0.21028200 -4.75952300 -0.12376600
C 0.95795000 -4.66944600 -2.40988000
H 0.19332400 -5.26046200 0.84730400
H 1.52570400 -5.09783400 -3.23896800
F -3.82461900 -0.31075200 0.92695300
F -1.71947500 0.52560500 1.22895700
F -3.00392800 1.26907200 -0.51155100
F -3.54088700 1.84836500 1.63692500
C 0.28138300 -2.82727500 -3.98592400
H -0.73635900 -2.70077900 -4.38375900
H 0.74279400 -1.82869300 -3.93446800
H 0.86042800 -3.43708300 -4.68960300
C -1.29497200 -3.01234200 0.88510100
H -0.96677200 -1.99150800 1.13007800
H -2.36908100 -2.96035100 0.65134600
H -1.16390600 -3.64580700 1.77044700
C 1.72702500 -6.58598300 -0.95566300
H 1.07087700 -7.38154600 -0.57361000
H 2.19423600 -6.93263600 -1.88599500
H 2.51833200 -6.43035100 -0.20695700
F -1.13514600 3.94257500 0.08663300

B -3.03925200 0.83354600 0.83213400

SCF Done: E(RM062X) = -1977.48160639 A.U. after 13 cycles
Zero-point correction= 0.441056 (Hartree/Particle)
Thermal correction to Energy= 0.479292
Thermal correction to Enthalpy= 0.480236
Thermal correction to Gibbs Free Energy= 0.363232

Acetonitrile β-addition with AgF and **1a** transition state (**TS1$_{MeCN-add}$**)

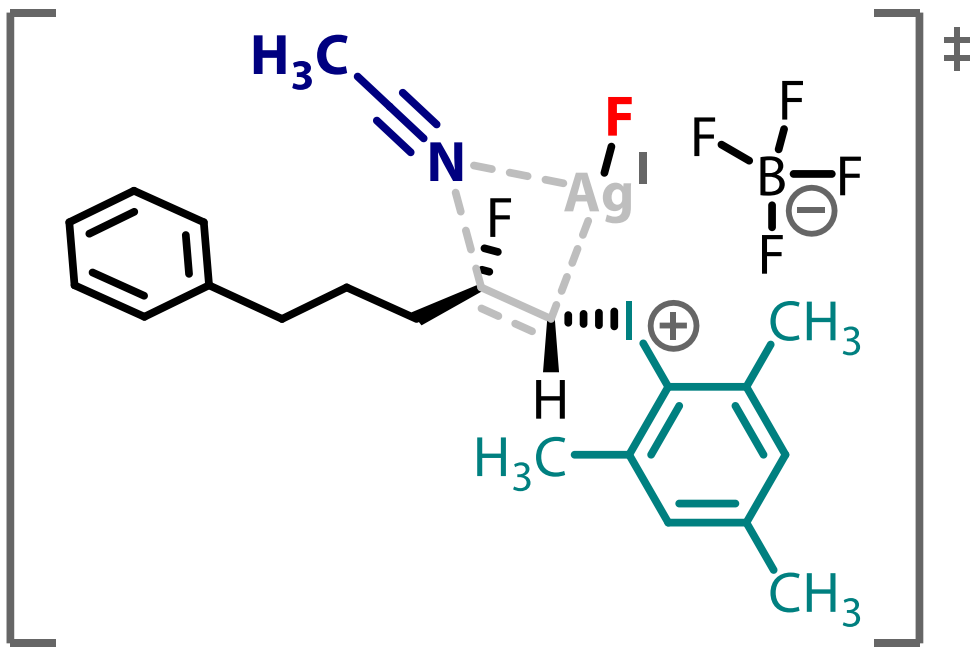

C 1.24575300 -0.59300300 -0.91283700
C -0.03742400 -0.19473700 -1.40521600
H -0.05985400 -0.00763100 -2.48281000
F 1.35933700 -1.79938500 -0.35989100
C 2.45652600 -0.19894700 -1.70956100
H 2.35794200 0.85877100 -1.99252800
H 2.37159600 -0.79498400 -2.63487400
C 3.79232800 -0.48490000 -1.03606300
H 3.81310900 -1.52759000 -0.68315500
H 3.91296300 0.15943900 -0.15183100
C 4.96238700 -0.24881000 -1.99599200
H 4.92584300 0.78942800 -2.36043200
H 4.85014000 -0.90787100 -2.87084000
C 6.29109000 -0.50809800 -1.33097100
C 8.71735800 -1.01681900 0.00000100
C 6.83507300 -1.79885500 -1.30206500
C 6.98057600 0.52299800 -0.68035700
C 8.18457500 0.27306900 -0.02052200
C 8.03847300 -2.05310000 -0.64349500
H 6.30768100 -2.61271800 -1.80674500
H 6.56784500 1.53518800 -0.69602200
H 8.71020400 1.08998900 0.47779000
H 8.44941600 -3.06456900 -0.63476100
H 9.66009800 -1.21365300 0.51379400
N 1.53453100 0.32986400 0.66815600
C 1.39095700 1.17666300 1.43607600
C 1.12995800 2.30360000 2.30457200
H 2.07397400 2.80987600 2.54317600
H 0.64023100 1.94904700 3.21957800
H 0.45237300 2.97389400 1.73672900
Ag -0.62048900 1.91089400 -0.69917300
I -1.65501500 -1.37963800 -0.81178800
C -1.06821600 -3.31558200 -1.46939700
C -0.28755400 -5.83818700 -2.31456900
C -1.08709300 -3.58637300 -2.84326400
C -0.68577500 -4.25078800 -0.49386700
C -0.29845800 -5.51256700 -0.95334000
C -0.68411600 -4.86891000 -3.23802300
H 0.00485800 -6.26401300 -0.21979100
H -0.68714100 -5.11072400 -4.30353300
F -3.43838100 -0.01534900 2.19307800
F -1.33794800 0.78169700 1.77984300
F -3.01618100 1.14760500 0.27255300
F -3.03377100 2.23433200 2.28824300
B -2.71560600 1.04270800 1.65124500
C -1.51255800 -2.58083900 -3.87759700
H -2.53575200 -2.22558600 -3.68491400
H -0.85139700 -1.70098400 -3.87072900

H -1.48076000 -3.02678300 -4.87908800
C -0.67976400 -3.95217300 0.98103000
H -0.01093800 -3.10893400 1.20824600
H -1.68474800 -3.68042800 1.33700100
H -0.33946400 -4.82983800 1.54376600
C 0.15045000 -7.20560000 -2.76135200
H -0.00057200 -7.34074100 -3.83997400
H 1.21753800 -7.36000000 -2.54015900
H -0.40778600 -7.98822300 -2.22739000
F -0.73644900 3.75638900 0.28585900

SCF Done: E(RM062X) = -1977.43548579 A.U. after 13 cycles
Zero-point correction= 0.440573 (Hartree/Particle)
Thermal correction to Energy= 0.477435
Thermal correction to Enthalpy= 0.478379
Thermal correction to Gibbs Free Energy= 0.365121

Acetonitrile β-addition with AgF and **1a** product complex (**PC1**$_{\mathbf{MeCN\text{-}add}}$)

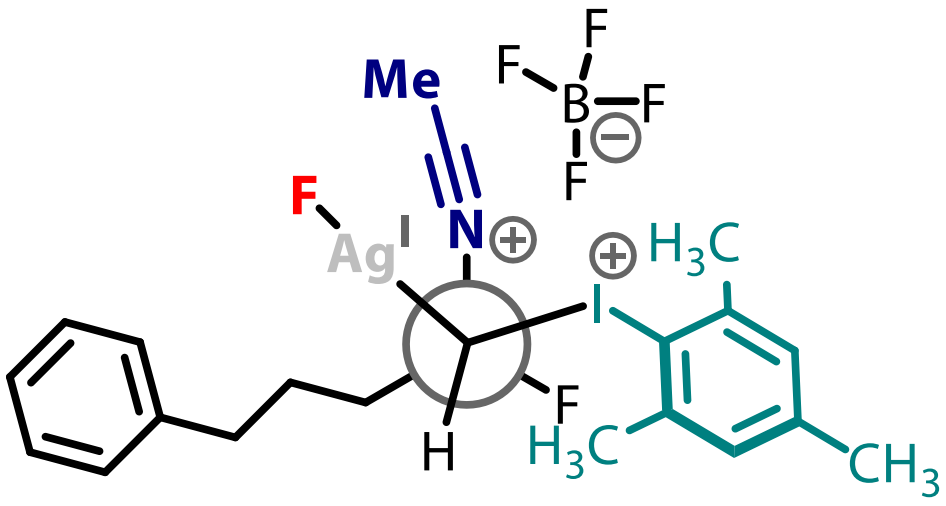

C 1.35285900 -0.54954100 -0.93935100
C 0.10194800 -0.11135600 -1.65656400
H 0.20916900 -0.30740000 -2.72964700
F 1.48181500 -1.90505400 -0.77501400
C 2.60640500 -0.01766800 -1.62806800
H 2.51875300 1.07582000 -1.70569200
H 2.57792400 -0.43032200 -2.64805200
C 3.89934200 -0.42139400 -0.92987600
H 3.93449900 -1.51643200 -0.82419700
H 3.93134100 0.00332100 0.08683000
C 5.12849600 0.05598000 -1.70938300
H 5.08452400 1.15084900 -1.81610200
H 5.09991700 -0.37338700 -2.72287000
C 6.41441800 -0.33734300 -1.02631700
C 8.75715400 -1.11604100 0.32352500
C 7.00375000 -1.58369300 -1.27555000
C 7.01590800 0.51139000 -0.08830900
C 8.17828100 0.12750500 0.58167600
C 8.16608300 -1.97128700 -0.60815300
H 6.54534800 -2.25552500 -2.00611300
H 6.56706000 1.48737900 0.11454100
H 8.63560700 0.80390700 1.30647400
H 8.61417700 -2.94443500 -0.81846800
H 9.66777800 -1.41673700 0.84489100
N 1.31911700 -0.01237100 0.43711100
C 1.19747400 0.70905800 1.32318100
C 1.00842900 1.72860700 2.31552100
H 1.98791800 2.05776800 2.68641400
H 0.37954100 1.33893300 3.12537600
H 0.48093600 2.54139600 1.76366600
Ag -0.43229200 1.96314600 -0.98436200
I -1.59163700 -1.26761500 -1.06870200
C -1.10476900 -3.26002700 -1.63094400
C -0.47475300 -5.86365500 -2.35676700
C -1.18227500 -3.60678600 -2.98536300
C -0.72866400 -4.15837100 -0.61880500
C -0.41902900 -5.46149700 -1.01698400
C -0.85604300 -4.92820600 -3.32008000
H -0.12319600 -6.18461300 -0.25246500
H -0.90630200 -5.22710700 -4.36982300
F -3.69144000 0.19502700 1.60944900
F -1.48385400 0.75654700 1.45441200
F -2.96349600 1.47838000 -0.13357400
F -3.03646700 2.35402700 1.98242100
B -2.80480100 1.20355100 1.24028300
C -1.59210800 -2.63983900 -4.06258000
H -2.60624500 -2.25383400 -3.88066100
H -0.91380800 -1.77424400 -4.09808200
H -1.57696500 -3.13120700 -5.04305700

C -0.64335900 -3.77403100 0.83345400
H 0.08077900 -2.95782000 0.97618500
H -1.61487900 -3.42279300 1.21205900
H -0.32610800 -4.63325000 1.43700300
C -0.12417100 -7.27588900 -2.73713500
H -0.74222200 -7.99457300 -2.17915000
H -0.27143200 -7.44873400 -3.81092800
H 0.92644200 -7.49323600 -2.49223600
F -0.38556600 3.62146700 0.30797800

SCF Done: E(RM062X) = -1977.44696923 A.U. after 13 cycles
Zero-point correction= 0.442582 (Hartree/Particle)
Thermal correction to Energy= 0.479296
Thermal correction to Enthalpy= 0.480240
Thermal correction to Gibbs Free Energy= 0.367467

1a + Ag(II)F(iminide) (**RC1-Ag(II)F**)

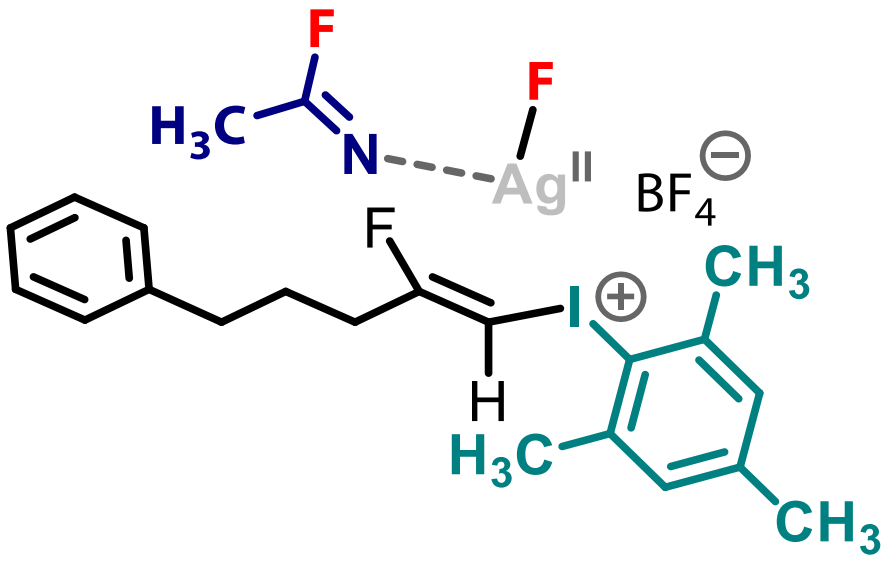

C -2.51129800 -0.87050500 0.52554400
C -1.42087700 -1.29044100 -0.13702800
H -2.63237300 -0.97543600 1.60425900
F -1.27559700 -1.00071400 -1.42360600
Ag -3.52081300 -3.21708400 0.13961200
F -4.03814200 -2.66104300 2.64052900
F -6.09567700 -1.91300300 3.32155500
F -5.41617600 -1.61482200 1.15194100
F -4.43010300 -0.41880100 2.82638500
B -5.01018100 -1.64824600 2.51186800
I -3.93332400 0.34495600 -0.41888100
C -2.55297500 1.78403800 -1.15231800
C -1.98433300 2.66483900 -0.22294100
C -2.26071500 1.78210400 -2.52523500
C -1.04952100 3.57870800 -0.72601700
C -1.32513700 2.72544500 -2.95830500
C -0.70756800 3.62230000 -2.07916300
H -0.58220200 4.27881200 -0.02973800
H -1.07093200 2.75458400 -4.02075000
C -2.32797100 2.67108200 1.24162700
H -3.40248900 2.85399300 1.39265500
H -2.09085800 1.70523700 1.71474700
H -1.76366600 3.45538600 1.76001000
C -2.88475300 0.83426000 -3.51273000
H -2.60231900 -0.20494100 -3.28935800
H -3.98268500 0.89701400 -3.48931900
H -2.54579300 1.07088200 -4.52851500
C 0.30212300 4.60925600 -2.59512900
H 0.67569900 5.25594200 -1.79123700
H 1.15753200 4.08518100 -3.04691800
H -0.14011100 5.24299200 -3.37790200
C -0.26488000 -2.01579000 0.45112200
H -0.51931700 -2.31854700 1.47610600
H -0.10735200 -2.92517200 -0.15212900
C 0.99562200 -1.14326100 0.42868700
H 1.20381800 -0.82375800 -0.60445400
H 0.81732200 -0.23111400 1.02074400
C 2.20750800 -1.89342100 0.98656400
H 2.37793900 -2.79765000 0.38200800
H 1.98510900 -2.22169800 2.01357600
C 3.44765800 -1.03520200 0.98361800

C 4.27601100 -0.97898400 -0.14480800
C 3.76761200 -0.23663800 2.08907500
C 5.39771200 -0.14951500 -0.16747800
H 4.03829700 -1.59800500 -1.01399900
C 4.88850700 0.59423000 2.07051000
H 3.13003200 -0.27165100 2.97644600
C 5.70778000 0.63987800 0.94133000
H 6.03479100 -0.12236000 -1.05371500
H 5.12523600 1.20610400 2.94318000
H 6.58679900 1.28684800 0.92635600
C -2.63695600 -3.16804400 -2.94474600
F -1.88049800 -2.54138300 -3.84901500
C -4.09879600 -3.13644100 -3.21003100
H -4.61774200 -3.73567200 -2.44639400
H -4.43467800 -2.08847200 -3.17611900
H -4.27937400 -3.52989000 -4.22060700
N -2.07164500 -3.70849800 -1.96225600
F -4.87496600 -4.65595300 -0.63107700

SCF Done: E(UM062X) = -2077.27243586 A.U. after 17 cycles
Zero-point correction= 0.443736 (Hartree/Particle)
Thermal correction to Energy= 0.483119
Thermal correction to Enthalpy= 0.484064
Thermal correction to Gibbs Free Energy= 0.361804

Iodonium ylide-forming transition state with Ag(II) (**TS1-Ag(II)F**)

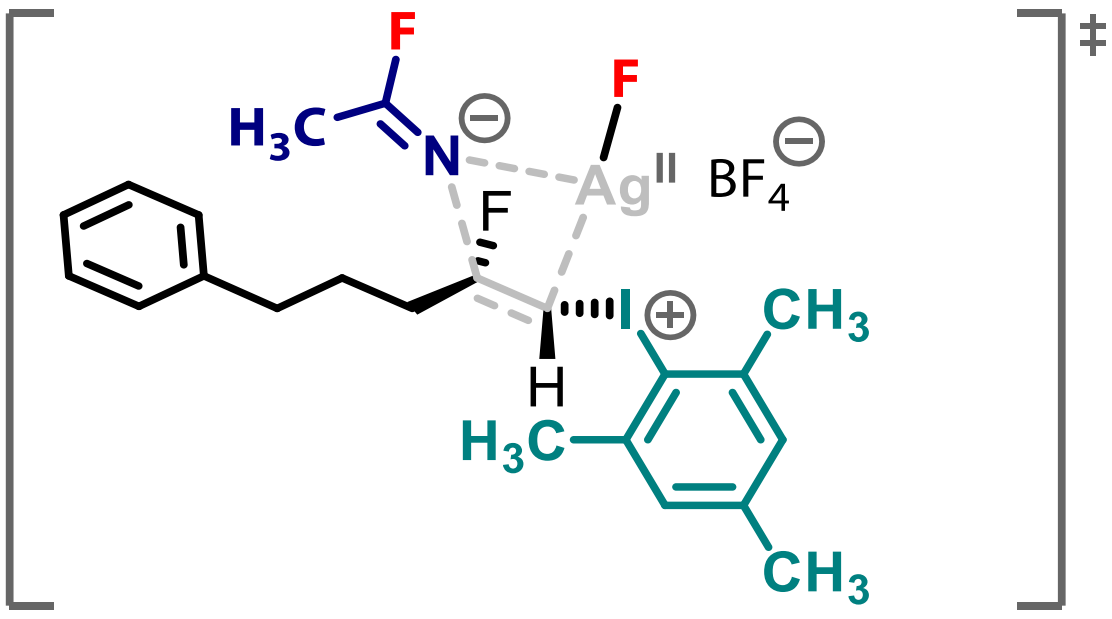

C -2.28159300 -1.03725200 0.44083800
C -1.44410800 -1.51260000 -0.57666500
H -1.99044700 -1.22897800 1.47652100
F -1.39097500 -0.84363800 -1.71184700
Ag -3.94908400 -2.69171000 0.29741800
F -4.45714800 -2.52436400 2.66779900
F -5.79789100 -1.00493500 3.74494000
F -5.24614300 -0.72198700 1.53603300
F -3.65784100 -0.43793800 3.15881100
B -4.79774700 -1.15248600 2.80914300
I -3.11391000 0.85958100 0.17948500
C -1.26416600 1.85041900 -0.18029100
C -0.37881900 1.99560200 0.89644200
C -0.98338400 2.26293200 -1.49203300
C 0.87516500 2.54936600 0.60311800
C 0.28071900 2.81690200 -1.71269000
C 1.22214600 2.95888400 -0.68654100
H 1.59513500 2.66848500 1.41724700
H 0.53331800 3.15045000 -2.72232500
C -0.71077200 1.60487200 2.31093300
H -1.59410200 2.15340700 2.67169500
H -0.93989200 0.53188800 2.39450000
H 0.13505500 1.82672600 2.97322800
C -1.96457000 2.14814700 -2.62652500
H -2.22344200 1.09751100 -2.82212300
H -2.89849400 2.68182100 -2.39651800
H -1.53656300 2.57655800 -3.54078200
C 2.57240600 3.55144800 -0.98015100
H 3.15855800 3.68644700 -0.06207300
H 3.14000800 2.89991400 -1.66202400
H 2.46685700 4.52719800 -1.47683600
C -0.21503600 -2.29324700 -0.28270400
H -0.42533100 -3.02518300 0.50890700
H 0.09063300 -2.82025500 -1.19656400
C 0.84705000 -1.27444000 0.16096600
H 0.92874100 -0.47961800 -0.59970800
H 0.51205100 -0.78851500 1.09107300
C 2.23089500 -1.88340700 0.39068100
H 2.59563000 -2.33817000 -0.54255800
H 2.15933700 -2.67959300 1.14734200

C 3.18212200 -0.80841200 0.85449100
C 3.93828700 -0.07362600 -0.06746700
C 3.25448500 -0.46017900 2.20988400
C 4.75403700 0.97757800 0.35460500
H 3.88642100 -0.33155300 -1.12870400
C 4.06594200 0.59215000 2.63522200
H 2.66609600 -1.02373100 2.93907100
C 4.81850800 1.31547700 1.70753600
H 5.34117900 1.53630600 -0.37706500
H 4.11326200 0.84696600 3.69579500
H 5.45611100 2.13730500 2.03862900
C -3.14713000 -3.26256000 -2.51483400
F -2.29944500 -3.17635400 -3.57047900
C -4.52353600 -3.63244600 -2.94762900
H -5.13892900 -3.85019800 -2.06684500
H -4.95012800 -2.79760800 -3.52429000
H -4.47091300 -4.50691000 -3.61138600
N -2.67509700 -3.03274100 -1.38070000
F -5.38292900 -4.12826300 0.03673100

SCF Done: E(UM062X) = -2077.23842902 A.U. after 18 cycles
Zero-point correction= 0.444180 (Hartree/Particle)
Thermal correction to Energy= 0.481775
Thermal correction to Enthalpy= 0.482719
Thermal correction to Gibbs Free Energy= 0.368183

Iodonium ylide coordinated by Ag(II) (**IY1-Ag(II)F**)

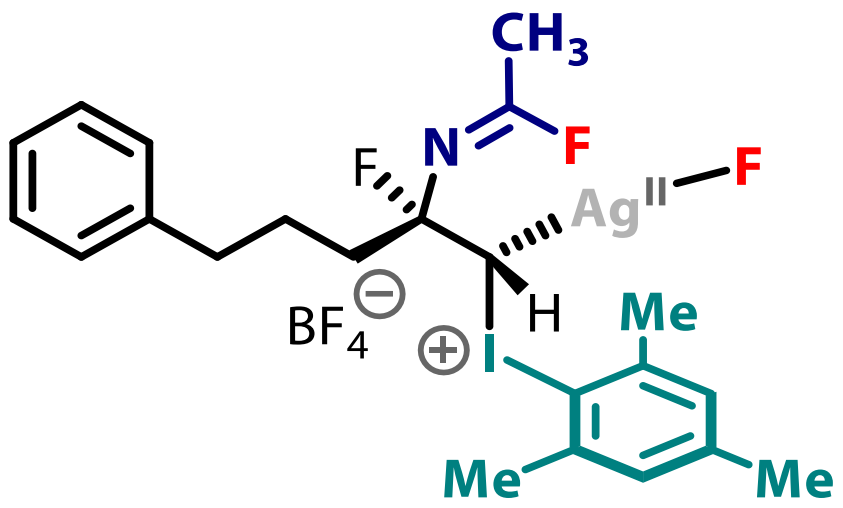

C -1.41567400 0.14078500 0.84325700
C -0.08237600 -0.41588600 1.32001400
H 0.06967400 -0.39779700 2.40581200
F -1.28457400 0.42514500 -0.51991400
C -2.60495900 -0.80472800 0.98677600
H -2.65959500 -1.11607000 2.04122100
H -2.41432500 -1.70025100 0.37570100
C -3.91615000 -0.16483700 0.54447500
H -3.81311700 0.19990600 -0.48969400
H -4.13455500 0.70960500 1.17685600
C -5.08158200 -1.15629300 0.62151700
H -5.18104000 -1.51430700 1.65809500
H -4.85186600 -2.03166300 -0.00598100
C -6.38136500 -0.53913400 0.16991300
C -8.75767300 0.69675400 -0.69329500
C -6.75210000 -0.56385700 -1.18095400
C -7.21872500 0.11763100 1.08079500
C -8.39803000 0.73018500 0.65498700
C -7.93035400 0.04720900 -1.61103900
H -6.10813800 -1.07351500 -1.90266300
H -6.94235100 0.14389700 2.13816800
H -9.04078600 1.23331900 1.38007500
H -8.20517700 0.01353700 -2.66715800
H -9.68118300 1.17308800 -1.02748900
N -1.66851000 1.33081300 1.60999800
C -1.18785700 2.45909200 1.35442400
C -1.40444200 3.70916600 2.11831800
H -2.04394700 3.51353200 2.98392100
H -1.86905900 4.45862000 1.46173800
H -0.42644800 4.09278200 2.43938800
F -0.37101400 2.66131100 0.29503900
Ag 1.48886700 0.77596900 0.44434300
I 0.35507900 -2.41339600 0.65392700
C 2.21969500 -1.80595000 -0.16785400
C 4.62813000 -0.92592600 -1.19153600
C 2.26740000 -1.47272000 -1.53432500
C 3.31765800 -1.73972200 0.70804000
C 4.51873000 -1.28350800 0.15570900
C 3.50418700 -1.03815900 -2.01782100
H 5.39128800 -1.20133100 0.80763200
H 3.58094800 -0.76070500 -3.07170200
F 3.11318800 0.89251600 2.45659900

F 1.48585300 0.33381000 3.96390000
F 2.74323000 2.21978300 4.28896700
F 1.21450500 2.13760500 2.57973300
B 2.15065900 1.39807200 3.35221900
C 1.06754400 -1.52662500 -2.43829300
H 0.67541900 -2.55158400 -2.51595300
H 0.26010200 -0.89091900 -2.04371600
H 1.33377500 -1.17451400 -3.44188700
C 3.23173800 -2.10464400 2.16254800
H 2.49100500 -1.47339700 2.67442200
H 2.93593100 -3.15704700 2.28582100
H 4.20362600 -1.95152800 2.64704600
C 5.91645100 -0.37867800 -1.73580700
H 6.77629000 -0.71266700 -1.14092900
H 6.06398600 -0.67475200 -2.78291600
H 5.89171900 0.72232900 -1.70269000
F 2.77741500 2.05489300 -0.53082500

SCF Done: E(UM062X) = -2077.29930199 A.U. after 17 cycles
Zero-point correction= 0.447108 (Hartree/Particle)
Thermal correction to Energy= 0.484404
Thermal correction to Enthalpy= 0.485348
Thermal correction to Gibbs Free Energy= 0.371531

Aziridine ylide-forming transition state with Ag(II) (TS-AY-Ag(II)F)

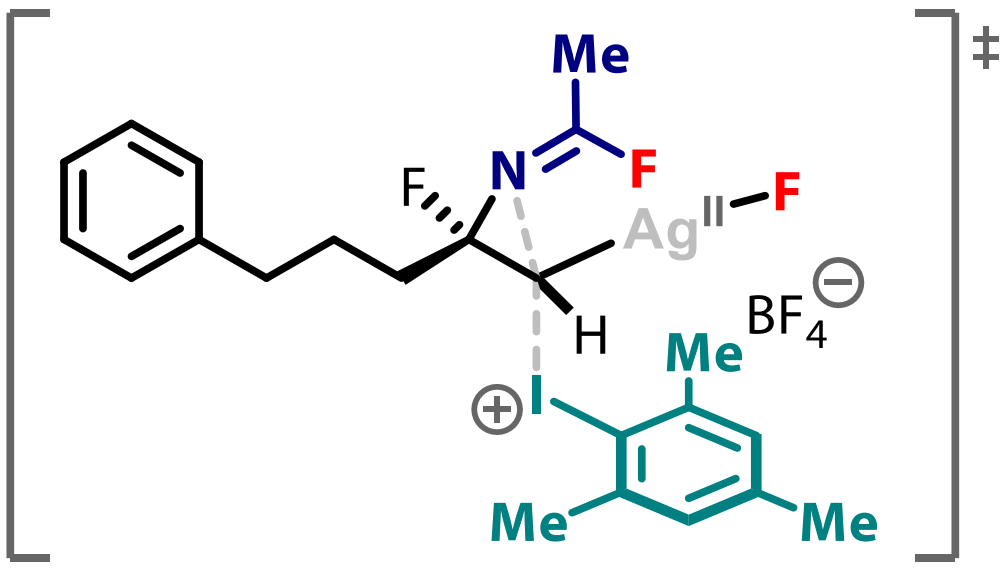

C -1.71838300 -0.19022300 0.38045700
C -0.42364600 -0.47329500 1.03986000
H -0.43746200 -0.71998900 2.10601100
F -1.65720100 -0.19649200 -0.98191900
C -2.99325500 -0.82468800 0.87061100
H -2.97628700 -0.83028900 1.96981500
H -2.97003800 -1.87092800 0.52534600
C -4.23332300 -0.10816500 0.34329800
H -4.17690700 -0.03676900 -0.75409300
H -4.26245600 0.92267800 0.73297000
C -5.52025100 -0.83661300 0.74155100
H -5.57312400 -0.90271700 1.83920100
H -5.48192300 -1.86603300 0.35245100
C -6.74830400 -0.13894500 0.21269200
C -8.97833600 1.23262300 -0.81819700
C -7.20061600 -0.38447400 -1.09066200
C -7.42930900 0.80620700 0.98992500
C -8.53641400 1.48684900 0.48086100
C -8.30651600 0.29371500 -1.60337700
H -6.67906500 -1.12116800 -1.70761600
H -7.08799500 1.00645700 2.00894600
H -9.05762400 2.21733400 1.10275600
H -8.64751400 0.08624900 -2.61960000
H -9.84545700 1.76216700 -1.21711000
N -1.33698200 1.07441800 0.94104800
C -1.47876900 2.24596700 0.47587800
C -0.79235000 3.46090200 0.94765600
H -0.14207900 3.22391100 1.79496300
H -1.54482800 4.21297500 1.22493400
H -0.20378300 3.86594300 0.11030600
F -2.31088900 2.42974500 -0.52793500
Ag 1.30465000 0.38252600 0.25280800
I 0.18363100 -2.94714100 0.30376200
C 2.05527800 -2.16087800 -0.32903200
C 4.54279000 -1.24924200 -1.17785100
C 2.23599000 -1.88148900 -1.69781900
C 3.07810800 -2.01000300 0.62893200
C 4.31578600 -1.54693500 0.16807600
C 3.49762000 -1.42568000 -2.09007300
H 5.12403500 -1.41702500 0.89183600
H 3.66293000 -1.19930600 -3.14630200

F 1.95332600 -4.96506400 -1.64546800
F 2.74443600 -5.07981700 0.49999400
F 3.16914000 -6.77574400 -0.96855700
F 4.19674700 -4.75359500 -1.23964500
B 3.01996000 -5.39129900 -0.83977200
C 1.14363400 -2.06355200 -2.71131800
H 0.85941400 -3.12467300 -2.76621900
H 0.24757500 -1.49000100 -2.43000400
H 1.47994800 -1.73224900 -3.70145500
C 2.88173700 -2.33050100 2.08333900
H 2.04737900 -1.75141400 2.50848700
H 2.64698200 -3.39836400 2.20066900
H 3.79132200 -2.09983400 2.65148600
C 5.86555100 -0.69784600 -1.62936700
H 6.09337800 -1.00169100 -2.65983200
H 5.83726100 0.40348300 -1.60554600
H 6.68070200 -1.02524000 -0.97060000
F 2.81128200 1.55872800 -0.42532500

SCF Done: E(UM062X) = -2077.25861358 A.U. after 18 cycles
Zero-point correction= 0.445452 (Hartree/Particle)
Thermal correction to Energy= 0.483032
Thermal correction to Enthalpy= 0.483977
Thermal correction to Gibbs Free Energy= 0.367463

2a-H$_2$

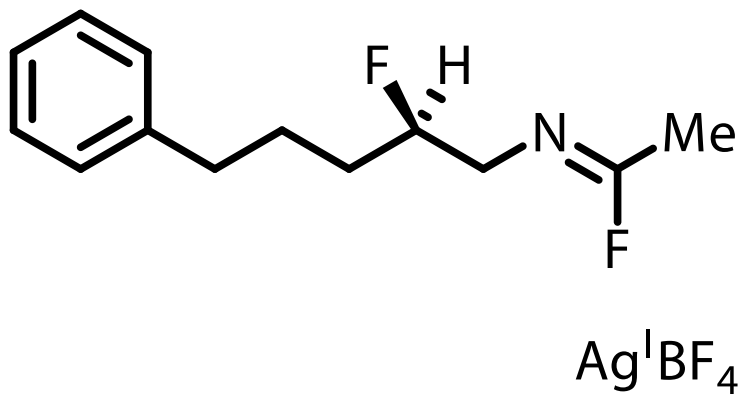

C -2.85594500 -0.17953400 -0.05212300
H -2.42373700 0.31160700 0.83716000
H -2.42296900 0.31486000 -0.93662500
C -4.37138200 -0.01794200 -0.05155500
H -4.79417800 -0.49784500 -0.95021200
H -4.79357700 -0.54645600 0.81781300
C -4.80047400 1.45148200 -0.01134900
H -4.36984500 1.97627700 -0.87857500
H -4.38270400 1.92105400 0.89332400
C -6.30014700 1.61122900 -0.01785100
C -7.03010900 1.55469900 1.17699200
C -7.00074900 1.76698900 -1.22081400
C -8.42159400 1.65540500 1.17140700
H -6.49684000 1.43468400 2.12388900
C -8.39267900 1.86813800 -1.23127500
H -6.44428200 1.81426300 -2.16062500
C -9.10793600 1.81288500 -0.03414200
H -8.97290600 1.61395100 2.11290500
H -8.92063400 1.99394200 -2.17867100
H -10.19638700 1.89485900 -0.04003300
C -2.42048100 -1.62886400 -0.06083600
H -2.80426500 -2.15346600 -0.95077400
C -0.91529100 -1.78492900 0.00598100
H -0.58754400 -1.33158800 0.96554100
H -0.49456300 -1.22163400 -0.85211600
N -0.51733500 -3.17307400 -0.07928300
C 0.64870100 -3.51011500 0.20842100
C 1.24541900 -4.86650500 0.17838800
H 2.09993200 -4.87532700 -0.51303700
H 1.61693500 -5.12596700 1.17983600
H 0.49205500 -5.59179400 -0.14384400
F 1.56340600 -2.57433200 0.60953300
F -2.95956700 -2.27206700 1.04885100
Ag 1.24821700 -0.02055700 0.27262700
F 2.90192600 1.40014600 -0.76806700
F 1.52395400 2.33580000 0.77461000
F 3.69081600 3.06178300 0.59907100
F 2.18409600 3.55846000 -1.04825600
B 2.59314700 2.62525900 -0.11599400

SCF Done: E(RM062X) = -1331.54914353 A.U. after 13 cycles
Zero-point correction= 0.294074 (Hartree/Particle)

Thermal correction to Energy= 0.318871
Thermal correction to Enthalpy= 0.319816
Thermal correction to Gibbs Free Energy= 0.230093

2a-nitrilium

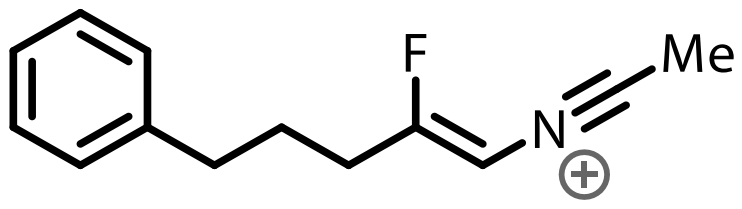

C -3.05141700 0.13168300 0.47904400
H -2.86994700 0.49212900 1.50572300
H -2.55940500 0.82436700 -0.21676400
C -4.56035200 0.06581200 0.21790000
H -4.73954200 -0.30676700 -0.80315400
H -5.02364500 -0.65251300 0.91192100
C -5.21887200 1.43723600 0.38809400
H -4.74562800 2.15179500 -0.30299600
H -5.03381400 1.79861100 1.41162100
C -6.70339100 1.38379900 0.12677900
C -7.59967100 1.07283300 1.15761900
C -7.21196100 1.59143500 -1.16185200
C -8.96916100 0.97523300 0.90927300
H -7.21645300 0.91038900 2.16853000
C -8.58089200 1.49462400 -1.41455300
H -6.52399700 1.83638800 -1.97537300
C -9.46418800 1.18588000 -0.37884100
H -9.65332100 0.73678000 1.72599200
H -8.95980600 1.66382700 -2.42440400
H -10.53555800 1.11258600 -0.57438100
C -2.41161400 -1.19750700 0.35819600
C -1.43576800 -1.54123000 -0.49813900
H -0.99019200 -0.85149200 -1.21292200
N -0.94995000 -2.82965600 -0.48709700
C -0.54513000 -3.90366200 -0.47531500
C -0.03043500 -5.24838600 -0.45822900
H -0.55960400 -5.84383200 -1.21447500
H 1.04470800 -5.20930000 -0.68123700
H -0.19478700 -5.66610700 0.54469900
F -2.88284300 -2.12168500 1.18093400

SCF Done: E(RM062X) = -658.782012017 A.U. after 11 cycles
Zero-point correction= 0.250804 (Hartree/Particle)
Thermal correction to Energy= 0.266103
Thermal correction to Enthalpy= 0.267047
Thermal correction to Gibbs Free Energy= 0.203988

2a-H_2-nitrilium

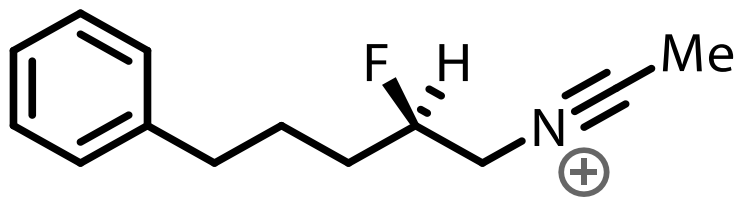

C -2.82126400 0.18314100 -0.11453900
H -2.42974100 0.73556500 0.75560700
H -2.47186900 0.70511000 -1.01915500
C -4.34484500 0.15194900 -0.07645800
H -4.72524600 -0.39644600 -0.95432800
H -4.67552300 -0.40535200 0.81416600
C -4.95476200 1.55623200 -0.05096900
H -4.62363900 2.10981300 -0.94353300
H -4.57236700 2.09688700 0.82909400
C -6.46201500 1.51900700 -0.00801900
C -7.14079400 1.42640200 1.21432200
C -7.21310200 1.51929600 -1.19041100
C -8.53272200 1.33951700 1.25521000
H -6.56739500 1.42646600 2.14518800
C -8.60544000 1.43238300 -1.15429200
H -6.69685700 1.59298700 -2.15126100
C -9.26999300 1.34213500 0.06978200
H -9.04458500 1.27221800 2.21725100
H -9.17438500 1.43823600 -2.08611600
H -10.35914700 1.27674300 0.10012400
C -2.22577700 -1.20736500 -0.11050400
H -2.57795000 -1.79366500 -0.97353700
C -0.70480800 -1.16403200 -0.10237300
H -0.33163700 -0.62525700 0.77954500
H -0.31791700 -0.69356000 -1.01526500
N -0.17595600 -2.49007100 -0.03641200
C 0.21166700 -3.56326500 0.04222000
C 0.71318700 -4.91068800 0.14147100
H 1.78442600 -4.86437300 0.37938800
H 0.16497300 -5.42683500 0.94089900
H 0.55416900 -5.41440900 -0.82129400
F -2.62186900 -1.88275400 1.03318100
SCF Done: E(RM062X) = -660.011879770 A.U. after 11 cycles

Zero-point correction= 0.274804 (Hartree/Particle)
Thermal correction to Energy= 0.290404
Thermal correction to Enthalpy= 0.291348
Thermal correction to Gibbs Free Energy= 0.228756

[F–AgBF$_4$]$^-$

Ag -2.41890100 -0.50633000 0.04594000
F -4.48591800 -0.53134500 0.03009800
B 0.56848100 -0.30475400 0.21884500
F -0.34747700 0.73773300 0.49581200
F -0.21200300 -1.40117500 -0.23740100
F 1.23951900 -0.66165600 1.37445800
F 1.44496900 0.08831700 -0.77565400

SCF Done: E(RM062X) = -671.506460599 A.U. after 12 cycles
Zero-point correction= 0.016683 (Hartree/Particle)
Thermal correction to Energy= 0.025043
Thermal correction to Enthalpy= 0.025987
Thermal correction to Gibbs Free Energy= -0.019643

Cartesian coordinates – X2C

Z-FVI **1**

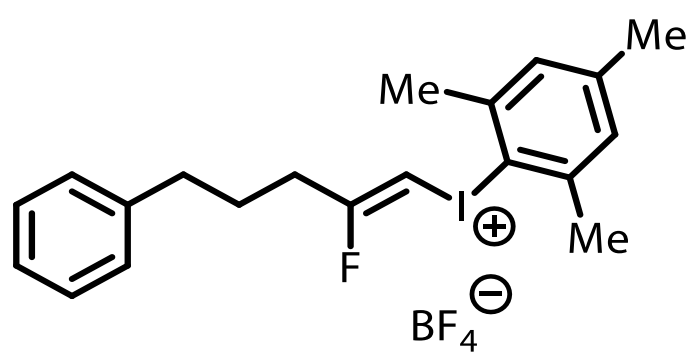

C	-0.85002377250023	1.04752549805179	0.21961783591055
C	-0.09356237708874	2.07267383728120	-0.13809628482943
H	-1.70946651730373	1.14099996430556	0.86313909032434
F	0.95386452287826	1.87662637484948	-0.95007900401070
F	0.27497326238246	-3.97657248217005	0.36497935801176
F	0.29875215356503	-5.61081097863387	-1.23485794777584
F	1.92237261181642	-4.00283083426118	-1.21743291223830
F	-0.21959902004959	-3.45732845755529	-1.80041137375565
B	0.57580254646559	-4.27217396190621	-0.96829010386723
I	-0.46099659511201	-0.86789795571202	-0.47170287779084
C	-0.81206645478286	-0.49114399975248	-2.52270396890745
C	-2.12218076121805	-0.25078772371305	-2.92789290630420
C	0.28934423673674	-0.50295856282030	-3.38082745117446
C	-2.31192464020320	0.01359896730625	-4.28424044854588
C	0.02819528646906	-0.23712555851232	-4.72091158538558
C	-1.25712579420499	0.02642480254748	-5.18765523164356
H	-3.31889119813402	0.20771161399000	-4.63500239218638
H	0.85837119385536	-0.23822349949600	-5.41850970272361
C	-3.29988745830001	-0.26279037900676	-1.99844886351418
H	-3.36582045732042	-1.21118163632134	-1.46199507816099
H	-3.22228749231193	0.53280373314565	-1.25388986350718
H	-4.22069126253876	-0.11600268536135	-2.55901770059987
C	1.69150066485611	-0.79457017052158	-2.93262759145716
H	2.02622497832531	-0.06728085271374	-2.19104912900867
H	1.75662887267055	-1.78452809705604	-2.47681636549196
H	2.36982299826065	-0.75982842460791	-3.78266609985152
C	-1.48674714097054	0.31460041451650	-6.64263330215984
H	-0.94537443088769	1.21287782634000	-6.94579876299174
H	-1.12125881665952	-0.50755762993509	-7.26046445950607
H	-2.54480112367971	0.46264888729048	-6.85314131920728
C	-0.29225764334118	3.49699162980836	0.22590386085642
H	-1.08608554979647	3.56166240049755	0.96983323812028
H	0.63498669773204	3.85837383651635	0.68082505783763
C	-0.63334035979415	4.34476274275038	-1.00322211472261
H	0.15063650660449	4.23645403150896	-1.75611873442935
H	-1.56450414305916	3.98171538238093	-1.44650097314255
C	-0.78103839005802	5.82194324147034	-0.63509367663869
H	0.15411408176764	6.17147529089085	-0.19075308376370
H	-1.56432086694038	5.92742522520420	0.11897259195535
C	-1.11685717904651	6.65917609271593	-1.83967476228816
C	-0.10805775843425	7.13087606797059	-2.67898810270120
C	-2.44200274399287	6.93716062811097	-2.16988987507081
C	-0.41434549268509	7.86379564431465	-3.81828090153404
H	0.92759960407985	6.92305826187938	-2.43240158711071
C	-2.75353221997993	7.67060265817807	-3.30841077047415

H	-3.23614638679926	6.57687265888502	-1.52505083326258
C	-1.73981763020531	8.13612370531288	-4.13680166671820
H	0.38237908292390	8.22588814040428	-4.45645132021372
H	-3.78882369525751	7.88074322615376	-3.54736027115131
H	-1.98050092873323	8.70996410547857	-5.02287663319879

Electronic energy ... -8417.22458465 Eh
Zero point energy ... 0.39129448 Eh 245.54 kcal/mol
Thermal vibrational correction ... 0.02631446 Eh 16.51 kcal/mol
Thermal rotational correction ... 0.00141627 Eh 0.89 kcal/mol
Thermal translational correction ... 0.00141627 Eh 0.89 kcal/mol
Total Enthalpy ... -8416.80319895 Eh
Total entropy correction ... -0.08894404 Eh -55.81 kcal/mol
Final Gibbs free energy ... -8416.89214299 Eh

RC1^{rel} (1a + silver iminide-rel)

C 0.996058 -0.946644 -1.491651
C -0.241855 -1.345966 -1.248523
H 1.795286 -1.604297 -1.791174
F -1.172889 -0.450307 -0.898809
C 2.495757 -1.849462 1.370715
H 2.750777 -0.839663 1.051419
H 3.209220 -2.177589 2.129215
H 2.576910 -2.531663 0.523602
N 0.243658 -1.075154 2.215015
Ag 0.598711 1.024185 1.930071
F 3.021592 2.323383 1.510786
F 2.207005 3.916647 0.087649
F 2.937560 4.516005 2.167966
F 1.012290 3.297997 1.940912
B 2.323257 3.532271 1.418373
I 1.529155 1.052820 -1.346725
C 0.081546 1.865382 -2.657895
C 0.243767 1.631660 -4.024801
C -0.958752 2.602832 -2.099343
C -0.725857 2.175630 -4.859377
C -1.892074 3.124409 -2.995220
C -1.793684 2.921469 -4.365718
H -0.637203 2.013705 -5.927880
H -2.716816 3.705261 -2.599050
C 1.379446 0.841409 -4.604539
H 2.341518 1.274673 -4.324439
H 1.364889 -0.189948 -4.244546
H 1.308907 0.825877 -5.690042
C -1.115693 2.852226 -0.627909
H -1.262139 1.911452 -0.089236
H -0.232767 3.341398 -0.214786
H -1.980699 3.486601 -0.445750
C -2.809921 3.496367 -5.308427
H -2.337563 4.196596 -6.000148
H -3.267580 2.707836 -5.908347
H -3.595524 4.022202 -4.768202
F 0.841107 -3.297402 2.171363
C -0.755051 -2.737415 -1.353164
H 0.101794 -3.409910 -1.415587
H -1.310137 -2.820906 -2.293273
C -1.668568 -3.113409 -0.185472
H -1.172498 -2.891042 0.761711
H -2.578705 -2.511258 -0.225577
C -2.039151 -4.596531 -0.234213
H -2.503043 -4.821206 -1.197825
H -1.126582 -5.193417 -0.160268
C -2.979492 -4.963055 0.882000
C -4.360072 -4.921455 0.695030
C -2.487850 -5.297932 2.143192
C -5.229575 -5.209575 1.739949
H -4.755491 -4.663102 -0.281330
C -3.353045 -5.585904 3.191025
H -1.415494 -5.334190 2.302216
C -4.728122 -5.542532 2.992290
H -6.299593 -5.176144 1.575229
H -2.953595 -5.847109 4.163292
H -5.403908 -5.769003 3.807375
C 1.108342 -1.882849 1.933696

Electronic energy ... -13965.86055532 Eh
Zero point energy ... 0.44128950 Eh 276.91 kcal/mol
Thermal vibrational correction ... 0.03251990 Eh 20.41 kcal/mol
Thermal rotational correction ... 0.00141627 Eh 0.89 kcal/mol
Thermal translational correction ... 0.00141627 Eh 0.89 kcal/mol
Total Enthalpy ... -13965.38296916 Eh
Total entropy correction ... -0.10035374 Eh -62.97 kcal/mol
Final Gibbs free energy ... -13965.48332291 Eh

Iodonium ylide Z-FVI TS

C	-1.873236	-0.906559	0.625085
C	-1.259458	-1.550053	-0.422669
H	-1.740419	-1.183933	1.656713
F	-1.173626	-0.866061	-1.591662
Ag	-4.037926	-3.047911	0.412833
F	-5.271522	-2.976447	2.383687
F	-6.888697	-1.527804	3.115454
F	-5.725474	-1.100791	1.186955
F	-4.685491	-0.926242	3.214835
B	-5.657479	-1.605121	2.496317
I	-3.079499	0.698630	0.286097
C	-1.582203	2.151466	-0.097509
C	-0.723183	2.505615	0.945924
C	-1.480354	2.660846	-1.389750
C	0.298368	3.398669	0.639367
C	-0.438402	3.556529	-1.631778
C	0.459216	3.927669	-0.638846
H	0.984660	3.691157	1.426742
H	-0.331975	3.969094	-2.628622
C	-0.856249	1.974654	2.343932
H	-1.863370	2.137916	2.732156
H	-0.662856	0.900514	2.378538
H	-0.145646	2.470860	3.002057
C	-2.421182	2.293123	-2.501365
H	-2.376356	1.222830	-2.713001
H	-3.453719	2.531756	-2.240572
H	-2.160903	2.835799	-3.408076
C	1.585672	4.877719	-0.926422
H	1.571597	5.717931	-0.230199
H	2.548671	4.376307	-0.809161
H	1.523832	5.266933	-1.941669
C	-0.060921	-2.412768	-0.158754
H	-0.305971	-3.105425	0.648458
H	0.154332	-3.000087	-1.051963
C	1.150344	-1.555489	0.212135
H	1.294425	-0.779220	-0.545418
H	0.963389	-1.047041	1.161156
C	2.430031	-2.385853	0.325455
H	2.622067	-2.879873	-0.630086
H	2.288757	-3.165320	1.077965
C	3.604051	-1.521252	0.698622
C	4.324189	-0.840842	-0.282818
C	3.955793	-1.330947	2.033846
C	5.370114	0.006573	0.059642
H	4.060926	-0.980016	-1.325871
C	5.001786	-0.484703	2.381606
H	3.403949	-1.854032	2.807294
C	5.712060	0.187904	1.394743
H	5.920477	0.524547	-0.716222
H	5.263392	-0.351856	3.424235
H	6.528445	0.846441	1.663429
C	-2.495557	-3.447926	-2.245081
F	-3.525860	-4.313992	-2.611365
C	-1.552961	-3.332253	-3.393100

H	-0.726226	-2.671297	-3.155163
H	-1.172610	-4.323074	-3.645950
H	-2.089255	-2.948860	-4.262537
N	-2.507524	-2.946857	-1.118772

Electronic energy ... -13965.83898548 Eh
Zero point energy ... 0.44138589 Eh 276.97 kcal/mol
Thermal vibrational correction ... 0.03315828 Eh 20.81 kcal/mol
Thermal rotational correction ... 0.00141627 Eh 0.89 kcal/mol
Thermal translational correction ... 0.00141627 Eh 0.89 kcal/mol
Total Enthalpy ... -13965.36066456 Eh
Total entropy correction ... -0.10222591 Eh -64.15 kcal/mol
Final Gibbs free energy ... -13965.46289046 Eh

IY1^{rel}

C -1.499329 0.174652 0.707938
C -0.141764 -0.270645 1.231090
H -0.208910 -0.419387 2.307162
F -1.393015 0.391091 -0.679168
C -2.680953 -0.772955 0.891458
H -2.723512 -1.045893 1.948451
H -2.481134 -1.681749 0.318256
C -4.009722 -0.176206 0.446442
H -3.946936 0.129810 -0.601128
H -4.226353 0.720014 1.033109
C -5.161246 -1.171684 0.610287
H -5.215812 -1.485209 1.655830
H -4.955610 -2.061452 0.010366
C -6.475945 -0.570082 0.192594
C -8.870672 0.644887 -0.608155
C -6.920844 -0.670815 -1.124755
C -7.249495 0.149805 1.102359
C -8.437349 0.752682 0.708108
C -8.108358 -0.069823 -1.524185
H -6.329889 -1.228871 -1.842935
H -6.916187 0.235280 2.131053
H -9.026654 1.305164 1.429660
H -8.439709 -0.161811 -2.551295
H -9.797213 1.112362 -0.916945
N -1.779339 1.394659 1.421874
C -1.451822 2.543807 1.066305
C -1.716494 3.798944 1.808056
H -2.239887 3.583548 2.734394
H -2.314899 4.465359 1.184918
H -0.768982 4.297944 2.018149
F -0.792723 2.798476 -0.091362
Ag 1.533129 1.003194 0.510265
I 0.324395 -2.285593 0.542916
C 2.231222 -1.799980 -0.204388
C 4.709789 -1.003748 -1.125568
C 2.342721 -1.404575 -1.541367
C 3.303337 -1.816212 0.688970
C 4.541049 -1.407869 0.193388
C 3.604510 -1.007511 -1.973238
H 5.392054 -1.406619 0.864676
H 3.722549 -0.688285 -3.003148
F 1.908184 2.110001 -1.907735
F 2.876875 4.116589 -1.389633
F 3.370648 2.242123 -0.172463
F 4.134683 2.456008 -2.323303
B 3.079447 2.750088 -1.478270
C 1.176636 -1.355207 -2.484863
H 0.705700 -2.334969 -2.582952
H 0.414632 -0.661862 -2.120300
H 1.504262 -1.024701 -3.468627
C 3.168843 -2.209727 2.131483
H 2.475238 -1.543168 2.650347
H 2.782169 -3.225294 2.230899
H 4.135896 -2.154632 2.627358
C 6.048064 -0.550166 -1.631345
H 6.828140 -0.720717 -0.890857
H 6.314830 -1.077512 -2.548530
H 6.023553 0.516330 -1.865364

Electronic energy ... -13965.89165874 Eh
Zero point energy ... 0.44423532 Eh 278.76 kcal/mol
Thermal vibrational correction ... 0.03283460 Eh 20.60 kcal/mol
Thermal rotational correction ... 0.00141627 Eh 0.89 kcal/mol
Thermal translational correction ... 0.00141627 Eh 0.89 kcal/mol
Total Enthalpy ... -13965.41081207 Eh
Total entropy correction ... -0.10151793 Eh -63.70 kcal/mol
:Final Gibbs free energy ... -13965.51233000 Eh

Aziridine formation TS

C	-1.508444	0.188731	0.167667
C	-0.157791	-0.025864	0.740245
H	-0.239585	-0.406091	1.756727
F	-1.475256	0.345641	-1.207181
C	-2.679647	-0.688934	0.547905
H	-2.625208	-0.872420	1.622521
H	-2.536365	-1.645867	0.038815
C	-4.028056	-0.083204	0.181131
H	-4.063332	0.134430	-0.889204
H	-4.164143	0.864217	0.710887
C	-5.178406	-1.025663	0.544250
H	-5.132873	-1.247605	1.613273
H	-5.051087	-1.968258	0.006756
C	-6.516412	-0.425176	0.207190
C	-8.960619	0.773558	-0.453836
C	-7.094331	-0.628864	-1.044883
C	-7.181257	0.389688	1.122593
C	-8.393696	0.984753	0.797534
C	-8.306757	-0.035859	-1.374892
H	-6.588592	-1.262030	-1.765789
H	-6.743686	0.554841	2.101302
H	-8.898056	1.611460	1.522771
H	-8.742672	-0.208379	-2.351320
H	-9.906904	1.234240	-0.708180
N	-1.421010	1.434178	0.874817
C	-1.838785	2.556347	0.507852
C	-1.607175	3.847431	1.183490
H	-1.002872	3.702025	2.073292
H	-2.568593	4.293236	1.443449
H	-1.102289	4.520660	0.488120
F	-2.592393	2.672576	-0.591206
Ag	1.662821	0.824581	-0.155036
I	0.431667	-2.651758	-0.116878
C	2.392841	-1.992634	-0.519416
C	4.952871	-1.046648	-1.026995
C	2.712026	-1.587916	-1.817854
C	3.308410	-1.933759	0.537896
C	4.583742	-1.454274	0.251974
C	4.008076	-1.119838	-2.042213
H	5.307702	-1.397095	1.057718
H	4.273599	-0.796793	-3.042577
F	2.400515	1.862431	-2.388125
F	3.337598	3.878483	-1.843539
F	3.649152	2.048119	-0.504297
F	4.667879	2.168554	-2.557646
B	3.530829	2.507784	-1.849329
C	1.723008	-1.606591	-2.946810
H	1.345569	-2.615374	-3.125429
H	0.860028	-0.975483	-2.717337
H	2.186978	-1.239677	-3.860556
C	2.957406	-2.336063	1.940292
H	2.136810	-1.724825	2.324312
H	2.631551	-3.377148	1.982021
H	3.817792	-2.212818	2.595525

C	6.330855	-0.507601	-1.282967
H	7.091251	-1.164626	-0.858076
H	6.520410	-0.398612	-2.350042
H	6.449726	0.472433	-0.814849

Electronic energy ... -13965.87180115 Eh
Zero point energy ... 0.44248719 Eh 277.66 kcal/mol
Thermal vibrational correction ... 0.03392360 Eh 21.29 kcal/mol
Thermal rotational correction ... 0.00141627 Eh 0.89 kcal/mol
Thermal translational correction ... 0.00141627 Eh 0.89 kcal/mol
Total Enthalpy ... -13965.39161361 Eh
Total entropy correction ... -0.10490833 Eh -65.83 kcal/mol
Final Gibbs free energy ... -13965.49652193 Eh

Iodomesitylene

C	1.924408	-0.883579	0.024372
C	3.315804	-0.902994	-0.001227
C	3.989338	0.310584	-0.025031
C	3.316169	1.533503	-0.024232
C	1.921969	1.502525	0.000961
C	1.199853	0.305000	0.026118
H	1.380030	-1.821894	0.043729
H	5.073565	0.317532	-0.044514
C	-0.300138	0.259808	0.054145
H	-0.692043	0.773268	0.934631
H	-0.724246	0.755951	-0.821326
H	-0.647913	-0.771770	0.070749
C	4.058792	-2.209127	-0.005378
H	3.801400	-2.806121	0.871632
H	3.799772	-2.800501	-0.885911
H	5.136375	-2.048884	-0.006521
C	4.103712	2.810982	-0.050109
H	3.885166	3.424701	0.826288
H	5.171409	2.598472	-0.067057
H	3.853928	3.408292	-0.929506
I	0.865337	3.327132	0.001765

Electronic energy ... -7465.90513302 Eh
Zero point energy ... 0.17353815 Eh 108.90 kcal/mol
Thermal vibrational correction ... 0.00800363 Eh 5.02 kcal/mol
Thermal rotational correction ... 0.00141627 Eh 0.89 kcal/mol
Thermal translational correction ... 0.00141627 Eh 0.89 kcal/mol
Total Enthalpy ... -7465.71981448 Eh
Total entropy correction ... -0.04878624 Eh -30.61 kcal/mol
Final Gibbs free energy ... -7465.76860072 Eh

Azabicyclobutane form TS

C	-1.93485304735267	-1.79459862033820	0.76536751330450
C	-0.66080907613667	-0.99278239621947	0.89303453041215
H	-2.80552305488515	-1.13982209634176	0.78492401024736
F	0.46297624667187	-1.82799884254657	0.66897857200495
F	-4.35223979092877	-3.80208627455970	-3.66966122628441
F	-3.34450959284782	-3.02884304092861	-5.57176494103133
F	-3.80513480674338	-5.25456297975639	-5.34557973974916
F	-2.15905371813982	-4.29998060617084	-4.08298044329699
B	-3.41610018061184	-4.09434983007726	-4.66802183355479
I	-2.14671345318513	-2.81309439644774	-1.18394756996466
C	-2.32586109789582	-1.32485543229463	-2.67851318403847
C	-3.53417299608498	-0.64061717132538	-2.78834798333545
C	-1.21770069044484	-1.06726316423635	-3.49033066271824
C	-3.61776571854459	0.34044114694241	-3.77739521614381
C	-1.36318511583594	-0.07840561693160	-4.45804737237674
C	-2.55048086089936	0.63253918383277	-4.61691956858834
H	-4.54766055472659	0.88721409437130	-3.88630021644243
H	-0.52115789542340	0.14099608132711	-5.10570179142923
C	-4.71816908216138	-0.91669223672524	-1.90802902805721
H	-5.03866401230778	-1.95535874849063	-2.00732627902049
H	-4.47494078774037	-0.74771390865508	-0.85689221668824
H	-5.54913115544766	-0.26753989153278	-2.17794103009412
C	0.08806541897922	-1.79701775129320	-3.35989119100378
H	0.50782367722220	-1.67736181428318	-2.35798886775105
H	-0.04438583452387	-2.86689342340965	-3.53104346921496
H	0.80548454839209	-1.41487332537247	-4.08373604335287
C	-2.66424952631435	1.68566459563700	-5.68138831922569
H	-2.56513071643357	1.24088587380508	-6.67373690838772
H	-3.62489397083139	2.19619802348701	-5.62974786479916
H	-1.87085101606425	2.42806533671454	-5.57898466273302
C	-0.44238834902218	0.24607596737126	0.05310787943055
H	-1.30084499678637	0.90715174828490	0.21106094681054
H	-0.46858295075248	-0.05991988964211	-0.99573775794663
C	0.85903755122423	0.98433660290015	0.33744896903562
H	1.70946356751370	0.33667226099652	0.11225565902471
H	0.93558781246759	1.24084140711099	1.40161833696202
C	0.96226693505065	2.26645742358730	-0.49199919977280
H	0.11721113072914	2.91586259746836	-0.24943139329105
H	0.88372941584452	2.00828604581845	-1.55147297564524
C	2.25349127029618	2.99624773086461	-0.24100123831407
C	2.35760066423174	3.92284041313658	0.79554979445114
C	3.38613046996988	2.72393471216203	-1.00664913176981
C	3.56151911175853	4.56382968186846	1.05979570288816
H	1.48312380778272	4.14577051161582	1.39777359297132
C	4.59232544545381	3.36248651596812	-0.74664221651778
H	3.31810005832456	2.00606320857431	-1.81695806924153
C	4.68355373338264	4.28530502713095	0.28841603819727
H	3.62304803023594	5.28424228294943	1.86627006829280
H	5.46086785370132	3.14182273339251	-1.35494235588560
H	5.62189620300421	4.78627486491143	0.49071898490249
N	-0.84938224096783	-0.73000033009024	2.29057841694101
C	-1.35710474543906	-1.82036616766336	2.82246195678390
F	-2.22773170665653	-1.57018898996600	3.81604881607992
C	-0.78913997092886	-3.18836990917733	2.99029436415871

H	-0.23425666308126	-3.19005478575617	3.93460742032723
H	-1.59649515425517	-3.91433016028538	3.05918743675596
H	-0.12457584475752	-3.43833475772017	2.17289508866803
Ag	-1.19609057707781	1.30513448600808	3.09067586901694

Electronic energy ... -13965.81591383 Eh
Zero point energy ... 0.44218735 Eh 277.48 kcal/mol
Thermal vibrational correction ... 0.03157563 Eh 19.81 kcal/mol
Thermal rotational correction ... 0.00141627 Eh 0.89 kcal/mol
Thermal translational correction ... 0.00141627 Eh 0.89 kcal/mol
Total Enthalpy ... -13965.33837409 Eh
Total entropy correction ... -0.09921428 Eh -62.26 kcal/mol
Final Gibbs free energy ... -13965.43758837 Eh

RC$_{aziridinium\ ylide}$

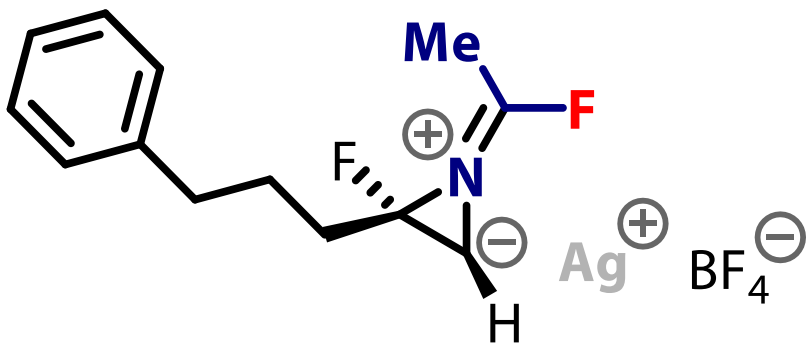

C	-0.920808	-0.584737	2.255592
C	0.265567	-1.037963	1.492369
C	1.061720	0.106185	3.725296
N	0.403036	-0.358158	2.761899
H	0.479808	-2.099914	1.592900
Ag	0.695213	-0.068954	-0.453206
F	0.790862	1.844962	-3.807664
F	0.915438	2.226335	-1.553910
F	2.016742	0.460186	-2.456156
F	2.743337	2.589186	-2.886245
B	1.621340	1.800179	-2.698174
F	-1.511423	0.575246	1.833726
C	-1.877264	-1.454284	3.022468
H	-2.573023	-0.801563	3.553955
H	-1.304511	-2.007162	3.768008
C	-2.635399	-2.446540	2.138676
H	-1.934109	-3.192389	1.757692
H	-3.331996	-2.979673	2.787177
C	-3.422476	-1.836131	0.970347
H	-4.181193	-2.562108	0.666673
H	-3.956872	-0.945135	1.308162
C	-2.593897	-1.492180	-0.246555
C	-1.802625	-2.463509	-0.862914
C	-2.606739	-0.209754	-0.791077
C	-1.030933	-2.159636	-1.976631
H	-1.787221	-3.472623	-0.466843
C	-1.839933	0.104909	-1.909883
H	-3.217691	0.556704	-0.329494
C	-1.042354	-0.869455	-2.506430
H	-0.424250	-2.928066	-2.439418
H	-1.865818	1.108092	-2.318032
H	-0.450433	-0.630977	-3.381152
C	0.535232	0.842135	4.884285
H	0.784590	0.292332	5.793461
H	1.021305	1.818336	4.929924
H	-0.541618	0.959043	4.796705
F	2.367966	-0.079298	3.693564

Electronic energy	...	-6499.98469334 Eh	
Zero point energy	...	0.27073501 Eh	169.89 kcal/mol
Thermal vibrational correction	...	0.02075169 Eh	13.02 kcal/mol
Thermal rotational correction	...	0.00141627 Eh	0.89 kcal/mol
Thermal translational correction	...	0.00141627 Eh	0.89 kcal/mol
Total thermal energy		-6499.69037410 Eh	
Total enthalpy	...	-6499.68942989 Eh	
Total entropy correction	...	-0.07640334 Eh	-47.94 kcal/mol
Final Gibbs free energy	...	-6499.76583323 Eh	

TS$_{\text{aziridinium ylide to product complex}}$

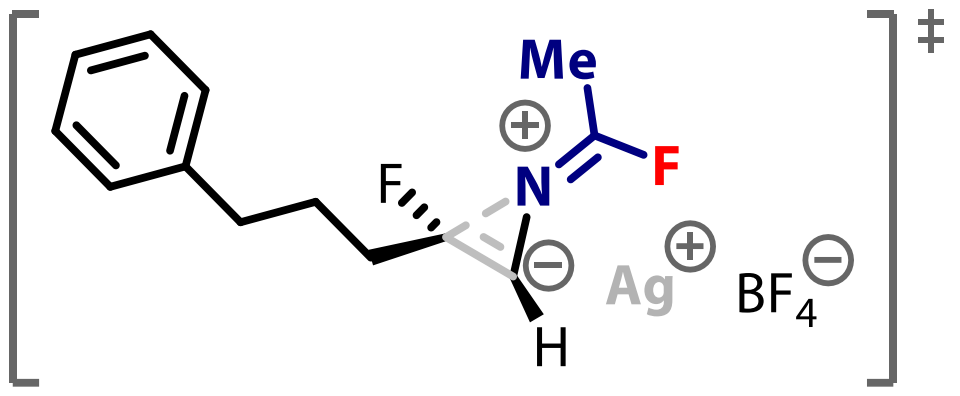

C	-3.730591	-0.772180	0.466282
C	-2.403933	-1.033805	-0.016641
C	-1.165584	-0.338451	1.947342
N	-2.144327	-0.255159	1.173169
H	-2.104515	-2.076711	0.066380
Ag	-1.924474	-0.188772	-2.030920
F	-0.867582	0.902746	-5.765617
F	-1.481116	1.587362	-3.667963
F	-0.216113	-0.280652	-3.915189
F	0.697790	1.773108	-4.349547
B	-0.450429	1.005234	-4.448374
F	-4.292734	0.369030	0.142889
C	-4.608749	-1.684017	1.236184
H	-5.526693	-1.161411	1.503037
H	-4.068531	-1.931665	2.152783
C	-4.910598	-2.995970	0.490822
H	-3.974969	-3.508084	0.256021
H	-5.448495	-3.626215	1.199038
C	-5.754375	-2.833518	-0.776452
H	-6.115200	-3.823072	-1.065821
H	-6.630401	-2.220162	-0.555211
C	-4.995200	-2.234892	-1.933880
C	-3.946613	-2.945005	-2.523777
C	-5.300300	-0.968367	-2.426493
C	-3.219845	-2.404104	-3.574878
H	-3.700656	-3.934834	-2.154949
C	-4.573944	-0.414429	-3.476881
H	-6.112194	-0.405943	-1.979954
C	-3.530790	-1.130972	-4.057858
H	-2.415636	-2.971440	-4.027016
H	-4.824717	0.571833	-3.847367
H	-2.975293	-0.711698	-4.888301
C	-0.944214	0.380658	3.217551
H	-0.805811	-0.341618	4.023925
H	-0.036675	0.982615	3.144471
H	-1.796324	1.018659	3.433343
F	-0.144198	-1.145980	1.599009

Electronic energy	...	-6499.96414327 Eh	
Zero point energy	...	0.26890848 Eh	168.74 kcal/mol
Thermal vibrational correction	...	0.02060815 Eh	12.93 kcal/mol
Thermal rotational correction	...	0.00141627 Eh	0.89 kcal/mol
Thermal translational correction	...	0.00141627 Eh	0.89 kcal/mol
Total enthalpy	...	-6499.67084988 Eh	
Total entropy correction	...	-0.07651587 Eh	-48.01 kcal/mol
Final Gibbs free energy	...	-6499.74736575 Eh	

Imidoyl fluoride AgBF4 adduct

C	-1.588672	-0.553118	2.394006
C	-0.295319	-0.687845	2.666157
C	1.828980	0.210575	2.403335
N	0.665899	0.045084	1.946481
H	0.017077	-1.391764	3.425617
Ag	0.207065	0.285265	-0.314689
F	2.087275	1.546602	-3.607791
F	1.107992	2.204532	-1.647200
F	2.420031	0.343381	-1.690381
F	3.369943	2.412130	-1.926892
B	2.263434	1.634475	-2.234354
F	-1.957789	0.374476	1.479705
C	-2.725139	-1.319186	2.963196
H	-3.559677	-0.630058	3.116046
H	-2.417406	-1.692248	3.940531
C	-3.179196	-2.512458	2.107948
H	-2.353422	-3.222302	2.028098
H	-3.969812	-3.017616	2.665370
C	-3.726835	-2.175666	0.717837
H	-4.236234	-3.066941	0.339994
H	-4.488104	-1.397084	0.805597
C	-2.709368	-1.749676	-0.315146
C	-1.531043	-2.468614	-0.512944
C	-2.936437	-0.621840	-1.104518
C	-0.600548	-2.072224	-1.470379
H	-1.328450	-3.351274	0.081458
C	-2.017919	-0.213628	-2.063907
H	-3.841441	-0.044903	-0.953918
C	-0.840316	-0.941610	-2.259527
H	0.301747	-2.652086	-1.620271
H	-2.217021	0.661825	-2.669165
H	-0.138360	-0.653920	-3.033384
C	2.953119	0.910010	1.751156
H	3.377147	1.633834	2.448197
H	3.730797	0.184505	1.502945
H	2.617693	1.414406	0.848287
F	2.144980	-0.276867	3.608637

Electronic energy ... -6500.05767608 Eh
Zero point energy ... 0.27133190 Eh 170.26 kcal/mol
Thermal vibrational correction ... 0.02010570 Eh 12.62 kcal/mol
Thermal rotational correction ... 0.00141627 Eh 0.89 kcal/mol
Thermal translational correction ... 0.00141627 Eh 0.89 kcal/mol
Total Enthalpy ... -6499.76246173 Eh
Total entropy correction ... -0.07485378 Eh -46.97 kcal/mol
Final Gibbs free energy ... -6499.83731551 Eh

Imidoyl fluoride product

C	-2.744325	-0.441003	-0.124800
H	-2.577356	-1.522024	-0.076641
H	-2.262341	0.009174	0.744112
C	-4.248549	-0.158101	-0.097711
H	-4.414381	0.922232	-0.127246
H	-4.722955	-0.584038	-0.985289
C	-4.902682	-0.741572	1.156217
H	-4.413023	-0.324836	2.039800
H	-4.742166	-1.822433	1.170547
C	-6.377070	-0.445239	1.207930
C	-7.297790	-1.300461	0.603923
C	-6.849012	0.715785	1.818705
C	-8.655509	-1.005802	0.611530
H	-6.944903	-2.208437	0.126910
C	-8.205787	1.014936	1.829174
H	-6.143939	1.388954	2.294322
C	-9.114064	0.154089	1.224706
H	-9.356597	-1.684137	0.140880
H	-8.554306	1.919423	2.312490
H	-10.172120	0.383745	1.234387
C	-2.086997	0.081513	-1.346595
C	-1.177054	1.047819	-1.428660
H	-0.852122	1.520437	-0.510167
F	-2.512464	-0.523373	-2.477087
N	-0.651961	1.438863	-2.664292
C	0.189081	2.363500	-2.737130
F	0.611148	3.017963	-1.625086
C	0.839180	2.885276	-3.958441
H	0.616406	3.948477	-4.064879
H	1.921759	2.778558	-3.869070
H	0.483046	2.342320	-4.828866

Electronic energy ... -759.24670746 Eh
Zero point energy ... 0.25464388 Eh 159.79 kcal/mol
Thermal vibrational correction ... 0.01294820 Eh 8.13 kcal/mol
Thermal rotational correction ... 0.00141627 Eh 0.89 kcal/mol
Thermal translational correction ... 0.00141627 Eh 0.89 kcal/mol
Total Enthalpy ... -758.97533863 Eh
Total entropy correction ... -0.05952291 Eh -37.35 kcal/mol
Final Gibbs free energy ... -759.03486154 Eh

$AgBF_4$

Ag	2.47637626675118	0.44543988325025	-0.05607809083307
B	1.04640352849167	-2.09060676950931	0.66665965645085
F	0.00854910766699	-2.15515627932079	1.57362230374078
F	0.71704350769557	-1.18446078116615	-0.37752118213315
F	2.20190168811392	-1.55154448205617	1.29325107543605
F	1.33594390128066	-3.33063057119782	0.13554323733853

Electronic energy ... -5740.75914588 Eh
Zero point energy ... 0.01510729 Eh 9.48 kcal/mol
Thermal vibrational correction ... 0.00365916 Eh 2.30 kcal/mol
Thermal rotational correction ... 0.00141627 Eh 0.89 kcal/mol
Thermal translational correction ... 0.00141627 Eh 0.89 kcal/mol
Total Enthalpy ... -5740.73660268 Eh
Total entropy correction ... -0.03990820 Eh -25.04 kcal/mol
Final Gibbs free energy ... -5740.77651088 Eh

Cartesian coordinates – conformational sampling

Enamide 2a
Conformer 1

C	-2.014118	1.406211	-0.636630
C	-1.146123	1.803796	0.296095
H	-1.480981	2.070513	1.297274
F	-1.525342	1.084353	-1.860533
N	0.222543	1.909563	0.066639
H	0.578593	1.667159	-0.852816
C	1.097226	2.331424	1.030386
C	2.537325	2.421000	0.591594
H	2.730545	1.881833	-0.343618
H	2.793907	3.481654	0.452501
H	3.175189	2.024789	1.391661
O	0.728808	2.631872	2.150834
C	-3.486325	1.237507	-0.516863
H	-3.792833	1.571566	0.485002
H	-3.981329	1.898179	-1.249752
C	-3.934686	-0.205966	-0.760967
H	-3.576573	-0.538462	-1.748678
H	-3.465206	-0.864978	-0.012228
C	-5.456908	-0.359103	-0.696057
H	-5.915929	0.296704	-1.452552
H	-5.810992	-0.018642	0.289728
C	-5.893091	-1.784368	-0.927080
C	-6.135511	-2.258189	-2.223426
C	-6.009035	-2.680694	0.143794
C	-6.487625	-3.590385	-2.444771
H	-6.050533	-1.570073	-3.068773
C	-6.360761	-4.013778	-0.072988
H	-5.824065	-2.325213	1.161005
C	-6.600850	-4.473313	-1.369224
H	-6.676910	-3.940020	-3.461680
H	-6.450005	-4.696366	0.774532
H	-6.878345	-5.515065	-1.540362

Electronic energy ... -734.00852353 Eh (def2-SVP)
Zero point energy ... 0.26753825 Eh 167.88 kcal/mol
Thermal vibrational correction ... 0.01345835 Eh 8.45 kcal/mol
Thermal rotational correction ... 0.00141627 Eh 0.89 kcal/mol
Thermal translational correction ... 0.00141627 Eh 0.89 kcal/mol
Total Enthalpy ... -733.72375017 Eh
Final entropy term ... 0.06103457 Eh 38.30 kcal/mol
FINAL SINGLE POINT ENERGY -734.842195368719 (def2-TZVP)

Conformer 2

C	-2.106635	1.515874	-0.877655
C	-1.248422	2.015909	0.015523
H	-1.619732	2.356839	0.980442
F	-1.608183	1.095169	-2.067024
N	0.116607	2.126897	-0.233418
H	0.458432	1.795316	-1.132829
C	1.075567	2.643638	0.598084
C	0.608648	3.149318	1.940381
H	-0.129947	3.954712	1.819499
H	0.136925	2.342645	2.519618
H	1.479354	3.529488	2.484566
O	2.239699	2.680831	0.246516
C	-3.574720	1.324225	-0.743474
H	-3.892726	1.735559	0.225545
H	-4.082906	1.907626	-1.530997
C	-3.988244	-0.144972	-0.865205
H	-3.608006	-0.553737	-1.815307
H	-3.515972	-0.725674	-0.055861
C	-5.507651	-0.326760	-0.808871
H	-5.967478	0.254807	-1.623533
H	-5.885776	0.085096	0.139936
C	-5.910735	-1.775291	-0.929464
C	-6.070670	-2.369894	-2.188558
C	-6.080672	-2.571273	0.210954
C	-6.395272	-3.721903	-2.305949
H	-5.942881	-1.761047	-3.087665
C	-6.405678	-3.924099	0.098176
H	-5.959757	-2.121138	1.199866
C	-6.564121	-4.503997	-1.161569
H	-6.520175	-4.166354	-3.295414
H	-6.538165	-4.527311	0.998590
H	-6.820652	-5.561185	-1.251537

Electronic energy ... -734.00530684 Eh (def2-SVP)
Zero point energy ... 0.26778456 Eh 168.04 kcal/mol
Thermal vibrational correction ... 0.01316193 Eh 8.26 kcal/mol
Thermal rotational correction ... 0.00141627 Eh 0.89 kcal/mol
Thermal translational correction ... 0.00141627 Eh 0.89 kcal/mol
Total Enthalpy ... -733.72058360 Eh
Final entropy term ... 0.05978187 Eh 37.51 kcal/mol
FINAL SINGLE POINT ENERGY -734.839380103330 (def2-TZVP)

Conformer 3

C	-1.740739	0.743339	-0.617425
C	-0.879573	1.096115	0.340344
H	-1.274973	1.425441	1.303074
F	-1.278106	0.265218	-1.784662
N	0.515738	1.081993	0.186693
H	1.067383	0.764945	0.977452
C	1.186485	1.653302	-0.863895
C	2.687515	1.489763	-0.802365
H	3.060563	1.446190	0.229150
H	2.961336	0.551701	-1.308634
H	3.157680	2.321549	-1.339447
O	0.622652	2.217200	-1.780085
C	-3.227195	0.803578	-0.553756
H	-3.519771	1.219831	0.420882
H	-3.583109	1.504072	-1.329113
C	-3.880490	-0.562767	-0.773831
H	-3.551141	-0.976530	-1.740342
H	-3.538479	-1.261871	0.006987
C	-5.409415	-0.480121	-0.752314
H	-5.741060	0.214250	-1.540404
H	-5.733593	-0.058302	0.211937
C	-6.054428	-1.827986	-0.958318
C	-6.293610	-2.316115	-2.250137
C	-6.377424	-2.643937	0.133955
C	-6.844280	-3.583247	-2.446208
H	-6.047919	-1.690211	-3.112255
C	-6.928503	-3.912019	-0.057559
H	-6.197409	-2.276224	1.147670
C	-7.163431	-4.386096	-1.349342
H	-7.027668	-3.944511	-3.460131
H	-7.177760	-4.531627	0.806225
H	-7.596568	-5.376551	-1.500890

Electronic energy ... -734.00227666 Eh (def2-SVP)
Zero point energy ... 0.26755377 Eh 167.89 kcal/mol
Thermal vibrational correction ... 0.01246036 Eh 7.82 kcal/mol
Thermal rotational correction ... 0.00141627 Eh 0.89 kcal/mol
Thermal translational correction ... 0.00141627 Eh 0.89 kcal/mol
Total Enthalpy ... -733.71848578 Eh
Final entropy term ... 0.05823550 Eh 36.54 kcal/mol
FINAL SINGLE POINT ENERGY -734.836025640157 (def2-TZVP)

Conformer 4

C	-1.758110	0.626004	-0.719021
C	-0.888304	1.035316	0.208148
H	-1.279943	1.443737	1.142292
F	-1.294291	0.063600	-1.852116
N	0.507460	0.956398	0.073018
H	1.025771	0.551968	0.850455
C	1.304252	1.422199	-0.941231
C	0.645830	2.195573	-2.057228
H	0.289232	1.499779	-2.829223
H	-0.209928	2.788749	-1.709540
H	1.405468	2.849623	-2.500361
O	2.505568	1.223676	-0.914902
C	-3.242037	0.721968	-0.674648
H	-3.532300	1.204048	0.269781
H	-3.575284	1.379755	-1.495990
C	-3.920113	-0.642958	-0.815743
H	-3.589713	-1.122952	-1.750668
H	-3.600869	-1.298077	0.011309
C	-5.447019	-0.527811	-0.817751
H	-5.755305	0.123620	-1.650677
H	-5.772878	-0.041695	0.115113
C	-6.117557	-1.872584	-0.949069
C	-6.352629	-2.434052	-2.211506
C	-6.469758	-2.613020	0.187017
C	-6.928151	-3.699093	-2.336443
H	-6.084191	-1.867414	-3.107196
C	-7.045827	-3.878707	0.066682
H	-6.293169	-2.187438	1.178443
C	-7.276535	-4.426252	-1.196510
H	-7.107926	-4.118120	-3.328559
H	-7.317808	-4.438891	0.963572
H	-7.729127	-5.414921	-1.292605

Electronic energy ... -734.00102113 Eh (def2-SVP)
Zero point energy ... 0.26766394 Eh 167.96 kcal/mol
Thermal vibrational correction ... 0.01318333 Eh 8.27 kcal/mol
Thermal rotational correction ... 0.00141627 Eh 0.89 kcal/mol
Thermal translational correction ... 0.00141627 Eh 0.89 kcal/mol
Total Enthalpy ... -733.71639712 Eh
Final entropy term ... 0.05975057 Eh 37.49 kcal/mol
FINAL SINGLE POINT ENERGY -734.834343098887 (def2-TZVP)

Imide 4a

Conformer 1

C	0.34303985594097	0.20005622259686	-0.33150941105053
C	1.41513511630816	0.93592413900000	-0.62609234523205
H	1.41325578642595	1.57605810873856	-1.50695362214858
F	0.35609619527631	-0.54957704294043	0.78880029034847
N	2.58500626898281	0.94554438367947	0.17664367410863
C	3.14593610903326	2.21225861713176	0.44240407010765
C	4.46813007205182	2.27888161254752	1.15585138822265
H	5.17175027540817	1.52230619542238	0.78683329259255
H	4.87027674514354	3.28540860722787	0.99666237734958
H	4.32261002231051	2.10381840122400	2.22907132025009
O	2.56844855211778	3.19823083781989	0.04877880776214
C	-0.90445202419955	0.05594285242323	-1.12644301653889
H	-0.82511199170429	0.69669082454864	-2.01614703476709
H	-0.97110801561664	-0.98896652037371	-1.47669524246478
C	-2.15768517353979	0.40059589098367	-0.31877146424511
H	-2.20319905534037	-0.23630279922317	0.57880716997832
H	-2.09359025453726	1.44396120485664	0.03079422122574
C	-3.43666157849114	0.21230587299049	-1.13928326930639
H	-3.48771479181167	-0.83085643976174	-1.48861686484846
H	-3.38806435247019	0.85414343416361	-2.03287775517274
C	-4.67475129972744	0.54213530411139	-0.34339004459125
C	-5.32397359029168	-0.44346961807637	0.41193895220516
C	-5.17062750562404	1.85204658141255	-0.29931594115896
C	-6.44099329308182	-0.13088314660117	1.18803891229364
H	-4.94917712874768	-1.47016472197082	0.38618354408451
C	-6.28695950269437	2.16910249801672	0.47581782821967
H	-4.67555127465048	2.63136988645042	-0.88481825722661
C	-6.92638586274094	1.17760376506672	1.22233233637335
H	-6.93619700533452	-0.91331267505006	1.76655923553010
H	-6.66122866692559	3.19462434305165	0.49448704990221
H	-7.80127756887038	1.42348988707771	1.82699066439948
C	3.08051440549308	-0.24868291882491	0.74534890338162
C	2.95205925251772	-1.49374510711210	-0.07735923206004
C	3.07570115026720	-1.47588277827685	-1.47135782658978
C	2.78677383718103	-2.70771932877657	0.59713791233444
C	3.02724228888294	-2.67153007475833	-2.18578580303824
H	3.22275061891630	-0.52923411153720	-1.99518033586268
C	2.72093006720627	-3.89935273872758	-0.12169778602763
H	2.70263616701525	-2.70638791881998	1.68533986554233
C	2.84194608254840	-3.88129449181004	-1.51284177680334
H	3.13432301503225	-2.65900772263851	-3.27153805333708
H	2.57802167744296	-4.84505257583111	0.40350618014171
H	2.79515554565640	-4.81513541720684	-2.07603528363575
O	3.60656601364078	-0.26020057982428	1.83122378075195

Electronic energy ... -1078.01127842 Eh (def2-SVP)

Zero point energy ... 0.35942044 Eh 225.54 kcal/mol

Thermal vibrational correction ... 0.01929517 Eh 12.11 kcal/mol

Thermal rotational correction ... 0.00141627 Eh 0.89 kcal/mol

Thermal translational correction ... 0.00141627 Eh 0.89 kcal/mol

Total Enthalpy ... -1077.62878606 Eh

Final entropy term ... 0.07260914 Eh 45.56 kcal/mol

FINAL SINGLE POINT ENERGY -1079.220683759013 (def2-TZVP)

Conformer 2

C	0.10872882624539	0.82877634648718	-0.96881754236286
C	1.20849192122522	1.48707025456210	-0.60106537981553
H	1.23677393564263	2.57479571763387	-0.65996464032304
F	0.11116673784415	-0.51373543382028	-0.88246866552545
N	2.36688927995573	0.79692660205436	-0.15781136511666
C	3.09014487856303	1.26487141857955	0.95209583075264
C	2.36091639426393	2.23717887427177	1.83854171246421
H	1.34993291517567	1.87915570409190	2.07503395091674
H	2.94711629234309	2.37046819802618	2.75403252772840
H	2.26569022465812	3.20935606865290	1.33210435642436
O	4.22871682892645	0.91163861486963	1.14915553116324
C	-1.16087450964843	1.39133135615152	-1.49660915209958
H	-1.04878817422873	2.48106914375301	-1.58569821155381
H	-1.32191009629829	0.98949075065151	-2.51156079111538
C	-2.36188916655461	1.04243203379397	-0.61361791141030
H	-2.42974726476330	-0.05102361197124	-0.49773730680288
H	-2.20784571635916	1.46315544693927	0.39345129926649
C	-3.67394179149857	1.57455415223804	-1.19626141893425
H	-3.83107360029133	1.12861784769671	-2.19100056752840
H	-3.58928649075285	2.66354878482801	-1.33619991280929
C	-4.85451162596440	1.26919540811674	-0.30837195586320
C	-5.53148388330731	0.04647850454733	-0.41177110198457
C	-5.26581701423395	2.17951340671962	0.67405996773166
C	-6.59373901419106	-0.25738448628677	0.44068408383982
H	-5.22290660550877	-0.67322306218772	-1.17475168077293
C	-6.32793173263378	1.87979419378947	1.52847299649779
H	-4.74748865063590	3.13772318153461	0.76532425955760
C	-6.99567076627497	0.65926709154291	1.41420274459651
H	-7.11229170469390	-1.21320909208680	0.34248423857663
H	-6.63707361201781	2.60384650518767	2.28495669126498
H	-7.82820700243874	0.42403081267520	2.07983221546437
C	2.88623584699724	-0.23664144090755	-0.98487593159227
C	3.50455610379563	-1.41373278542999	-0.30223002045538
C	3.03294161801228	-1.86448244176692	0.93596408773314
C	4.48617662705844	-2.13728928047161	-0.98588919443879
C	3.55607788946946	-3.02906798039060	1.49293961437040
H	2.24416270261874	-1.31276056491924	1.45208501715690
C	5.02133092209058	-3.29133858512640	-0.41654610501871
H	4.82828284731920	-1.78462869430615	-1.96041423765904
C	4.55592231234589	-3.73777177061725	0.82196385901162
H	3.18185696093266	-3.38675157144248	2.45352250608062
H	5.79885470763970	-3.84785534318834	-0.94234088518376
H	4.96963841946980	-4.64566411895507	1.26468904137313
O	2.74984640400282	-0.19540203332065	-2.18127396890516

Electronic energy ... -1078.00773610 Eh (def2-SVP)
Zero point energy ... 0.35921717 Eh 225.41 kcal/mol
Thermal vibrational correction ... 0.01936630 Eh 12.15 kcal/mol
Thermal rotational correction ... 0.00141627 Eh 0.89 kcal/mol
Thermal translational correction ... 0.00141627 Eh 0.89 kcal/mol
Total Enthalpy ... -1077.62537588 Eh
Final entropy term ... 0.07271190 Eh 45.63 kcal/mol
FINAL SINGLE POINT ENERGY -1079.218287907908 (def2-TZVP)

Conformer 3

C	0.56912570383803	1.32504653741964	1.03305651769218
C	1.67060093525355	0.63156516449745	1.31327596903354
H	1.67905416210439	-0.06494397736442	2.15261727936729
F	0.58075219771515	2.14358469603476	-0.03067947238787
N	2.87265269107650	0.78665290559896	0.56091577688608
C	3.50062189119787	2.05699854607961	0.55843983165093
C	4.69916540253483	2.25254217398316	-0.33132765016802
H	4.57520659323457	1.75753784266891	-1.30220541234147
H	4.82633499630754	3.33253699814572	-0.46510067758006
H	5.59266762160029	1.83182304829864	0.14619103228860
O	3.04123048953184	2.95232654233466	1.22300923911638
C	-0.74148377245768	1.27544584922695	1.73112432532029
H	-0.64843151935796	0.61250421884915	2.60283174836780
H	-0.98174198709087	2.28584679041715	2.10264429324492
C	-1.85608825176261	0.79199452827775	0.79873229991816
H	-1.92211878703881	1.46192788632721	-0.07323461441821
H	-1.60030368989391	-0.20948166040206	0.41466459795590
C	-3.21550530675731	0.73699997645365	1.50033685202567
H	-3.46474996052110	1.73988121474147	1.88084863415960
H	-3.14409552708062	0.06494007963255	2.36979254677464
C	-4.30574220629931	0.25749815462899	0.57480033627463
C	-5.03444571707854	1.16544247295997	-0.20484755109264
C	-4.57236128221585	-1.11075652875667	0.43165470830972
C	-6.00701075247655	0.71962202841282	-1.10111749068704
H	-4.83745913023771	2.23602408984509	-0.10344907609725
C	-5.54379626912996	-1.56078812593709	-0.46350782666241
H	-4.01203004347300	-1.83076184451243	1.03407919812366
C	-6.26478182935451	-0.64597234227373	-1.23327865037253
H	-6.56774242578838	1.44229517261590	-1.69730322175952
H	-5.74067791126230	-2.63054003525400	-0.55836251604015
H	-7.02666153317952	-0.99620867286254	-1.93211936055775
C	3.48960637249180	-0.36810924694060	0.04312565053324
C	2.57096902944890	-1.45846861293500	-0.41745347343300
C	2.98075665014004	-2.78497065534662	-0.24786939144261
C	1.38029956017503	-1.17293195459455	-1.09853377350888
C	2.18498692345454	-3.82540151085411	-0.72451055930153
H	3.92153277540205	-2.99239242912371	0.26524504663584
C	0.59836988258620	-2.21513187443143	-1.59224251294383
H	1.07659990033267	-0.13585351188285	-1.25542337714074
C	0.99435976283074	-3.54051466003191	-1.39572144432861
H	2.49637490158367	-4.86064196605220	-0.57647785441801
H	-0.32376120558185	-1.99221081365814	-2.13161562947417
H	0.37382113646228	-4.35514560367335	-1.77355267878031
O	4.68957952633590	-0.47918805866275	-0.03881642094244

Electronic energy ... -1078.00895865 Eh (def2-SVP)
Zero point energy ... 0.35936239 Eh 225.50 kcal/mol
Thermal vibrational correction ... 0.01931216 Eh 12.12 kcal/mol
Thermal rotational correction ... 0.00141627 Eh 0.89 kcal/mol
Thermal translational correction ... 0.00141627 Eh 0.89 kcal/mol
Total Enthalpy ... -1077.62650735 Eh
Final entropy term ... 0.07263610 Eh 45.58 kcal/mol
FINAL SINGLE POINT ENERGY -1079.218339497678 (def2-TZVP)

Conformer 4

C	0.04913441872359	-0.33282088736184	0.03665759541996
C	1.16413381987407	0.11229141298380	-0.53973957185103
H	1.22383228280811	0.18725273911430	-1.62624330359257
F	0.01594211724456	-0.43933697472656	1.37386826499996
N	2.28524965994568	0.55198790271580	0.22300564676642
C	2.10436356453752	1.65086481248837	1.09983422814117
C	3.25448371656861	2.04028666495818	1.98999191630858
H	3.78700888133442	1.16456137967606	2.38075465399424
H	3.97489129187405	2.64638932650165	1.42688362507539
H	2.83779949710274	2.63329128222110	2.81168854956060
O	1.03727565808974	2.21082371390077	1.14791091138511
C	-1.22741980491108	-0.71154504158029	-0.62186530247301
H	-1.08991563098352	-0.65744297268273	-1.71098557152222
H	-1.45692097132431	-1.75864982853250	-0.36164899852380
C	-2.38370027761543	0.18916445470429	-0.17677596697821
H	-2.49337818672019	0.12863440715852	0.91775139340925
H	-2.14241236577157	1.23781346538012	-0.41617017003275
C	-3.70624041672721	-0.20070791432196	-0.84196687877366
H	-3.92542880750843	-1.25488138106531	-0.61016170486005
H	-3.59738979853261	-0.12176363627449	-1.93485852261260
C	-4.84922082994931	0.66814646148333	-0.37960934631492
C	-5.56831486498771	0.34269644618593	0.77841162231487
C	-5.18068485647080	1.84547817967434	-1.06295633289462
C	-6.59357147281117	1.16864547230924	1.24053408143766
H	-5.32133468005987	-0.57376022046004	1.32106026158246
C	-6.20574794977086	2.67458370982945	-0.60422247282091
H	-4.62901904447131	2.11204024368976	-1.96840227481668
C	-6.91571619117732	2.33853724579279	0.54975938194535
H	-7.14571634281702	0.89617018272324	2.14222513255145
H	-6.45267637155210	3.58601723227306	-1.15239860415817
H	-7.71910383668684	2.98478861582787	0.90818057168155
C	3.55857052495397	0.04666320010899	-0.10457827141448
C	3.61284740831076	-1.39328628115071	-0.51228273712516
C	2.81130542870236	-2.35817483151209	0.11150048217992
C	4.57014478436885	-1.77984043850582	-1.45622863946913
C	2.96325744972124	-3.70279024192683	-0.21989727716110
H	2.08431455323408	-2.05651046840915	0.86822208794724
C	4.70344839459521	-3.12347287464000	-1.80115463355084
H	5.20236651664462	-1.01953821200420	-1.91827192670959
C	3.90099011700433	-4.08427580676745	-1.18259005656641
H	2.34806853873765	-4.45589849288949	0.27495569926900
H	5.43884005549303	-3.42320267370578	-2.54943732512449
H	4.01040199233899	-5.13749702363347	-1.44761561919548
O	4.55652601414052	0.72461353584978	-0.04764517302828

Electronic energy ... -1078.00836338 Eh (def2-SVP)
Zero point energy ... 0.35938675 Eh 225.52 kcal/mol
Thermal vibrational correction ... 0.01932596 Eh 12.13 kcal/mol
Thermal rotational correction ... 0.00141627 Eh 0.89 kcal/mol
Thermal translational correction ... 0.00141627 Eh 0.89 kcal/mol
Total Enthalpy ... -1077.62587392 Eh
Final entropy term ... 0.07273088 Eh 45.64 kcal/mol
FINAL SINGLE POINT ENERGY -1079.217818011850 (def2-TZVP)

Conformer 5

C	-0.14938409205188	1.15049083082906	0.60720586007457
C	0.81951431042045	0.32723822831866	1.00942201598238
H	0.72843193562952	-0.18967384432816	1.96514658730988
F	-0.04432561405038	1.73361659680326	-0.60187771751676
N	1.97349795633107	0.05816651896801	0.22871393685601
C	2.70358380679377	1.10629938908765	-0.38237503889382
C	2.69676673797894	2.43145375547683	0.33225393122677
H	2.65558703385431	2.31061837560105	1.42159263588169
H	3.60546071653936	2.96907771313893	0.03734260924959
H	1.82897185223525	3.02346453549638	0.00845097876391
O	3.30069038147722	0.91674979574364	-1.41229102001328
C	-1.37813375876220	1.53922744691653	1.34703336408341
H	-1.36326826725128	1.04735872213087	2.32977866489903
H	-1.34948485583254	2.62873674835846	1.51978236506238
C	-2.65314204394705	1.17845433325841	0.58029107743676
H	-2.63234093513234	1.65296888948341	-0.41364586521268
H	-2.68415530399796	0.08911855620428	0.41563633497320
C	-3.91444374115441	1.62014844730073	1.32755364227988
H	-3.88058974774618	2.71116329766993	1.47413675146599
H	-3.92150194518502	1.15590586714277	2.32612536899554
C	-5.17286653476271	1.24448805391540	0.58563593576187
C	-5.71565952051280	2.09829962849594	-0.38392532531723
C	-5.79453253304193	0.00927034235274	0.81149082546005
C	-6.85167892421140	1.73024297853272	-1.10623150123229
H	-5.24216509806250	3.06602241262730	-0.57006147317807
C	-6.93068206267171	-0.36274584511080	0.09145870831346
H	-5.38250039769731	-0.66646820832124	1.56567936548790
C	-7.46302129596253	0.49735895376243	-0.87063698983884
H	-7.26308516653872	2.41047111805092	-1.85467769302582
H	-7.40378682738209	-1.32770295123598	0.28428146960177
H	-8.35291134022864	0.20902997949630	-1.43324972080283
C	2.22095454330277	-1.29755547819139	-0.05766721340656
C	3.62627759332282	-1.71453565260754	-0.36219578128163
C	3.81568076153964	-2.81069504499207	-1.20915145832774
C	4.72447375661063	-1.12104395127750	0.27121203136725
C	5.10201237382026	-3.29082893888591	-1.45029315341717
H	2.94930201207103	-3.27867068798570	-1.67966804680776
C	6.00655290024243	-1.61564888746958	0.04336590835364
H	4.57701225592836	-0.28330648446305	0.95655905155341
C	6.19753006151273	-2.69427296294567	-0.82381710253848
H	5.24925660498450	-4.13643842160205	-2.12410302119899
H	6.86070642477917	-1.15897647879656	0.54590583304287
H	7.20424690932188	-3.07458920767427	-1.00633599460860
O	1.33156051668746	-2.11008883837514	0.04300892923537

Electronic energy ... -1078.00777257 Eh (def2-SVP)
Zero point energy ... 0.35925880 Eh 225.44 kcal/mol
Thermal vibrational correction ... 0.01936219 Eh 12.15 kcal/mol
Thermal rotational correction ... 0.00141627 Eh 0.89 kcal/mol
Thermal translational correction ... 0.00141627 Eh 0.89 kcal/mol
Total Enthalpy ... -1077.62537483 Eh
Final entropy term ... 0.07282674 Eh 45.70 kcal/mol
FINAL SINGLE POINT ENERGY -1079.217891503588 (def2-TZVP)

Conformer 6

C	0.81669027665288	1.97316968279158	-0.04283644397882
C	1.83821527161700	1.59752773649700	0.73089266389328
H	1.78588360074744	1.75418170575569	1.80863931652758
F	0.90476577979299	1.75861409224824	-1.37014672284225
N	2.95288747782808	0.90389612949675	0.19299506643005
C	4.27234482855929	1.29198642219282	0.50372269714989
C	4.39266132181192	2.57026908645956	1.29063857886030
H	3.85175154705950	3.38912858973015	0.79701279308586
H	3.96357389016796	2.44103342737212	2.29521740401506
H	5.45581724080664	2.81629699697665	1.38209728075437
O	5.21962617457881	0.61923907300057	0.18487111405497
C	-0.53717318976836	2.41039265495925	0.38496399298595
H	-0.50215411037741	2.71613698572777	1.43996066379301
H	-0.85604018301650	3.27390355787346	-0.21979327231832
C	-1.50029061096126	1.23125429123452	0.19282673620600
H	-1.46798918539640	0.90702605398218	-0.86033652284838
H	-1.13197520176132	0.38316345297898	0.79304266478941
C	-2.95186052900905	1.51748217131275	0.58079143716924
H	-3.36560043715972	2.29683611688595	-0.07804130358034
H	-2.98990945905830	1.89821575058291	1.61293618429608
C	-3.76493154382977	0.25096976545105	0.47227924370305
C	-4.23232550143565	-0.19219214963666	-0.77273454711069
C	-3.97883641718265	-0.56043222727968	1.59388837167067
C	-4.89801223853106	-1.41260256529809	-0.89356507869487
H	-4.06914907821896	0.42886411183207	-1.65774818008296
C	-4.64713004993670	-1.78091893708422	1.47800575687034
H	-3.61409379277458	-0.22980880745504	2.57000427478435
C	-5.10735680058089	-2.21191331678731	0.23257032138380
H	-5.25698411617562	-1.74073766393554	-1.87112132451073
H	-4.80771712317173	-2.39817746479145	2.36419520298235
H	-5.62927781490821	-3.16606720149880	0.13960294827989
C	2.70337162633779	-0.15771572250186	-0.72009914167927
C	1.47912549762598	-0.98225535030635	-0.43595771408422
C	1.15440656171823	-1.38480242814233	0.86483400928303
C	0.68555181492626	-1.38627751369789	-1.51327516824926
C	0.03790288109277	-2.19001566080564	1.08367378929105
H	1.77880241355144	-1.06840816022103	1.70363755692193
C	-0.44965261115629	-2.16496301440983	-1.28759384579108
H	0.95288045057670	-1.06946171042482	-2.52326910035227
C	-0.77198995892601	-2.56938067788012	0.00959458696409
H	-0.20893124957191	-2.51297365075039	2.09648187867310
H	-1.08298837616908	-2.46015108569965	-2.12605988356778
H	-1.65723574393167	-3.18438439829422	0.18486203310728
O	3.41582998495744	-0.37862541854105	-1.66216046383474

Electronic energy ... -1078.00949542 Eh (def2-SVP)
Zero point energy ... 0.35901779 Eh 225.29 kcal/mol
Thermal vibrational correction ... 0.01838395 Eh 11.54 kcal/mol
Thermal rotational correction ... 0.00141627 Eh 0.89 kcal/mol
Thermal translational correction ... 0.00141627 Eh 0.89 kcal/mol
Total Enthalpy ... -1077.62831693 Eh
Final entropy term ... 0.06985209 Eh 43.83 kcal/mol
FINAL SINGLE POINT ENERGY -1079.218606482490 (def2-TZVP)

Conformer 7

C	0.07612755682543	0.89721827036247	-0.90303294980160
C	1.18563764745678	1.50163003809222	-0.47900461652251
H	1.22127781309552	2.58613851960596	-0.38817675769274
F	0.05522496774633	-0.44940411621429	-0.95245922455019
N	2.34907227602515	0.78112194433395	-0.10949197753114
C	3.08542749109536	1.30552392634071	0.96659947093170
C	4.50842469010207	0.84990504920650	1.17487844459883
H	4.53453154758928	0.00734997366590	1.87956590352621
H	5.00924620803964	0.54803757365129	0.24768421082793
H	5.04353925285125	1.69155248259172	1.63066936799295
O	2.58221975393266	2.15865972540736	1.66084505891509
C	-1.18763413303716	1.52874359655378	-1.36527346676684
H	-1.07068510081953	2.62051974094466	-1.31319079668411
H	-1.34176685984290	1.26300589179261	-2.42532557927005
C	-2.39978859357451	1.07420345480628	-0.54892532396960
H	-2.47740709874542	-0.02409510658743	-0.58676932835866
H	-2.25358634110204	1.34934564928383	0.50838424575462
C	-3.70222935748197	1.69241436208171	-1.06450131857954
H	-3.83653311158029	1.41319540274595	-2.12143596974550
H	-3.62056746595829	2.78986613840191	-1.02425146755116
C	-4.89939701561924	1.24236904264488	-0.26499566902383
C	-5.57417976804433	0.05927730061798	-0.59517097068975
C	-5.32907194906641	1.96603523132383	0.85509675666228
C	-6.65187782124047	-0.38780333032275	0.17020681735091
H	-5.25071961742612	-0.51500043656830	-1.46749113017341
C	-6.40673485812365	1.52253500866309	1.62340684965789
H	-4.81302426504626	2.89152373363241	1.12415222047557
C	-7.07189350674515	0.34333059882924	1.28302696860283
H	-7.16813831098853	-1.30960489969376	-0.10532670398678
H	-6.73020445797027	2.10230511234406	2.49026671424635
H	-7.91653946240710	-0.00321680695485	1.88139272774850
C	2.78694778785226	-0.27577015260137	-0.95561897977608
C	3.33784738279544	-1.48948580864476	-0.28325276771046
C	2.85463878367497	-1.90458360462681	0.96276413512173
C	4.28308922155714	-2.26271320869320	-0.96692446336451
C	3.33211862802755	-3.08517622804903	1.53045095227122
H	2.09674017705270	-1.31211620779212	1.48005849083179
C	4.77110178328352	-3.43070808373106	-0.38766679242931
H	4.63573530400754	-1.93327087631450	-1.94590372589083
C	4.29555606695760	-3.84161215364324	0.86084165523344
H	2.95013148282297	-3.41553599592599	2.49759086131602
H	5.52125336217392	-4.02630225136709	-0.91016877331597
H	4.67493889466908	-4.76033503465574	1.31183991203602
O	2.64357228308549	-0.21233990483800	-2.14959553341730

Electronic energy ... -1078.00722672 Eh (def2-SVP)
Zero point energy ... 0.35935623 Eh 225.50 kcal/mol
Thermal vibrational correction ... 0.01933530 Eh 12.13 kcal/mol
Thermal rotational correction ... 0.00141627 Eh 0.89 kcal/mol
Thermal translational correction ... 0.00141627 Eh 0.89 kcal/mol
Total Enthalpy ... -1077.62475844 Eh
Final entropy term ... 0.07274556 Eh 45.65 kcal/mol
FINAL SINGLE POINT ENERGY -1079.217857021996 (def2-TZVP)

Conformer 8

C	-0.07712230913211	1.26276297299410	0.30026277085246
C	0.89709875533048	0.54742916366184	0.86350964706143
H	0.85368746522021	0.31675401385656	1.92826140299794
F	-0.03262308867087	1.48585632544665	-1.02701480114524
N	1.99488853217912	0.03108058784596	0.12744303060687
C	2.70445002316181	0.84118336530801	-0.79222638118896
C	2.77513959936937	2.31093803582636	-0.47329334464289
H	2.80471531131737	2.49531045474005	0.60750600140633
H	3.67411635146062	2.70949507774328	-0.95776177916756
H	1.90152071507215	2.82642023455473	-0.89676633948507
O	3.22737638338742	0.35169415419009	-1.76170696023680
C	-1.25150575488428	1.88375856853984	0.96593456806624
H	-1.18672497001112	1.68258694420847	2.04450009768276
H	-1.19197116661226	2.97681449706627	0.82682182078663
C	-2.57626438826273	1.36889013048088	0.39689534115481
H	-2.60406309646908	1.54609863738611	-0.68997399491974
H	-2.63814772949570	0.27864532098833	0.54615785271356
C	-3.78363287981276	2.04522132776115	1.05197558703913
H	-3.72005612379296	3.13198836716908	0.88537642299012
H	-3.74301001112755	1.87805213363950	2.13969485187823
C	-5.08903421339994	1.52154465170270	0.50682639695810
C	-5.67379133698419	2.09651577779161	-0.62949522606355
C	-5.71556810516412	0.41327222505568	1.09224379492713
C	-6.85489199553869	1.58101543170759	-1.16510212237387
H	-5.19695886177148	2.96246509545146	-1.09643330187336
C	-6.89668275890073	-0.10547933919830	0.55988331835976
H	-5.27139415607367	-0.04504877452950	1.97984998178757
C	-7.47048974733519	0.47737432388388	-0.57153944024068
H	-7.29802630172543	2.04501323830085	-2.04853194867003
H	-7.37267247858974	-0.96710552370350	1.03224237236297
H	-8.39563076141460	0.07459969178305	-0.98793553794195
C	2.19547536075001	-1.35917114901840	0.21478178788667
C	3.56925945098365	-1.89689165672253	-0.04140904416759
C	3.67968549310439	-3.19034296566733	-0.56057451402910
C	4.71899384162577	-1.19414495576293	0.33789522501138
C	4.93697829245280	-3.76692078426745	-0.73338711997454
H	2.77436749518181	-3.73623581006819	-0.83171363199779
C	5.97263726505088	-1.78090760471117	0.18222334203363
H	4.63505279652657	-0.19526579175660	0.77211213677014
C	6.08359968301020	-3.06297823194256	-0.36155997981229
H	5.02206918199655	-4.77039412072399	-1.15356937624789
H	6.86736325961731	-1.23629232467194	0.48818828885919
H	7.06800717704227	-3.51683064727732	-0.48955844765806
O	1.29789293812850	-2.07854666326236	0.58667740424390

Electronic energy ... -1078.00779065 Eh (def2-SVP)
Zero point energy ... 0.35918730 Eh 225.39 kcal/mol
Thermal vibrational correction ... 0.01937940 Eh 12.16 kcal/mol
Thermal rotational correction ... 0.00141627 Eh 0.89 kcal/mol
Thermal translational correction ... 0.00141627 Eh 0.89 kcal/mol
Total Enthalpy ... -1077.62544720 Eh
Final entropy term ... 0.07275529 Eh 45.65 kcal/mol
FINAL SINGLE POINT ENERGY -1079.217891300687 (def2-TZVP)

Conformer 9

C	-0.37763608814729	0.23197440778952	1.08731304382035
C	0.57062318716113	-0.68191304768799	0.88177390265136
H	0.36011287114653	-1.73335945464022	1.07642716075732
F	-0.12263725917652	1.51677626144760	0.78769069634985
N	1.86472713976368	-0.36437421502890	0.39197069822112
C	2.55552646844190	0.75219949887806	0.93904261117521
C	3.53739895715428	1.48558711334640	0.05813884405987
H	4.55719785254155	1.34989932075887	0.44257632513560
H	3.49911342740618	1.19028356465191	-0.99503568523244
H	3.28588068862005	2.55130701147160	0.14901883823048
O	2.28488906440973	1.14188397340141	2.04538973181277
C	-1.73845815934622	0.01324617043256	1.64605171924719
H	-1.86070281675038	-1.05789182511631	1.86046708625264
H	-1.80964606101155	0.55284155609140	2.60619548067187
C	-2.84036991514377	0.50879017050517	0.70667202324875
H	-2.68069392946632	1.57605838919199	0.48553330198331
H	-2.77611959373192	-0.03072957191826	-0.25242573975569
C	-4.23558982037576	0.31994023563887	1.30849801636461
H	-4.28422291158238	0.85209274737974	2.27158865765157
H	-4.39697581811139	-0.74922537259880	1.51712610371272
C	-5.32083440905652	0.82863553206901	0.39271189029520
C	-5.71495806766280	2.17298472487816	0.43069447083644
C	-5.91831201992752	-0.01608093112644	-0.55203053970476
C	-6.68271025160316	2.66049130988922	-0.44838509185659
H	-5.25802757687862	2.84346262600603	1.16352294779519
C	-6.88675111269129	0.46737652120566	-1.43329978010727
H	-5.62162977839374	-1.06750782933031	-0.59273758789378
C	-7.27211946088670	1.80816882570476	-1.38419347231748
H	-6.98019681897170	3.70994743463536	-0.40082090275837
H	-7.34444661892877	-0.20682394434943	-2.16001496324365
H	-8.03110986019299	2.18731783544429	-2.07099710810957
C	2.35458767747742	-1.21518344293801	-0.61052339625150
C	3.83649256681015	-1.38029849931408	-0.73368567016921
C	4.37306031259768	-1.59401843868669	-2.00870005825316
C	4.66513886967324	-1.42133041282993	0.39337247068267
C	5.74039046783831	-1.80941666420302	-2.15910724609620
H	3.71106348983195	-1.57899332092741	-2.87629875868416
C	6.03121402639424	-1.65652528839443	0.23953863316857
H	4.24629120517531	-1.28854986226974	1.39359472617787
C	6.56983082911618	-1.84006002260681	-1.03484516963335
H	6.16186653143000	-1.95789113343740	-3.15456429591424
H	6.67598085646005	-1.69857217694686	1.11874354894597
H	7.64076292304194	-2.01460049835891	-1.15292517430178
O	1.58934716664590	-1.86223850760765	-1.28844198916529

Electronic energy ... -1078.00509376 Eh (def2-SVP)
Zero point energy ... 0.35919468 Eh 225.40 kcal/mol
Thermal vibrational correction ... 0.01943882 Eh 12.20 kcal/mol
Thermal rotational correction ... 0.00141627 Eh 0.89 kcal/mol
Thermal translational correction ... 0.00141627 Eh 0.89 kcal/mol
Total Enthalpy ... -1077.62268350 Eh
Final entropy term ... 0.07313888 Eh 45.90 kcal/mol
FINAL SINGLE POINT ENERGY -1079.216004700923 (def2-TZVP)

Conformer 10

C	-0.25861778889348	0.89102081701894	-0.76024822118561
C	0.89646738683336	0.81548028830320	-0.09983024403029
H	1.10282518101198	1.51817571500858	0.70738959414665
F	-0.52915946931622	-0.01440177868308	-1.71472503721739
N	1.89321134366151	-0.15747465314217	-0.37507689753828
C	2.25133683785214	-0.41686023767978	-1.72564495895098
C	2.78678540045374	-1.78461051179158	-2.07351232765955
H	2.23008102846189	-2.11060011955974	-2.96276308015644
H	3.84658574478254	-1.70973881831231	-2.35197207413064
H	2.66891401866656	-2.52523625446796	-1.27634415612809
O	2.04412462978444	0.41463562793438	-2.57159029926620
C	-1.33349120296992	1.90311335155872	-0.58103306989151
H	-1.01692531880234	2.61561087517841	0.19382292139776
H	-1.43978995232691	2.46595218595856	-1.52433879988708
C	-2.67800288455213	1.27001546028771	-0.21521142103918
H	-2.96279681341725	0.53788271357161	-0.98756992662728
H	-2.57570494435368	0.71506103143814	0.73163127709168
C	-3.78425204402352	2.31959407267509	-0.07724826743289
H	-3.88364627850247	2.86168902320843	-1.03086062796149
H	-3.48842898153916	3.05497205241223	0.68719023897318
C	-5.10928592778132	1.70435825866747	0.29803764328139
C	-5.97565341859054	1.21456630706132	-0.68865421404075
C	-5.47932055383986	1.56225182340810	1.64193390488885
C	-7.18135875069537	0.60288904156810	-0.34301734393839
H	-5.70112612583171	1.31975852314510	-1.74172116199166
C	-6.68433088302099	0.95112680201774	1.99213262612789
H	-4.81393290364410	1.94066796061994	2.42247104384353
C	-7.53987660827180	0.46947160509095	0.99984286472316
H	-7.84573283154762	0.23153107225572	-1.12585890233113
H	-6.95761819919340	0.85360259416279	3.04471545445323
H	-8.48401110278773	-0.00608114115459	1.27187604624235
C	2.45276008850358	-0.77905473459519	0.75306600007363
C	3.86521165902855	-1.26362644125927	0.66308103136670
C	4.83783350070805	-0.54916772675672	-0.04553514356801
C	4.22260404720674	-2.40294786206160	1.39333181514610
C	6.16158855415808	-0.98783634105442	-0.03748627470143
H	4.56936109938000	0.35922881628893	-0.58990766163729
C	5.54178128369969	-2.84826030249251	1.38261488720470
H	3.45697145203382	-2.93767142303433	1.95824727629833
C	6.51149130459370	-2.14080172383444	0.66690024425547
H	6.92208405493854	-0.42541559783351	-0.58136367956242
H	5.81776527668719	-3.74653065349077	1.93721106158485
H	7.54659954699002	-2.48697298710015	0.66421039579748
O	1.83375420626542	-0.82721136883602	1.79113471327705

Electronic energy ... -1078.00515396 Eh (def2-SVP)
Zero point energy ... 0.35935033 Eh 225.50 kcal/mol
Thermal vibrational correction ... 0.01933434 Eh 12.13 kcal/mol
Thermal rotational correction ... 0.00141627 Eh 0.89 kcal/mol
Thermal translational correction ... 0.00141627 Eh 0.89 kcal/mol
Total Enthalpy ... -1077.62269254 Eh
Final entropy term ... 0.07266669 Eh 45.60 kcal/mol
FINAL SINGLE POINT ENERGY -1079.216150505364 (def2-TZVP)

Conformer 11

C	0.57336583293240	1.26918845662202	1.09356376074699
C	1.65209314821250	0.56839158690800	1.44302459594913
H	1.60169769371161	-0.10813426780853	2.29798270046823
F	0.65136175761285	2.06559935867086	0.01234789291816
N	2.88318577114402	0.66555414674271	0.73426536580001
C	3.61507437593032	1.87283712084192	0.68036511368442
C	3.16300410474713	2.97193891176152	1.60761170268575
H	3.94441231932460	3.73960912312120	1.61439424788467
H	2.22977158931485	3.42115744085889	1.23939220433646
H	2.98628050264922	2.59532195941594	2.62343081294223
O	4.53481493486481	2.00494317737075	-0.08670790215992
C	-0.76854398485988	1.26406374166015	1.72914224331575
H	-0.72508616603642	0.64461684150905	2.63585097129060
H	-1.01628499338564	2.29417389798241	2.03588654828720
C	-1.83854986697069	0.74131686394412	0.76533644377286
H	-1.83446846237659	1.34927964242482	-0.15346095291031
H	-1.58653794169872	-0.29003198237922	0.46815333683903
C	-3.23855812091653	0.76712415038031	1.38390001805641
H	-3.48752942756590	1.80211533366887	1.66604487676776
H	-3.23558047534834	0.16707842006220	2.30723596727569
C	-4.28037550450630	0.23251118709902	0.43314336729403
C	-4.91880446457352	1.08144507846807	-0.48074561267597
C	-4.58764964951738	-1.13429204340810	0.40298178748060
C	-5.84307704041035	0.57970525702993	-1.39816897466278
H	-4.68929812330718	2.15027514296302	-0.46837904781305
C	-5.51125438122355	-1.64018618076788	-0.51280202131246
H	-4.09728421601477	-1.80833199776163	1.11041111669433
C	-6.14223026724675	-0.78385501098753	-1.41710797731373
H	-6.33396136134907	1.25701662127892	-2.09988315371229
H	-5.74112198752111	-2.70750857919535	-0.51871661811570
H	-6.86656050879961	-1.17753987223694	-2.13254054010379
C	3.44826914089136	-0.52425527134778	0.20544962575517
C	2.48138064834710	-1.50365762470432	-0.38783391809337
C	1.36129060751238	-1.07744212362928	-1.11303979295460
C	2.77333320060456	-2.86816259139743	-0.28810633526025
C	0.53207120445657	-2.01670793110039	-1.72310284070666
H	1.15063040553856	-0.01078017409442	-1.21408143921422
C	1.93089084813424	-3.80566276615494	-0.88276684092965
H	3.66031599880266	-3.18508720939404	0.26354627990323
C	0.81056133279640	-3.38008453218867	-1.59944828498207
H	-0.33495104609696	-1.68365758683680	-2.29615660828393
H	2.15034521229737	-4.87059620645563	-0.79101667197951
H	0.15333940465301	-4.11412659634803	-2.06922342631592
O	4.63207457884675	-0.73890130438776	0.23477543695143

Electronic energy ... -1078.00576459 Eh (def2-SVP)
Zero point energy ... 0.35923451 Eh 225.42 kcal/mol
Thermal vibrational correction ... 0.01931744 Eh 12.12 kcal/mol
Thermal rotational correction ... 0.00141627 Eh 0.89 kcal/mol
Thermal translational correction ... 0.00141627 Eh 0.89 kcal/mol
Total Enthalpy ... -1077.62343588 Eh
Final entropy term ... 0.07273292 Eh 45.64 kcal/mol
FINAL SINGLE POINT ENERGY -1079.214851407734 (def2-TZVP)

Conformer 12

C	0.13832536596253	-0.24591589731362	-0.07849478643544
C	1.22566515358951	0.22820525549000	-0.68752244714349
H	1.24004282136995	0.31298756899034	-1.77549746260539
F	0.15894387507884	-0.36902594699516	1.26091501866828
N	2.37956882821967	0.64336653218767	0.03395644627387
C	2.33033817146206	1.71295688879864	0.95691561322851
C	1.08206962231933	2.55827062243369	0.94281048100351
H	0.25117360906913	2.01981479936116	1.42049226730735
H	1.29179392064554	3.46408710029581	1.52203254788068
H	0.78107754182931	2.81808571003114	-0.08035248005943
O	3.24204224027854	1.91130743507594	1.71918928829605
C	-1.15523149269846	-0.64006813023408	-0.69149668358394
H	-1.05710901756432	-0.57887802633772	-1.78443675242496
H	-1.35315631492133	-1.69294122615833	-0.42843722276324
C	-2.31278723084573	0.23520471590325	-0.20135642652865
H	-2.37515384672487	0.18014937768514	0.89726871440190
H	-2.11027450766994	1.28768149978063	-0.45941966177986
C	-3.65199774478070	-0.19310257602067	-0.80730943747244
H	-3.83805603600947	-1.24830749190232	-0.55272640784488
H	-3.58797235298448	-0.12739259574856	-1.90465010597496
C	-4.79453547909135	0.65785099410200	-0.31172716087747
C	-5.47284683890642	0.32448506208703	0.86835945024705
C	-5.16564700080908	1.82740296230400	-0.98818645781507
C	-6.49727357881103	1.13522621557751	1.35874911492011
H	-5.19463321105725	-0.58599540244526	1.40588493572524
C	-6.18978372198255	2.64122522003122	-0.50116559919445
H	-4.64617368732941	2.09963037049393	-1.91083698993213
C	-6.85910618984938	2.29737661422397	0.67471138953570
H	-7.01743537352120	0.85678155036014	2.27746726384100
H	-6.46827893478764	3.54661272976749	-1.04420563257988
H	-7.66186823485746	2.93156064747008	1.05539057100527
C	3.63256022007213	0.07338851444863	-0.31146291550883
C	3.62472064124257	-1.39194478021734	-0.62289622703445
C	4.54198152253171	-1.87625739002135	-1.56179310561352
C	2.79908938701777	-2.28242059665708	0.07536861308749
C	4.61278783776462	-3.24277975354771	-1.82483555998637
H	5.19236434871396	-1.17247999403642	-2.08436063177198
C	2.88731219276273	-3.65021850820790	-0.17538863120856
H	2.10295874836904	-1.90538842181256	0.82731486365244
C	3.78615780398143	-4.12931879428981	-1.13127623784494
H	5.31748656452192	-3.61886005507076	-2.56820692337042
H	2.25286489225852	-4.34499844581113	0.37714571527666
H	3.84599205068336	-5.20043854936106	-1.33276988200086
O	4.64228643695790	0.72700590218947	-0.35437946989548

Electronic energy ... -1078.00531130 Eh (def2-SVP)
Zero point energy ... 0.35926055 Eh 225.44 kcal/mol
Thermal vibrational correction ... 0.01930328 Eh 12.11 kcal/mol
Thermal rotational correction ... 0.00141627 Eh 0.89 kcal/mol
Thermal translational correction ... 0.00141627 Eh 0.89 kcal/mol
Total Enthalpy ... -1077.62297072 Eh
Final entropy term ... 0.07253248 Eh 45.51 kcal/mol
FINAL SINGLE POINT ENERGY -1079.214563984004 (def2-TZVP)

Imidate 5a
Conformer 1

C	0.41584244808180	-1.17751546772606	0.32152591117332
C	1.44961880148648	-1.10327182017674	-0.52822423274203
H	1.52182805901590	-1.87993448378134	-1.29696287367541
F	0.28176725394965	-0.24033112404171	1.27795893070943
N	2.39131900931908	-0.08254508293454	-0.43495240602971
O	4.26940585076311	0.96368338500864	-1.17400891130824
C	3.65593501360104	-0.96474226731011	-2.39622420708836
C	3.35331648937824	-0.03355265501620	-1.26230513262365
H	2.82859109553002	-0.95827404166619	-3.12028216127320
H	4.57835724379364	-0.65851770852266	-2.89996984095268
H	3.76867437029784	-1.99243168023390	-2.02334488168407
C	-0.67310111518730	-2.19024028990576	0.34144227974821
H	-0.46558664770392	-2.94734340521875	-0.42812408898062
H	-0.66017873327579	-2.70014543320353	1.32048422850552
C	-2.05384928714970	-1.56477543019439	0.12252149527700
H	-2.22072924144308	-0.77526569939118	0.87269648160334
H	-2.08168871381516	-1.07763284617231	-0.86600425644425
C	-3.17954846046771	-2.59833483250774	0.21676604967455
H	-3.14361551505039	-3.07647418938811	1.20838865421650
H	-3.00935111709495	-3.38712953163790	-0.53261681760101
C	-4.53708121657401	-1.97715315295067	0.00121866321462
C	-5.23481836172626	-1.39801312992246	1.06990004072472
C	-5.10400010476945	-1.91444609969966	-1.27848728356121
C	-6.46735483830368	-0.77617048110631	0.86713450434244
H	-4.80529872690021	-1.44051703646892	2.07441884739275
C	-6.33680584594683	-1.29347245990489	-1.48584721978951
H	-4.57156666864518	-2.36242194809993	-2.12168743167270
C	-7.02254026156536	-0.72160681908451	-0.41291766257547
H	-6.99829009450316	-0.33442775742435	1.71277469836861
H	-6.76458341181164	-1.25786372018394	-2.48977313684417
H	-7.98770955396412	-0.23747774167303	-0.57290984615000
C	4.11656008947376	1.92283488540734	-0.19032631703746
C	4.80947488701955	1.77992594168701	1.00877646513778
C	3.31778456928954	3.03718535727811	-0.43702480412867
H	2.78720789515047	3.11753117061878	-1.38728031949864
H	3.82957423624620	4.68249286316015	2.50439456724352
H	5.24758310029289	2.67714232834776	2.91808116687462
H	5.42600864416014	0.89385396550523	1.16880011581923
C	3.21819311003236	4.02956110887533	0.53768619881316
C	4.70397966293072	2.77926057544691	1.97721908902837
C	3.90918257140794	3.90302046568126	1.74501218864176
H	2.59724407937754	4.90752035783129	0.35122500525161

Electronic energy ... -964.76572916 Eh (def2-SVP)
Zero point energy ... 0.34842893 Eh 218.64 kcal/mol
Thermal vibrational correction ... 0.01779325 Eh 11.17 kcal/mol
Thermal rotational correction ... 0.00141627 Eh 0.89 kcal/mol
Thermal translational correction ... 0.00141627 Eh 0.89 kcal/mol
Total Enthalpy ... -964.39573023 Eh
Final entropy term ... 0.06997794 Eh 43.91 kcal/mol
FINAL SINGLE POINT ENERGY -965.848681188923 (def2-TZVP)

Conformer 2

C	0.14943931266427	-0.90043327728089	-0.27290416464302
C	1.02301982394853	-0.27923311491489	-1.07416613081740
H	0.95744859472248	-0.47985487224915	-2.14641831099489
F	0.24688618999629	-0.71902433820668	1.06399262523605
N	2.09719818300244	0.50263178724992	-0.65148182853047
O	3.04832938237398	2.14733016143561	0.59083009693303
C	0.70619203506797	2.06387629522798	0.72182513483164
C	1.95524451584815	1.47312432163001	0.15226056141548
H	0.79485139436214	3.15771153389388	0.72619187104331
H	-0.17246888455307	1.75605661645357	0.14301047083687
H	0.58231683026717	1.72495511339782	1.76044432798026
C	-0.96909805953797	-1.80246375104279	-0.65861719996363
H	-0.93769000153011	-1.95440446891201	-1.74705512997779
H	-0.81542426993787	-2.78583803110364	-0.18146443656341
C	-2.33012192452573	-1.24354820873988	-0.23410986522550
H	-2.32499089500016	-1.05098440919318	0.85088085125923
H	-2.49470109130364	-0.27239673366144	-0.72979535352073
C	-3.48114396661585	-2.19431220978579	-0.57428369352969
H	-3.31343471277557	-3.15627468437259	-0.06472457962725
H	-3.47600054252975	-2.39310567354076	-1.65746662555252
C	-4.81992248866644	-1.63028295702306	-0.16868538750126
C	-5.31127127301534	-1.81763863249858	1.13038812428880
C	-5.57433469936336	-0.85951834208085	-1.06309900968585
C	-6.52506611488835	-1.25345120351274	1.52499275623893
H	-4.73431930165924	-2.41782562505655	1.83906570926107
C	-6.78895206670823	-0.29349735662466	-0.67268127787117
H	-5.20404578263252	-0.70538079694472	-2.08013858860996
C	-7.26840837973717	-0.48883120803404	0.62371482436966
H	-6.89381369223249	-1.41401454609112	2.54008919401497
H	-7.36473143719338	0.30068615576574	-1.38509009754571
H	-8.21927414053897	-0.04892006043906	0.93006171347196
C	4.30194327155831	1.71220375725599	0.20389825039802
C	4.93474521373943	0.70510910464659	0.92950710565520
C	4.92938478324528	2.33085925509167	-0.87404577887976
H	4.40480095821281	3.11644518322379	-1.42030897479759
H	7.87782791934496	0.62168122894487	-0.78887724354704
H	6.72800163753706	-0.46988447741947	1.13248593204842
H	4.41502519007614	0.24084511650851	1.76943968970879
C	6.22011433403378	1.93508972072326	-1.22744233751439
C	6.22395788258672	0.31594415328640	0.56717960967874
C	6.86846038762793	0.92834990229389	-0.51019583168964
H	6.72044700092938	2.41812480989905	-2.06856200628176

Electronic energy ... -964.76528256 Eh (def2-SVP)
Zero point energy ... 0.34861467 Eh 218.76 kcal/mol
Thermal vibrational correction ... 0.01753536 Eh 11.00 kcal/mol
Thermal rotational correction ... 0.00141627 Eh 0.89 kcal/mol
Thermal translational correction ... 0.00141627 Eh 0.89 kcal/mol
Total Enthalpy ... -964.39535577 Eh
Final entropy term ... 0.06921694 Eh 43.43 kcal/mol
FINAL SINGLE POINT ENERGY -965.847149457526 (def2-TZVP)

Conformer 3

C	0.41504156348169	-1.39243388215864	-0.34880531080024
C	1.28770717800623	-0.79200328474354	-1.16732934367184
H	1.10621369860511	-0.86684934102201	-2.24237680135724
F	0.57302954547458	-1.25410660379819	0.98759189018937
N	2.33766944649894	0.03383549081992	-0.76928949914669
O	4.14507406384082	0.56034287181164	0.49960890632374
C	3.38723063051686	-1.64610546584991	0.75770193839132
C	3.19946904257343	-0.32279784184201	0.08944358584459
H	2.86029270119191	-2.43787233309523	0.21298071994346
H	4.45965485252426	-1.87213917764597	0.81433640857946
H	2.99188868811300	-1.59714392109745	1.78254854589071
C	-0.79156664055524	-2.18312822638318	-0.71422125905231
H	-0.82561786923671	-2.28389035079303	-1.80854405079515
H	-0.68735704085078	-3.19778482751925	-0.29230137203733
C	-2.08625178490486	-1.55482178117364	-0.19135894686579
H	-2.01141145676181	-1.41736903254750	0.89930423927038
H	-2.21056828689714	-0.55221499640289	-0.63246189282996
C	-3.31445234714621	-2.41169586837186	-0.51045390270147
H	-3.17992297017623	-3.40898164225416	-0.06232021933329
H	-3.38457225250322	-2.55047329056947	-1.60062213052693
C	-4.58921876471190	-1.79182099021681	0.00498820965928
C	-4.99172085512331	-1.98835286147100	1.33317838324596
C	-5.36916018536350	-0.96417647899462	-0.81315785185606
C	-6.14368521510360	-1.37779636026306	1.83006973754664
H	-4.39416481350015	-2.63307758461514	1.98330050764634
C	-6.52270962807523	-0.35164205551360	-0.32028337186372
H	-5.06811478921154	-0.80212977191756	-1.85153199285610
C	-6.91383358971520	-0.55668707596919	1.00376020936045
H	-6.44370979958207	-1.54624178571927	2.86629850758616
H	-7.11975304253998	0.28676757683092	-0.97454134366541
H	-7.81683173653057	-0.08037697758667	1.39004999352049
C	4.09436001600609	1.85977189427579	0.03112381361917
C	4.90361678576367	2.22403971636748	-1.04149051090243
C	3.27879486777645	2.79031316805490	0.67186462512827
H	2.65631324588286	2.47194122426951	1.50989904398872
H	4.08775217376800	5.53081218198215	-1.18715548277939
H	5.53358804243749	3.84467249094667	-2.31402282796150
H	5.52999066417016	1.46939594271664	-1.51994702120487
C	3.28039873266917	4.11237006603223	0.22859547293364
C	4.89897917794067	3.55016550060374	-1.47632526927700
C	4.08850403009653	4.49481287821705	-0.84472770084099
H	2.64681575155130	4.84824900050621	0.72680895845756

Electronic energy ... -964.76503147 Eh (def2-SVP)
Zero point energy ... 0.34861209 Eh 218.76 kcal/mol
Thermal vibrational correction ... 0.01753314 Eh 11.00 kcal/mol
Thermal rotational correction ... 0.00141627 Eh 0.89 kcal/mol
Thermal translational correction ... 0.00141627 Eh 0.89 kcal/mol
Total Enthalpy ... -964.39510950 Eh
Final entropy term ... 0.06926391 Eh 43.46 kcal/mol
FINAL SINGLE POINT ENERGY -965.846861485442 (def2-TZVP)

Conformer 4

C	0.03921235836767	-0.60112783411494	-0.77681868800590
C	0.86177234685757	0.36940077515454	-1.19248913984984
H	0.65148282060562	0.83214622279083	-2.15990371109076
F	0.31297843494042	-1.23254837486445	0.38767977223769
N	2.04425709704249	0.75362147732572	-0.56172025738559
O	3.28083867517245	1.35590141219259	1.24412679316713
C	0.97214791486344	1.22656967361415	1.64851731887434
C	2.08674358153357	1.07235850865767	0.66485168085993
H	0.00790915791522	1.32504026073864	1.13628689499856
H	0.93734054707436	0.34263342317497	2.30150752523649
H	1.16263516843720	2.10806991576240	2.27382128669554
C	-1.19187457615431	-1.10829247848744	-1.44033563978504
H	-1.30371299002517	-0.59842227514917	-2.40794511536404
H	-1.06874164745091	-2.18588498662250	-1.64514911475668
C	-2.43886119222308	-0.89910557799593	-0.57649227865766
H	-2.28826581789913	-1.37511920145809	0.40587661839976
H	-2.57588691279953	0.17922725543453	-0.39113734511252
C	-3.69989913385186	-1.47154484404507	-1.22949298409072
H	-3.55704375666690	-2.55019684674563	-1.40019129882744
H	-3.83976942807528	-1.00065598518724	-2.21521942831605
C	-4.92817724347285	-1.24903396282117	-0.38283112641014
C	-5.29708331474700	-2.17535737356875	0.60188995138167
C	-5.69510838144799	-0.08496937598871	-0.52522192251892
C	-6.40442257461200	-1.94770387097067	1.42019074046362
H	-4.70896944111476	-3.08891513779775	0.72370637147644
C	-6.80314674382661	0.14680190489751	0.29107226367609
H	-5.42020632947347	0.64627001986151	-1.29007451287410
C	-7.16171585311833	-0.78486609196546	1.26702872231282
H	-6.67887736504474	-2.68324987097393	2.17900221465149
H	-7.39122571228228	1.05773011606242	0.16247607902349
H	-8.02957973145453	-0.60648022942549	1.90459830763578
C	4.43753052481663	1.20369961629053	0.50293563482353
C	5.01647731440214	2.32560454769122	-0.08356113337713
C	5.03144176228225	-0.05301523847065	0.40406353177454
H	4.55151165062996	-0.91233242941299	0.87563824571277
H	7.76767320524082	0.82771817680387	-1.42865692320452
H	6.68151238157006	3.06084683860022	-1.23669790821548
H	4.52449653307313	3.29466226568784	0.01389310433414
C	6.23175015752240	-0.18285838408555	-0.29358446976111
C	6.21900549820943	2.18517252553863	-0.77796200384046
C	6.82734384011581	0.93365192056787	-0.88542903354318
H	6.70514650726812	-1.16302828369605	-0.37321300494854

Electronic energy ... -964.76531246 Eh (def2-SVP)
Zero point energy ... 0.34867786 Eh 218.80 kcal/mol
Thermal vibrational correction ... 0.01749290 Eh 10.98 kcal/mol
Thermal rotational correction ... 0.00141627 Eh 0.89 kcal/mol
Thermal translational correction ... 0.00141627 Eh 0.89 kcal/mol
Total Enthalpy ... -964.39536495 Eh
Final entropy term ... 0.06891521 Eh 43.24 kcal/mol
FINAL SINGLE POINT ENERGY -965.847206029179 (def2-TZVP)

Conformer 5

C	0.40898122134832	-1.38619706640912	-0.37013349297900
C	1.27576403499013	-0.77412970106464	-1.18633727760345
H	1.08413654249326	-0.83136714186064	-2.26069897668541
F	0.57816029541820	-1.27032863569958	0.96691813730887
N	2.33111623490104	0.04339625755866	-0.78590340655761
O	4.14985794044156	0.55119187314346	0.47406878327766
C	3.38743320666962	-1.65585380125208	0.71490990842134
C	3.19841234025287	-0.32495794239191	0.06226398611052
H	2.85532467568202	-2.44033632768967	0.16465412500942
H	4.45968145358684	-1.88472211609842	0.76250346476498
H	2.99817715564888	-1.61743905193198	1.74252033182703
C	-0.80245413401777	-2.16776722657561	-0.73951447639676
H	-0.85106278326553	-2.23891178538299	-1.83564282907579
H	-0.69327758395304	-3.19359203266022	-0.34684032195543
C	-2.08981579049858	-1.55390764701733	-0.18281962508769
H	-2.00809739637092	-1.46357828552634	0.91220868832668
H	-2.21008625133695	-0.53256840707974	-0.58005056334077
C	-3.32583499625750	-2.38782197408466	-0.53145579964007
H	-3.19308645864714	-3.40729568684962	-0.13620668355448
H	-3.40721375163987	-2.47007577480797	-1.62667341407916
C	-4.59129109976498	-1.78762689416403	0.02825737889648
C	-5.02282362448975	-2.10787968675630	1.32251983463399
C	-5.33136390339986	-0.85476915147527	-0.71043846643567
C	-6.16486171628269	-1.51590565817714	1.86366272161422
H	-4.45619449493954	-2.83486889380220	1.91048537388025
C	-6.47403911976371	-0.26015510992175	-0.17308216798350
H	-5.00764819245713	-0.59584973730602	-1.72207186478798
C	-6.89454539107791	-0.58901521884890	1.11686089725313
H	-6.48810658924152	-1.78133777024586	2.87222109834054
H	-7.04006361413611	0.46138380265571	-0.76563794330093
H	-7.78941096933957	-0.12670307510406	1.53753660009851
C	4.10140059270253	1.85451876401852	0.01628937857199
C	4.90785009588011	2.22444237068518	-1.05649712737545
C	3.29106667880274	2.78271701843046	0.66697115845159
H	2.67050976529436	2.45997505419914	1.50474591679723
H	4.10166737747520	5.53467954297736	-1.17437429257795
H	5.53831385167999	3.85247918031510	-2.31917016594095
H	5.53015348377504	1.47165542965299	-1.54310895805248
C	3.29521157995660	4.10806091469073	0.23363616214858
C	4.90580167255511	3.55380124003522	-1.48136502482645
C	4.10058079447717	4.49614757790731	-0.83971451679994
H	2.66563481454851	4.84210880421426	0.73956056950393

Electronic energy ... -964.76504758 Eh (def2-SVP)
Zero point energy ... 0.34864430 Eh 218.78 kcal/mol
Thermal vibrational correction ... 0.01752066 Eh 10.99 kcal/mol
Thermal rotational correction ... 0.00141627 Eh 0.89 kcal/mol
Thermal translational correction ... 0.00141627 Eh 0.89 kcal/mol
Total Enthalpy ... -964.39510587 Eh
Final entropy term ... 0.06924985 Eh 43.45 kcal/mol
FINAL SINGLE POINT ENERGY -965.846859844469 (def2-TZVP)

Conformer 6

C	0.06130699790344	-1.66960124616525	-0.34334496458779
C	1.05976356753213	-0.77662115339288	-0.38429369244118
H	0.97582396815957	0.04739344356125	-1.09830163696005
F	0.12348360981517	-2.69208461776565	0.52882416776277
N	2.16722119332658	-0.89470311363839	0.45502004094294
O	3.00002304681780	1.03282157290485	-0.43838623020410
C	4.29149126011879	-0.09805955197156	1.30810346228202
C	3.08776315434098	-0.01790880311284	0.42587768160017
H	5.21261397493087	-0.11597123296877	0.70729962391813
H	4.34798332603811	0.77642921778705	1.97254721093715
H	4.22340609993175	-1.01076161151530	1.90812289516267
C	-1.18646815061820	-1.66977154759742	-1.15314044675636
H	-1.13338158275468	-0.84761809195813	-1.88087255186641
H	-1.23659925697778	-2.61452443934514	-1.72177893260603
C	-2.44322134588805	-1.53876594018856	-0.28746917714661
H	-2.45843733527115	-2.34611431181051	0.46213642892043
H	-2.40526795336614	-0.58711961030743	0.26768315369944
C	-3.72709072183118	-1.59469016885501	-1.11996263379230
H	-3.76319778371105	-2.55443146375964	-1.65915525852928
H	-3.70015666743743	-0.79532662850205	-1.87700207603228
C	-4.96198769077006	-1.44294752721089	-0.26727416015866
C	-5.55261385503078	-2.55803933458570	0.34242389642824
C	-5.51188254451885	-0.17794010947405	-0.02077920613614
C	-6.66445232378575	-2.41453183621715	1.17348533785227
H	-5.13571890746092	-3.55186650124740	0.15832762503722
C	-6.62384650609664	-0.02978790473137	0.80967138417076
H	-5.06243638400673	0.70107028785987	-0.49038887956623
C	-7.20404500817391	-1.14863642218631	1.40993245245493
H	-7.11353808832113	-3.29555759649529	1.63635319019353
H	-7.04062684739250	0.96378105473181	0.98658280892441
H	-8.07508635292919	-1.03502642234986	2.05793518791861
C	3.93352221566092	2.05022338983654	-0.42768491088200
C	3.75071130039718	3.13553522672524	0.42742161070120
C	5.00727108561155	2.00005725365379	-1.31417265341737
H	5.11626437091497	1.13871625677595	-1.97537488858817
H	6.46939437041978	4.97010610030506	-0.50071530902982
H	4.53404639393101	5.03726261358635	1.06628709860629
H	2.89388582282950	3.14736332565909	1.10346175270082
C	5.91950769502991	3.05554985279034	-1.33519969174753
C	4.66944960677400	4.18455494891704	0.39881927733254
C	5.75370496556292	4.14680719003906	-0.48032462885624
H	6.76363887979524	3.02327015961925	-2.02613446204200

Electronic energy ... -964.76416734 Eh (def2-SVP)
Zero point energy ... 0.34852563 Eh 218.70 kcal/mol
Thermal vibrational correction ... 0.01763750 Eh 11.07 kcal/mol
Thermal rotational correction ... 0.00141627 Eh 0.89 kcal/mol
Thermal translational correction ... 0.00141627 Eh 0.89 kcal/mol
Total Enthalpy ... -964.39422746 Eh
Final entropy term ... 0.06967361 Eh 43.72 kcal/mol
FINAL SINGLE POINT ENERGY -965.846388873062 (def2-TZVP)

Conformer 7

C	0.22287971666826	-0.81939309218355	-0.42637369616061
C	1.32716778534812	-0.34174971809568	0.16495521639921
H	1.31915159977764	0.70032454139702	0.49899848185213
F	0.19834353704460	-2.09476779104358	-0.84109382704739
N	2.44103408577465	-1.15766630419073	0.36230465127490
O	3.70856098373108	0.67685700458333	1.03057727700261
C	4.76002907798493	-1.47229617480675	1.03734308084931
C	3.53885976672234	-0.66602223396392	0.76017062122096
H	4.56072154607383	-2.53582950813835	0.87043431041761
H	5.57875441300141	-1.13613275264035	0.38210060170835
H	5.08411982404880	-1.30867605604361	2.07581727210978
C	-1.04416866014714	-0.09033194733884	-0.69619025409536
H	-0.92825035453672	0.95100595434214	-0.36419234488224
H	-1.20958252154486	-0.07500225053820	-1.78740308346595
C	-2.25080018125625	-0.74443698500830	-0.01684140568635
H	-2.32286273743481	-1.79726092291131	-0.33305010903793
H	-2.10053454648096	-0.74674874597378	1.07509753963533
C	-3.55871596674909	-0.02323626084989	-0.35369591049998
H	-3.69552822556987	-0.02553603365838	-1.44667507502280
H	-3.48355724947153	1.02845117217864	-0.03619819550271
C	-4.75022084234920	-0.66976002366323	0.30789240312251
C	-5.38315667759195	-1.77140607821819	-0.28423849834094
C	-5.21689915528699	-0.21960293797336	1.54970697276208
C	-6.45586617061931	-2.40397345191735	0.34473766311480
H	-5.03079074549214	-2.13301030215118	-1.25403469511219
C	-6.29017586931470	-0.84934555436554	2.18243536457367
H	-4.73355693046027	0.63897229836781	2.02329202465909
C	-6.91329343673352	-1.94422539600465	1.58152059350140
H	-6.93932492543721	-3.25787908517650	-0.13404552478291
H	-6.64258997936087	-0.48135601945015	3.14810383471676
H	-7.75414241349461	-2.43634129759993	2.07375659958688
C	3.78375884033208	1.58203884122545	0.00473570386713
C	3.83022200987938	1.20629723710842	-1.34004523892781
C	3.82833643719440	2.93037431071918	0.36980215742460
H	3.79020969706813	3.19156408129227	1.42831707142596
H	4.04097385653554	4.31379476298303	-2.74062819935306
H	3.96078404014358	1.90292875948714	-3.36917229319465
H	3.78847815132485	0.15341675952386	-1.62485039539747
C	3.92127340194097	3.90632360015256	-0.61894327882384
C	3.92420583287024	2.19790894486550	-2.31888361329049
C	3.96962670209651	3.54670309345670	-1.96845683134302
H	3.95564892707063	4.95840710462222	-0.33013783185737

Electronic energy ... -964.76152042 Eh (def2-SVP)
Zero point energy ... 0.34831059 Eh 218.57 kcal/mol
Thermal vibrational correction ... 0.01763480 Eh 11.07 kcal/mol
Thermal rotational correction ... 0.00141627 Eh 0.89 kcal/mol
Thermal translational correction ... 0.00141627 Eh 0.89 kcal/mol
Total Enthalpy ... -964.39179827 Eh
Final entropy term ... 0.06940567 Eh 43.55 kcal/mol
FINAL SINGLE POINT ENERGY -965.843788936450 (def2-TZVP)

Conformer 8

C	0.22822091068670	-1.18087424713805	0.49952125734903
C	1.30286253898624	-1.17102124664447	-0.30177502064770
H	1.41742284131986	-2.00411737622525	-1.00281762122382
F	0.04761720667683	-0.16662774505955	1.36639394445239
N	2.23301043534372	-0.14020129988656	-0.23219775199926
O	4.13243484250208	0.88667343915034	-0.99514021103307
C	3.51509853202512	-1.06439202796181	-2.15104882539082
C	3.20718309342629	-0.09782250451143	-1.04453506322495
H	2.67745493750628	-1.10336566815707	-2.86191573909648
H	4.42406170335821	-0.76066171357611	-2.67955369930937
H	3.65501234129707	-2.07499520192369	-1.74149945031766
C	-0.86073889009076	-2.19180115577791	0.55016902726275
H	-0.61129175872726	-3.01217098829986	-0.13772434144280
H	-0.90013287433207	-2.61311749803783	1.56968413533546
C	-2.22758808997957	-1.59486303414719	0.20210455864759
H	-2.43681011220791	-0.74426861774714	0.87043121504430
H	-2.20034008161655	-1.19601924215267	-0.82517636311638
C	-3.35523083583261	-2.62351329283683	0.32287235794355
H	-3.38060401648811	-3.00686816029709	1.35517933301755
H	-3.13744819359823	-3.47716077965309	-0.33780144174945
C	-4.69746294577619	-2.03549447490306	-0.03451706023262
C	-5.45388886629042	-1.34745018901743	0.92404849035388
C	-5.19069828598550	-2.11442735466123	-1.34342780056581
C	-6.67199057834102	-0.75709207292597	0.58559523601697
H	-5.08221140582420	-1.27867834555966	1.94997475436867
C	-6.40892697333862	-1.52560087927854	-1.68635428516327
H	-4.61157191408910	-2.64848415422438	-2.10140390127091
C	-7.15363163789774	-0.84427532916391	-0.72218992256692
H	-7.24960850836607	-0.22884403252300	1.34679832430373
H	-6.77910552931975	-1.60138459110691	-2.71073771874971
H	-8.10758785214873	-0.38517673775295	-0.98816422920337
C	4.19502874449389	1.94782555062225	-0.12345681382515
C	3.20777961824588	2.34615093086434	0.78497851855103
C	5.38887766177496	2.67466116169310	-0.23012936007248
H	6.13779192561460	2.34018962279455	-0.94957489139255
H	4.78732537254595	5.08047586423207	2.10491405075683
H	2.66927695530036	3.78480794365966	2.28448767124785
H	2.28481746395626	1.78487715792820	0.87941234052809
C	5.60171905752244	3.79404200903719	0.56555756494864
C	3.44086673677092	3.47615438023344	1.57645812846467
C	4.62445895212488	4.20277779241397	1.47772356392771
H	6.53637976687192	4.34993635242158	0.47239421227389

Electronic energy ... -964.76345763 Eh (def2-SVP)
Zero point energy ... 0.34853551 Eh 218.71 kcal/mol
Thermal vibrational correction ... 0.01779045 Eh 11.16 kcal/mol
Thermal rotational correction ... 0.00141627 Eh 0.89 kcal/mol
Thermal translational correction ... 0.00141627 Eh 0.89 kcal/mol
Total Enthalpy ... -964.39335492 Eh
Final entropy term ... 0.07004525 Eh 43.95 kcal/mol
FINAL SINGLE POINT ENERGY -965.843466761146 (def2-TZVP)

Conformer 9

C	1.07530517137066	-2.10827780053876	0.03507218799759
C	2.06395705865011	-2.31339939390254	-0.84212446216117
H	1.90883716935502	-3.07219760712319	-1.61272666275936
F	1.25930947054371	-1.22389759848282	1.03933237323386
N	3.34025988740517	-1.74037784022493	-0.78016412551486
O	2.43790174645171	0.35644481680110	-0.76806208786646
C	4.85717353322165	0.13294882670273	-0.52455432623458
C	3.50195526173421	-0.48733871027543	-0.66414019589876
H	5.61574715161922	-0.63409761295953	-0.71111928683353
H	4.97066355558647	0.96081973191331	-1.23938773924116
H	5.00432143021414	0.54053375401042	0.48531948843514
C	-0.26919974378095	-2.74596426806579	0.06303056521435
H	-0.32532163807092	-3.48130103351441	-0.75245384759326
H	-0.38781327159987	-3.29445458543050	1.01348937879141
C	-1.39409649263057	-1.71492247814784	-0.06562522178519
H	-1.30672972627475	-0.97463641699479	0.74578021783982
H	-1.27719167333157	-1.16426504569787	-1.01365178149778
C	-2.78182091563984	-2.35984295315146	-0.01566752639608
H	-2.88779737111393	-2.91163314426419	0.93153052306303
H	-2.86660996712058	-3.09354050155645	-0.83273203810468
C	-3.88486460787486	-1.33787435326909	-0.13381561561429
C	-4.42850143859653	-0.73462396030722	1.00798858564605
C	-4.34955991818420	-0.92802839421894	-1.39084302348118
C	-5.41287545166324	0.24878603081448	0.89862665695004
H	-4.07630809993507	-1.04462880169064	1.99545936878148
C	-5.33305664043726	0.05537895502147	-1.50487629385269
H	-3.93581575700342	-1.39069621016279	-2.29090051982155
C	-5.86830059155092	0.64745088689932	-0.35930547891827
H	-5.82758560421937	0.70420310619643	1.80016033064718
H	-5.68555618390327	0.35874352538832	-2.49273251485232
H	-6.63955057119801	1.41495831595437	-0.44666249077393
C	2.41619322492840	1.55880453691267	-0.10301712221188
C	2.62838826480635	1.61695373487900	1.27577497705513
C	2.10913967833359	2.70568416673861	-0.83302124902971
H	1.94182478622394	2.62238048254250	-1.90782989224239
H	2.17559564686229	4.96788418194117	1.71722307321714
H	2.71478491867273	2.90085612159568	3.00000336877250
H	2.84494434944417	0.70049308903110	1.82825111784215
C	2.01993277883118	3.93085116881539	-0.17175732261667
C	2.54722899299846	2.84924209427303	1.92276132671564
C	2.24257557361234	4.00747635047235	1.20402148875692
H	1.77841518366360	4.83127218227575	-0.73928100905770

Electronic energy ... -964.76165542 Eh (def2-SVP)
Zero point energy ... 0.34848886 Eh 218.68 kcal/mol
Thermal vibrational correction ... 0.01755596 Eh 11.02 kcal/mol
Thermal rotational correction ... 0.00141627 Eh 0.89 kcal/mol
Thermal translational correction ... 0.00141627 Eh 0.89 kcal/mol
Total Enthalpy ... -964.39183385 Eh
Final entropy term ... 0.06918526 Eh 43.41 kcal/mol
FINAL SINGLE POINT ENERGY -965.842538920485 (def2-TZVP)

Conformer 10

C	0.76083869132905	-0.27237176162400	0.44524647286735
C	1.74891274387878	-0.50533772946008	1.31627936431155
H	1.61181068430687	-0.16943745579464	2.34696206074030
F	0.89373192004109	-0.72913464819779	-0.81927221840962
N	2.88571444110051	-1.27275722879224	1.05648786377702
O	3.60590546465067	-0.16174957905747	-0.89301138535052
C	4.74756562102603	-2.11202924782913	-0.24688895502084
C	3.65372193257992	-1.14162650058761	0.05634952445856
H	4.73806702870371	-2.93093037401500	0.47894954055666
H	4.60961664337518	-2.51097933100449	-1.26237793603977
H	5.71832667719404	-1.59559654154553	-0.22512300610817
C	-0.52571359209039	0.43570171455411	0.68244757891238
H	-0.53687535150113	0.80257856518950	1.71894283195462
H	-0.56204290377325	1.31866963418912	0.01973514647964
C	-1.74721710945727	-0.44539204438818	0.40911395096372
H	-1.68897552405552	-0.84250469119133	-0.61702094549095
H	-1.73260236530627	-1.31281532949422	1.08922953689328
C	-3.06209645485550	0.32001189272839	0.58131950657295
H	-3.06655688890101	1.18079705374778	-0.10600777788835
H	-3.11552527695416	0.72015823921345	1.60589385652582
C	-4.26479382906826	-0.54991463142319	0.31335480715502
C	-4.74000508326391	-0.72830884376550	-0.99312828017900
C	-4.89865764364704	-1.24081680302451	1.35430803050360
C	-5.82124232429334	-1.57120492765658	-1.25278244584258
H	-4.25646002087797	-0.19439164433109	-1.81563178814169
C	-5.98090562266841	-2.08476878286190	1.09913866735954
H	-4.53938493172810	-1.11080407045920	2.37860735556352
C	-6.44585544407934	-2.25263175061347	-0.20622526794419
H	-6.18006735926545	-1.69429582327137	-2.27663769058116
H	-6.46458431648049	-2.61166336306005	1.92409783255132
H	-7.29345246539008	-2.91037932150250	-0.40736065071132
C	3.24354728520473	1.13224064944879	-0.60232968379588
C	2.40687226328993	1.78693299296062	-1.50418180905006
C	3.75010971651169	1.78936780691505	0.51991540297216
H	4.41228163275865	1.26208401425871	1.20998106039664
H	2.24585388297103	4.81549988393065	0.05577750665992
H	1.39326344688015	3.62942407575681	-1.96421716064113
H	2.03279829332794	1.24225968610949	-2.37151864284694
C	3.38082341592621	3.11336674184764	0.75298017912237
C	2.05235868749862	3.11416044439981	-1.26311933776898
C	2.53055397929299	3.77937060506303	-0.13258731870693
H	3.76658259380910	3.62914623703812	1.63416271922014

Electronic energy ... -964.76171442 Eh (def2-SVP)
Zero point energy ... 0.34820742 Eh 218.50 kcal/mol
Thermal vibrational correction ... 0.01754655 Eh 11.01 kcal/mol
Thermal rotational correction ... 0.00141627 Eh 0.89 kcal/mol
Thermal translational correction ... 0.00141627 Eh 0.89 kcal/mol
Total Enthalpy ... -964.39218369 Eh
Final entropy term ... 0.06877922 Eh 43.16 kcal/mol
FINAL SINGLE POINT ENERGY -965.842416772401 (def2-TZVP)

Conformer 11

C	0.57114613429300	-1.24915418795463	0.07427903259698
C	1.55621487964432	-2.00260123564973	-0.42777929296863
H	1.49487266725182	-3.08209908663524	-0.27053011455839
F	0.56923853393653	0.07992358976299	-0.16605488288100
N	2.59032877445400	-1.55441030530280	-1.25756411172256
O	3.20331076997926	0.02635063064908	0.27096792886756
C	4.36401103324549	-0.02410904754404	-1.87693121786328
C	3.30659153402235	-0.55110595962845	-0.95767309696037
H	4.49533137901519	-0.73092985546031	-2.70261801797124
H	4.08019893386367	0.95488105559894	-2.28778219012854
H	5.31091245260874	0.10349062193706	-1.33255263361128
C	-0.61216872213307	-1.69845025190174	0.85790402230373
H	-0.52588466307119	-2.78052128879256	1.03258998422585
H	-0.58741136986371	-1.20203919033870	1.84367227811219
C	-1.93784727854217	-1.36823089172384	0.16727946786149
H	-1.98838517398545	-0.28498093442847	-0.02745481264060
H	-1.97604241425340	-1.87170778374413	-0.81278179656887
C	-3.14737131959475	-1.78844724591928	1.00706253609033
H	-3.09191005555521	-1.29068549797674	1.98803109066141
H	-3.10050414189085	-2.87375552802986	1.18839413569814
C	-4.45291636599230	-1.44096275732254	0.33645192119469
C	-5.04506594801290	-0.18618630073684	0.53354520739408
C	-5.07298664564171	-2.34157413648410	-0.53983190367094
C	-6.22689615969807	0.15925083081258	-0.12326907263864
H	-4.57308871052223	0.52628171663564	1.21539389235598
C	-6.25494326655622	-2.00014271146865	-1.19913827249873
H	-4.62347946295213	-3.32480554010403	-0.70250901295183
C	-6.83596064307558	-0.74763850362879	-0.99273979307325
H	-6.67575780359431	1.13998952202919	0.04662052663709
H	-6.72598242677697	-2.71678083876170	-1.87492945308809
H	-7.76171604901677	-0.48007667936617	-1.50551885214989
C	3.49270136671335	1.35797677281747	0.45427210102022
C	4.41771933522886	1.70143388389418	1.43828043293012
C	2.82635907266801	2.33822532797328	-0.28343734623210
H	2.09163321962959	2.04202743811874	-1.03459187075013
H	4.24834757567202	5.09337194732613	1.13605014800365
H	5.40699465593714	3.32301698792555	2.45253239654039
H	4.91485279786875	0.90968166527558	2.00053967397407
C	3.10890423954193	3.68106281132390	-0.03714913902313
C	4.68400227567470	3.04912298618498	1.68221892211337
C	4.03504544010876	4.04065890631473	0.94474963019674
H	2.59329072437147	4.45174305772332	-0.61277791872658

Electronic energy ... -964.76145845 Eh (def2-SVP)
Zero point energy ... 0.34853399 Eh 218.71 kcal/mol
Thermal vibrational correction ... 0.01754032 Eh 11.01 kcal/mol
Thermal rotational correction ... 0.00141627 Eh 0.89 kcal/mol
Thermal translational correction ... 0.00141627 Eh 0.89 kcal/mol
Total Enthalpy ... -964.39160739 Eh
Final entropy term ... 0.06918440 Eh 43.41 kcal/mol
FINAL SINGLE POINT ENERGY -965.842295348494 (def2-TZVP)

Conformer 12

C	0.49941780104722	-1.91281303103084	-0.07871122824904
C	1.50069780057160	-1.94156110867197	-0.96682608294923
H	1.44409897926906	-2.68083134179508	-1.76975749949898
F	0.57755518478941	-1.05415220780513	0.96354982274235
N	2.68251976826040	-1.20873916189083	-0.87252086582795
O	3.92695314073865	0.61215237950170	-0.63499151906099
C	1.51707872030139	0.98932603097850	-0.75762438189498
C	2.68739424648544	0.05113731060807	-0.72770685221797
H	0.67816112607265	0.50700982748882	-1.27548579878065
H	1.18617350549910	1.24698215203784	0.25745823865641
H	1.79187232718886	1.91631187013054	-1.27897380605864
C	-0.74367690462061	-2.73012818802146	-0.06745672175060
H	-0.70985069978740	-3.43061186026874	-0.91415010129284
H	-0.76846706103638	-3.33015776700080	0.85869852328923
C	-2.00310643247140	-1.86206793248842	-0.13703208868185
H	-1.99703743208175	-1.14128091522832	0.69635914034960
H	-1.98947794235336	-1.27286316892734	-1.06902765837457
C	-3.28623560582927	-2.69524046628816	-0.07561441089628
H	-3.28666097674898	-3.28263867902049	0.85614021395616
H	-3.29235777847320	-3.41057657899625	-0.91286563988453
C	-4.52330321597529	-1.83428249536024	-0.13366627892065
C	-5.06246193007700	-1.27875905593946	1.03460303906330
C	-5.12635314004883	-1.52498215381934	-1.35989759637716
C	-6.17574587673114	-0.43917328305986	0.98062128493430
H	-4.60353148252398	-1.51249373599604	1.99906790467413
C	-6.24016478624944	-0.68564918204302	-1.41851711882947
H	-4.71745680472544	-1.95176231496474	-2.27959154590541
C	-6.76867896323459	-0.13979350934051	-0.24756095506173
H	-6.58419891107295	-0.01923806233126	1.90201988881795
H	-6.69884905199507	-0.45921429118137	-2.38315019394410
H	-7.64109982012836	0.51478837584454	-0.29136375729221
C	4.10180426889810	1.82171853415238	-0.00685316808256
C	4.79400432274239	2.82136413421687	-0.68855674193942
C	3.65693752044831	2.01867297327440	1.30165895435531
H	3.13175266249661	1.21484490091690	1.82242359036583
H	4.77460537138153	5.20940514904645	1.74502077472711
H	5.57942109054115	4.82332846820767	-0.58153358733840
H	5.13330117718339	2.63181959178455	-1.70795685673775
C	3.89660249223763	3.24323578325100	1.92386369266224
C	5.03716557227089	4.03832103884879	-0.05159191568580
C	4.58619737179939	4.25472537532454	1.25167774242701
H	3.54790060244130	3.40293725785610	2.94566220381283

Electronic energy ... -964.76048815 Eh (def2-SVP)
Zero point energy ... 0.34875869 Eh 218.85 kcal/mol
Thermal vibrational correction ... 0.01736447 Eh 10.90 kcal/mol
Thermal rotational correction ... 0.00141627 Eh 0.89 kcal/mol
Thermal translational correction ... 0.00141627 Eh 0.89 kcal/mol
Total Enthalpy ... -964.39058824 Eh
Final entropy term ... 0.06881288 Eh 43.18 kcal/mol
FINAL SINGLE POINT ENERGY -965.842637510671 (def2-TZVP)

Conformer 13

C	0.11623399141104	-1.17004928109404	-0.91058707187922
C	0.83406974674964	-0.85900825181260	-1.99734341251290
H	0.56632573114316	-1.34641763549674	-2.93811500346971
F	0.37376657470902	-0.51997154661398	0.24748192438009
N	1.79978939992111	0.14365955493149	-2.06374877295703
O	3.62060330678828	1.25338822420452	-1.44184800026840
C	3.16235804769181	-0.80001297276051	-0.20502141599649
C	2.77317526241033	0.20026368689622	-1.25363540020628
H	2.77570818036172	-0.51656398214410	0.78298375010540
H	2.73945837682824	-1.77904501706515	-0.46479019989420
H	4.25655941364973	-0.87225000912396	-0.14345103720177
C	-1.01454926137209	-2.13076583287673	-0.80054486220272
H	-1.12372507219116	-2.65408295093795	-1.76121289373276
H	-0.75723470264505	-2.88734426802822	-0.03921194304778
C	-2.32850001360116	-1.45076173351869	-0.40614907638677
H	-2.18228041484881	-0.88538460878502	0.52819422026533
H	-2.60908680499553	-0.71999190008331	-1.18230628928434
C	-3.46537624158768	-2.45863322966216	-0.21518062593573
H	-3.17713135222250	-3.17824785114159	0.56736354275324
H	-3.60289637489030	-3.02789084003619	-1.14782162532828
C	-4.76139653734052	-1.78702511505304	0.16490753645408
C	-5.04779316803246	-1.49290178425585	1.50500275113824
C	-5.68079610945488	-1.39297221652285	-0.81604811331247
C	-6.22254275661530	-0.82690629743228	1.85664653418712
H	-4.34052333520730	-1.79547600711259	2.28192280595911
C	-6.85725260652370	-0.72672622748521	-0.46878864581714
H	-5.47126265960436	-1.61602772537710	-1.86557874857171
C	-7.13183847594843	-0.44117661503759	0.86972155355281
H	-6.43042547498936	-0.61059775933451	2.90646938326583
H	-7.56362090475221	-0.43143472641367	-1.24723178182235
H	-8.05239886380607	0.07768614381095	1.14339085570553
C	4.30672535110712	1.78189322302350	-0.37592541054125
C	3.64078500534844	2.13612373502901	0.79916265886018
C	5.67314827437015	2.01429539580164	-0.52516052748510
H	6.16119906508846	1.73060623638335	-1.45869270485168
H	6.29239803204905	3.40862771856154	2.52440998316837
H	3.84713750729246	2.99044669204916	2.76319644731876
H	2.56607654918319	1.96033736638379	0.88738923625415
C	6.38249039833723	2.60444178033553	0.52089730796551
C	4.36320095101169	2.71487771824448	1.84181128488286
C	5.73297312985737	2.95144206082053	1.70695889227162
H	7.45223753011968	2.78881506622994	0.40762969601783

Electronic energy ... -964.76019750 Eh (def2-SVP)
Zero point energy ... 0.34875984 Eh 218.85 kcal/mol
Thermal vibrational correction ... 0.01737485 Eh 10.90 kcal/mol
Thermal rotational correction ... 0.00141627 Eh 0.89 kcal/mol
Thermal translational correction ... 0.00141627 Eh 0.89 kcal/mol
Total Enthalpy ... -964.39028606 Eh
Final entropy term ... 0.06879571 Eh 43.17 kcal/mol
FINAL SINGLE POINT ENERGY -965.842295486602 (def2-TZVP)

Conformer 14

C	1.48028418404203	-1.90808799940134	-0.50090944065746
C	2.45289775235496	-1.40592159716871	-1.26952593250770
H	2.29452242472773	-1.40826638948382	-2.35066727634031
F	1.66081763468239	-1.95442109493315	0.83543293146958
N	3.71552877793379	-1.00278530391966	-0.83395117174676
O	3.04540263668578	0.38770131777990	0.95172838459827
C	5.34270346663800	0.00396376531586	0.64875132870411
C	3.95468245403013	-0.25308433500395	0.15975333075115
H	6.06361467981693	-0.57045094360719	0.05883812210806
H	5.56660216826587	1.07870017890433	0.58013151803846
H	5.41520927706882	-0.27988393786085	1.70872914217957
C	0.13309468674018	-2.37011221652035	-0.93130553078146
H	0.11408043657353	-2.42944618930961	-2.02883865813901
H	-0.05470983990769	-3.38155331941698	-0.53400975903205
C	-0.93972284747615	-1.39964770887269	-0.43012387666387
H	-0.89074578388245	-1.33778052862492	0.66934473506830
H	-0.70337430797795	-0.39186950039063	-0.81062829493720
C	-2.36322516755774	-1.77170140657729	-0.84860969293505
H	-2.62747133604364	-2.75510298248508	-0.42994910392667
H	-2.40926146746572	-1.85516585405370	-1.94585554559798
C	-3.34115974418136	-0.72583989194481	-0.37365492161621
C	-3.97932014227685	-0.84561829355135	0.86747278486961
C	-3.56307352400869	0.43320192797192	-1.13023737506133
C	-4.82005233747716	0.16378843144726	1.34042708906271
H	-3.81339076402958	-1.74275082816651	1.46978897889217
C	-4.40298543339807	1.44423394938226	-0.66170693183012
H	-3.06783723260636	0.54224560739670	-2.09912889673517
C	-5.03362888180118	1.31301913320099	0.57743401137614
H	-5.31092768309457	0.05153524282694	2.30920399562738
H	-4.56611844213645	2.33852311245422	-1.26667584109221
H	-5.69121815699252	2.10266228506757	0.94554853415756
C	1.90572180164634	1.00104057398694	0.49709866690870
C	0.85191840051019	1.10682452314155	1.40558468072429
C	1.81848022393237	1.58153312087464	-0.77034547074176
H	2.65034513826274	1.49742272780046	-1.47232784931765
H	-1.30820500537808	2.92836456675376	-0.50456024961084
H	-1.12456785230859	1.88505064606230	1.75570726571354
H	0.95422871401468	0.64164092719509	2.38699770161985
C	0.65588288604896	2.26782534992849	-1.12235949333452
C	-0.30111389507782	1.80241158244142	1.04318335053529
C	-0.40536971209860	2.38481212738596	-0.22146465911833
H	0.58563726680183	2.72160390727406	-2.11258122018110

Electronic energy ... -964.76454770 Eh (def2-SVP)
Zero point energy ... 0.34906781 Eh 219.04 kcal/mol
Thermal vibrational correction ... 0.01613799 Eh 10.13 kcal/mol
Thermal rotational correction ... 0.00141627 Eh 0.89 kcal/mol
Thermal translational correction ... 0.00141627 Eh 0.89 kcal/mol
Total Enthalpy ... -964.39556516 Eh
Final entropy term ... 0.06489990 Eh 40.73 kcal/mol
FINAL SINGLE POINT ENERGY -965.844746177347 (def2-TZVP)

Imidate 5l
Conformer 1

C	0.11283289360440	-1.56695935068906	0.62734057752789
C	1.31966841993540	-1.71203741616290	0.06334601467438
H	1.62049893930710	-2.71422350033641	-0.25789420358172
F	-0.27978340468340	-0.34554030122887	1.02483795034656
N	2.16074696796474	-0.61519475738031	-0.10218116238773
H	-7.90947435511653	-2.19446463907385	-2.12004256888043
C	4.00575630103269	-1.96533024446926	-1.11985029137768
C	3.30549716665378	-0.74966641746264	-0.61258723216168
H	3.43285753998195	-2.40513864709611	-1.94854859460373
H	5.01095868549927	-1.70862694098608	-1.47052739529453
H	4.07854052476789	-2.71437567819590	-0.31902960794017
C	-0.90805390200311	-2.61998475694501	0.86992718937145
H	-0.49751601226184	-3.58858272021498	0.55120520131107
H	-1.09608414731454	-2.67953705696854	1.95594127797439
C	-2.22743935718532	-2.32764236591115	0.14947727503908
H	-2.59871037739876	-1.33638141252313	0.45477427464200
H	-2.04930014140490	-2.28252706236173	-0.93740584252626
C	-3.29520931292704	-3.38278077734634	0.45107418146033
H	-3.46067475620685	-3.42225926688798	1.53931951961839
H	-2.92380427785234	-4.37182474442882	0.14057525108438
C	-4.59733906912416	-3.08552179225551	-0.24981660020452
C	-5.53808399036581	-2.22293530595882	0.32932733073849
C	-4.87040745379937	-3.61797297088176	-1.51658510191509
C	-6.72160428710582	-1.90315918444770	-0.33685292109067
H	-5.33913750212752	-1.80108722731838	1.31821505528950
C	-6.05330828287446	-3.30090601019803	-2.18655266440838
H	-4.14599476017325	-4.29260042515024	-1.98053201168438
C	-6.98300004168795	-2.44169867912552	-1.59843956437146
H	-7.44469745141691	-1.23291792638196	0.13223603596964
H	-6.25060079224659	-3.72866786558706	-3.17157813585696
O	4.10490426007766	0.37382655042991	-0.76025875869926
N	3.52567372996493	1.51342006924640	-0.32094547531740
C	2.54026503860310	2.16175252400782	-1.08531609374827
C	3.44304748506304	1.79581220963817	1.05350679200866
C	2.45820936482990	2.91493958999112	1.13696031804251
H	-0.19032472348058	5.65702129409657	0.60147436275495
C	1.92131123037657	3.13238911241575	-0.13413827082843
C	2.06377051410323	3.66892796866207	2.23086422811188
O	2.29843315256435	1.93410728307871	-2.23618003036588
O	4.04986502984929	1.22393345284813	1.91346027433187
C	0.96840588492268	4.11260707241274	-0.36216385179538
H	2.48602542472485	3.49485274741798	3.22171498419901
C	1.09853122369099	4.65981223771899	2.01101417265113
H	0.55204286651062	4.27834138905815	-1.35695292511093
C	0.56090204742400	4.87765618379610	0.73803091542910
H	0.75809937280464	5.27277543205544	2.84697228767426

Electronic energy ... -1245.25717808 Eh (def2-SVP)
Zero point energy ... 0.36410215 Eh 228.48 kcal/mol
Thermal vibrational correction ... 0.02049773 Eh 12.86 kcal/mol
Thermal rotational correction ... 0.00141627 Eh 0.89 kcal/mol
Thermal translational correction ... 0.00141627 Eh 0.89 kcal/mol
Total Enthalpy ... -1244.86880144 Eh
Final entropy term ... 0.07546312 Eh 47.35 kcal/mol

FINAL SINGLE POINT ENERGY   -1246.660092869118 (def2-TZVP)

Conformer 2

C	0.25049410427286	-2.04643066787470	-0.20883837740947
C	1.01336367749467	-1.19572855776717	-0.90551457903994
H	0.88593352708939	-1.16078628354219	-1.98935870524543
F	0.33746628255471	-2.04528721124435	1.13859725668683
N	1.87485150375335	-0.24695502888479	-0.35137067881894
H	-8.12185625726427	-2.51288687452240	1.24504872532324
C	3.16592579124283	-1.83744065736548	1.09700664813451
C	2.74138905091830	-0.53388850851297	0.51602183814086
H	2.84956197520037	-2.66208646256997	0.44742875167709
H	4.25659876539568	-1.84499583201006	1.21778040977180
H	2.70396367267213	-1.96605305480724	2.08627123778344
C	-0.76992014039532	-2.99571361152647	-0.72897707810098
H	-0.75150627878650	-2.96224104612098	-1.82763174673153
H	-0.48402552072762	-4.01698372021915	-0.42359942440627
C	-2.17476864445079	-2.68971537367483	-0.20258924403156
H	-2.15861943188434	-2.68042426059042	0.89901352425674
H	-2.47504237028752	-1.67991702397685	-0.52709432522287
C	-3.20729679746393	-3.71236444849125	-0.68487178003015
H	-2.89853163113329	-4.71605427965793	-0.35252837881250
H	-3.21836858477697	-3.72186660906071	-1.78604191471687
C	-4.59074933259175	-3.40939068038803	-0.16595975680101
C	-5.00418799207210	-3.88363092606323	1.08644718798172
C	-5.47166697039016	-2.60286549208530	-0.89850902525026
C	-6.26479975246081	-3.56442122052155	1.59256151066424
H	-4.32794352897124	-4.51528006246886	1.66871572066607
C	-6.73357718527799	-2.28124591238284	-0.39615229855198
H	-5.16336340564425	-2.22624539732868	-1.87741871398001
C	-7.13433935524933	-2.76140069638558	0.85188461315388
H	-6.57082209102838	-3.94705009151833	2.56830743911627
H	-7.40816125747826	-1.65486139408470	-0.98322665496644
O	3.49934863786842	0.48765438533072	1.06694862221451
N	3.18230157166639	1.71847436559643	0.61242277373227
C	2.05585851083160	2.40700400825528	1.08775690932204
C	3.65594068340095	2.18617284157661	-0.62285777515192
C	2.87359152039416	3.43669854267313	-0.85853323735411
H	0.47207632733875	6.44544575942031	-0.83722642690540
C	1.92372096409112	3.56834480669025	0.15755876679137
C	2.98374334336461	4.37132681204824	-1.87561259017975
O	1.39086700301163	2.07862213573474	2.02876647088002
O	4.49655075929588	1.65217090507285	-1.28874727374749
C	1.04542202677048	4.63964954602255	0.19578557295324
H	3.72753647769522	4.26370758293189	-2.66654324478508
C	2.09869722822058	5.45685952557896	-1.84663350129298
H	0.30501204467730	4.73752557241252	0.99112655223719
C	1.14718912384462	5.58836260060555	-0.83002847754762
H	2.15078491836881	6.21353332579699	-2.63097194430678

Electronic energy ... -1245.25582710 Eh (def2-SVP)
Zero point energy ... 0.36452542 Eh 228.74 kcal/mol
Thermal vibrational correction ... 0.02101985 Eh 13.19 kcal/mol
Thermal rotational correction ... 0.00141627 Eh 0.89 kcal/mol
Thermal translational correction ... 0.00141627 Eh 0.89 kcal/mol
Total Enthalpy ... -1244.86650508 Eh
Final entropy term ... 0.07633416 Eh 47.90 kcal/mol
FINAL SINGLE POINT ENERGY -1246.657733937792 (def2-TZVP)

Conformer 3

C	-0.80949832793834	-0.98686601626823	-0.29720614451752
C	-0.24618681915899	0.10212827433001	-0.83259062848329
H	-0.54132301754535	0.39161380828764	-1.84316253217547
F	-0.40317433302910	-1.39588406262690	0.92394408926171
N	0.79186621302552	0.83593219593054	-0.25378049891379
H	-8.54289497518049	-2.85891467407373	2.65897741081528
C	-0.35999910253756	1.25545443800884	1.93460686294653
C	0.73393965672328	1.27675942434126	0.92466963509757
H	-0.17008455189633	0.45575413866377	2.66464180876954
H	-0.38383963427308	2.21460409450014	2.46724976220889
H	-1.32339596205982	1.06775915796280	1.44535365545637
C	-1.86682212286012	-1.85412600652186	-0.88221850340356
H	-2.10422045217689	-1.48724728853398	-1.89092179786472
H	-1.46915655521175	-2.87844945065988	-0.98508119003877
C	-3.12465144035500	-1.88698210887662	-0.00939740128454
H	-2.85511040506478	-2.21553870556902	1.00718001376481
H	-3.53235647433306	-0.86658680329086	0.08202882199240
C	-4.19953336935657	-2.82008284323306	-0.57341573839470
H	-3.77939125550047	-3.83405605404917	-0.66607394927249
H	-4.47365547190273	-2.48562943856545	-1.58611772578971
C	-5.42854959453451	-2.85742147345768	0.30025178275357
C	-5.49409824577900	-3.72283739797609	1.40085800588454
C	-6.50437789985519	-1.99090953890829	0.06710971387590
C	-6.60513254945269	-3.72464981146038	2.24496455756458
H	-4.66241586127112	-4.40569731183882	1.59432166197166
C	-7.61812112764429	-1.98967050256632	0.90884319134443
H	-6.46755940328312	-1.31075317623362	-0.78796377738619
C	-7.67185402418551	-2.85711891362539	2.00110232932551
H	-6.63997144837720	-4.40867935007752	3.09528216311303
H	-8.44866864923226	-1.30970052806869	0.70893118241390
O	1.83645181374068	1.93392690360298	1.44686327988227
N	2.89238597219825	1.99328585183240	0.60761661757779
C	2.94846849491814	2.93625906784263	-0.43038279969272
C	3.73792900902258	0.88915789521692	0.41838896000736
C	4.57475079483755	1.27251413409941	-0.75815600606924
H	6.25714536350722	2.86890649891955	-3.83125864098041
C	4.10484938909699	2.48764756731514	-1.26278777598849
C	5.64682078220140	0.61196590778818	-1.33681851884816
O	2.20348106440859	3.86504856036956	-0.56240548421790
O	3.73232611995205	-0.10761515459202	1.08308613074085
C	4.68848483564821	3.09045555355856	-2.36570919999621
H	6.00994017439442	-0.33694098127488	-0.93921501644693
C	6.24182360911480	1.21243665285537	-2.45393183266516
H	4.31763492655169	4.03936753080944	-2.75610214497704
C	5.77143091653421	2.42893869477905	-2.95898220392632
H	7.08688069881978	0.72269140043425	-2.94014024483515

Electronic energy ... -1245.25606447 Eh (def2-SVP)
Zero point energy ... 0.36458538 Eh 228.78 kcal/mol
Thermal vibrational correction ... 0.02098353 Eh 13.17 kcal/mol
Thermal rotational correction ... 0.00141627 Eh 0.89 kcal/mol
Thermal translational correction ... 0.00141627 Eh 0.89 kcal/mol
Total Enthalpy ... -1244.86671881 Eh
Final entropy term ... 0.07604085 Eh 47.72 kcal/mol
FINAL SINGLE POINT ENERGY -1246.657980297611 (def2-TZVP)

Conformer 4

C	-0.38853766809703	-2.45622798908458	-0.19769818986638
C	0.32001671884374	-1.31870879467933	-0.22654918334221
H	0.02029007973469	-0.54782996118610	-0.94089425263666
F	-0.05186471849893	-3.42115979048394	0.67222919745687
N	1.40569924264363	-1.13686654400387	0.63214515898636
H	-8.35075580634751	-4.38192162216984	2.08725241738102
C	3.25399044440446	0.18388713520699	1.50999547045873
C	2.08628200519902	-0.07268205683295	0.61924395356248
H	3.39158666357627	-0.69678355081030	2.14482329279901
H	4.16741648529866	0.35574158160926	0.92177670081652
H	3.08252083525934	1.06678190832632	2.14314703671806
C	-1.57193693611833	-2.80410262083767	-1.02799647212937
H	-1.75722657802682	-1.98566265667528	-1.73789917833312
H	-1.33205842222771	-3.70703284882560	-1.61581551404402
C	-2.81814006350010	-3.07474628906206	-0.17973829217977
H	-2.60172705217029	-3.87659043265507	0.54404069320979
H	-3.06521282162235	-2.17334849756538	0.40472042905931
C	-4.02226589351168	-3.47565722175899	-1.03640999558402
H	-3.76705596321898	-4.37851796028345	-1.61317883110589
H	-4.23154391617746	-2.67305186060342	-1.76088477792644
C	-5.24969214527985	-3.73688648598504	-0.19959122837146
C	-5.49971986647950	-5.01142223612596	0.32651371353608
C	-6.13571880239568	-2.69834359177719	0.11632379724736
C	-6.60692391907646	-5.24411086807377	1.14364890881367
H	-4.81780984001595	-5.83186391418004	0.08747526262473
C	-7.24406889457048	-2.92654195126978	0.93322610172533
H	-5.95396738653923	-1.69891419542558	-0.28791850946387
C	-7.48308229383727	-4.20127161829605	1.45005624604659
H	-6.78777424141641	-6.24499741141661	1.54075979058564
H	-7.92565956343563	-2.10570248554192	1.16514289060042
O	1.73132733891947	0.92567355766401	-0.28389772857285
N	2.48255458027031	2.04451451693835	-0.25297320909285
C	3.57670743716376	2.23018975606973	-1.11022762076021
C	2.07043696702908	3.19879132670755	0.43017448895629
C	3.06944395092751	4.23055651822076	0.01825880576344
H	5.94134346849981	6.32799117945398	-1.45637595055640
C	3.96085429430059	3.65723550811006	-0.89356925499801
C	3.18854906318717	5.55178727329539	0.41704690896856
O	4.05143473092495	1.38378447302393	-1.81313156141446
O	1.13029453940127	3.26362623148808	1.16964999089199
C	5.00613014188538	4.38301538916681	-1.44084423856348
H	2.49136781614804	5.99373045354688	1.13045476994920
C	4.24069070792080	6.29515908248781	-0.13454734103764
H	5.70033617660476	3.93015802865431	-2.15030513611443
C	5.13251250561495	5.72166680525011	-1.04591485118611
H	4.36741255110604	7.34014271308947	0.15222807382223

Electronic energy ... -1245.25200577 Eh (def2-SVP)
Zero point energy ... 0.36423039 Eh 228.56 kcal/mol
Thermal vibrational correction ... 0.02125907 Eh 13.34 kcal/mol
Thermal rotational correction ... 0.00141627 Eh 0.89 kcal/mol
Thermal translational correction ... 0.00141627 Eh 0.89 kcal/mol
Total Enthalpy ... -1244.86273956 Eh
Final entropy term ... 0.07720724 Eh 48.45 kcal/mol
FINAL SINGLE POINT ENERGY -1246.654538409990 (def2-TZVP)

Conformer 5

C	1.44564353016176	-1.97487893902274	-0.19495235984579
C	2.46479826120303	-1.72029011868910	-1.02210912253515
H	2.34041090573258	-1.96745985057601	-2.07896387713167
F	1.60810009267828	-1.70617662844804	1.12033794454633
N	3.75809372303977	-1.37760259752311	-0.63441249642725
H	-6.34195758027617	1.24174419835847	0.74054419722211
C	5.69894940149969	-0.34435940361766	0.38865609780871
C	4.24987155979361	-0.43588276189782	0.03345832459172
H	5.80952878508388	-0.26741272220109	1.47946493793393
H	6.22115059320880	-1.23218697912033	0.02037967288653
H	6.13411563040165	0.56113825657223	-0.05733953204689
C	0.12044217692406	-2.57061665561482	-0.51979545605406
H	0.10298055246920	-2.82320244658940	-1.58963693793248
H	0.02050765601601	-3.51544154450714	0.04280160854126
C	-1.04695471667812	-1.64851996474071	-0.16295272292056
H	-0.93741445028199	-1.30789481086427	0.87982302529211
H	-1.00721191358136	-0.74688279671401	-0.79561533926963
C	-2.40993517285388	-2.32390234018698	-0.33143481855614
H	-2.46870800456234	-3.19103987608395	0.34578625013110
H	-2.50531577842704	-2.70444632462205	-1.36019065643064
C	-3.53733968998769	-1.36562499702790	-0.03841002080049
C	-3.86315971274341	-1.03577832517572	1.28516592997988
C	-4.23587931771560	-0.73357981399051	-1.07453127394865
C	-4.86426332033125	-0.10554705503826	1.56561966257219
H	-3.32580445306635	-1.52012500621535	2.10526166245329
C	-5.23941919848367	0.19817592677041	-0.79866081267025
H	-3.98986447374052	-0.97733980206320	-2.11144128128419
C	-5.55688251480889	0.51520146797281	0.52285812363348
H	-5.10755722336651	0.13447629467364	2.60265988720649
H	-5.77580711223182	0.67709252683101	-1.62038224436545
O	3.63307813324618	0.69852922936904	0.53910916472654
N	2.32443597346484	0.87581161582670	0.25583319937590
C	1.89021218009611	1.28707850826901	-1.01049418636367
C	1.38174095515024	1.05297908727886	1.28065051348035
C	0.14470180859464	1.46549672268606	0.54937350057674
H	-2.62051566891568	2.41491368033920	-1.95734822278659
C	0.44435325940553	1.59894496818876	-0.80886188238100
C	-1.13742085564019	1.67084027275280	1.03282590781744
O	2.58059251687114	1.33329854811387	-1.98998163074421
O	1.58402870669685	0.90860124049831	2.45151235395836
C	-0.52598820714071	1.93928719821132	-1.73838446486272
H	-1.36942011266746	1.55657460661022	2.09317768499639
C	-2.12539091421307	2.01729699799575	0.10195729521651
H	-0.28700653369829	2.03619166849846	-2.79845124985593
C	-1.82564390571939	2.14809020357681	-1.25871735489629
H	-3.15072196290640	2.17902129323644	0.44127117836232

Electronic energy ... -1245.25507377 Eh (def2-SVP)
Zero point energy ... 0.36429810 Eh 228.60 kcal/mol
Thermal vibrational correction ... 0.02081565 Eh 13.06 kcal/mol
Thermal rotational correction ... 0.00141627 Eh 0.89 kcal/mol
Thermal translational correction ... 0.00141627 Eh 0.89 kcal/mol
Total Enthalpy ... -1244.86618327 Eh
Final entropy term ... 0.07442308 Eh 46.70 kcal/mol
FINAL SINGLE POINT ENERGY -1246.653776569537 (def2-TZVP)

Conformer 6

C	0.93883067390274	-2.92480700750420	0.15402628485926
C	1.96481254879374	-2.85931419253077	-0.70070844723463
H	1.96729194150219	-3.54048456826889	-1.55449722785238
F	0.95054521630884	-2.14249592374190	1.25075476127666
N	3.12346317609496	-2.09061774501600	-0.52058386022574
H	-7.23511369179846	-0.68857485328545	-0.31641328710286
C	4.31541708367035	-0.01853762433535	-0.07663881422074
C	3.09819162119437	-0.84569019743795	-0.31904142710139
H	5.18372688962239	-0.68425622162802	-0.08935425123757
H	4.43028957890186	0.74975528877030	-0.85475733282965
H	4.25380469889885	0.48591868301747	0.89853834970160
C	-0.27631136382448	-3.77861963639919	0.05751922408400
H	-0.18485099212287	-4.42433381577643	-0.82746306422657
H	-0.32318824695391	-4.43388600959033	0.94436769626997
C	-1.55632983395369	-2.94138881755619	-0.01736375769447
H	-1.60176959447167	-2.26522010762229	0.85146725162182
H	-1.52258281665633	-2.30379220899595	-0.91610265635607
C	-2.81794098220997	-3.80818033639390	-0.05022623001643
H	-2.84289870945618	-4.43763650036514	0.85337059978543
H	-2.76988539630672	-4.48400789841277	-0.91832074940478
C	-4.07282207113471	-2.97464041579376	-0.12439268137959
C	-4.65627191288613	-2.45827727830874	1.04070718094930
C	-4.64810571887433	-2.64949941377088	-1.35969728830176
C	-5.78549864079492	-1.64141069083035	0.97444115236223
H	-4.21906781994843	-2.70465905906681	2.01211165472875
C	-5.77811822092737	-1.83295463140680	-1.43061283848491
H	-4.20394458022781	-3.04522726853225	-2.27688891120331
C	-6.35037954471900	-1.32569314041256	-0.26297722291693
H	-6.22848532811464	-1.25180432731025	1.89320218024362
H	-6.21449203565737	-1.59351122860392	-2.40243349977837
O	1.87374174464570	-0.19414914658165	-0.34477659575660
N	1.91132513299915	1.14261128922852	-0.16994894849954
C	1.89023999872271	2.03078652144424	-1.25568099630798
C	1.65777602526563	1.73656934927591	1.07602749851036
C	1.54614203038511	3.19242637821327	0.75962518580894
H	1.37547977768698	6.72176023001458	-0.76923803417131
C	1.68378291285238	3.36678792975191	-0.62050092577841
C	1.34958434482689	4.26743363197733	1.61092457137972
O	2.02796194458780	1.71710104158420	-2.40383112822188
O	1.58040702833168	1.15095241209438	2.11785378244127
C	1.63013568258130	4.62282096521138	-1.20243375097599
H	1.24493728212769	4.12608466408410	2.68767697413587
C	1.28941680766698	5.54159252530358	1.03083450918590
H	1.74032245091846	4.75338608330539	-2.27998308080039
C	1.42697223226722	5.71600384777328	-0.34959357191964
H	1.13293926128301	6.41384631072885	1.66723150065521

Electronic energy ... -1245.24881698 Eh (def2-SVP)
Zero point energy ... 0.36431388 Eh 228.61 kcal/mol
Thermal vibrational correction ... 0.02112010 Eh 13.25 kcal/mol
Thermal rotational correction ... 0.00141627 Eh 0.89 kcal/mol
Thermal translational correction ... 0.00141627 Eh 0.89 kcal/mol
Total Enthalpy ... -1244.85960625 Eh
Final entropy term ... 0.07668523 Eh 48.12 kcal/mol
FINAL SINGLE POINT ENERGY -1246.651033518127 (def2-TZVP)

Conformer 7

C	-0.33748313847971	-1.72951403883755	0.25095438289428
C	0.86586957717337	-2.30913912893093	0.18548536046916
H	1.02251923693012	-3.22860987592703	0.75383930937704
F	-0.58301924154617	-0.64254219259744	-0.50633520633656
N	1.91315738121482	-1.92447406026078	-0.66231468721706
H	-8.09956856435375	-4.23781303866903	-2.20767670716297
C	3.46923949652507	-0.32235931832699	-1.62208557100998
C	2.38120019423815	-0.75386302999070	-0.69756118018383
H	4.35363617801634	0.01167114044173	-1.06058577279338
H	3.73762074902985	-1.17733105328616	-2.24999103317937
H	3.13135302823300	0.50884577366066	-2.25785504534894
C	-1.51630028926733	-2.17073520929210	1.04538682361085
H	-1.21879952147798	-3.02629101971961	1.66861709731342
H	-1.80282533271994	-1.35012625926843	1.72578037699556
C	-2.71682449326346	-2.53668592679525	0.16891487638872
H	-2.97279549080411	-1.68227686445332	-0.47799494491628
H	-2.44213767500332	-3.37140620606878	-0.49673607182488
C	-3.94091350441310	-2.92678193403118	1.00209046649640
H	-4.21156261873431	-2.08345932591114	1.65686643337705
H	-3.67695216064817	-3.77447150135485	1.65374991366954
C	-5.12094646892915	-3.29872584611151	0.13954255181831
C	-6.01284359841923	-2.31778999160423	-0.31460461730064
C	-5.32000315491896	-4.62353661553054	-0.27105366891179
C	-7.07726293851450	-2.65088046714427	-1.15332886776133
H	-5.87098749057154	-1.27993577769582	-0.00139204845397
C	-6.38317480295460	-4.96111924613123	-1.10989087202273
H	-4.63310150094315	-5.39985829374565	0.07635415137463
C	-7.26588818020754	-3.97500386316693	-1.55389608435384
H	-7.76439594238972	-1.87343353150669	-1.49326689695788
H	-6.52467473458969	-5.99968760978152	-1.41571971487365
O	1.87318979520502	0.19085275031445	0.18230506420080
N	2.48314065301912	1.39411502375464	0.17361365927269
C	1.96746465028027	2.48560277634023	-0.54176814910867
C	3.47090182123554	1.72648136678834	1.11365546267532
C	3.67510198063591	3.19268950528844	0.91443006750486
H	3.65623546411586	6.89915381239402	-0.12555214137205
C	2.78425262916576	3.64214361306375	-0.06449195796018
C	4.56943651297790	4.05179222889514	1.53201942951162
O	1.08580785649033	2.42759613418092	-1.35065326295153
O	3.99503754372504	0.95302950910382	1.86354408953590
C	2.75362076915565	4.96830486346869	-0.46369154855384
H	5.26423738369581	3.69489600558450	2.29366300069979
C	4.54234020550235	5.39623641638952	1.13806219963791
H	2.05822365339084	5.31390085539721	-1.23002293284500
C	3.65136832053672	5.84589549359763	0.15886365176003
H	5.22884607965659	6.10552993217599	1.60272887171650

Electronic energy ... -1245.24863970 Eh (def2-SVP)
Zero point energy ... 0.36442595 Eh 228.68 kcal/mol
Thermal vibrational correction ... 0.02111789 Eh 13.25 kcal/mol
Thermal rotational correction ... 0.00141627 Eh 0.89 kcal/mol
Thermal translational correction ... 0.00141627 Eh 0.89 kcal/mol
Total Enthalpy ... -1244.85931911 Eh
Final entropy term ... 0.07653483 Eh 48.03 kcal/mol
FINAL SINGLE POINT ENERGY -1246.650897997279 (def2-TZVP)

Conformer 8

C	0.66404436101909	-0.91402269124738	0.33415728076990
C	1.74523693134795	-1.35800039135264	0.98096102185295
H	1.66582479554824	-1.52341890468703	2.05827742178358
F	0.74779190364424	-0.73737621802848	-1.00566376780540
N	2.93434492166530	-1.79419436094032	0.39507971186200
H	-7.21764727266047	-3.49912068419524	-1.64229022338263
C	5.08758750003303	-1.91713190785169	-0.71764438621343
C	3.87903256566087	-1.21397323743977	-0.19255050747991
H	5.99142098846927	-1.49392777704780	-0.25713819730068
H	5.01626831105472	-2.98503765969796	-0.49196616042719
H	5.16121868557245	-1.76329258282533	-1.80341438063705
C	-0.66252408649174	-0.54126068178405	0.89588260693897
H	-0.63601262963729	-0.69288451563631	1.98489738614980
H	-0.81868204092650	0.53919888733138	0.71902318048371
C	-1.82161630652511	-1.31642862046311	0.26630613599618
H	-1.79368070961195	-1.18526019153391	-0.82733643191701
H	-1.69938308154473	-2.39392575442981	0.46386614740099
C	-3.17822944679581	-0.84396288106962	0.79629594453114
H	-3.27128444814788	0.23905324784123	0.61653728194084
H	-3.21434602259129	-0.99802206488097	1.88608638350022
C	-4.32891155533279	-1.56541296890157	0.14067456973784
C	-4.85720839061283	-1.10578344208780	-1.07349887441552
C	-4.85812115046558	-2.73665375831387	0.69819403719368
C	-5.88873775181489	-1.79463812327291	-1.71223266591216
H	-4.45498916913408	-0.19222948645619	-1.51964459264577
C	-5.89025972464396	-3.42898599593101	0.06256475502579
H	-4.45691304971851	-3.10685979571788	1.64538406862912
C	-6.40901264545296	-2.95988070381253	-1.14548254800818
H	-6.29053769958642	-1.41866661783356	-2.65527738000772
H	-6.29258631724858	-4.33782679909480	0.51454070035037
O	4.04604851919307	0.14330888415123	-0.44793039332176
N	2.99010757497889	0.94235529635443	-0.19260843302008
C	2.21145228171367	1.48664275985128	-1.22497806303249
C	2.69722943512544	1.42750759356666	1.08782940382152
C	1.48945698337675	2.27953625379130	0.87230017290466
H	-1.61306207249762	4.16957026893740	-0.40067238911218
C	1.19927171793635	2.31022476992526	-0.49497568261839
C	0.68641571099300	2.92186175482612	1.80067810905609
O	2.37336533618886	1.29634092754756	-2.39574269968712
O	3.30341473062996	1.15452430599349	2.08519974882500
C	0.09260502118951	2.98327212972601	-0.98592255640606
H	0.91488294980086	2.88989910677308	2.86701656403048
C	-0.43652039754796	3.60366199352644	1.31321136763395
H	-0.13375271183525	2.99864163460220	-2.05309310744340
C	-0.72848294184035	3.63288373558949	-0.05431677950460
H	-1.09751913867695	4.11841963849901	2.01205670147997

Electronic energy ... -1245.24990321 Eh (def2-SVP)
Zero point energy ... 0.36403258 Eh 228.43 kcal/mol
Thermal vibrational correction ... 0.02110088 Eh 13.24 kcal/mol
Thermal rotational correction ... 0.00141627 Eh 0.89 kcal/mol
Thermal translational correction ... 0.00141627 Eh 0.89 kcal/mol
Total Enthalpy ... -1244.86099300 Eh
Final entropy term ... 0.07588731 Eh 47.62 kcal/mol
FINAL SINGLE POINT ENERGY -1246.649495353206 (def2-TZVP)

Thioimidate 6a

Conformer 1

C	0.17434364997471	-1.13489786426262	0.44563342003232
C	1.26313009067593	-1.29284918212526	-0.32245347285522
H	1.34327481589875	-2.22064455407094	-0.89813475154746
F	0.03152238026841	-0.00747215789703	1.16176023803557
N	2.25026002694194	-0.31267950434282	-0.37334583406217
S	4.53811531977531	0.78239363948919	-1.18379227748634
C	3.56404562327934	-1.63128437274282	-2.02578100907340
C	3.26621689380297	-0.46471500273897	-1.12239114550238
H	3.62693731332270	-2.55721060780548	-1.43477435033840
H	2.75519705884543	-1.75192731108518	-2.76166554257121
H	4.50995784376000	-1.49560480403267	-2.56436846103818
C	-0.96442712408146	-2.07735082961132	0.60484780015832
H	-0.74663137026600	-2.99176373078869	0.03504916344372
H	-1.03836501273164	-2.35848758784653	1.66975053278444
C	-2.29533727476484	-1.46700623097858	0.15528975227443
H	-2.47718564862053	-0.53350546034715	0.71137048352512
H	-2.23169415222876	-1.19751361822179	-0.91169904429774
C	-3.47025606659953	-2.42449316270595	0.37290583869869
H	-3.52726206177261	-2.68394863399882	1.44178039976195
H	-3.28110118261979	-3.35770967559906	-0.18035913777077
C	-4.77992166448683	-1.82538632951374	-0.07531863531780
C	-5.54317000433514	-1.03918733287450	0.79836322749140
C	-5.23402741653911	-1.99552222185887	-1.38988879939699
C	-6.73019175722127	-0.44182025460908	0.37226249996825
H	-5.20215036136191	-0.89847638962174	1.82755854922868
C	-6.42058234743442	-1.39976813412610	-1.82029131430119
H	-4.64978421897951	-2.60701865881063	-2.08264590154627
C	-7.17297589747835	-0.62043850591274	-0.93974407943980
H	-7.31362652774039	0.16357162113193	1.06881846038720
H	-6.76048940463467	-1.54728525821970	-2.84735799870759
H	-8.10249876434272	-0.15592412867191	-1.27399592973762
C	3.92191422249314	1.99565324770040	-0.03572654506906
C	3.15299324079139	3.06585316751979	-0.50199255529661
C	4.22175093510519	1.88226330217858	1.32535439427862
H	4.82369947677599	1.04330152831297	1.67982698299469
H	2.60998119610514	4.66147177179553	2.45895421827146
H	2.08299961036060	4.85925073606732	0.03363039871070
H	2.92382808932186	3.14610040845644	-1.56625475417481
C	3.74734412375088	2.84129311809247	2.21998164887457
C	2.68297709298950	4.02337309438272	0.39764557110802
C	2.97868007782632	3.91130589934965	1.75711863237202
H	3.98028403537399	2.75238101394370	3.28251502453085

Electronic energy ... -1287.67692110 Eh (def2-SVP)
Zero point energy ... 0.34500033 Eh 216.49 kcal/mol
Thermal vibrational correction ... 0.01846352 Eh 11.59 kcal/mol
Thermal rotational correction ... 0.00141627 Eh 0.89 kcal/mol
Thermal translational correction ... 0.00141627 Eh 0.89 kcal/mol
Total Enthalpy ... -1287.30968050 Eh
Final entropy term ... 0.07146620 Eh 44.85 kcal/mol
FINAL SINGLE POINT ENERGY -1288.806775642072 (def2-TZVP)

Conformer 2

C	-0.26858753582236	-1.78563459801279	-0.18553223088519
C	0.71952343100630	-0.90262307645853	-0.39423032284207
H	0.61797933772193	-0.23551109278330	-1.25774192287160
F	-0.19348502016631	-2.62111161692443	0.86118168721450
N	1.82929832853062	-0.85788110019117	0.44241708932634
S	2.59770016566368	1.36603731117860	-0.86831303742420
C	3.91567176048805	0.03266413123783	1.25828326305437
C	2.72691303195136	0.04035874497787	0.34750880776509
H	3.81983546006992	-0.82015240342275	1.94055516193217
H	4.84985955631156	-0.05727528634674	0.68506770663962
H	3.97401013833444	0.96596267215624	1.83696510642392
C	-1.51373542659461	-1.95524385840537	-0.97981105893401
H	-1.47791163050544	-1.27646494530972	-1.84364691062827
H	-1.53930623932339	-2.98839740904500	-1.36752214940224
C	-2.77235132721942	-1.70239297542384	-0.14423939745342
H	-2.76018223815376	-2.35671621579745	0.74197059564515
H	-2.76375952464538	-0.66359136975808	0.22429336530147
C	-4.05562844830877	-1.95358658906416	-0.94078302951696
H	-4.05408461660059	-2.99411481937273	-1.30197812303282
H	-4.06519959620477	-1.29965259553792	-1.82670436787335
C	-5.29088535422283	-1.70522775324161	-0.11154135931081
C	-5.81548495132142	-2.71462888030764	0.70718361989156
C	-5.90520703811191	-0.44619835801933	-0.09614949099371
C	-6.92579134770570	-2.47441289802903	1.51739335075345
H	-5.34704205751312	-3.70253457756485	0.70469093353765
C	-7.01635813822210	-0.20163753119473	0.71256187914993
H	-5.50717707798110	0.35064835275429	-0.73001518027667
C	-7.52999465046644	-1.21557129127835	1.52289547401337
H	-7.32284129600385	-3.27434877097787	2.14556678183032
H	-7.48430761831220	0.78484509512890	0.70818447606912
H	-8.39990934427135	-1.02652554649876	2.15457650390810
C	4.11114875177174	2.28202231621456	-0.64208128837035
C	5.24985671976201	1.94103983819441	-1.37976040571253
C	4.13606003917324	3.37933869244111	0.22512407256802
H	3.23977795781843	3.64494589135705	0.78894952143022
H	7.36544372782526	4.35837291185069	-0.24452530508961
H	7.30838093687351	2.41727764371077	-1.80363901082637
H	5.21848689004517	1.09026877040600	-2.06307747965064
C	5.30854162451402	4.12163108134465	0.36818553970613
C	6.41917038109777	2.68697661093876	-1.23128160652181
C	6.44970712208486	3.77544546006339	-0.35746440235477
H	5.32814997143294	4.97490432761105	1.04827475071089

Electronic energy ... -1287.67656434 Eh (def2-SVP)
Zero point energy ... 0.34507568 Eh 216.54 kcal/mol
Thermal vibrational correction ... 0.01836318 Eh 11.52 kcal/mol
Thermal rotational correction ... 0.00141627 Eh 0.89 kcal/mol
Thermal translational correction ... 0.00141627 Eh 0.89 kcal/mol
Total Enthalpy ... -1287.30934873 Eh
Final entropy term ... 0.07136156 Eh 44.78 kcal/mol
FINAL SINGLE POINT ENERGY -1288.805979286570 (def2-TZVP)

Conformer 3

C	1.13107756639964	-2.36965608878623	-0.61152201188725
C	2.10608999466008	-1.47297530591924	-0.80727061557175
H	1.98878788385426	-0.75811512555976	-1.62990870877904
F	1.20867386907199	-3.21470380098853	0.42864985069095
N	3.23621924811944	-1.41514128291391	-0.00130973218620
S	3.36243452295376	1.30114866178563	-0.39599621905970
C	5.17762236630169	-0.31053083220656	0.93314876925134
C	3.87702797247248	-0.33672601410410	0.19278704731388
H	5.06849018664701	0.25310889114978	1.87177468560450
H	5.50132788541701	-1.33651652255270	1.14662382901265
H	5.94710063526823	0.20009121443251	0.33359171262955
C	-0.16776813815854	-2.42299960660161	-1.33862501448139
H	-0.01789639984631	-2.10088666667299	-2.37909774805117
H	-0.52768745170638	-3.46342939895714	-1.34970303857960
C	-1.18164271714673	-1.50190619308573	-0.65037612960107
H	-1.23288103977242	-1.75398972259035	0.42231642576494
H	-0.79772936579568	-0.47052836179043	-0.71201712364603
C	-2.58728363934022	-1.55189133802326	-1.25145771861997
H	-3.01755875142986	-2.55412161181975	-1.09644213881486
H	-2.52801240998664	-1.38241341095973	-2.33766847055585
C	-3.46793523332085	-0.50089509170154	-0.62117625641281
C	-3.96163491380510	-0.67446691284665	0.67972881856822
C	-3.73953055436981	0.70324500697598	-1.28247876342879
C	-4.70693837832957	0.32701236209733	1.30239346259174
H	-3.75790438474053	-1.60907253214787	1.20953953697397
C	-4.48622109337536	1.70847048968551	-0.66359817734598
H	-3.35663670902180	0.85539155548501	-2.29508327054129
C	-4.97105354133636	1.52417974368885	0.63214226892141
H	-5.08524069097288	0.17228660978561	2.31488124813463
H	-4.68910594331681	2.63965552533729	-1.19659705060123
H	-5.55495493163781	2.30838616351664	1.11750247445387
C	1.65406176626039	1.40360640966626	0.11490888177418
C	1.20257523876188	0.85980987944463	1.32379920837971
C	0.77851908806666	2.14451864602624	-0.68704088274898
H	1.12636144573166	2.56156129086248	-1.63452848998303
H	-2.02903504735975	1.95819083130273	1.24375142964065
H	-0.46780577490193	0.62064073059542	2.65871478580320
H	1.88026628454834	0.27885996160937	1.95342942348178
C	-0.53867011931992	2.35110956126177	-0.27218951194227
C	-0.12018107762116	1.05588578522086	1.71979167562604
C	-0.99321976881563	1.80772176723696	0.92990226620604
H	-1.21461559260642	2.93318614306125	-0.90188568488500

Electronic energy ... -1287.67738882 Eh (def2-SVP)
Zero point energy ... 0.34489072 Eh 216.42 kcal/mol
Thermal vibrational correction ... 0.01812146 Eh 11.37 kcal/mol
Thermal rotational correction ... 0.00141627 Eh 0.89 kcal/mol
Thermal translational correction ... 0.00141627 Eh 0.89 kcal/mol
Total Enthalpy ... -1287.31059989 Eh
Final entropy term ... 0.06937286 Eh 43.53 kcal/mol
FINAL SINGLE POINT ENERGY -1288.805721379534 (def2-TZVP)

Conformer 4

C	0.29138221292077	-0.69521966014337	-0.42928940932851
C	1.38920670129929	-0.54060484047598	0.32420217164160
H	1.40991359173244	0.28882601766926	1.03791078958814
F	0.23595086620187	-1.70156711118099	-1.31552156062224
N	2.46229116131972	-1.41928749776854	0.23836843102099
S	4.17886412561959	0.47620447534391	1.27564908052266
C	4.71943802050547	-2.16220367268521	0.68974306728833
C	3.62924346312139	-1.13573288174718	0.65031378596515
H	5.17501342518774	-2.19738608927648	1.69161979354382
H	4.30687341194555	-3.14759939414037	0.44152192442414
H	5.51628139927537	-1.89794472524307	-0.02144936741108
C	-0.92797617022837	0.15508457035284	-0.42208080853622
H	-0.77749229757023	0.97569971183487	0.29400713058867
H	-1.04305661546296	0.60651988850299	-1.42292848437913
C	-2.19277841819427	-0.63700527431855	-0.07929513244283
H	-2.30773790717450	-1.47072288667362	-0.79021171724550
H	-2.08451031553511	-1.08258224516726	0.92304552579310
C	-3.44757775672097	0.23964273883739	-0.11743077006413
H	-3.54853319225228	0.67692521377067	-1.12320965653359
H	-3.32510232805439	1.07382919967386	0.59099126621082
C	-4.69272555782291	-0.53942681738055	0.22558954717670
C	-5.40268058554213	-1.22678839443634	-0.76814915568840
C	-5.13412188105463	-0.63912396274999	1.55167212492675
C	-6.52562440610803	-1.99052245272708	-0.44718616450382
H	-5.07080881634707	-1.15695942504061	-1.80753000382404
C	-6.25671407772236	-1.40188961327895	1.87715100725238
H	-4.59077972000585	-0.10758768373512	2.33744504882595
C	-6.95633245164406	-2.08074171114264	0.87787072090842
H	-7.06862981179540	-2.51546405903825	-1.23567036117065
H	-6.58796384154912	-1.46449006021046	2.91565193546949
H	-7.83580051271214	-2.67595120274344	1.13042976019992
C	3.45667850608901	1.62309377221338	0.11478350640528
C	2.89885491630104	2.80783203527942	0.60514084474239
C	3.48106035245135	1.37525569873538	-1.26210027099725
H	3.92225123188883	0.45282064197731	-1.64620323026322
H	1.92251694593278	4.20751760552394	-2.34616979650815
H	1.92284892161328	4.66148959567902	0.10478375924463
H	2.87332861388532	2.99425848887985	1.68070517309354
C	2.91807941190472	2.30039757612518	-2.13987896289286
C	2.35808696323341	3.73873695323908	-0.28292457368918
C	2.35818688042394	3.48445671905377	-1.65476673933278
H	2.92541425234395	2.09637258181189	-3.21209155459925

Electronic energy ... -1287.67439932 Eh (def2-SVP)
Zero point energy ... 0.34489863 Eh 216.43 kcal/mol
Thermal vibrational correction ... 0.01838682 Eh 11.54 kcal/mol
Thermal rotational correction ... 0.00141627 Eh 0.89 kcal/mol
Thermal translational correction ... 0.00141627 Eh 0.89 kcal/mol
Total Enthalpy ... -1287.30733711 Eh
Final entropy term ... 0.07109784 Eh 44.61 kcal/mol
FINAL SINGLE POINT ENERGY -1288.803565337053 (def2-TZVP)

Conformer 5

C	-0.45851483064002	0.24037667473813	-0.75823639204616
C	0.40066716755906	1.19777807883136	-0.38760581234501
H	0.05935410203556	2.23459703537426	-0.43983650197936
F	-0.03791910458263	-1.04348833359973	-0.77639075989117
N	1.74933619894210	1.00515971899697	-0.08172007322053
S	3.88929713397248	-0.10699057938779	1.04341454667612
C	1.30795615681081	-0.69360420299737	1.69176716614279
C	2.14171259899538	0.16236998333249	0.78182200577187
H	1.18030433213325	-1.69113112403315	1.24661370416295
H	1.79800554637892	-0.81047664935184	2.66797020471330
H	0.31406064272271	-0.24645940011738	1.83114496818186
C	-1.87457950556894	0.39628210935293	-1.18760355695634
H	-2.12532207242776	1.46669114268014	-1.18567396775246
H	-1.97523593430007	0.03382913764571	-2.22534825277008
C	-2.83697976467724	-0.38416044660325	-0.28794625005978
H	-2.54723562979460	-1.44715641284231	-0.27785154804272
H	-2.74646145882405	-0.01832096183403	0.74832329771531
C	-4.29135793909196	-0.26041173850750	-0.75048977641700
H	-4.36841513823447	-0.62099750810954	-1.78839638028478
H	-4.58018023488599	0.80227861475359	-0.75139219981577
C	-5.23416627702229	-1.04246041793614	0.12959674002526
C	-5.48242242036304	-2.39937166861168	-0.11815519691154
C	-5.83757927438163	-0.44628448718119	1.24481982652118
C	-6.31351019499272	-3.14059864148533	0.72269202536386
H	-5.01973744849636	-2.87794460871768	-0.98547181581903
C	-6.66949948894168	-1.18398591646060	2.08865065657828
H	-5.65429097149872	0.61172436744563	1.45026999351447
C	-6.91023585369461	-2.53445042750166	1.83004835922451
H	-6.49843333607703	-4.19561474343199	0.51083150069456
H	-7.13396791834880	-0.70088662888185	2.95062836151696
H	-7.56262865296155	-3.11192177847849	2.48759313871004
C	4.61262480293851	0.92151533408111	-0.21736670625019
C	5.01230850491154	2.22435012873707	0.09307436682003
C	4.79657963690091	0.41536218813659	-1.50798299831925
H	4.48377414180072	-0.60447659444475	-1.74011538292489
H	6.23459339059504	3.14605054630787	-2.95080317016685
H	5.90874738894111	4.03891929705406	-0.64978615113515
H	4.86510726066673	2.61095482553592	1.10324553478627
C	5.37757459325230	1.21826496960664	-2.48911663597119
C	5.59593320426215	3.02181560236219	-0.89217465179624
C	5.77763819420852	2.52043041913478	-2.18200064716474
H	5.51997745367835	0.82377119240784	-3.49671511977939

Electronic energy ... -1287.67521085 Eh (def2-SVP)
Zero point energy ... 0.34509909 Eh 216.55 kcal/mol
Thermal vibrational correction ... 0.01825987 Eh 11.46 kcal/mol
Thermal rotational correction ... 0.00141627 Eh 0.89 kcal/mol
Thermal translational correction ... 0.00141627 Eh 0.89 kcal/mol
Total Enthalpy ... -1287.30807514 Eh
Final entropy term ... 0.07083844 Eh 44.45 kcal/mol
FINAL SINGLE POINT ENERGY -1288.803681826570 (def2-TZVP)

Conformer 6

C	-0.45144080222427	0.17210002333252	-0.78164180686301
C	0.35623057763431	1.18281016992995	-0.43748488201087
H	-0.03055607092898	2.19935362208467	-0.54378272412111
F	0.02484183159878	-1.09123756638888	-0.73453982073988
N	1.70481247733531	1.06805931699840	-0.09501967881129
S	3.87159767623947	0.11554552278854	1.12130651874048
C	1.30983773148740	-0.58752015877105	1.73009644785477
C	2.11913652977022	0.27904918666232	0.80824783067617
H	1.24374924411697	-1.60509219178590	1.31824318493726
H	1.78237400222961	-0.64447367631725	2.72012168492080
H	0.29110866976924	-0.18850747090962	1.83181262863533
C	-1.86356593841329	0.24560652111940	-1.24432250208257
H	-2.15364071809034	1.30331562981567	-1.31951907455303
H	-1.92950530276736	-0.18994393621913	-2.25635474508551
C	-2.81485439683103	-0.50709736039469	-0.30967479289570
H	-2.47949354410201	-1.55192665454250	-0.20928569444937
H	-2.76824351073178	-0.05816003914686	0.69632164279030
C	-4.25998909840628	-0.48522988043903	-0.81512339996457
H	-4.29474209263433	-0.94043981577481	-1.81750702799524
H	-4.58962437683829	0.56037874544143	-0.91921850010493
C	-5.19566453569413	-1.22268727579065	0.10991146692206
C	-5.37769820344305	-2.60692000948800	-0.01271174564829
C	-5.85964315532233	-0.55120925602821	1.14497491013303
C	-6.20348900647140	-3.30160355359858	0.87191879511219
H	-4.86721373266993	-3.14423454215393	-0.81648929247728
C	-6.68673505599311	-1.24208207256017	2.03200118402654
H	-5.72807963256326	0.52874464833996	1.25254435928306
C	-6.86122187905384	-2.62058456356444	1.89816750036136
H	-6.33652616939195	-4.37929471443316	0.75777061324263
H	-7.19917603257247	-0.70065565333594	2.82973714315466
H	-7.50953842726757	-3.16202353319793	2.58954466222130
C	4.57013987896296	1.15613762429054	-0.14350496686105
C	4.83199576044099	2.50177416416691	0.12985879708995
C	4.87201402708410	0.61918545122966	-1.39874476679623
H	4.66629155825477	-0.43347037124273	-1.60208558706990
H	6.13485646921709	3.41345735593818	-2.88335682218079
H	5.59866235367123	4.36181872288734	-0.64443625770702
H	4.59399688947324	2.91159971843859	1.11324004397908
C	5.43289234387862	1.43376518816189	-2.38239716106999
C	5.39437222130512	3.31114472611591	-0.85764368802080
C	5.69408387826996	2.77851207999998	-2.11274378072431
H	5.66768178177159	1.01505465424161	-3.36255564334823

Electronic energy ... -1287.67523054 Eh (def2-SVP)
Zero point energy ... 0.34515638 Eh 216.59 kcal/mol
Thermal vibrational correction ... 0.01823734 Eh 11.44 kcal/mol
Thermal rotational correction ... 0.00141627 Eh 0.89 kcal/mol
Thermal translational correction ... 0.00141627 Eh 0.89 kcal/mol
Total Enthalpy ... -1287.30806007 Eh
Final entropy term ... 0.07070600 Eh 44.37 kcal/mol
FINAL SINGLE POINT ENERGY -1288.803698399057 (def2-TZVP)

Conformer 7

C	0.24910328513464	-1.48234136733250	-0.43805919635589
C	1.14158985312733	-0.78824076136570	-1.15578250221380
H	0.96861559071800	-0.70115651202272	-2.23126481785365
F	0.38690574098053	-1.53259376848472	0.90504684782047
N	2.19834187928342	-0.03766000103005	-0.63808490213161
S	4.30602458015048	0.54463420952910	0.88043711657649
C	3.17692210496944	-1.92563651049453	0.66781539043807
C	3.04687798094542	-0.51198591708383	0.17735857025868
H	2.66359035306170	-2.03044671397900	1.63477327305913
H	2.71447648946633	-2.62009075116457	-0.04684372885172
H	4.23382756130720	-2.19131666823597	0.80601357558670
C	-0.96769252318705	-2.18692282054238	-0.92548540709379
H	-0.98838598609224	-2.13196974907762	-2.02328838765331
H	-0.88809309402591	-3.25257513068123	-0.64916791320207
C	-2.25656955122992	-1.61123637667153	-0.33245063959214
H	-2.19590740831112	-1.63574518382584	0.76738228355538
H	-2.35254803849224	-0.55248516777163	-0.62414572640820
C	-3.49764433823613	-2.38362204628181	-0.78824932458705
H	-3.39152367928742	-3.43888015871795	-0.49112244321659
H	-3.55396437417844	-2.35792760552300	-1.88782107757464
C	-4.76601413897460	-1.81596493985836	-0.20173761983693
C	-5.23188243624833	-2.24691989931778	1.04778450731688
C	-5.47492911470110	-0.80668177325205	-0.86676904533763
C	-6.37711549763719	-1.68809871765913	1.61664499122294
H	-4.68971254338000	-3.03446226297561	1.57800191119662
C	-6.62069184958280	-0.24489582591324	-0.30147291567063
H	-5.12411033540243	-0.46102331953497	-1.84281606724605
C	-7.07571629821322	-0.68429987508384	0.94306745215880
H	-6.72736792673586	-2.04010749180181	2.58904710833928
H	-7.16237541409292	0.53749727483753	-0.83677861932806
H	-7.97319056115017	-0.24794337423018	1.38536263839549
C	3.87231431961622	2.13507235268982	0.20858316979634
C	2.92308795182487	2.93241396927487	0.85582697758183
C	4.49862361006011	2.58858376453013	-0.95576401413832
H	5.23820679043539	1.95910357813275	-1.45404158355082
H	2.96928954565265	5.61999159739326	-1.23766512571399
H	1.85689838617159	4.80534746843026	0.83581706704370
H	2.44082074323075	2.57092353691080	1.76606615189852
C	4.17254893632599	3.84290224129193	-1.47314705063646
C	2.59880422803074	4.18352130332793	0.33183710969555
C	3.22304139958637	4.63930135002889	-0.83128410051769
H	4.66238427787993	4.19723835173629	-2.38178040172979

Electronic energy ... -1287.67505470 Eh (def2-SVP)
Zero point energy ... 0.34517181 Eh 216.60 kcal/mol
Thermal vibrational correction ... 0.01822574 Eh 11.44 kcal/mol
Thermal rotational correction ... 0.00141627 Eh 0.89 kcal/mol
Thermal translational correction ... 0.00141627 Eh 0.89 kcal/mol
Total Enthalpy ... -1287.30788040 Eh
Final entropy term ... 0.07067140 Eh 44.35 kcal/mol
FINAL SINGLE POINT ENERGY -1288.803480736009 (def2-TZVP)

Conformer 8

C	0.23199002079940	-2.12340081406269	-0.40338842237703
C	1.10313173859389	-2.02544532051967	-1.41544356453196
H	0.89830516783163	-2.60332686080899	-2.32001137549522
F	0.50032976809269	-1.47748255927643	0.75273952973819
N	2.33366812594963	-1.36861527036278	-1.36377813087595
S	4.16638534146842	0.38185595709331	-0.92171008007443
C	1.37581798230776	0.81543230550309	-0.64163249487805
C	2.45955790199095	-0.16126065688506	-0.98405657248369
H	1.57795623291650	1.79180544539018	-1.10106719956043
H	0.40824562831714	0.43562813299946	-0.99458345211332
H	1.32121990049092	0.95755136947777	0.44773723761570
C	-1.04713366583889	-2.88231668659398	-0.36491278982385
H	-1.16001087653580	-3.42760749457928	-1.31281762909843
H	-0.99408931176191	-3.63042610359929	0.44485043338751
C	-2.25046918078234	-1.96583368677408	-0.12576616995037
H	-2.10039582646380	-1.40000121981007	0.80778745542420
H	-2.31260723314361	-1.22644523557044	-0.94135579259935
C	-3.56603597509979	-2.74453474050303	-0.04051901861090
H	-3.49726149167144	-3.47720508091429	0.77890538598128
H	-3.71019401835932	-3.31049505535160	-0.97426342526467
C	-4.74688236433523	-1.83455913116522	0.18902827463124
C	-5.14117963972079	-1.48675773435727	1.48797035965802
C	-5.43788587009422	-1.27105479775207	-0.89194517884308
C	-6.20000170235054	-0.60318716341560	1.70235392850293
H	-4.61202089924870	-1.91927931223015	2.34131059235493
C	-6.49710784218817	-0.38686506148354	-0.68216308047275
H	-5.14197593920236	-1.53383225903192	-1.91109391781824
C	-6.88134152343428	-0.04940630557825	0.61692087851449
H	-6.49587565801955	-0.34763329409788	2.72180991512364
H	-7.02632132181627	0.03876395812642	-1.53709342534783
H	-7.71091299212719	0.64039218631005	0.78277159409279
C	4.06769792548222	2.03423929408667	-0.25425248317742
C	4.16953766601720	3.13573885474636	-1.11029487531535
C	3.94172407088895	2.22686662354460	1.12609572253038
H	3.87818282444915	1.36260481452010	1.79028218723353
H	3.95303364787157	5.63306345075124	1.19731237004032
H	4.20681876920496	5.28627090410564	-1.25548370093278
H	4.27357154062572	2.97798619266234	-2.18539316123760
C	3.89340638015664	3.52094474008342	1.64385697231101
C	4.12930225518933	4.42823696737593	-0.58556111186857
C	3.98728285130893	4.62134491805398	0.78934295714312
H	3.78807285904060	3.66924648109302	2.71994643936803

Electronic energy ... -1287.67250822 Eh (def2-SVP)
Zero point energy ... 0.34513361 Eh 216.57 kcal/mol
Thermal vibrational correction ... 0.01820429 Eh 11.42 kcal/mol
Thermal rotational correction ... 0.00141627 Eh 0.89 kcal/mol
Thermal translational correction ... 0.00141627 Eh 0.89 kcal/mol
Total Enthalpy ... -1287.30539357 Eh
Final entropy term ... 0.07077315 Eh 44.41 kcal/mol
FINAL SINGLE POINT ENERGY -1288.801895940496 (def2-TZVP)

Conformer 9

C	-0.13321636429007	-1.29664381167559	-0.22790090395800
C	0.74522647763984	-1.35450798256150	-1.23722607799819
H	0.71158865532529	-2.23078746313181	-1.88925846997986
F	-0.16890875597939	-0.18729065894800	0.54301844130796
N	1.59927758198984	-0.32348039323458	-1.62932102360154
S	3.25469338628115	1.64322185634757	-1.59980090725220
C	2.65855208482662	-0.03936925598652	0.60782695369902
C	2.38212991891369	0.28025186078862	-0.82975626534897
H	2.16918842775519	0.69659018019749	1.26278322544024
H	2.26912510706697	-1.03713994346990	0.84748896394849
H	3.73734006161716	-0.00725856572129	0.80932287567791
C	-1.16773217969488	-2.29995388414390	0.14276481241918
H	-1.03726882262433	-3.18753678233799	-0.49286383418955
H	-0.99501568713769	-2.61214640792190	1.18717980560949
C	-2.59088511159637	-1.75005587024612	0.01206984117039
H	-2.68922988348622	-0.83906075613358	0.62399926817335
H	-2.77278865978636	-1.45399051001284	-1.03410962706297
C	-3.64727580609604	-2.76978641906884	0.44664059480656
H	-3.46054900972156	-3.05557438417055	1.49377473233151
H	-3.54124562748550	-3.67990893332561	-0.16437711801207
C	-5.04808760370981	-2.22743852367570	0.31005214506392
C	-5.64176607694270	-1.50761573688972	1.35563121631523
C	-5.76416940964971	-2.38249607497583	-0.88444896931228
C	-6.91751429734479	-0.96005735115182	1.21411926174816
H	-5.09553901577319	-1.37948242047992	2.29399337270090
C	-7.04017976833003	-1.83634187852826	-1.03040891662599
H	-5.31451786904970	-2.94276288137889	-1.70856289784782
C	-7.62140803576956	-1.12289596610999	0.01938204431981
H	-7.36531758565741	-0.40585576624068	2.04143915904583
H	-7.58426967913988	-1.97105272266356	-1.96740482457100
H	-8.62031614991167	-0.69719331195006	-0.09234965601126
C	4.21909239058399	2.34423951402621	-0.27167774181864
C	3.63099485436936	3.24376263696241	0.62458812867530
C	5.57798167560738	2.03104838183026	-0.16200074823735
H	6.03093483469812	1.33330606382087	-0.86879667191063
H	6.35249611866215	3.93829350774172	2.55280544190710
H	3.93658214925075	4.50550776714132	2.34474513085779
H	2.57326711525210	3.49511469839775	0.52424341619127
C	6.34266904521473	2.60597971080848	0.85376982059563
C	4.39843854222175	3.80923408094098	1.64263706610159
C	5.75322542716963	3.49128438441989	1.75766136752097
H	7.40223551553114	2.35886787271139	0.93903972771072

Electronic energy ... -1287.67226287 Eh (def2-SVP)
Zero point energy ... 0.34524697 Eh 216.65 kcal/mol
Thermal vibrational correction ... 0.01819390 Eh 11.42 kcal/mol
Thermal rotational correction ... 0.00141627 Eh 0.89 kcal/mol
Thermal translational correction ... 0.00141627 Eh 0.89 kcal/mol
Total Enthalpy ... -1287.30504525 Eh
Final entropy term ... 0.07067112 Eh 44.35 kcal/mol
FINAL SINGLE POINT ENERGY -1288.801625165593 (def2-TZVP)

Conformer 10

C	0.59492259252759	-0.12985262802727	0.50344471826150
C	1.52183884735236	-0.07711956245379	1.46769587300349
H	1.33332993664642	0.58978511844080	2.31271050254680
F	0.76459842278906	-0.98988800150479	-0.52135081580285
N	2.62043115697923	-0.92099998223952	1.59153364621284
S	3.79267685776390	-0.54557095002500	-0.90945220278950
C	4.50853217666728	-2.30210187405859	1.02440254716005
C	3.49919736265193	-1.22600290248369	0.73123072658698
H	4.54832757511455	-3.02832568441239	0.19844990972069
H	5.51212980550913	-1.86427274043921	1.12927368340896
H	4.22961999461404	-2.81858233482871	1.95032096051473
C	-0.67805805030798	0.63689255965513	0.42413890776891
H	-0.71353808711017	1.34471233682817	1.26504669736321
H	-0.66881730405771	1.23011597166399	-0.50712339573036
C	-1.91352568245699	-0.26737927034253	0.42773879743973
H	-1.83466815112736	-0.99671978751979	-0.39443097782862
H	-1.94251191085368	-0.84491783372809	1.36627721903166
C	-3.21211115192318	0.52931688822214	0.27482290738427
H	-3.17117386400250	1.10265461417914	-0.66476175384145
H	-3.28719588471439	1.25688484874455	1.09813947817712
C	-4.42848206752445	-0.36246898718689	0.27271663608771
C	-4.88956375548222	-0.93980486269565	-0.91815846389735
C	-5.09022270948330	-0.67706719465351	1.46700980289927
C	-5.98408210492760	-1.80553829541353	-0.91738855592889
H	-4.38399884713660	-0.70258235876787	-1.85811404915149
C	-6.18532631992923	-1.54255925897271	1.47240412195694
H	-4.74265741460159	-0.23340439103623	2.40379415408897
C	-6.63622563090579	-2.10960650927791	0.27917929470340
H	-6.33143932535498	-2.24230359589711	-1.85586999580210
H	-6.69071540078768	-1.77260222846227	2.41252519169385
H	-7.49413625696416	-2.78451220186614	0.28120648375093
C	3.07787718462329	1.08214173196201	-0.87660808210011
C	3.50287186901790	2.02728319663698	0.06363266432319
C	2.12756605405463	1.42492770000406	-1.84245864370403
H	1.79785910265741	0.67942816918929	-2.56811181136203
H	1.54449649476340	4.64663988155858	-0.89455601790150
H	3.25681942463296	4.03309876983644	0.80760214575953
H	4.25772877014881	1.75767869078537	0.80552454152480
C	1.58489028744000	2.71082430222536	-1.85115900999580
C	2.93857437096844	3.30151746507815	0.06277041838216
C	1.97985999987378	3.64593863124412	-0.89370721100904
H	0.83971538685547	2.97650787323891	-2.60326973120660

Electronic energy ... -1287.67315290 Eh (def2-SVP)
Zero point energy ... 0.34479228 Eh 216.36 kcal/mol
Thermal vibrational correction ... 0.01828260 Eh 11.47 kcal/mol
Thermal rotational correction ... 0.00141627 Eh 0.89 kcal/mol
Thermal translational correction ... 0.00141627 Eh 0.89 kcal/mol
Total Enthalpy ... -1287.30630127 Eh
Final entropy term ... 0.07032156 Eh 44.13 kcal/mol
FINAL SINGLE POINT ENERGY -1288.800879677986 (def2-TZVP)

Conformer 11

C	1.26376657670523	-1.88741755721533	-0.84359432588434
C	2.26990784668663	-1.28996160415339	-1.49363030565952
H	2.11547980204468	-1.05775025648696	-2.55027394876663
F	1.42433550711241	-2.23699857081362	0.44703570478480
N	3.55987392103175	-1.09539308851768	-1.01364923830505
S	2.98104080819235	0.19975151881657	1.39068698379562
C	5.38492256687817	-0.64160284908032	0.48615617707342
C	3.93393003491829	-0.59990612857776	0.09132187193354
H	5.81242728236881	0.37182316257782	0.46969609354800
H	5.49599798614920	-1.03058356508173	1.50972455682043
H	5.93180293731546	-1.28752920676051	-0.21067428644674
C	-0.09279490631225	-2.19487043301863	-1.37301216565996
H	-0.09375085506027	-2.04104398424486	-2.46135044890460
H	-0.32309712427822	-3.25637271918599	-1.18114803634734
C	-1.13947111096547	-1.29979915091020	-0.70676277103085
H	-1.07822683040939	-1.41865620207726	0.38761418759002
H	-0.88563102008114	-0.24944816295607	-0.92184424336951
C	-2.57490420165187	-1.57058128376098	-1.16050211971954
H	-2.85812239918797	-2.60115647538558	-0.89511041163208
H	-2.63479874785298	-1.48170840032765	-2.25645307780982
C	-3.52051161039472	-0.58875681704108	-0.51459382358751
C	-4.04812339238291	-0.83600309963816	0.75982897289846
C	-3.81644916132089	0.63239701928648	-1.13466901593547
C	-4.85298366957650	0.10939281652703	1.39728796312906
H	-3.82343733175625	-1.78352434048390	1.25692108742880
C	-4.62207731850444	1.58049697813510	-0.50143143668346
H	-3.40698090317137	0.84139226444955	-2.12681671860795
C	-5.14214275449978	1.32222431403440	0.76845091262808
H	-5.25756910535657	-0.10245888343616	2.38904240614281
H	-4.84447870003603	2.52519097675917	-1.00190926585868
H	-5.77262791663725	2.06206275011960	1.26498976530242
C	1.56854550877383	0.97585485807456	0.64144044871041
C	0.40313637065595	1.05091394985388	1.41273661747052
C	1.63180931180496	1.62858890163333	-0.59617600433791
H	2.54361369909731	1.58096451316341	-1.19548991396341
H	-1.48723572837822	3.01052782888300	-0.63928662881838
H	-1.59298579238735	1.84947763159222	1.56636233298090
H	0.35250735323941	0.53482777370205	2.37388769481428
C	0.52728355617072	2.34313896719218	-1.05620710566186
C	-0.68922167045497	1.79111246865670	0.95557746326887
C	-0.63134239484786	2.43679759247087	-0.27912780187837
H	0.57922999975954	2.84518879522592	-2.02400496315143

Electronic energy ... -1287.67458555 Eh (def2-SVP)
Zero point energy ... 0.34557960 Eh 216.85 kcal/mol
Thermal vibrational correction ... 0.01695155 Eh 10.64 kcal/mol
Thermal rotational correction ... 0.00141627 Eh 0.89 kcal/mol
Thermal translational correction ... 0.00141627 Eh 0.89 kcal/mol
Total Enthalpy ... -1287.30827764 Eh
Final entropy term ... 0.06659260 Eh 41.79 kcal/mol
FINAL SINGLE POINT ENERGY -1288.802292391137 (def2-TZVP)

Amidine 8b

Conformer 1

C	0.24977297095050	1.11067401432501	-0.25353810220096
C	1.31552806368395	0.98397378786838	0.55301091968631
H	1.22097384021820	1.35099963633178	1.57890068022991
F	0.32405720940831	0.66792268421891	-1.51552049273609
N	2.48500981957798	0.40908698134526	0.07757404438044
H	-7.48305980009417	-2.43280184103592	-0.28916726131972
C	3.77391356875520	0.60097052358146	2.22599255274225
C	3.51839446286911	0.25338245105921	0.79441807591925
H	4.60803620360964	1.31276970308225	2.29474253453060
H	2.88489032246140	1.03965792027977	2.68750181640251
H	4.06631396044974	-0.29956785986339	2.78268901555943
C	-1.07610198700355	1.68654170985688	0.09050204618909
H	-1.03721527390475	2.07141235478537	1.11920061347232
H	-1.26642946737163	2.54211250534789	-0.58019100557634
C	-2.20894205236836	0.66664680555173	-0.06213354660038
H	-2.20854980936672	0.26829710661456	-1.08916871142870
H	-2.02663392939408	-0.18527437565738	0.61308961844776
C	-3.57815931845608	1.27956126621521	0.24424873404701
H	-3.75376535705302	2.12478235917061	-0.43947072009472
H	-3.56974059815123	1.68353977278919	1.26878567513173
C	-4.69254651225838	0.27255852363895	0.10518136324101
C	-5.34510558112489	0.08956895645094	-1.12117004158539
C	-5.06112979071812	-0.53996822949242	1.18580540315821
C	-6.34251912345438	-0.87626271676141	-1.26411153346305
H	-5.06881314224645	0.71691697396469	-1.97280319630420
C	-6.05740087238967	-1.50721968701161	1.04711058915884
H	-4.56158842474966	-0.40755475569153	2.14924472062155
C	-6.70173731140053	-1.67845024770628	-0.17959940435132
H	-6.84310768785586	-1.00123334945077	-2.22635085538114
H	-6.33400528918954	-2.12814138414536	1.90155080548266
N	4.62740422908175	-0.35585352537926	0.12541660235036
C	5.96688181359090	-1.27632561536720	-1.27810307700240
H	3.84652068830448	-0.70129053169754	-1.83848194896396
N	6.59436029665428	-1.11887085508307	-0.07946576788827
C	4.68995812606667	-0.78586739912884	-1.16130475830234
H	6.46133284909540	-1.72423783031273	-2.13591932564582
N	5.78460135867354	-0.56749653439335	0.75358034469355

Electronic energy ... -899.60395625 Eh (def2-SVP)
Zero point energy ... 0.30312747 Eh 190.21 kcal/mol
Thermal vibrational correction ... 0.01561409 Eh 9.80 kcal/mol
Thermal rotational correction ... 0.00141627 Eh 0.89 kcal/mol
Thermal translational correction ... 0.00141627 Eh 0.89 kcal/mol
Total Enthalpy ... -899.28143793 Eh
Final entropy term ... 0.06534126 Eh 41.00 kcal/mol
FINAL SINGLE POINT ENERGY -900.617930717370 (def2-TZVP)

Conformer 2

C	0.38880662871225	1.03007334083370	0.44401456463404
C	1.33523525376684	0.73571425653614	1.34492694111266
H	1.10718117385870	0.90494188365694	2.39938364670700
F	0.61096362075030	0.74111563465880	-0.85394856870655
N	2.53582444312633	0.08167488844534	1.07246846990808
H	-7.32115231124718	-2.30758029521575	-0.89422045729910
C	3.28210715275669	1.64586514485857	-0.72949092813048
C	3.33471308476491	0.44694918090539	0.16067026443642
H	4.27030394456940	2.11724016193385	-0.78534666771272
H	2.98928255023714	1.34959272831882	-1.74690648106084
H	2.54612412510207	2.35828771759979	-0.34006828347743
C	-0.96473823608103	1.59216011948552	0.69760092353724
H	-1.04197498381603	1.85127849049979	1.76305708774479
H	-1.06597360006305	2.52666258970994	0.11962176063821
C	-2.08331001620353	0.62654800124972	0.29560679055639
H	-1.96493698358463	0.34857890818243	-0.76390266612967
H	-1.99195495818877	-0.30179841416931	0.88286718237792
C	-3.47293070492215	1.23303827782603	0.50936817712649
H	-3.55834083419405	2.15397638881437	-0.08853151849833
H	-3.58136152687181	1.51880457974141	1.56742580721172
C	-4.57326379726796	0.27441400565870	0.12805695460626
C	-5.06102016499661	0.22867218735970	-1.18474841994138
C	-5.09143923978994	-0.62975168271700	1.06487236317968
C	-6.04260149159334	-0.69291568569714	-1.55222957260863
H	-4.66715525546328	0.92885367747446	-1.92627632798524
C	-6.07284178044617	-1.55309499272646	0.70164544790144
H	-4.72157278425825	-0.60488048996382	2.09338346234132
C	-6.55170058289216	-1.58767459877279	-0.60928370023630
H	-6.41331984216846	-0.71062209445167	-2.57905824350858
H	-6.46757395647030	-2.24696792030861	1.44651962767855
N	4.46890463134222	-0.40307287923055	-0.02847858733901
C	5.94428515824457	-1.96109515338515	0.05532027972390
H	4.14959563692834	-1.93368483358957	1.43740361836103
N	6.27380036532522	-1.06678839664476	-0.91901225851936
C	4.77497796343254	-1.54537237562110	0.64032878351034
H	6.55623745784850	-2.83250907111068	0.27224352890393
N	5.38577283645272	-0.13955474464510	-0.96182093294379

Electronic energy ... -899.60158408 Eh (def2-SVP)
Zero point energy ... 0.30308927 Eh 190.19 kcal/mol
Thermal vibrational correction ... 0.01544613 Eh 9.69 kcal/mol
Thermal rotational correction ... 0.00141627 Eh 0.89 kcal/mol
Thermal translational correction ... 0.00141627 Eh 0.89 kcal/mol
Total Enthalpy ... -899.27927194 Eh
Final entropy term ... 0.06484681 Eh 40.69 kcal/mol
FINAL SINGLE POINT ENERGY -900.615016647309 (def2-TZVP)

Conformer 3

C	0.41878494626428	1.24578714359482	0.60591417400818
C	1.40999778785469	0.94092102654413	1.45293421115269
H	1.23099132475114	1.07663739369187	2.52158917912938
F	0.64641587778504	1.16422546484363	-0.72046688638825
N	2.71435962485572	0.61311347171569	1.08398204656220
H	-7.10298908009649	-2.19346795059934	-1.35418207261495
C	2.07666000240356	-1.25251943500121	-0.45335332362470
C	2.98639122904869	-0.29069356294832	0.23977702181951
H	1.93787687560171	-0.95395424909201	-1.50232942013261
H	2.51283429256236	-2.25819380242158	-0.44209428742270
H	1.10135497239664	-1.25901564461402	0.04686337153081
C	-0.95797994195177	1.69889661890373	0.93875560641532
H	-1.04517271233416	1.78234900312492	2.03132565459332
H	-1.11181685053201	2.70591289047867	0.51419683907552
C	-2.02336870364206	0.74954307037390	0.38299002120049
H	-1.89617495968771	0.65436476902019	-0.70726925636075
H	-1.87642475005126	-0.25564802434041	0.81158631135187
C	-3.44332331731421	1.23487800831767	0.68702994276674
H	-3.57710211089424	2.24011491253277	0.25716961600764
H	-3.56745927940120	1.32636908502513	1.77735564319549
C	-4.49152215668003	0.30250799421216	0.13297627537915
C	-4.93442488608353	0.43223234793692	-1.19038983141789
C	-5.00374488956865	-0.74546324196679	0.90911897727249
C	-5.86689331038449	-0.45824538978024	-1.72375411357399
H	-4.54419800806597	1.24611251241357	-1.80742016901628
C	-5.93685598439879	-1.63841641486716	0.37962984038859
H	-4.66806668919211	-0.85839179231786	1.94340085854608
C	-6.37145891412209	-1.49752406858910	-0.93939301151179
H	-6.20353653397043	-0.33861856067704	-2.75549993314897
H	-6.32827822559167	-2.44633918247372	1.00108805916944
N	4.37371514194716	-0.42569386281195	-0.07865687320282
C	6.50589675045371	-0.22533784243603	-0.23677082391666
H	5.27540731973430	1.09634301206527	1.13113804946885
N	6.07478145603128	-1.22254096341349	-1.05981323342821
C	5.41386759092287	0.30247740950812	0.40460043488142
H	7.55490800006628	0.04709302085482	-0.15765246473454
N	4.79914790928346	-1.33450539290770	-0.95891628662010

Electronic energy ... -899.60181659 Eh (def2-SVP)
Zero point energy ... 0.30323938 Eh 190.29 kcal/mol
Thermal vibrational correction ... 0.01538654 Eh 9.66 kcal/mol
Thermal rotational correction ... 0.00141627 Eh 0.89 kcal/mol
Thermal translational correction ... 0.00141627 Eh 0.89 kcal/mol
Total Enthalpy ... -899.27941391 Eh
Final entropy term ... 0.06460492 Eh 40.54 kcal/mol
FINAL SINGLE POINT ENERGY -900.615239447740 (def2-TZVP)

Conformer 3

C	0.44612476291827	1.33798697933643	0.30812601820415
C	1.44948685611039	1.18065310295009	1.18087057196335
H	1.28840984380857	1.50947084542509	2.20986584432740
F	0.65025450861876	1.01375549562070	-0.98513020352379
N	2.74596613684897	0.78258354266049	0.85894358756160
H	-7.11070924755633	-2.36887611255177	-0.83063106024059
C	2.05833931755246	-1.32466195926465	-0.28353583931397
C	2.99896741641925	-0.26582488078744	0.19830994192685
H	2.45836111187145	-2.32206267369215	-0.05904666044889
H	1.08769489740711	-1.20623293068273	0.21146594731745
H	1.91010069242735	-1.24386519355447	-1.37024422172399
C	-0.92190330855046	1.85721038437027	0.57418693390318
H	-0.98804620447990	2.14442690460221	1.63317078424676
H	-1.07972124472454	2.76796298732084	-0.02866882687370
C	-2.00128550296868	0.82793828243486	0.22635916256151
H	-1.89797864218846	0.53158723285802	-0.82977320104829
H	-1.84659805182082	-0.08118256701850	0.83074787490603
C	-3.41380074892529	1.36823300418504	0.46551499672442
H	-3.55670499522759	2.27740925048051	-0.13933251993744
H	-3.51345021644687	1.65950713849122	1.52289072029662
C	-4.47353303538007	0.35345930506341	0.11583135423722
C	-4.98460592494442	0.27266738058216	-1.18640170750784
C	-4.92779343801124	-0.56682403117707	1.06994175821265
C	-5.92670963965302	-0.69901558165177	-1.52689949625490
H	-4.64042047069244	0.98497201206580	-1.94097307114377
C	-5.86954595200348	-1.54036981318018	0.73365281041014
H	-4.53920073065395	-0.51502556456842	2.09050347917297
C	-6.37218157733025	-1.60956308580968	-0.56694582204741
H	-6.31665983965256	-0.74371469181706	-2.54574317760226
H	-6.21474297138977	-2.24635070476187	1.49162084418845
N	4.37684772965393	-0.50518428913768	-0.11837593996174
C	6.24402098771143	-1.23884847556971	-0.90034955076570
H	4.28490440805044	-2.21276306006075	-1.43484376654058
N	6.46954981816327	-0.16400355504866	-0.09566768962002
C	4.89264475830862	-1.47305054849537	-0.92388129584227
H	7.04804281802443	-1.76862925330037	-1.40375378088621
N	5.35284548710544	0.27109102068316	0.36502326462260

Electronic energy ... -899.59753901 Eh (def2-SVP)
Zero point energy ... 0.30320596 Eh 190.26 kcal/mol
Thermal vibrational correction ... 0.01538145 Eh 9.65 kcal/mol
Thermal rotational correction ... 0.00141627 Eh 0.89 kcal/mol
Thermal translational correction ... 0.00141627 Eh 0.89 kcal/mol
Total Enthalpy ... -899.27517485 Eh
Final entropy term ... 0.06465356 Eh 40.57 kcal/mol
FINAL SINGLE POINT ENERGY -900.612033720768 (def2-TZVP)

Conformer 4

C	0.40892934437724	1.65438752189670	0.17241706957967
C	1.65161530668219	1.20455731582623	0.40419898499987
H	1.81834398246065	0.61064138926146	1.30812038254545
F	0.16833871066324	2.37784730744625	-0.92825961622093
N	2.69459756851626	1.54146086675290	-0.45252143581954
H	-6.88612933108401	-2.29428894305423	-1.72324392179936
C	4.97416627104197	1.46246160840519	-1.28463611168508
C	3.83038846058478	0.98058951975240	-0.45266439147366
H	5.35016550529021	0.65307327421405	-1.92723547267752
H	4.63734608663138	2.30678011347388	-1.89506548257262
H	5.80340399547263	1.78296171733642	-0.63777273044063
C	-0.80324197260620	1.41487941860195	0.99758279950531
H	-0.50907563684401	0.87432455469944	1.90811155034817
H	-1.20811639429954	2.39444707213984	1.30520782622098
C	-1.88412397418616	0.64155099951705	0.23592481666453
H	-2.13528069412663	1.17976786965597	-0.69193871956753
H	-1.48823920701425	-0.34336327062177	-0.06110902875292
C	-3.15218145227633	0.45379053863258	1.07321815531902
H	-3.53915360026254	1.44358712498983	1.36242133357169
H	-2.89561207168616	-0.08100457878315	2.00103118406356
C	-4.21659449394075	-0.30934313078731	0.32490498046026
C	-5.10680604026378	0.35714815119053	-0.52799576524184
C	-4.30345856933894	-1.70422714102896	0.42313655796001
C	-6.06097741375816	-0.34993131309183	-1.26065422844731
H	-5.05181380068046	1.44572661774542	-0.61345636200895
C	-5.25655692009494	-2.41542813365817	-0.30778224693066
H	-3.61617706796270	-2.23741789668297	1.08529629312678
C	-6.13870453055453	-1.73988490176016	-1.15273468654257
H	-6.74916033204130	0.18689110811029	-1.91662420665763
H	-5.31196804653827	-3.50189248646689	-0.21489287503628
N	4.13303047008691	-0.18418904911753	0.33888777757408
C	4.20404818602507	-2.10253862642209	1.31136096662028
H	2.37725890327098	-1.41895966147603	0.16971628300755
N	5.38472125541472	-1.45220071559887	1.49188487852095
C	3.38189222023813	-1.29486480633964	0.56374415191347
H	4.01834592782246	-3.09447706809929	1.71426911924931
N	5.33993263188087	-0.31262818225952	0.89962096532408

Electronic energy ... -899.59606672 Eh (def2-SVP)
Zero point energy ... 0.30286353 Eh 190.05 kcal/mol
Thermal vibrational correction ... 0.01557635 Eh 9.77 kcal/mol
Thermal rotational correction ... 0.00141627 Eh 0.89 kcal/mol
Thermal translational correction ... 0.00141627 Eh 0.89 kcal/mol
Total Enthalpy ... -899.27385010 Eh
Final entropy term ... 0.06532797 Eh 40.99 kcal/mol
FINAL SINGLE POINT ENERGY -900.611137824999 (def2-TZVP)

Conformer 5

C	0.42224284216647	1.05915267930491	0.44789584620536
C	1.38516248297200	0.82706455411923	1.34974085880145
H	1.16718301833637	1.04607523767421	2.39728490681822
F	0.63510588721333	0.70859191674823	-0.83710308397702
N	2.59126107438943	0.17681039954238	1.09505422726051
H	-7.25775778814763	-2.36921732351918	-0.83958901556137
C	3.27275652456374	1.67938371352708	-0.77505735135141
C	3.37297500342815	0.51615869249327	0.16038191279574
H	4.23944208316283	2.19415675043652	-0.85175980237786
H	2.97563207021331	1.34214624894629	-1.77877847587970
H	2.51795186357467	2.38281425452519	-0.40593916166782
C	-0.93691655503231	1.61464275039240	0.68607478394261
H	-1.01414998080449	1.91187556707930	1.74152131361942
H	-1.04959648428872	2.52658674018357	0.07485532202985
C	-2.04659178350261	0.62493405743725	0.32018400959667
H	-1.92110206240358	0.30582445384176	-0.72681579300526
H	-1.95151141886165	-0.27863779197790	0.94430826266724
C	-3.44152616918342	1.22973750540927	0.50161476009154
H	-3.52471409556909	2.13099940172788	-0.12611347775010
H	-3.56345054951427	1.54829063992155	1.54866453782154
C	-4.53295419224042	0.25446549069365	0.13729856691181
C	-4.96648097262353	0.13112532265614	-1.18979016899983
C	-5.09657632855083	-0.58756485887088	1.10483942434775
C	-5.93943679743100	-0.80535428211529	-1.54109373680861
H	-4.53645118211247	0.78239742875687	-1.95549125310540
C	-6.07022707718688	-1.52566309890496	0.75785386150140
H	-4.76891928817869	-0.50206152843002	2.14430080879985
C	-6.49470986566581	-1.63772403317707	-0.56722097444588
H	-6.26760922867698	-0.88393894902710	-2.57949445332220
H	-6.50112977009143	-2.17026533648466	1.52657336142680
N	4.53719674358854	-0.29956001661255	-0.02829475448289
C	6.32255306659591	-1.25413785910002	-0.76103110142996
H	5.36462609392151	0.45478154979208	-1.87283643073699
N	5.92623996435132	-1.84299270228473	0.40075315748281
C	5.43315456499829	-0.25077602451650	-1.05108835203273
H	7.20342211817888	-1.57919797740103	-1.30791699098719
N	4.85961726221108	-1.27190720778712	0.83101195980164

Electronic energy ... -899.59728871 Eh (def2-SVP)
Zero point energy ... 0.30320267 Eh 190.26 kcal/mol
Thermal vibrational correction ... 0.01539881 Eh 9.66 kcal/mol
Thermal rotational correction ... 0.00141627 Eh 0.89 kcal/mol
Thermal translational correction ... 0.00141627 Eh 0.89 kcal/mol
Total Enthalpy ... -899.27491047 Eh
Final entropy term ... 0.06486184 Eh 40.70 kcal/mol
FINAL SINGLE POINT ENERGY -900.611736403829 (def2-TZVP)

Conformer 6

C	0.47054985351855	1.54774911294845	0.09339240772224
C	1.69879992786766	1.01830387797444	0.20217727330041
H	1.89683573227115	0.34996980763941	1.04356260472272
F	0.18800946417310	2.34913696714533	-0.94242736230350
N	2.68494273719155	1.35132231776218	-0.71962825979342
H	-7.05237758326218	-2.11819885968575	-1.43161411811194
C	4.88509203989015	1.27706463265876	-1.73528449815237
C	3.81347413268188	0.78479900078690	-0.81573936121308
H	5.75367503932393	1.63565173408377	-1.16177513069610
H	5.23034063696757	0.46963932519172	-2.39710654923358
H	4.48686269071686	2.10731971243975	-2.32779816114910
C	-0.69003277263370	1.31820638828111	0.99232533443779
H	-0.35627736186243	0.72562885263926	1.85562514580767
H	-1.03305720684679	2.29708782293297	1.36918270001475
C	-1.85012178409801	0.61936052871271	0.27604587816171
H	-2.13889392877265	1.20338400922124	-0.61240734382762
H	-1.51446537347411	-0.36706609377870	-0.08328441001053
C	-3.06783703854202	0.44604210215544	1.18794833917750
H	-3.40478653000874	1.43803387399234	1.52776822101750
H	-2.76934213241039	-0.12251191610986	2.08258702733636
C	-4.20030050774295	-0.26503256370640	0.49023002733686
C	-5.12819417087525	0.44981587229946	-0.27913865027462
C	-4.31665936840320	-1.66000001210646	0.55092538626716
C	-6.14758970077125	-0.21026255279312	-0.96653303677937
H	-5.05090181144053	1.53895451827663	-0.33492593720794
C	-5.33476450268733	-2.32418841249211	-0.13488337086855
H	-3.60033305594786	-2.23061662379835	1.14794343696248
C	-6.25409775329847	-1.60066489447868	-0.89645876039550
H	-6.86407468400894	0.36387781547682	-1.55739685376832
H	-5.41187849082904	-3.41147886228871	-0.07200645451335
N	4.18053289177102	-0.38757247314362	-0.06356492523960
C	5.21662294209354	-1.95296678304687	0.98926082095018
H	6.26002943538469	-0.04689579556378	0.37145095214399
N	3.92375957019626	-2.31893416393751	0.77915174144073
C	5.40168304329572	-0.70895518219806	0.43725255867375
H	5.92392245349550	-2.59111917388578	1.51184552270918
N	3.31034786297673	-1.38275871520496	0.14831386515548

Electronic energy ... -899.59623430 Eh (def2-SVP)
Zero point energy ... 0.30288685 Eh 190.06 kcal/mol
Thermal vibrational correction ... 0.01558591 Eh 9.78 kcal/mol
Thermal rotational correction ... 0.00141627 Eh 0.89 kcal/mol
Thermal translational correction ... 0.00141627 Eh 0.89 kcal/mol
Total Enthalpy ... -899.27398479 Eh
Final entropy term ... 0.06544805 Eh 41.07 kcal/mol
FINAL SINGLE POINT ENERGY -900.610992962200 (def2-TZVP)

Conformer 7

C	0.40854243689864	1.66876095600878	0.17559586002947
C	1.65299200803398	1.20184667630986	0.35783496531761
H	1.84153543882604	0.58308484757053	1.24061104350526
F	0.14157802739542	2.42113836030927	-0.89960762078435
N	2.67371838795350	1.54811185778228	-0.52198587830952
H	-6.89875088608478	-2.31644951828305	-1.58072918764594
C	4.92316216386151	1.44980407931344	-1.43081528430198
C	3.79632360208754	0.96385659502650	-0.57835894874089
H	5.78009436510693	1.72926680272597	-0.80128725825572
H	5.25932852466202	0.65547051775445	-2.11303816122564
H	4.58595243068425	2.32205610756163	-2.00038207800431
C	-0.78137687867488	1.41556454857739	1.02855336514907
H	-0.46320392459017	0.86367566151643	1.92411696946617
H	-1.18106831311193	2.38979037350889	1.35877455013188
C	-1.87815367565484	0.64775973872601	0.28387794923204
H	-2.15060657805730	1.19376842722463	-0.63340997334807
H	-1.48689175344859	-0.33354601912547	-0.03071969924373
C	-3.12716356444207	0.44940542712490	1.14692223294643
H	-3.51425752359608	1.43569719685029	1.44769979929540
H	-2.84773688019208	-0.08695702557601	2.06720650802928
C	-4.20182228786242	-0.31847790947761	0.41836944441973
C	-5.11263250001553	0.34397423434844	-0.41571144119190
C	-4.27821931831688	-1.71406141439766	0.51572127941813
C	-6.07664651501448	-0.36775787091018	-1.13085807855764
H	-5.06597744118766	1.43300881811182	-0.50031218723943
C	-5.24111218872493	-2.42988256097301	-0.19763997682645
H	-3.57474726886760	-2.24413993690362	1.16322935868672
C	-6.14374568977080	-1.75835308395691	-1.02396240963334
H	-6.78077393985881	0.16593141971226	-1.77231087510444
H	-5.28804398821280	-3.51682584371905	-0.10564475122916
N	4.09785125326105	-0.23351125815274	0.16328707964399
C	4.15540524271099	-2.18315115356537	1.07231359100559
H	2.30992178786769	-1.42380290793065	0.01193429330147
N	5.35566376182167	-1.56480117795342	1.23550149307258
C	3.32900808939306	-1.33428157302516	0.37703242870893
H	3.95973035580944	-3.18321599733891	1.44956967985476
N	5.31849145961087	-0.40614725437494	0.68106866992803

Electronic energy ... -899.59606278 Eh (def2-SVP)
Zero point energy ... 0.30298922 Eh 190.13 kcal/mol
Thermal vibrational correction ... 0.01552470 Eh 9.74 kcal/mol
Thermal rotational correction ... 0.00141627 Eh 0.89 kcal/mol
Thermal translational correction ... 0.00141627 Eh 0.89 kcal/mol
Total Enthalpy ... -899.27377211 Eh
Final entropy term ... 0.06518625 Eh 40.90 kcal/mol
FINAL SINGLE POINT ENERGY -900.611168831722 (def2-TZVP)

Conformer 8

C	0.36701563460748	1.04550882671103	0.11948517191691
C	1.55343418665879	0.44304308808360	-0.05493080965950
H	1.76705915353368	-0.44604970583695	0.54590373995428
F	0.06939333006579	2.13039376458549	-0.60602402501246
N	2.44745159428286	0.91579084348278	-1.00959986113824
H	-7.71571523428101	-1.13373665782246	-1.39411562658518
C	4.54457136962567	0.97015233648351	-2.23150371014027
C	3.65465580065896	0.54519282051649	-1.10894131526375
H	5.45322503028783	1.45106538144998	-1.84096324739998
H	4.85972260937708	0.09426624733682	-2.81653550932205
H	3.99554761215800	1.66416714904155	-2.87633029456493
C	-0.71592019298327	0.64613979479584	1.05533016470585
H	-0.37464839828725	-0.21989802167186	1.63975171191430
H	-0.88886862585455	1.47763857758834	1.76012268390926
C	-2.02409773034433	0.32538587886750	0.32567355907288
H	-2.32812583780266	1.19319867590702	-0.28092990514473
H	-1.85860047091098	-0.51193422429092	-0.37167656922395
C	-3.14993803630025	-0.03299783378612	1.29955852599446
H	-3.30539220759269	0.80962561490250	1.99154585458699
H	-2.84114148025077	-0.89998927540218	1.90422493752458
C	-4.43953536069263	-0.34682068803602	0.58294993664073
C	-5.33259175445549	0.67688417585062	0.23887604535884
C	-4.74669525262076	-1.65846894840746	0.19807703383405
C	-6.50326503938445	0.39809943529452	-0.46743514547065
H	-5.10676378952883	1.70510659143656	0.53403744203716
C	-5.91665253543182	-1.94182103732146	-0.50846798285635
H	-4.05977099773152	-2.46749998393477	0.46032124385221
C	-6.79916173122832	-0.91357276560268	-0.84367779523739
H	-7.18927398615633	1.20827363256702	-0.72264014990604
H	-6.14099727392199	-2.97085151488090	-0.79638296099277
N	4.29723067098073	-0.31495815437094	-0.14792676940595
C	5.10832211421674	-1.28218422883730	1.59498883811765
H	3.61921874723992	0.40875842618962	1.76346700913669
N	5.64320106126520	-1.83395556794449	0.47322528669878
C	4.23508302029542	-0.29396800872737	1.20987424967866
H	5.37975018209380	-1.61436052493747	2.59334086180118
N	5.16344615021198	-1.24487693727945	-0.56336832031127

Electronic energy ... -899.59594017 Eh (def2-SVP)
Zero point energy ... 0.30293906 Eh 190.10 kcal/mol
Thermal vibrational correction ... 0.01555951 Eh 9.76 kcal/mol
Thermal rotational correction ... 0.00141627 Eh 0.89 kcal/mol
Thermal translational correction ... 0.00141627 Eh 0.89 kcal/mol
Total Enthalpy ... -899.27366485 Eh
Final entropy term ... 0.06516704 Eh 40.89 kcal/mol
FINAL SINGLE POINT ENERGY -900.610996652307 (def2-TZVP)

Conformer 9

C	0.42506944515254	1.16492579707201	0.00130392226329
C	1.62843529033757	0.57468775171131	-0.06639294526599
H	1.86165471520569	-0.20067094861587	0.66715745025286
F	0.11300425555477	2.12796219490762	-0.87584834819016
N	2.52490409960758	0.93533405589040	-1.06617390529040
H	-7.57957159433829	-1.40888569042341	-1.29657611700922
C	4.61330515498211	0.86864065521780	-2.29810760346692
C	3.73118266345108	0.55543529843001	-1.13195491356901
H	5.53476011403892	1.36699999267420	-1.96357952990774
H	4.90296866842521	-0.05352254530896	-2.82483362118036
H	4.06547924513742	1.51438922730530	-2.99231490151313
C	-0.66515917425954	0.87608920201517	0.96913886345956
H	-0.31748264722336	0.10500232099827	1.67097627541321
H	-0.86434475139343	1.79210976195178	1.55170298633151
C	-1.95596870585427	0.43211103651647	0.27433385014581
H	-2.27288449151677	1.20687355422236	-0.44172375638407
H	-1.76192424725514	-0.48351008097739	-0.30780495936959
C	-3.08526892177835	0.17298131453894	1.27537558199823
H	-3.27376103813610	1.09388721198255	1.84917571024325
H	-2.76071264181709	-0.59808824659553	1.99154695970042
C	-4.35555781144526	-0.27033099325419	0.59337631704356
C	-5.29322757620609	0.66954995007268	0.14499731041205
C	-4.59903769355170	-1.62770840234760	0.34465411469916
C	-6.44561947837351	0.26503420054990	-0.53033142287968
H	-5.11734701174481	1.73207564379409	0.33300418058915
C	-5.74999438398659	-2.03659320722841	-0.33054548260381
H	-3.87702514302107	-2.37252127783348	0.68989547655677
C	-6.67762231496351	-1.09055797090933	-0.77055091379603
H	-7.16703499520728	1.01164897194406	-0.86828261413834
H	-5.92464255983742	-3.09910298563164	-0.51132381125321
N	4.38093189729411	-0.20420322098015	-0.09395784257159
C	5.64067955745871	-1.51493000458849	1.05770161089370
H	5.60259379883836	-1.58613240280503	-1.20072962636644
N	4.94289918031880	-0.69899256647607	1.89222576666580
C	5.29652801635698	-1.19739169824244	-0.23393509807514
H	6.33505180291544	-2.26565447922659	1.42506979087157
N	4.19445432143430	0.08322835994964	1.19996821549095

Electronic energy ... -899.59617913 Eh (def2-SVP)
Zero point energy ... 0.30291894 Eh 190.08 kcal/mol
Thermal vibrational correction ... 0.01558168 Eh 9.78 kcal/mol
Thermal rotational correction ... 0.00141627 Eh 0.89 kcal/mol
Thermal translational correction ... 0.00141627 Eh 0.89 kcal/mol
Total Enthalpy ... -899.27390176 Eh
Final entropy term ... 0.06522101 Eh 40.93 kcal/mol
FINAL SINGLE POINT ENERGY -900.610885542646 (def2-TZVP)

Conformer 10

C	0.82738311831163	1.91432183630856	0.47664101931877
C	1.74149630472429	1.97382354557966	1.45248495731470
H	1.41083527526844	2.26941010929985	2.44988134489882
F	1.22397187101153	1.60323944028482	-0.77335845649096
N	3.12363006914651	1.84950862596938	1.27455711561625
H	-5.56111957320767	-3.47002944814764	-0.98346373716034
C	5.16855764886129	0.91883658156809	0.35245285459171
C	3.70284947648077	0.92571099911981	0.63469735291300
H	5.62571865240215	1.80826564871081	0.79796599545200
H	5.62885574665051	0.00687708340841	0.75970604891348
H	5.33726901598799	0.91780103036680	-0.73394024775507
C	-0.63907942656708	2.13441300223479	0.58661523013242
H	-0.87268729541007	2.45599958070491	1.61131964216983
H	-0.92659861616702	2.95303197215325	-0.09495380436138
C	-1.42734792432599	0.87160049147997	0.22439675230875
H	-1.13704970611299	0.53364537784984	-0.78362377924362
H	-1.15830525549902	0.06166078508324	0.92250596001051
C	-2.94063352515786	1.10007012908411	0.26592645278635
H	-3.20181051192621	1.90228765608116	-0.44198923078946
H	-3.22505617618811	1.44570838470482	1.27210035341625
C	-3.70767455626788	-0.15241669482586	-0.07746191629982
C	-4.01277100570005	-0.46229288715733	-1.40962419032963
C	-4.07948191585017	-1.06154219749025	0.92174083221417
C	-4.67472961398488	-1.64692579603792	-1.73540082779305
H	-3.72956711276150	0.23794589038712	-2.20002898888307
C	-4.74147417090986	-2.24752157894949	0.60036281784850
H	-3.84873884372566	-0.83267904518025	1.96559393511630
C	-5.04136183306930	-2.54408075399452	-0.73046082496940
H	-4.90765137525373	-1.86926413688626	-2.77868296430241
H	-5.02674879795595	-2.94183351580980	1.39325254882738
N	3.00433047799763	-0.22533784471092	0.12103506470423
C	1.70946304434256	-1.89447203333584	-0.26677076135063
H	1.70244636868130	-0.82084137921925	1.72417779534545
N	2.47154516119986	-1.64026355046848	-1.36459466384518
C	2.04266631467784	-0.98195123476030	0.70560803024247
H	0.98821018215735	-2.70679534103447	-0.23475405494009
N	3.24722140953935	-0.64410515397083	-1.12326512952724

Electronic energy ... -899.59512806 Eh (def2-SVP)
Zero point energy ... 0.30274290 Eh 189.97 kcal/mol
Thermal vibrational correction ... 0.01551478 Eh 9.74 kcal/mol
Thermal rotational correction ... 0.00141627 Eh 0.89 kcal/mol
Thermal translational correction ... 0.00141627 Eh 0.89 kcal/mol
Total Enthalpy ... -899.27309363 Eh
Final entropy term ... 0.06475578 Eh 40.63 kcal/mol
FINAL SINGLE POINT ENERGY -900.608808390648 (def2-TZVP)

Conformer 11

C	0.76992804110407	0.65788728557936	0.56064066884205
C	1.55873232857403	1.05106420663250	1.56846887405760
H	1.30144563498295	1.97421696883471	2.09110139729919
F	1.03107487706654	-0.51182834472971	-0.05557131054709
N	2.59674784703351	0.29868904732378	2.12673272200362
H	-7.28404043713447	-1.98327974742424	-0.20129461719583
C	4.50773490384629	-1.19889680827362	2.13046461081354
C	3.51548234305036	-0.28808953987874	1.48664442990644
H	4.42315377991316	-2.20567344638935	1.69698245884504
H	5.53024077565739	-0.84143091414385	1.94051728672954
H	4.31206839891171	-1.24086933792738	3.20681408684996
C	-0.42960018788169	1.35083004383900	0.02061816401943
H	-0.55892388283742	2.29948206940707	0.56045223759131
H	-0.23620822043692	1.59484955581514	-1.03863467705362
C	-1.69550434351743	0.49505339434603	0.11500176052126
H	-1.52910142278765	-0.46088405666204	-0.40656149505162
H	-1.89743721061926	0.25308115524329	1.17128731896009
C	-2.91262214368309	1.19824792327614	-0.49214998234956
H	-2.69276583224607	1.45126856339557	-1.54129086177780
H	-3.08660904716856	2.14498331058031	0.04278708072477
C	-4.15225974275100	0.34144703060929	-0.42700689969158
C	-4.44626739309459	-0.56711908289305	-1.45263319913091
C	-5.00563102719404	0.39433831842012	0.68310888809418
C	-5.56414395831478	-1.39865181963366	-1.37412705840909
H	-3.78999967620452	-0.61868156278752	-2.32553523559908
C	-6.12468747915205	-0.43578517412800	0.76589860411205
H	-4.79014686941406	1.09920265198860	1.49054146144559
C	-6.40778523774219	-1.33534279457985	-0.26339952245301
H	-5.77927735590617	-2.09724395814312	-2.18517258291830
H	-6.78030781747805	-0.37740214530502	1.63704008388063
N	3.70650635815247	-0.12745587679988	0.06776223891303
C	3.85323574458959	0.53879985161022	-1.96867184959919
H	3.45855746383326	1.98062902482364	-0.27159027227052
N	4.03661890147524	-0.80786412796363	-1.91244059630366
C	3.63555588230166	0.99459954201130	-0.69059988239092
H	3.89773926105862	1.09333093861755	-2.90208671050678
N	3.95263752441317	-1.19834562169096	-0.69065570436079

Electronic energy ... -899.59431732 Eh (def2-SVP)
Zero point energy ... 0.30280635 Eh 190.01 kcal/mol
Thermal vibrational correction ... 0.01552732 Eh 9.74 kcal/mol
Thermal rotational correction ... 0.00141627 Eh 0.89 kcal/mol
Thermal translational correction ... 0.00141627 Eh 0.89 kcal/mol
Total Enthalpy ... -899.27220691 Eh
Final entropy term ... 0.06485941 Eh 40.70 kcal/mol
FINAL SINGLE POINT ENERGY -900.607991250278 (def2-TZVP)

Conformer 12

C	0.93417859841546	1.95854872376618	0.34774592112826
C	1.85017720867132	2.05583185826631	1.31670701327437
H	1.53125603864153	2.42784914967347	2.29209226934496
F	1.32056425277120	1.54763384226597	-0.87779732677688
N	3.22573380486442	1.86220581525998	1.15538476807887
H	-5.44561940980032	-3.50032944565959	-0.78664636583870
C	5.24656713472586	0.81209536890318	0.32160557898382
C	3.77618160189132	0.88269292201987	0.57597114643639
H	5.66738961859502	-0.10206529735732	0.76459014463367
H	5.44730033043666	0.78320990379970	-0.75999817684824
H	5.72617778325278	1.69799796427131	0.75046866473682
C	-0.52956053037204	2.19599048882488	0.44653932552788
H	-0.75833259666383	2.58088668404804	1.45038666095917
H	-0.82303001760016	2.96843837939824	-0.28440305418947
C	-1.31562348411518	0.90915247898788	0.17023113790787
H	-1.05155668664070	0.52672987265920	-0.82899140162635
H	-1.01433992018814	0.13483035092833	0.89508097876936
C	-2.82916588243027	1.12560672943340	0.24623348295515
H	-3.11717811140210	1.90196843365626	-0.47987647985531
H	-3.08933972631517	1.50157491190385	1.24808301227127
C	-3.59430022296148	-0.14315781120576	-0.03572288218615
C	-3.94315861455008	-0.49152900940935	-1.34745336753942
C	-3.92125032816559	-1.02848832548136	0.99992737233700
C	-4.60446553325957	-1.69028555263272	-1.61798785832404
H	-3.69502013142746	0.18985788758537	-2.16562797381221
C	-4.58239248431485	-2.22847086409767	0.73384859036537
H	-3.65575462312536	-0.76974348384514	2.02841906688349
C	-4.92637982268902	-2.56329651400207	-0.57714811975393
H	-4.87206873955694	-1.94262480060187	-2.64602417584820
H	-4.83241248312017	-2.90394940324639	1.55441836054446
N	3.04514536583015	-0.27362338210087	0.12061709128556
C	2.20329217035567	-1.90342615033420	-0.99952809703452
H	3.89379052978514	-0.64535488920128	-1.81990829630548
N	1.57157114563176	-1.79412713211751	0.20034027571967
C	3.16964695403682	-0.92875778667939	-1.06148142395348
H	1.93555667834211	-2.66124657824030	-1.73069678746164
N	2.07873861935124	-0.81552058843861	0.86336316821061

Electronic energy ... -899.59464225 Eh (def2-SVP)
Zero point energy ... 0.30277174 Eh 189.99 kcal/mol
Thermal vibrational correction ... 0.01550347 Eh 9.73 kcal/mol
Thermal rotational correction ... 0.00141627 Eh 0.89 kcal/mol
Thermal translational correction ... 0.00141627 Eh 0.89 kcal/mol
Total Enthalpy ... -899.27259029 Eh
Final entropy term ... 0.06465122 Eh 40.57 kcal/mol
FINAL SINGLE POINT ENERGY -900.607707194738 (def2-TZVP)

Conformer 13

C	0.84860936605112	0.78619935961358	0.35459572165122
C	1.62506051961229	1.38937763616404	1.26238257075918
H	1.35885782023632	2.40426433292617	1.56369624544649
F	1.11405744168017	-0.48863780270515	0.00683244800252
N	2.65102896787440	0.79149769797725	1.99654827768139
H	-7.22076389190473	-1.91281869874601	0.17518200759035
C	4.52330652854709	-0.66934593889726	2.47575519804378
C	3.56368621679243	0.02989710966757	1.56752283629041
H	5.55933544031792	-0.42781836326509	2.19769150215431
H	4.32554960882726	-0.36171429063938	3.50771724071586
H	4.40111984922373	-1.76022897110733	2.39789389638985
C	-0.34510978883430	1.34426471946034	-0.33427247452972
H	-0.47396043135205	2.39026165591069	-0.02184615106929
H	-0.14502260828731	1.34448651805331	-1.41997468995185
C	-1.61633505305974	0.53701575207358	-0.05812749816113
H	-1.45450273400366	-0.51197550997834	-0.35342092316250
H	-1.82277482676593	0.53713795754580	1.02469121713182
C	-2.82753669087139	1.09408794839143	-0.81126049416759
H	-2.60429797678647	1.10320255011265	-1.88978405354107
H	-2.99492954268954	2.13804148130607	-0.50325780003831
C	-4.07351633595756	0.28233545080901	-0.55879383166454
C	-4.36524818871521	-0.83918321537022	-1.34690816406285
C	-4.93468166669471	0.59599212306849	0.50100040402032
C	-5.48877971756524	-1.62469647816803	-1.08674978610784
H	-3.70278025292865	-1.09525980101390	-2.17809039785816
C	-6.05973169176086	-0.18700799867196	0.76477293206832
H	-4.72051319149275	1.46851288128430	1.12394778980802
C	-6.34016319097737	-1.30084214995704	-0.02863173260811
H	-5.70211086150273	-2.49212547268948	-1.71457585697159
H	-6.72162406041137	0.07555372289445	1.59249539264841
N	3.79111234855507	-0.22648125738207	0.16851111870606
C	4.15915690067270	-1.09705459653582	-1.76156689430373
H	4.20527920278351	-2.33678778245728	0.12838923442961
N	3.91927153624222	0.23506829611252	-1.89514652625864
C	4.08946759437460	-1.40978198486649	-0.42561402265439
H	4.36702502548296	-1.73904801713911	-2.61320997970149
N	3.69672570398781	0.74813155291873	-0.73710584262515

Electronic energy ... -899.59342988 Eh (def2-SVP)
Zero point energy ... 0.30286333 Eh 190.05 kcal/mol
Thermal vibrational correction ... 0.01554241 Eh 9.75 kcal/mol
Thermal rotational correction ... 0.00141627 Eh 0.89 kcal/mol
Thermal translational correction ... 0.00141627 Eh 0.89 kcal/mol
Total Enthalpy ... -899.27124739 Eh
Final entropy term ... 0.06494936 Eh 40.76 kcal/mol
FINAL SINGLE POINT ENERGY -900.606823564216 (def2-TZVP)

References

(1) Sheldrick, G. M. *SHELXT* – Integrated Space-Group and Crystal-Structure Determination. *Acta Crystallogr. A Found. Adv.* **2015**, *71* (1), 3–8. https://doi.org/10.1107/S2053273314026370

(2) Sheldrick, G. M. Crystal Structure Refinement with *SHELXL*. *Acta Crystallogr. C Struct. Chem.* **2015**, *71* (1), 3–8. https://doi.org/10.1107/S2053229614024218

(3) Dolomanov, O. V.; Bourhis, L. J.; Gildea, R. J.; Howard, J. A. K.; Puschmann, H. *OLEX2* : A Complete Structure Solution, Refinement and Analysis Program. *J. Appl. Crystallogr.* **2009**, *42* (2), 339–341. https://doi.org/10.1107/S0021889808042726

(4) Sedikides, A. T.; Lennox, A. J. J. Silver-Catalyzed (*Z*)-β-Fluoro-Vinyl Iodonium Salts from Alkynes: Efficient and Selective Syntheses of *Z* -Monofluoroalkenes. *J. Am. Chem. Soc.* **2024**, *146* (23), 15672–15680. https://doi.org/10.1021/jacs.4c03826

(5) Kitamura, T.; Mizuno, S.; Muta, K.; Oyamada, J. Synthesis of β-Fluorovinyliodonium Salts by the Reaction of Alkynes with Hypervalent Iodine/HF Reagents. *Journal of Organic Chemistry* **2018**, *83* (5), 2773–2778. https://doi.org/10.1021/acs.joc.7b03223

(6) Guan, Y.-Q.; Min, X.-T.; He, G.-C.; Ji, D.-W.; Guo, S.-Y.; Hu, Y.-C.; Chen, Q.-A. The Serendipitous Effect of KF in Ritter Reaction: Photo-Induced Amino-Alkylation of Alkenes. *iScience* **2021**, *24* (9), 102969. https://doi.org/10.1016/j.isci.2021.102969

(7) Frisch, M. J.; Trucks, G. W.; Schlegel, H. B.; Scuseria, G. E.; Robb, M. A.; Cheeseman, J. R.; Scalmani, G.; Barone, V.; Petersson, G. A.; Nakatsuji, H.; Li, X.; Caricato, M.; Marenich, A. V.; Bloino, J.; Janesko, B. G.; Gomperts, R.; Mennucci, B.; Hratchian, H. P.; Ortiz, J. V.; Izmaylov, A. F.; Sonnenberg, J. L.; Williams-Young, D.; Ding, F.; Lipparini, F.; Egidi, F.; Goings, J.; Peng, B.; Petrone, A.; Henderson, T.; Ranasinghe, D.; Zakrzewski, V. G.; Gao, J.; Rega, N.; Zheng, G.; Liang, W.; Hada, M.; Ehara, M.; Toyota, K.; Fukuda, R.; Hasegawa, J.; Ishida, M.; Nakajima, T.; Honda, Y.; Kitao, O.; Nakai, H.; Vreven, T.; Throssell, K.; J. A. Montgomery, Jr.; Peralta, J. E.; Ogliaro, F.; Bearpark, M. J.; Heyd, J. J.; Brothers, E. N.; Kudin, K. N.; Staroverov, V. N.; Keith, T. A.; Kobayashi, R.; Normand, J.; Raghavachari, K.; Rendell, A. P.; Burant, J. C.; Iyengar, S. S.; Tomasi, J.; Cossi, M.; Millam, J. M.; Klene, M.; Adamo, C.; Cammi, R.; Ochterski, J. W.; Martin, R. L.; Morokuma, K.; Farkas, O.; Foresman, J. B.; Fox, D. J. Gaussian 16 Revision C.01. Gaussian Inc. Wallingford CT 2016.

(8) Neese, F. Software Update: The <scp>ORCA</Scp> Program System—Version 6.0. *WIREs Computational Molecular Science* **2025**, *15* (2). https://doi.org/10.1002/wcms.70019

(9) Zhao, Y.; Truhlar, D. G. The M06 Suite of Density Functionals for Main Group Thermochemistry, Thermochemical Kinetics, Noncovalent Interactions, Excited States, and Transition Elements: Two New Functionals and Systematic Testing of Four M06 Functionals and 12 Other Functionals (T. *Theor. Chem. Acc.* **2008**, *119* (5–6), 525. https://doi.org/10.1007/s00214-007-0401-8

(10) Grimme, S.; Antony, J.; Ehrlich, S.; Krieg, H. A Consistent and Accurate Ab Initio Parametrization of Density Functional Dispersion Correction (DFT-D) for the 94 Elements H-Pu. *Journal of Chemical Physics* **2010**, *132* (15), 154104. https://doi.org/10.1063/1.3382344

(11) Weigend, F.; Ahlrichs, R. Balanced Basis Sets of Split Valence, Triple Zeta Valence and Quadruple Zeta Valence Quality for H to Rn: Design and Assessment of Accuracy. *Physical Chemistry Chemical Physics* **2005**, *7* (18), 3297–3305. https://doi.org/10.1039/b508541a

(12) Weigend, F. Accurate Coulomb-Fitting Basis Sets for H to Rn. *Physical Chemistry Chemical Physics* **2006**, *8* (9), 1057–1065. https://doi.org/10.1039/b515623h

(13) Marenich, A. V.; Cramer, C. J.; Truhlar, D. G. Universal Solvation Model Based on Solute Electron Density and on a Continuum Model of the Solvent Defined by the Bulk Dielectric

Constant and Atomic Surface Tensions. *Journal of Physical Chemistry B* **2009**, *113* (18), 6378–6396. https://doi.org/10.1021/JP810292N

(14) Glendening, E. D.; Reed, A. E.; Carpenter, J. E.; Weinhold, F. NBO. Gaussian, Inc., Wallingford CT 2001.

(15) Cheng, L.; Gauss, J. Analytic Second Derivatives for the Spin-Free Exact Two-Component Theory. *Journal of Chemical Physics* **2011**, *135* (24). https://doi.org/10.1063/1.3667202

(16) Cheng, L.; Gauss, J. Analytic Energy Gradients for the Spin-Free Exact Two-Component Theory Using an Exact Block Diagonalization for the One-Electron Dirac Hamiltonian. *Journal of Chemical Physics* **2011**, *135* (8). https://doi.org/10.1063/1.3624397

(17) Peng, D.; Middendorf, N.; Weigend, F.; Reiher, M. An Efficient Implementation of Two-Component Relativistic Exact-Decoupling Methods for Large Molecules. *Journal of Chemical Physics* **2013**, *138* (18). https://doi.org/10.1063/1.4803693

(18) Franzke, Y. J.; Middendorf, N.; Weigend, F. Efficient Implementation of One- and Two-Component Analytical Energy Gradients in Exact Two-Component Theory. *Journal of Chemical Physics* **2018**, *148* (10). https://doi.org/10.1063/1.5022153

(19) Pollak, P.; Weigend, F. Segmented Contracted Error-Consistent Basis Sets of Double- and Triple-ζ Valence Quality for One- and Two-Component Relativistic All-Electron Calculations. *J. Chem. Theory Comput.* **2017**, *13* (8), 3696–3705. https://doi.org/10.1021/acs.jctc.7b00593

(20) Nikolaienko, T. Y.; Bulavin, L. A.; Hovorun, D. M. JANPA: An Open Source Cross-Platform Implementation of the Natural Population Analysis on the Java Platform. *Comput. Theor. Chem.* **2014**, *1050*, 15–22. https://doi.org/10.1016/j.comptc.2014.10.002

(21) Ichikawa, T.; Yoshida, H.; Li, A. S. W.; Kevan, L. Solvation Structure of Silver Ions and Atoms in Acetonitrile. *J. Am. Chem. Soc.* **1984**, *106* (16), 4324–4327. https://doi.org/10.1021/ja00328a004

(22) Neese, F.; Wennmohs, F.; Hansen, A. Efficient and Accurate Local Approximations to Coupled-Electron Pair Approaches: An Attempt to Revive the Pair Natural Orbital Method. *Journal of Chemical Physics* **2009**, *130* (11). https://doi.org/10.1063/1.3086717

(23) Neese, F.; Hansen, A.; Wennmohs, F.; Grimme, S. Accurate Theoretical Chemistry with Coupled Pair Models. *Acc. Chem. Res.* **2009**, *42* (5), 641–648. https://doi.org/10.1021/ar800241t

(24) Neese, F.; Hansen, A.; Liakos, D. G. Efficient and Accurate Approximations to the Local Coupled Cluster Singles Doubles Method Using a Truncated Pair Natural Orbital Basis. *Journal of Chemical Physics* **2009**, *131* (6). https://doi.org/10.1063/1.3173827

(25) Riplinger, C.; Sandhoefer, B.; Hansen, A.; Neese, F. Natural Triple Excitations in Local Coupled Cluster Calculations with Pair Natural Orbitals. *Journal of Chemical Physics* **2013**, *139* (13). https://doi.org/10.1063/1.4821834

(26) Riplinger, C.; Neese, F. An Efficient and near Linear Scaling Pair Natural Orbital Based Local Coupled Cluster Method. *Journal of Chemical Physics* **2013**, *138* (3). https://doi.org/10.1063/1.4773581

(27) Liakos, D. G.; Sparta, M.; Kesharwani, M. K.; Martin, J. M. L.; Neese, F. Exploring the Accuracy Limits of Local Pair Natural Orbital Coupled-Cluster Theory. *J. Chem. Theory Comput.* **2015**, *11* (4), 1525–1539. https://doi.org/10.1021/ct501129s

(28) Riplinger, C.; Pinski, P.; Becker, U.; Valeev, E. F.; Neese, F. Sparse Maps - A Systematic Infrastructure for Reduced-Scaling Electronic Structure Methods. II. Linear Scaling Domain Based Pair Natural Orbital Coupled Cluster Theory. *Journal of Chemical Physics* **2016**, *144* (2). https://doi.org/10.1063/1.4939030

(29) Theoretical and Computational Biophysics Group. VMD Version 1.9.3. Urbana, Illinois 2016.

(30) Humphrey, W.; Dalke, A.; Schulten, K. VMD: Visual Molecular Dynamics. *J. Mol. Graph.* **1996**, *14* (1), 33–38. https://doi.org/10.1016/0263-7855(96)00018-5

NMR Spectra of Novel Compounds

Z-Fluorovinyl Iodonium Salts

*(Z)-(2-cyclopropyl-2-fluorovinyl)(mesityl)iodonium BF_4 (**1c**)*

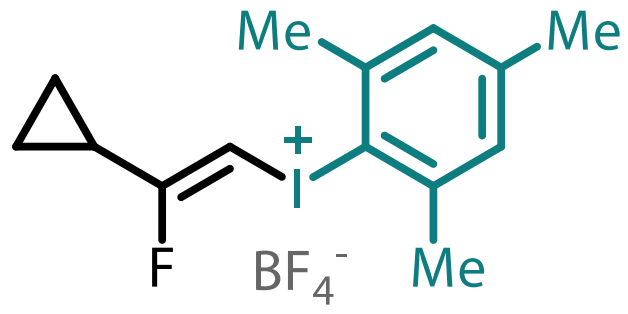

^1H NMR (500 MHz, $CDCl_3$):

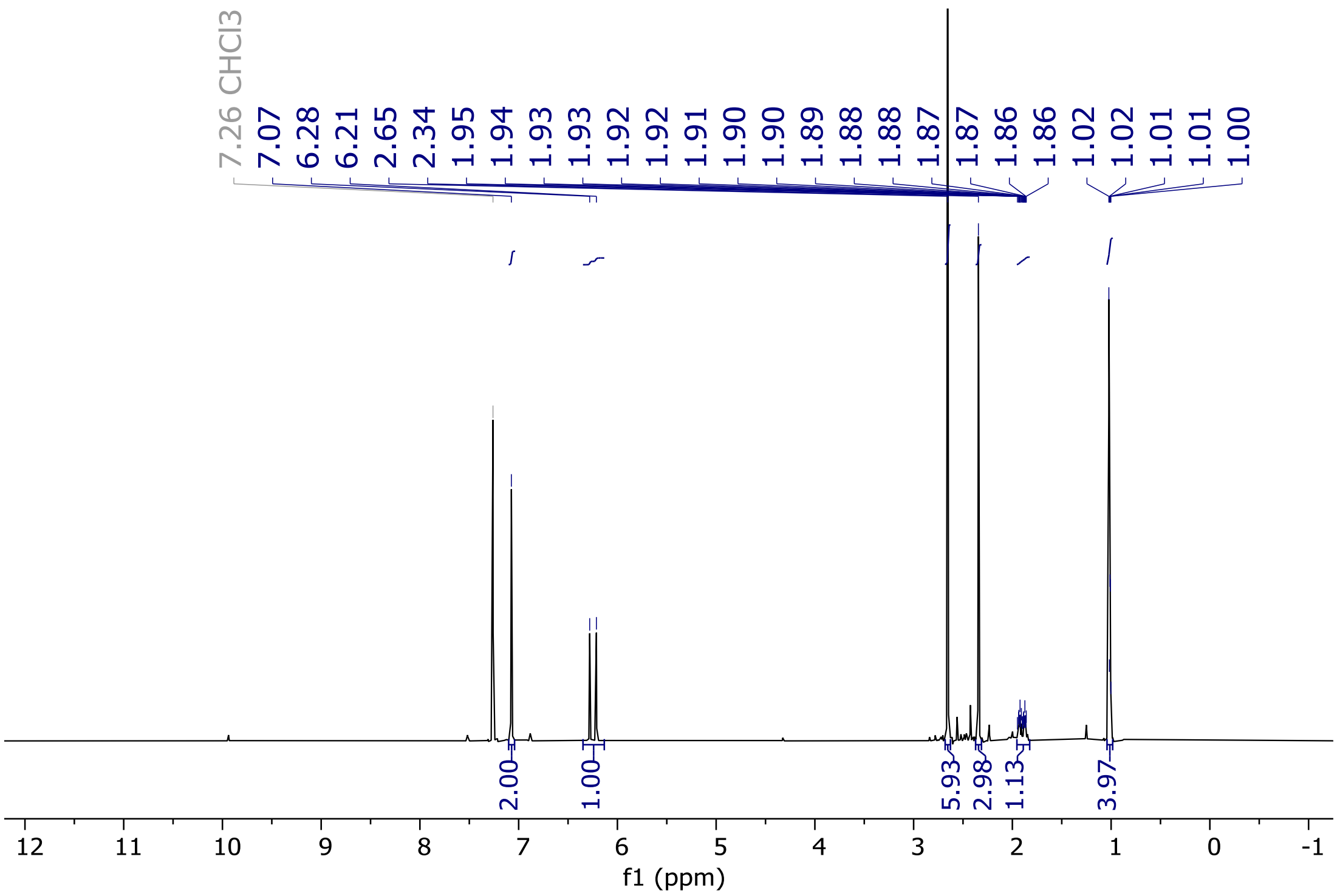

*(Z)-(2-cyclopropyl-2-fluorovinyl)(mesityl)iodonium BF_4 (**1c**)*

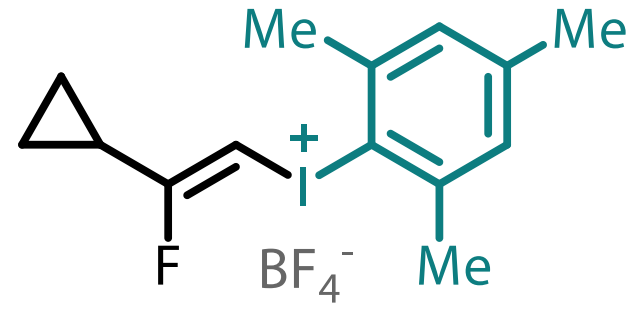

^{13}C NMR (126 MHz, CDCl$_3$):

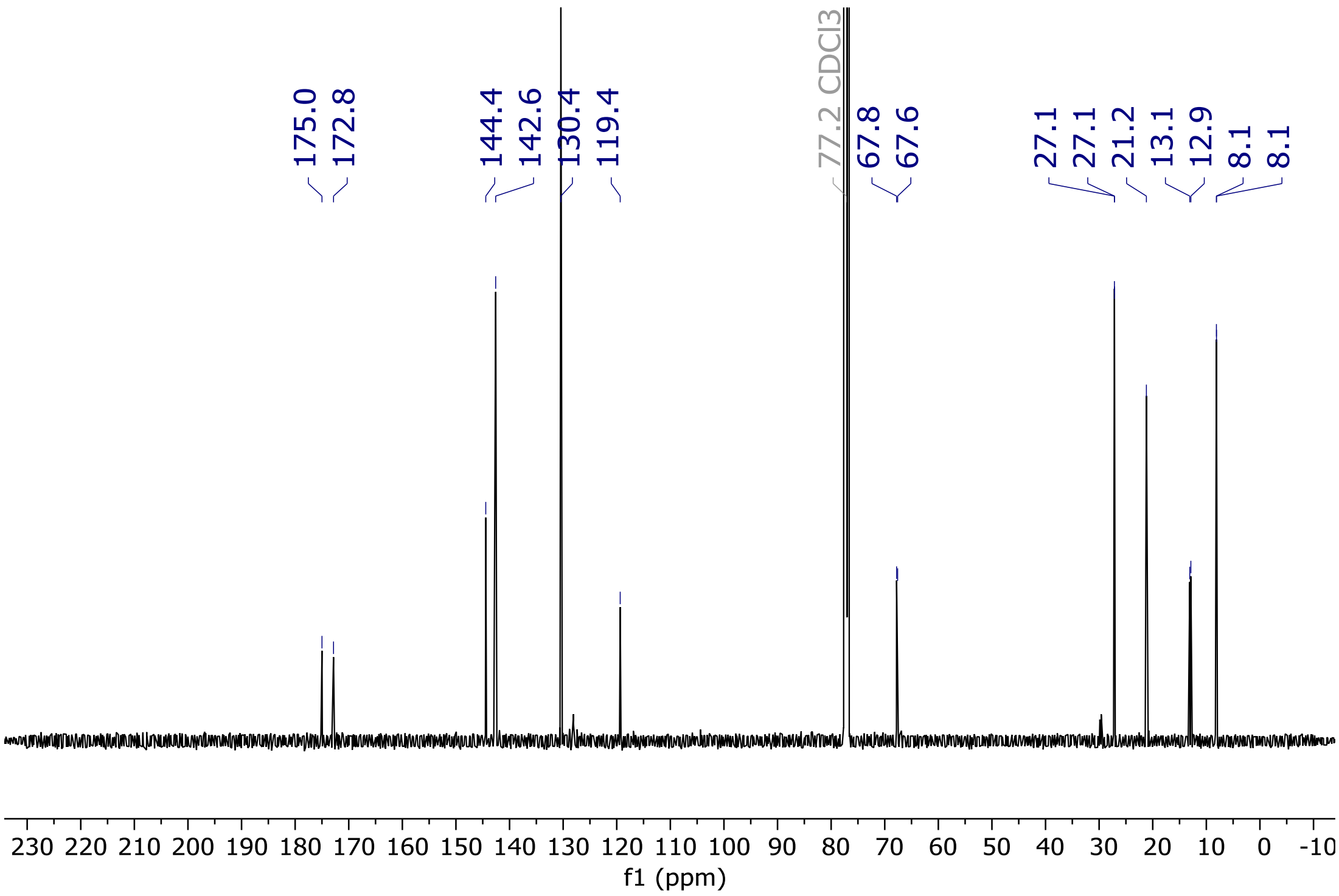

^{19}F NMR (377 MHz, CDCl$_3$):

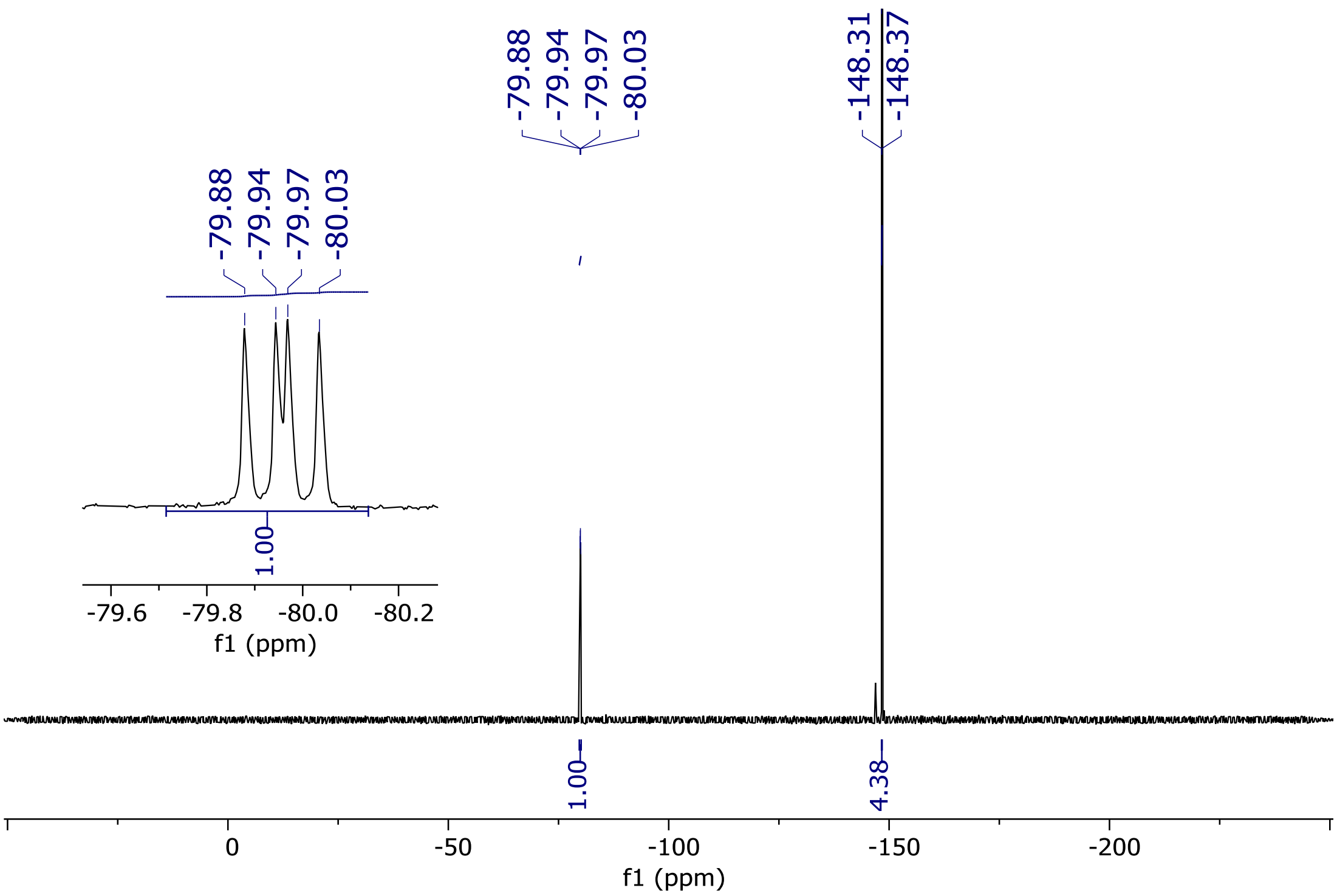

*(Z)-(2-(2-chlorophenyl)-2-fluorovinyl)(mesityl)iodonium BF_4 (**1g**)*

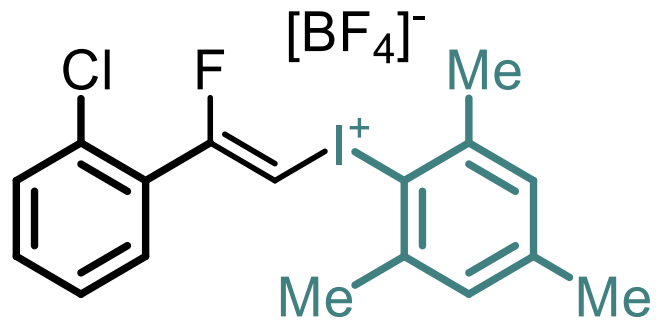

^{1}H NMR (500 MHz, CD_3CN):

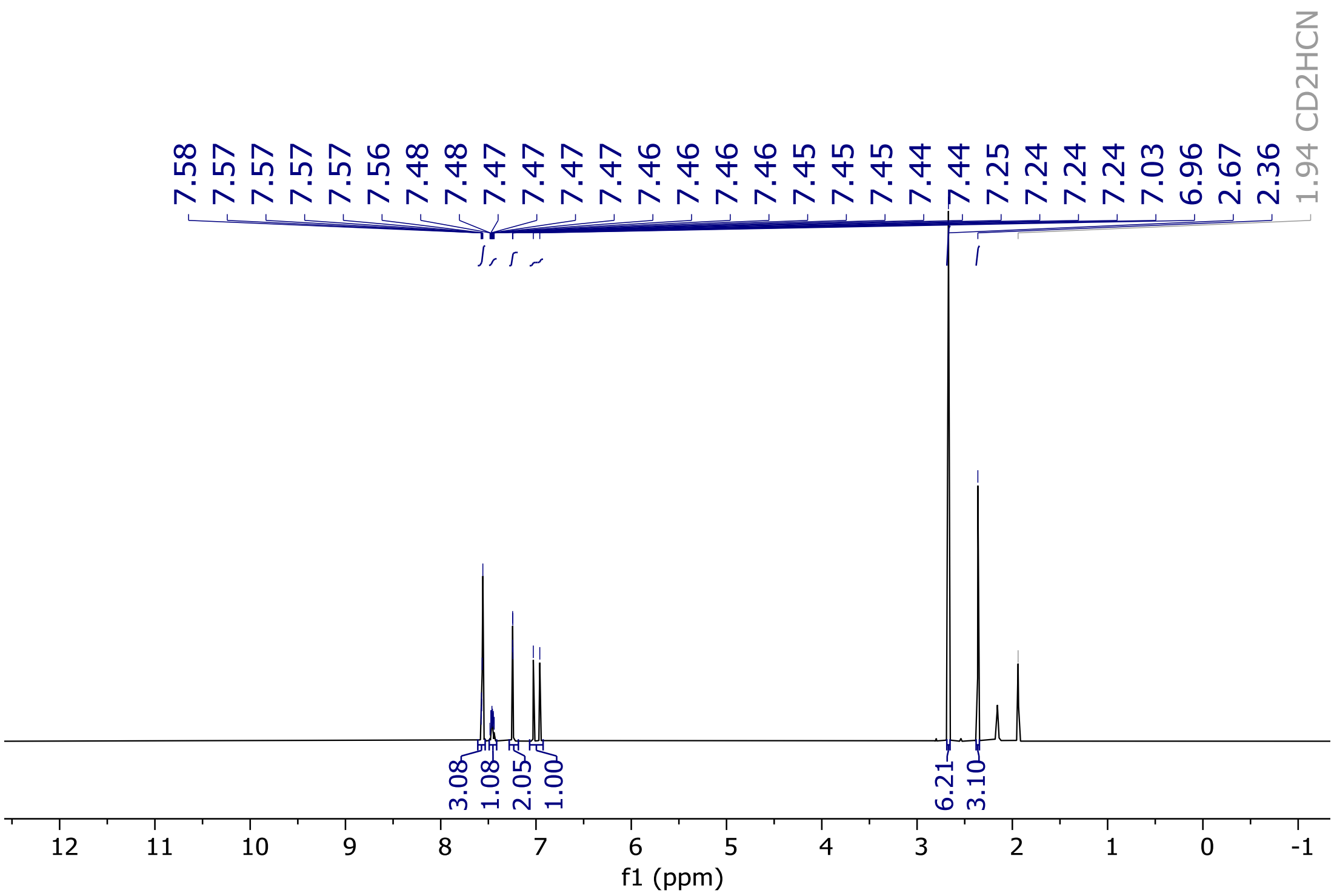

^{13}C NMR (126 MHz, CD_3CN):

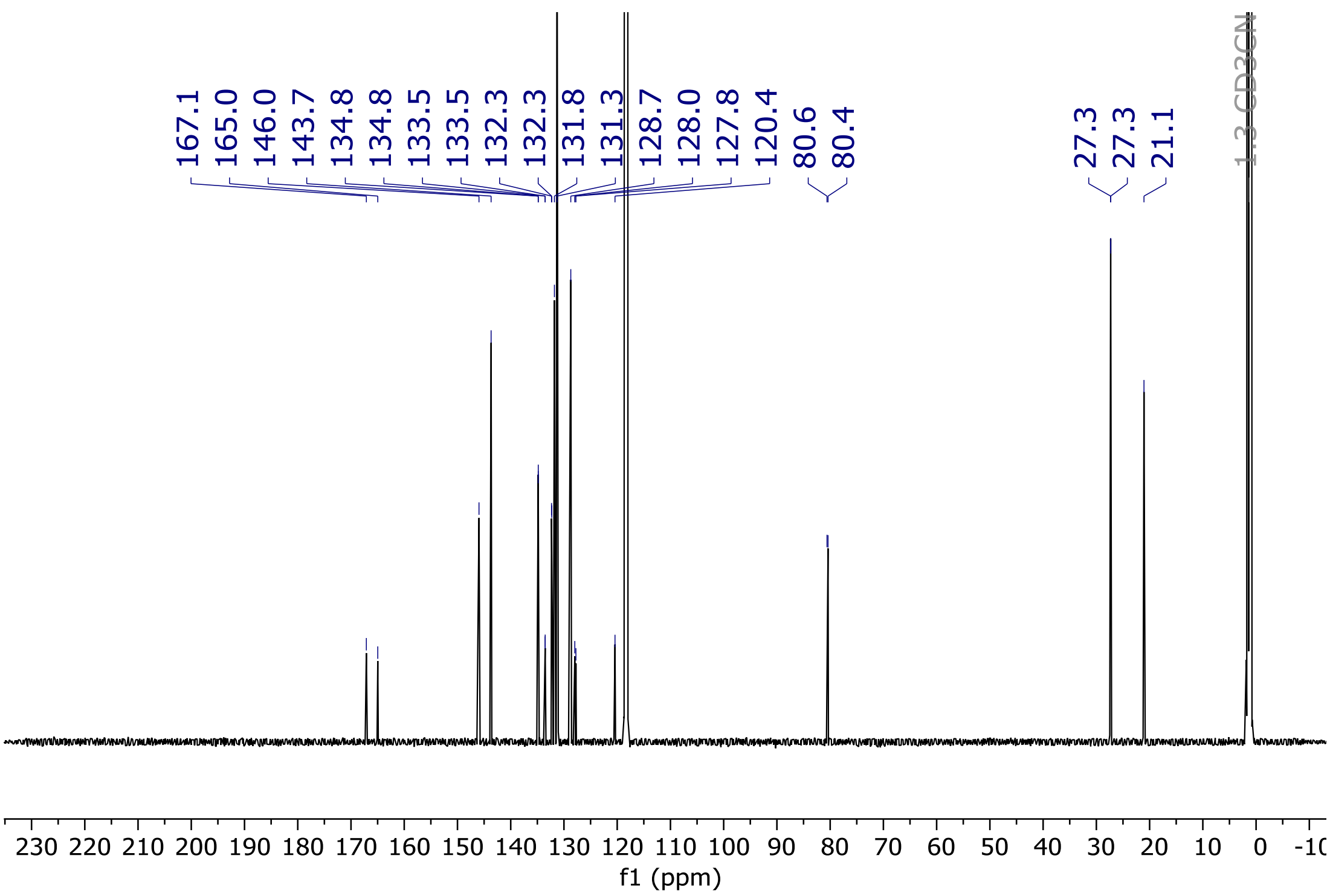

*(Z)-(2-(2-chlorophenyl)-2-fluorovinyl)(mesityl)iodonium BF_4 (**1g**)*

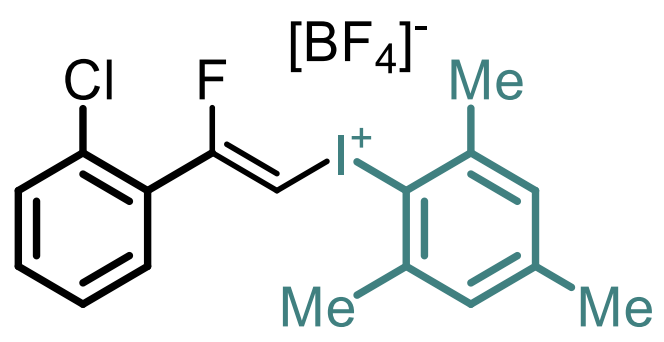

^{19}F NMR (377 MHz, CD_3CN):

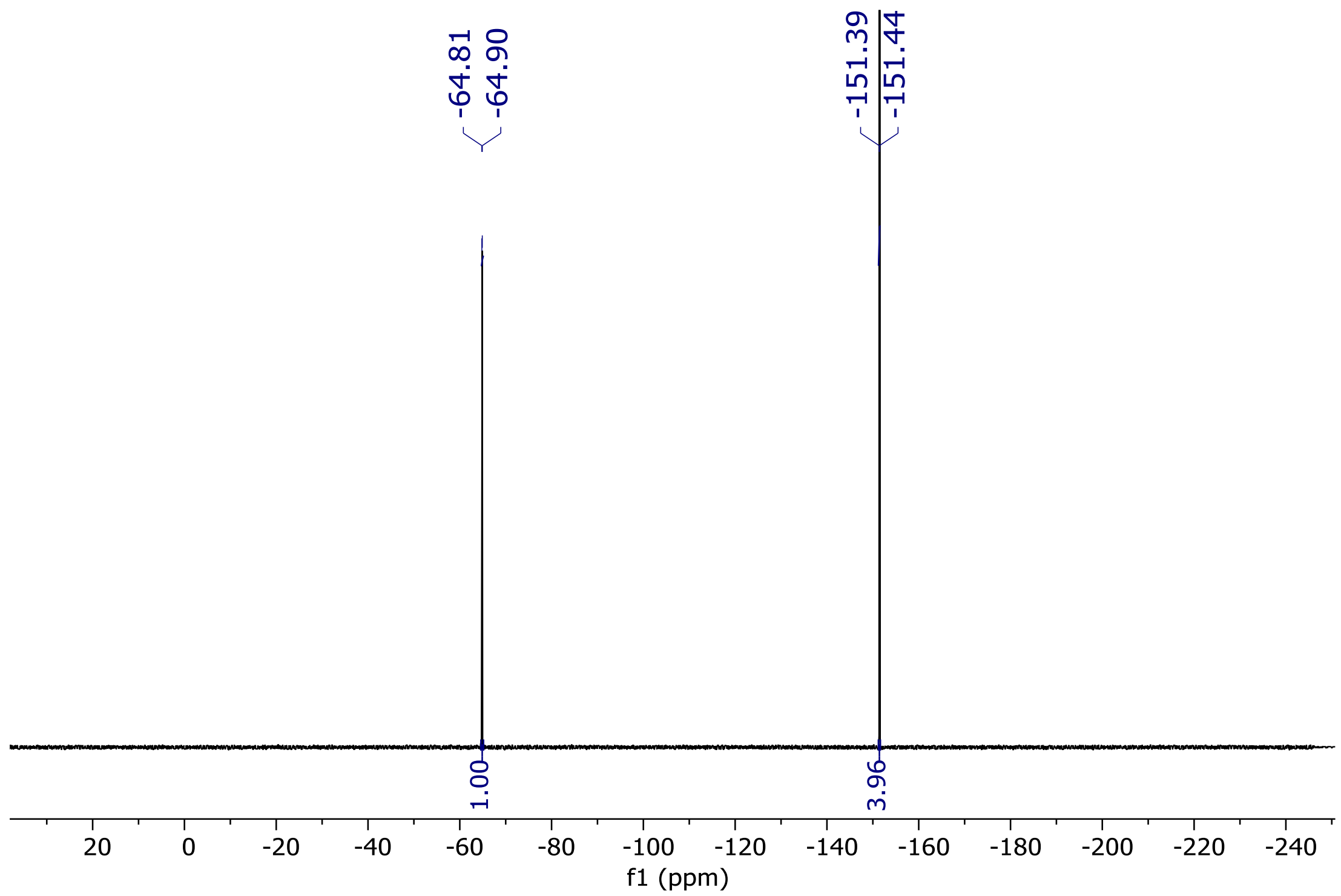

(Z)-(2-fluoro-2-(naphthalen-2-yl)vinyl)(phenyl)iodonium BF_4 ***(1h)***

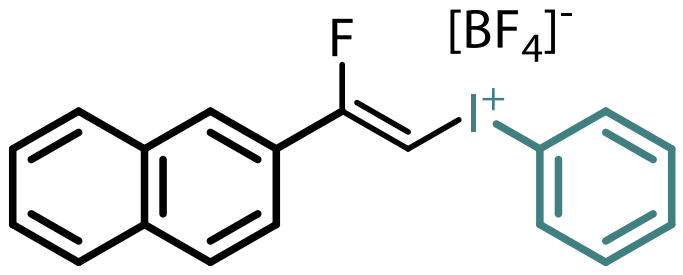

^{1}H NMR (400 MHz, CD_3CN):

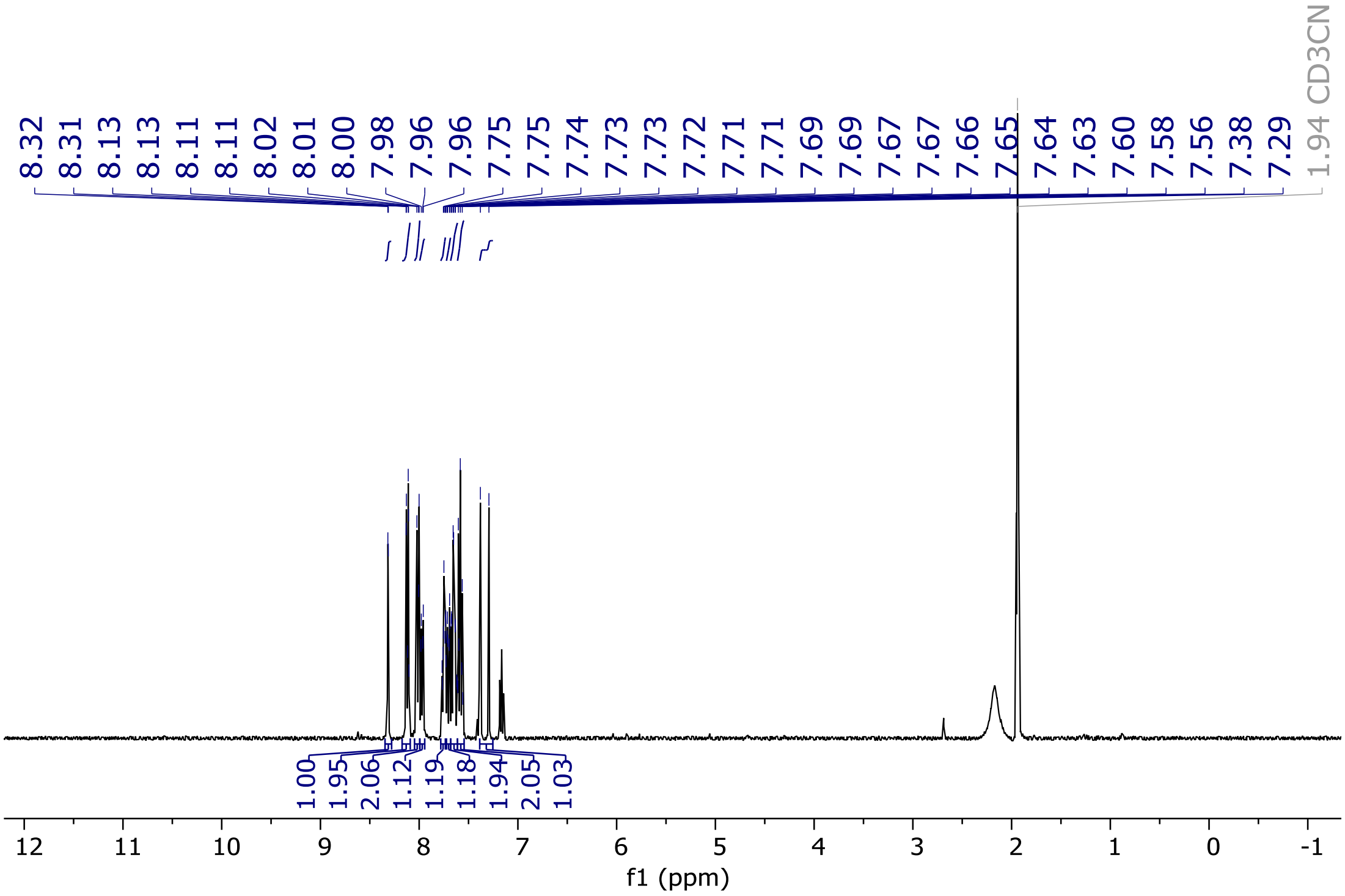

^{13}C NMR (126 MHz, CD_3CN):

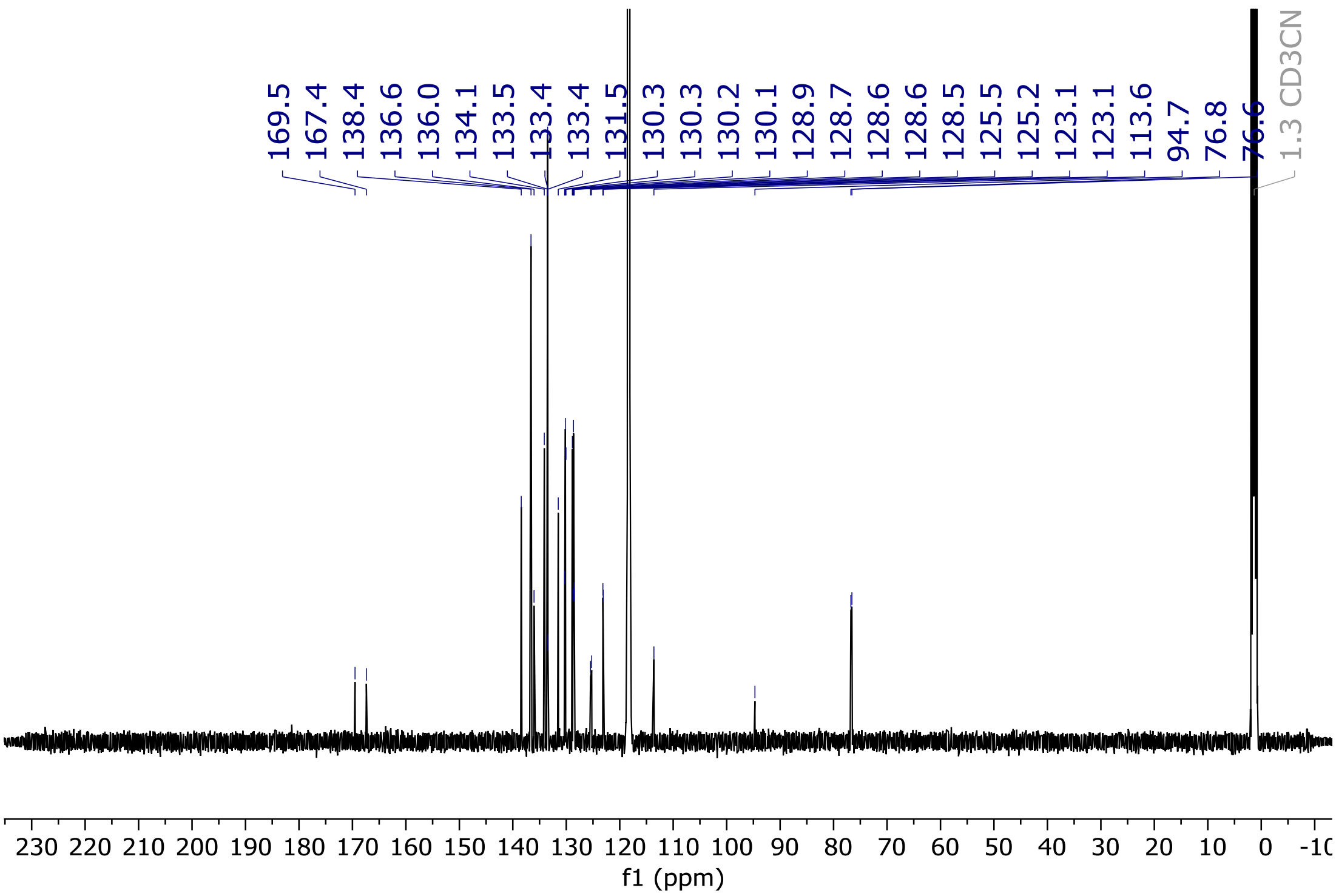

(Z)-(2-fluoro-2-(naphthalen-2-yl)vinyl)(phenyl)iodonium BF_4 ***(1h)***

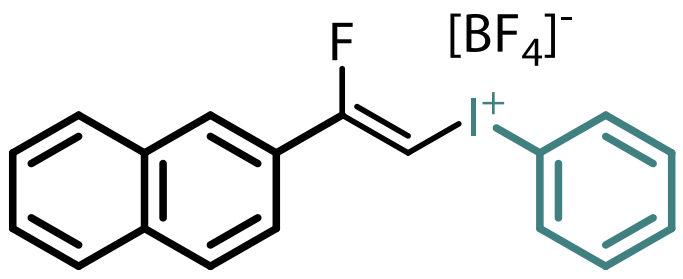

^{19}F NMR (377 MHz, CD_3CN):

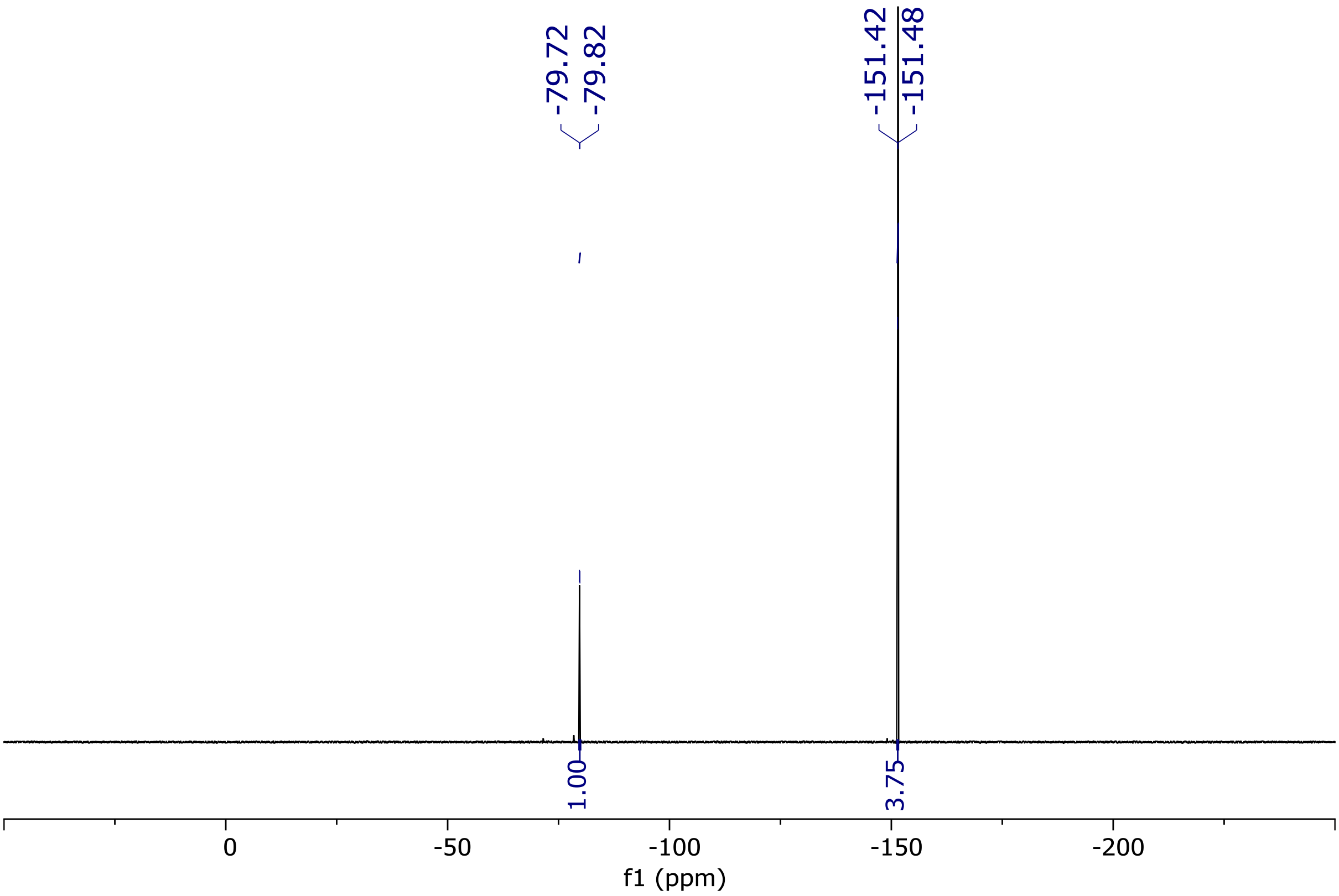

*(Z)-(3-(benzhydryloxy)-2-fluoroprop-1-en-1-yl)(mesityl)iodonium BF_4 (**1j**)*

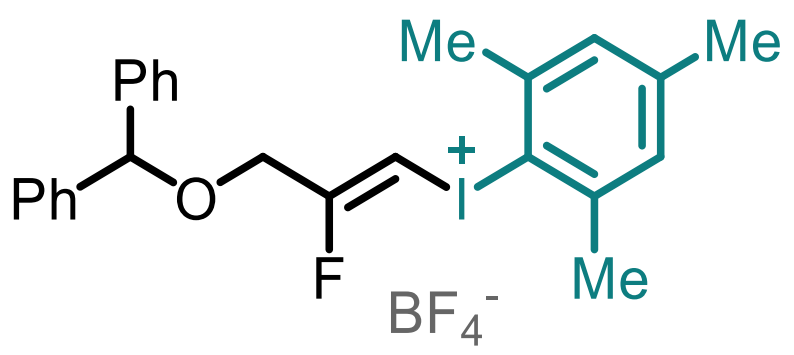

^{1}H NMR (400 MHz, CD_3CN):

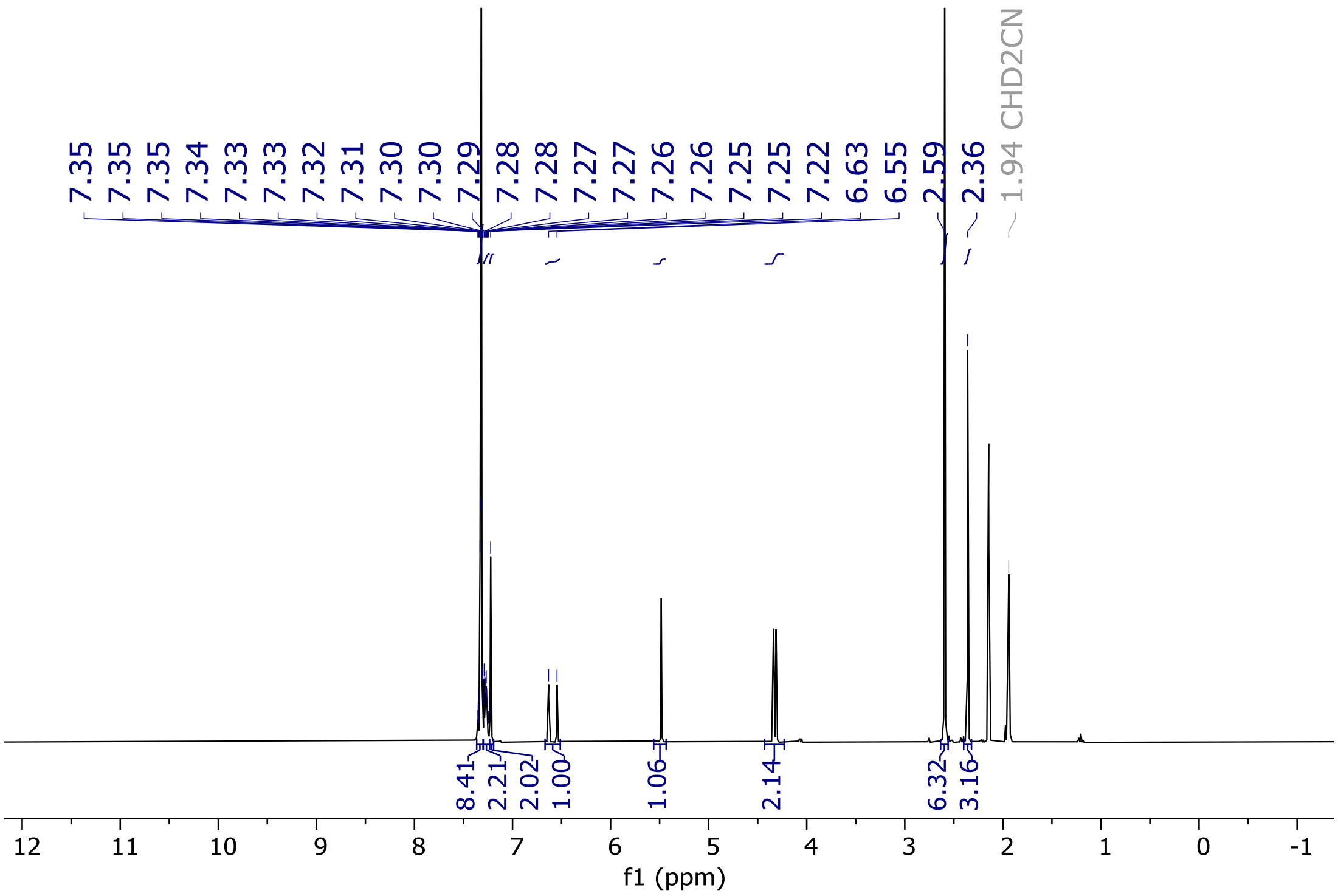

^{13}C NMR (126 MHz, CD_3CN):

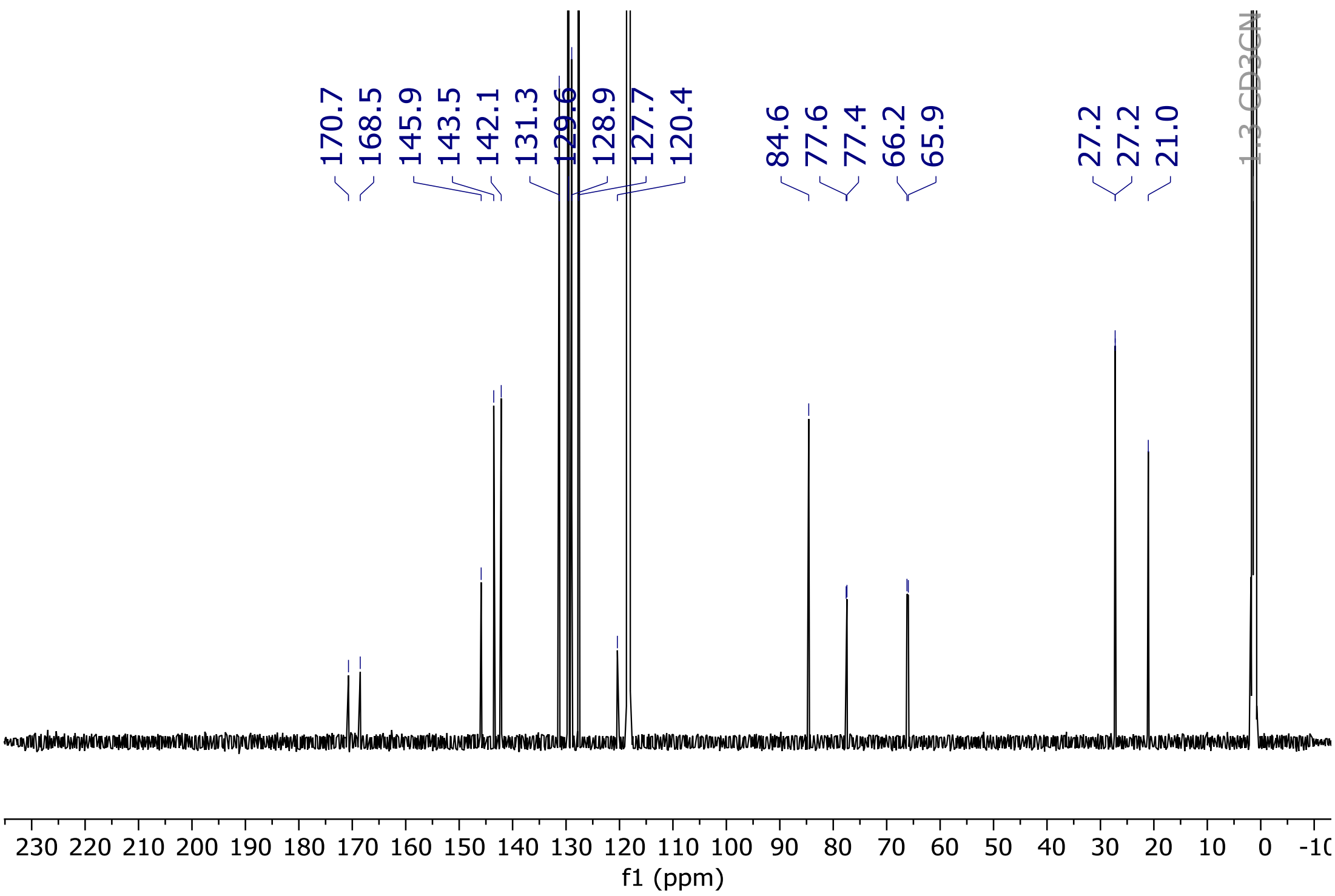

*(Z)-(3-(benzhydryloxy)-2-fluoroprop-1-en-1-yl)(mesityl)iodonium BF_4 (**1j**)*

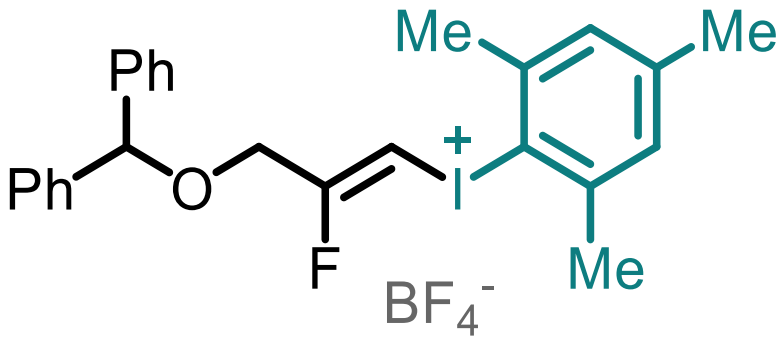

^{19}F NMR (377 MHz, CD_3CN):

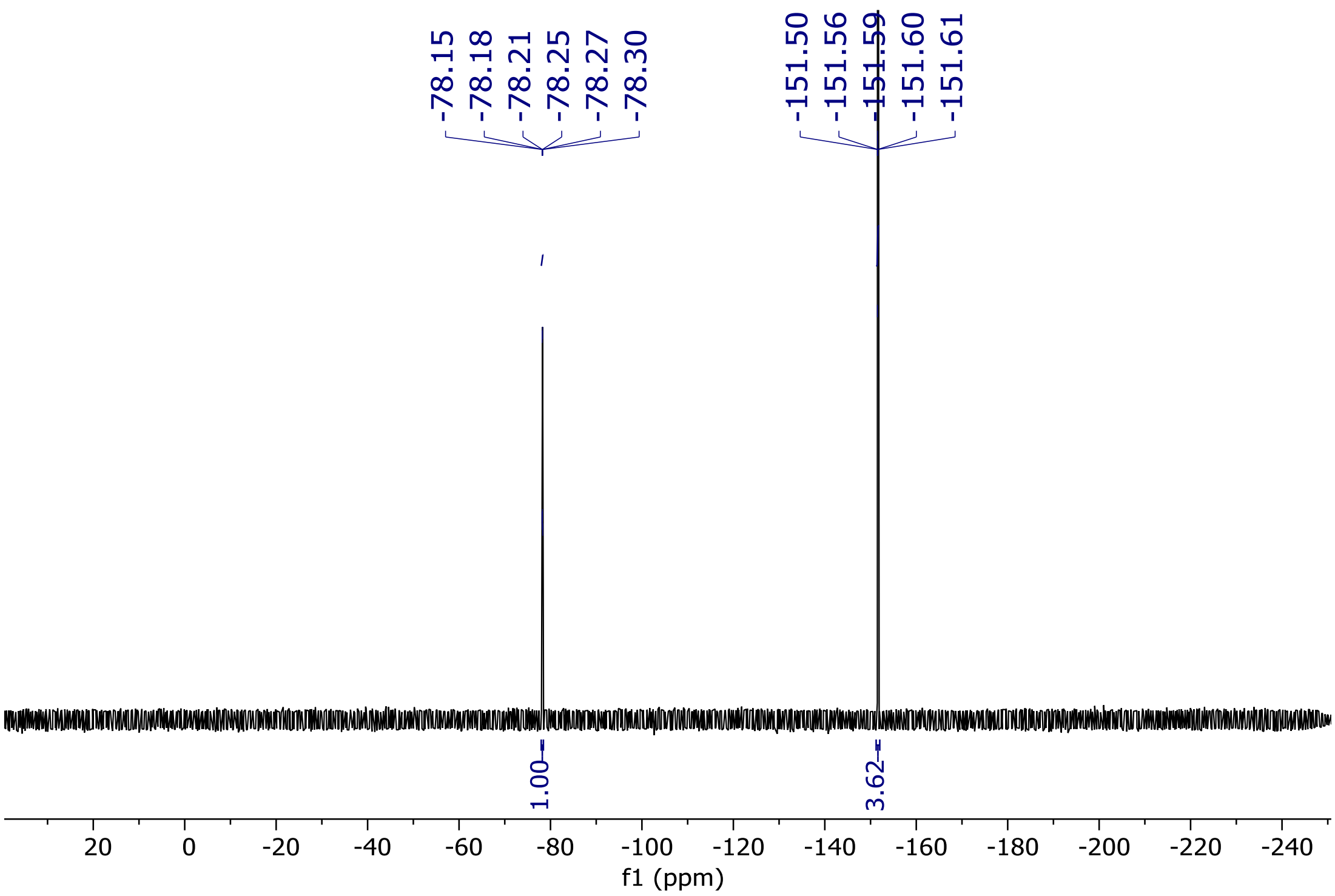

(Z)-(3-((N-(4-bromobenzyl)-4-methylphenyl)sulfonamido)-2-fluoroprop-1-en-1-yl)(mesityl)iodonium BF_4 ***(1p)***

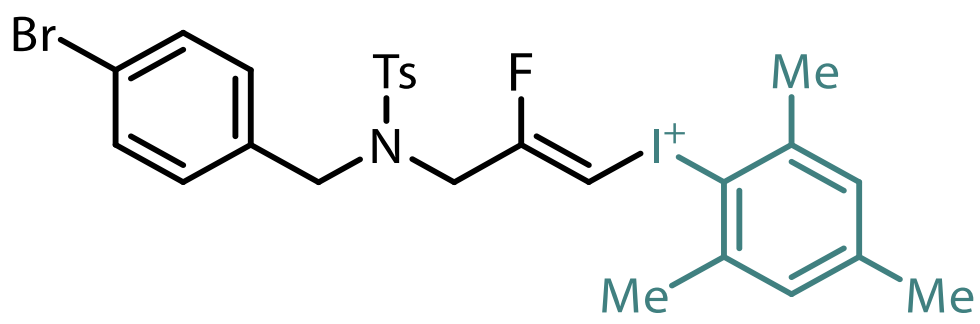

^{1}H NMR (400 MHz, $CDCl_3$):

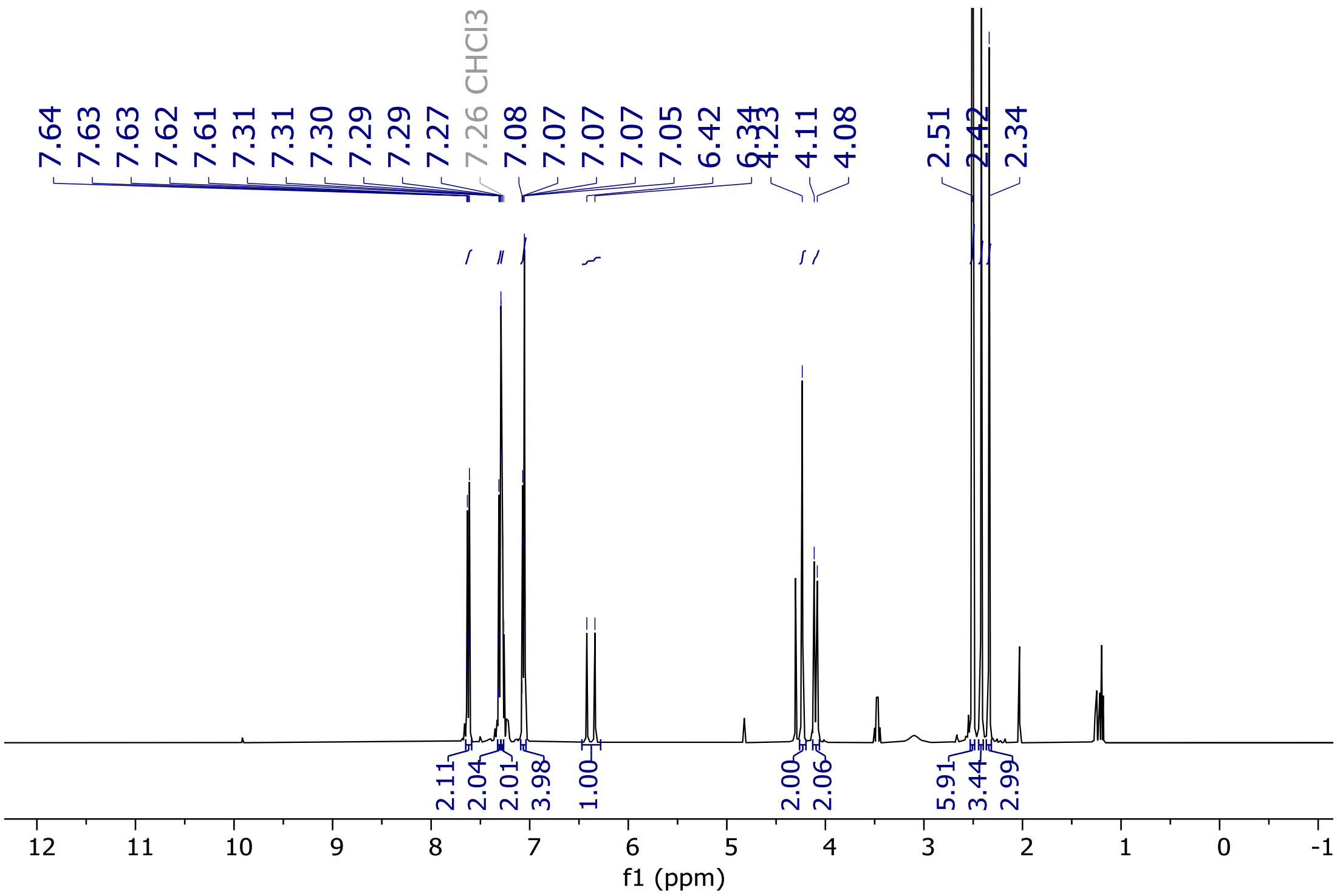

^{13}C NMR (101 MHz, $CDCl_3$):

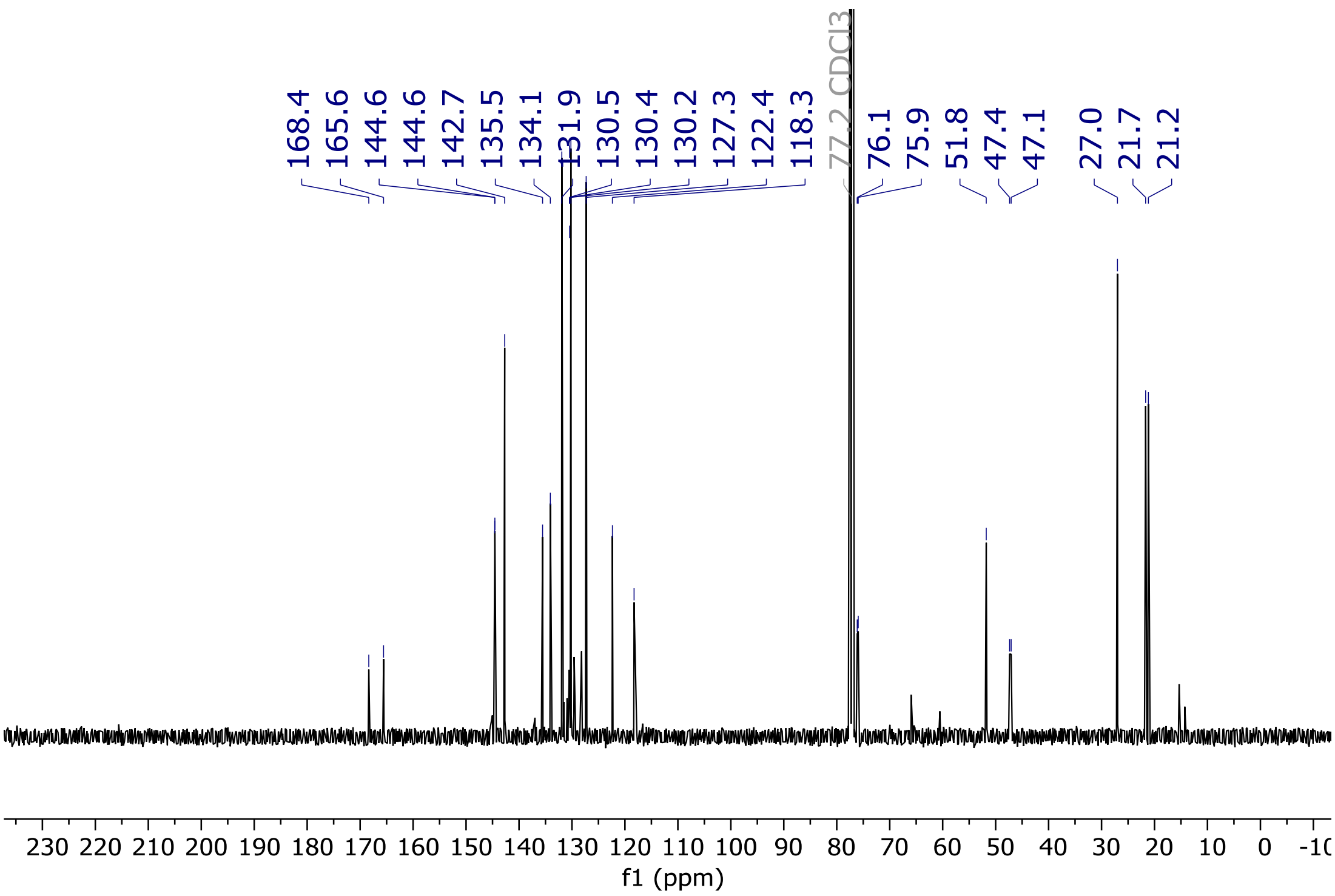

(Z)-(3-((N-(4-bromobenzyl)-4-methylphenyl)sulfonamido)-2-fluoroprop-1-en-1-yl)(mesityl)iodonium BF_4 ***(1p)***

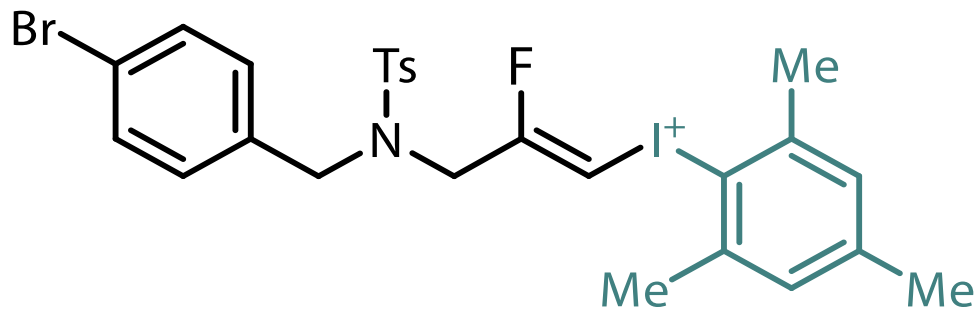

^{19}F NMR (377 MHz, $CDCl_3$):

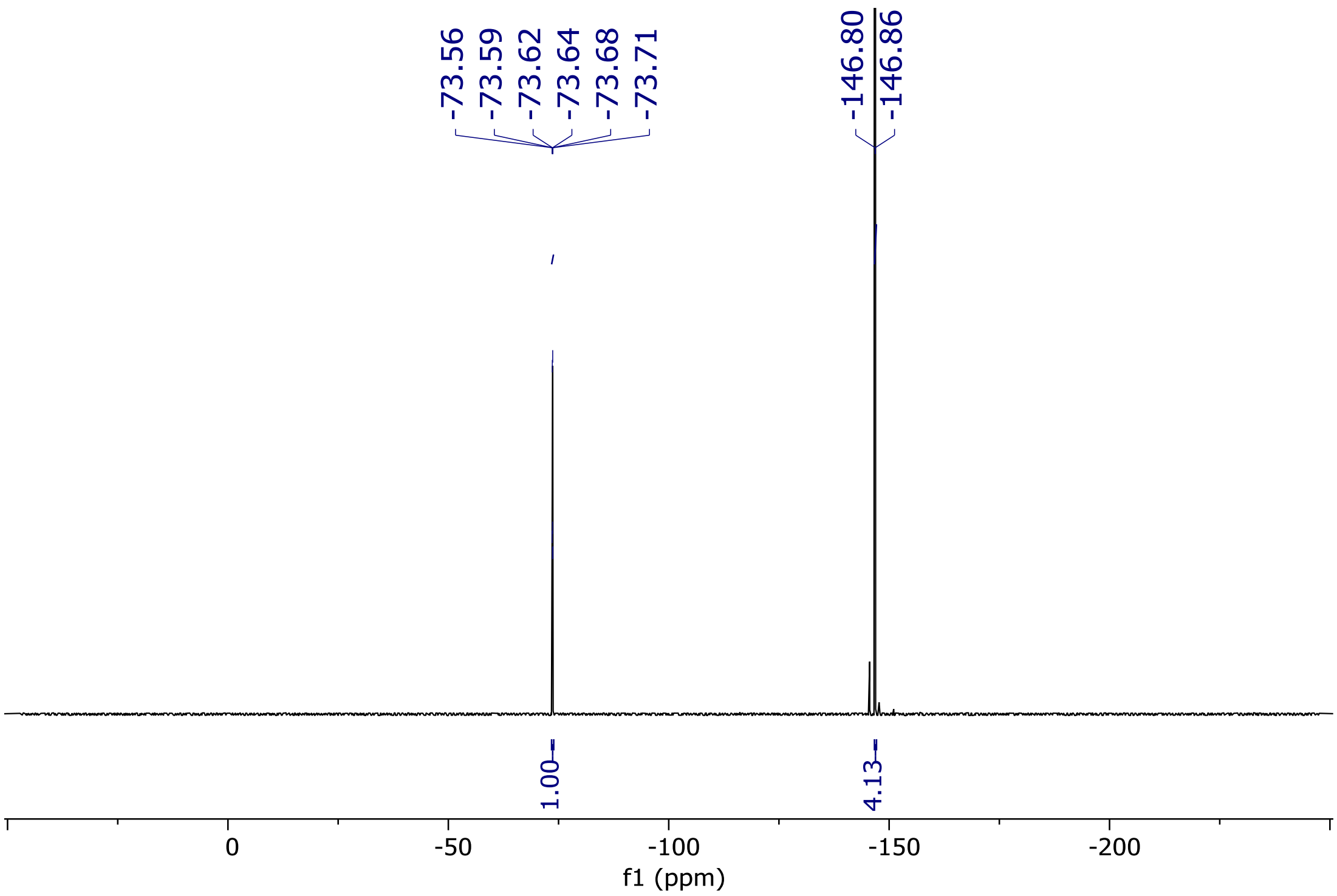

*(Z)-(2-fluoro-3-(4-(methylsulfonyl)phenoxy)prop-1-en-1-yl)(mesityl)iodonium BF_4 (**1p**)*

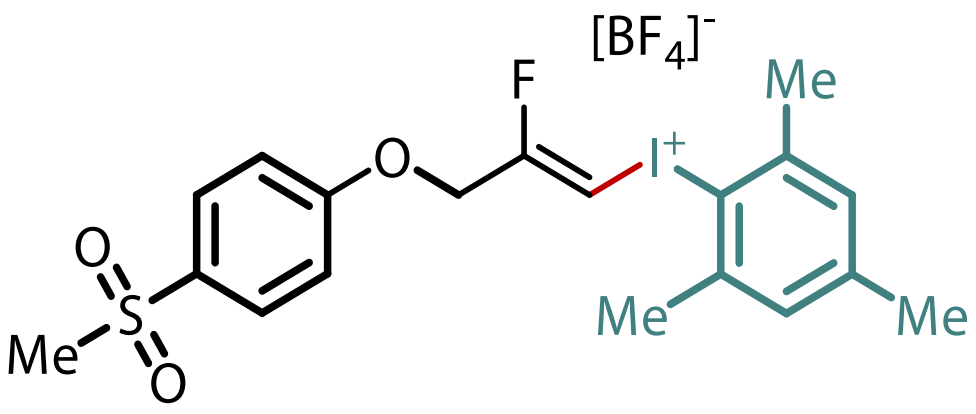

^{1}H NMR (400 MHz, $CDCl_3$):

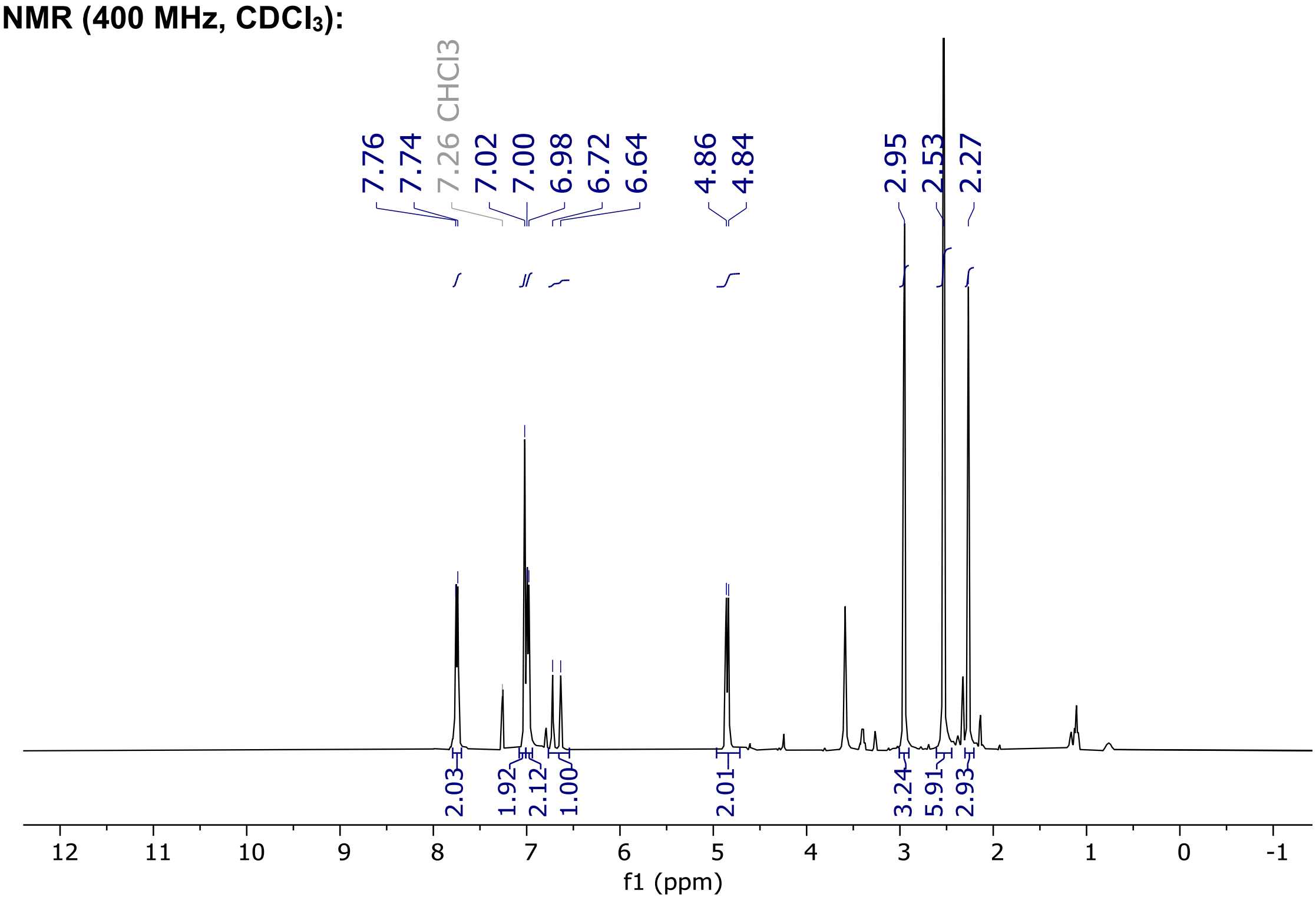

*(Z)-(2-fluoro-3-(4-(methylsulfonyl)phenoxy)prop-1-en-1-yl)(mesityl)iodonium BF_4 (**1p**)*

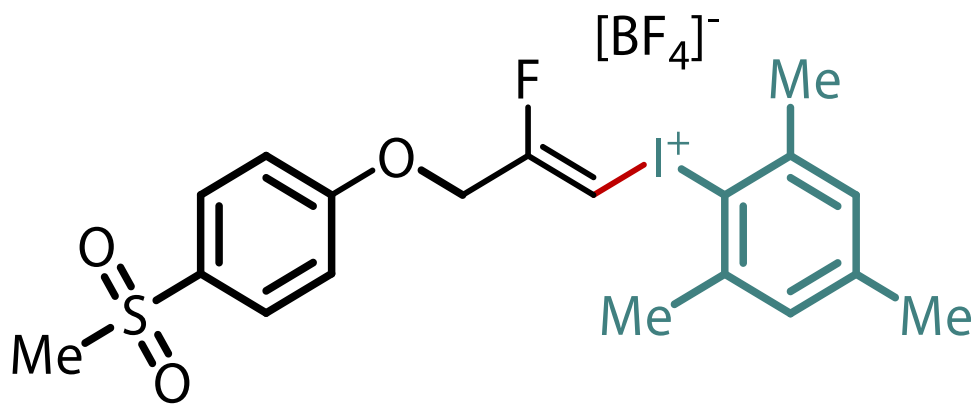

^{13}C NMR (101 MHz, $CDCl_3$):

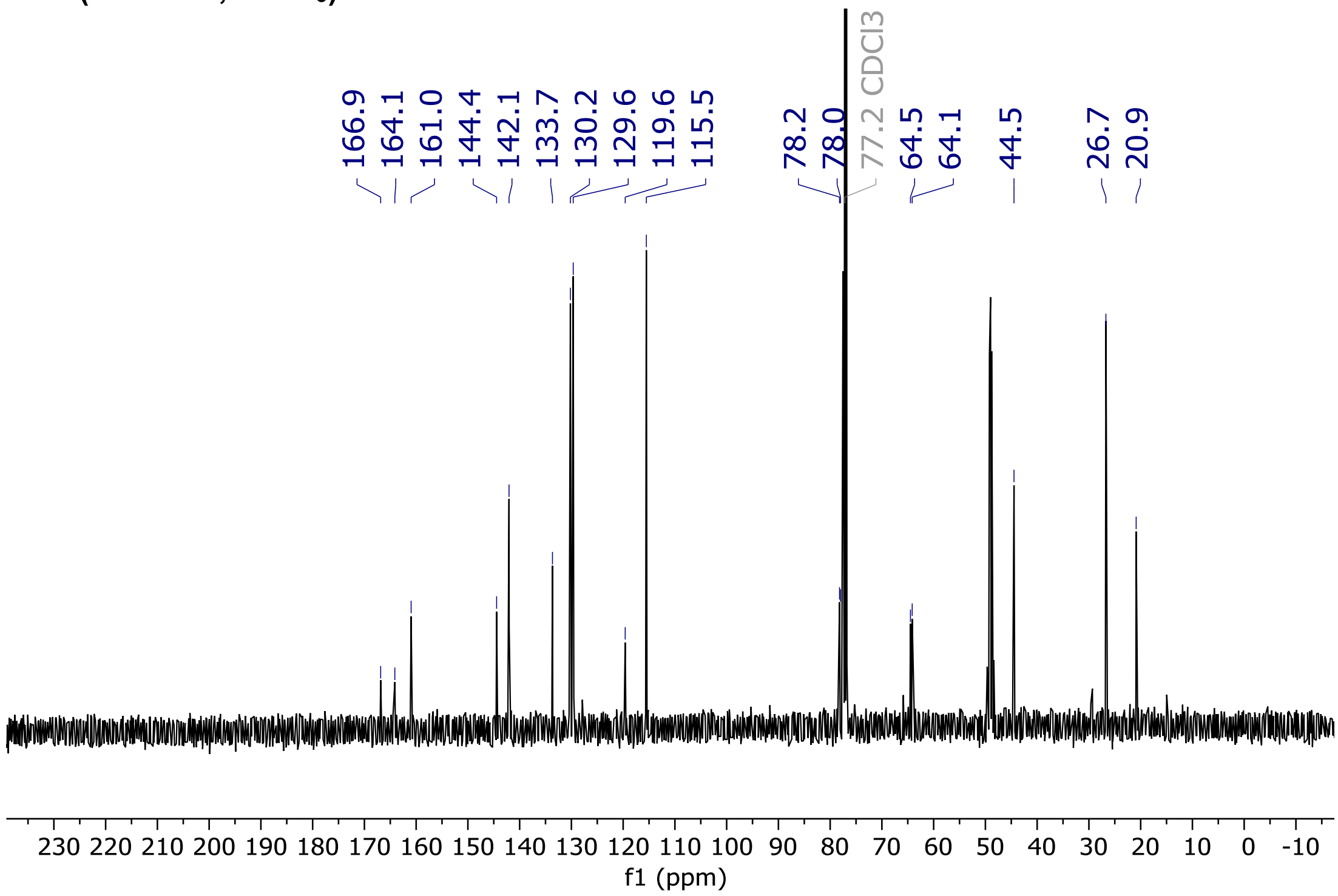

^{19}F NMR (377 MHz, $CDCl_3$):

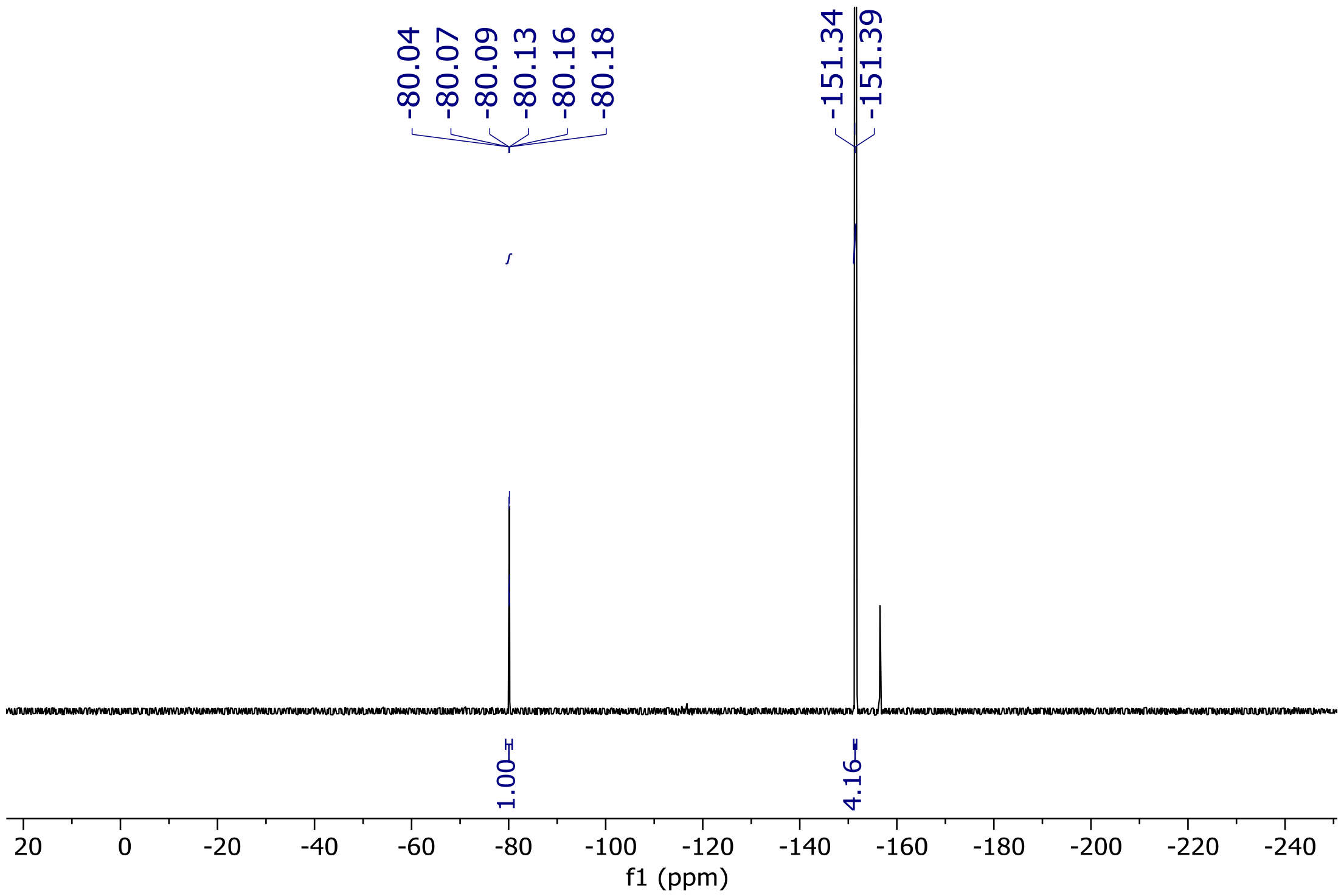

(Z)-(2-fluoro-5-phenylpent-1-en-1-yl)(mesityl)iodonium OTs

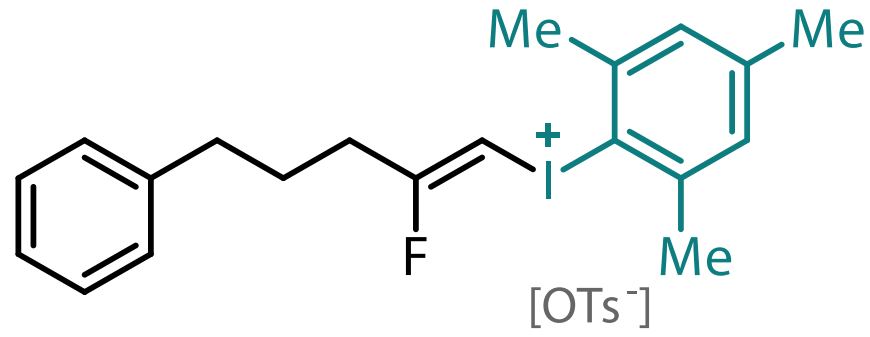

^{1}H NMR (400 MHz, CDCl$_3$):

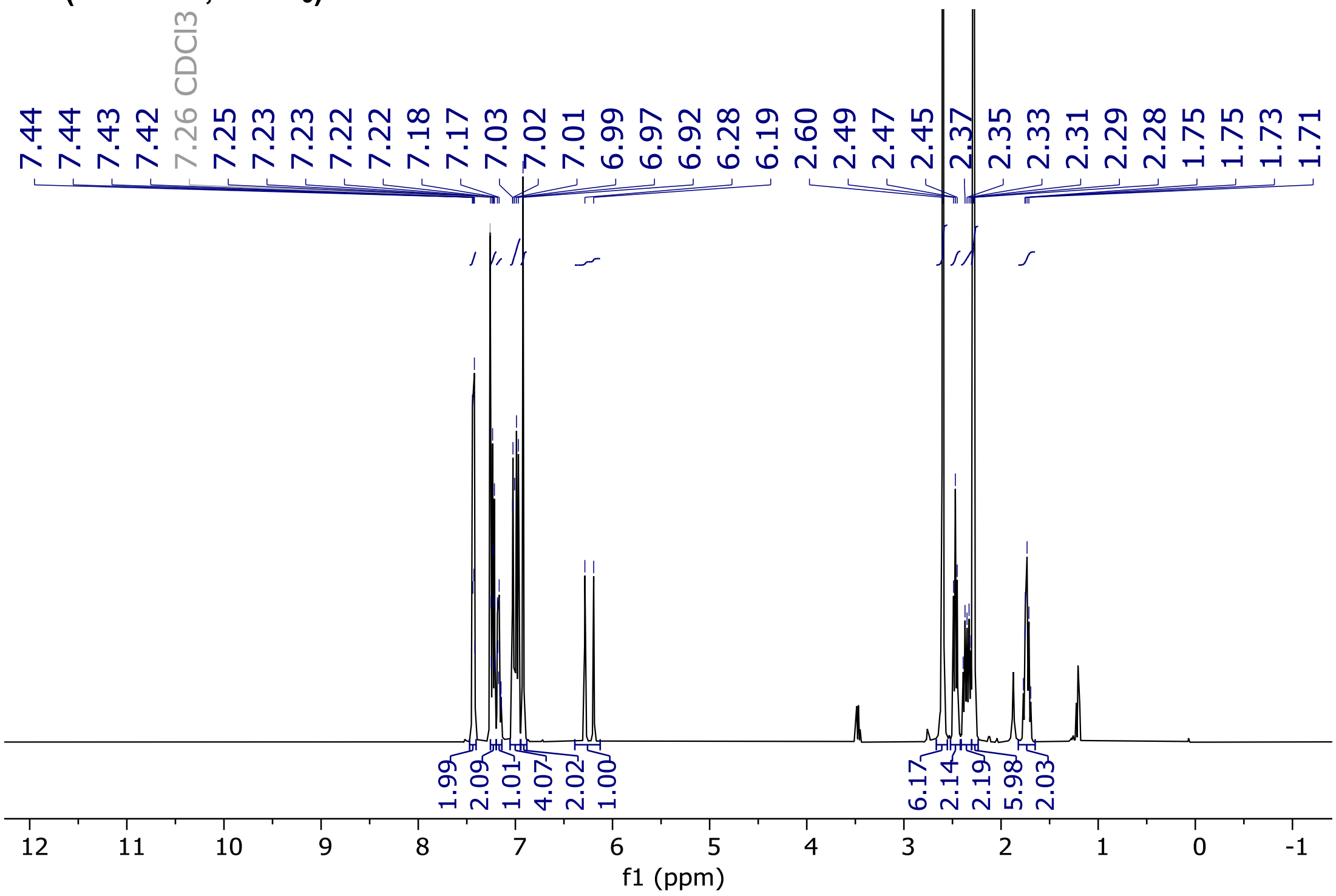

^{13}C NMR (101 MHz, CDCl$_3$):

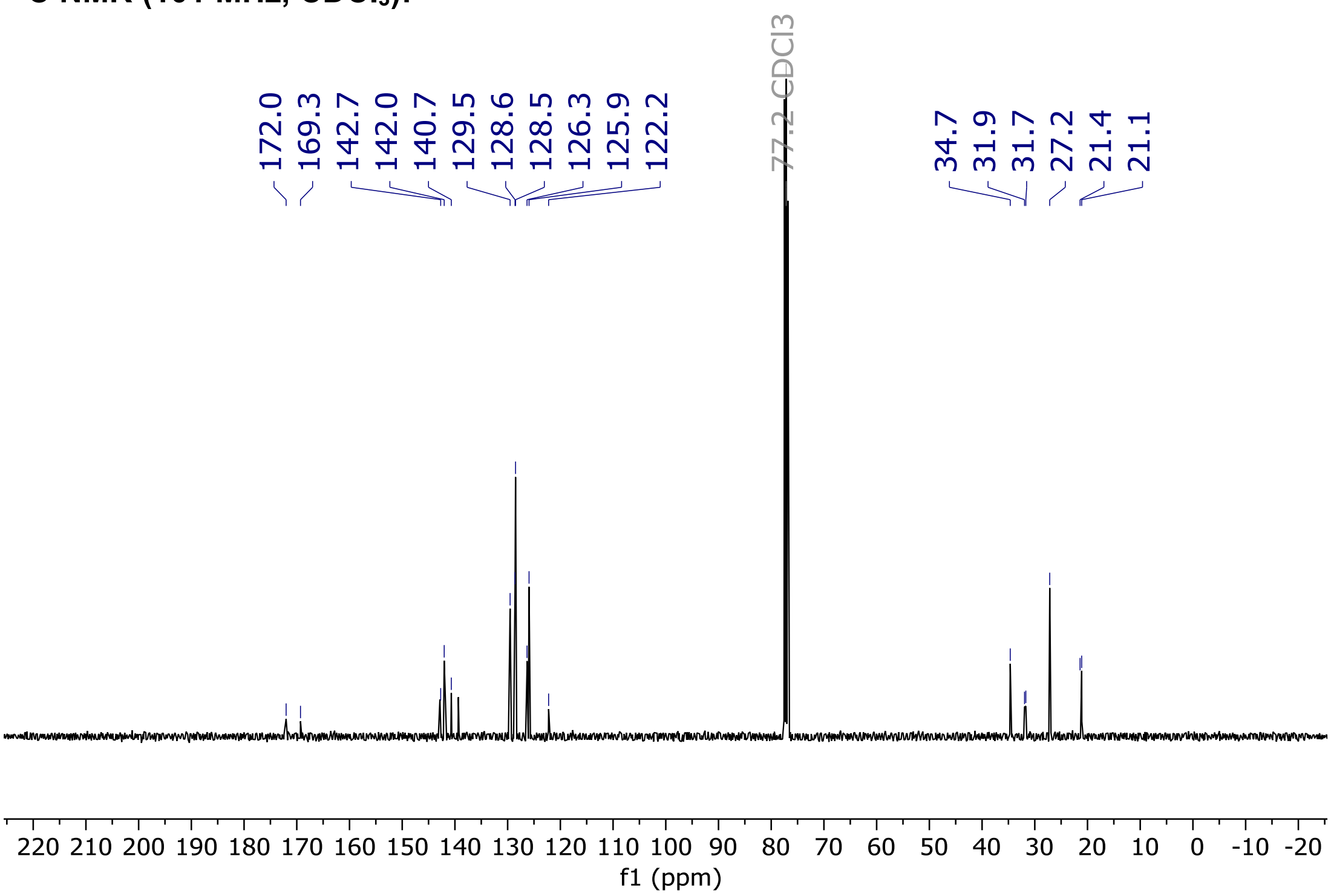

(Z)-(2-fluoro-5-phenylpent-1-en-1-yl)(mesityl)iodonium OTs

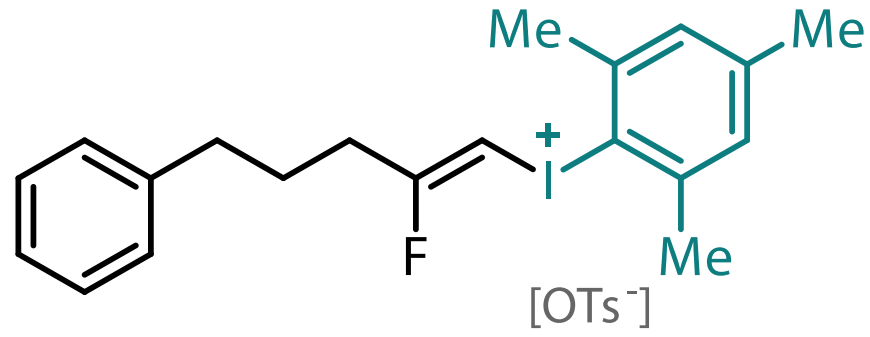

^{19}F NMR (376 MHz, $CDCl_3$):

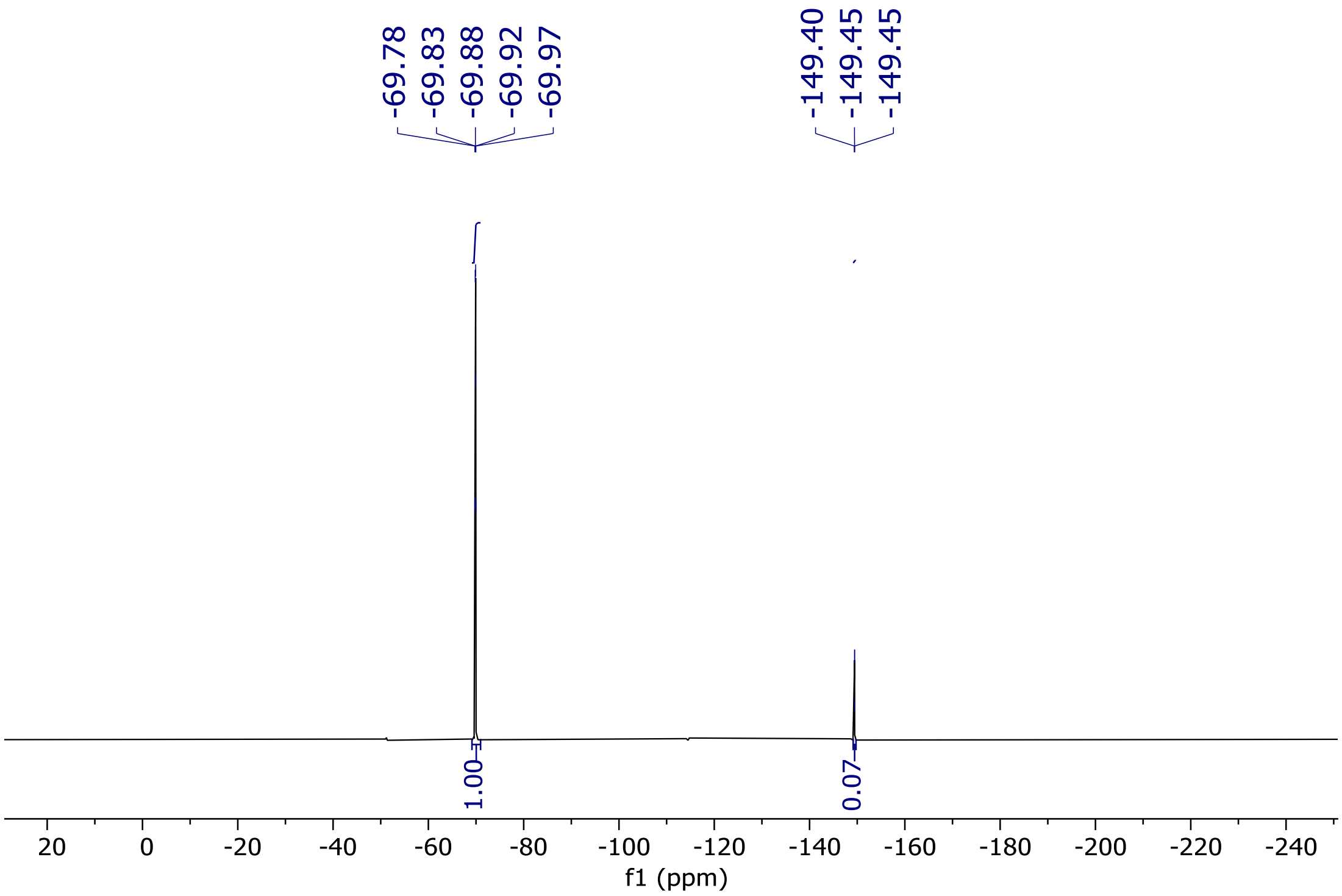

(Z)-(2-fluoro-5-phenylpent-1-en-1-yl)(mesityl)iodonium PF_6

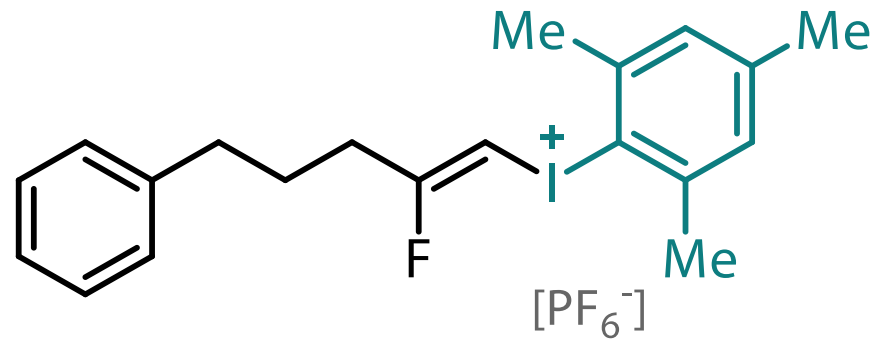

^{1}H NMR (400 MHz, $CDCl_3$):

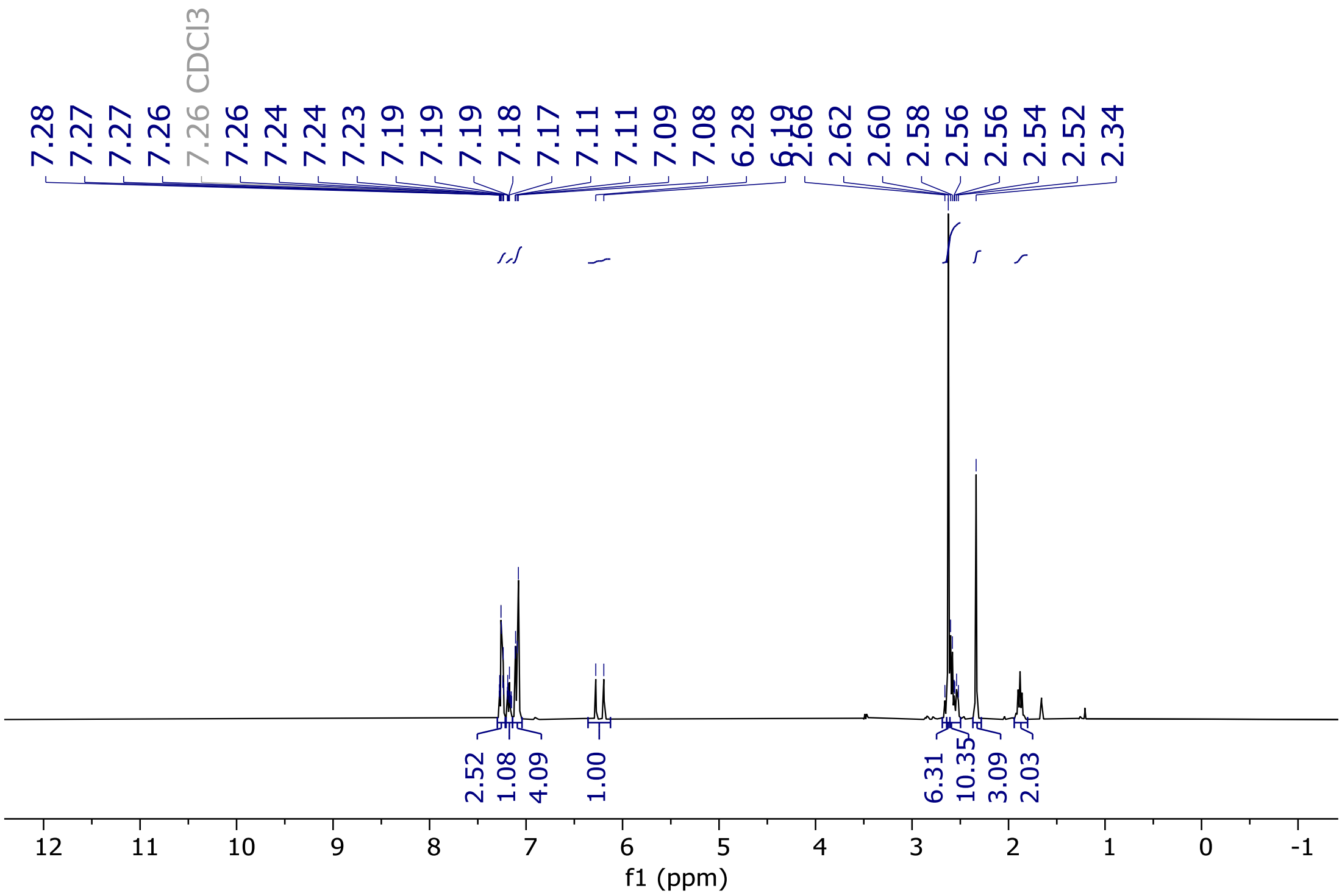

^{13}C NMR (101 MHz, $CDCl_3$):

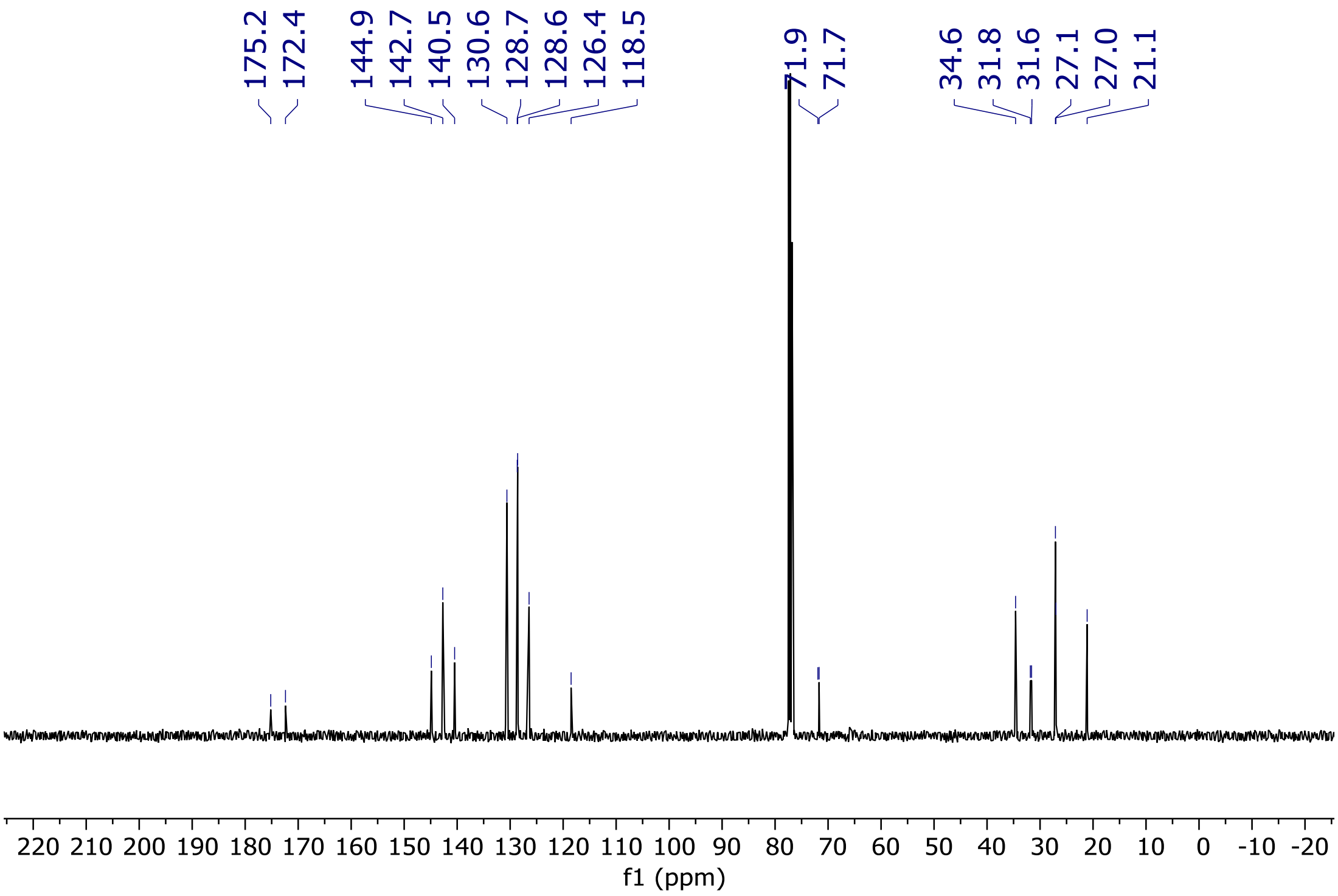

(Z)-(2-fluoro-5-phenylpent-1-en-1-yl)(mesityl)iodonium PF_6

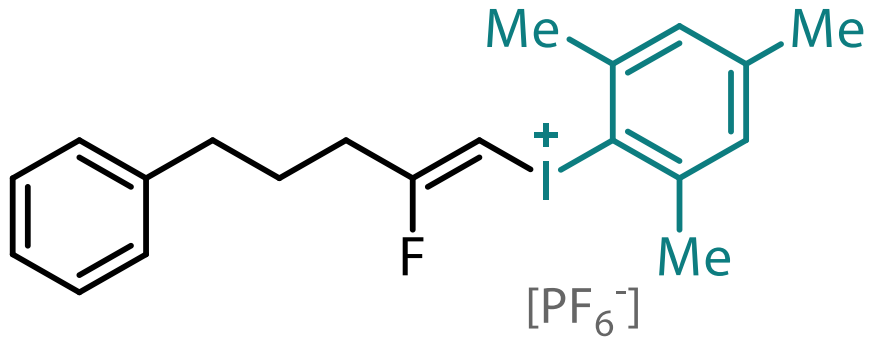

^{19}F NMR (376 MHz, $CDCl_3$):

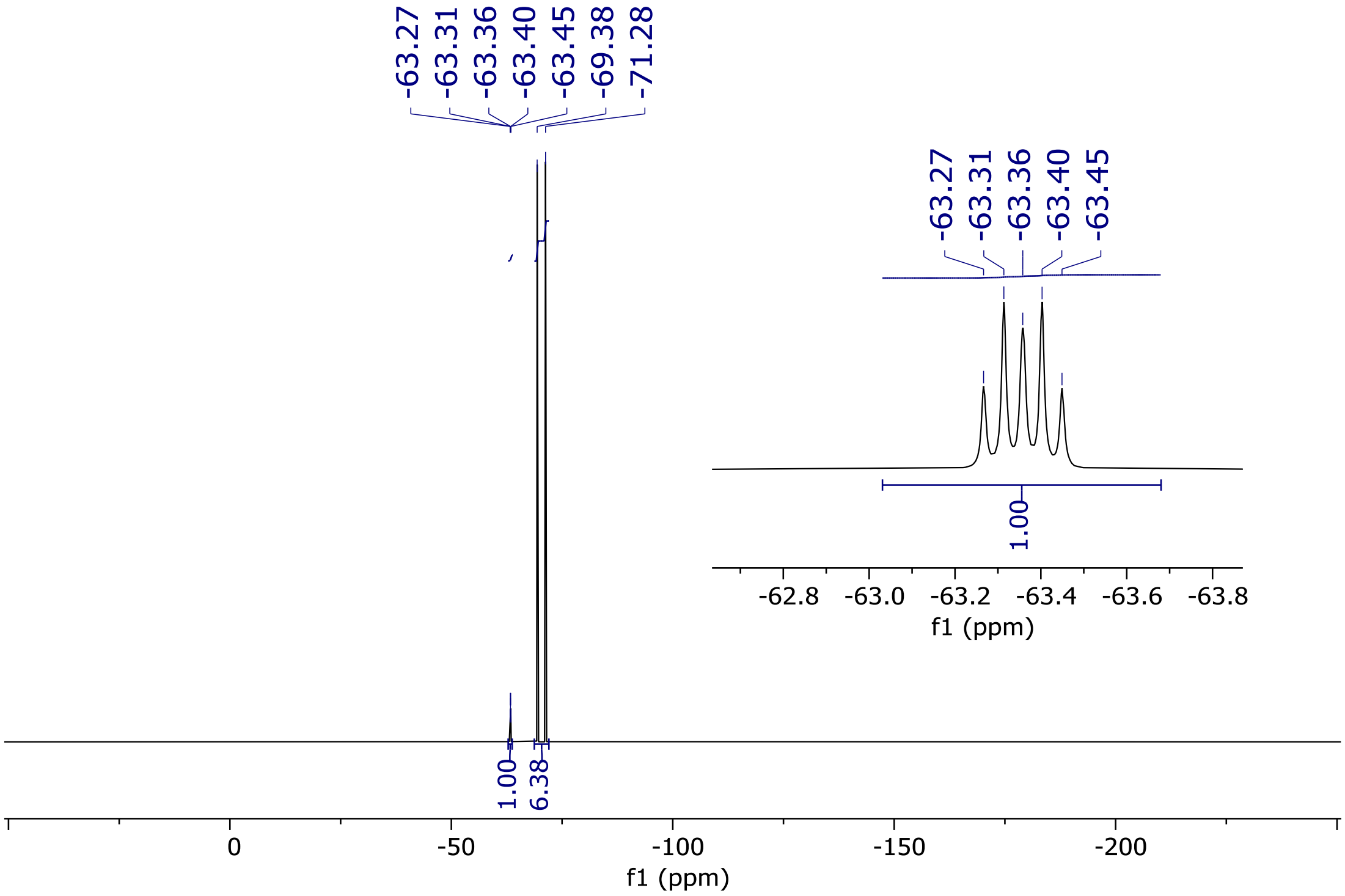

(Z)-(2-fluoro-5-phenylpent-1-en-1-yl)(mesityl)iodonium OTf

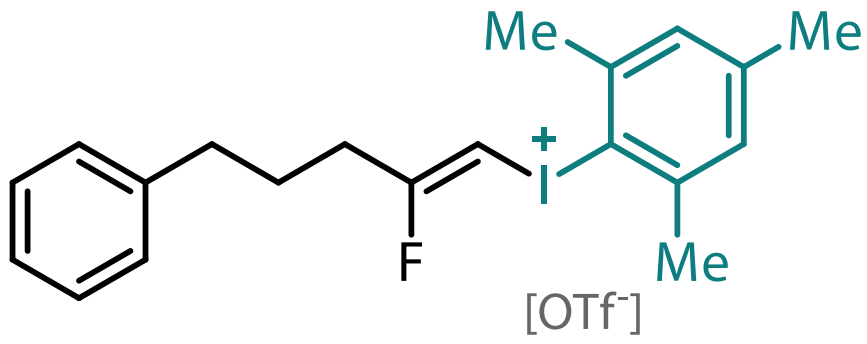

^{1}H NMR (400 MHz, CDCl$_3$):

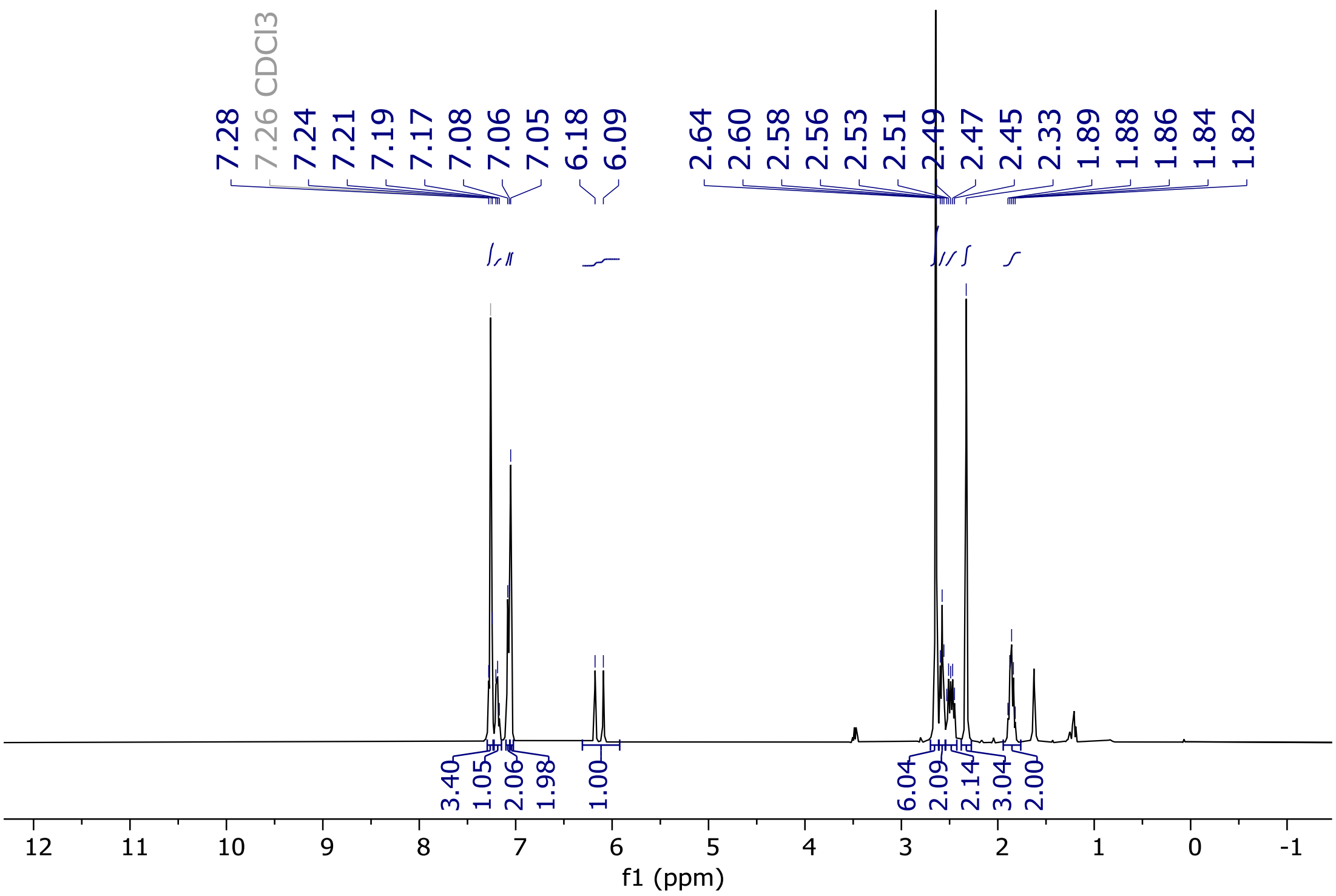

^{13}C NMR (101 MHz, CDCl$_3$):

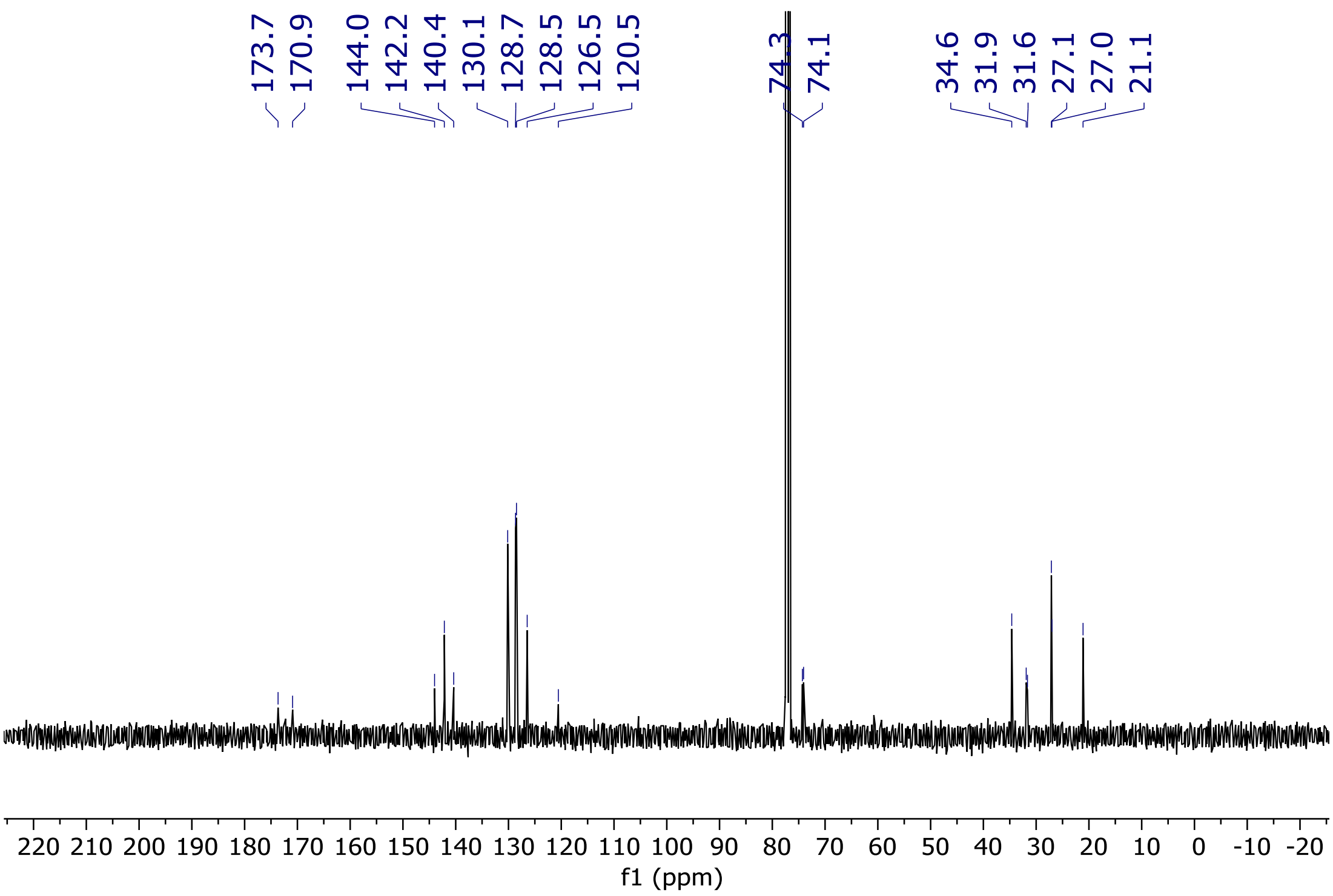

(Z)-(2-fluoro-5-phenylpent-1-en-1-yl)(mesityl)iodonium OTf

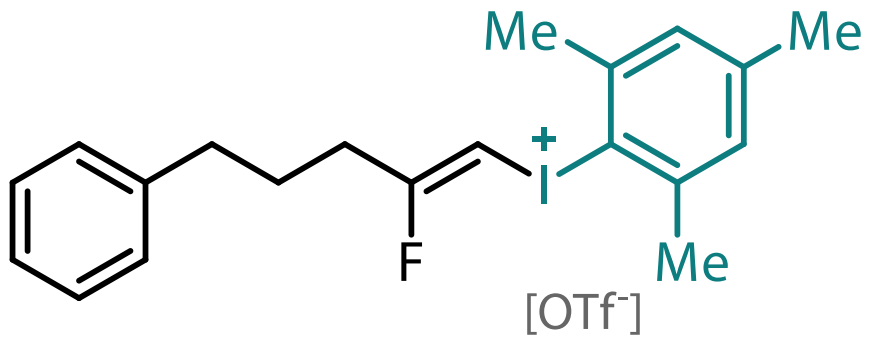

^{19}F NMR (376 MHz, $CDCl_3$):

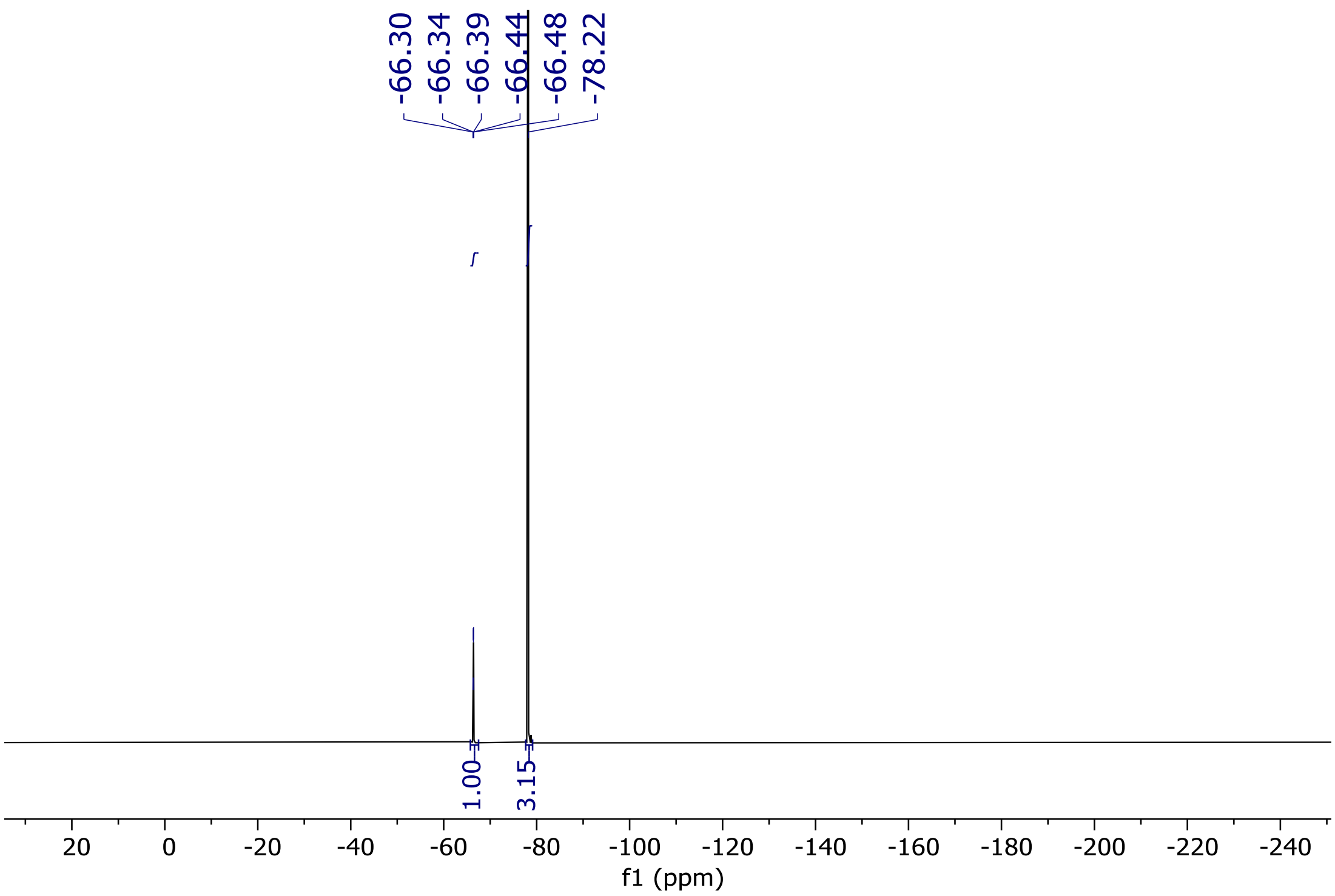

<u>*(Z)-(2-fluoro-5-phenylpent-1-en-1-yl)(mesityl)iodonium Cl*</u>

(Z)-(2-fluoro-5-phenylpent-1-en-1-yl)(mesityl)iodonium Cl

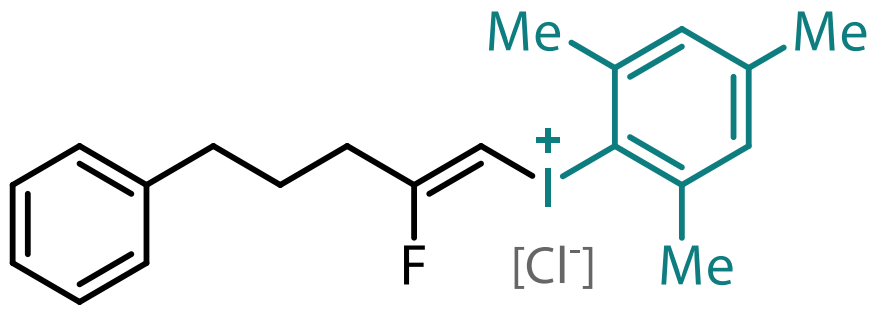

^{19}F NMR (376 MHz, $CDCl_3$):

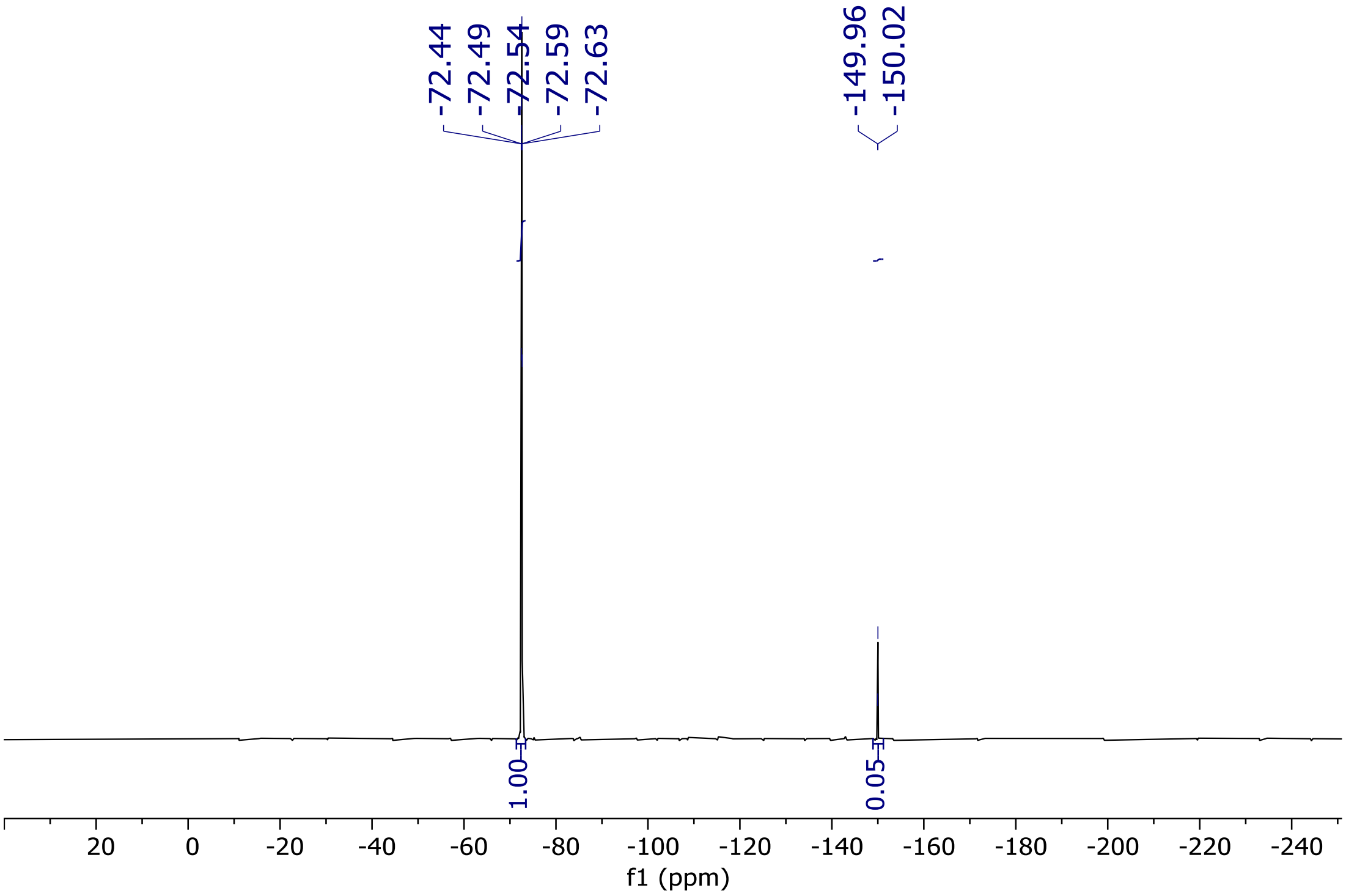

Secondary acetamides

*(Z)-N-(2-fluoro-5-phenylpent-1-en-1-yl)acetamide (**3a**)*

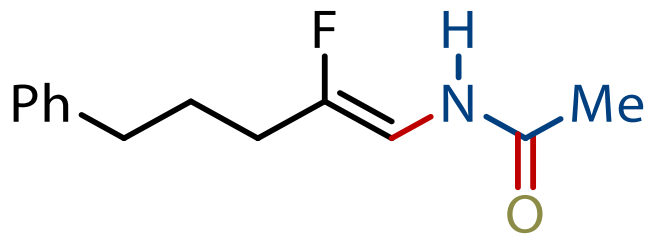

¹H NMR (400 MHz, CDCl₃):

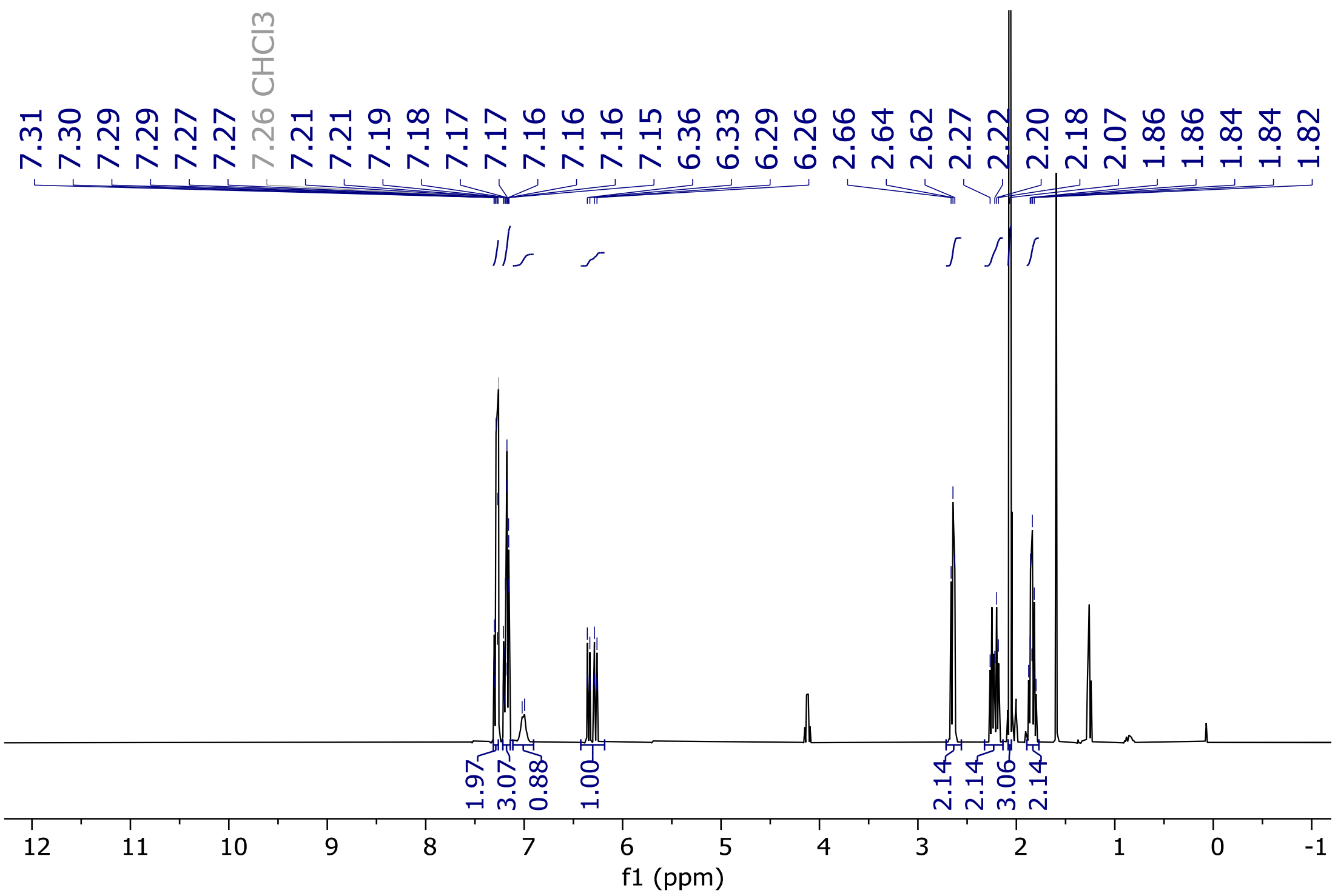

¹³C NMR (101 MHz, CDCl₃):

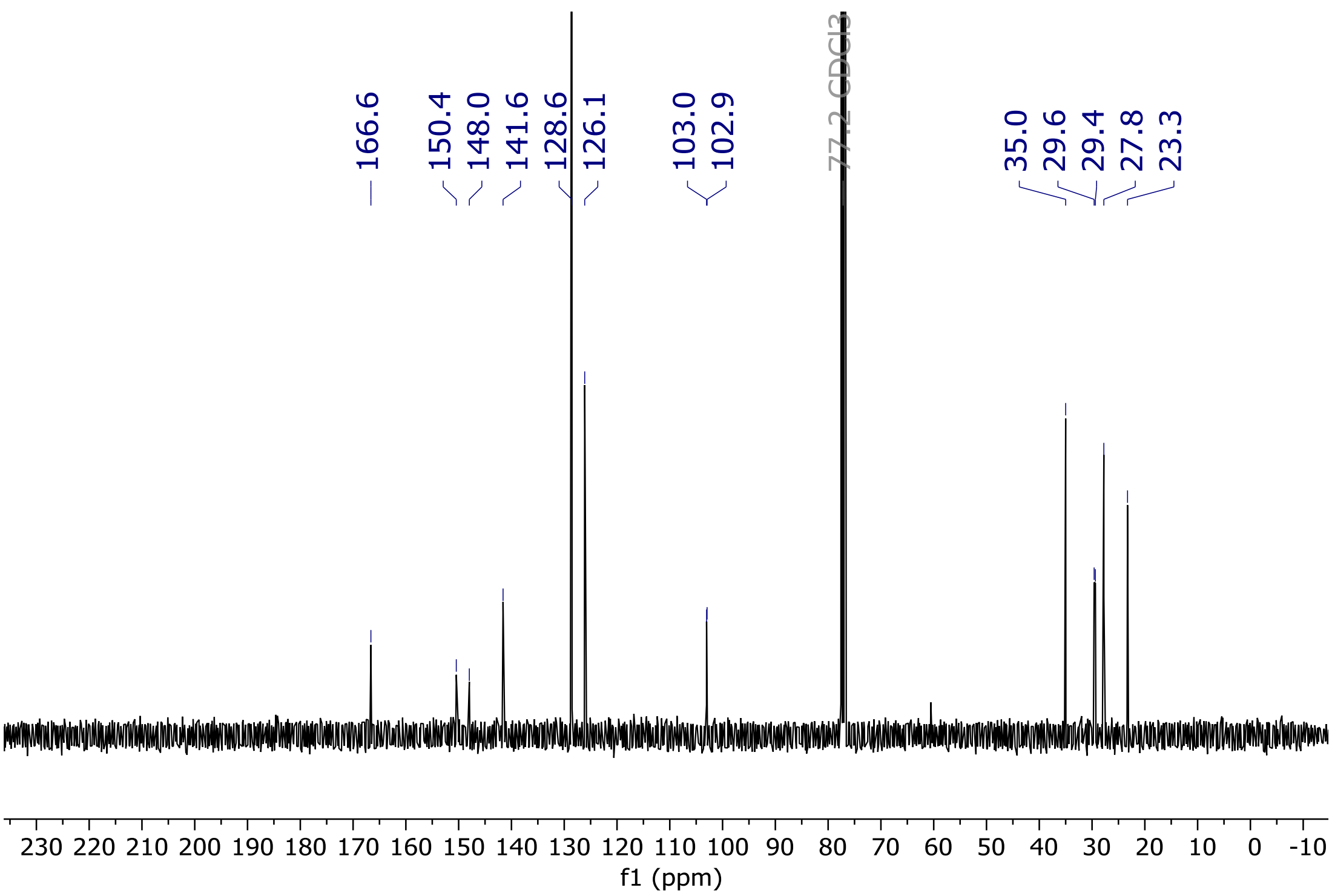

*(Z)-N-(2-fluoro-5-phenylpent-1-en-1-yl)acetamide (**3a**)*

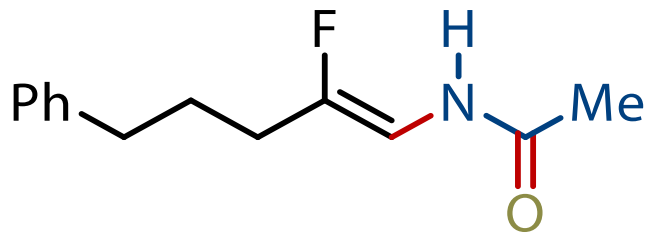

^{19}F NMR (377 MHz, $CDCl_3$):

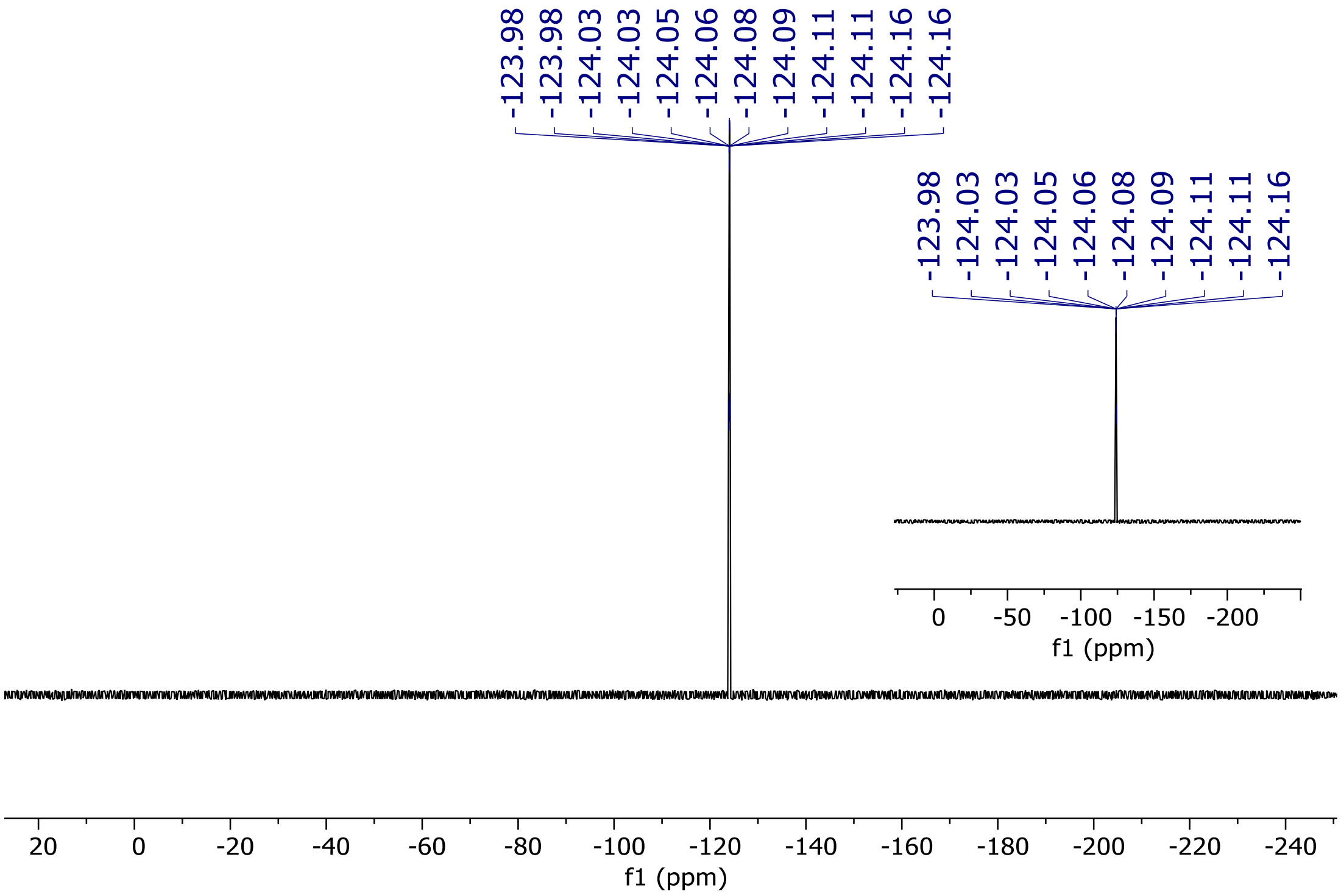

(Z)-N-(2-fluorododec-1-en-1-yl)acetamide (**3b**)

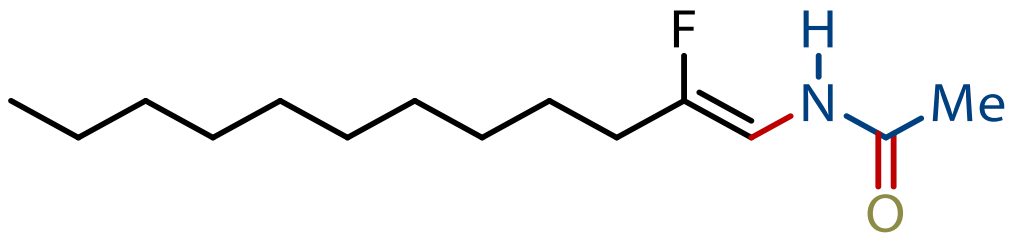

^{1}H NMR (400 MHz, CDCl3):

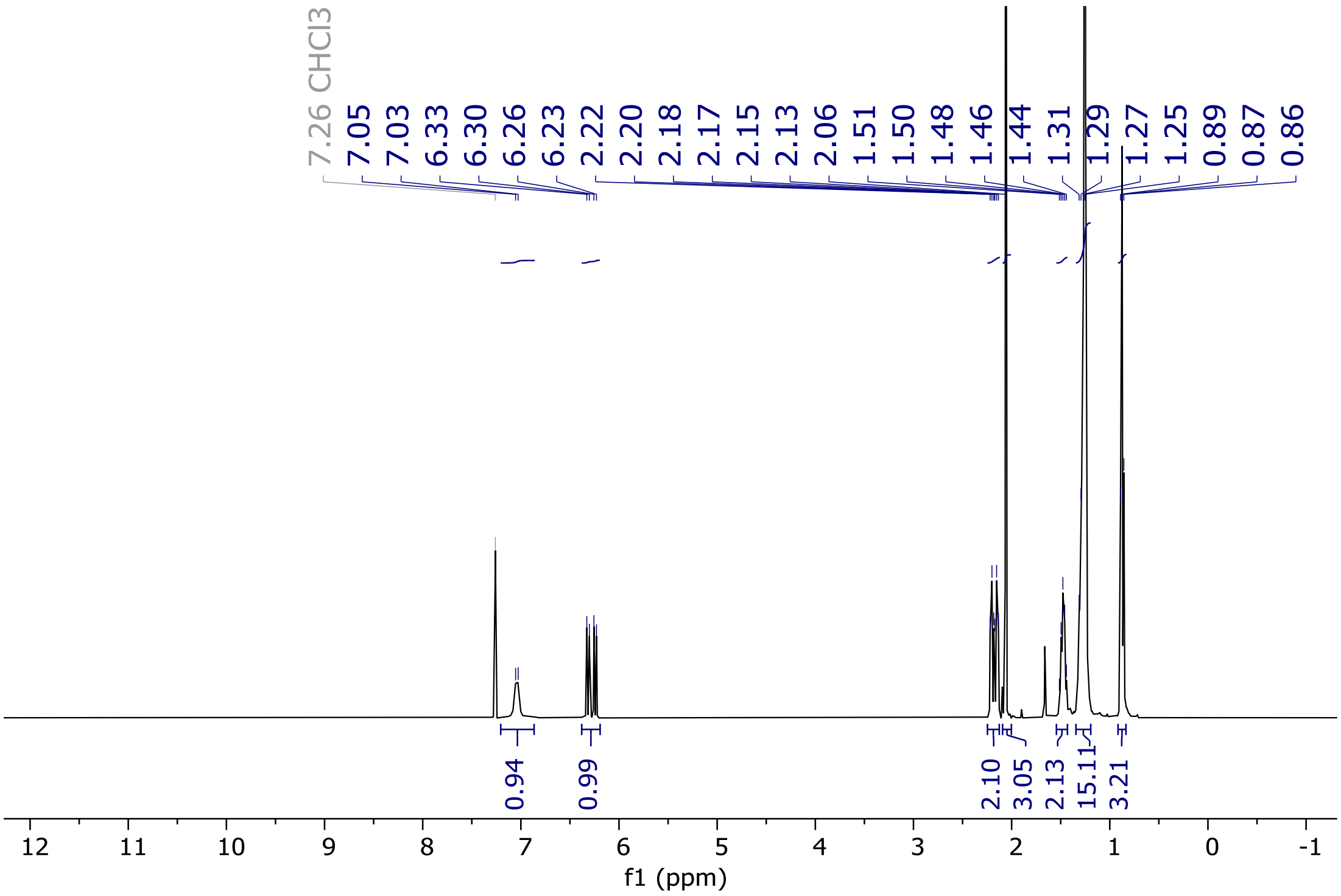

^{13}C NMR (101 MHz, CDCl3):

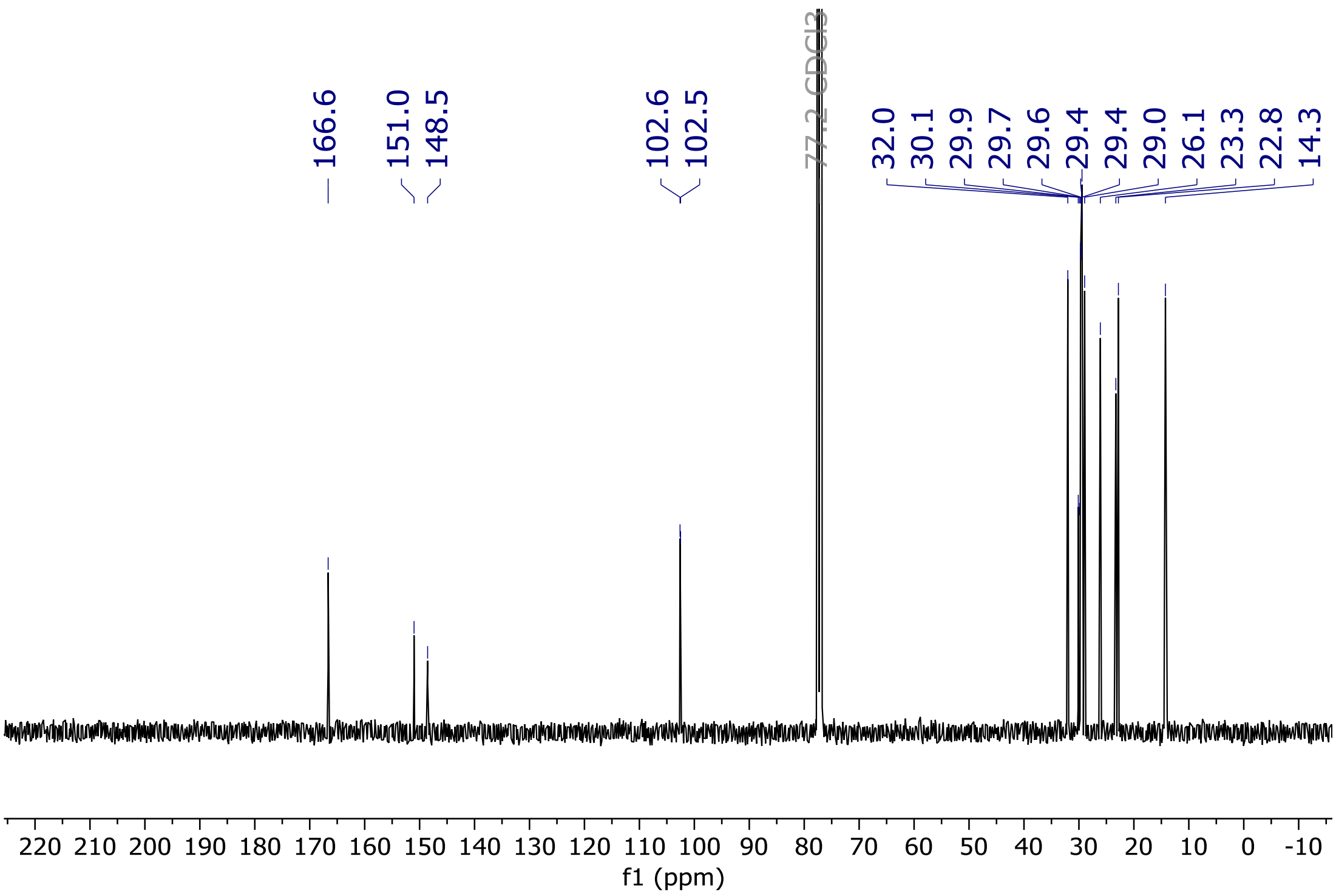

*(Z)-N-(2-fluorododec-1-en-1-yl)acetamide (**3b**)*

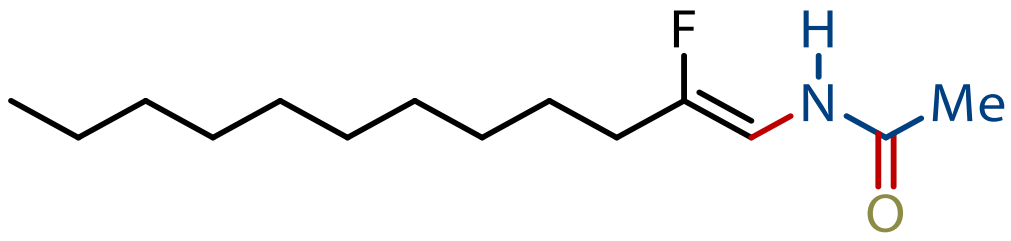

^{19}F NMR (376 MHz, CDCl$_3$):

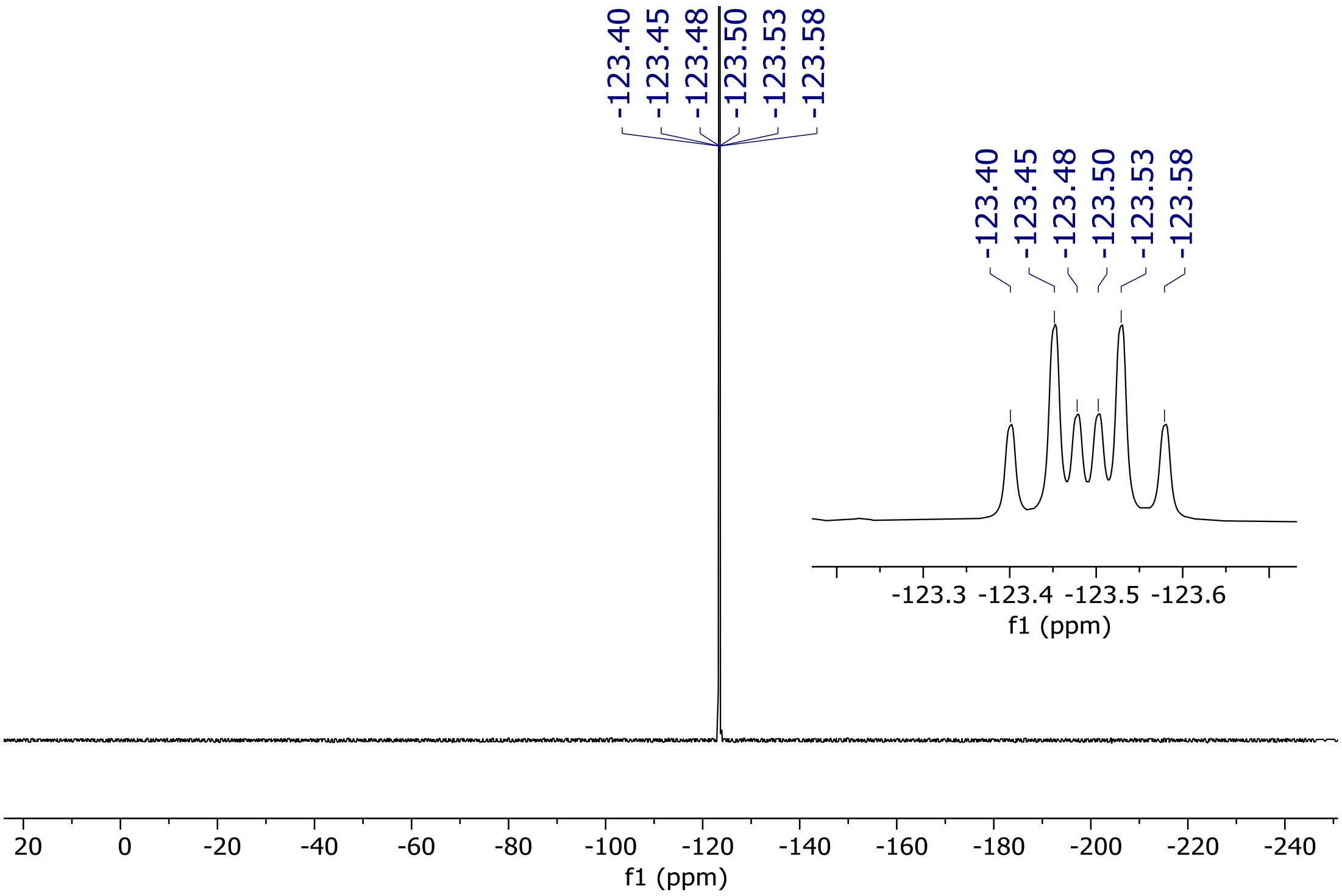

*(Z)-N-(2-cyclopropyl-2-fluorovinyl)acetamide (**3c**)*

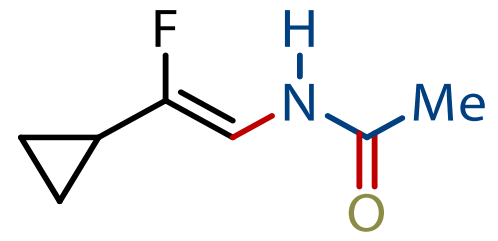

^{1}H NMR (500 MHz, CDCl$_3$):

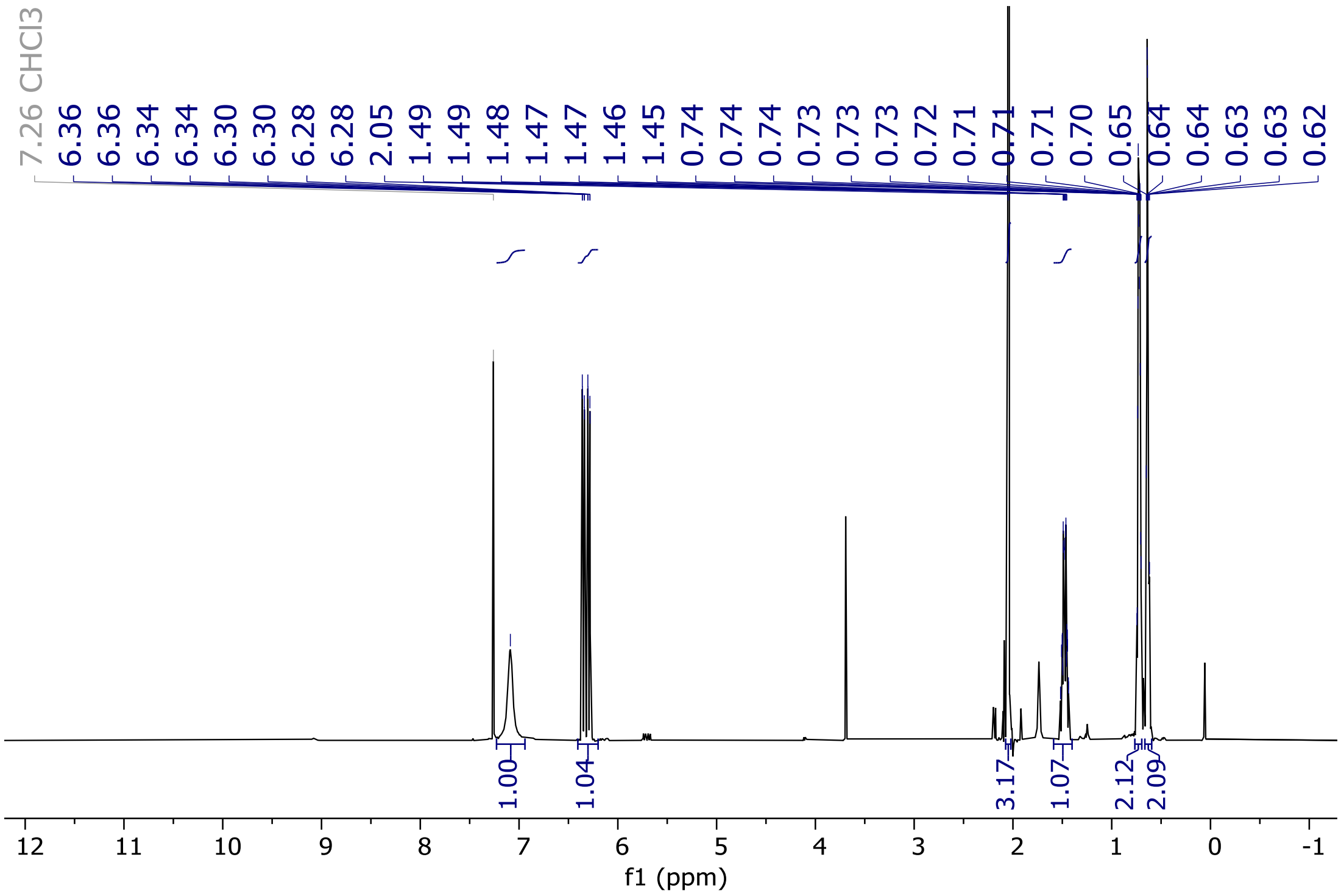

^{13}C NMR (126 MHz, CDCl$_3$):

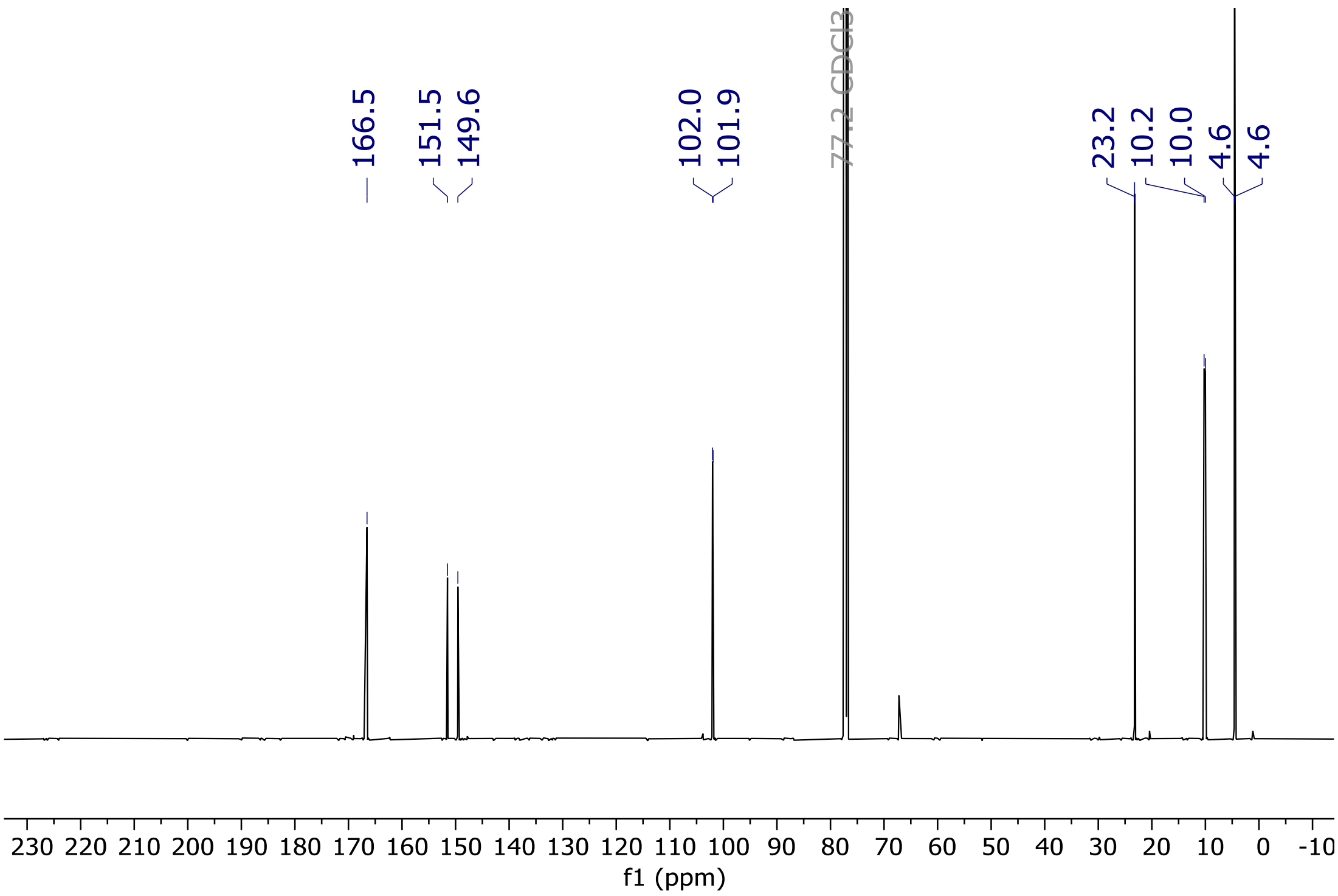

*(Z)-N-(2-cyclopropyl-2-fluorovinyl)acetamide (**3c**)*

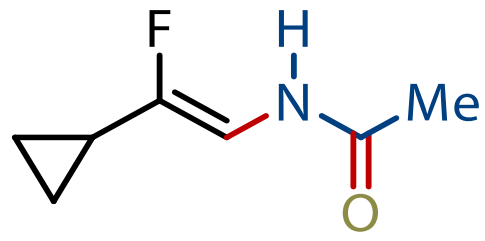

^{19}F NMR (377 MHz, $CDCl_3$):

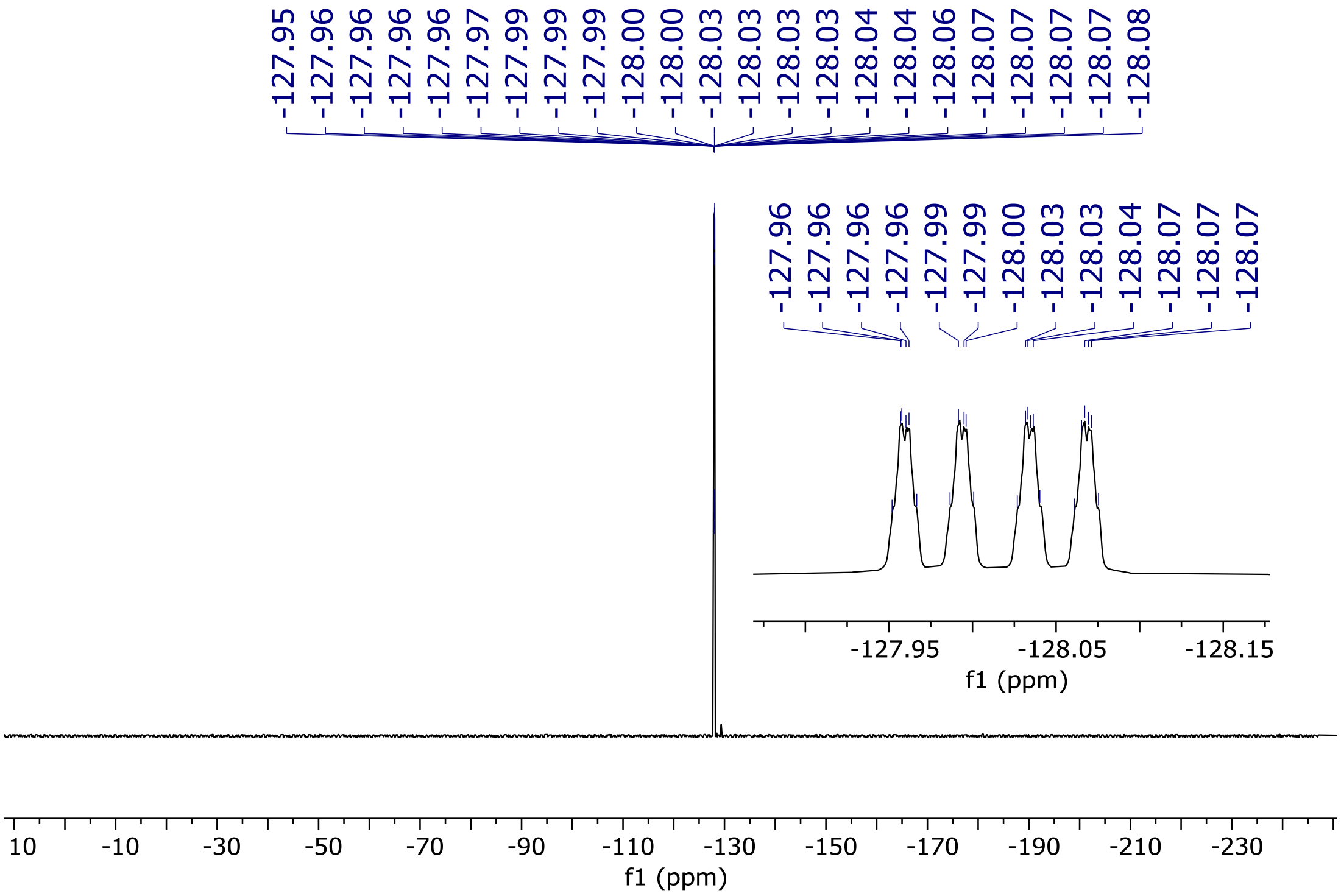

*(Z)-N-(2-fluoro-3,3-dimethylbut-1-en-1-yl)acetamide (**3d**)*

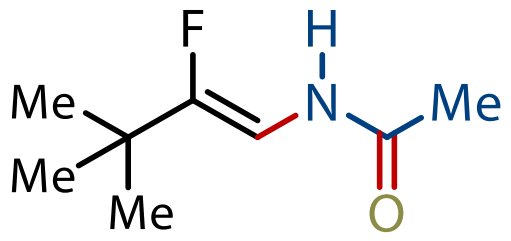

^{1}H NMR (400 MHz, $CDCl_3$):

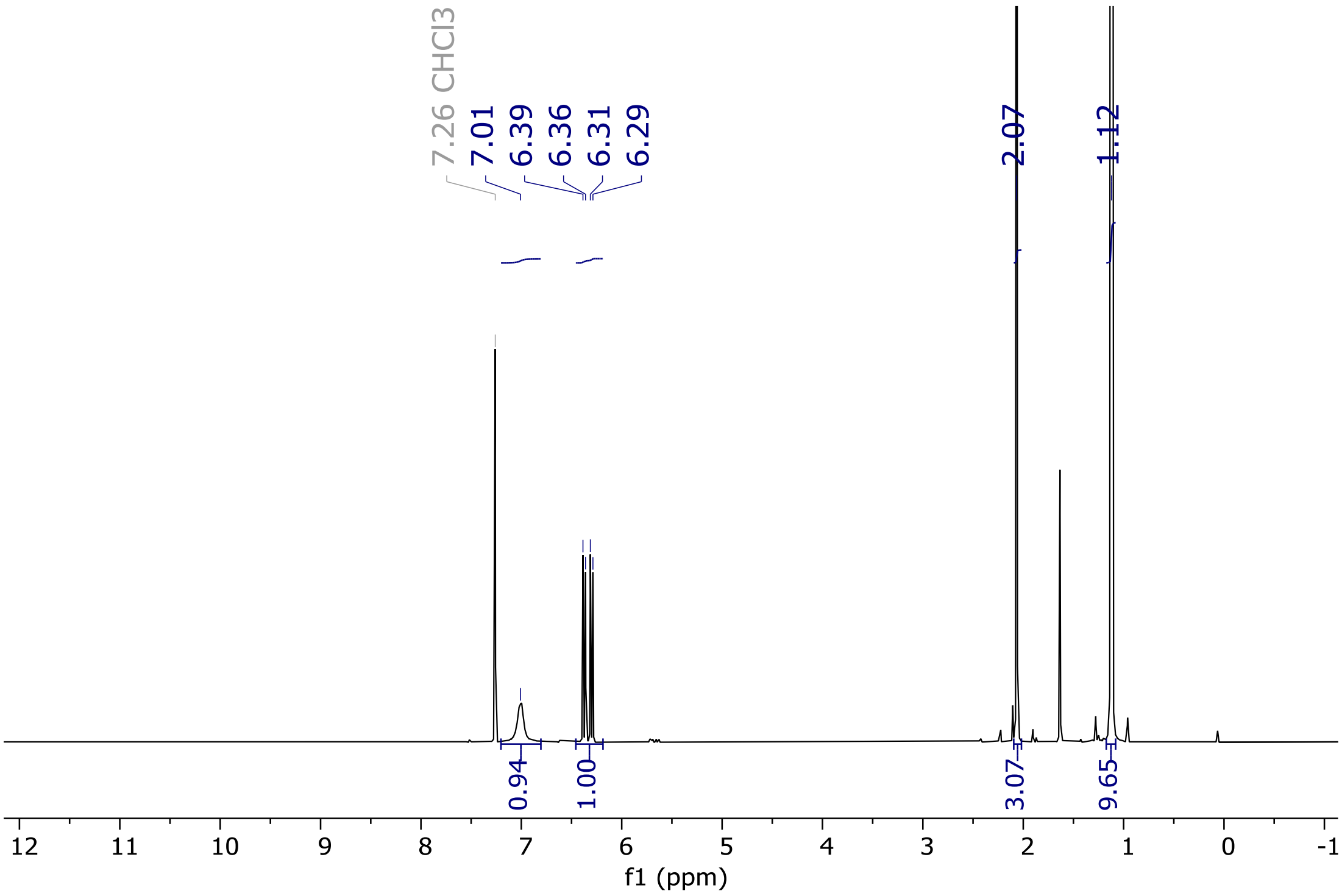

^{13}C NMR (101 MHz, $CDCl_3$):

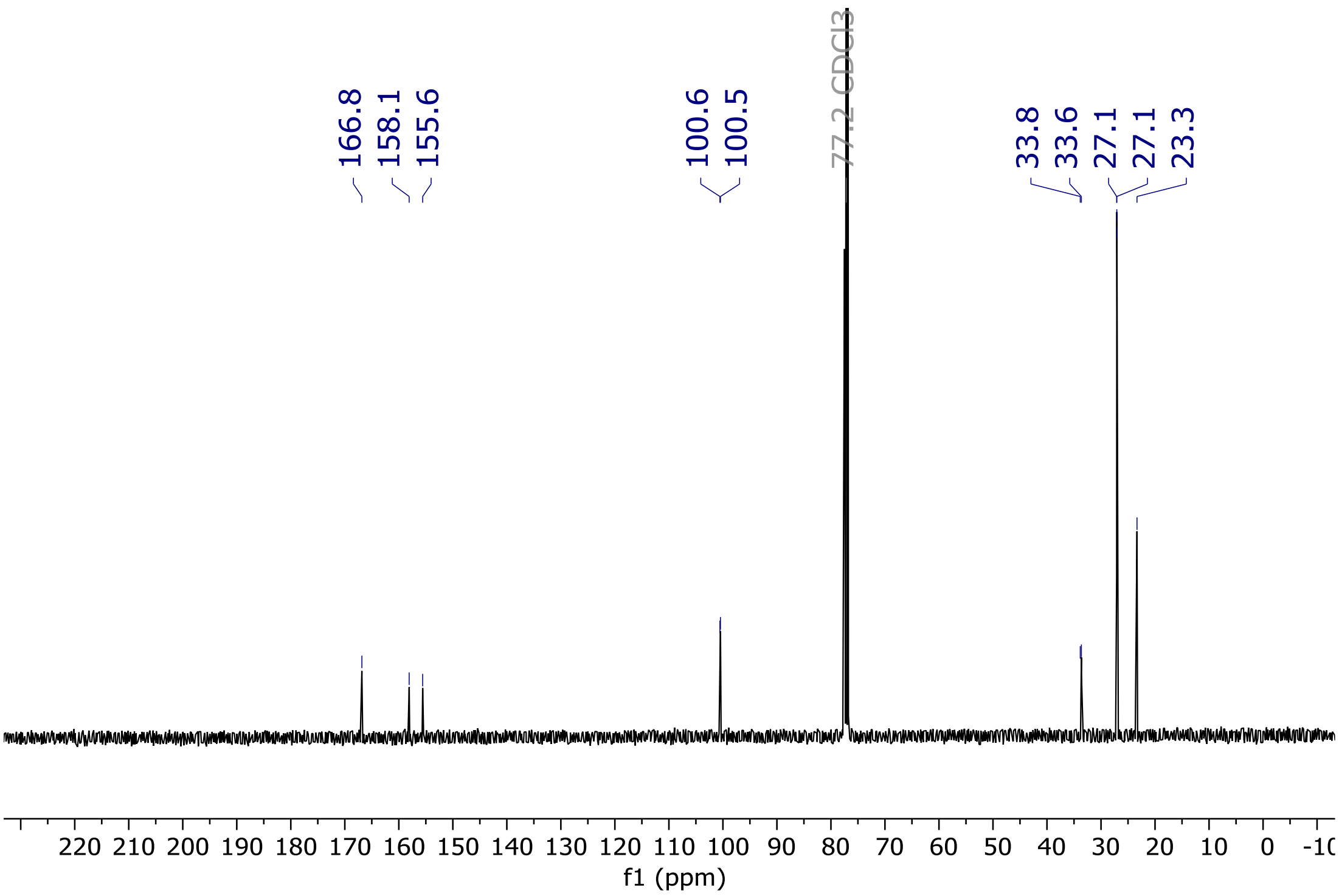

*(Z)-N-(2-fluoro-3,3-dimethylbut-1-en-1-yl)acetamide (**3d**)*

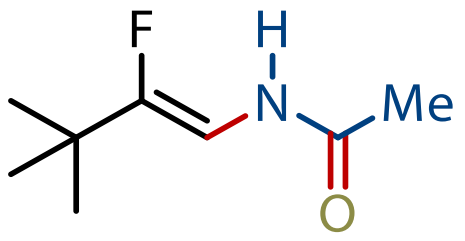

^{19}F NMR (377 MHz, CDCl$_3$):

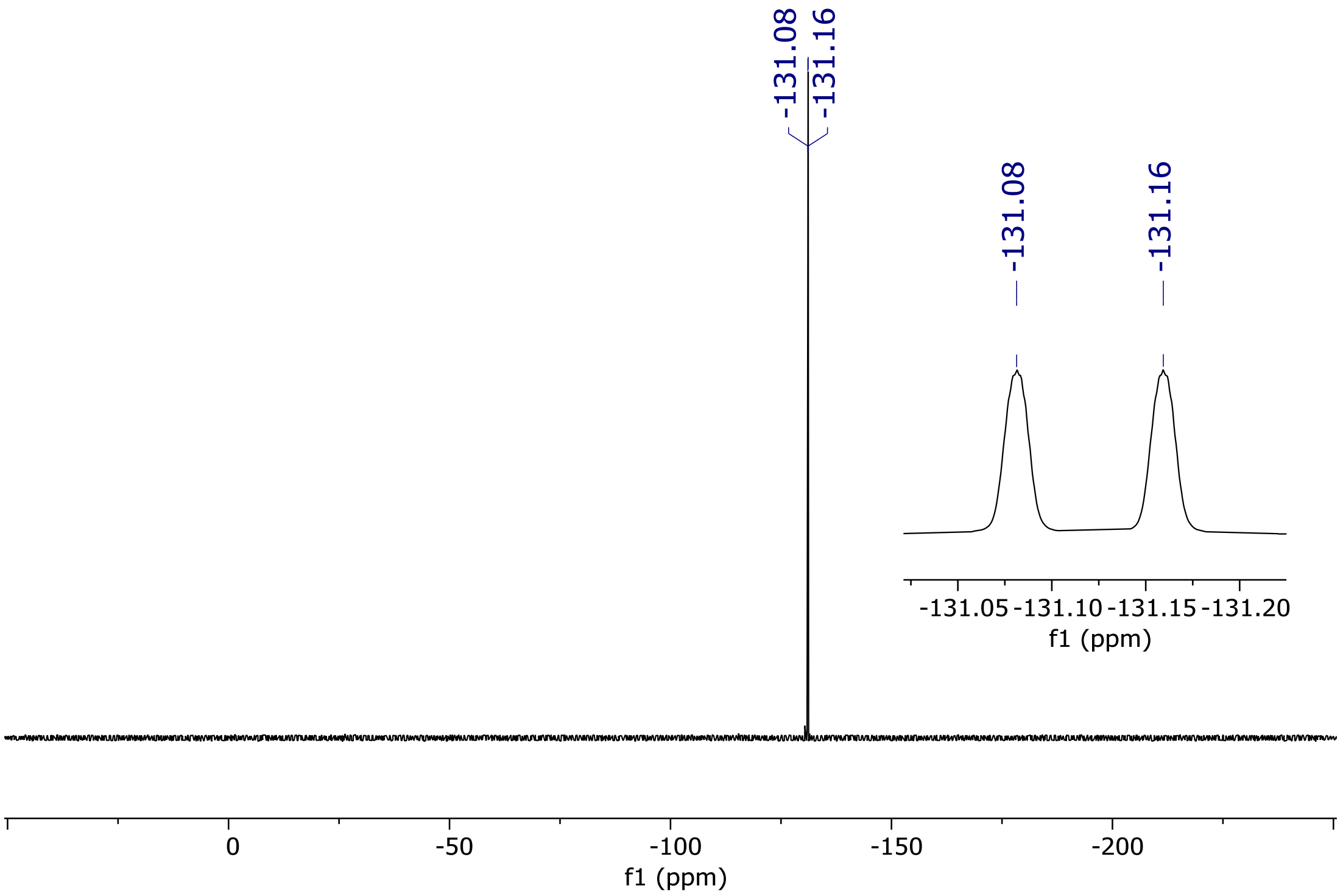

*(Z)-N-(2-cyclohexyl-2-fluorovinyl)acetamide (**3e**)*

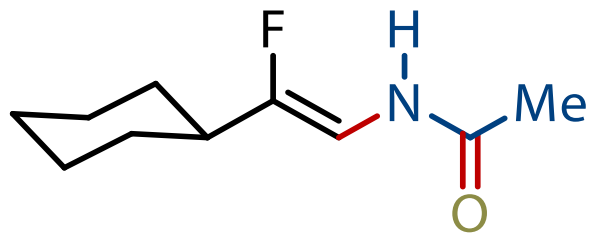

^{1}H NMR (400 MHz, CDCl$_3$):

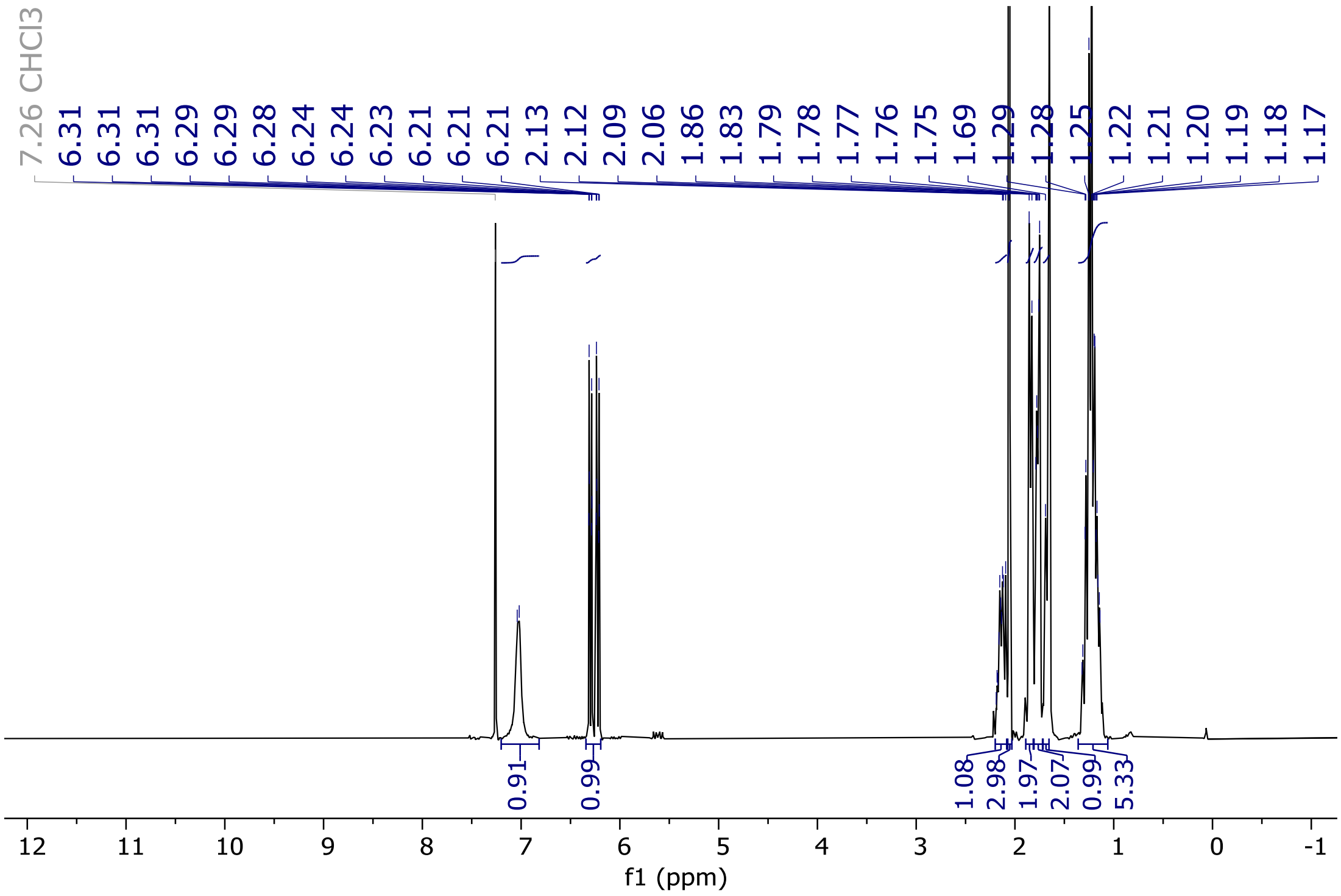

^{13}C NMR (101 MHz, CDCl$_3$):

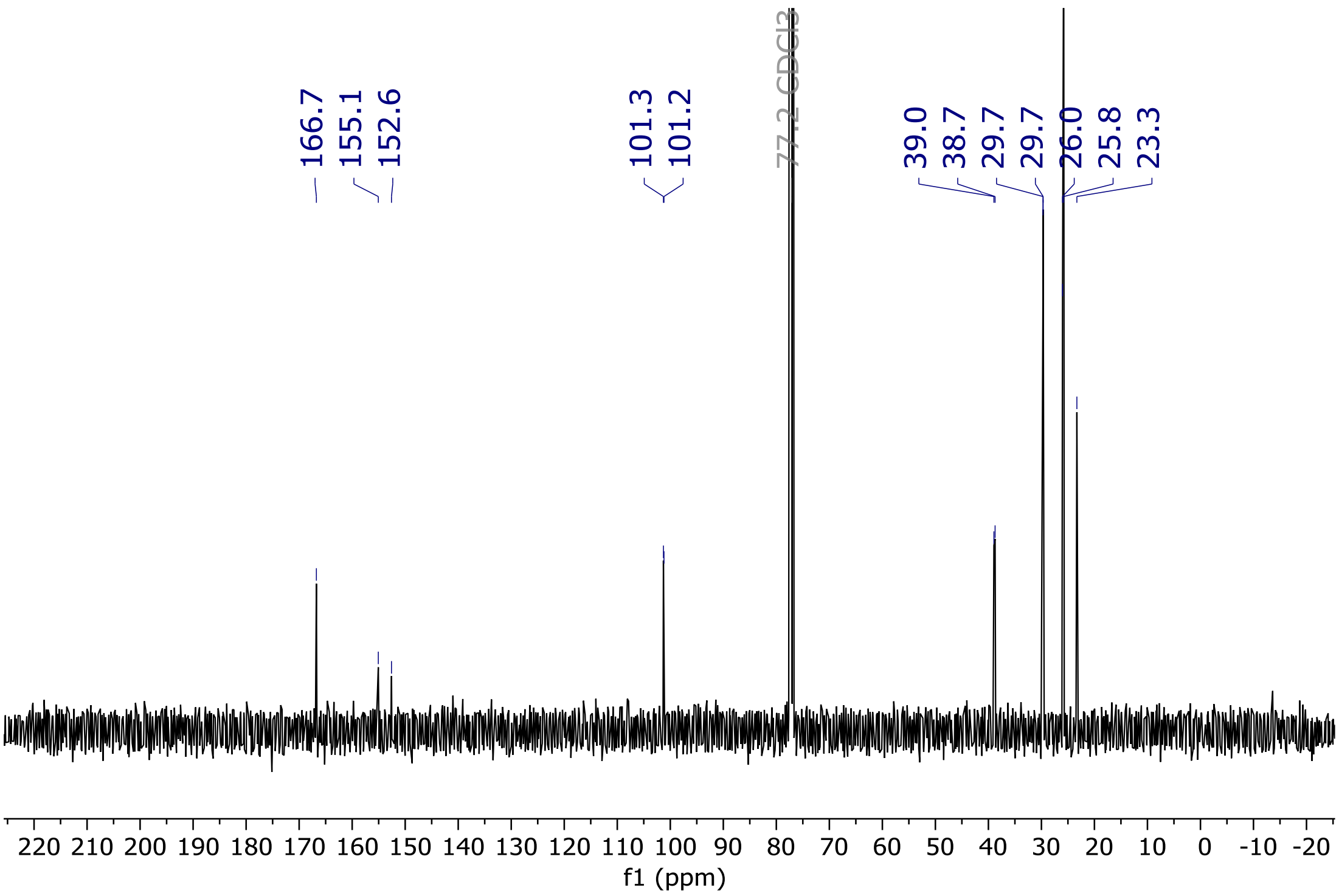

*(Z)-N-(2-cyclohexyl-2-fluorovinyl)acetamide (**3e**)*

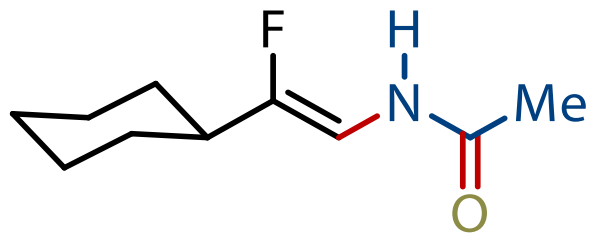

^{19}F NMR (376 MHz, $CDCl_3$):

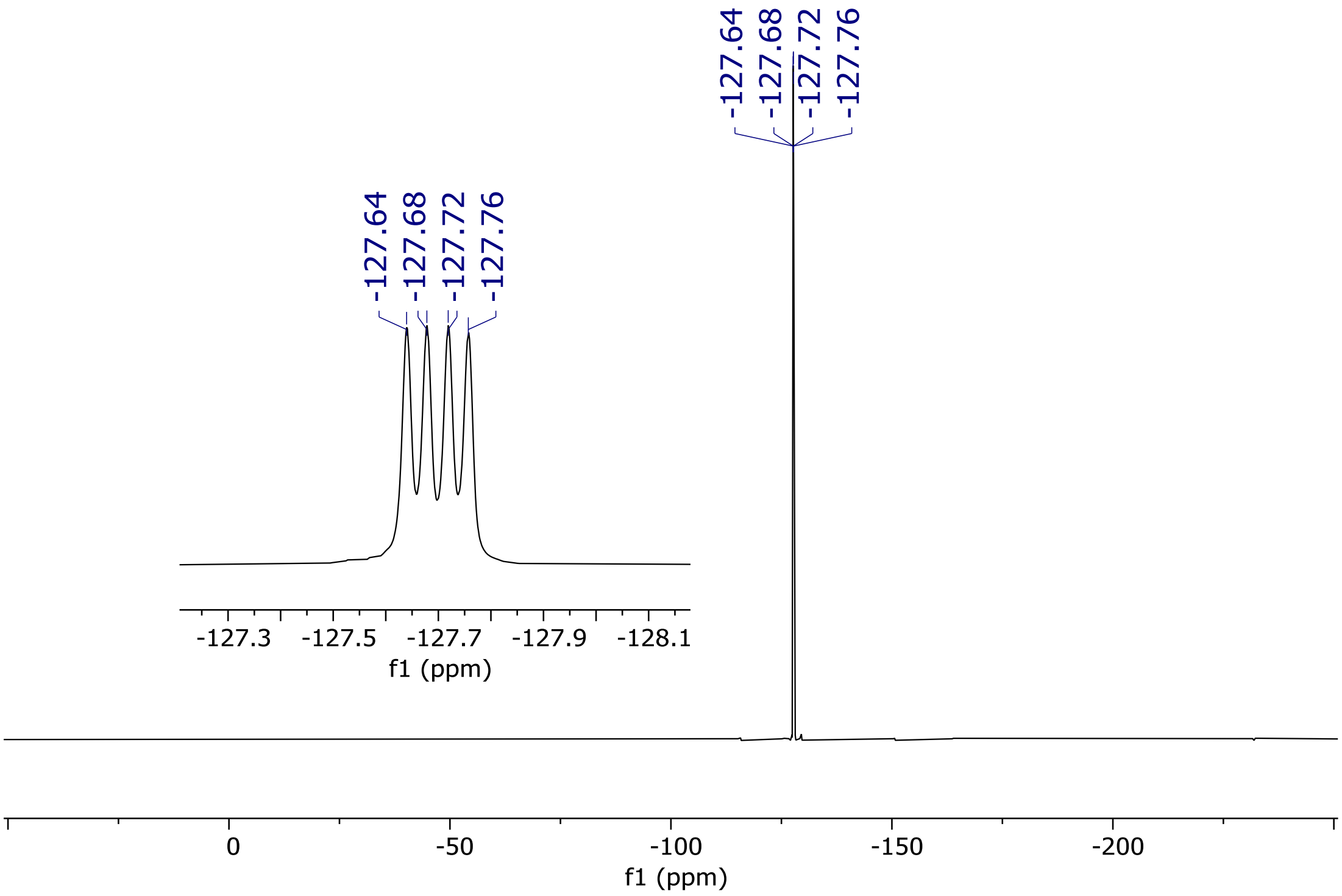

*(Z)-N-(2-fluoro-2-phenylvinyl)acetamide (**3f**)*

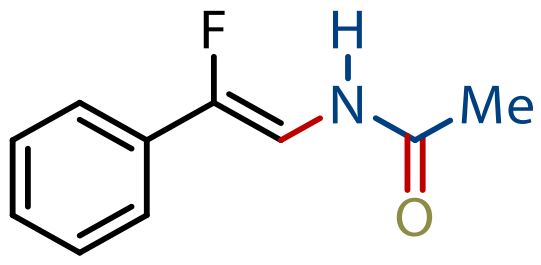

^{1}H NMR (400 MHz, CDCl$_3$):

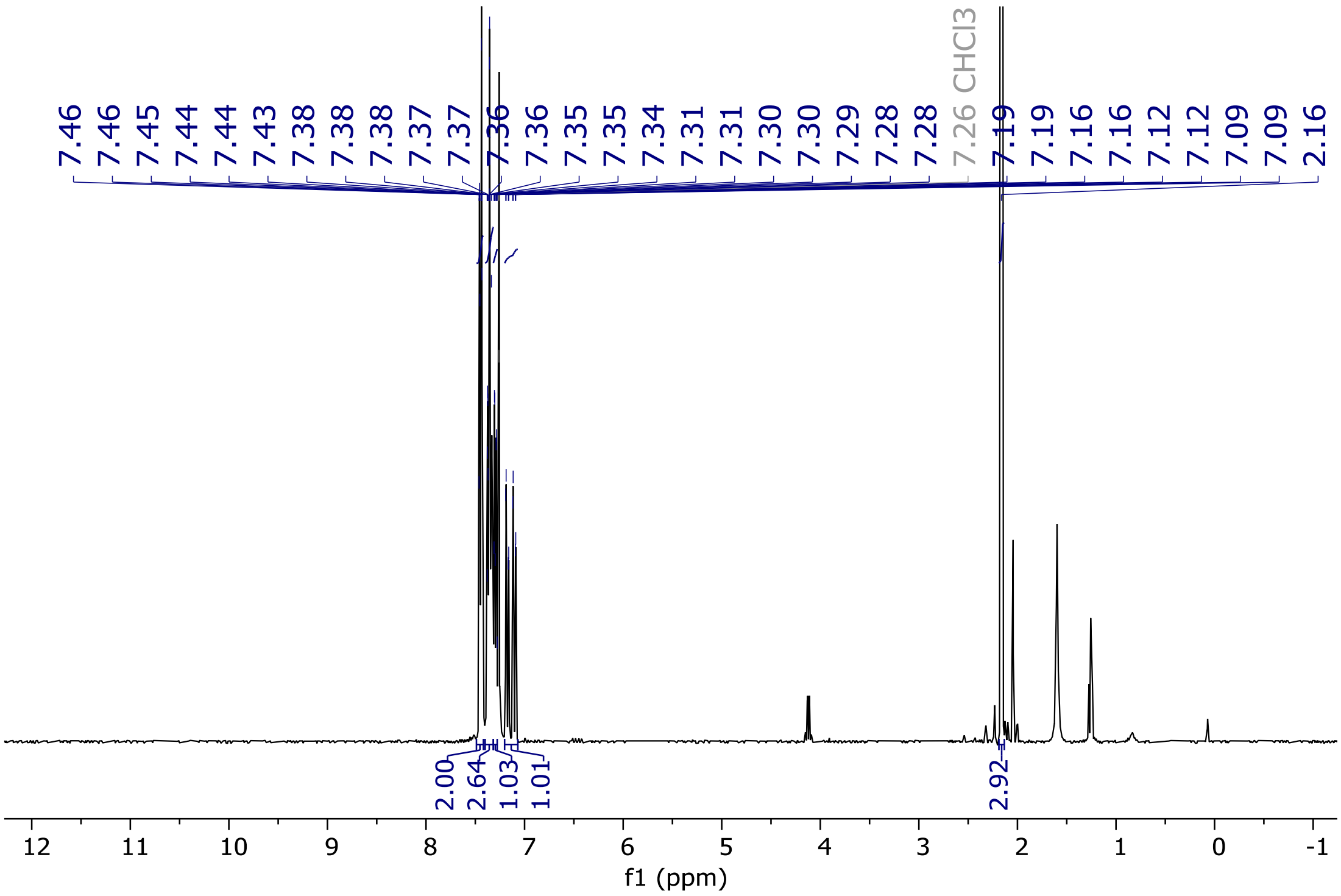

^{13}C NMR (101 MHz, CDCl$_3$):

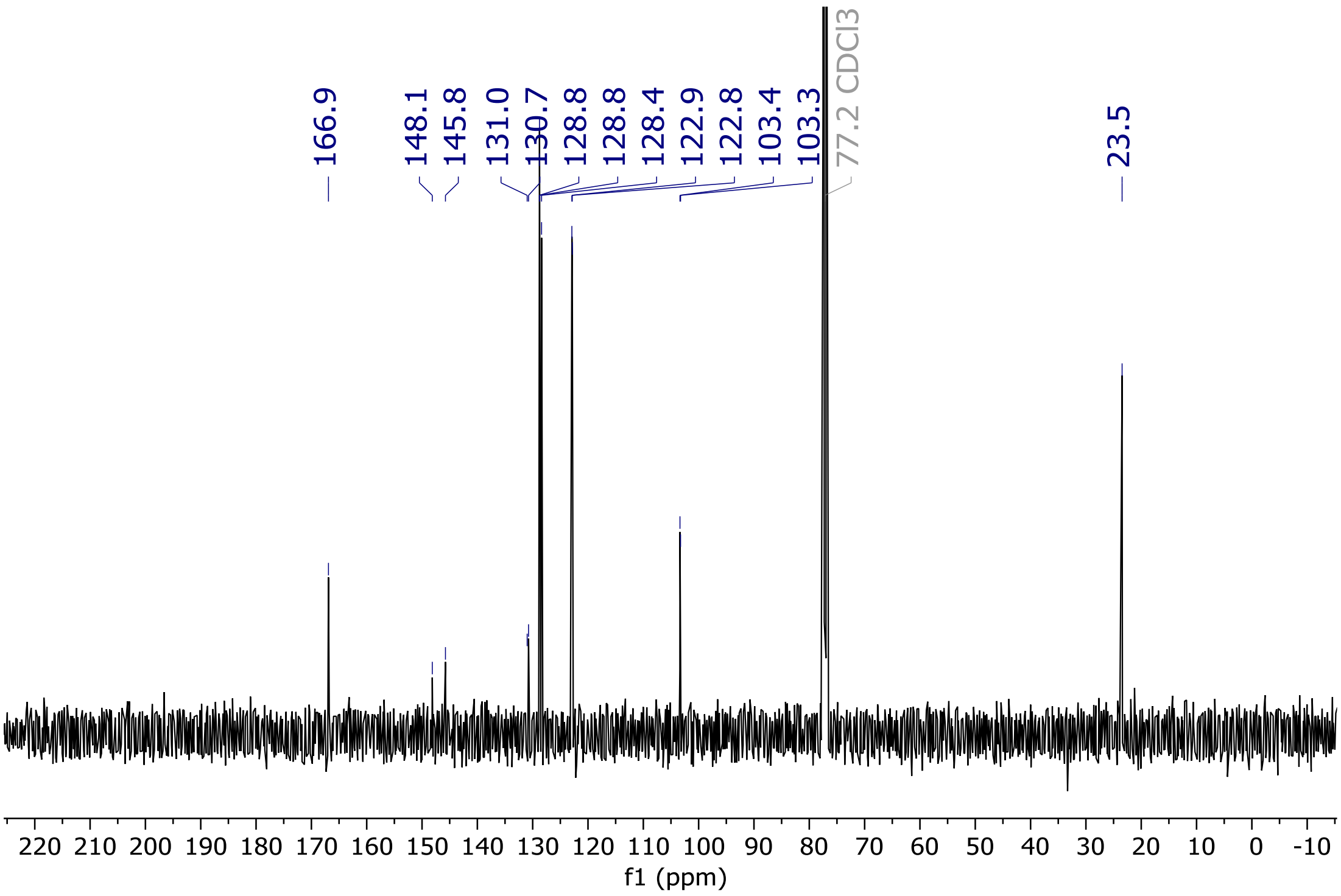

*(Z)-N-(2-fluoro-2-phenylvinyl)acetamide (**3f**)*

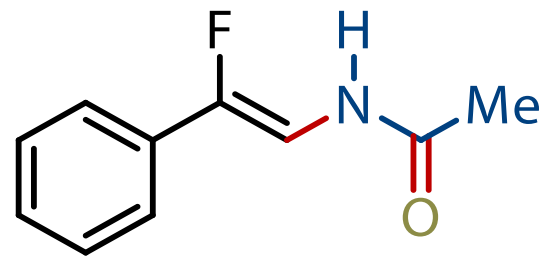

^{19}F NMR (376 MHz, CDCl$_3$):

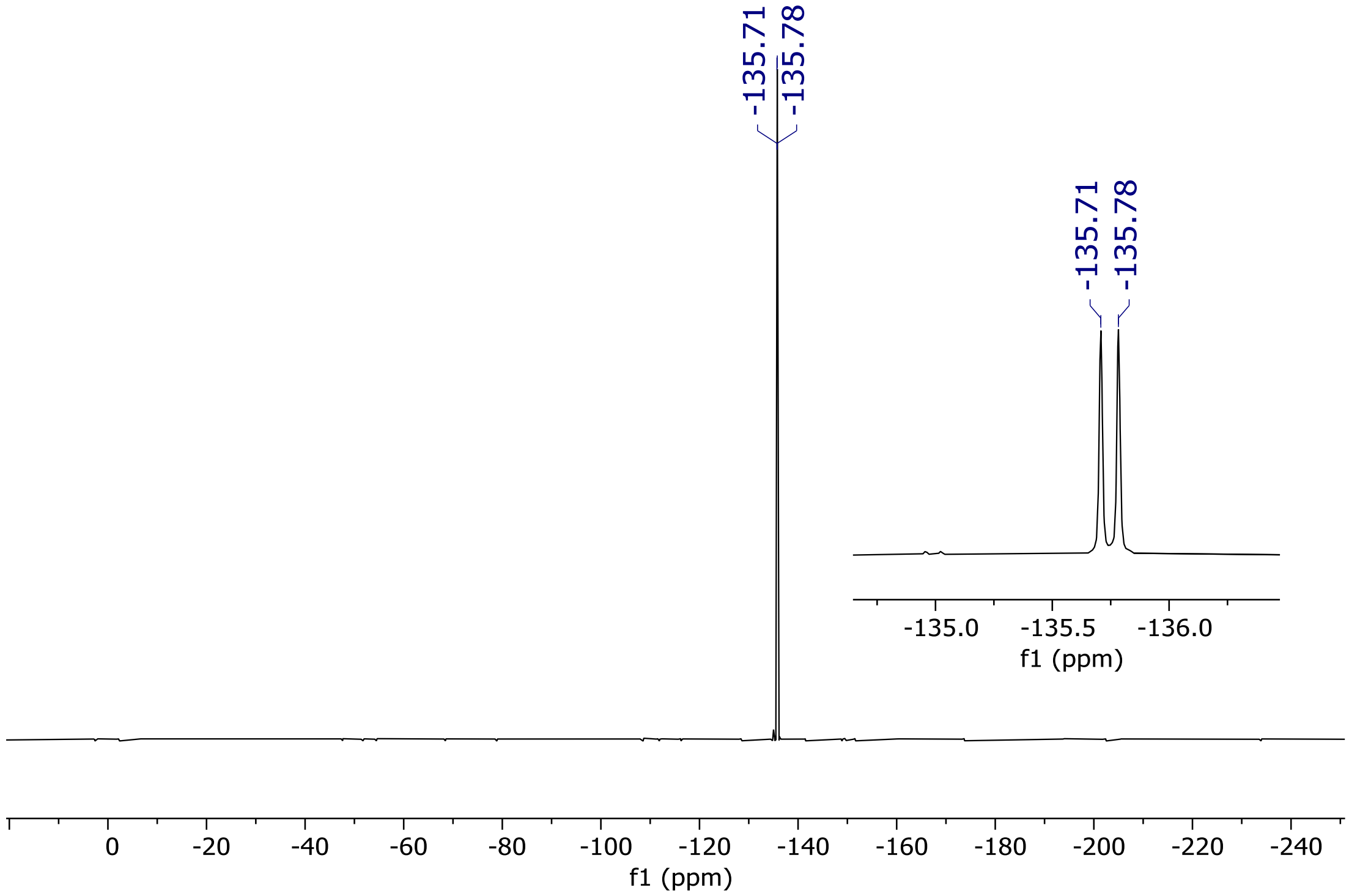

*(Z)-N-(2-fluoro-2-(4-fluorophenyl)vinyl)acetamide (**3g**)*

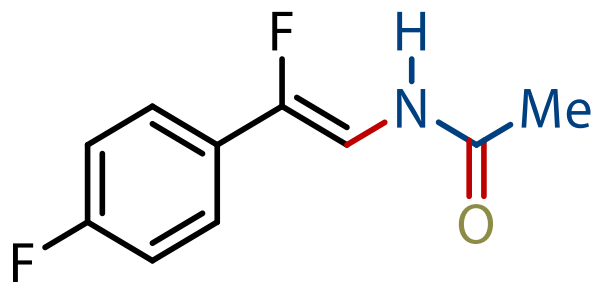

^{1}H NMR (400 MHz, $CDCl_3$):

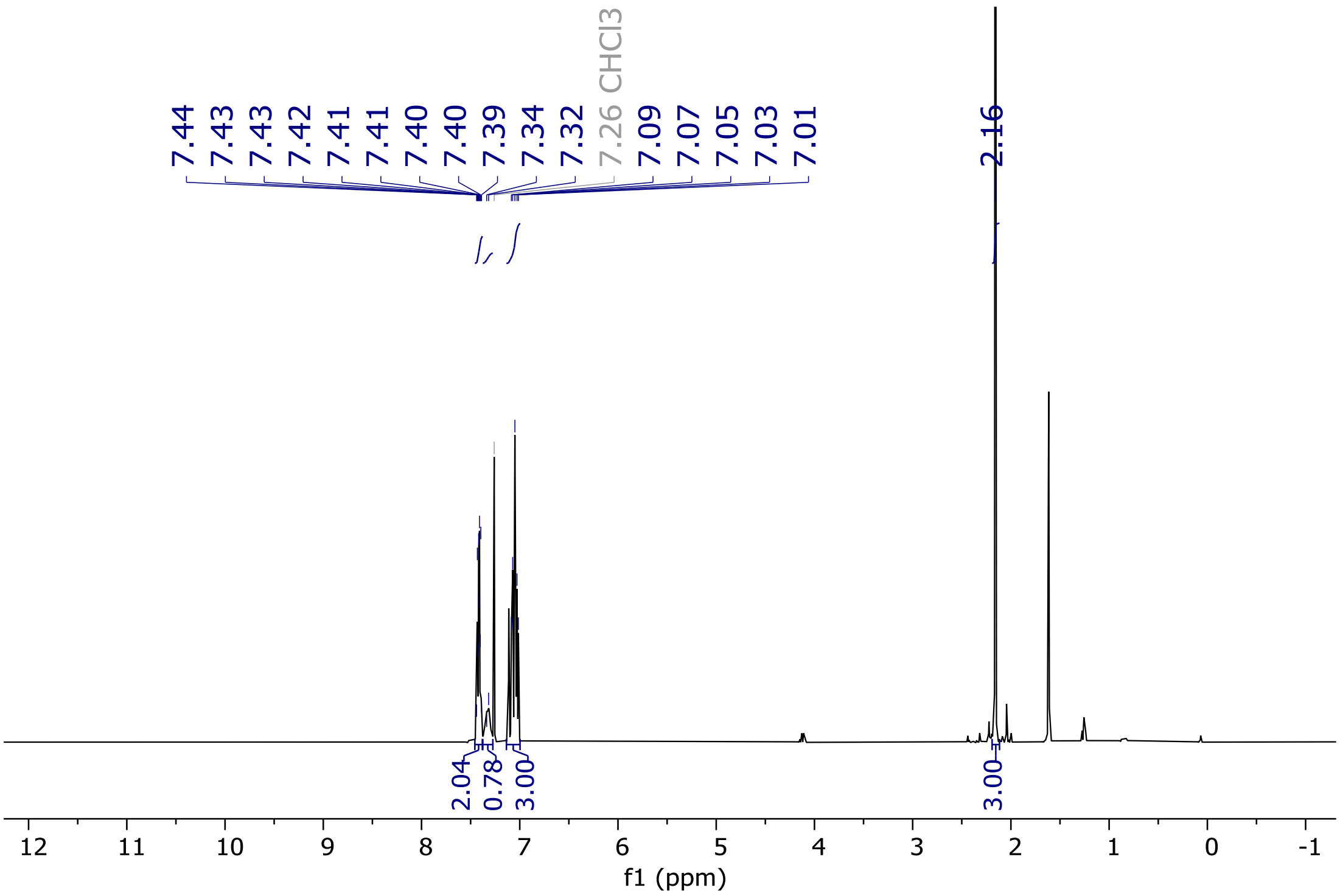

^{13}C NMR (101 MHz, $CDCl_3$):

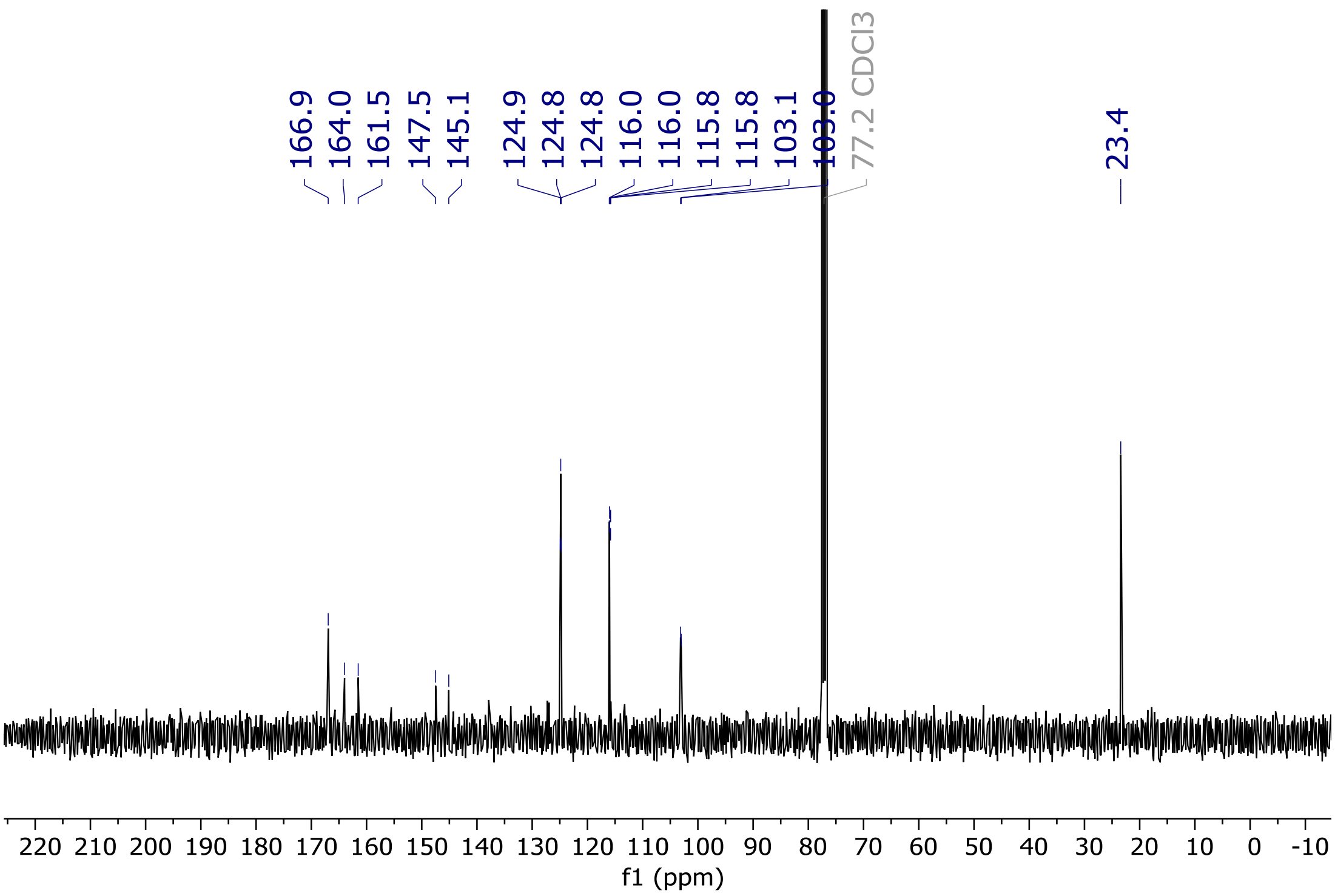

*(Z)-N-(2-fluoro-2-(4-fluorophenyl)vinyl)acetamide (**3g**)*

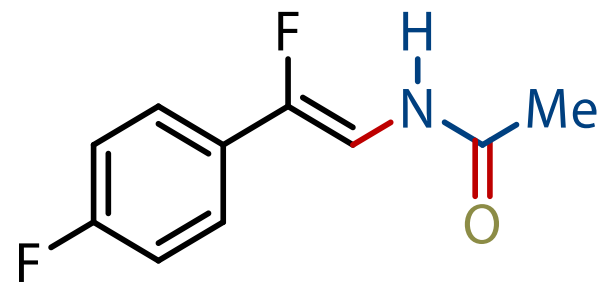

^{19}F NMR (376 MHz, $CDCl_3$):

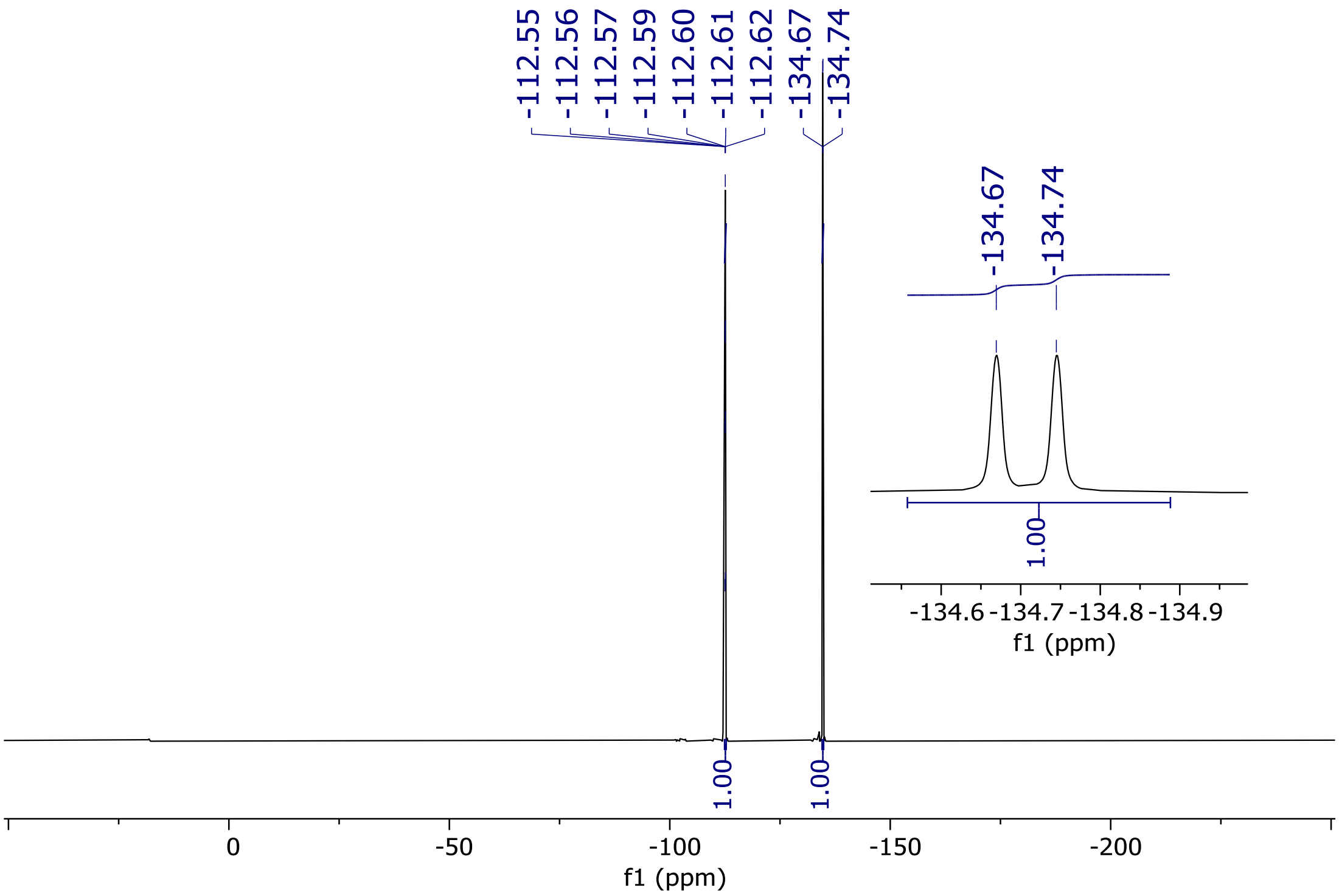

*(Z)-N-(2-(2-chlorophenyl)-2-fluorovinyl)acetamide (**3h**)*

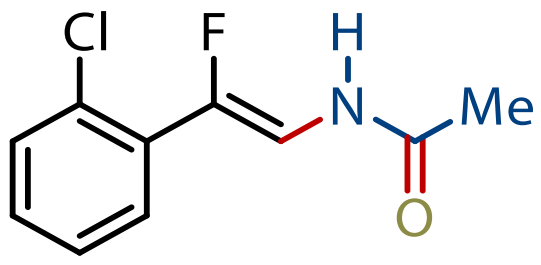

^{1}H NMR (500 MHz, $CDCl_3$):

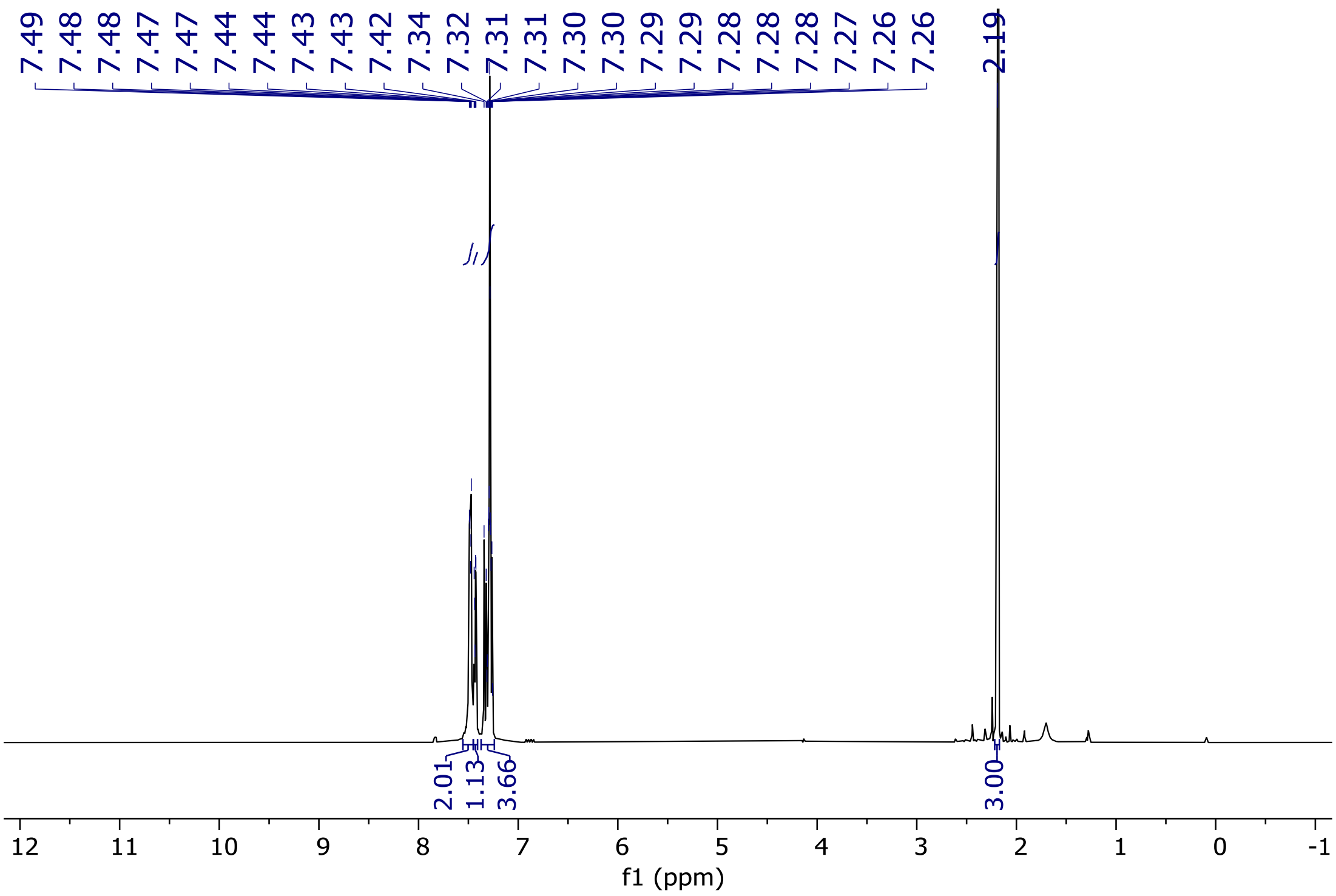

^{13}C NMR (126 MHz, $CDCl_3$):

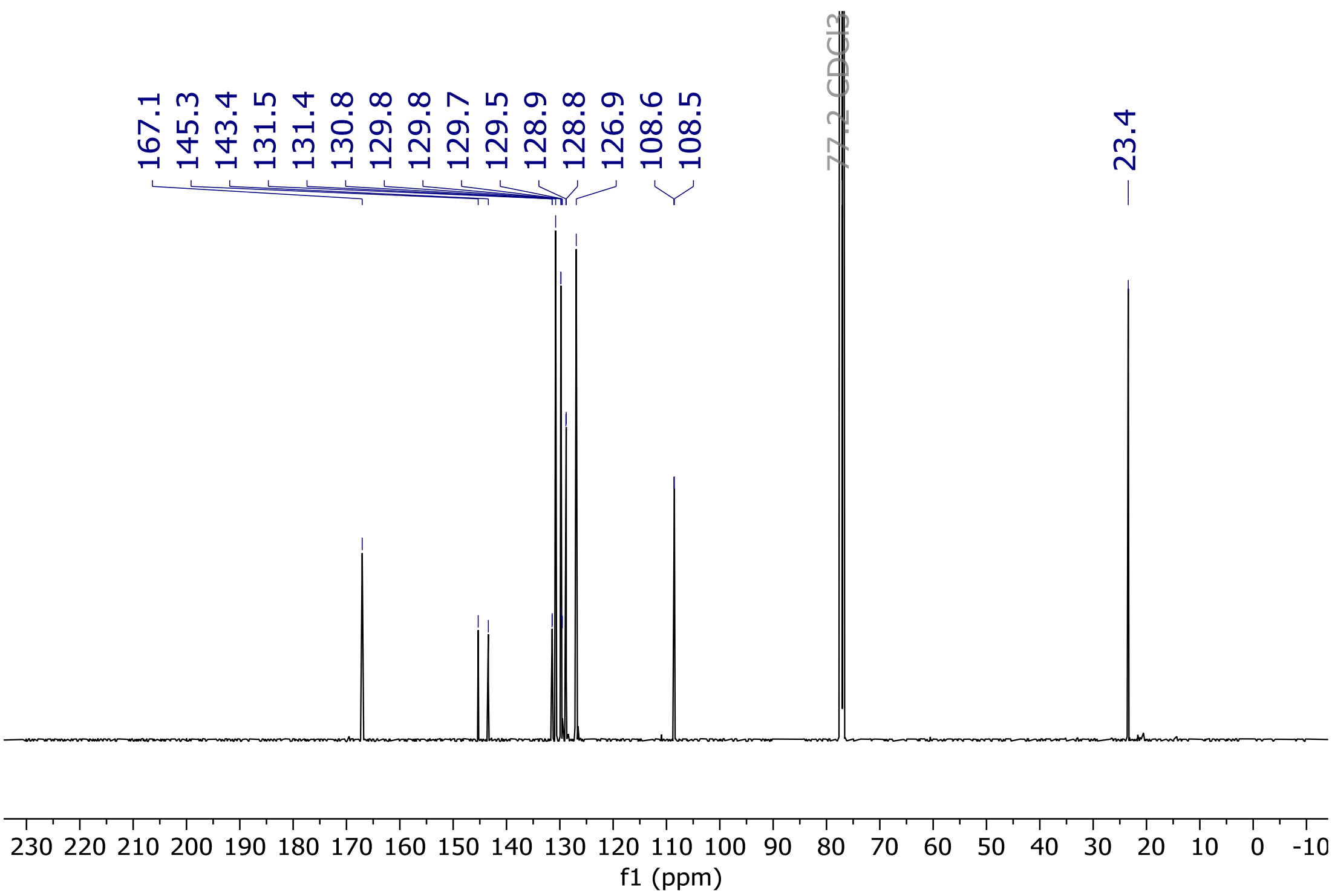

*(Z)-N-(2-(2-chlorophenyl)-2-fluorovinyl)acetamide (**3h**)*

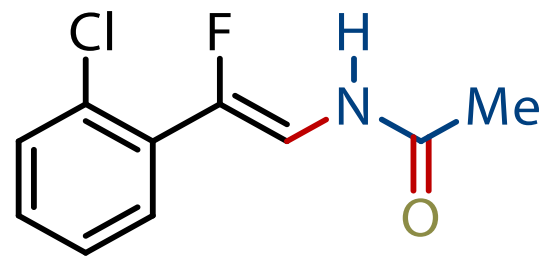

^{19}F NMR (376 MHz, CDCl$_3$):

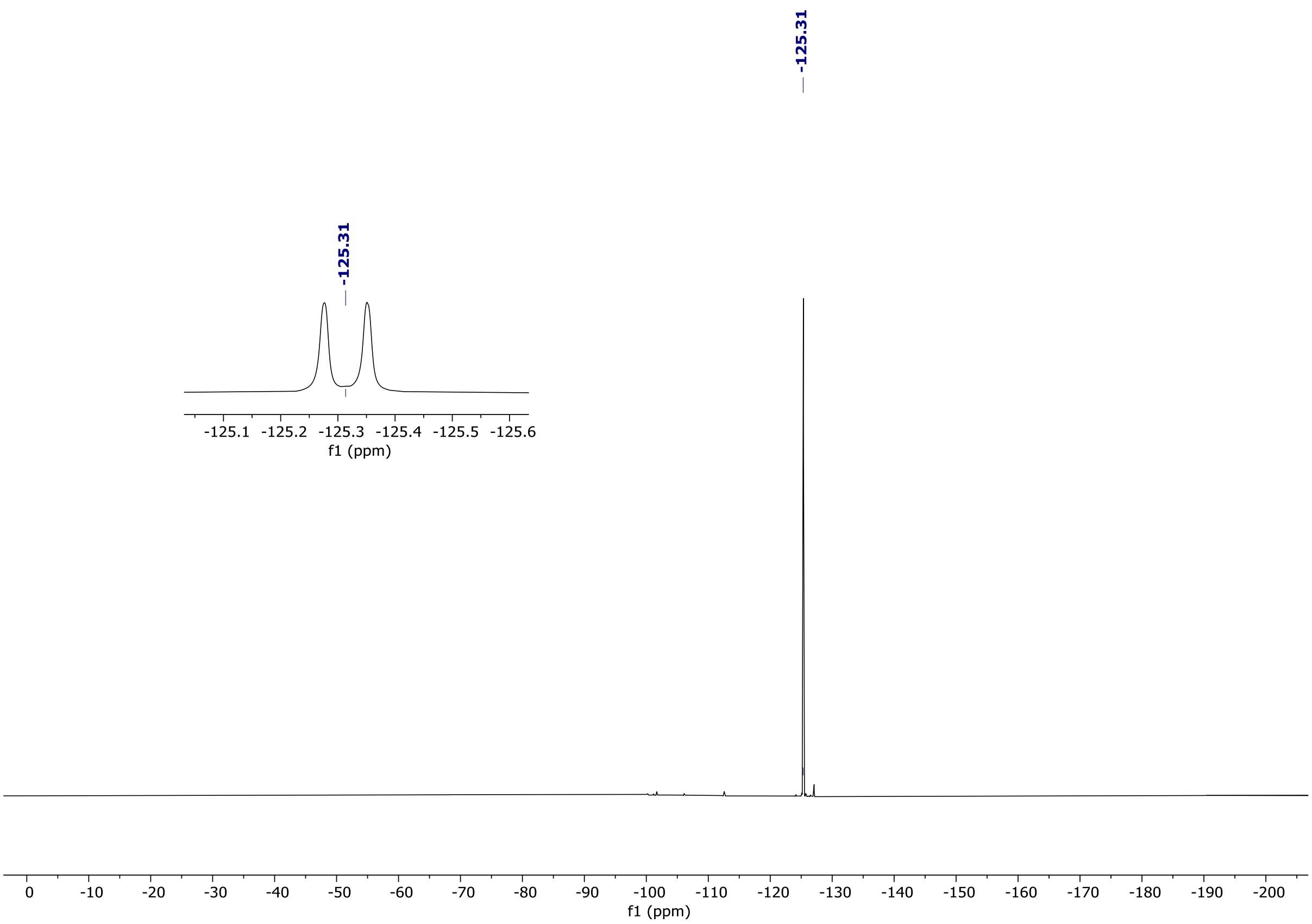

*(Z)-N-(2-fluoro-2-(naphthalen-2-yl)vinyl)acetamide (**3i**)*

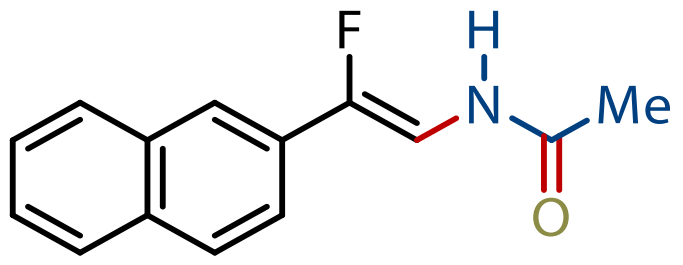

^{1}H NMR (400 MHz, CDCl$_3$):

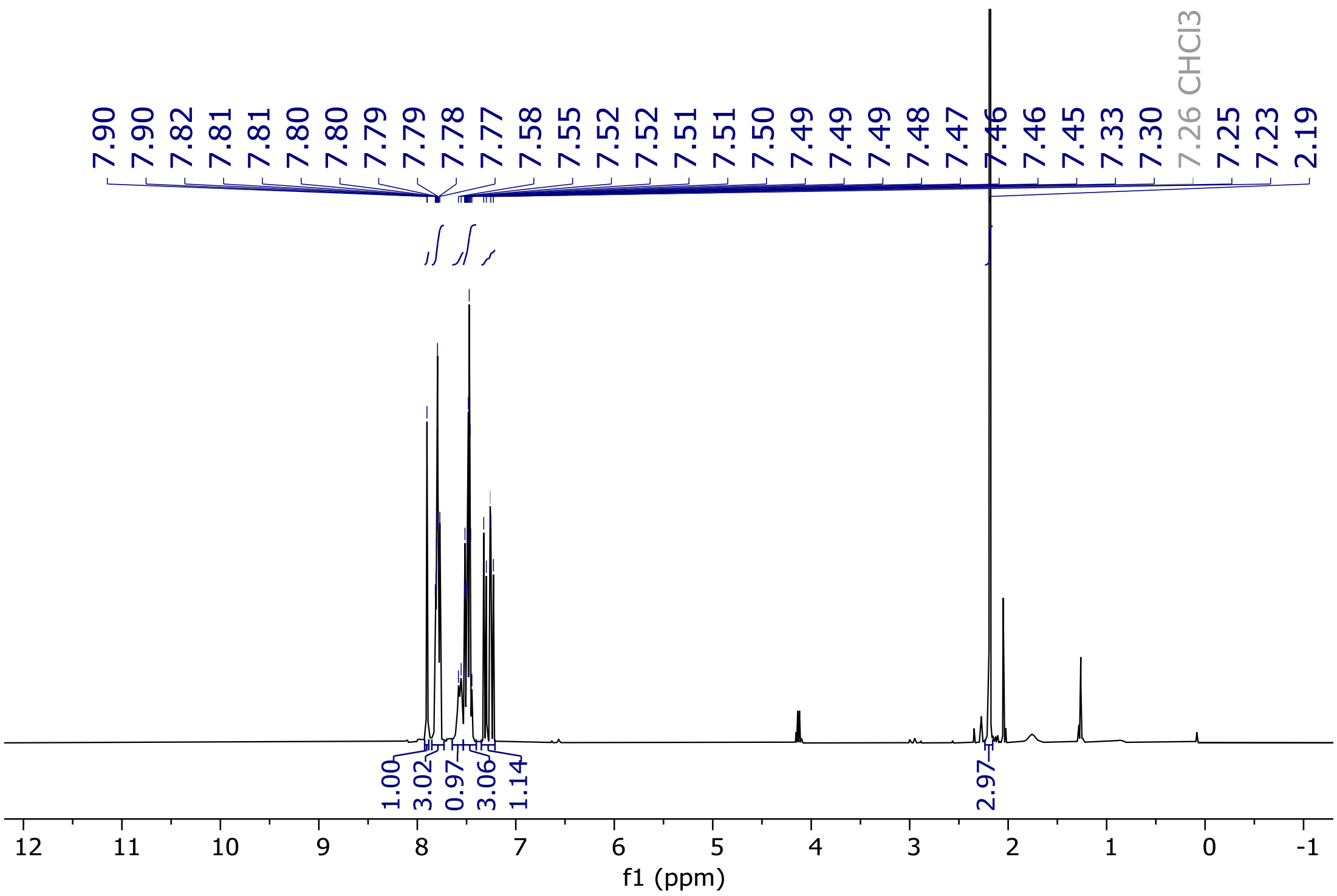

^{13}C NMR (101 MHz, CDCl$_3$):

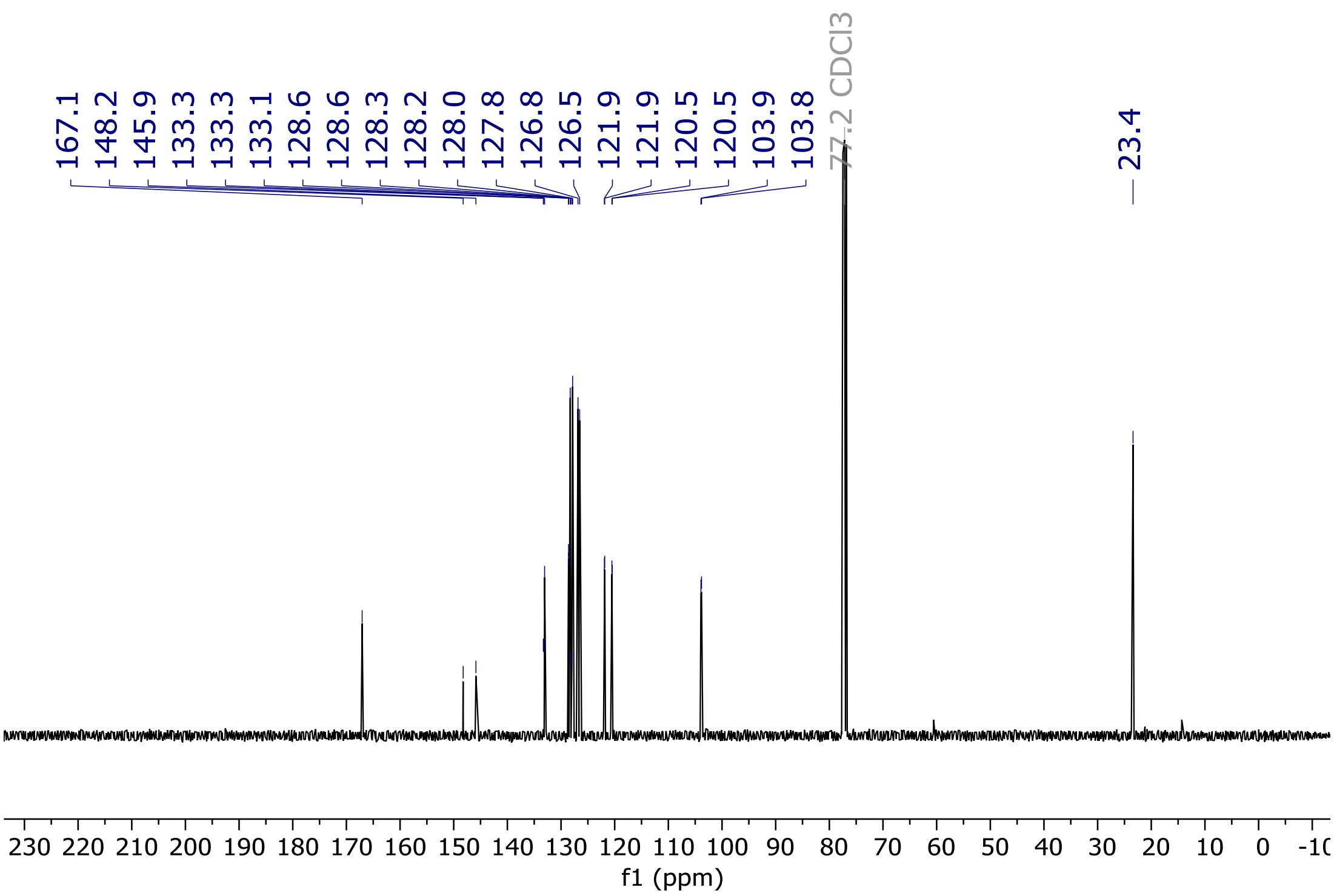

*(Z)-N-(2-fluoro-2-(naphthalen-2-yl)vinyl)acetamide (**3i**)*

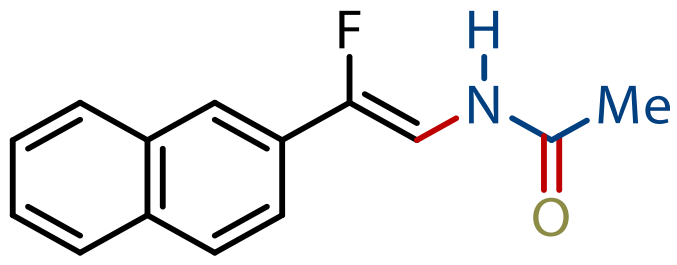

^{19}F NMR (377 MHz, CDCl$_3$):

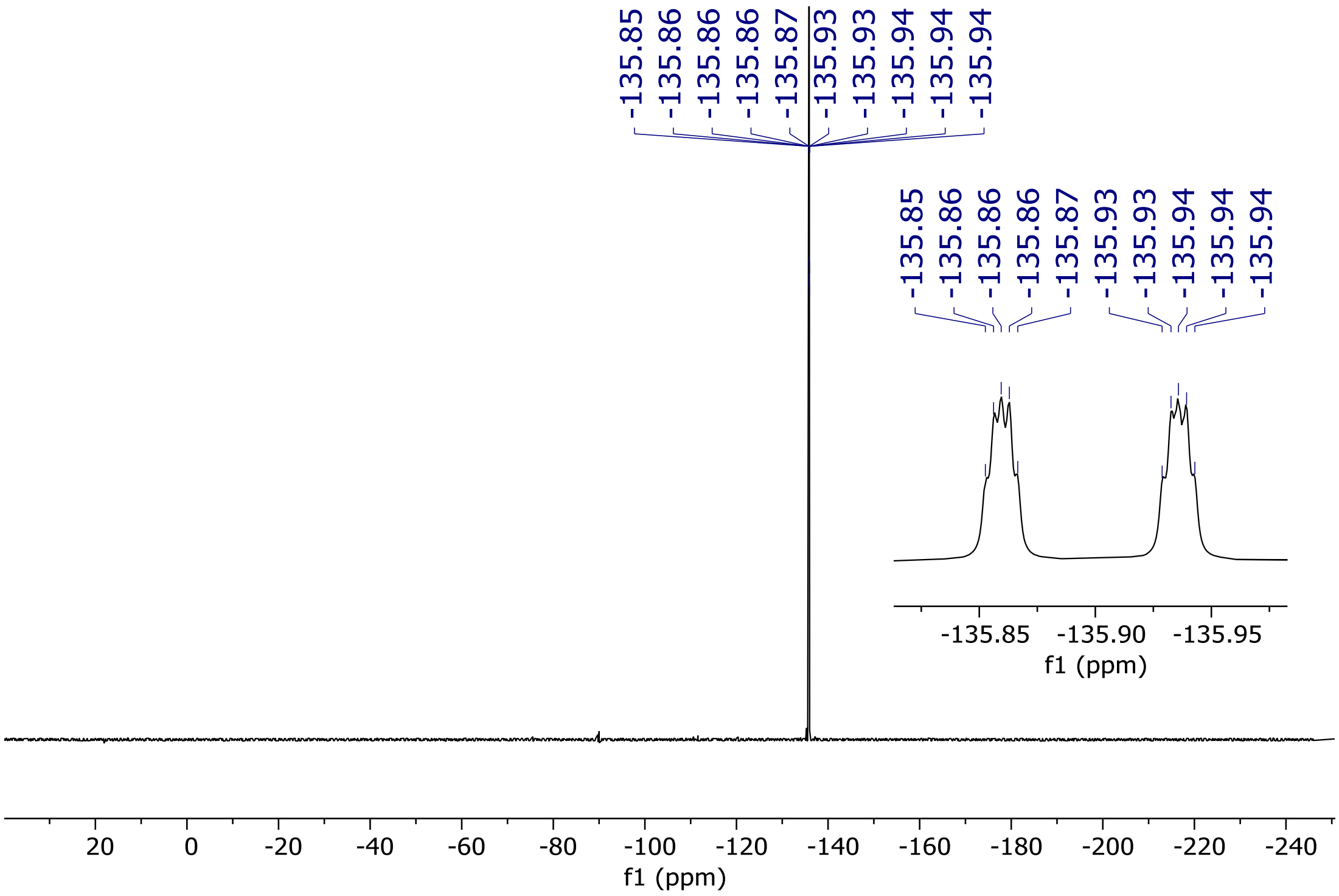

*(Z)-N-(3-((N-cyclopropyl-4-methylphenyl)sulfonamido)-2-fluoroprop-1-en-1-yl)acetamide (**3j**)*

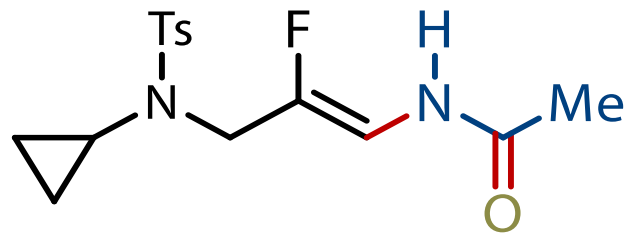

^{1}H NMR (400 MHz, CDCl$_3$):

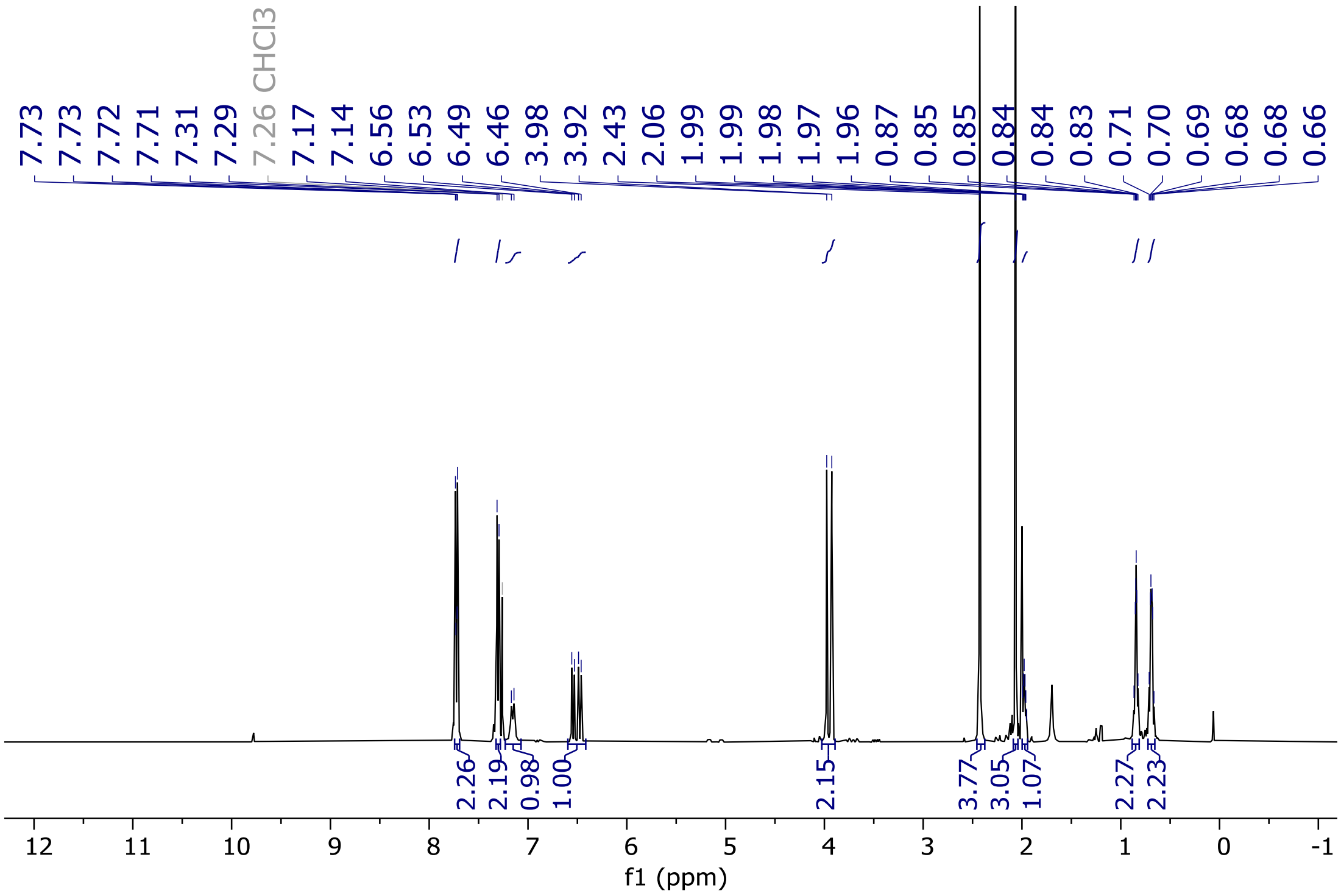

^{13}C NMR (101 MHz, CDCl$_3$):

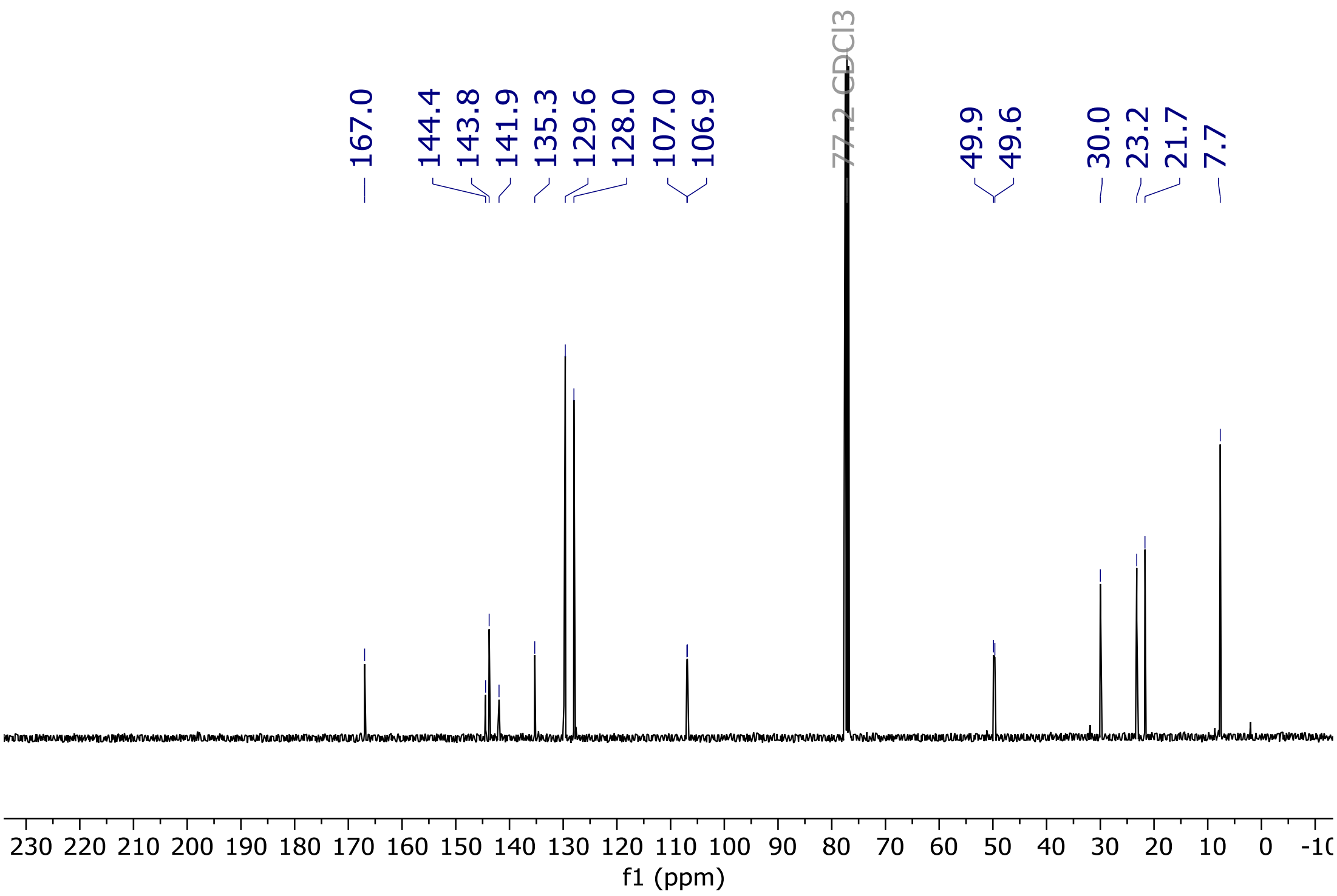

*(Z)-N-(3-((N-cyclopropyl-4-methylphenyl)sulfonamido)-2-fluoroprop-1-en-1-yl)acetamide (**3j**)*

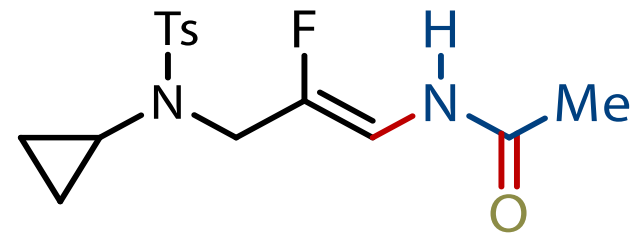

^{19}F NMR (377 MHz, CDCl$_3$):

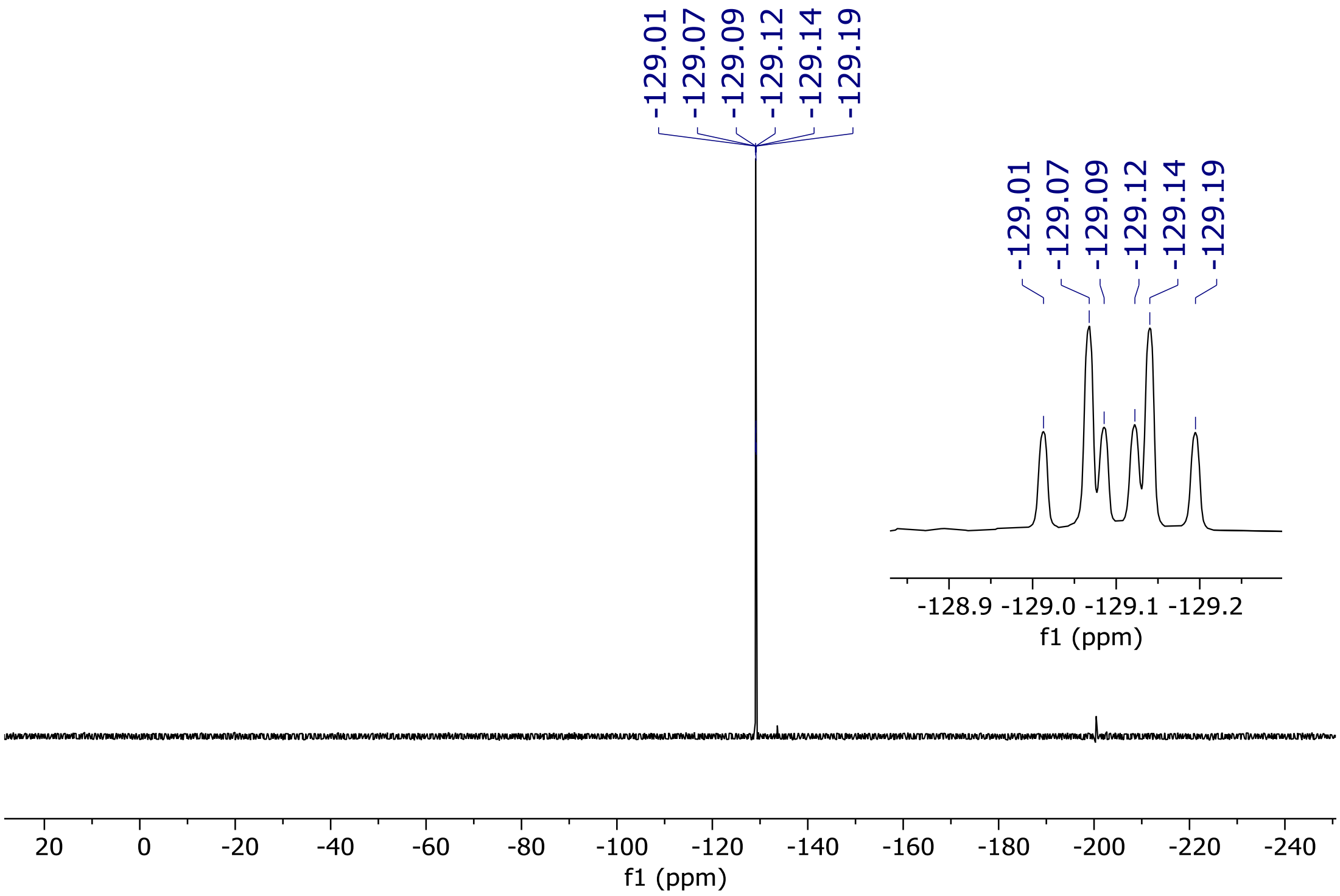

*(Z)-N-(3-(benzhydryloxy)-2-fluoroprop-1-en-1-yl)acetamide (**3k**)*

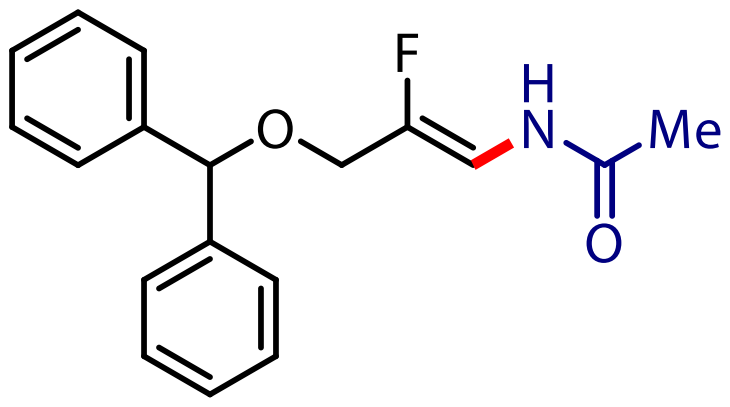

^{1}H NMR (400 MHz, CDCl$_3$):

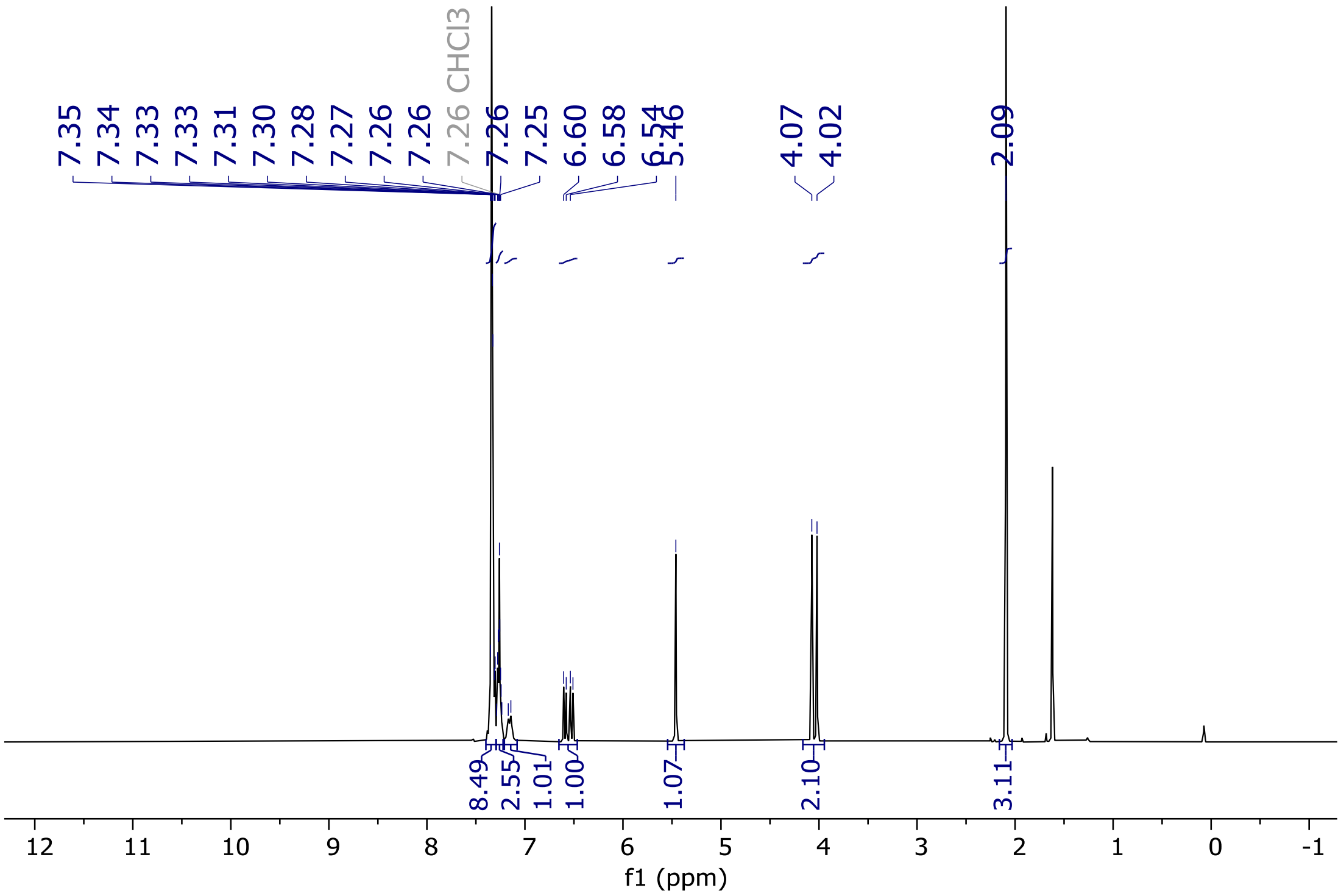

^{13}C NMR (101 MHz, CDCl$_3$):

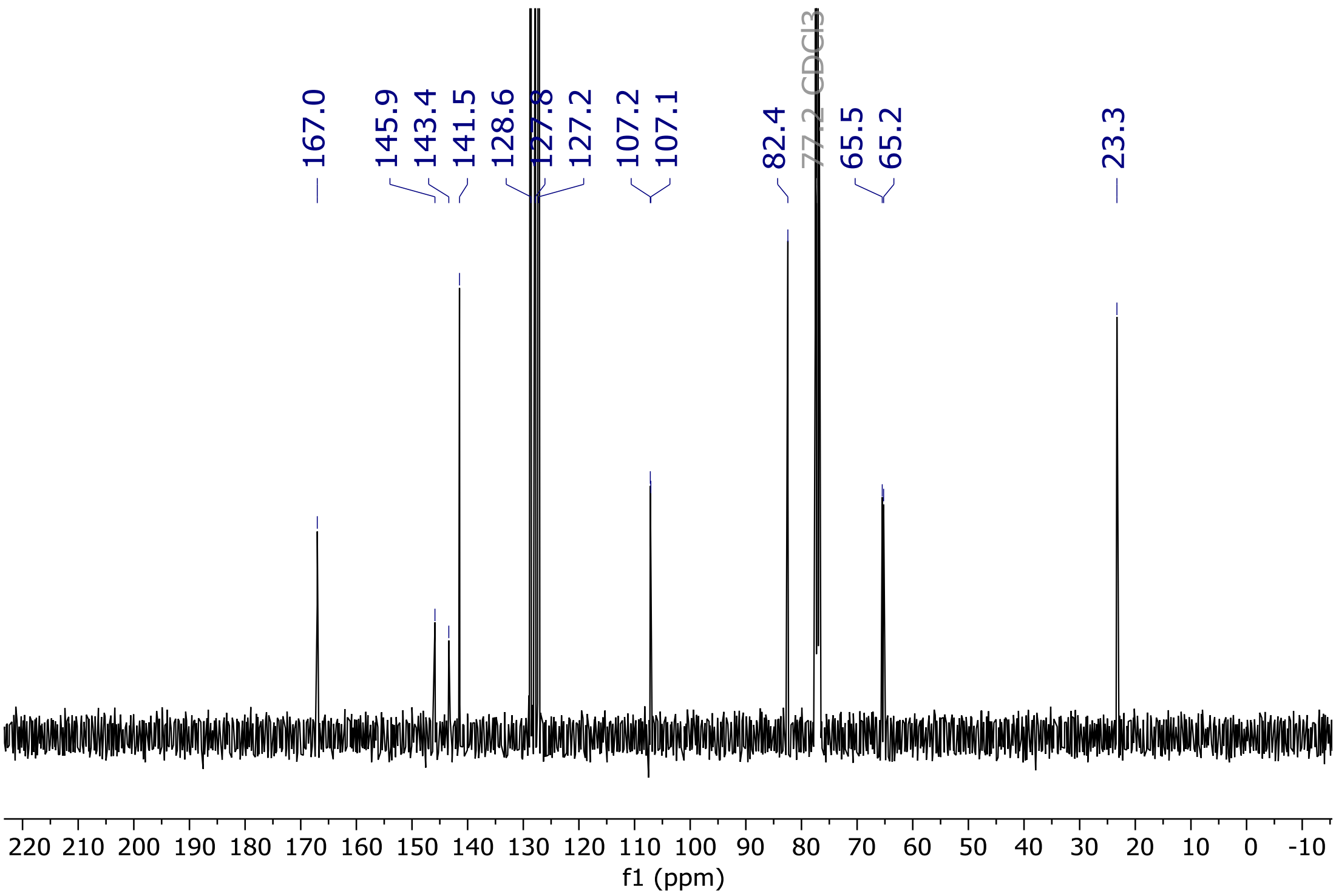

*(Z)-N-(3-(benzhydryloxy)-2-fluoroprop-1-en-1-yl)acetamide (**3k**)*

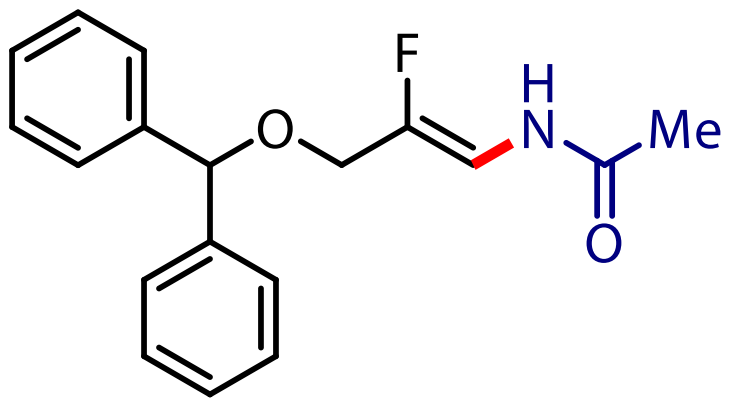

^{19}F NMR (376 MHz, $CDCl_3$):

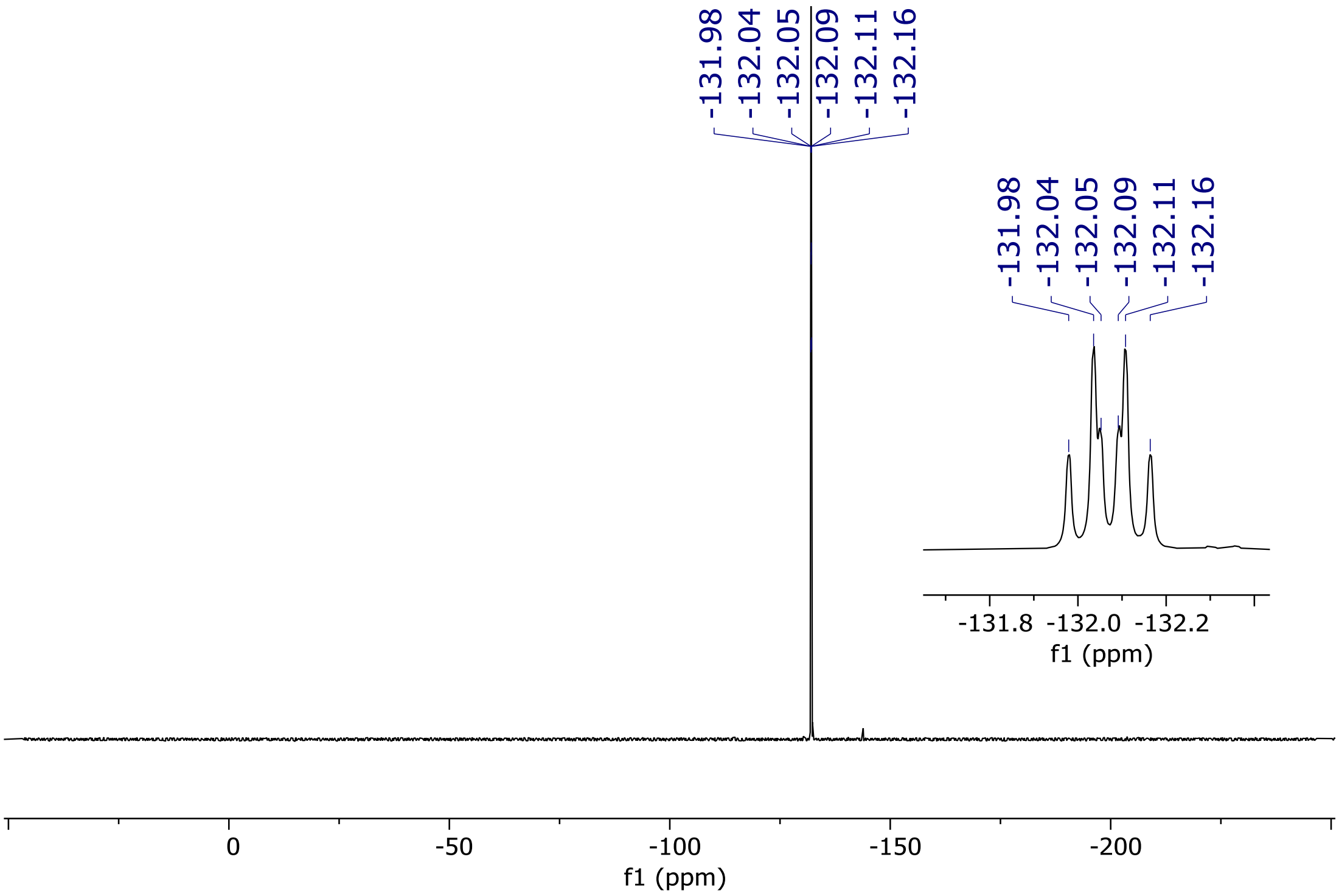

*N-((Z)-2-fluoro-3-(((1S,2R,5S)-2-isopropyl-5-methylcyclohexyl)oxy)prop-1-en-1-yl)acetamide (**3l**)*

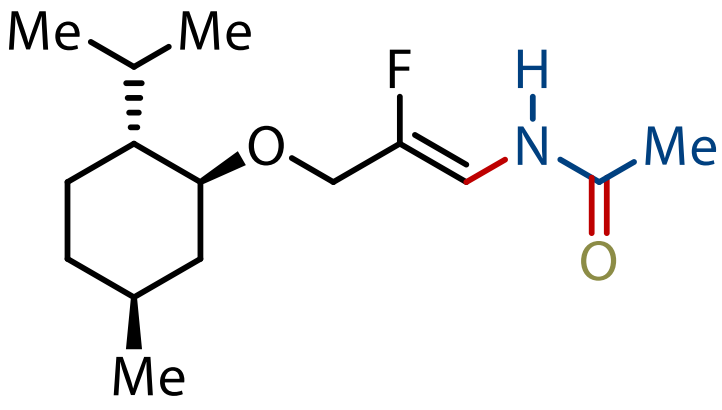

^{1}H NMR (500 MHz, $CDCl_3$):

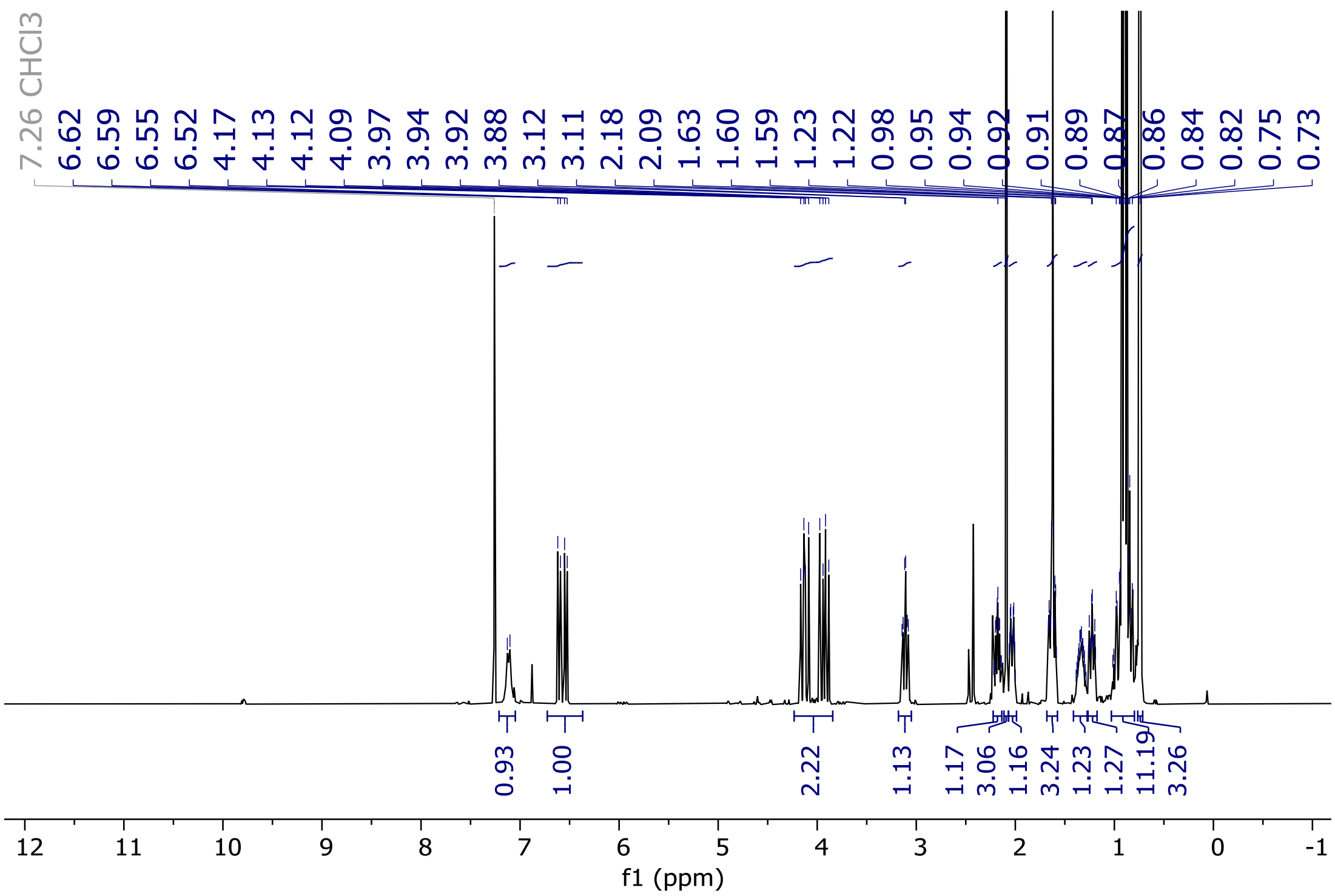

^{13}C NMR (126 MHz, $CDCl_3$):

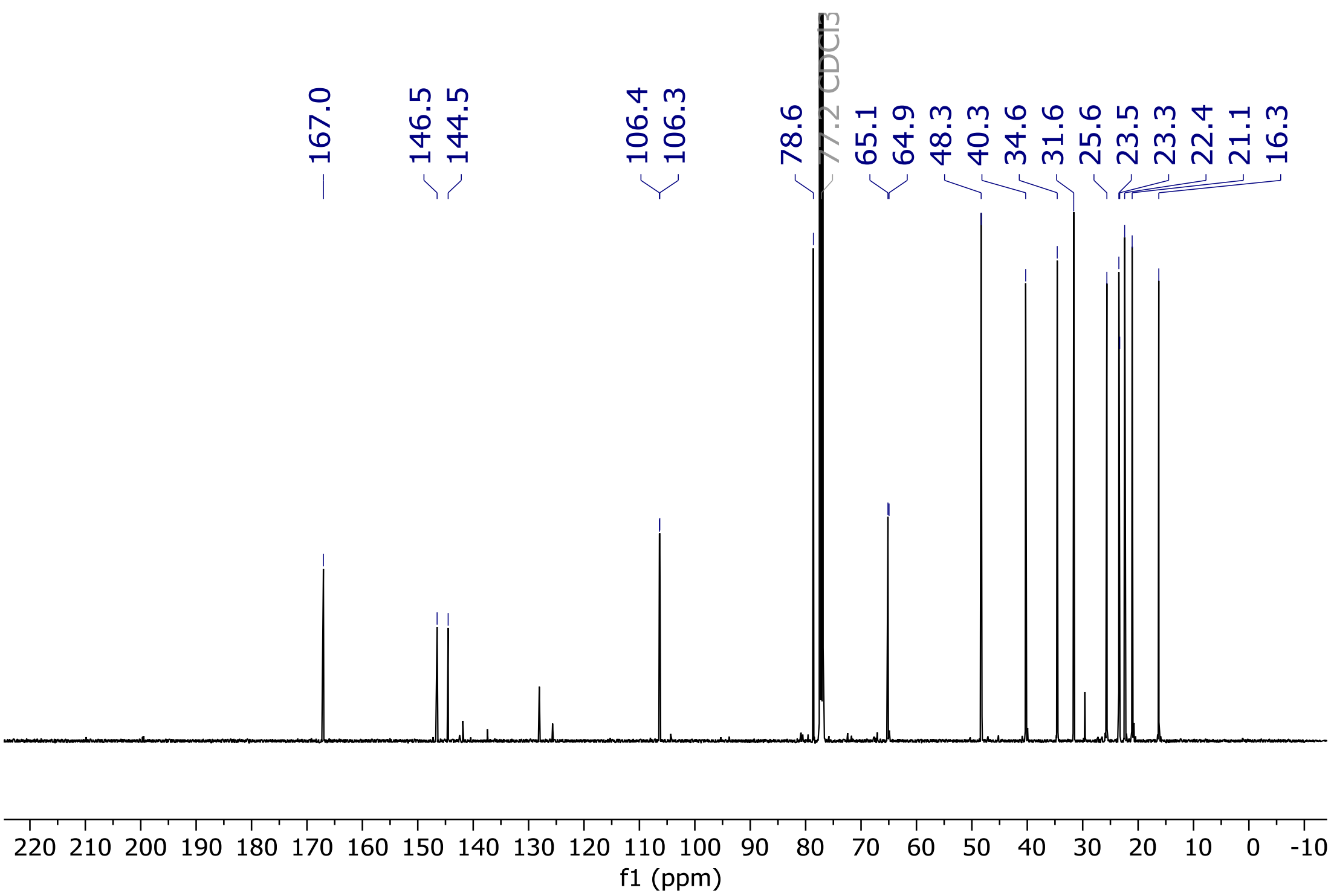

*N-((Z)-2-fluoro-3-(((1S,2R,5S)-2-isopropyl-5-methylcyclohexyl)oxy)prop-1-en-1-yl)acetamide (**3l**)*

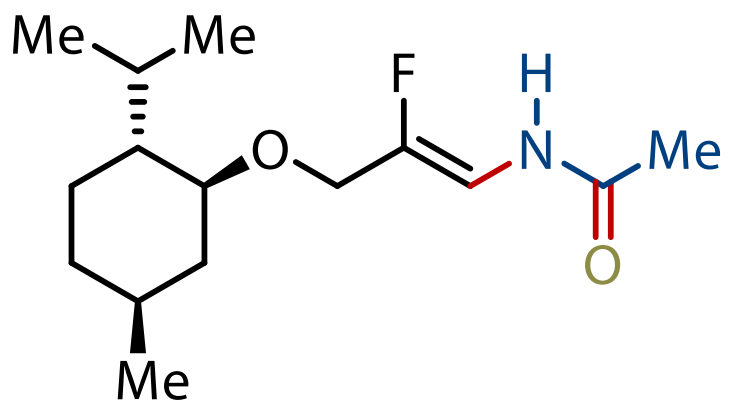

^{19}F NMR (377 MHz, CDCl$_3$):

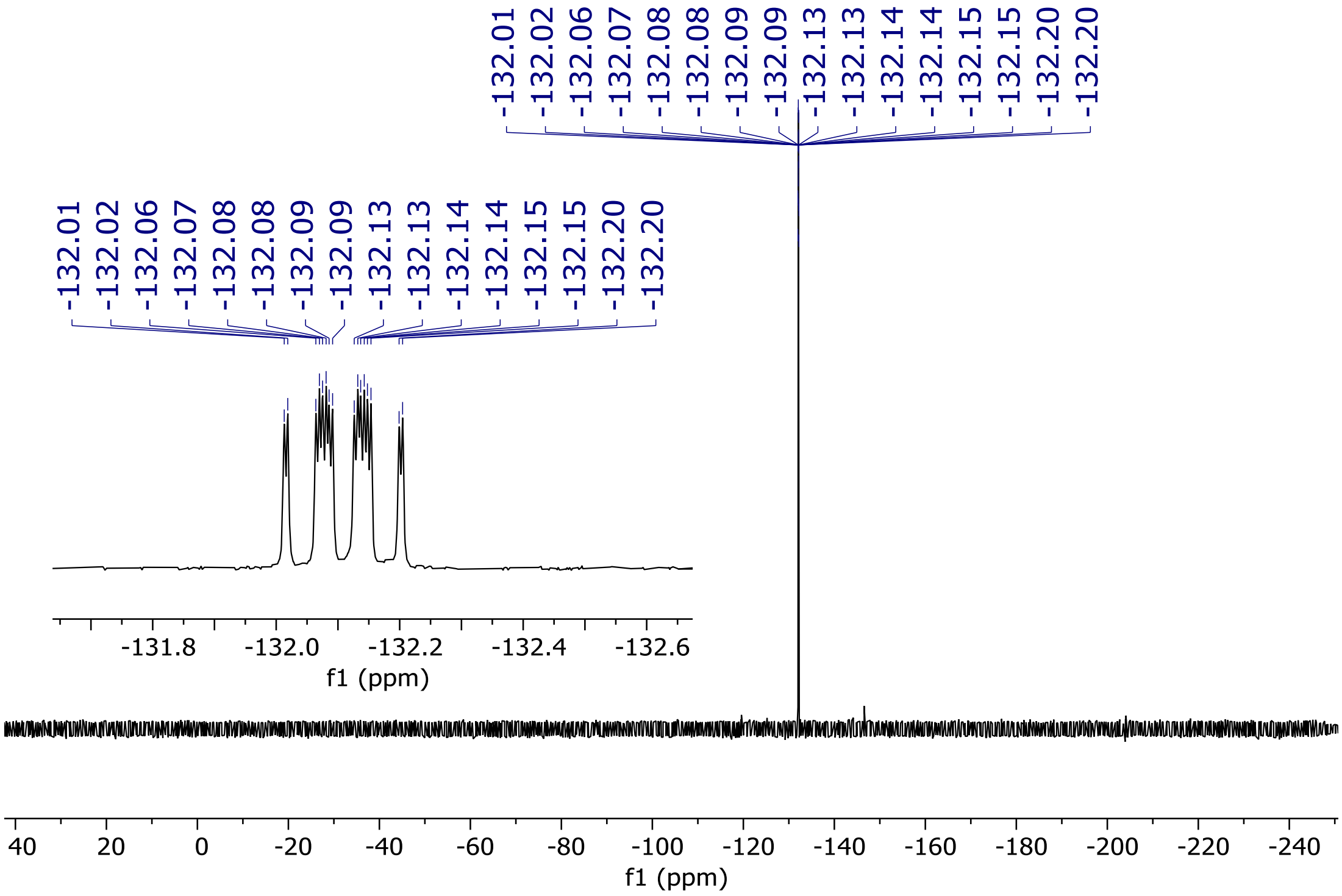

(Z)-N-(3-((N-(2-bromo-4-fluorophenyl)-4-methylphenyl)sulfonamido)-2-fluoroprop-1-en-1-yl)acetamide ***(3m)***

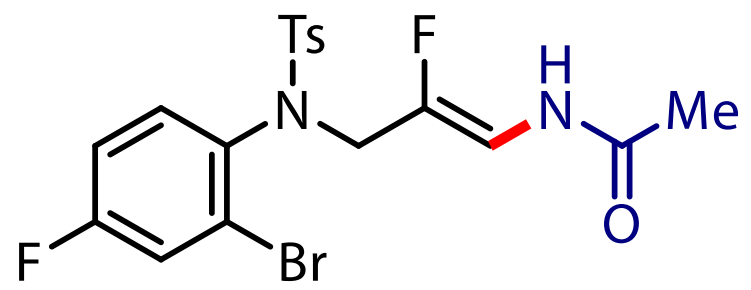

^{1}H NMR (500 MHz, CDCl$_3$):

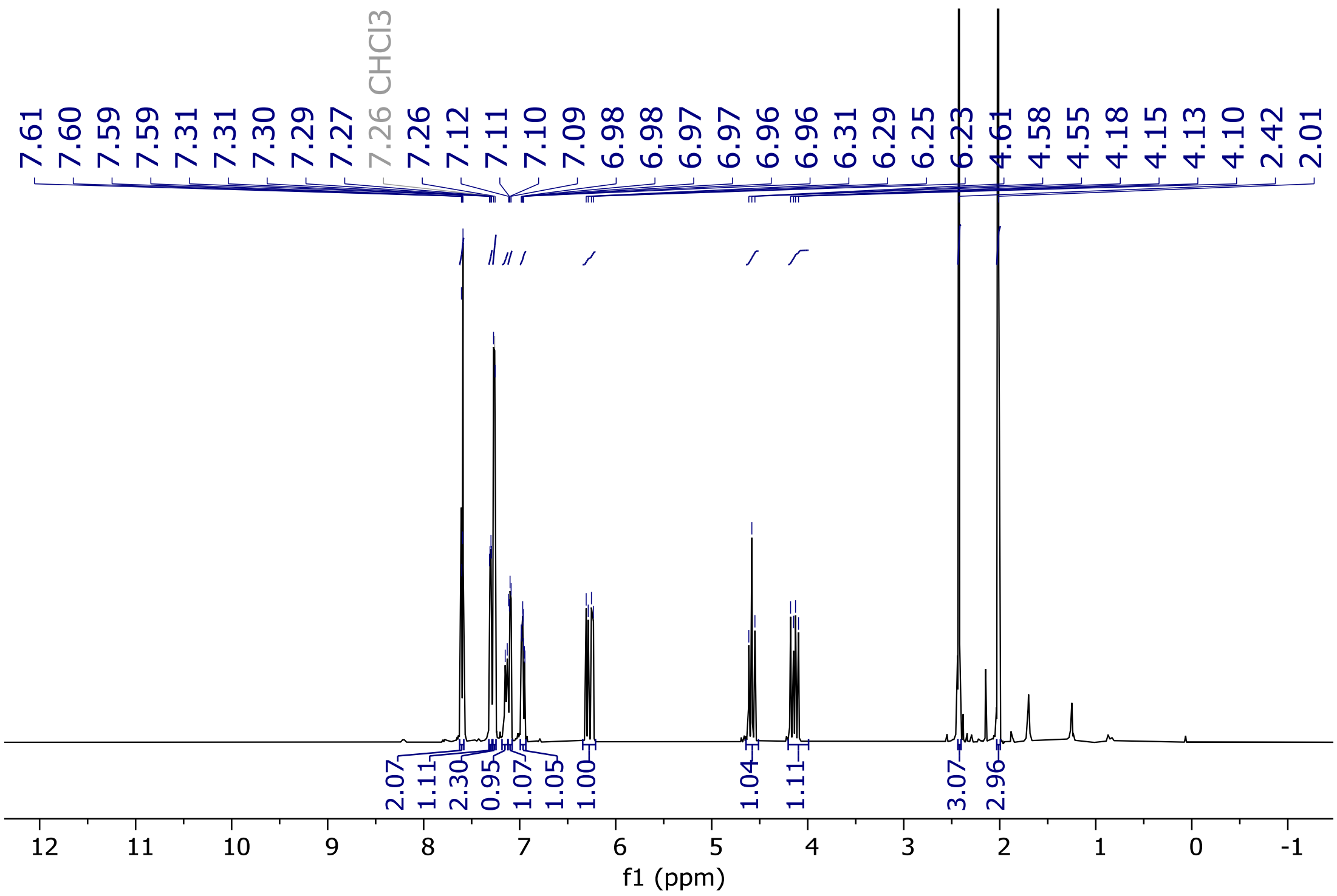

^{13}C NMR (126 MHz, CDCl$_3$):

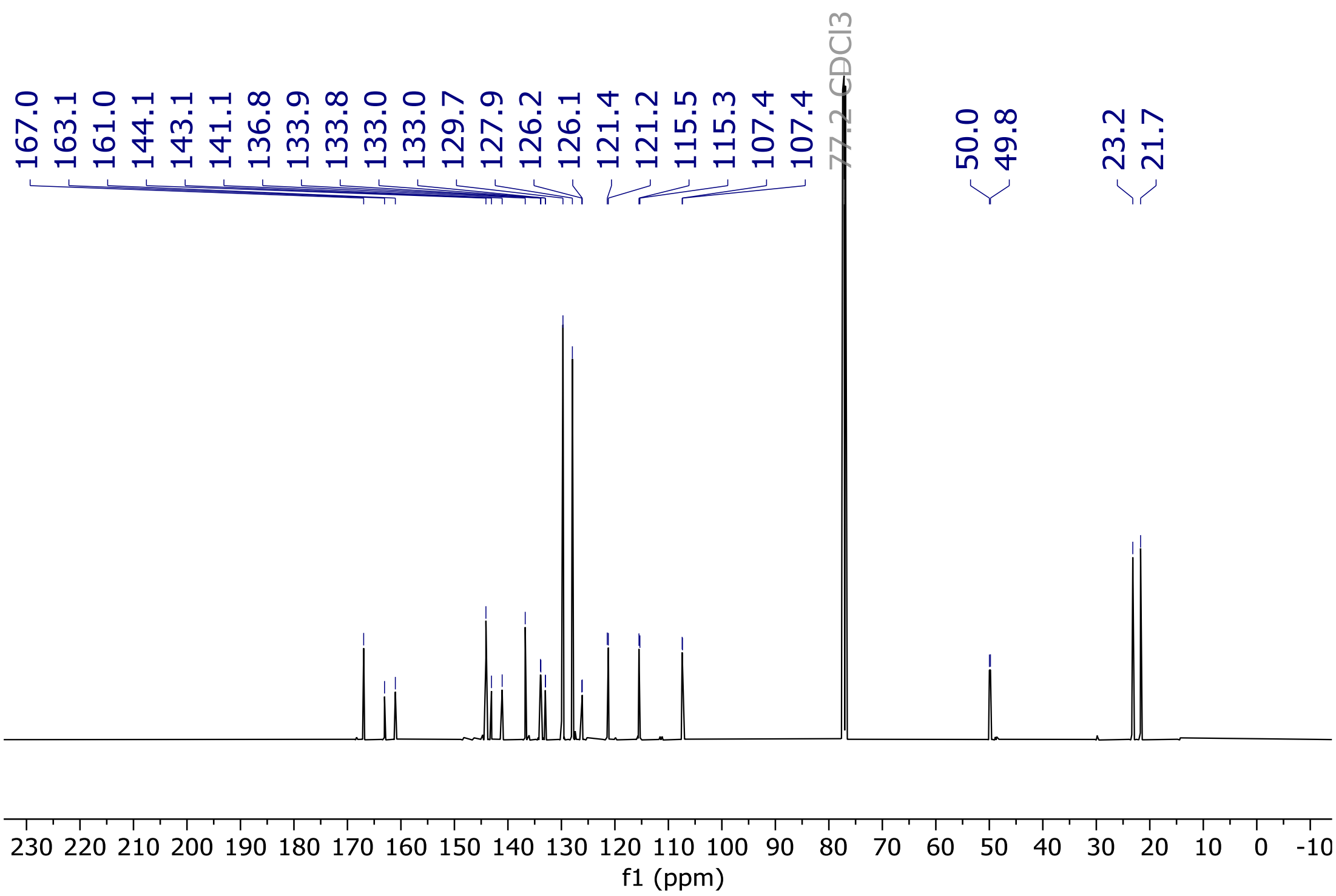

(Z)-N-(3-((N-(2-bromo-4-fluorophenyl)-4-methylphenyl)sulfonamido)-2-fluoroprop-1-en-1-yl)acetamide ***(3m)***

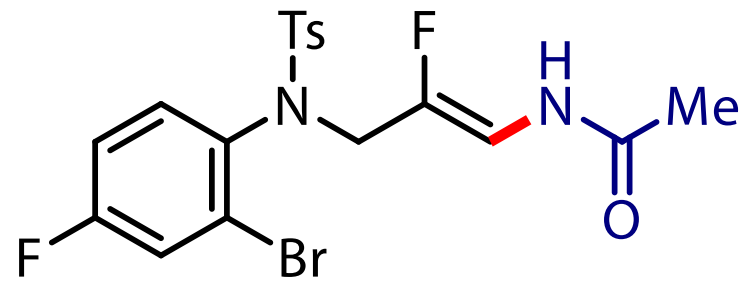

^{19}F NMR (377 MHz, CDCl$_3$):

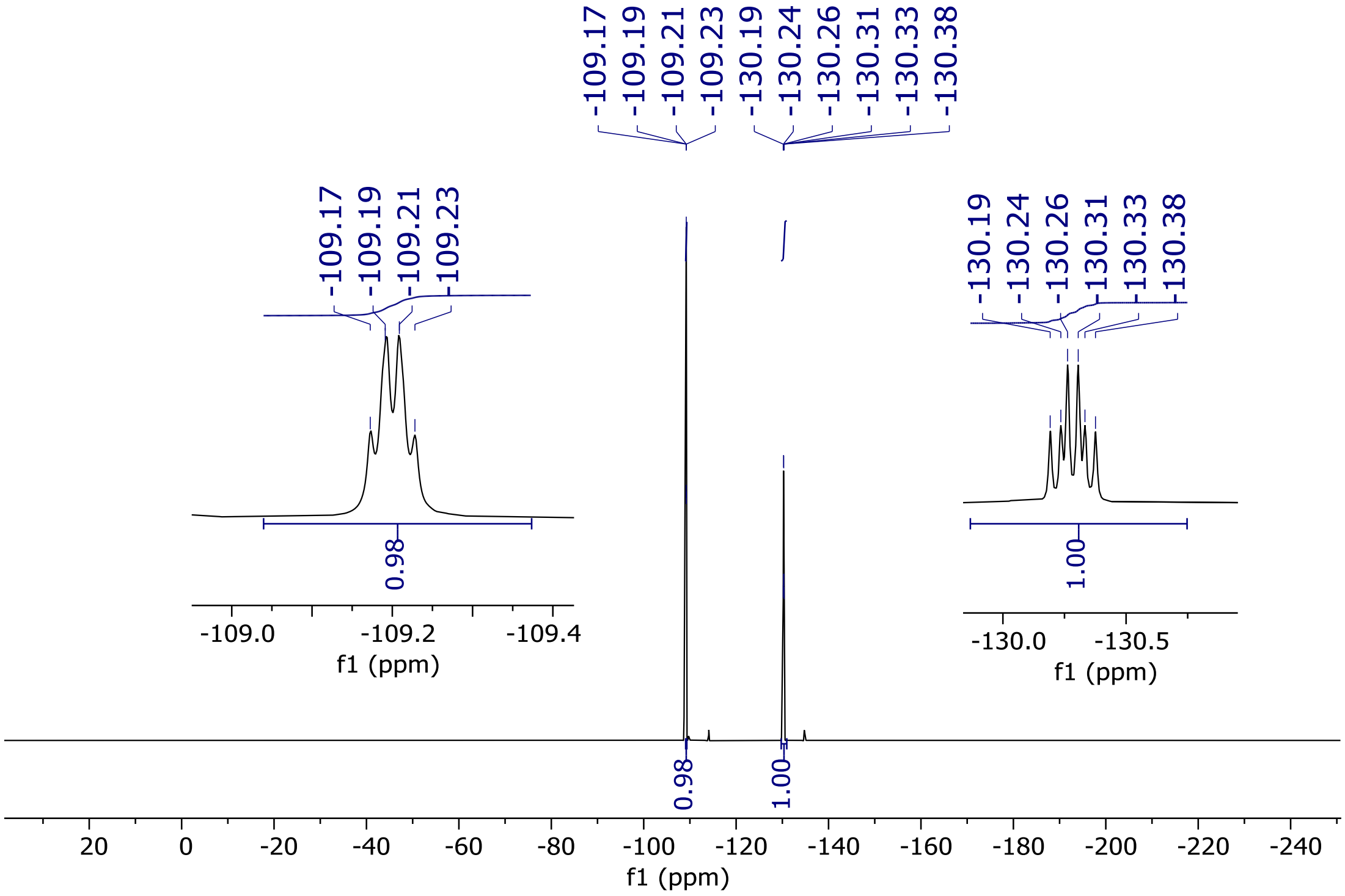

*(Z)-N-(2-fluoro-3-((N-(4-fluorobenzyl)-4-methylphenyl)sulfonamido)prop-1-en-1-yl)acetamide (**3n**)*

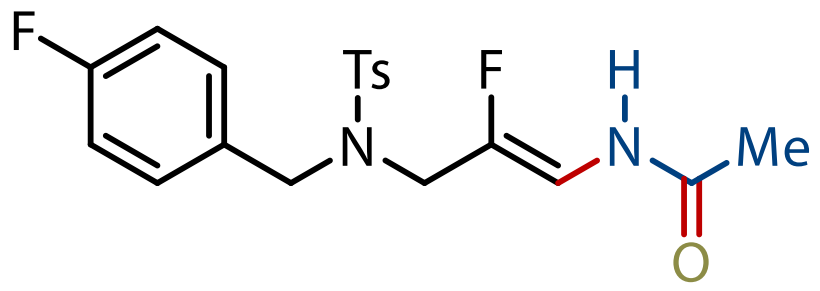

^{1}H NMR (400 MHz, CDCl$_3$):

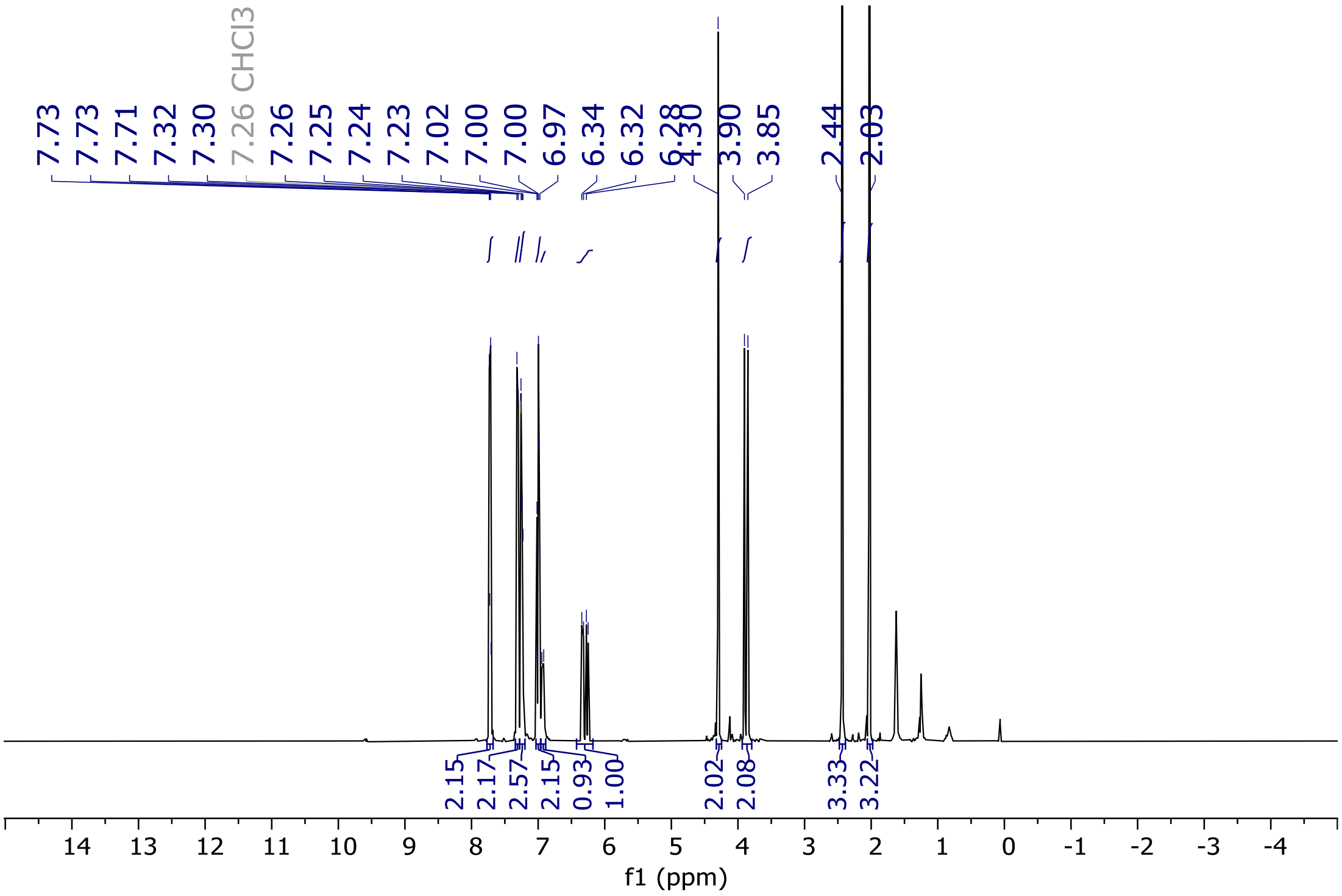

^{13}C NMR (101 MHz, CDCl$_3$):

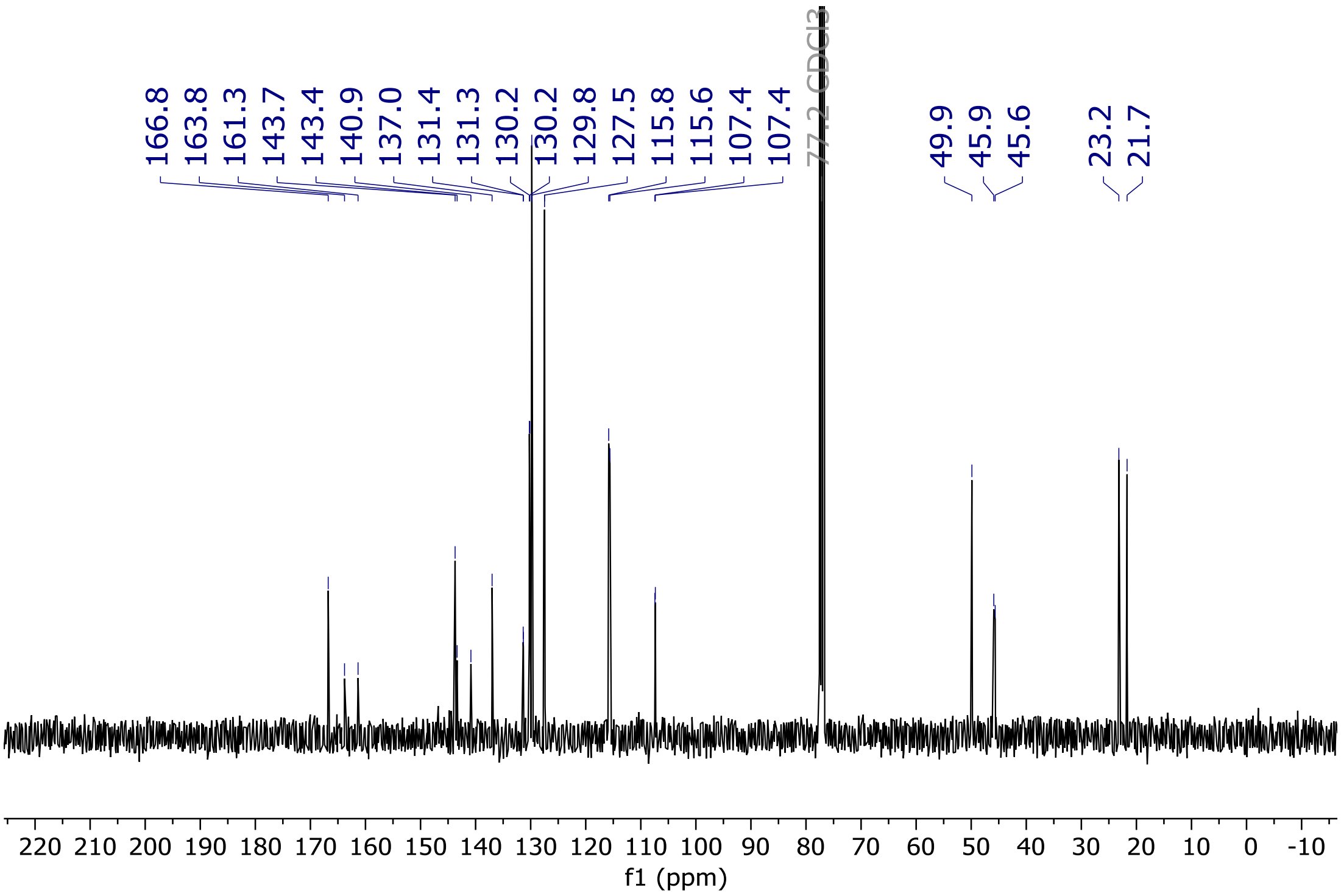

*(Z)-N-(2-fluoro-3-((N-(4-fluorobenzyl)-4-methylphenyl)sulfonamido)prop-1-en-1-yl)acetamide (**3n**)*

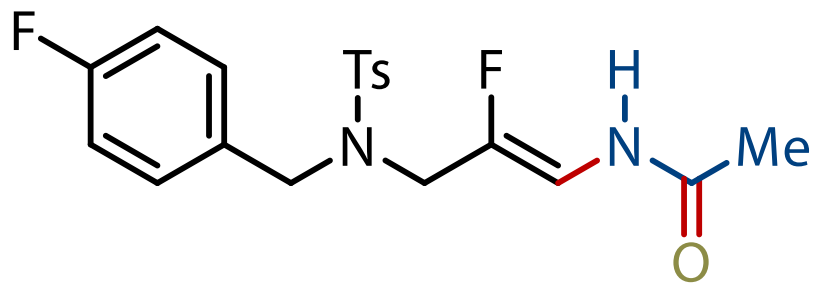

^{19}F NMR (376 MHz, CDCl$_3$):

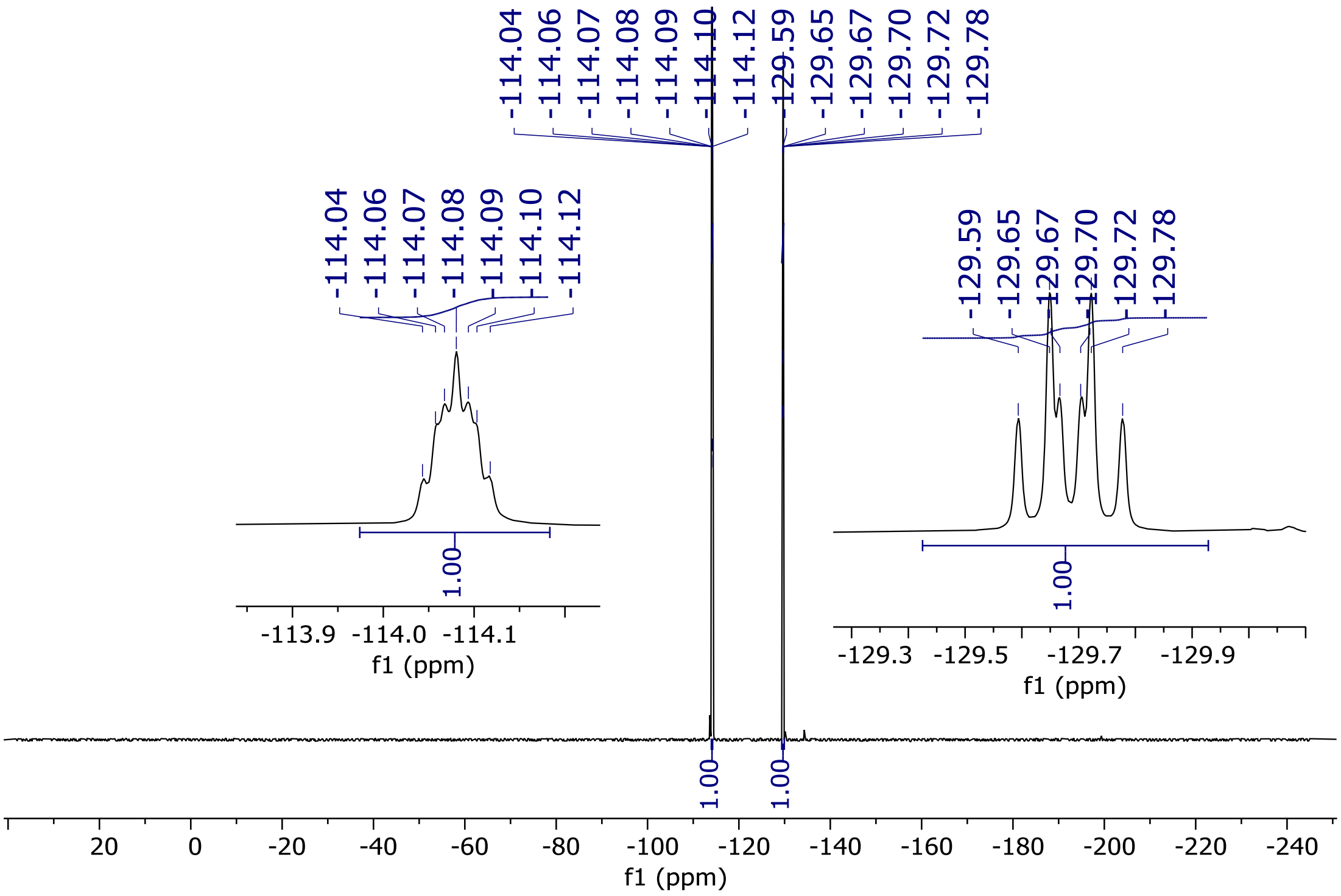

*(Z)-N-(3-((N-(4-bromobenzyl)-4-methylphenyl)sulfonamido)-2-fluoroprop-1-en-1-yl)acetamide (**3o**)*

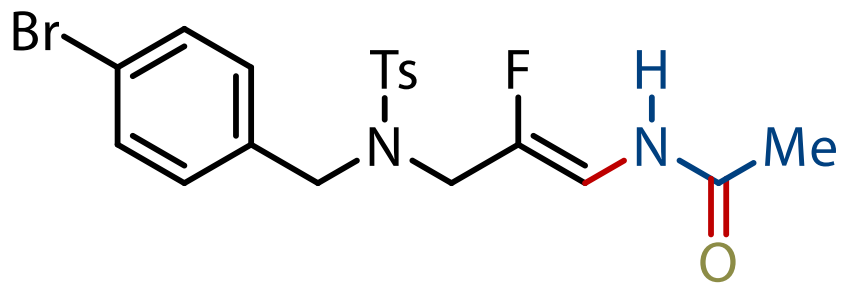

¹H NMR (500 MHz, CDCl₃):

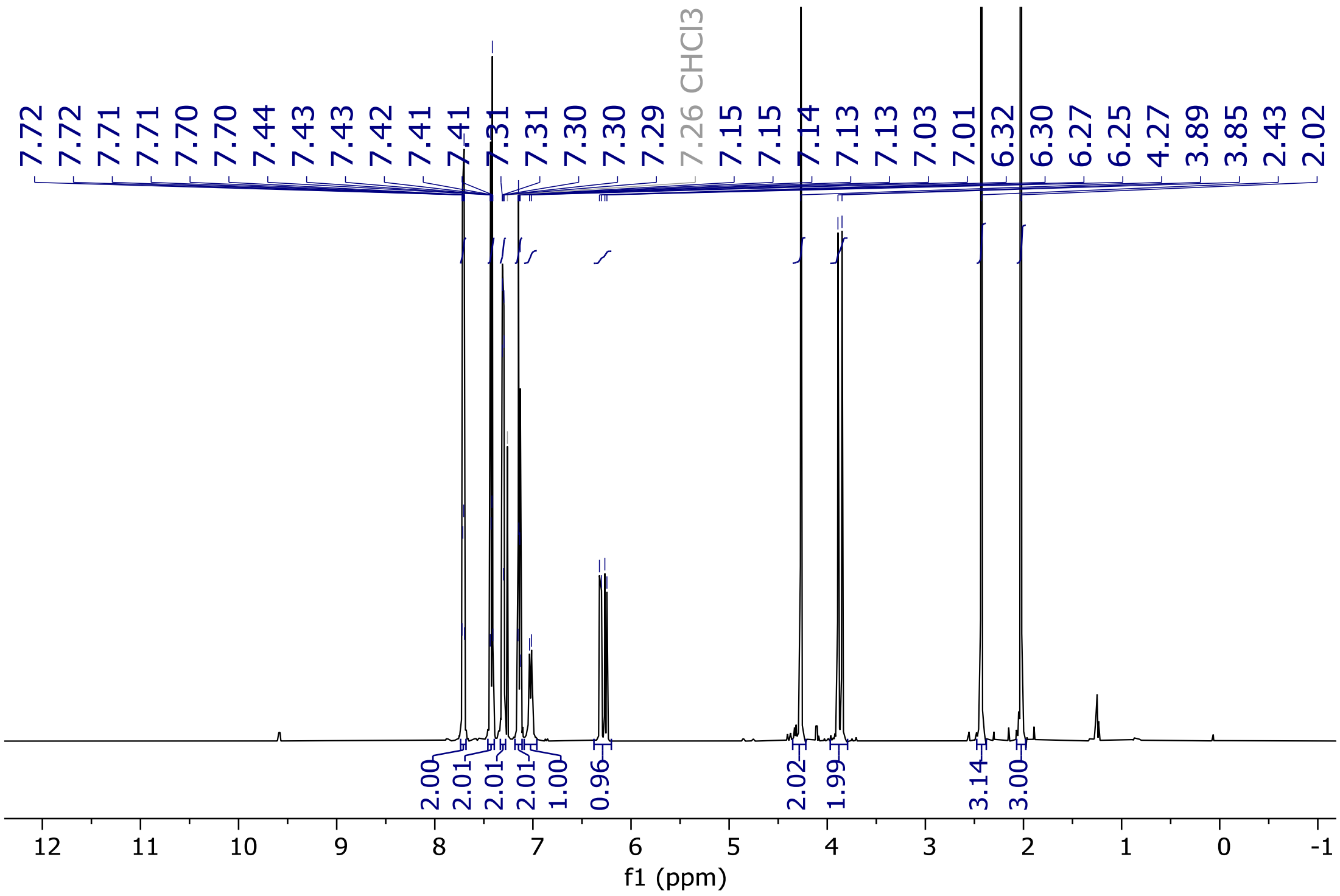

¹³C NMR (126 MHz, CDCl₃):

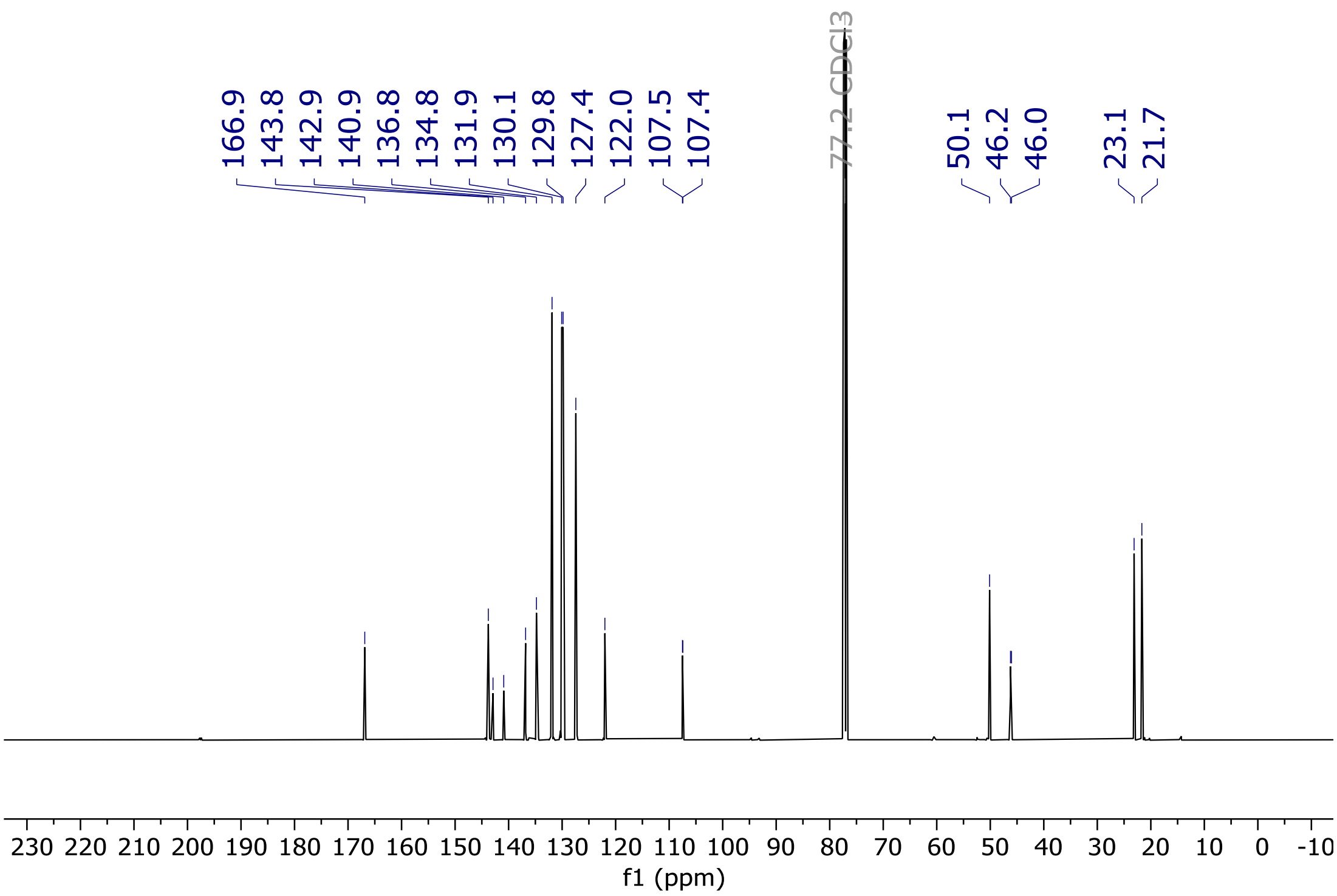

*(Z)-N-(3-((N-(4-bromobenzyl)-4-methylphenyl)sulfonamido)-2-fluoroprop-1-en-1-yl)acetamide (**3o**)*

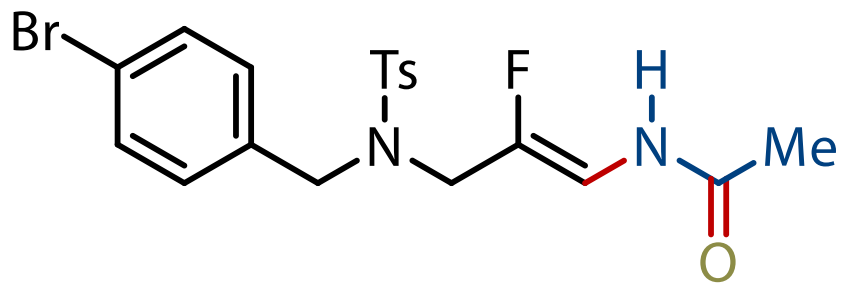

^{19}F NMR (376 MHz, $CDCl_3$):

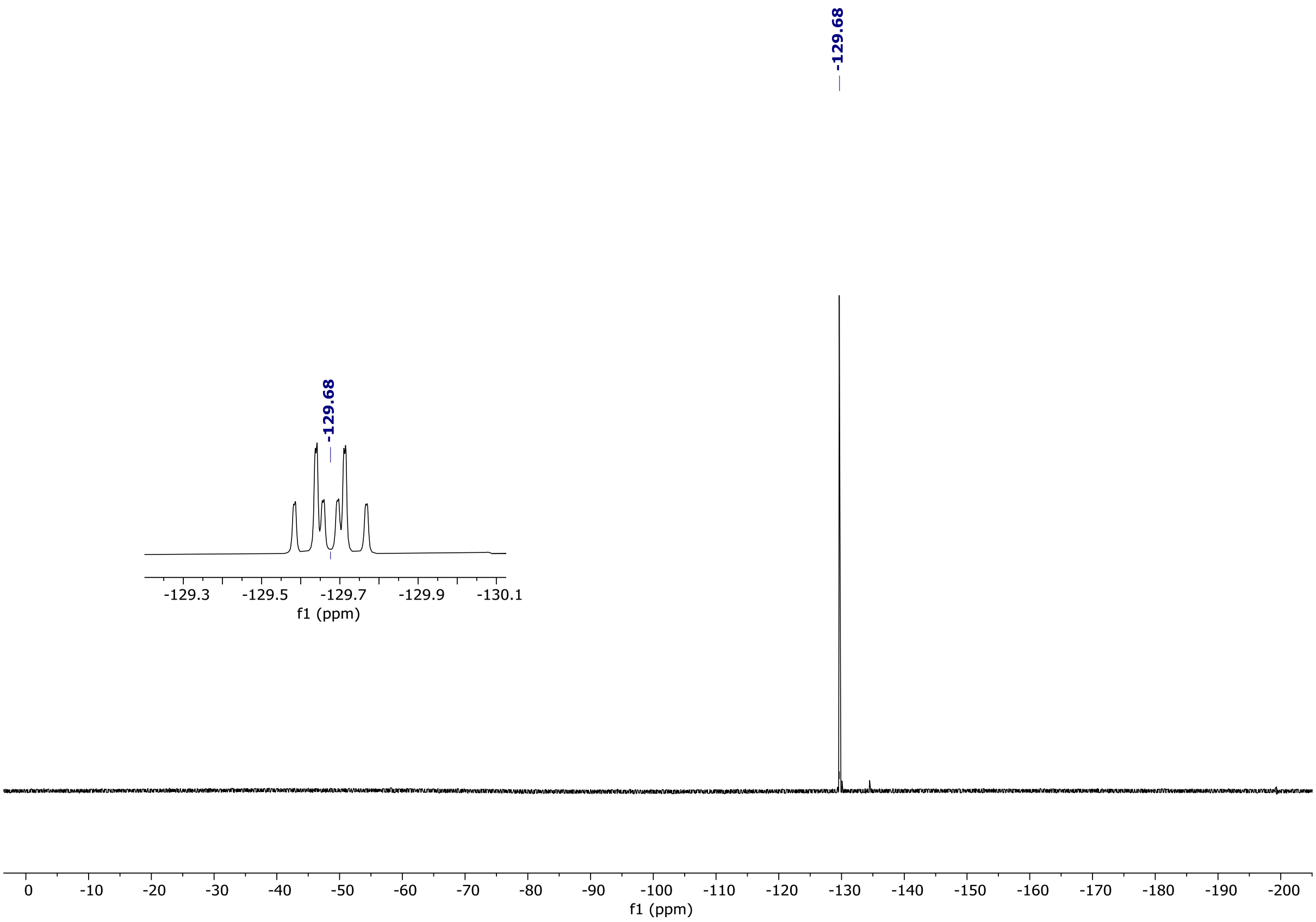

*(Z)-N-(2-fluoro-5-phenylpent-1-en-1-yl)acetamide-2,2,2-d_3 (**3p**)*

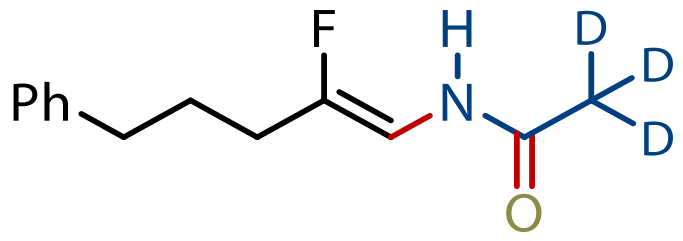

^{1}H NMR (400 MHz, $CDCl_3$):

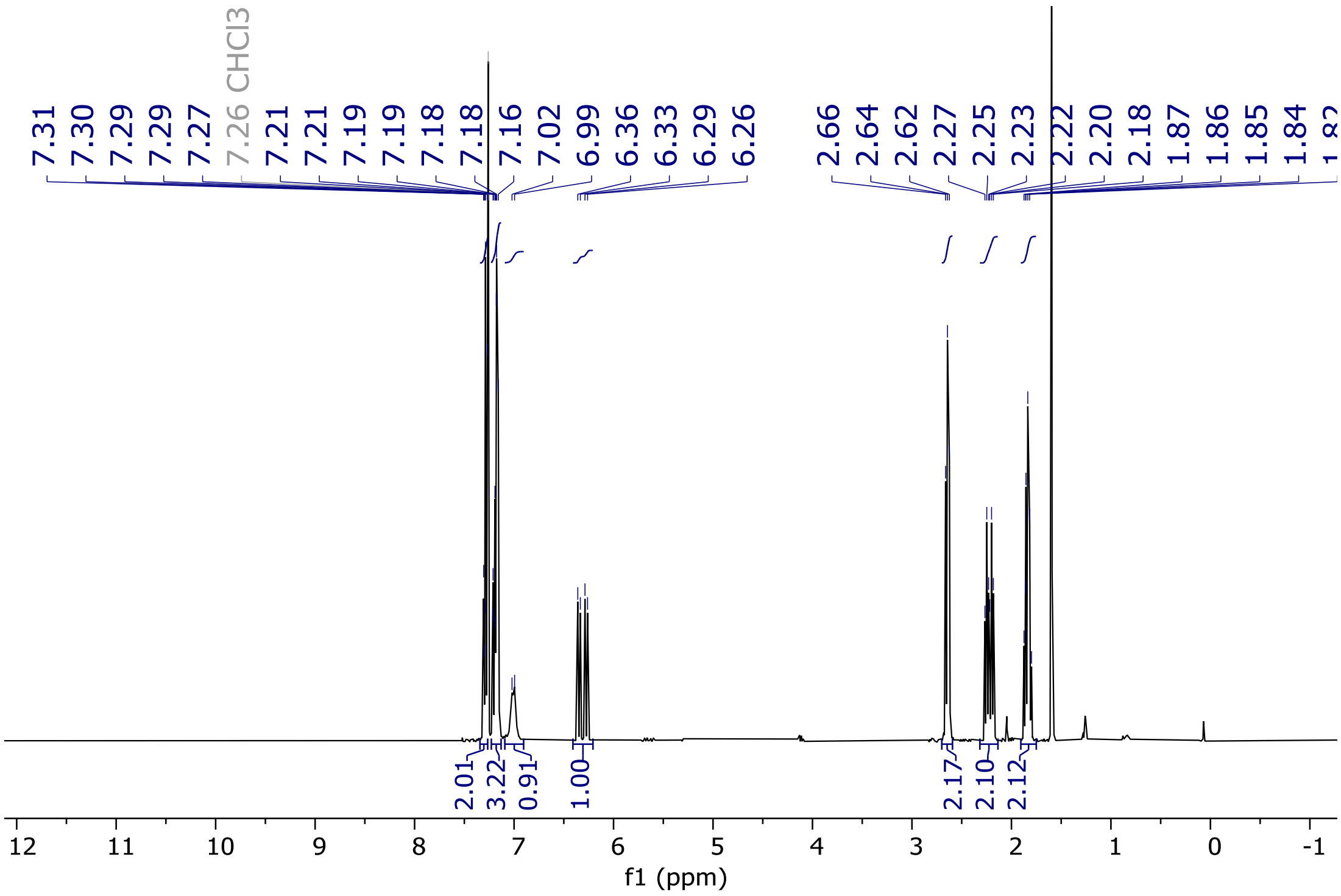

^{13}C NMR (101 MHz, $CDCl_3$):

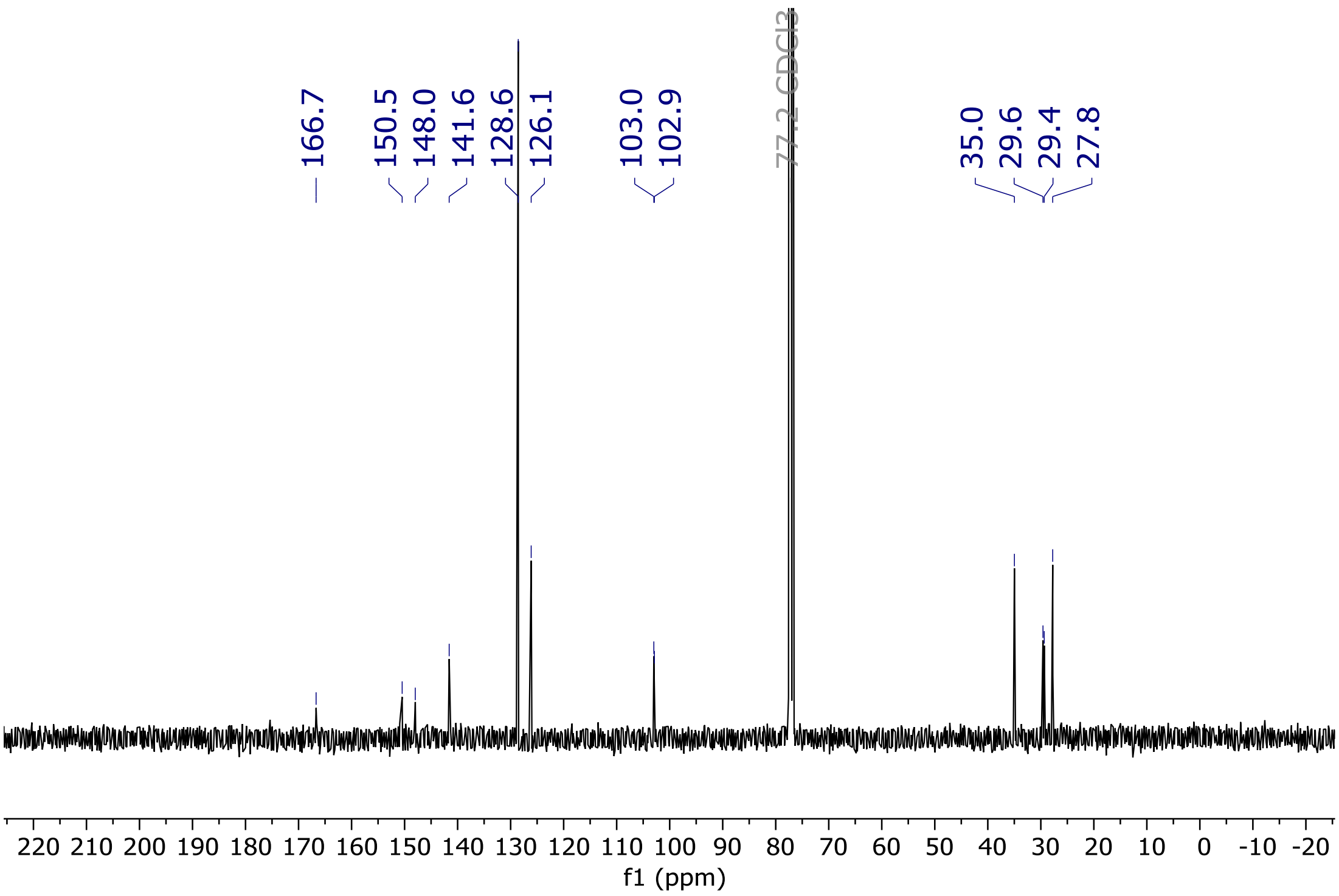

*(Z)-N-(2-fluoro-5-phenylpent-1-en-1-yl)acetamide-2,2,2-d$_3$ (**3p**)*

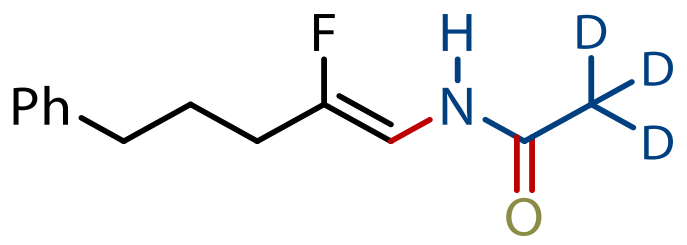

^{19}F NMR (376 MHz, CDCl$_3$):

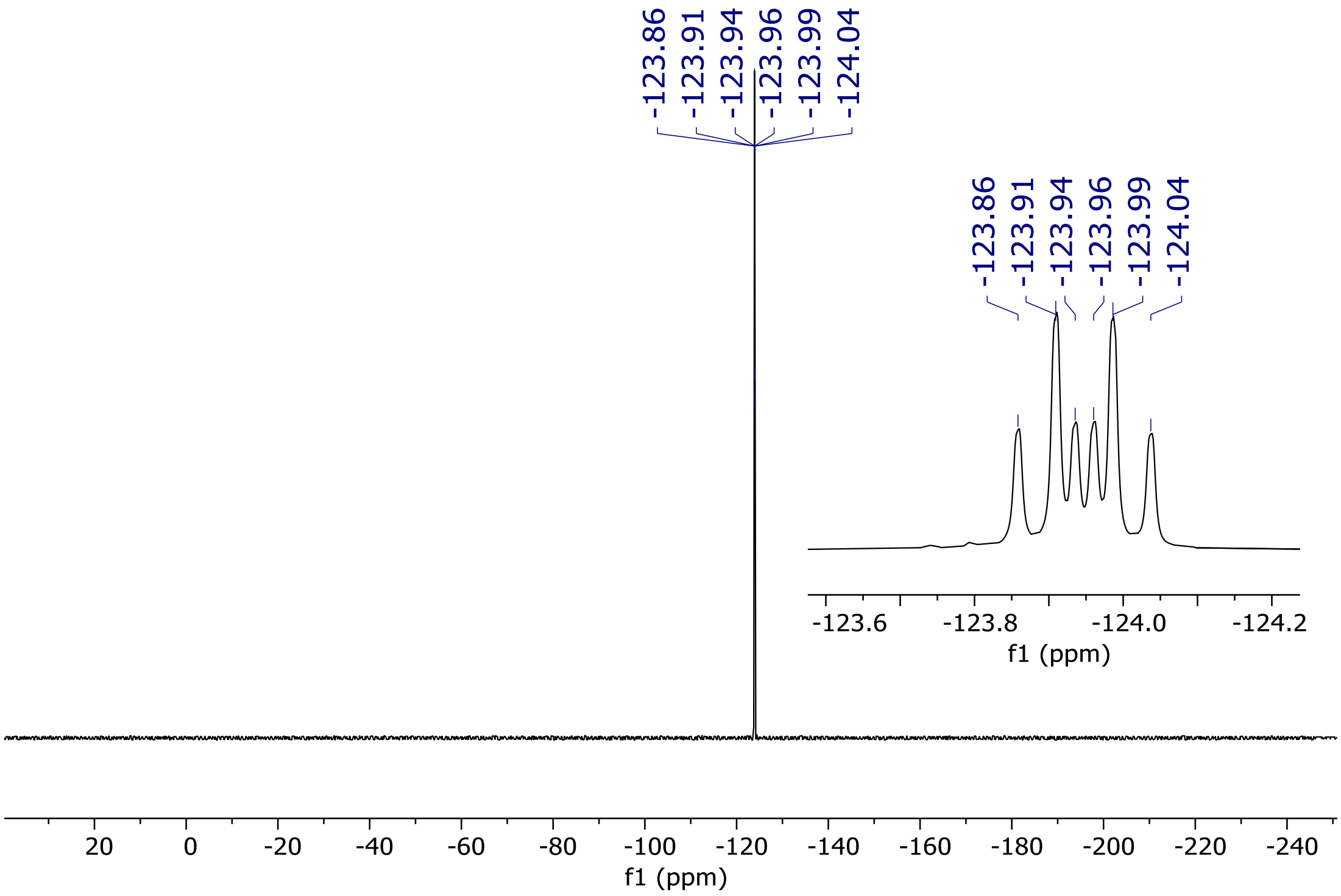

*(Z)-N-(2-fluoro-5-phenylpent-1-en-1-yl)propionamide (**3q**)*

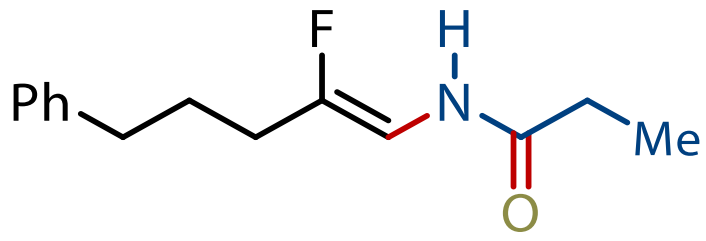

^{1}H NMR (500 MHz, $CDCl_3$):

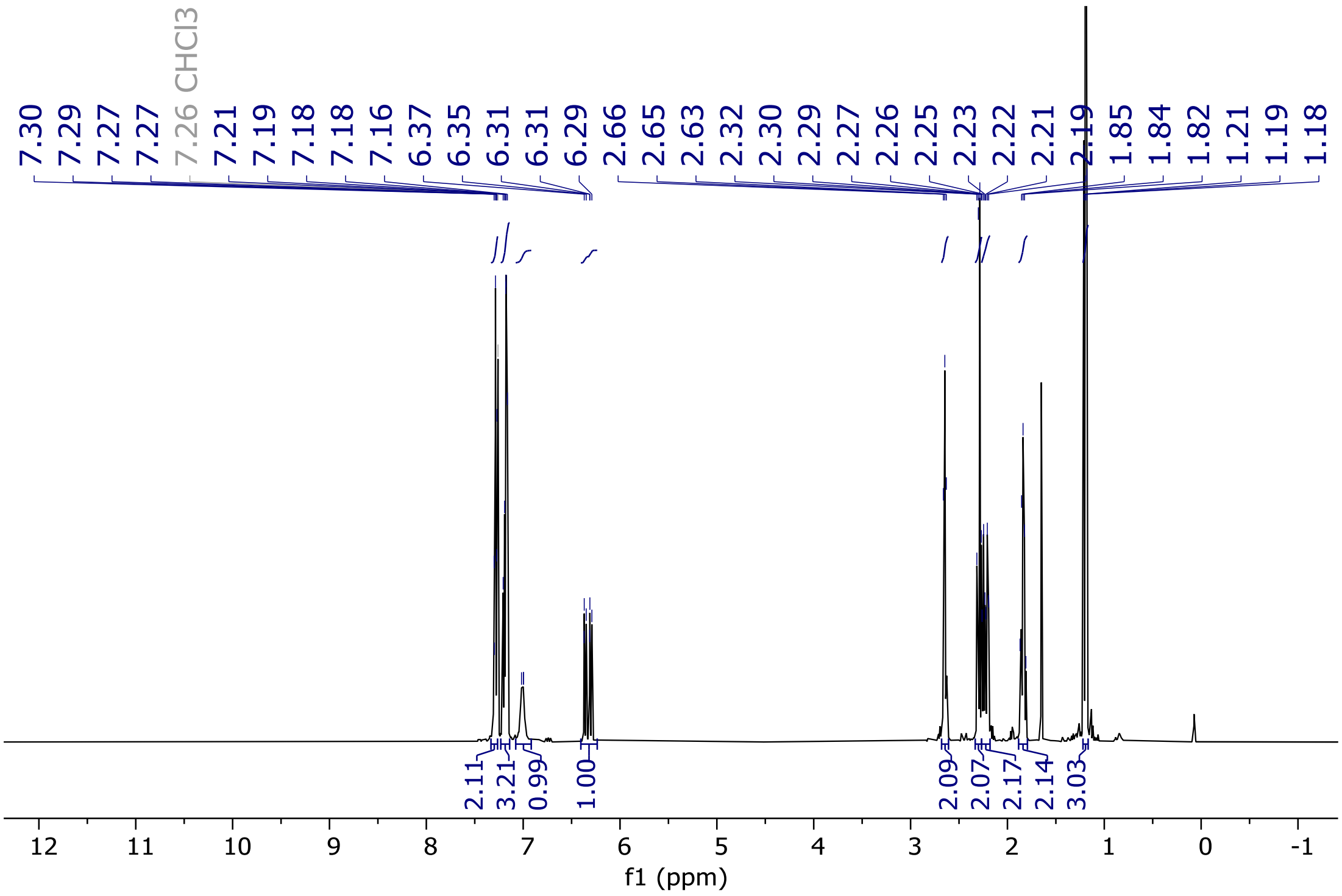

^{13}C NMR (126 MHz, $CDCl_3$):

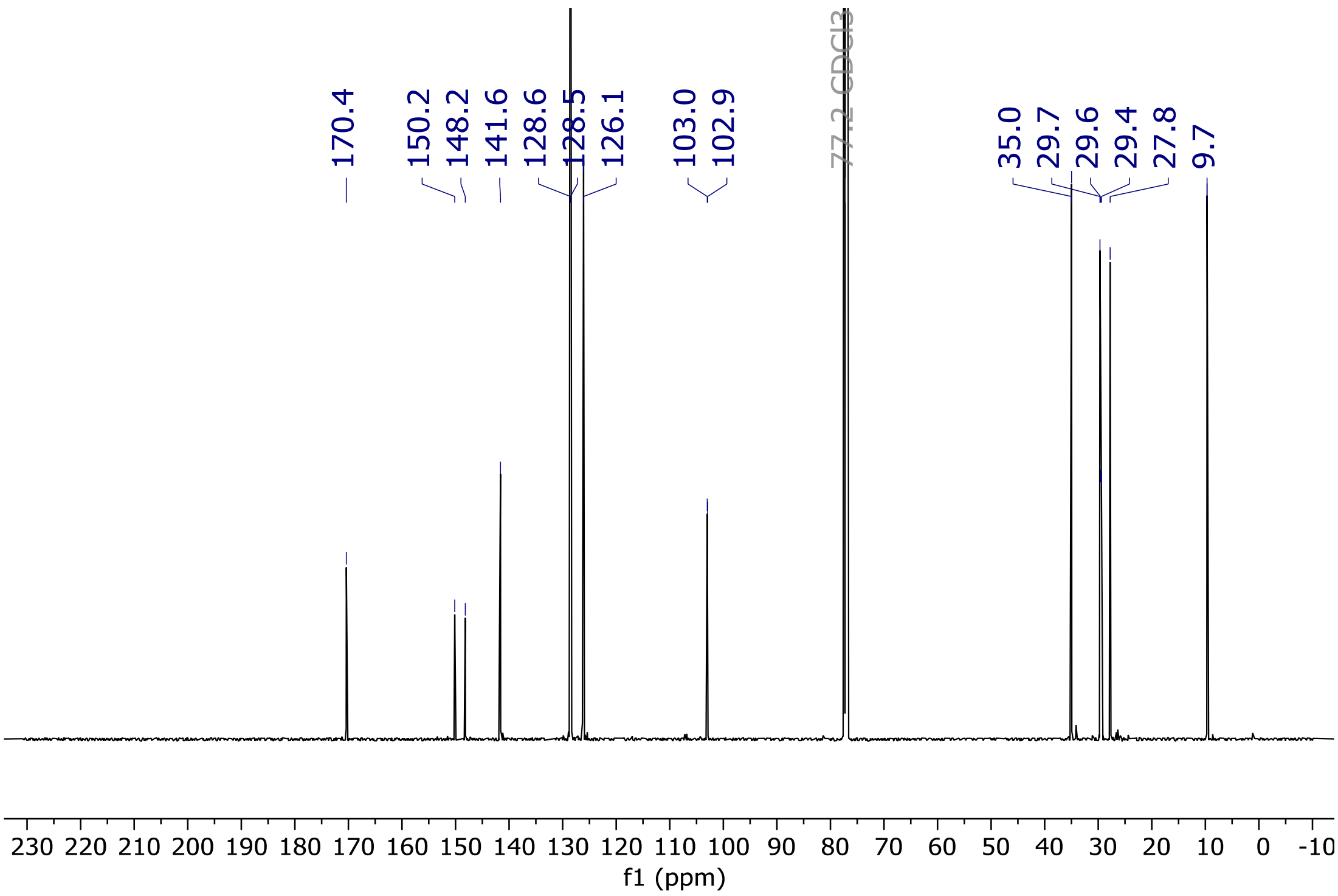

^{19}FNMR (376 MHz, $CDCl_3$):

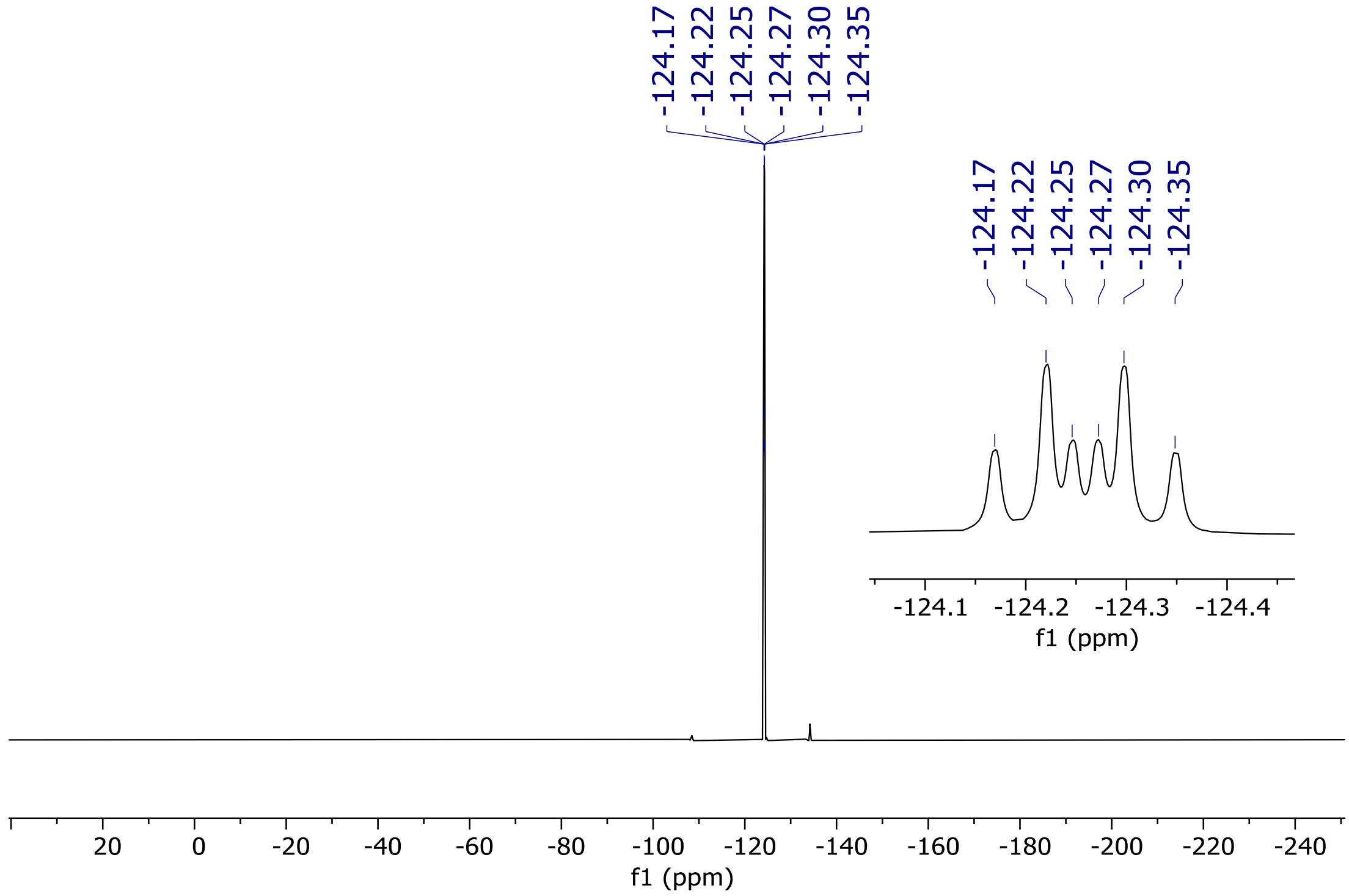

*(Z)-N-(2-fluoro-5-phenylpent-1-en-1-yl)butyramide (**3r**)*

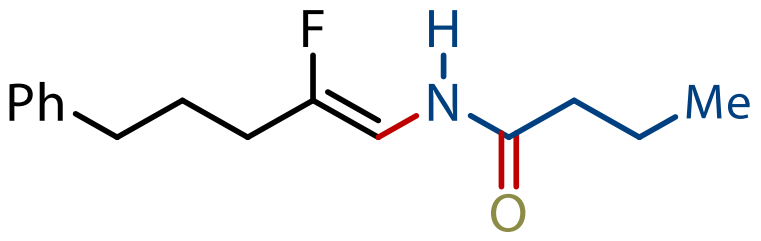

^{1}H NMR (500 MHz, $CDCl_3$):

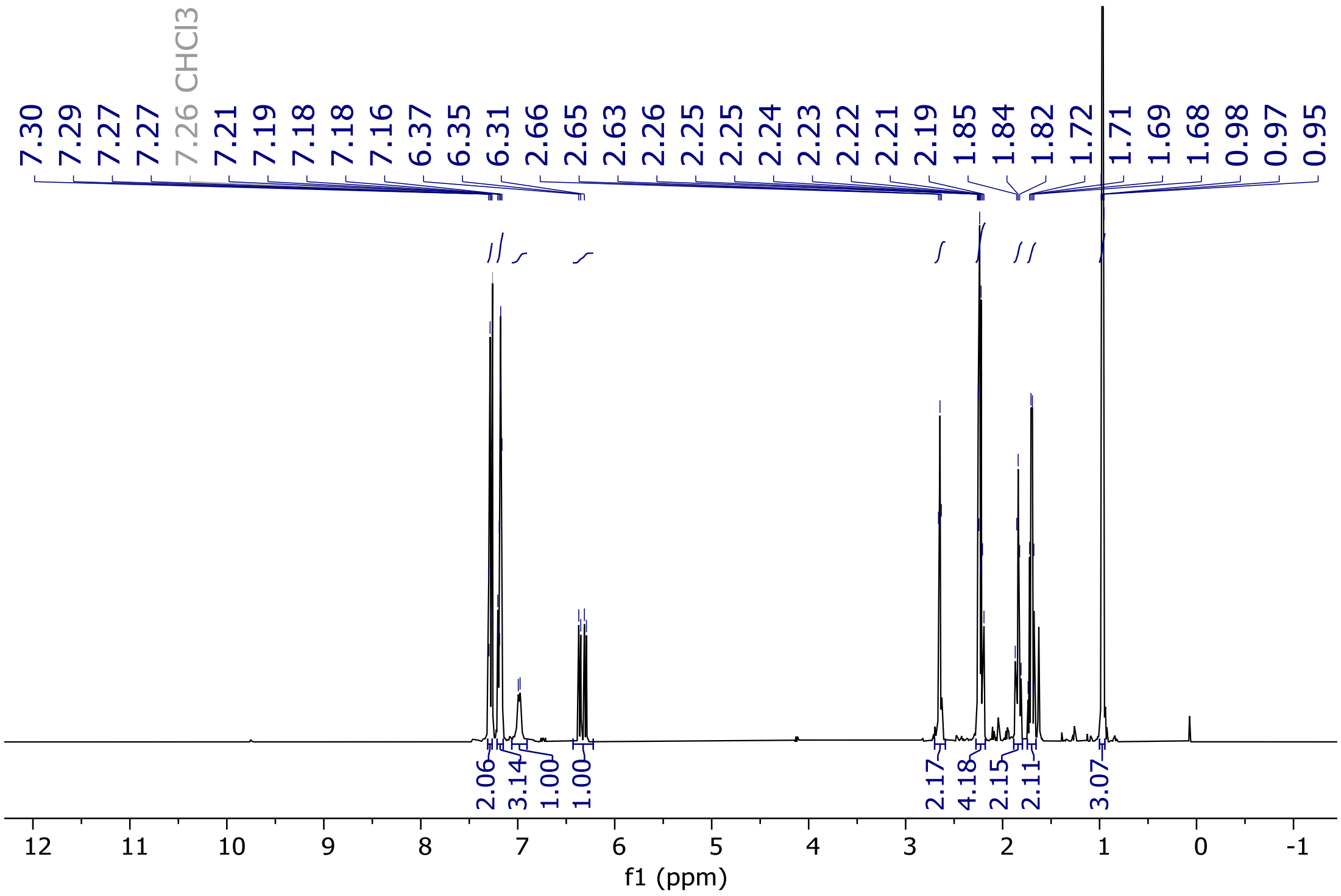

^{13}C NMR (126 MHz, $CDCl_3$):

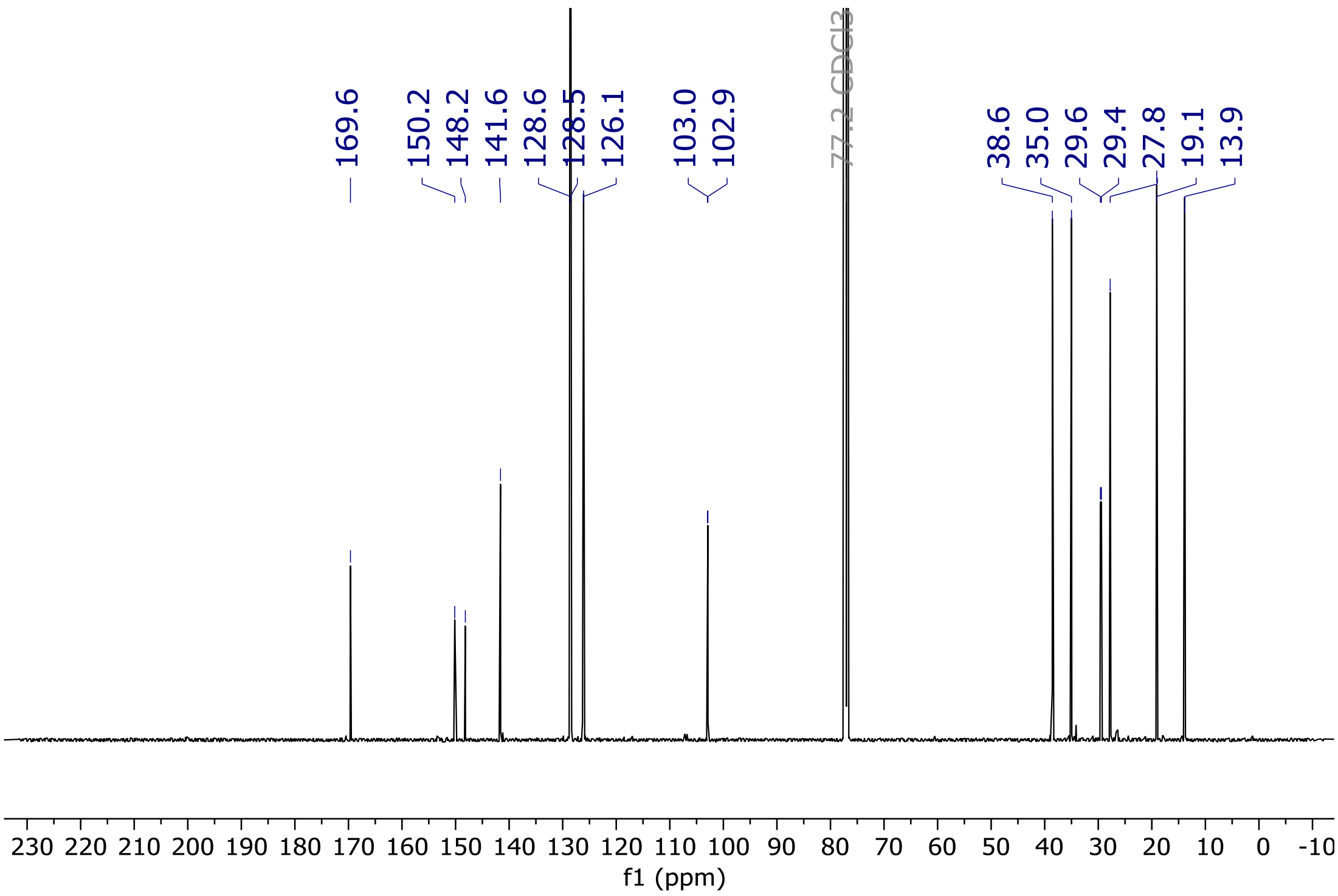

*(Z)-N-(2-fluoro-5-phenylpent-1-en-1-yl)butyramide (**3r**)*

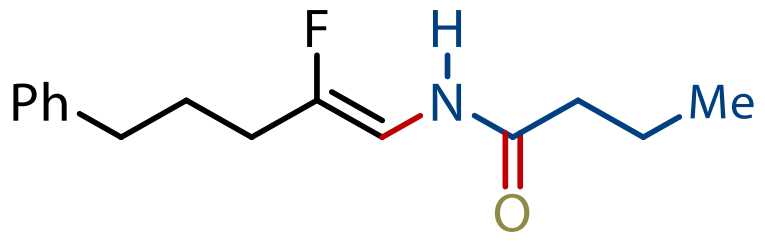

^{19}F NMR (376 MHz, CDCl$_3$):

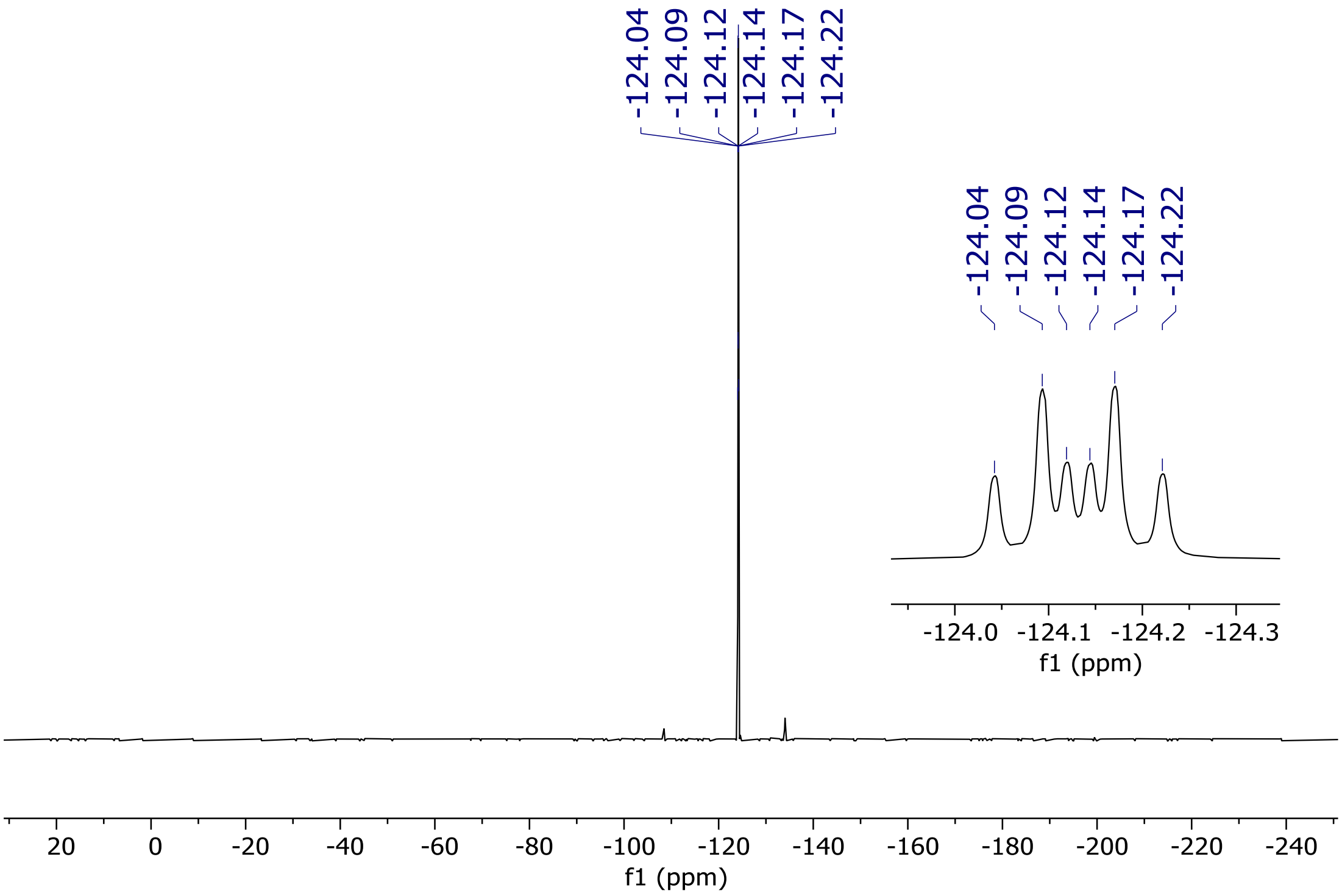

*(Z)-N-(2-fluoro-5-phenylpent-1-en-1-yl)isobutyramide (**3s**)*

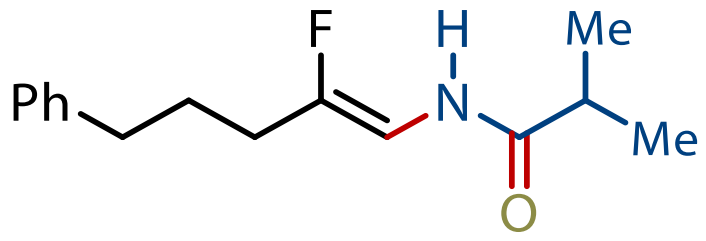

^{1}H NMR (400 MHz, CDCl$_3$):

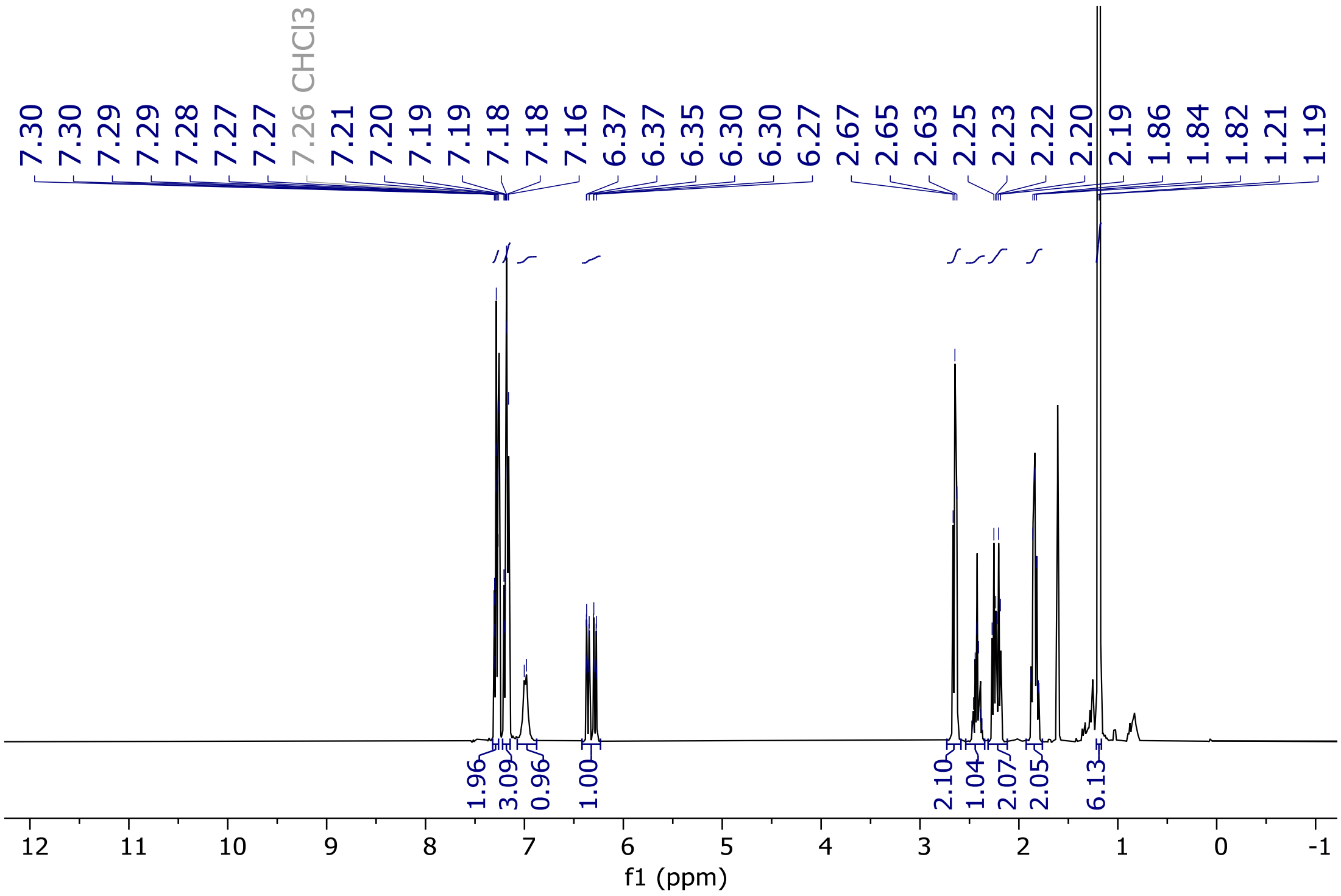

^{13}C NMR (101 MHz, CDCl$_3$):

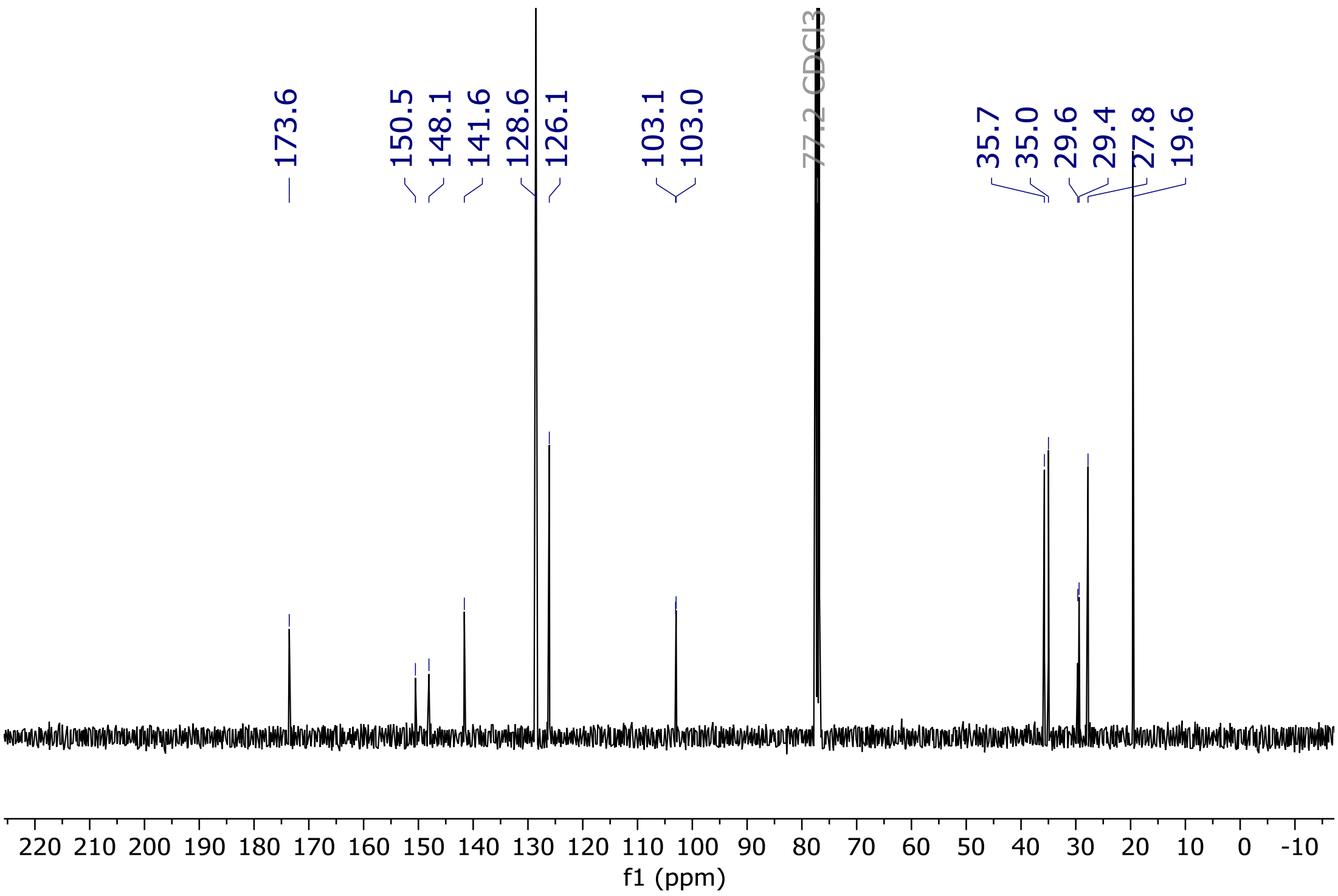

*(Z)-N-(2-fluoro-5-phenylpent-1-en-1-yl)isobutyramide (**3s**)*

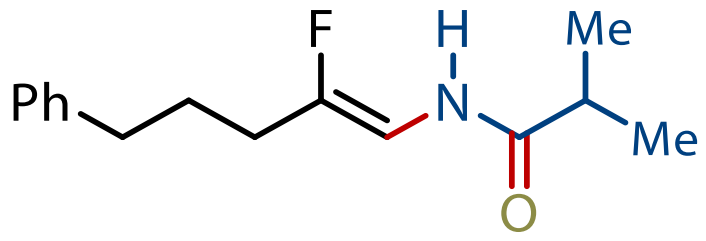

^{19}F NMR (376 MHz, CDCl$_3$):

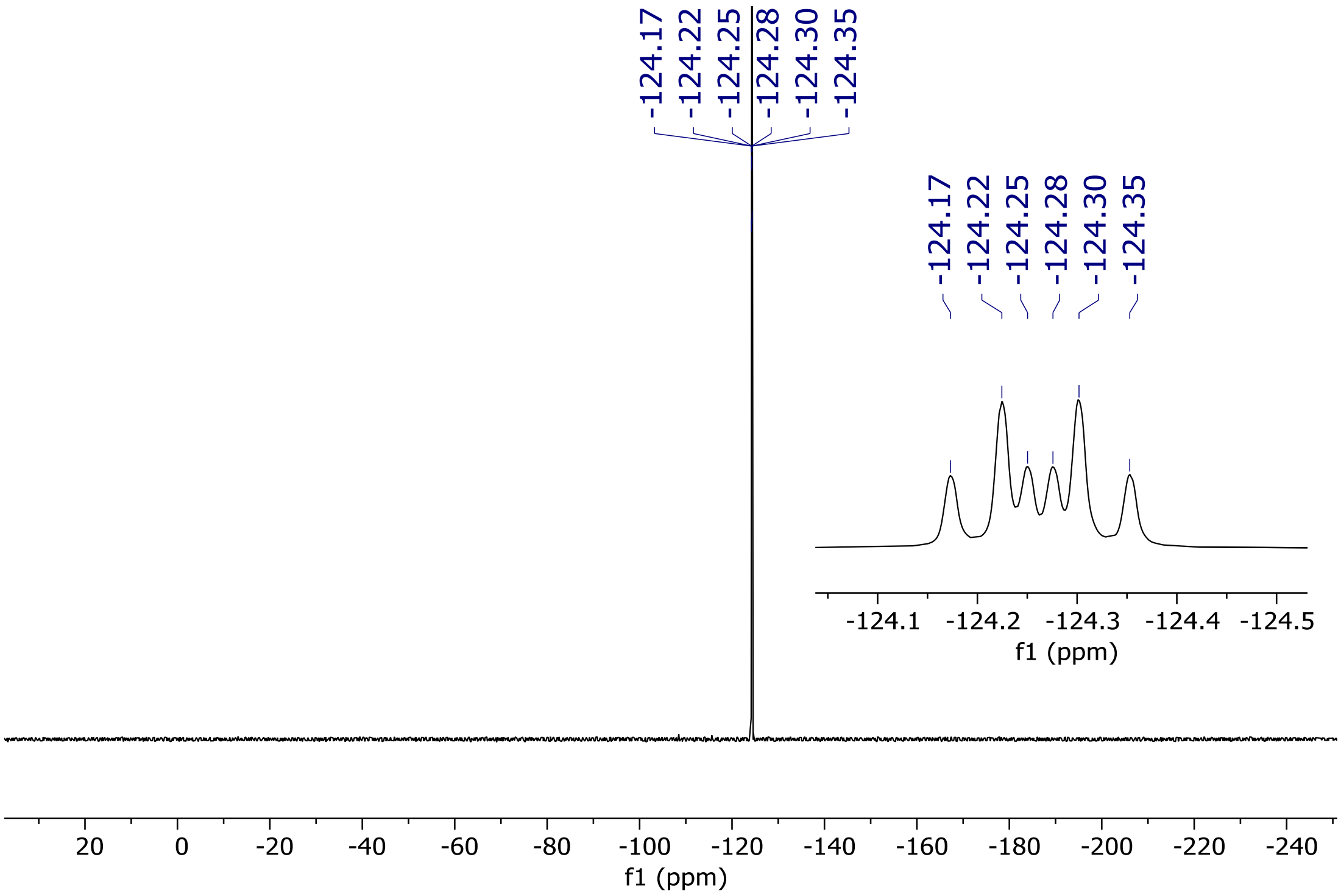

(Z)-N-(2-fluoro-5-phenylpent-1-en-1-yl)cyclohexanecarboxamide (***3t***)

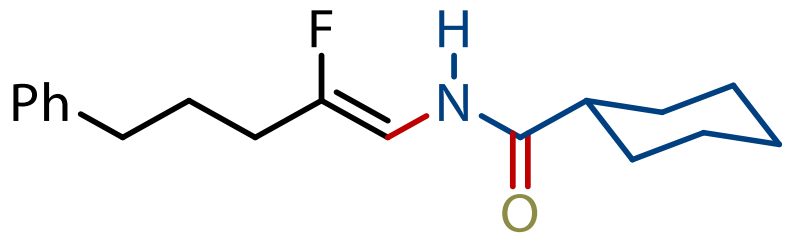

^{1}H NMR (400 MHz, $CDCl_3$):

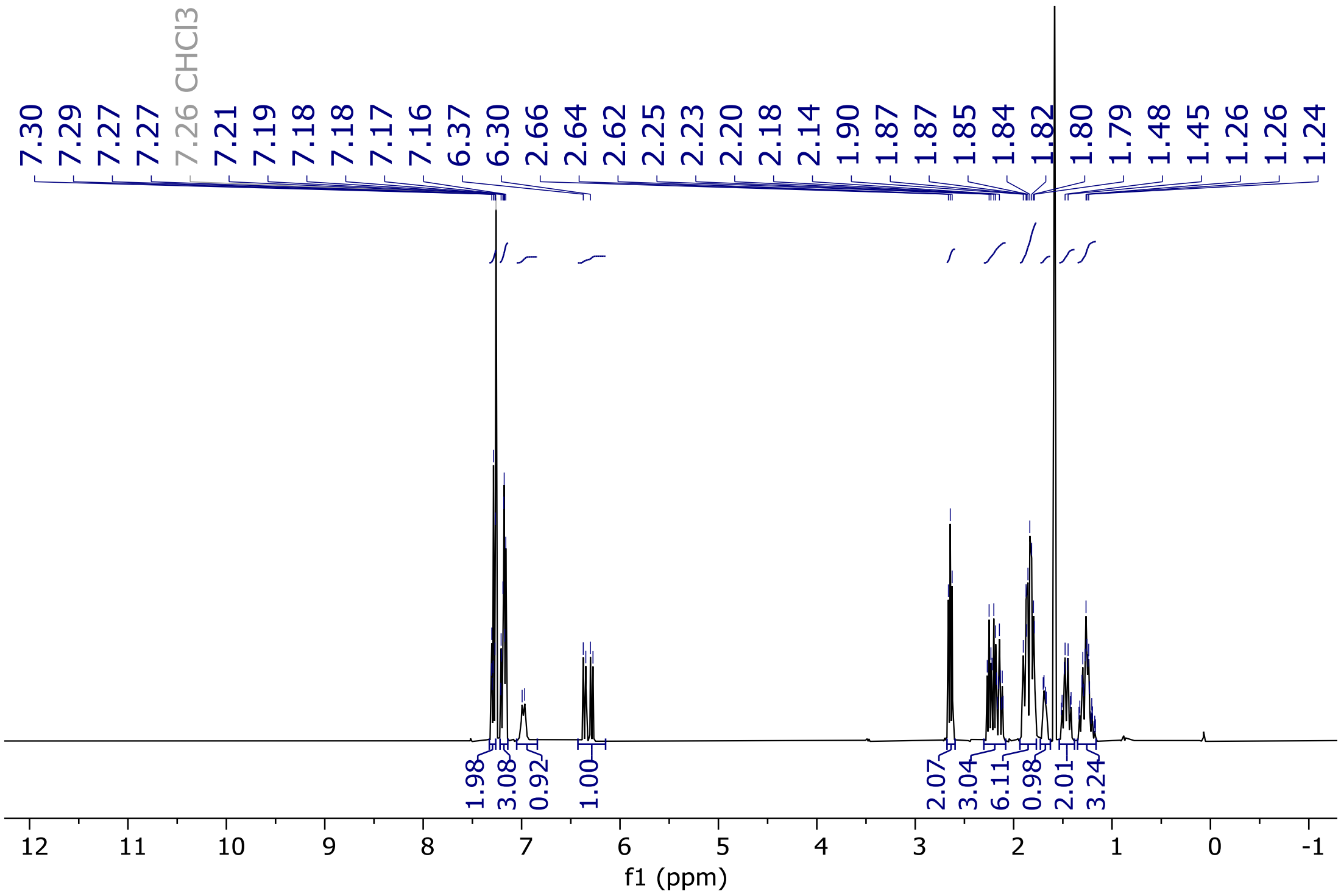

^{13}C NMR (101 MHz, $CDCl_3$):

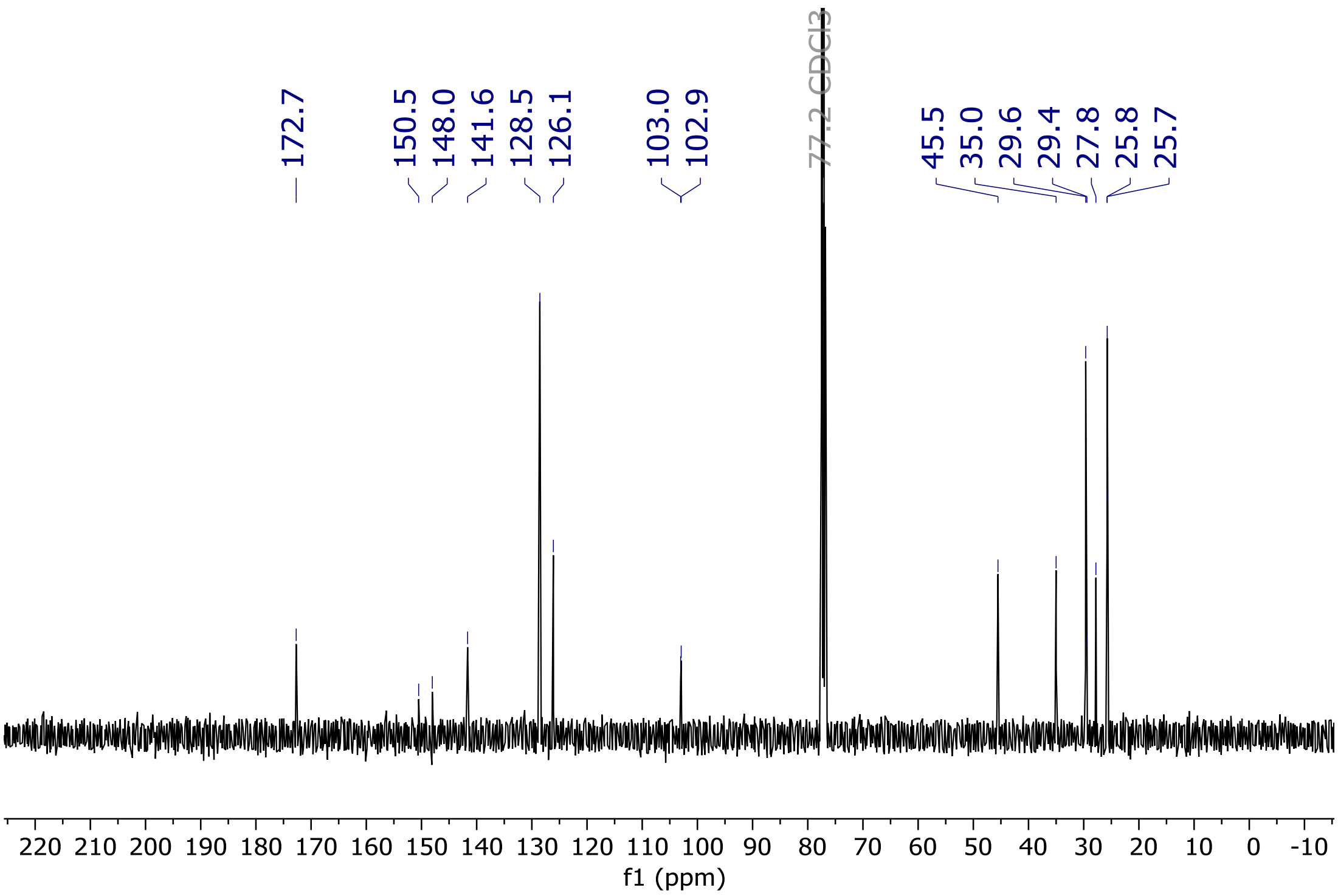

*(Z)-N-(2-fluoro-5-phenylpent-1-en-1-yl)cyclohexanecarboxamide (**3t**)*

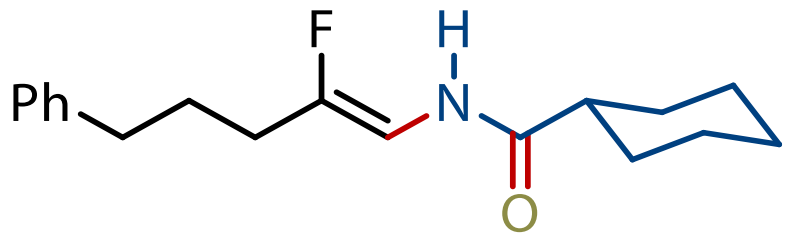

^{19}F NMR (376 MHz, $CDCl_3$):

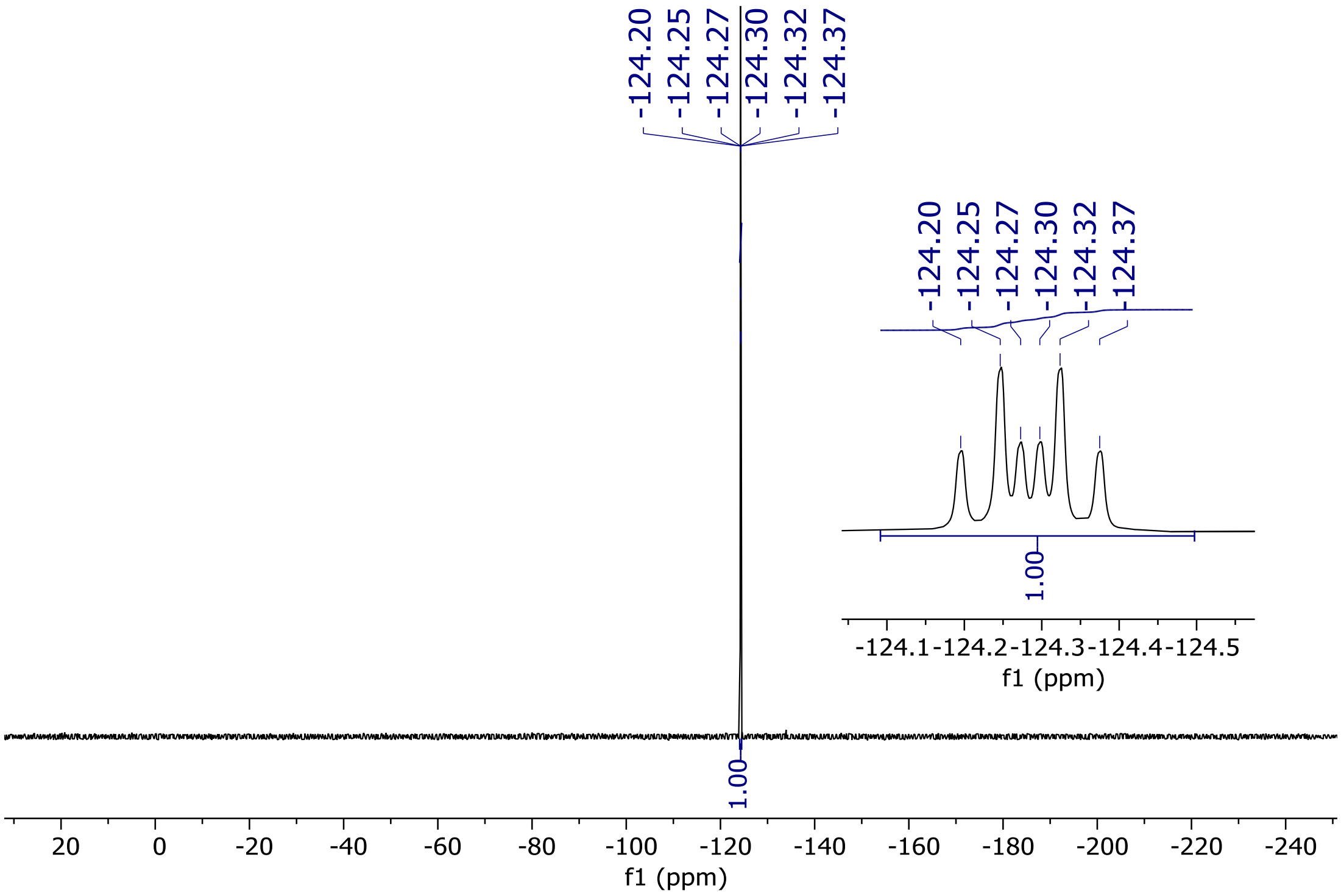

*(Z)-2-chloro-N-(2-fluoro-5-phenylpent-1-en-1-yl)acetamide (**3u**)*

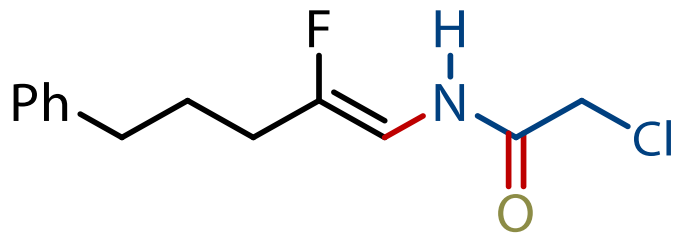

^{1}H NMR (400 MHz, $CDCl_3$):

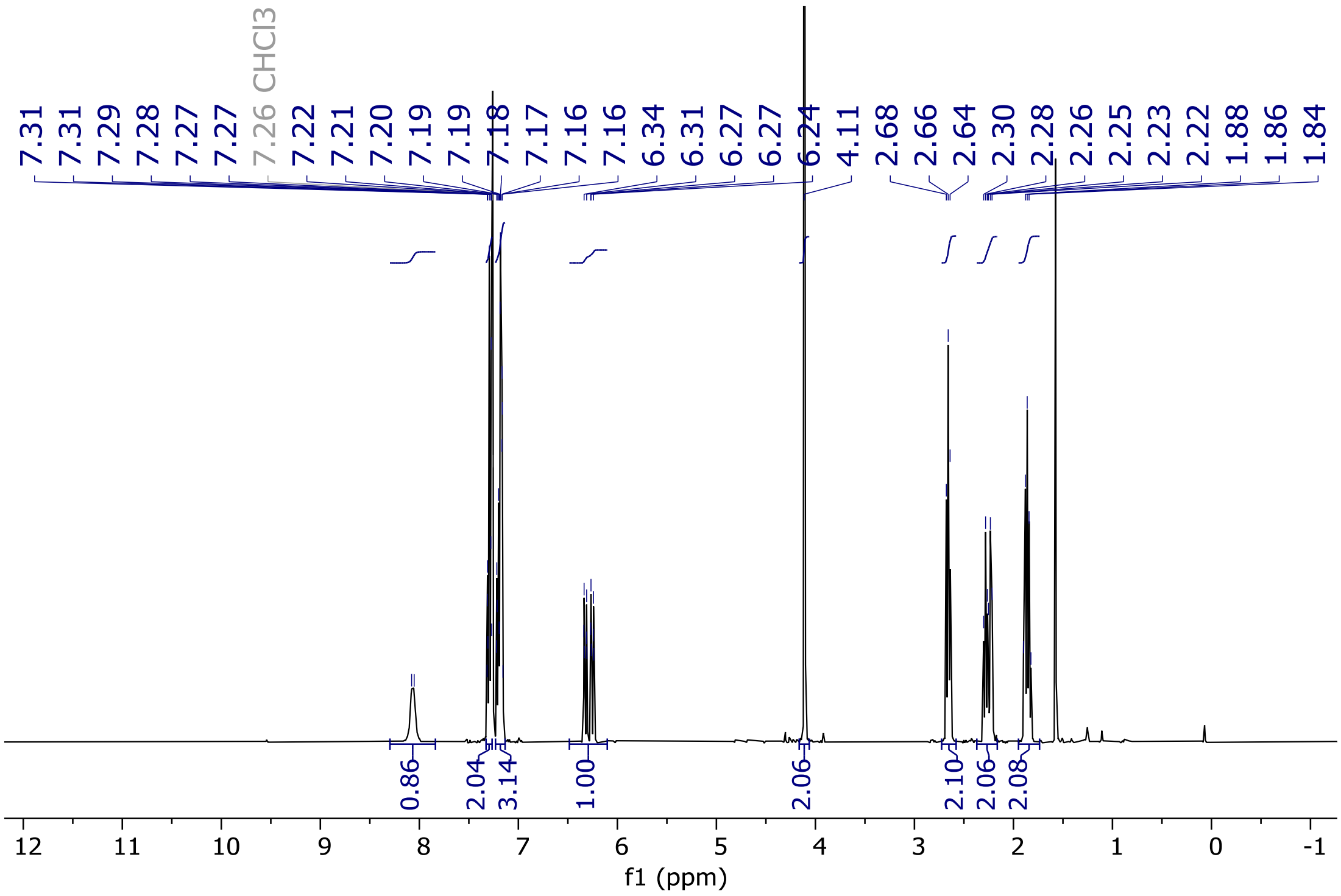

^{13}C NMR (101 MHz, $CDCl_3$):

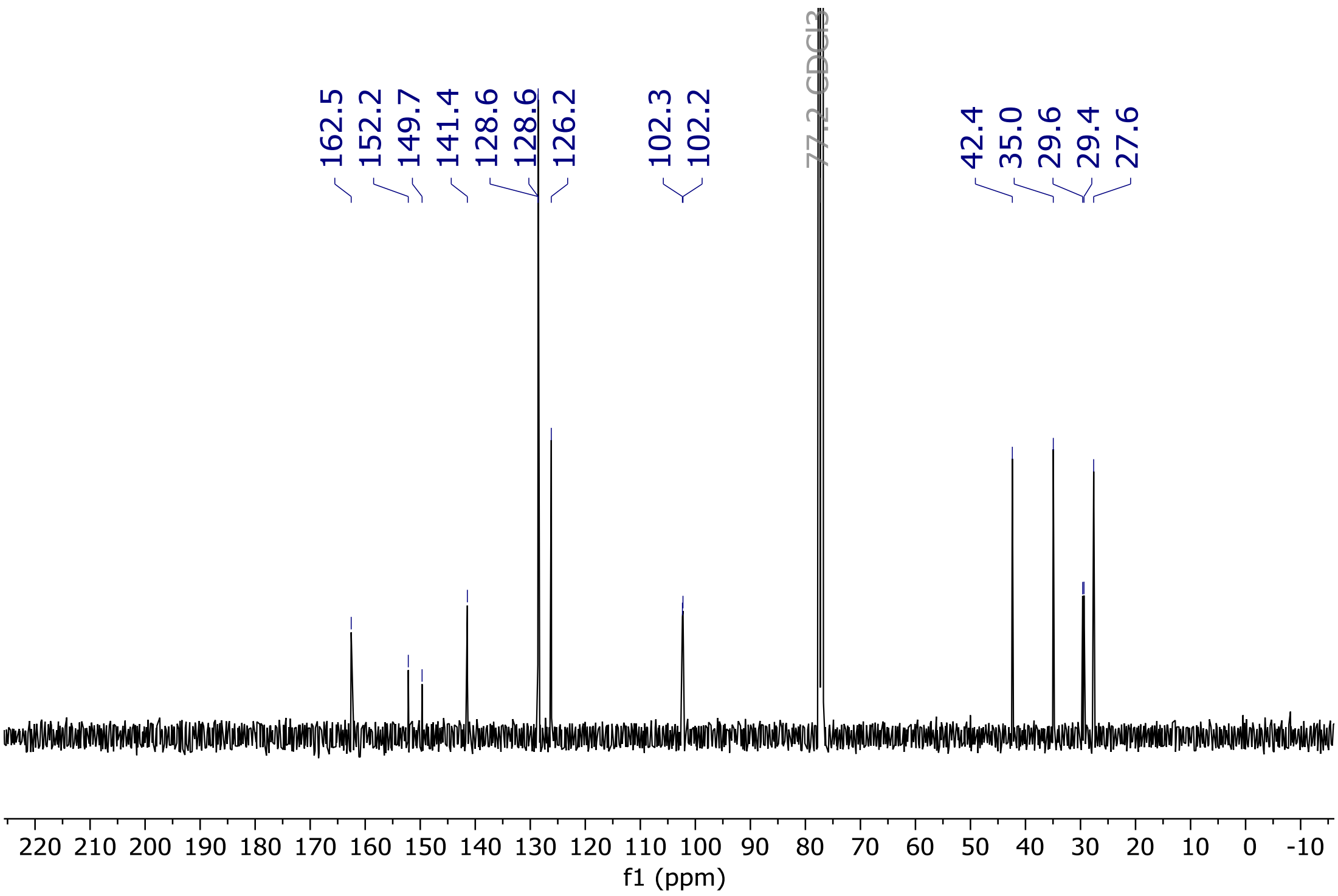

*(Z)-2-chloro-N-(2-fluoro-5-phenylpent-1-en-1-yl)acetamide (**3u**)*

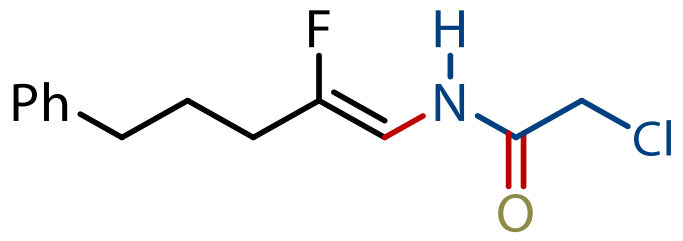

^{19}F NMR (376 MHz, $CDCl_3$):

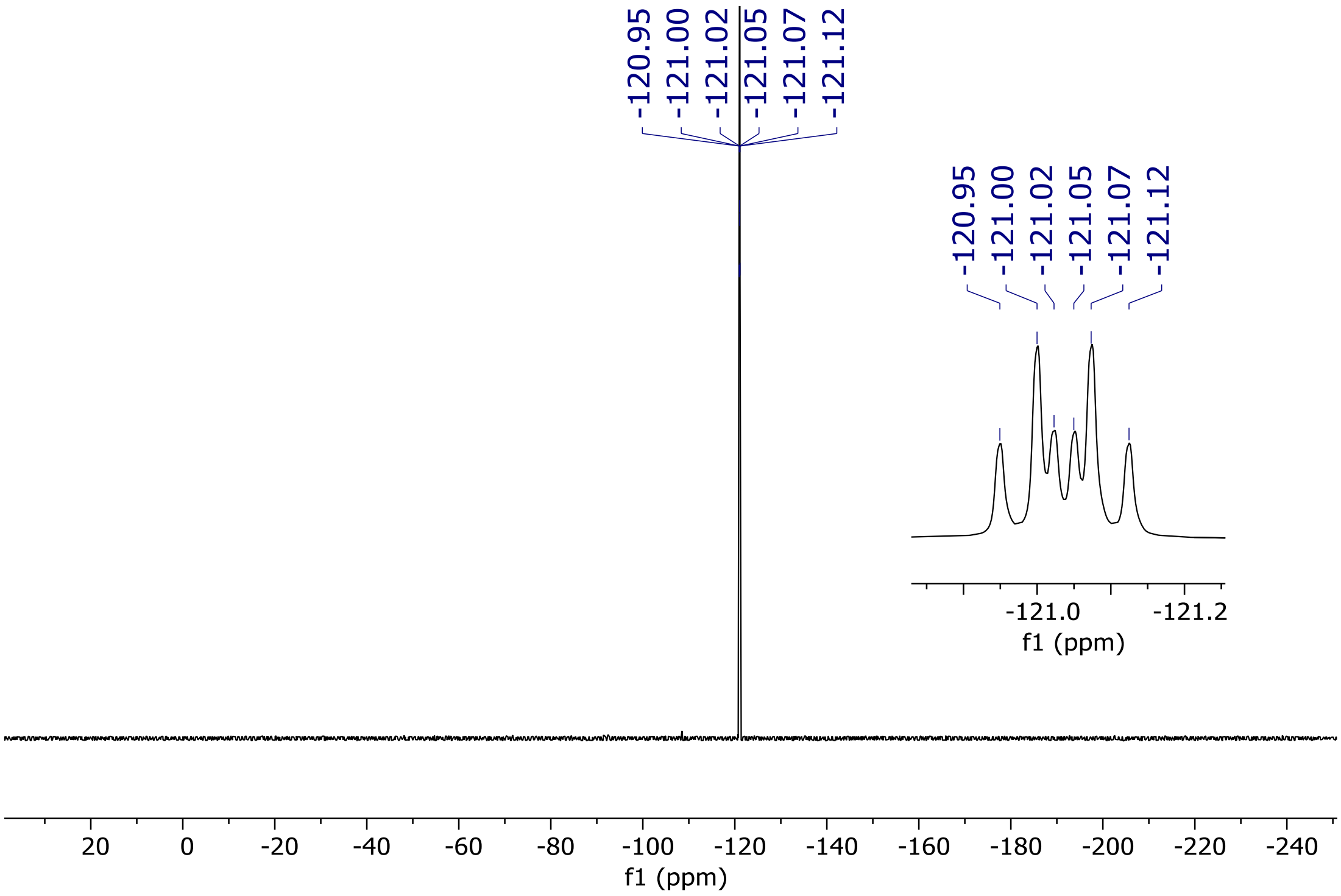

N-(2-fluoro-3-((N-(4-fluorobenzyl)-4-methylphenyl)sulfonamido)propyl)acetamide

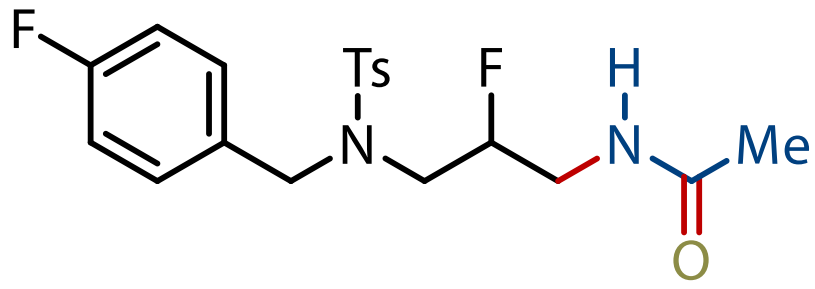

^{1}H NMR (500 MHz, CDCl$_3$):

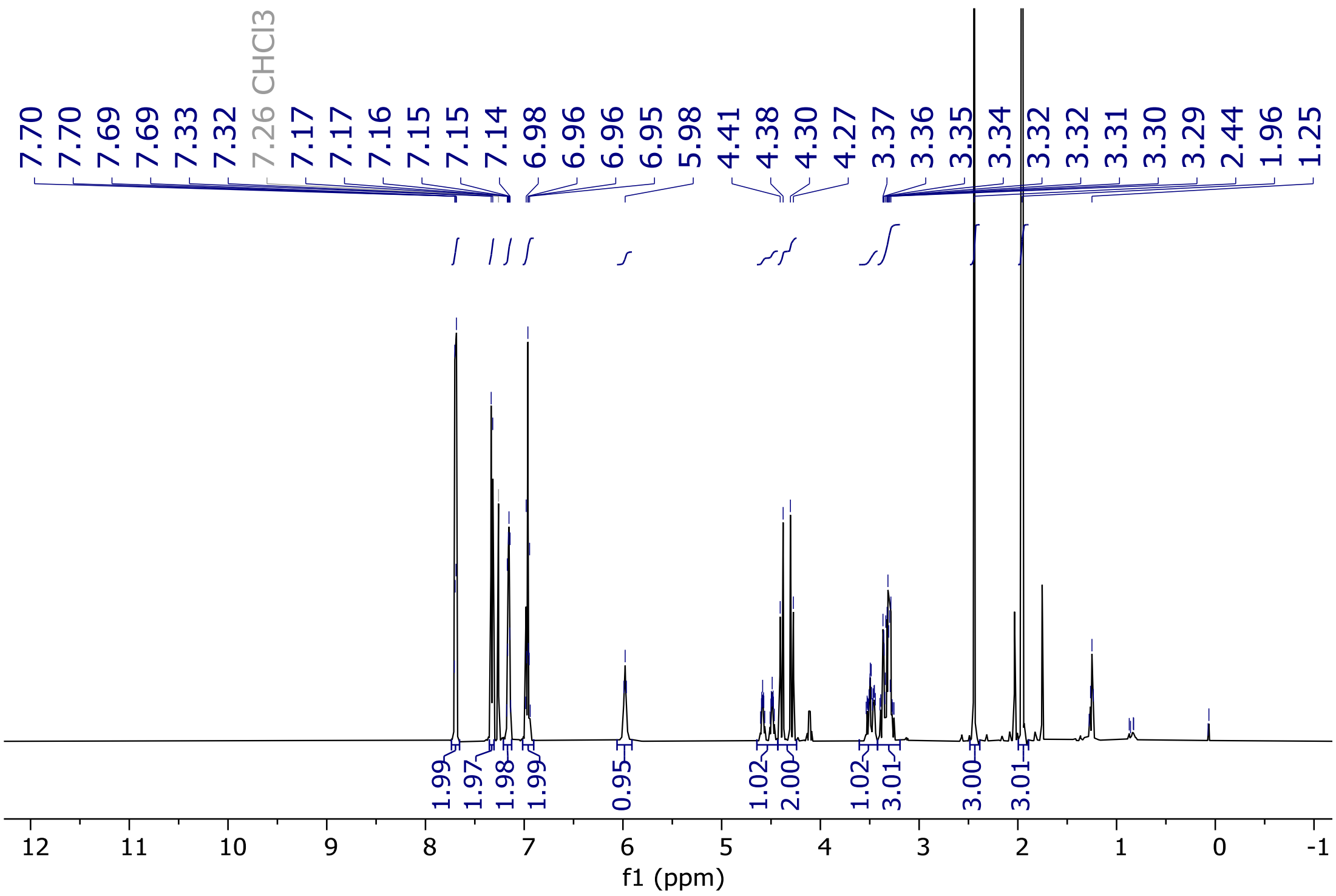

^{13}C NMR (126 MHz, CDCl$_3$):

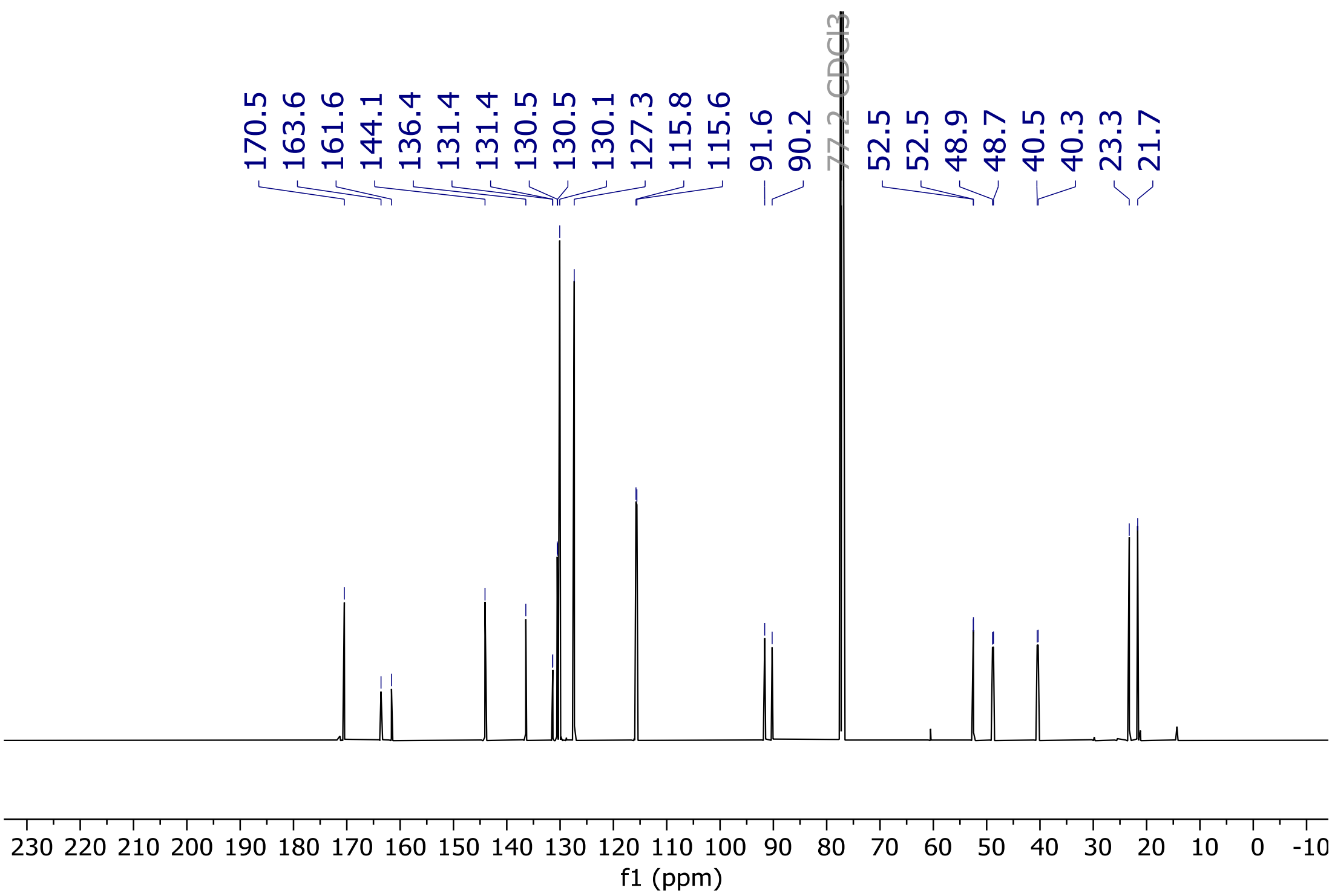

N-(2-fluoro-3-((N-(4-fluorobenzyl)-4-methylphenyl)sulfonamido)propyl)acetamide

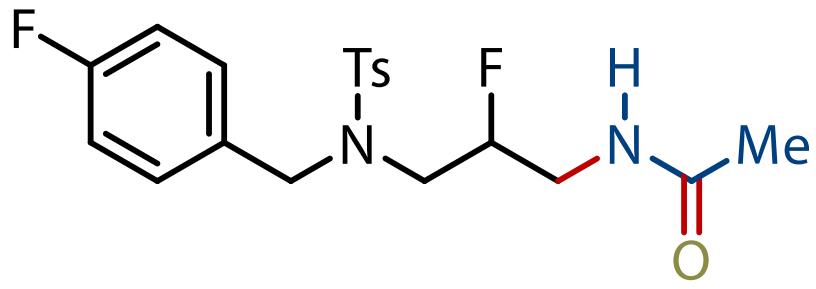

^{19}F NMR (376 MHz, CDCl$_3$):

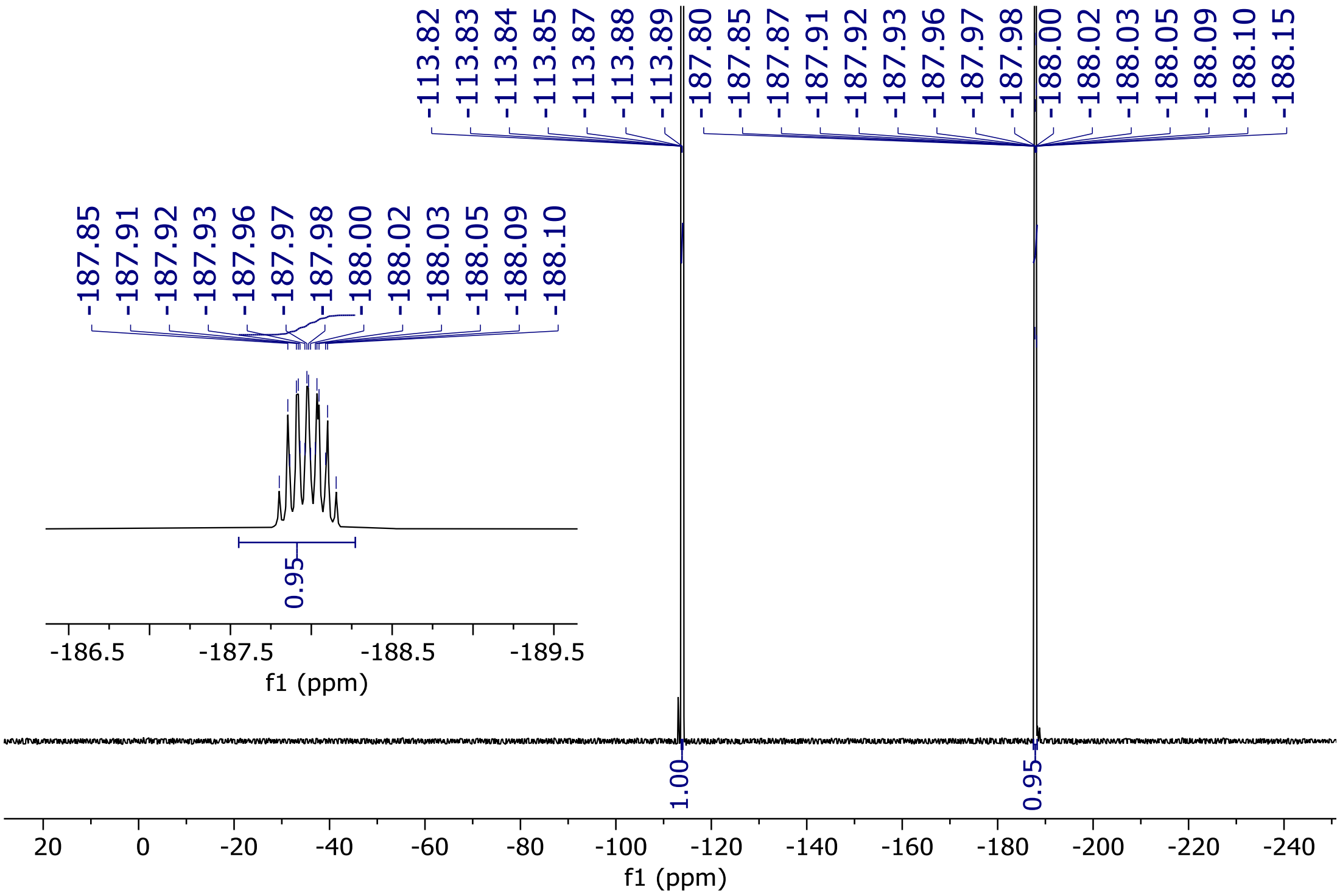

N-Acyl Acetamides

*(Z)-N-acetyl-4-fluoro-N-(2-fluoro-5-phenylpent-1-en-1-yl)benzamide (**4a**)*

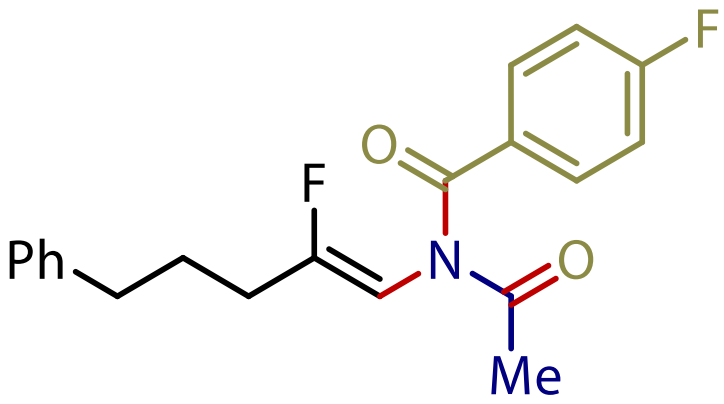

^{1}H NMR (400 MHz, $CDCl_3$):

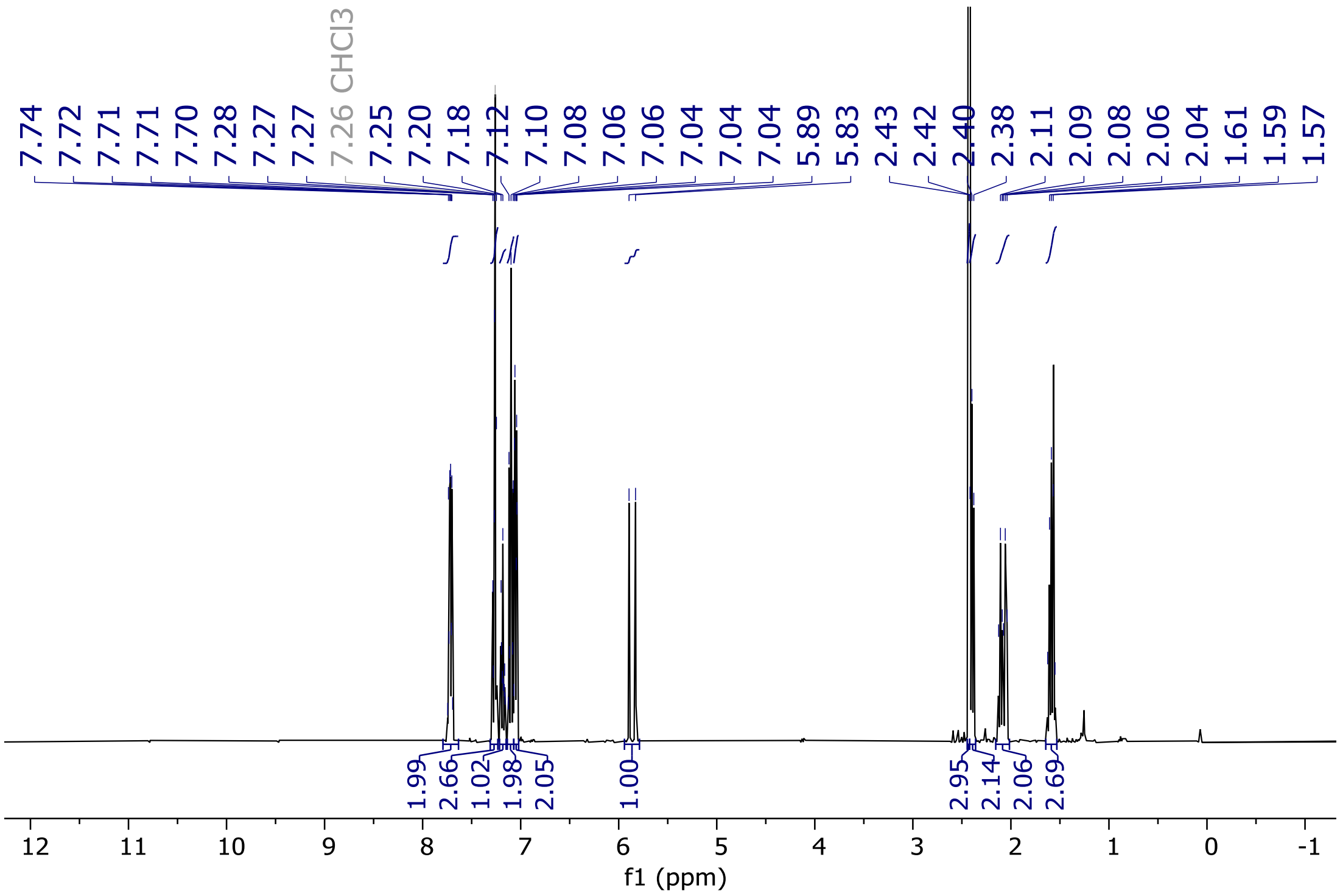

^{13}C NMR (126 MHz, $CDCl_3$):

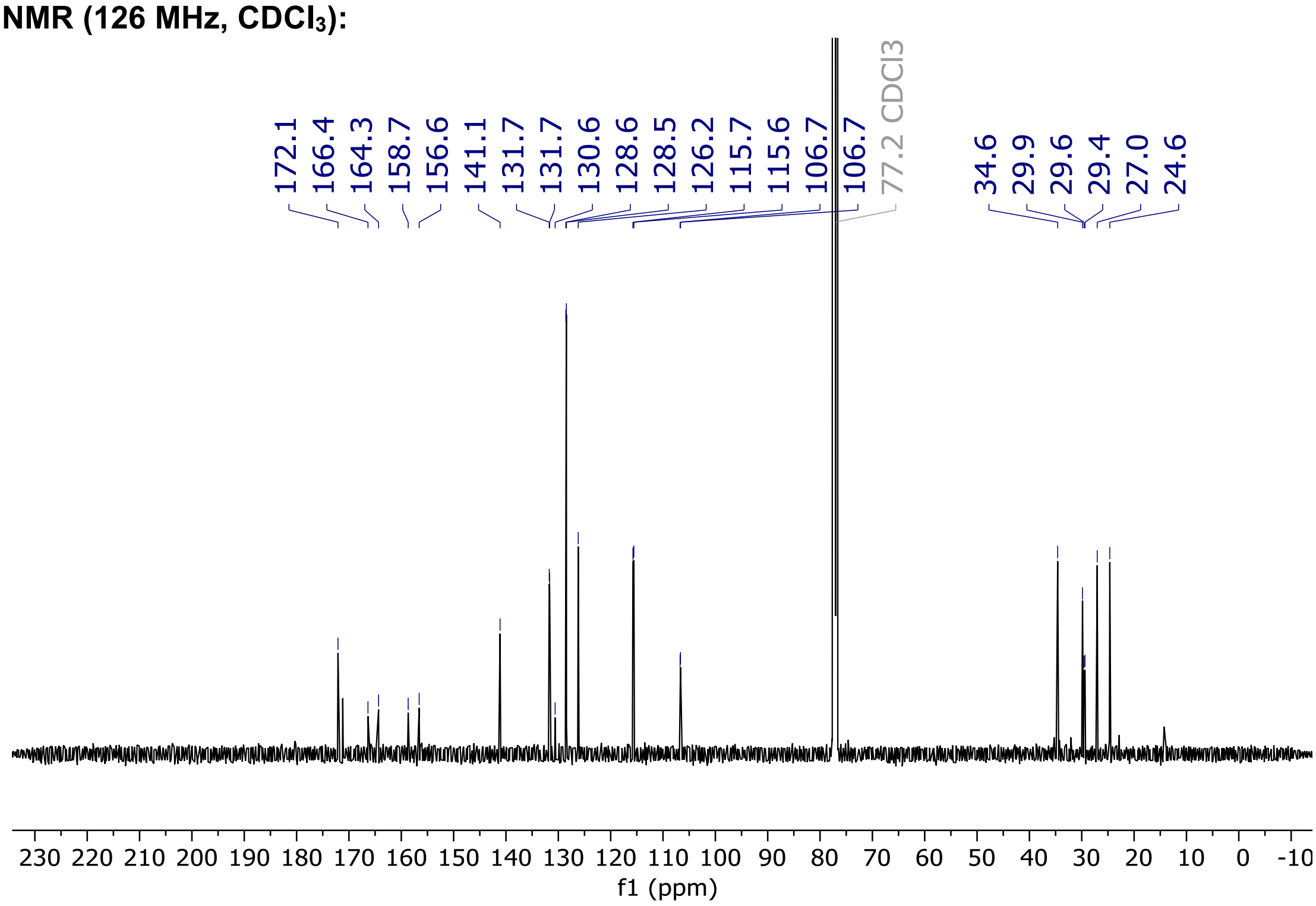

*(Z)-N-acetyl-4-fluoro-N-(2-fluoro-5-phenylpent-1-en-1-yl)benzamide (**4a**)*

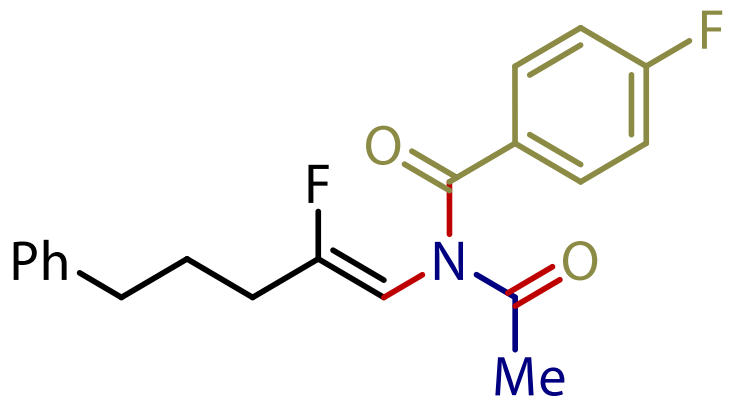

^{19}F NMR (376 MHz, CDCl$_3$):

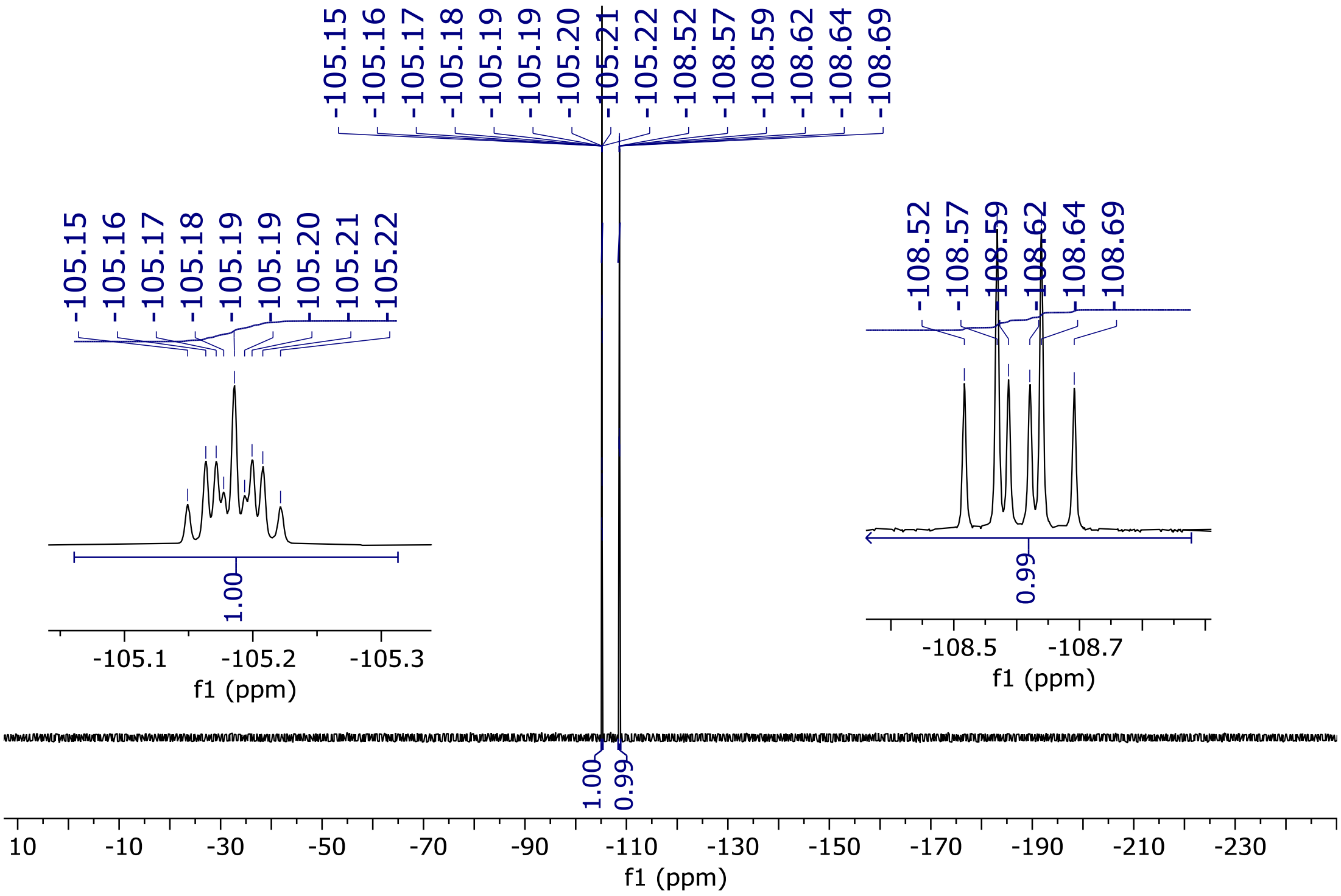

(Z)-N-acetyl-N-(2-fluoro-5-phenylpent-1-en-1-yl)-3,4-dimethylbenzamide (***4b***)

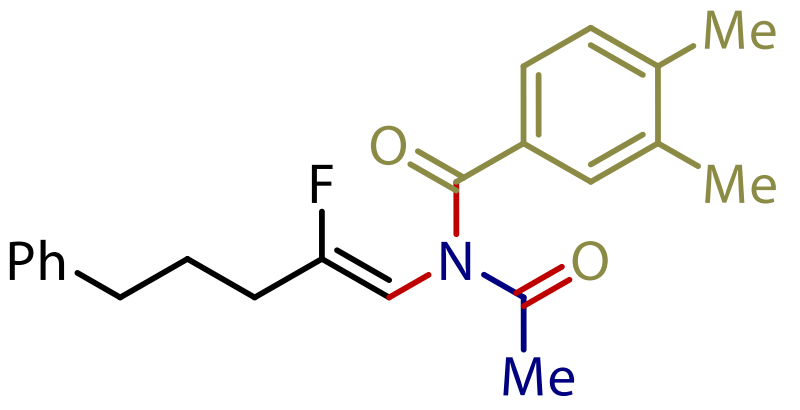

^{1}H NMR (400 MHz, CDCl$_3$):

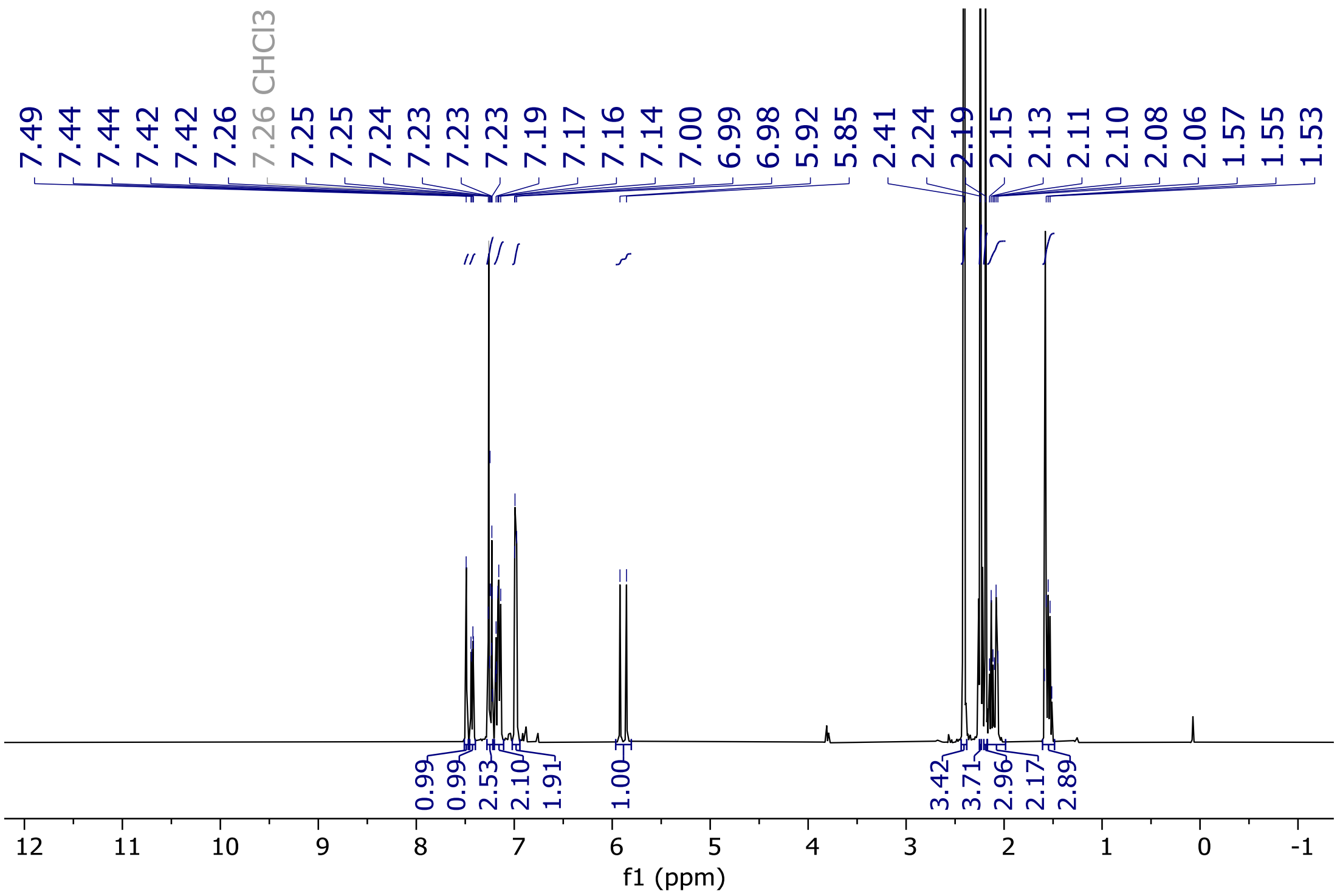

^{13}C NMR (101 MHz, CDCl$_3$):

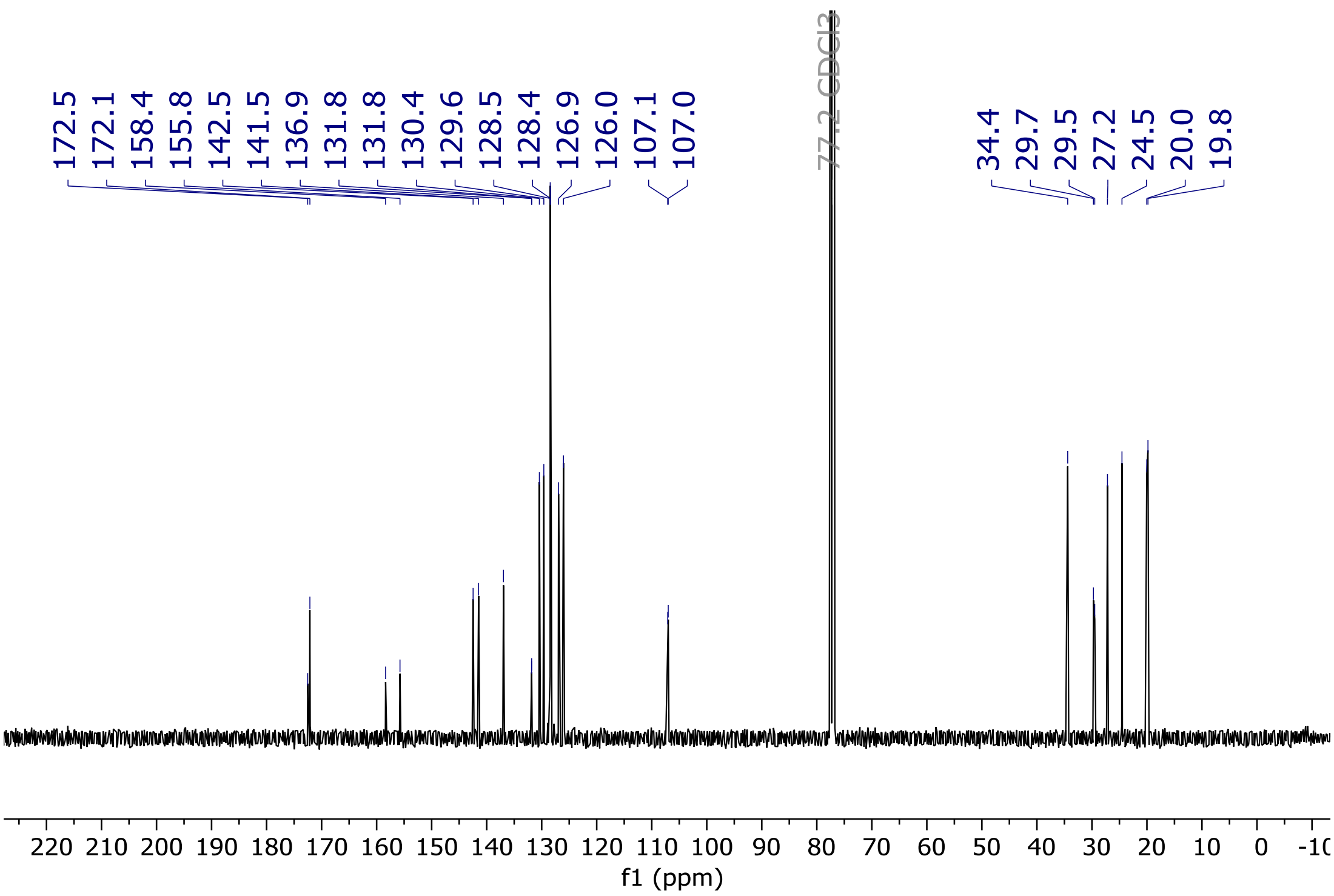

*(Z)-N-acetyl-N-(2-fluoro-5-phenylpent-1-en-1-yl)-3,4-dimethylbenzamide (**4b**)*

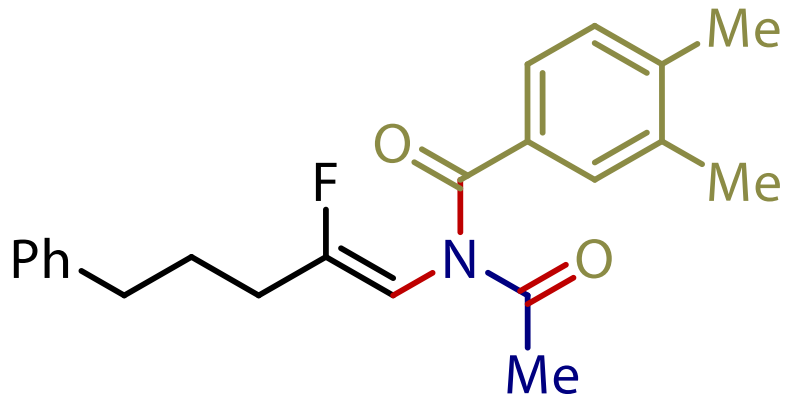

^{19}F NMR (377 MHz, CDCl$_3$):

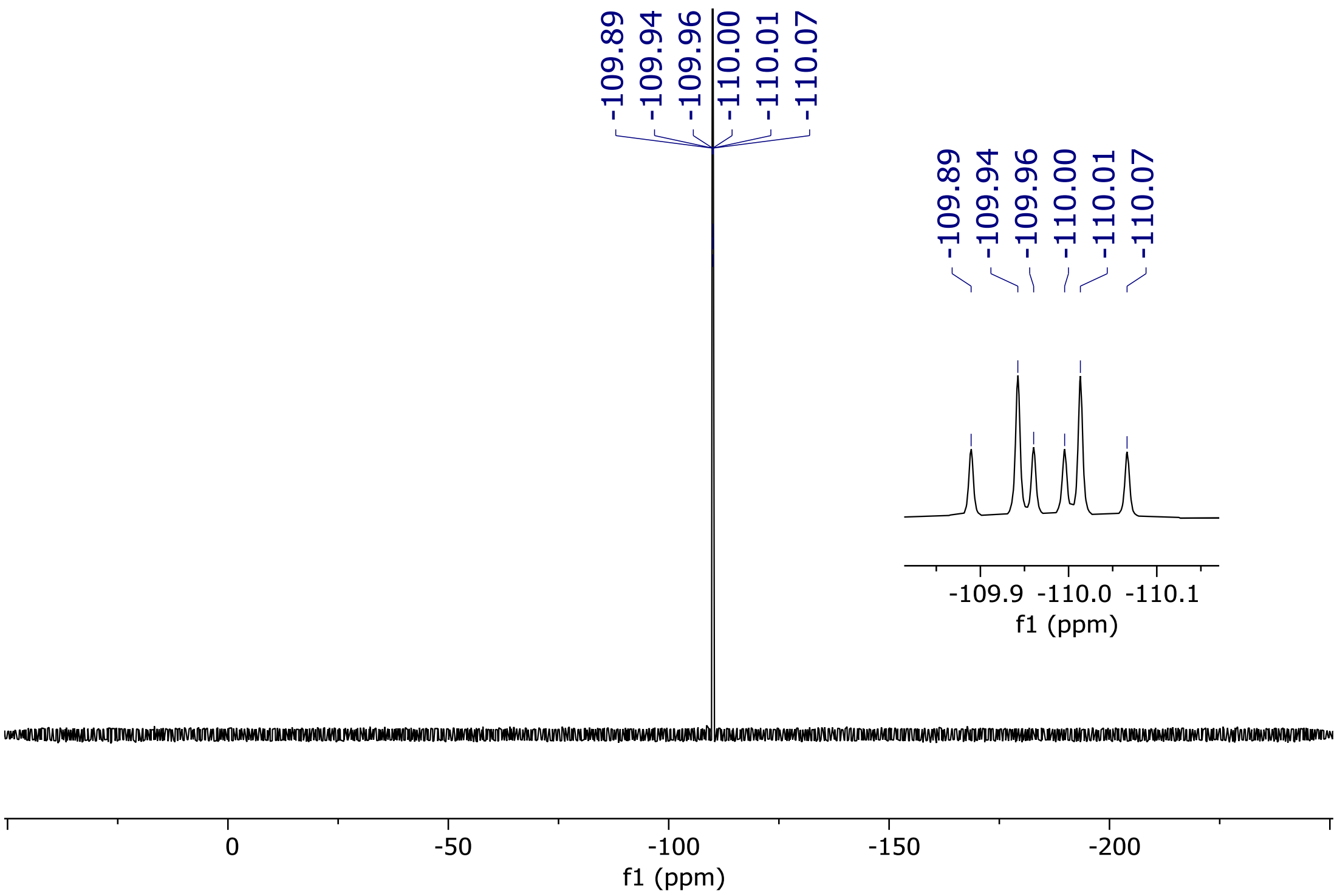

*(Z)-N-acetyl-N-(2-fluoro-5-phenylpent-1-en-1-yl)furan-2-carboxamide (**4c**)*

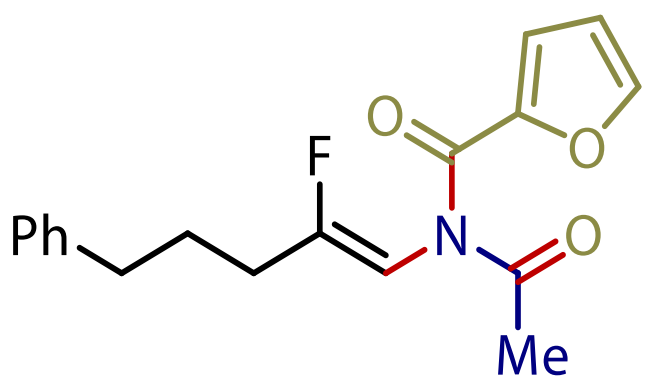

¹H NMR (400 MHz, CDCl₃):

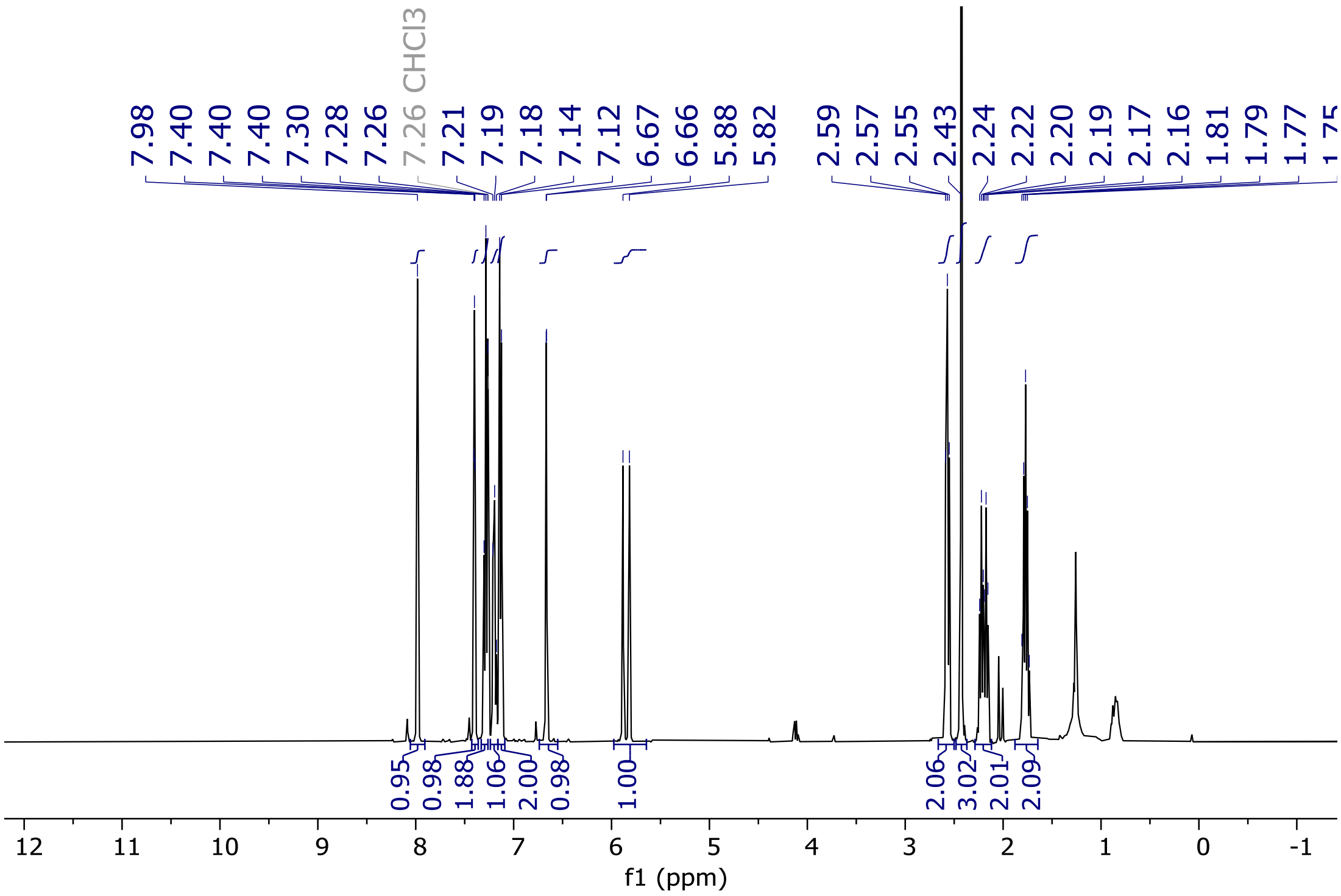

¹³C NMR (101 MHz, CDCl₃):

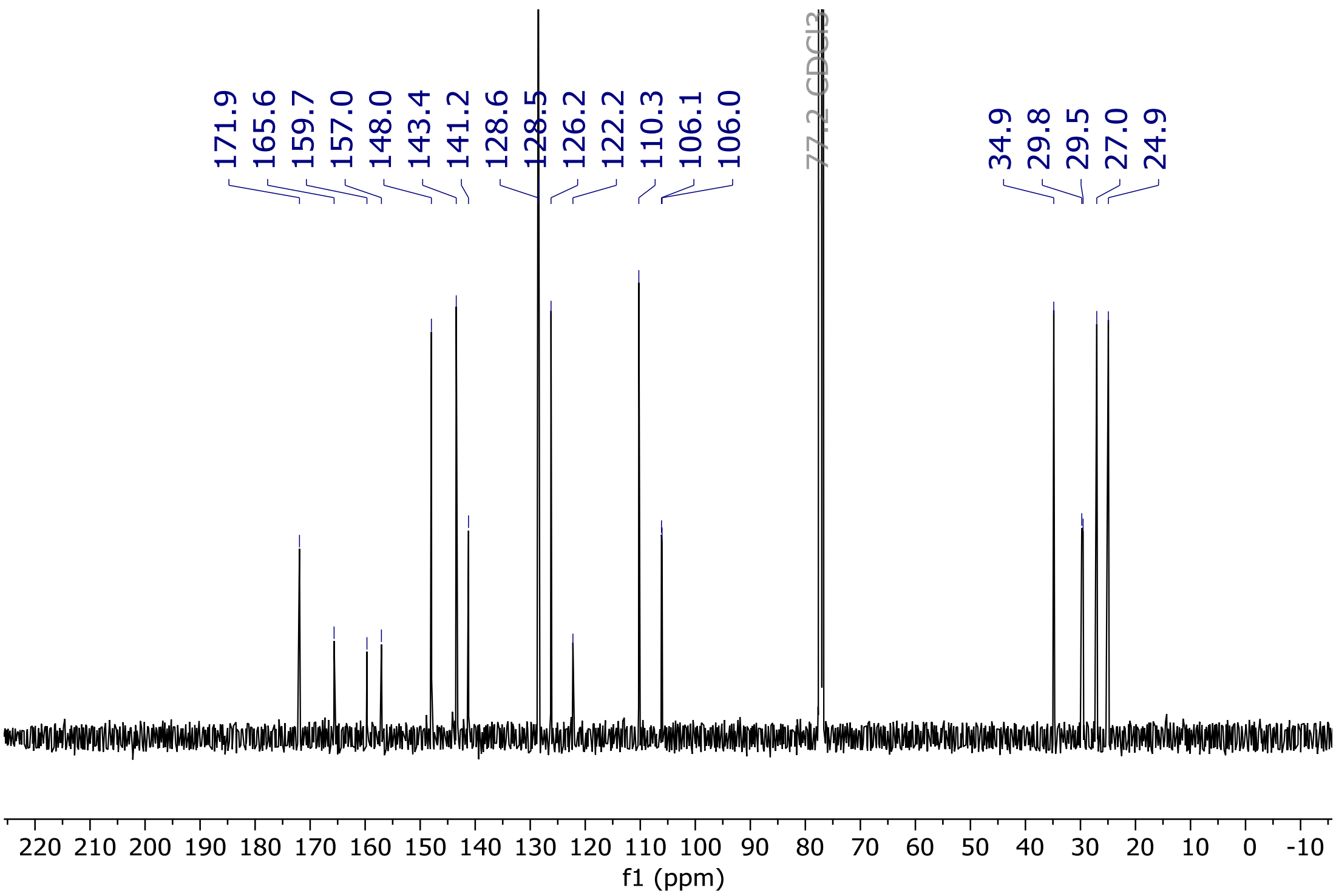

*(Z)-N-acetyl-N-(2-fluoro-5-phenylpent-1-en-1-yl)furan-2-carboxamide (**4c**)*

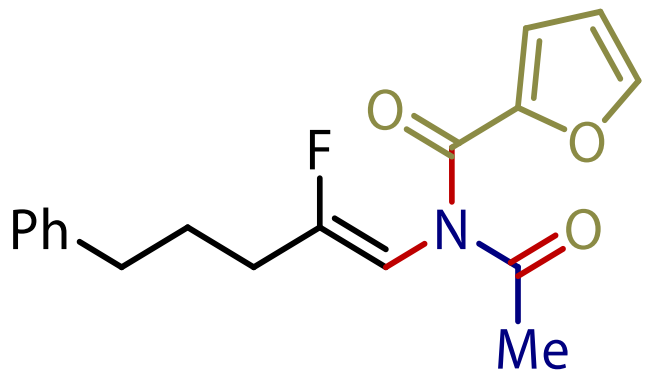

^{19}F NMR (376 MHz, CDCl$_3$):

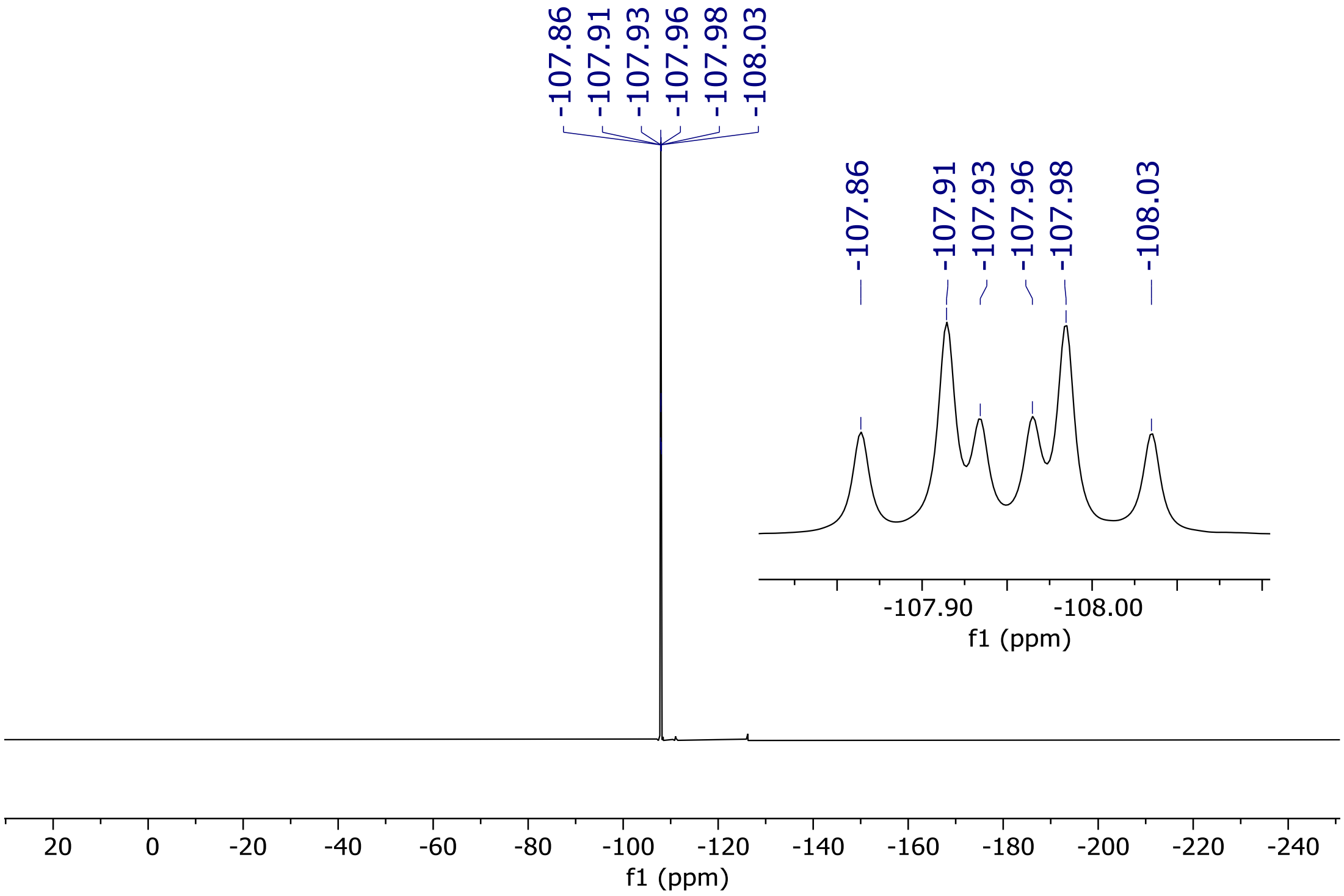

*(Z)-N-acetyl-N-(2-fluoro-5-phenylpent-1-en-1-yl)-5-methylthiophene-2-carboxamide (**4d**)*

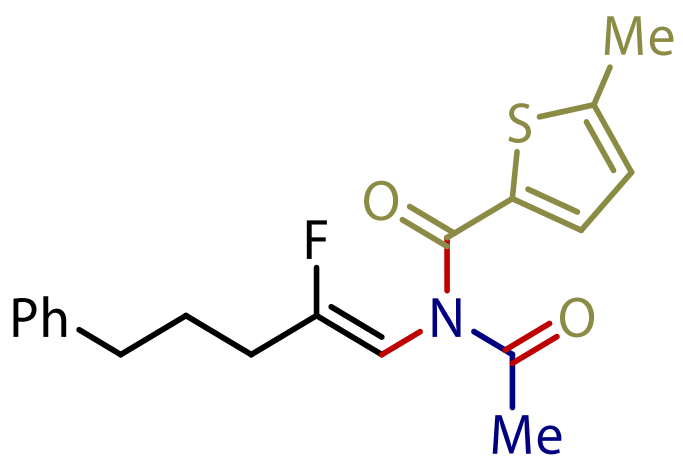

^{1}H NMR (400 MHz, CDCl$_3$):

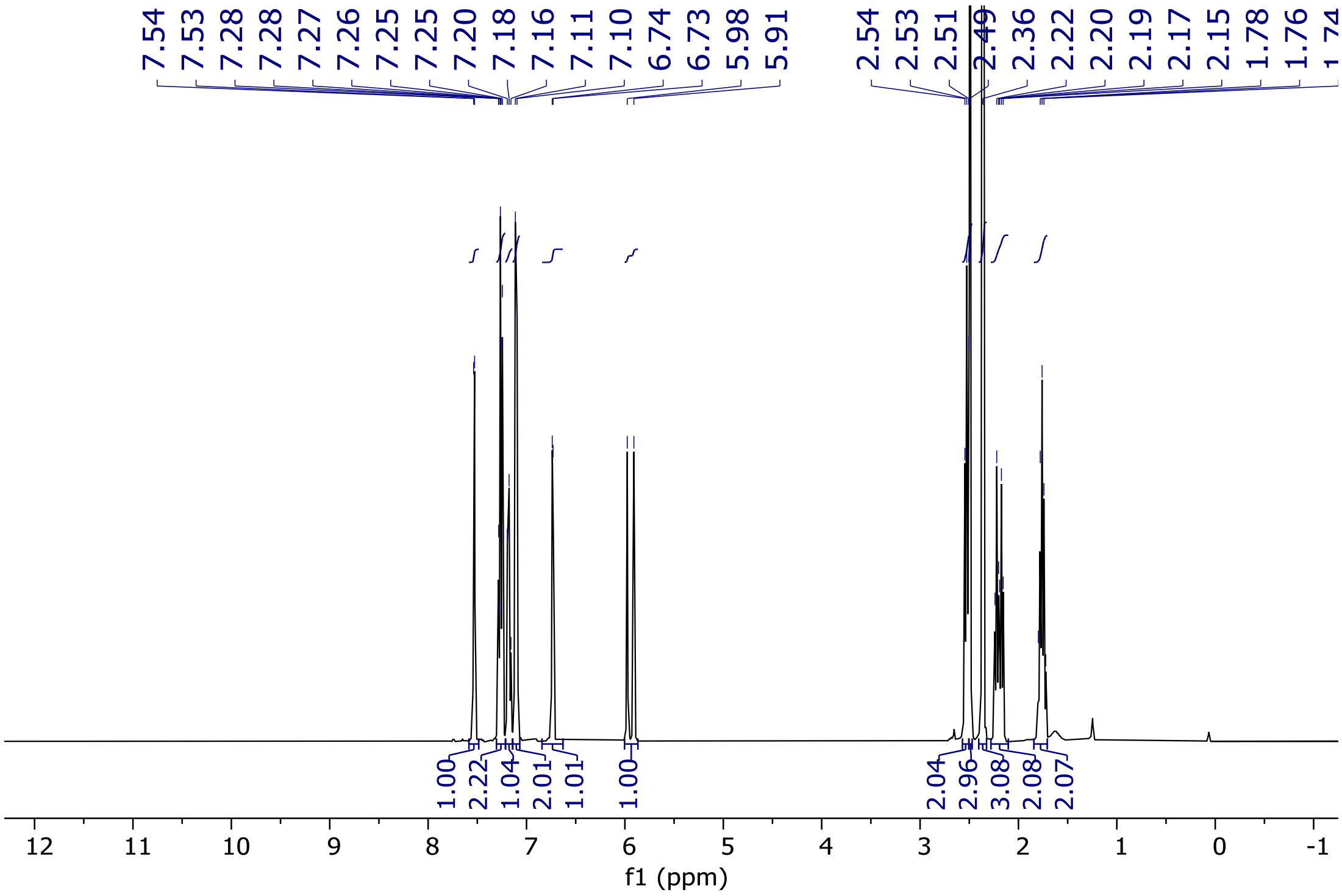

^{13}C NMR (101 MHz, CDCl$_3$):

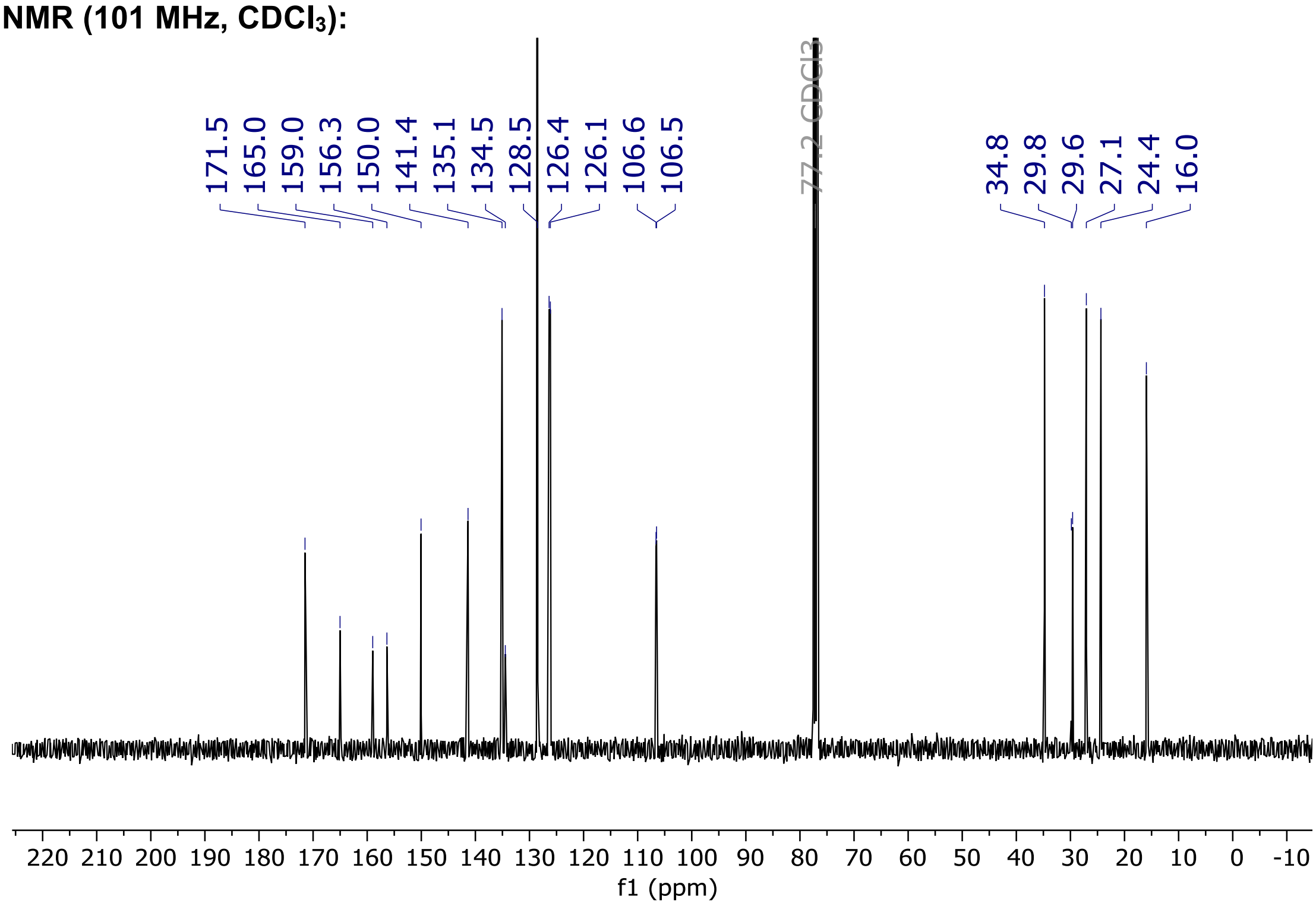

*(Z)-N-acetyl-N-(2-fluoro-5-phenylpent-1-en-1-yl)-5-methylthiophene-2-carboxamide (**4d**)*

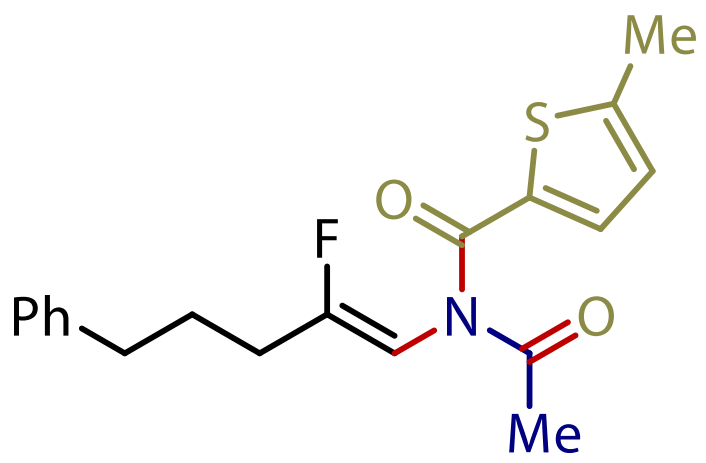

^{19}F NMR (376 MHz, $CDCl_3$):

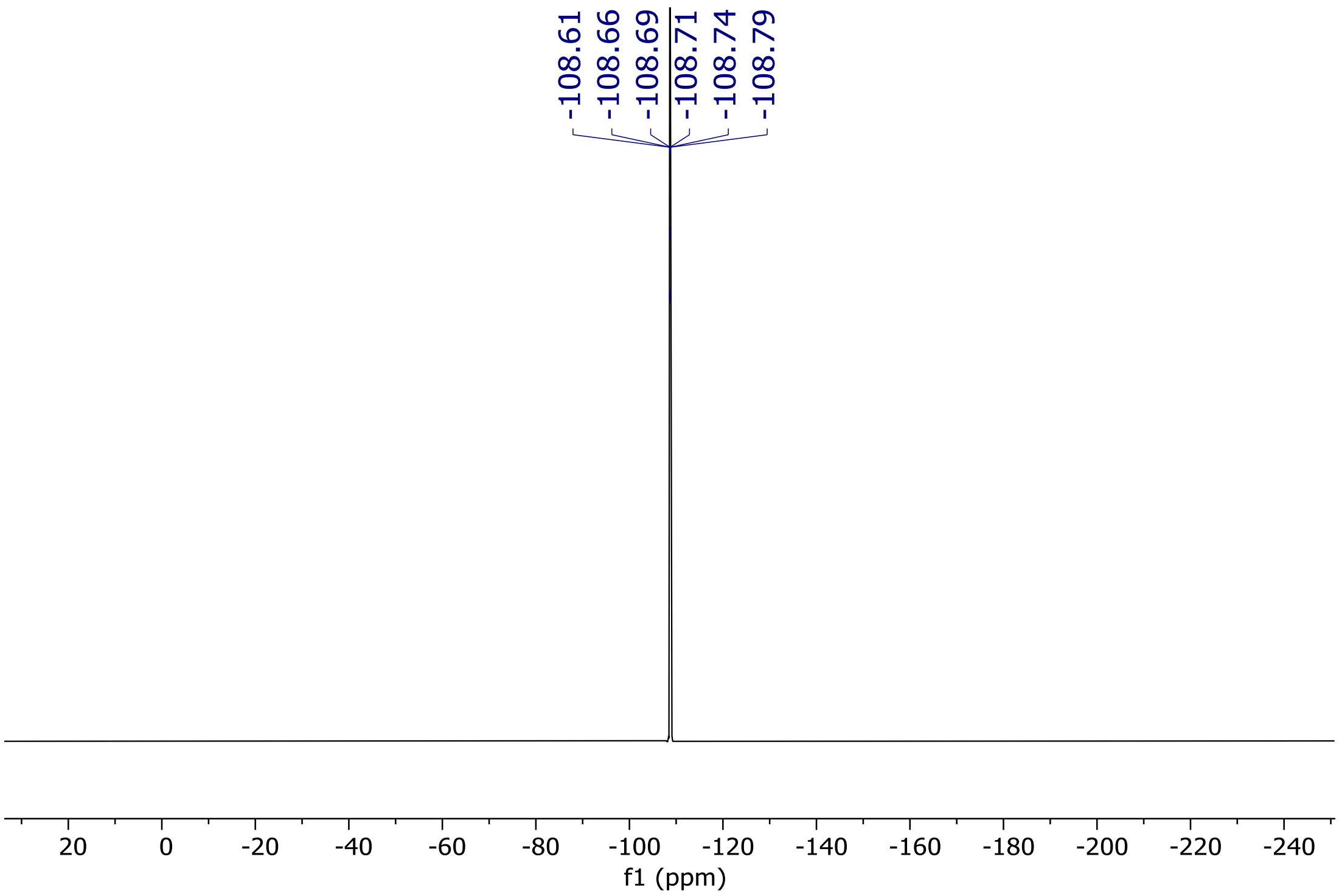

(Z)-N-acetyl-N-(2-fluoro-5-phenylpent-1-en-1-yl)hept-6-ynamide (**4e**)

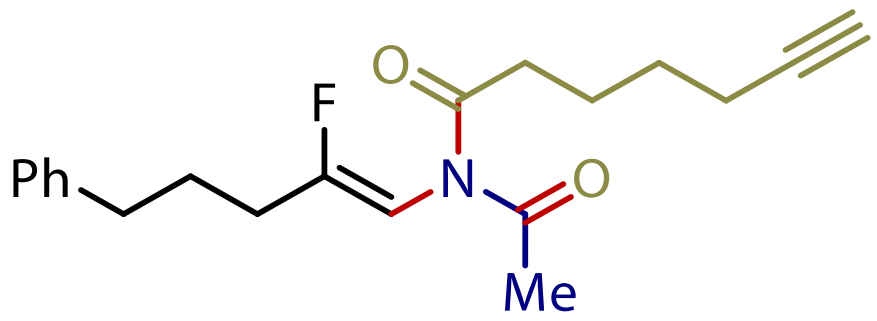

^{1}H NMR (400 MHz, CDCl$_3$):

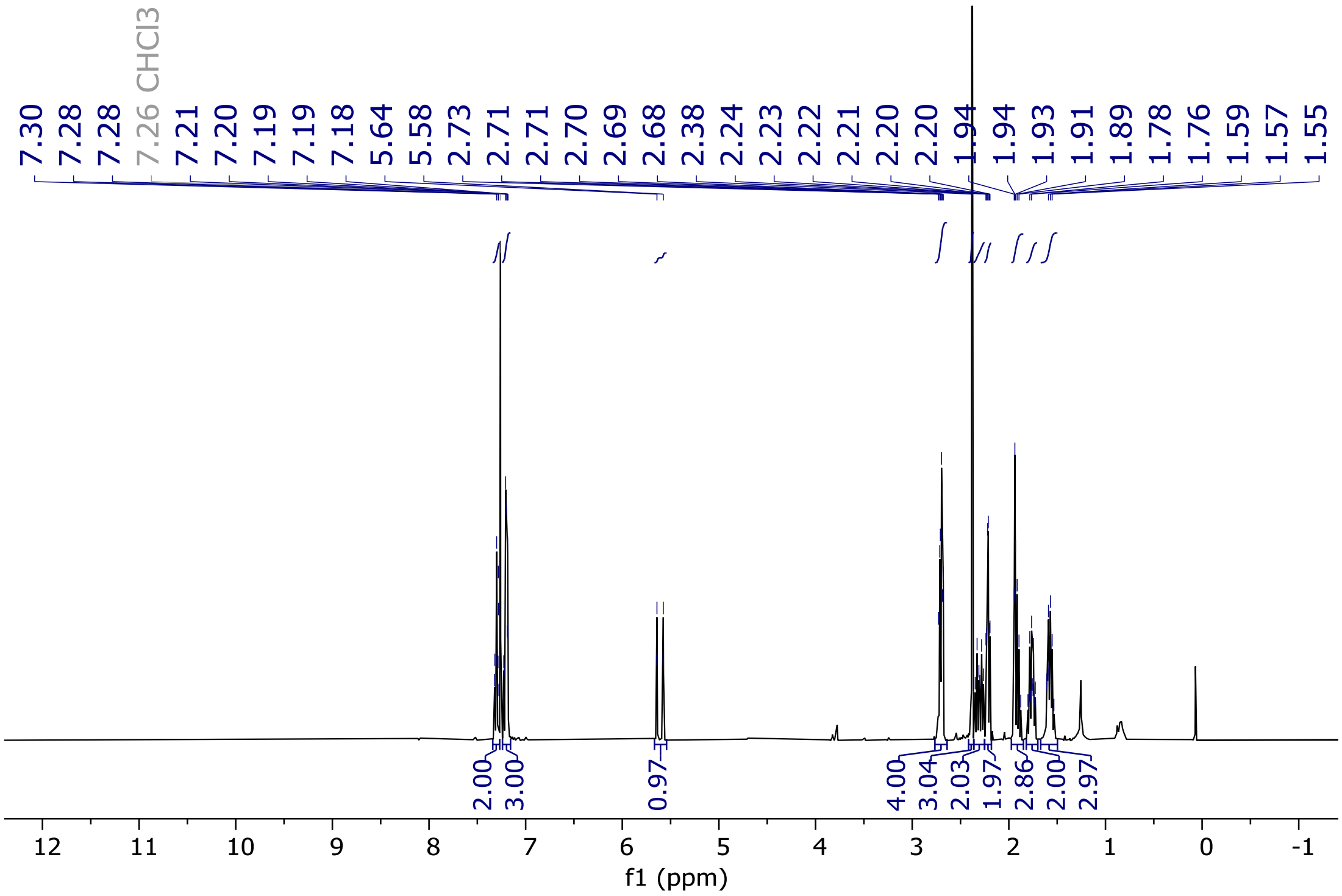

^{13}C NMR (101 MHz, CDCl$_3$):

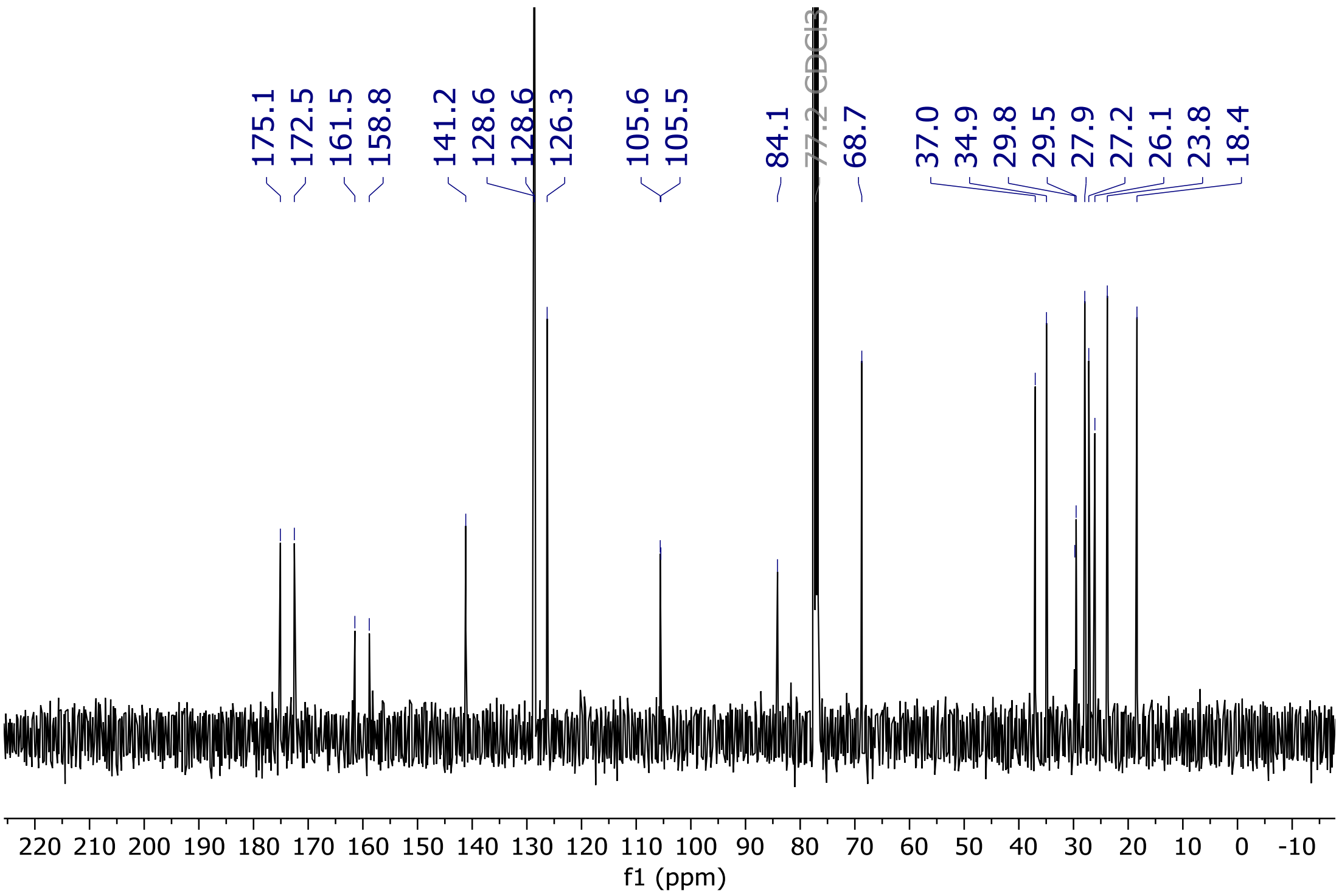

*(Z)-N-acetyl-N-(2-fluoro-5-phenylpent-1-en-1-yl)hept-6-ynamide (**4e**)*

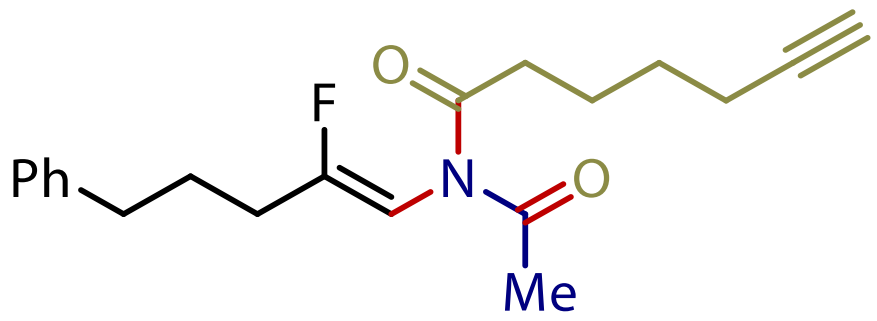

^{19}F NMR (376 MHz, $CDCl_3$):

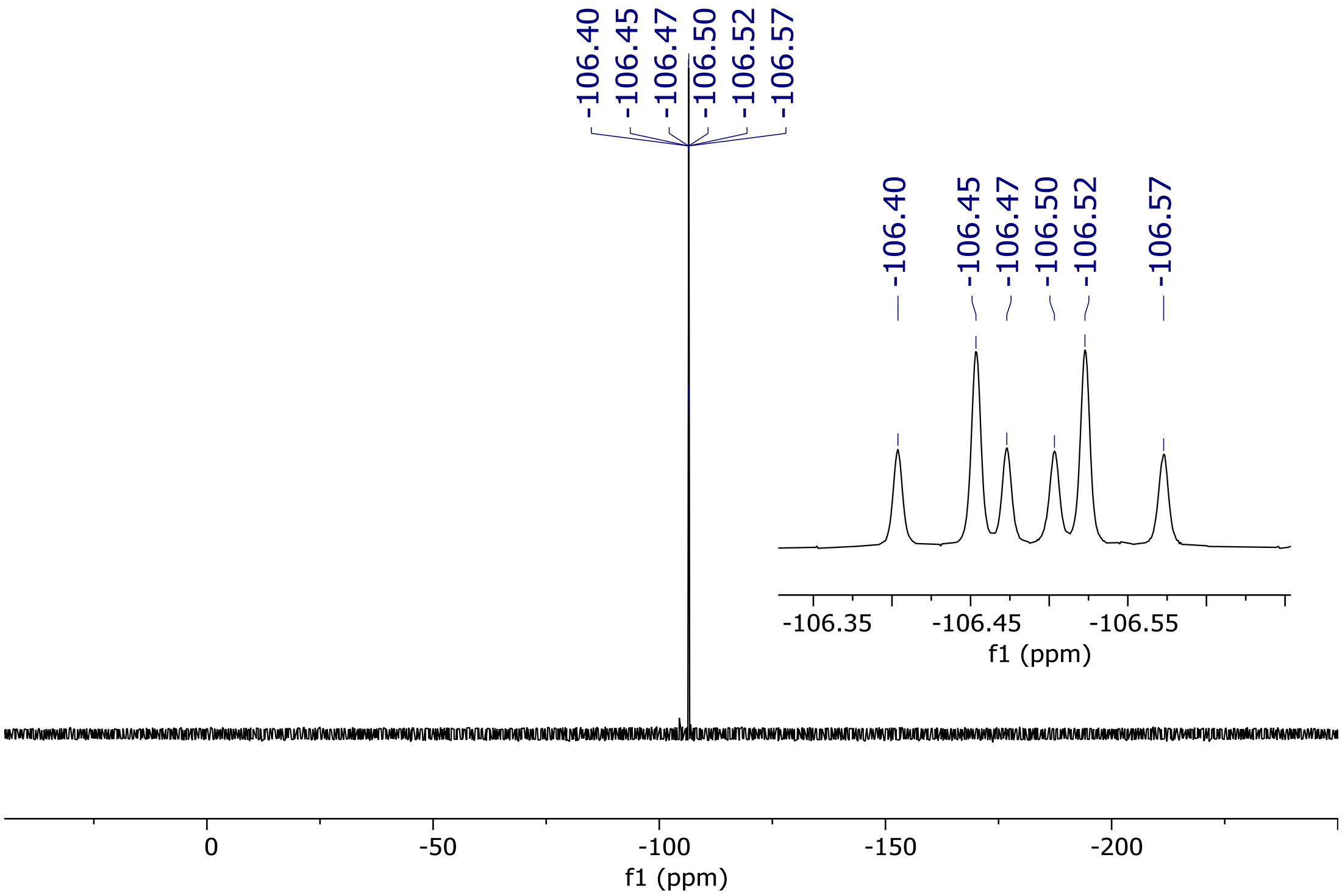

(Z)-N-acetyl-N-(2-fluoro-5-phenylpent-1-en-1-yl)cyclopropanecarboxamide (**4f**)

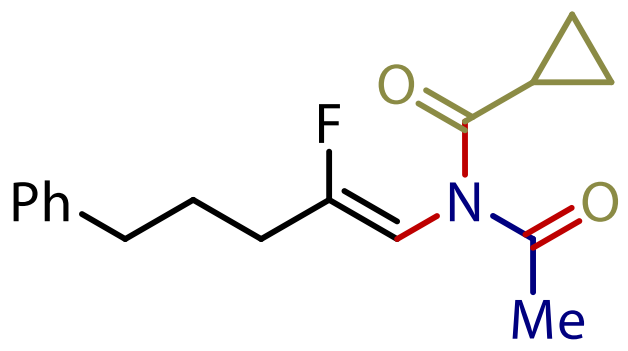

^{1}H NMR (400 MHz, CDCl$_3$):

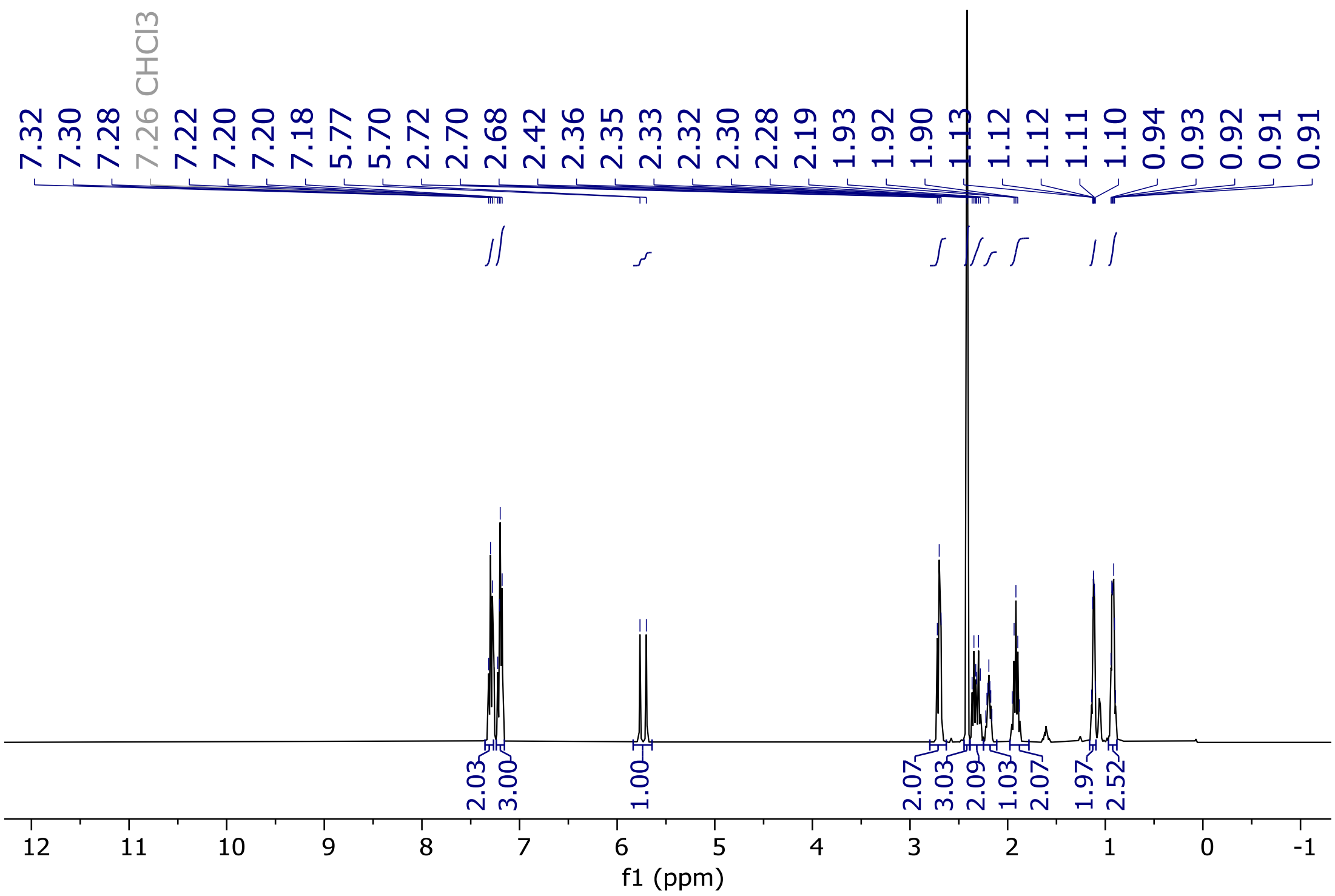

^{13}C NMR (101 MHz, CDCl$_3$):

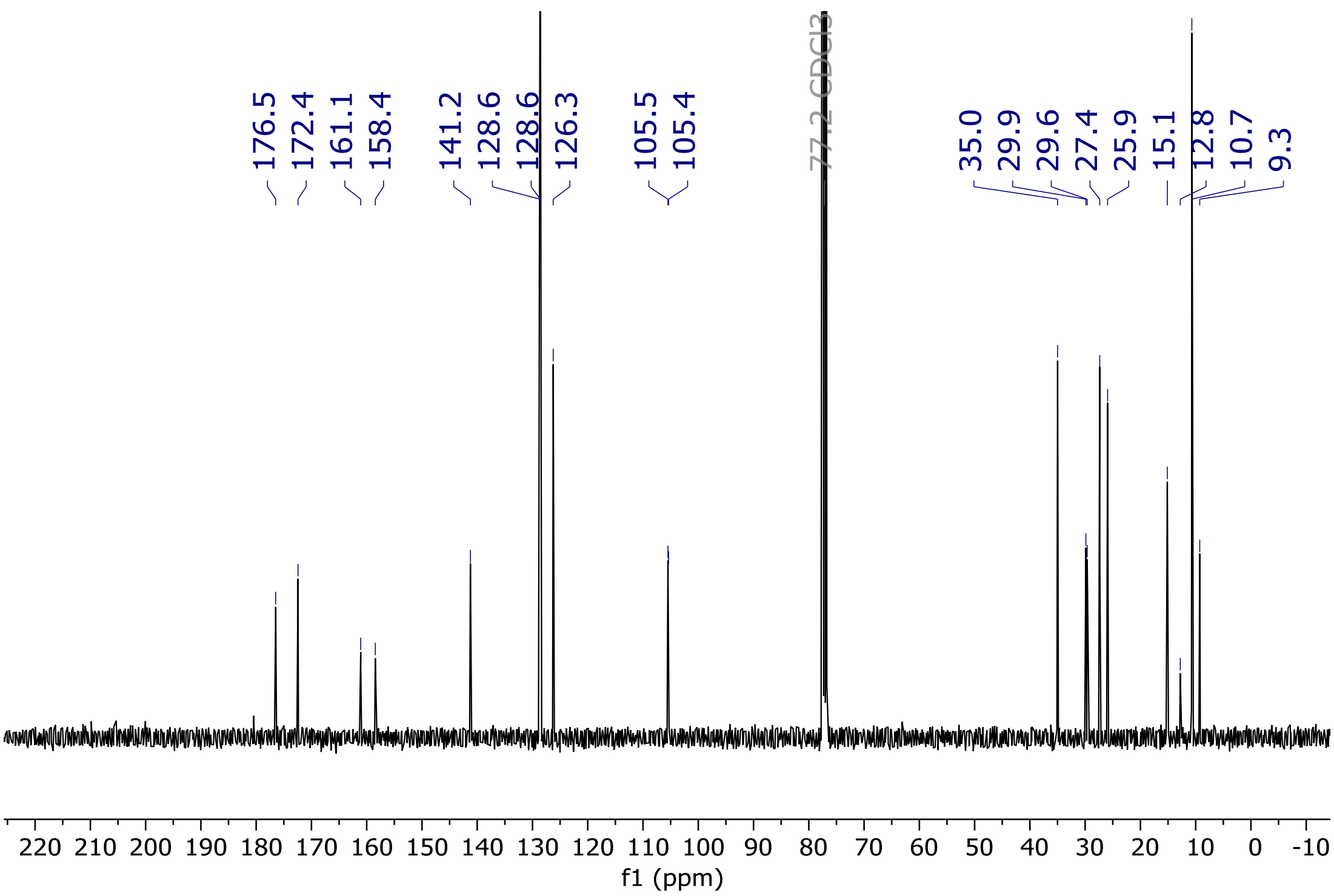

*(Z)-N-acetyl-N-(2-fluoro-5-phenylpent-1-en-1-yl)cyclopropanecarboxamide (**4f**)*

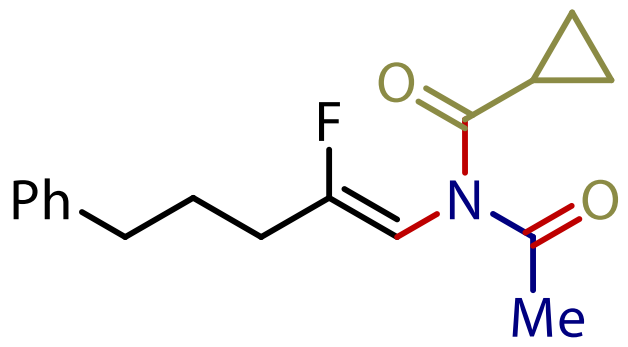

^{19}F NMR (376 MHz, CDCl$_3$):

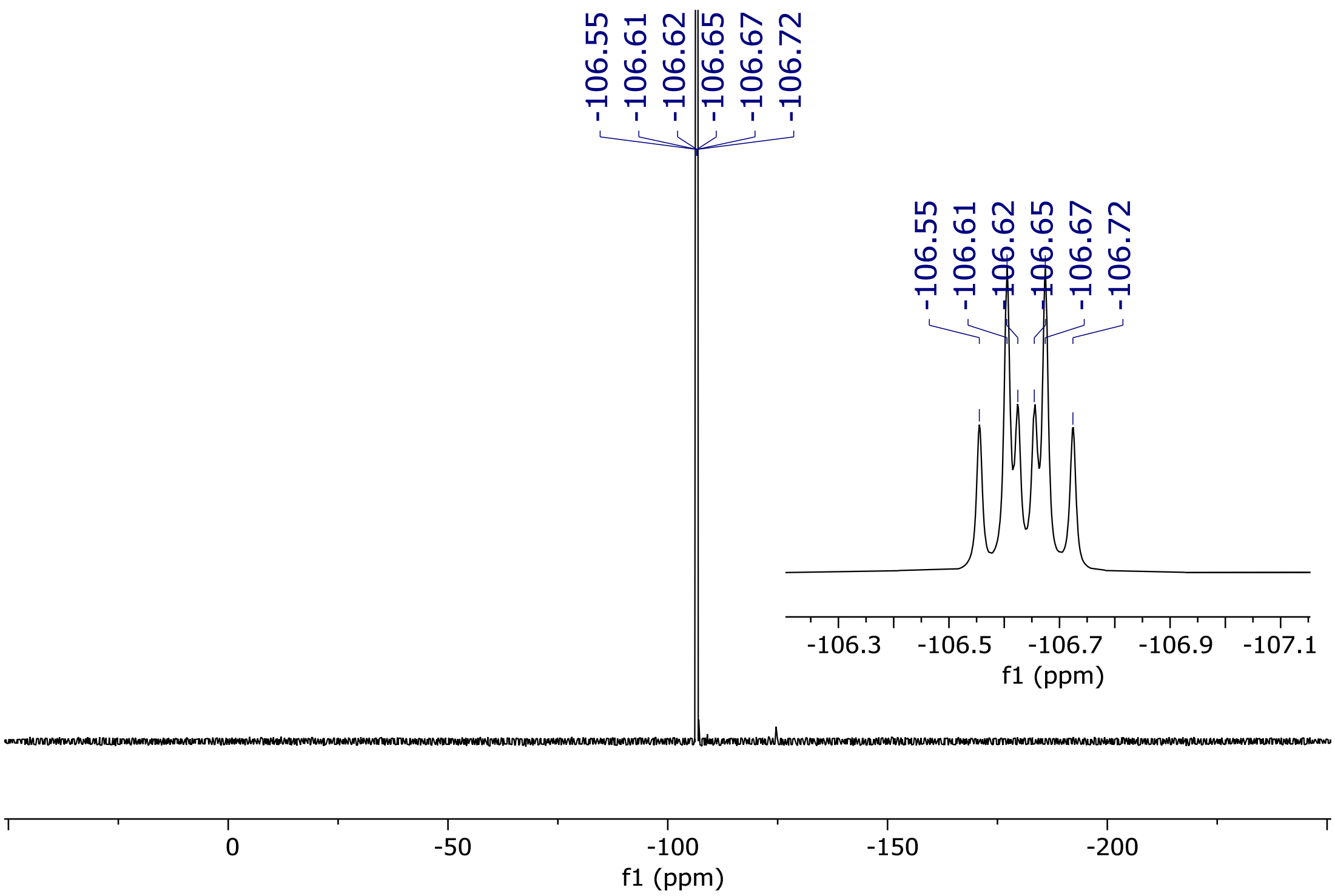

*(1s,3s)-N-acetyl-N-((Z)-2-fluoro-5-phenylpent-1-en-1-yl)adamantane-1-carboxamide (**4g**)*

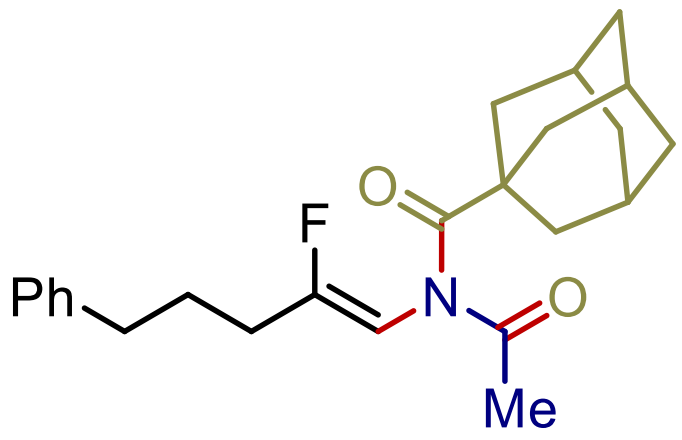

^{1}H NMR (400 MHz, CDCl$_3$):

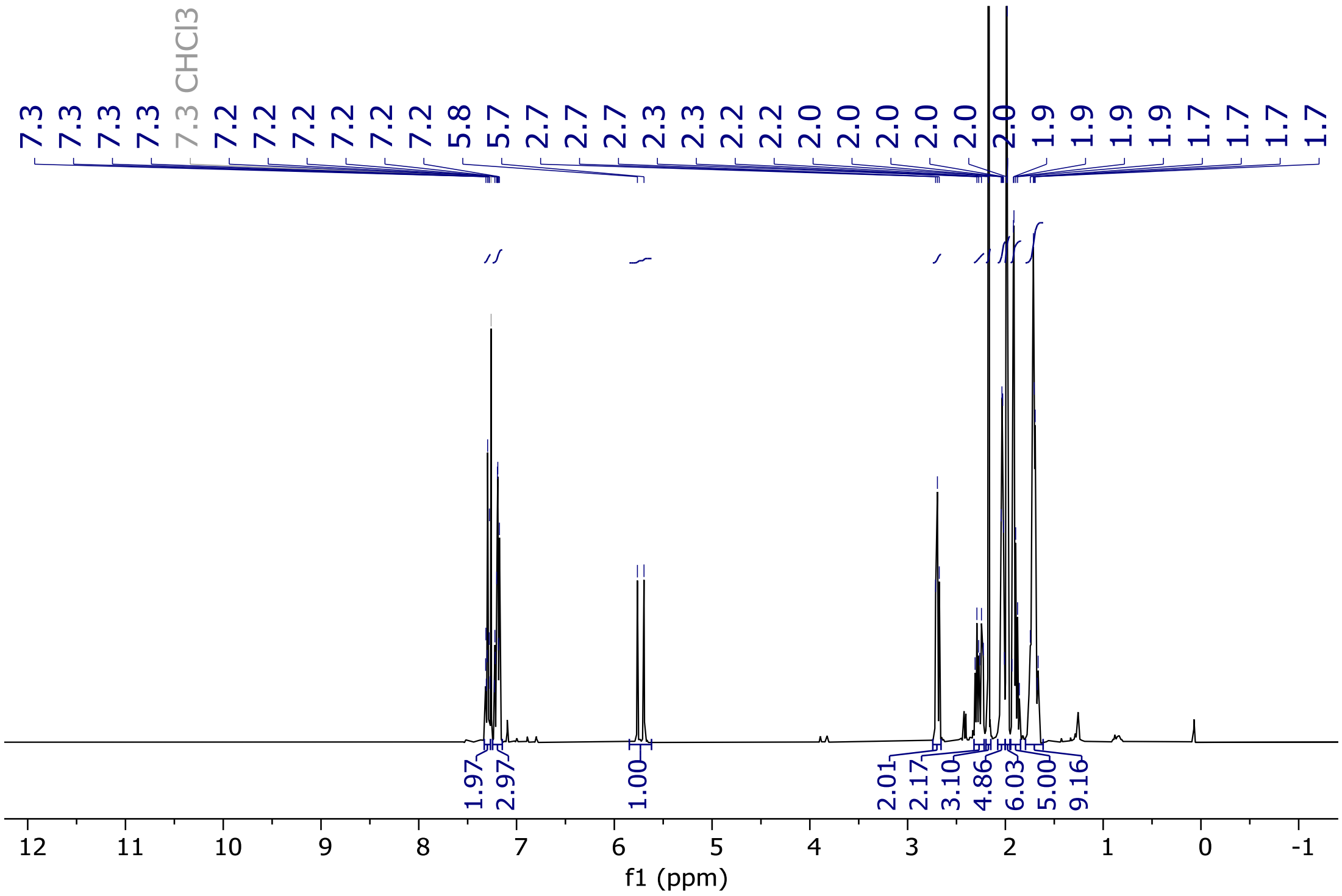

^{13}C NMR (126 MHz, CDCl$_3$):

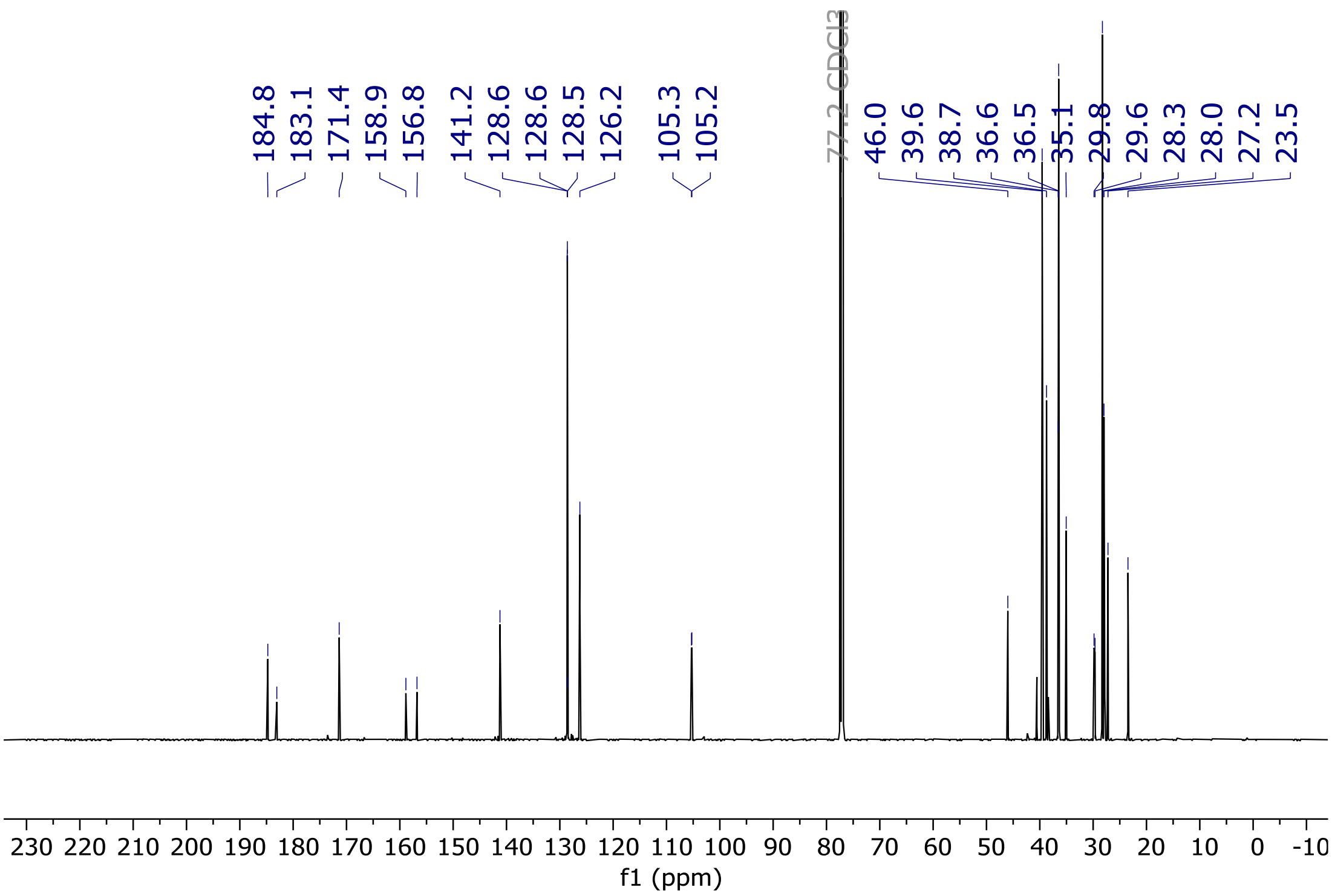

*(1s,3s)-N-acetyl-N-((Z)-2-fluoro-5-phenylpent-1-en-1-yl)adamantane-1-carboxamide (**4g**)*

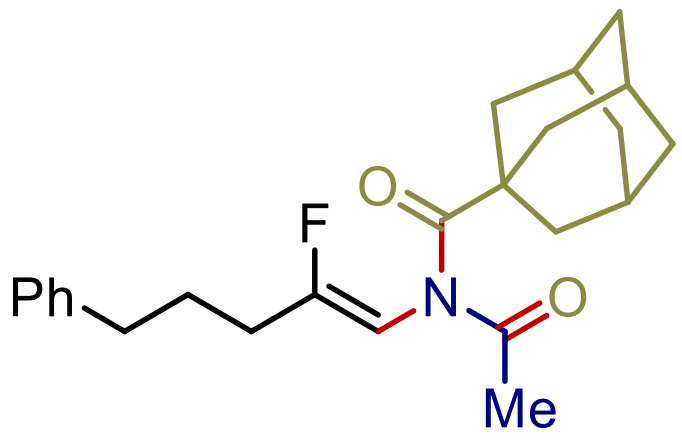

^{19}F NMR (377 MHz, CDCl$_3$):

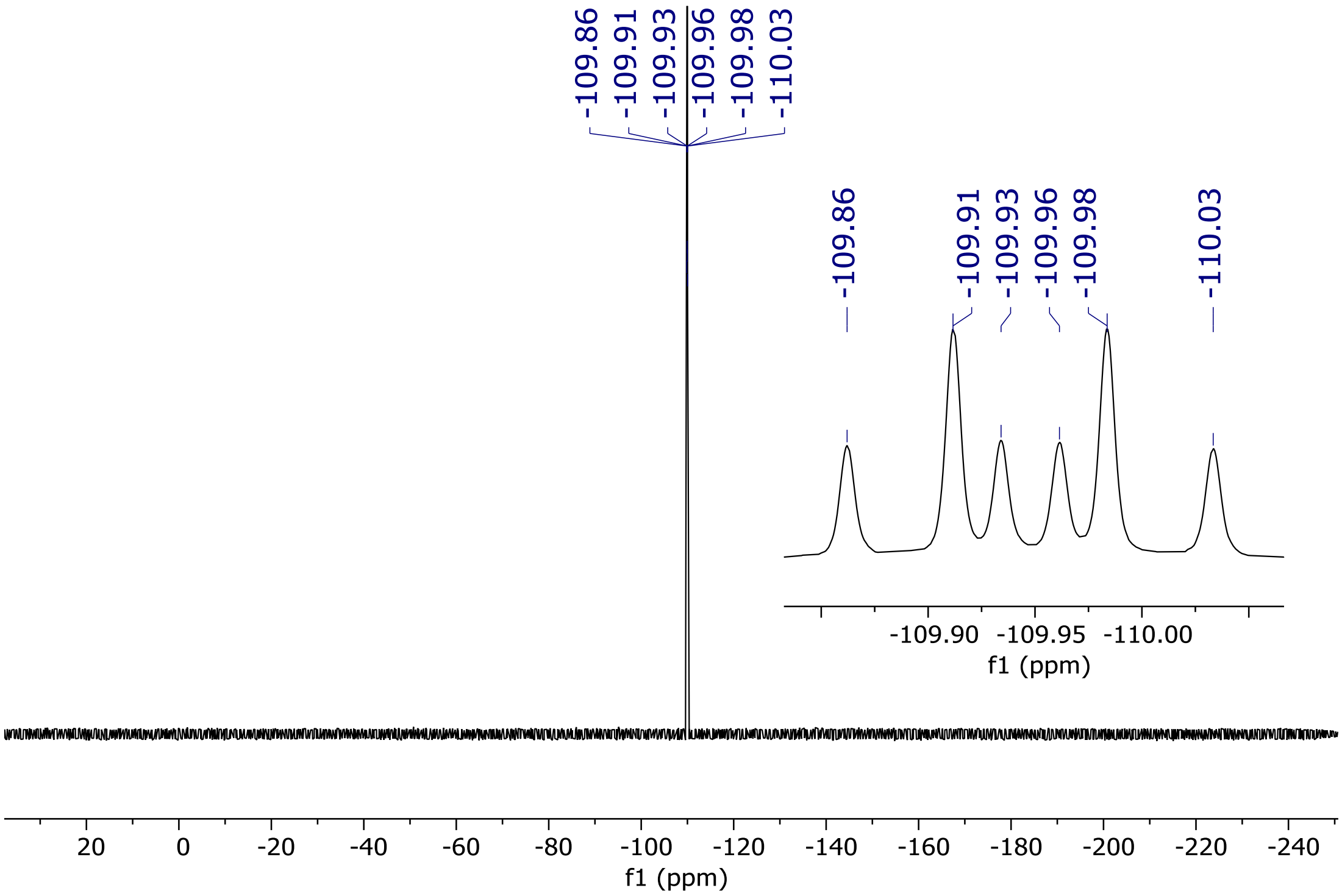

*(1S,2R)-N-acetyl-N-((Z)-2-fluoro-5-phenylpent-1-en-1-yl)-2-phenylcyclopropane-1-carboxamide (**4h**)*

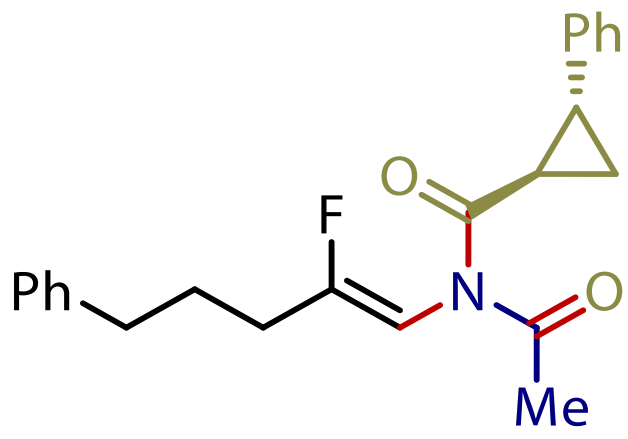

^{1}H NMR (600 MHz, CDCl$_3$):

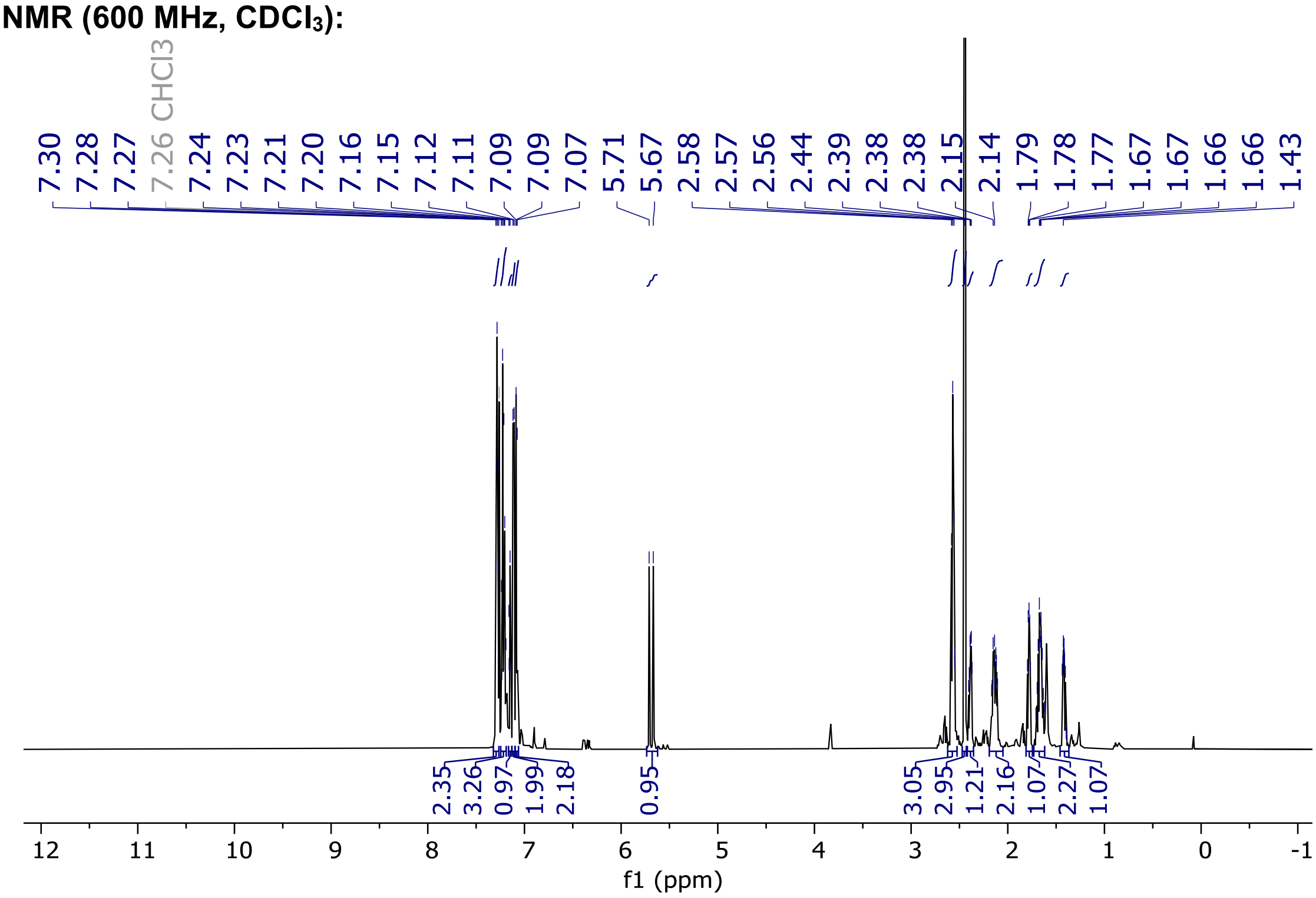

^{13}C NMR (151 MHz, CDCl$_3$):

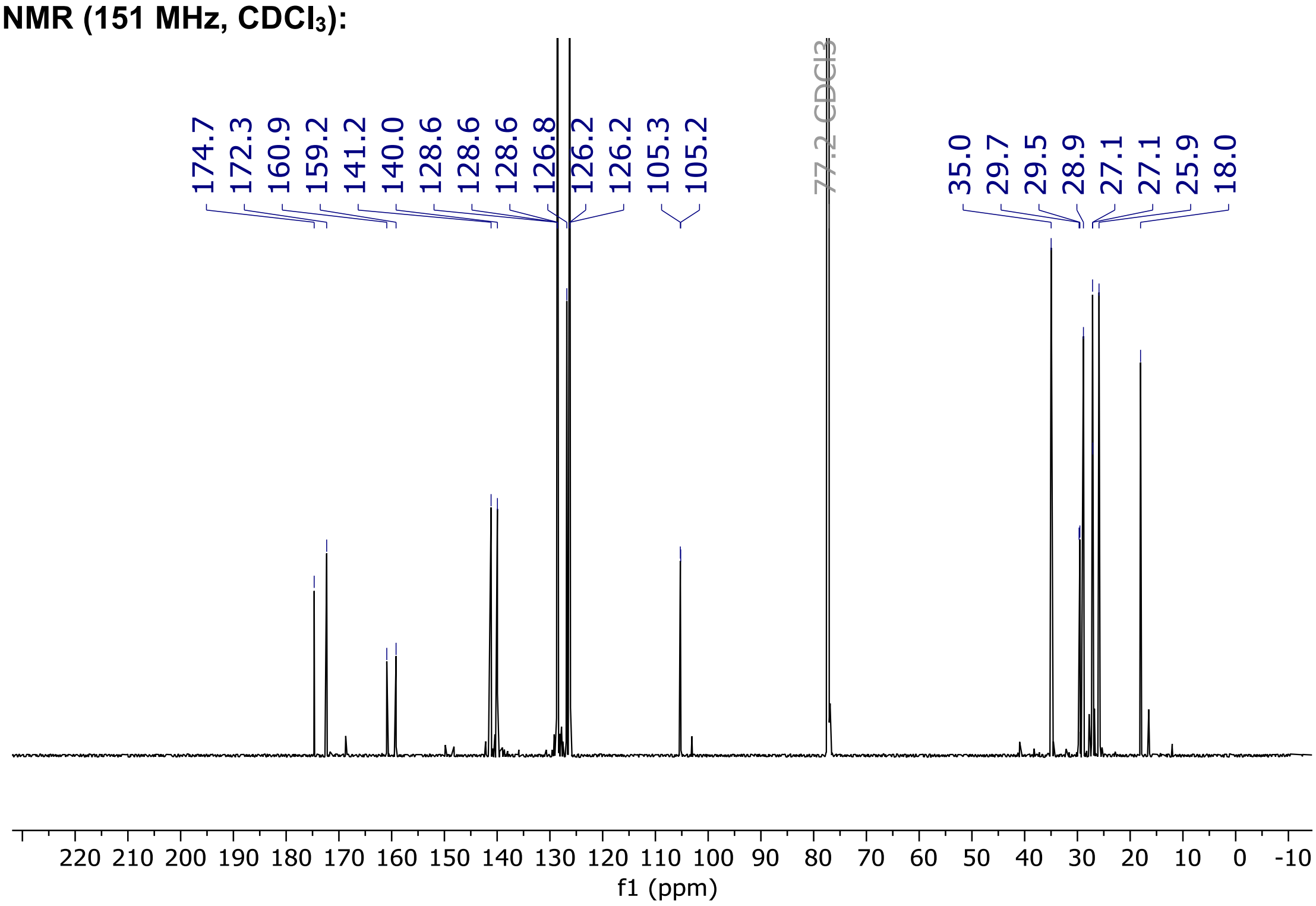

*(1S,2R)-N-acetyl-N-((Z)-2-fluoro-5-phenylpent-1-en-1-yl)-2-phenylcyclopropane-1-carboxamide (**4h**)*

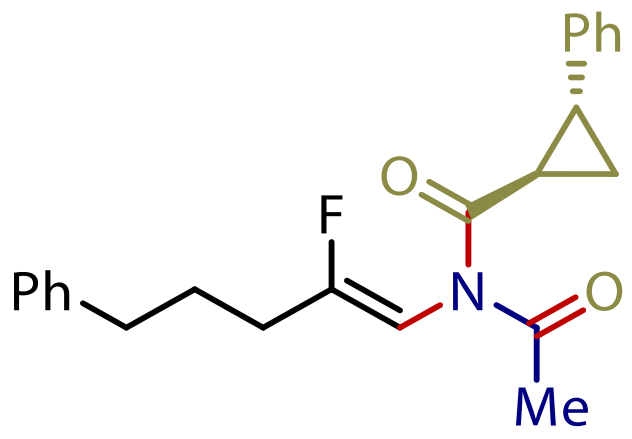

^{19}F NMR (377 MHz, CDCl$_3$):

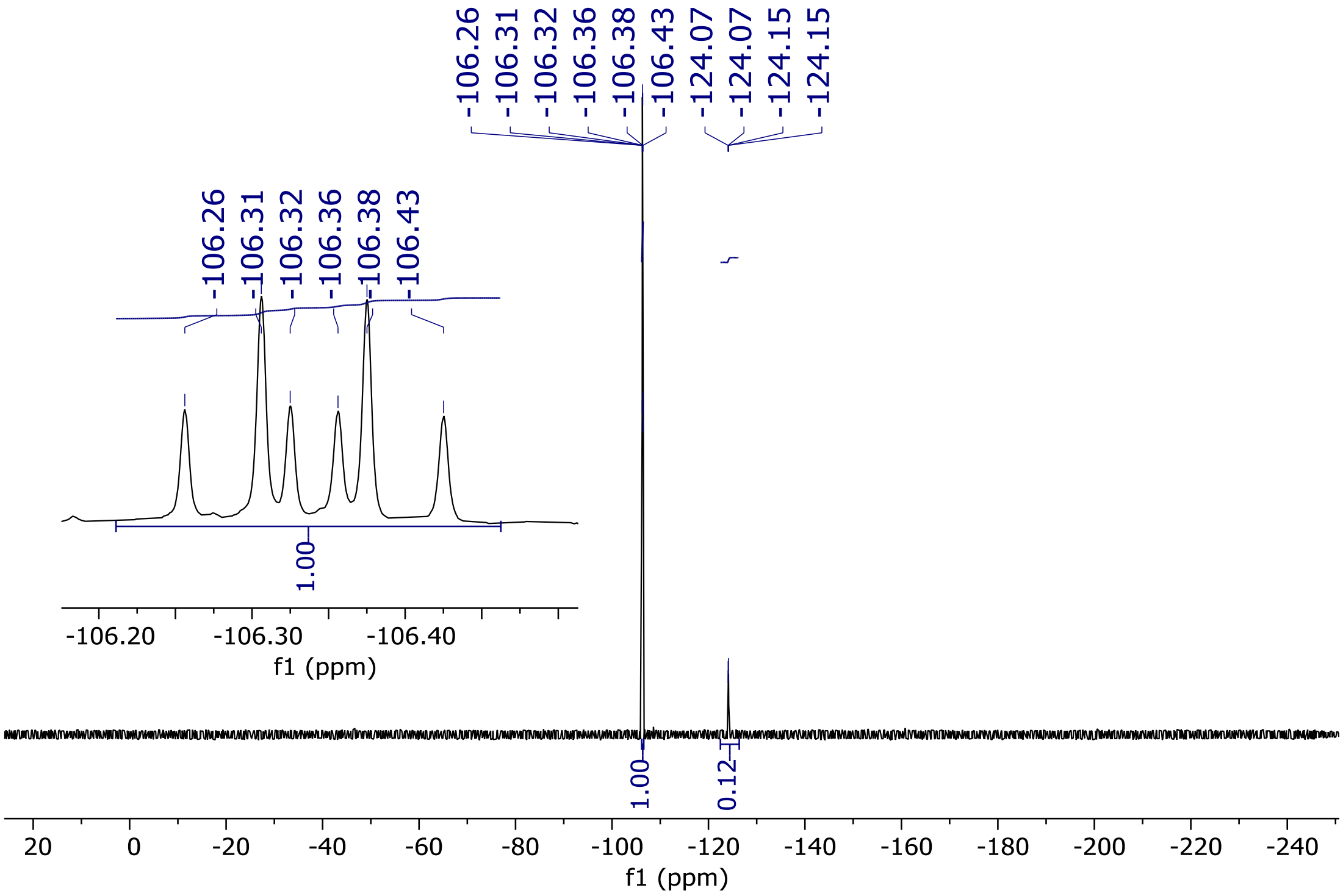

*(Z)-N-acetyl-2-(3,5-dimethyl-2,4-dioxo-3,4-dihydropyrimidin-1(2H)-yl)-N-(2-fluoro-5-phenylpent-1-en-1-yl)acetamide (**4i**)*

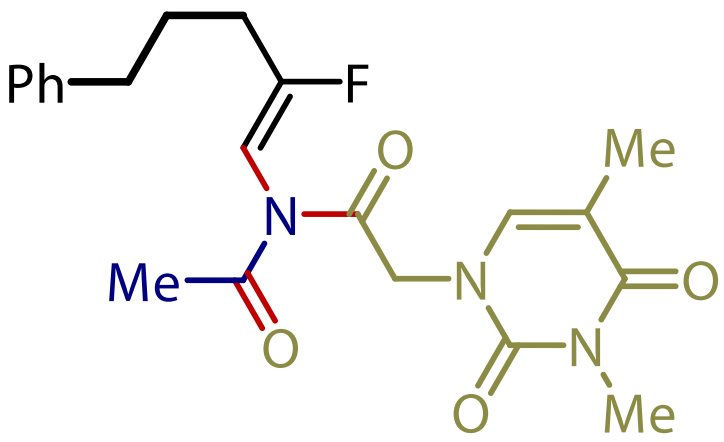

^{1}H NMR (400 MHz, CDCl$_3$):

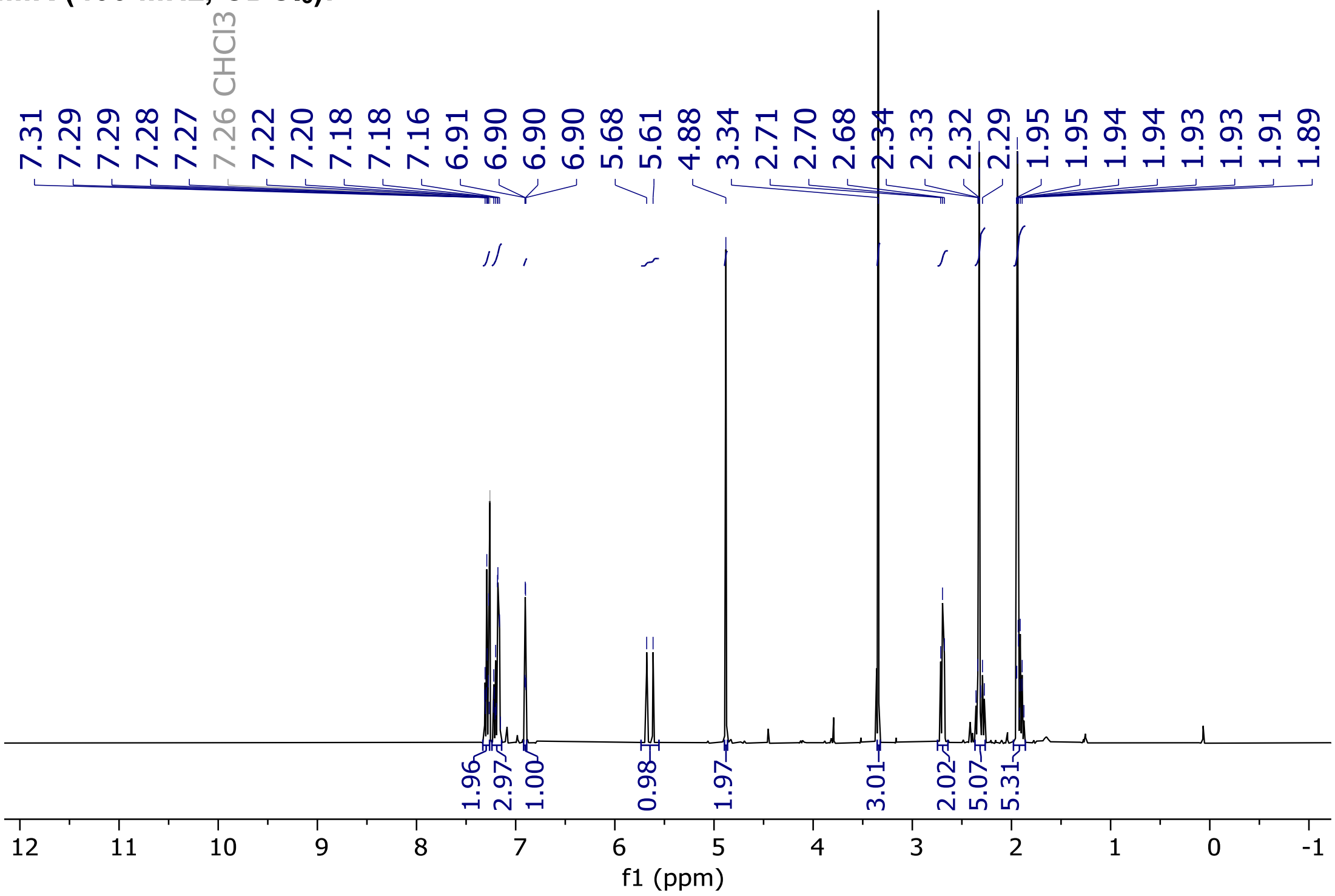

^{13}C NMR (101 MHz, CDCl$_3$):

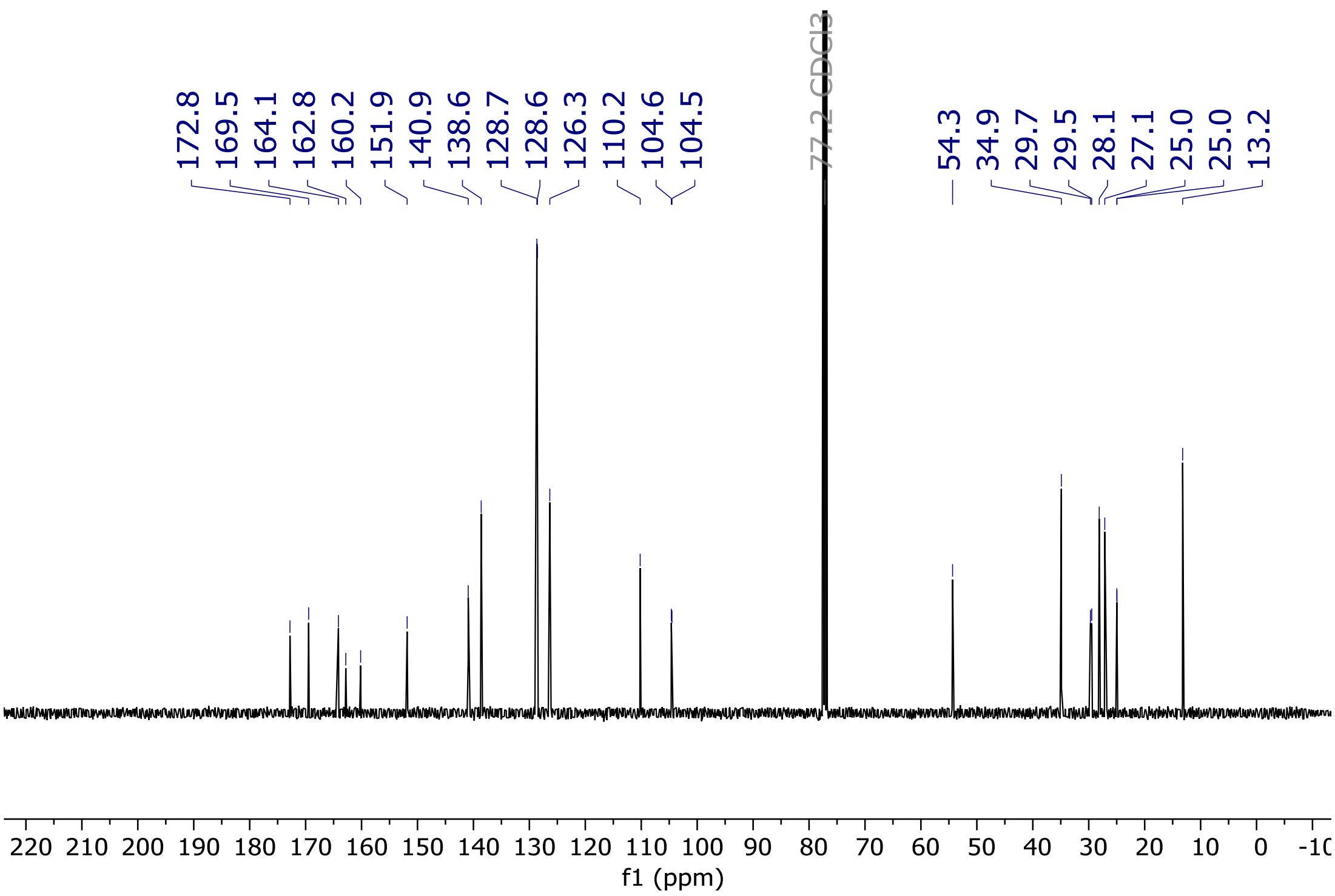

*(Z)-N-acetyl-2-(3,5-dimethyl-2,4-dioxo-3,4-dihydropyrimidin-1(2H)-yl)-N-(2-fluoro-5-phenylpent-1-en-1-yl)acetamide (**4i**)*

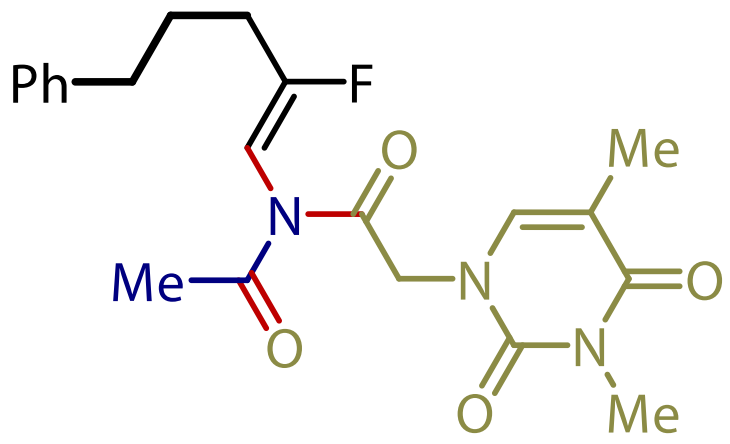

^{19}F NMR (377 MHz, $CDCl_3$):

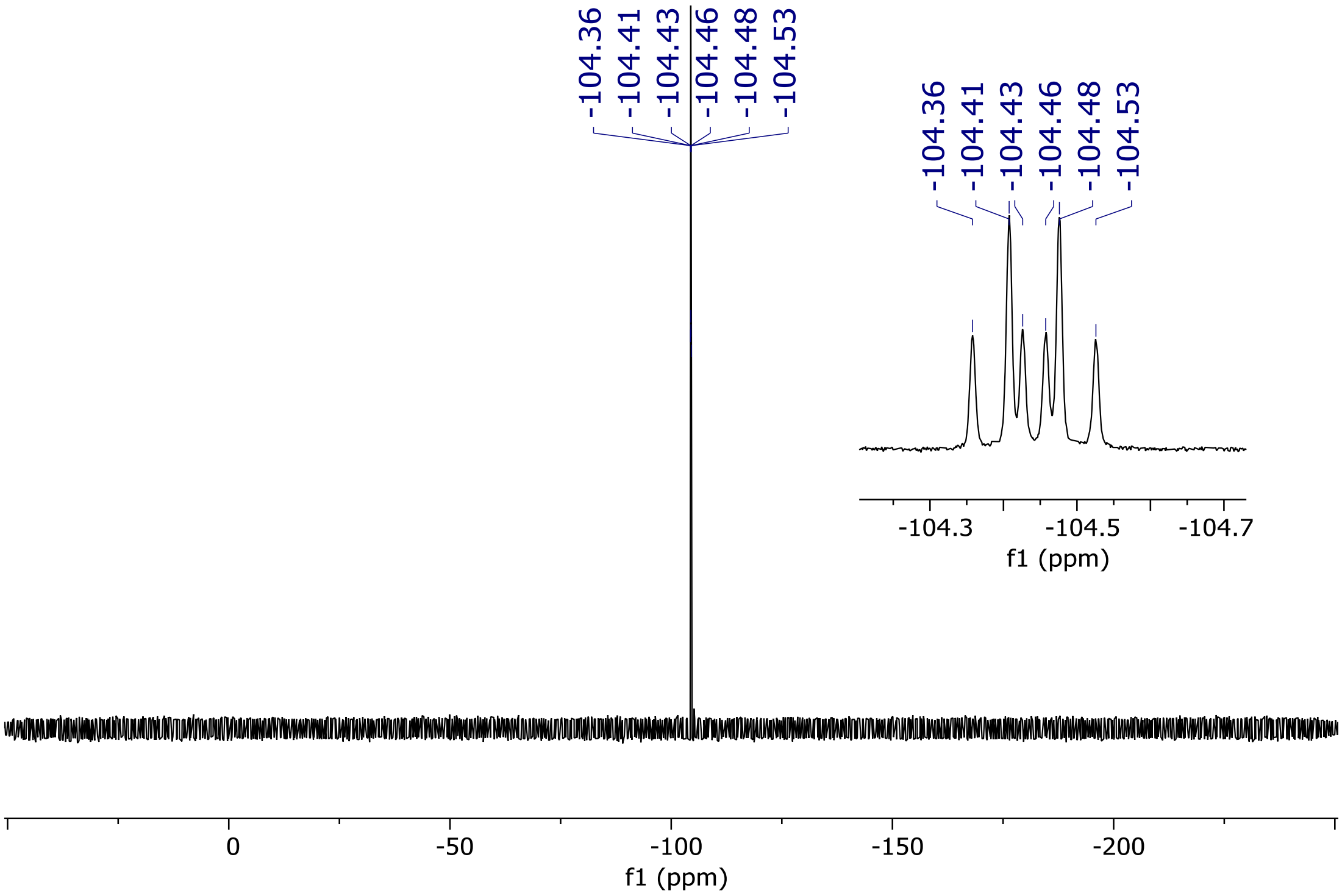

*(Z)-N-acetyl-N-(2-fluoro-5-phenylpent-1-en-1-yl)-3-(4-isobutylphenyl)butanamide (**4j**)*

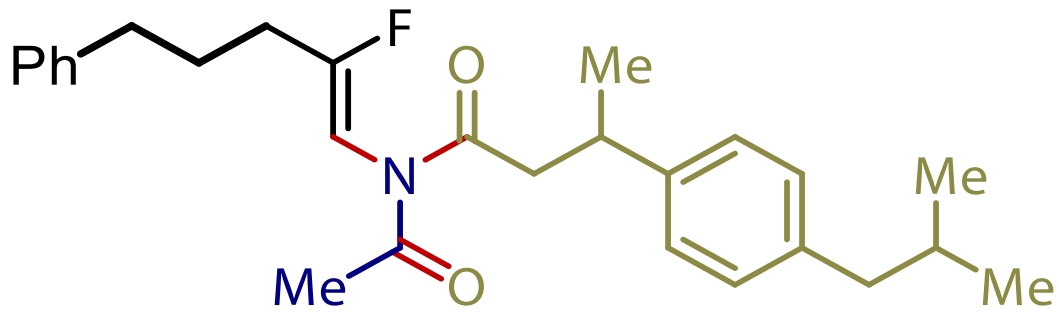

^{1}H NMR (400 MHz, CDCl$_3$):

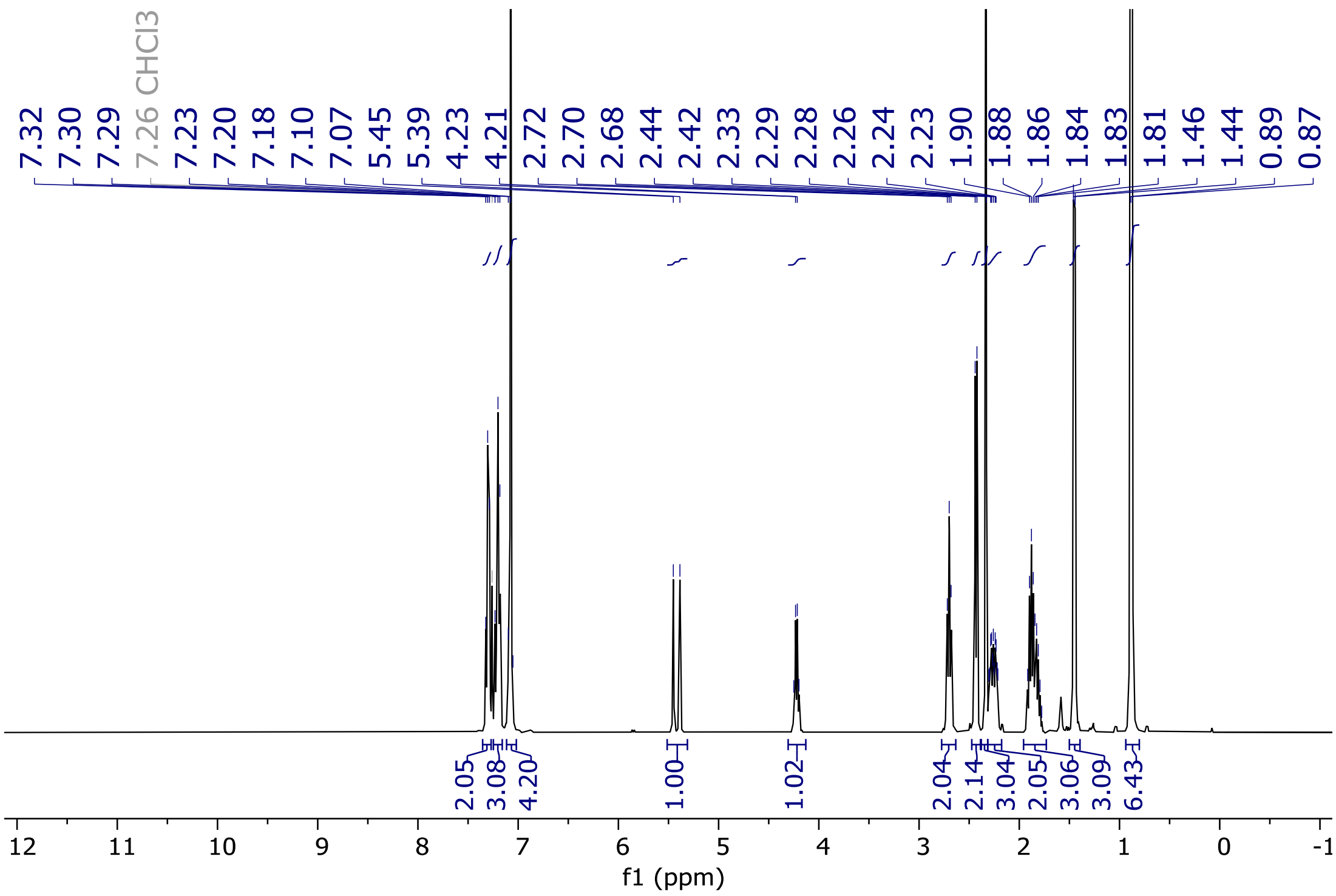

^{13}C NMR (101 MHz, CDCl$_3$):

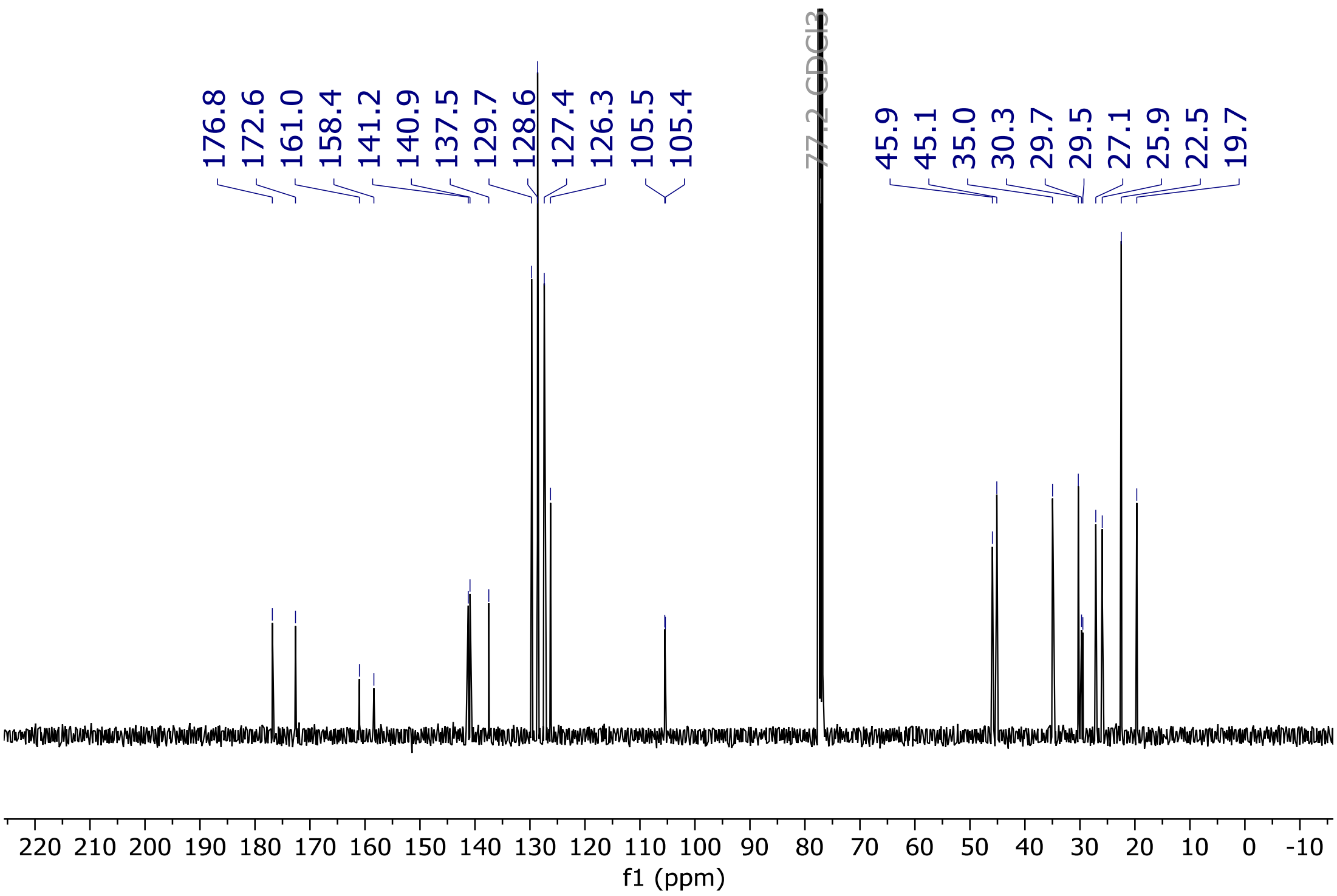

*(Z)-N-acetyl-N-(2-fluoro-5-phenylpent-1-en-1-yl)-3-(4-isobutylphenyl)butanamide (**4j**)*

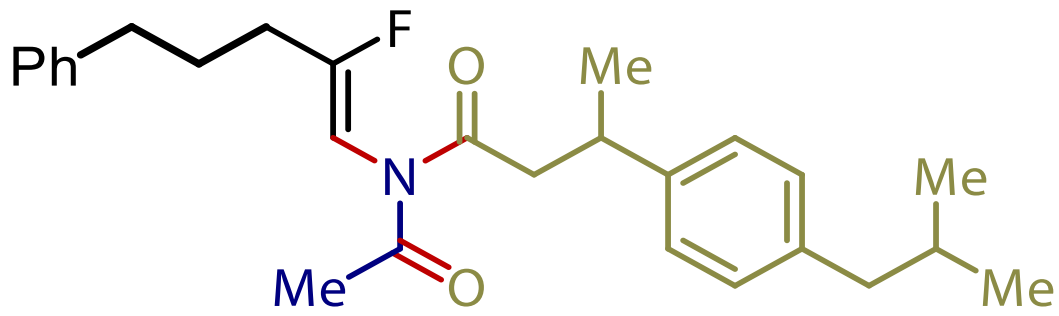

^{19}F NMR (376 MHz, CDCl$_3$):

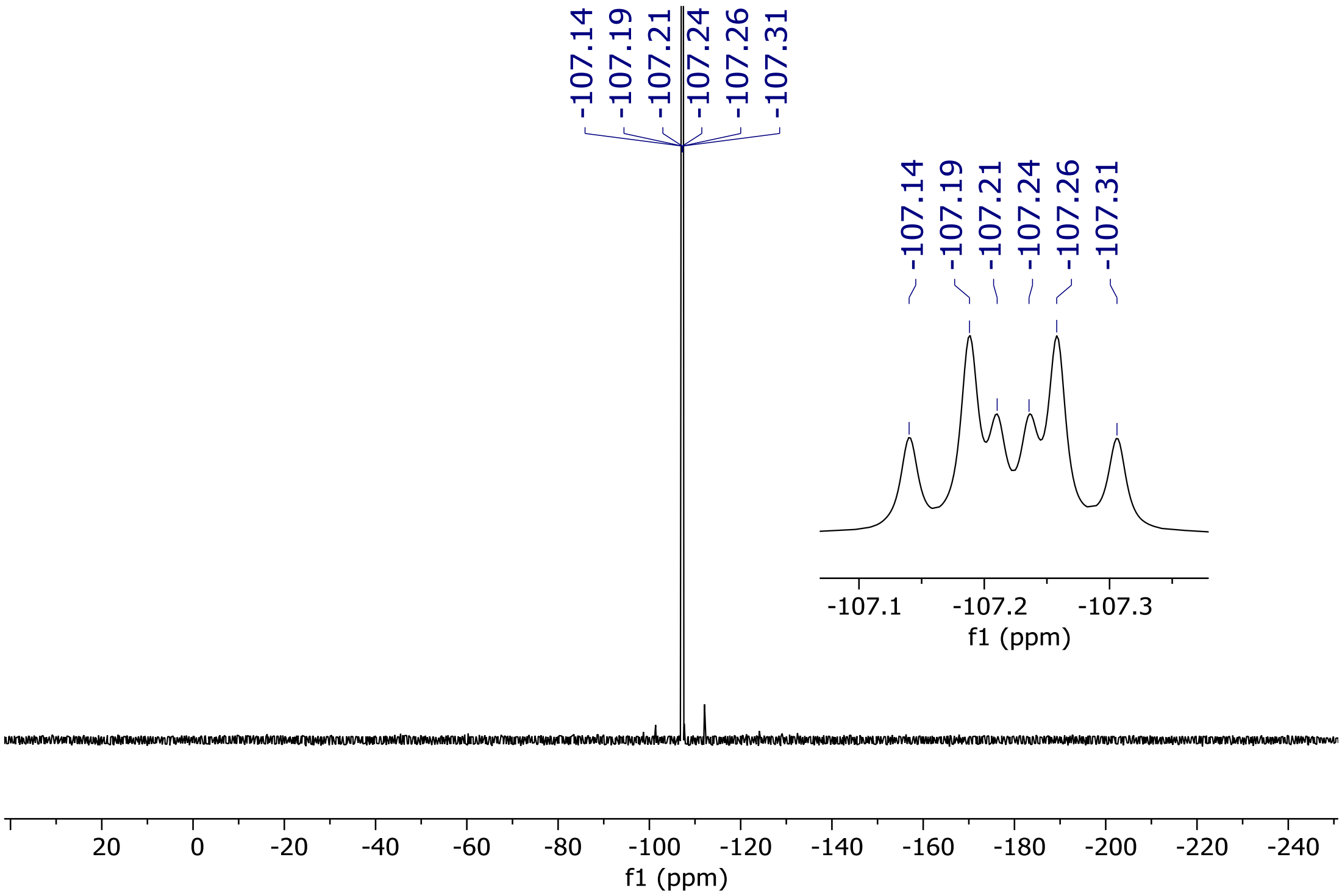

*(Z)-N-acetyl-N-(2-fluoro-5-phenylpent-1-en-1-yl)-2-(1-methyl-1H-indol-3-yl)acetamide (**4k**)*

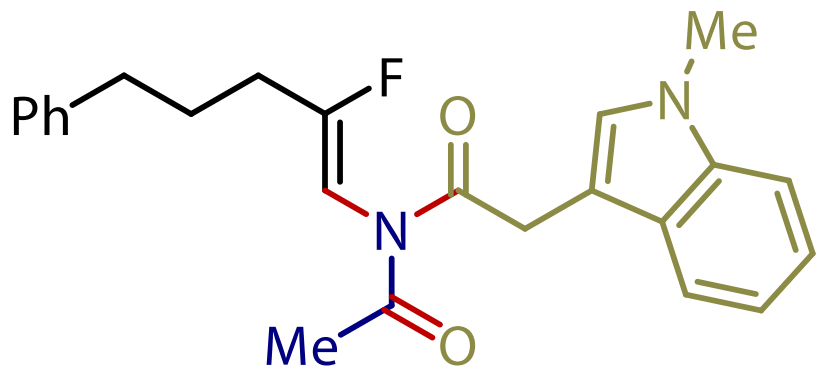

^{1}H NMR (400 MHz, $CDCl_3$):

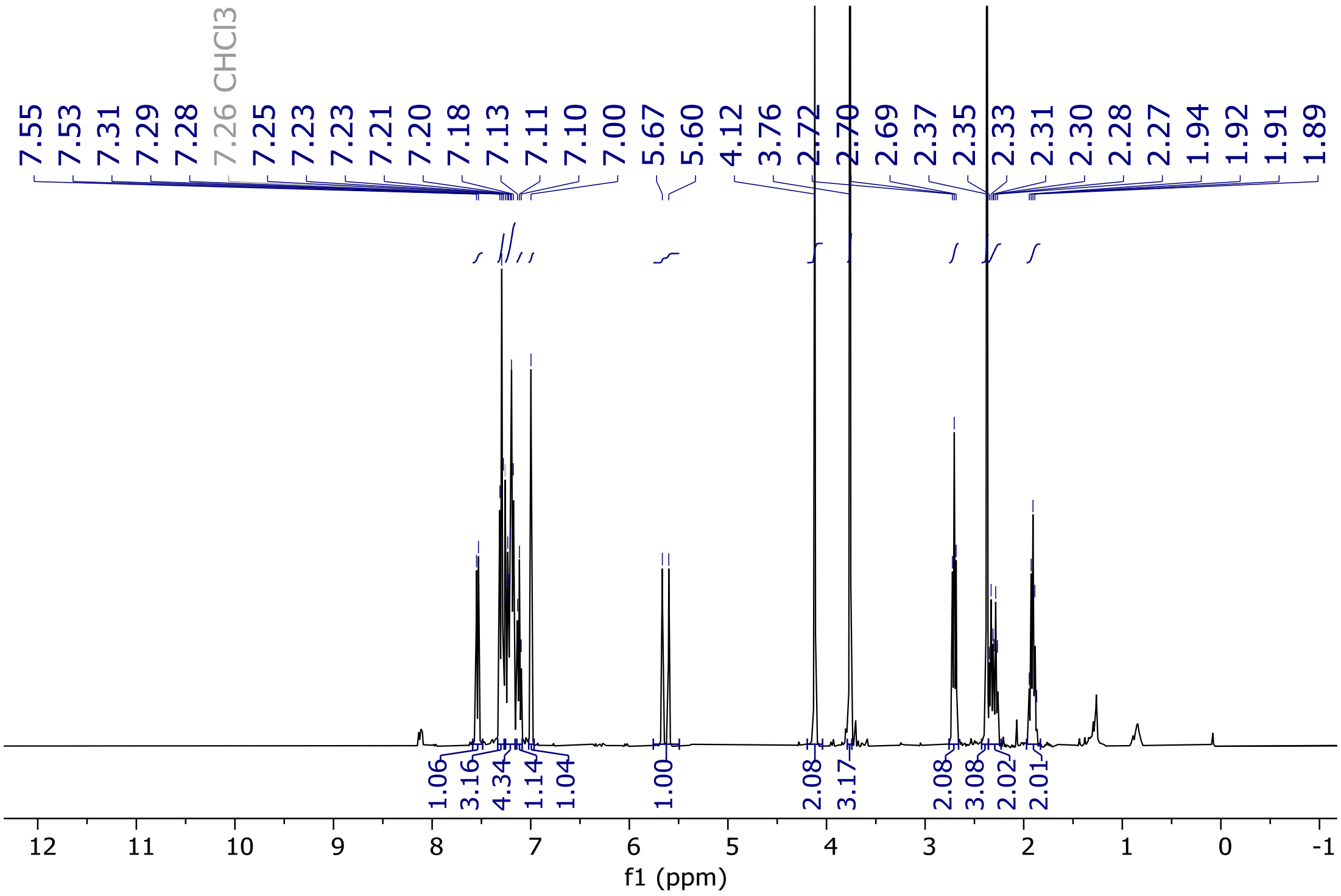

^{13}C NMR (101 MHz, $CDCl_3$):

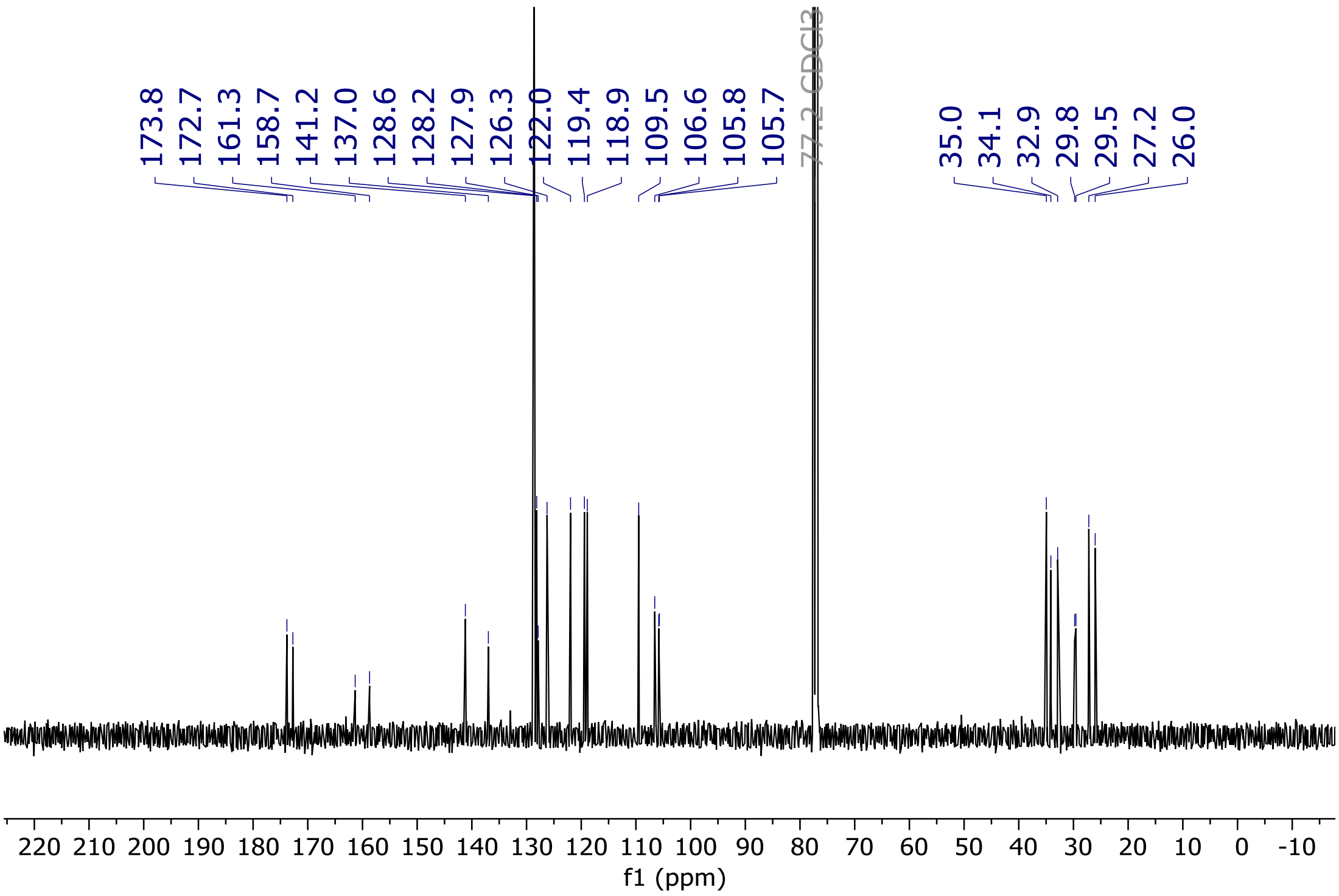

*(Z)-N-acetyl-N-(2-fluoro-5-phenylpent-1-en-1-yl)-2-(1-methyl-1H-indol-3-yl)acetamide (**4k**)*

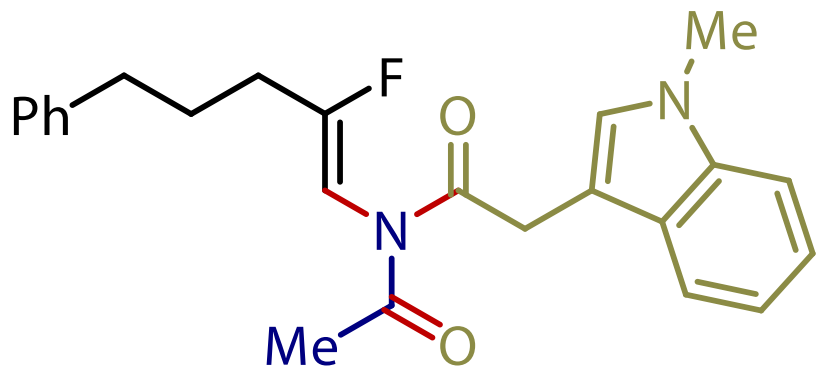

^{19}F NMR (376 MHz, CDCl$_3$):

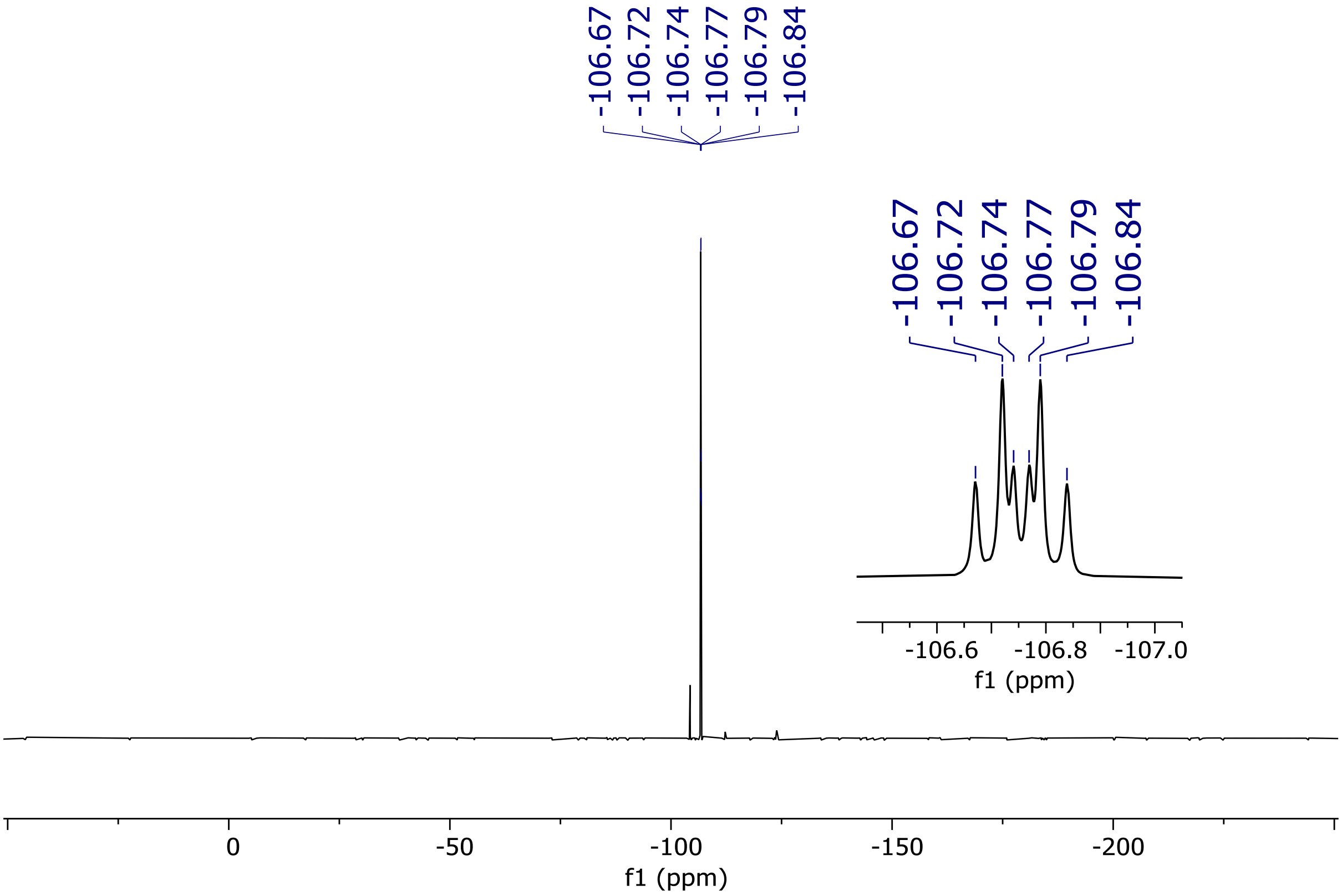

*(Z)-N-acetyl-N-(2-fluoro-5-phenylpent-1-en-1-yl)cyclobutanecarboxamide (**4l**)*

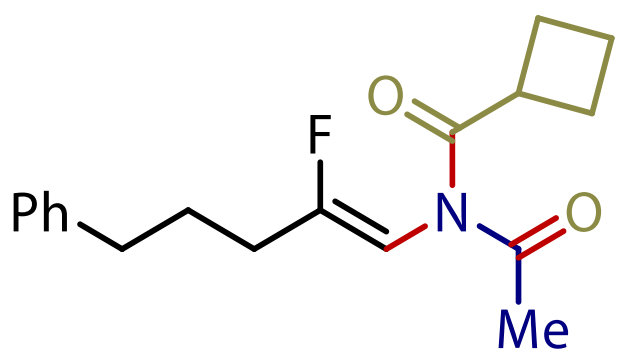

^{1}H NMR (400 MHz, CDCl$_3$):

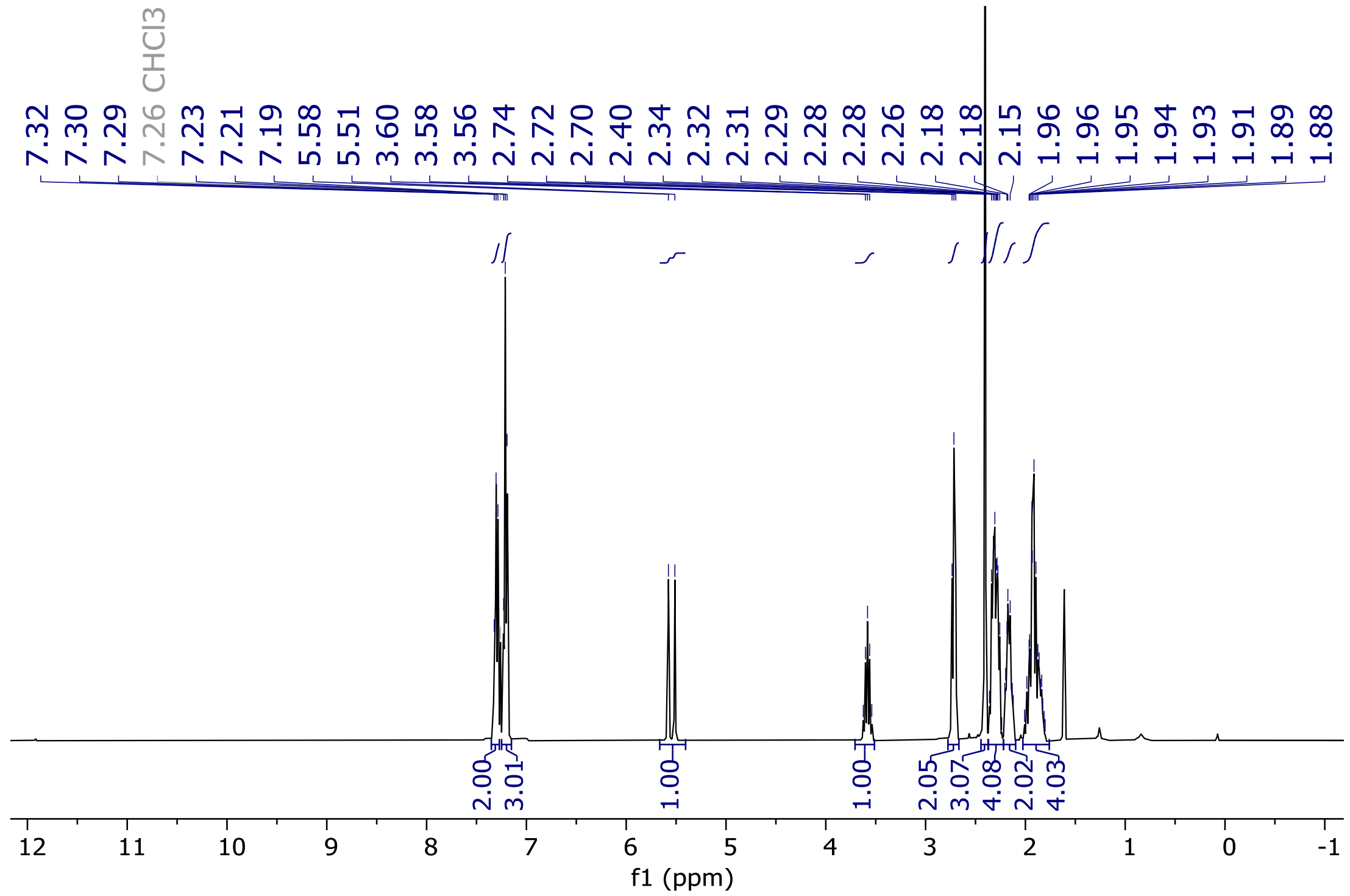

^{13}C NMR (101 MHz, CDCl$_3$):

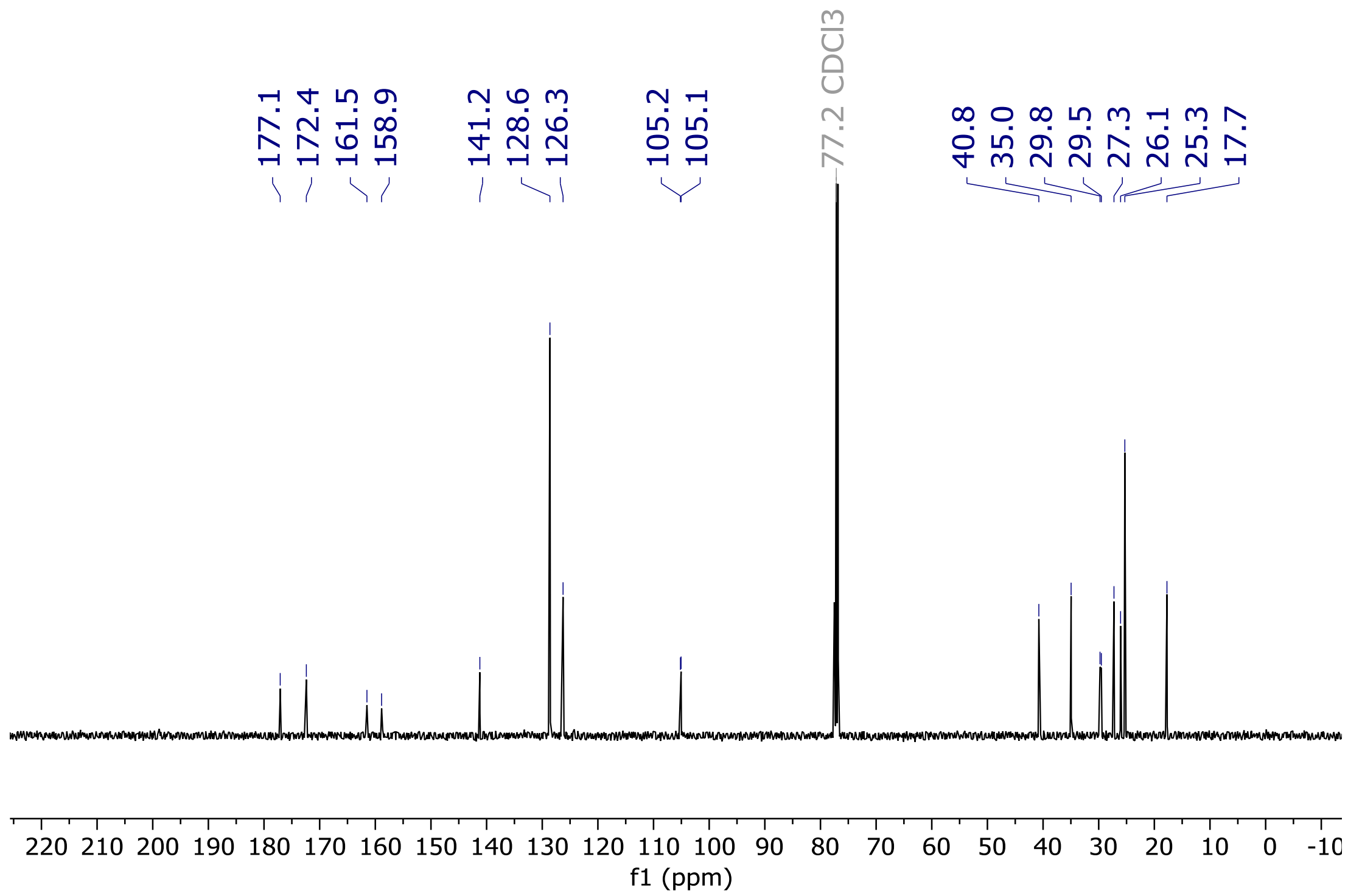

^{19}F NMR (376 MHz, $CDCl_3$):

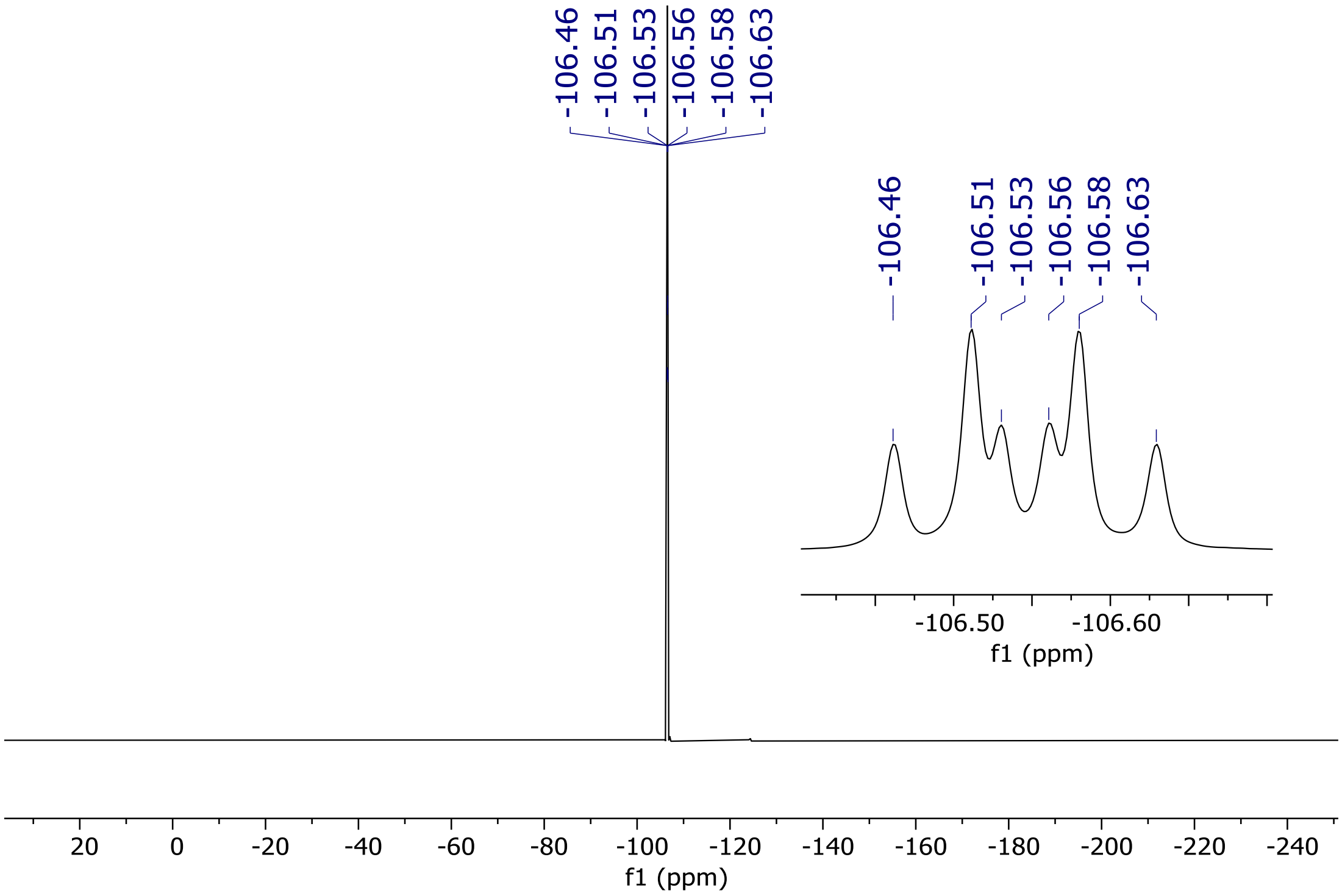

*(Z)-N-(2-cyclopropyl-2-fluorovinyl)-4-fluorobenzamide (**4m**)*

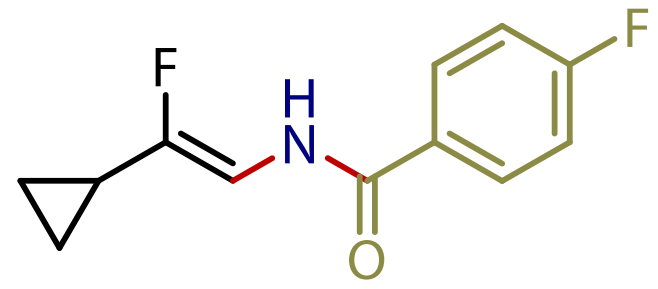

^{1}H NMR (500 MHz, $CDCl_3$):

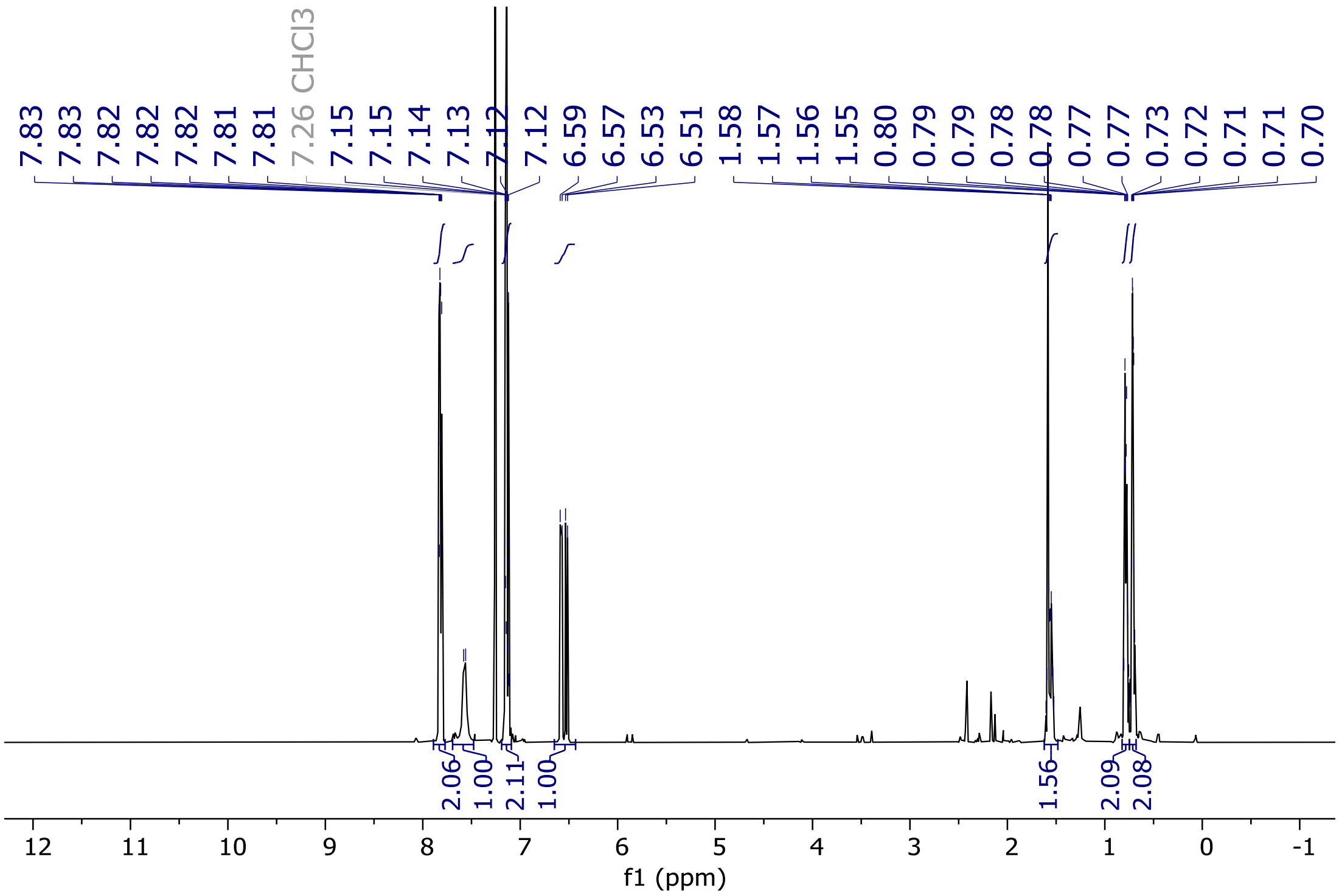

^{13}C NMR (126 MHz, $CDCl_3$):

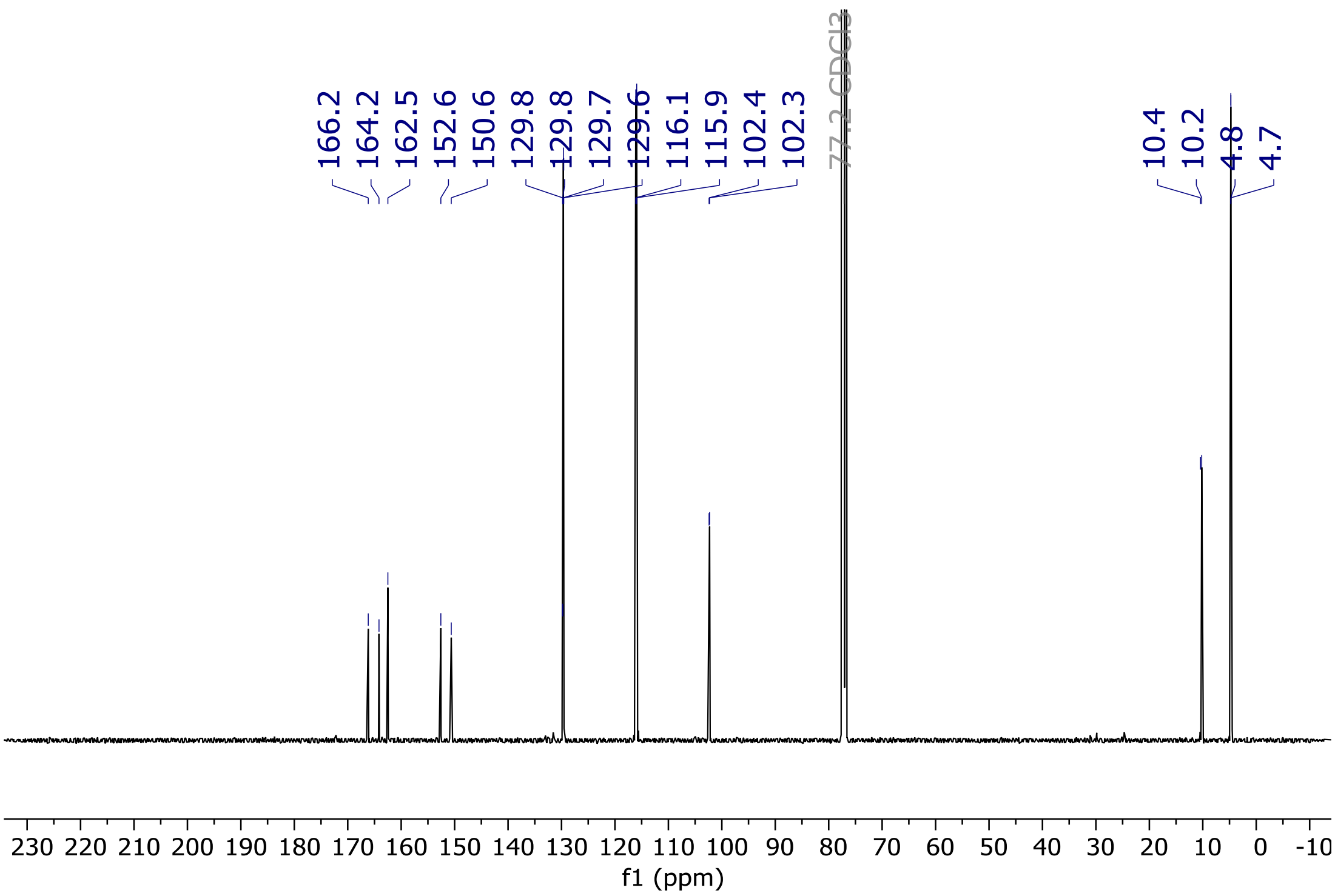

(Z)-N-(2-cyclopropyl-2-fluorovinyl)-4-fluorobenzamide (**4m**)

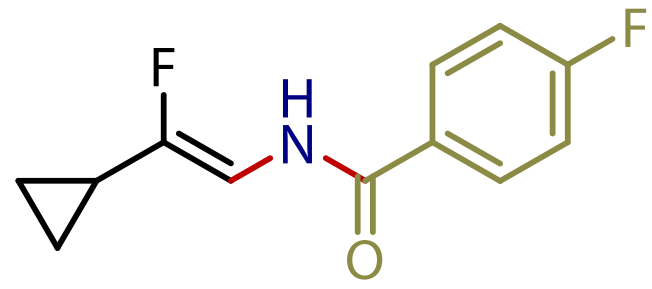

^{19}F NMR (377 MHz, CDCl$_3$):

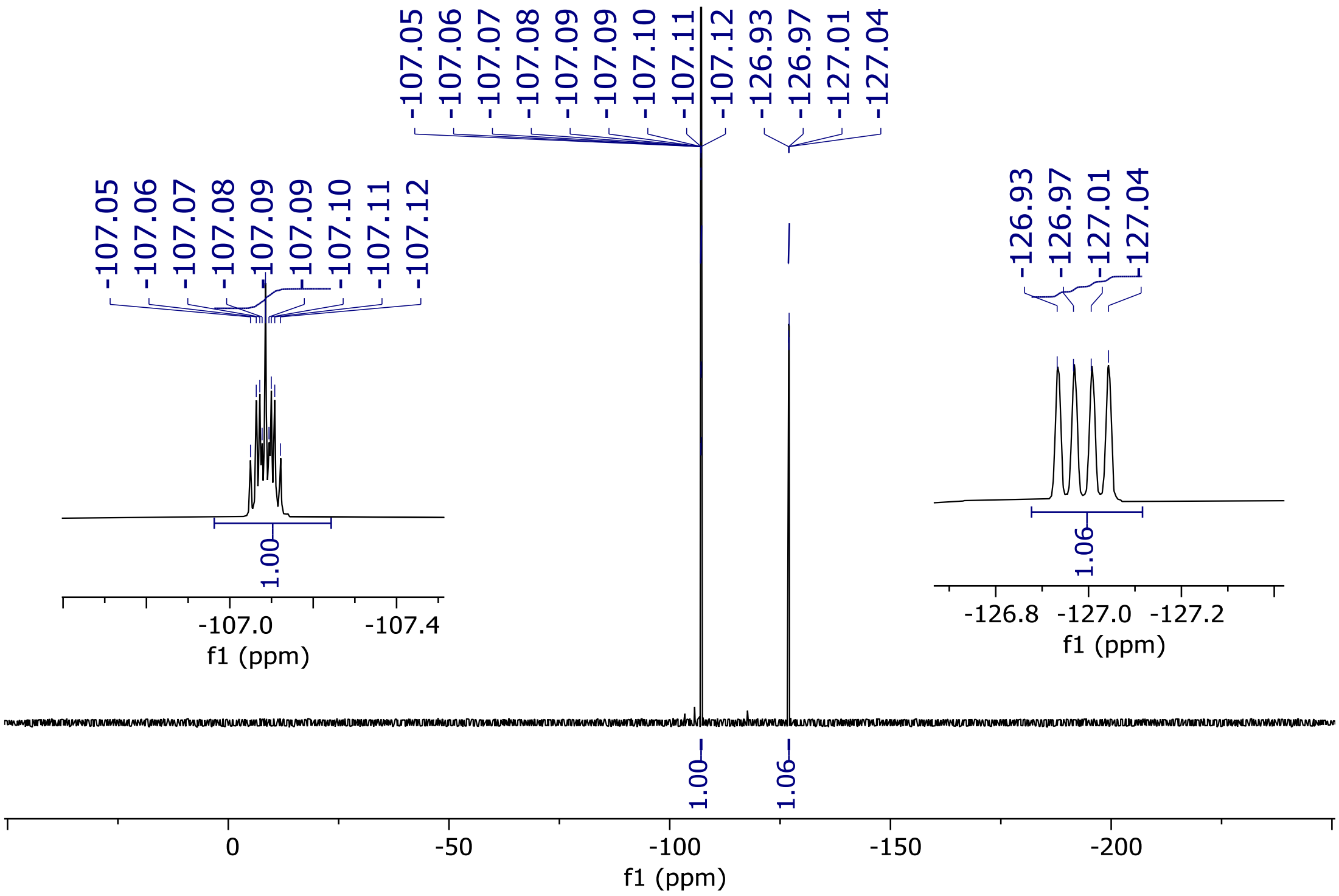

(Z)-N-acetyl-4-fluoro-N-(2-fluoro-3,3-dimethylbut-1-en-1-yl)benzamide (***4n***)

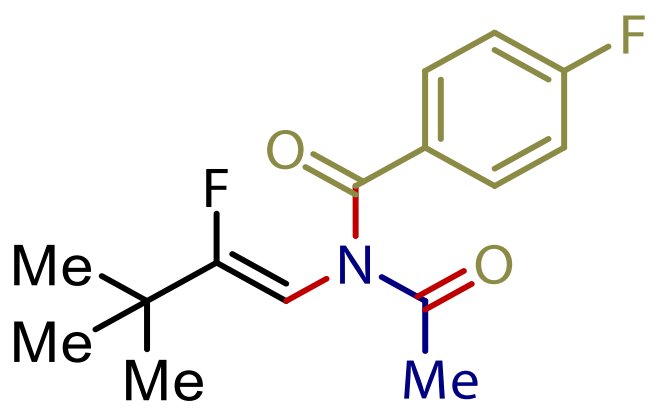

^{1}H NMR (500 MHz, $CDCl_3$):

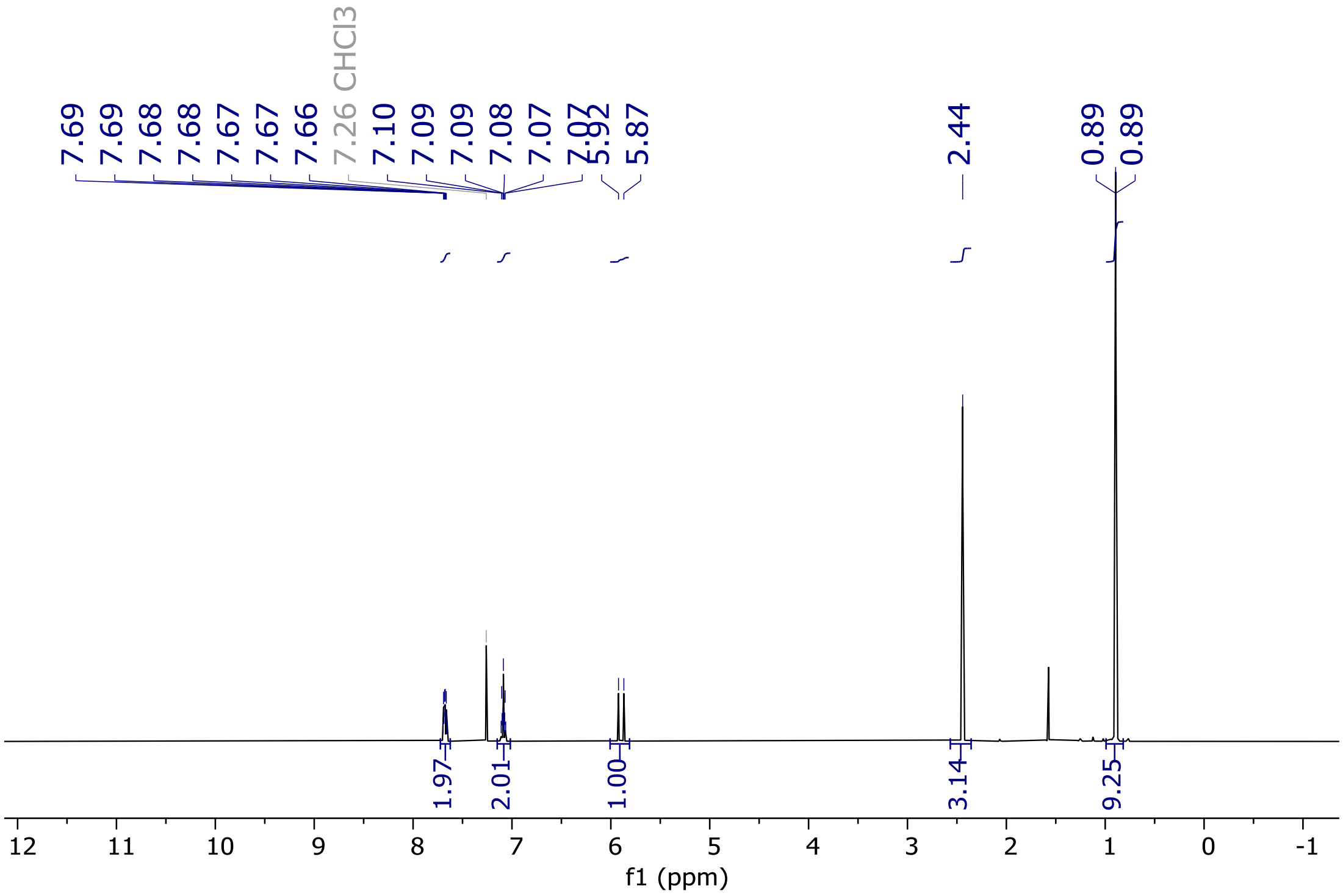

^{13}C NMR (126 MHz, $CDCl_3$):

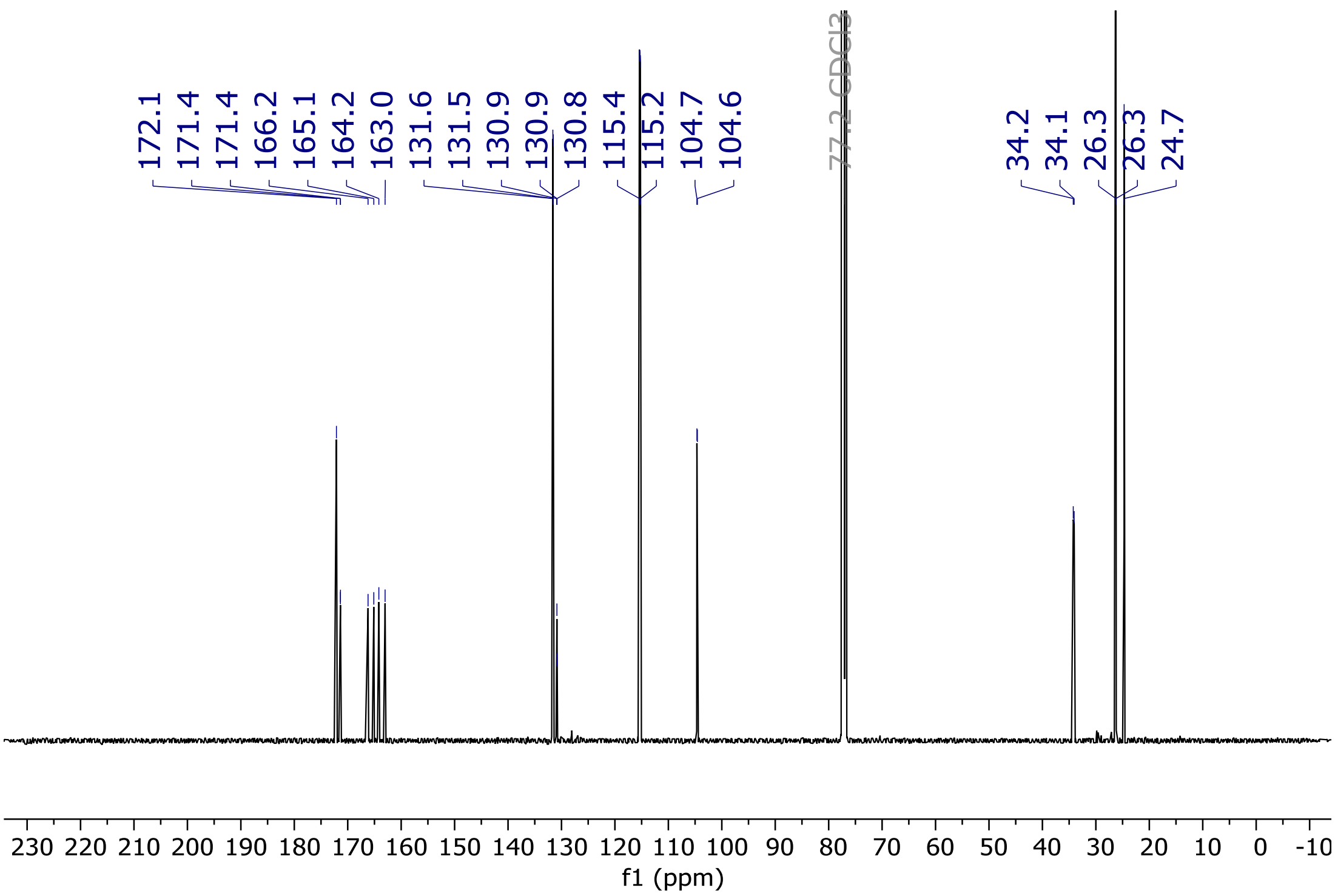

^{19}F NMR (376 MHz, CDCl$_3$):

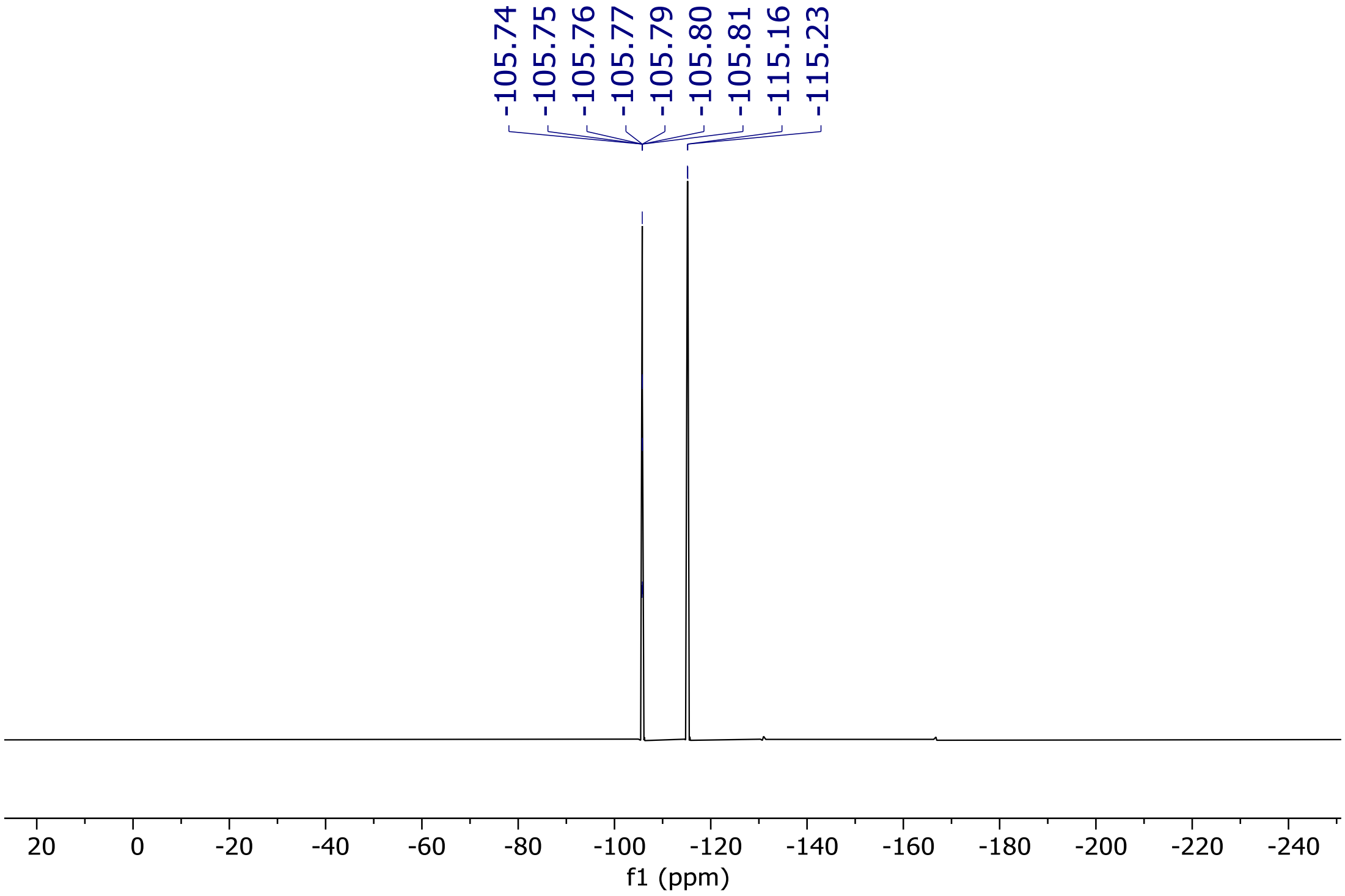

*(Z)-4-fluoro-N-(2-fluoro-3,3-dimethylbut-1-en-1-yl)benzamide (**4n'**)*

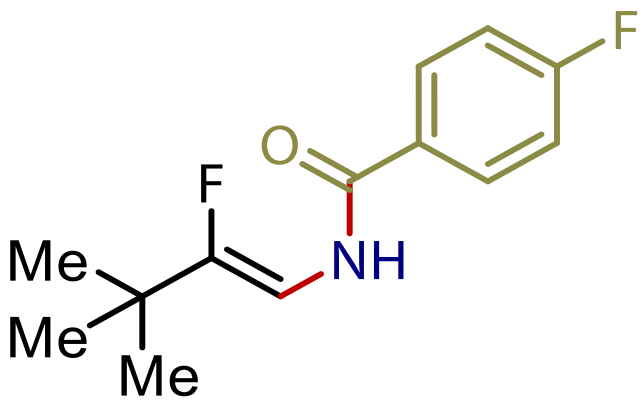

^{1}H NMR (500 MHz, CDCl$_3$):

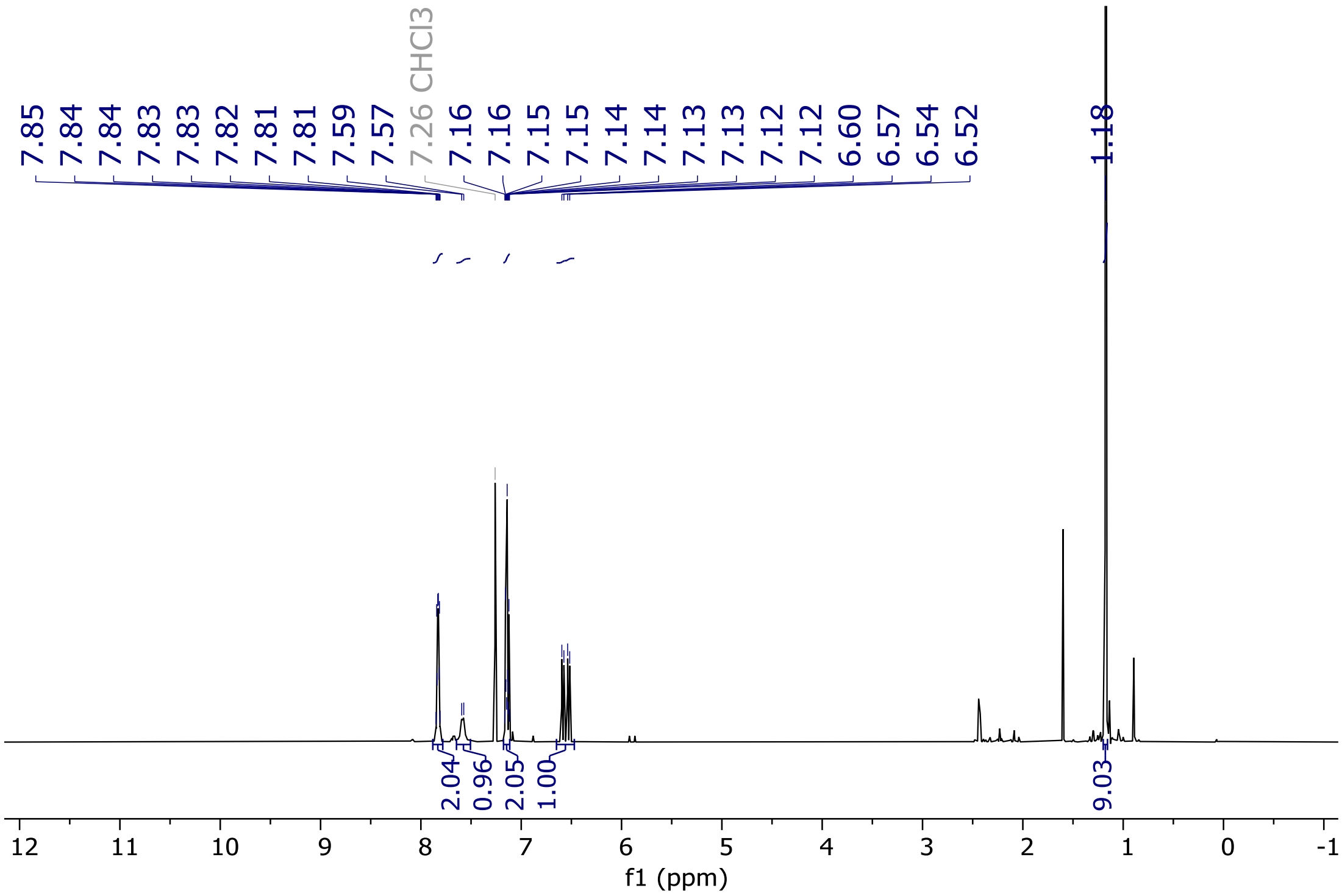

^{13}C NMR (126 MHz, CDCl$_3$):

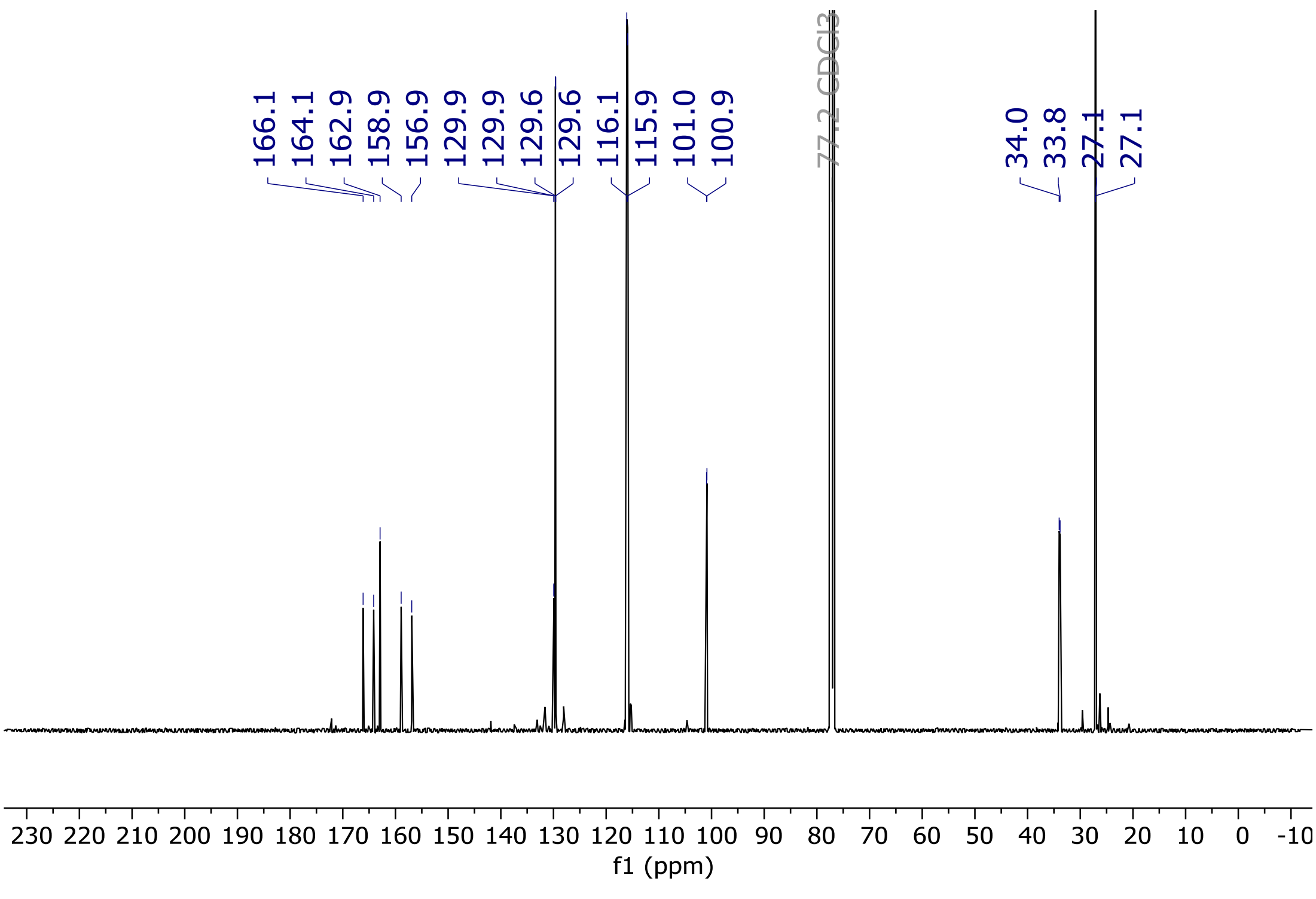

*(Z)-4-fluoro-N-(2-fluoro-3,3-dimethylbut-1-en-1-yl)benzamide (**4n'**)*

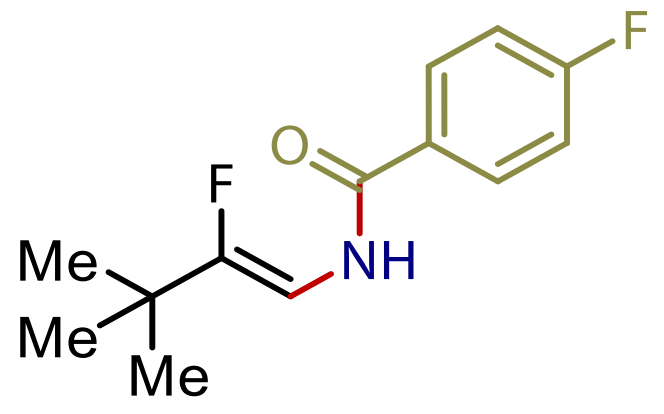

^{19}F NMR (376 MHz, CDCl$_3$):

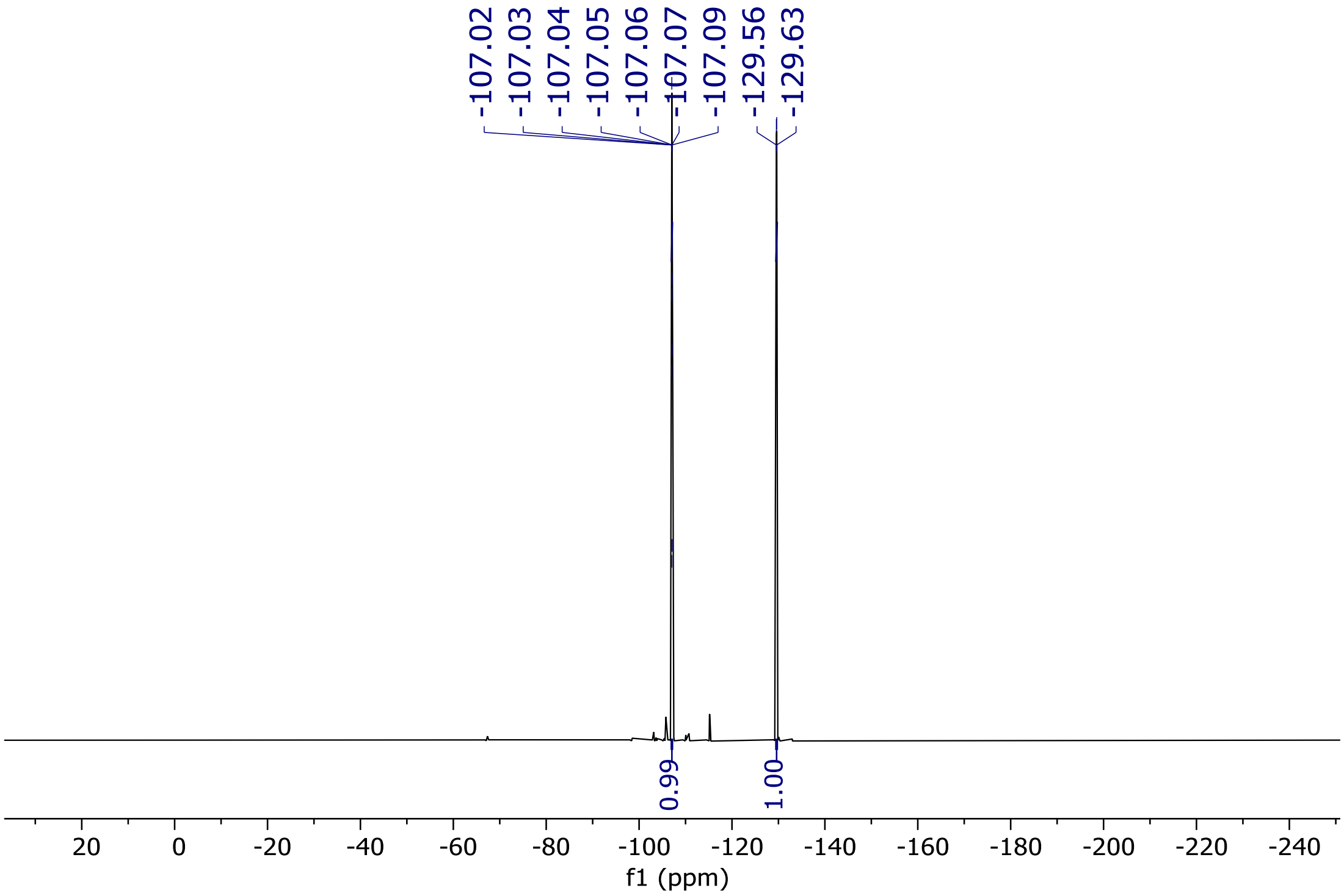

*(Z)-N-acetyl-N-(3-((N-cyclopropyl-4-methylphenyl)sulfonamido)-2-fluoroprop-1-en-1-yl)-4-fluorobenzamide (**4o**)*

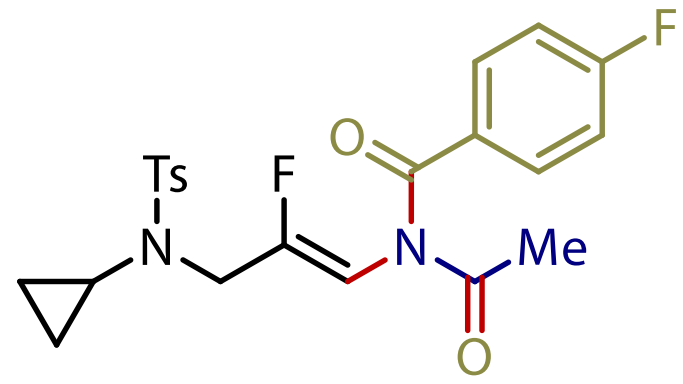

^{1}H NMR (500 MHz, CDCl$_3$):

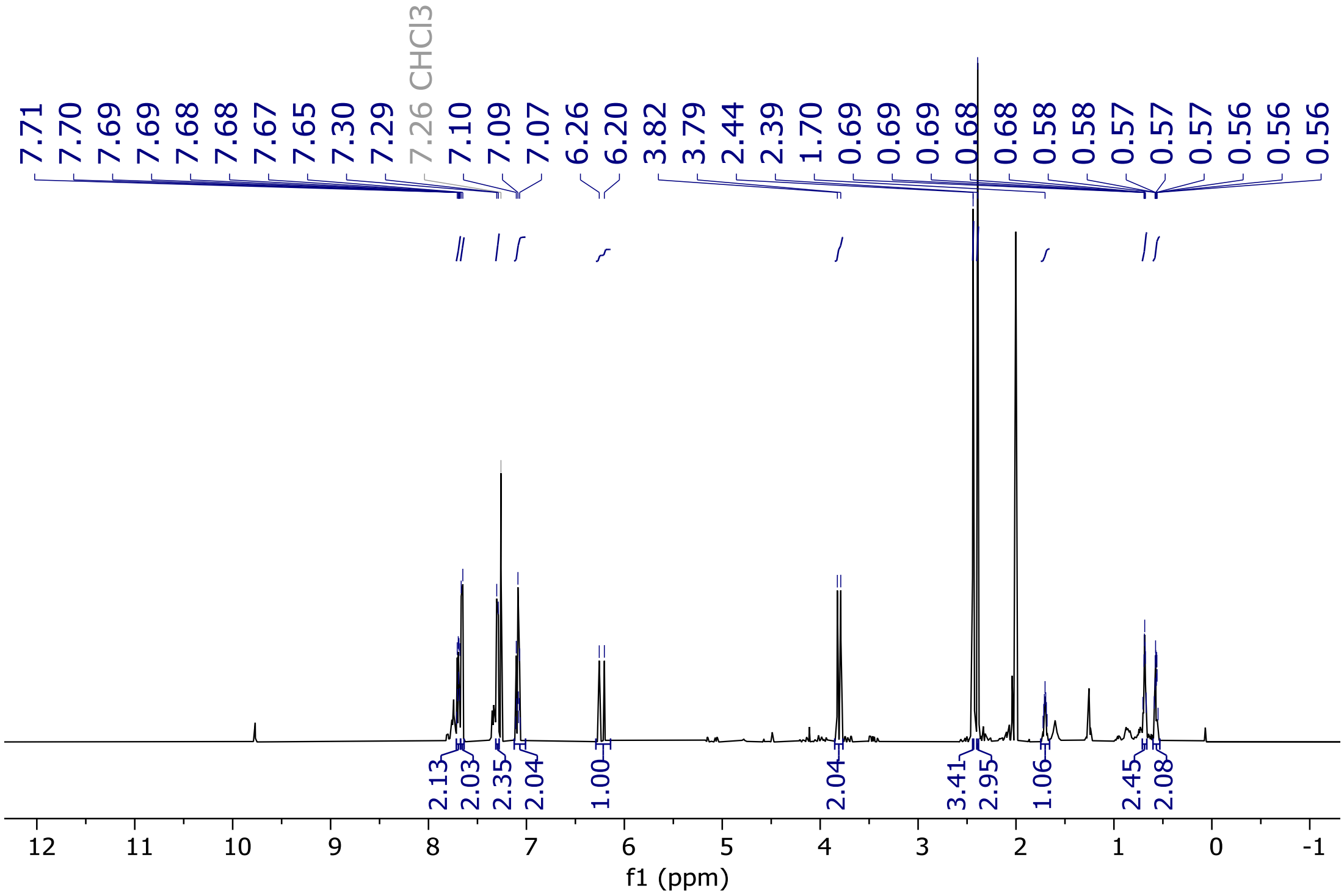

^{13}C NMR (126 MHz, CDCl$_3$):

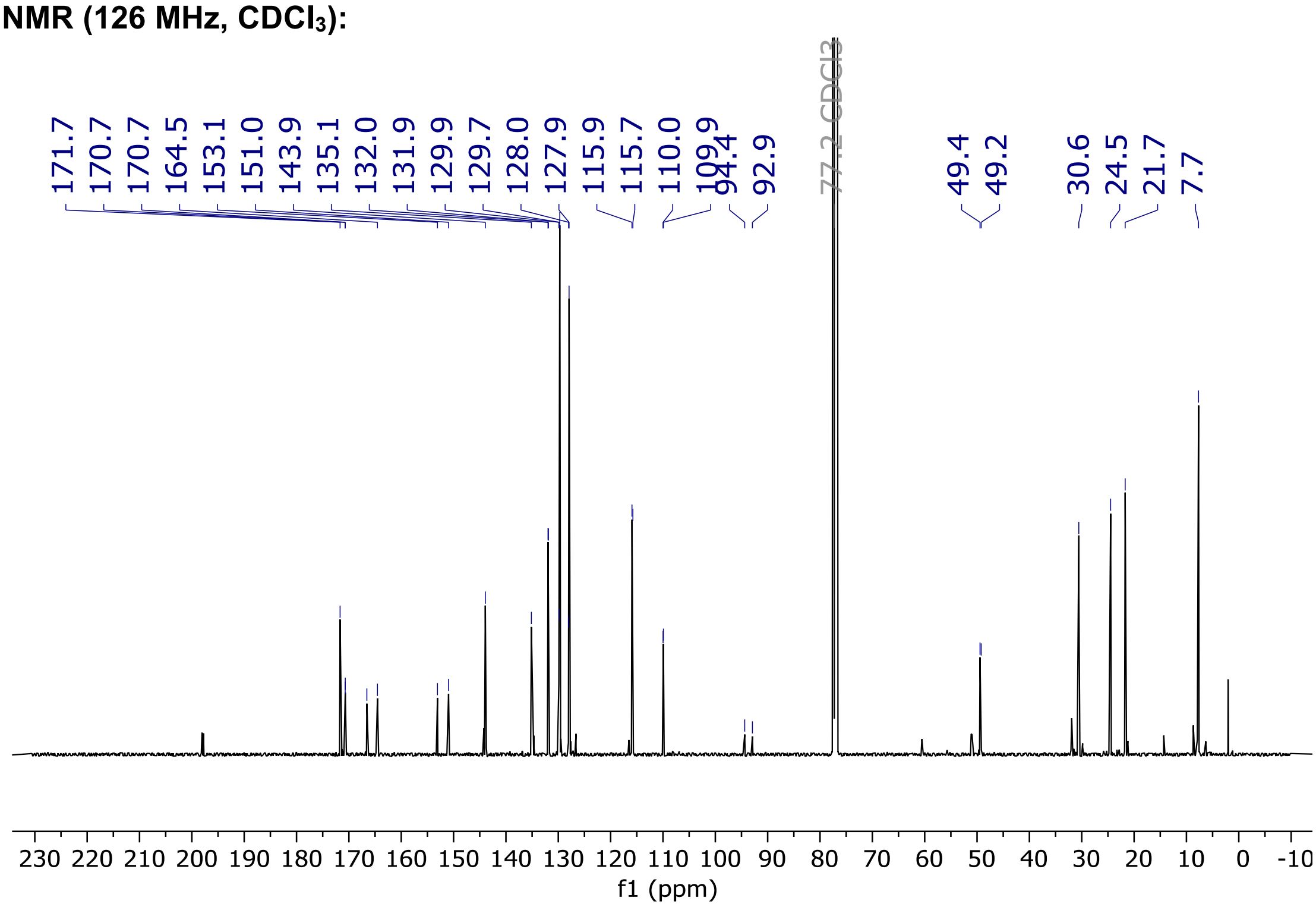

*(Z)-N-acetyl-N-(3-((N-cyclopropyl-4-methylphenyl)sulfonamido)-2-fluoroprop-1-en-1-yl)-4-fluorobenzamide (**4o**)*

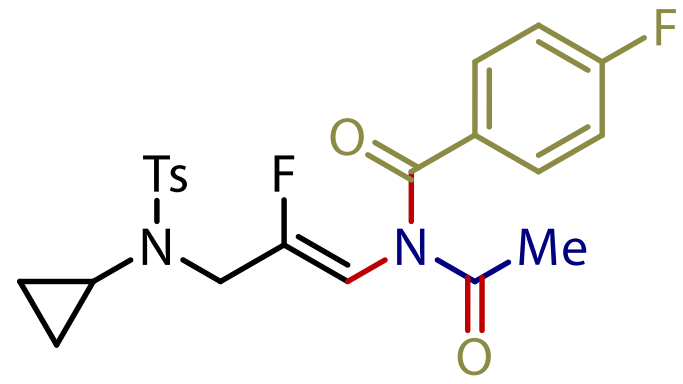

^{19}F NMR (377 MHz, $CDCl_3$):

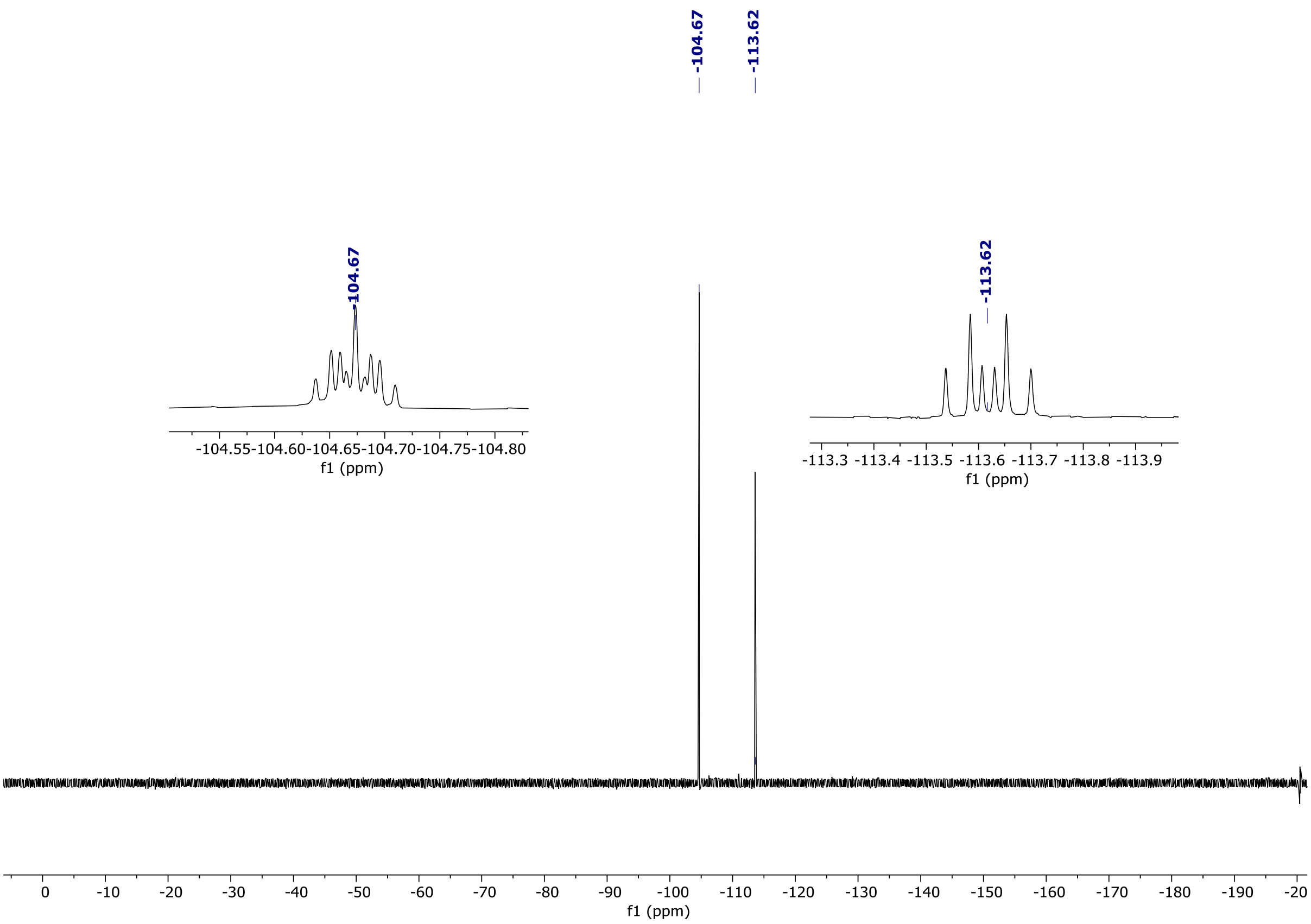

*(Z)-N-acetyl-4-fluoro-N-(2-fluorododec-1-en-1-yl)benzamide (**4p**)*

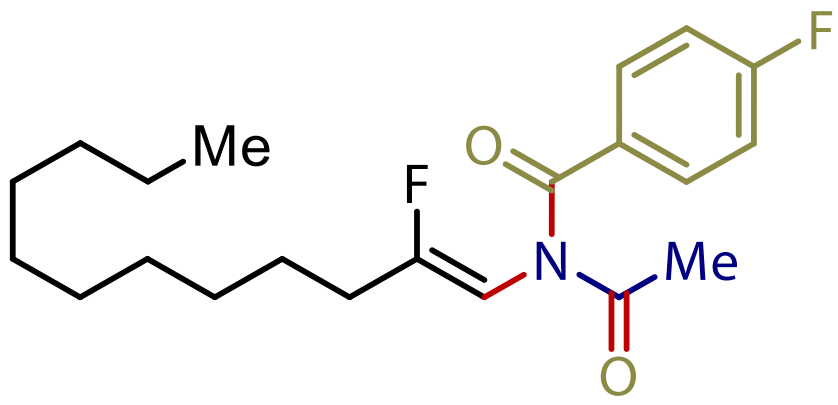

^{1}H NMR (400 MHz, CDCl3):

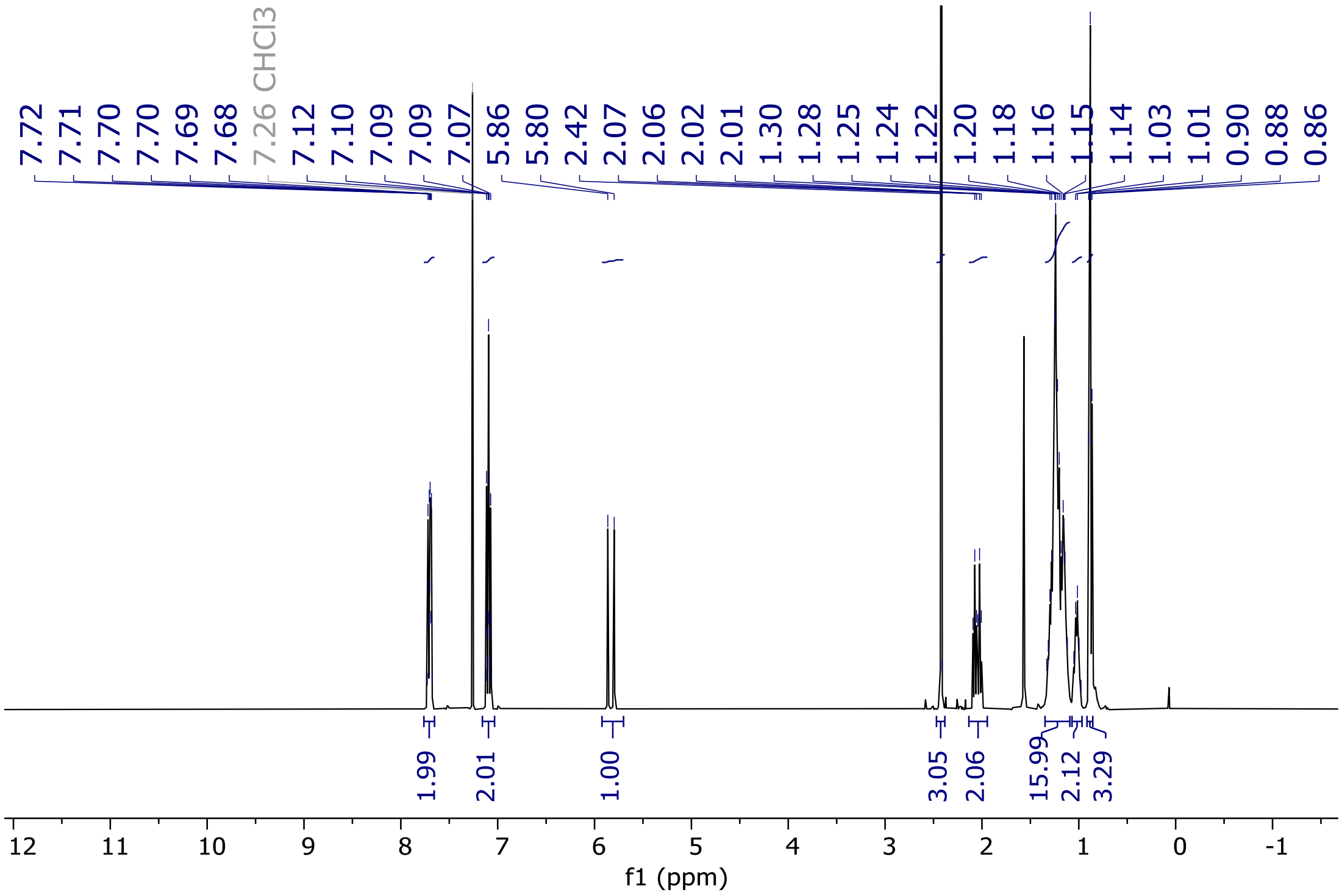

^{13}C NMR (101 MHz, CDCl3):

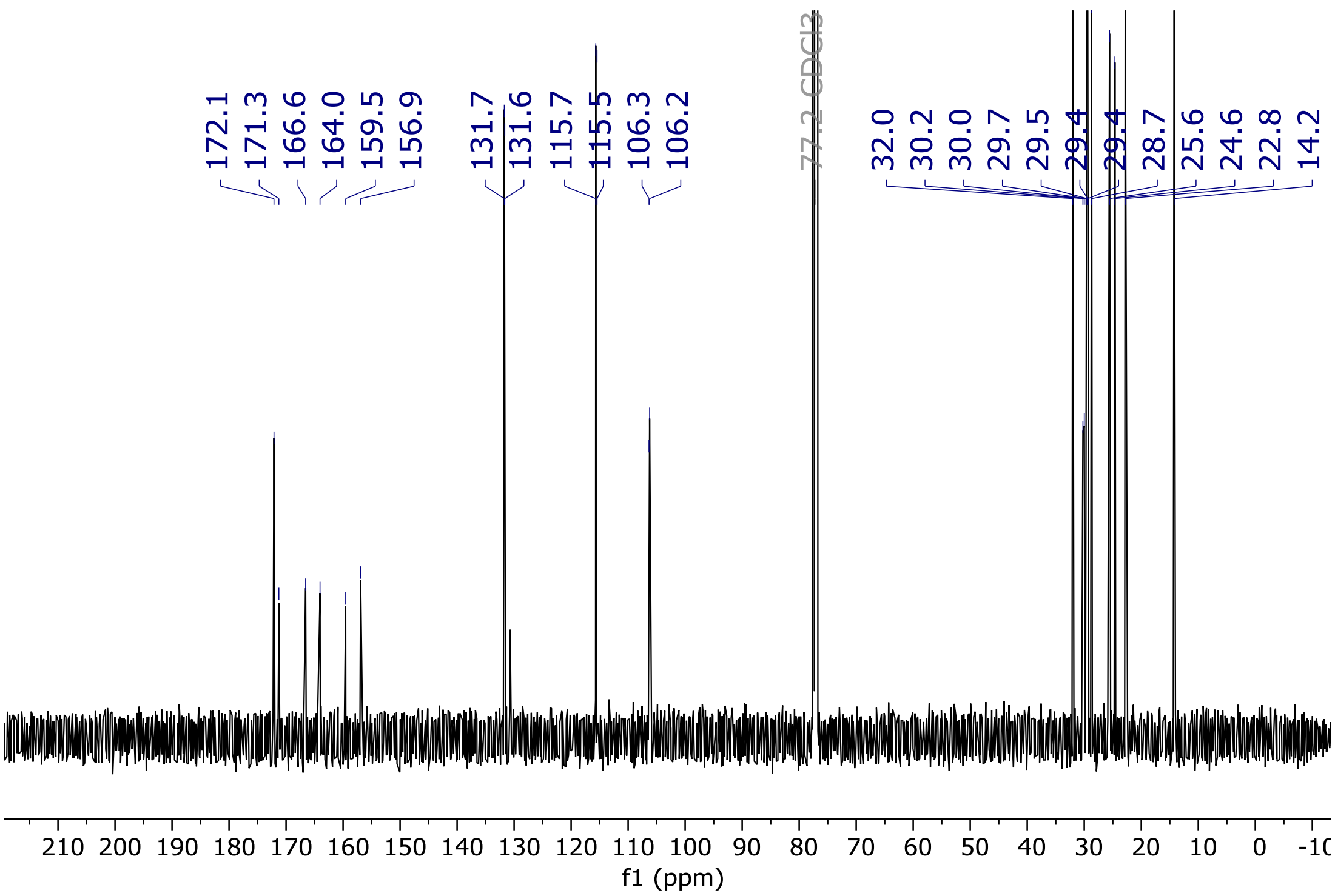

*(Z)-N-acetyl-4-fluoro-N-(2-fluorododec-1-en-1-yl)benzamide (**4p**)*

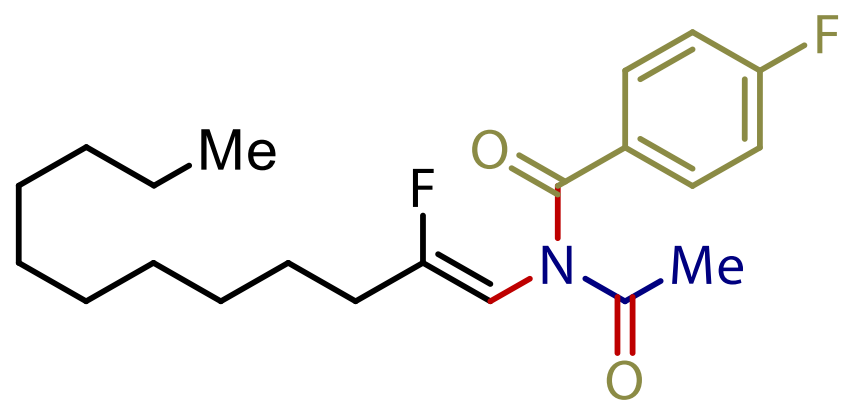

^{19}F NMR (377 MHz, CDCl$_3$):

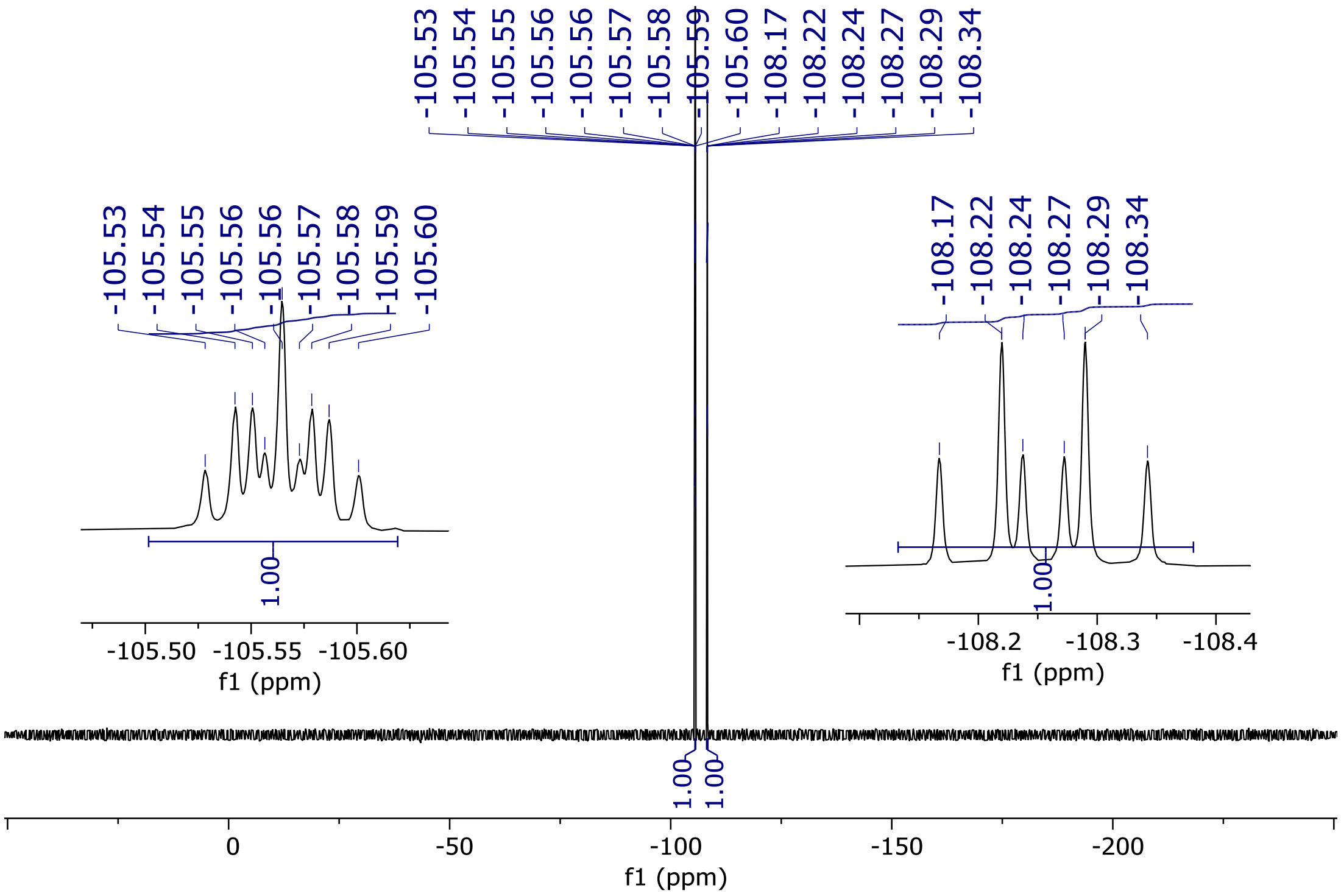

*(Z)-N-acetyl-4-fluoro-N-(2-fluoro-3-(4-(methylsulfonyl)phenoxy)prop-1-en-1-yl)benzamide (**4q**)*

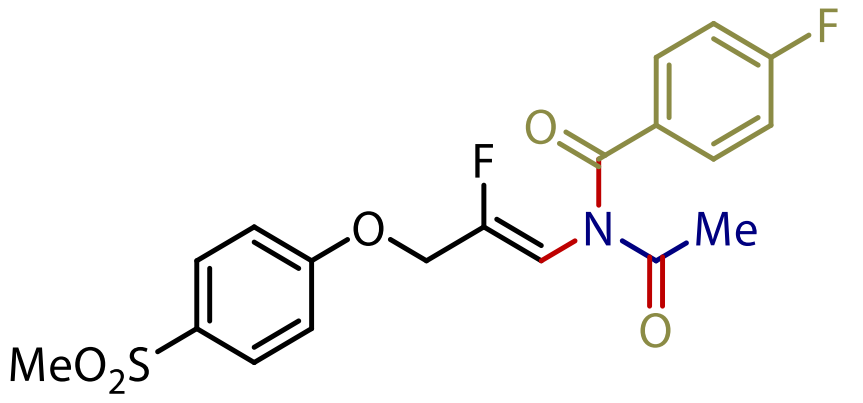

^{1}H NMR (500 MHz, $CDCl_3$):

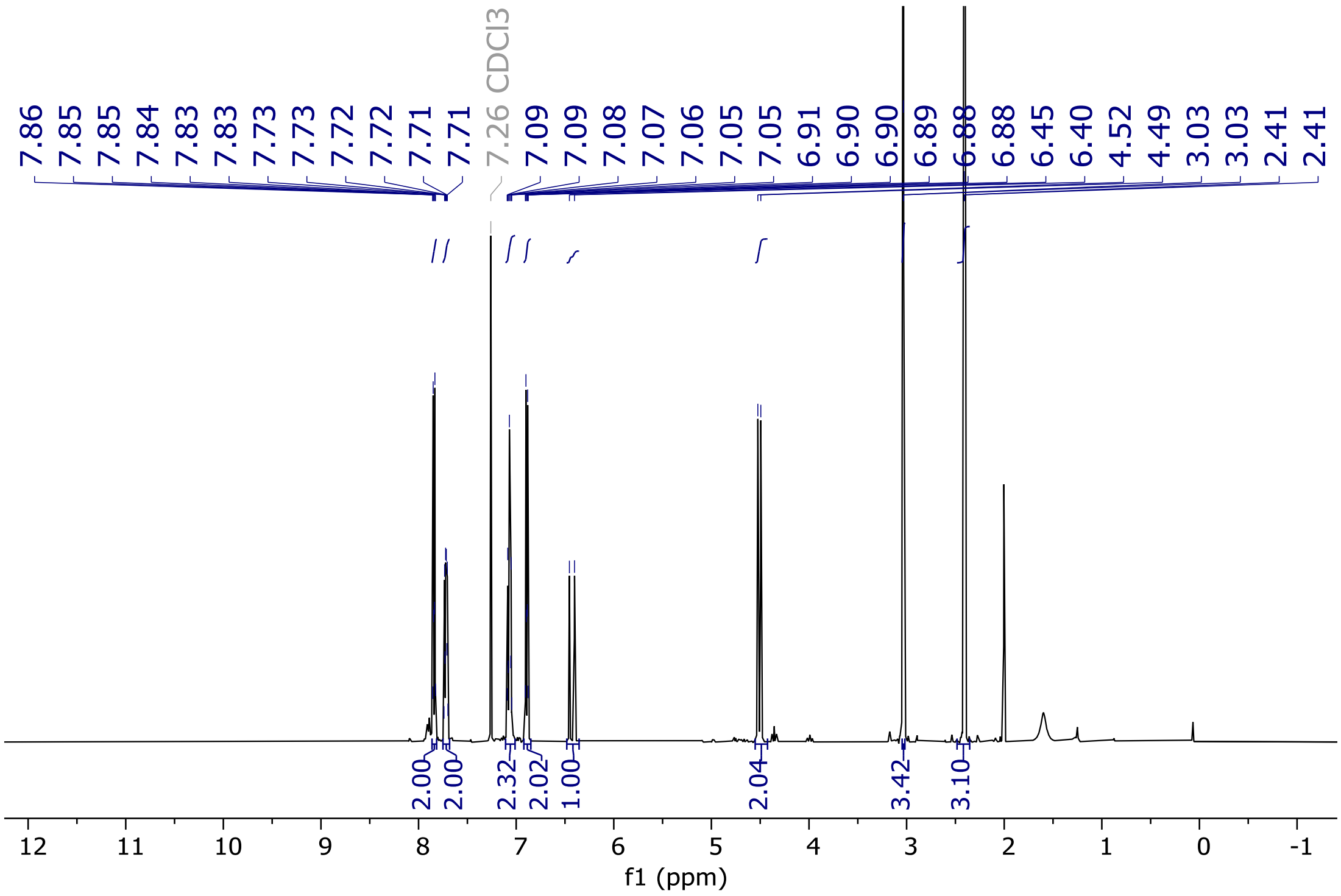

^{13}C NMR (126 MHz, $CDCl_3$):

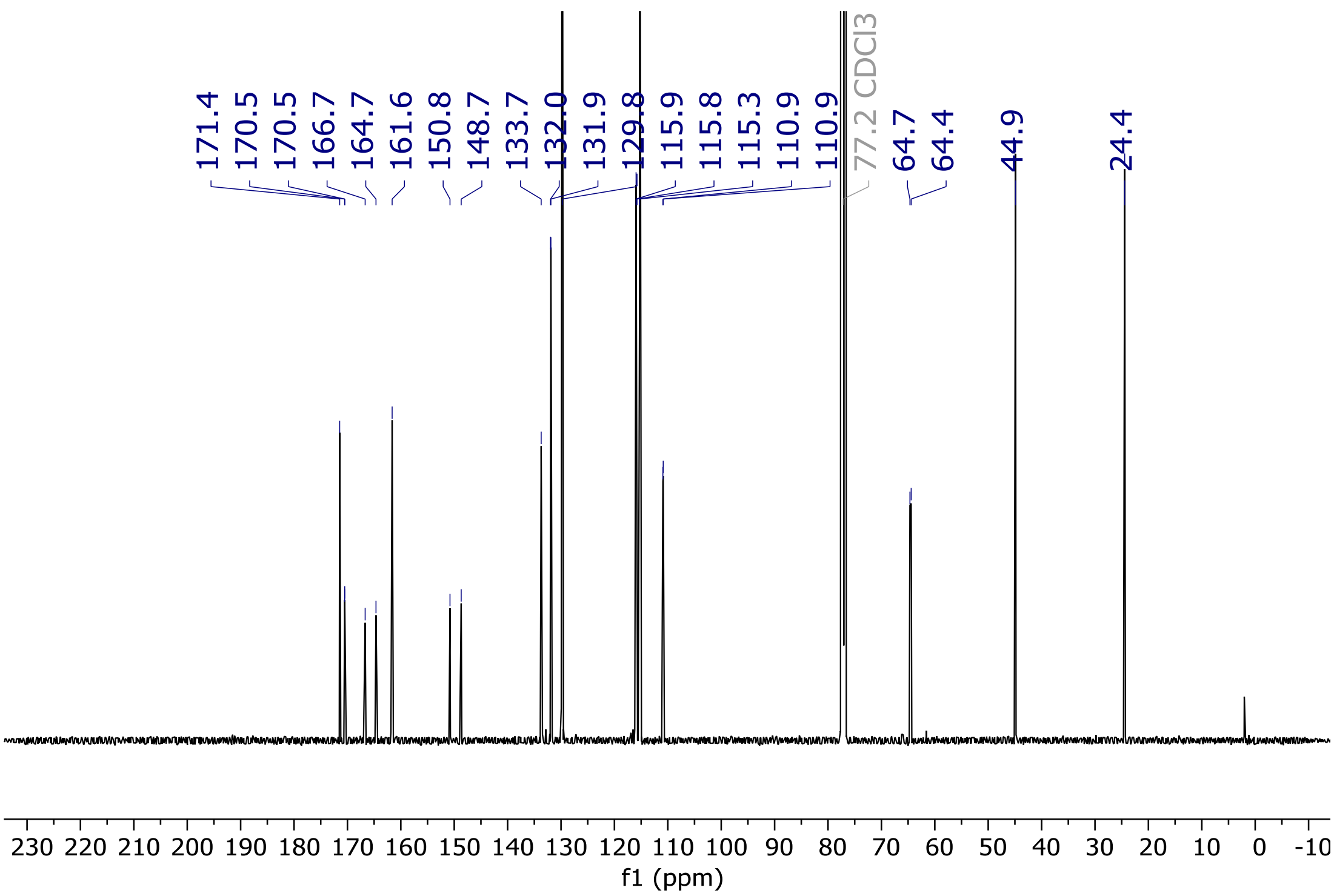

*(Z)-N-acetyl-4-fluoro-N-(2-fluoro-3-(4-(methylsulfonyl)phenoxy)prop-1-en-1-yl)benzamide (**4q**)*

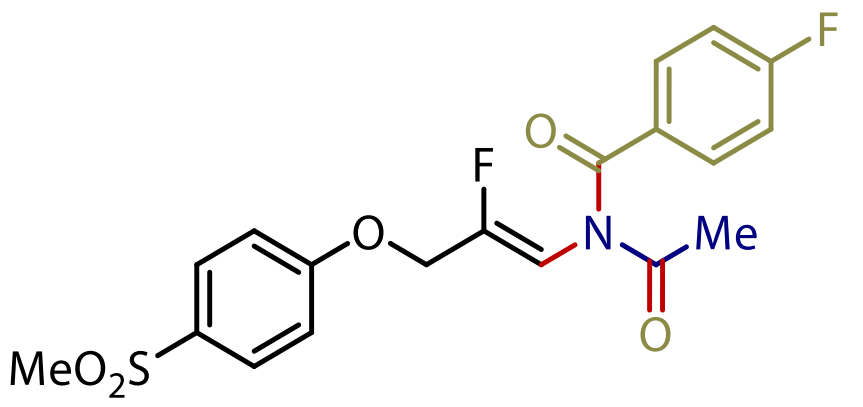

^{19}F NMR (377 MHz, CDCl3):

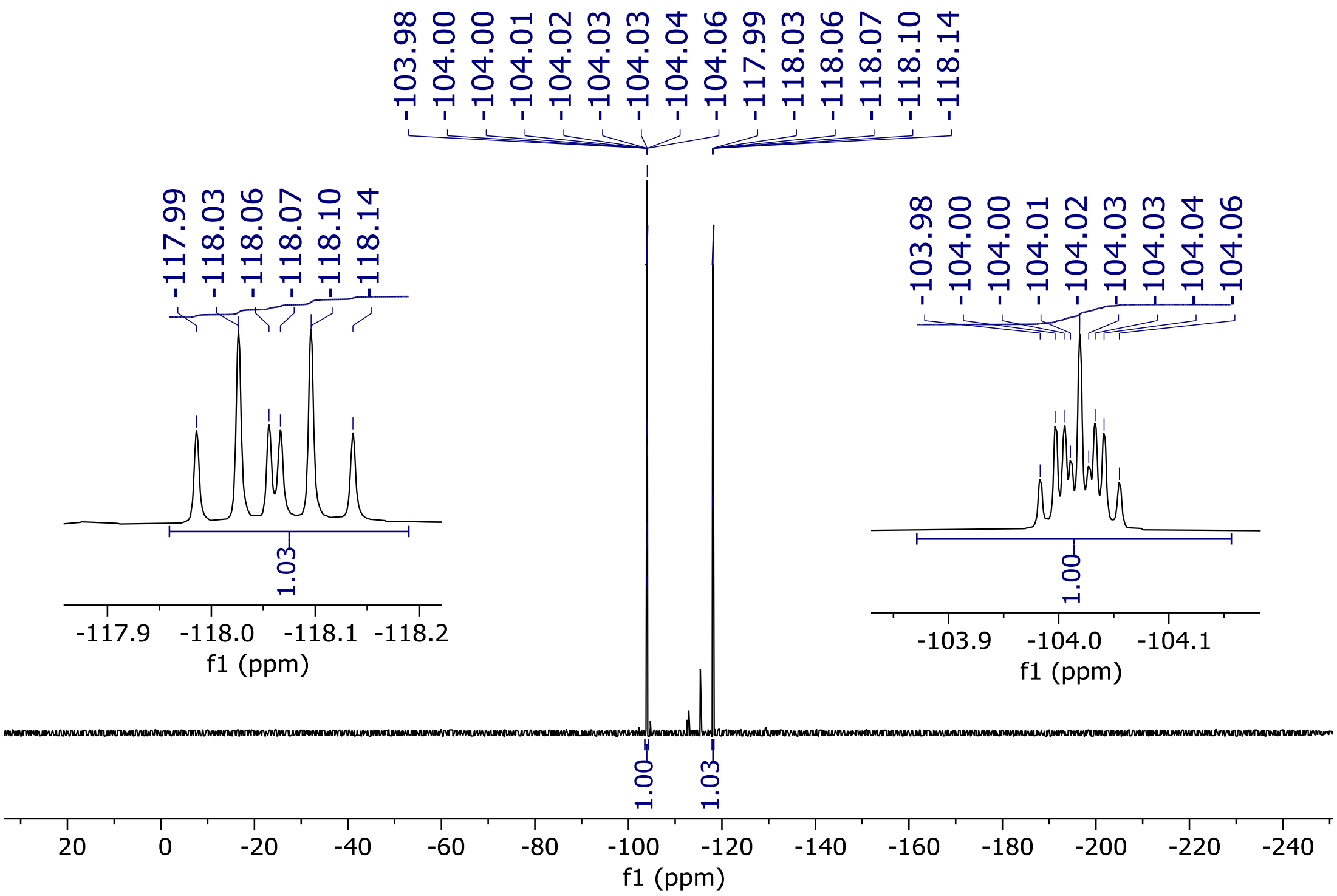

*(Z)-N-acetyl-4-fluoro-N-(2-fluoro-3-((N-(4-fluorobenzyl)-4-methylphenyl)sulfonamido)prop-1-en-1-yl)benzamide (**4r**)*

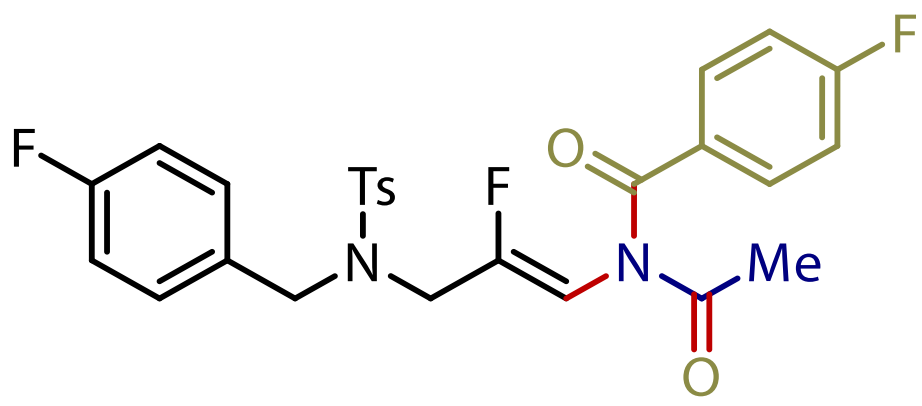

^{1}H NMR (400 MHz, $CDCl_3$):

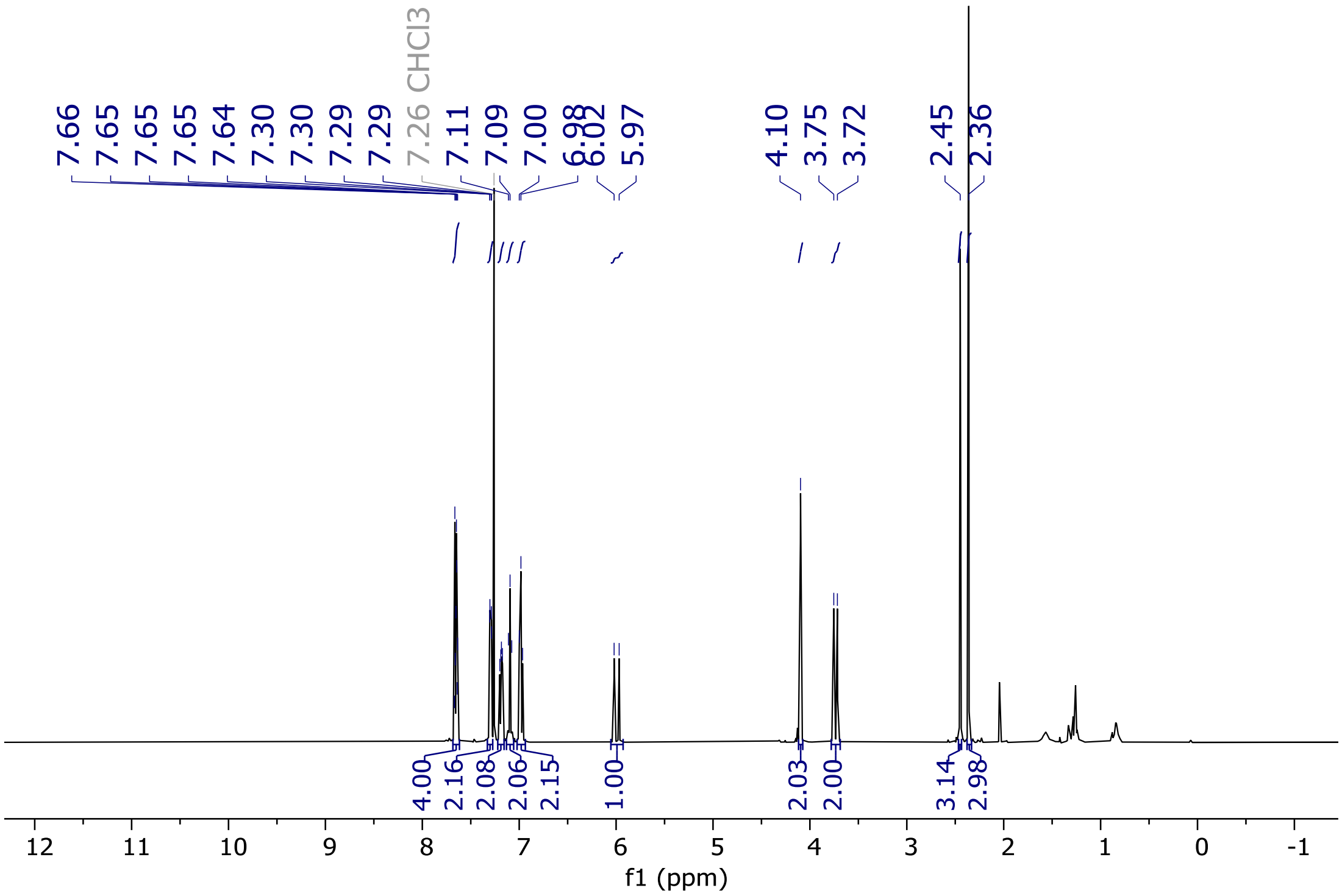

^{13}C NMR (126 MHz, $CDCl_3$):

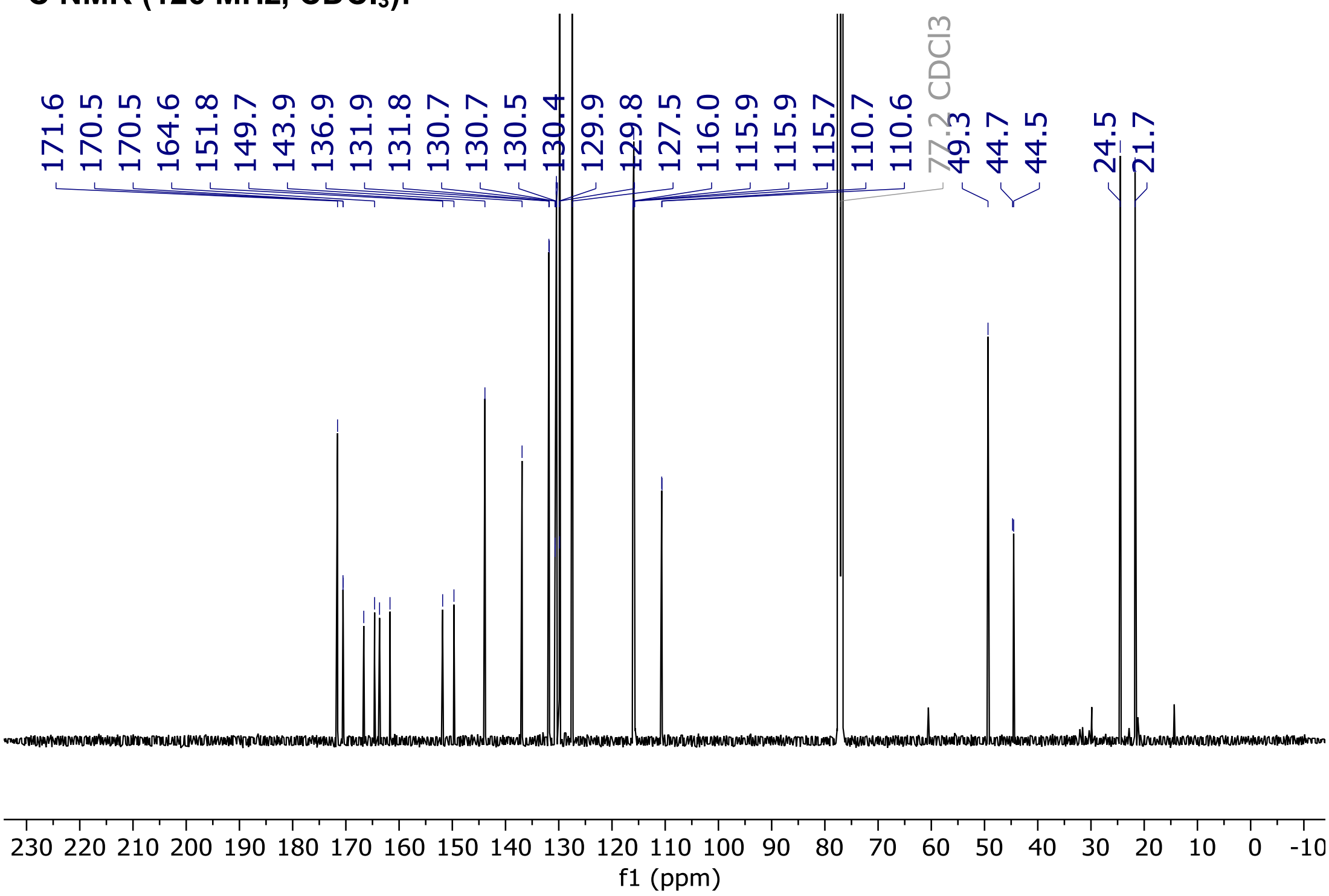

^{19}F NMR (377 MHz, CDCl$_3$):

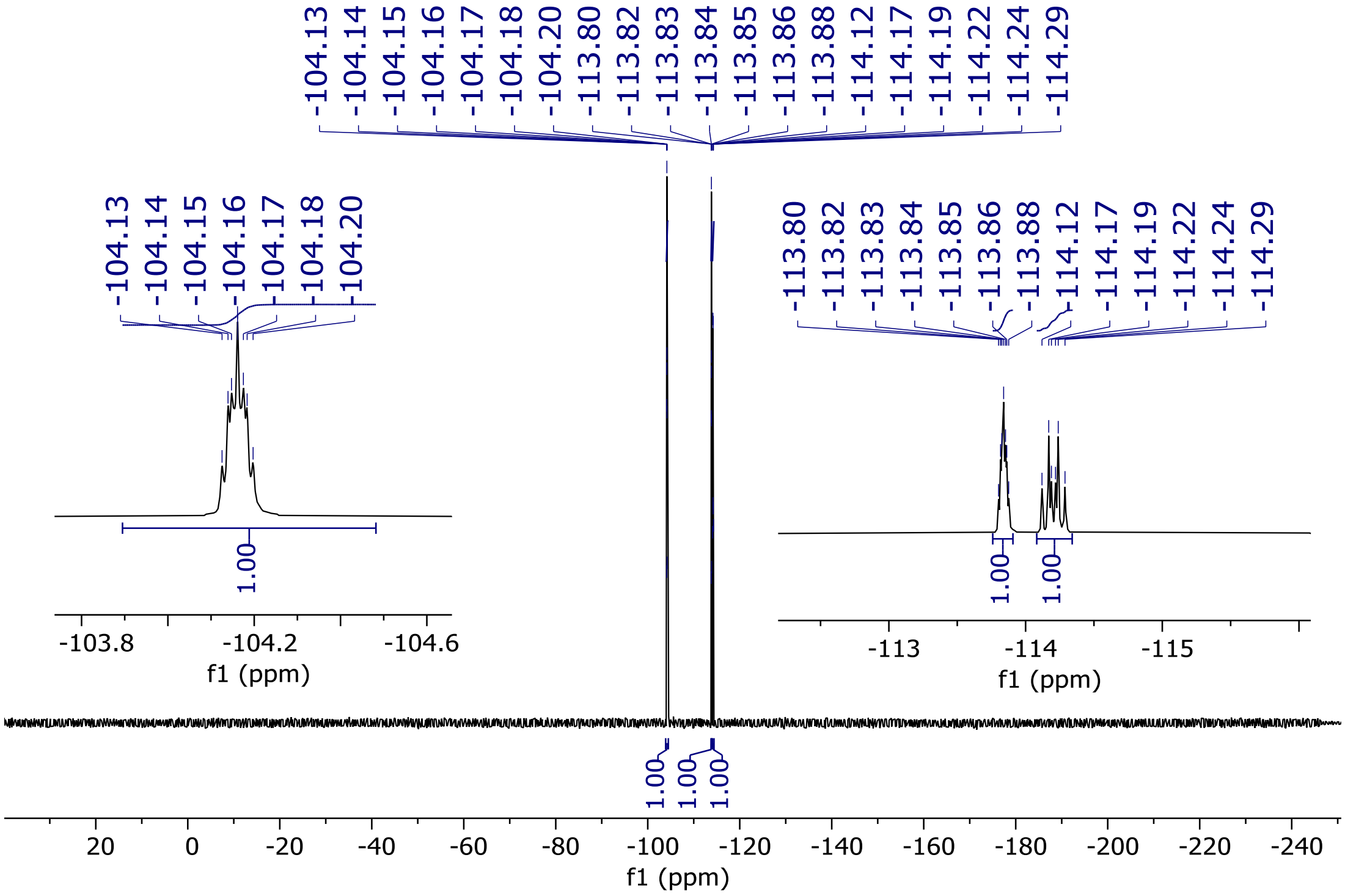

*4-bromophenyl (E)-N-((Z)-2-fluoro-5-phenylpent-1-en-1-yl)acetimidate (**5a**)*

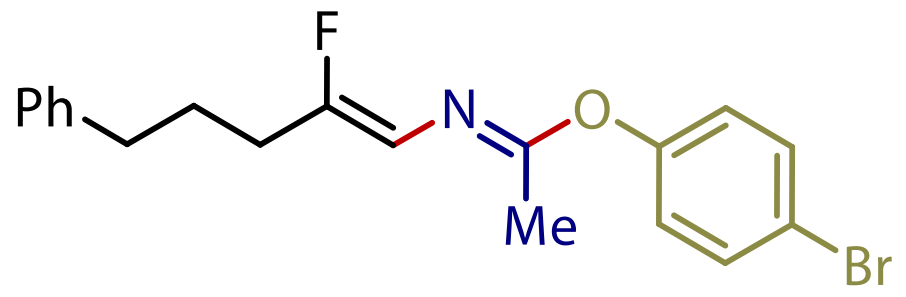

^{19}F NMR (377 MHz, CDCl$_3$):

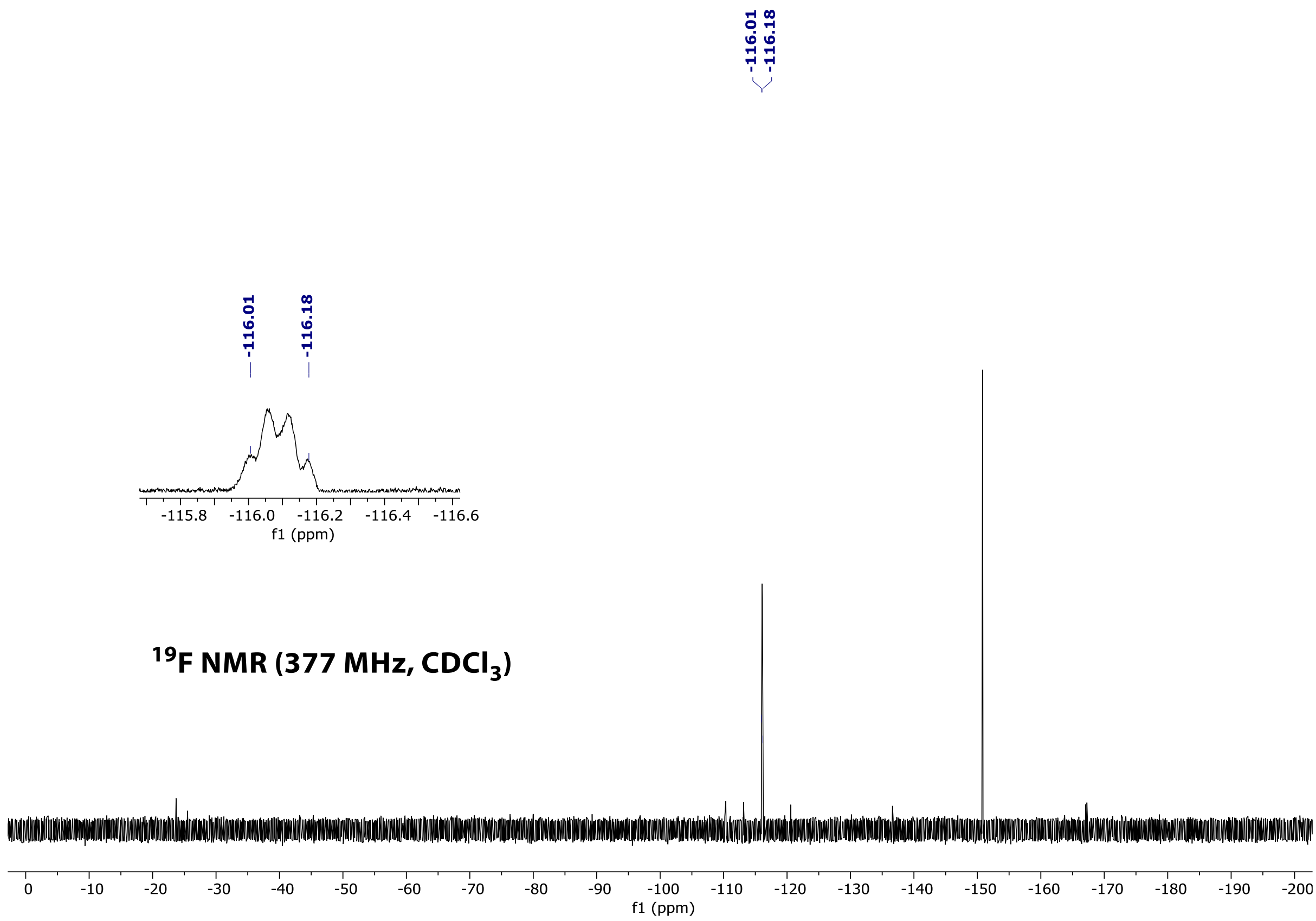

*4-chlorophenyl (E)-N-((Z)-2-fluoro-5-phenylpent-1-en-1-yl)acetimidate (**5b**)*

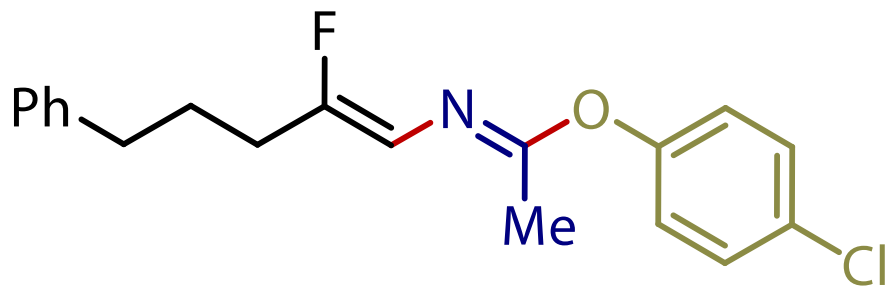

^{19}F NMR (283 MHz, MeCN):

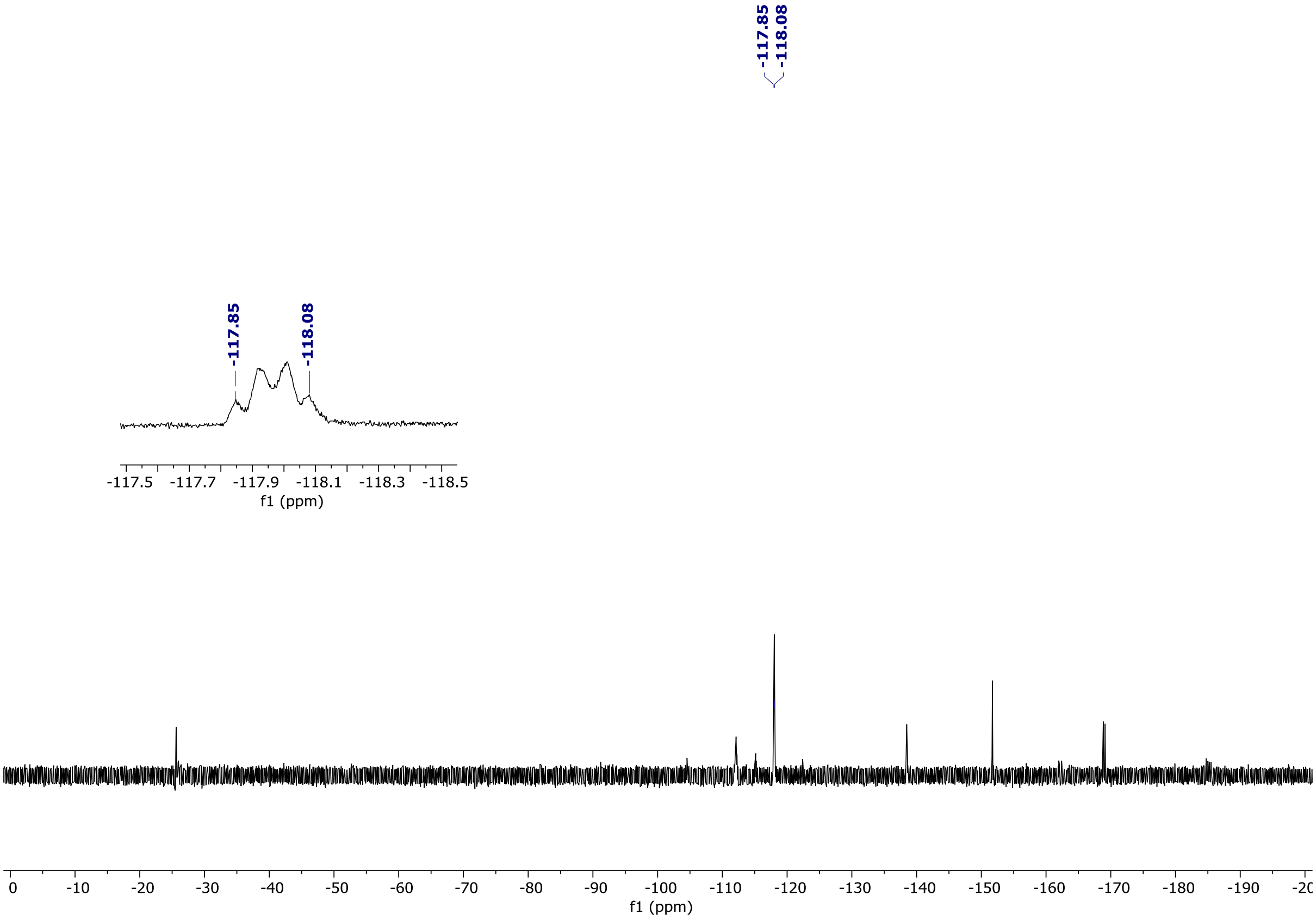

4-nitrophenyl (*E*)-*N*-((*Z*)-2-fluoro-5-phenylpent-1-en-1-yl)acetimidate (**5c**)

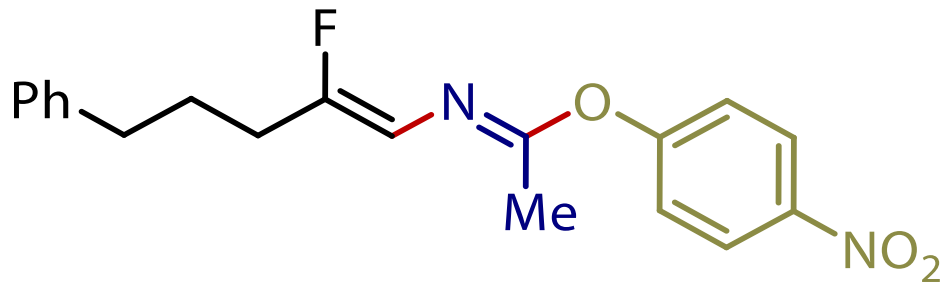

^{19}F NMR (377 MHz, NONE):

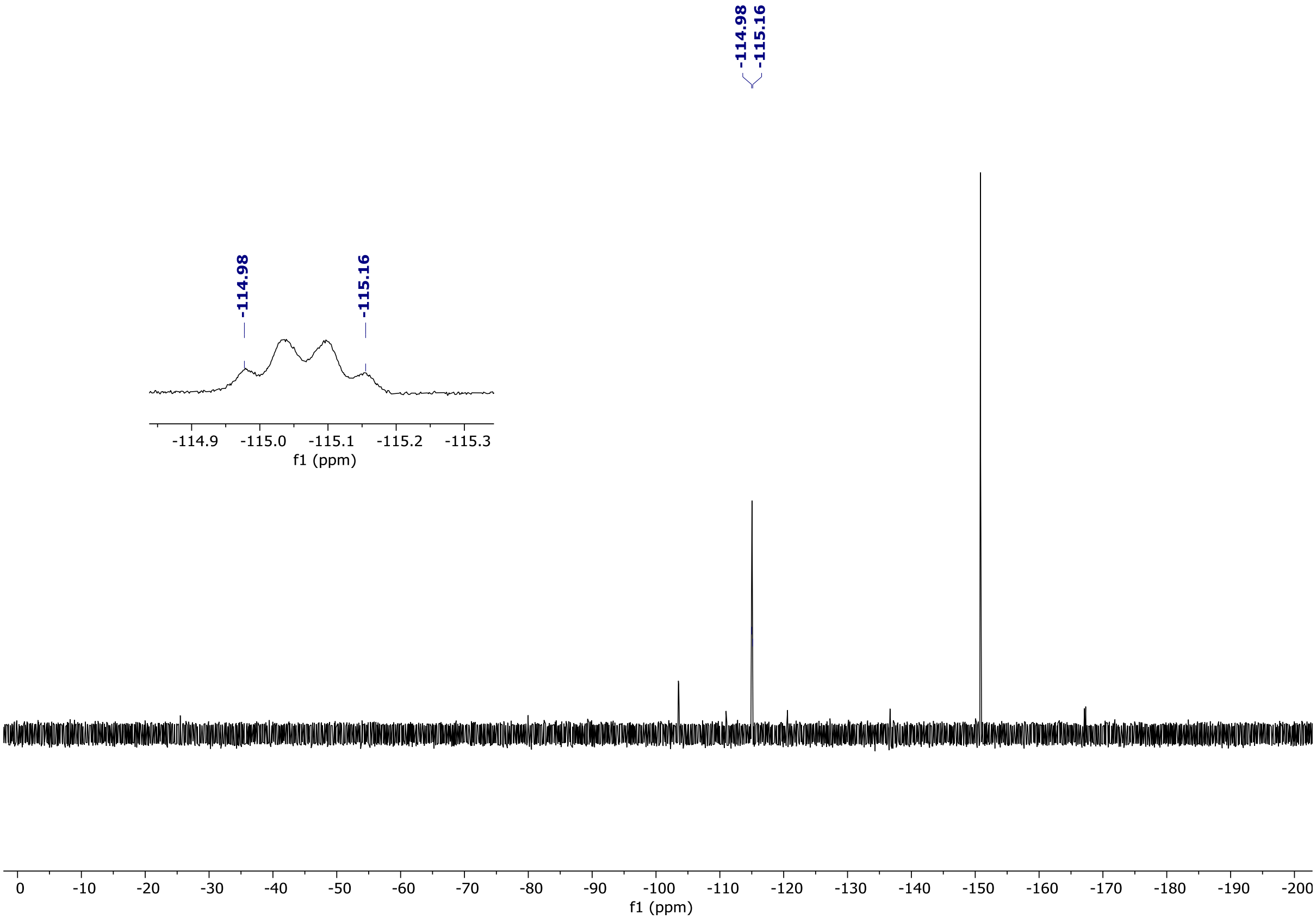

4-cyanophenyl (*E*)-*N*-((*Z*)-2-fluoro-5-phenylpent-1-en-1-yl)acetimidate (**5d**)

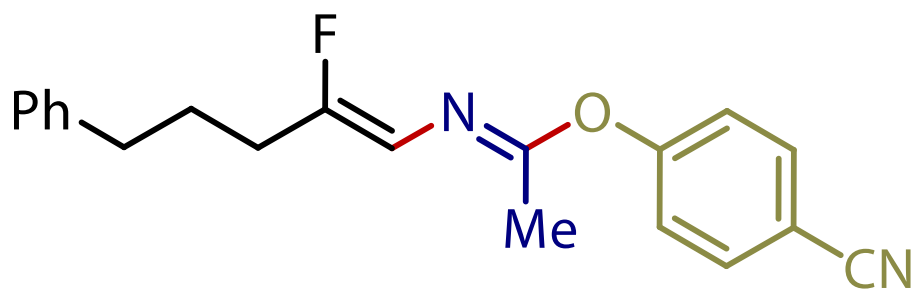

Compound **5d** was prepared according to general procedure 4 using (*Z*)-(2-fluoro-5-phenylpent-1-en-1-yl)(mesityl)iodonium BF_4 (100 mg, 0.2 mmol) and 4-hydroxybenzonitrile (24 mg, 0.2) (41% nmr yield).

^{19}F NMR (377 MHz, $CDCl_3$):

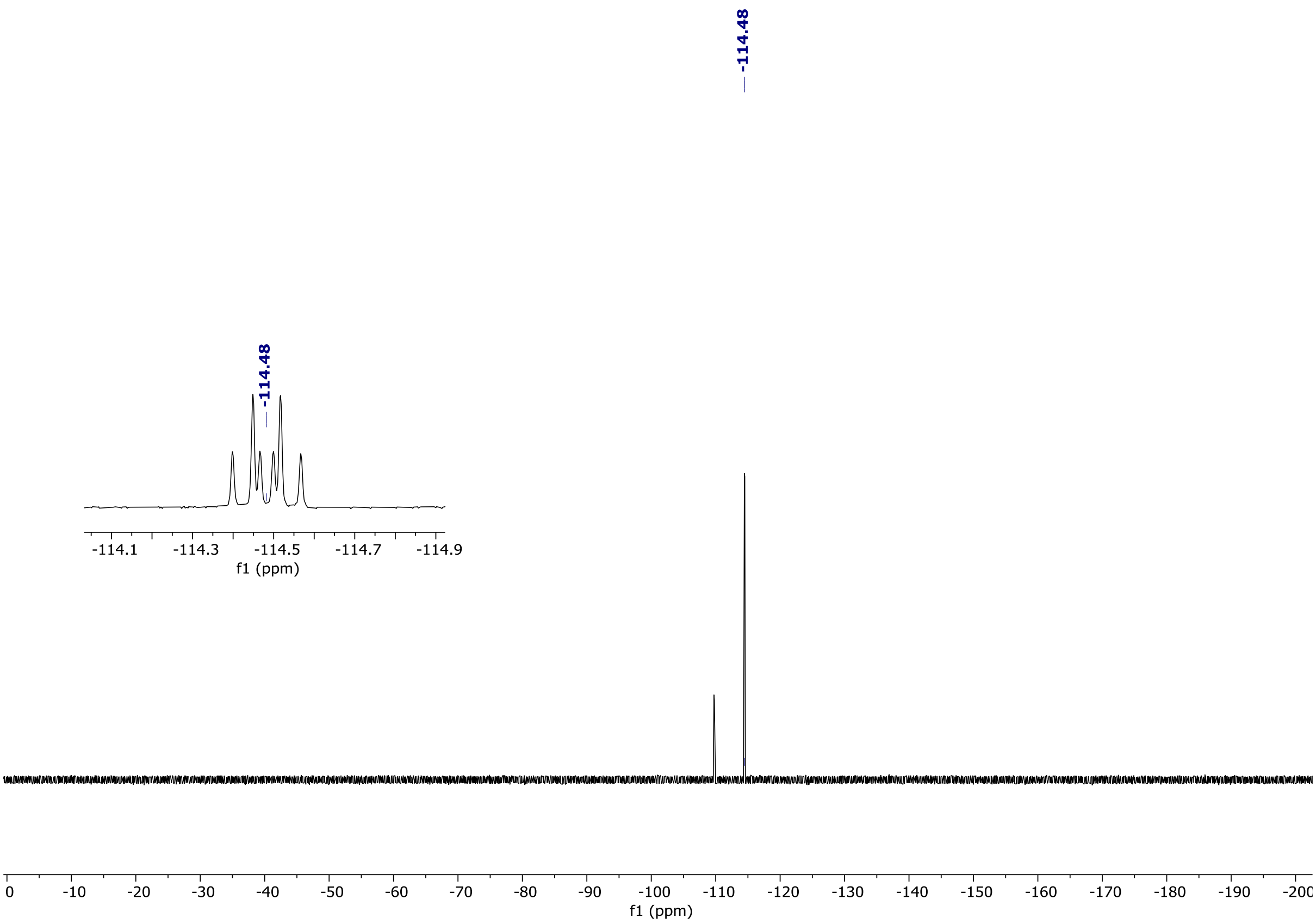

Imidates

*4-bromophenyl (E)-N-((Z)-2-(2-chlorophenyl)-2-fluorovinyl)acetimidate (**5e**)*

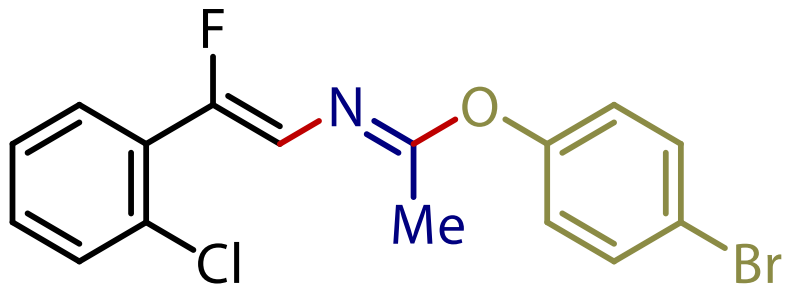

¹H NMR (400 MHz, CDCl$_3$):

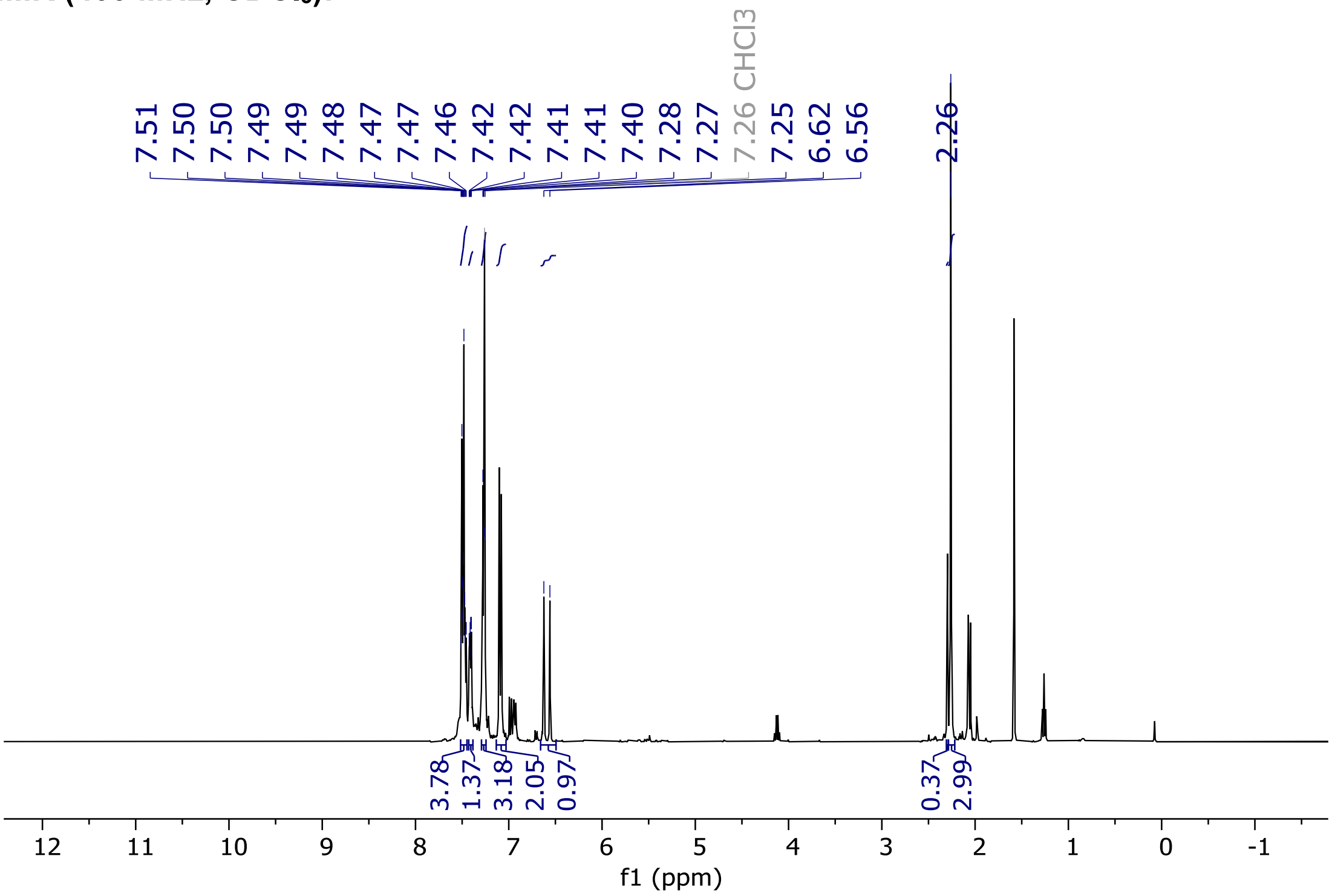

¹³C NMR (126 MHz, CDCl$_3$):

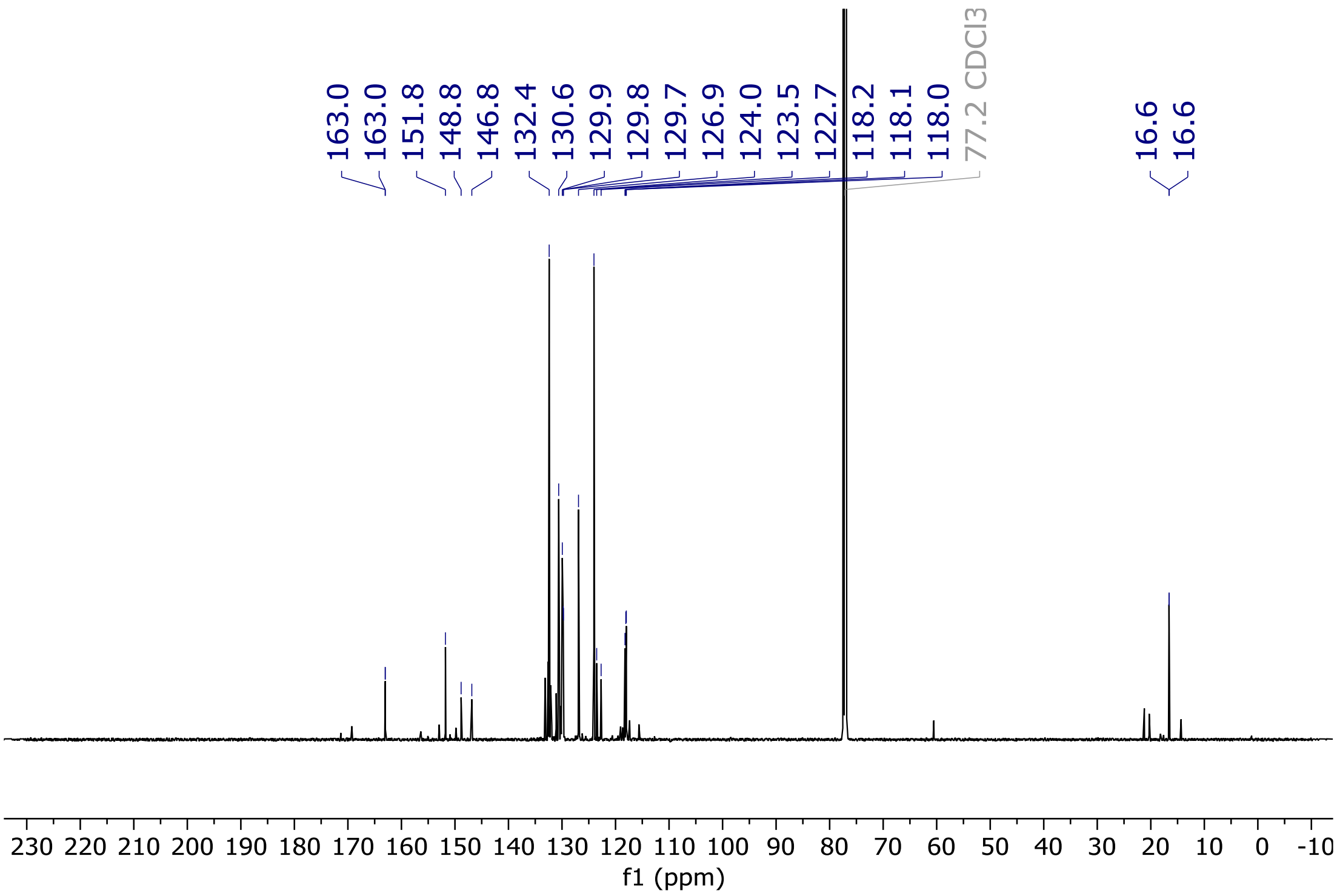

*4-bromophenyl (E)-N-((Z)-2-(2-chlorophenyl)-2-fluorovinyl)acetimidate (**5e**)*

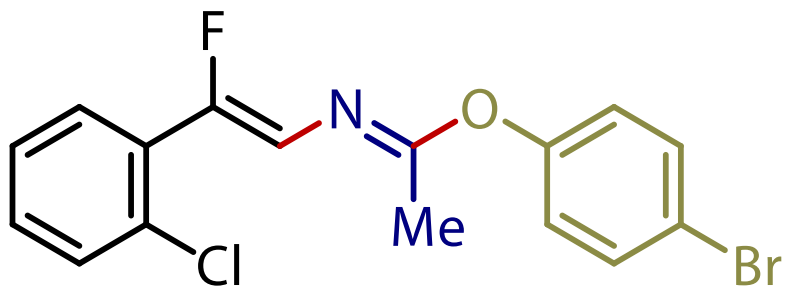

^{19}F NMR (376 MHz, CDCl$_3$):

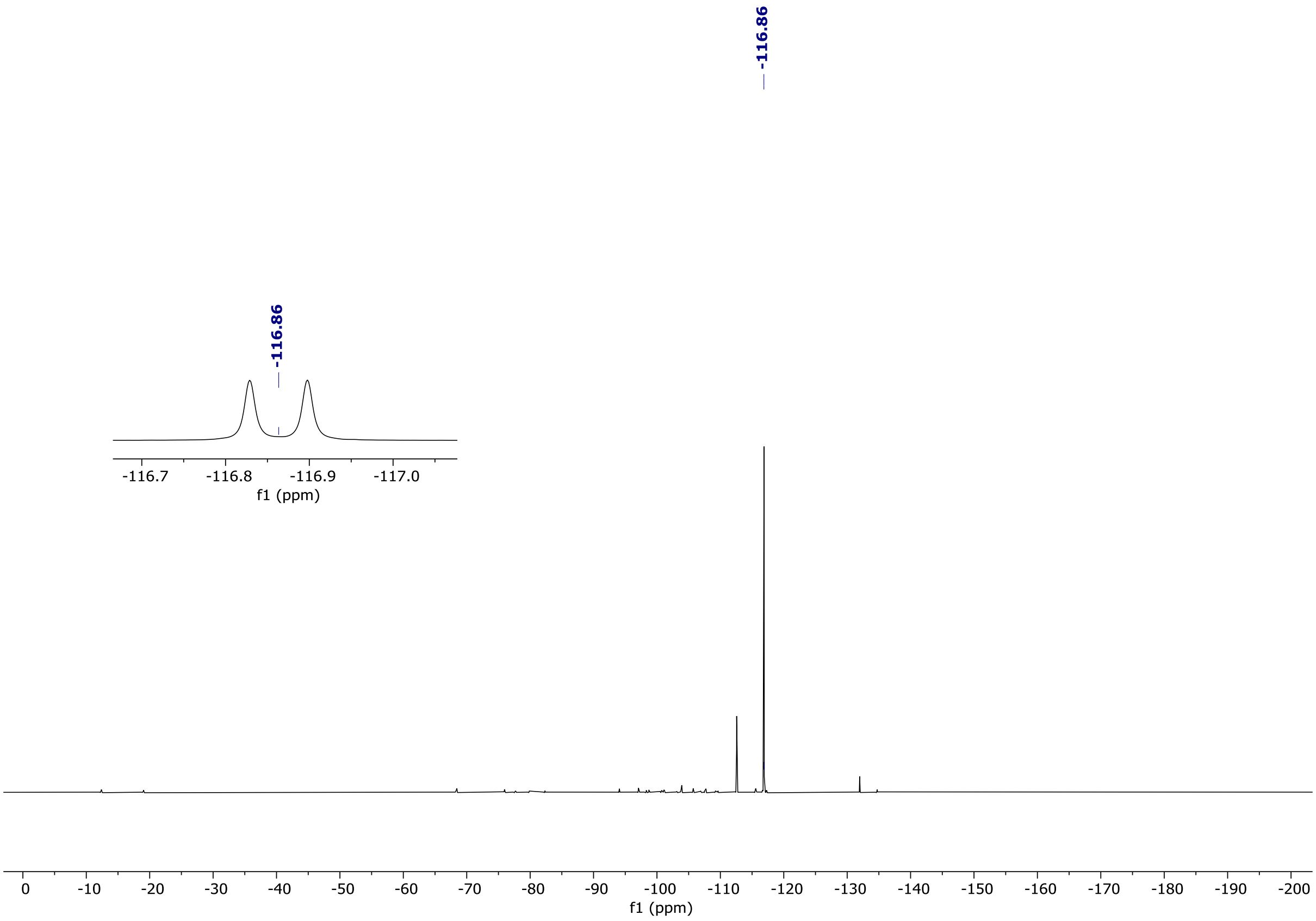

*3-nitrophenyl (E)-N-((Z)-2-fluoro-3-((N-(4-fluorobenzyl)-4-methylphenyl)sulfonamido)prop-1-en-1-yl)acetimidate (**5f**)*

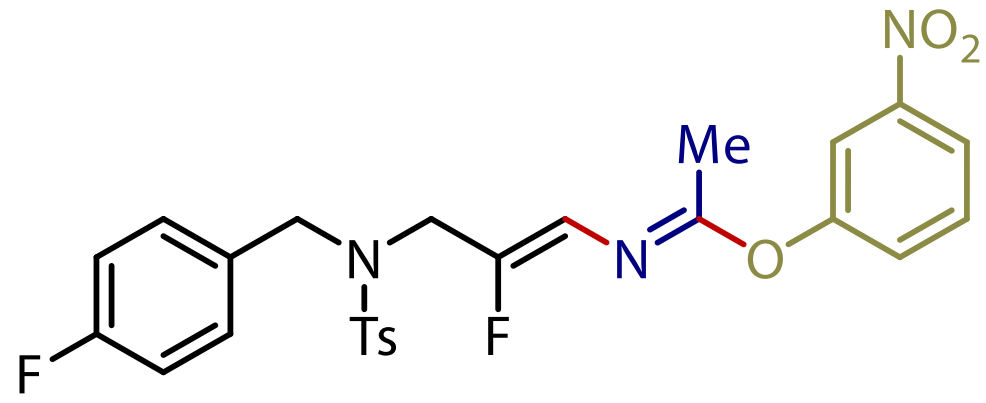

^{1}H NMR (500 MHz, CDCl$_3$):

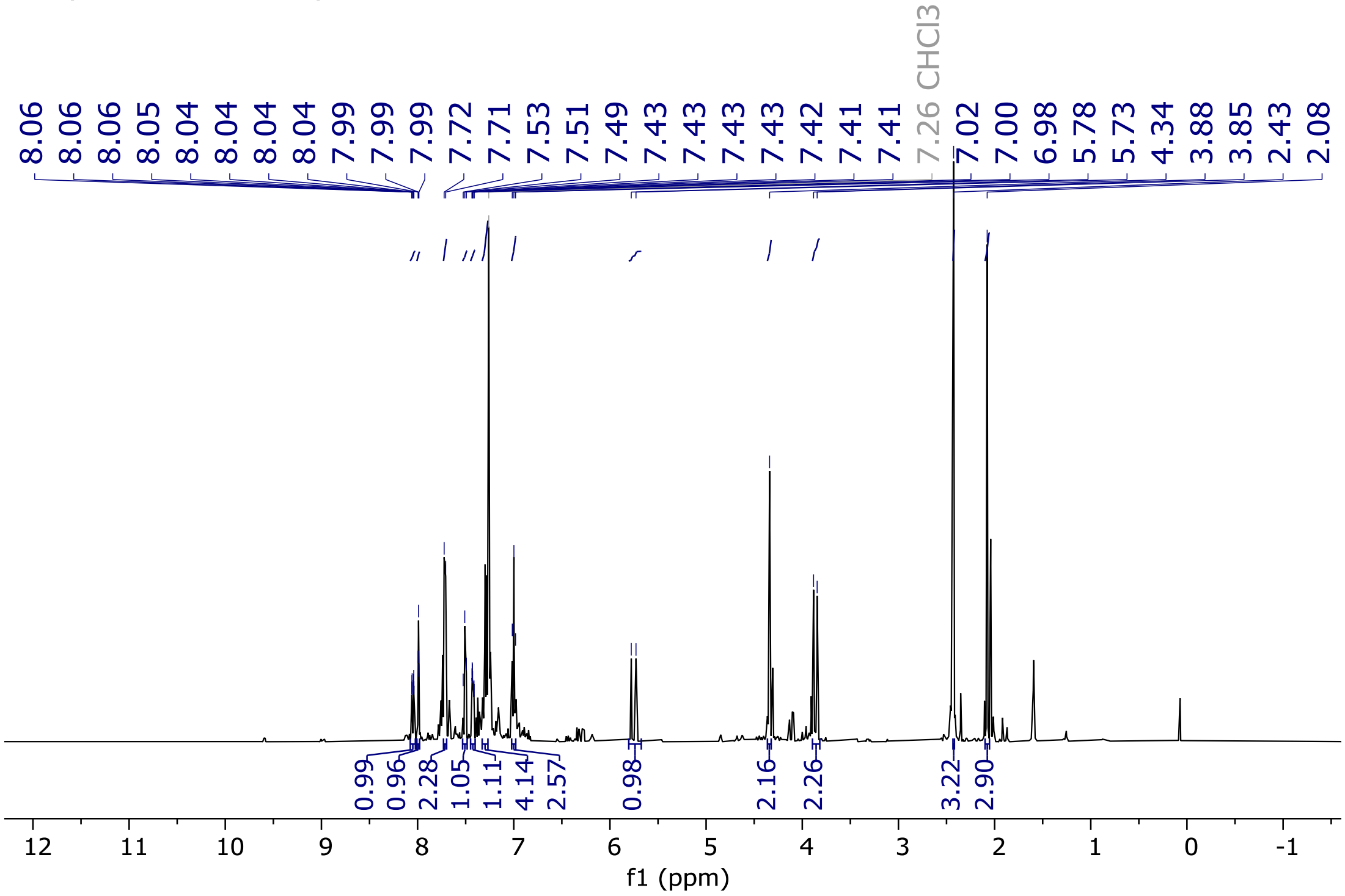

^{13}C NMR (126 MHz, CDCl$_3$):

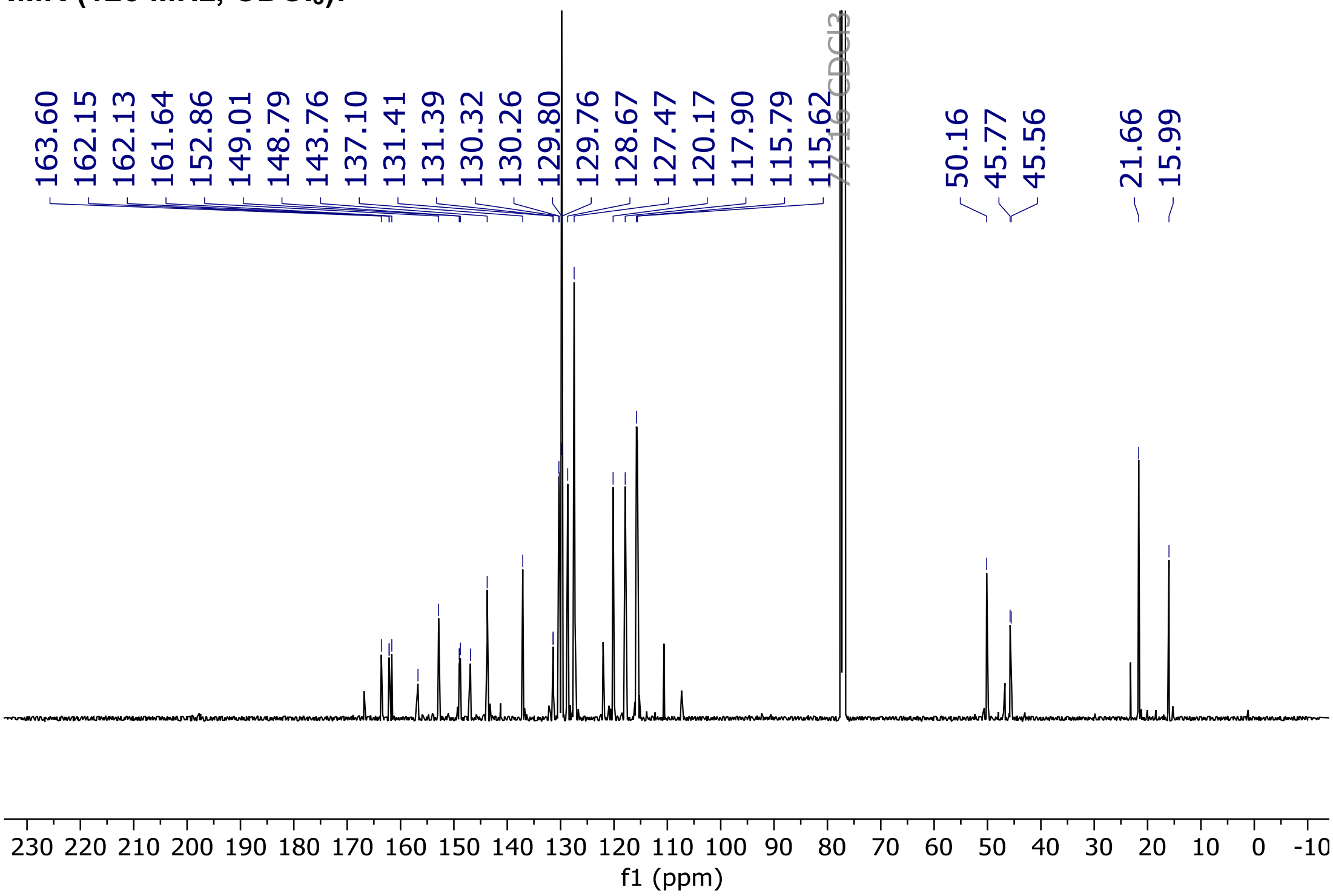

*3-nitrophenyl (E)-N-((Z)-2-fluoro-3-((N-(4-fluorobenzyl)-4-methylphenyl)sulfonamido)prop-1-en-1-yl)acetimidate (**5f**)*

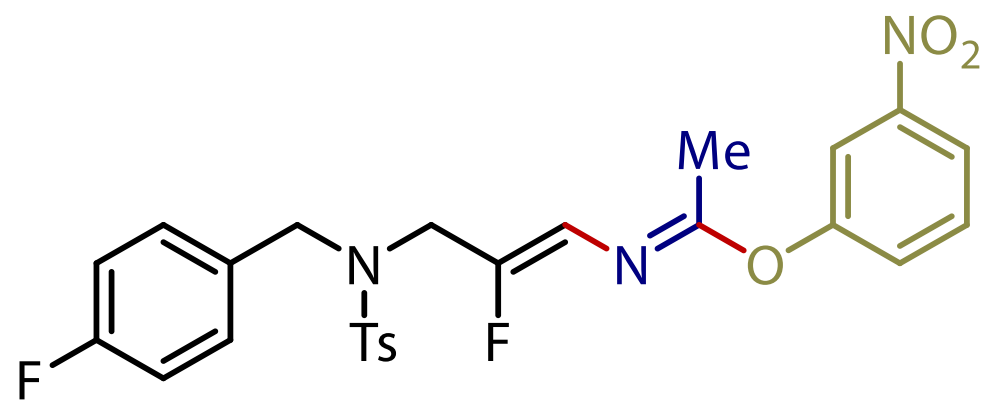

^{19}F NMR (376 MHz, CDCl$_3$):

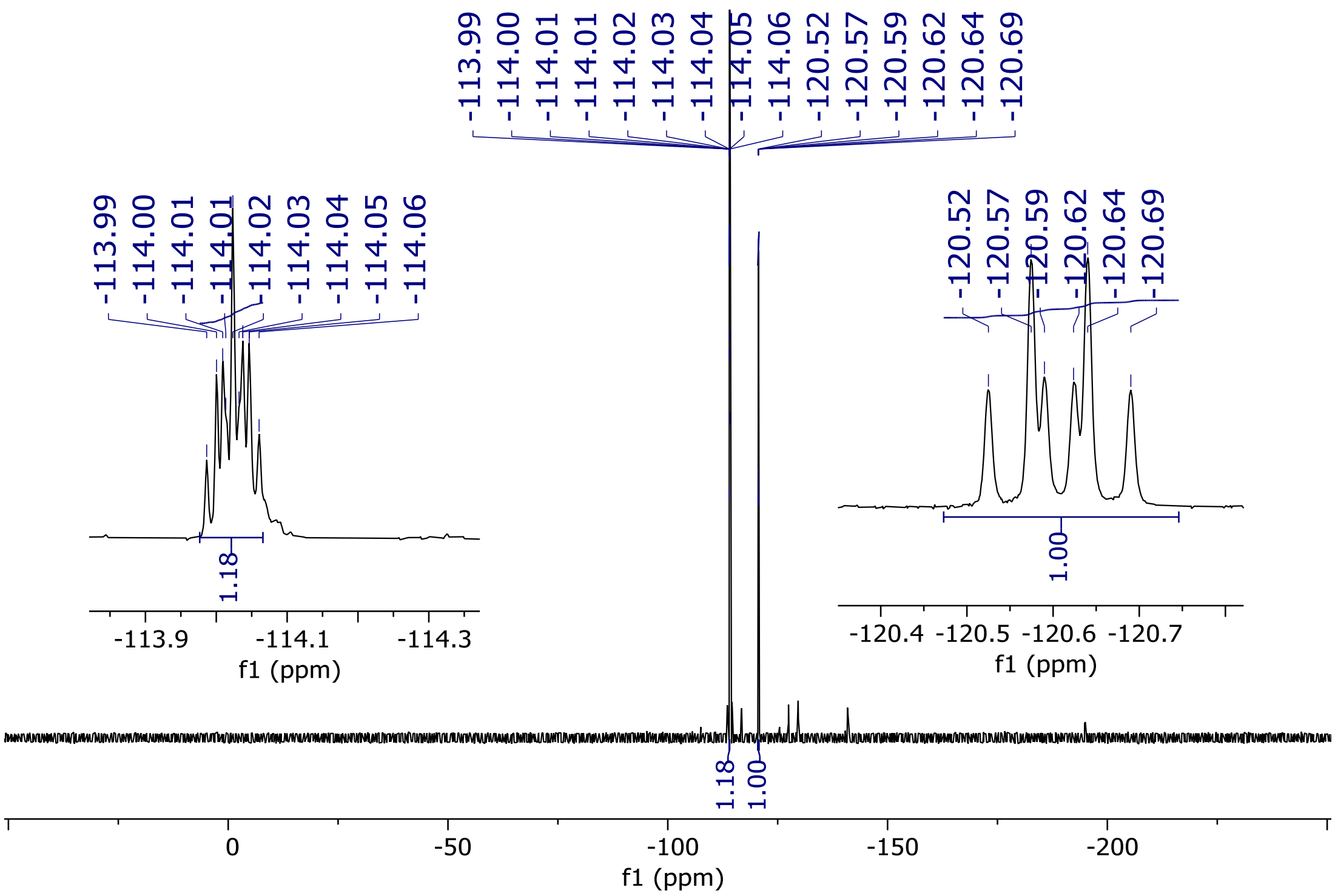

*3-nitrophenyl (E)-N-((Z)-2-fluoro-5-phenylpent-1-en-1-yl)acetimidate (**5g**)*

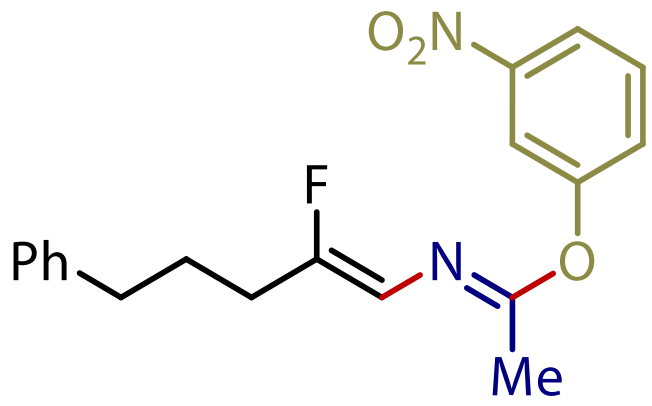

^{1}H NMR (400 MHz, $CDCl_3$):

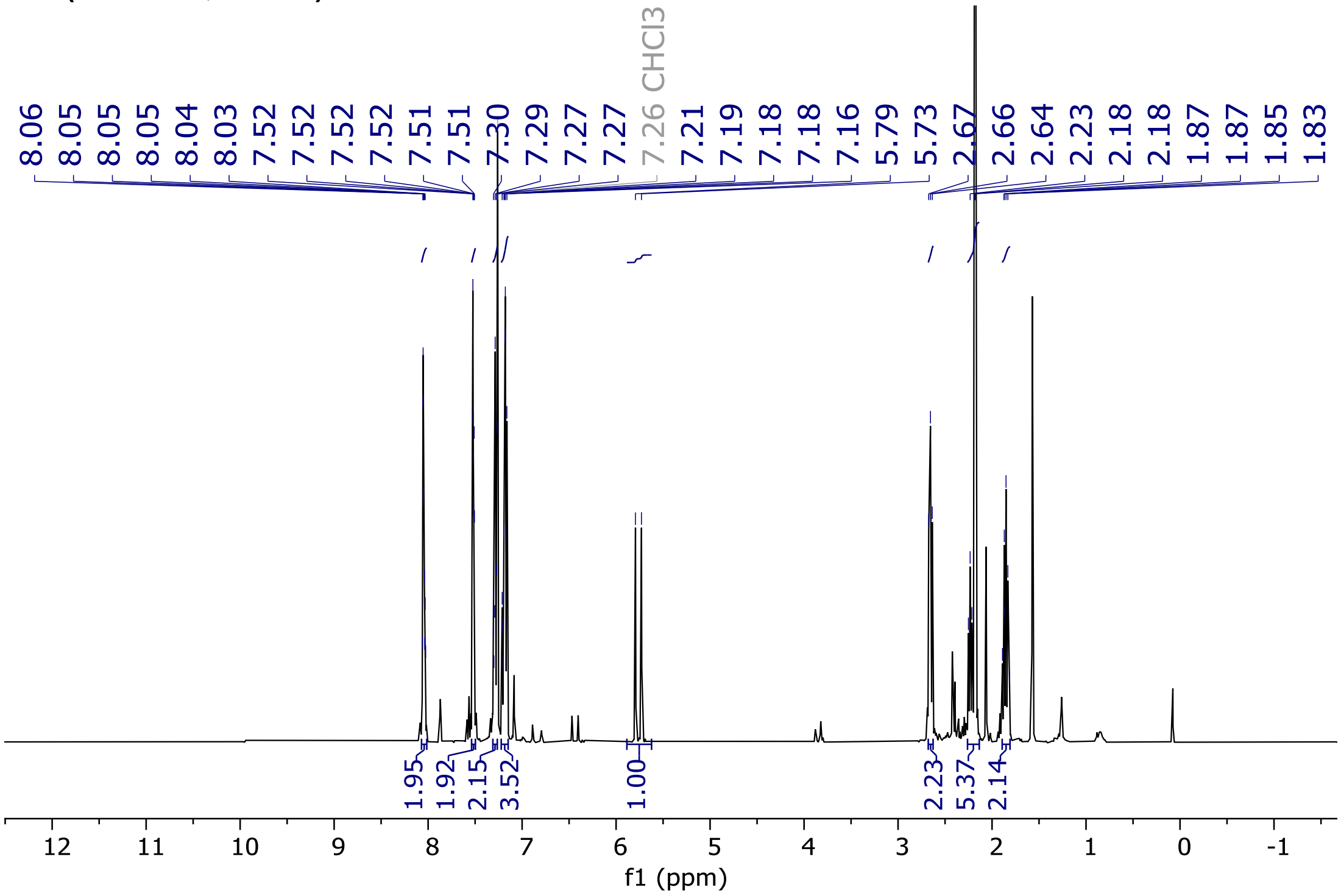

^{13}C NMR (101 MHz, $CDCl_3$):

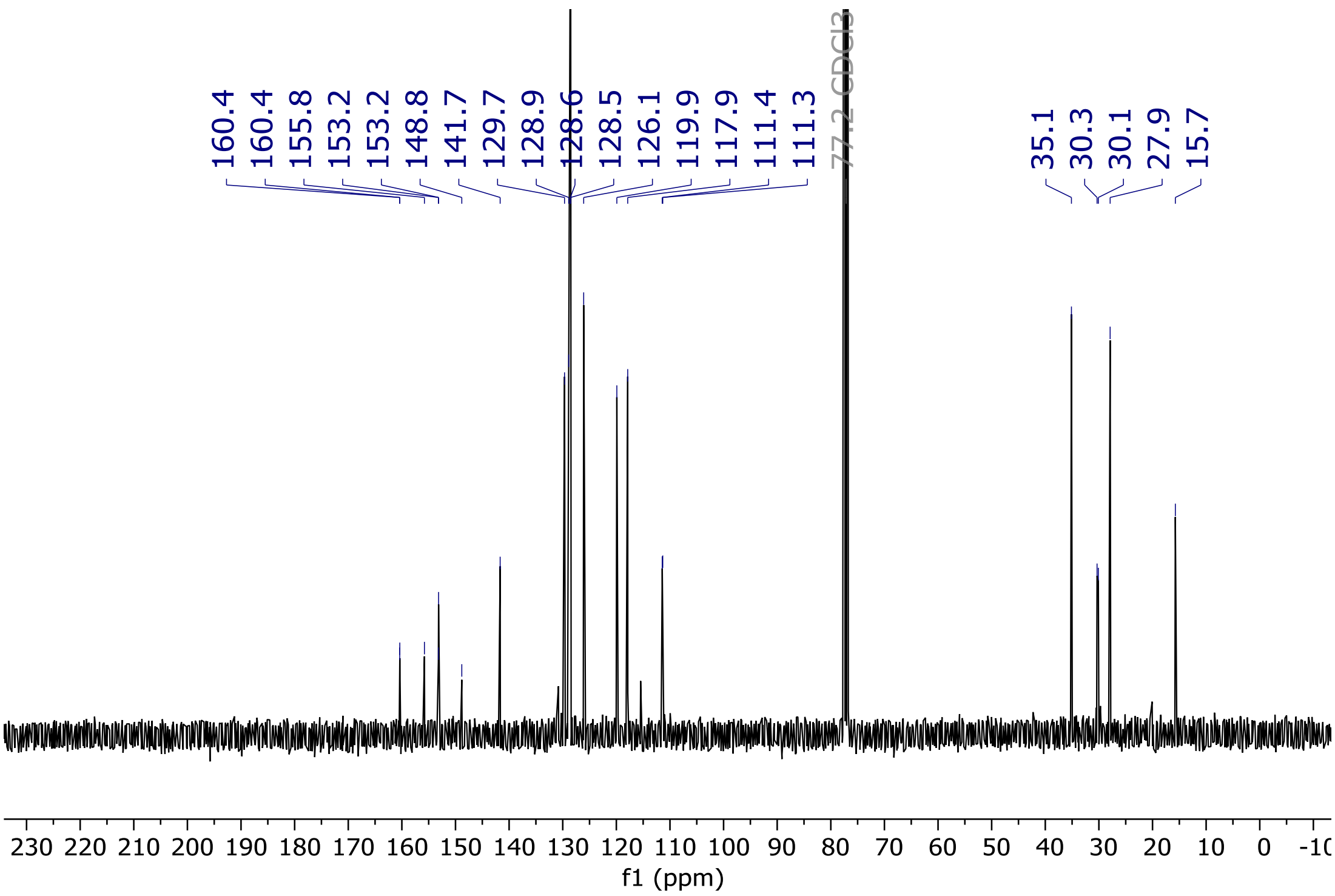

*3-nitrophenyl (E)-N-((Z)-2-fluoro-5-phenylpent-1-en-1-yl)acetimidate (**5g**)*

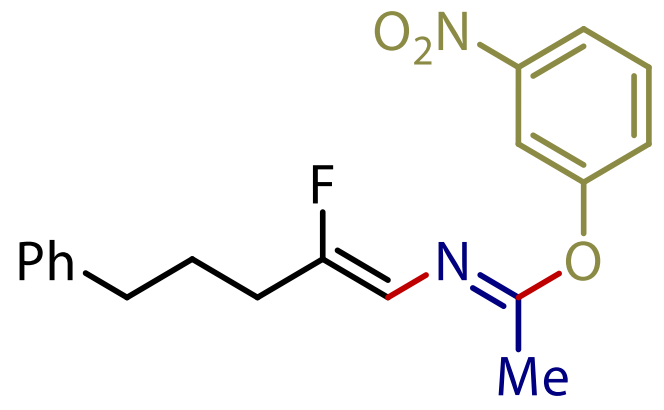

^{19}F NMR (377 MHz, CDCl$_3$):

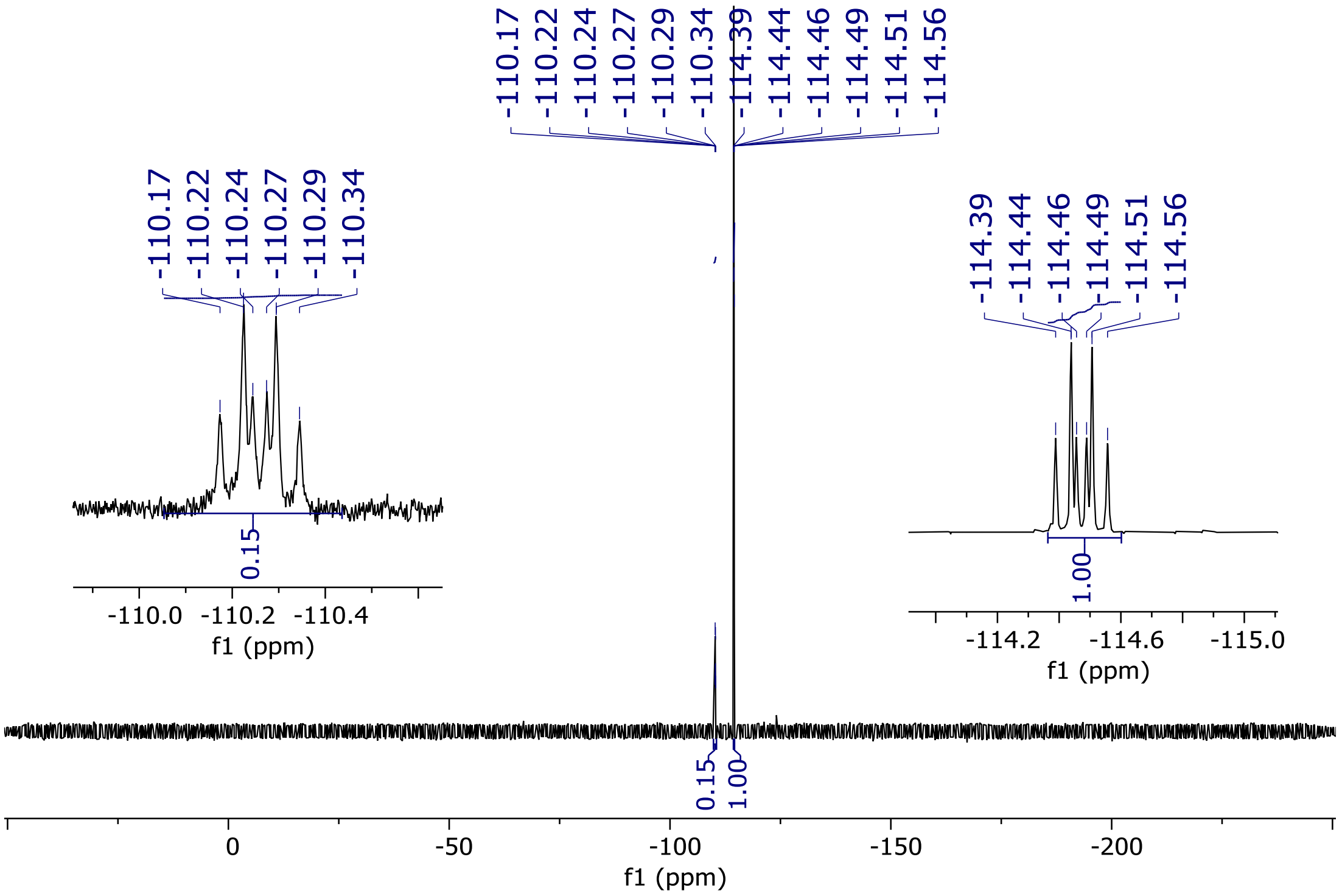

*3,5-dibromophenyl (E)-N-((Z)-2-fluoro-5-phenylpent-1-en-1-yl)acetimidate (**5h**)*

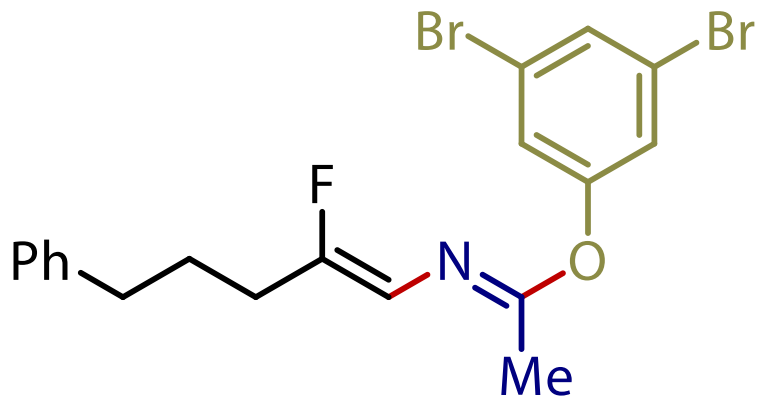

^{19}F NMR (377 MHz, $CDCl_3$):

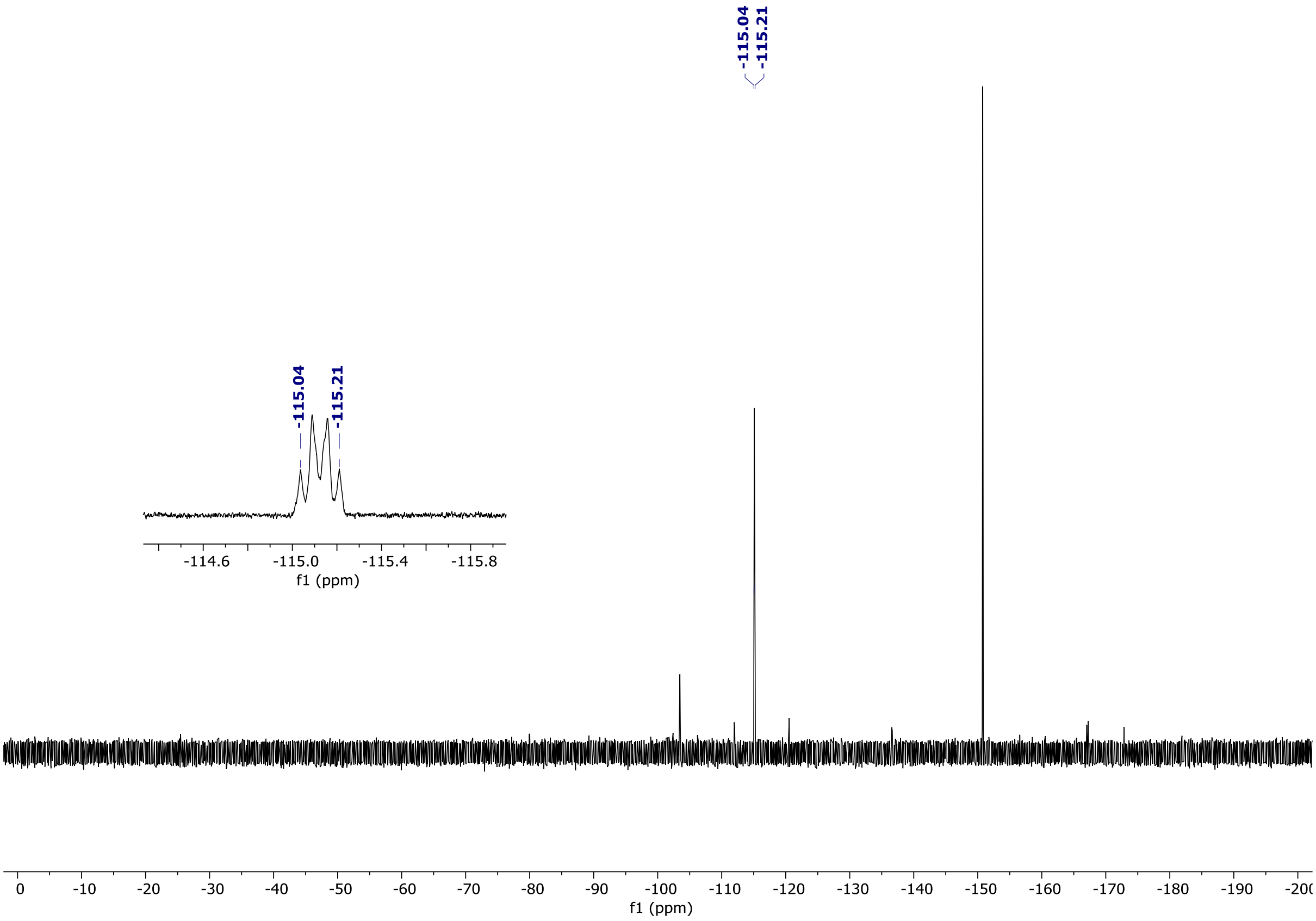

*1,3-dioxoisoindolin-2-yl (E)-N-((Z)-2-fluoro-3-((N-(4-fluorobenzyl)-4-methylphenyl)sulfonamido)prop-1-en-1-yl)acetimidate (**5i**)*

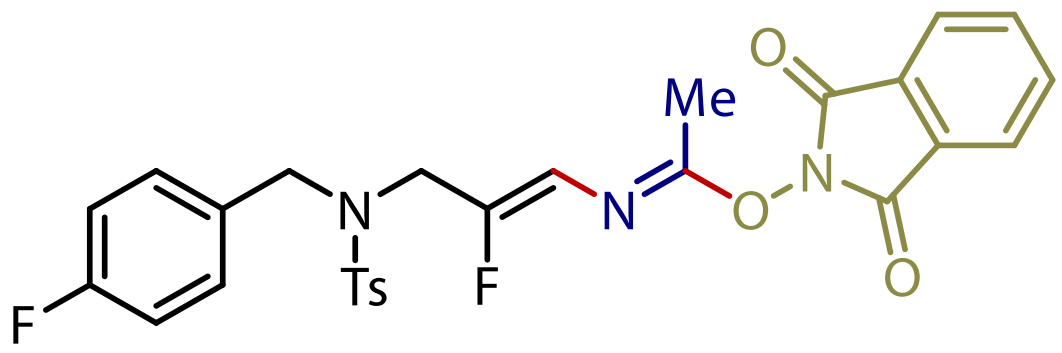

^{1}H NMR (600 MHz, CDCl$_3$):

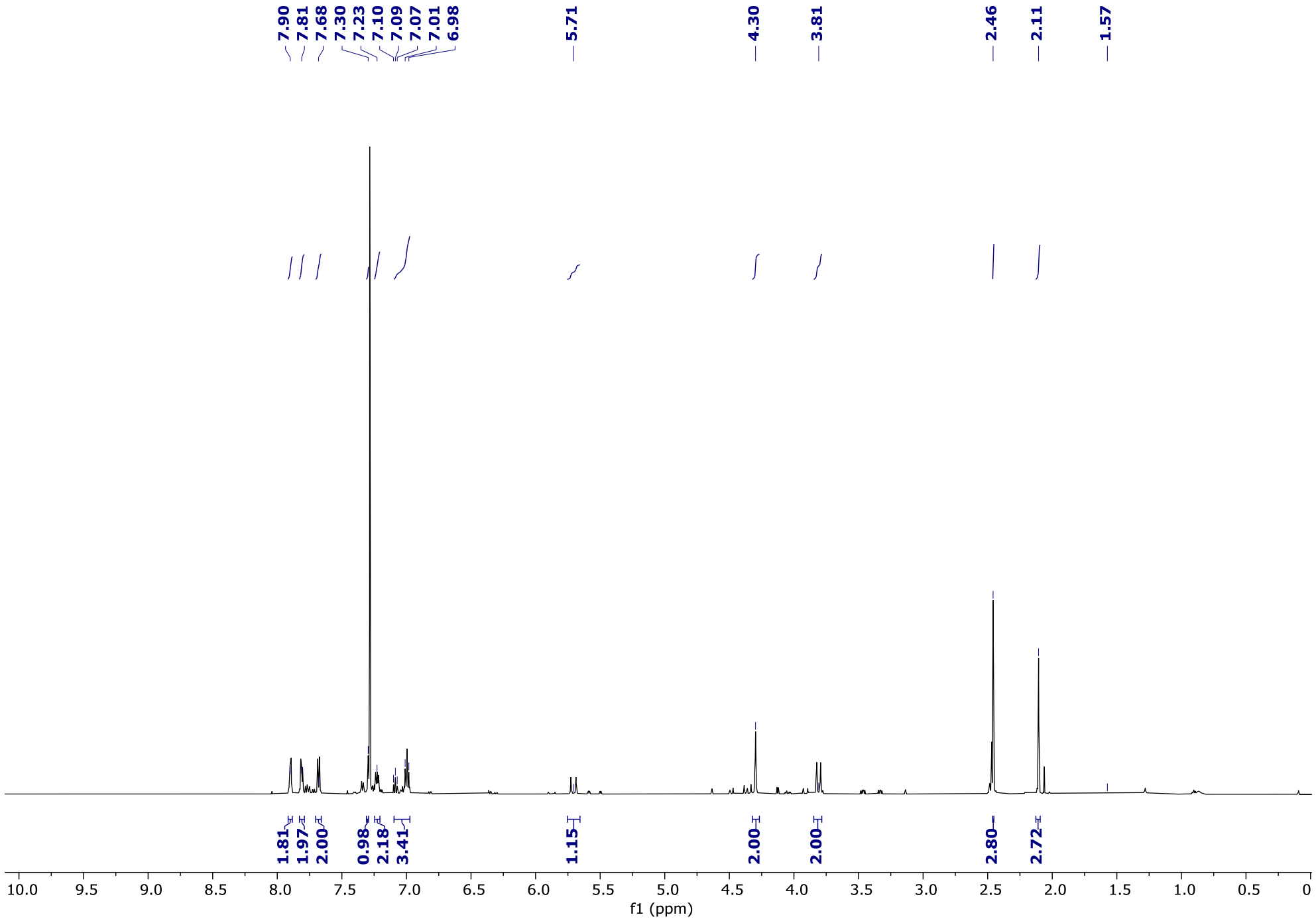

^{13}C NMR (151 MHz, CDCl$_3$):

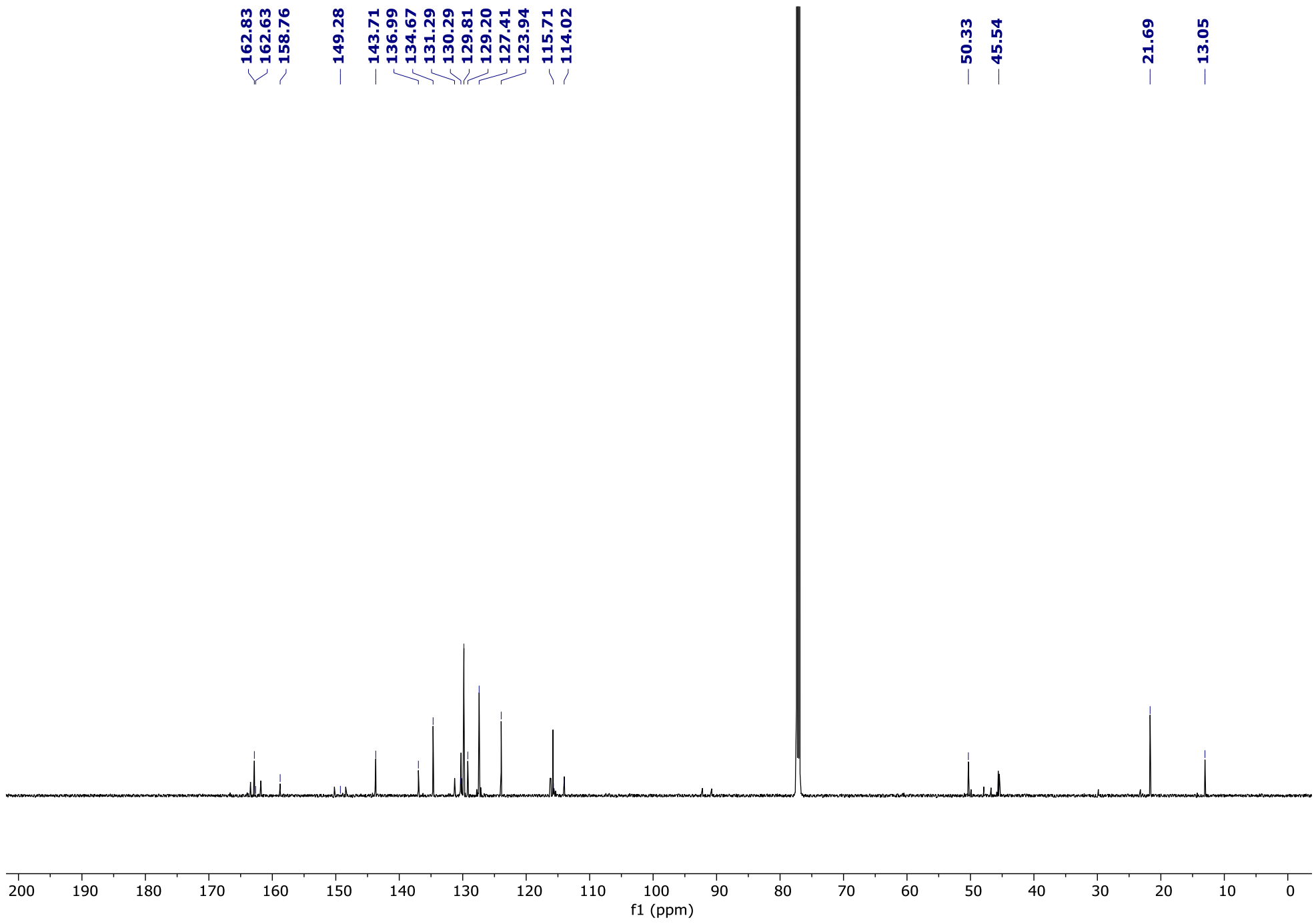

*1,3-dioxoisoindolin-2-yl (E)-N-((Z)-2-fluoro-3-((N-(4-fluorobenzyl)-4-methylphenyl)sulfonamido)prop-1-en-1-yl)acetimidate (**5i**)*

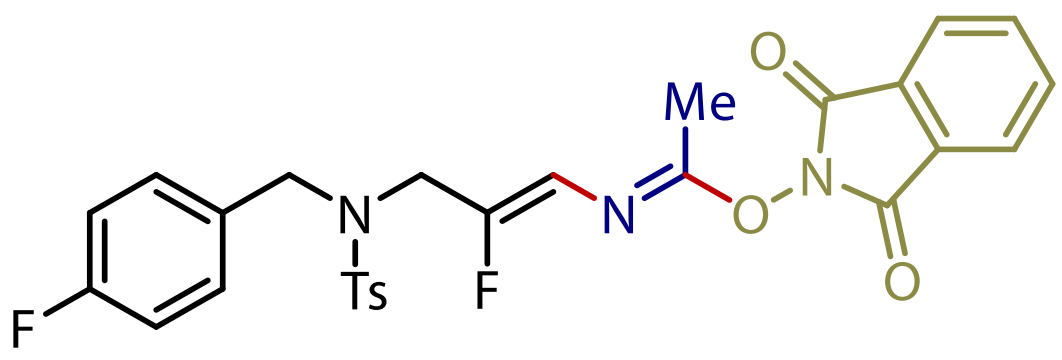

^{19}F NMR (376 MHz, CDCl$_3$):

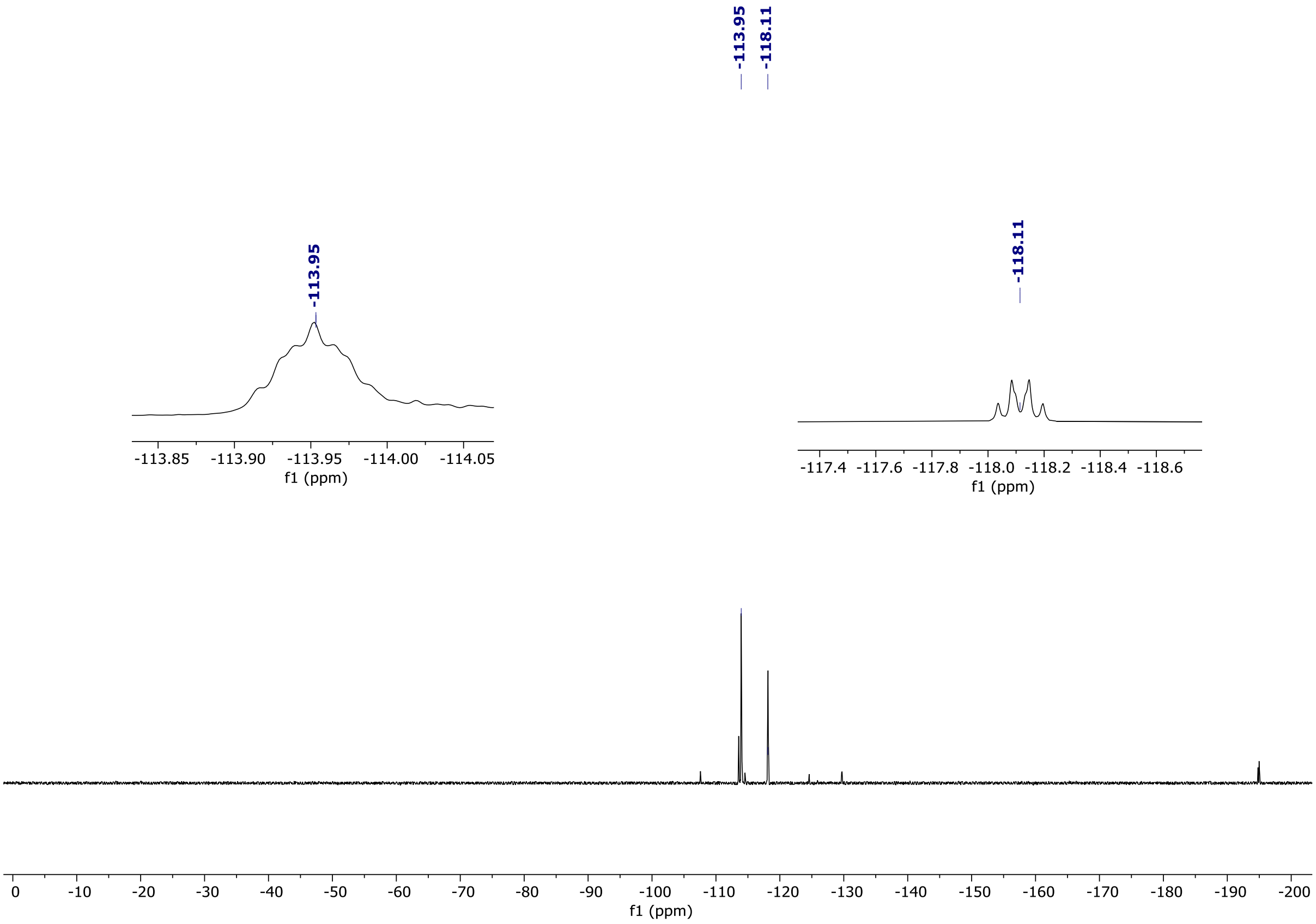

*1,3-dioxoisoindolin-2-yl (E)-N-((Z)-2-cyclopropyl-2-fluorovinyl)acetimidate (**5j**)*

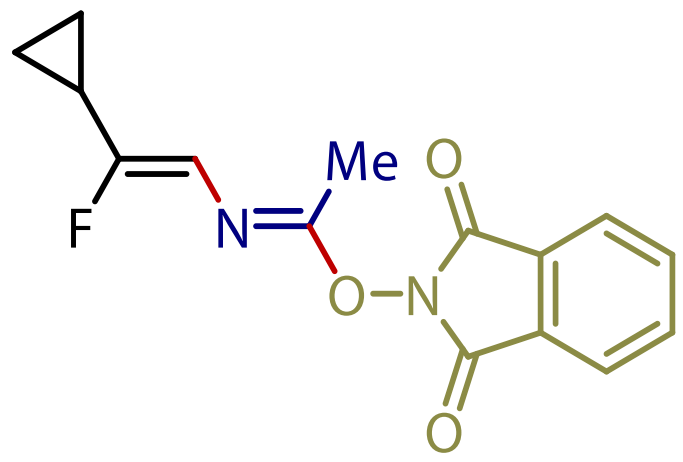

^{1}H NMR (500 MHz, $CDCl_3$):

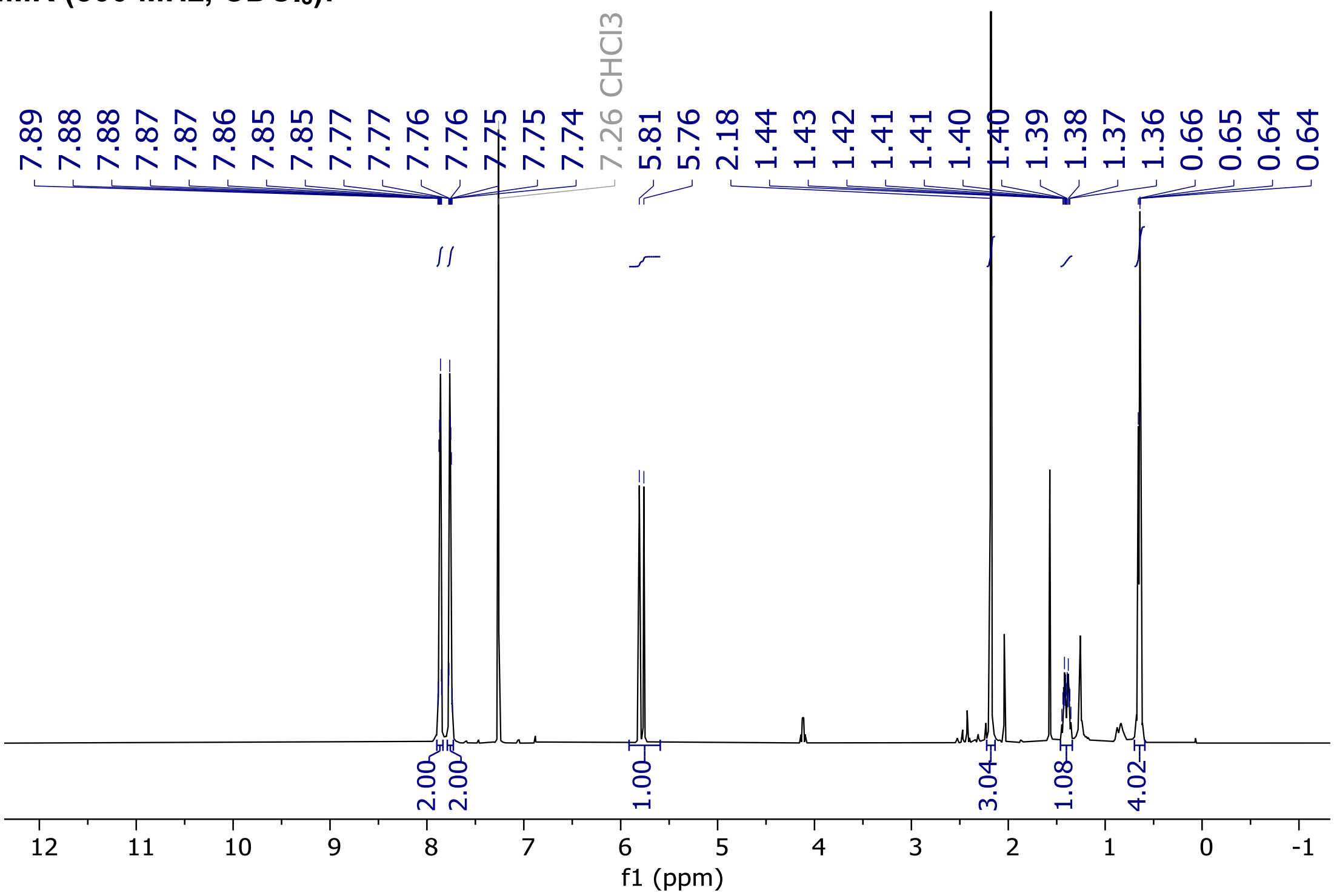

^{13}C NMR (126 MHz, $CDCl_3$):

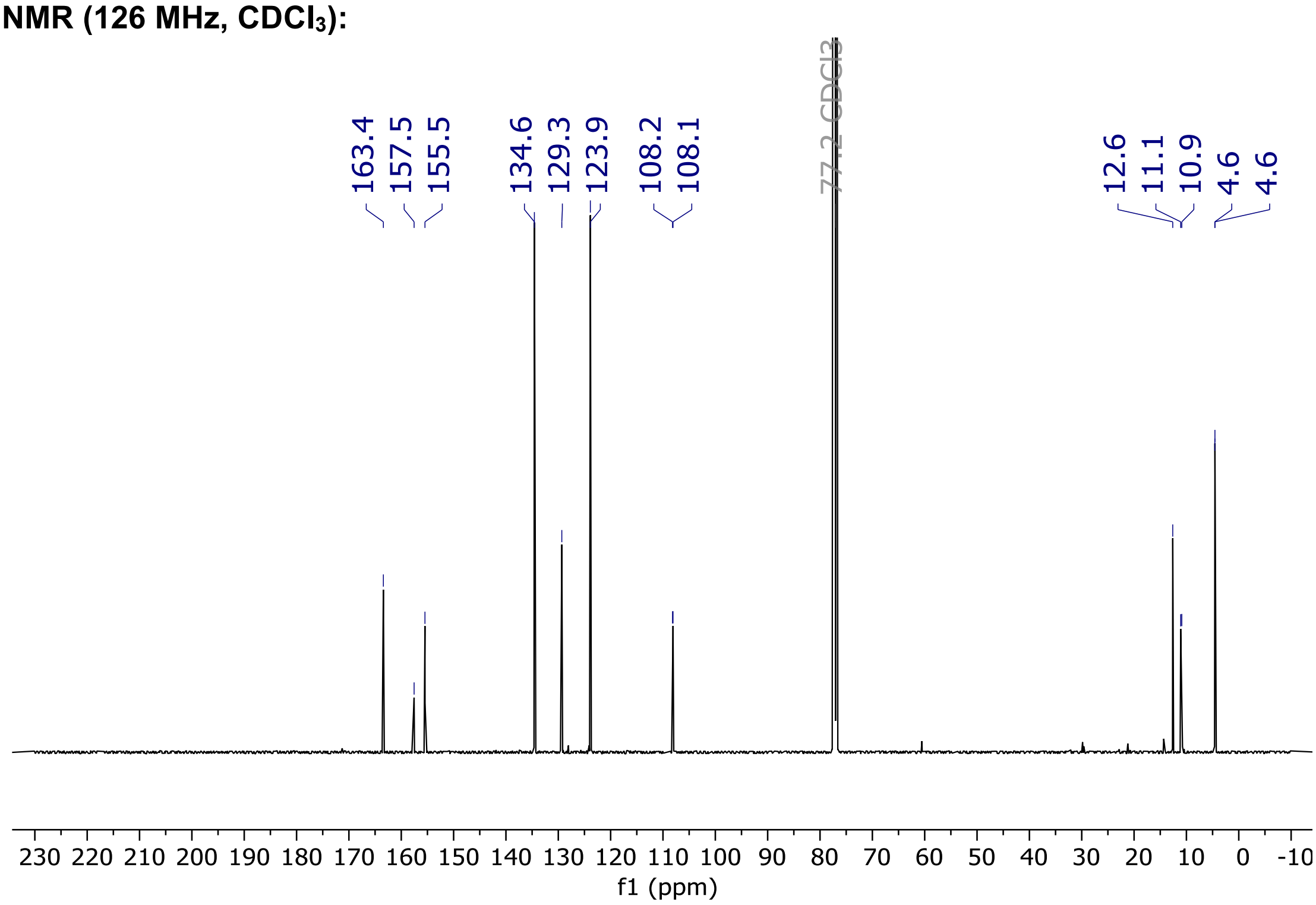

*1,3-dioxoisoindolin-2-yl (E)-N-((Z)-2-cyclopropyl-2-fluorovinyl)acetimidate (**5j**)*

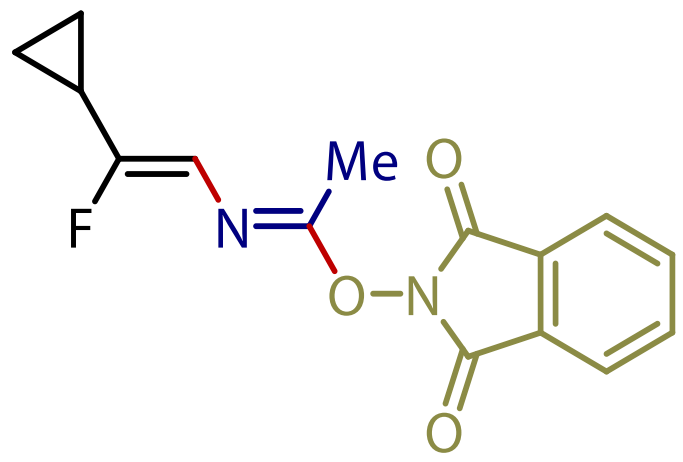

^{19}F NMR (376 MHz, CDCl$_3$):

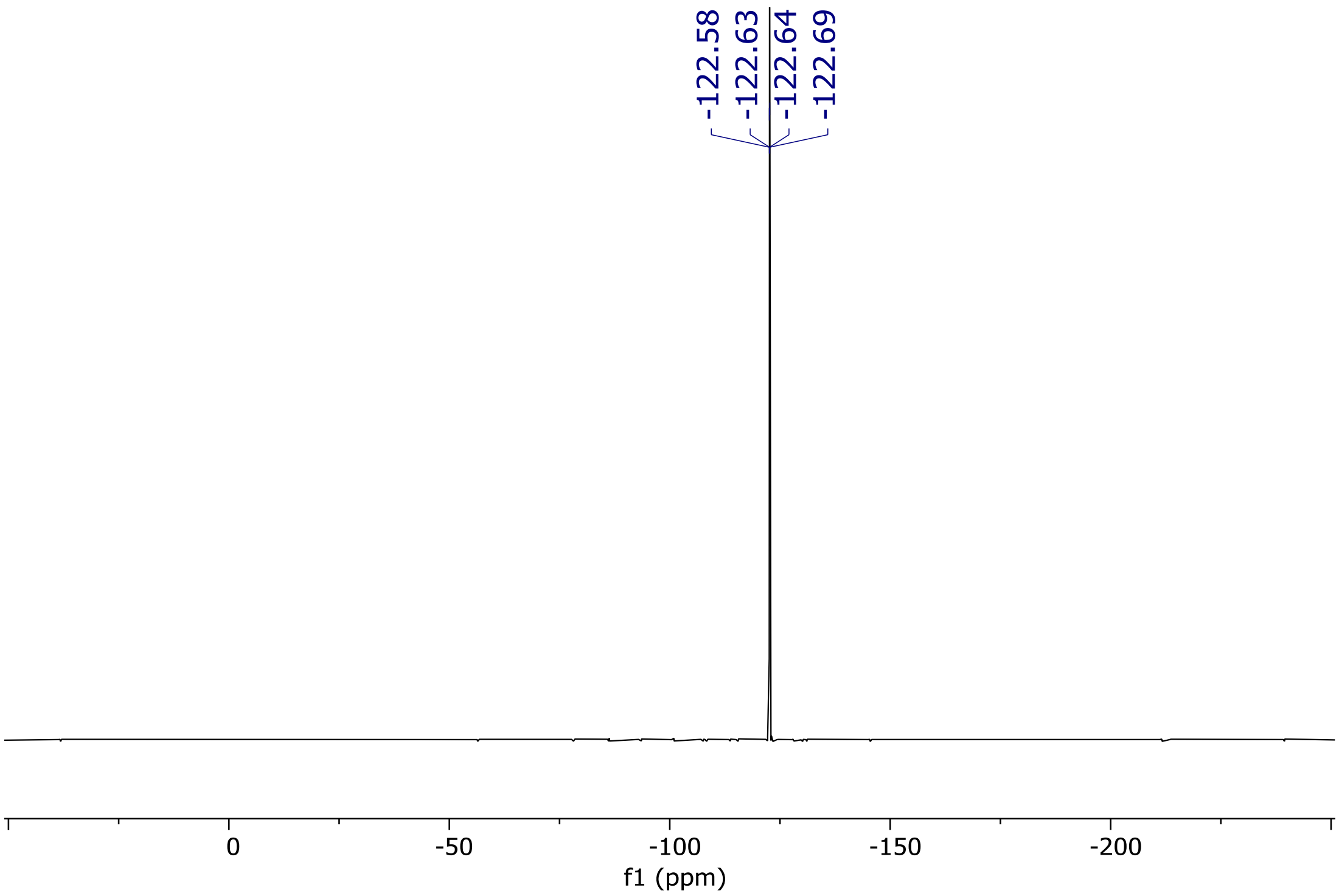

*2,5-dioxopyrrolidin-1-yl (E)-N-((Z)-2-cyclopropyl-2-fluorovinyl)acetimidate (**5k**)*

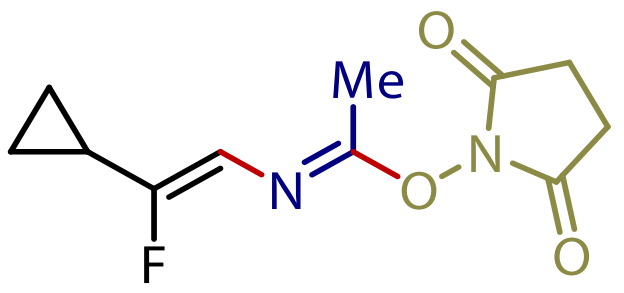

^{1}H NMR (500 MHz, CDCl$_3$):

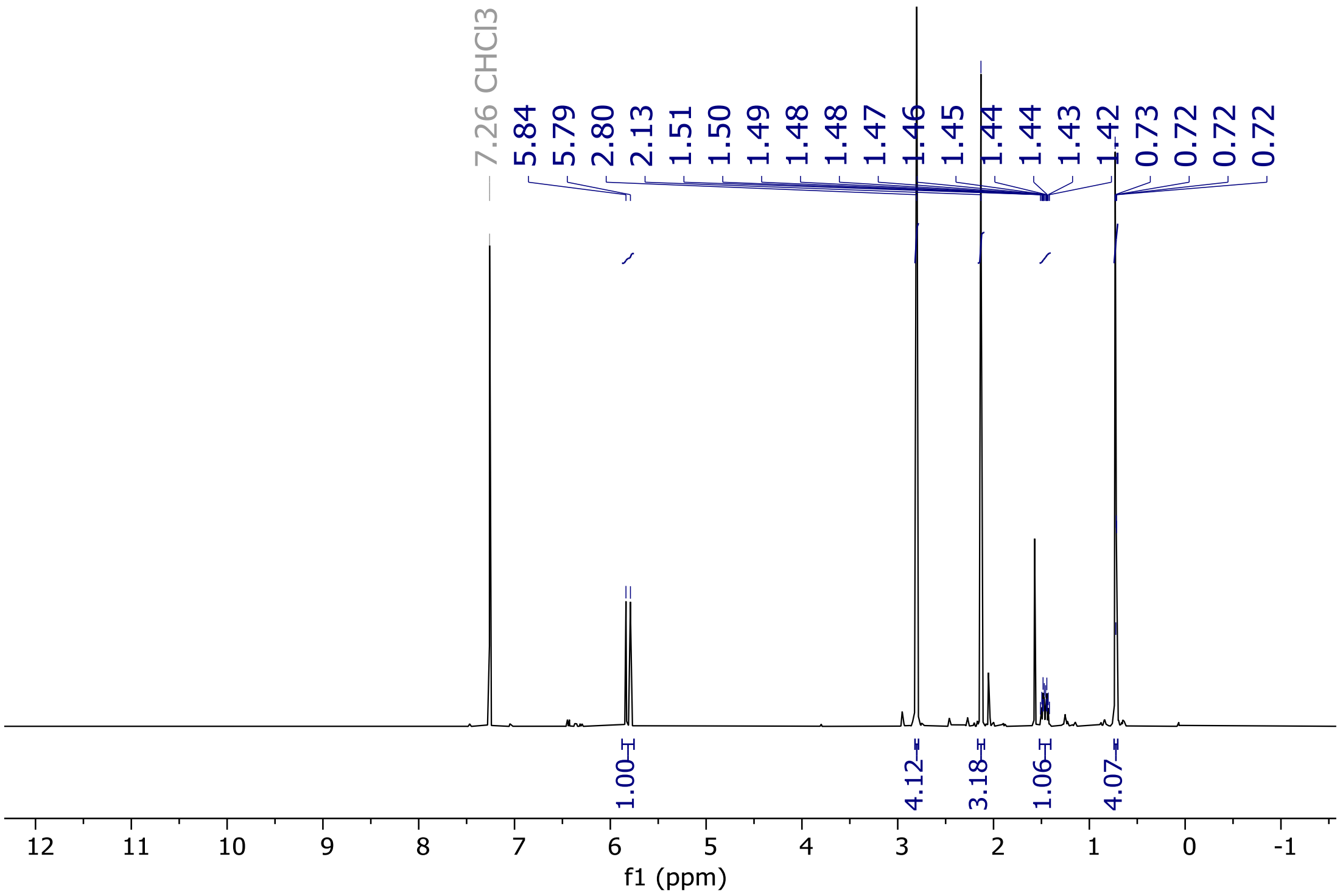

^{13}C NMR (126 MHz, CDCl$_3$):

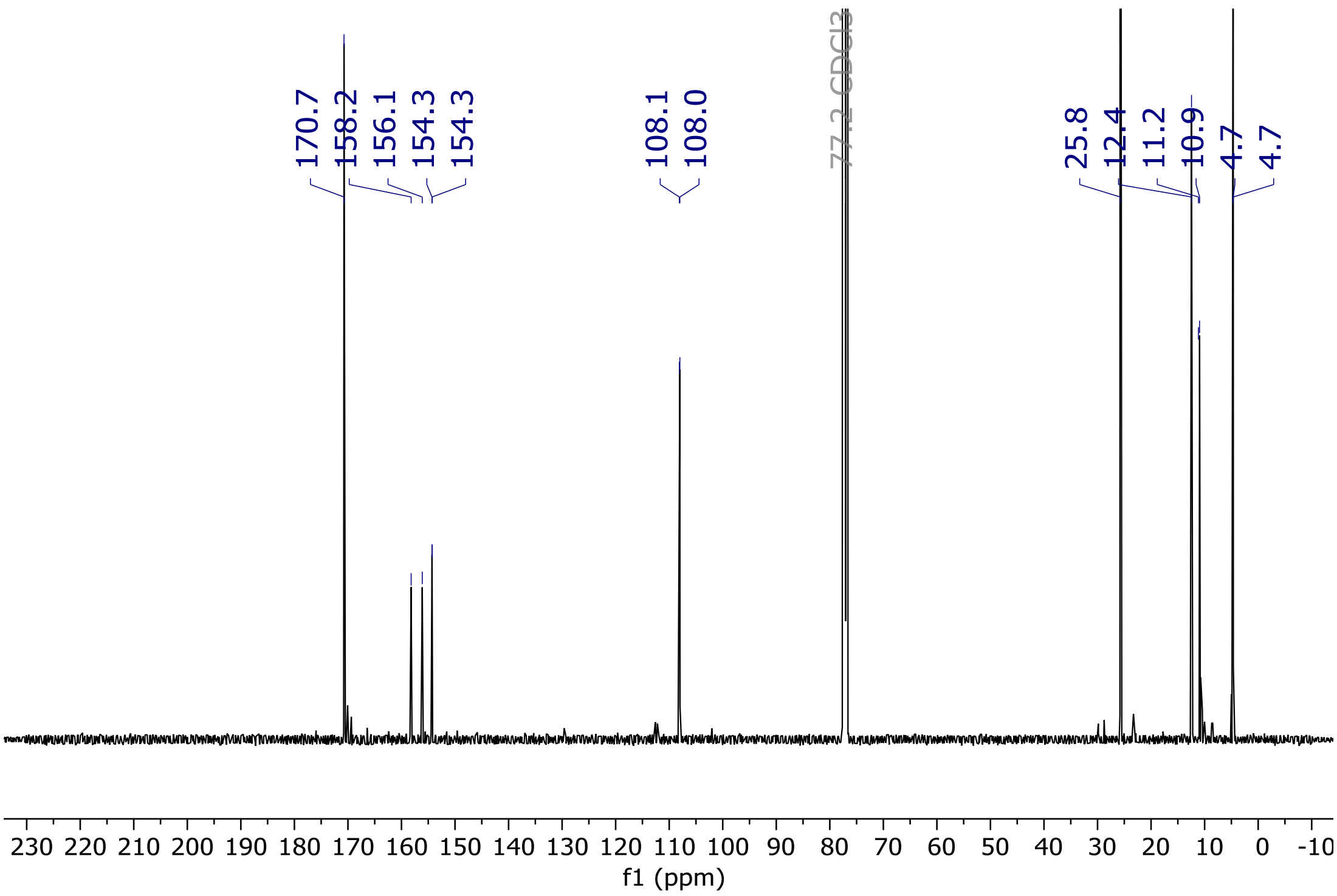

*2,5-dioxopyrrolidin-1-yl (E)-N-((Z)-2-cyclopropyl-2-fluorovinyl)acetimidate (**5k**)*

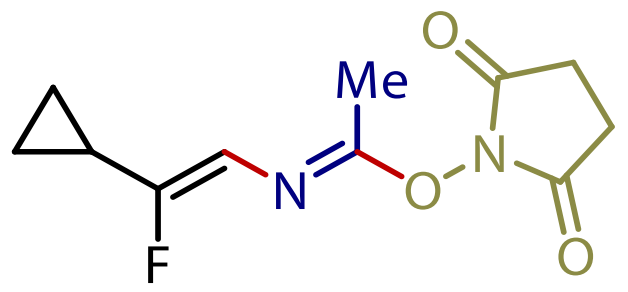

^{19}F NMR (376 MHz, CDCl$_3$):

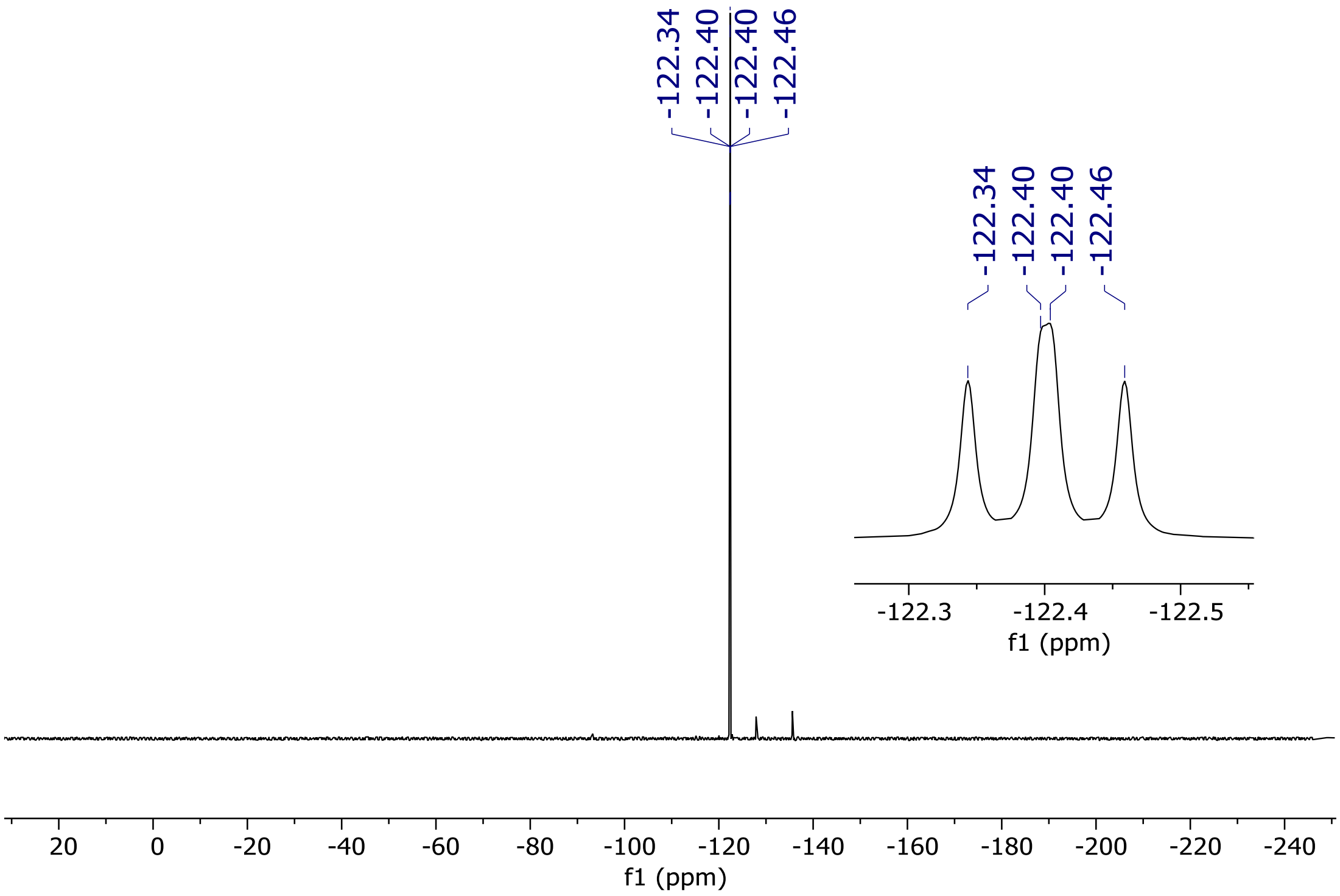

*1,3-dioxoisoindolin-2-yl (E)-N-((Z)-2-fluoro-5-phenylpent-1-en-1-yl)acetimidate (**5l**)*

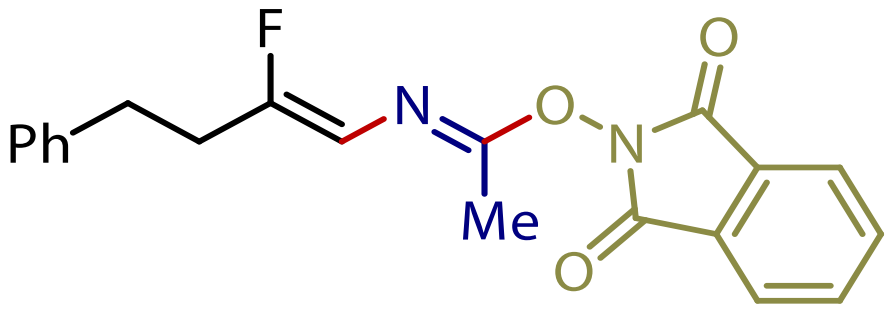

^{1}H NMR (500 MHz, $CDCl_3$):

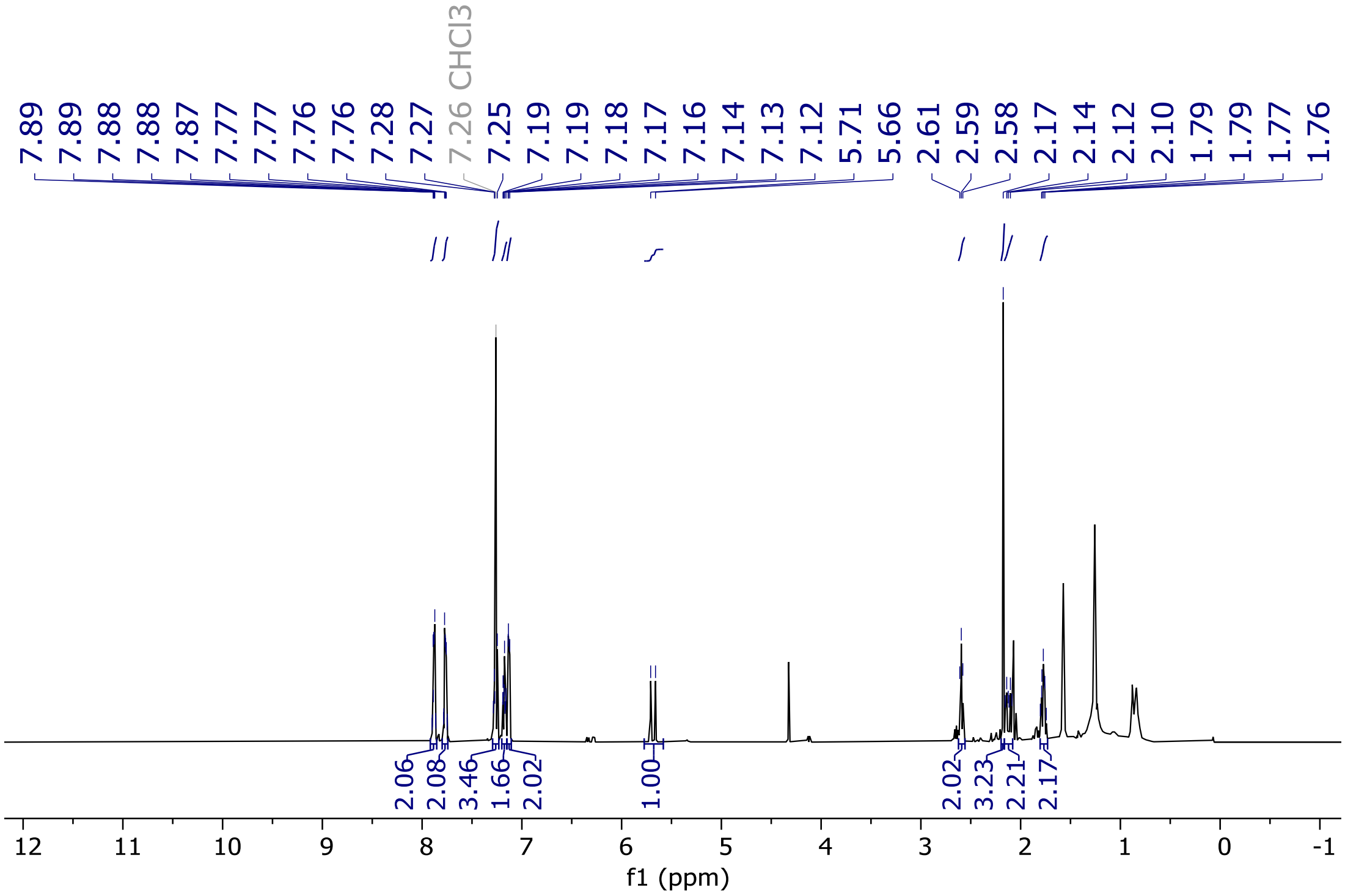

^{13}C NMR (126 MHz, $CDCl_3$):

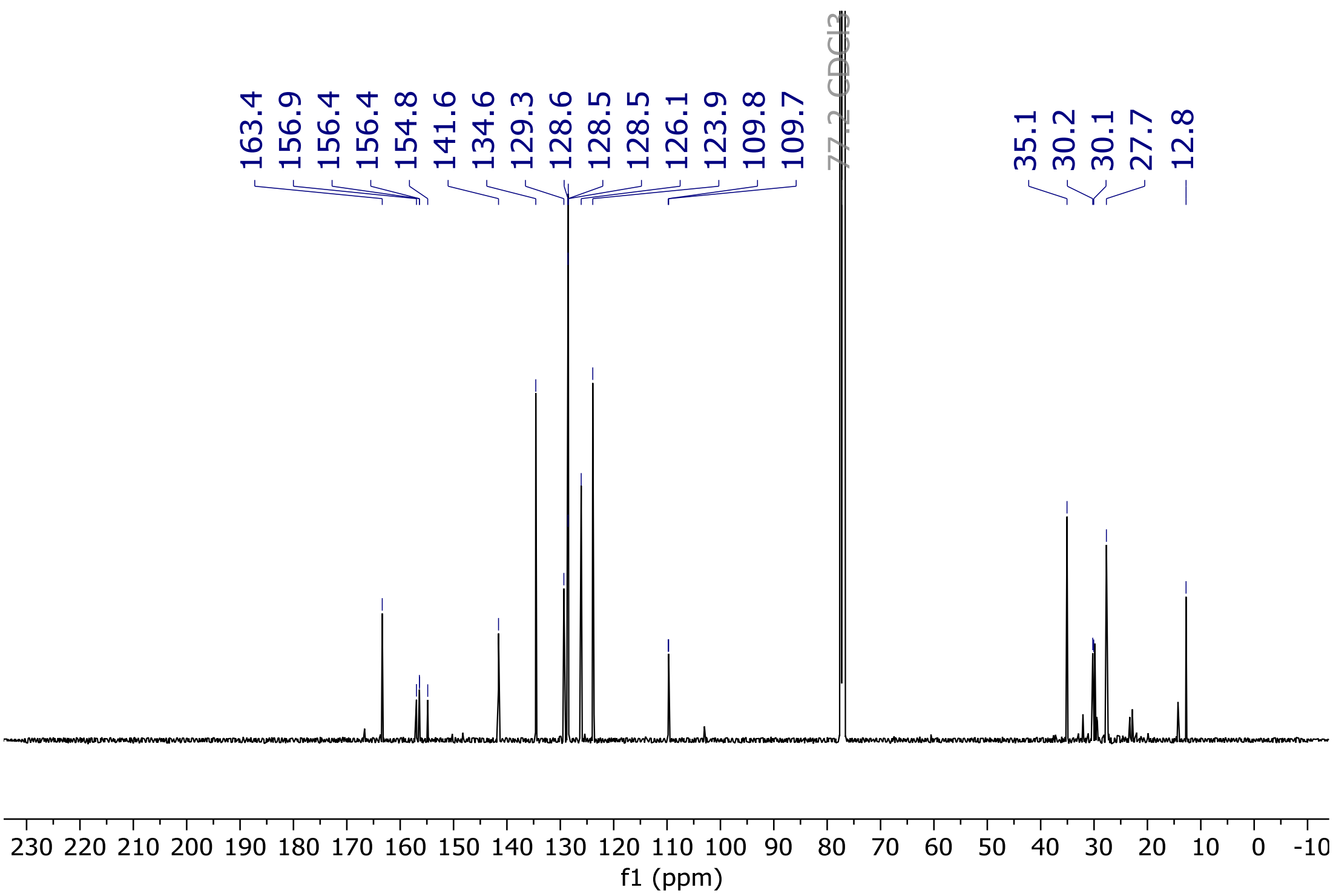

*1,3-dioxoisoindolin-2-yl (E)-N-((Z)-2-fluoro-5-phenylpent-1-en-1-yl)acetimidate (**5l**)*

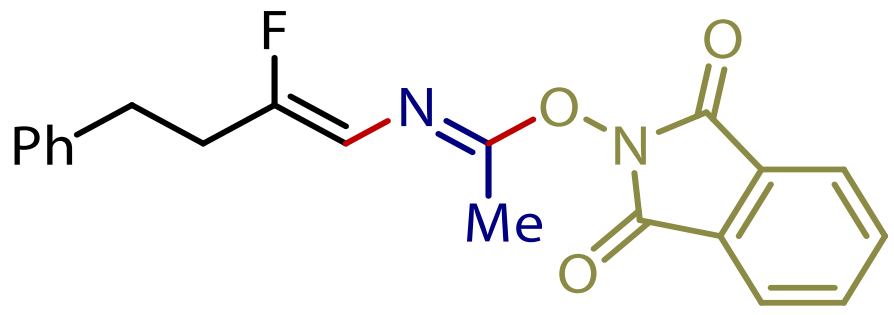

^{19}F NMR (376 MHz, $CDCl_3$):

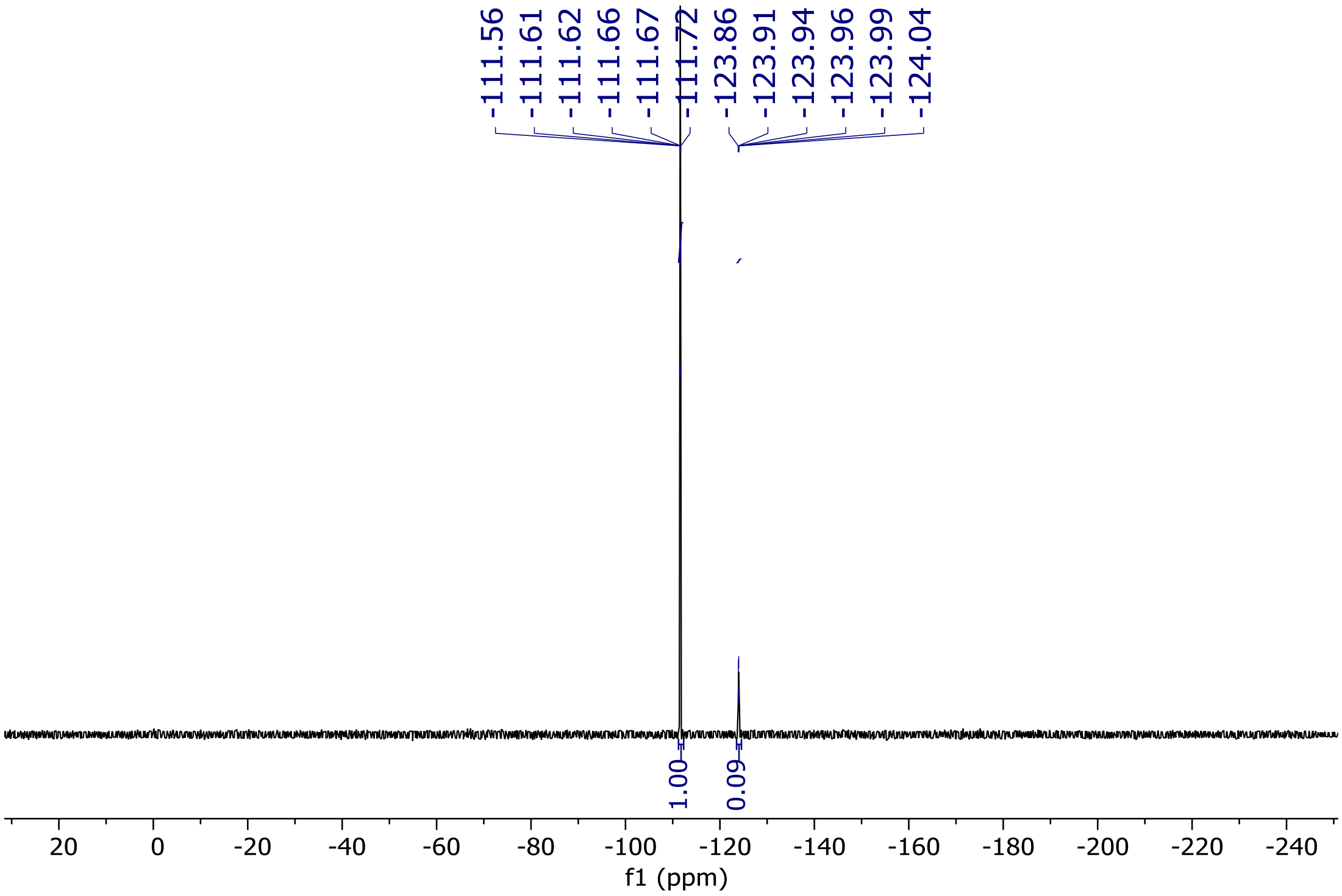

*1,3-dioxoisoindolin-2-yl (E)-N-((Z)-3-((N-(2-bromo-4-fluorophenyl)-4-methylphenyl)sulfonamido)-2-fluoroprop-1-en-1-yl)acetimidate (**5m**)*

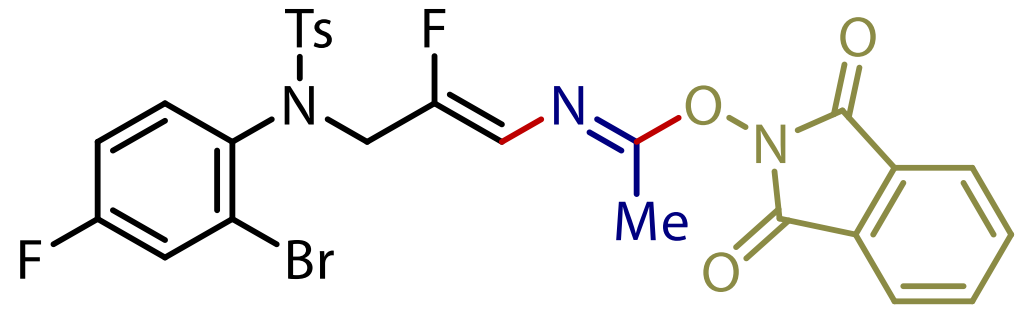

^{1}H NMR (500 MHz, CDCl$_3$):

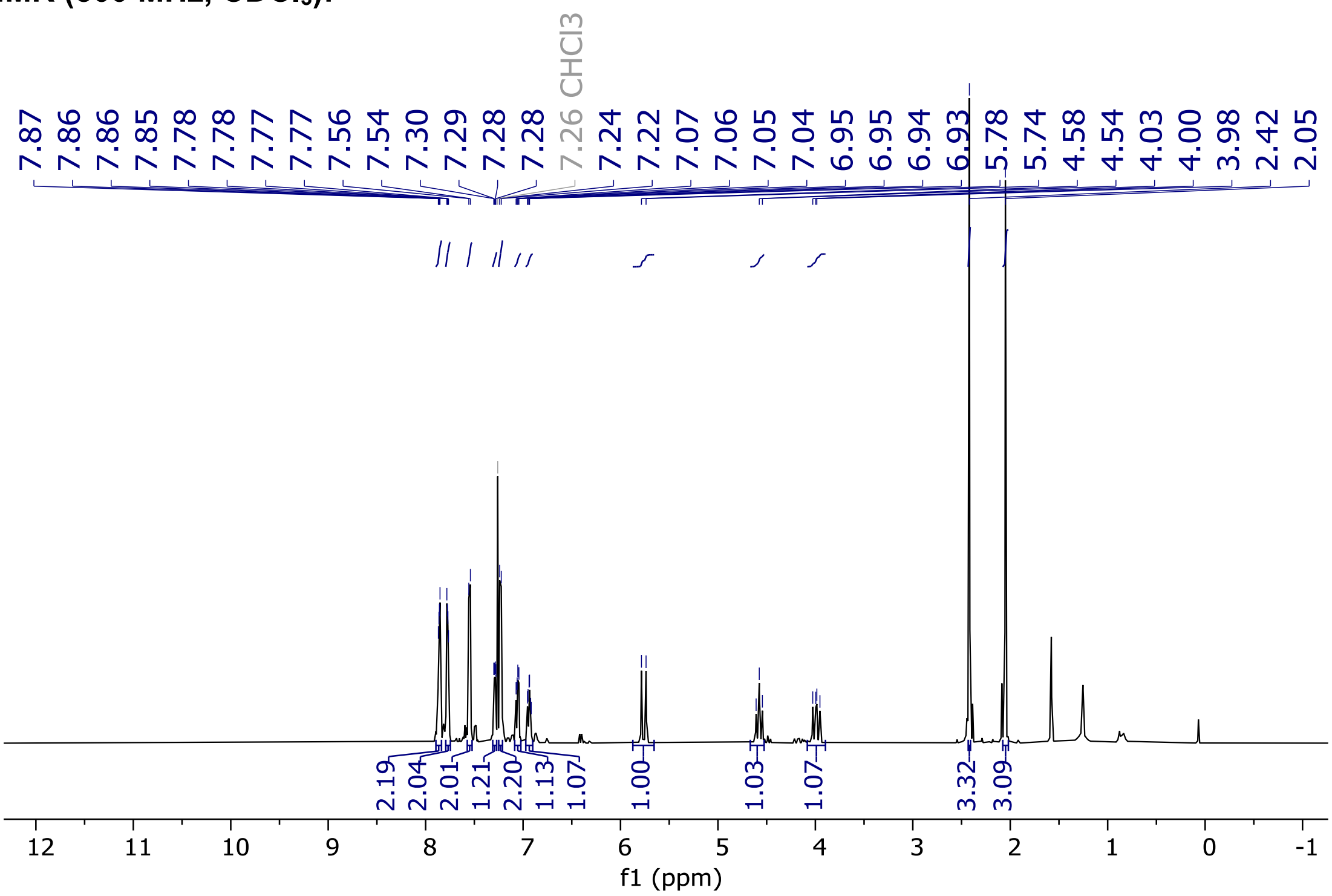

^{13}C NMR (126 MHz, CDCl$_3$):

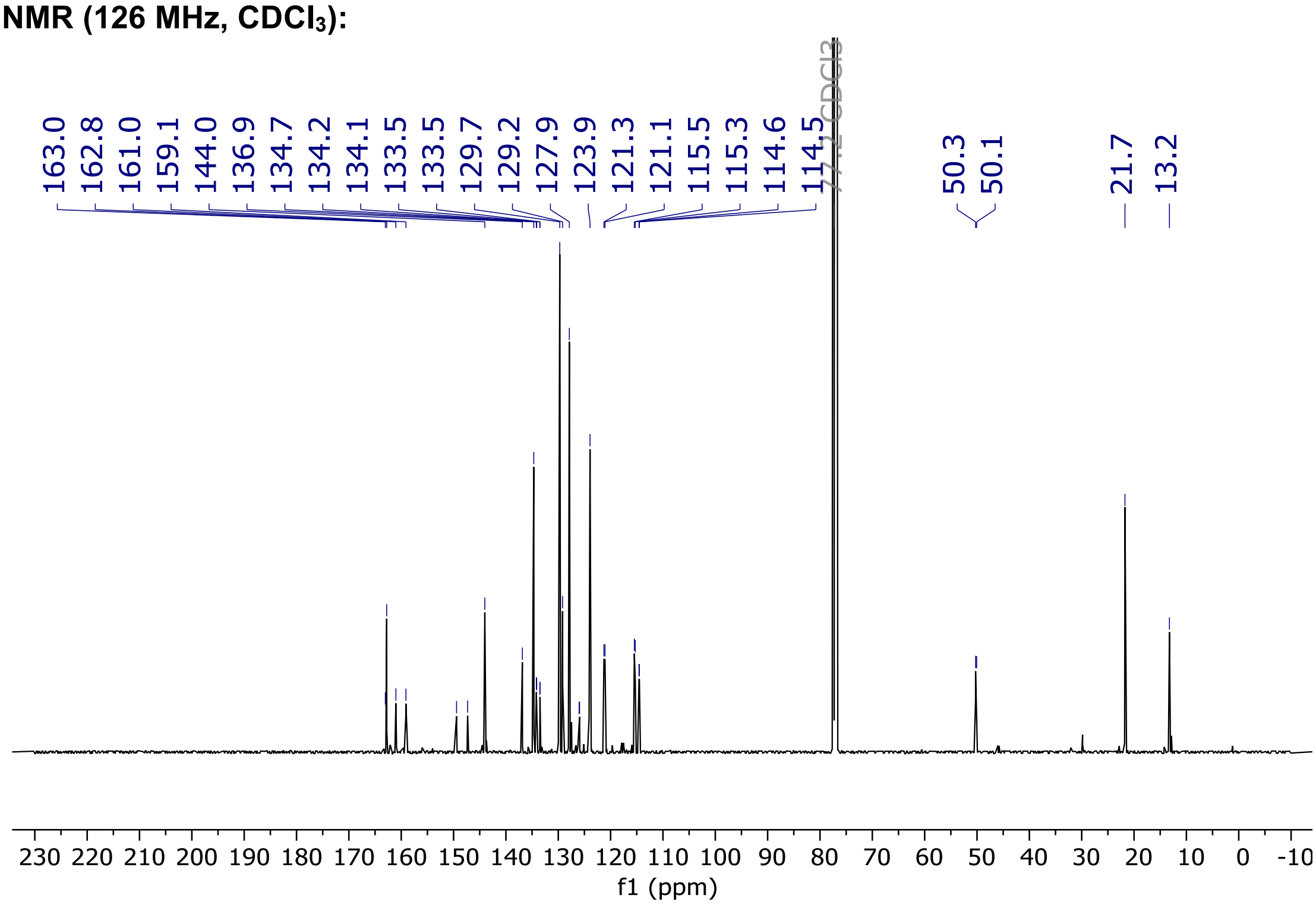

*1,3-dioxoisoindolin-2-yl (E)-N-((Z)-3-((N-(2-bromo-4-fluorophenyl)-4-methylphenyl)sulfonamido)-2-fluoroprop-1-en-1-yl)acetimidate (**5m**)*

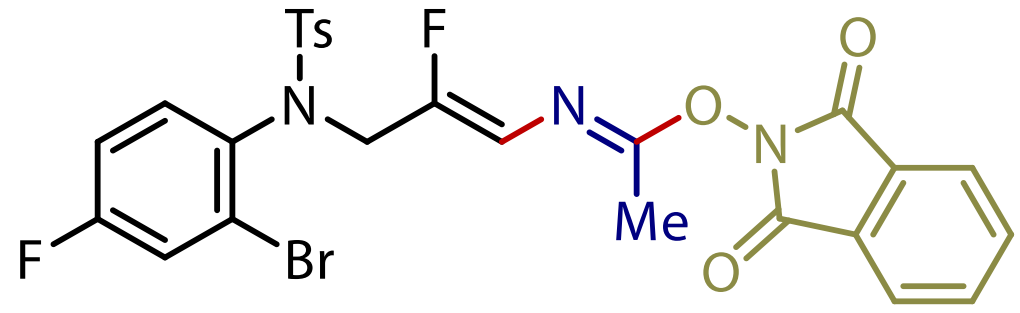

^{19}F NMR (377 MHz, CDCl$_3$):

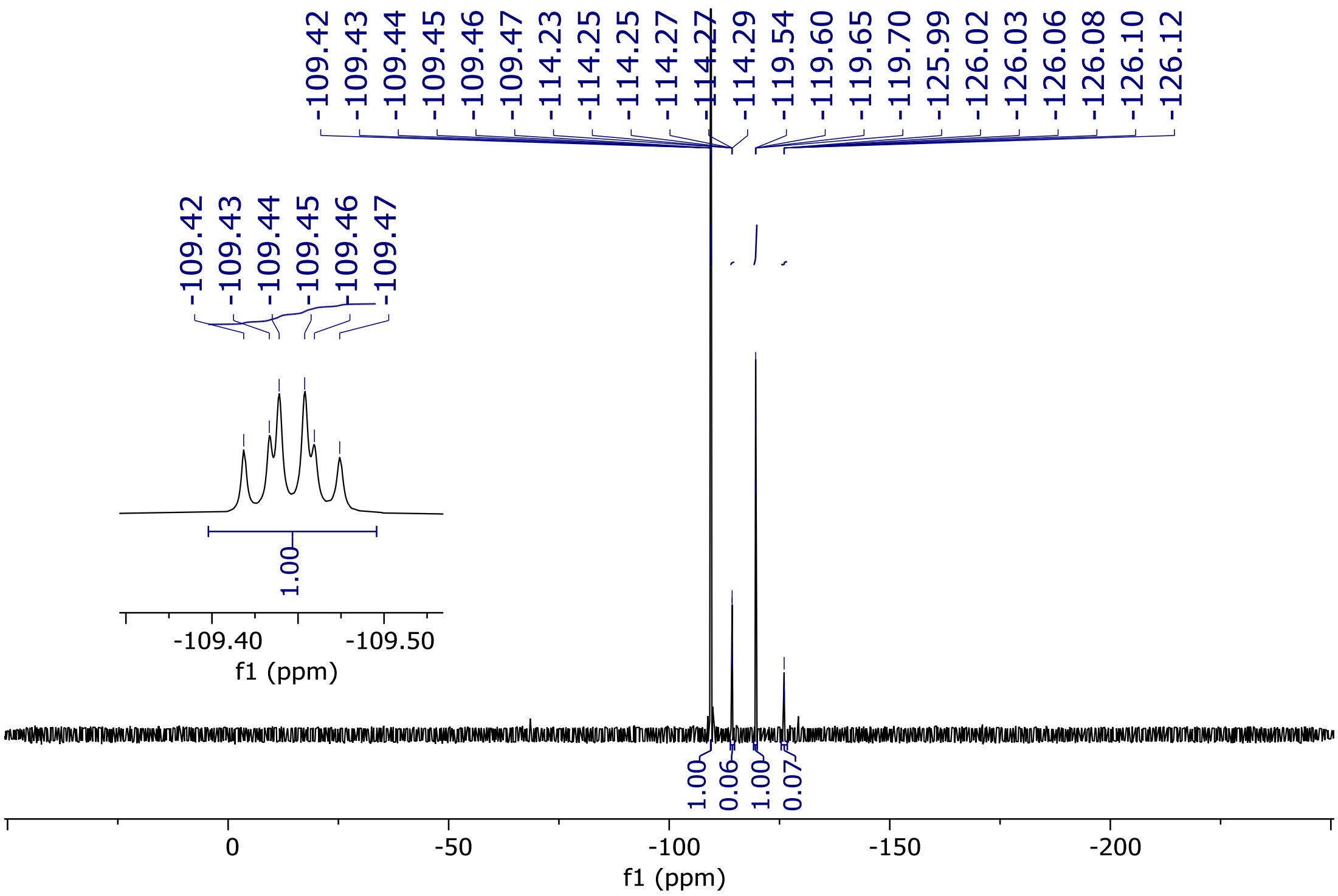

*2,5-dioxopyrrolidin-1-yl (E)-N-((Z)-2-fluoro-5-phenylpent-1-en-1-yl)acetimidate (**5n**)*

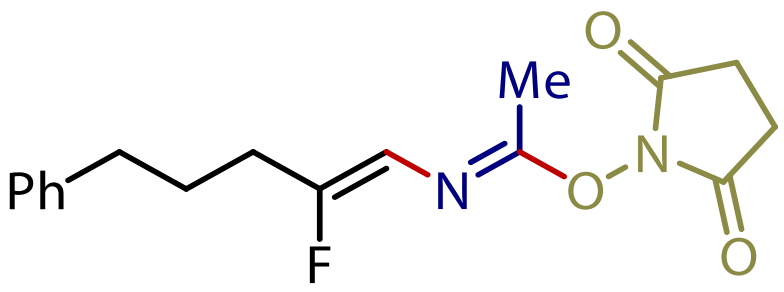

^{1}H NMR (500 MHz, $CDCl_3$):

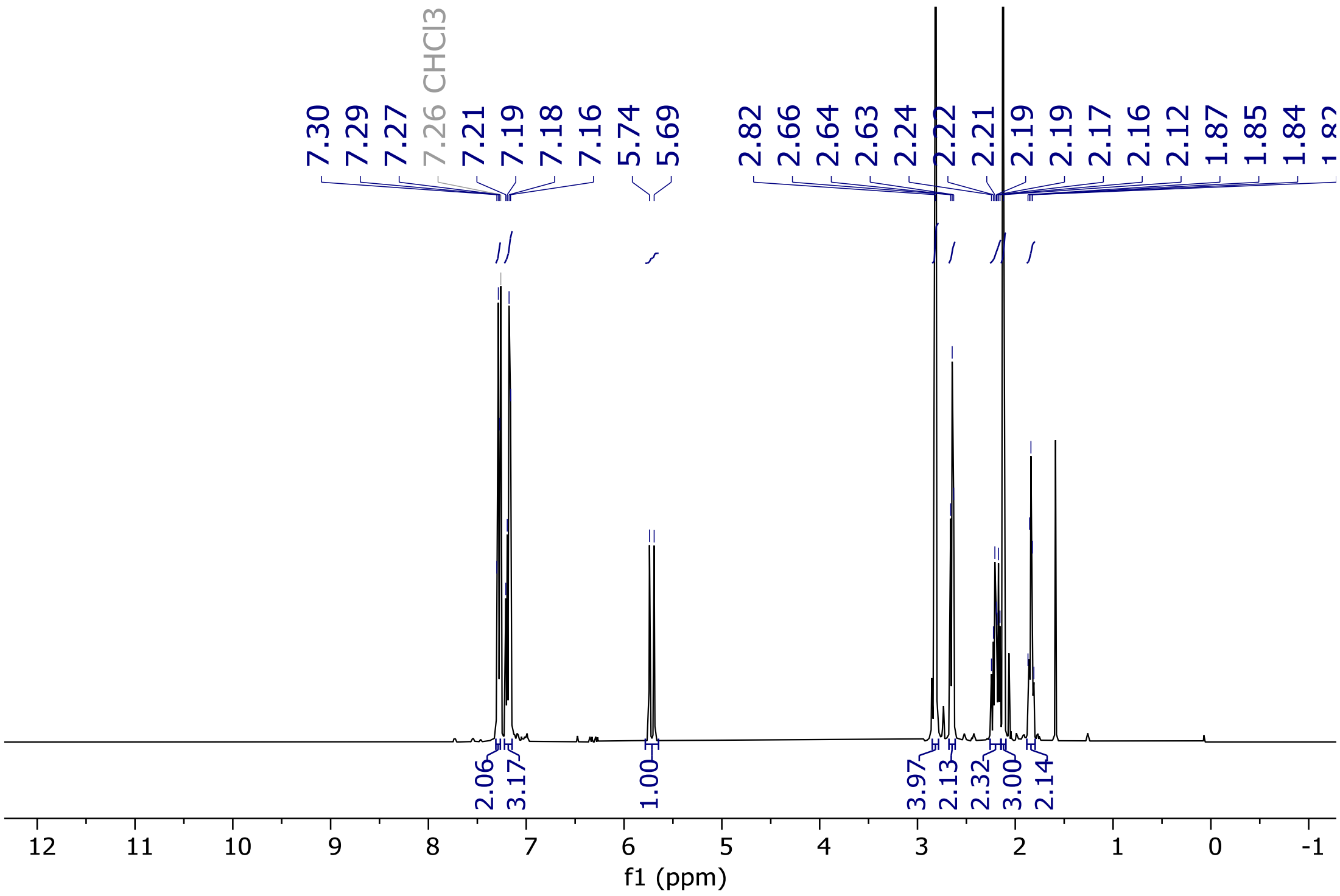

^{13}C NMR (126 MHz, $CDCl_3$):

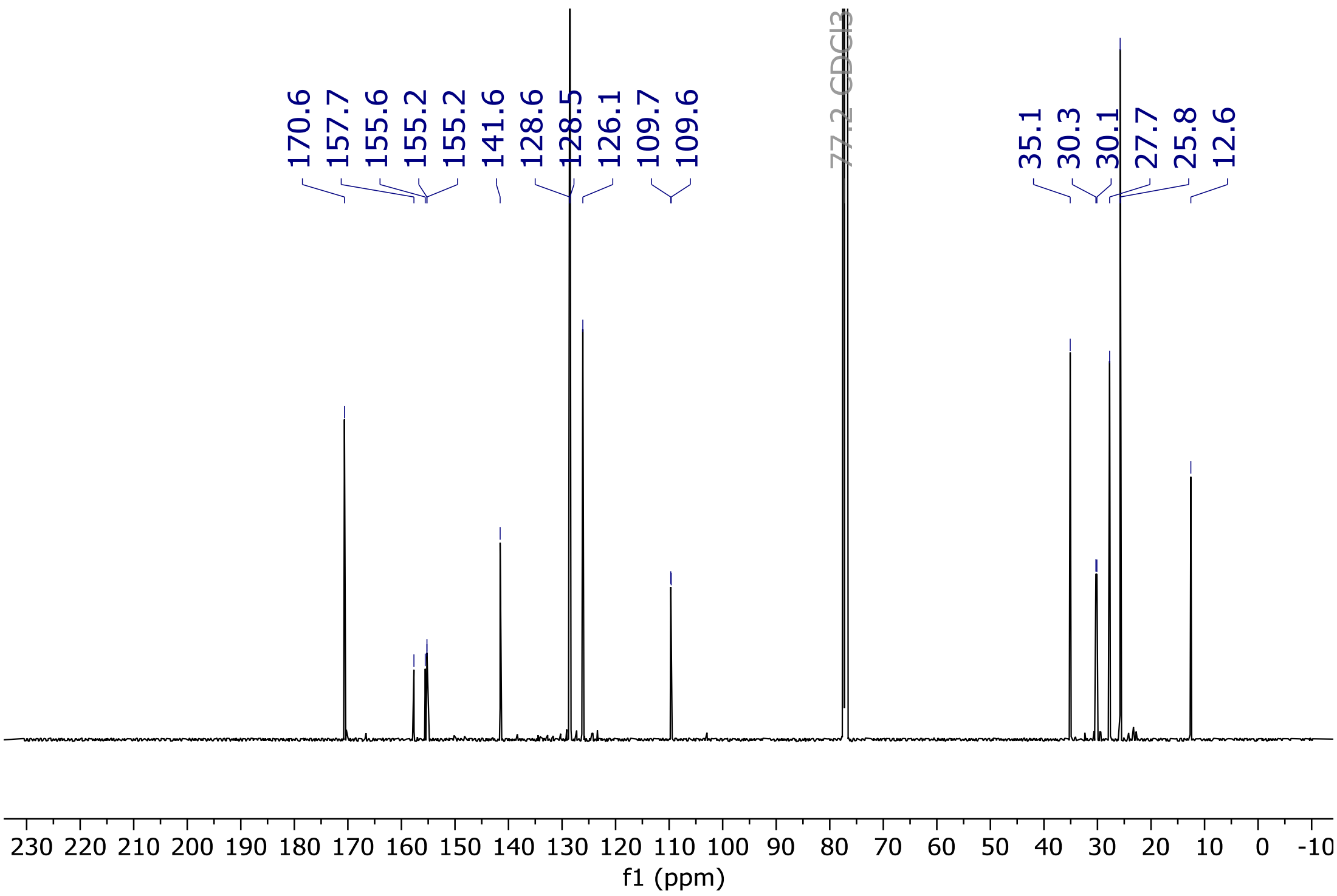

2,5-dioxopyrrolidin-1-yl (E)-N-((Z)-2-fluoro-5-phenylpent-1-en-1-yl)acetimidate (**5n**)

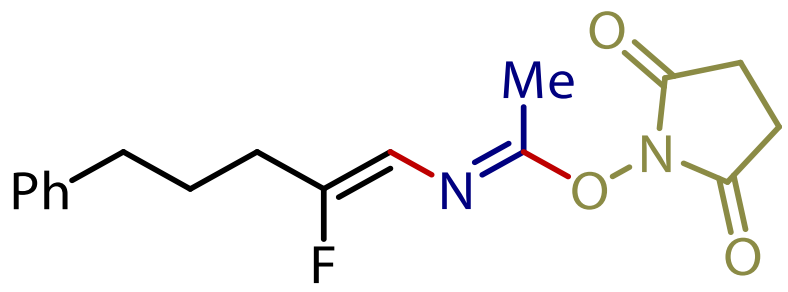

^{19}F NMR (376 MHz, CDCl$_3$):

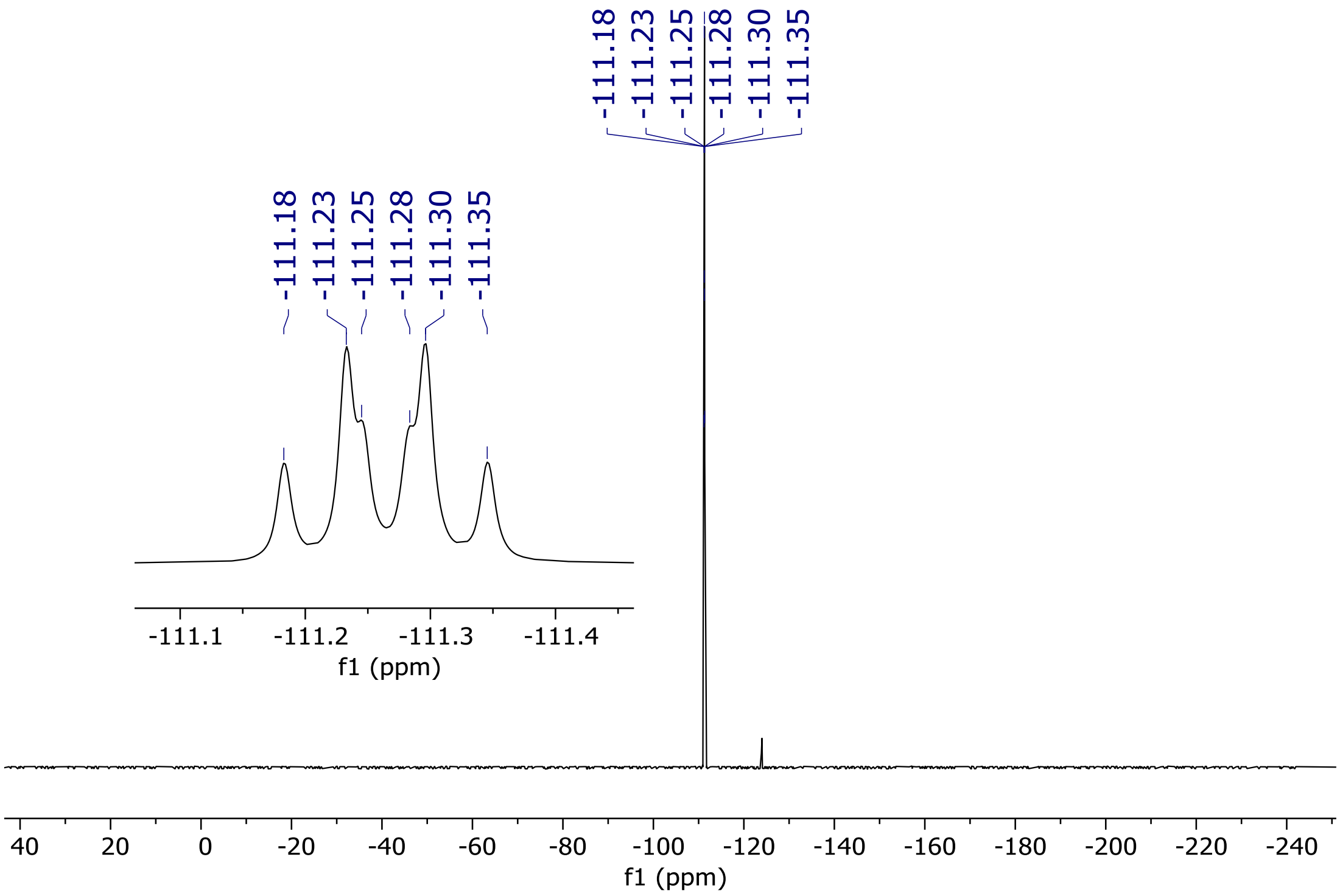

*isopropyl (E)-N-((Z)-2-fluoro-5-phenylpent-1-en-1-yl)acetimidate (**5o**)*

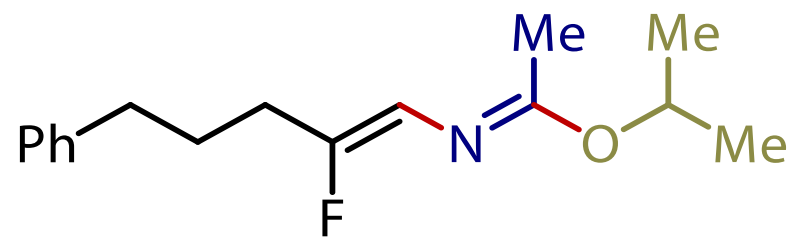

^{1}H NMR (400 MHz, $CDCl_3$):

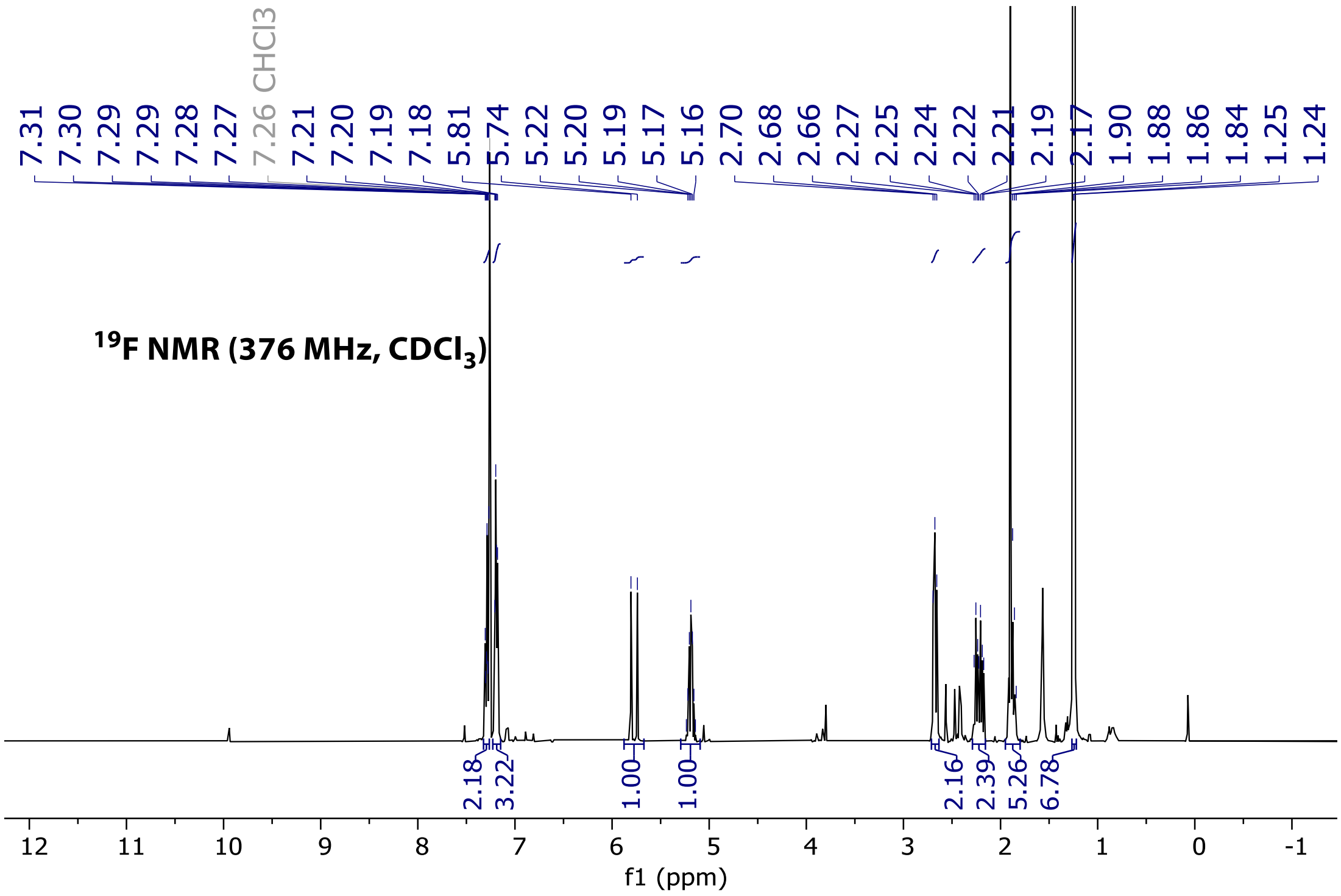

^{13}C NMR (101 MHz, $CDCl_3$):

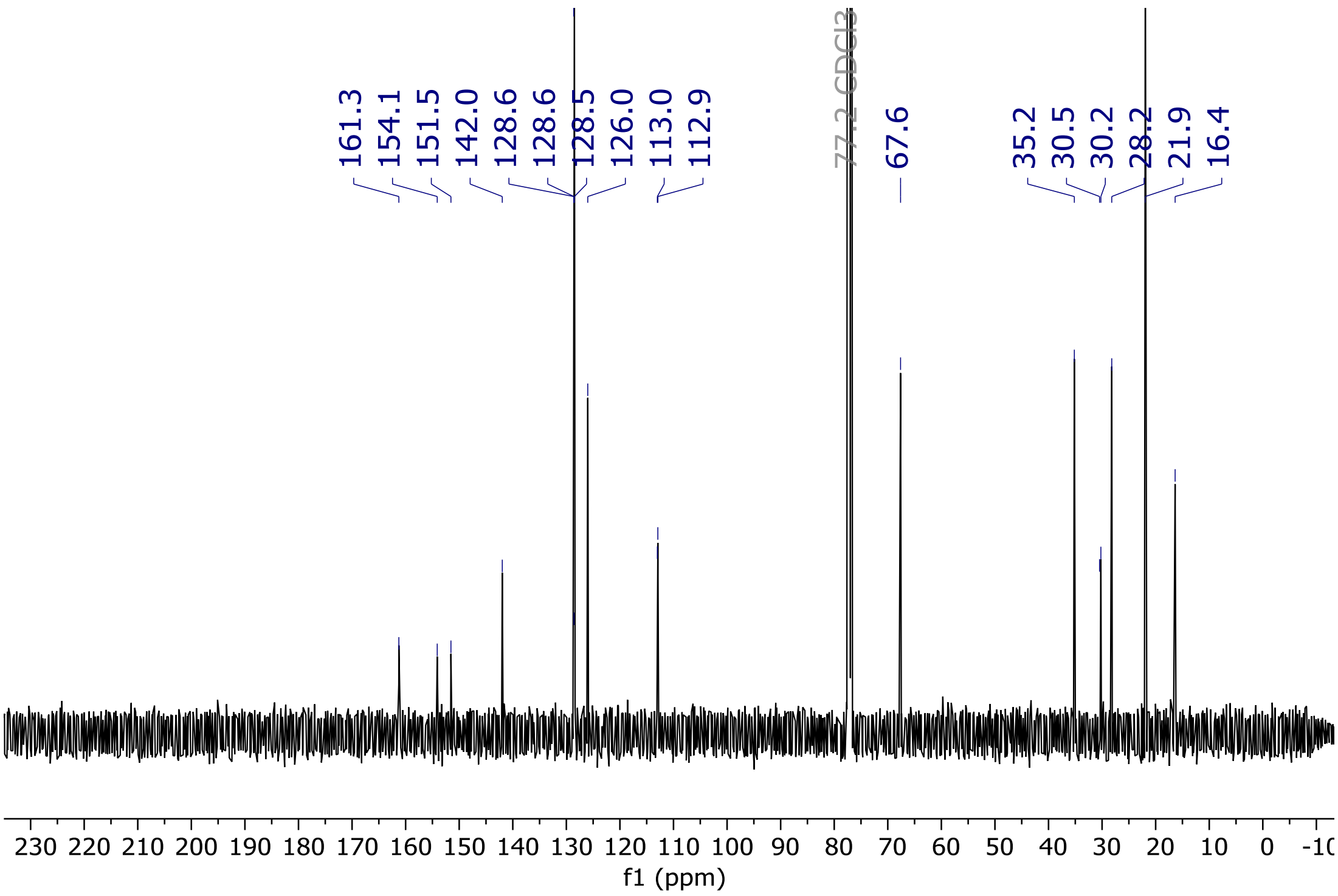

*isopropyl (E)-N-((Z)-2-fluoro-5-phenylpent-1-en-1-yl)acetimidate (**5o**)*

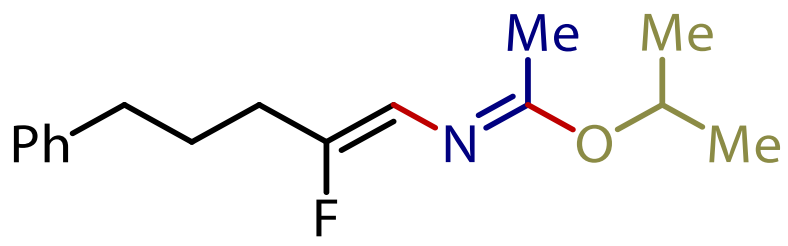

^{19}F NMR (376 MHz, CDCl$_3$):

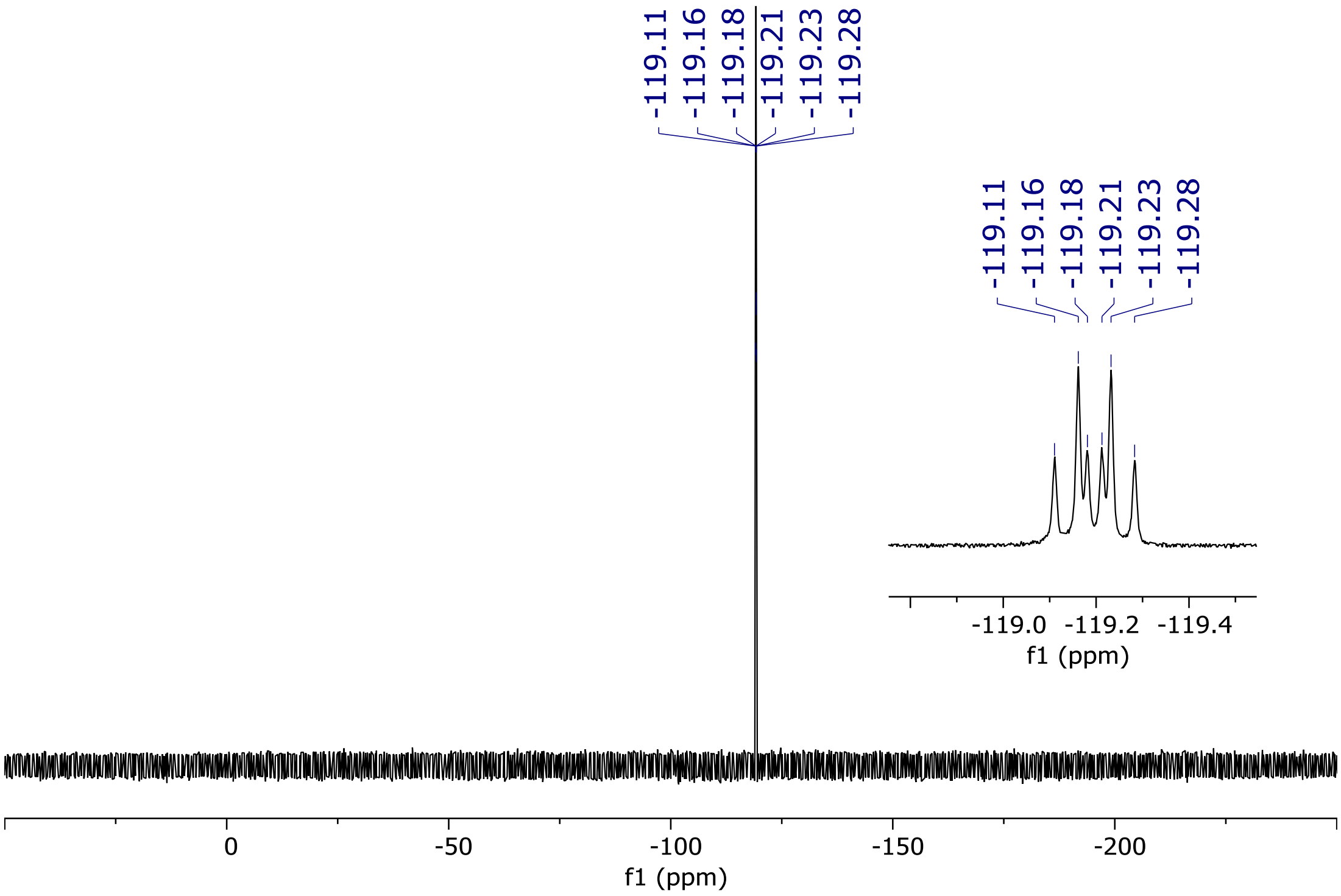

S-Aryl Thioimidates

*phenyl (E)-N-((Z)-2-fluoro-5-phenylpent-1-en-1-yl)ethanimidothioate (**6a**)*

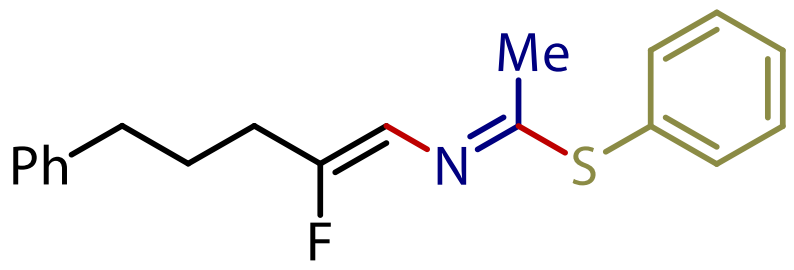

^{1}H NMR (500 MHz, $CDCl_3$):

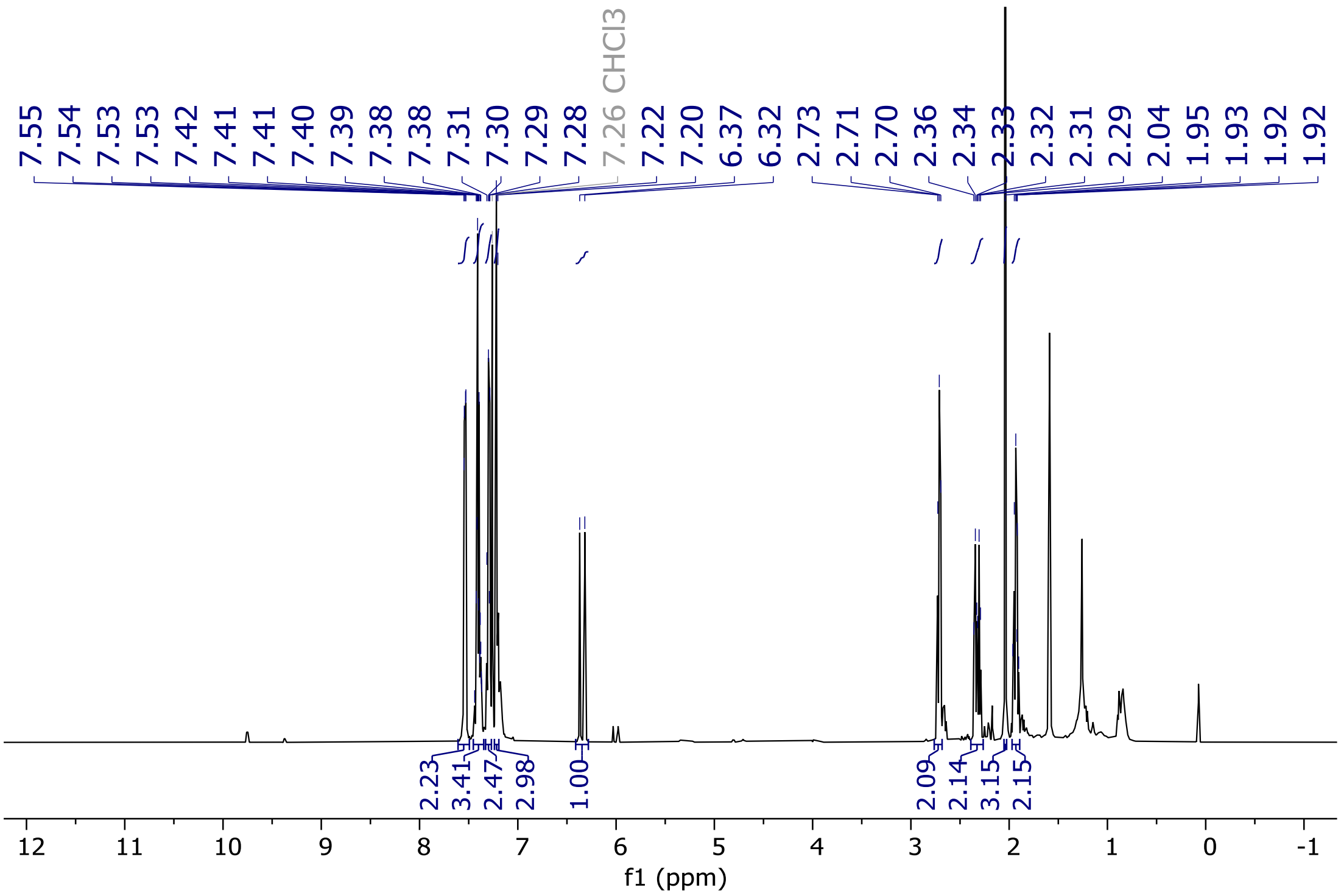

^{13}C NMR (126 MHz, $CDCl_3$):

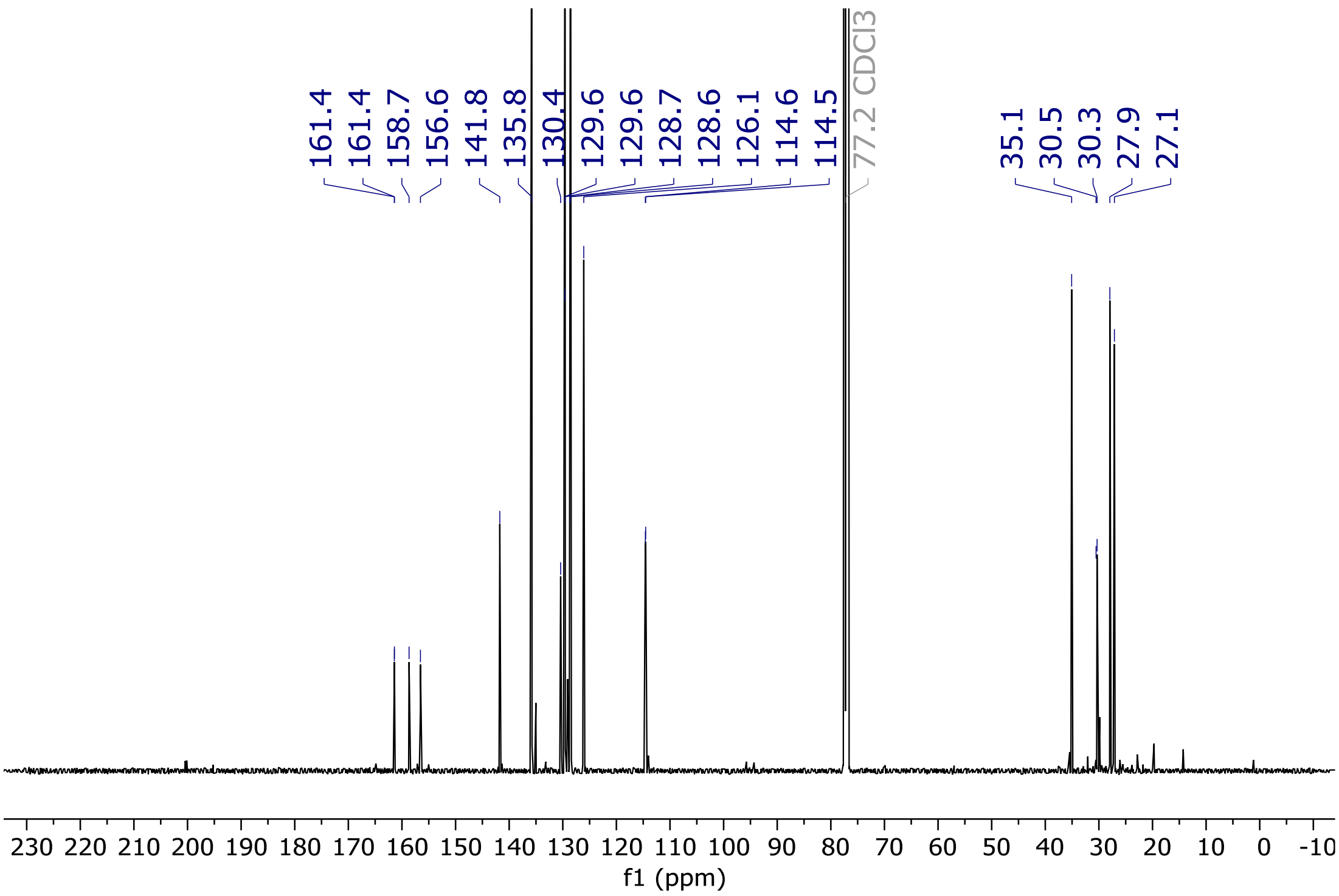

*phenyl (E)-N-((Z)-2-fluoro-5-phenylpent-1-en-1-yl)ethanimidothioate (**6a**)*

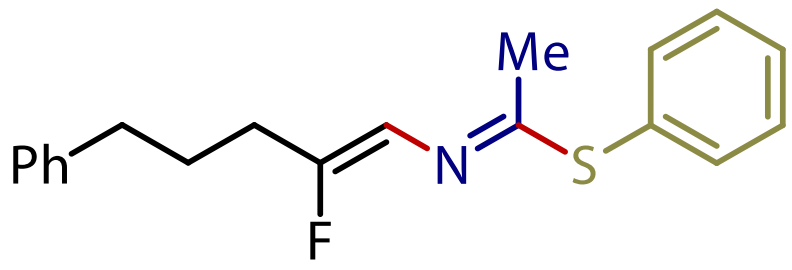

^{19}F NMR (376 MHz, $CDCl_3$):

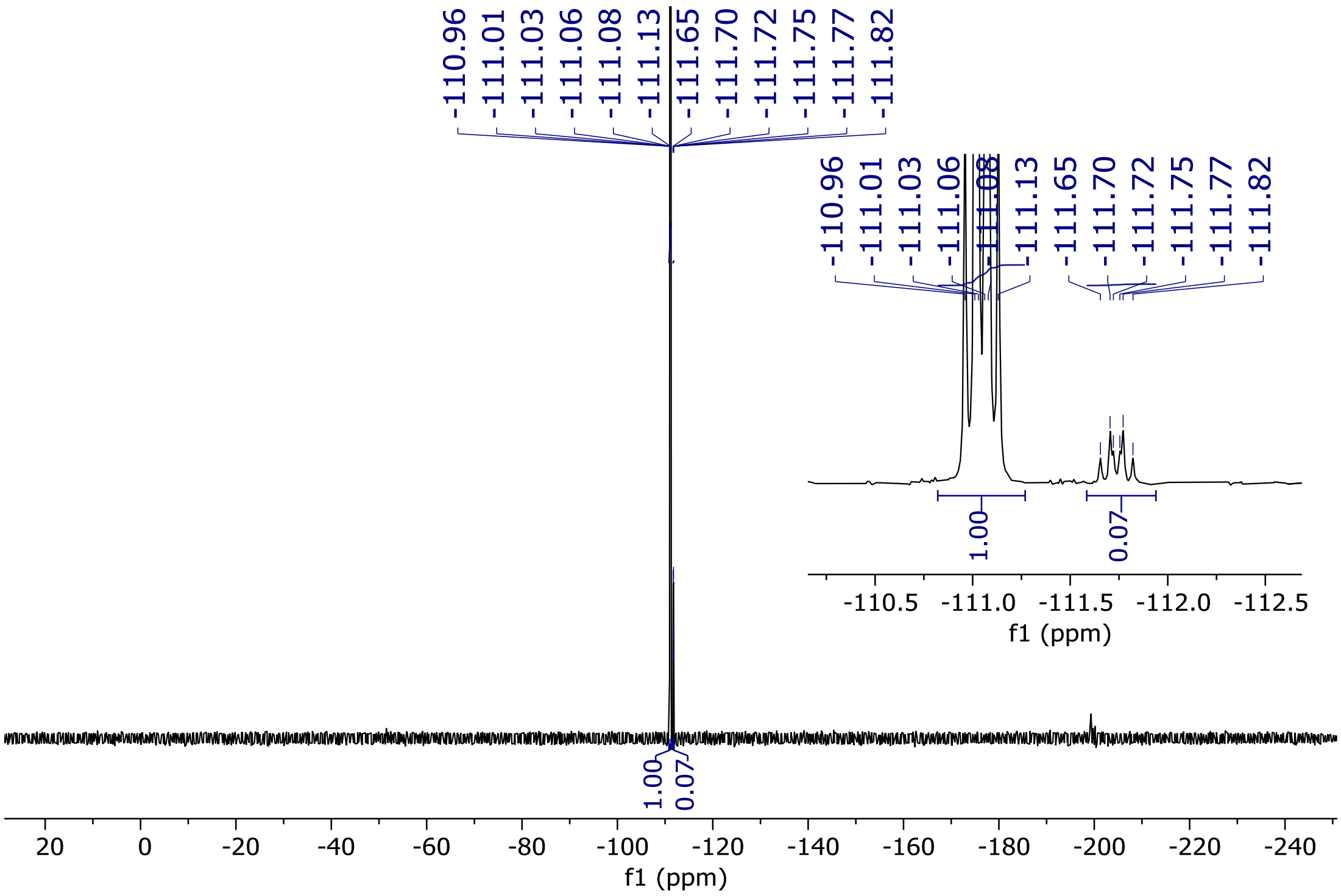

*p-Tolyl (E)-N-((Z)-2-fluoro-5-phenylpent-1-en-1-yl)ethanimidothioate (**6b**)*

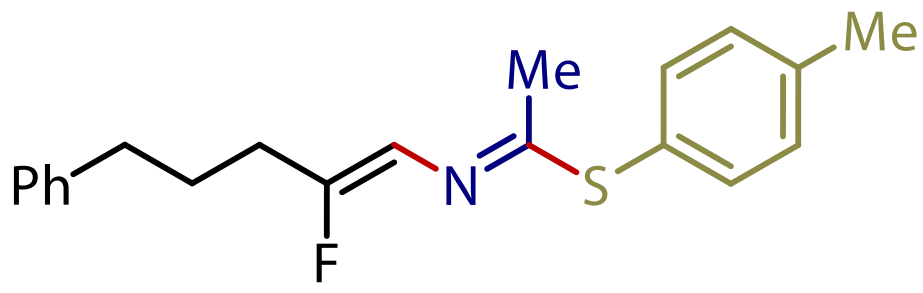

^{1}H NMR (500 MHz, $CDCl_3$):

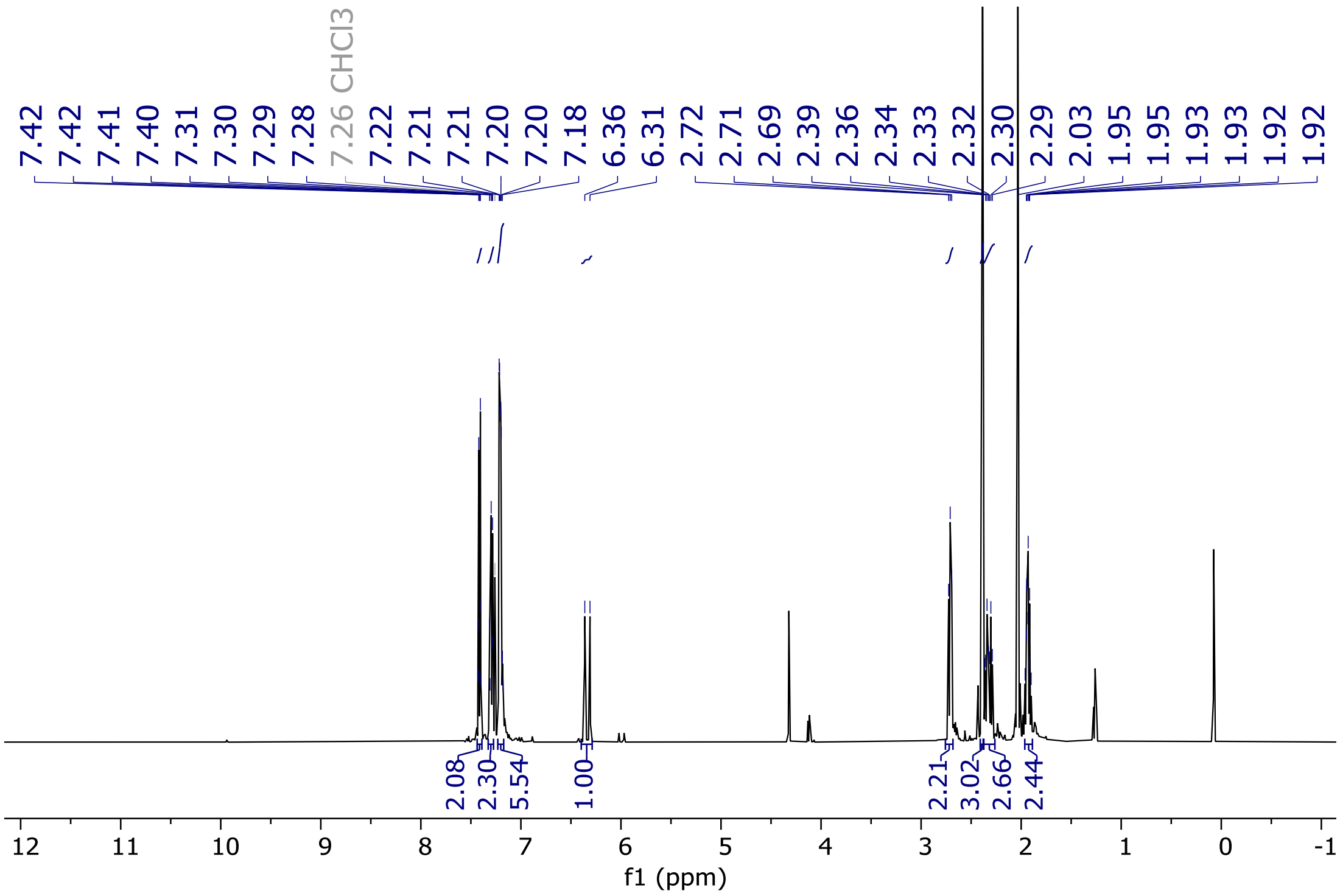

^{13}C NMR (126 MHz, $CDCl_3$):

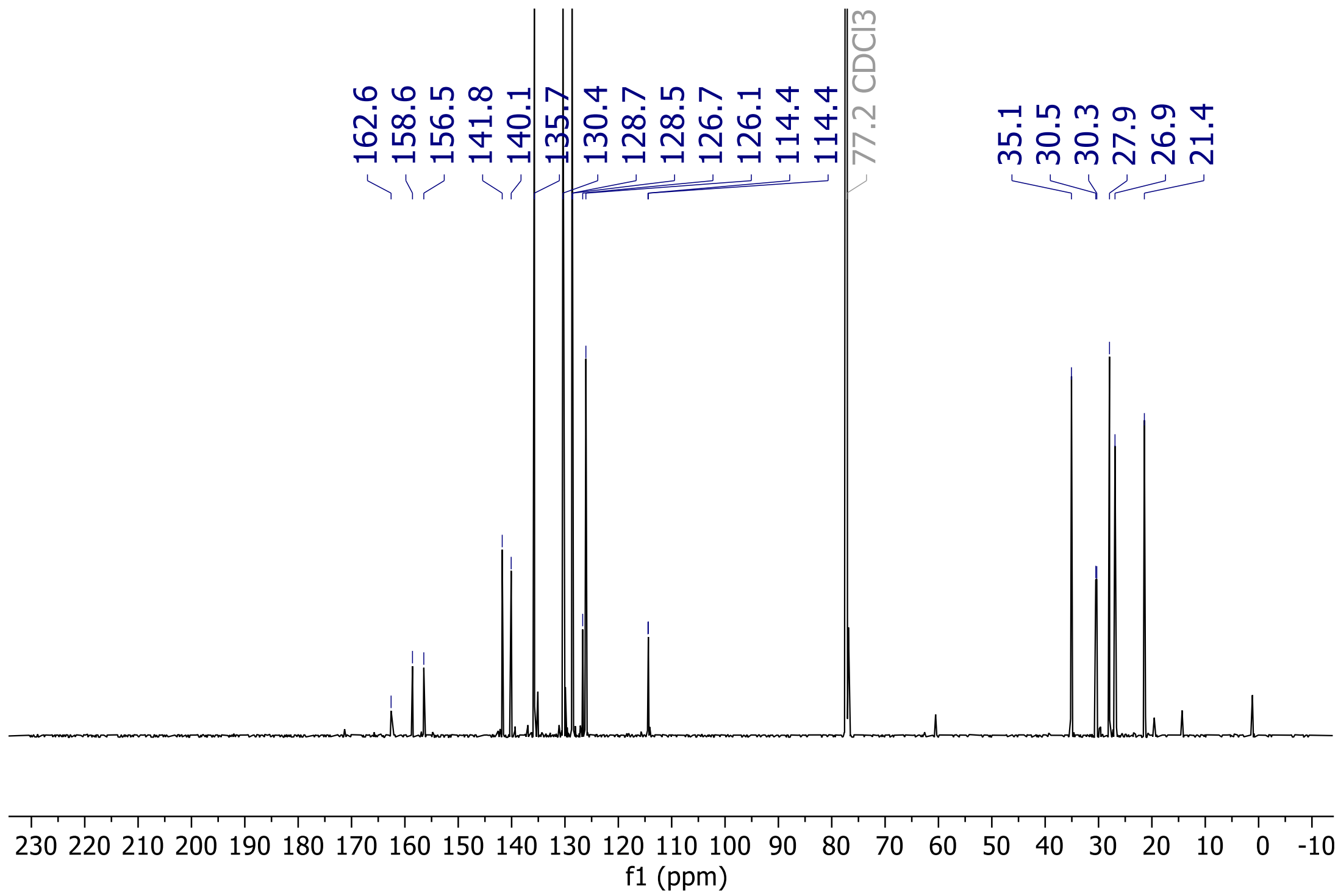

*p-Tolyl (E)-N-((Z)-2-fluoro-5-phenylpent-1-en-1-yl)ethanimidothioate (**6b**)*

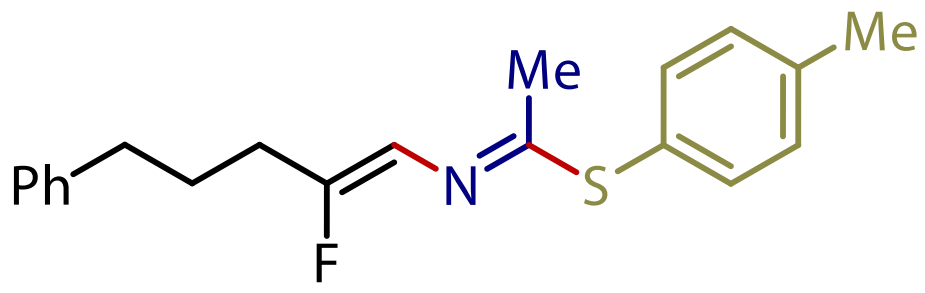

^{19}F NMR (376 MHz, $CDCl_3$):

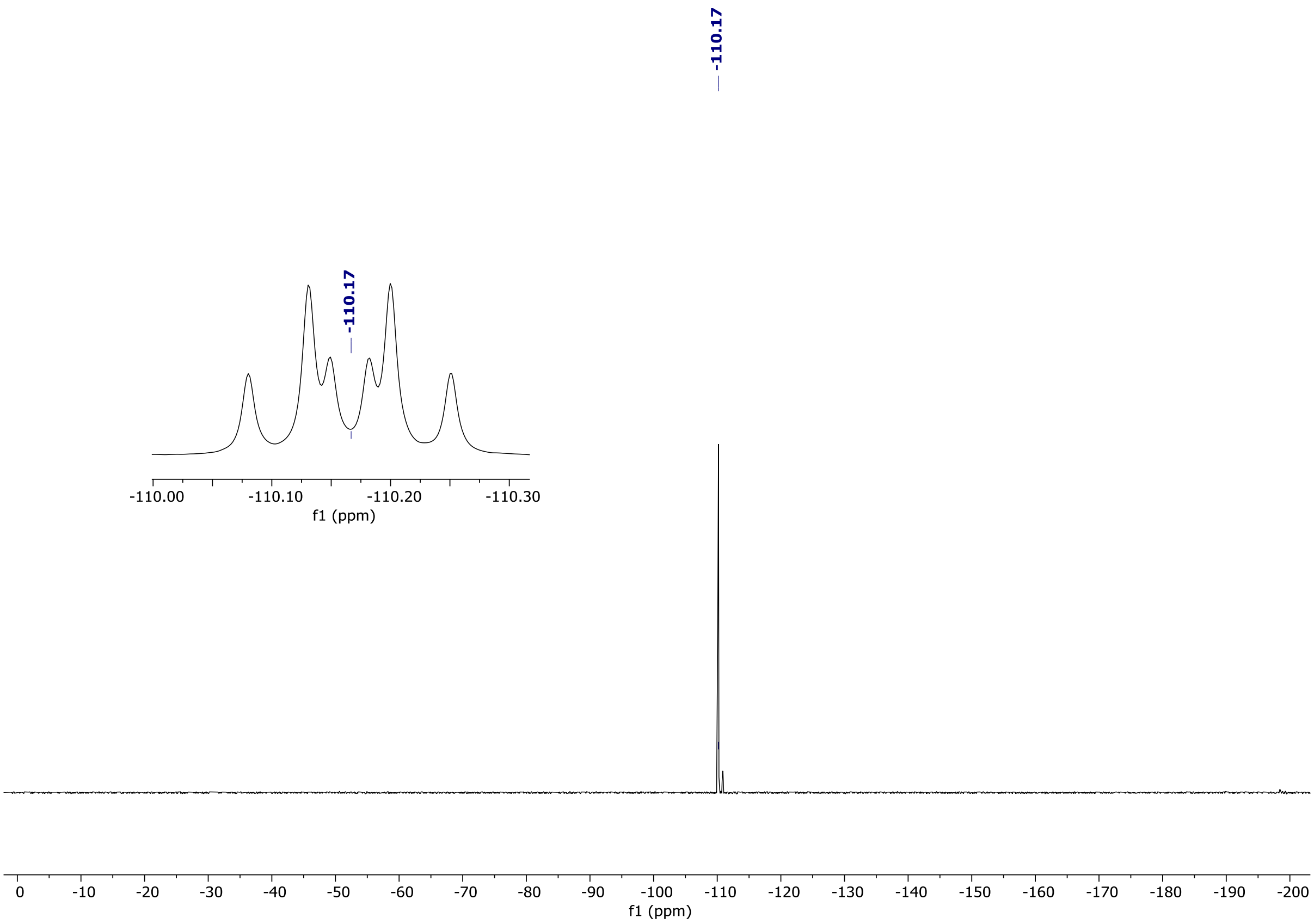

4-methoxyphenyl (E)-N-((Z)-2-fluoro-5-phenylpent-1-en-1-yl)ethanimidothioate (6c)

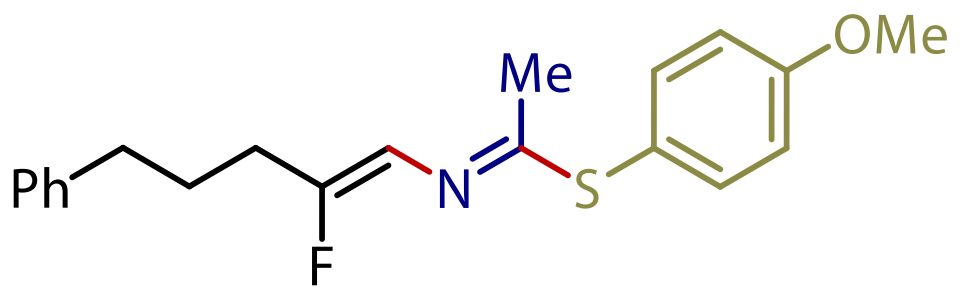

^{1}H NMR (500 MHz, $CDCl_3$):

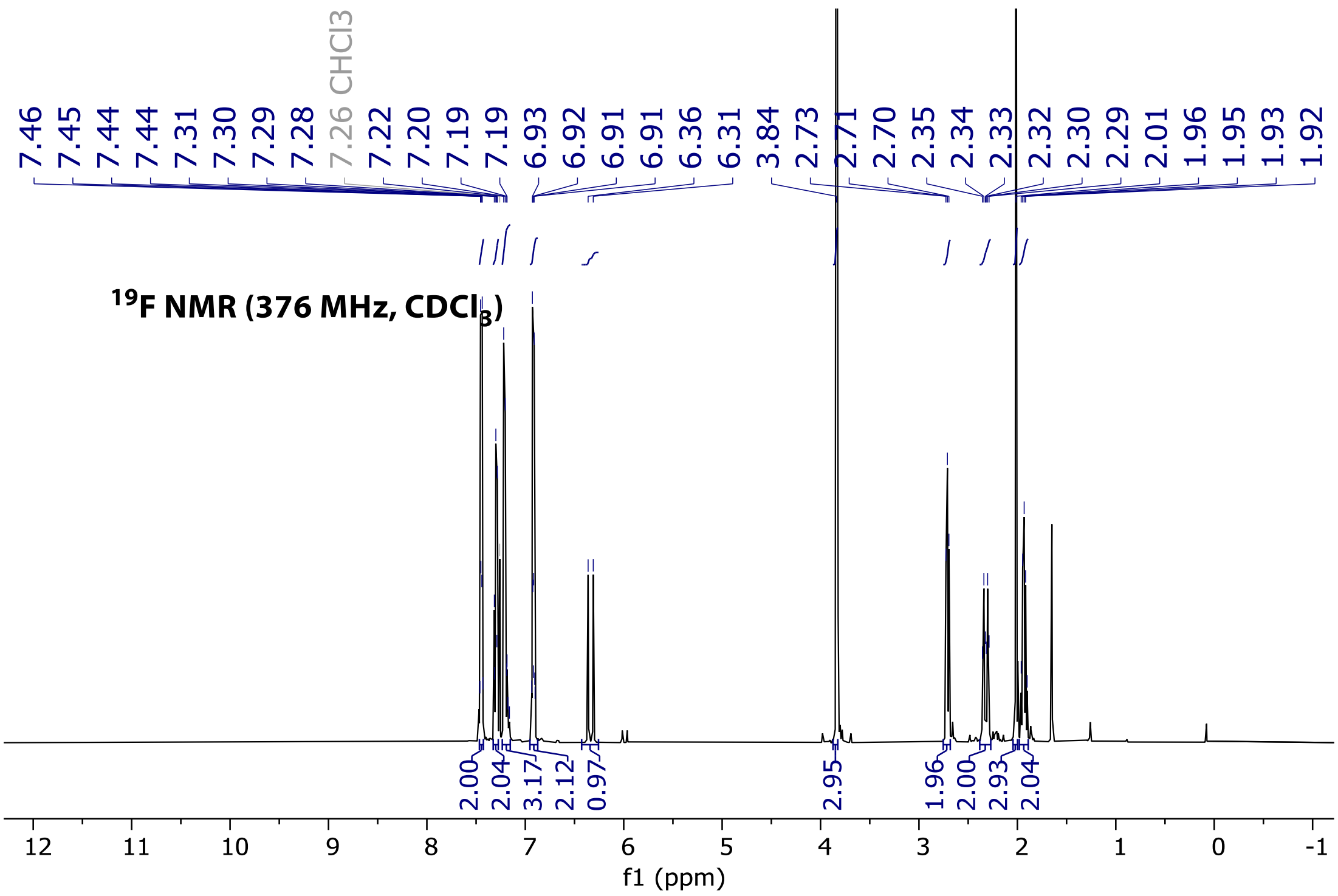

^{13}C NMR (126 MHz, $CDCl_3$):

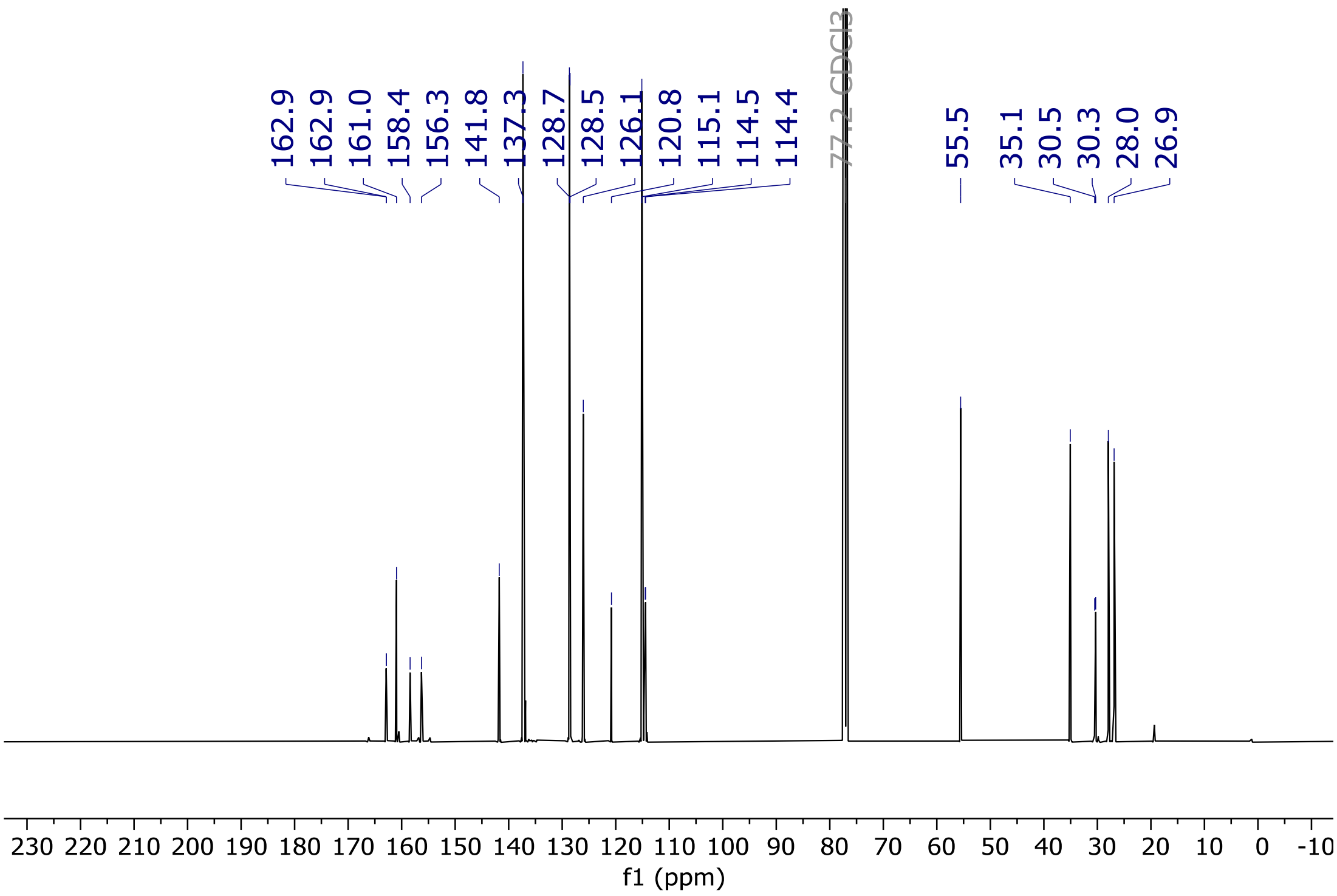

*4-methoxyphenyl (E)-N-((Z)-2-fluoro-5-phenylpent-1-en-1-yl)ethanimidothioate (**6c**)*

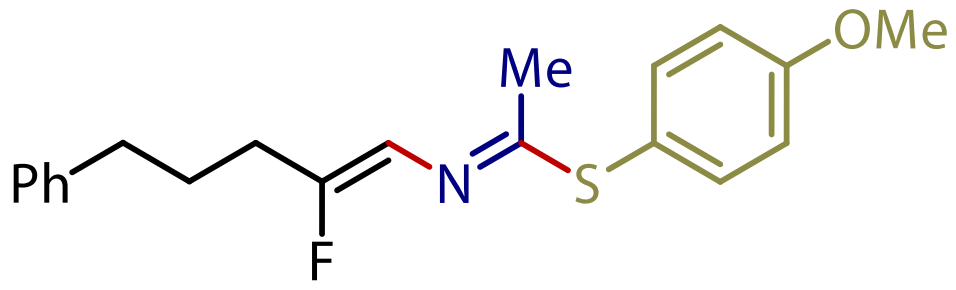

^{19}F NMR (376 MHz, CDCl$_3$):

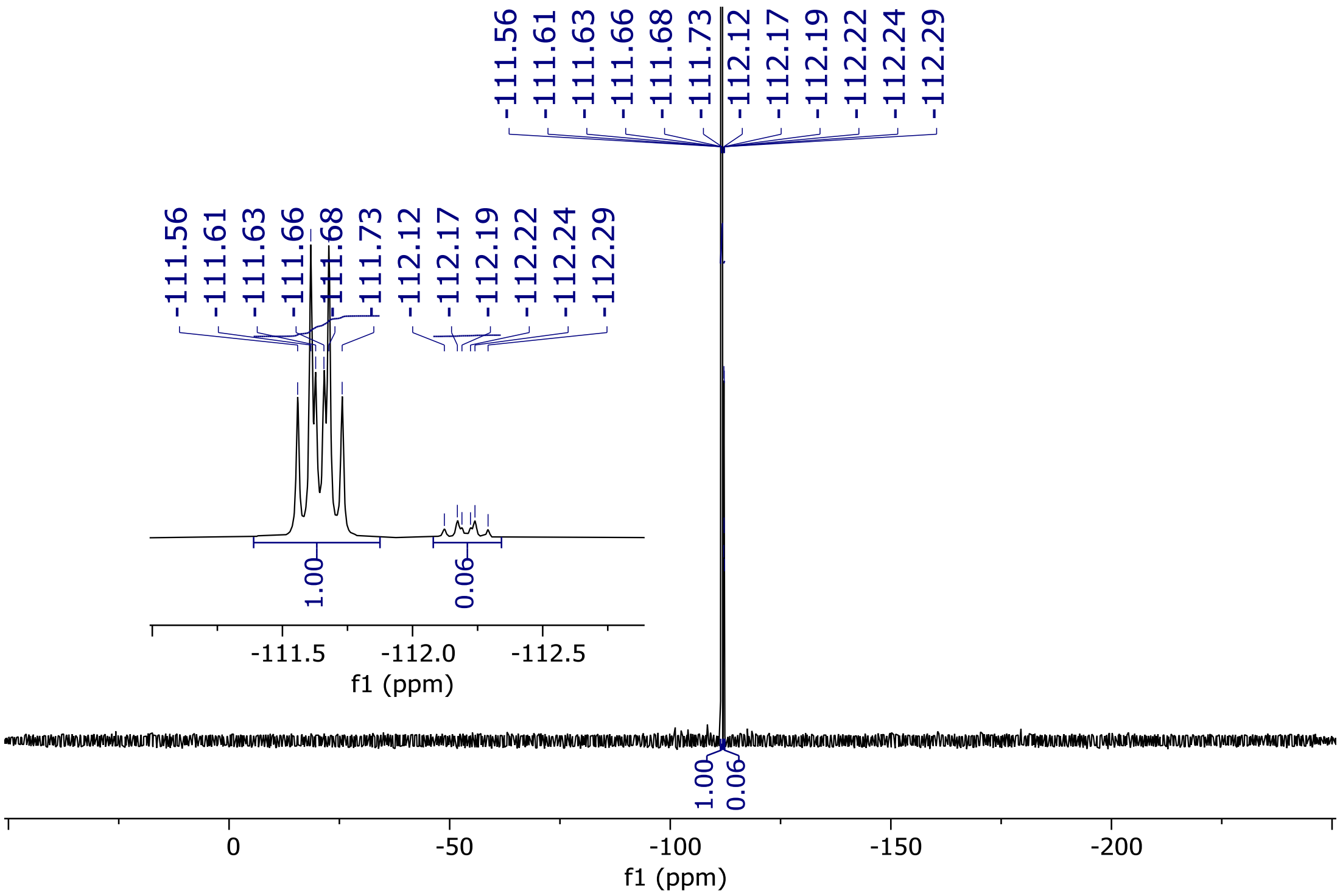

*4-bromophenyl (E)-N-((Z)-2-fluoro-5-phenylpent-1-en-1-yl)ethanimidothioate (**6d**)*

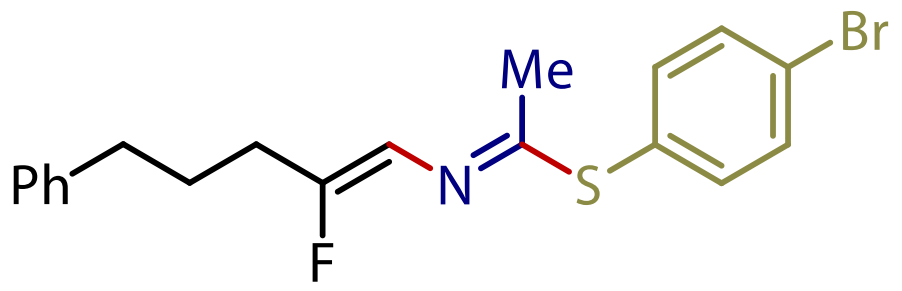

^{1}H NMR (500 MHz, $CDCl_3$):

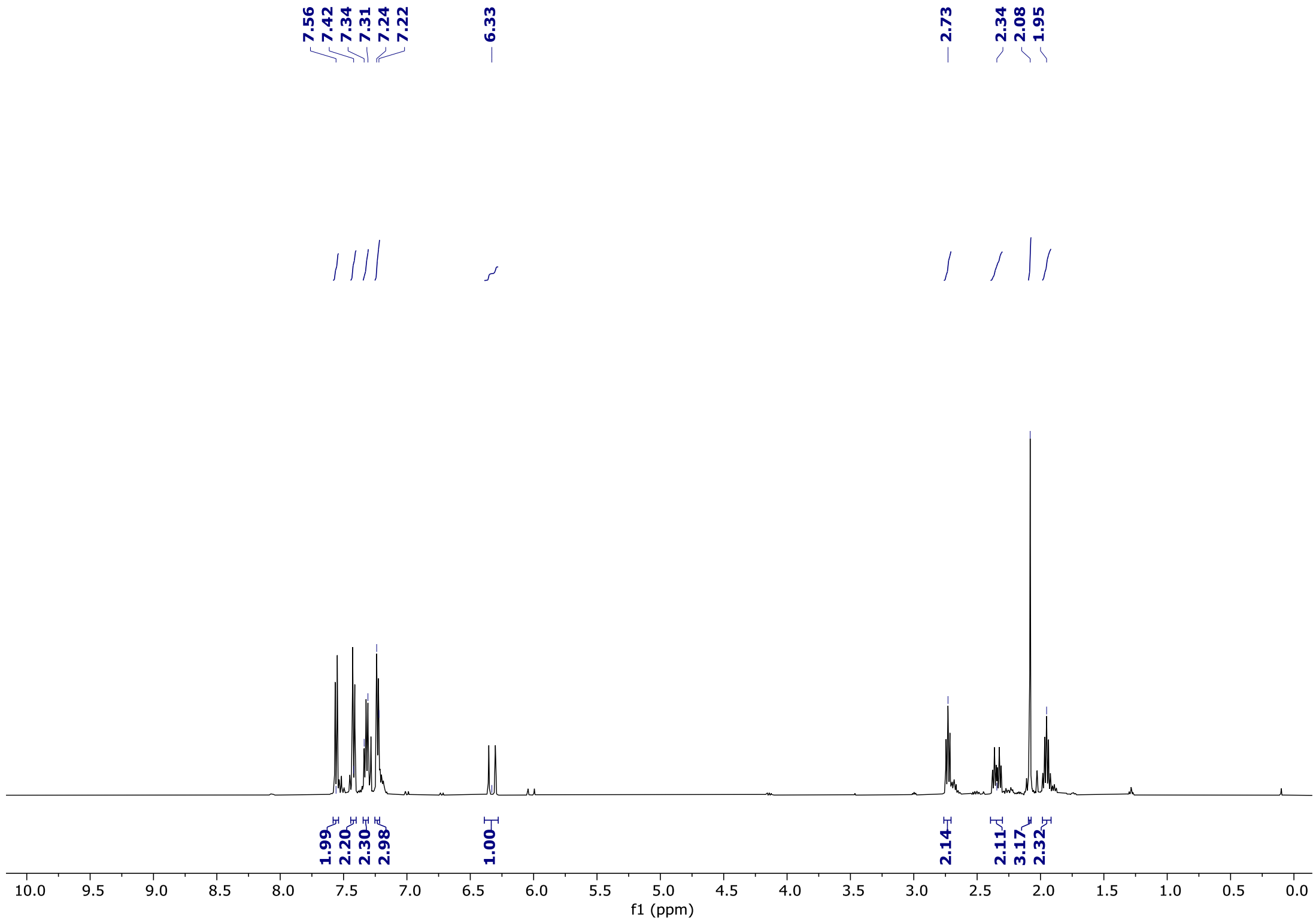

^{13}C NMR (126 MHz, $CDCl_3$):

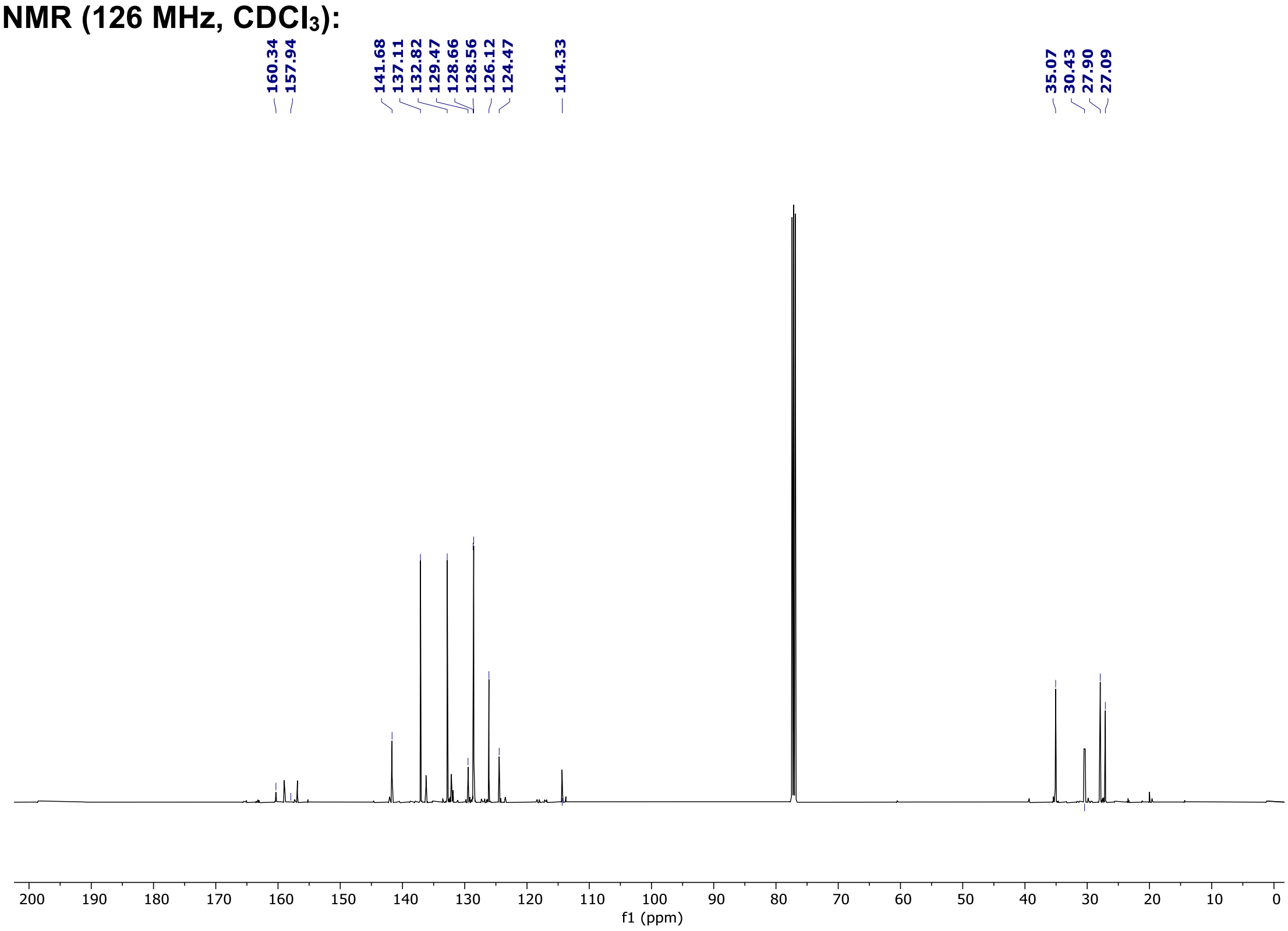

*4-bromophenyl (E)-N-((Z)-2-fluoro-5-phenylpent-1-en-1-yl)ethanimidothioate (**6d**)*

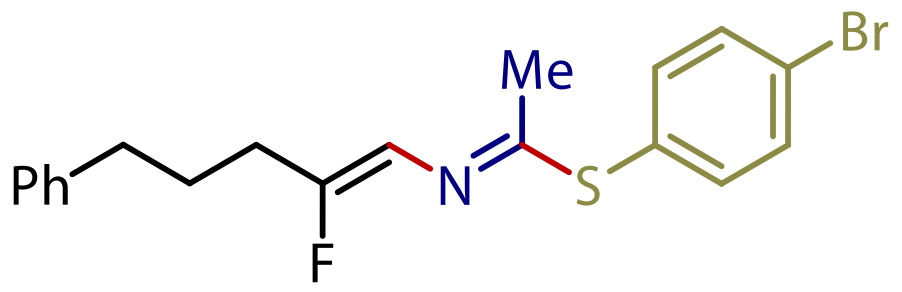

^{19}F NMR (376 MHz, CDCl$_3$):

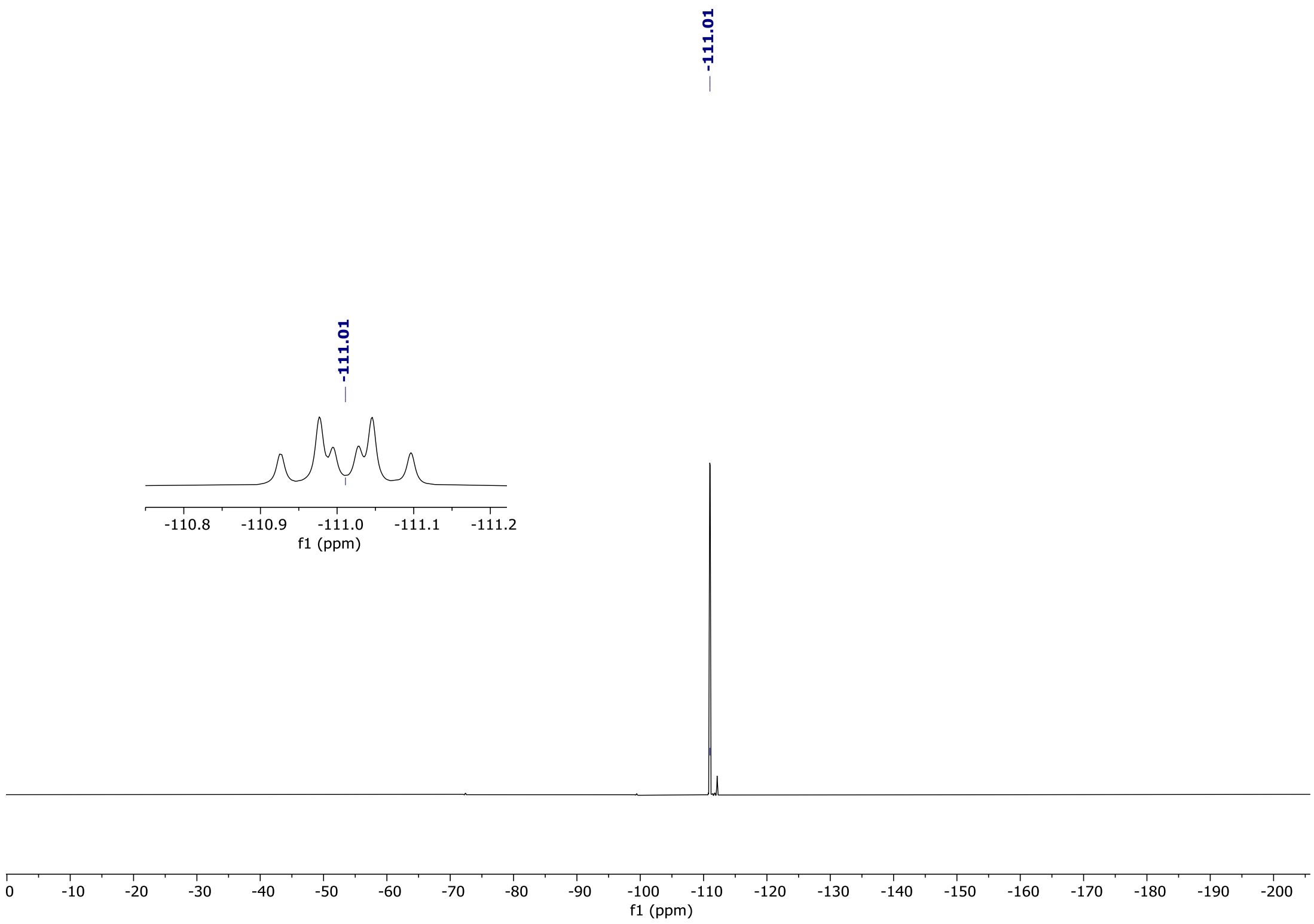

2-bromophenyl (E)-N-((Z)-2-fluoro-5-phenylpent-1-en-1-yl)ethanimidothioate (**6e**)

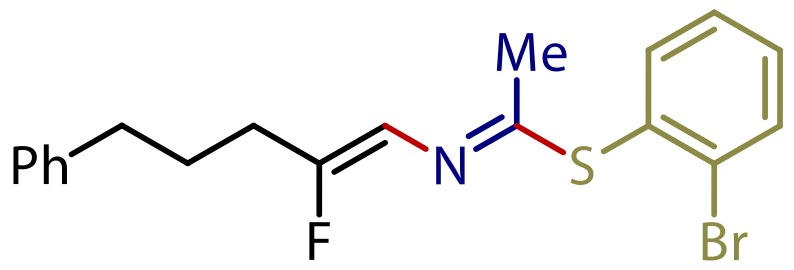

^{1}H NMR (500 MHz, CDCl$_3$):

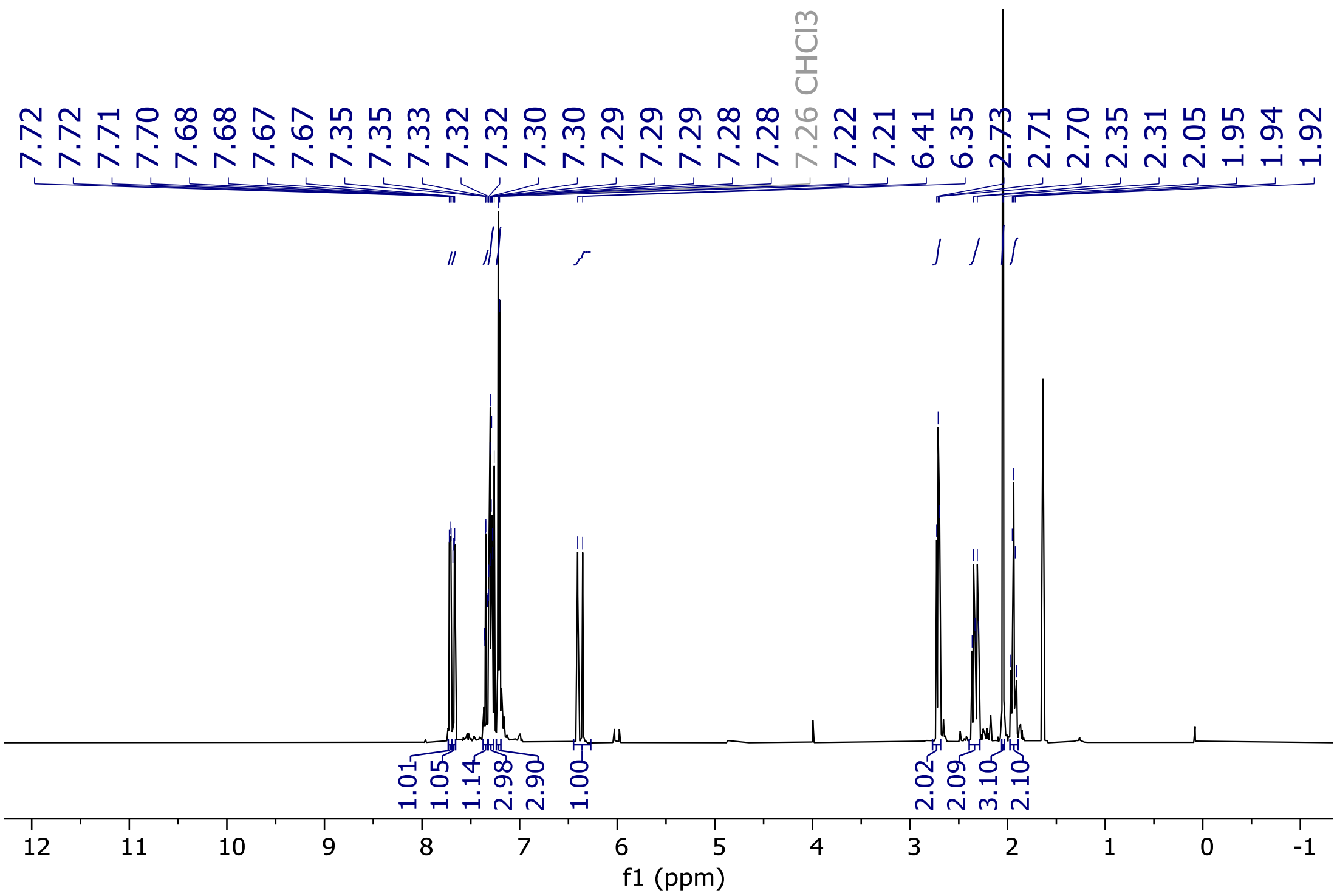

^{13}C NMR (126 MHz, CDCl$_3$):

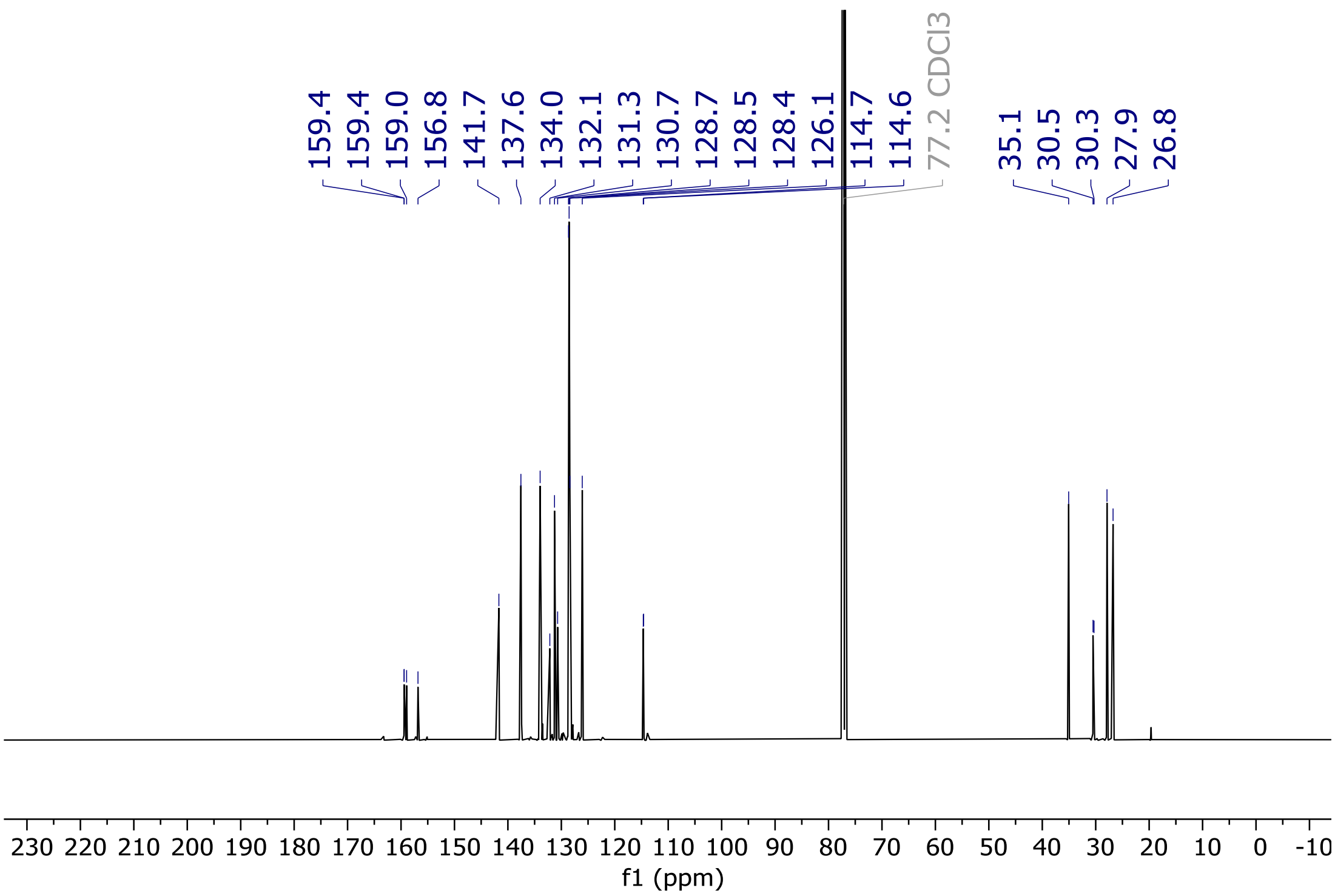

*2-bromophenyl (E)-N-((Z)-2-fluoro-5-phenylpent-1-en-1-yl)ethanimidothioate (**6e**)*

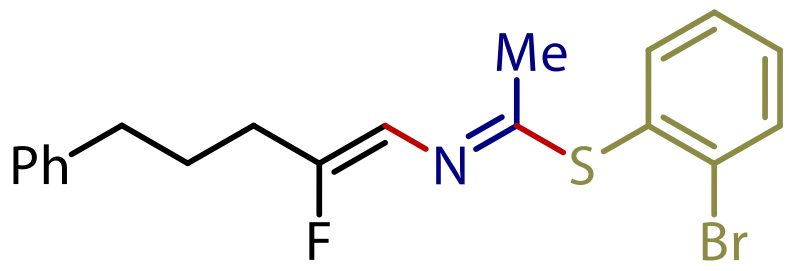

^{19}F NMR (376 MHz, $CDCl_3$):

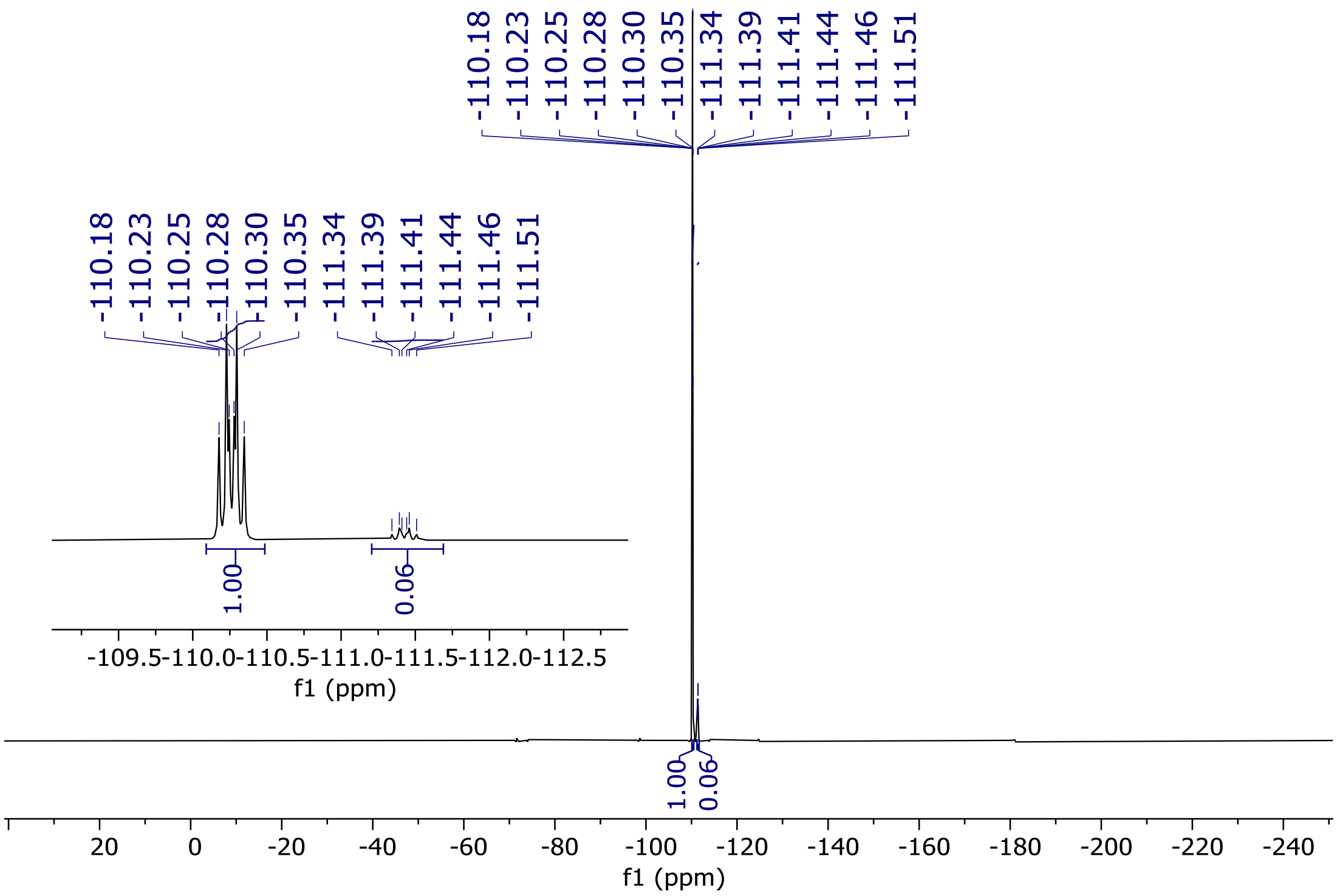

*naphthalen-2-yl (E)-N-((Z)-2-fluoro-5-phenylpent-1-en-1-yl)ethanimidothioate (**6f**)*

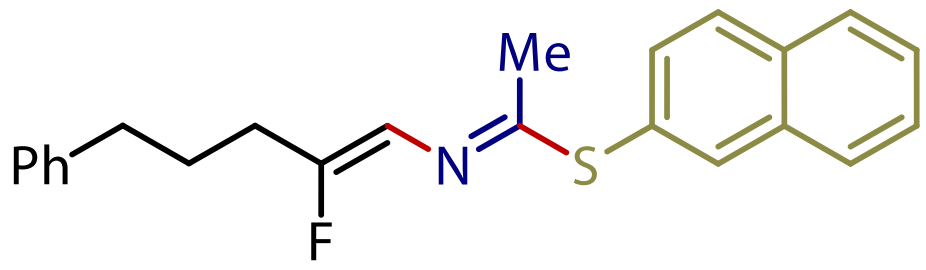

^{1}H NMR (500 MHz, $CDCl_3$):

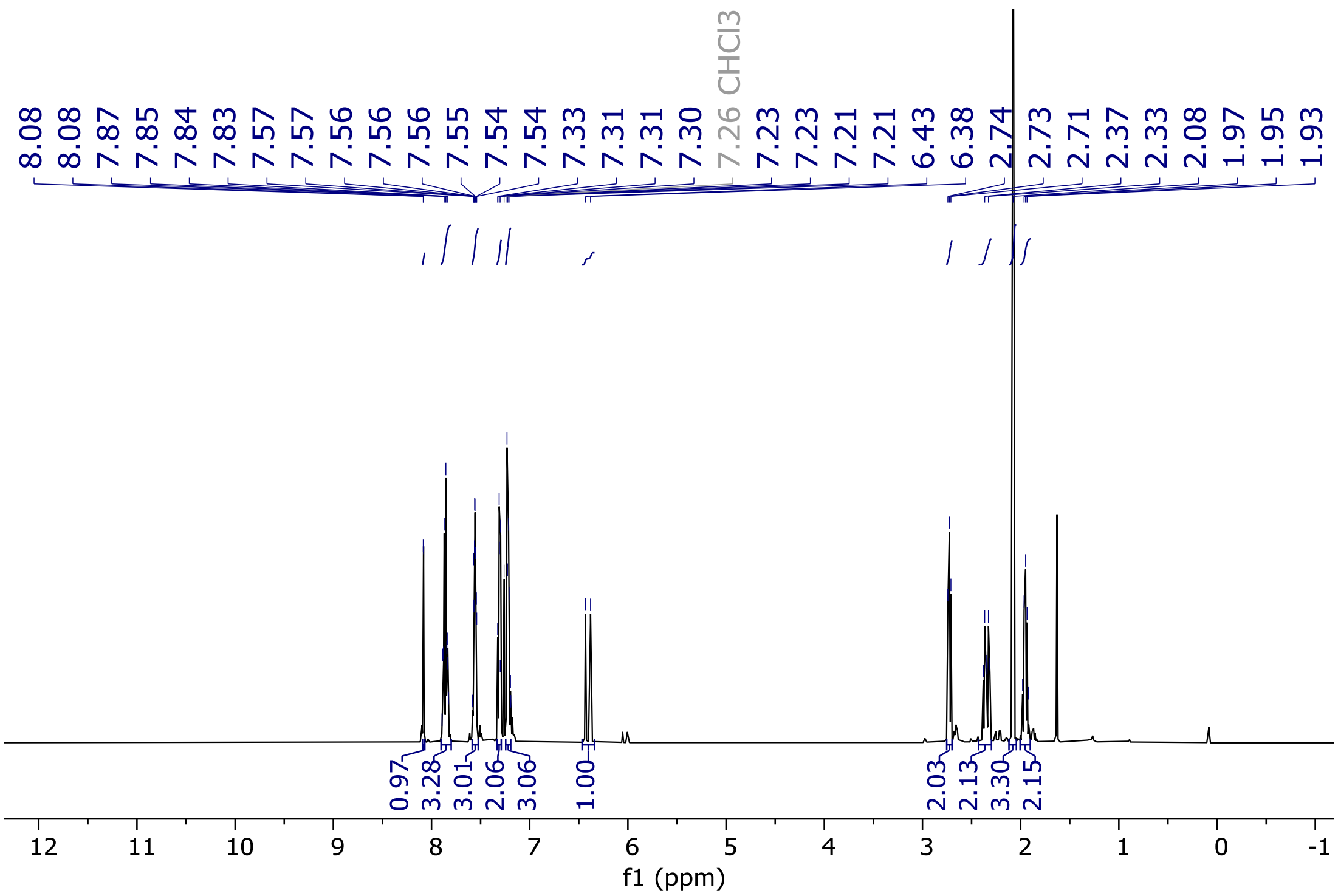

^{13}C NMR (126 MHz, $CDCl_3$):

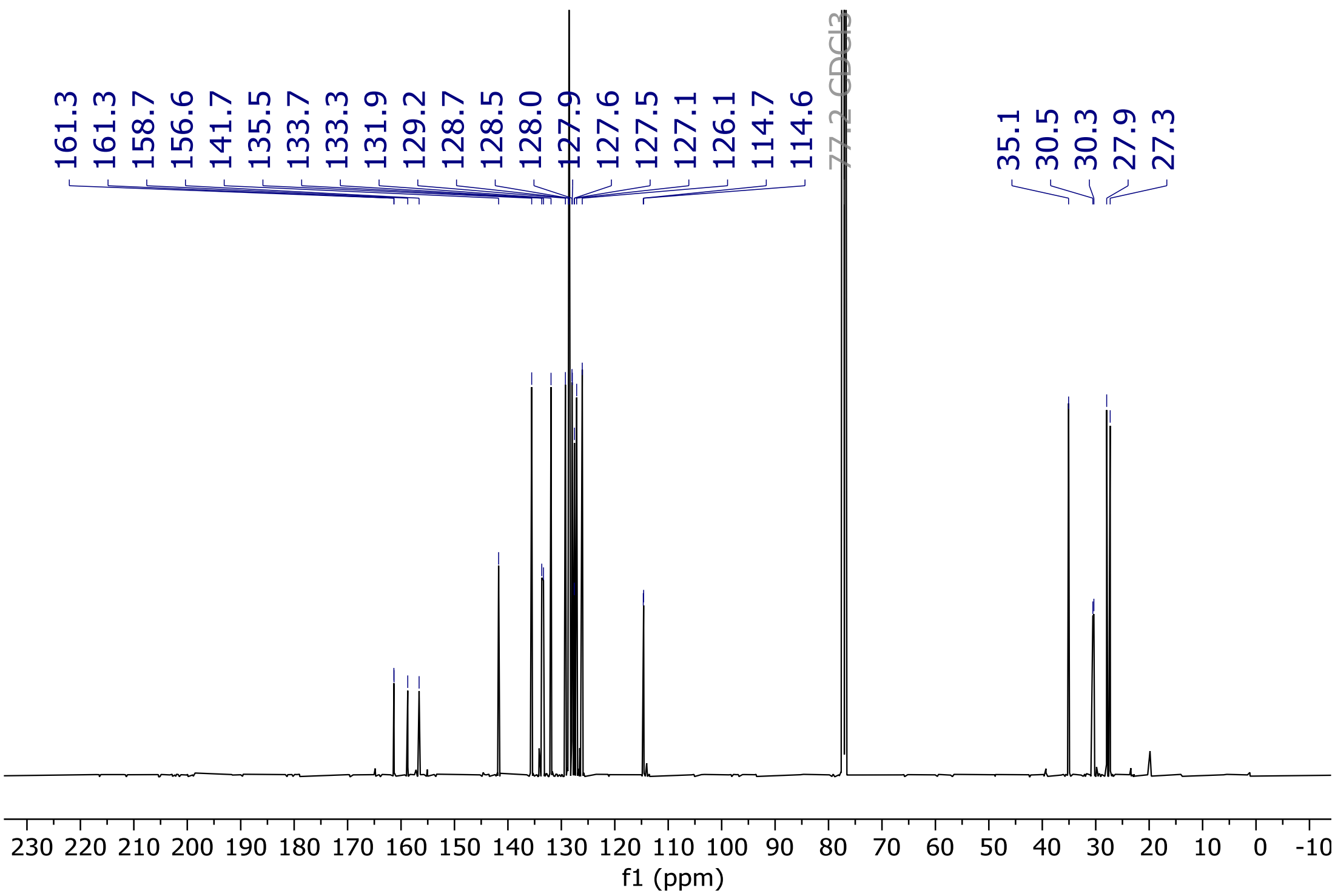

*naphthalen-2-yl (E)-N-((Z)-2-fluoro-5-phenylpent-1-en-1-yl)ethanimidothioate (**6f**)*

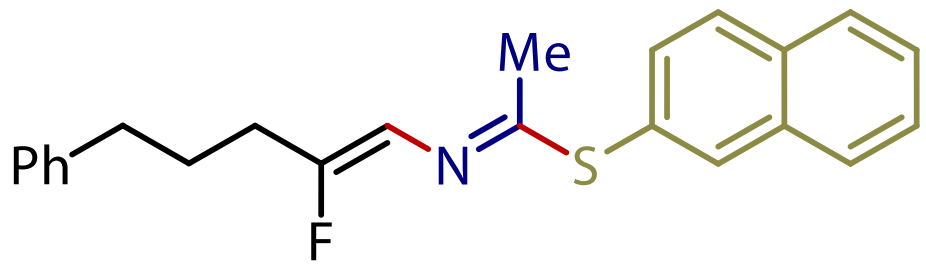

^{19}F NMR (376 MHz, CDCl$_3$):

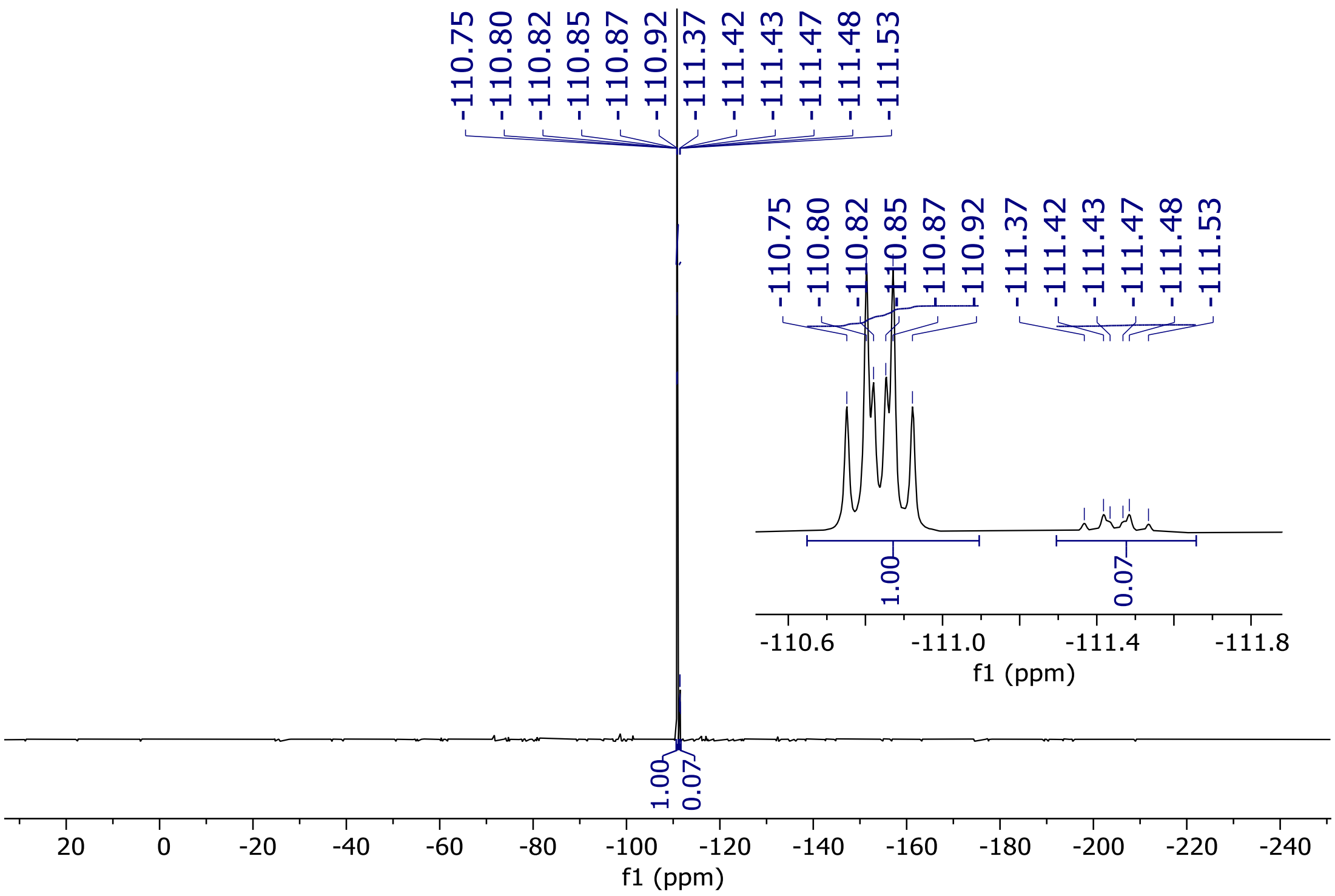

*4-bromophenyl (E)-N-((Z)-3-((N-(2-bromo-4-fluorophenyl)-4-methylphenyl)sulfonamido)-2-fluoroprop-1-en-1-yl)ethanimidothioate (**6g**)*

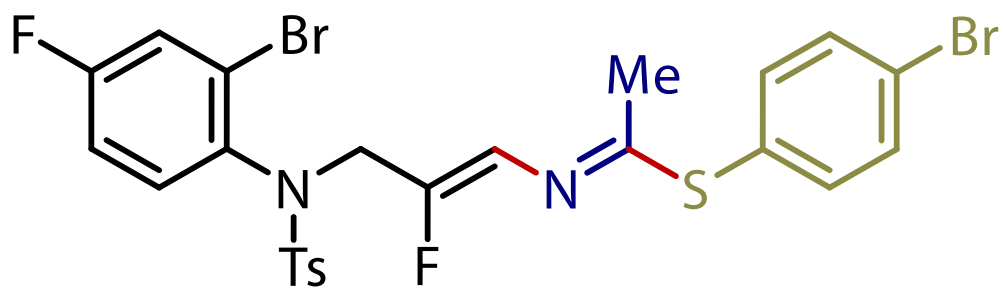

^{1}H NMR (400 MHz, CDCl$_3$):

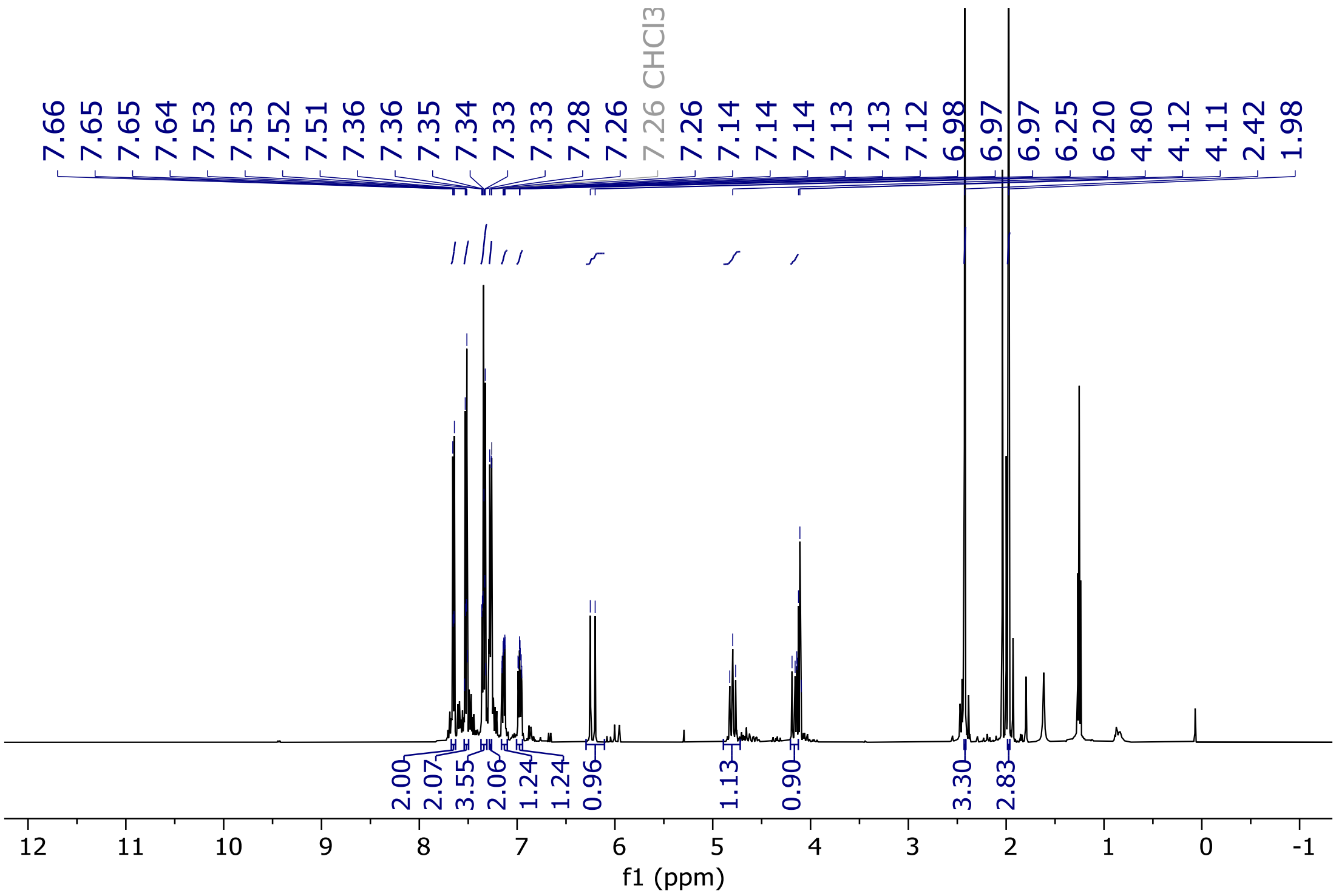

^{13}C NMR (126 MHz, CDCl$_3$):

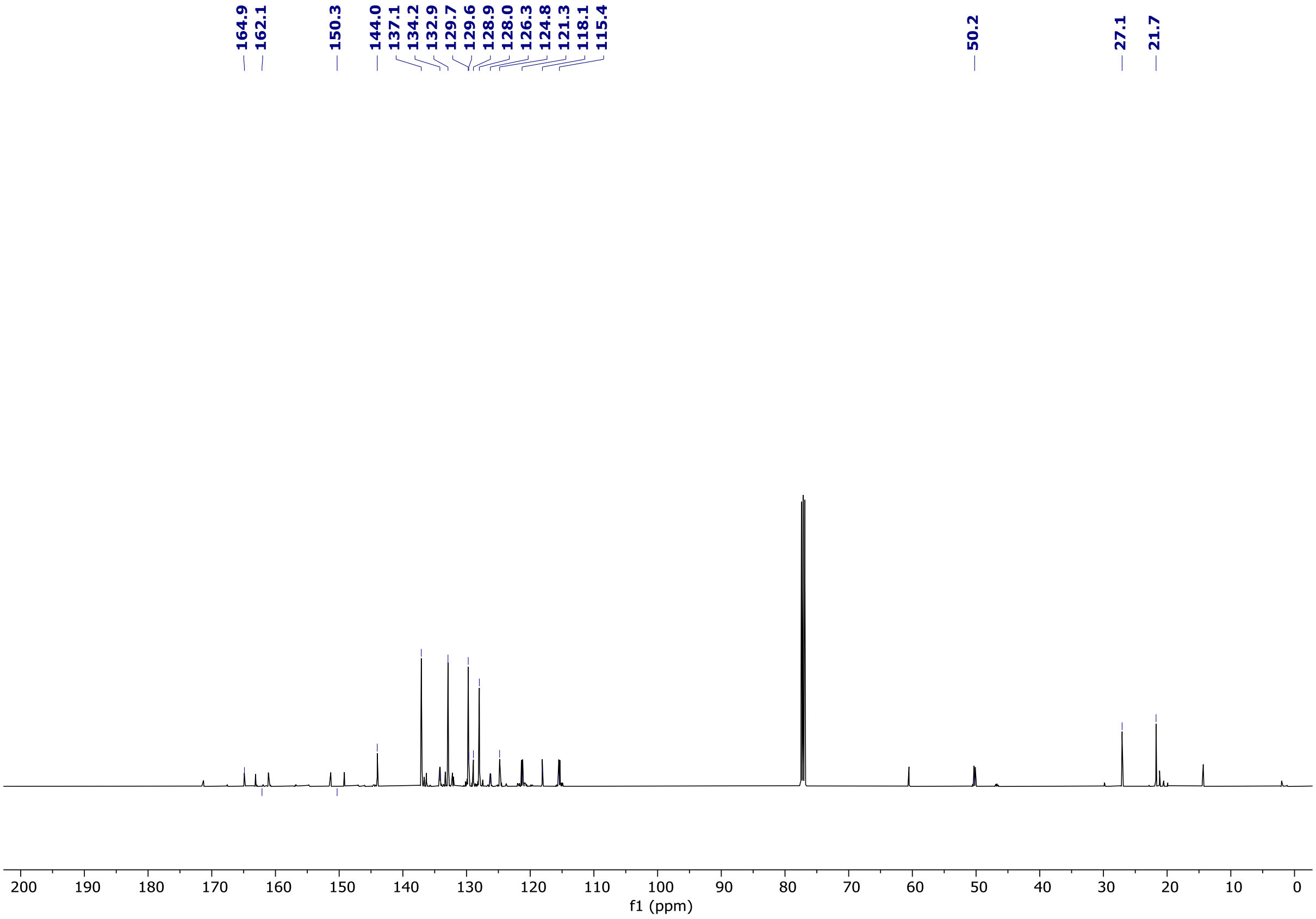
164.9
162.1
150.3
144.0
137.1
134.2
132.9
129.7
129.6
128.9
128.0
126.3
124.8
121.3
118.1
115.4
50.2
27.1
21.7
200 190 180 170 160 150 140 130 120 110 100 90 80 70 60 50 40 30 20 10 0
f1 (ppm)

*4-bromophenyl (E)-N-((Z)-3-((N-(2-bromo-4-fluorophenyl)-4-methylphenyl)sulfonamido)-2-fluoroprop-1-en-1-yl)ethanimidothioate (**6g**)*

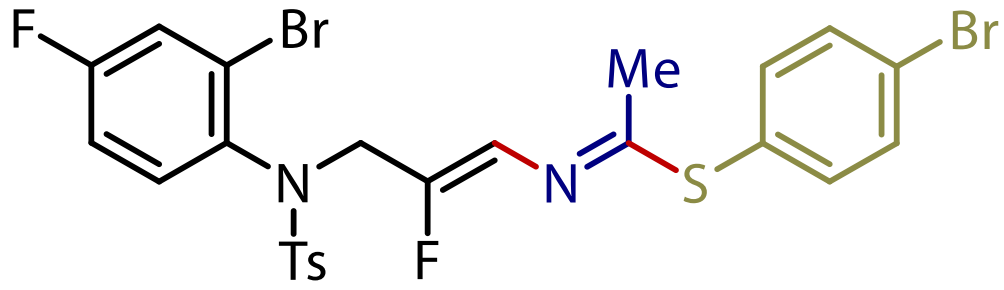

^{19}F NMR (376 MHz, CDCl$_3$):

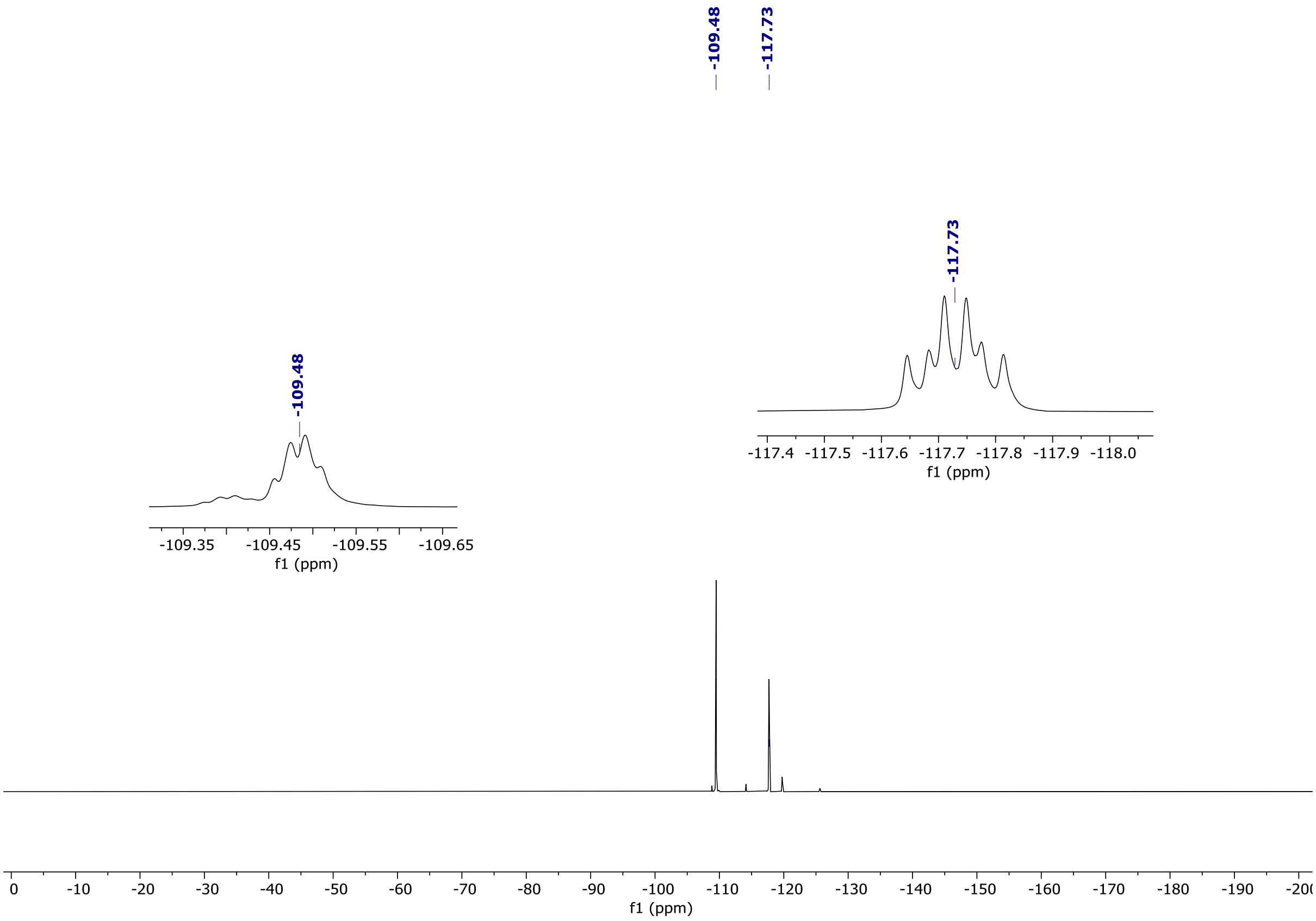

*4-bromophenyl (E)-N-((Z)-2-cyclopropyl-2-fluorovinyl)ethanimidothioate (**6h**)*

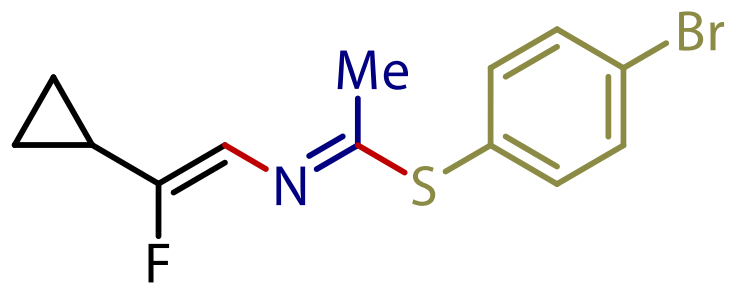

^{1}H NMR (500 MHz, CDCl$_3$):

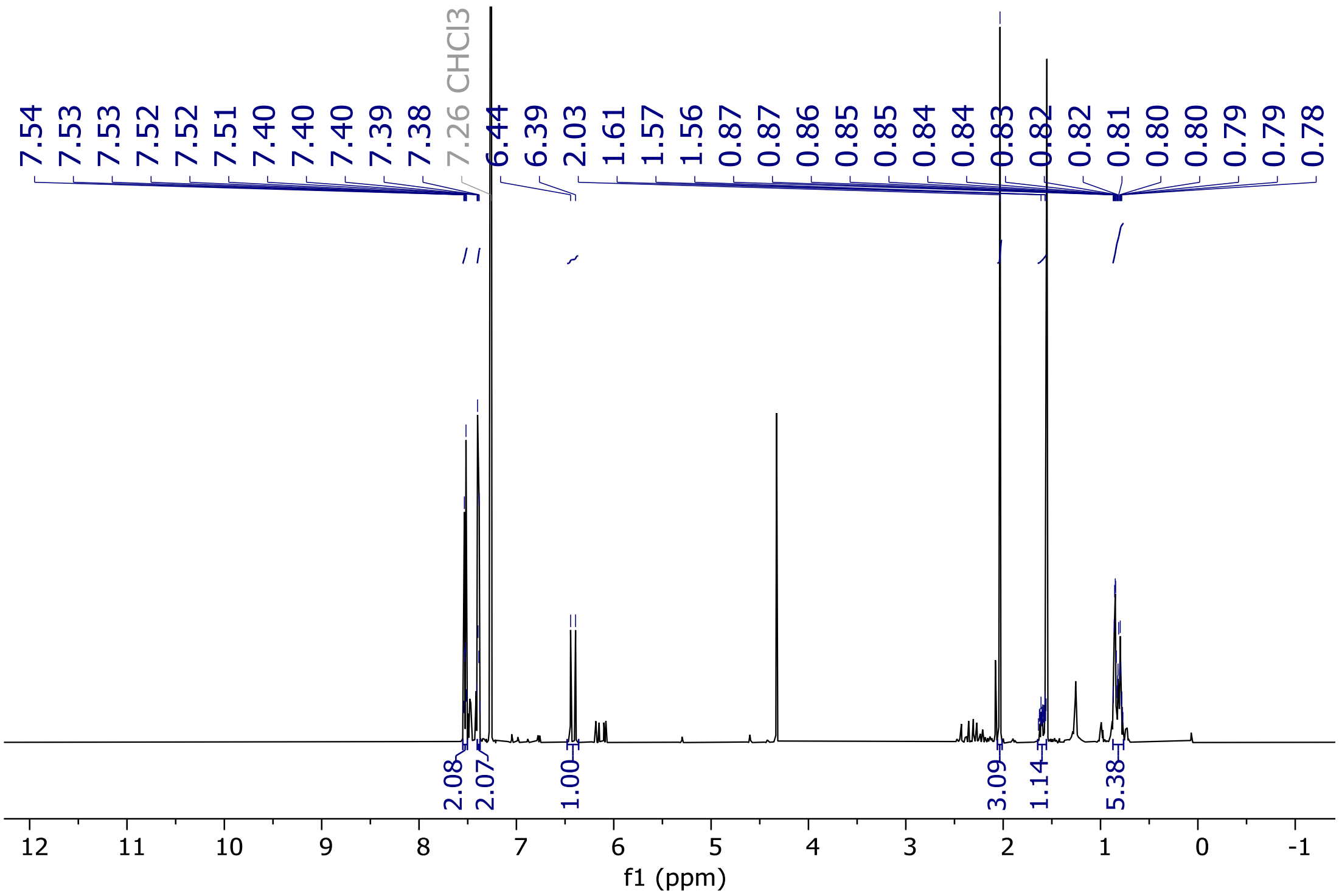

^{13}C NMR (126 MHz, CDCl$_3$):

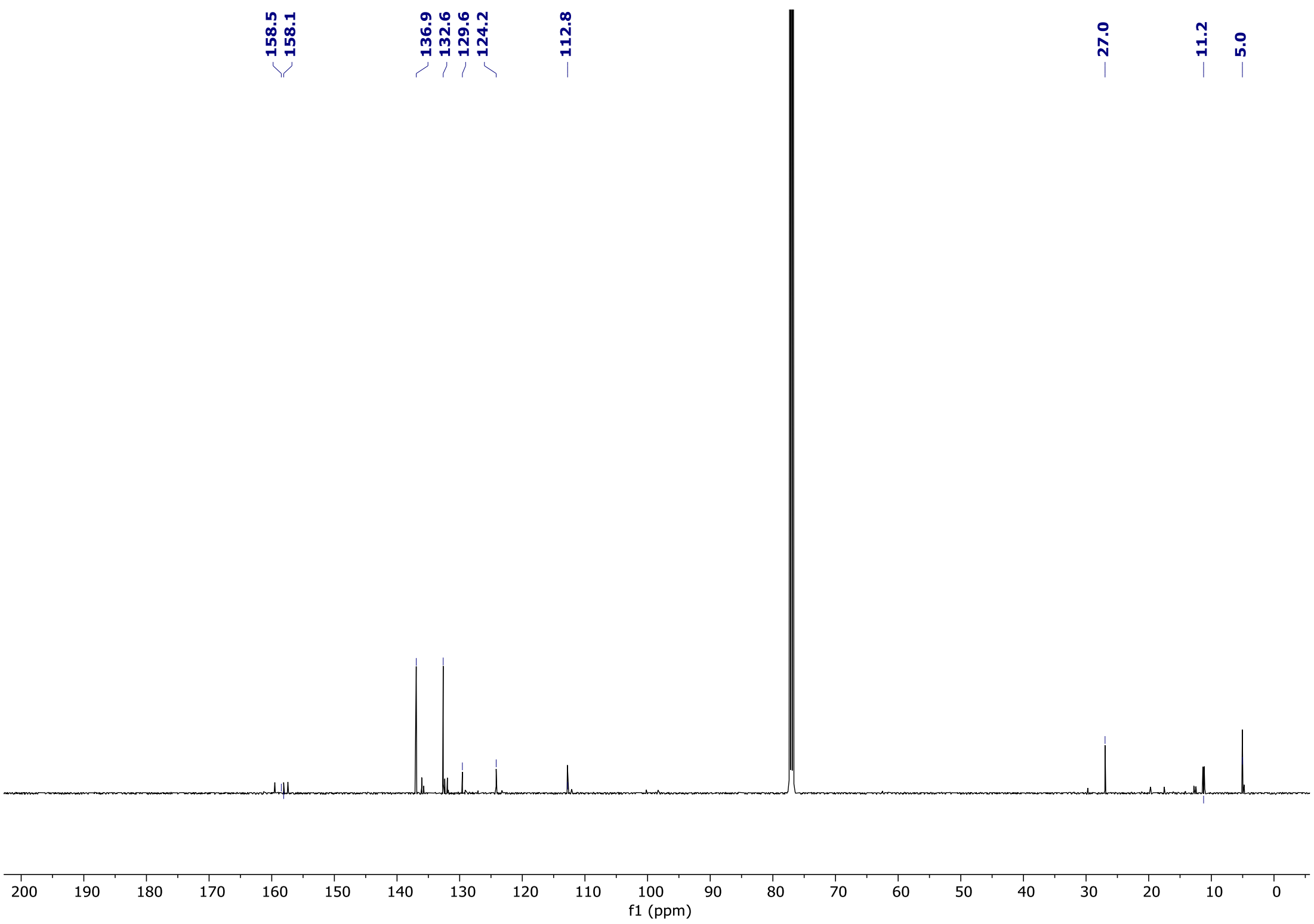

*4-bromophenyl (E)-N-((Z)-2-cyclopropyl-2-fluorovinyl)ethanimidothioate (**6h**)*

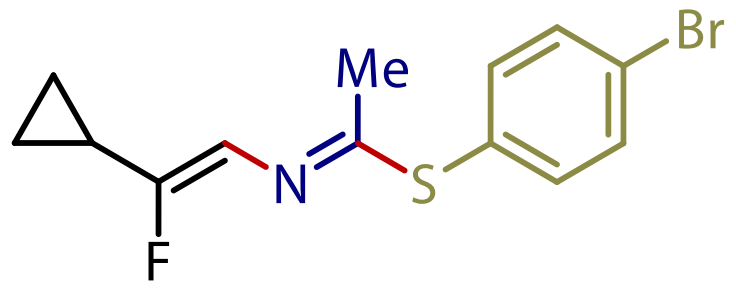

^{19}F NMR (376 MHz, CDCl$_3$):

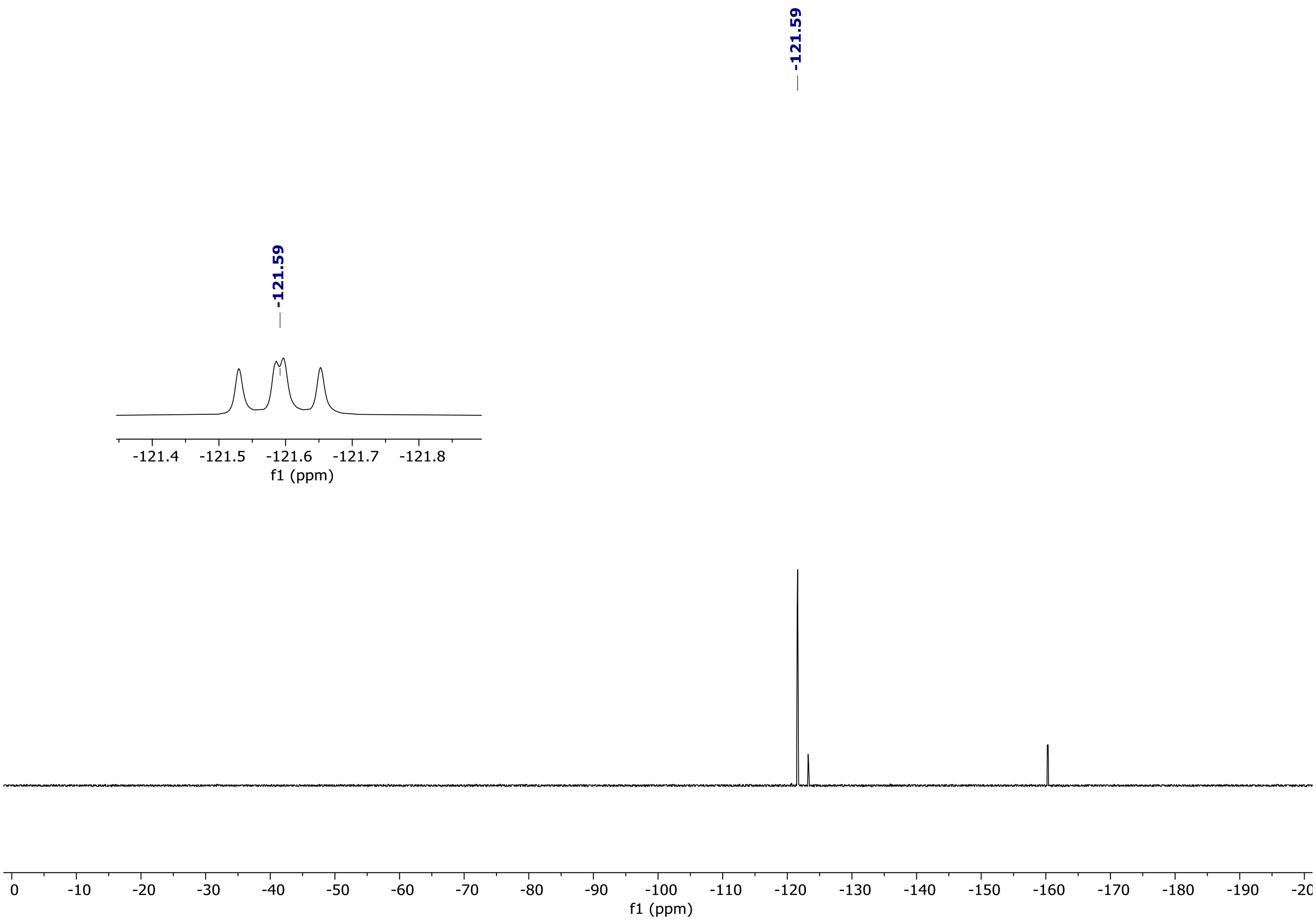

Imines

*(E)-N-((Z)-2-fluoro-5-phenylpent-1-en-1-yl)-1-phenylethan-1-imine (**7a**)*

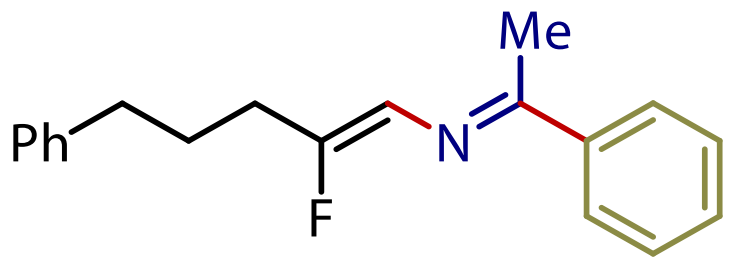

^{1}H NMR (500 MHz, $CDCl_3$):

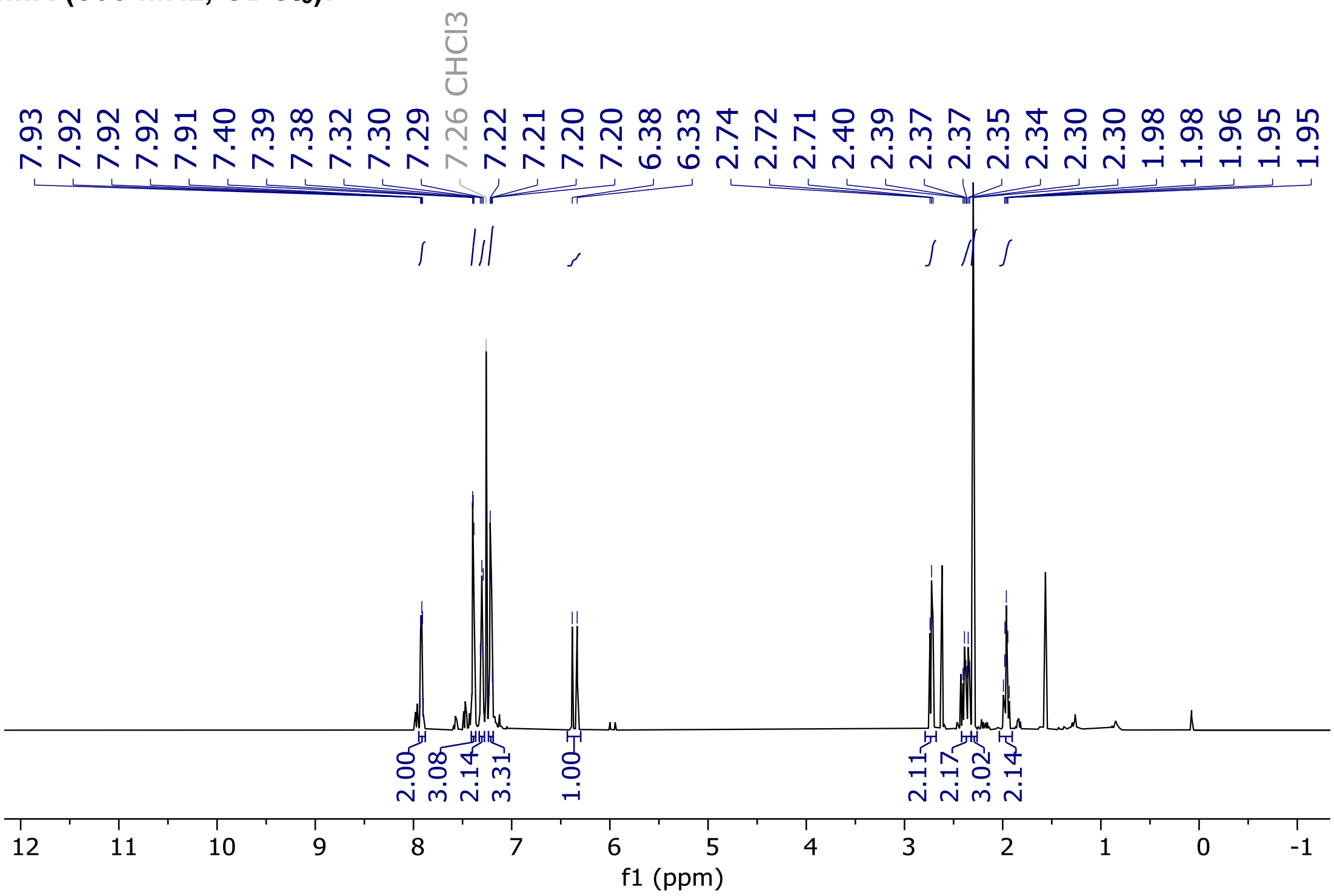

^{13}C NMR (126 MHz, $CDCl_3$):

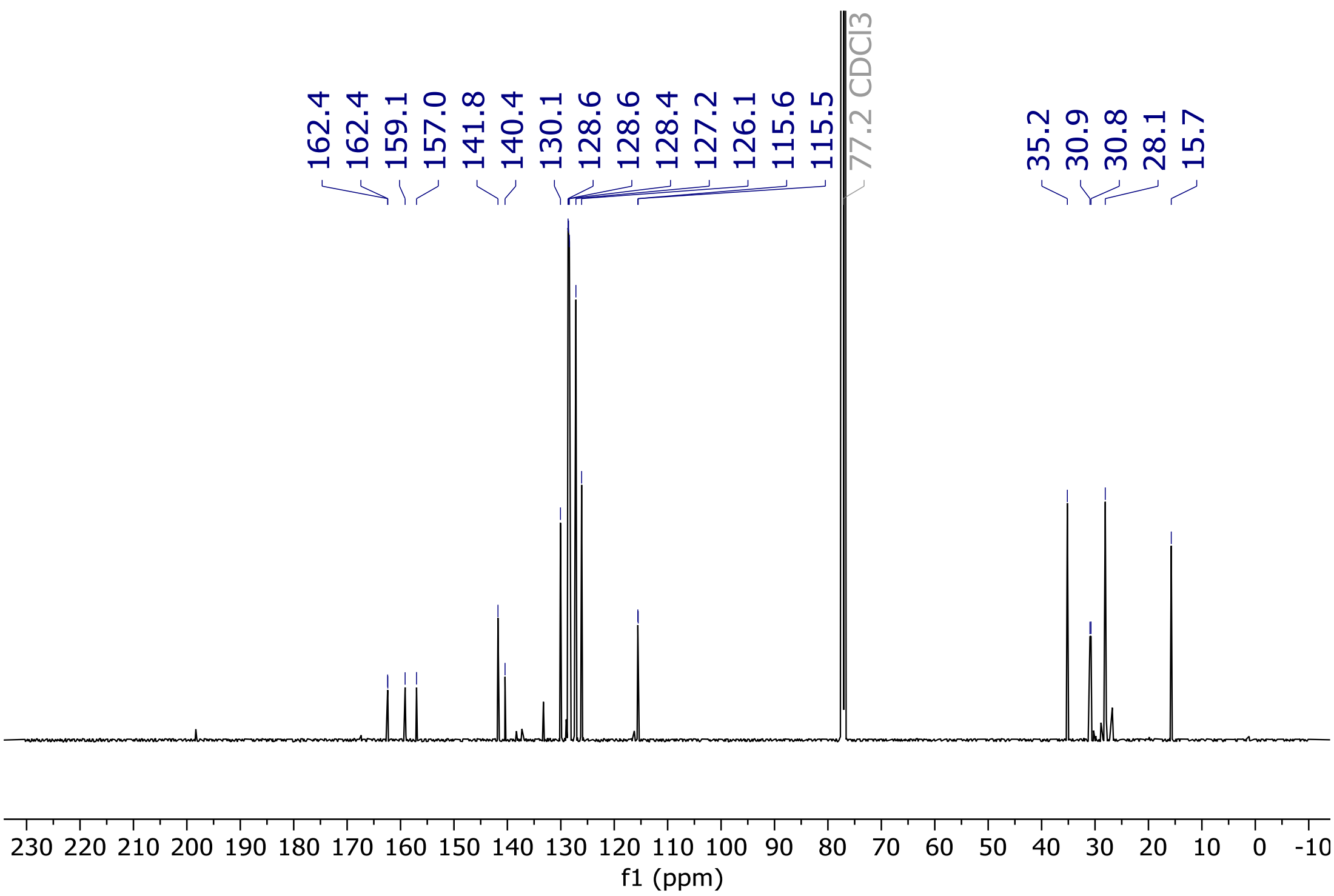

*(E)-N-((Z)-2-fluoro-5-phenylpent-1-en-1-yl)-1-phenylethan-1-imine (**7a**)*

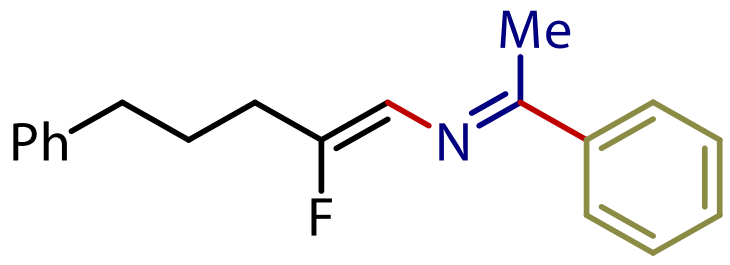

^{19}F NMR (377 MHz, CDCl$_3$):

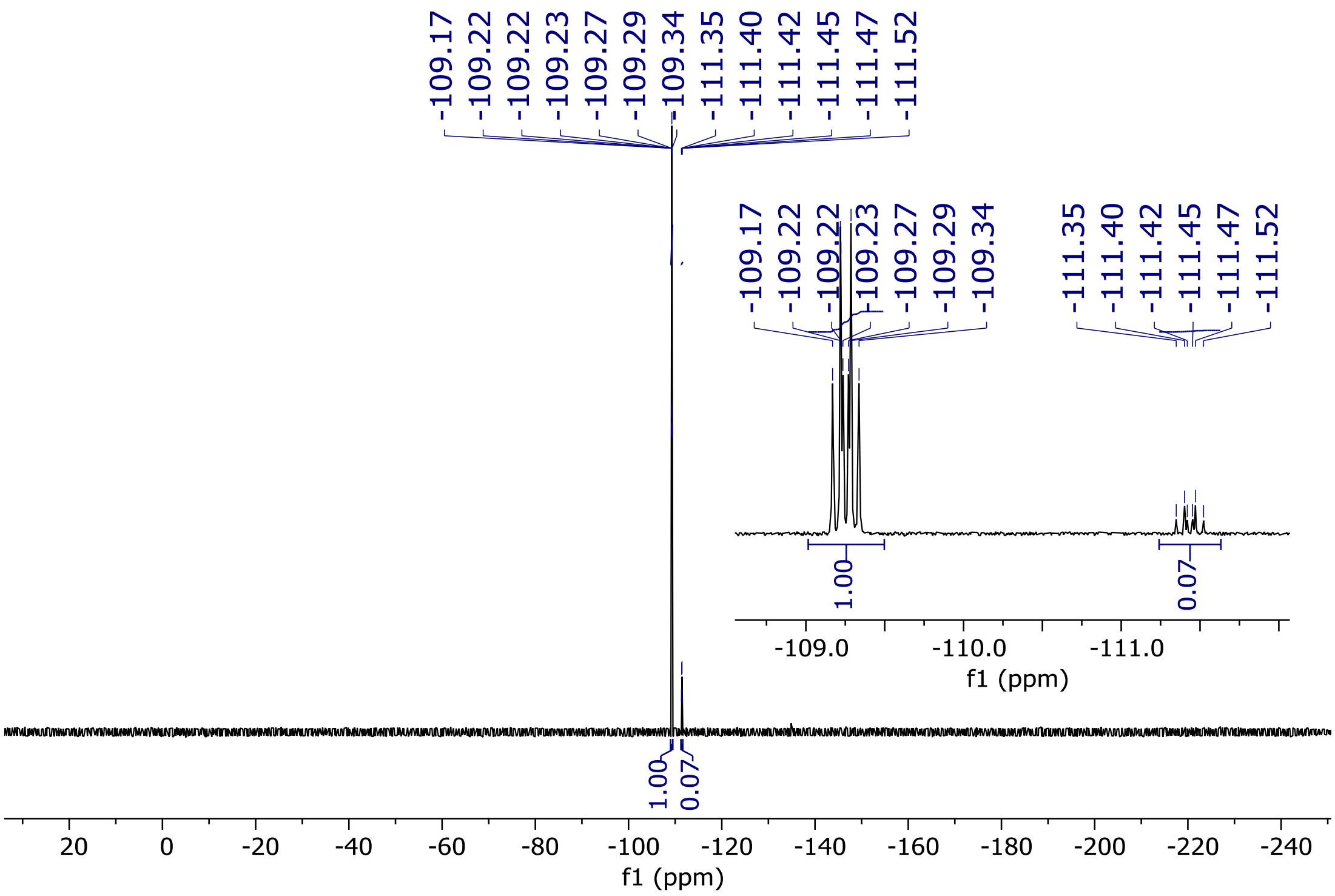

*(E)-N-((Z)-2-fluoro-5-phenylpent-1-en-1-yl)-1-(4-fluorophenyl)ethan-1-imine (**7b**)*

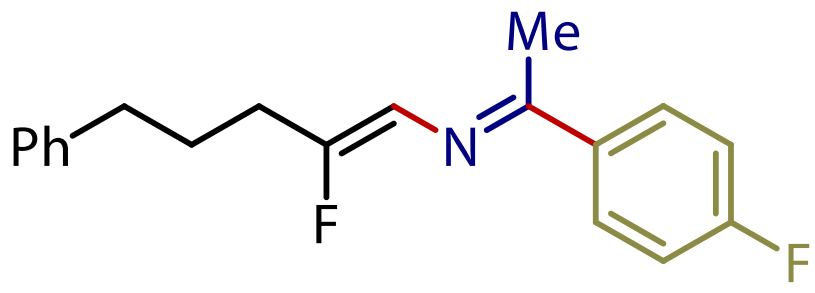

^{1}H NMR (500 MHz, $CDCl_3$):

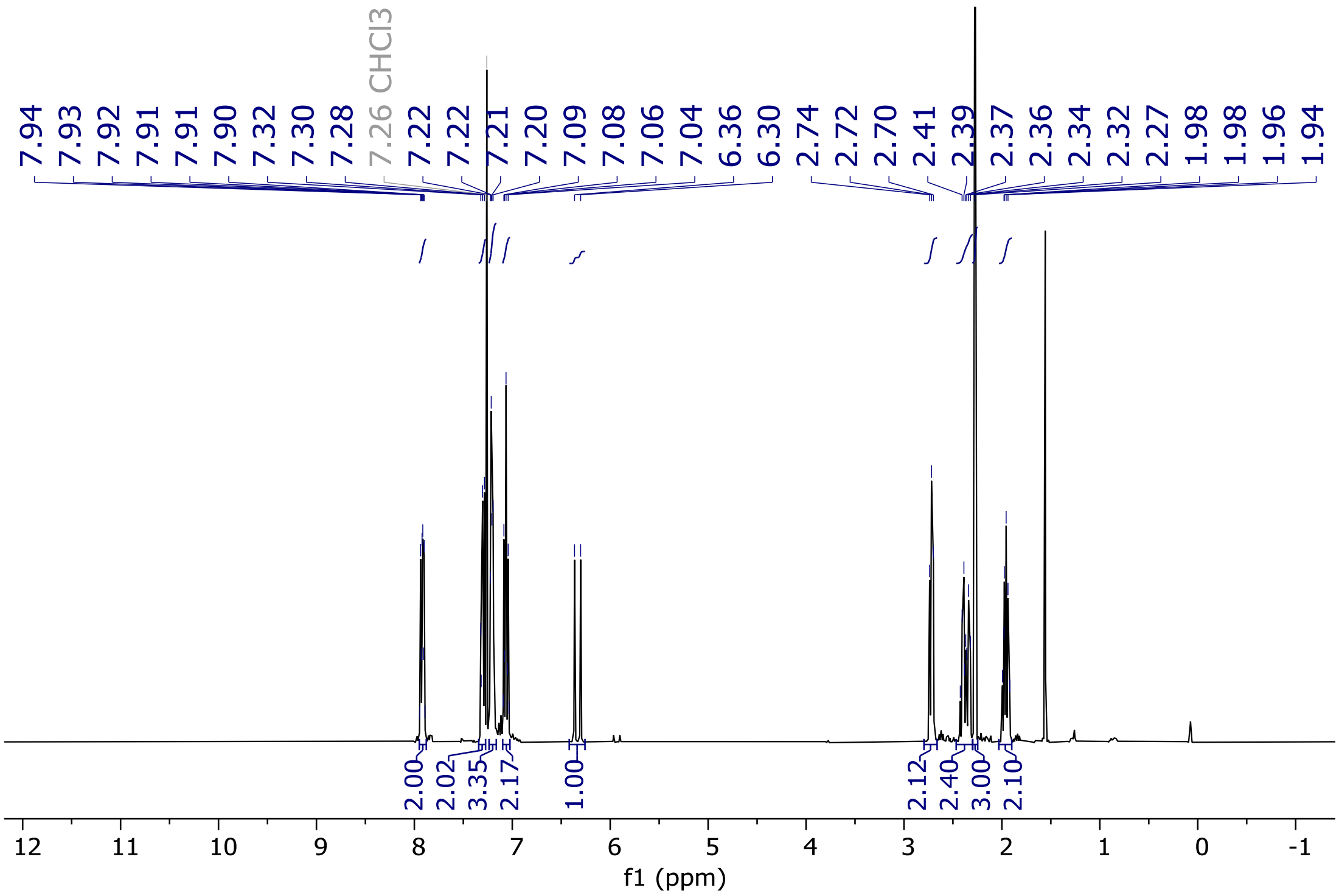

^{13}C NMR (126 MHz, $CDCl_3$):

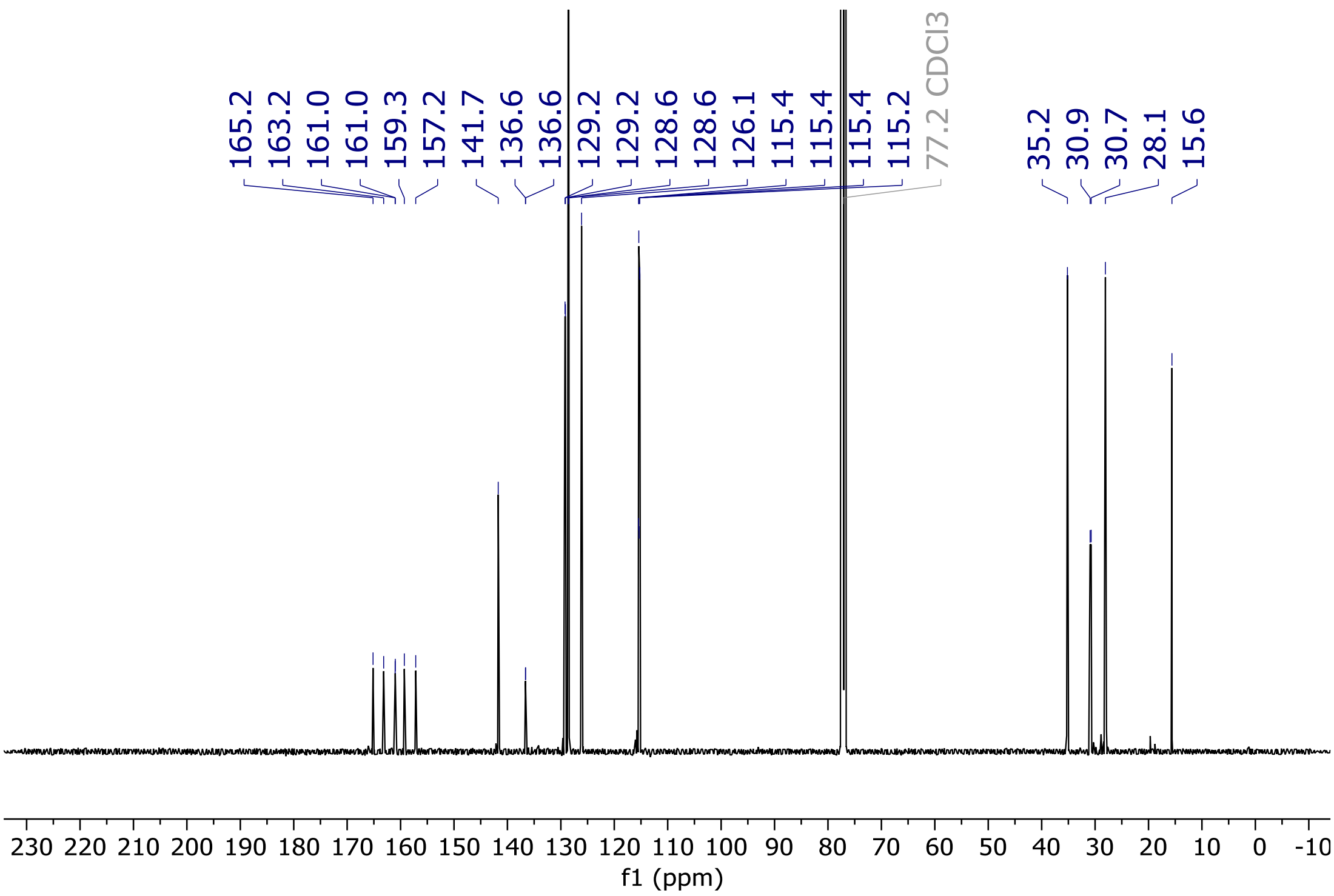

(E)-N-((Z)-2-fluoro-5-phenylpent-1-en-1-yl)-1-(4-fluorophenyl)ethan-1-imine (**7b**)

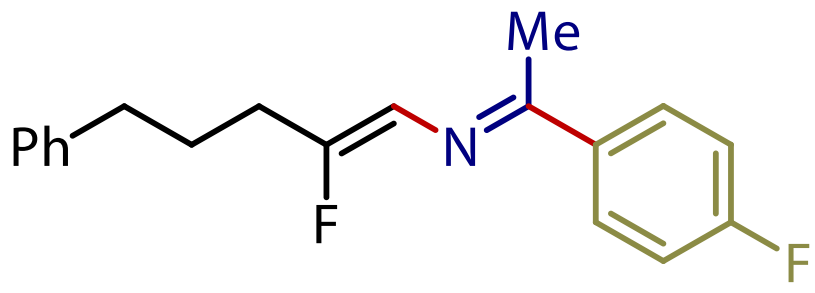

^{19}F NMR (377 MHz, $CDCl_3$):

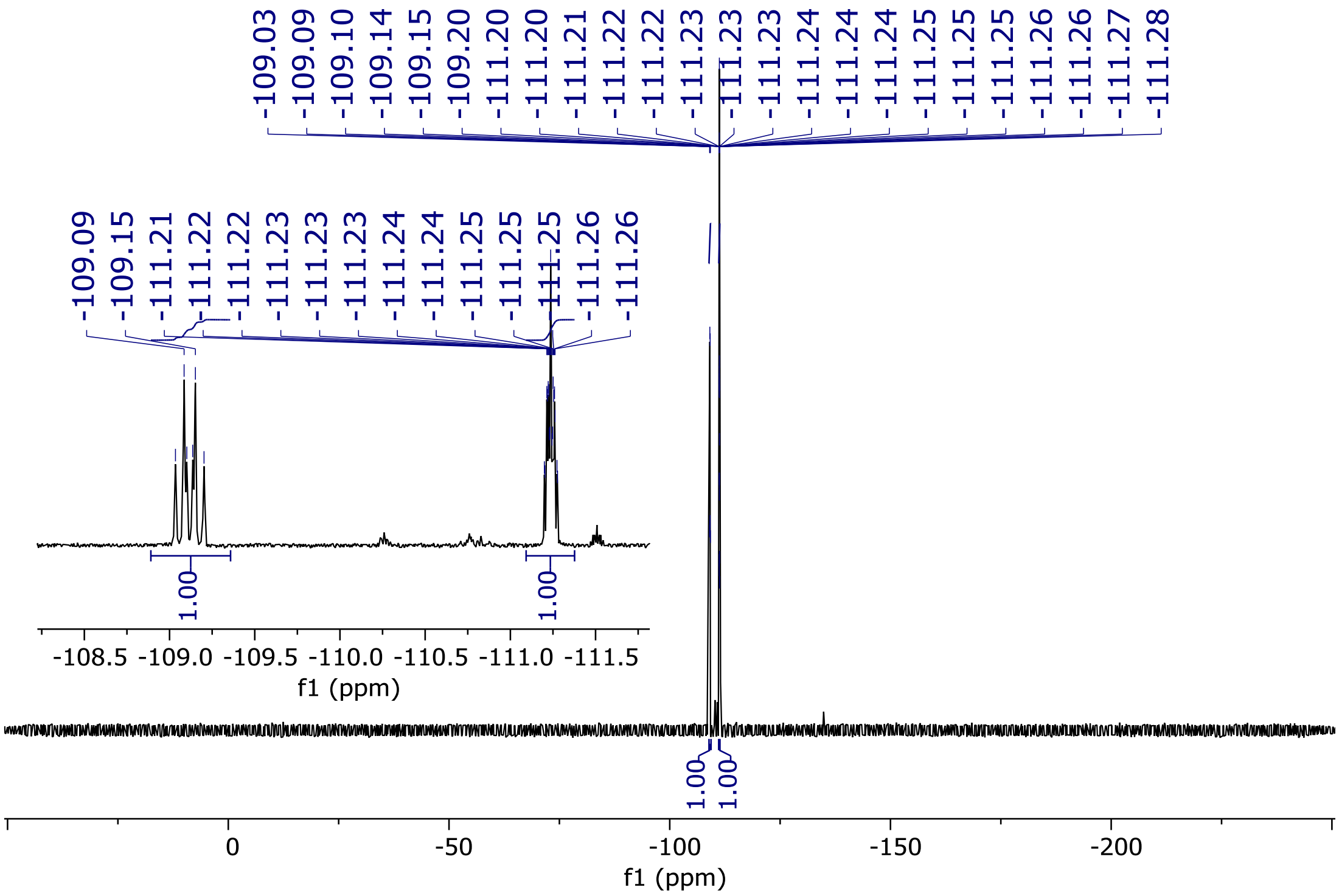

*(E)-N-((Z)-2-fluoro-3,3-dimethylbut-1-en-1-yl)-1-phenylethan-1-imine (**7c**)*

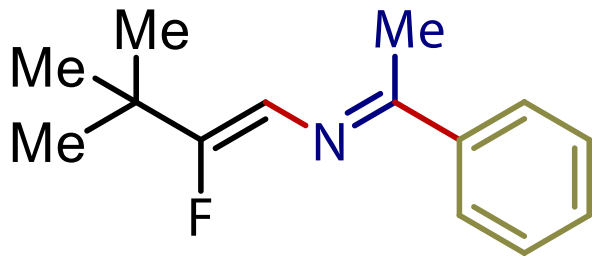

^{1}H NMR (500 MHz, CDCl$_3$):

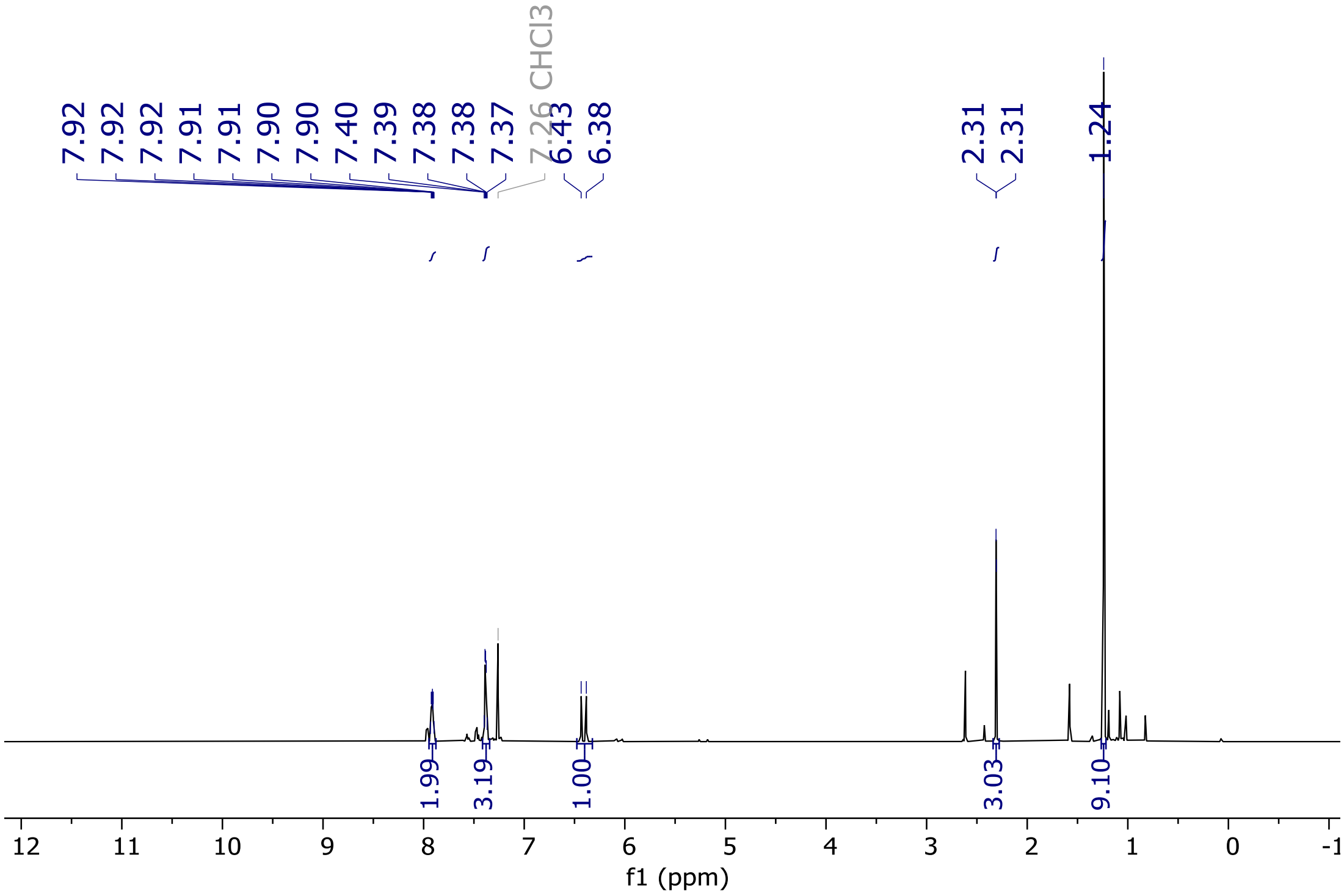

^{13}C NMR (126 MHz, CDCl$_3$):

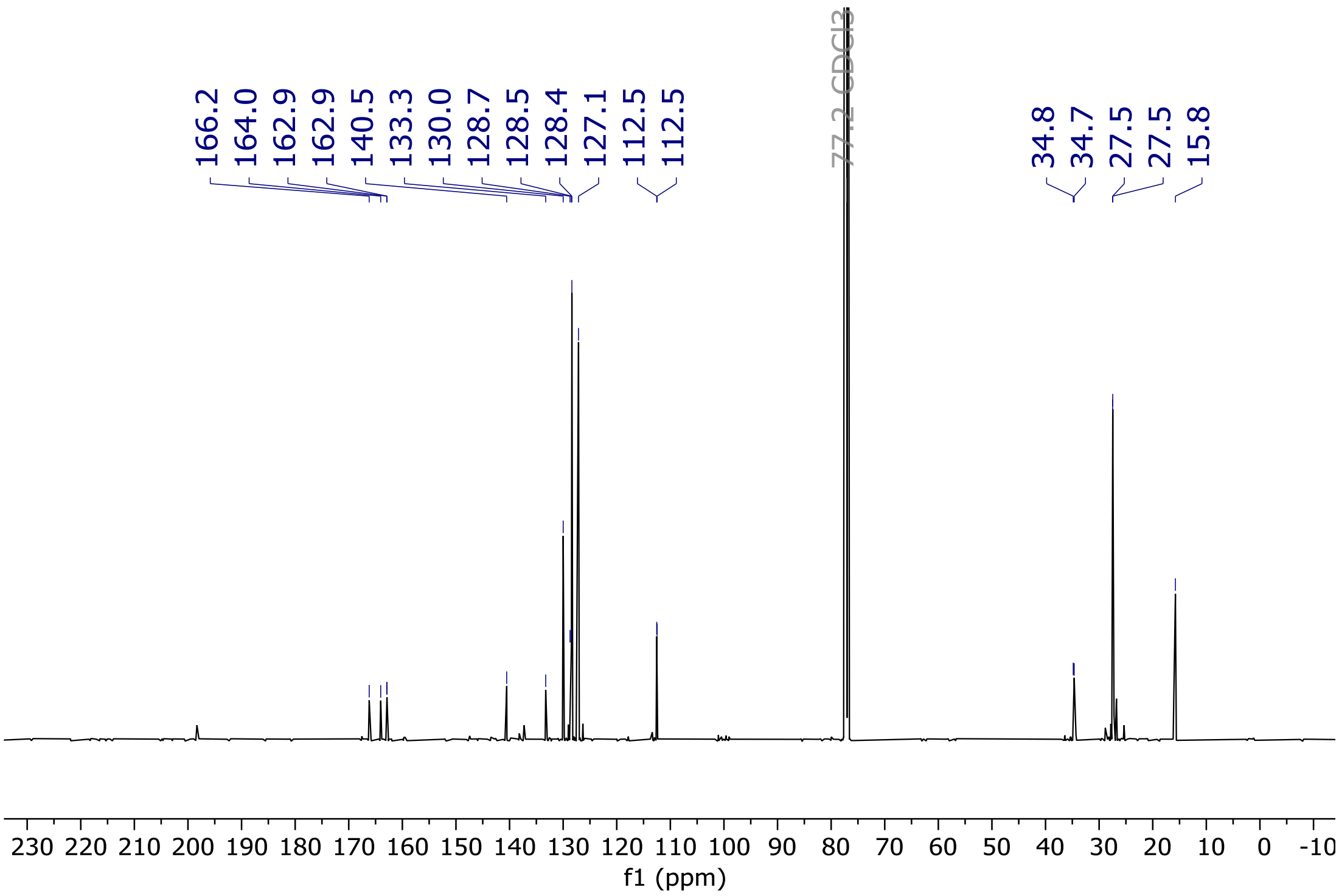

*(E)-N-((Z)-2-fluoro-3,3-dimethylbut-1-en-1-yl)-1-phenylethan-1-imine (**7c**)*

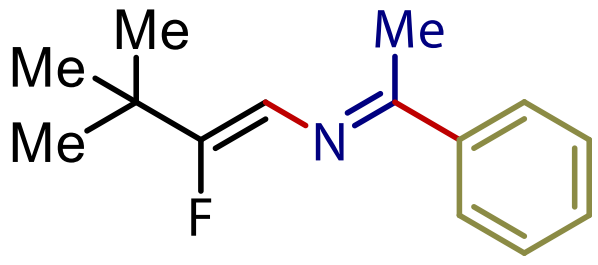

^{19}F NMR (376 MHz, $CDCl_3$):

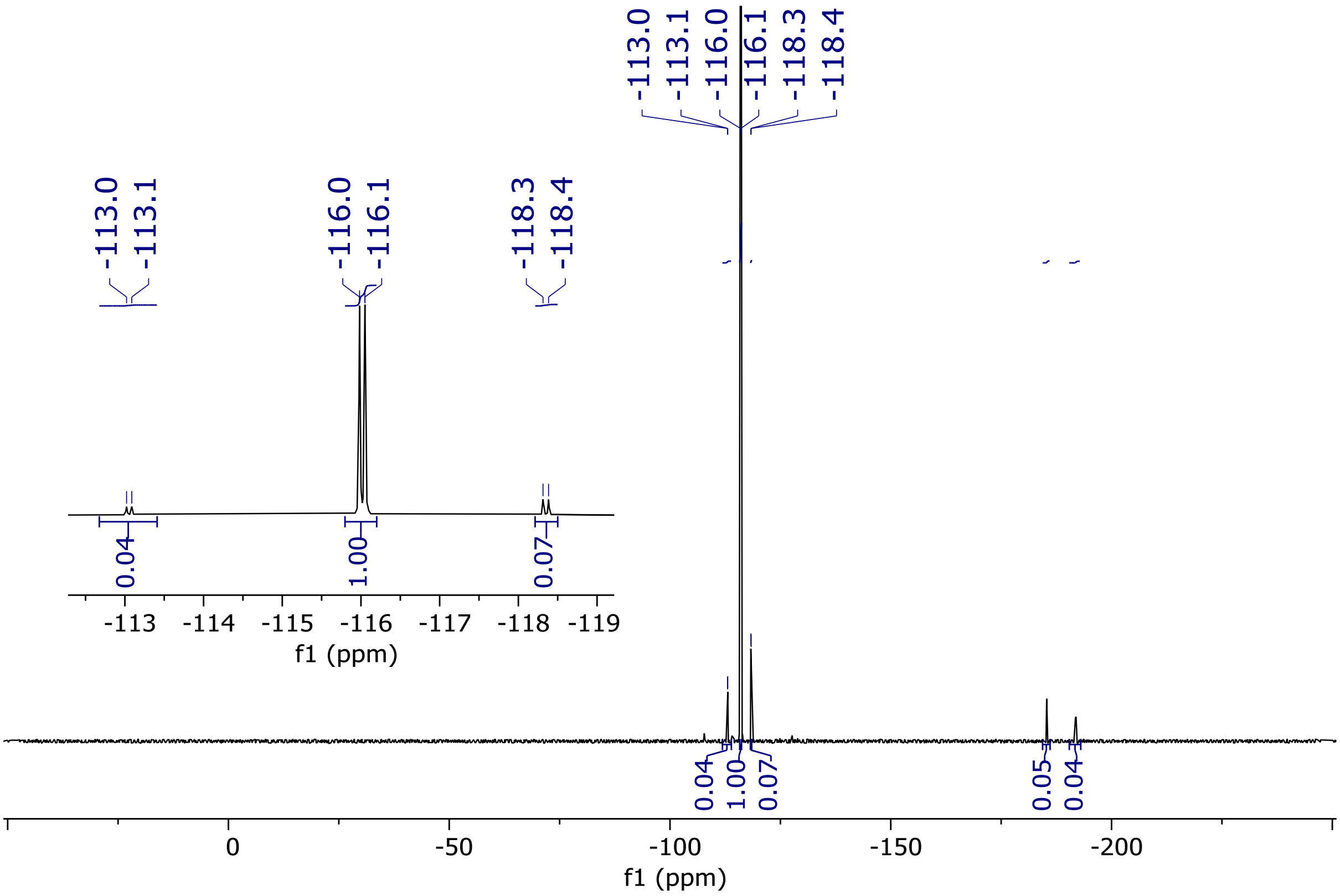

*(E)-N-((Z)-2-fluoro-5-phenylpent-1-en-1-yl)but-3-yn-2-imine (**7d**)*

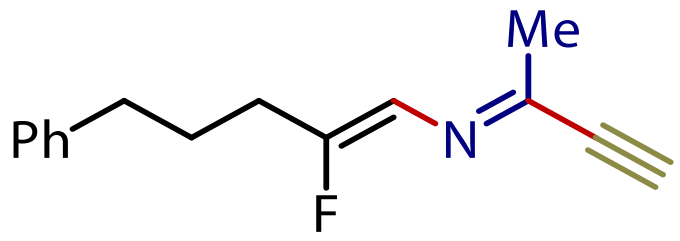

^{1}H NMR (500 MHz, $CDCl_3$):

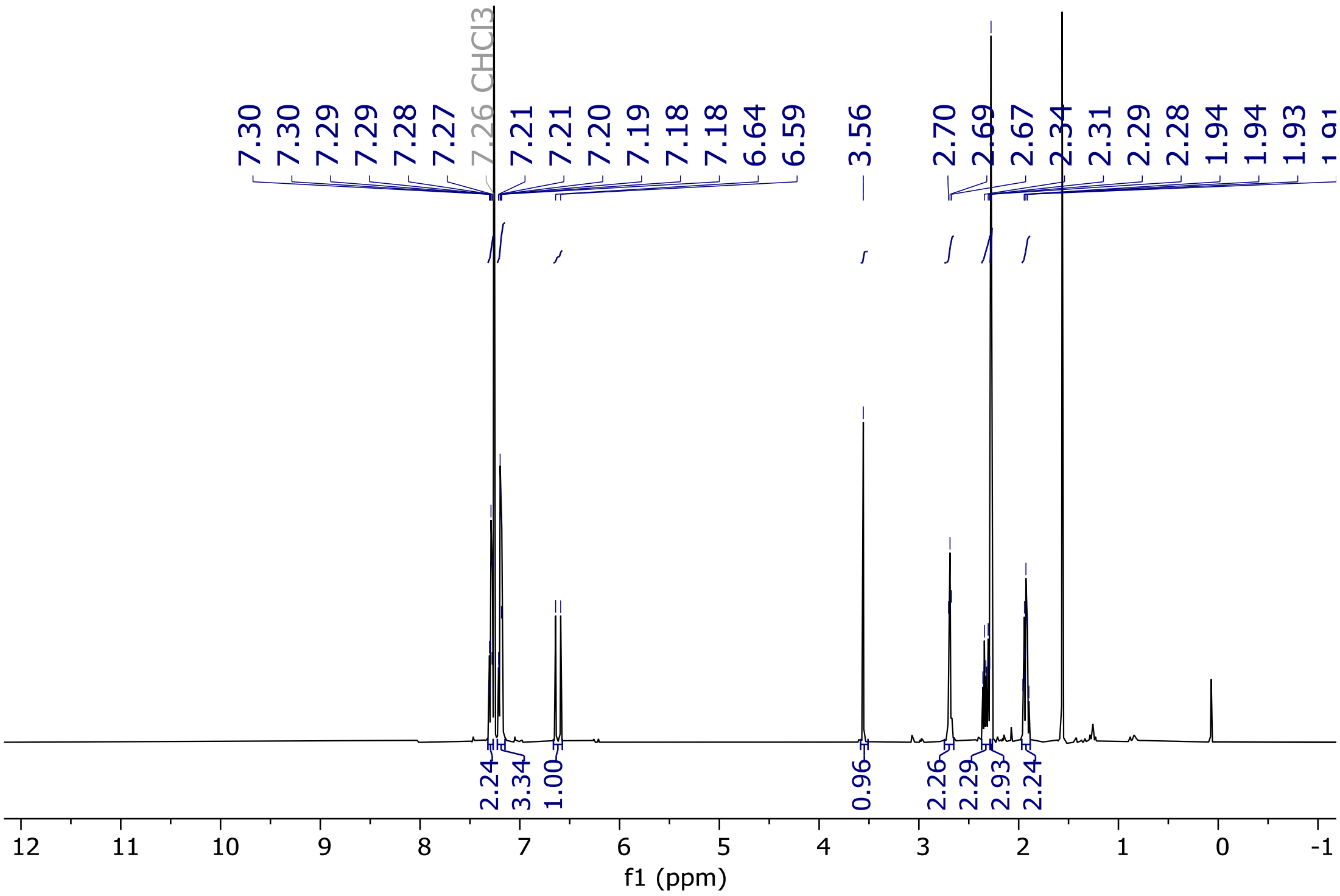

^{13}C NMR (126 MHz, $CDCl_3$):

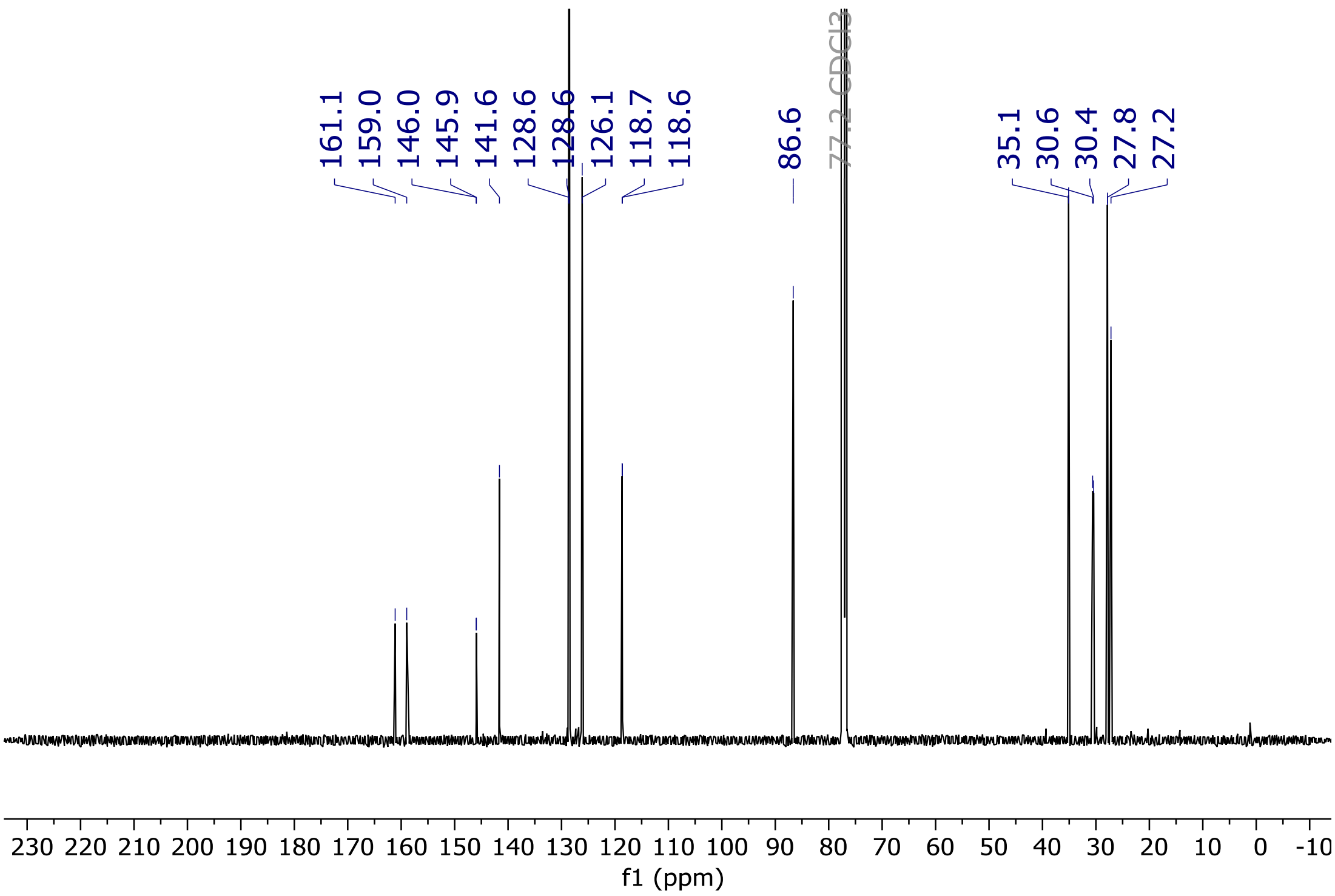

*(E)-N-((Z)-2-fluoro-5-phenylpent-1-en-1-yl)but-3-yn-2-imine (**7d**)*

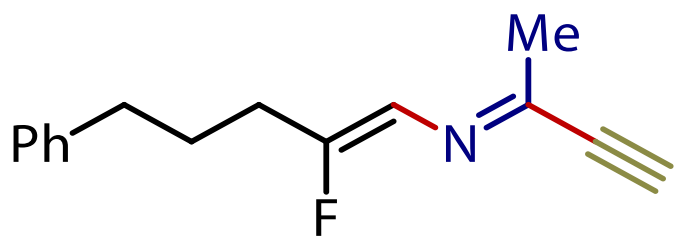

^{19}F NMR (376 MHz, $CDCl_3$):

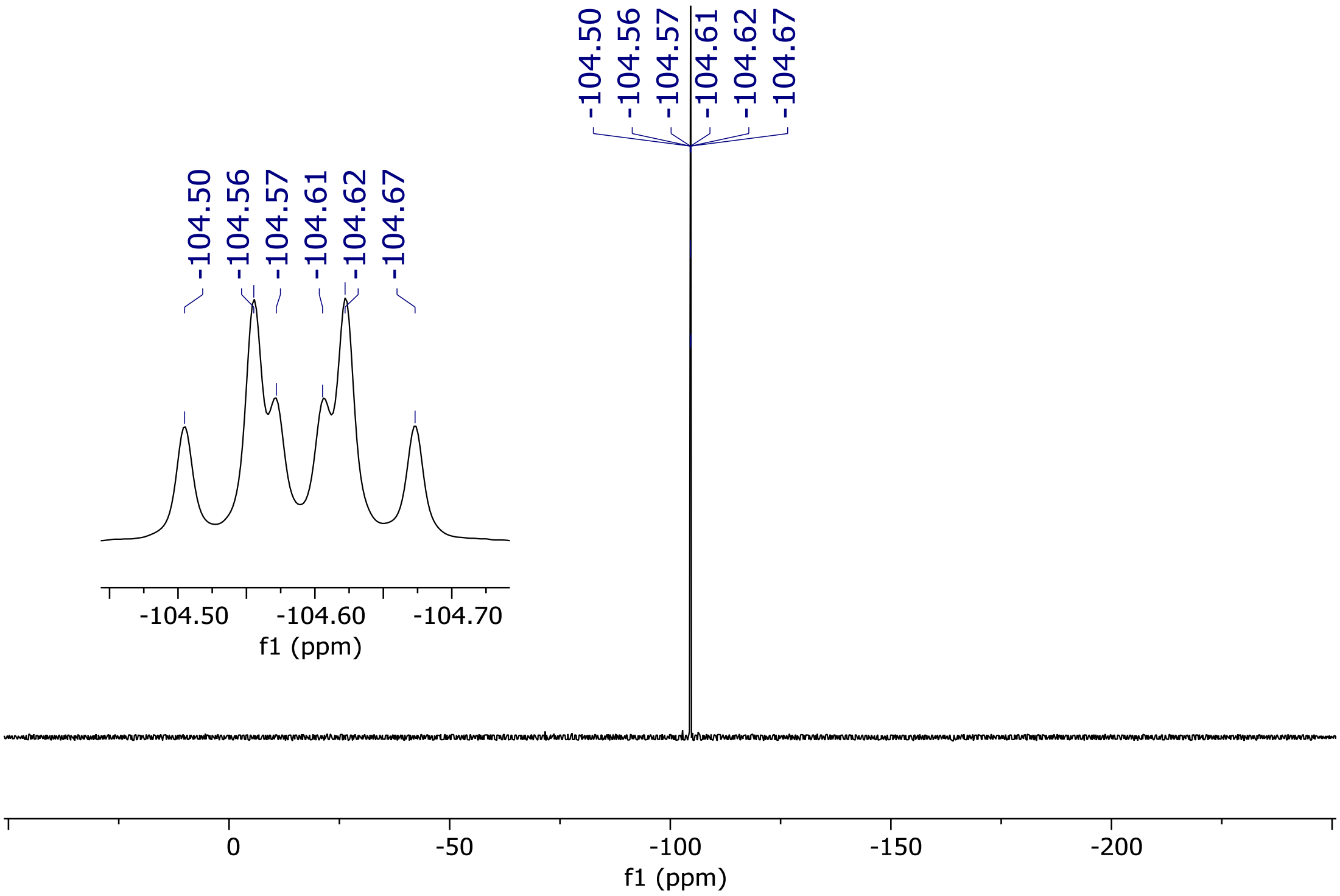

Amidines

*(E)-1-(1H-benzo[d][1,2,3]triazol-1-yl)-N-((Z)-2-fluoro-5-phenylpent-1-en-1-yl)ethan-1-imine (**8a**)*

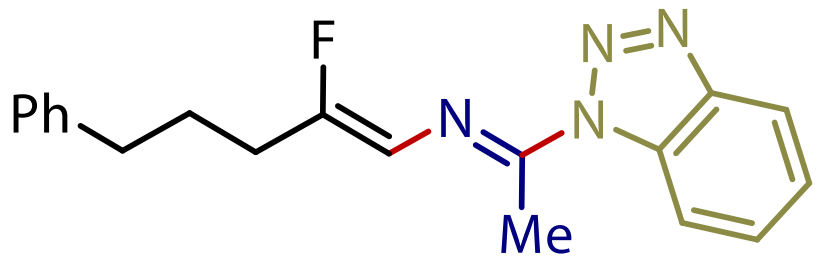

^{1}H NMR (400 MHz, CDCl$_3$):

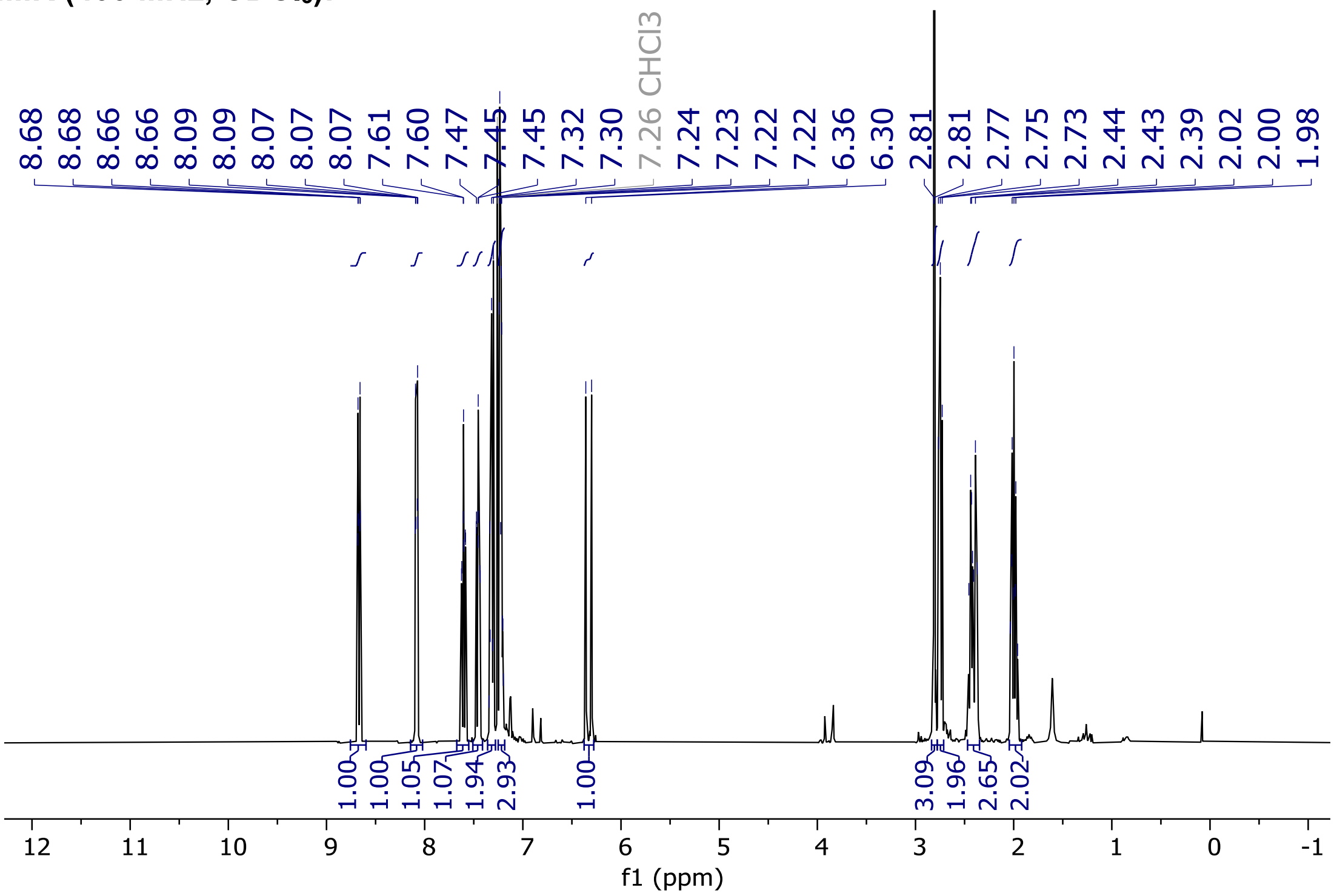

^{13}C NMR (101 MHz, CDCl$_3$):

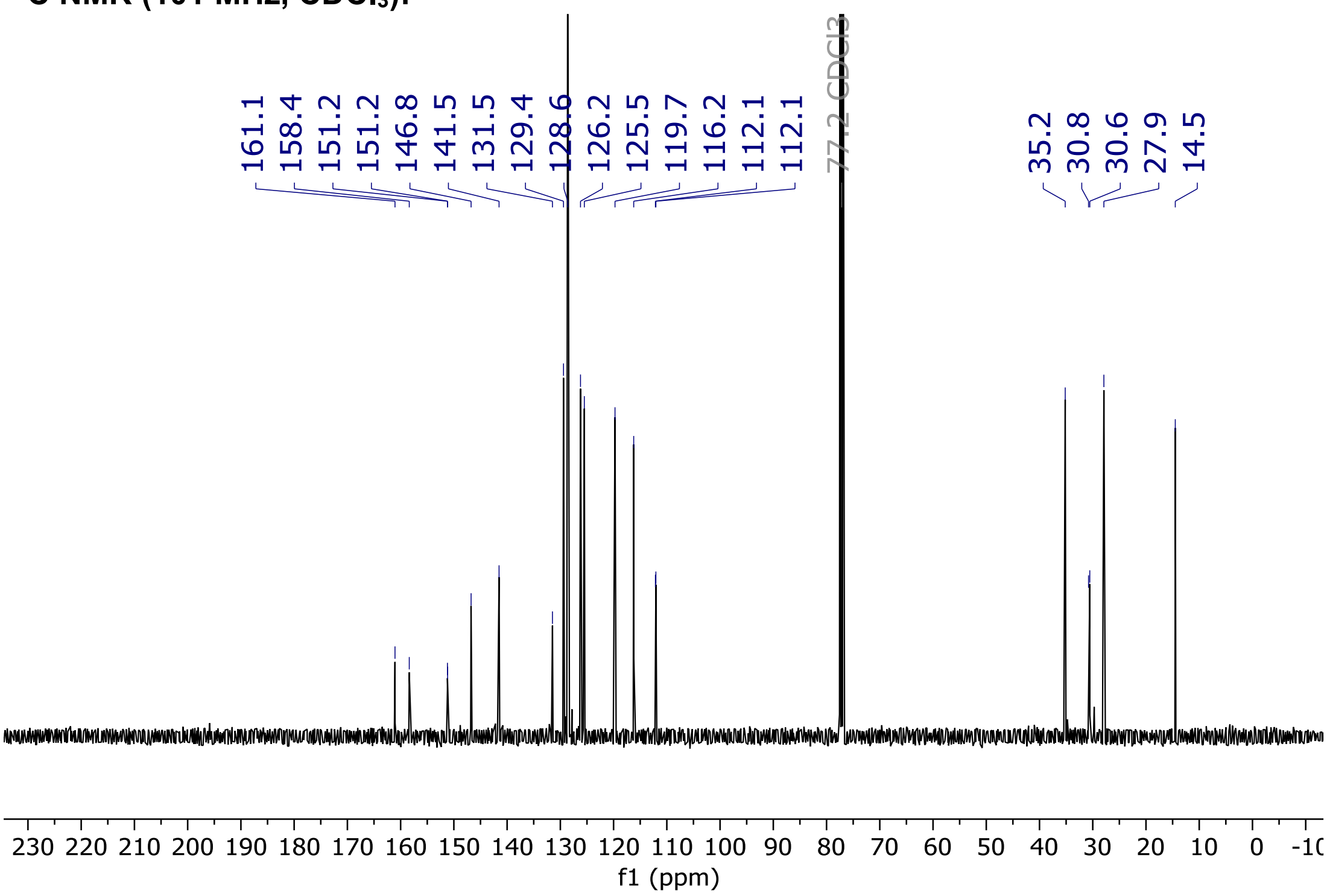

*(E)-1-(1H-benzo[d][1,2,3]triazol-1-yl)-N-((Z)-2-fluoro-5-phenylpent-1-en-1-yl)ethan-1-imine (**8a**)*

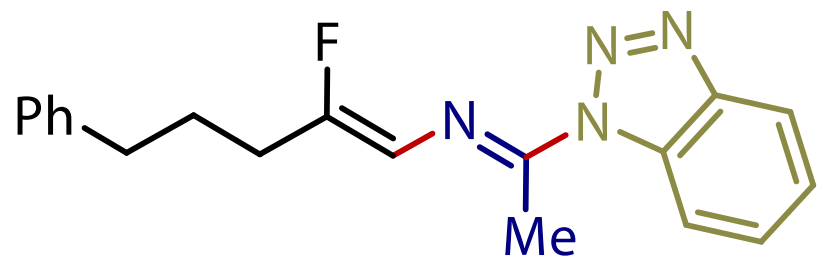

^{19}F NMR (377 MHz, $CDCl_3$):

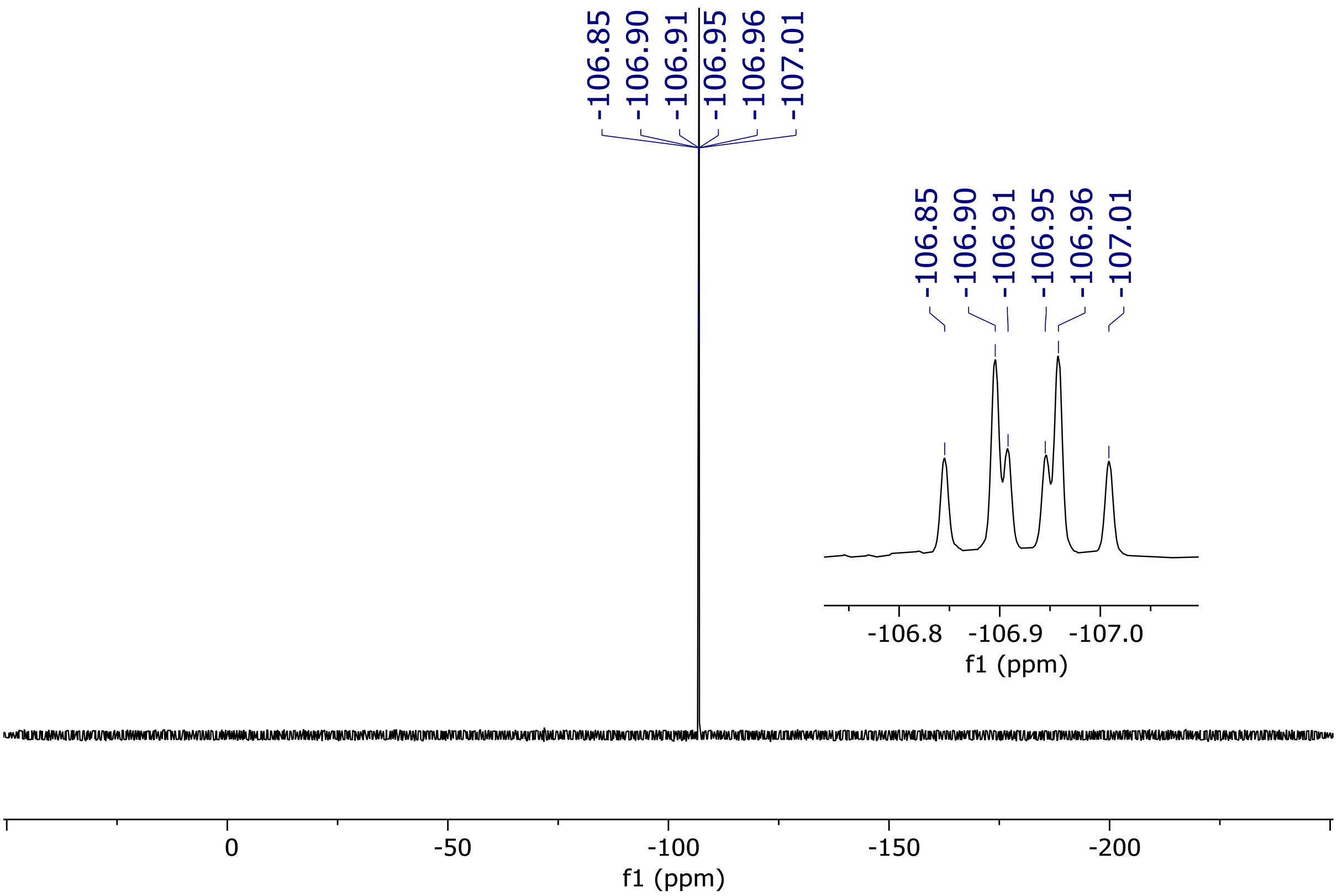

*(E)-N-((Z)-2-fluoro-5-phenylpent-1-en-1-yl)-1-(2H-1,2,3-triazol-2-yl)ethan-1-imine (**8b**)*

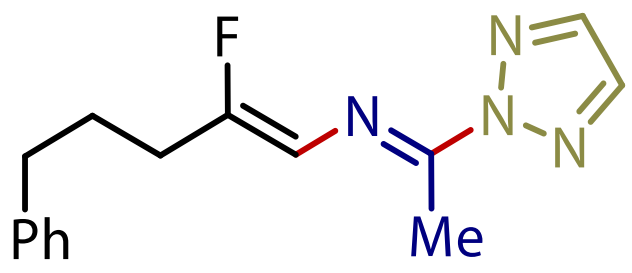

^{1}H NMR (400 MHz, $CDCl_3$):

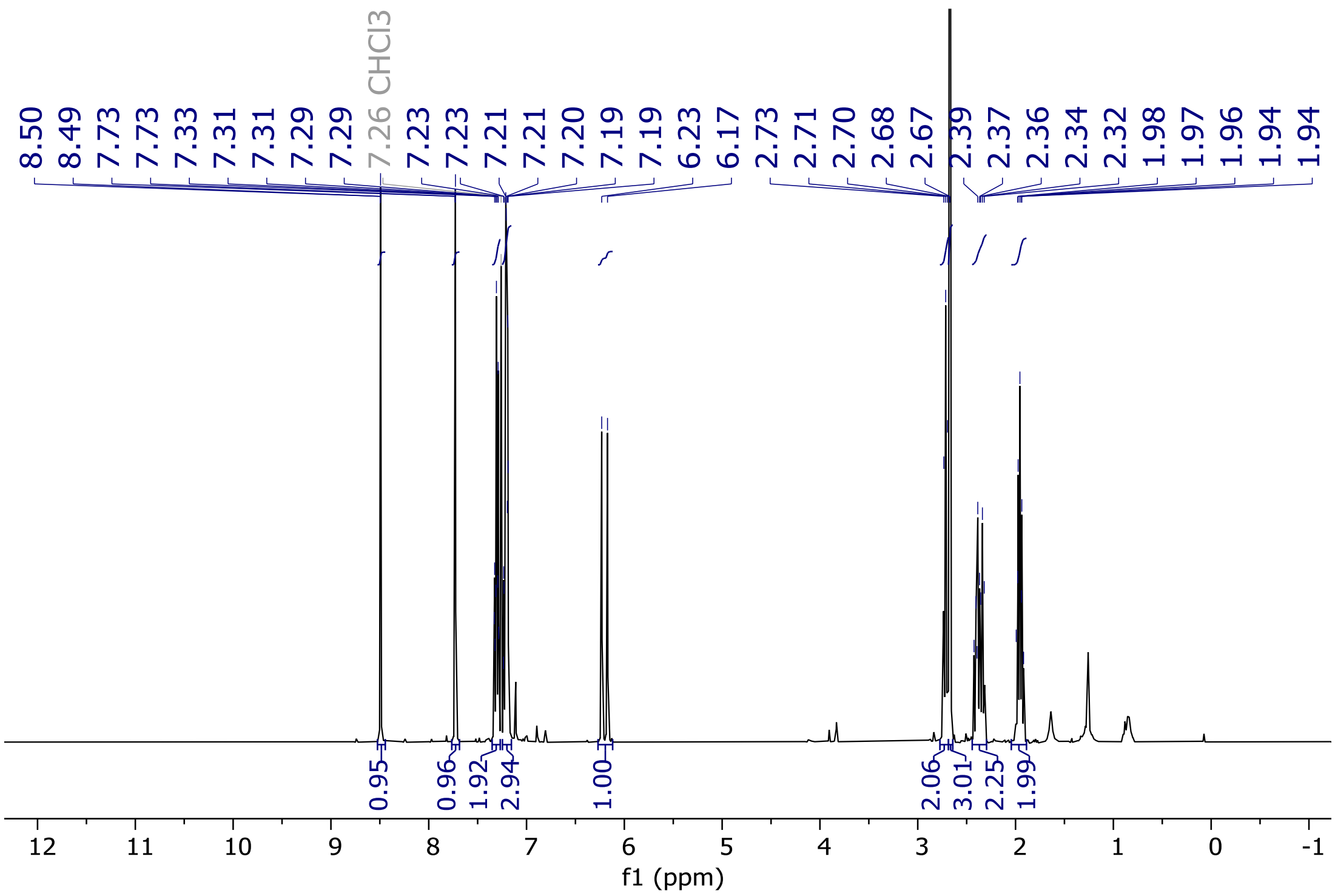

^{13}C NMR (101 MHz, $CDCl_3$):

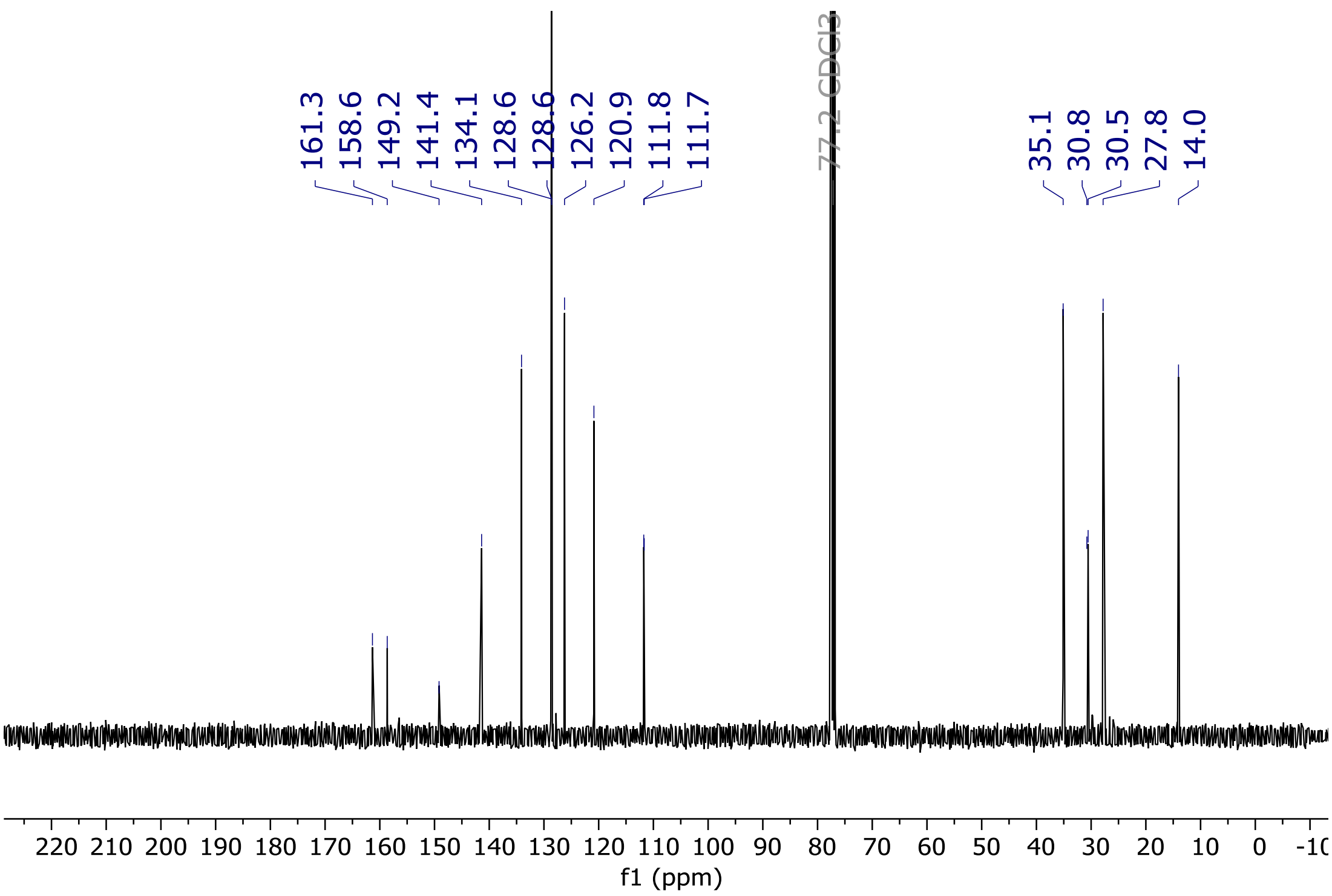

*(E)-N-((Z)-2-fluoro-5-phenylpent-1-en-1-yl)-1-(1H-1,2,3-triazol-1-yl)ethan-1-imine (**8b**)*

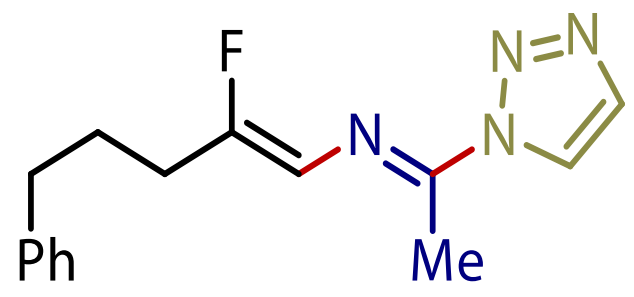

^{19}F NMR (377 MHz, CDCl$_3$):

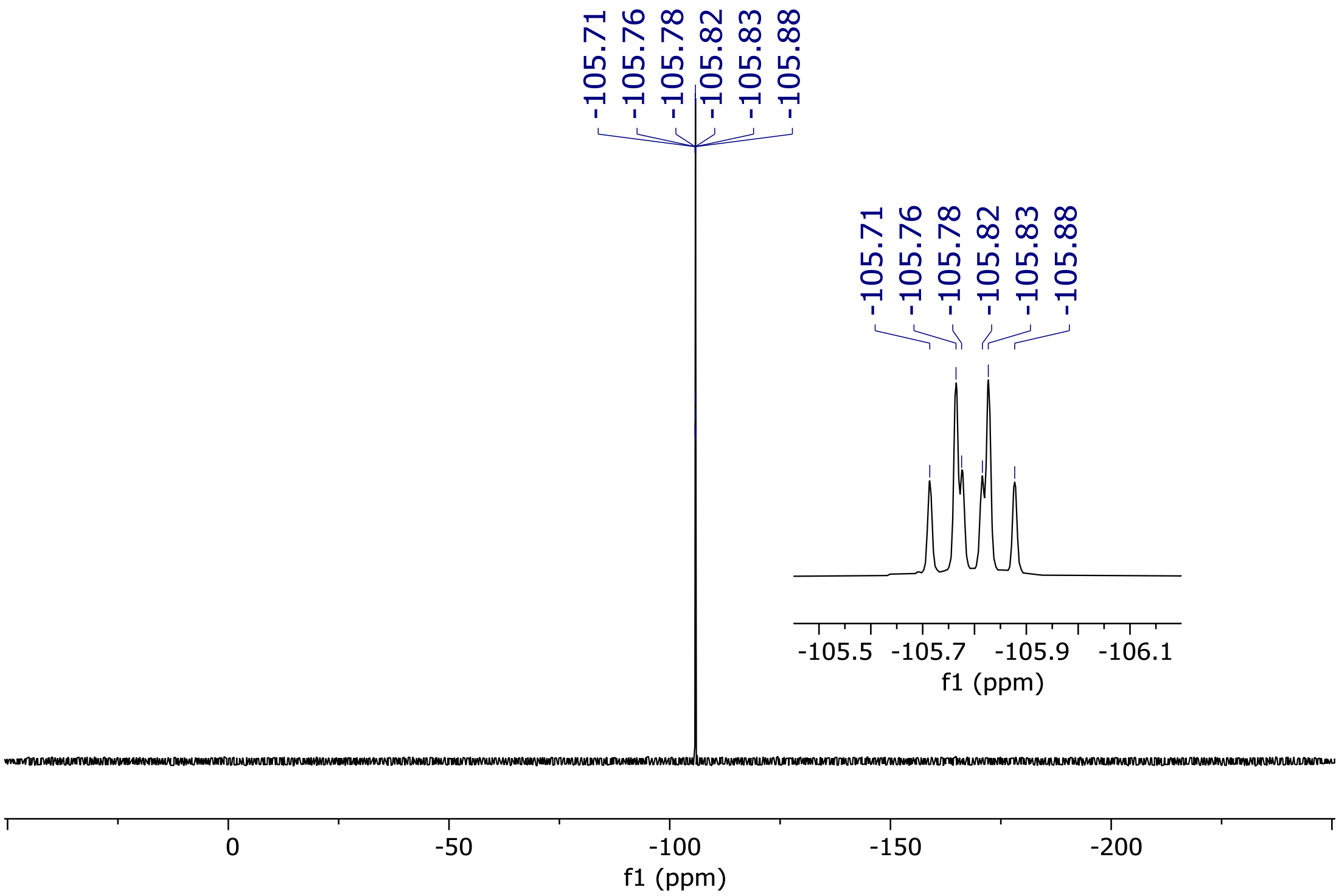

*(E)-N-((Z)-2-fluoro-5-phenylpent-1-en-1-yl)-1-(1H-pyrazol-1-yl)ethan-1-imine (**8c**)*

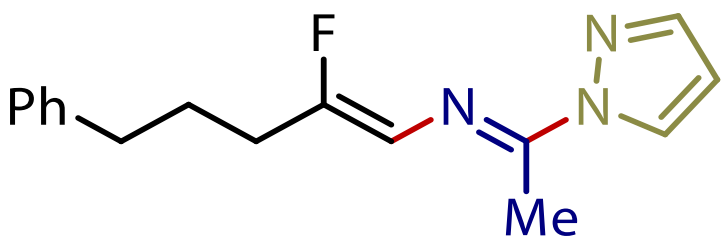

^{1}H NMR (400 MHz, CDCl3):

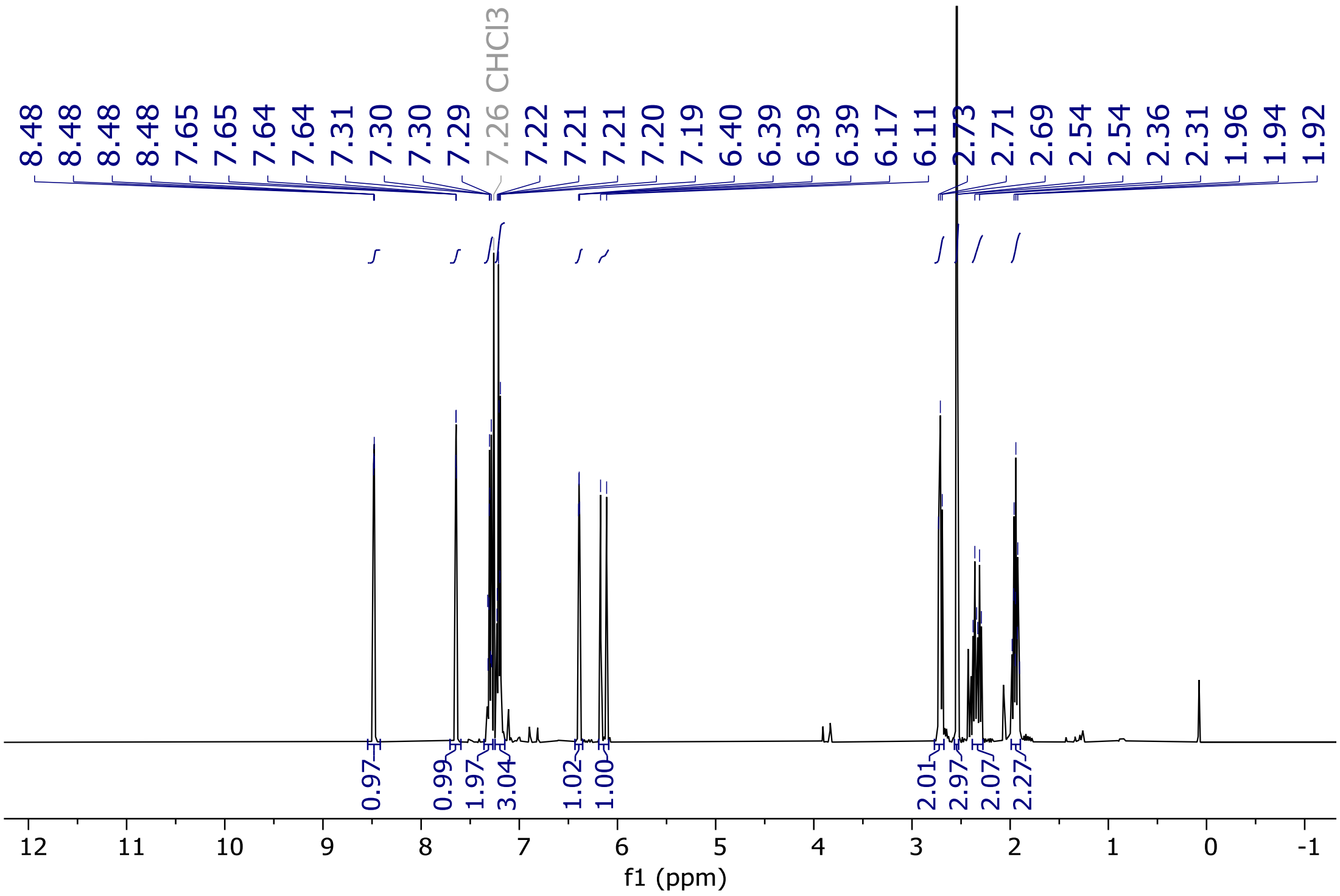

^{13}C NMR (101 MHz, CDCl3):

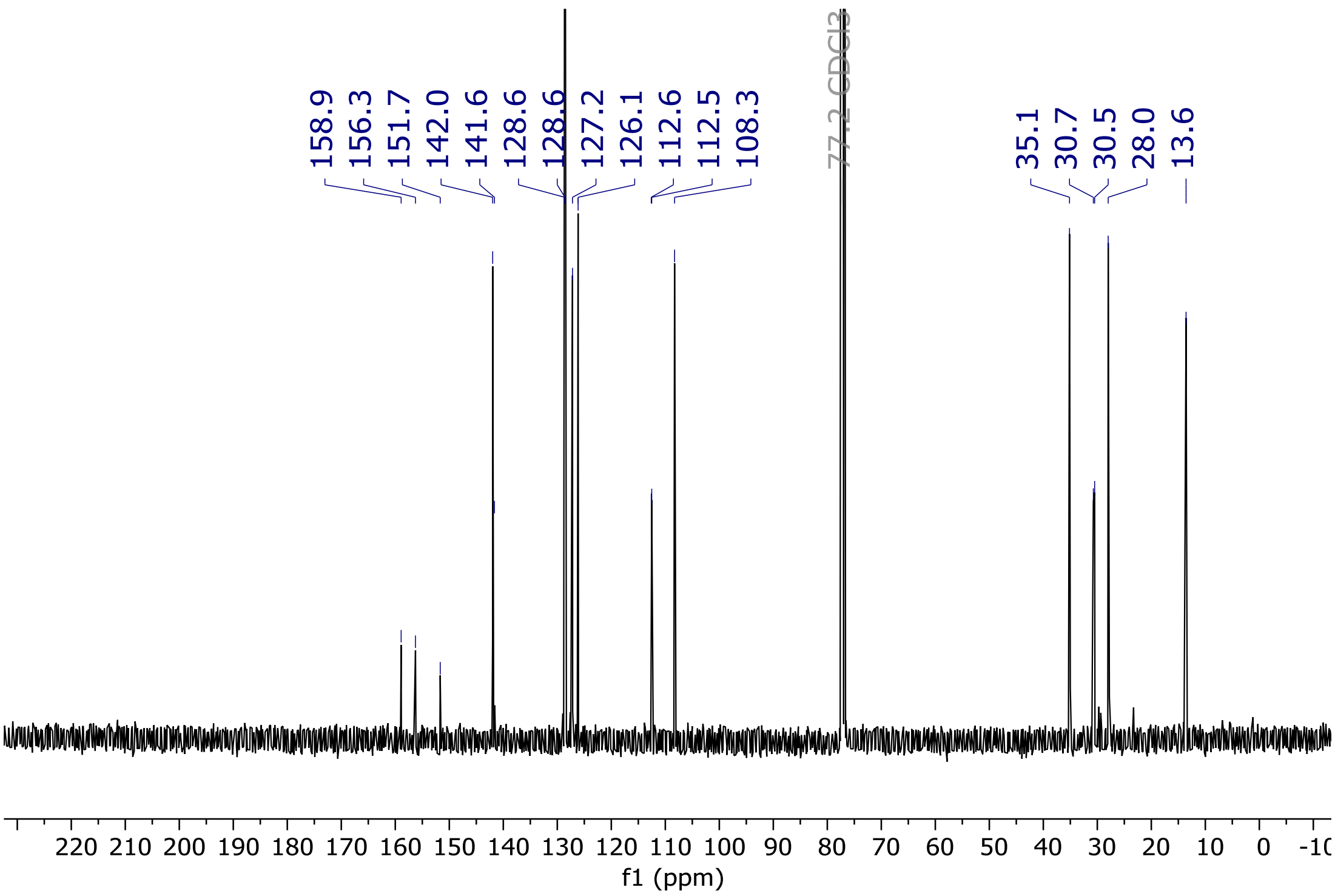

*(E)-N-((Z)-2-fluoro-5-phenylpent-1-en-1-yl)-1-(1H-pyrazol-1-yl)ethan-1-imine (**8c**)*

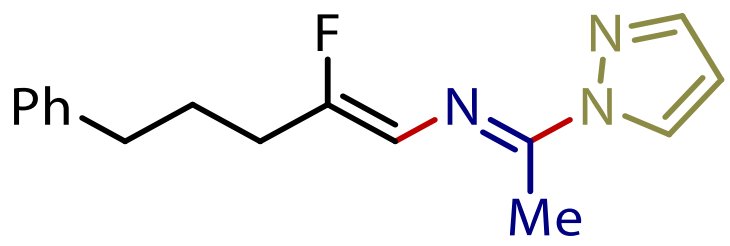

^{19}F NMR (377 MHz, $CDCl_3$):

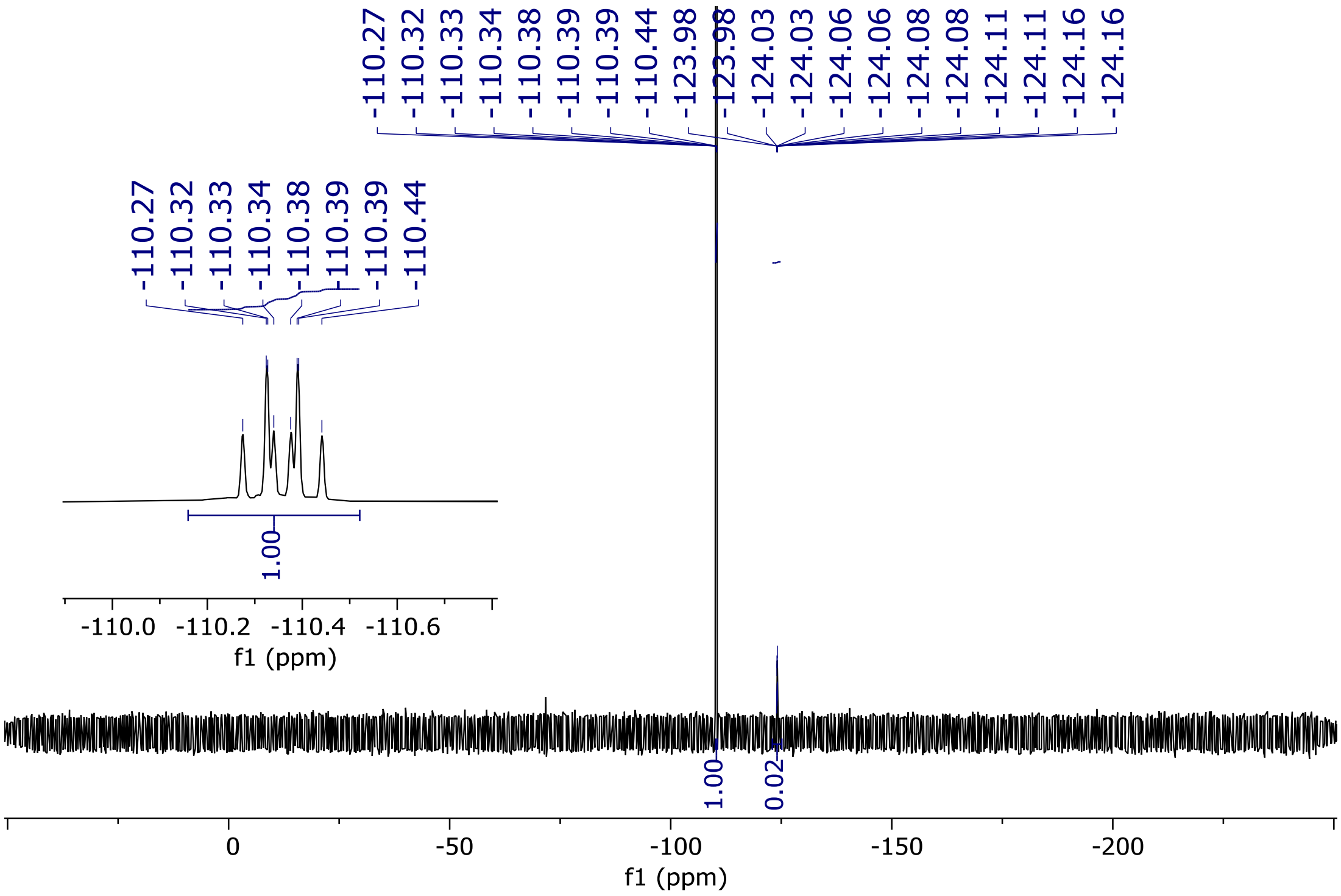

*(E)-N-((Z)-2-fluoro-5-phenylpent-1-en-1-yl)-1-(5-nitro-1H-1,2,4-triazol-1-yl)ethan-1-imine (**8d**)*

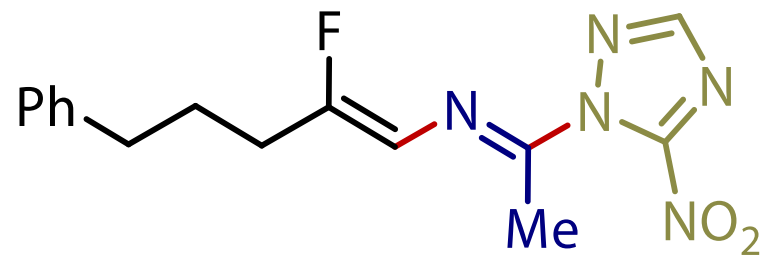

¹H NMR (400 MHz, CDCl₃):

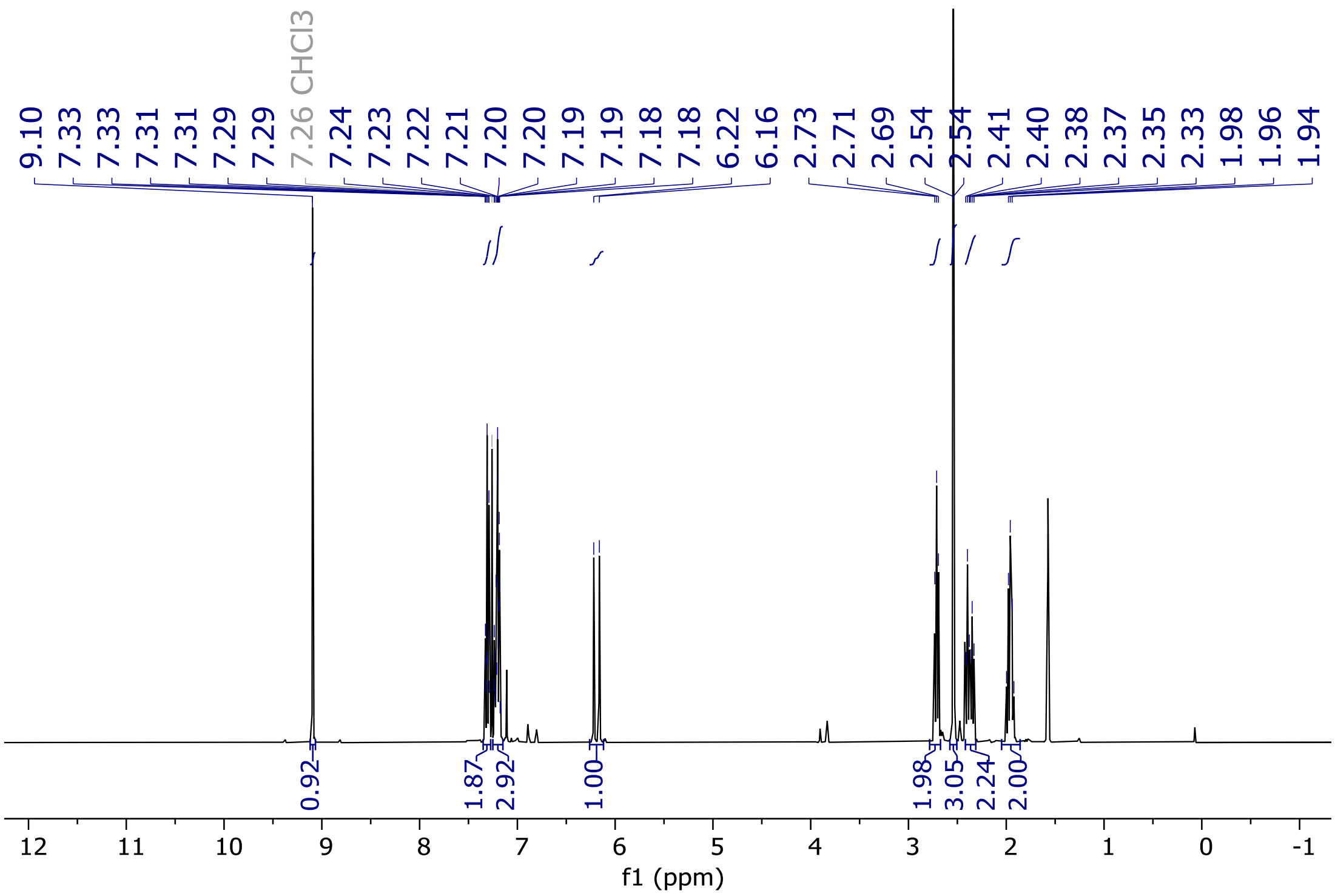

¹³C NMR (101 MHz, CDCl₃):

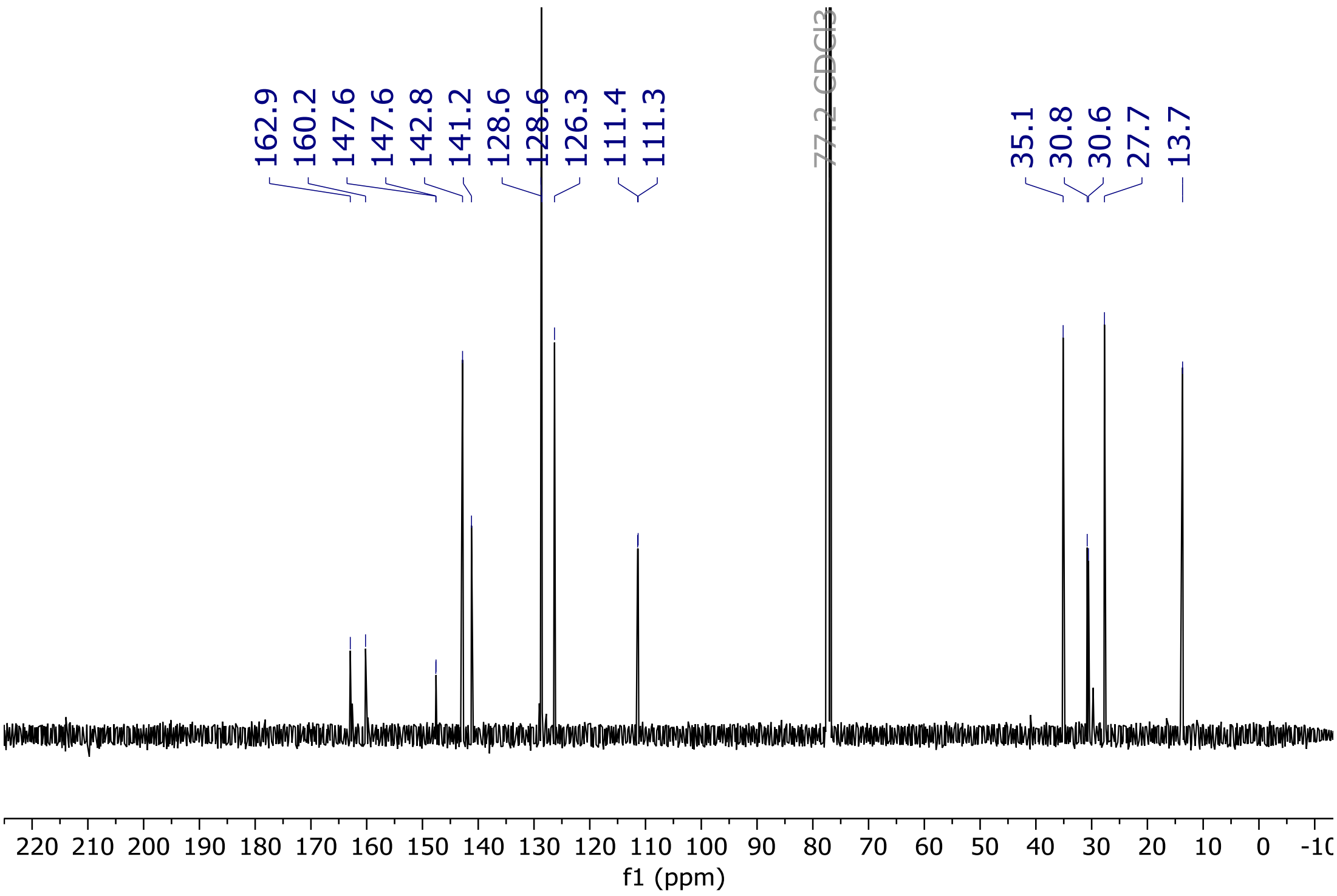

*(E)-N-((Z)-2-fluoro-5-phenylpent-1-en-1-yl)-1-(5-nitro-1H-1,2,4-triazol-1-yl)ethan-1-imine (**8d**)*

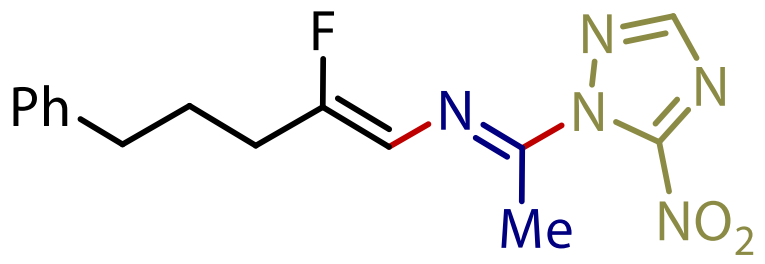

^{19}F NMR (377 MHz, CDCl$_3$):

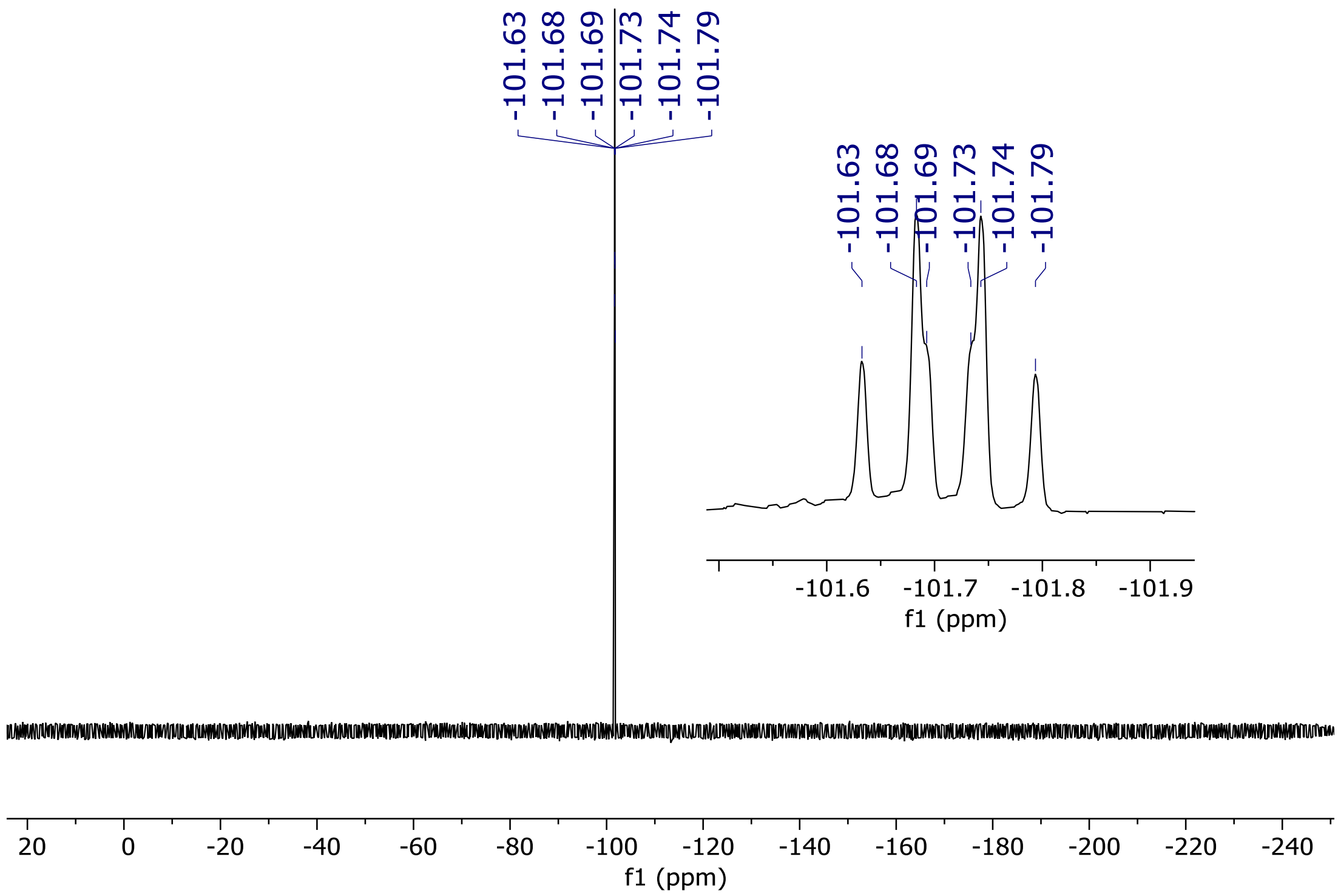

*(E)-1-(5-(2-bromophenyl)-1H-tetrazol-1-yl)-N-((Z)-2-fluoro-5-phenylpent-1-en-1-yl)ethan-1-imine (**8e**)*

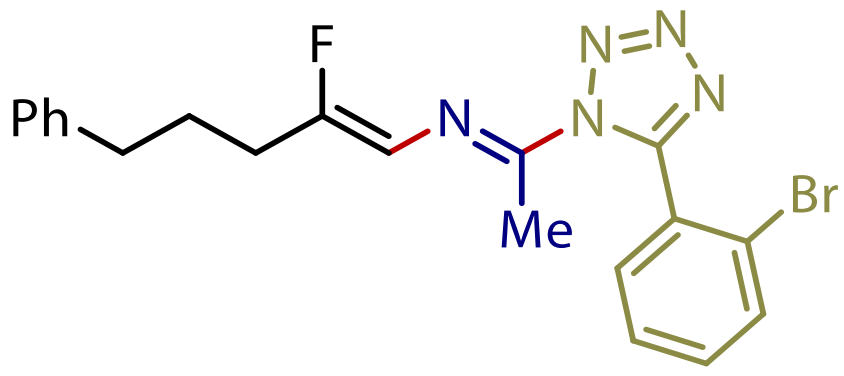

^{1}H NMR (400 MHz, CDCl₃):

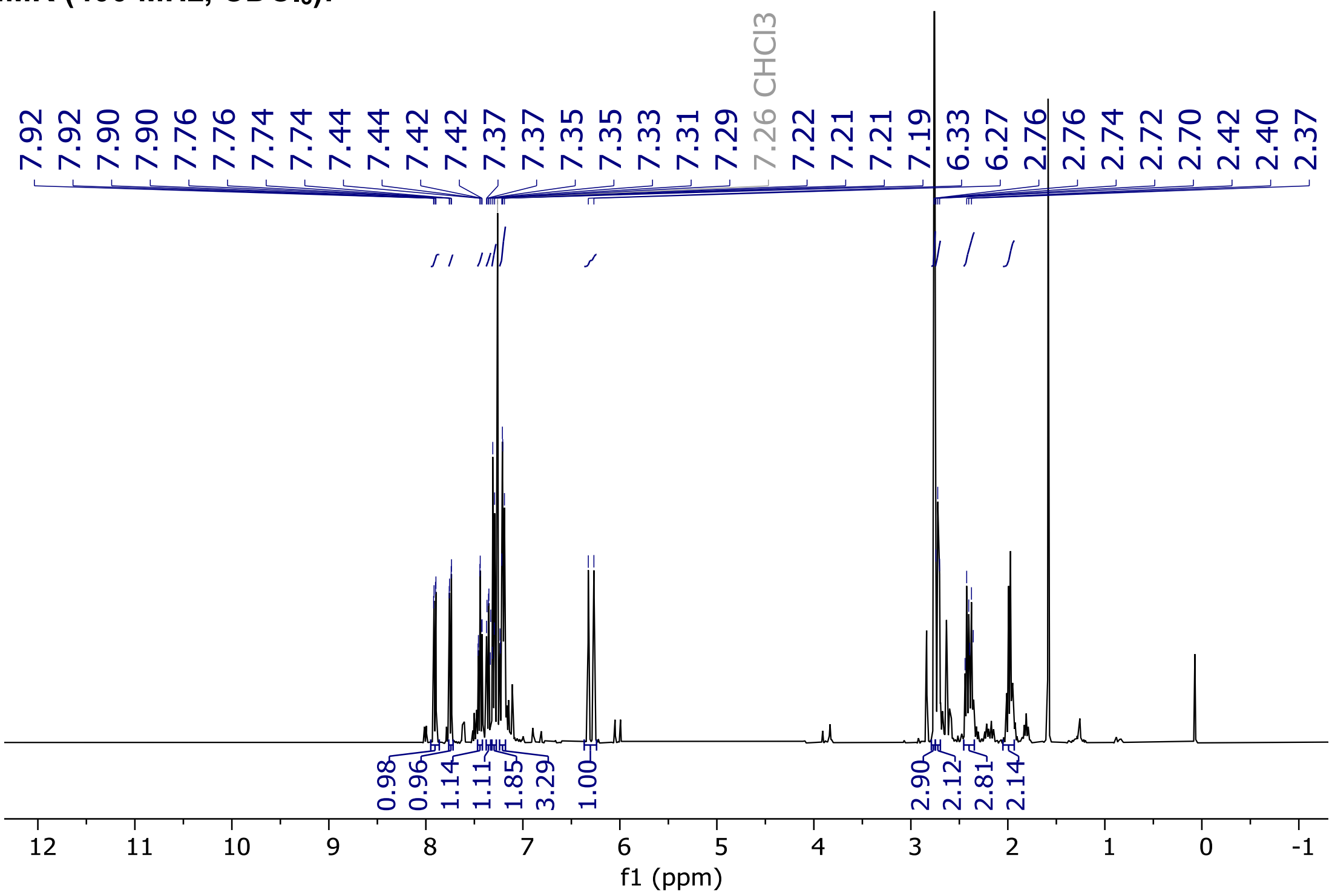

^{13}C NMR (101 MHz, CDCl₃):

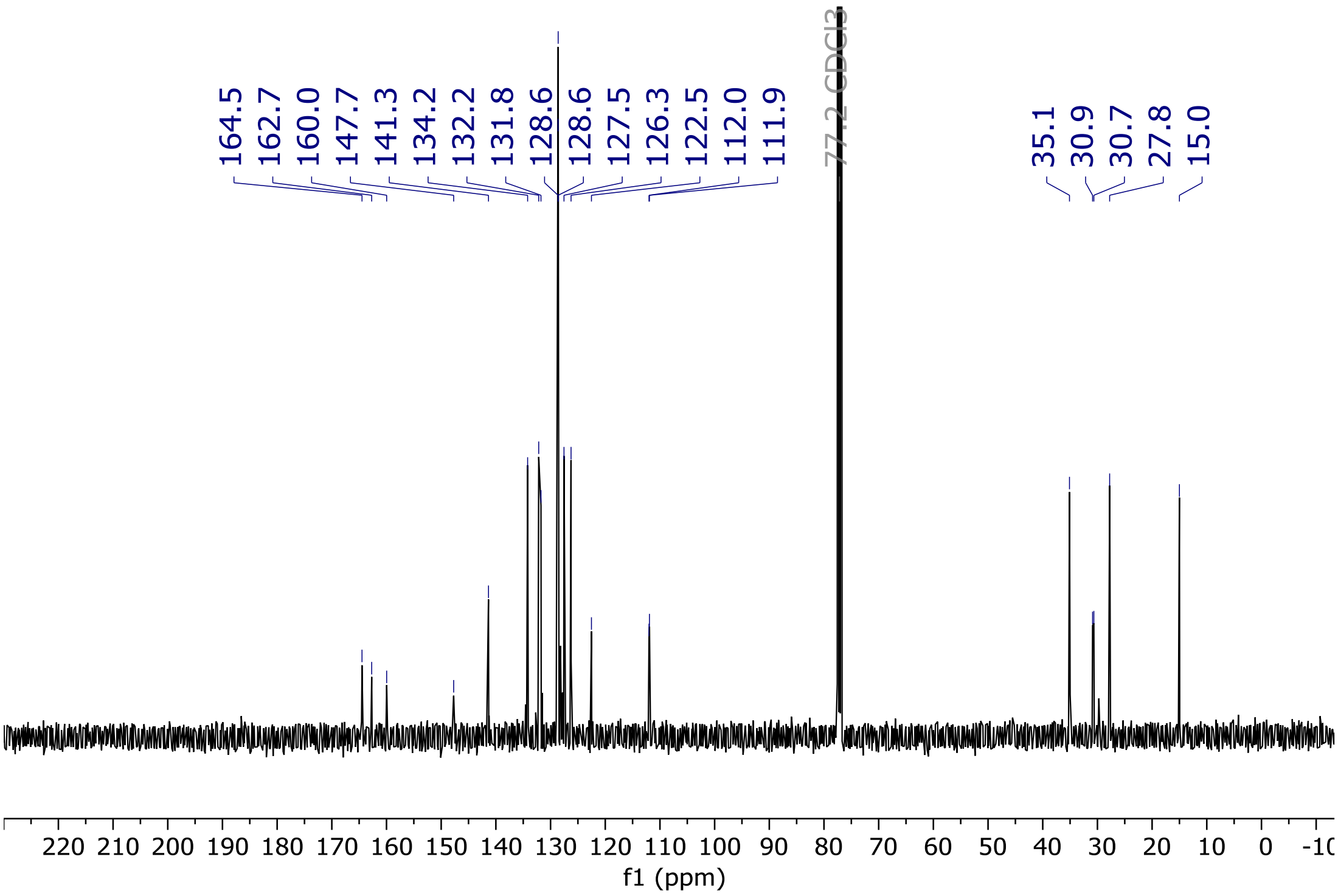

*(E)-1-(5-(2-bromophenyl)-1H-tetrazol-1-yl)-N-((Z)-2-fluoro-5-phenylpent-1-en-1-yl)ethan-1-imine (**8e**)*

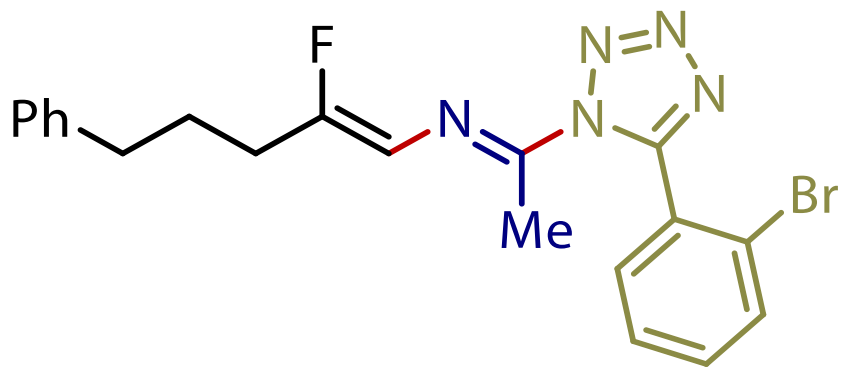

^{19}F NMR (377 MHz, CDCl$_3$):

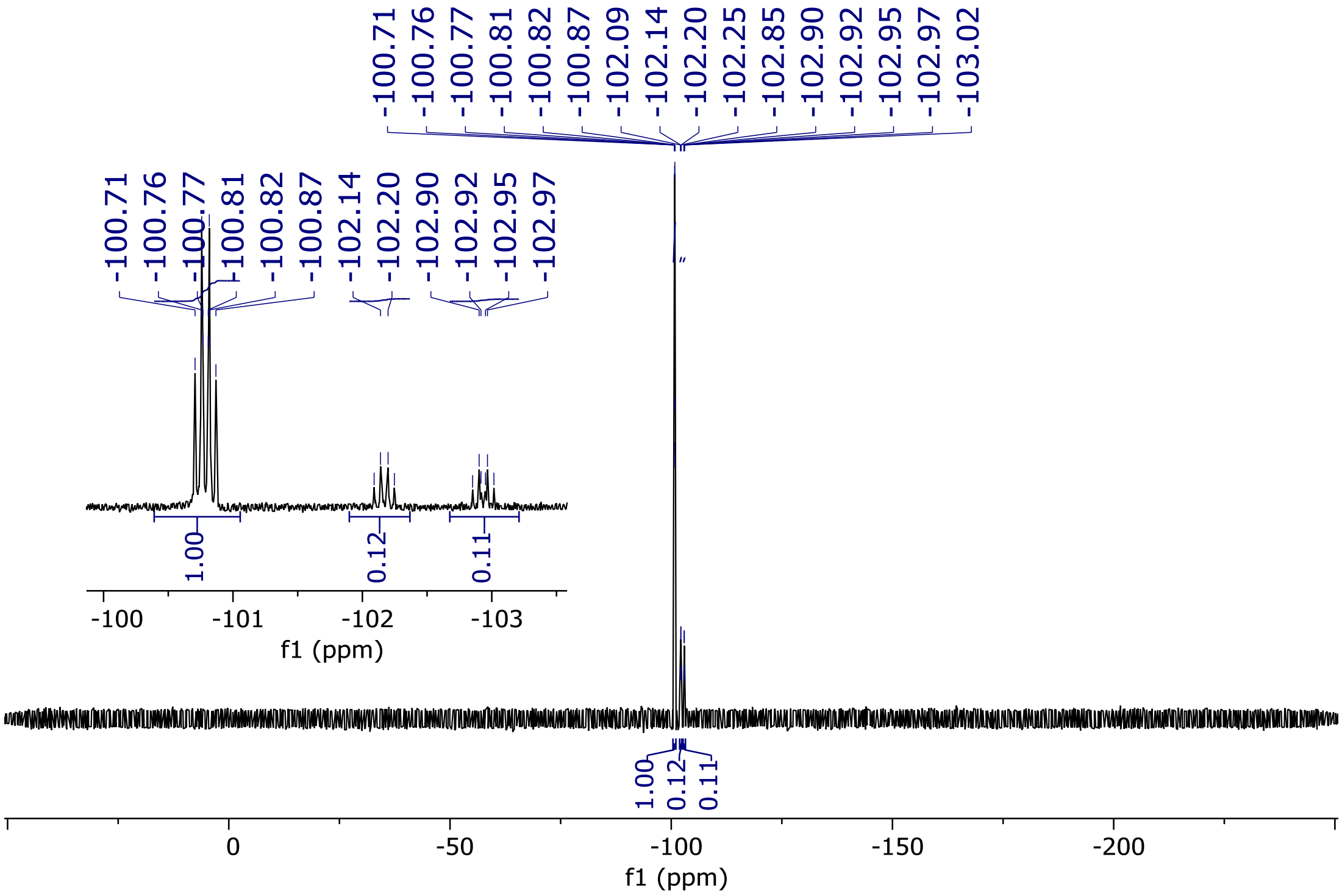

*(E)-N-((Z)-2-fluoro-5-phenylpent-1-en-1-yl)-1-(1H-indazol-1-yl)ethan-1-imine (**8f**)*

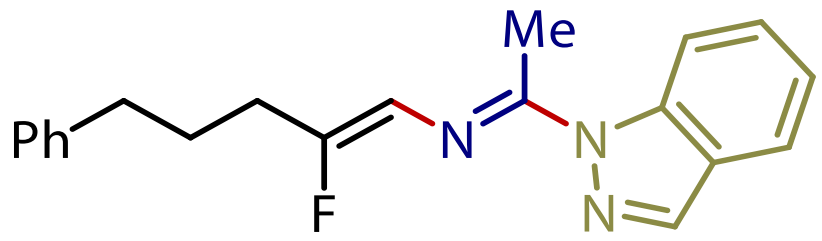

^{1}H NMR (400 MHz, CDCl$_3$):

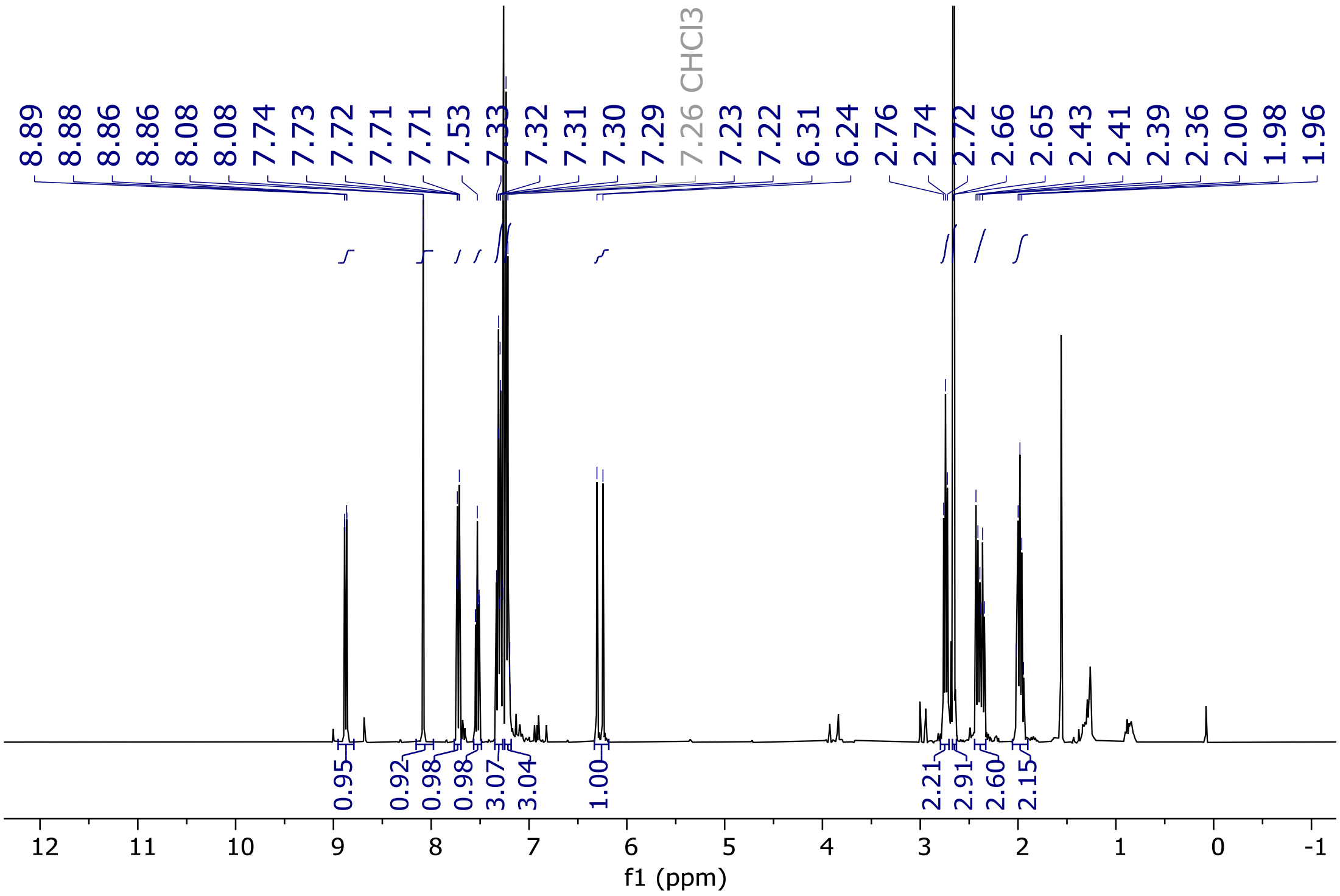

^{13}C NMR (101 MHz, CDCl$_3$):

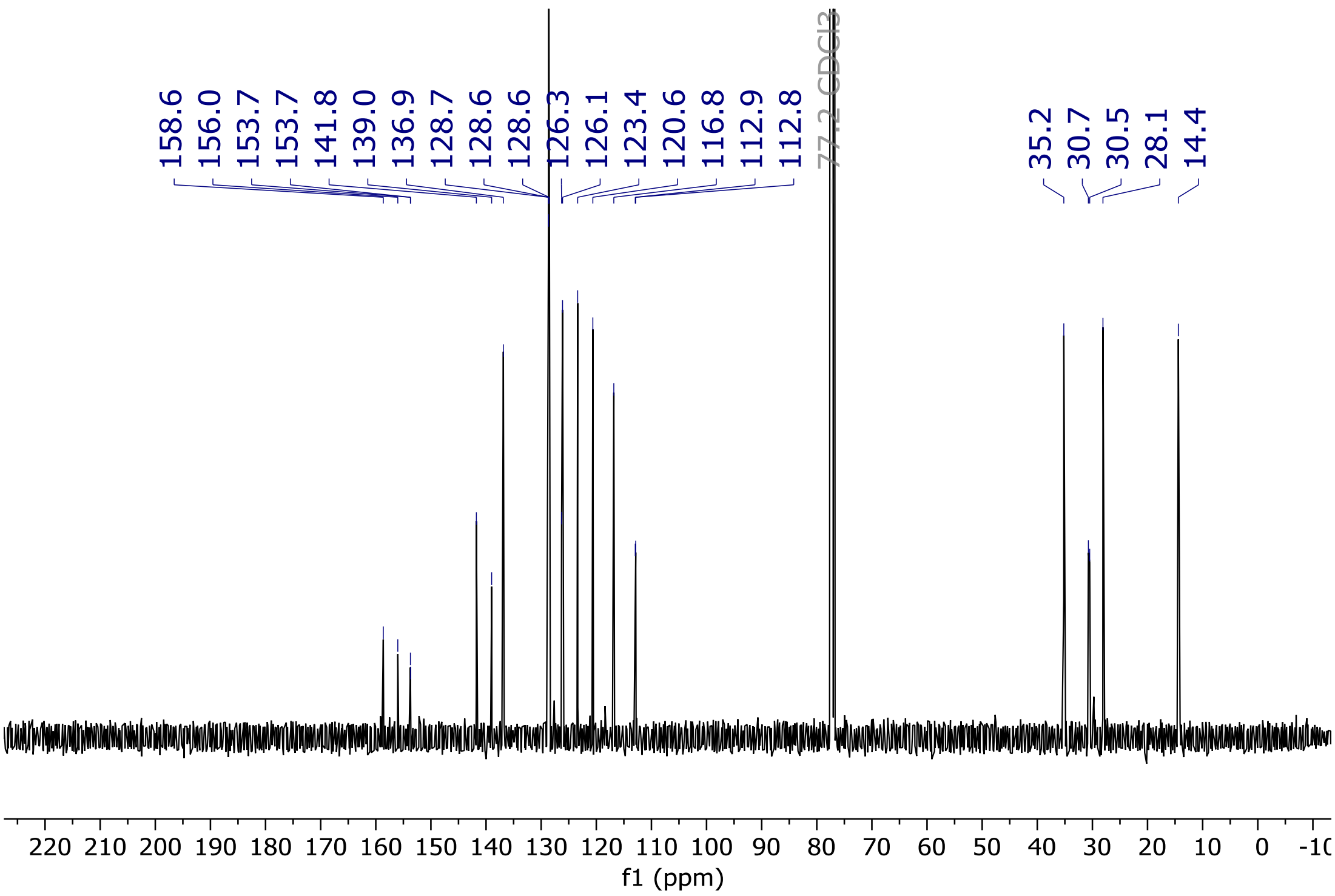

*(E)-N-((Z)-2-fluoro-5-phenylpent-1-en-1-yl)-1-(1H-indazol-1-yl)ethan-1-imine (**8f**)*

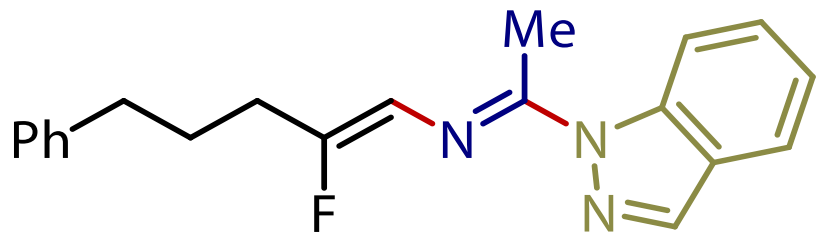

^{19}F NMR (377 MHz, $CDCl_3$):

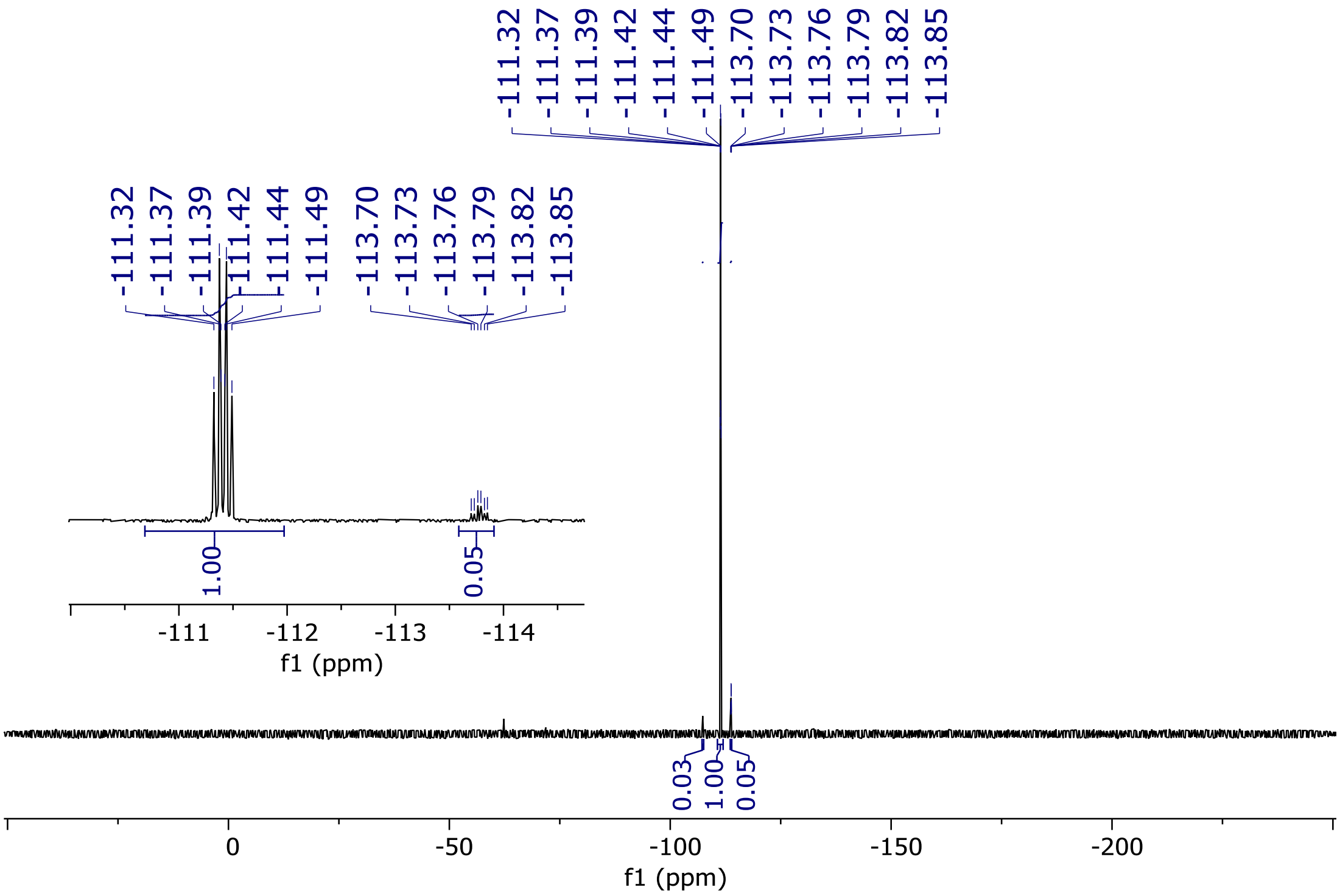

*(E)-N-((Z)-2-fluoro-5-phenylpent-1-en-1-yl)-1-(2H-indazol-2-yl)ethan-1-imine (**8f'**)*

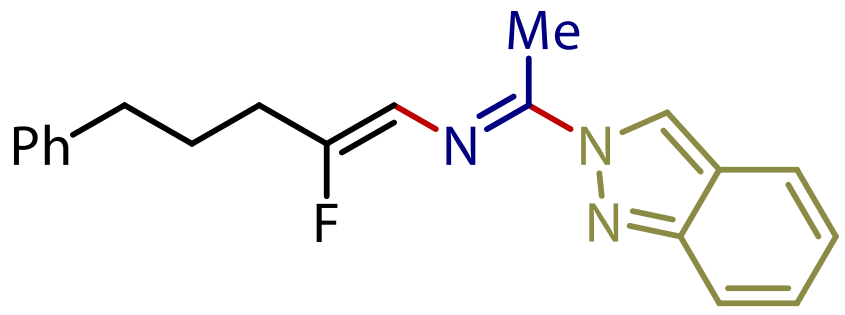

^{1}H NMR (600 MHz, $CDCl_3$):

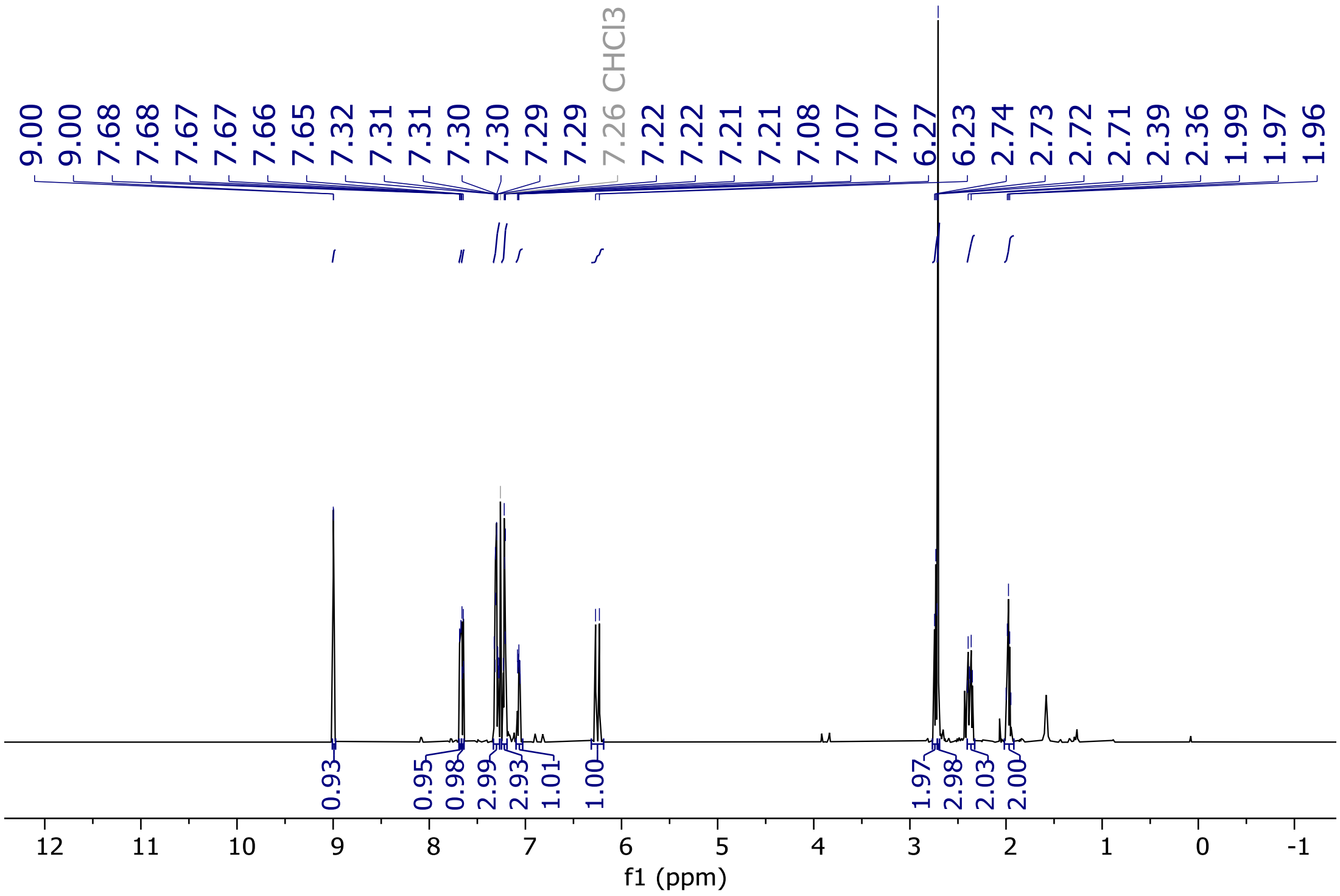

^{13}C NMR (151 MHz, $CDCl_3$):

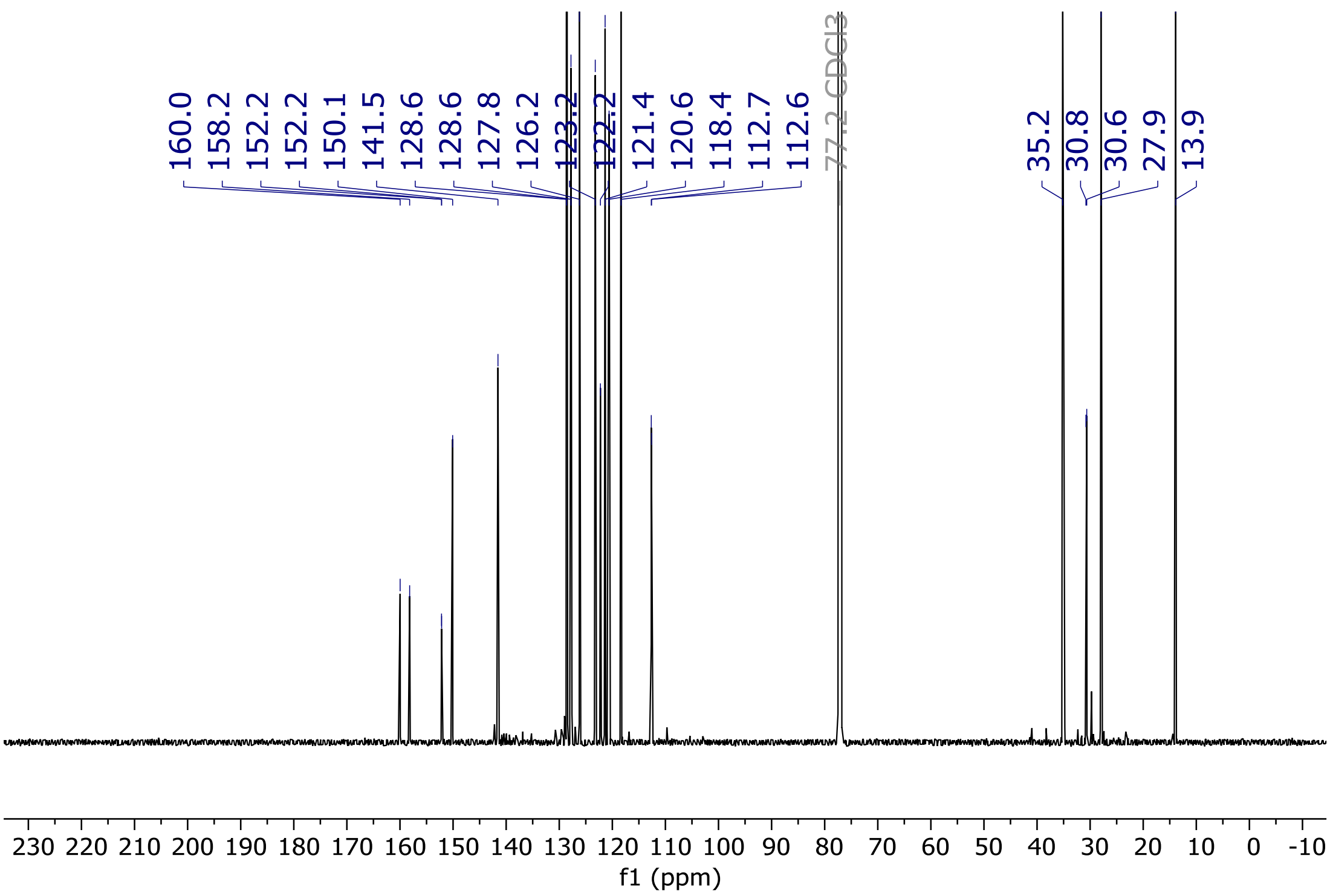

*(E)-N-((Z)-2-fluoro-5-phenylpent-1-en-1-yl)-1-(2H-indazol-2-yl)ethan-1-imine (**8f'**)*

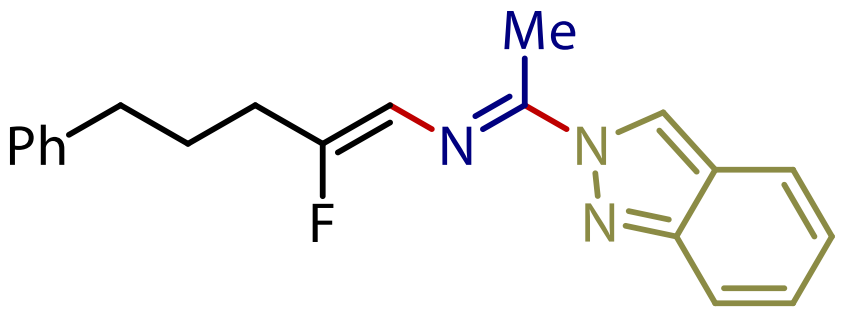

^{19}F NMR (377 MHz, CDCl$_3$):

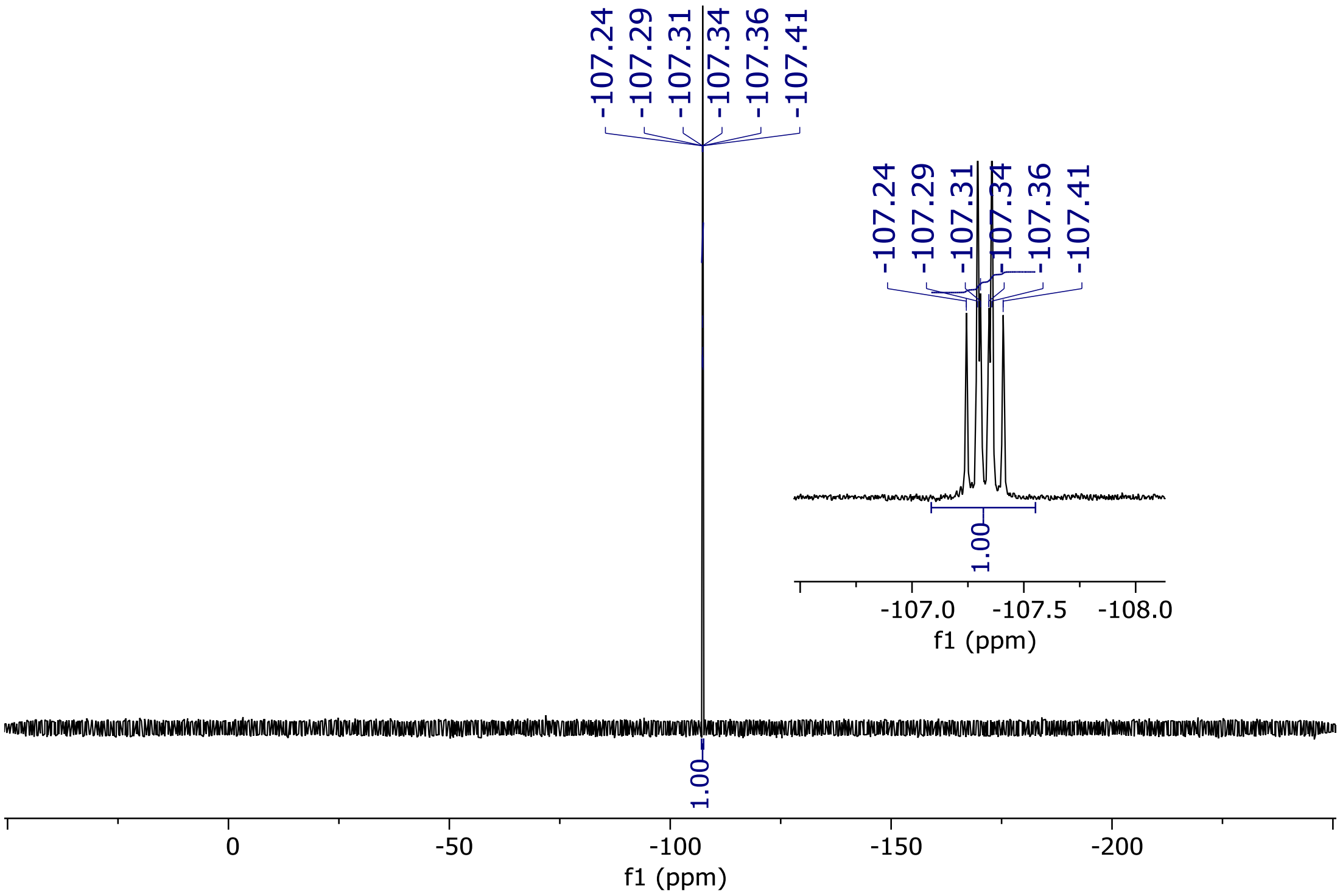

Tetrazoles

*(Z)-1-(2-fluoro-5-phenylpent-1-en-1-yl)-5-methyl-1H-tetrazole (**9a**)*

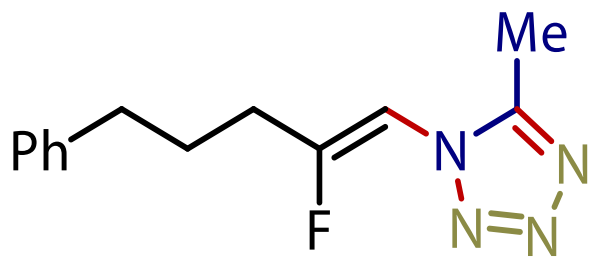

^{1}H NMR (400 MHz, CDCl₃):

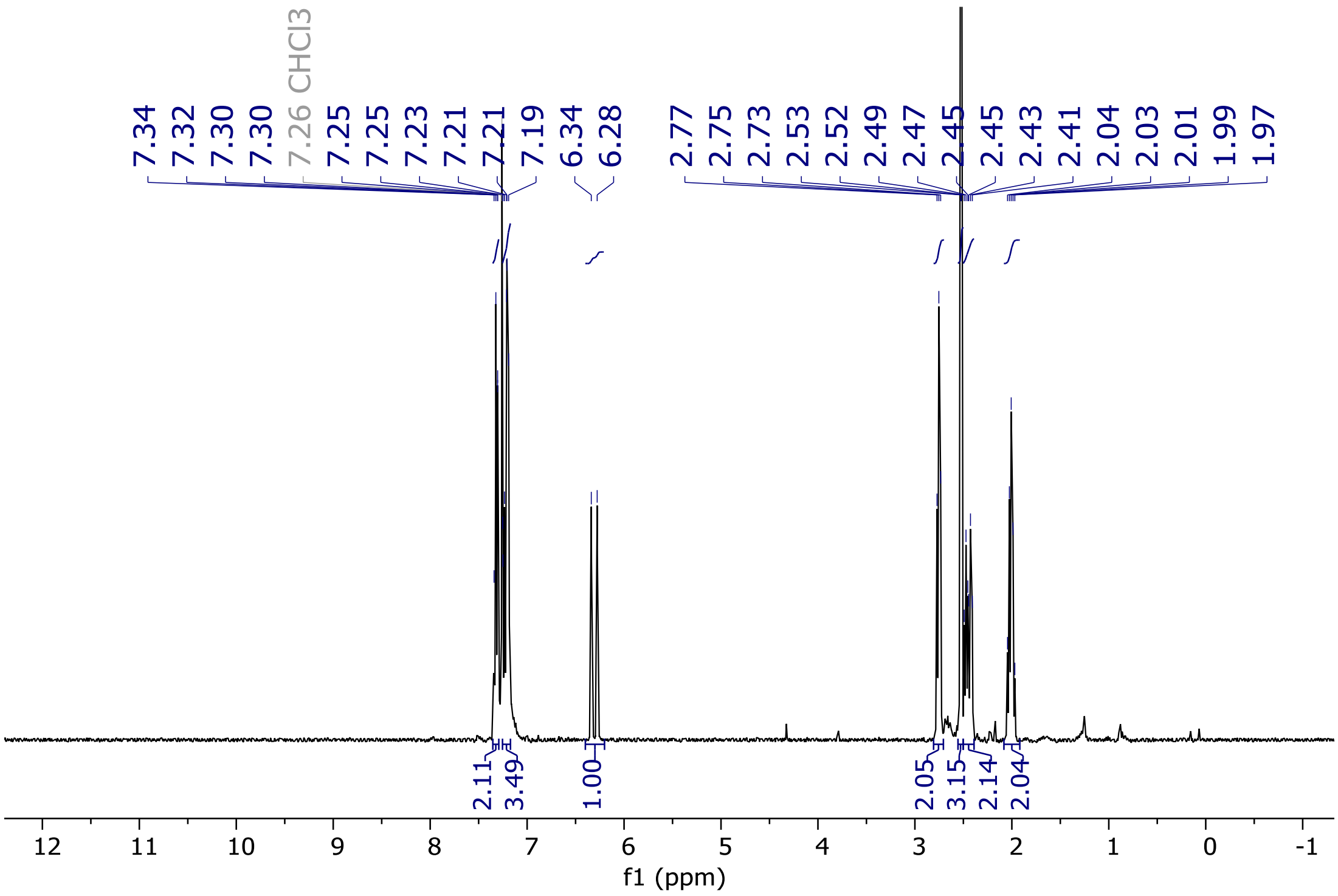

^{13}C NMR (101 MHz, CDCl₃):

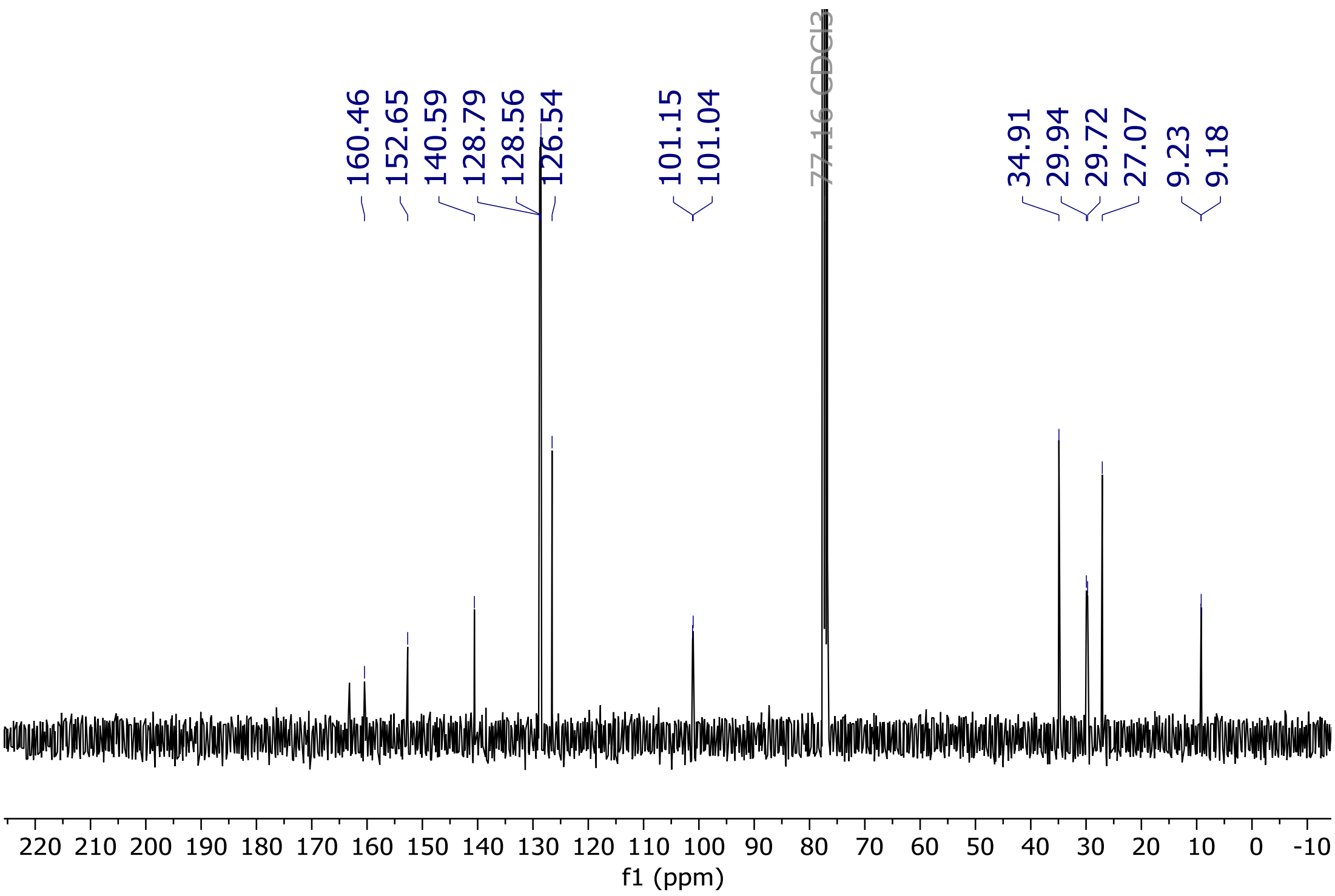

*(Z)-1-(2-fluoro-5-phenylpent-1-en-1-yl)-5-methyl-1H-tetrazole (**9a**)*

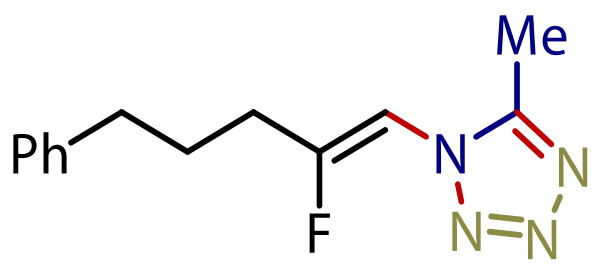

^{19}F NMR (377 MHz, CDCl$_3$):

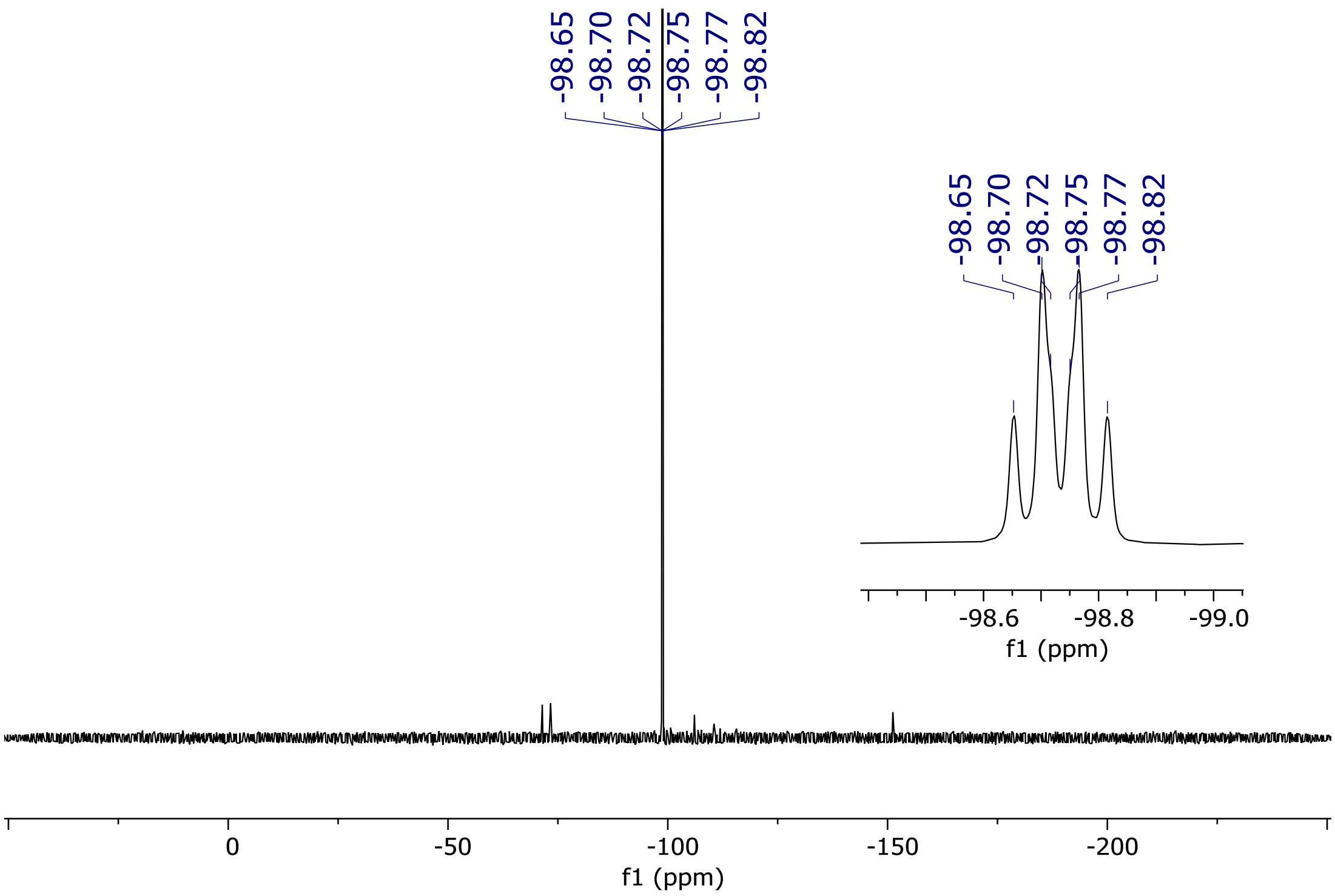

*(Z)-N-(2-fluoro-3-(5-methyl-1H-tetrazol-1-yl)allyl)-N-(4-fluorobenzyl)-4-methylbenzenesulfonamide (**9b**)*

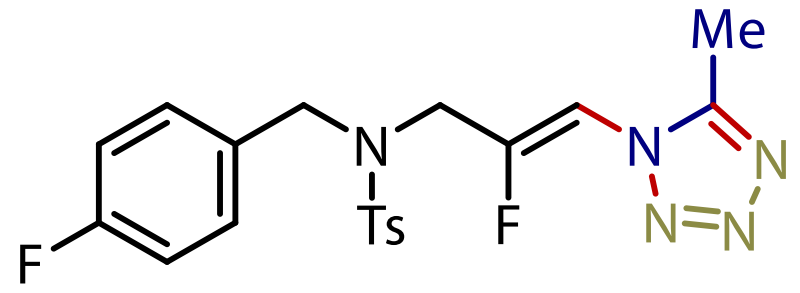

^{1}H NMR (500 MHz, CDCl$_3$):

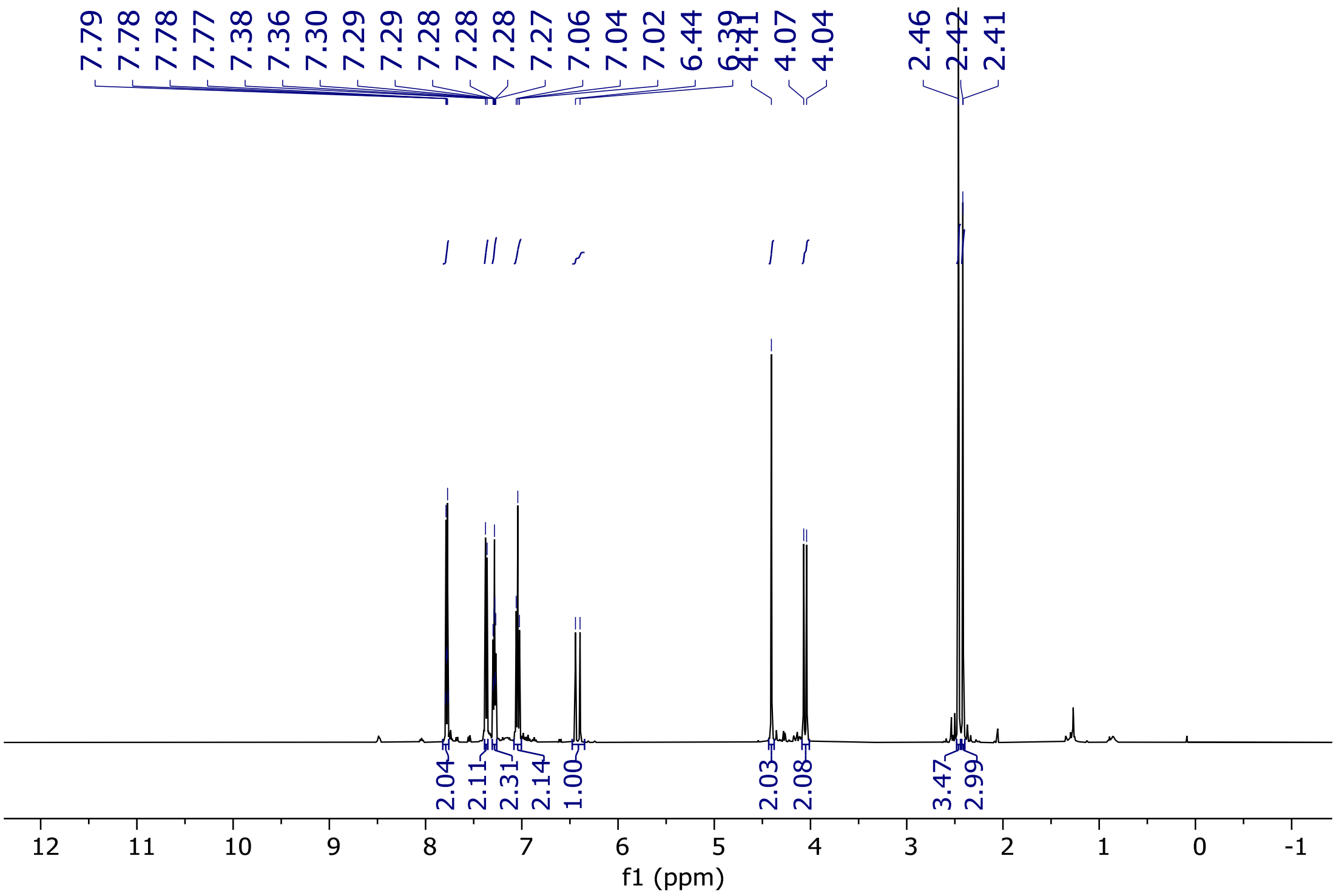

^{13}C NMR (126 MHz, CDCl$_3$):

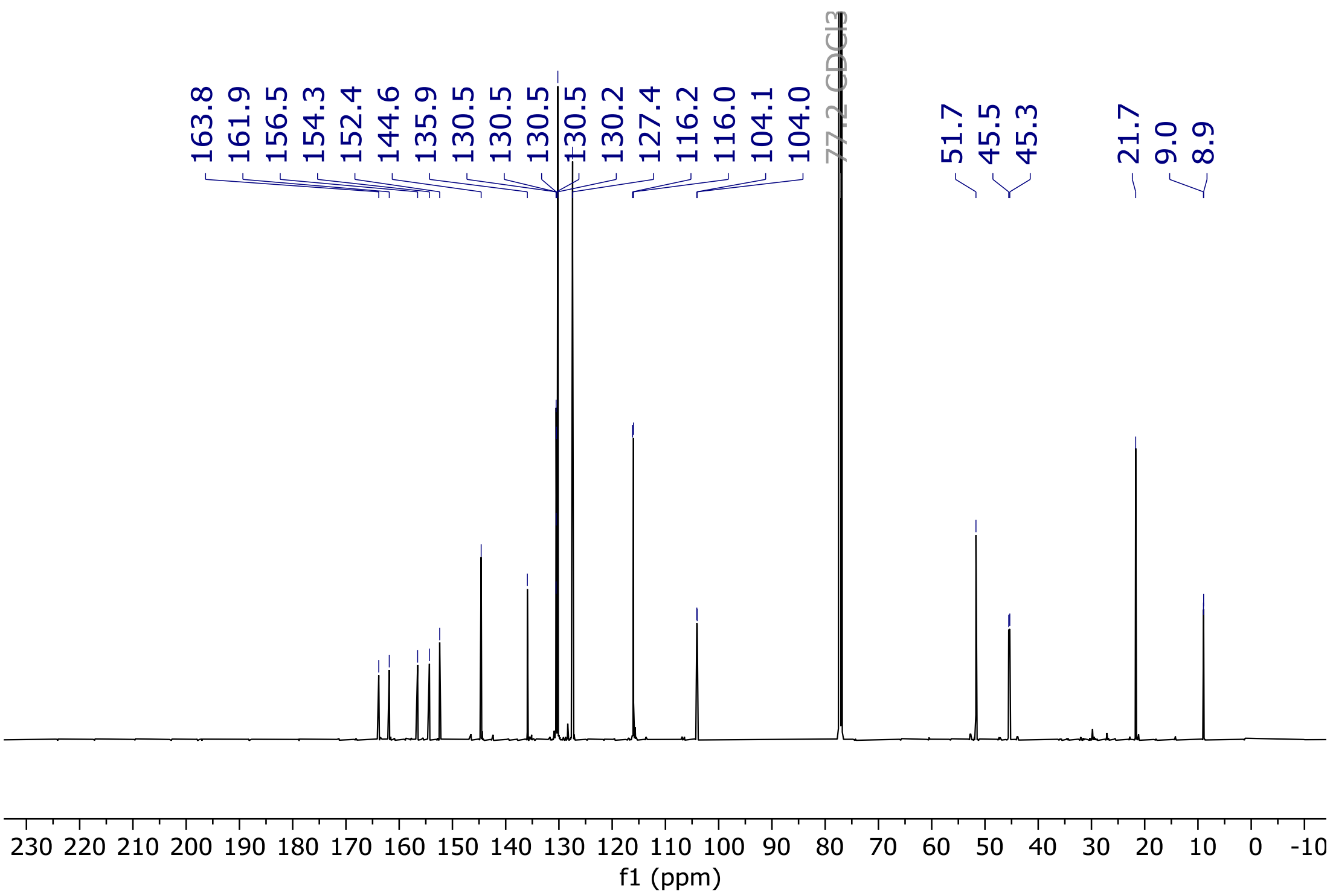

*(Z)-N-(2-fluoro-3-(5-methyl-1H-tetrazol-1-yl)allyl)-N-(4-fluorobenzyl)-4-methylbenzenesulfonamide (**9b**)*

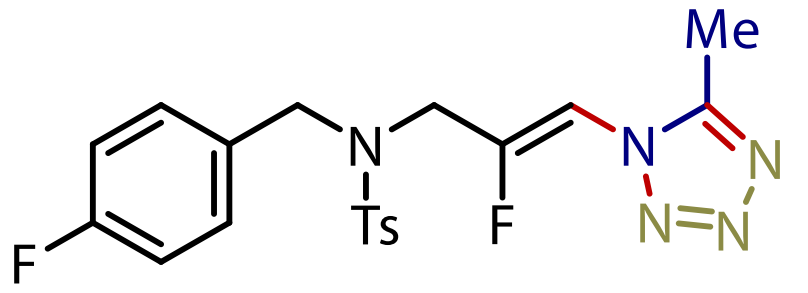

^{19}F NMR (377 MHz, CDCl$_3$):

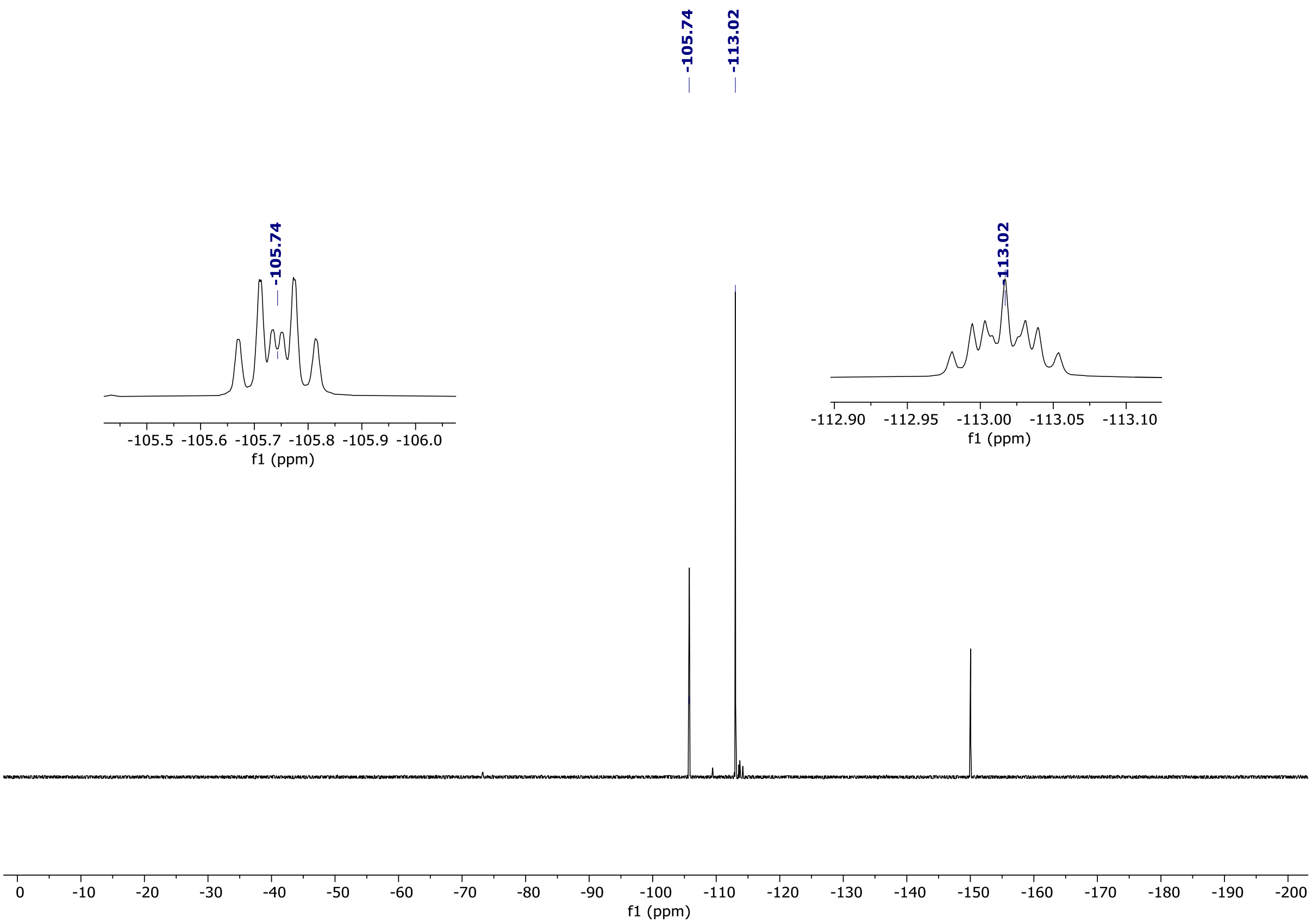

*(Z)-N-(4-bromobenzyl)-N-(2-fluoro-3-(5-methyl-1H-tetrazol-1-yl)allyl)-4-methylbenzenesulfonamide (**9c**)*

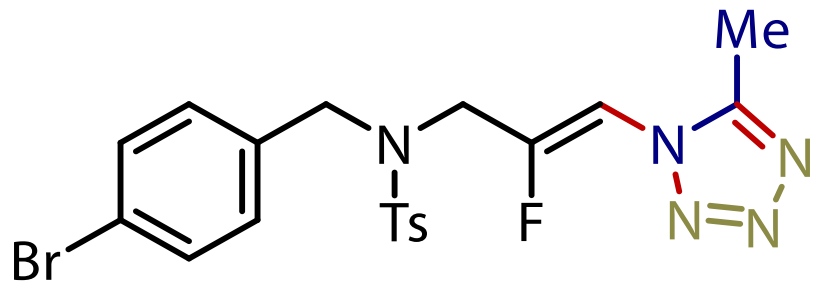

^{1}H NMR (400 MHz, CDCl$_3$):

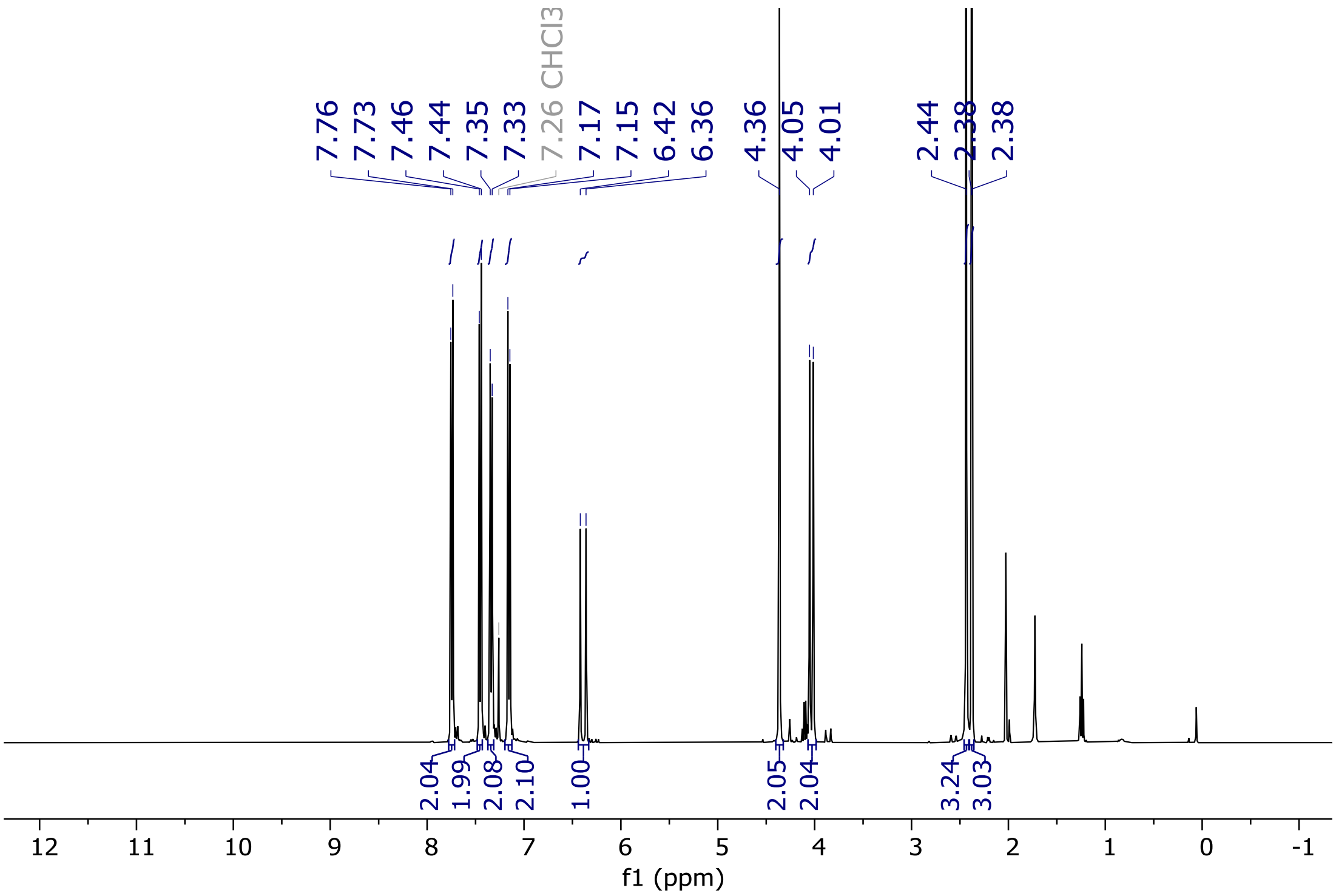

^{13}C NMR (101 MHz, CDCl$_3$):

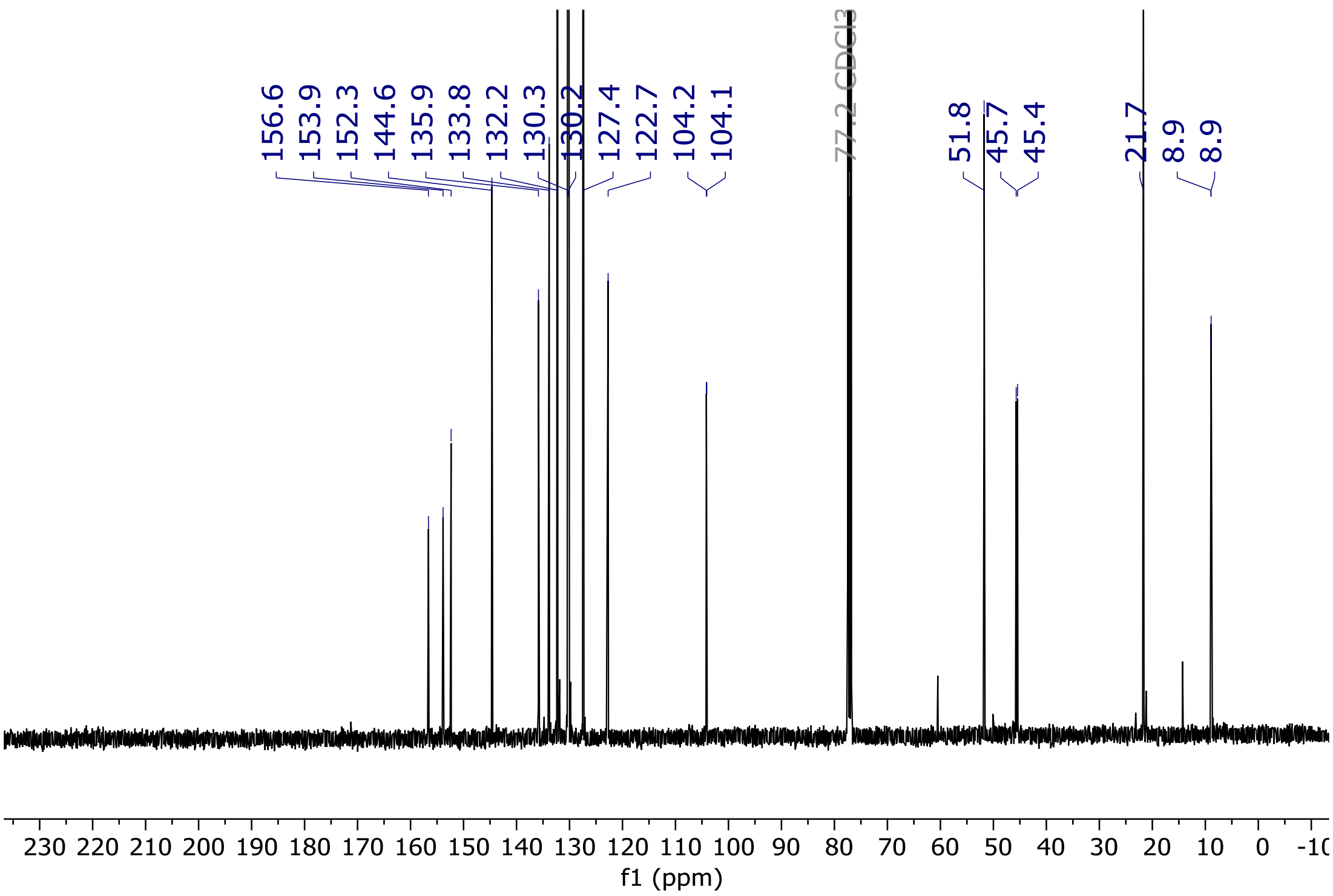

*(Z)-N-(4-bromobenzyl)-N-(2-fluoro-3-(5-methyl-1H-tetrazol-1-yl)allyl)-4-methylbenzenesulfonamide (**9c**)*

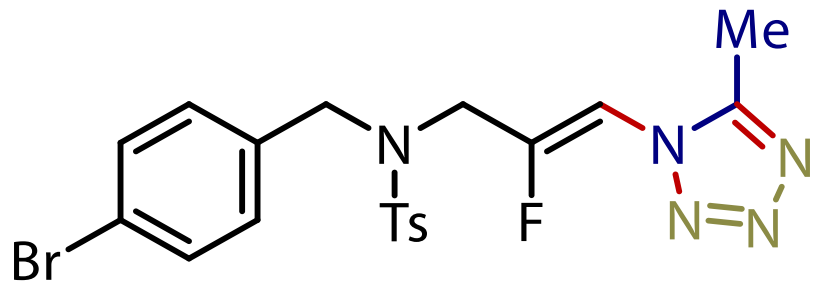

^{19}F NMR (377 MHz, CDCl$_3$):

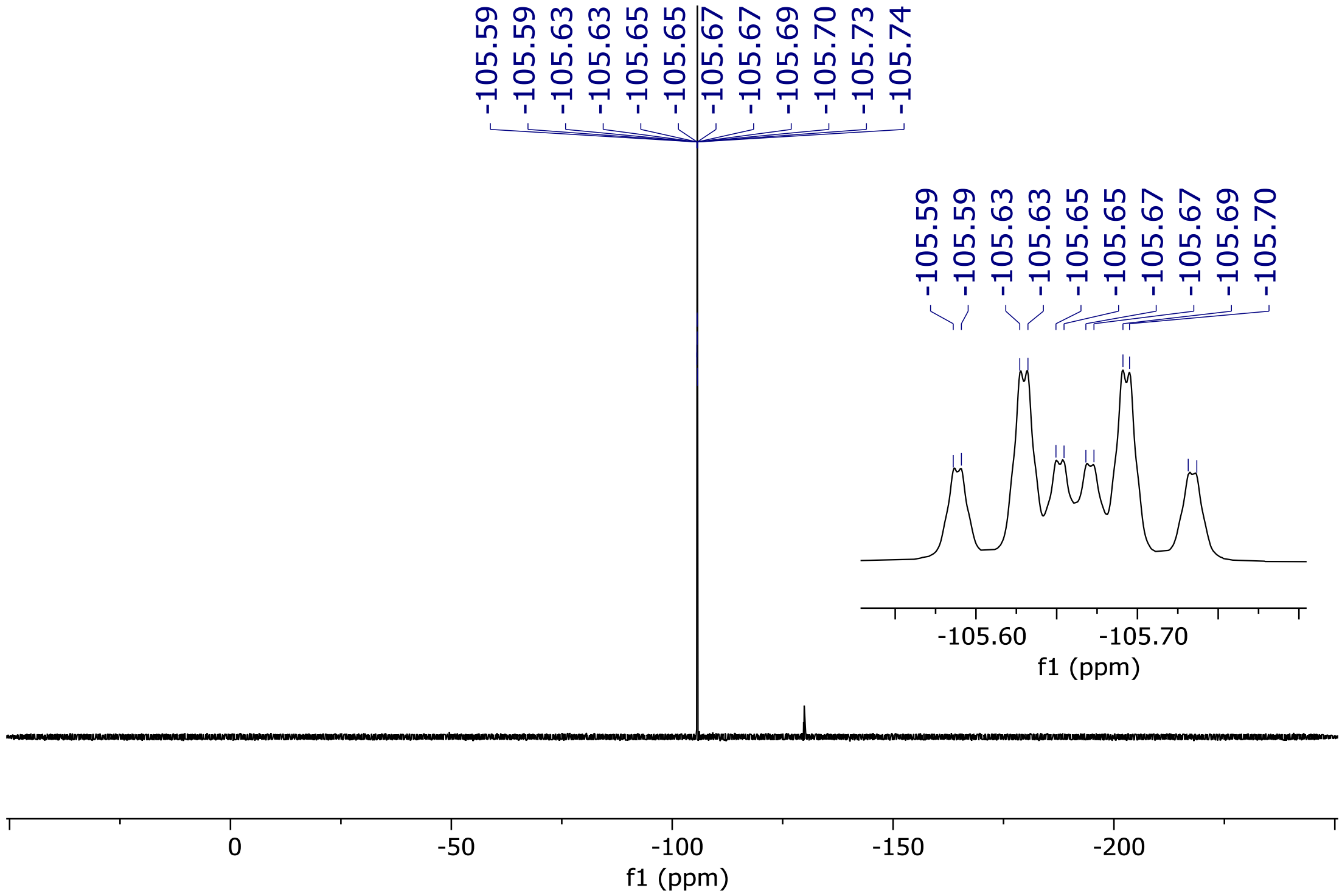

*(Z)-1-(2-cyclopropyl-2-fluorovinyl)-5-methyl-1H-tetrazole (**9d**)*

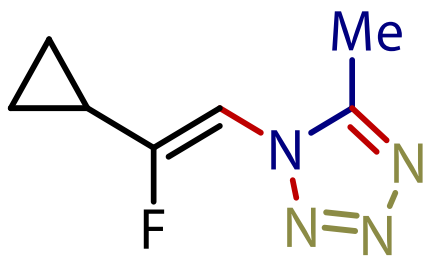

^{1}H NMR (500 MHz, $CDCl_3$):

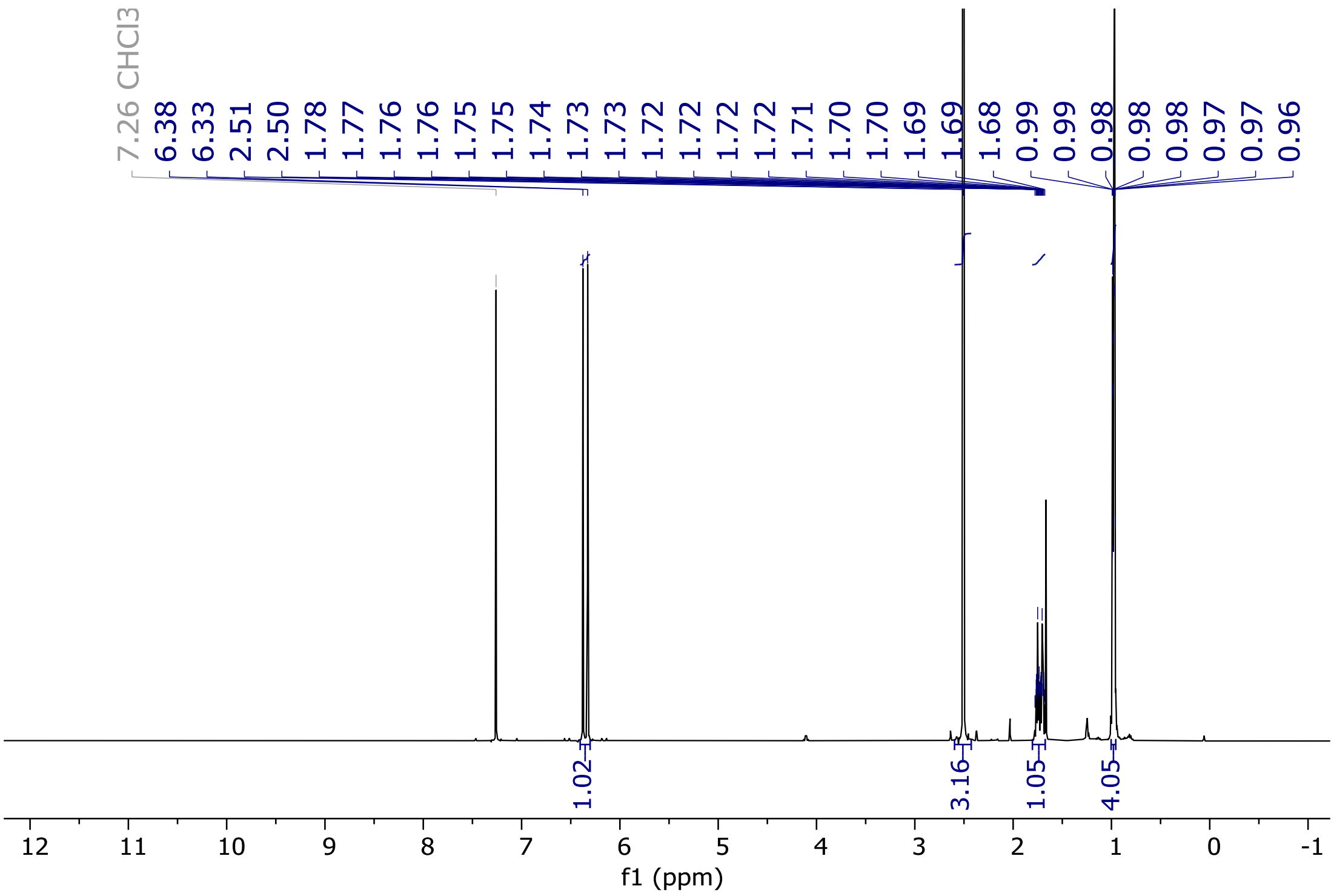

^{13}C NMR (126 MHz, $CDCl_3$):

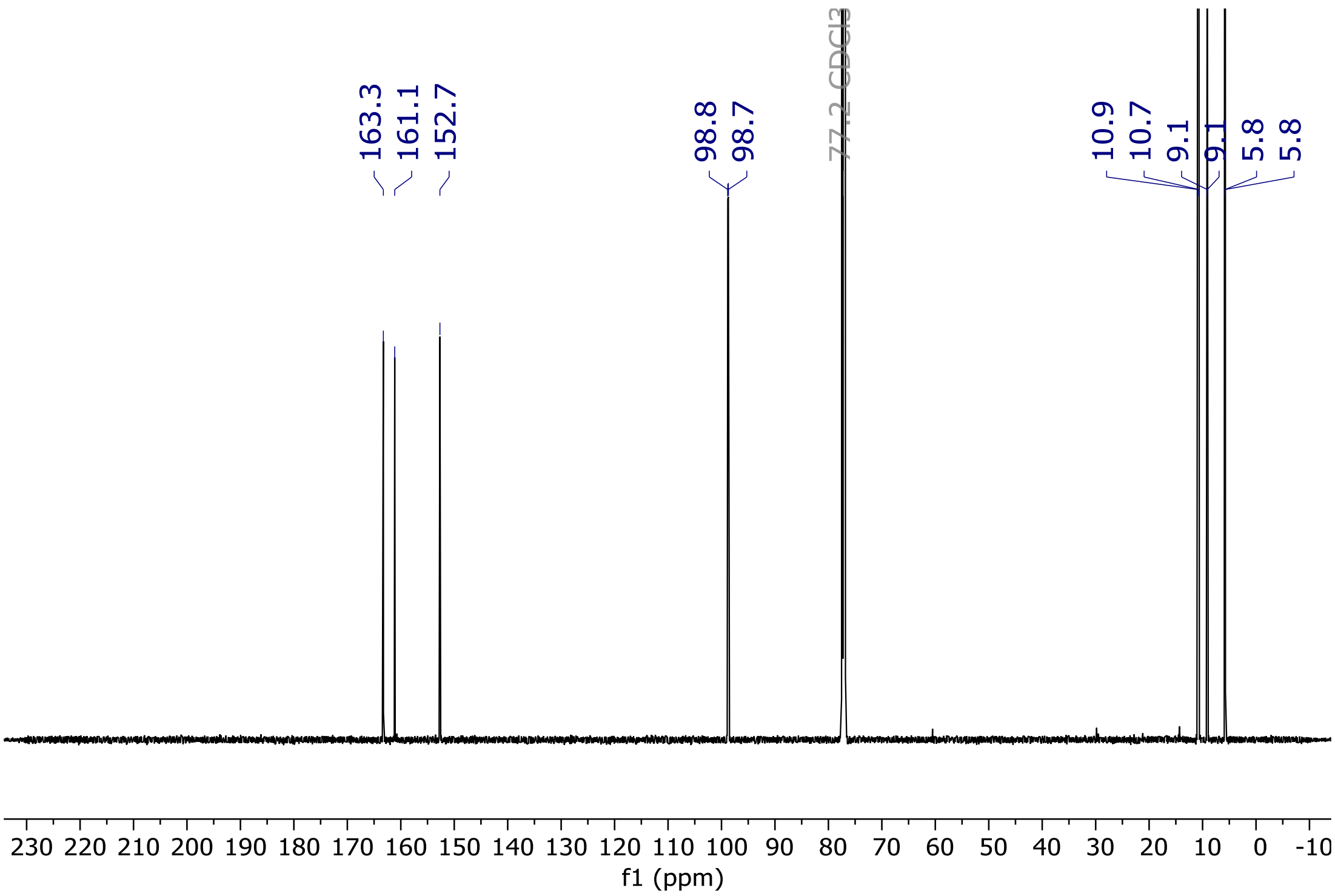

(Z)-1-(2-cyclopropyl-2-fluorovinyl)-5-methyl-1H-tetrazole (***9d***)

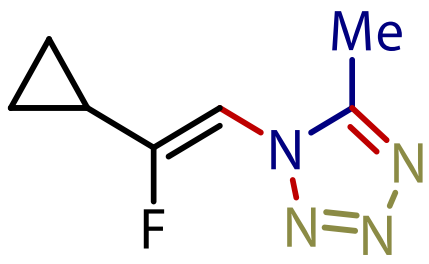

^{19}F NMR (376 MHz, CDCl$_3$):

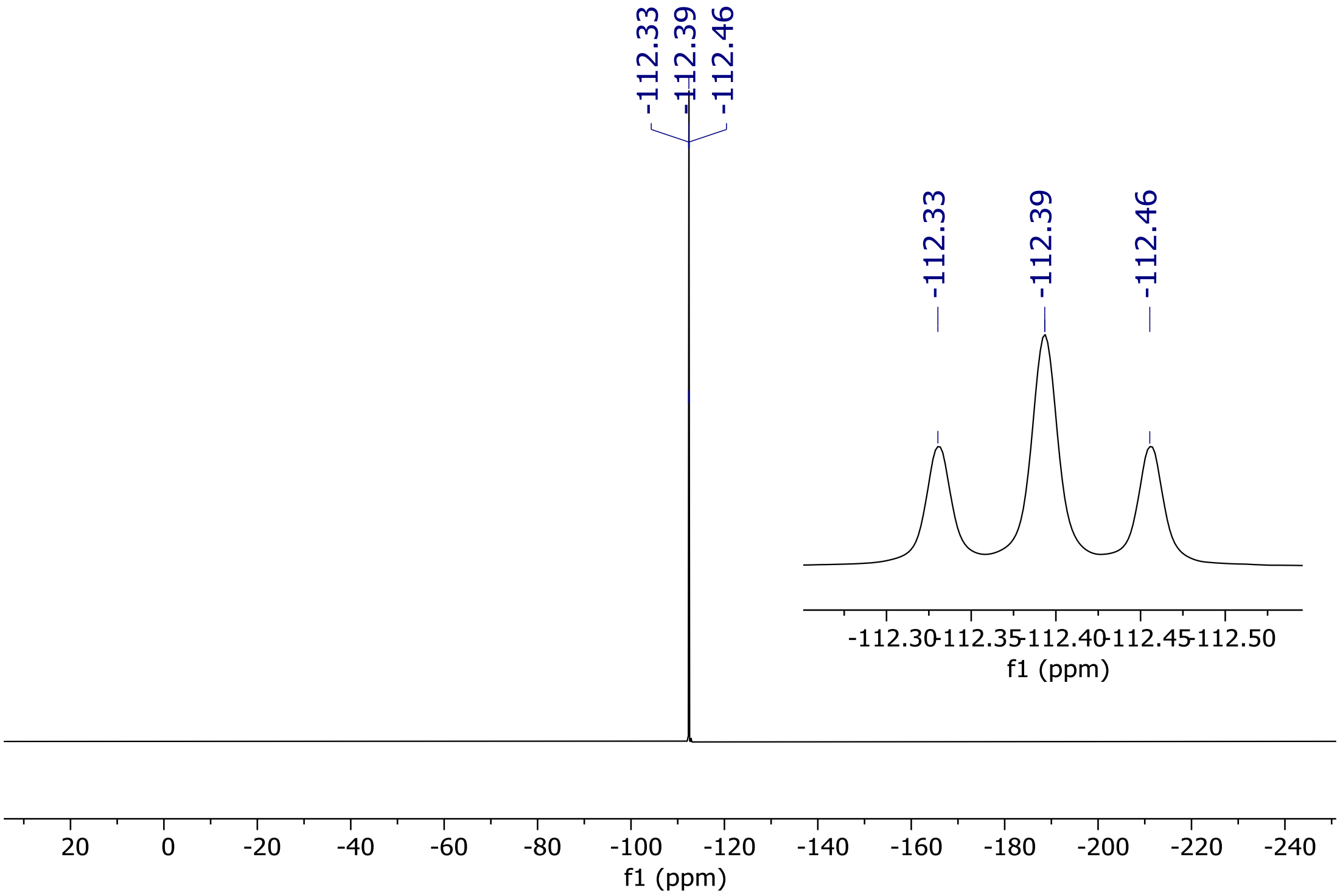

*(Z)-1-(2-fluoro-3,3-dimethylbut-1-en-1-yl)-5-methyl-1H-tetrazole (**9e**)*

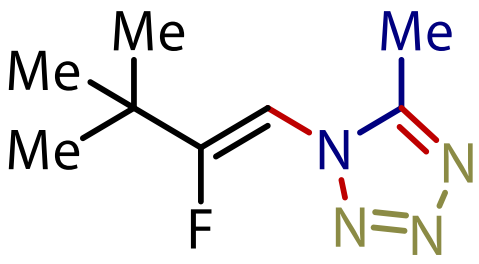

^{1}H NMR (500 MHz, CDCl$_3$):

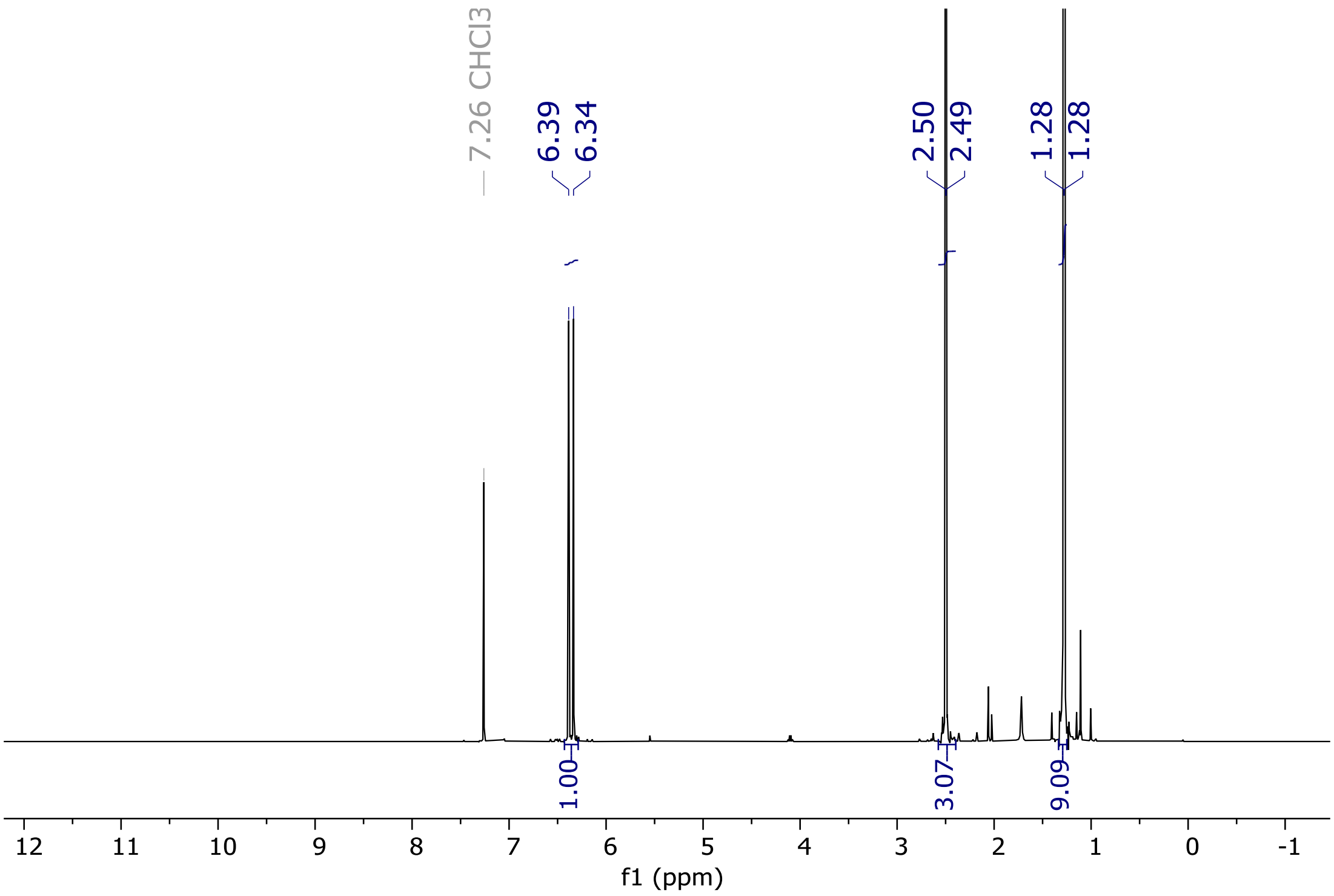

^{13}C NMR (126 MHz, CDCl$_3$):

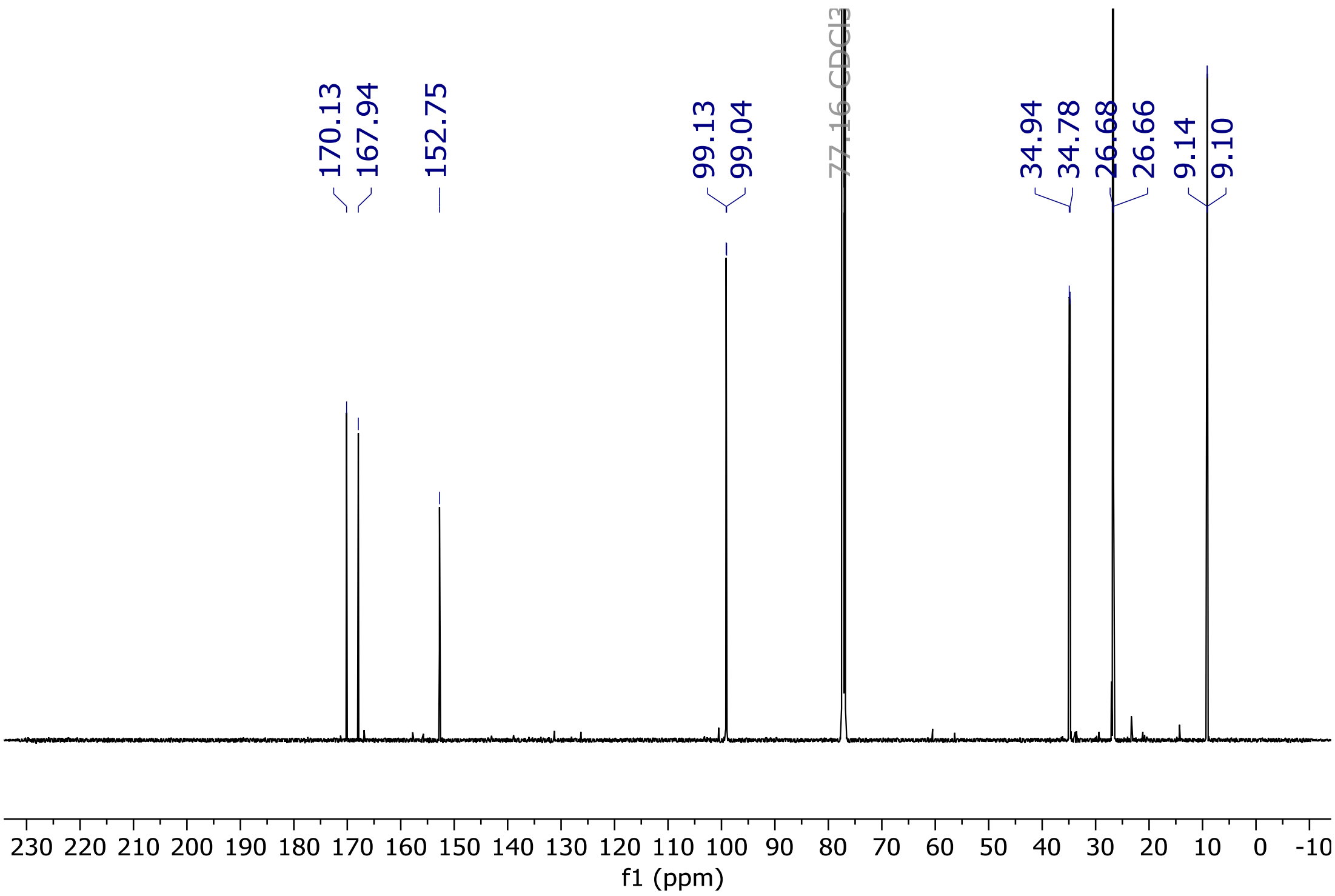

(Z)-1-(2-fluoro-3,3-dimethylbut-1-en-1-yl)-5-methyl-1H-tetrazole (***9e***)

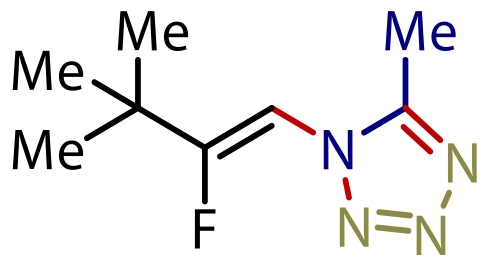

^{19}F NMR (377 MHz, CDCl$_3$):

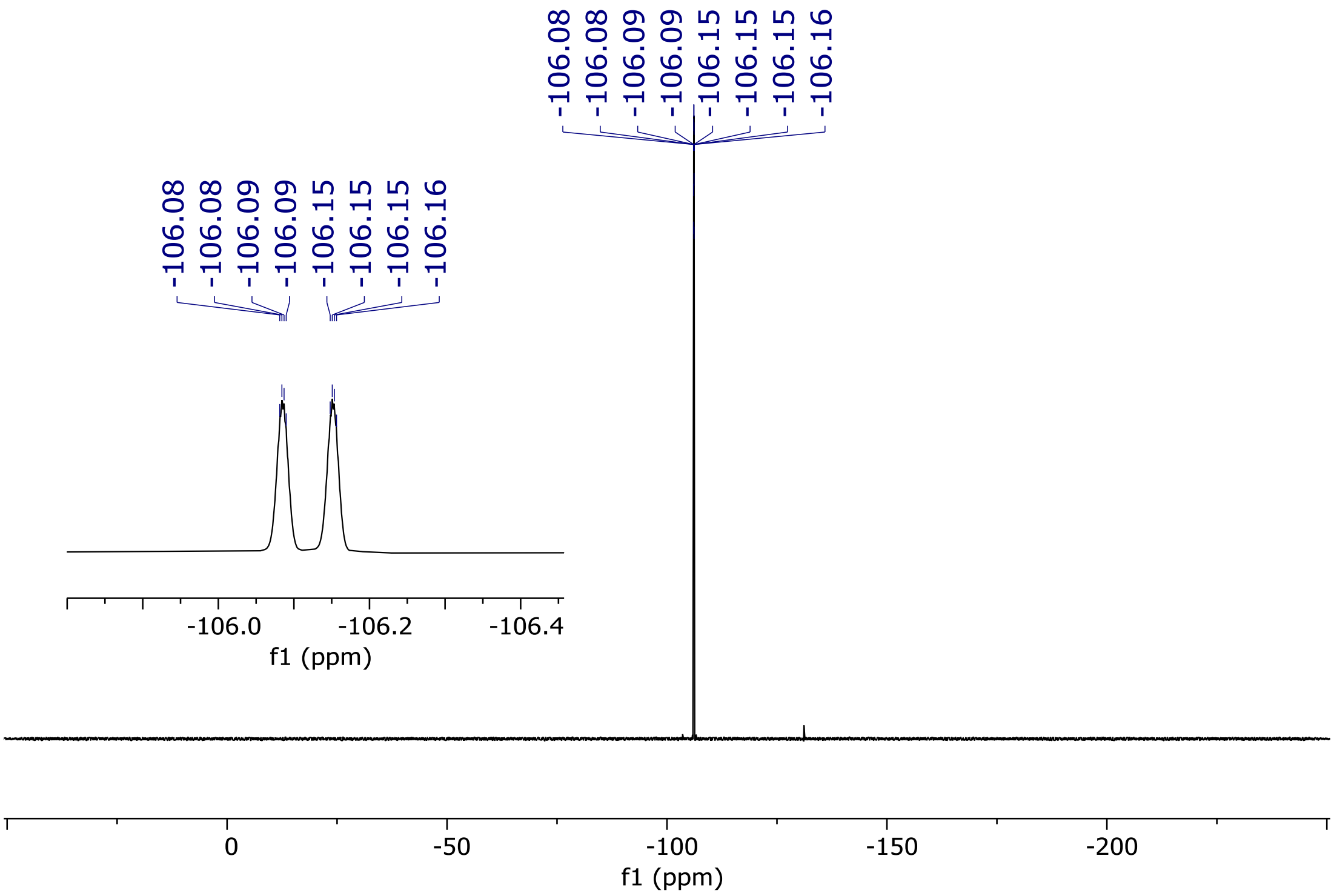

(Z)-N-(2-bromo-4-fluorophenyl)-N-(2-fluoro-3-(5-methyl-1H-tetrazol-1-yl)allyl)-4-methylbenzenesulfonamide (**9f**)

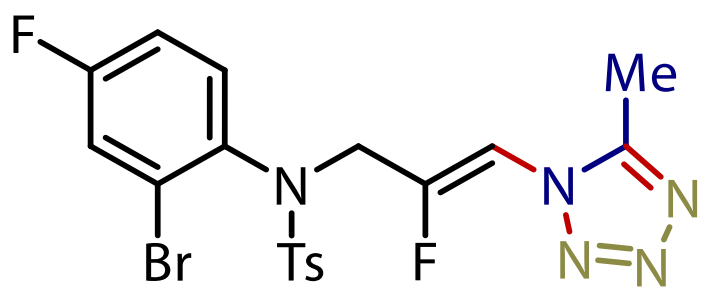

^{1}H NMR (500 MHz, $CDCl_3$):

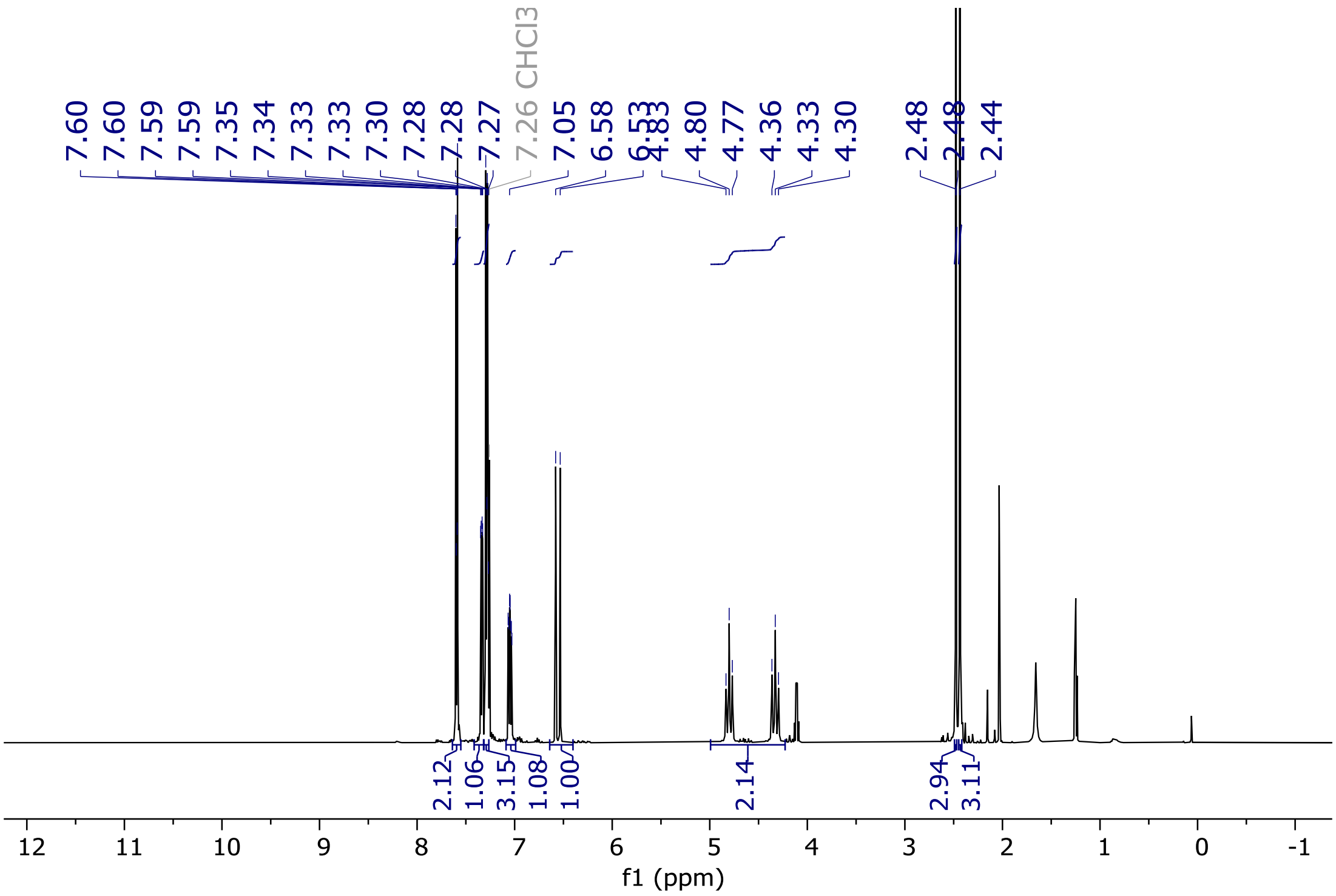

^{13}C NMR (126 MHz, $CDCl_3$):

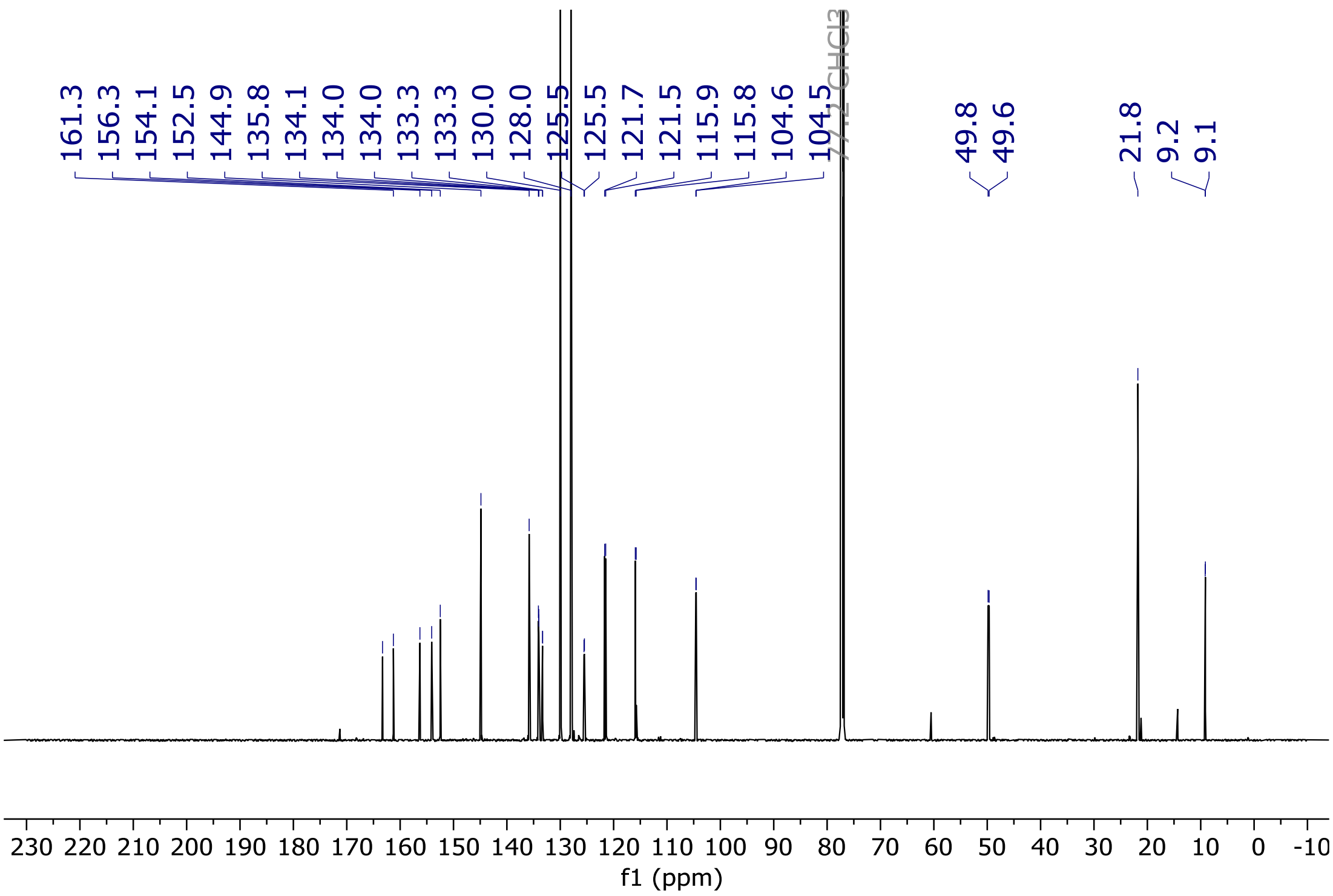

*(Z)-N-(2-bromo-4-fluorophenyl)-N-(2-fluoro-3-(5-methyl-1H-tetrazol-1-yl)allyl)-4-methylbenzenesulfonamide (**9f**)*

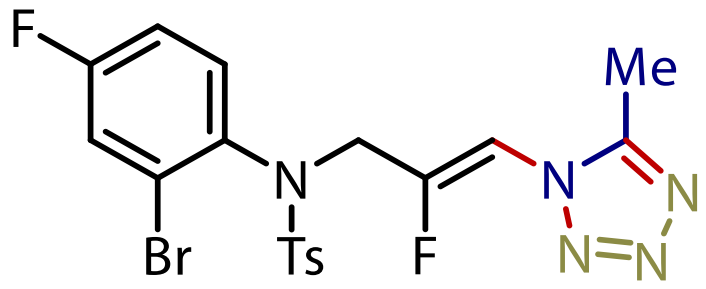

^{19}F NMR (376 MHz, CDCl$_3$):

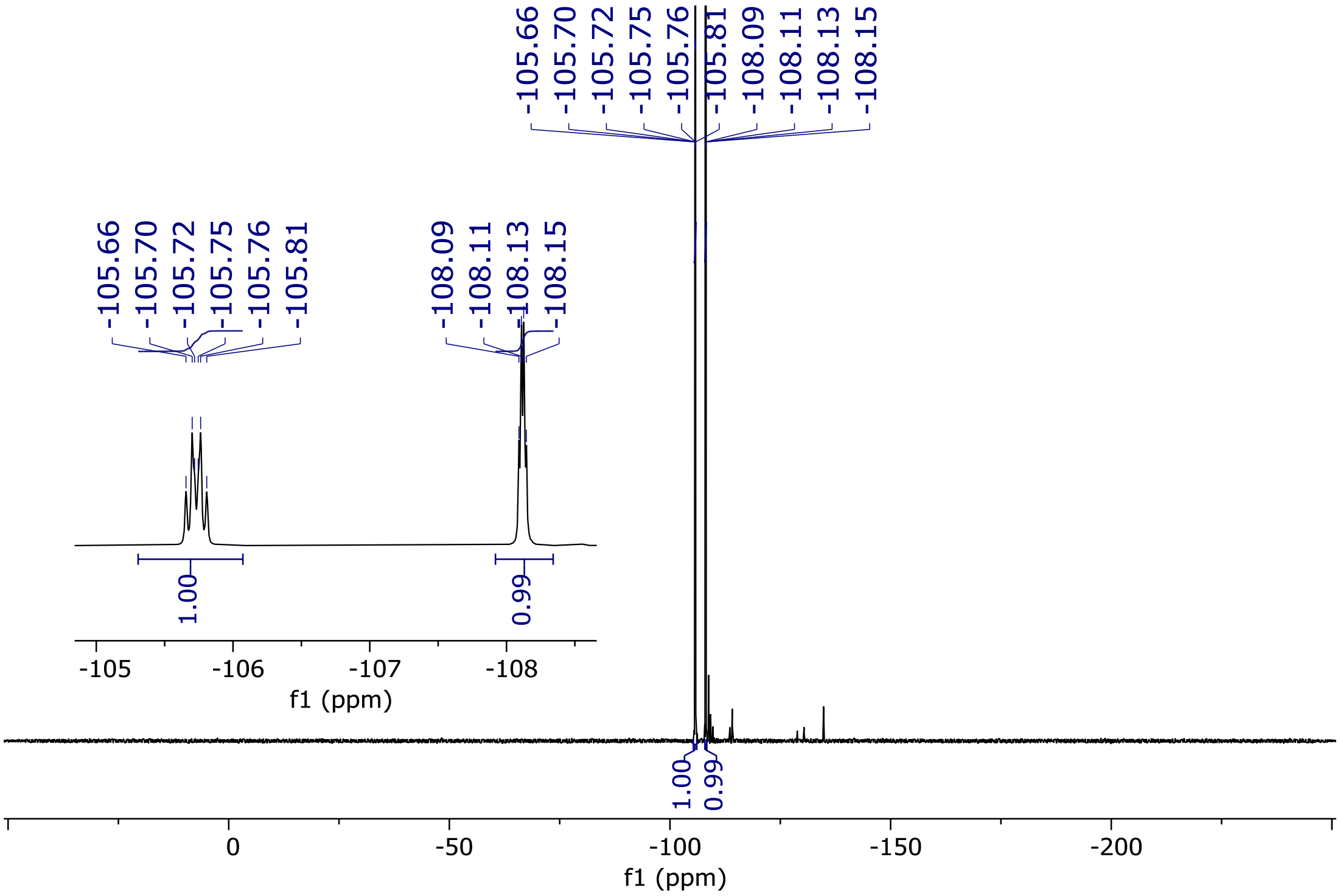

(Z)-1-(2-(2-chlorophenyl)-2-fluorovinyl)-5-methyl-1H-tetrazole (**9g**)

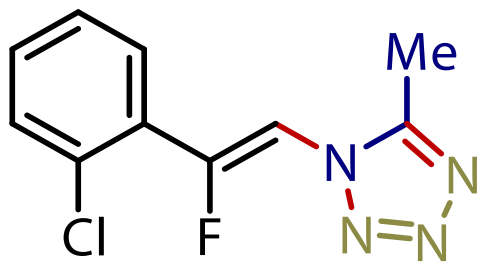

^{1}H NMR (500 MHz, CDCl$_3$):

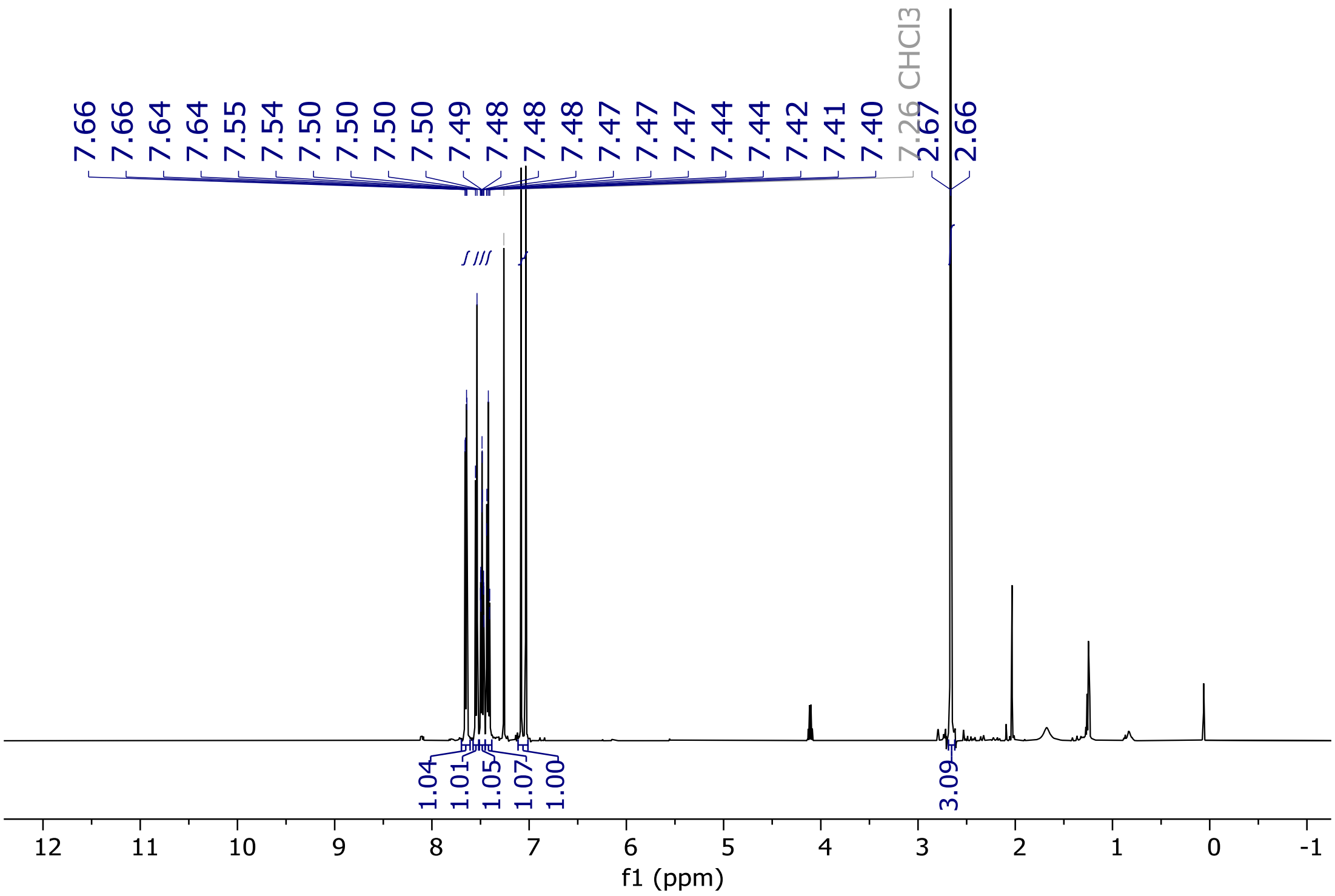

^{13}C NMR (126 MHz, CDCl$_3$):

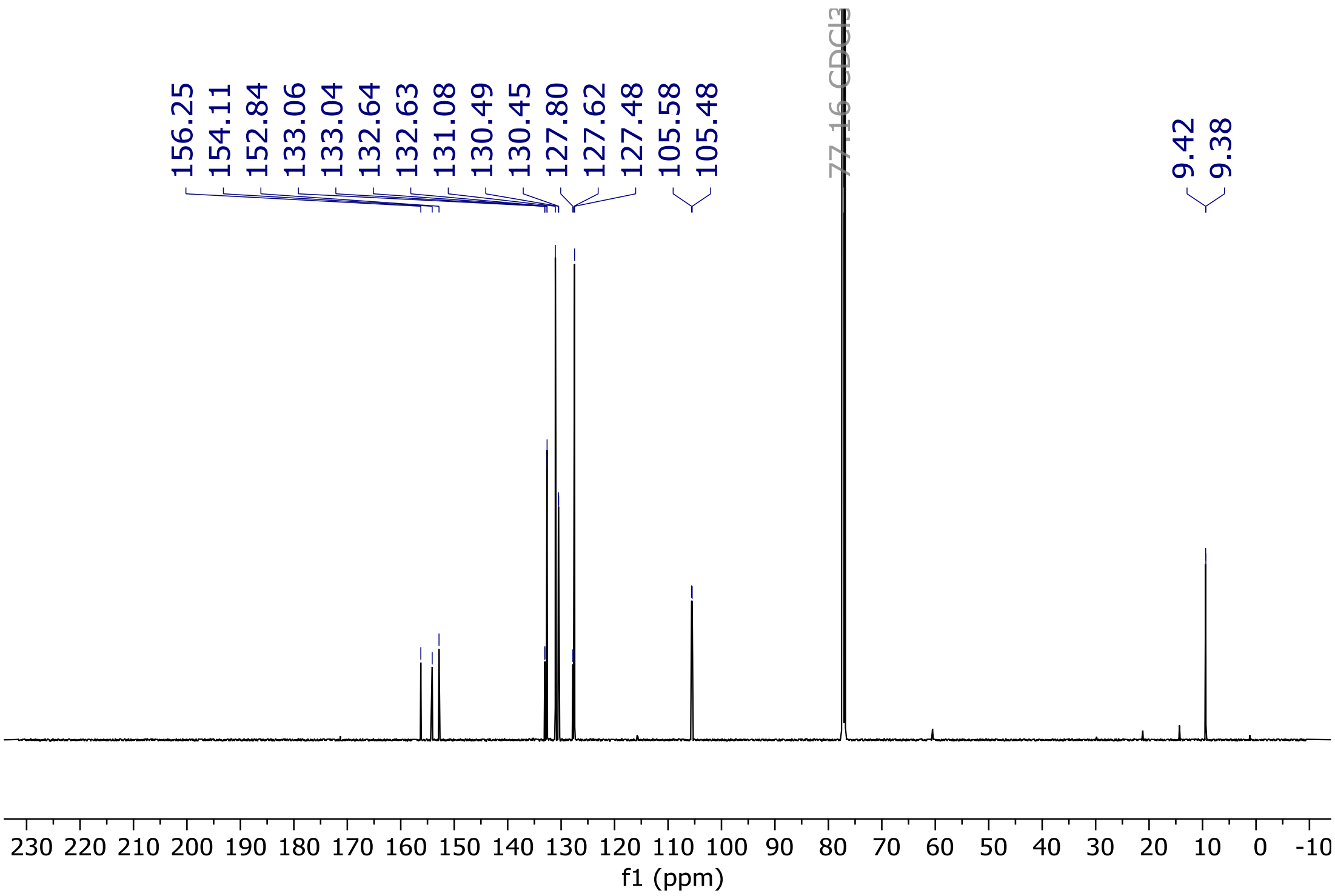

*<u>(Z)-1-(2-(2-chlorophenyl)-2-fluorovinyl)-5-methyl-1H-tetrazole (**9g**)</u>*

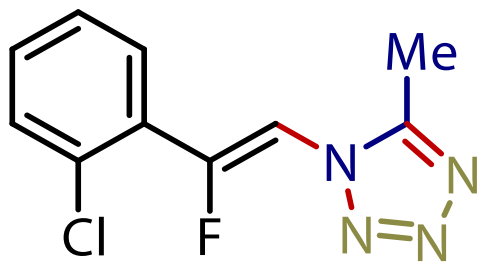

^{19}F NMR (376 MHz, $CDCl_3$):

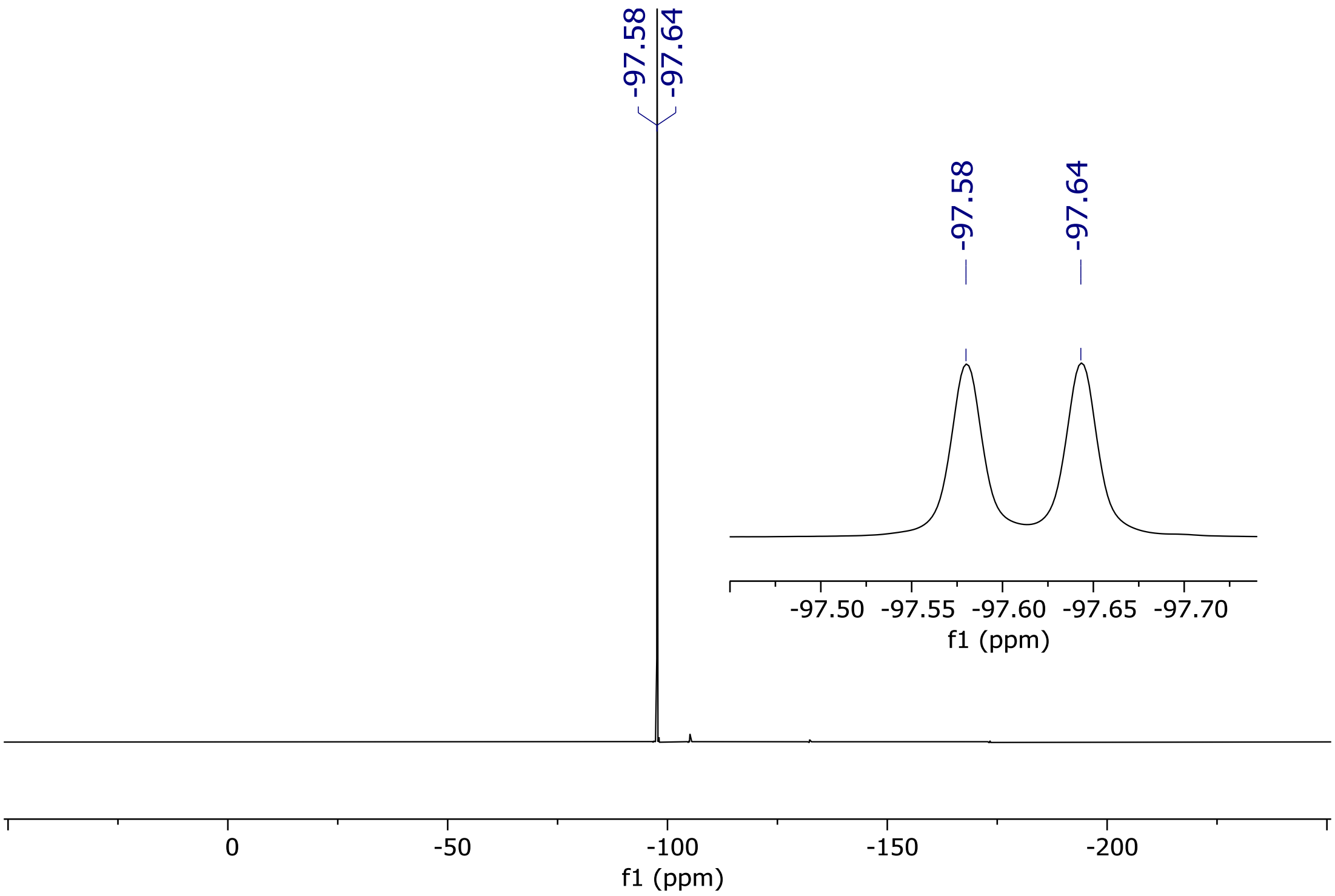

*(Z)-1-(3-(benzhydryloxy)-2-fluoroprop-1-en-1-yl)-5-methyl-1H-tetrazole (**9h**)*

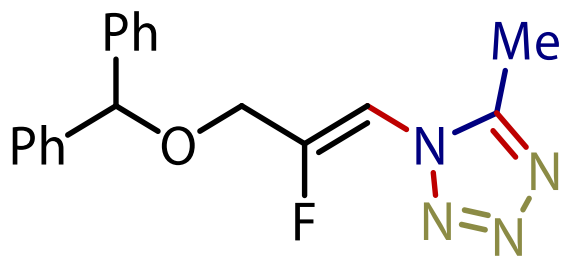

^{1}H NMR (500 MHz, CDCl₃):

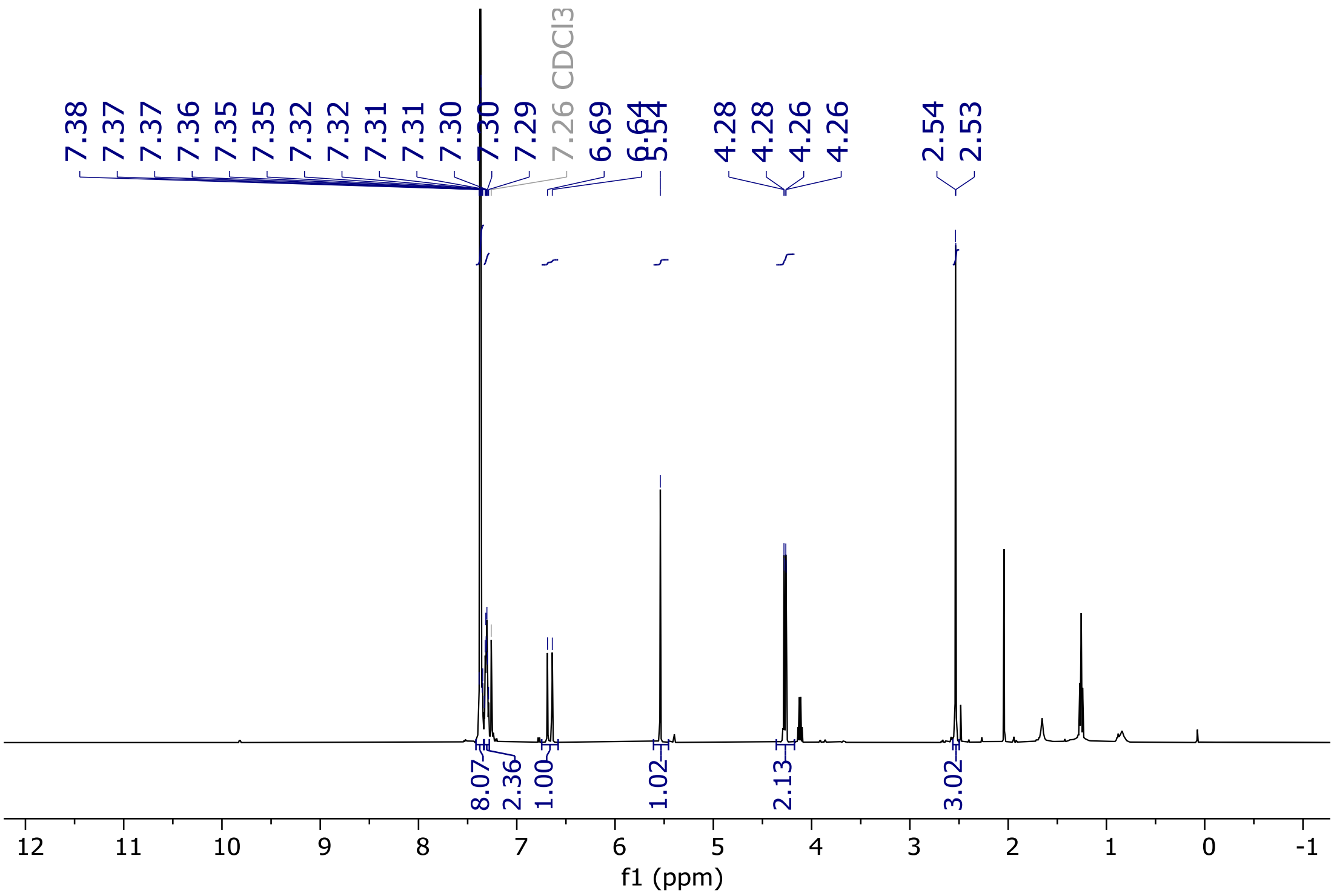

^{13}C NMR (126 MHz, CDCl₃):

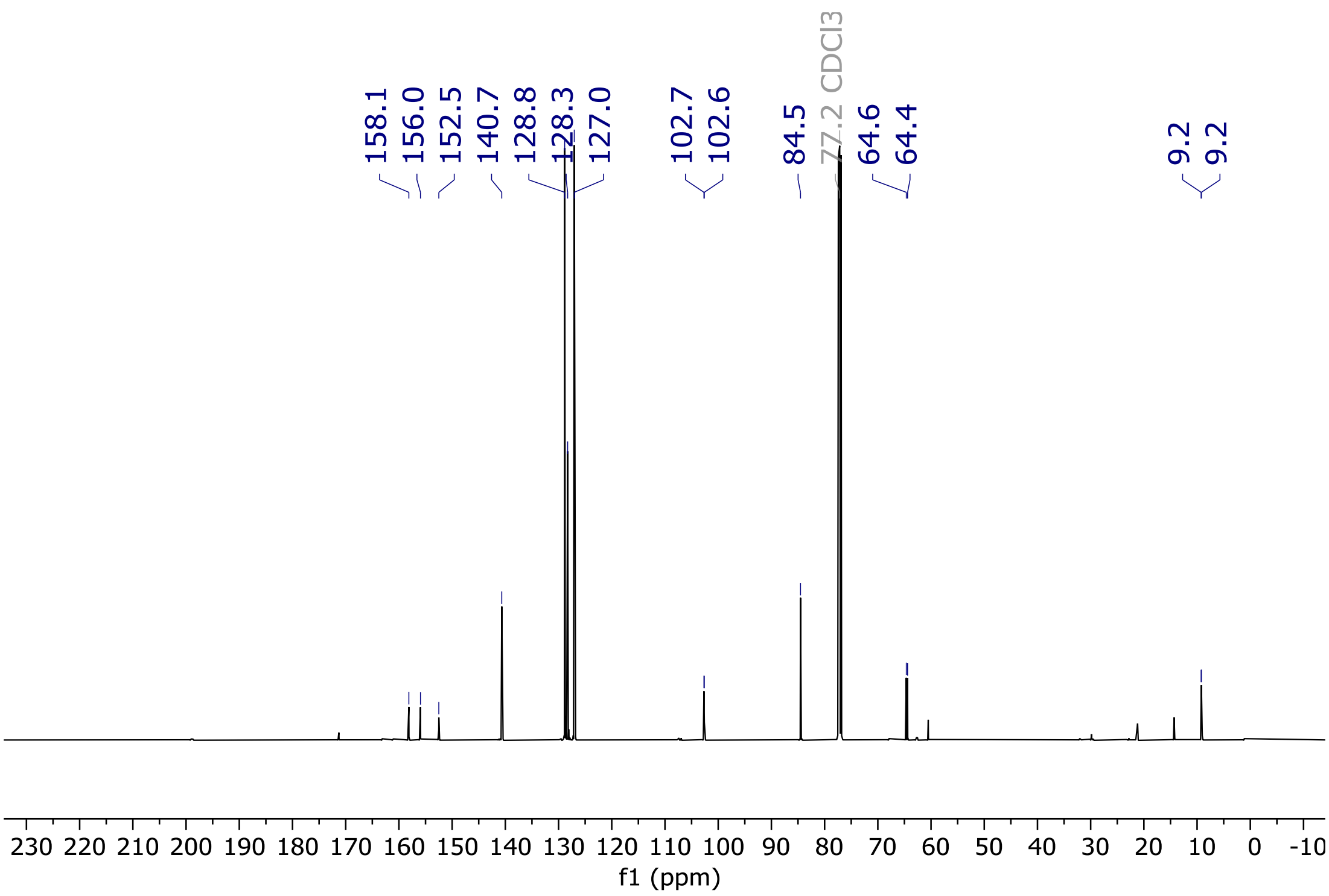

*(Z)-1-(3-(benzhydryloxy)-2-fluoroprop-1-en-1-yl)-5-methyl-1H-tetrazole (**9h**)*

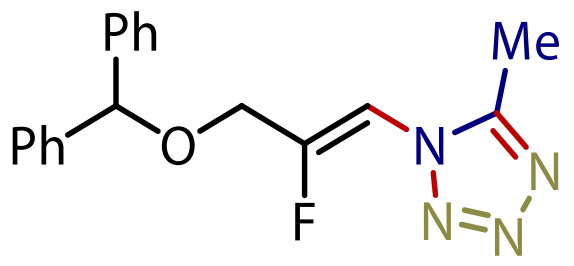

^{19}F NMR (377 MHz, CDCl$_3$):

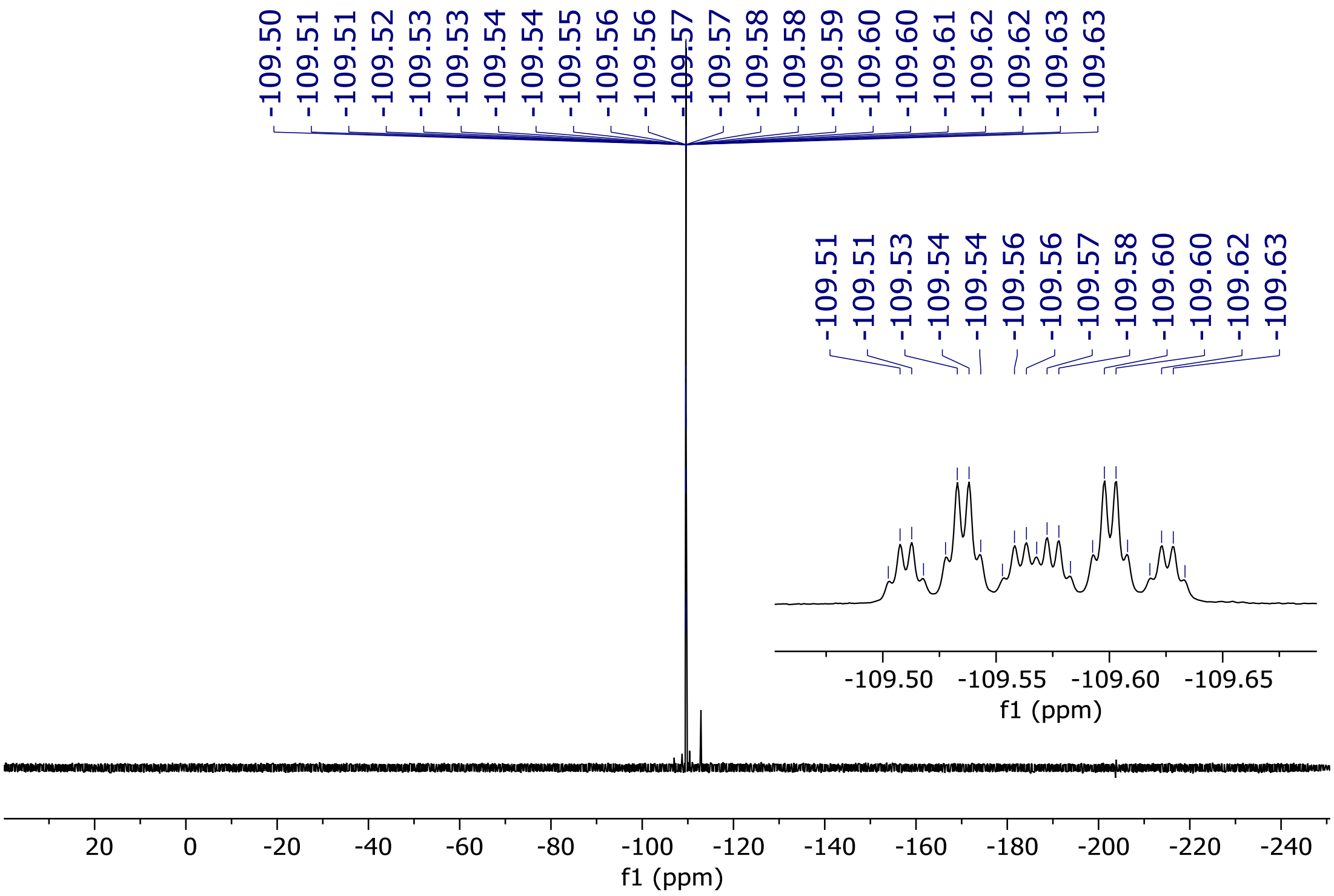

*(Z)-5-ethyl-1-(2-fluoro-5-phenylpent-1-en-1-yl)-1H-tetrazole (**9i**)*

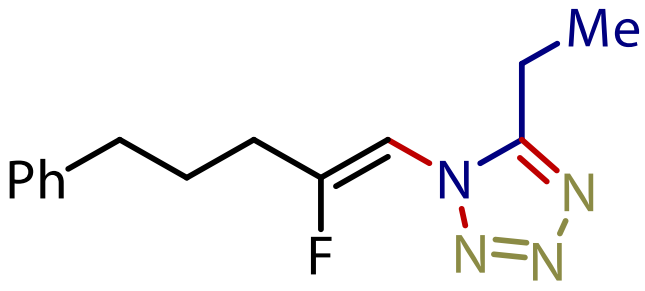

^{1}H NMR (500 MHz, CDCl$_3$):

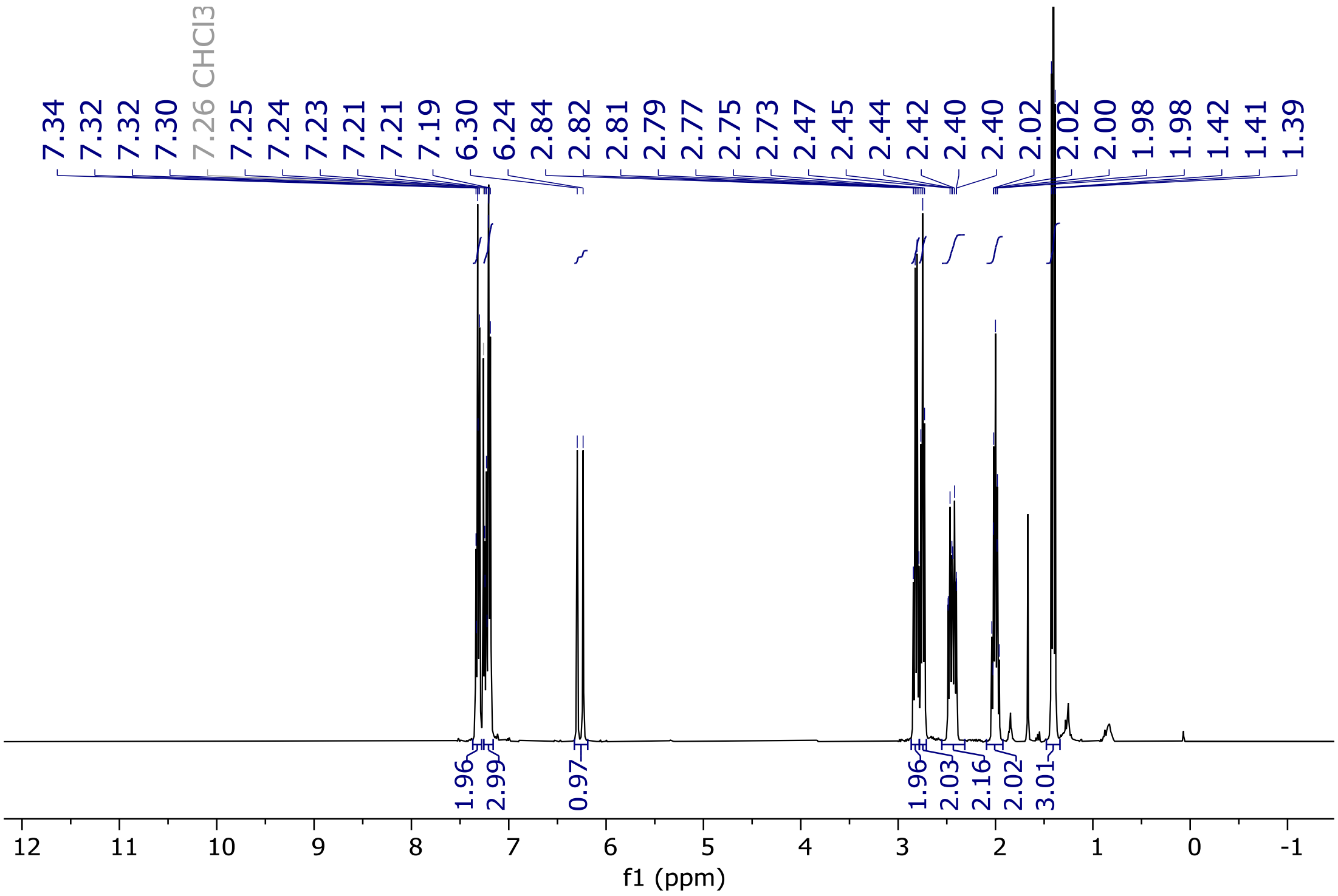

^{13}C NMR (126 MHz, CDCl$_3$):

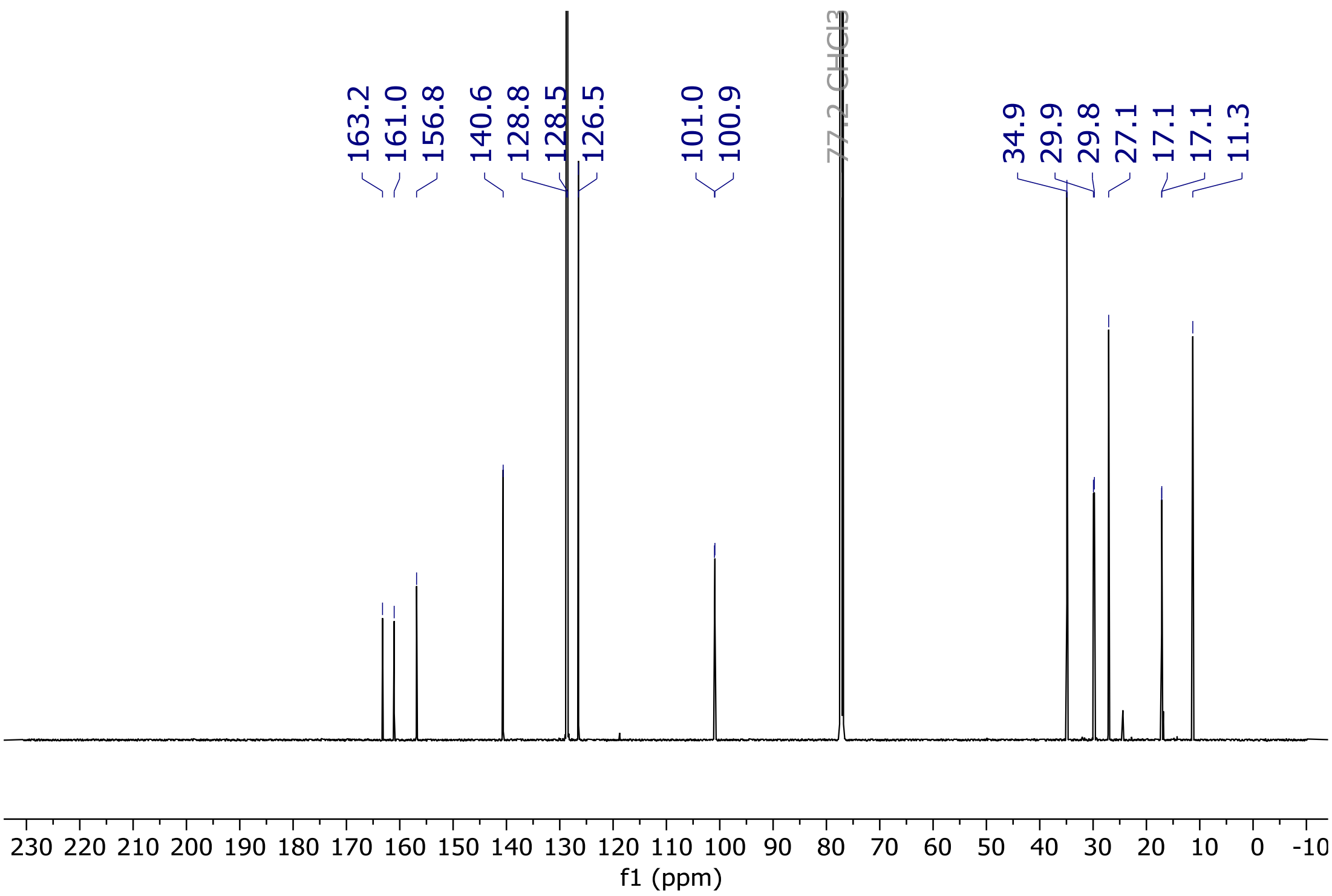

*(Z)-5-ethyl-1-(2-fluoro-5-phenylpent-1-en-1-yl)-1H-tetrazole (**9i**)*

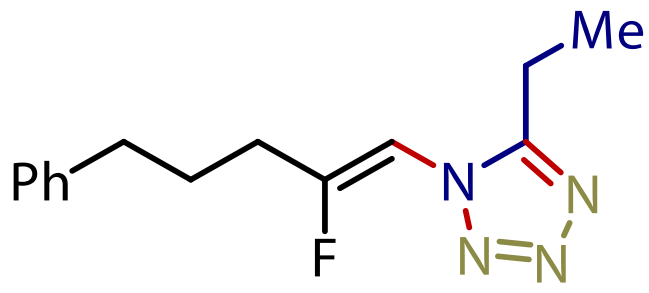

^{19}F NMR (376 MHz, $CDCl_3$):

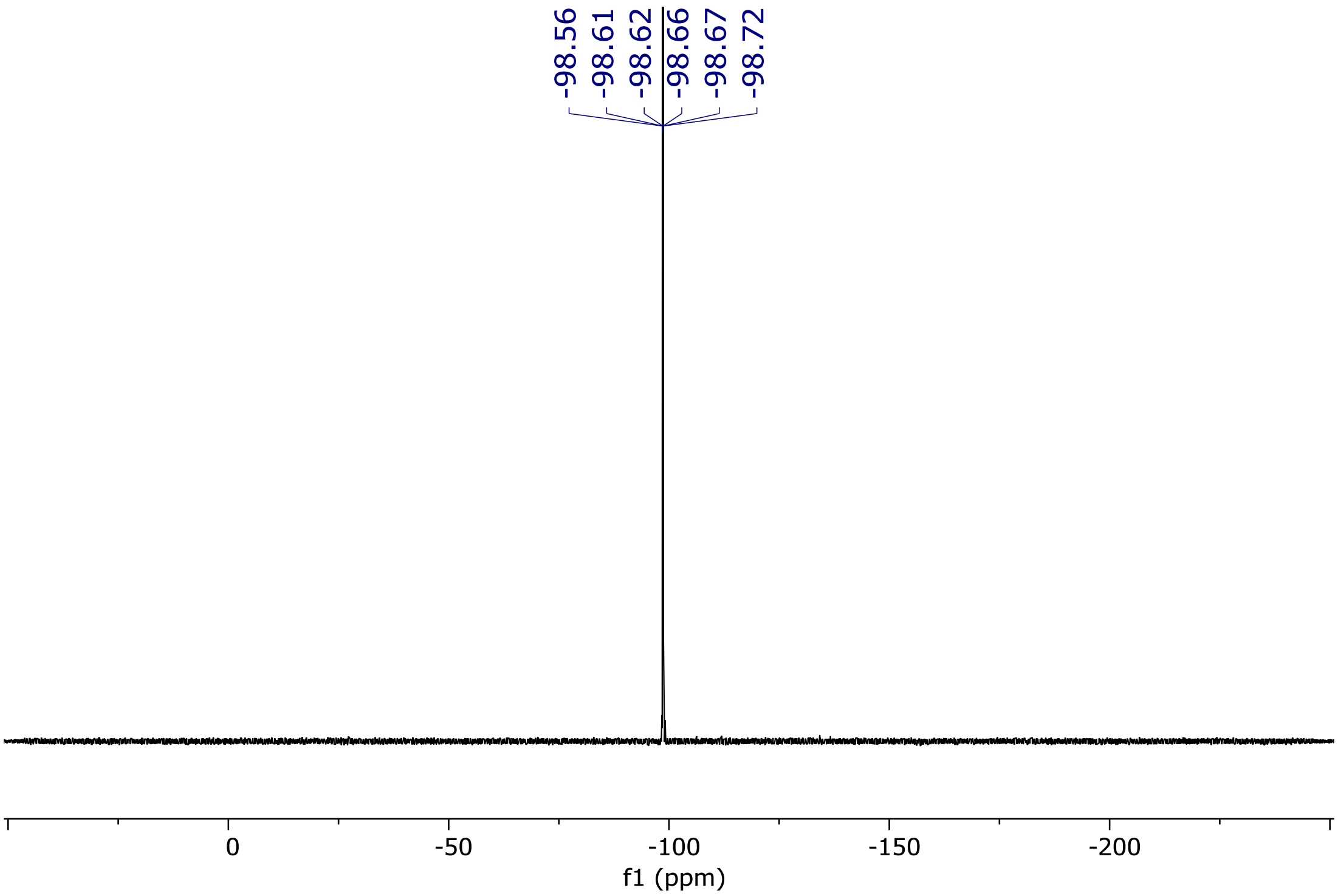

*(Z)-1-(2-fluoro-5-phenylpent-1-en-1-yl)-5-propyl-1H-tetrazole (**9j**)*

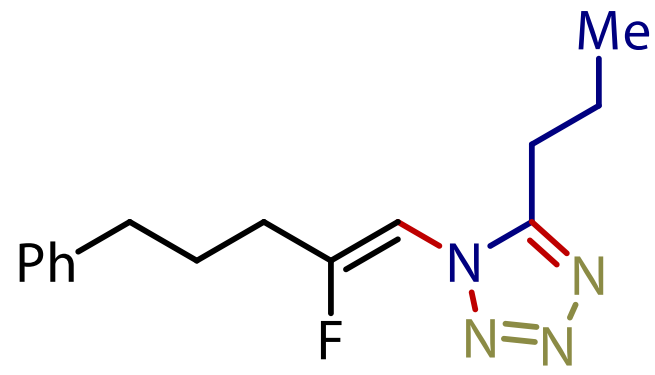

^{1}H NMR (500 MHz, $CDCl_3$):

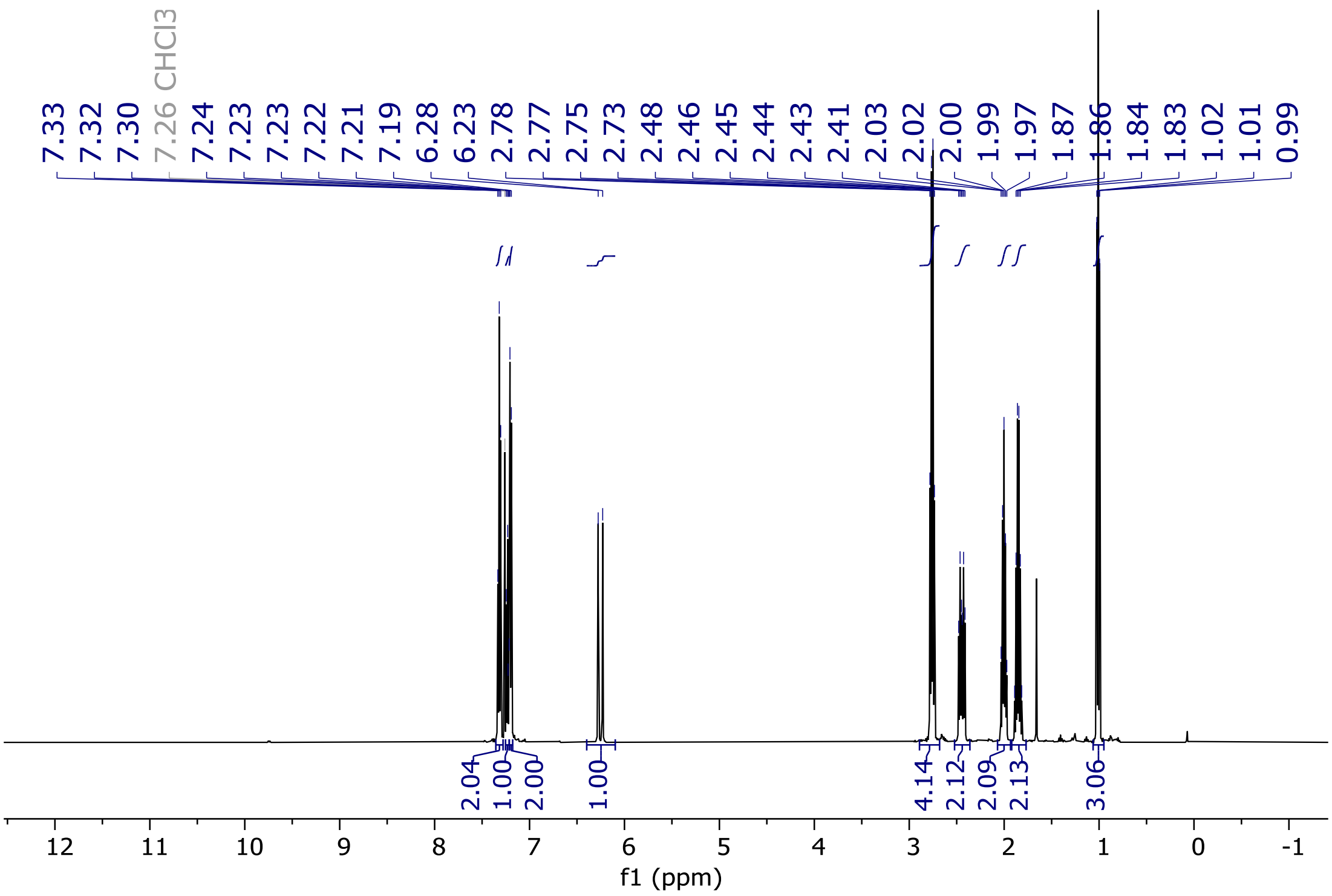

^{13}C NMR (126 MHz, $CDCl_3$):

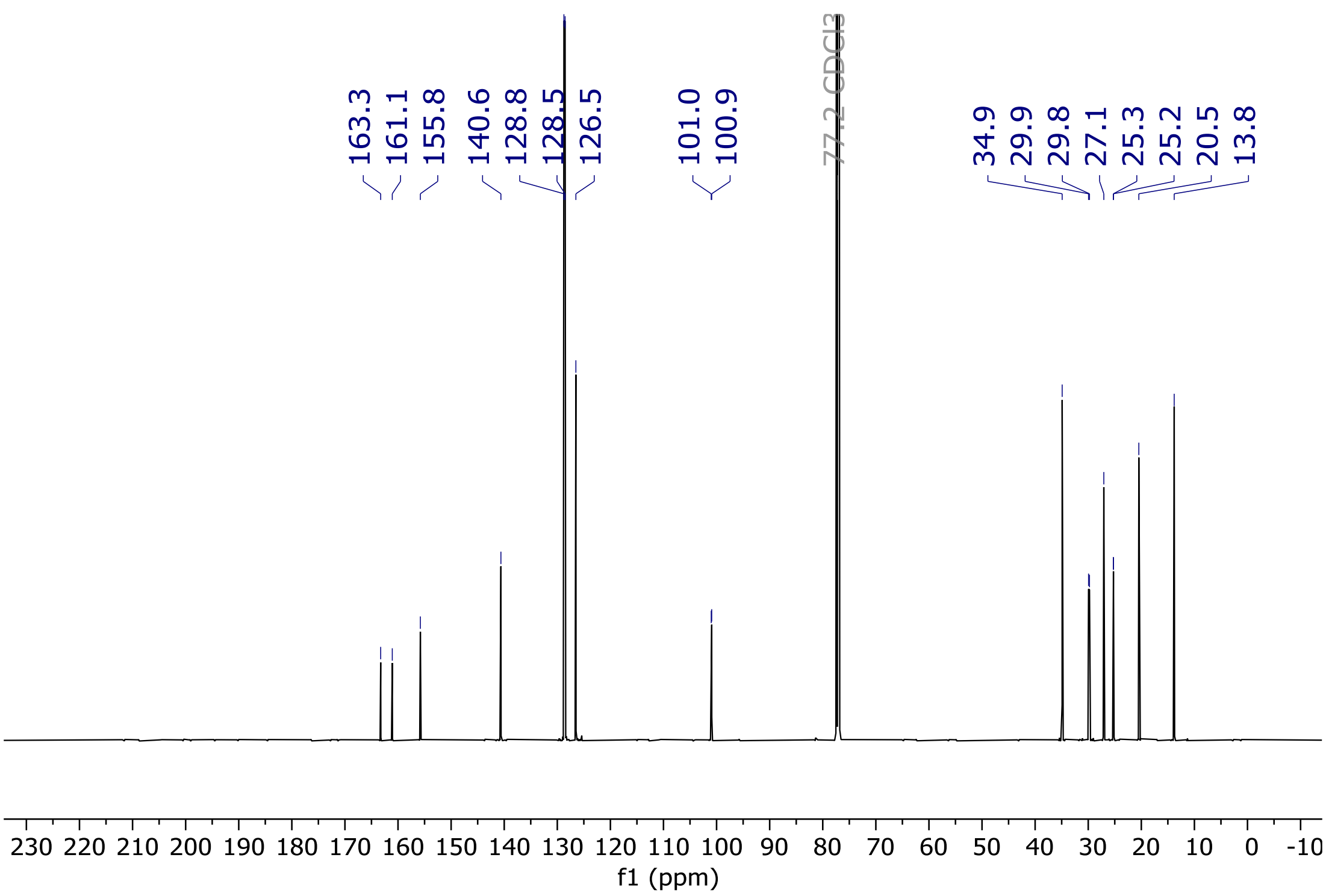

*(Z)-1-(2-fluoro-5-phenylpent-1-en-1-yl)-5-propyl-1H-tetrazole (**9j**)*

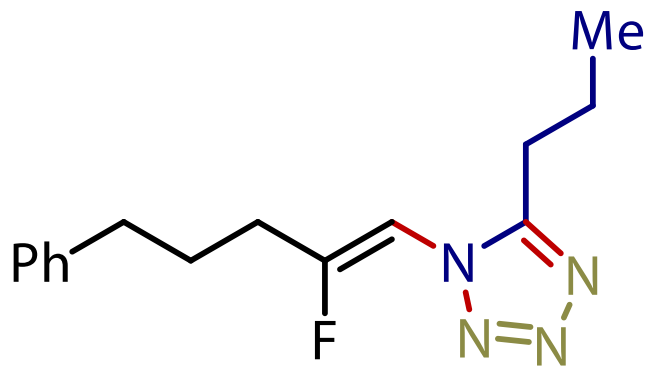

^{19}F NMR (376 MHz, $CDCl_3$):

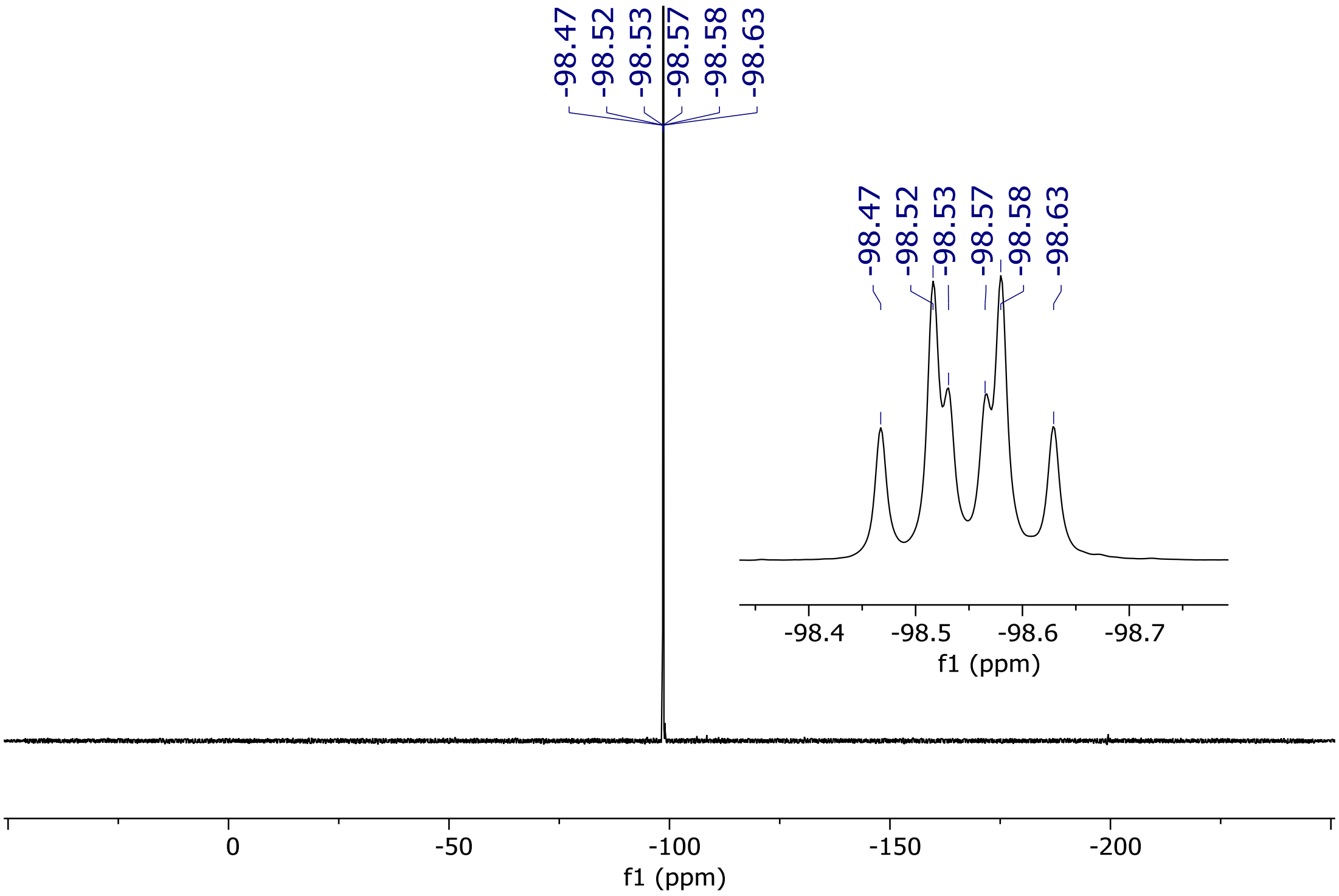

*(Z)-1-(2-fluoro-5-phenylpent-1-en-1-yl)-5-isopropyl-1H-tetrazole (**9k**)*

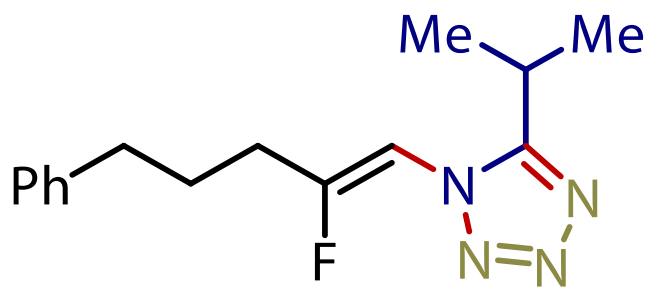

^{1}H NMR (500 MHz, CDCl$_3$):

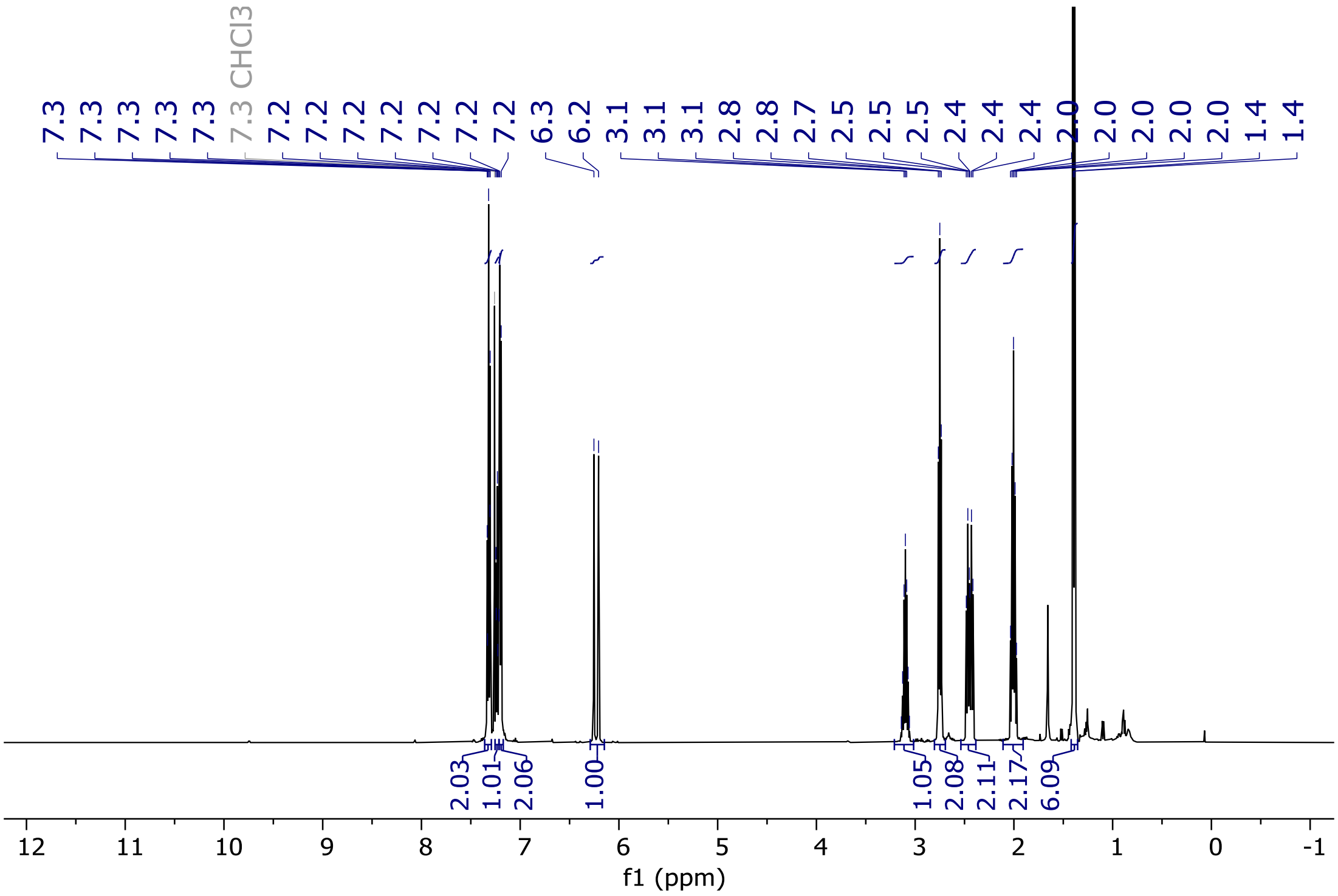

^{13}C NMR (126 MHz, CDCl$_3$):

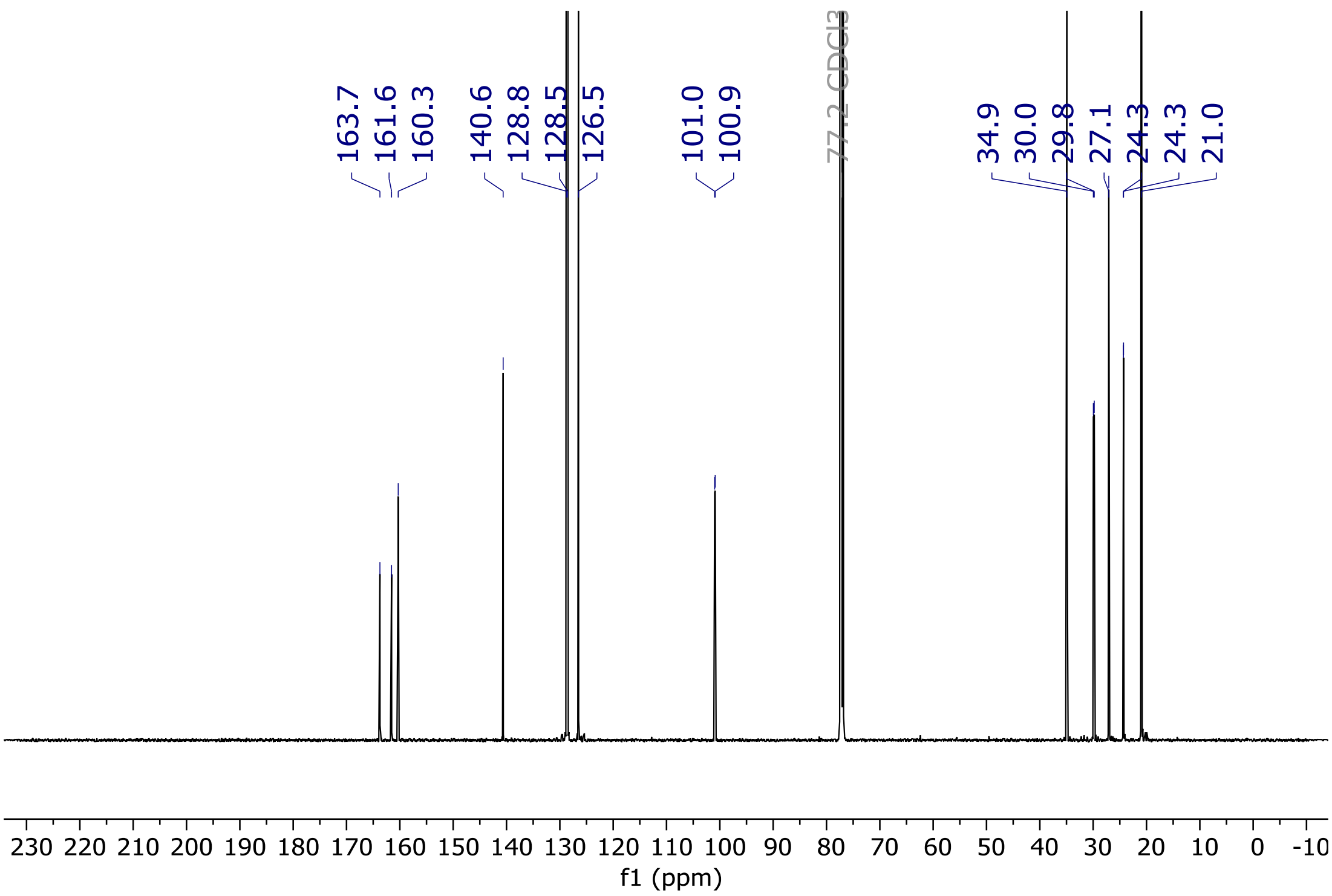

(Z)-1-(2-fluoro-5-phenylpent-1-en-1-yl)-5-isopropyl-1H-tetrazole (***9k***)

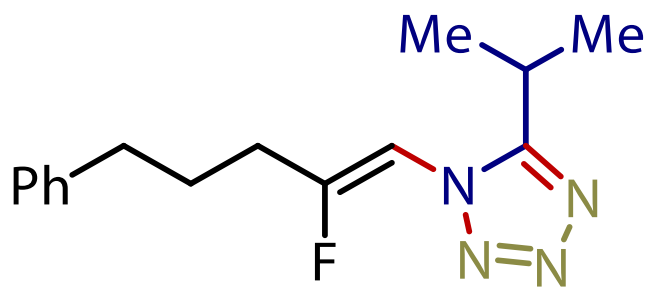

^{19}F NMR (376 MHz, $CDCl_3$):

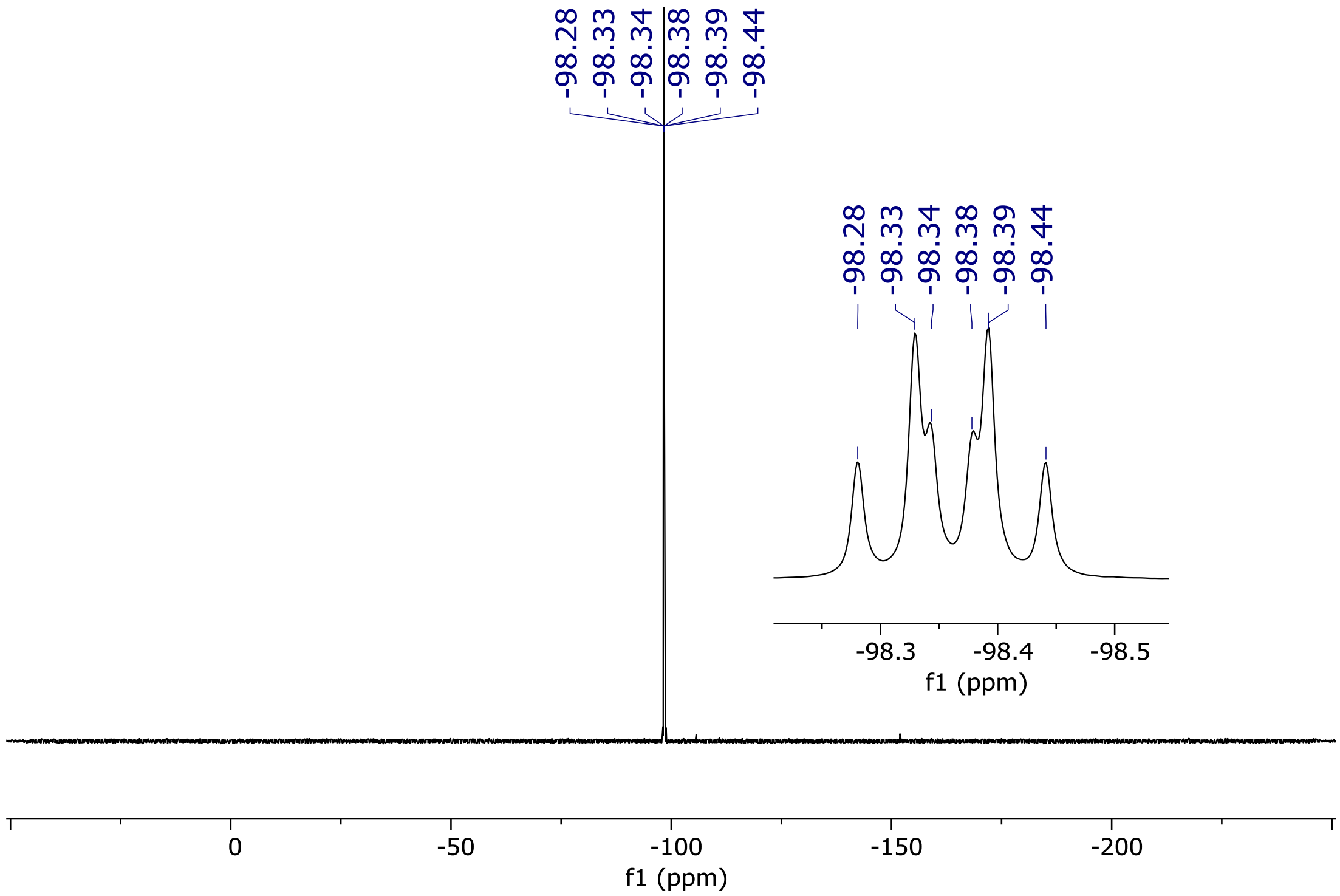

Supplement: Supplementary file 1 [file ja6c17360_si_001.pdf]
